# Lipidomic profiles, lipid trajectories and clinical biomarkers in female elite endurance athletes

**DOI:** 10.1038/s41598-020-59127-8

**Published:** 2020-02-11

**Authors:** Tibor V. Varga, Ashfaq Ali, Jose A. R. Herrera, Linda L. Ahonen, Ismo M. Mattila, Naba H. Al-Sari, Cristina Legido-Quigley, Sven Skouby, Søren Brunak, Åsa B. Tornberg

**Affiliations:** 1 Novo Nordisk Foundation Center for Protein Research, Translational Disease Systems Biology Group, Faculty of Health and Medical Sciences, University of Copenhagen, Copenhagen, Denmark; 2 Department of Clinical Sciences, Genetic and Molecular Epidemiology Unit, Lund University, Skåne University Hospital Malmö, Malmö, Sweden; 3 Steno Diabetes Center Copenhagen, Gentofte, Denmark; 4 National Bioinformatics Infrastructure Sweden (NBIS), SciLifeLab, Lund, Sweden; 5 Biosyntia ApS, Copenhagen, Denmark; 6 Institute of Pharmaceutical Science, Faculty of Life Sciences and Medicine, King’s College London, London, United Kingdom; 7 Faculty of Health and Medical Sciences, University of Copenhagen, Copenhagen, Denmark; 8 Endocrinological and Reproductive Unit, Department of Obstetrics and Gynecology, Herlev Hospital, Herlev, Denmark; 9 Department of Health Sciences, Lund University, Lund, Sweden

**Keywords:** Predictive markers, Dyslipidaemias, Time series, Machine learning, Bone, Metabolism, Reproductive biology, Biomarkers, Endocrinology, Medical research, Risk factors

## Abstract

We assessed whether blood lipid metabolites and their changes associate with various cardiometabolic, endocrine, bone- and energy-related comorbidities of Relative Energy Deficiency in Sport (RED-S) in female elite endurance athletes. Thirty-eight Scandinavian female elite athletes underwent a day-long exercise test. Five blood samples were obtained during the day - at fasting state and before and after two standardized exercise tests. Clinical biomarkers were assessed at fasting state, while untargeted lipidomics was undertaken using all blood samples. Linear and logistic regression was used to assess associations between lipidomic features and clinical biomarkers. Overrepresentations of findings with *P* < 0.05 from these association tests were assessed using Fisher’s exact tests. Self-organizing maps and a trajectory clustering algorithm were utilized to identify informative clusters in the population. Twenty associations *P*_*FDR*_ < 0.05 were detected between lipidomic features and clinical biomarkers. Notably, cortisol demonstrated an overrepresentation of associations with *P* < 0.05 compared to other traits (*P*_*Fisher*_ = 1.9×10^−14^). Mean lipid trajectories were created for 201 named features for the cohort and subsequently by stratifying participants by their energy availability and menstrual dysfunction status. This exploratory analysis of lipid trajectories indicates that participants with menstrual dysfunction might have decreased adaptive response to exercise interventions.

## Introduction

Relative Energy Deficiency in Sport (RED-S) represents a metabolically altered state that is associated with imparied metabolic rate and bone health, decreased immunity and protein synthesis, and cardiovascular comorbidities^1^. Although RED-S occurs both in males and females, detection is easier in females, partly due to the fact that RED-S is likely to be exacerbated by symptoms related to menstrual dysfunction^1,2^. Low energy availability (LEA), one of the most important comorbidities of RED-S, represents a chronic negative energy balance resulting from a failure to satisfy real-time energy requirements. Consequently, LEA often results in prolonged states of undernourishment, a metabolic state with distinct biomarker profiles^3^. Functional hypothalamic amenorrhea (FHA), another common comorbidity of RED-S, is also associated with markedly altered metabolic profiles^4^.

LEA, as a negative energy balance state, can be used as a model to identify metabolic processes that are activated during energy deficiency. Identifying biomarkers associated with various cardiovascular, hormonal and anthropometric comorbidities in RED-S may improve our understanding of the biology of undernutrition and may highlight metabolic pathways which are important in states of metabolic imbalance.

Acute changes of metabolomic profiles in response to various exposures, such as exercise, and oral glucose- or lipid tolerance tests, have been investigated in the Human Metabolome Study in 15 healthy males^5^, but no such studies have been undertaken in undernourished females. Nonetheless, unfavourable lipid profiles have been associated with RED-S comorbidities, such as menstrual dysfunction and impaired bone health, in female athletes^6,7^. In this study, we mapped acute changes in lipidomic profiles in Danish and Swedish female elite athletes at risk of RED-S from the LEA Study, who underwent a day-long experiment involving standardized exercise tests. We hypothesized that i) fasting levels of lipidomic features will associate and/or predict various RED-S comorbidities and ii) intensive exercise will induce marked changes in lipidomic profiles and these adaptations will differ between participants demonstrating RED-S comorbidities, such as LEA or FHA status.

## Materials and Methods

### Study population

**Figure 1:**
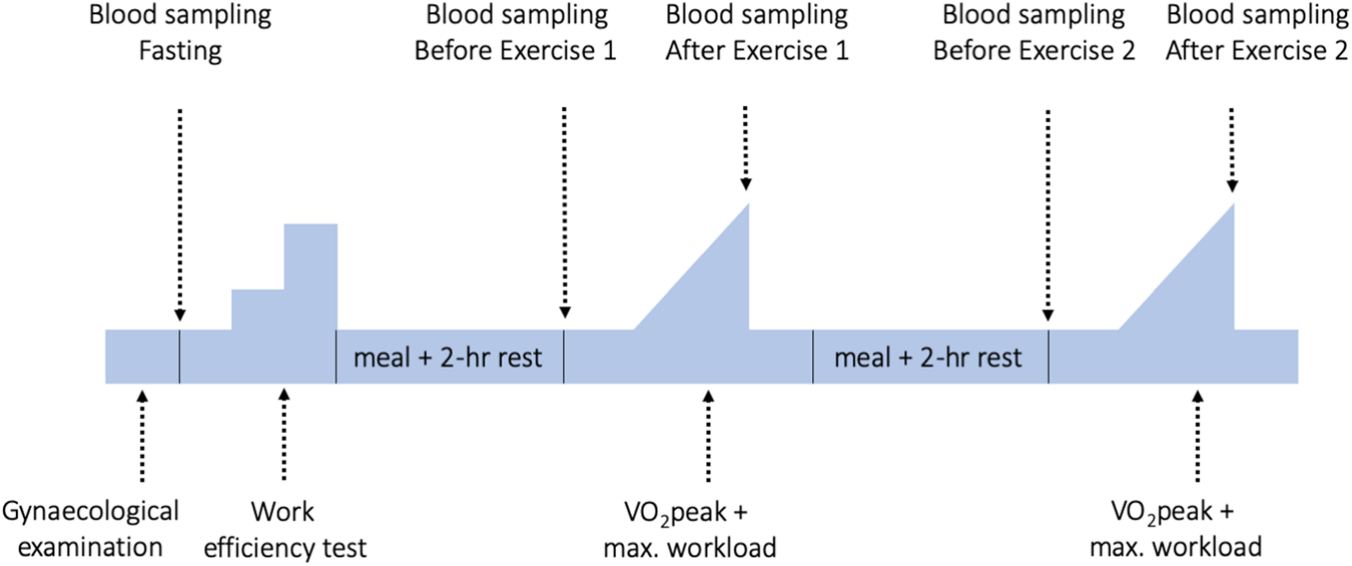
LEA study protocol.

The LEA Study comprises Swedish and Danish female elite athletes (at the national level or competitive endurance athletes from regional sports clubs) aged between 18 and 38, training a minimum of five times per week. The study is described in detail elsewhere^3,8–10^. In short, all participants underwent a day-long exercise protocol, and blood samples were obtained in fasting state and four additional times: before and after two intensive exercise tests (designed to obtain VO_2_peak values) that participants undertook during the day. In total, 38 participants had the necessary data for this study. Figure 1 shows the study protocol with all the times of data collection pertaining to this project. All subjects signed a written informed consent and the study was approved by the Swedish and Danish Confederation of Sports, Team Denmark and the data inspectorate. Ethical approval was obtained from the Regional Ethical Committee in Lund, Sweden (2011/576, 2016/1051) and the Regional Ethical Committee of the Capital Region, Denmark (H-4-2011-096). The LEA Study was conducted in accordance with the Declaration of Helsinki.

### Clinical biomarkers

Venous blood was drawn five times throughout the study day: at fasting state, and before and after two separate exercise tests in the morning and in the afternoon. Blood samples were prepared and the resulting plasma and serum aliquots were stored at −80 °C. Blood lipid levels were assessed using VITROS Chemistry Products DT slides (Ortho-Clinical Diagnostics, Buckinghamshire, UK) as described previously^3^. Circulating hormone levels were assessed using a wide range of assays described elsewhere^3,4^. Antropometric measures and bone health was measured as described previously^3^. LEA status (1/0) was determined as <45 kcal energy availability (188 kJ)/kg fat-free mass/day^8^. FHA status (1/0) was defined as menstrual cycles >35 days, no menarche after 15 yrs of age, or the absence of at least three consecutive menstrual cycles, where other causes than hypothalamic suppression had been ruled out^11^. In this study, 26 outcomes were considered, organized into four subgroups: i) *laboratory lipids*: triglycerides, high-density lipoprotein cholesterol (HDL-C), low-density lipoprotein cholesterol (LDL-C) and total cholesterol (TC); ii) *sex hormones and related*: total testosterone, free testosterone, estrogen, progesterone, prolactin, luteinizing hormone (LH), follicle stimulating hormone (FSH), androstendione, dehydroepiandrosterone (DHAS), sex hormone binding globulin (SHBG) and FHA; iii) *other hormones*: thyroid stimulating hormone (TSH), leptin, cortisol, adrenocorticotropic hormone (ACTH), brain-derived neurotrophic factor (BDNF), growth hormone (GH) and insulin; iv) *anthropometric and energy-related*: BMI, bone mineral density (BMD), fat mass percentage and LEA. While FHA and LEA status were binary (0/1 for no/yes), the rest of the variables were continuous.

### LC-MS lipidomics

The 190 (N = 38×5) frozen blood serum samples were sent to the Metabolomics Laboratory at the Steno Diabetes Center Copenhagen (Gentofte, Denmark). The samples had not been thawed before this analysis. The laboratory procedures and the list of lipids used as standards and calibrants have been previously described^12^. In brief, during sample preprocessing, 10 μl of 0.9% NaCl, 92 μl of chloroform:methanol (2:1, v/v) and 28 μl of a lipid standard solution (10 µg/ml) were added to 10 μl of serum samples. All samples and controls were mixed and placed on ice for a minimum of 30 minutes and subsequently centrifuged (3 min, 4 °C, 9400 *g*). Following centrifugation, 60 μl from the lower layer of each sample was transferred to a glass vial and mixed with 60 μl chloroform:methanol (2:1, v/v). Untargeted lipidomics analyses were performed by an ultra-high performance liquid chromatography quadrupole time-of-flight mass spectrometry (UHPLC-Q-TOF-MS) equipment by Agilent Technologies (Santa Clara, CA, USA). The raw lipidomic data was processed using MZmine 2^13^, an open-source software for mass-spectrometry data processing. In brief, the processing steps included mass detection, chromatogram builder, chromatogram deconvolution, isotopic peak grouper, peak filter, join aligner, peak list row filter, gap filling and annotation to in-house library^14^. In total, 201 lipids could be annotated using the Steno Diabetes Center internal peak library based on m/z values and retention time. These metabolites represent a subset that we consider to be annotated with higher confidence and thus, are the main focus of our investigation. The rest of the lipids were annotated based on their m/z values using LIPID MAPS^15,16^, a comprehensive and widely used online resource for lipidomics annotations. As compounds annotated using LIPID MAPS have lower confidence annotations, we exercised caution in presenting individual associations with these features in downstream analyses. After data processing and annotation, the available lipids (n = 1,060) were further filtered. Lipids with amigous annotations were removed: these included lipids used as standards (n = 17), lipids with no annotations (n = 167) and duplicated LIPID MAPS annotations (n = 242). The remaining named, unique lipids (n = 634) comprise the final lipidomics dataset used in the study.

### Statistical analyses

Statistical analyses were performed using R v3.5.2^17^ and Python 3.6^18^. The median, mean and maximum feature missingness rates in the dataset were 1.1%, 3.7% and 45%, respectively. Missing data of the unscaled data was imputed using the *missForest* R package^19^, an algorithm utilizing random forest to impute missing data in the data matrix based on observed datapoints.

The main data analysis can be separated into two main parts:

#### Analyses at fasting state

For the first part of the analysis, the dataset was restricted to the first timepoint (fasting status). Highly correlated features (Pearson’s |r| > 0.8) were removed, which resulted in 216 lipidomic features for further analysis. All lipid features were scaled and centered (mean = 0, standard deviation = 1). Linear regression (24 unscaled numeric outcomes) and logistic regression (2 categorical outcomes) models were constructed with the individual lipid features being the independent variables. In these models, age was added as a covariate. In total, 26 × 216 = 5,616 statistical tests were performed. *P* values from these tests were corrected using the Benjamini-Hochberg false discovery rate (FDR) method (α = 0.05)^20^. To assess whether *P* < 0.05 findings were overrepresented among results for a given trait (compared to the other 25 traits), Fisher’s exact tests were employed. Here, *P* < 0.002 (α = 0.05/26, adjusted using the Bonferroni method) were considered statistically significant. To evaluate the cumulative predictive utilities of the 216 lipids towards the 24 scaled numeric outcomes and the 2 categorical outcomes, linear and logistic regression was utilized in a leave-one-out cross validation (LOOCV) framework, which has been shown to be better suited for small datasets compared to regular *k*-fold cross-validation. In this framework, for each outcome, models are trained on 37 samples and tested on the 38^th^ sample. This results in 38 test sets, each test set being a participant in the study. For numeric outcomes, prediction error, measured by mean absolute error (MAE), and explained variance (R^2^) were subsequently averaged between the 38 test sets. For categorical outcomes, prediction accuracy is reported (number of correct predictions/number of all predictions).

#### Analyses considering all timepoints

For the second part of the analysis, data at all five timepoints were considered. All 634 lipids were stratified into distinct clusters using the Compass hybrid method^21^. First, the 634 lipid features were scaled and centered into z-scores. Subsequently, Self-Organizing Maps (SOM)^22^ were employed to reduce dimensionality and cluster participants based on the lipidomics data at all five timepoints, separately. In this method, a set of interconnected neurons is trained to adapt to the original data set. The way nodes are connected is defined beforehand; here we tested hexagonal (6 connections) grids of sizes 4 × 4, 5 × 5, 6 × 6 and 7 × 7. The training process consists in reducing the Euclidean distances between the model neurons and the data points in a process called competitive learning. For each data sample, distances to the model vectors are calculated, and the closest neuron, also called as Best Matching Unit, is selected as the “winner”. Next, the values of the “winning” neuron and its neighbour nodes are adjusted towards the sample. This process is iterated until the network approximates to the original data space. Once the training process is finished, it is possible to assign samples to their Best Matching Units, which can be considered as clusters. In this study, the most optimal grid, based on fitness, was a 4 × 4 neuron structure. The resulting neurons were aggregated into larger groups using the Partitioning Around Medoids algorithm, estimating the number of clusters based on the optimum average silhouette width of the SOM nodes. Associations between the resulting SOM clusters and the two categorical variables, LEA and FHA were assessed using chi-squared tests. Associations with *P* < 0.05 were considered statistically significant.

Lipid trajectory clustering was undertaken using the *traj* R package. Here, highly correlated features (Pearson’s |r| > 0.8) were removed, which resulted in 245 lipid features in analysis. Using *traj*, trajectory clustering for all individual lipid features were undertaken separately and for each iteration, 24 meta-features describing the lipid trajectories were extracted. These meta-features included trajectory properties such as feature range, mean over time, change, slope, standard deviation, maximum differences between timepoints, etc. The obtained meta-features were subsequently visualized using a heatmap.

## Results

### Participant characteristics

Descriptive statistics of various biomarkers in LEA study participants have been published before^3,4^, but these statistics described study populations with slight differences in size and constitution. Therefore, medians and interquartile ranges (IQR) for all continuous clinical biomarkers at fasting state for all participants eligible for this study (together and stratified by LEA and FHA status) are shown in Table 1. Amongst the participants, 12 individuals had LEA, while 26 had sufficient energy availability (EA). Furthermore, while 27 participants were diagnosed with FHA, 11 participants had eumenorrhea (healthy menstrual cycle).

**Table 1:** Characteristics of LEA participants at fasting status (N = 38).

Trait	All	Sufficient EA (n = 26)	LEA (n = 12)	EUM (n = 11)	FHA (n = 27)
Age (years)	26.5 [21.25;29]	26 [21;30]	27 [23.75;28]	27 [23;31]	26 [21;29]
HDL-C (mmol/l)	1.8 [1.6;2.07]	1.79 [1.43;2]	1.99 [1.74;2.12]	1.76 [1.29;2]	1.86 [1.66;2.06]
LDL-C (mmol/l)	2.3 [2.12;2.9]	2.25 [1.95;2.9]	2.3 [2.27;2.82]	2.3 [2.25;2.5]	2.3 [2;3.1]
TC (mmol/l)	4.55 [4.1;5.1]	4.35 [3.82;5.07]	4.7 [4.45;5.12]	4.6 [4;4.9]	4.5 [4.1;5.3]
TG (mmol/l)	0.67 [0.61;0.87]	0.66 [0.58;0.79]	0.68 [0.64;1]	0.7 [0.66;0.91]	0.66 [0.59;0.81]
Androstendion (nmol/l)	4.82 [3.38;5.63]	5.28 [3.26;5.63]	4.09 [3.59;5.69]	4.08 [3.06;4.76]	5.26 [3.52;6.15]
DHAS (nmol/l)	3238 [2455;4045]	3300 [2675;5065]	3229 [2134;3797]	3020 [2176;3883]	3360 [2955;4240]
Estrogen (ng/ml)	0.12 [0.09;0.16]	0.12 [0.09;0.15]	0.14 [0.07;0.17]	0.13 [0.12;0.18]	0.11 [0.07;0.16]
Free testosterone (nmol/l)	0.01 [0.01;0.02]	0.02 [0.01;0.02]	0.01 [0.01;0.02]	0.01 [0.01;0.01]	0.02 [0.01;0.02]
FSH (IU/l)	6.5 [5.7;8.1]	6.5 [5.92;8.1]	6.8 [5.2;7.87]	6.5 [5.85;7.75]	6.5 [5.7;8.4]
LH (IU/l)	4.15 [2.72;6.1]	4.15 [2.35;6.7]	4.5 [3.22;5.8]	3.4 [2.9;5.15]	4.9 [2.75;7.05]
Progesteron (ng/ml)	1.23 [0.84;1.48]	1.14 [0.74;1.47]	1.28 [1.18;1.48]	1.32 [0.96;1.55]	1.18 [0.77;1.36]
Prolactin (IU/l)	0.18 [0.13;0.24]	0.15 [0.12;0.19]	0.22 [0.18;0.27]	0.22 [0.18;0.26]	0.15 [0.11;0.21]
SHBG (nmol/l)	69 [60;88]	69 [59;87]	70 [63;89]	69 [56;83]	70 [61;97]
Total testosterone (nmol/l)	1.14 [0.82;1.34]	1.17 [0.82;1.34]	1.06 [0.82;1.3]	0.81 [0.51;1.19]	1.17 [0.91;1.46]
ACTH (µg/l)	22 [17;36]	21 [16;36]	24 [22;30]	15 [13;24]	23 [19;37]
BDNF (µg/l)	117 [78;164]	124 [90;160]	111 [64;168]	138 [112;163]	107 [72;163]
Cortisol (mmol/l)	470 [393;550]	478 [398;554]	458 [380;537]	424 [279;461]	510 [423;555]
GH (ng/ml)	4.2 [2.46;6.96]	4.36 [2.46;8.14]	3.76 [2.73;4.79]	2.9 [2.01;5.77]	4.73 [2.74;7.16]
Insulin (mIE/l)	3.25 [2.41;5.4]	3.7 [2.62;6.62]	2.7 [2.25;3.6]	2.7 [2.2;4.6]	3.3 [2.58;6.15]
Leptin (µg/ml)	2942 [1621;3875]	2151 [397;3549]	3170 [2915;4190]	3119 [1772;4988]	2608 [794;3524]
TSH (mIU/l)	2.16 [1.69;2.86]	2.23 [1.53;2.86]	2.1 [1.96;2.66]	2.05 [1.4;2.54]	2.18 [1.77;2.92]
BMD (kg/cm^2^)	1.11 [1.05;1.14]	1.1 [1.05;1.14]	1.12 [1.05;1.13]	1.13 [1.09;1.13]	1.09 [1.05;1.14]
BMI (kg/m^2^)	20.35 [19.21;21.37]	20.19 [18.83;21.19]	20.8 [20;21.52]	20.8 [20.29;22.03]	20.2 [18.85;21.04]
Fat mass (%)	20.25 [18.07;22.3]	19.7 [17.47;21.37]	21.35 [19.87;23.7]	20.8 [19.9;24.45]	19.8 [17.25;21.75]

Abbreviations: ACTH - adrenocorticotropic hormone; BDNF - brain-derived neurotrophic factor; BMD - bone mineral density; BMI - body mass index; DHAS - dehydroepiandrosterone; EA - energy availability; EUM - eumenorrhea; FHA - functional hypothalamic amenorrhea; FSH - follicle stimulating hormone; GH - growth hormone; HDL-C - high-density lipoprotein cholesterol; LDL-C - low-density lipoprotein cholesterol; LEA - low energy availability; LH - luteinizing hormone; SHBG - sex hormone binding globulin; TC - total cholesterol; TG - triglycerides; TSH - thyroid stimulating hormone.

The values shown are median values and interquartile ranges.

### Classification of lipidomic features

The 634 identified named lipid features were organized into four major categories. The first category, *Glycerolipids,* was comprised of diacylglycerols (DG) and triacylglycerols (TG). The second category, *Glycerophospholipids,* was comprised of lysophosphatidylcholines (LPC), phosphatidylcholines (PC), phosphatidylethanolamines (PE), phosphatidylglycerols (PG), phosphatidylinositols (PI), phosphatidylserines (PS) and phosphatidic acids (PA). The third category, *Sphingolipids,* was comprised of ceramides (Cer) and sphingomyelins (SM). The fourth category was termed *Miscellaneous,* and included the rest of the features including cholesteryl esters (CE), vitamins, and other named features.

### Association and prediction using lipidomics

First, linear regression (for the 24 continuous traits) and logistic regression models (for the two categorical traits, LEA and FHA) were employed to test associations with the correlation filtered lipidomic features (n = 216). In total, 5,616 (26 outcomes × 216 features) statistical tests were performed. After FDR correction, 20 statistically significant associations were detected, from which nine had annotations using the Steno Diabetes Center in-house library (Table 2) and eleven using LIPID MAPS (Supplementary Table 1). In this set of results, fat mass percentage, HDL-C, TC, free testosterone and cortisol demonstrated statistically significant associations with lipidomic features. We present spectral data for the pool samples for lipidomic features annotated using the Steno Diabetes Center in-house library (Supplementary Text 1).

**Table 2:** Associations between lipidomic features with Steno Diabetes Center in-house library annotations and clinical biomarkers at fasting state (N = 38).

Outcome	Lipidomic feature	β	SE	*P*	*P*_*FDR*_	Annotation type	m/z value	Retention time
Cortisol (mmol/l)	LPC(22:5)	81,63	17,83	5,42E-05	0,035	SDC in-house	570.36	3.80
Cortisol (mmol/l)	LPC(18:2)	77,08	18,39	1,71E-04	0,048	SDC in-house	520.34	3.79
Cortisol (mmol/l)	PC(42:6)	77,36	18,35	1,60E-04	0,048	SDC in-house	862.63	7.11
HDL-C (mmol/l)	SM(d38:1)	0,233	0,041	1,56E-06	0,009	SDC in-house	759.64	7.82
HDL-C (mmol/l)	SM(d32:1)	0,200	0,045	9,29E-05	0,041	SDC in-house	675.54	6.35
HDL-C (mmol/l)	PC(O-36:2)	0,200	0,045	9,38E-05	0,041	SDC in-house	772.62	7.58
TC (mmol/l)	SM(d33:1)	0,488	0,111	9,41E-05	0,041	SDC in-house	689.56	6.63
TC (mmol/l)	SM(d18:2/18:1)	0,482	0,112	1,18E-04	0,047	SDC in-house	727.57	6.48
TC (mmol/l)	CE(18:2) + Unknown CE(667.6219)	0,474	0,113	1,62E-04	0,048	SDC in-house	1315.20	9.72

Abbreviations: β - effect sizes from linear regression models; CE - cholesteryl ester; HDL-C - high-density lipoprotein cholesterol; LPC - lysophosphatidylcholine; m/z - mass-to-charge ratio; PC - phosphatidylcholine; SDC - Steno Diabetes Center; SE - standard error; SM - sphingomyelin; TC - total cholesterol.

*P* values were calculated using linear regression models. *P*_*FDR*_ values were adjusted using the Benjamini-Hochberg false discovery rate (FDR = 0.05). Annotation types are described in Materials and Methods.

**Figure 2:**
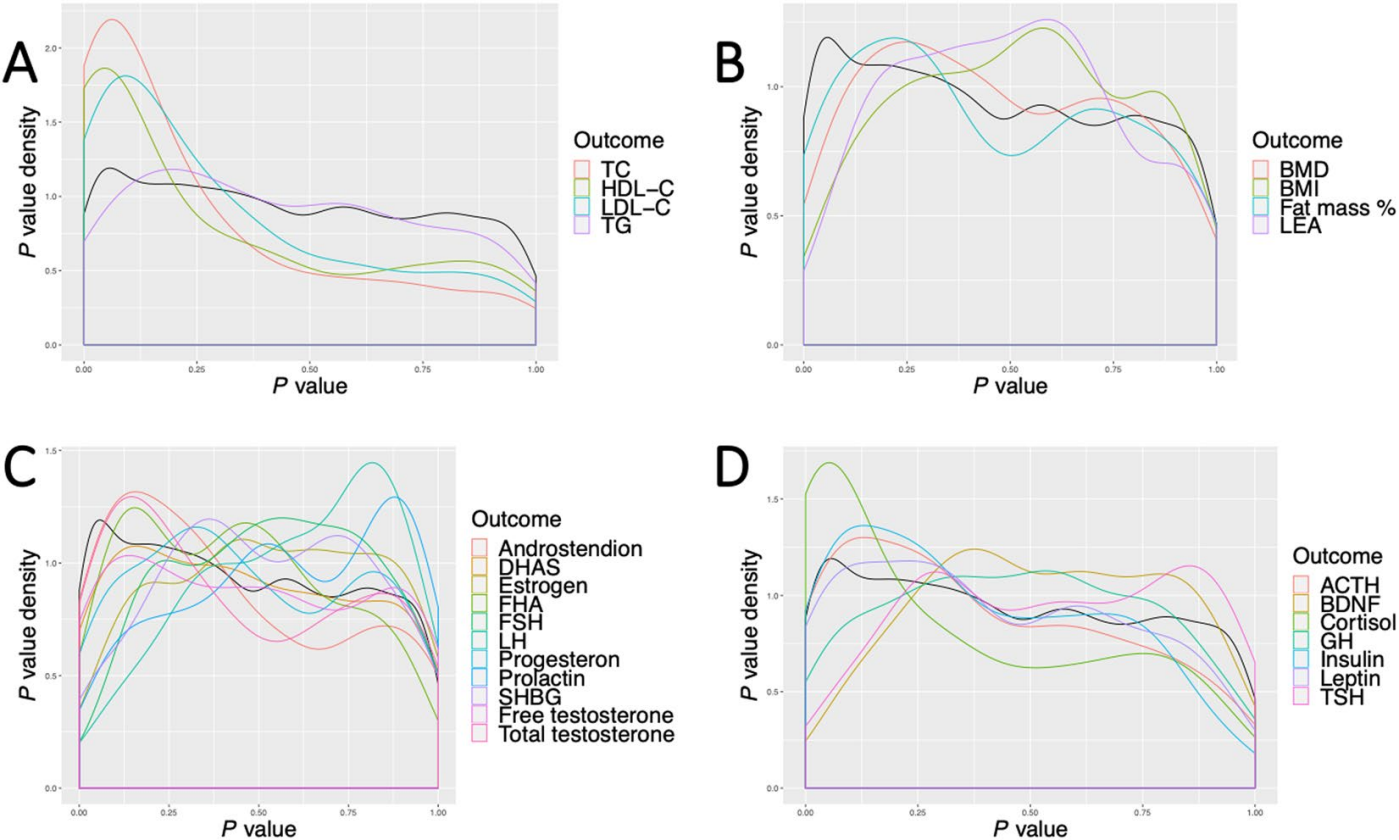
Density plots of *P* value distributions for all associations between 26 clinical biomarkers and 216 lipidomic features. The four panels demonstrate the *P* value distributions for the four main trait categories, (**A**) laboratory lipid traits, (**B**) energy-related traits, (**C**) sex hormones and (**D**) other hormones. *P* < 0.05 are overrepresented for HDL-C, LDL-C, TC and cortisol levels based on Fisher’s exact tests.

Second, overrepresentation of associations with *P* < 0.05 were tested for each sets of results for the 26 outcome traits using Fisher’s exact tests (Bonferroni-adjusted, *P* < 0.002). In this analysis, the 216 *P* values for a given outcome were compared to the rest of the 5,400 *P* values for all other outcomes. Four outcomes, HDL-C (*P*_*Fisher*_ = 2.9 × 10^−22^), LDL-C (*P*_*Fisher*_ = 4.2 × 10^−6^), TC (*P*_*Fisher*_ = 8.0 × 10^−19^) and cortisol (*P*_*Fisher*_ = 1.9 × 10^−14^) demonstrated overrepresentation of results with *P* < 0.05. All other traits did not demonstrate overrepresentation (*P* > 0.002). Density plots of distributions of *P* values for all outcomes are shown in Figure 2.

Third, the cumulative predictive utility of the scaled lipidomic features was assessed in relation to the 24 continuous outcomes (scaled to mean = 0 and standard deviation = 1 here) and the two categorical outcomes. In this analysis, explained variance and prediction error were recorded for numeric outcomes and prediction accuracy was recorded for categorical outcomes. The lipidomic profiles explained 41% of the variance in BMI levels, 14% of the variance of fat mass percentage, 10% of the variance of leptin levels and predicted BMI levels with a MAE of 0.4 standard deviations. For all other outcomes, the lipidomic features demonstrated poor predictive utilities with explained variances <5% and MAE > 0.5 standard deviations. The lipidomic features also demonstrated poor to mediocre predictive utilities for LEA (accuracy = 0.5) and FHA status (accuracy = 0.7).

### Clustering and lipid trajectories

**Figure 3:**
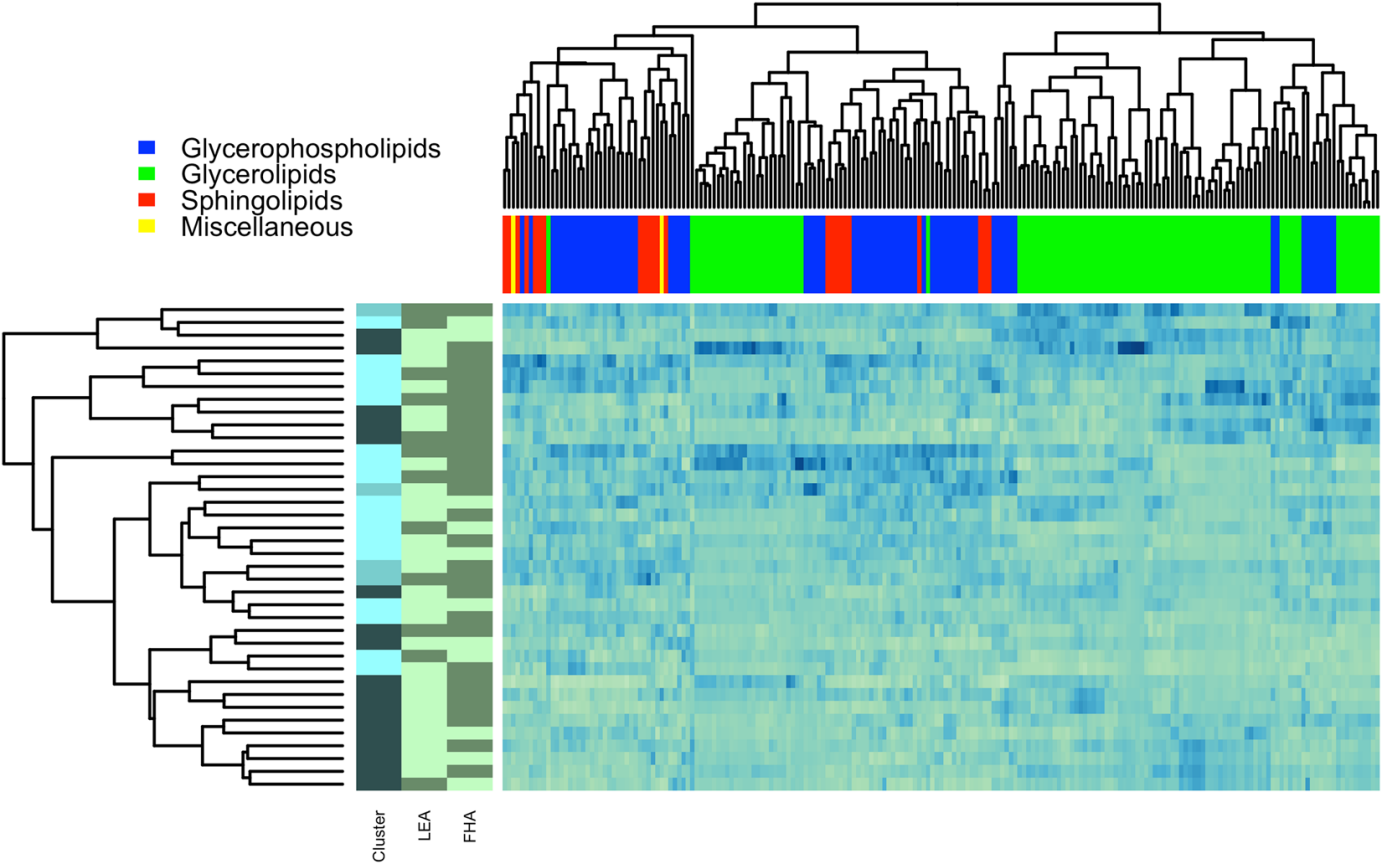
Heatmap and clustering of lipids and LEA participants. The heatmap visualizes 201 lipidomic features at fasting state in all LEA participants (N = 38). Lipids are categorized as *Glycerolipids*, *Glycerophospholipids*, *Sphingolipids* or *Miscellaneous*. The heatmap shows the hierarchical clustering dendrograms of 201 lipidomic features with Steno Diabetes Center in-house annotations and for the participants. The bars on the left side of the heatmap show LEA status, FHA status and the clustering based on SOM. In the LEA sidebar, the darker color reflects LEA status, while lighter color reflects sufficient EA. In the FHA sidebar, the darker color reflects FHA status, while lighter color reflects EUM.

Self-organizing maps were utilized to cluster individuals based on their lipidomic profiles. No correlation filtering was utilized before this analysis, therefore all 634 lipidomic features were used. The resulting clusters (as categorical variables) were subsequently tested for associations with LEA and FHA status using chi-squared tests. In these analyses, LEA status and the clusters at fasting state (three clusters identified) were nominally statistically significantly associated (*P* = 0.03). LEA and FHA status were not associated with the SOM clusters at any other timepoints (*P* > 0.05). A heatmap of the 201 lipidomic features annotated using the Steno Diabetes Center in-house library (higher confidence annotations) at fasting state, together with individual FHA status, LEA status, and SOM clusters are shown in Figure 3.

Trajectory clustering was undertaken using the *traj* R package based on 245 uncorrelated lipidomic features. The heatmap based on the results of the lipid trajectory clustering algorithm (Supplementary Figure 1) did not reveal an informative internal data structure.

**Figure 4:**
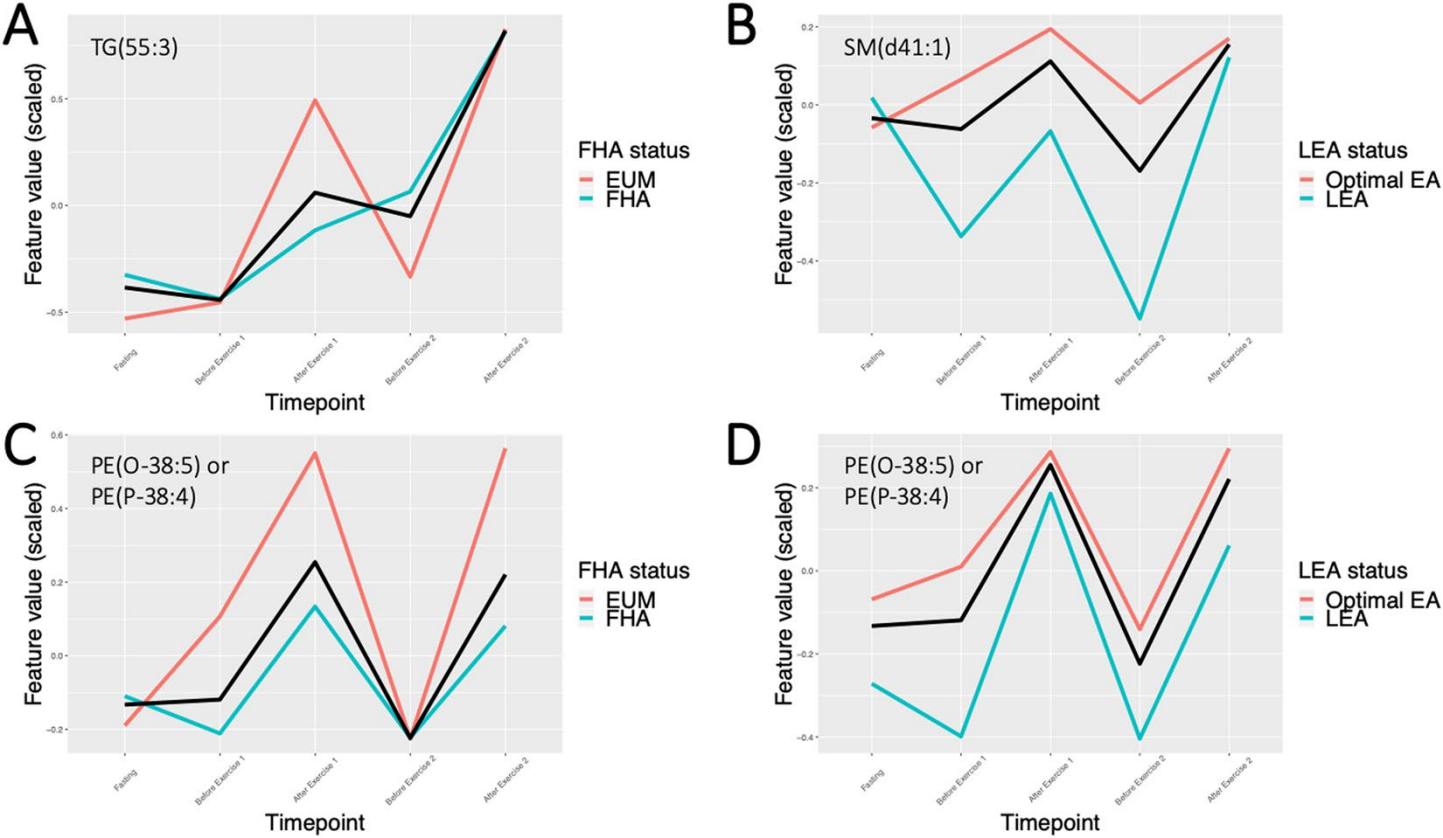
Lipid trajectories of TG(55:3), SM(d41:1) and PE(O-38:5) or PE(P-38:4). The figures show mean trajectories (using locally estimated scatterplot smoothing) for all LEA participants (black line), participants with sufficient EA/eumenorrhea (red) and LEA/FHA (cyan). Panel (**A**) shows trajectories for TG(55:3) stratified by FHA status. Panel (**B**) shows trajectories for SM(d41:1) stratified by LEA status. Panels (**C,D**) show trajectories for PE(O-38:5) or PE(P-38:4) stratified by FHA and LEA status, respectively.

Visual presentation of the lipidomics trajectories for 201 lipids annotated using the Steno Diabetes Center in-house library over the five timepoints for all participants, and stratified by LEA and FHA status, organized by lipid classes, are available at Supplementary Text 2. This browsable material serves as a resource to generate hypotheses on candidate lipidomic features in future studies. The visual inspection of the stratified mean lipid trajectories showed interesting patterns for a number of features. Multiple triglycerides with long-chain fatty acids, for instance TG(47:1), TG(51:1), or TG(55:3), demonstrate a pattern reactive to the exercise interventions in participants with eumenorrhea, while participants with FHA demonstrate markedly different patterns, for instance a steady increase throughout the study day (Figure 4A). SM(d41:1), a sphingomyelin, demonstrates trajectories differing in magnitude between individuals with LEA and sufficient EA status (Figure 4B). Another example is PE(O-38:5) or PE(P-38:4), a phosphatidylethanolamine, where participants with eumenorrhea and with sufficient EA status show a trajectory reactive to the exercise interventions. Here, while individuals with FHA appear to demonstrate a blunted response to the interventions, individuals with LEA show a similar reactive trajectory, however mean levels are considerably lower compared to those with sufficient EA (Figure 4C,D).

## Discussion

In this study, fasting lipidomic profiles and acute adaptations of lipidomic features to standardized exercise tests were investigated in female elite athletes at risk of RED-S. The focus of this investigation was: (i) to assess whether lipidomic features associate with clinical biomarkers at fasting state and (ii) test whether trajectories of lipidomic features could provide information on metabolic responses to intensive exercise.

Previous investigations showed that unhealthful lipid profiles associate with RED-S comorbidities such as menstrual dysfunction^7^ and BMD^6^ in female athletes. Here, we investigated whether the lipidome, which offers a higher resolution of lipid profiles compared to standard laboratory lipid assessments, would associate with a wide range of RED-S comorbidities. Such comorbidities included lipid, hormonal, anthropometric and energy-related clinical biomarkers. In total, 20 associations were detected at fasting and four traits showed overrepresentation of statistically significant associations. While the associations with standard laboratory lipids, such as TC, HDL-C and LDL-C cannot be considered surprising, it is noteworthy that cortisol levels positively associated with seven features and also showed a general overrepresentation of statistically significant findings. It has been shown that cortisol levels are chronically mildly elevated in elite athletes, and that FHA status is shown to be associated with higher levels of fasting serum cortisol levels^4^. Thus, we hypothesize that the LPC and PC features associated with cortisol might serve as diagnostic proxies for a metabolically altered state resulting from RED-S, and are likely correlates of RED-S severity.

The study of acute and long-term trajectories of lipidomic profiles has the ability to reveal metabolic adaptations that would normally remain hidden, as standard clinical biomarkers cannot provide the data resolution achieved by various omics platforms^23,24^. Acute changes of metabolomic profiles (lipids and amino acids) in response to various metabolic stressors have been tested in smaller studies with strict protocols^5,25–27^, whereas long-term adaptations have been studied in larger epidemiological studies^28,29^. These studies showcase that complex metabolic profiles are highly adaptive and demonstrate marked differences between various groups of individuals, for instance between males and females^28,29^.

The authors acknowledge that the individual lipidomic feature trajectories stratified by LEA and FHA status, due to the low sample size, are exploratory in nature. While no obvious clusters could be identified using the trajectory clustering algorithm, the presented individual lipidomic trajectories serve as a resource for future investigations which seek candidate biomarkers showing promising clinical or predictive utility, based on lipid trajectory differences between individuals stratified by menstrual dysfunction and EA. The visual inspection of all plots revealed a number of lipidomic features, belonging to various biological subclasses (e.g. LPC, PC, SM, TG), demonstrating tentative evidence for lipid trajectory differences between individuals stratified by FHA status. Lipid trajectory differences between individuals with sufficient EA and LEA were lower in numbers. These results tentatively indicate that, in this cohort of female elite athletes, FHA status is associated with an altered ability for metabolic adaptation to exercise interventions.

Our study has a number of important strengths. First, deep phenotyping was undertaken, i.e. a wide range of lipid, hormonal, anthropometric, and energy-related biomarkers were assessed together with lipidomic profiles. Second, this study is prospective in nature; the participants underwent a day-long protocol and lipidomic profiles were obtained at five different timepoints throughout the day, at fasting state, and before and after two exercise sessions^3^. This study design and data collection presents a rare opportunity to study adaptive, acute changes in metabolic profiles. Third, a robust statistical framework used biomarkers at fasting state, along with the longitudinal aspect of the study, the lipid trajectories. Fourth, all participants adhered to a strict study protocol with standardized exercise interventions. Fifth, objective measurements of anthropometric and energy-related traits and reproductive function (e.g. the thorough classification of menstrual functions) were available. Sixth, we provide reference trajectories for 201 lipidomic features, which may provide interesting candidate features for future investigations.

The most obvious limitation of this project is the low sample size. The authors acknowledge that due to the small number of participants, findings from this study are exploratory in nature, and larger cohorts must be utilized for validation. However, as expressed above, due to challenges related to establishing studies with standardized prospective designs with high-frequency sampling and deep phenotyping, our sample size is not unusual. Indeed, previous studies that demonstrated the dynamic nature of metabolomic adaptations in response to prolonged fasting, cold stress^5^, exercise^27^, meal challenge^26^ and oral glucose tolerance tests^25^ had lower sample sizes and lower number of metabolites assessed. To ameliorate the challenges posed by low sample size, we utilized statistical approaches appropriate for smaller samples sizes, such as presenting nonparametric test statistics and using LOOCV for prediction analyses. A high number of statistical tests were undertaken, which can result in increased type 1 error rates. We addressed this by applying multiple testing corrections, Benjamini-Hochberg FDR for lipidomics associations and the more conservative Bonferroni correction for other comparisons. We acknowledge that annotations obtained by LIPID MAPS yielded a number of metabolites that are unlikely to be present in human serum samples. Thus, when presenting our results, we strived to primarily present findings with higher confidence annotations obtained by our in-house library, all confirmed to be present in human blood. Last, we acknowledge that LEA is a highly specific cohort; while female elite athletes at risk for RED-S represent an interesting population to study, findings from this project are likely to have limited generalizability to different populations, for instance males at risk for RED-S or for non-athlete females.

In summary, we demonstrate associations between a number of lipidomic features and cortisol and standard laboratory lipids while in a fasting state. We present reference lipid trajectories for hundreds of lipidomic features, many of which demonstrate distinct patterns for participants with menstrual dysfunction or undernourishment.

## Supplementary Material

Supplementary information.

Supplementary information 2.

Supplementary information 3.

Supplementary information 4.
