# Supplementary material for: Lipidomic profiles, lipid trajectories and clinical biomarkers in female elite endurance athletes
Source: Sci Rep. 2020 Feb 11;10:2349. doi: 10.1038/s41598-020-59127-8 (PMC7012926; doi:10.1038/s41598-020-59127-8)
Supplement: Supplementary file 1 — Supplementary information. [file 41598_2020_59127_MOESM1_ESM.pdf]

SUPPLEMENTARY TEXT 2

Lipid trajectories of 201 individual lipidomics features

Feature	Page
GLYCEROLIPIDS	
TG(14:0/16:0/18:1)	10
TG(14:0/18:1/18:1)	13
TG(14:0/18:2/18:2)	16
TG(16:0/18:0/18:1)	19
TG(16:0/18:2/18:2)	22
TG(16:0/18:2/18:3)	25
TG(16:0/18:2/22:6)	28
TG(16:0/22:5/18:1) or TG(20:4/18:1/18:1)	31
TG(18:0/18:1/20:4)	34
TG(18:1/12:0/18:1) or TG(18:2/16:0/14:0)	37
TG(18:1/18:1/16:0)	40
TG(18:1/18:1/18:1)	43
TG(18:1/18:1/22:6)	46
TG(18:1/18:2/18:2)	49
TG(18:2/18:1/16:0)	52
TG(18:2/18:1/18:1)	55
TG(18:2/18:2/18:2) or TG(18:3/18:2/18:1)	58
TG(18:2/22:5/16:0)	61
TG(40:0)	64
TG(40:1)	67
TG(42:0)	70
TG(42:1)	73
TG(42:2)	76
TG(44:1)	79
TG(44:2)	82
TG(45:0)	85
TG(46:0)	88
TG(46:1)	91
TG(46:2)	94

TG(47:1)	97
TG(47:2)	100
TG(48:1)	103
TG(49:0)	106
TG(49:1)	109
TG(49:2)	112
TG(49:3)	115
TG(50:0)	118
TG(50:0).1	121
TG(50:1)	124
TG(50:2)	127
TG(50:3)	130
TG(50:3).1	133
TG(50:4)	136
TG(50:5)	139
TG(51:1)	142
TG(51:2)	145
TG(51:2).1	148
TG(51:3)	151
TG(51:4)	154
TG(52:0)	157
TG(52:1)	160
TG(52:2)	163
TG(52:3)	166
TG(52:4)	169
TG(52:5)	172
TG(52:6)	175
TG(52:7)	178
TG(53:1)	181
TG(53:2)	184
TG(53:2).1	187

TG(53:3)	190
TG(53:4)	193
TG(53:5)	196
TG(54:1)	199
TG(54:2)	202
TG(54:3)	205
TG(54:4)	208
TG(54:5)	211
TG(54:6)	214
TG(54:7)	217
TG(54:8)	220
TG(55:2)	223
TG(55:3)	226
TG(55:4)	229
TG(55:5)	232
TG(55:6)	235
TG(55:7)	238
TG(56:1)	241
TG(56:2)	244
TG(56:3)	247
TG(56:4)	250
TG(56:5)	253
TG(56:6)	256
TG(56:7)	259
TG(56:8)	262
TG(56:9)	265
TG(58:10)	268
TG(58:11)	271
TG(58:3)	274
TG(58:4)	277
TG(58:5)	280

TG(58:6)	283
TG(58:7)	286
TG(58:8)	289
TG(58:9)	292
TG(60:10)	295
TG(60:11)	298
TG(60:12)	301
TG(60:7)	304
TG(60:9)	307
TG(62:13)	310
GLYCEROPHOSPHOLIPIDS	
LPC(16:0e)	314
LPC(16:0p)	317
LPC(16:1)	320
LPC(18:1)	323
LPC(18:2)	326
LPC(20:3)	329
LPC(20:4)	332
LPC(22:5)	335
LPC(22:6)	338
PC(30:0)	341
PC(31:1)	344
PC(32:0)	347
PC(32:1)	350
PC(32:2)	353
PC(33:1)	356
PC(34:1)	359
PC(34:2)	362
PC(34:3)	365
PC(35:1)	368
PC(35:2)	371

PC(35:3)	374
PC(35:4)	377
PC(36:1)	380
PC(36:2)	383
PC(36:3)	386
PC(36:4)	389
PC(36:5)	392
PC(36:6)	395
PC(37:1)	398
PC(37:2)	401
PC(37:3)	404
PC(37:4)	407
PC(37:6)	410
PC(38:1)	413
PC(38:2)	416
PC(38:3)	419
PC(38:4)	422
PC(38:5)	425
PC(38:6)	428
PC(38:7)	431
PC(40:3)	434
PC(40:4)	437
PC(40:5)	440
PC(40:6)	443
PC(40:7)	446
PC(40:8)	449
PC(42:5)	452
PC(42:6)	455
PC(O-20:0/22:4)	458
PC(O-20:0/22:6)	461
PC(O-22:2/22:3)	464

PC(O-32:0)	467
PC(O-32:1)	470
PC(O-34:2)	473
PC(O-34:3)	476
PC(O-36:2)	479
PC(O-36:3)	482
PC(O-36:4)	485
PC(O-36:5)	488
PC(O-38:3) or PC(P-38:2)	491
PC(O-38:4)	494
PC(O-38:5)	497
PC(O-38:6)	500
PC(O-40:4)	503
PC(O-40:6)	506
PC(O-42:3)	509
PC(P-18:0/22:6)	512
PC(P-20:0/22:4)	515
PE(34:2)	518
PE(36:4)	521
PE(38:4)	524
PE(38:6)	527
PE(O-16:0/22:6) or PE(P-18:0/20:5)	530
PE(O-38:5) or PE(P-38:4)	533
PE(P-18:0/22:6)	536
SPHINGOLIPIDS	
SM(42:2)	540
SM(d16:1/18:1) or SM(d18:2/16:0)	543
SM(d18:0/14:0)	546
SM(d18:1/12:0)	549
SM(d18:1/24:0) or SM(d18:0/24:1)	552
SM(d18:1/24:0)	555

SM(d18:1/26:1)	558
SM(d18:2/14:0)	561
SM(d18:2/18:1)	564
SM(d18:2/24:1)	567
SM(d32:1)	570
SM(d33:1)	573
SM(d34:1)	576
SM(d36:1)	579
SM(d36:2)	582
SM(d38:0)	585
SM(d38:1)	588
SM(d38:2)	591
SM(d39:1)	594
SM(d40:1)	597
SM(d40:2)	600
SM(d41:1)	603
SM(d41:2)	606
MISCELLANEOUS	
CE(18:2) + Unknown CE(667.6219)	610
Fragment: CE signature ion	613

GLYCEROLIPIDS

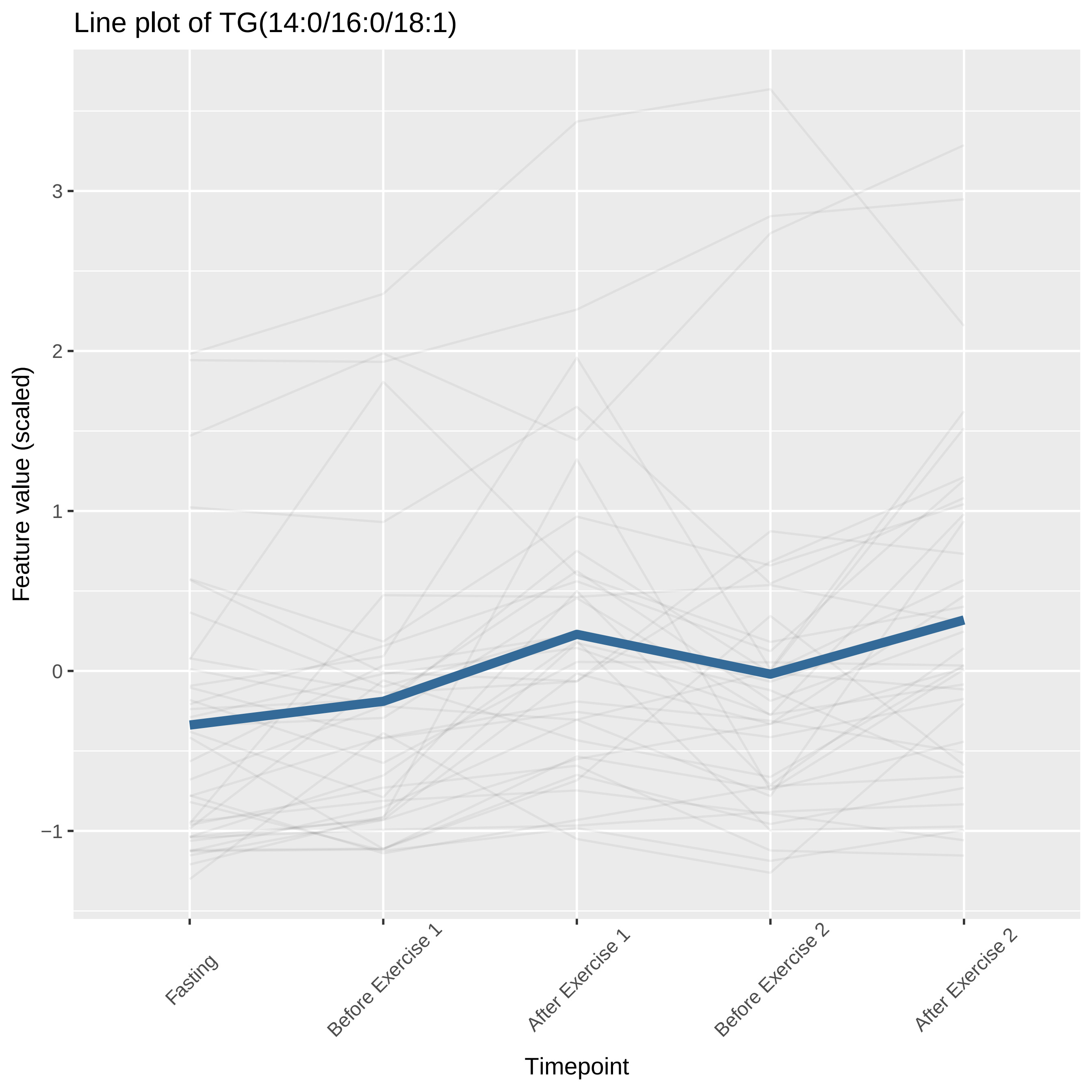

Line plot of TG(14:0/16:0/18:1)
Feature value (scaled)
3
2
1
0
−1
Fasting
Before Exercise 1
After Exercise 1
Before Exercise 2
After Exercise 2
Timepoint

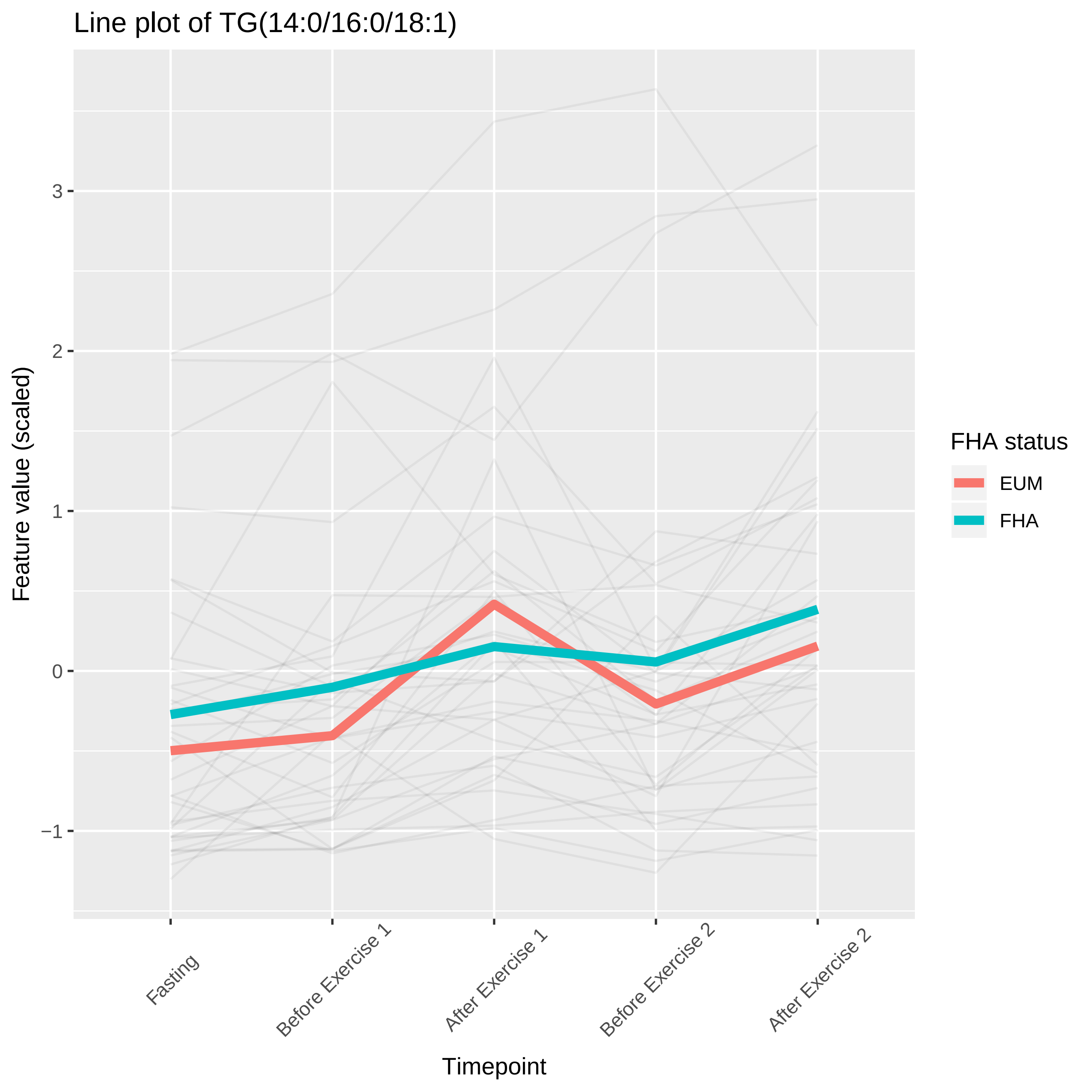

Line plot of TG(14:0/16:0/18:1)
Feature value (scaled)
3
2
1
0
−1
Fasting
Before Exercise 1
After Exercise 1
Before Exercise 2
After Exercise 2
Timepoint
FHA status
EUM
FHA

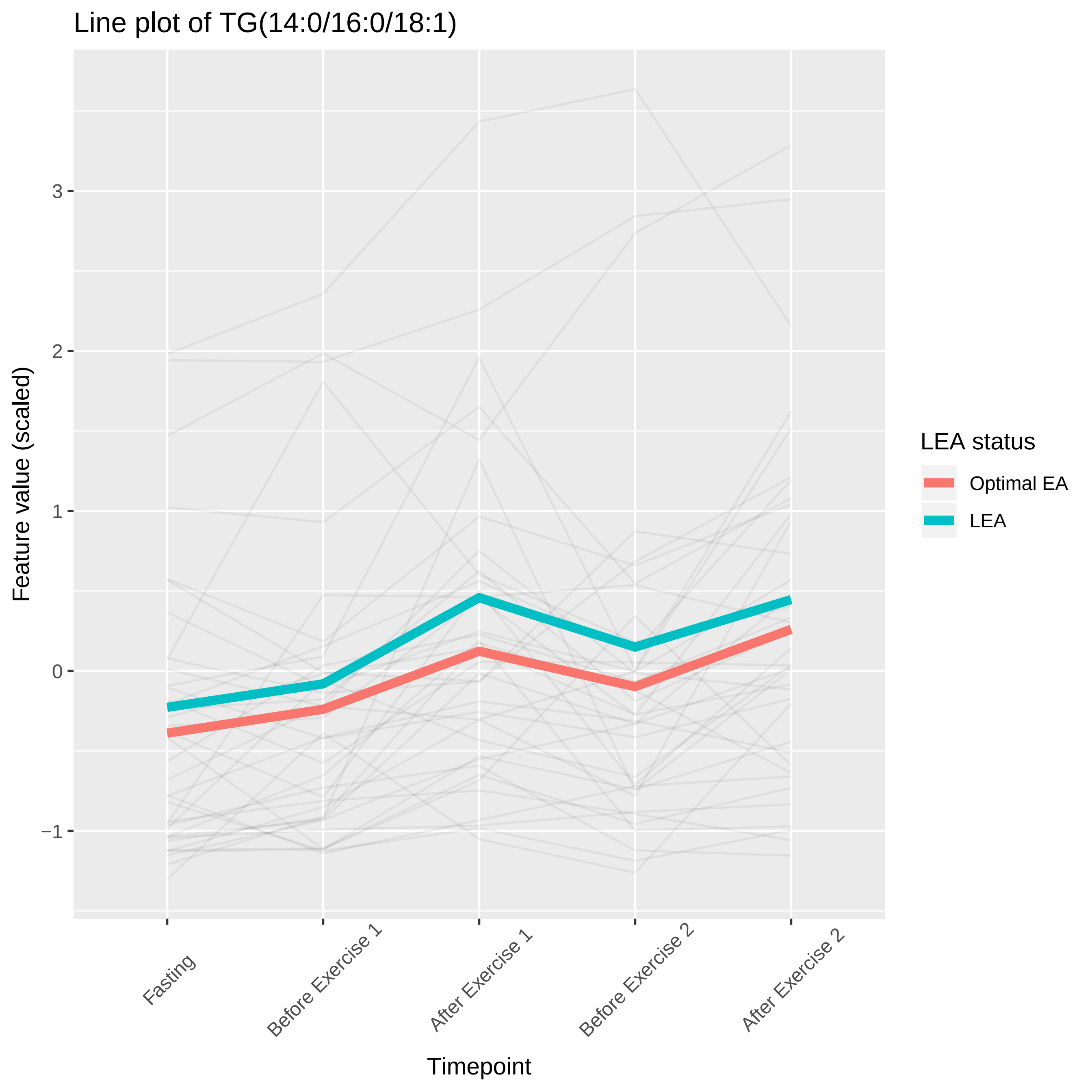
Line plot of TG(14:0/16:0/18:1)
Feature value (scaled)
3
2
1
0
−1
Fasting
Before Exercise 1
After Exercise 1
Before Exercise 2
After Exercise 2
Timepoint
LEA status
Optimal EA
LEA

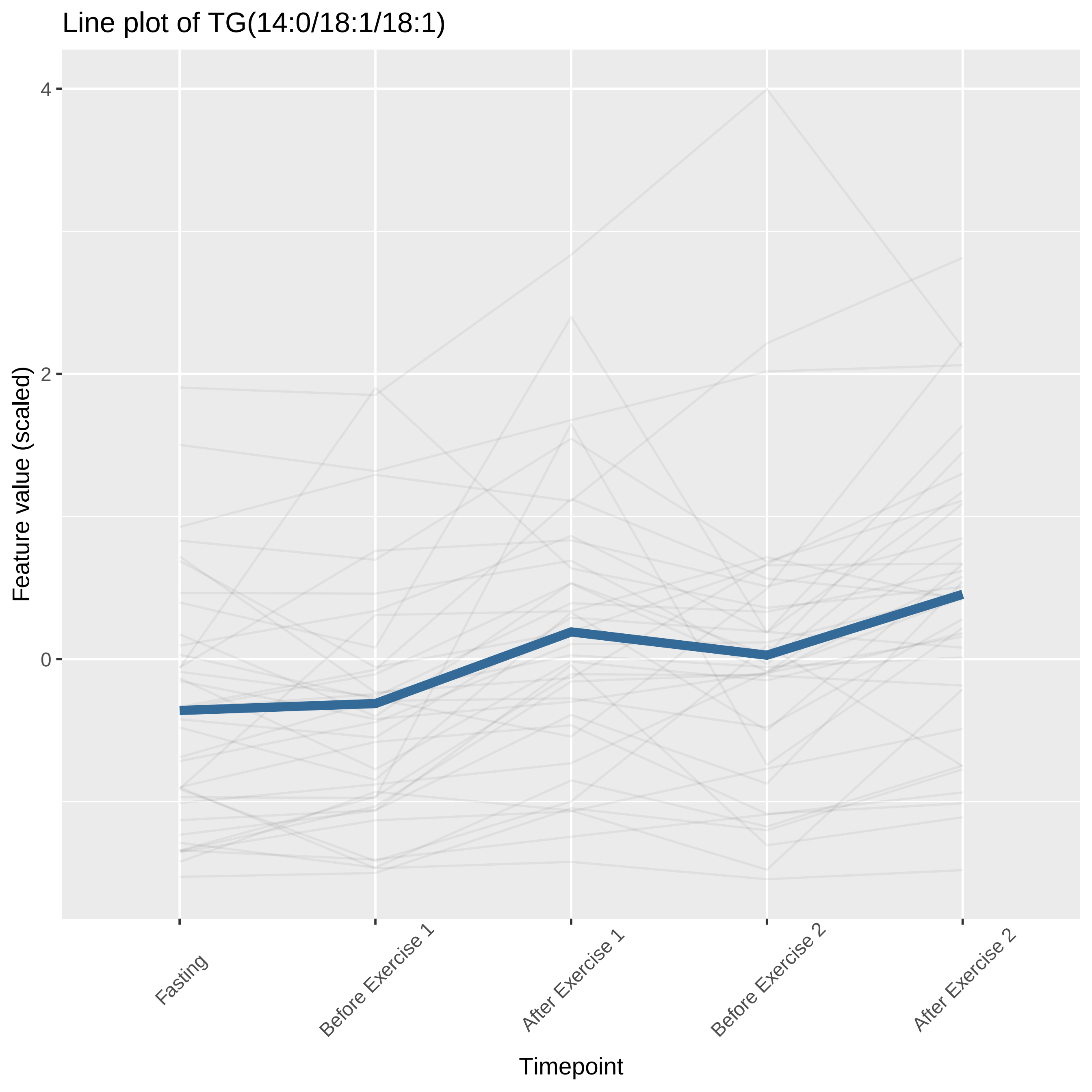

Line plot of TG(14:0/18:1/18:1)
Feature value (scaled)
4
2
0
Fasting
Before Exercise 1
After Exercise 1
Before Exercise 2
After Exercise 2
Timepoint

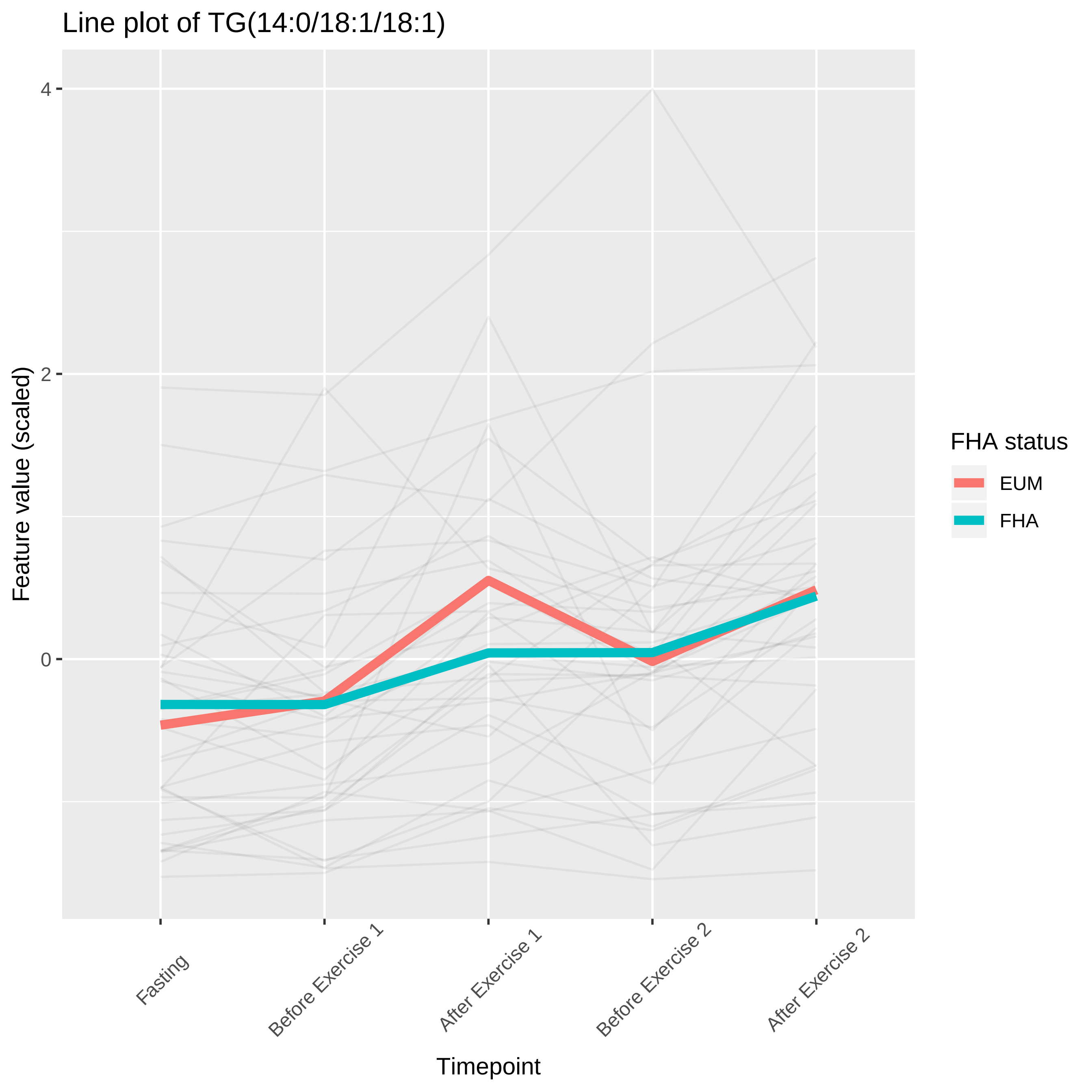

Line plot of TG(14:0/18:1/18:1)
Feature value (scaled)
4
2
0
Fasting
Before Exercise 1
After Exercise 1
Before Exercise 2
After Exercise 2
Timepoint
FHA status
EUM
FHA

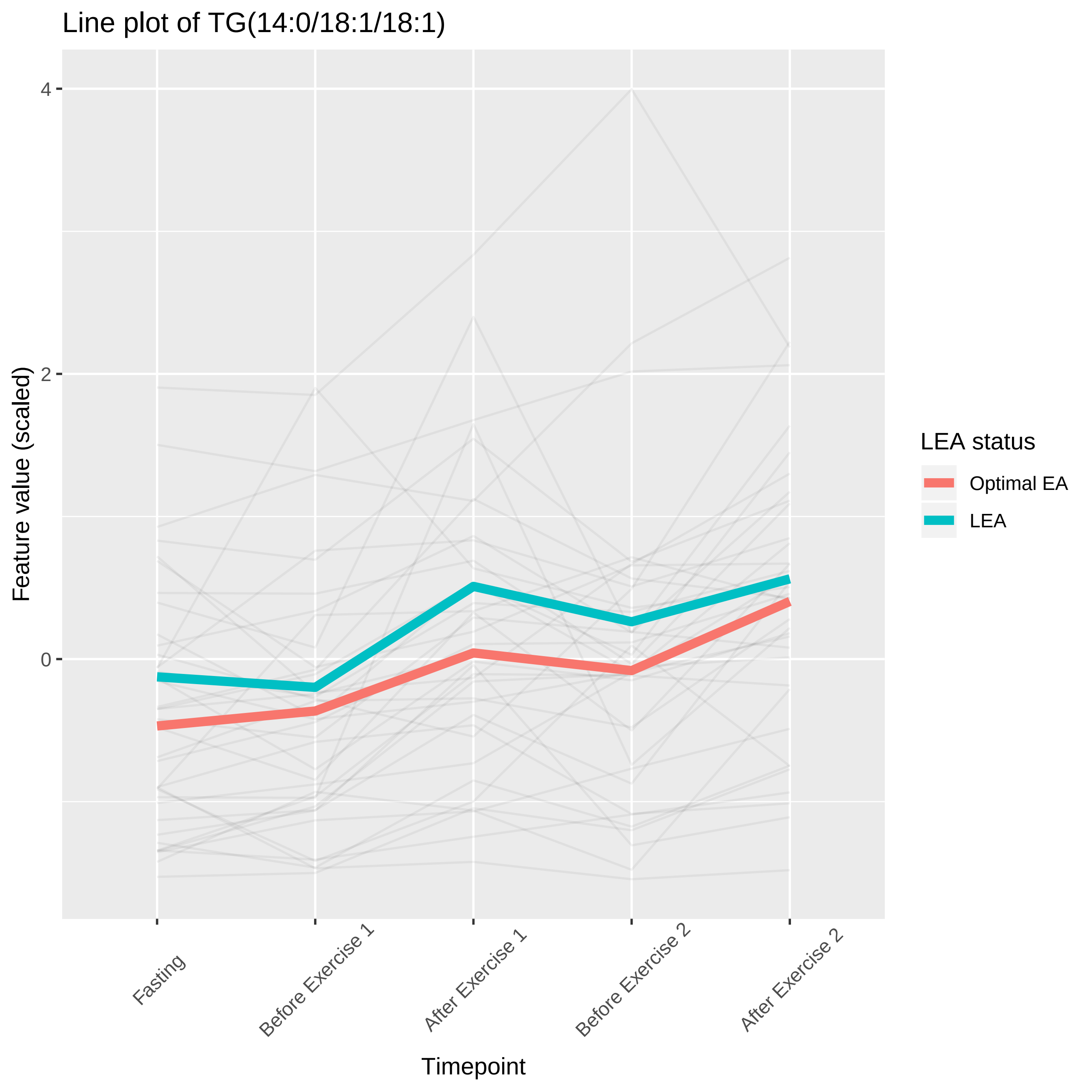

Line plot of TG(14:0/18:1/18:1)
Feature value (scaled)
4
2
0
Fasting
Before Exercise 1
After Exercise 1
Before Exercise 2
After Exercise 2
Timepoint
LEA status
Optimal EA
LEA

Line plot of TG(14:0/18:2/18:2)

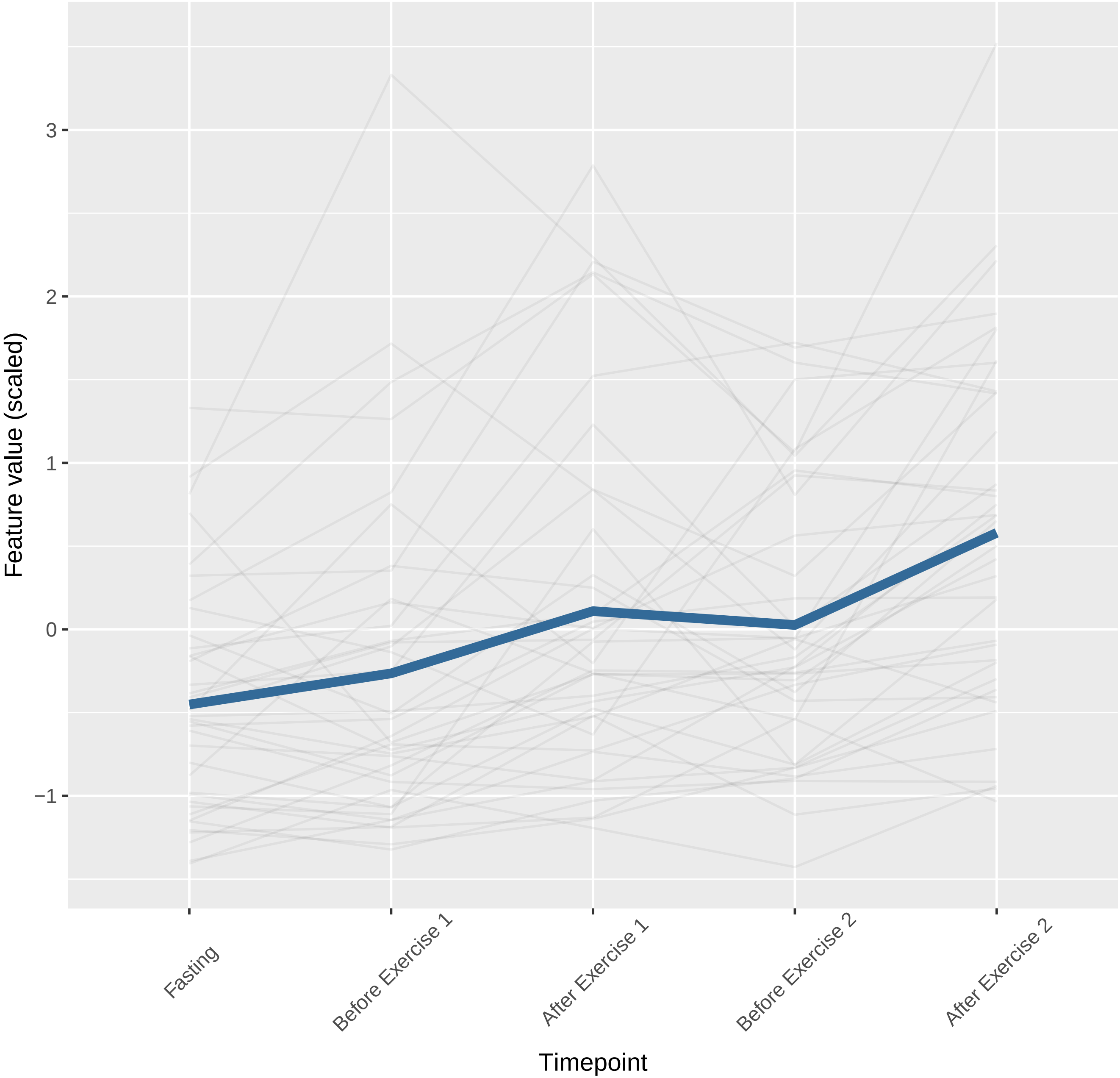

Line plot of TG(14:0/18:2/18:2)

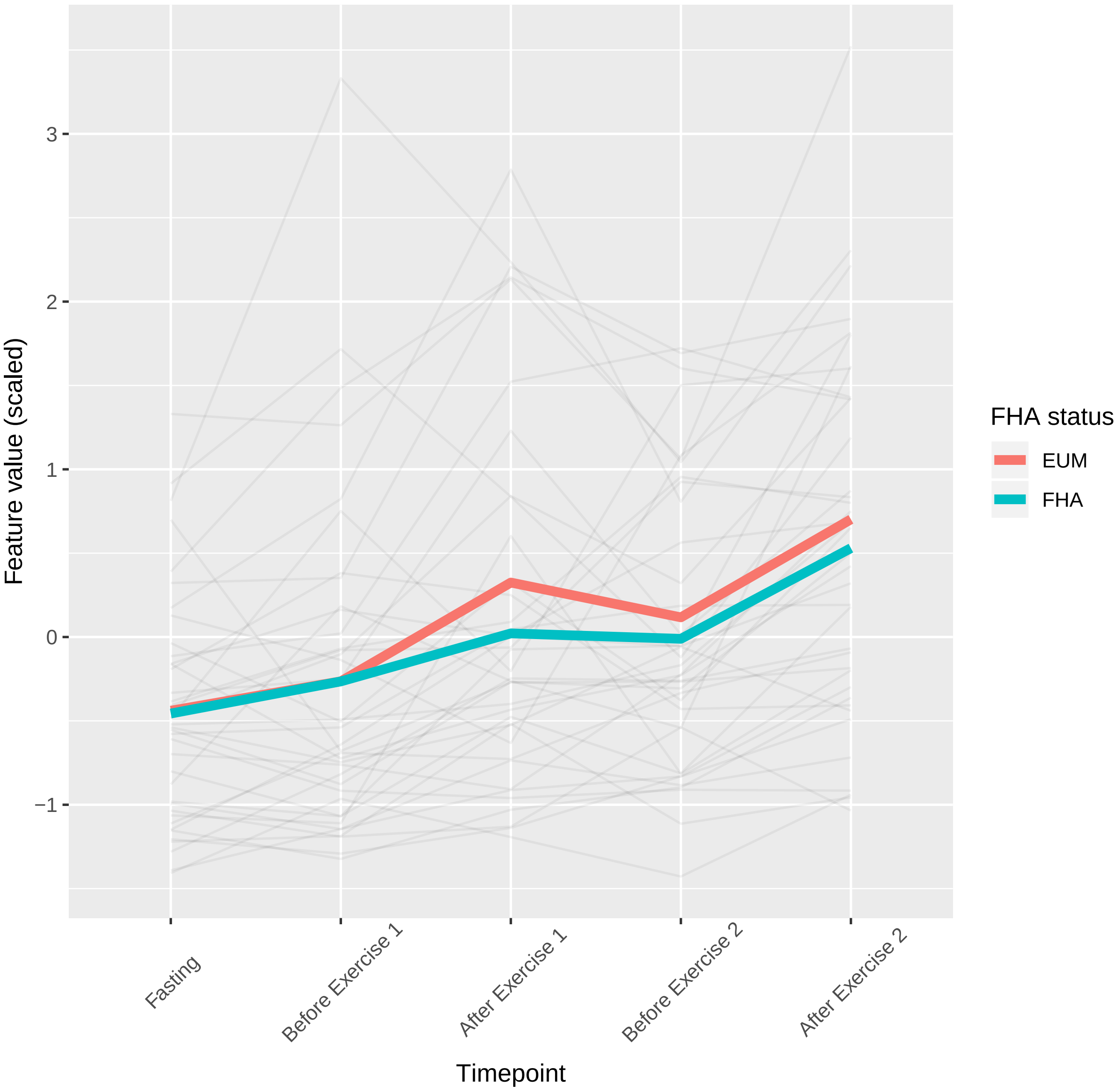

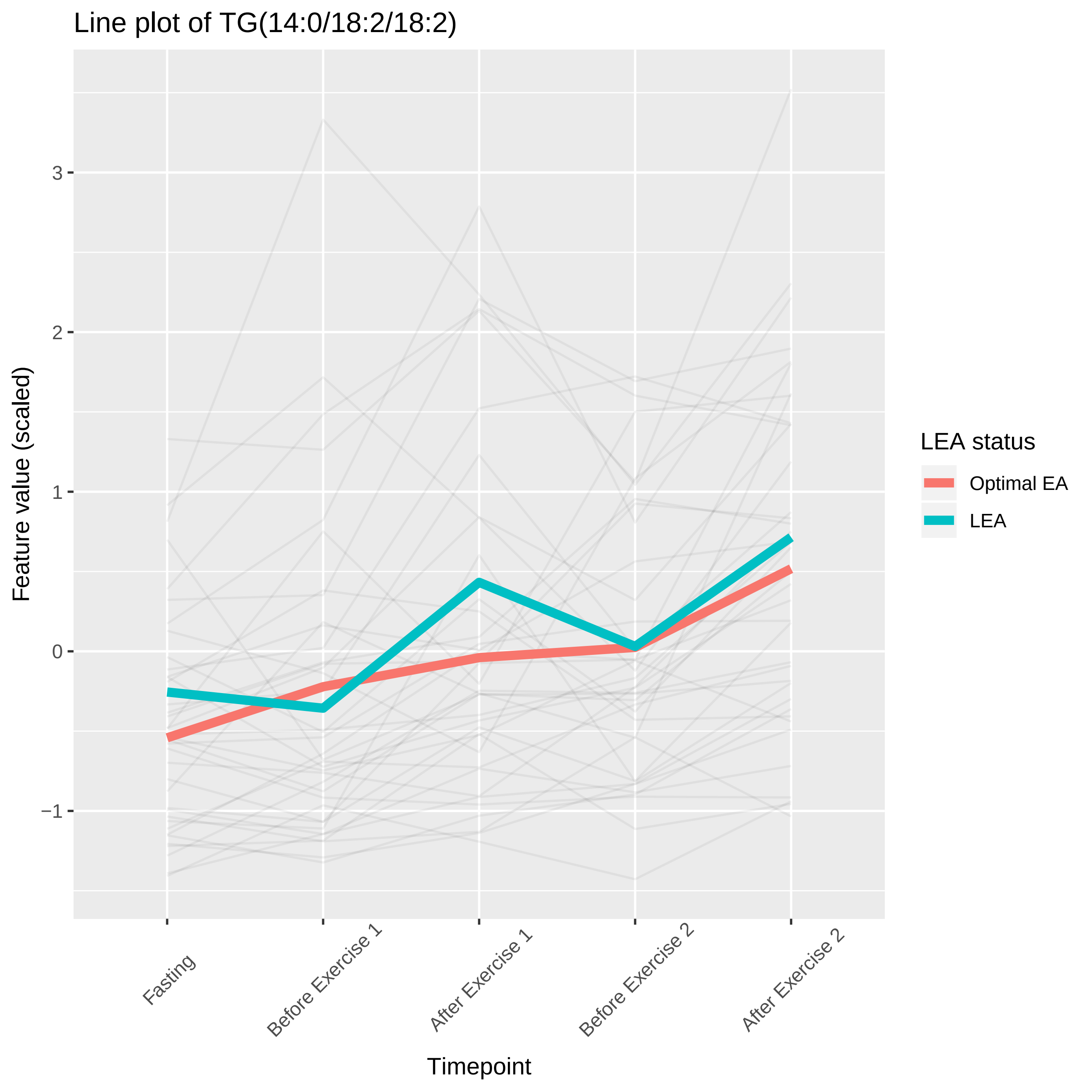
Line plot of TG(14:0/18:2/18:2)
Feature value (scaled)
3
2
1
0
−1
Fasting
Before Exercise 1
After Exercise 1
Before Exercise 2
After Exercise 2
Timepoint
LEA status
Optimal EA
LEA

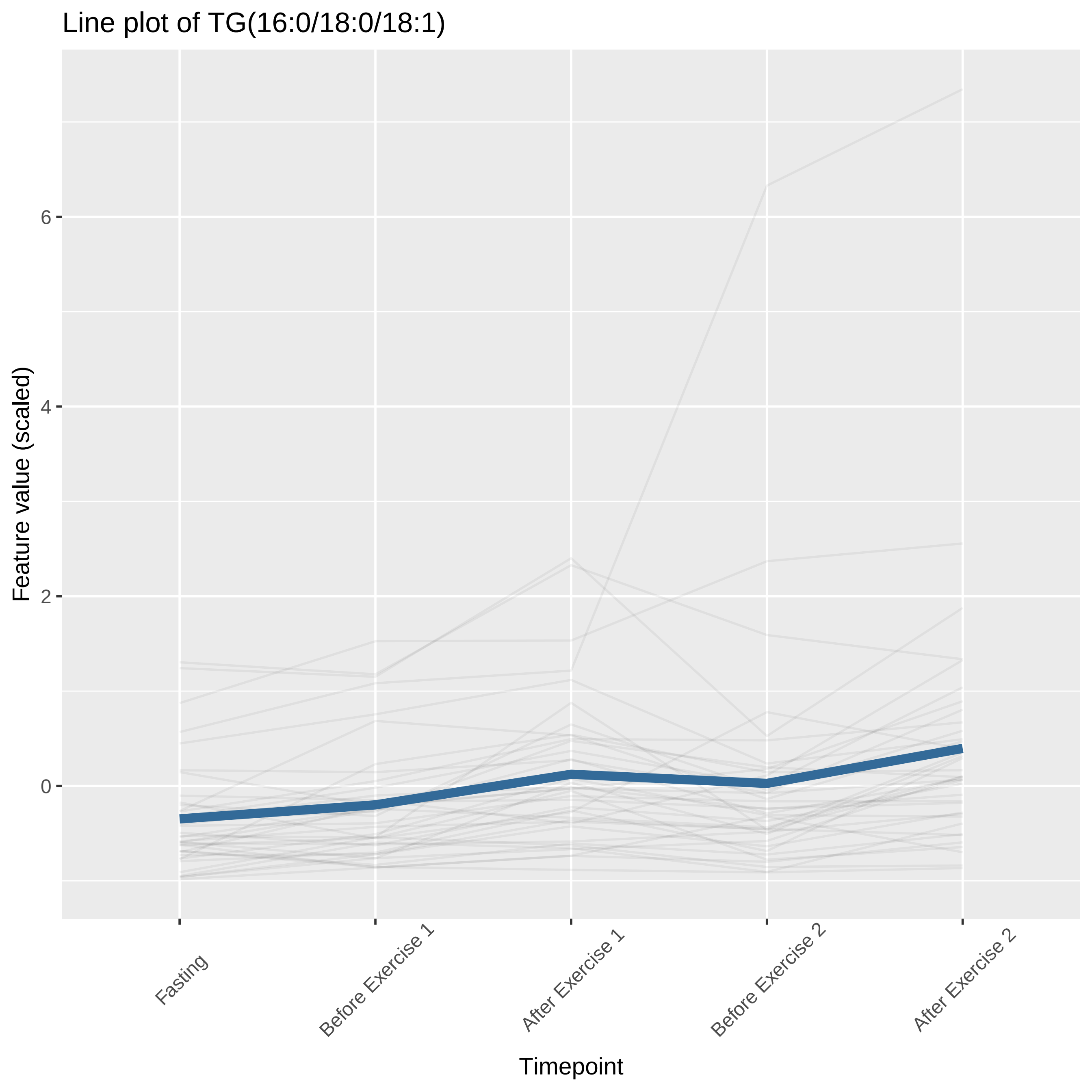

Line plot of TG(16:0/18:0/18:1)
Feature value (scaled)
6
4
2
0
Fasting
Before Exercise 1
After Exercise 1
Before Exercise 2
After Exercise 2
Timepoint

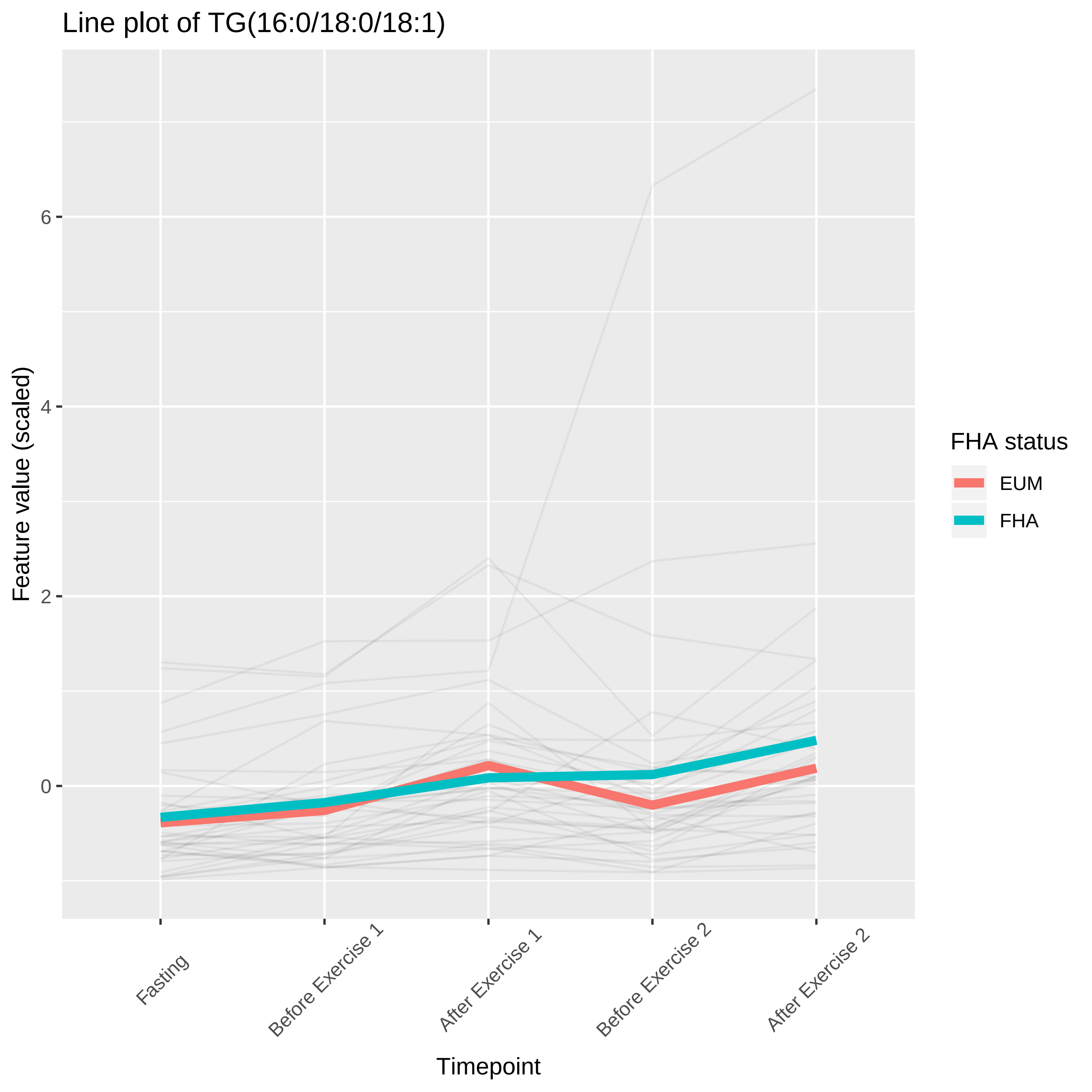

Line plot of TG(16:0/18:0/18:1)
Feature value (scaled)
6
4
2
0
Fasting
Before Exercise 1
After Exercise 1
Before Exercise 2
After Exercise 2
Timepoint
FHA status
EUM
FHA

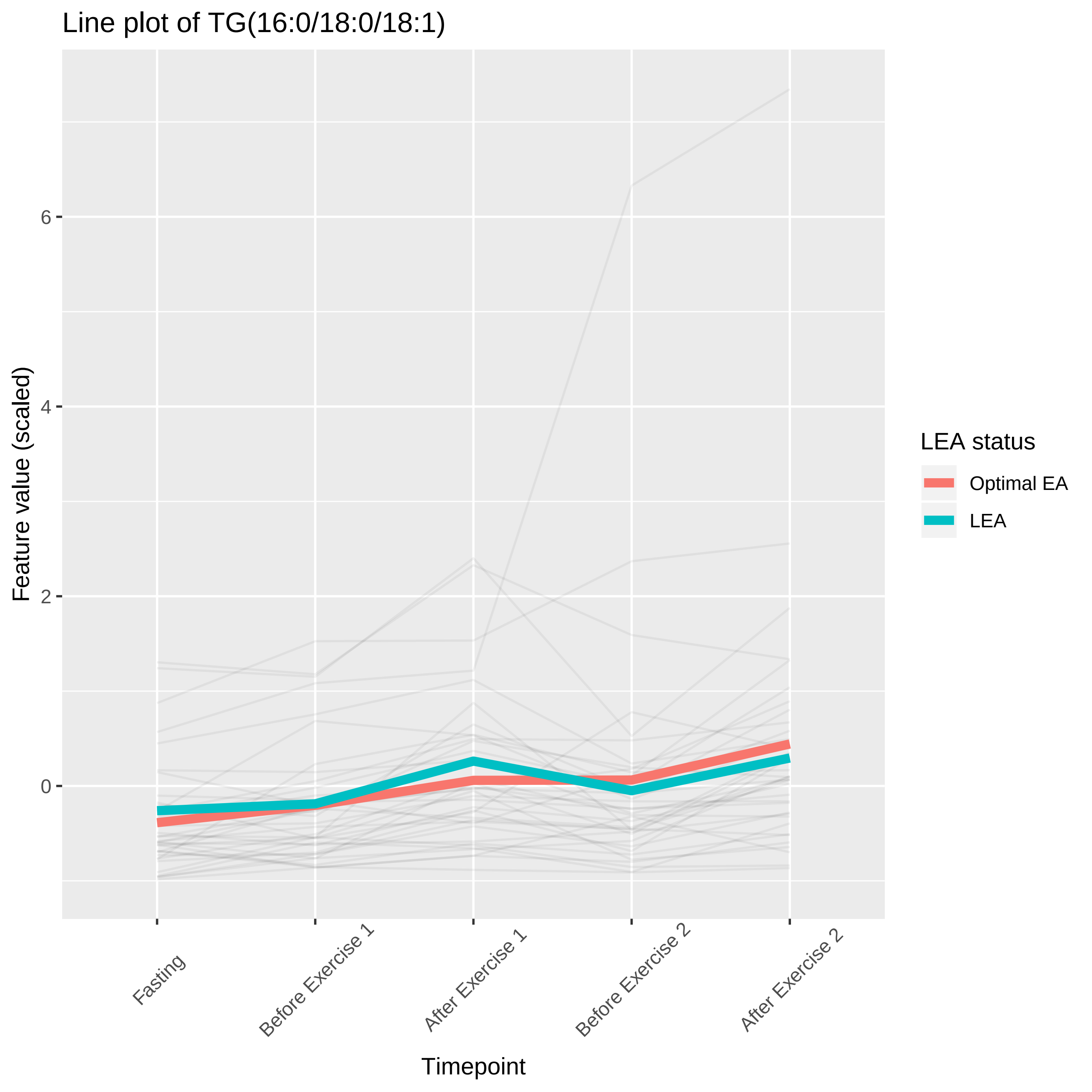

Line plot of TG(16:0/18:0/18:1)
Feature value (scaled)
6
4
2
0
Fasting
Before Exercise 1
After Exercise 1
Before Exercise 2
After Exercise 2
Timepoint
LEA status
Optimal EA
LEA

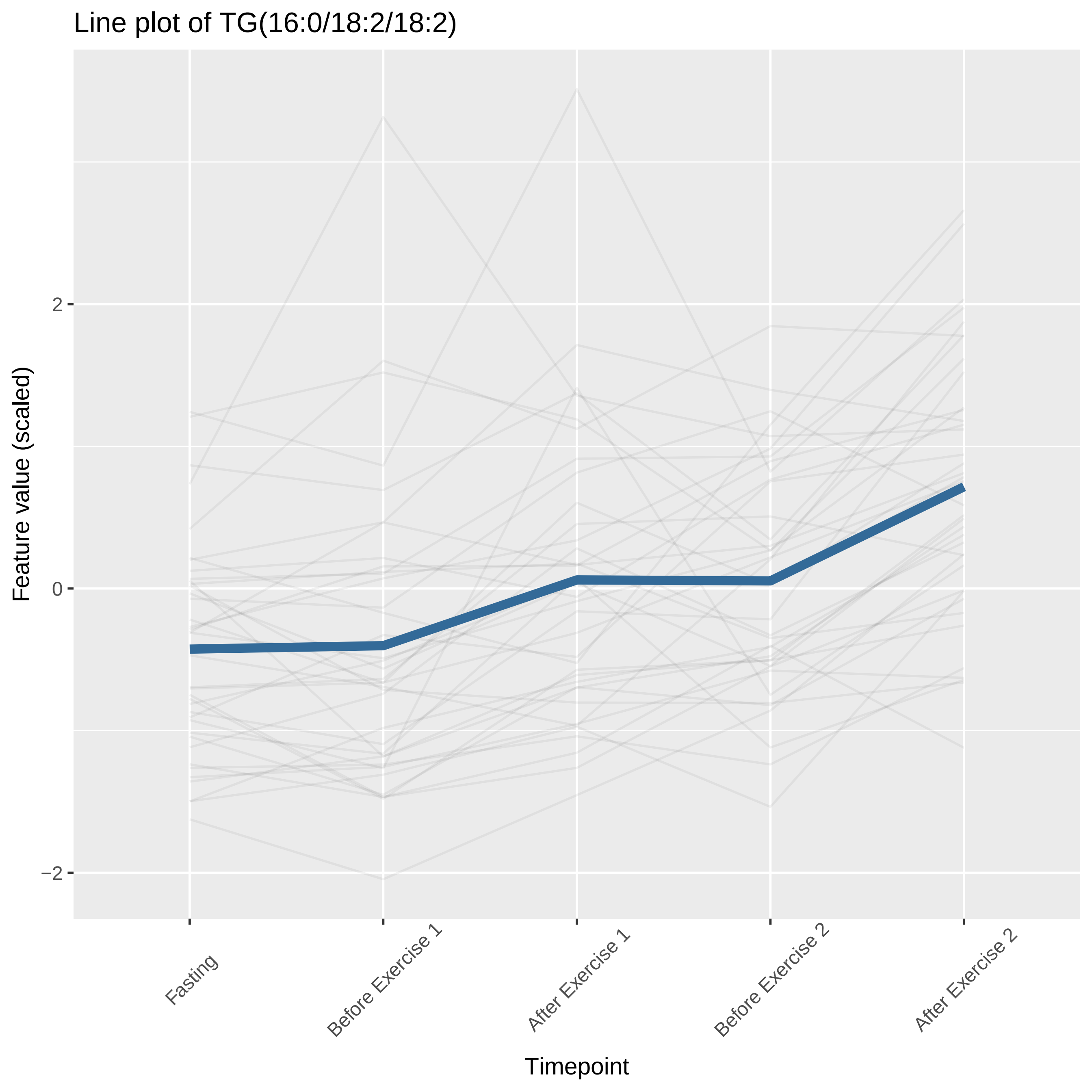

Line plot of TG(16:0/18:2/18:2)
Feature value (scaled)
2
0
−2
Fasting
Before Exercise 1
After Exercise 1
Before Exercise 2
After Exercise 2
Timepoint

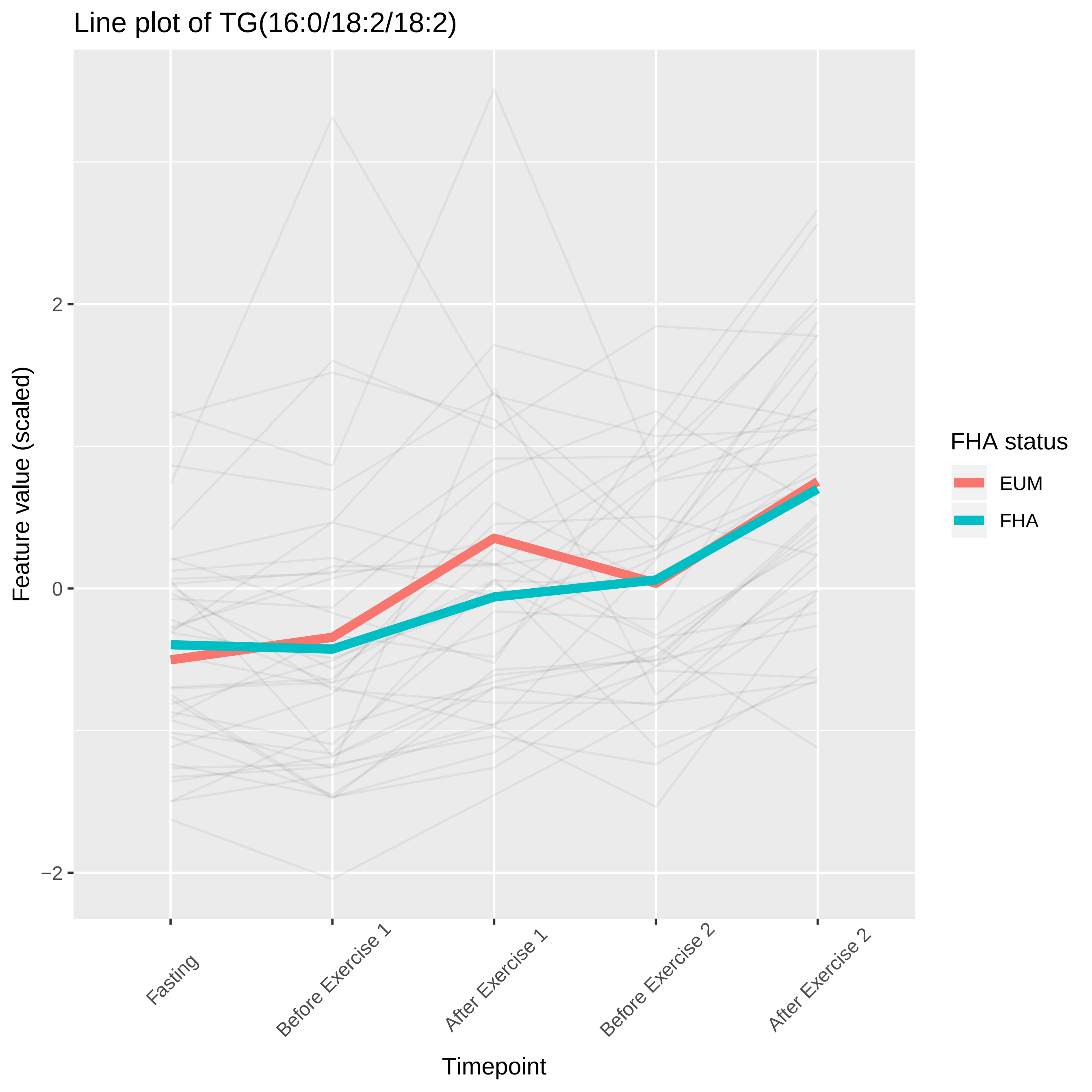

Line plot of TG(16:0/18:2/18:2)
Feature value (scaled)
2
0
−2
Fasting
Before Exercise 1
After Exercise 1
Before Exercise 2
After Exercise 2
Timepoint
FHA status
EUM
FHA

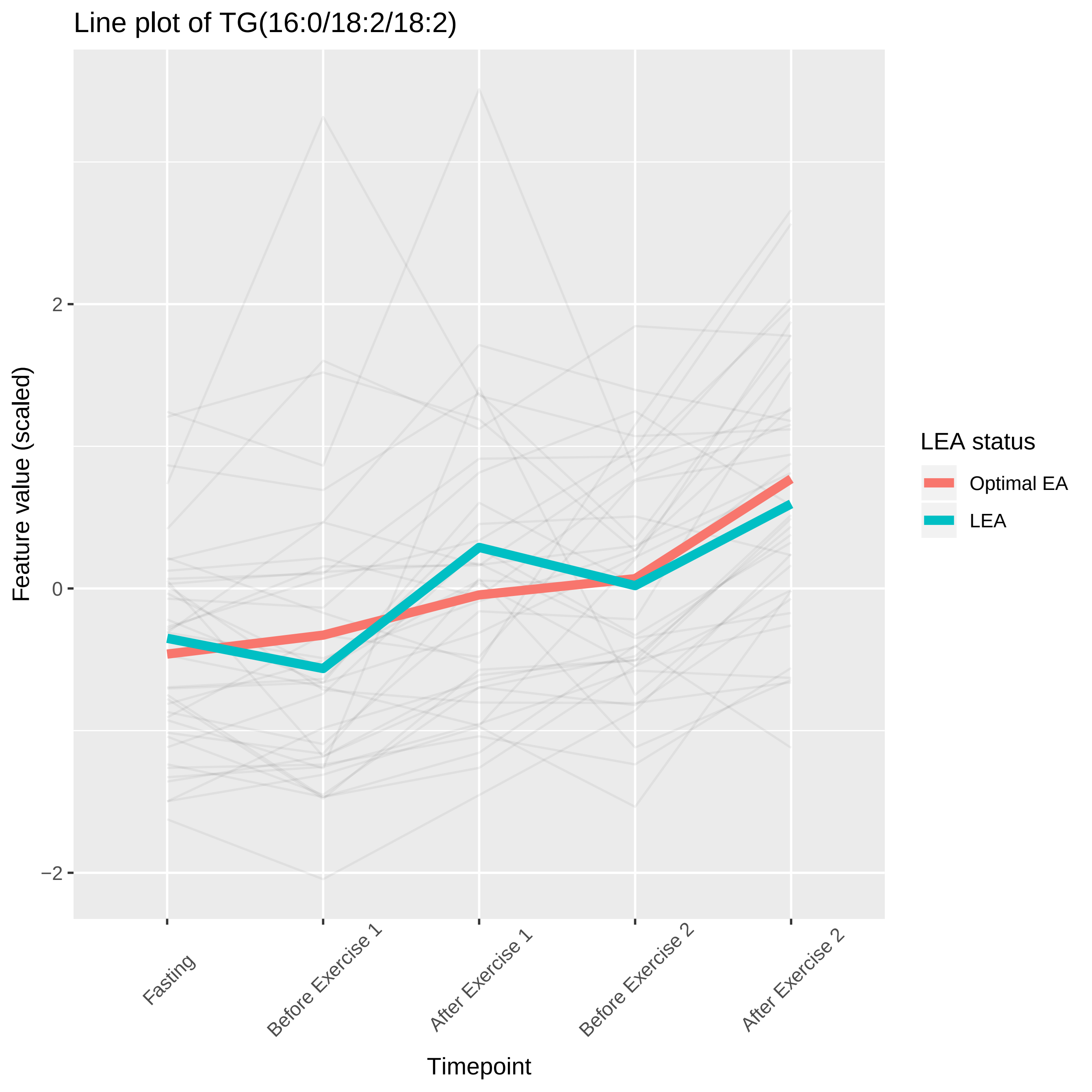

Line plot of TG(16:0/18:2/18:2)
Feature value (scaled)
2
0
−2
Fasting
Before Exercise 1
After Exercise 1
Before Exercise 2
After Exercise 2
Timepoint
LEA status
Optimal EA
LEA

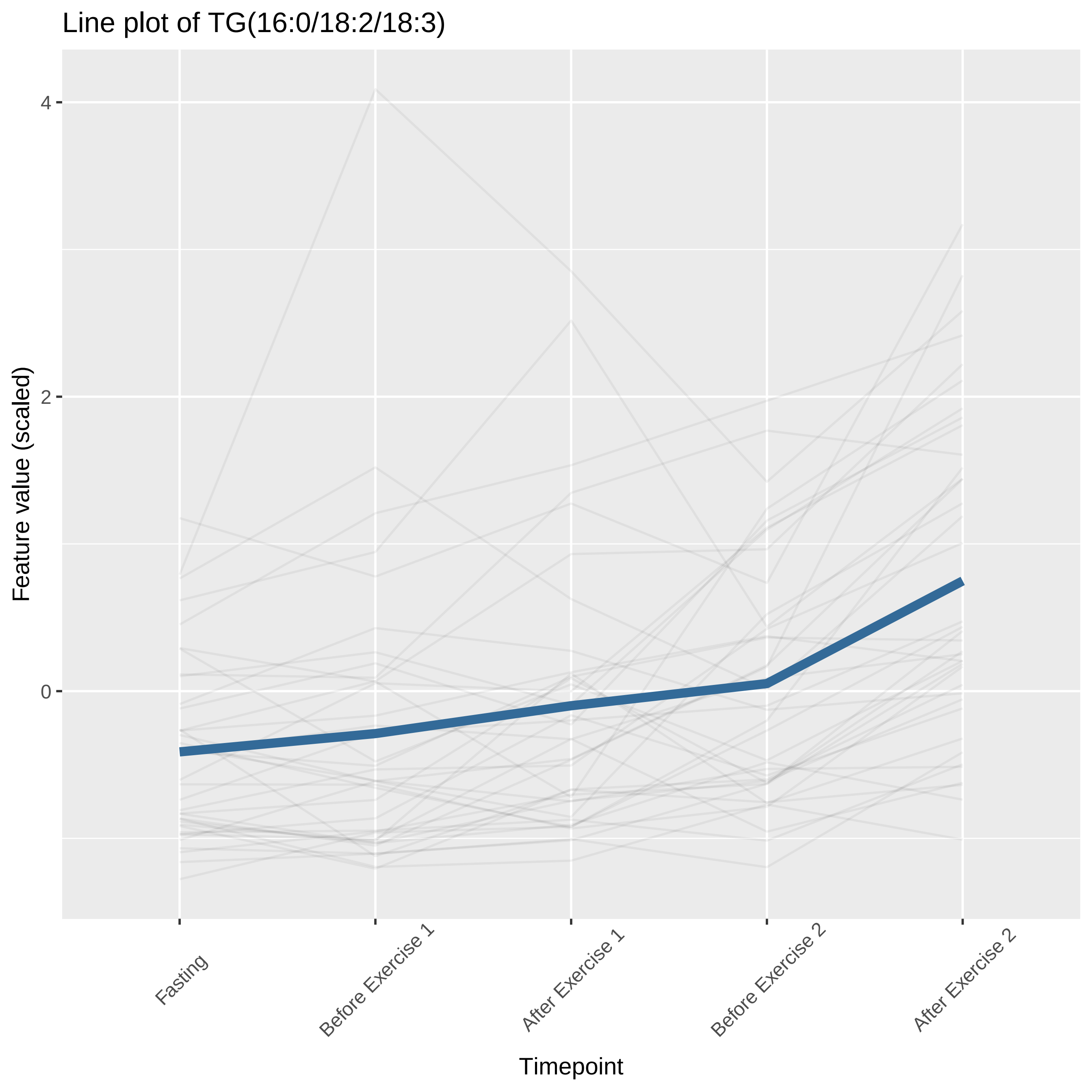

Line plot of TG(16:0/18:2/18:3)
Feature value (scaled)
4
2
0
Fasting
Before Exercise 1
After Exercise 1
Before Exercise 2
After Exercise 2
Timepoint

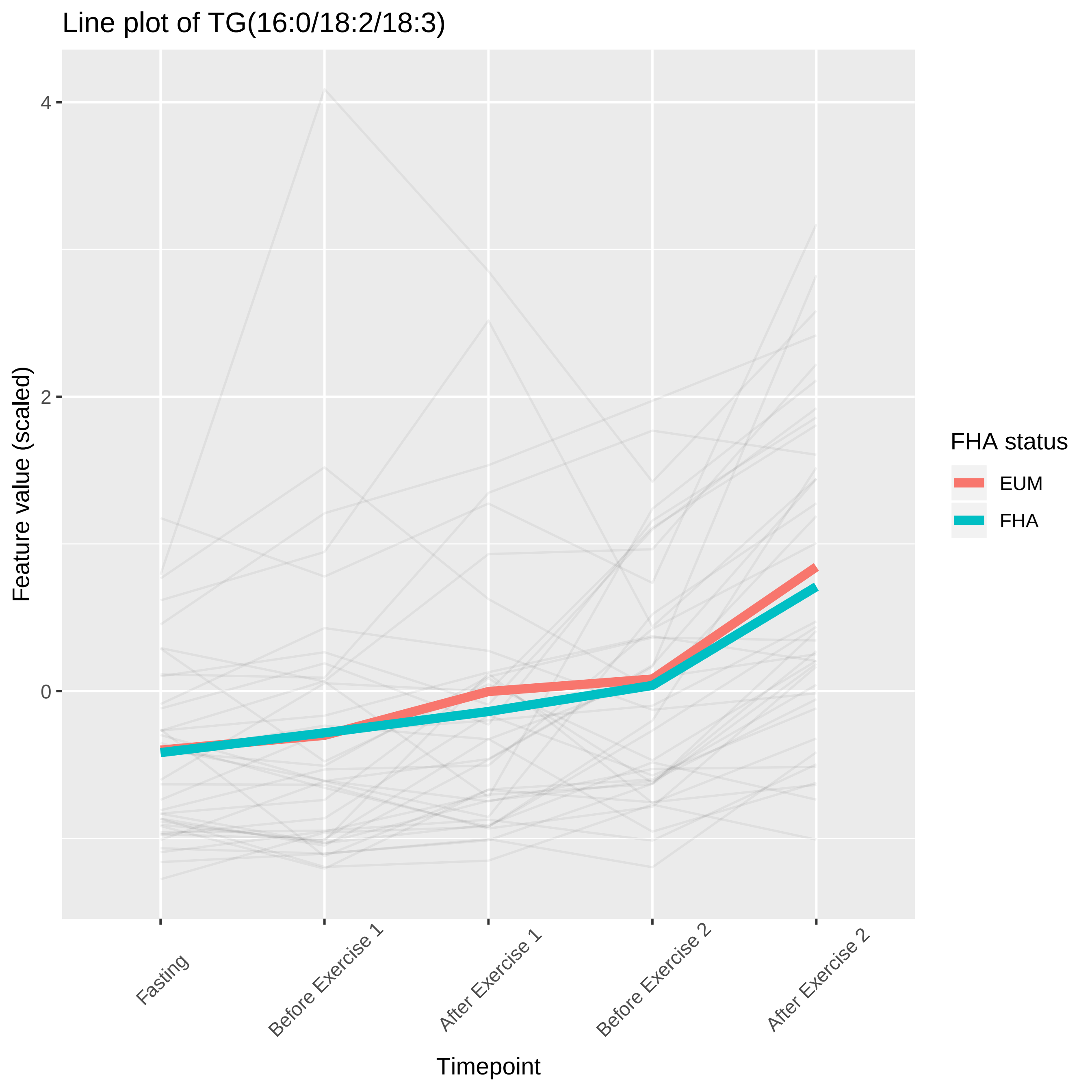

Line plot of TG(16:0/18:2/18:3)
Feature value (scaled)
4
2
0
Fasting
Before Exercise 1
After Exercise 1
Before Exercise 2
After Exercise 2
Timepoint
FHA status
EUM
FHA

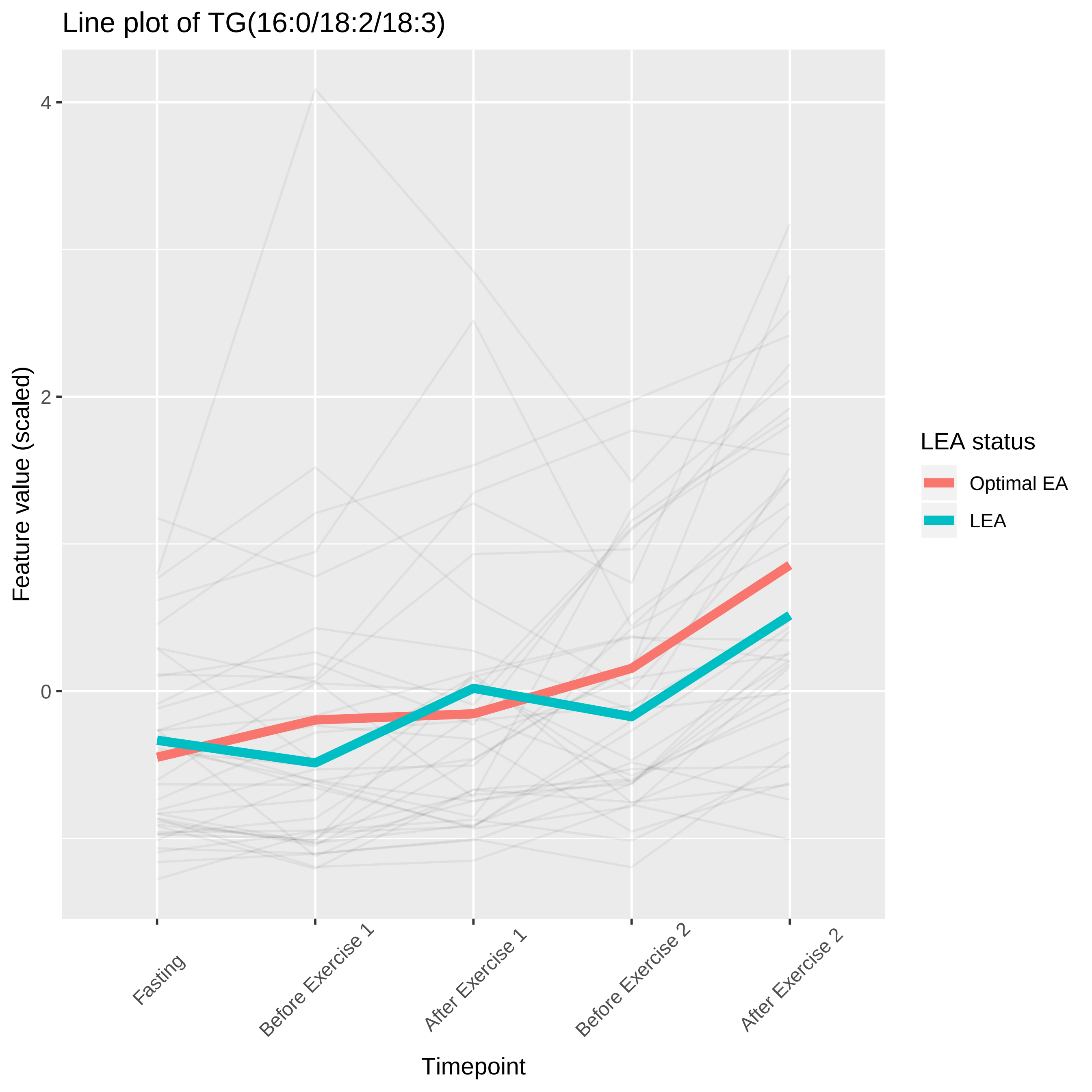

Line plot of TG(16:0/18:2/18:3)
Feature value (scaled)
4
2
0
Fasting
Before Exercise 1
After Exercise 1
Before Exercise 2
After Exercise 2
Timepoint
LEA status
Optimal EA
LEA

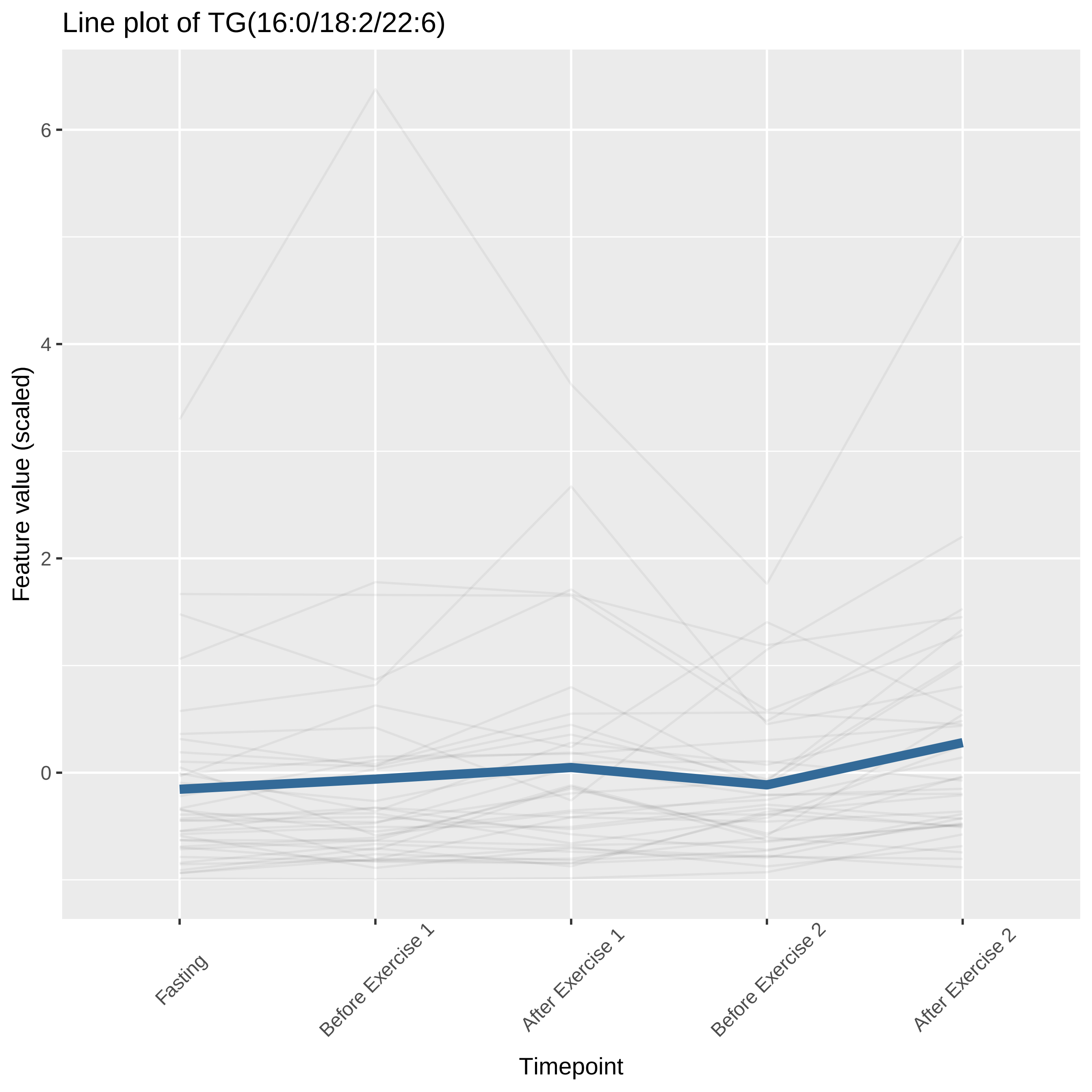

Line plot of TG(16:0/18:2/22:6)
Feature value (scaled)
6
4
2
0
Fasting
Before Exercise 1
After Exercise 1
Before Exercise 2
After Exercise 2
Timepoint

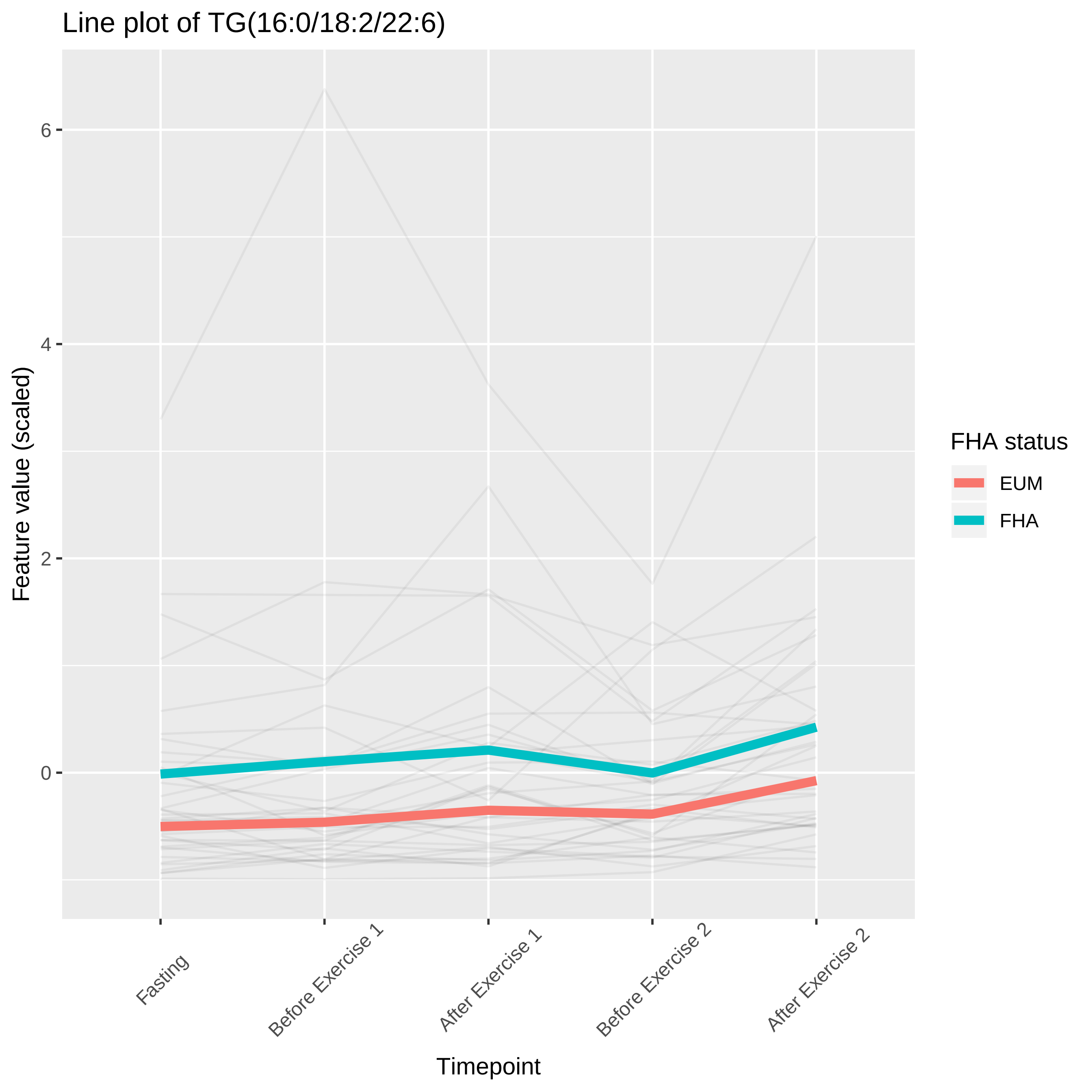

Line plot of TG(16:0/18:2/22:6)
Feature value (scaled)
6
4
2
0
Fasting
Before Exercise 1
After Exercise 1
Before Exercise 2
After Exercise 2
Timepoint
FHA status
EUM
FHA

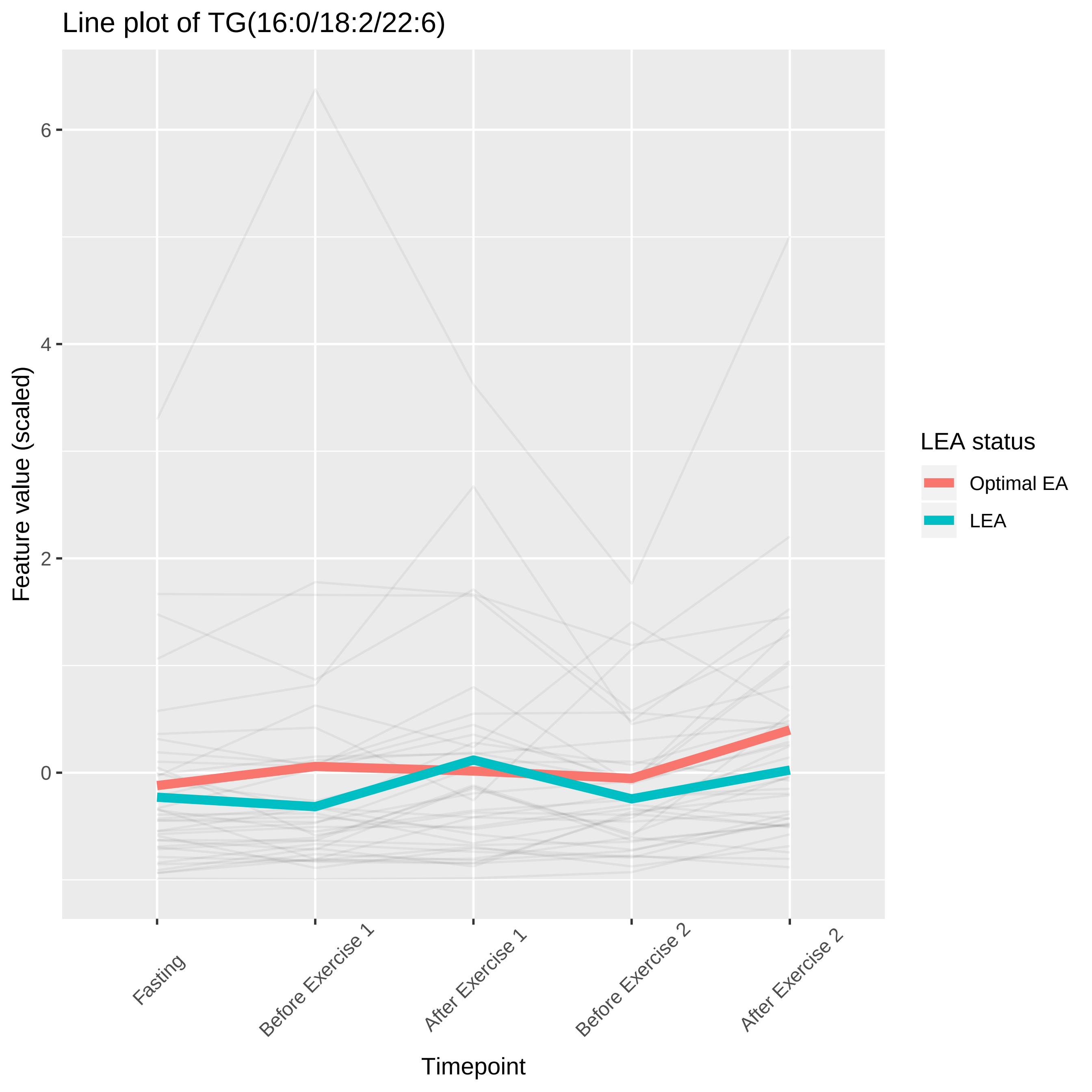

Line plot of TG(16:0/18:2/22:6)
Feature value (scaled)
6
4
2
0
Fasting
Before Exercise 1
After Exercise 1
Before Exercise 2
After Exercise 2
Timepoint
LEA status
Optimal EA
LEA

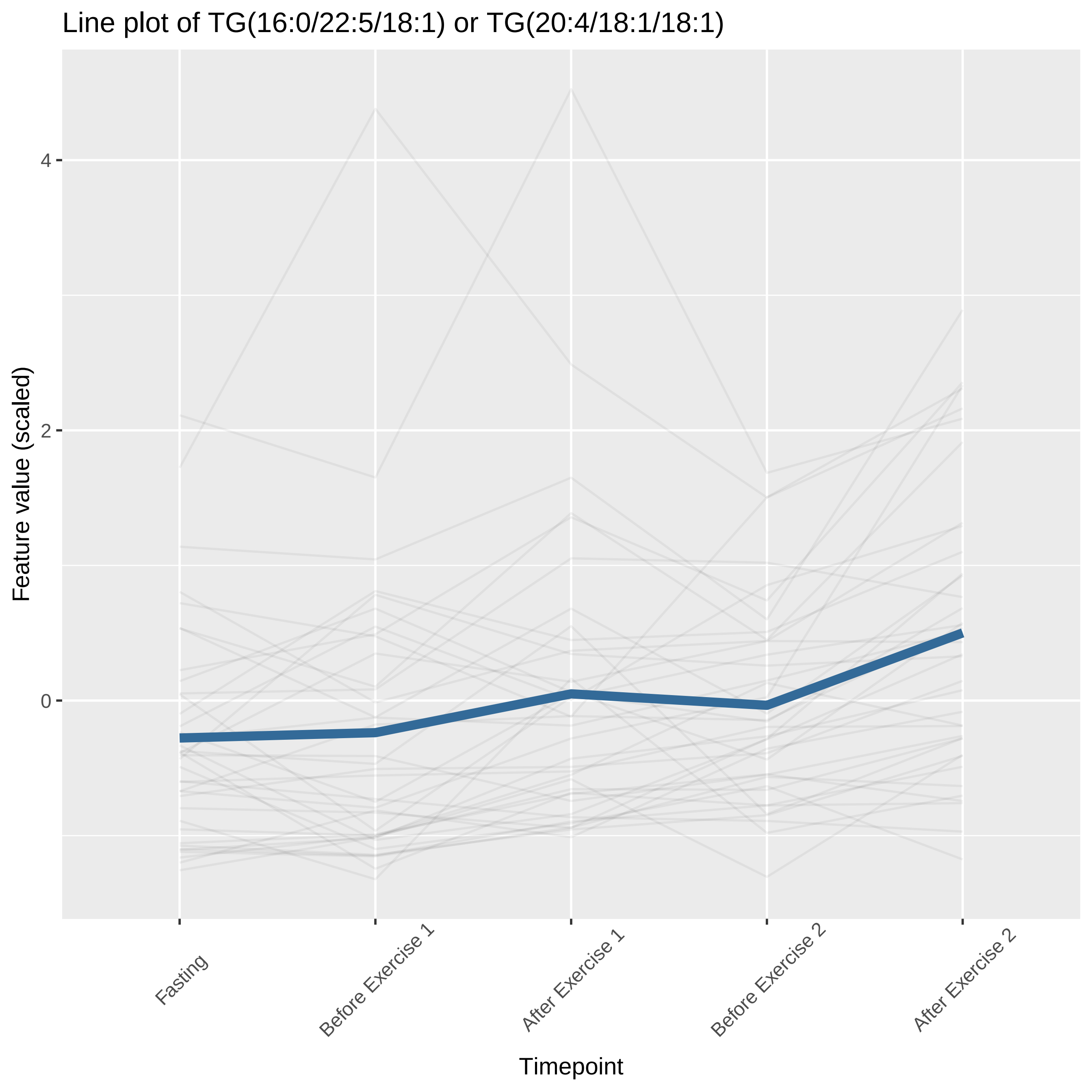

Line plot of TG(16:0/22:5/18:1) or TG(20:4/18:1/18:1)
Feature value (scaled)
4
2
0
Fasting
Before Exercise 1
After Exercise 1
Before Exercise 2
After Exercise 2
Timepoint

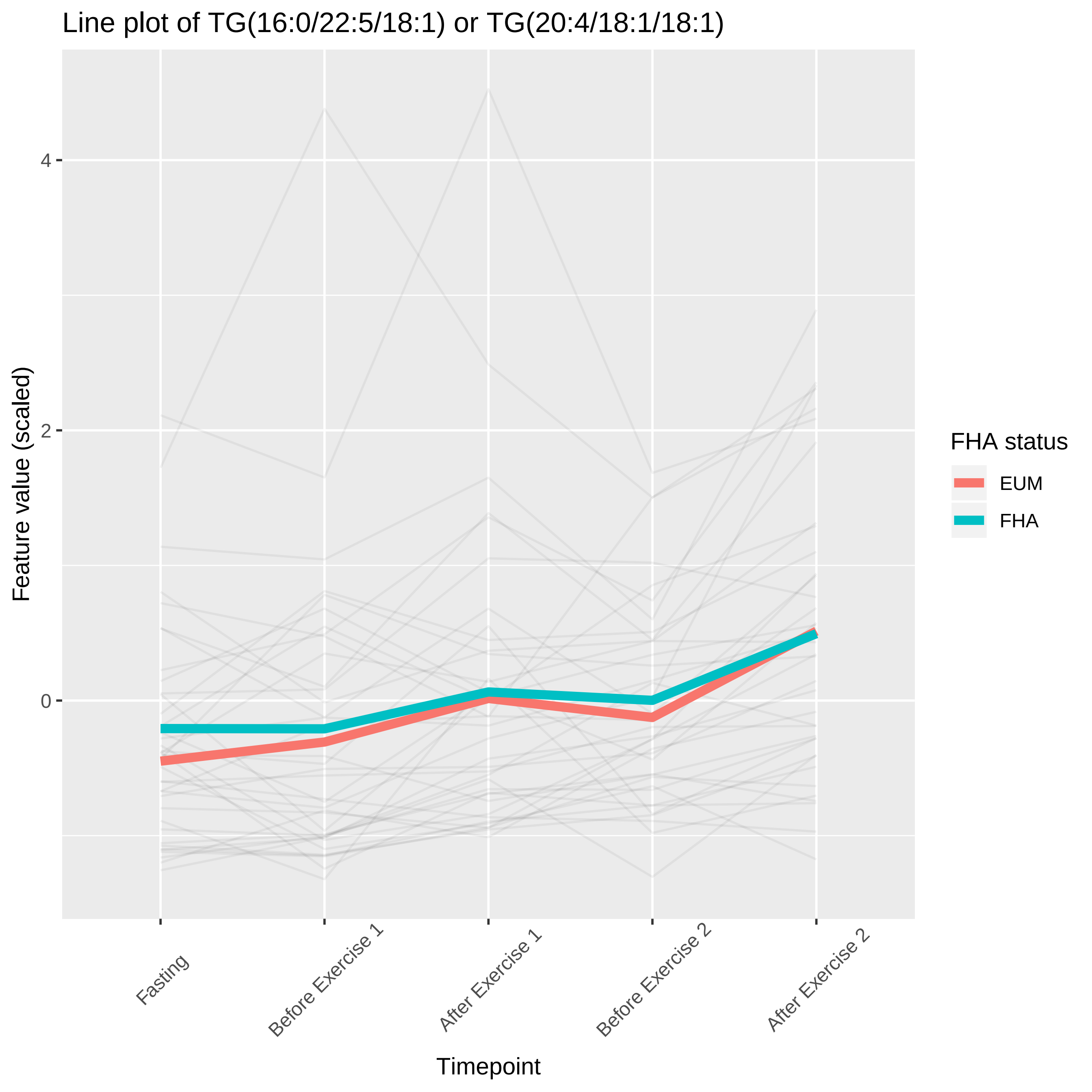

Line plot of TG(16:0/22:5/18:1) or TG(20:4/18:1/18:1)
Feature value (scaled)
4
2
0
Fasting
Before Exercise 1
After Exercise 1
Before Exercise 2
After Exercise 2
Timepoint
FHA status
EUM
FHA

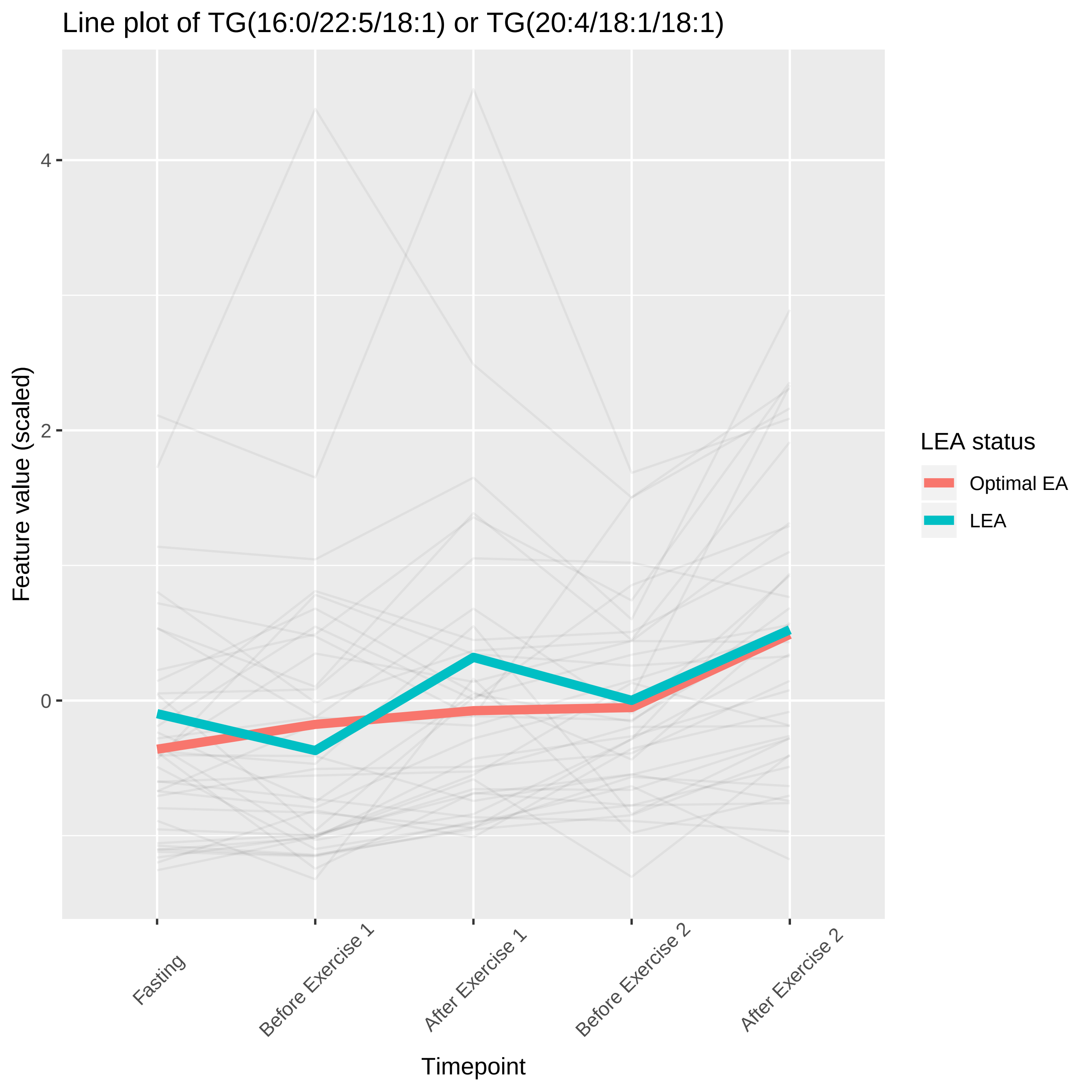
Line plot of TG(16:0/22:5/18:1) or TG(20:4/18:1/18:1)
Feature value (scaled)
4
2
0
Fasting
Before Exercise 1
After Exercise 1
Before Exercise 2
After Exercise 2
Timepoint
LEA status
Optimal EA
LEA

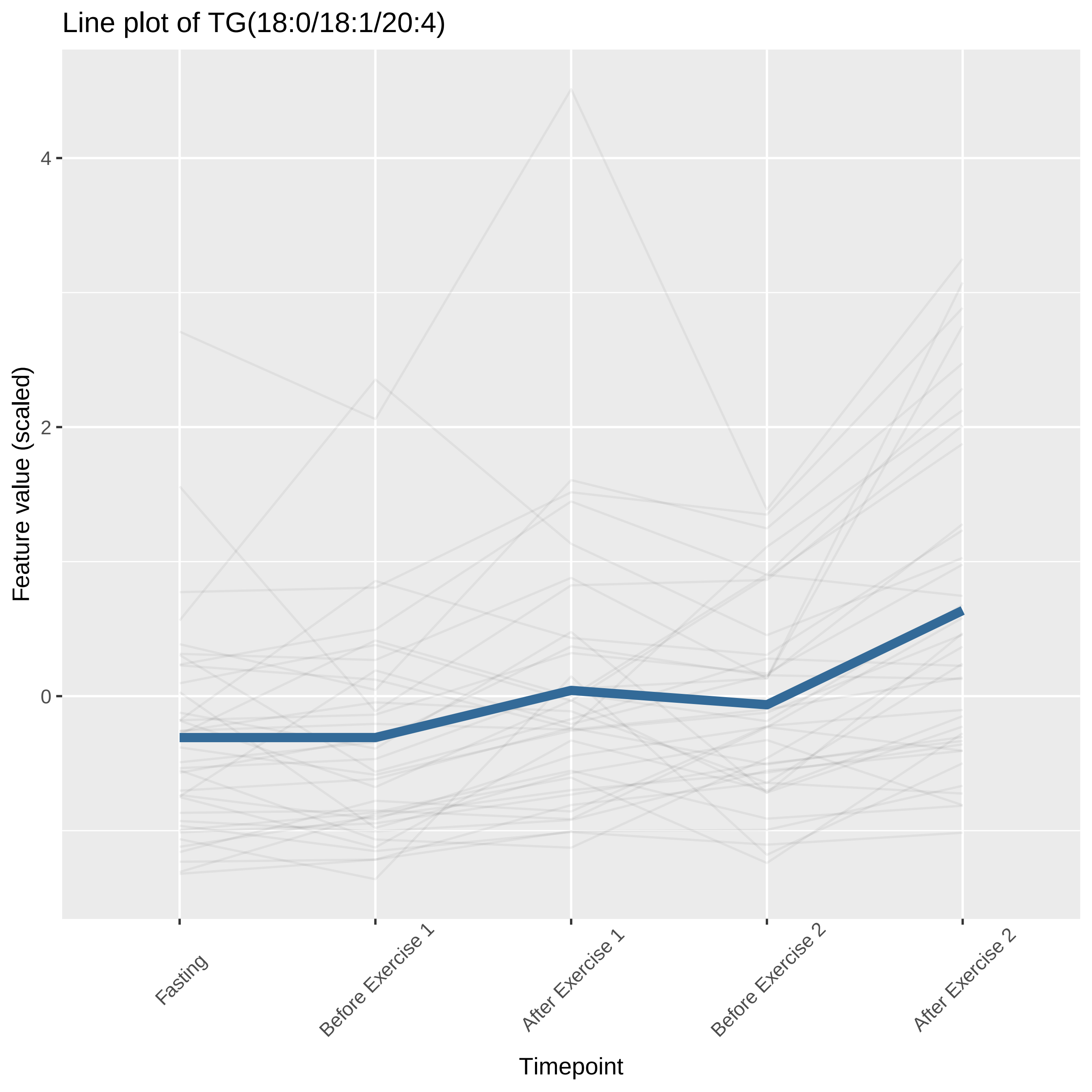
Line plot of TG(18:0/18:1/20:4)
Feature value (scaled)
4
2
0
Fasting
Before Exercise 1
After Exercise 1
Before Exercise 2
After Exercise 2
Timepoint

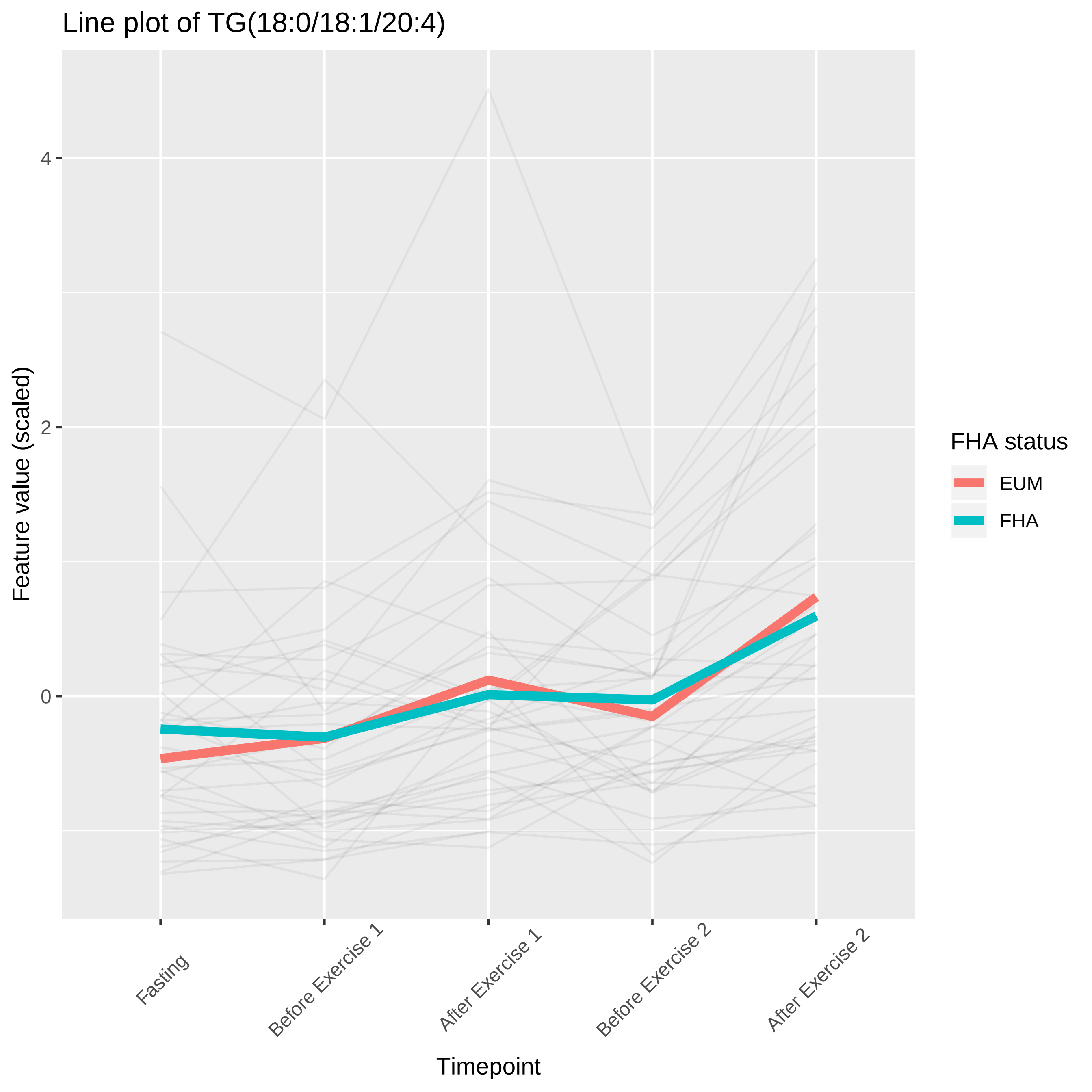

Line plot of TG(18:0/18:1/20:4)
Feature value (scaled)
4
2
0
Fasting
Before Exercise 1
After Exercise 1
Before Exercise 2
After Exercise 2
Timepoint
FHA status
EUM
FHA

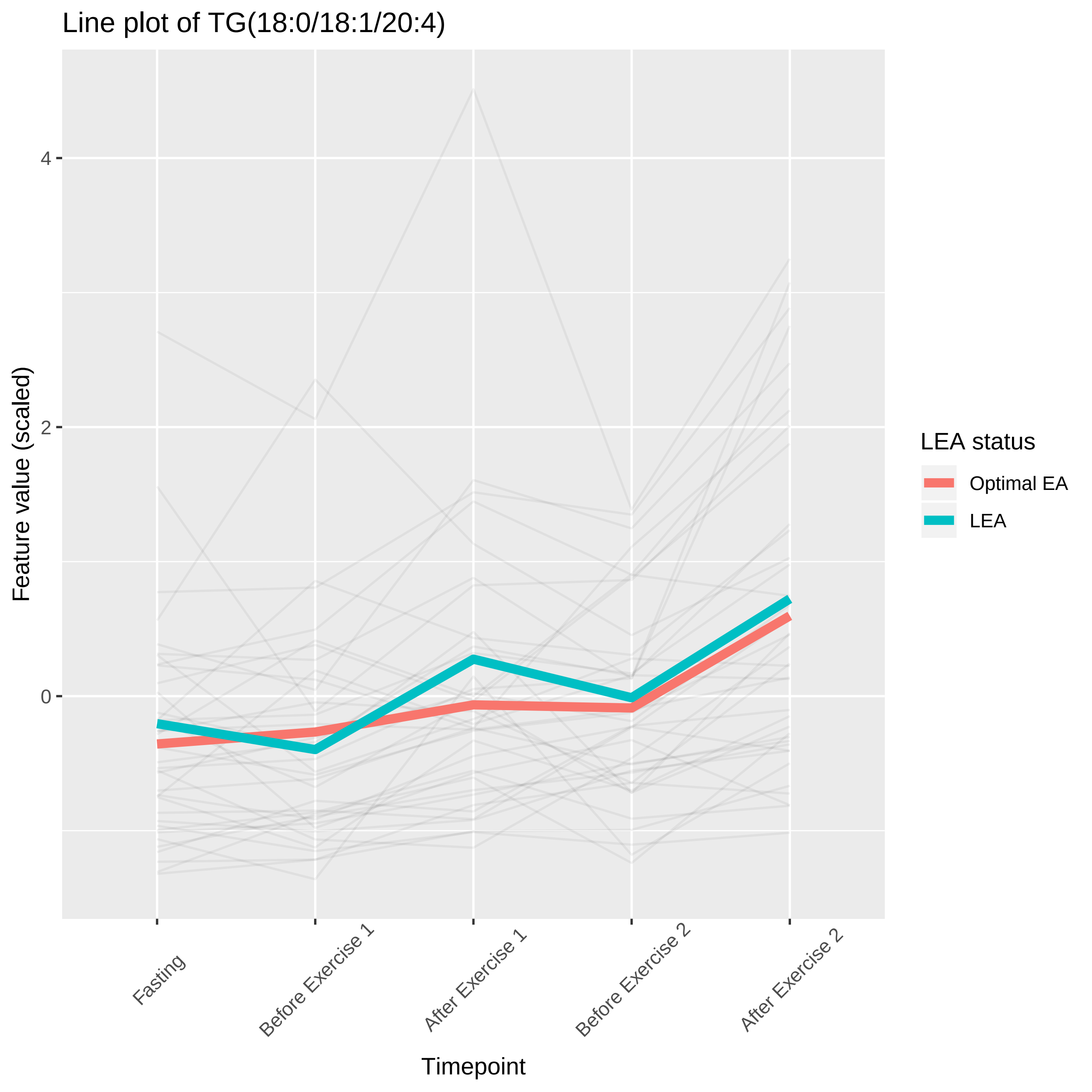

Line plot of TG(18:0/18:1/20:4)
Feature value (scaled)
4
2
0
Fasting
Before Exercise 1
After Exercise 1
Before Exercise 2
After Exercise 2
Timepoint
LEA status
Optimal EA
LEA

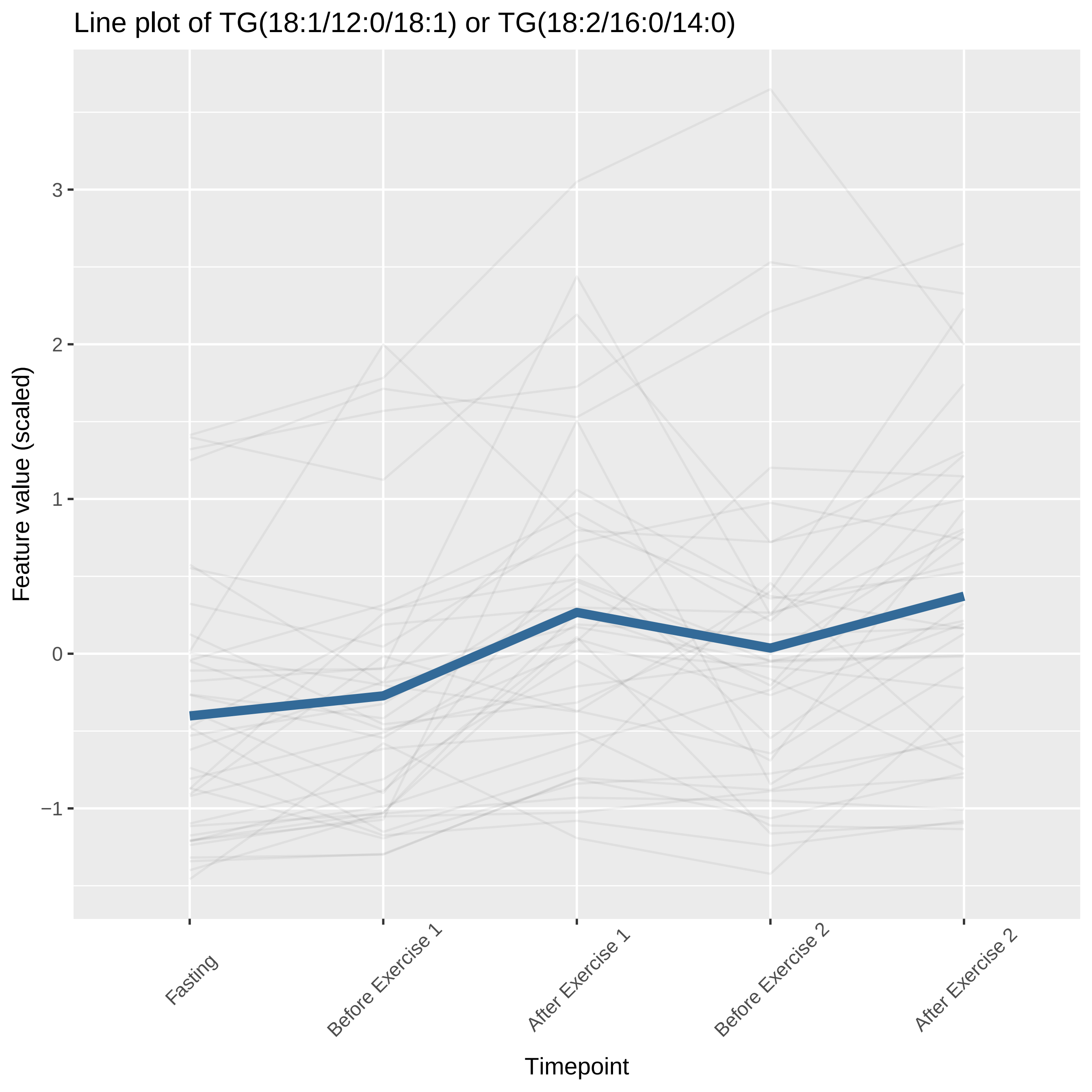

Line plot of TG(18:1/12:0/18:1) or TG(18:2/16:0/14:0)
Feature value (scaled)
3
2
1
0
−1
Fasting
Before Exercise 1
After Exercise 1
Before Exercise 2
After Exercise 2
Timepoint

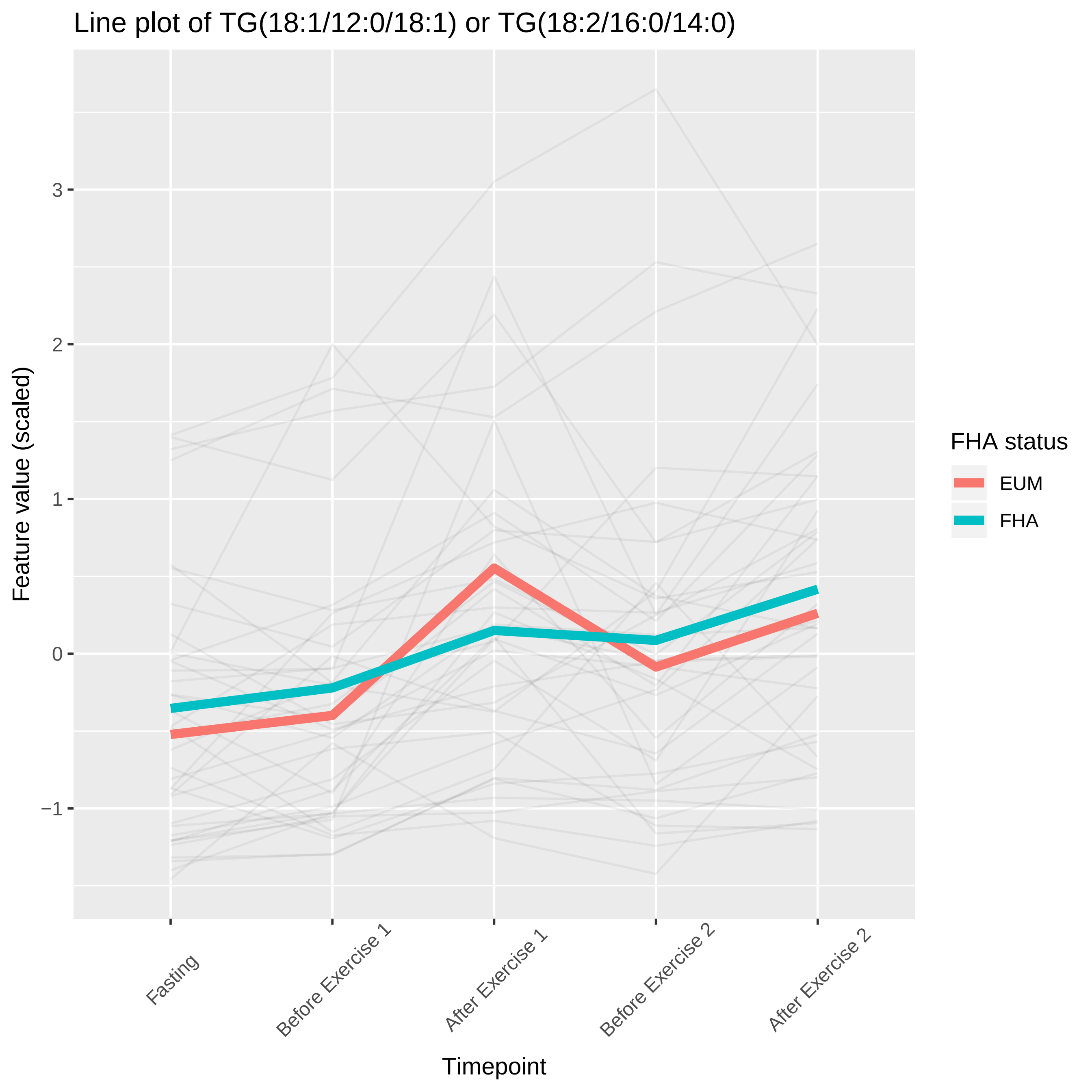

Line plot of TG(18:1/12:0/18:1) or TG(18:2/16:0/14:0)
Feature value (scaled)
3
2
1
0
−1
Fasting
Before Exercise 1
After Exercise 1
Before Exercise 2
After Exercise 2
Timepoint
FHA status
EUM
FHA

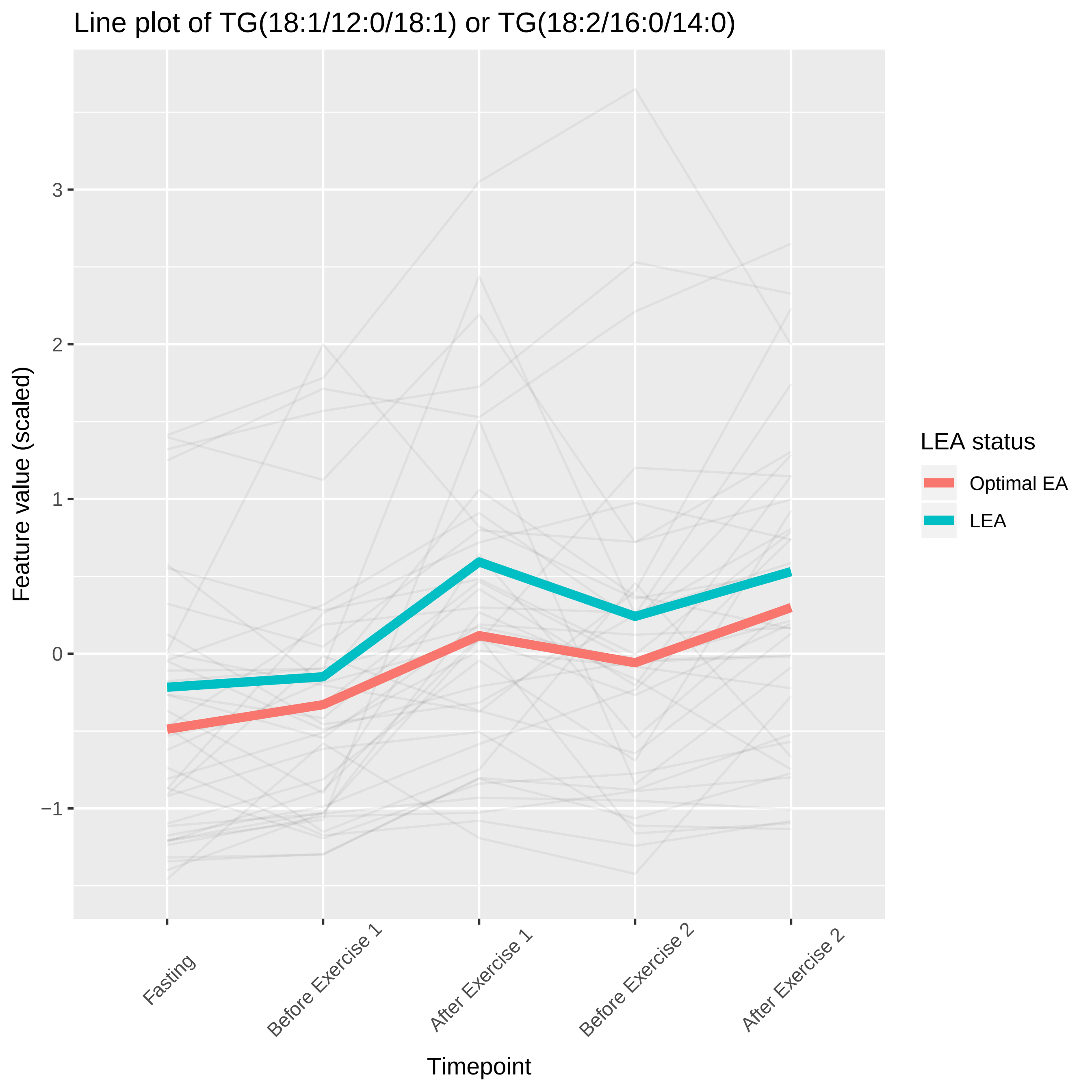

Line plot of TG(18:1/12:0/18:1) or TG(18:2/16:0/14:0)
Feature value (scaled)
3
2
1
0
−1
Fasting
Before Exercise 1
After Exercise 1
Before Exercise 2
After Exercise 2
Timepoint
LEA status
Optimal EA
LEA

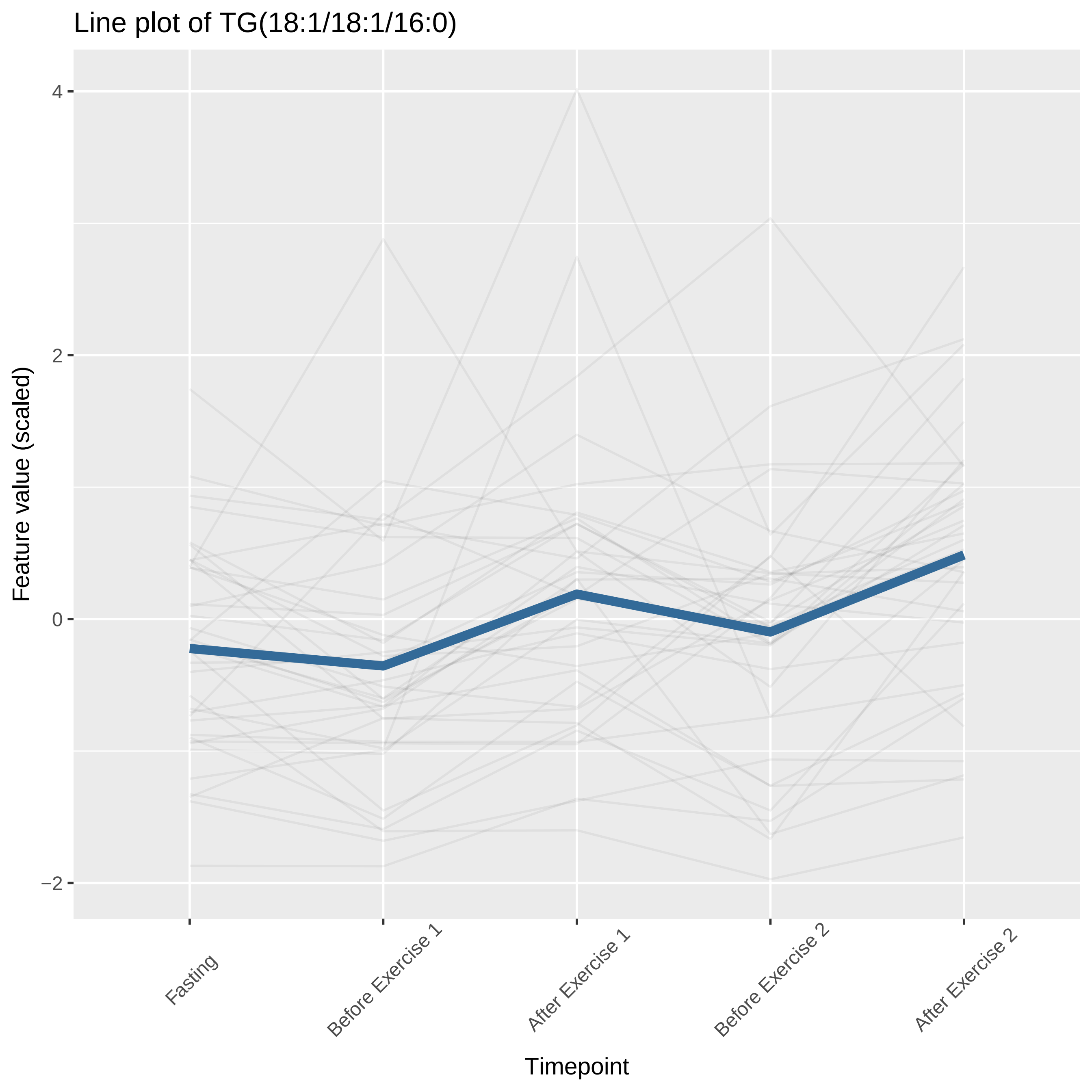
Line plot of TG(18:1/18:1/16:0)
Feature value (scaled)
4
2
0
−2
Fasting
Before Exercise 1
After Exercise 1
Before Exercise 2
After Exercise 2
Timepoint

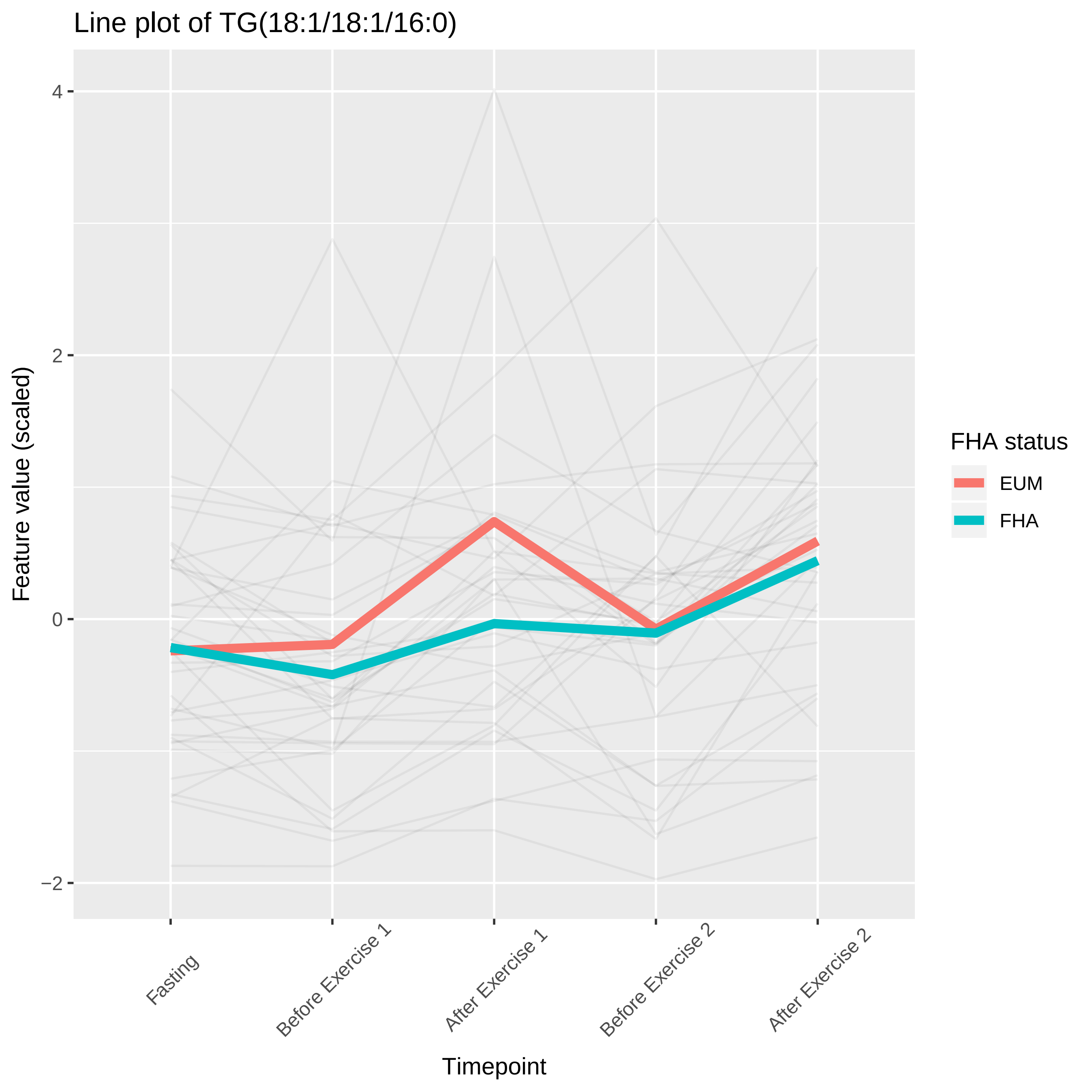

Line plot of TG(18:1/18:1/16:0)
Feature value (scaled)
4
2
0
−2
Fasting
Before Exercise 1
After Exercise 1
Before Exercise 2
After Exercise 2
Timepoint
FHA status
EUM
FHA

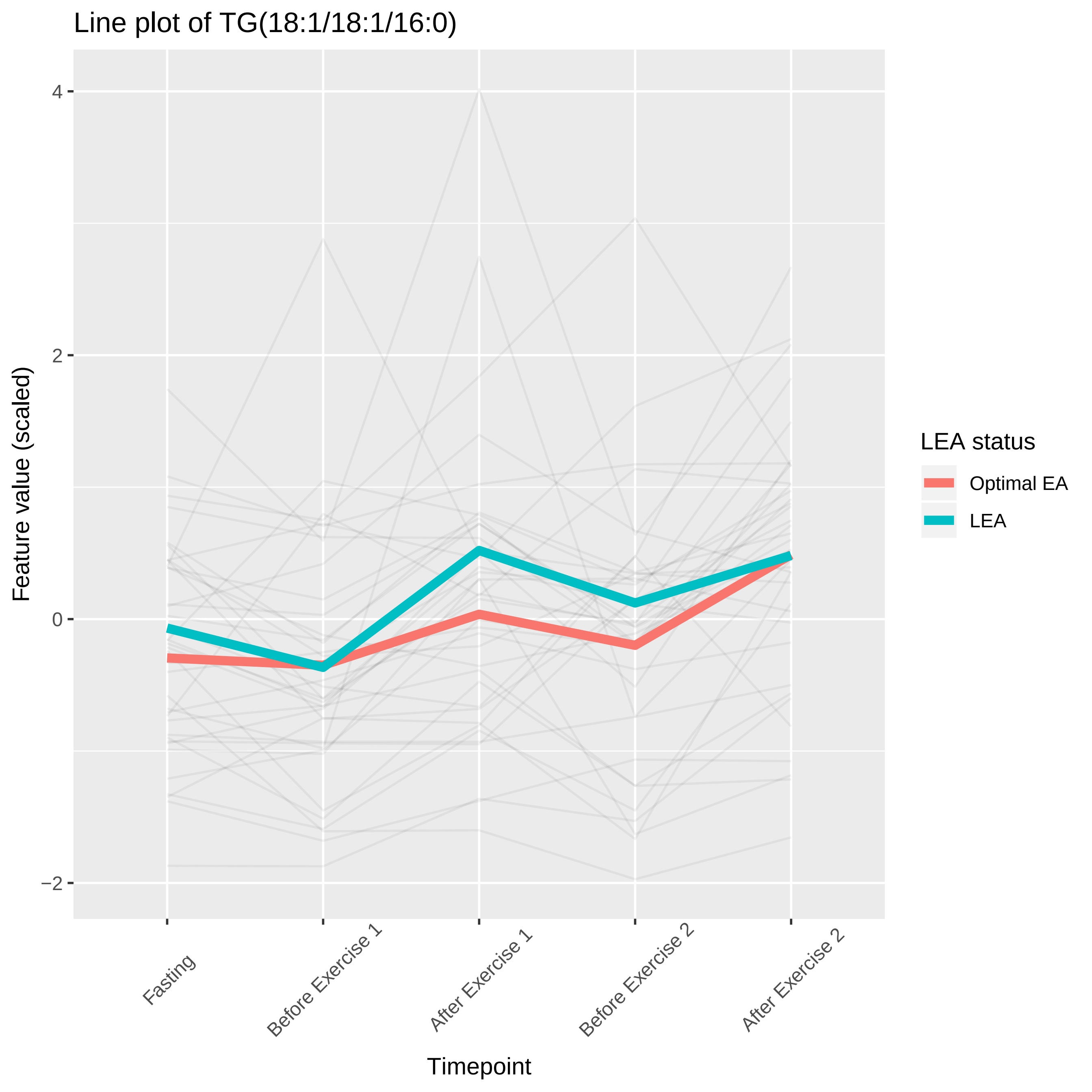

Line plot of TG(18:1/18:1/16:0)
Feature value (scaled)
4
2
0
−2
Fasting
Before Exercise 1
After Exercise 1
Before Exercise 2
After Exercise 2
Timepoint
LEA status
Optimal EA
LEA

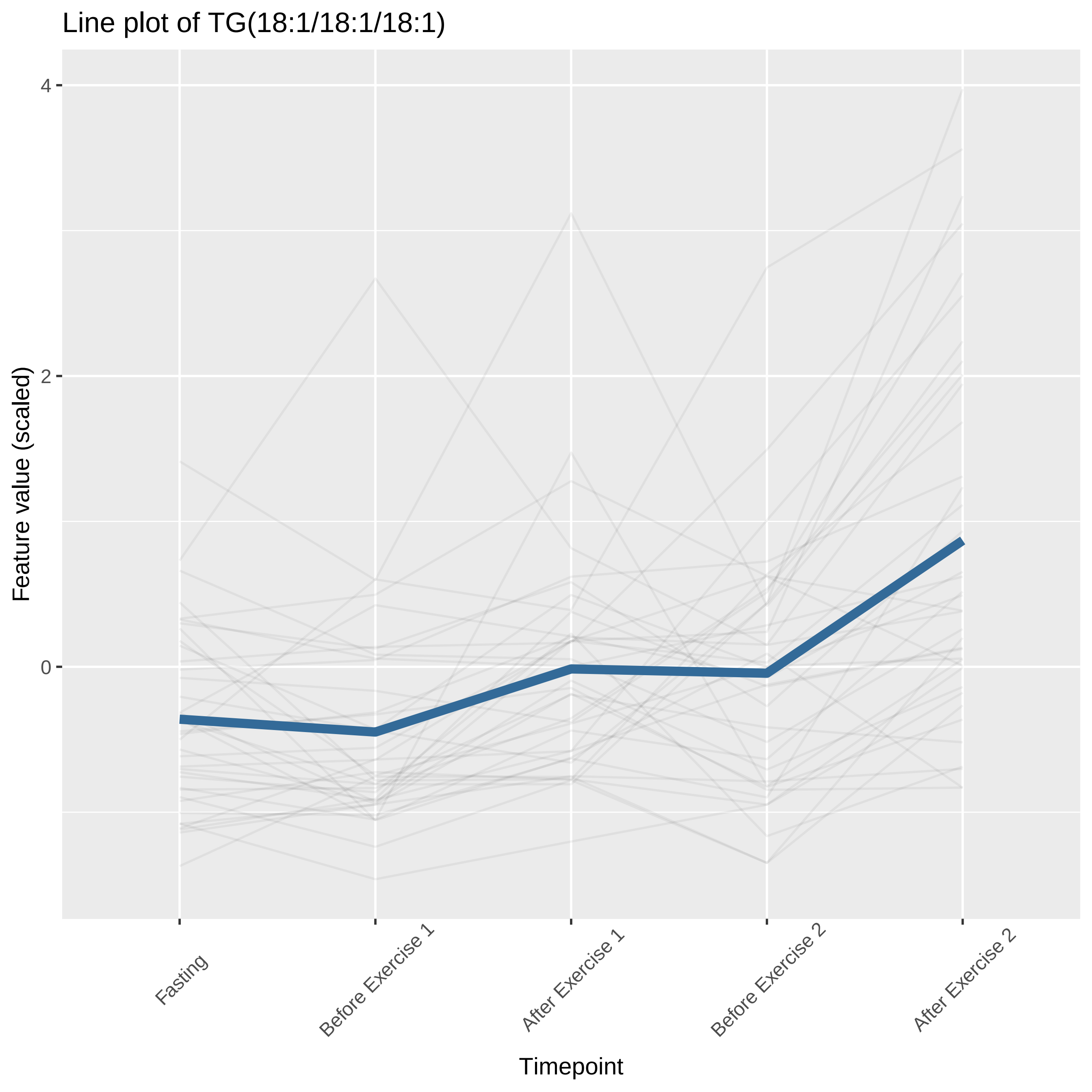
Line plot of TG(18:1/18:1/18:1)
Feature value (scaled)
4
2
0
Fasting
Before Exercise 1
After Exercise 1
Before Exercise 2
After Exercise 2
Timepoint

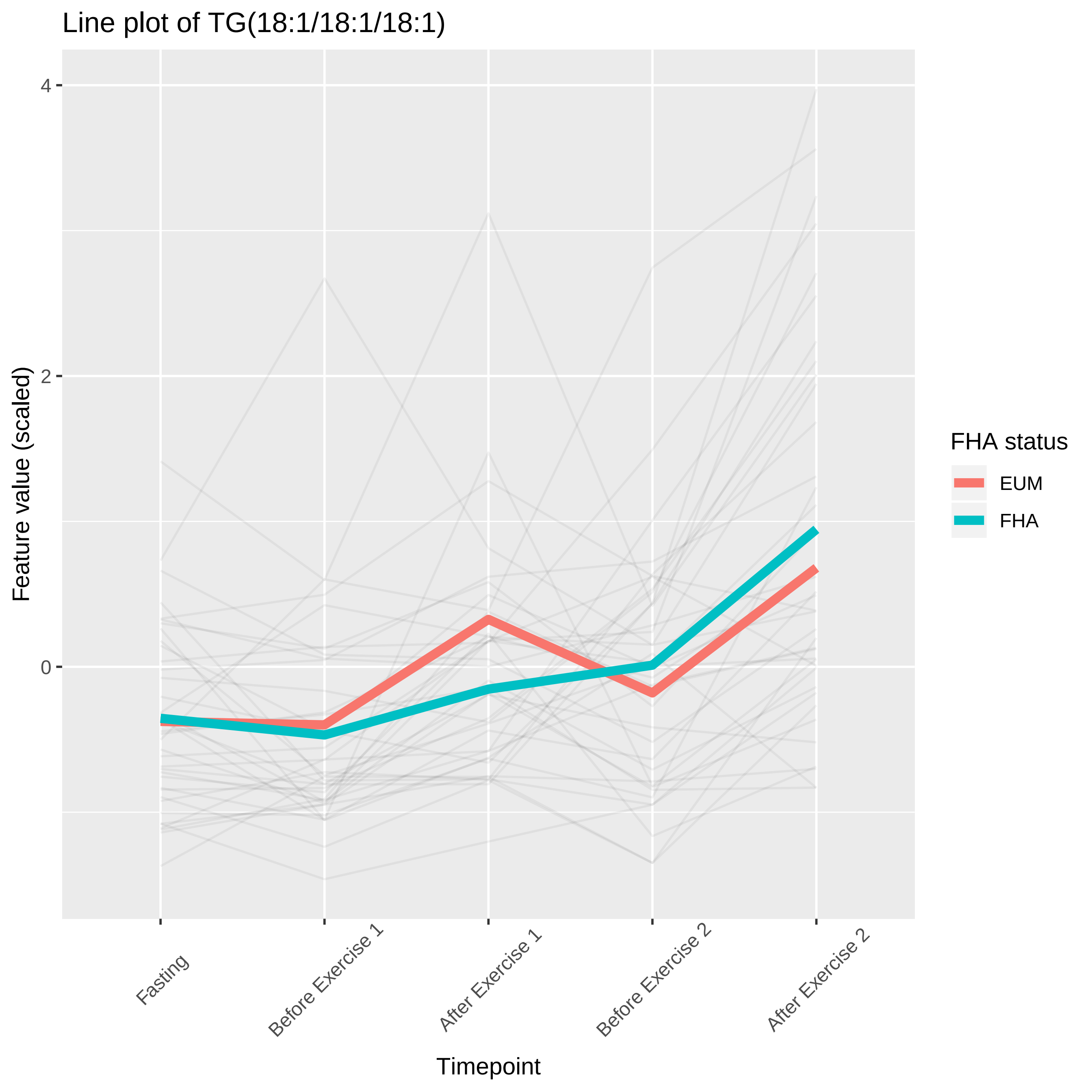
Line plot of TG(18:1/18:1/18:1)
Feature value (scaled)
4
2
0
Fasting
Before Exercise 1
After Exercise 1
Before Exercise 2
After Exercise 2
Timepoint
FHA status
EUM
FHA

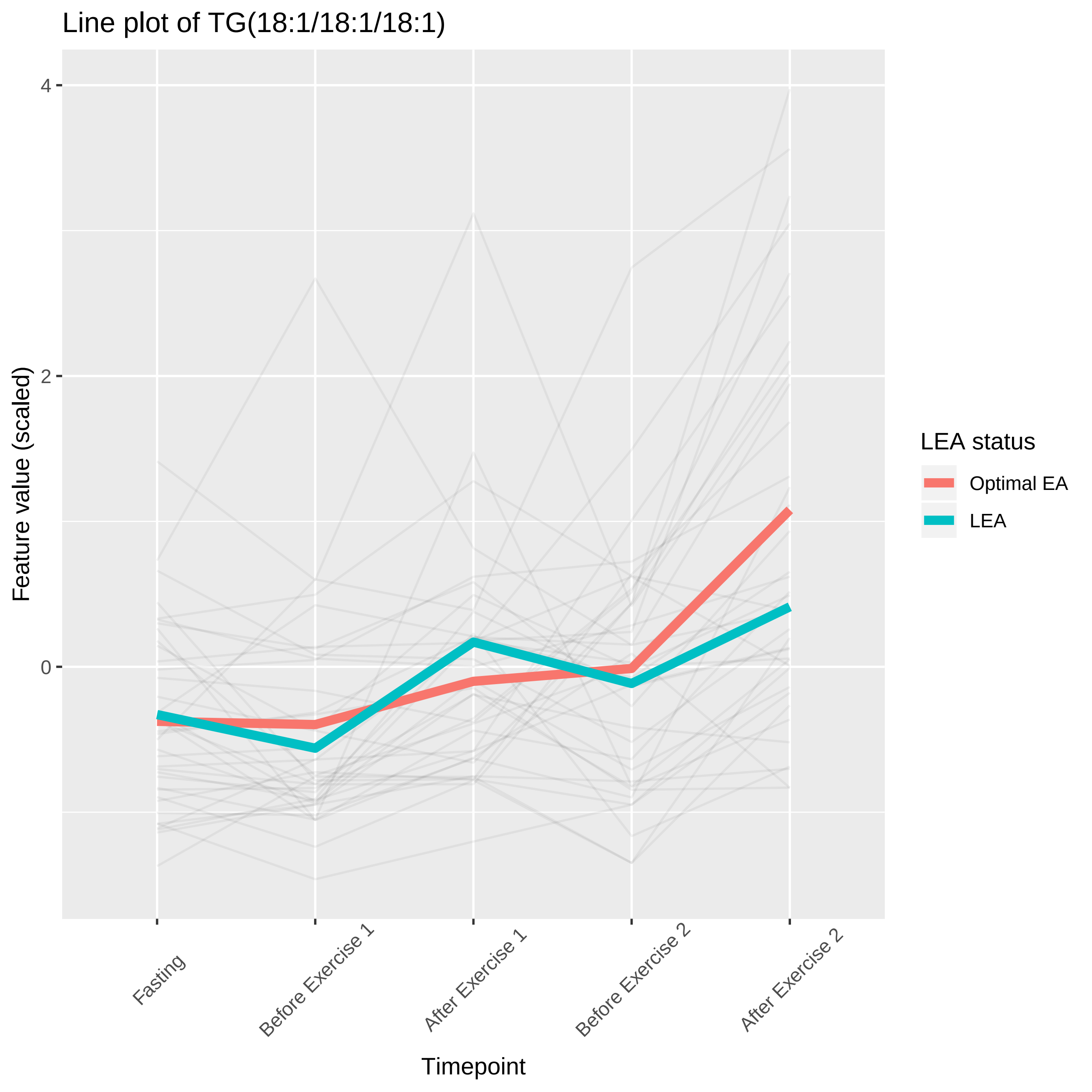

Line plot of TG(18:1/18:1/18:1)
Feature value (scaled)
4
2
0
Fasting
Before Exercise 1
After Exercise 1
Before Exercise 2
After Exercise 2
Timepoint
LEA status
Optimal EA
LEA

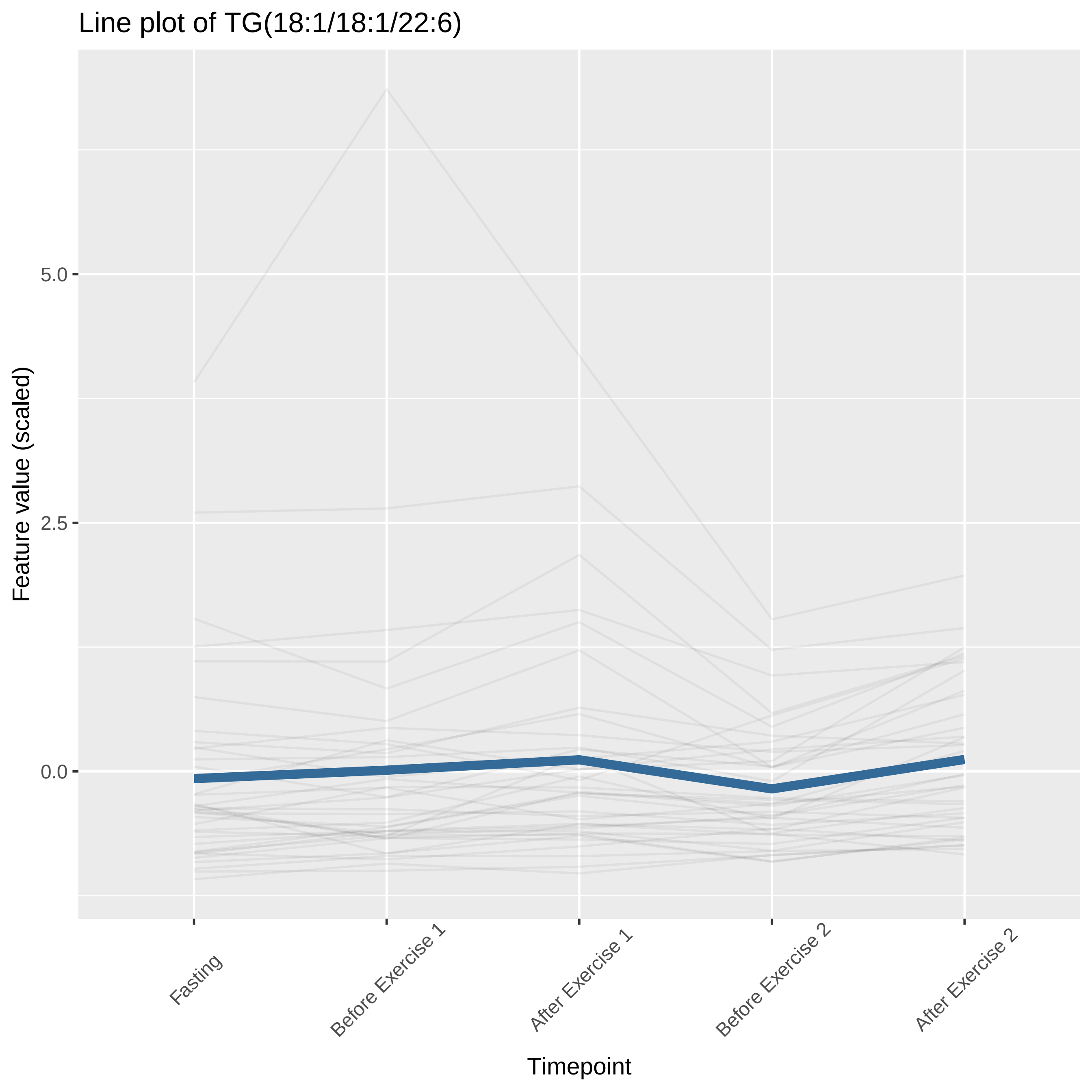

Line plot of TG(18:1/18:1/22:6)
Feature value (scaled)
5.0
2.5
0.0
Fasting
Before Exercise 1
After Exercise 1
Before Exercise 2
After Exercise 2
Timepoint

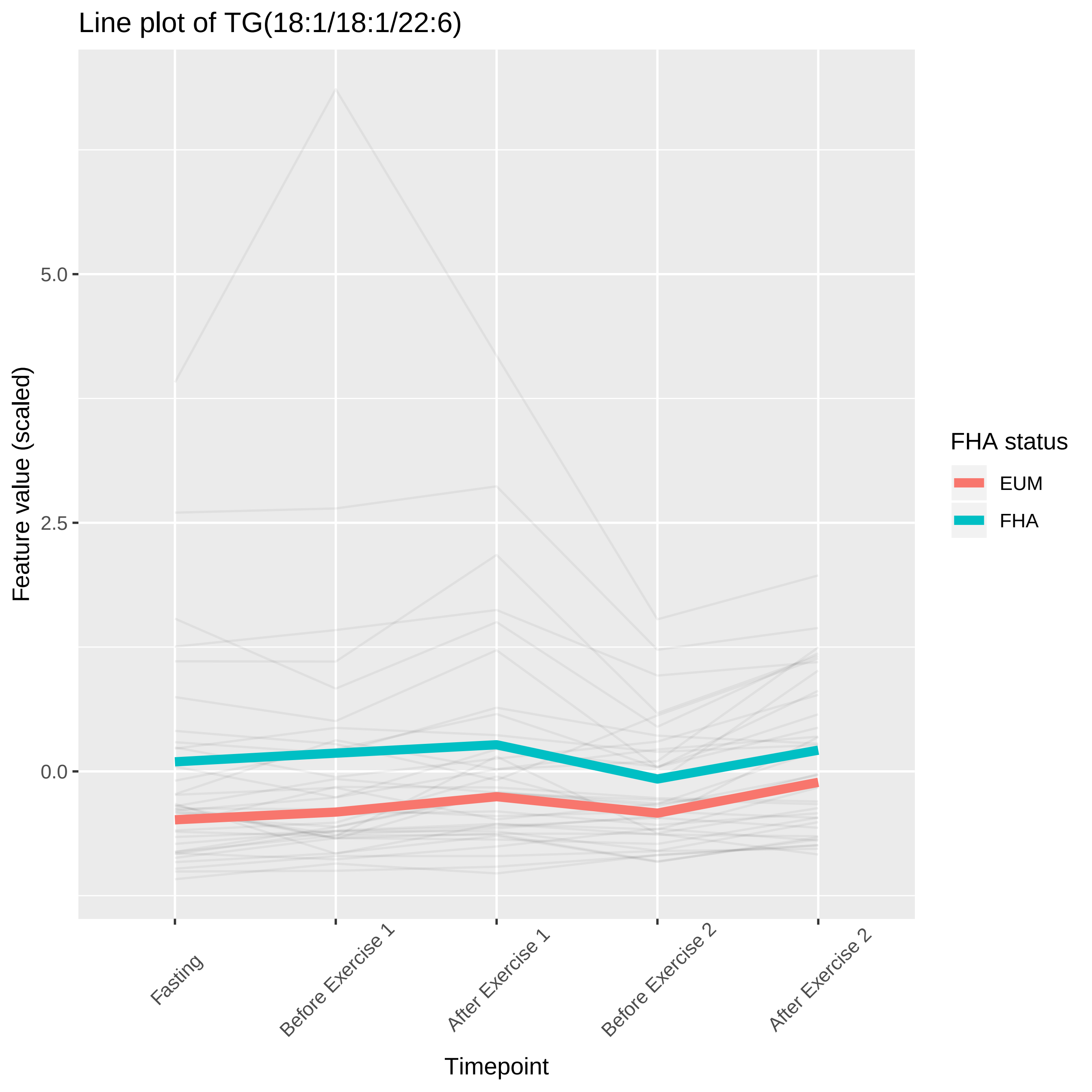

Line plot of TG(18:1/18:1/22:6)
Feature value (scaled)
5.0
2.5
0.0
Fasting
Before Exercise 1
After Exercise 1
Before Exercise 2
After Exercise 2
Timepoint
FHA status
EUM
FHA

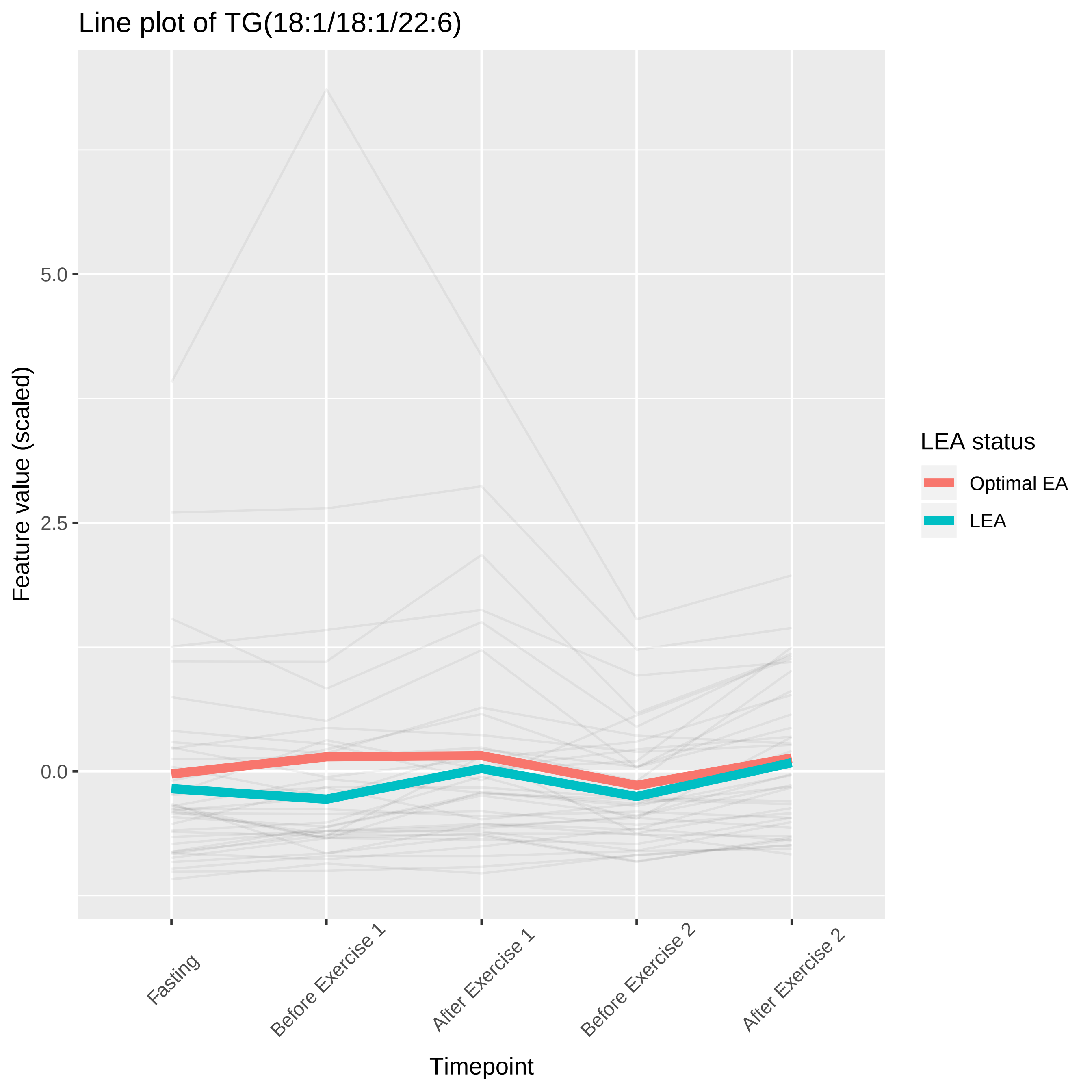

Line plot of TG(18:1/18:1/22:6)
Feature value (scaled)
5.0
2.5
0.0
Fasting
Before Exercise 1
After Exercise 1
Before Exercise 2
After Exercise 2
Timepoint
LEA status
Optimal EA
LEA

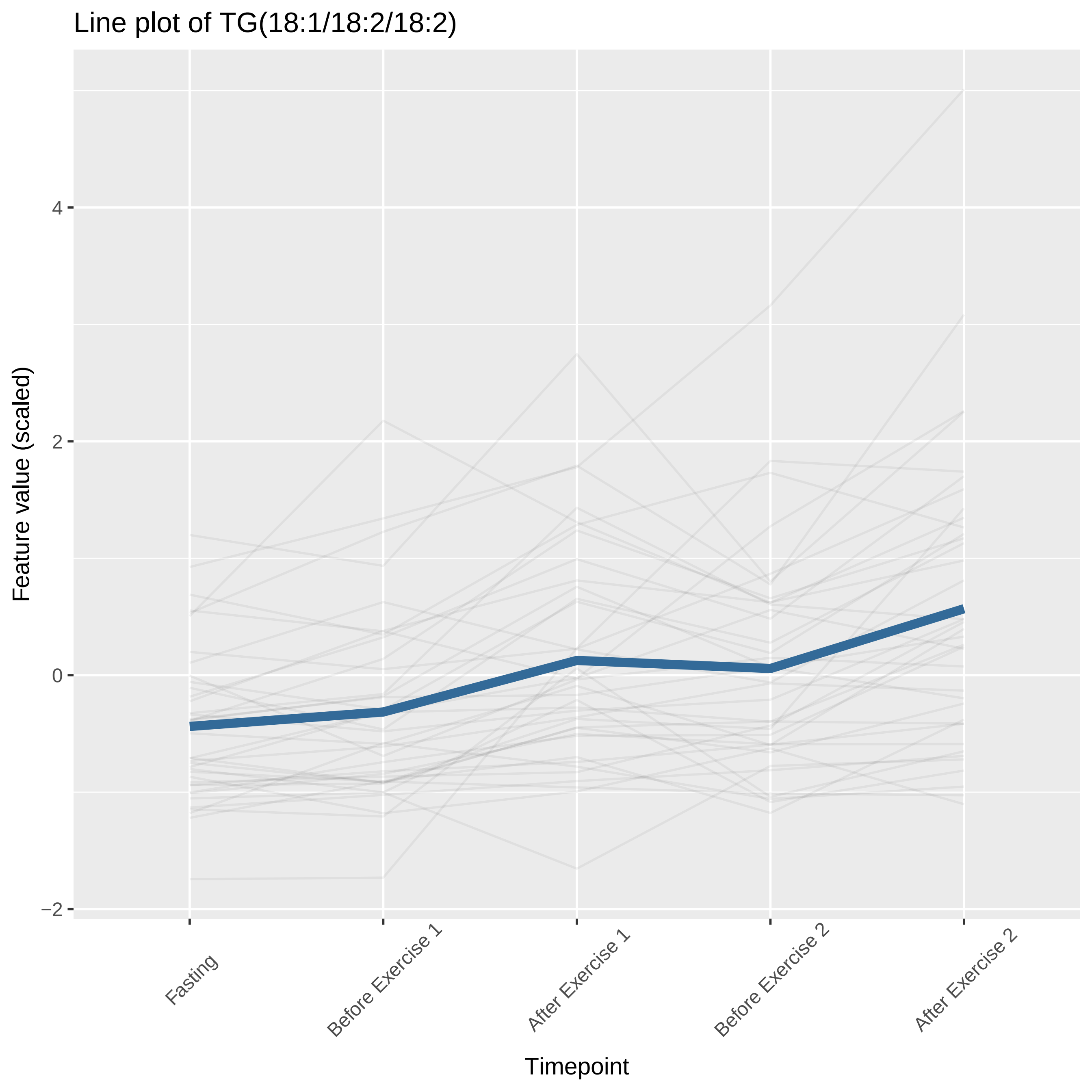

Line plot of TG(18:1/18:2/18:2)
Feature value (scaled)
4
2
0
−2
Fasting
Before Exercise 1
After Exercise 1
Before Exercise 2
After Exercise 2
Timepoint

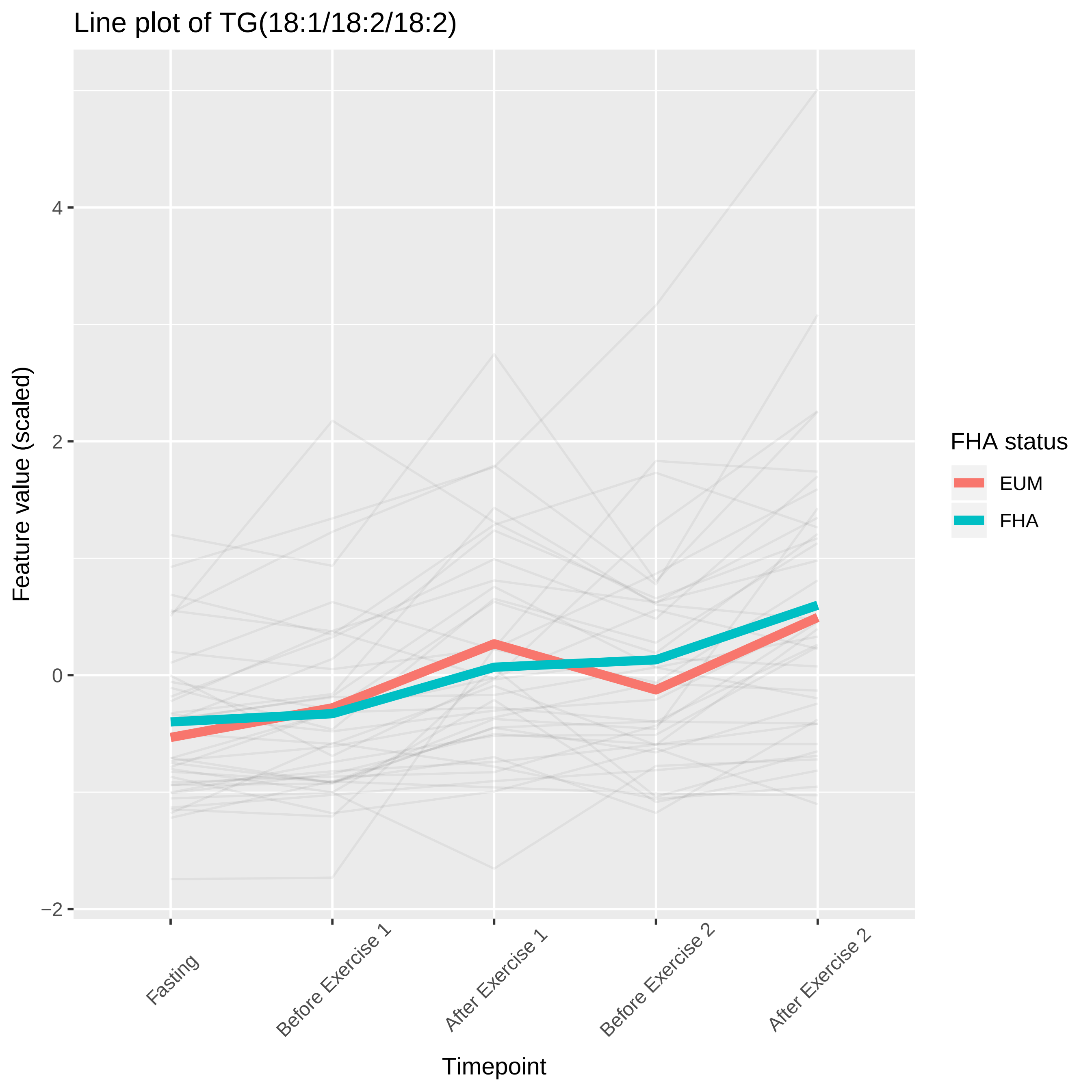
Line plot of TG(18:1/18:2/18:2)
Feature value (scaled)
4
2
0
−2
Fasting
Before Exercise 1
After Exercise 1
Before Exercise 2
After Exercise 2
Timepoint
FHA status
EUM
FHA

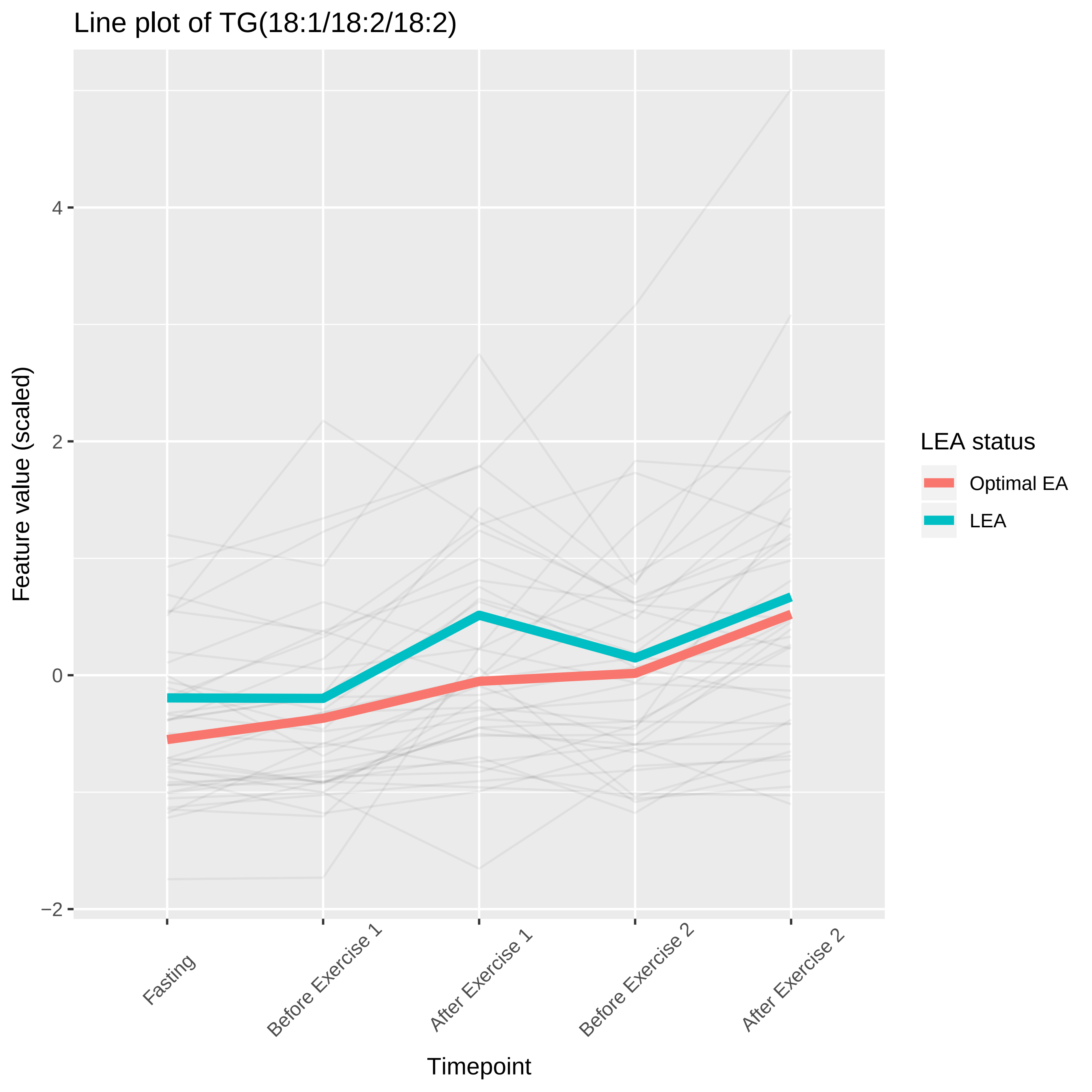

Line plot of TG(18:1/18:2/18:2)
Feature value (scaled)
4
2
0
−2
Fasting
Before Exercise 1
After Exercise 1
Before Exercise 2
After Exercise 2
Timepoint
LEA status
Optimal EA
LEA

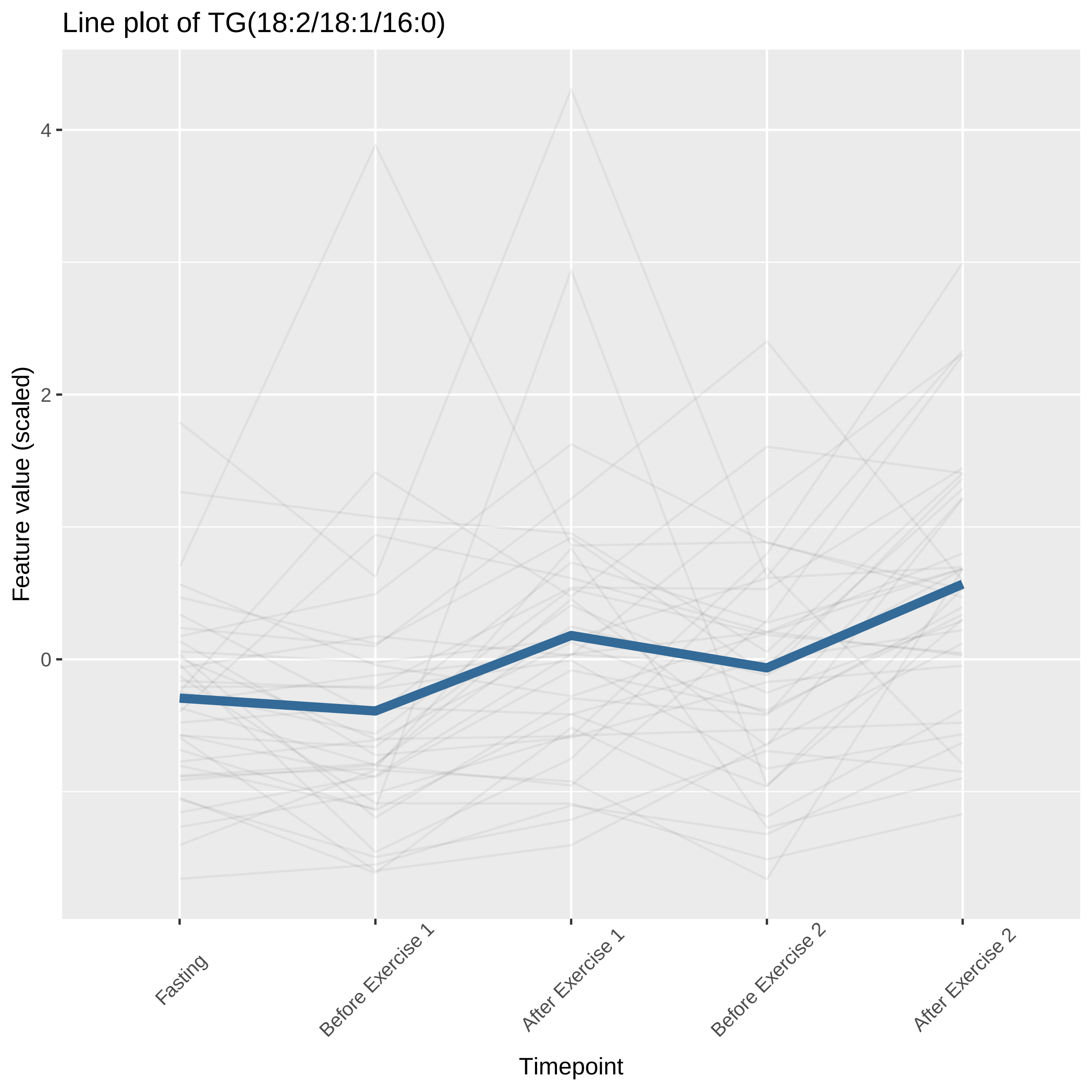

Line plot of TG(18:2/18:1/16:0)
Feature value (scaled)
4
2
0
Fasting
Before Exercise 1
After Exercise 1
Before Exercise 2
After Exercise 2
Timepoint

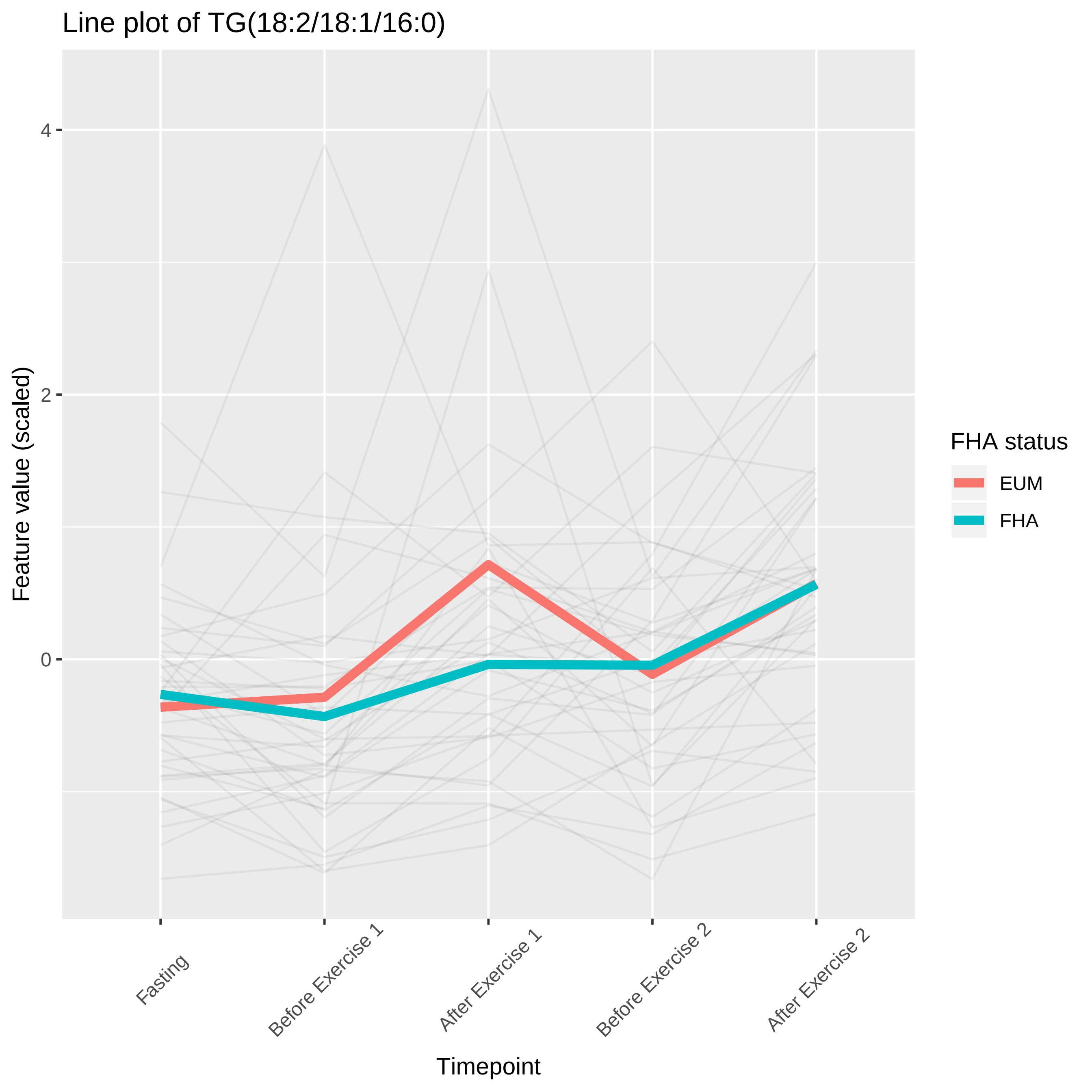

Line plot of TG(18:2/18:1/16:0)
Feature value (scaled)
4
2
0
Fasting
Before Exercise 1
After Exercise 1
Before Exercise 2
After Exercise 2
Timepoint
FHA status
EUM
FHA

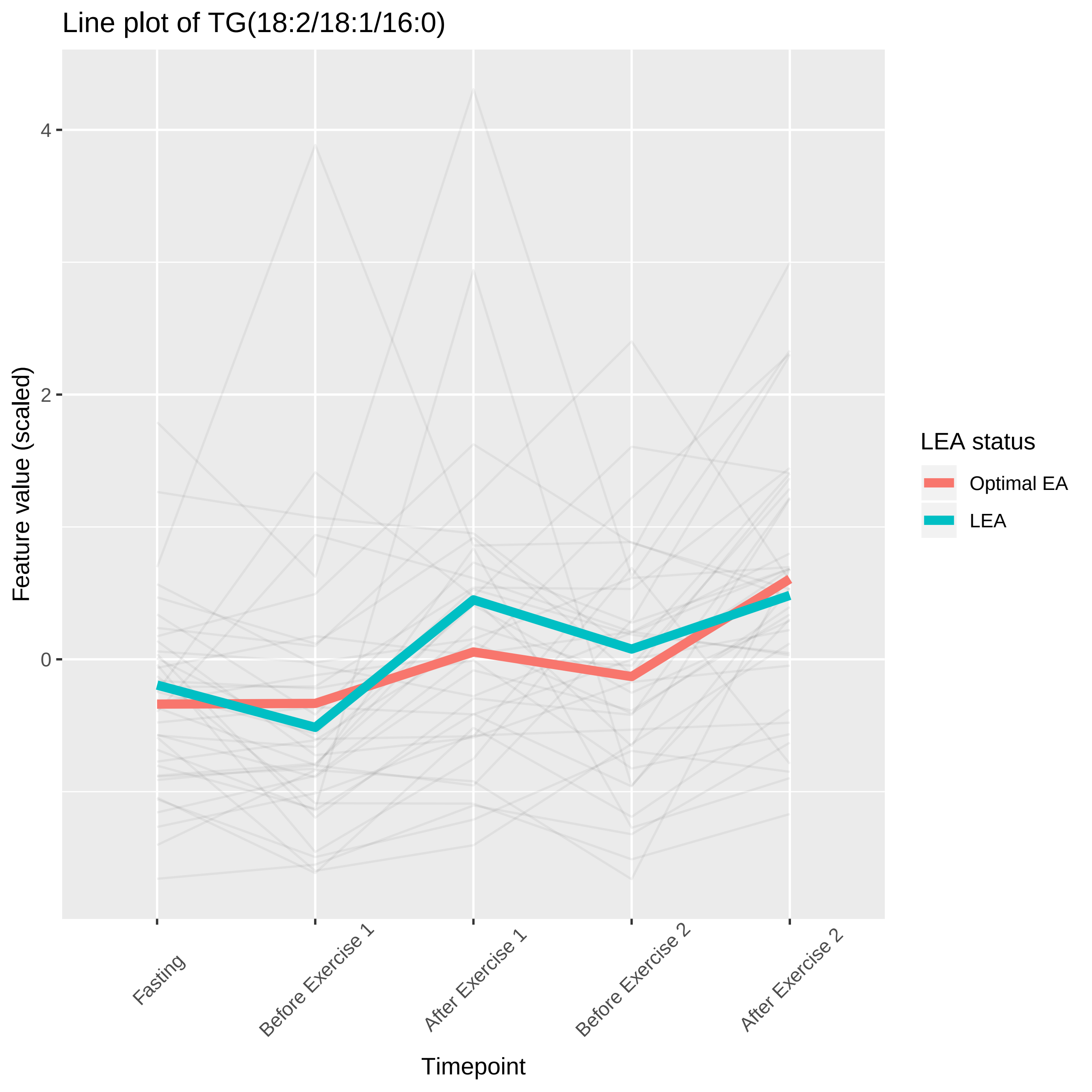
Line plot of TG(18:2/18:1/16:0)
Feature value (scaled)
4
2
0
Fasting
Before Exercise 1
After Exercise 1
Before Exercise 2
After Exercise 2
Timepoint
LEA status
Optimal EA
LEA

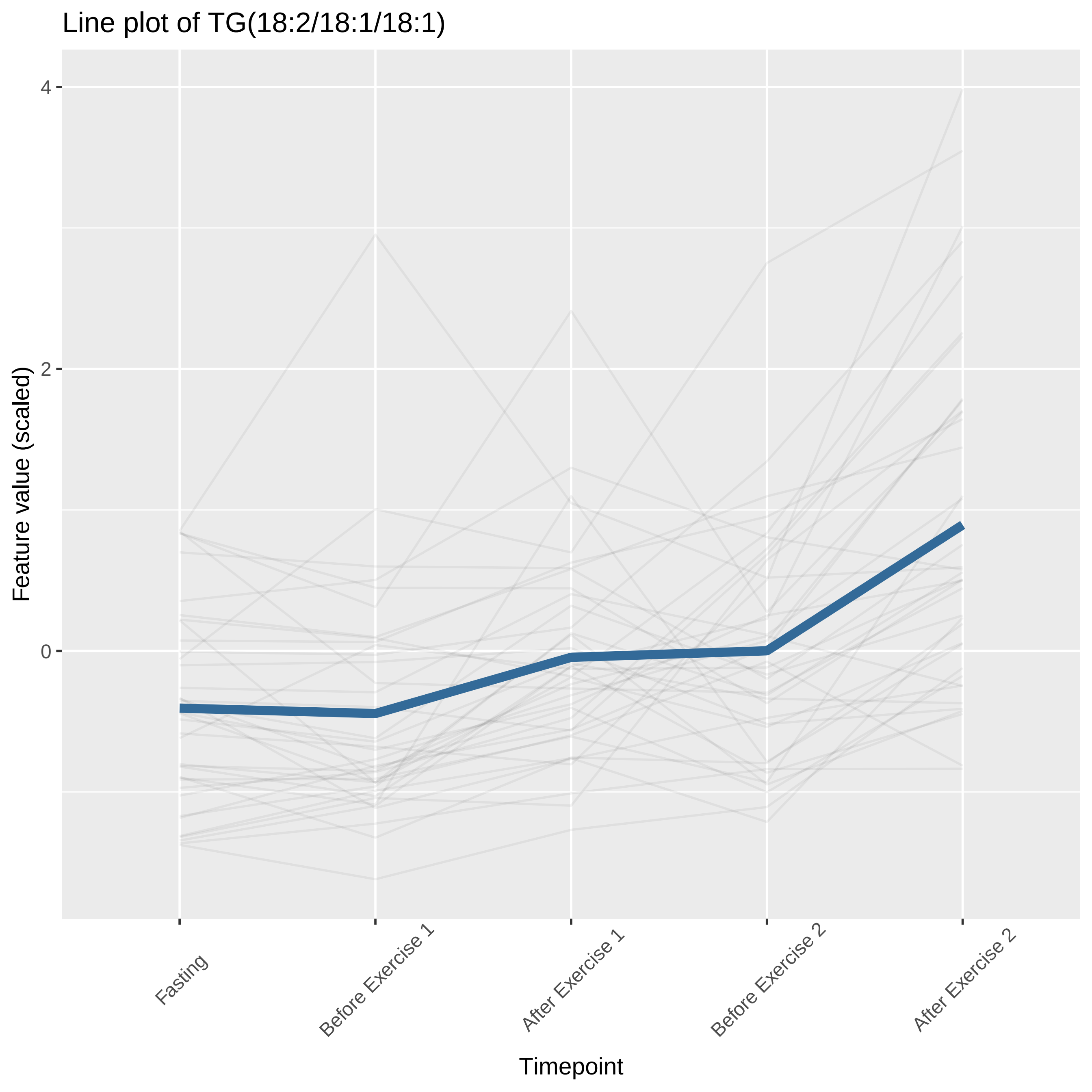

Line plot of TG(18:2/18:1/18:1)
4
2
0
Feature value (scaled)
Fasting
Before Exercise 1
After Exercise 1
Before Exercise 2
After Exercise 2
Timepoint

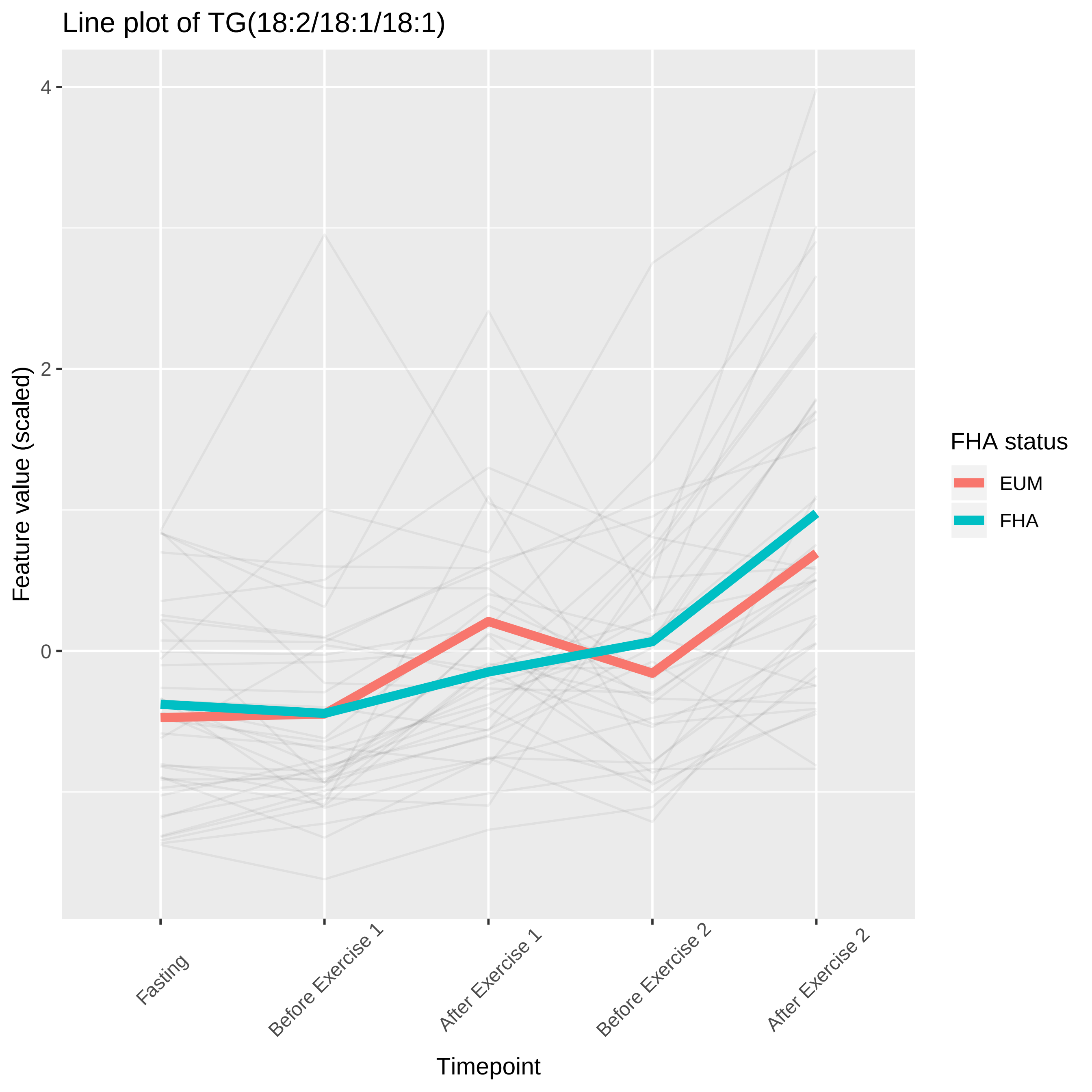

Line plot of TG(18:2/18:1/18:1)
Feature value (scaled)
4
2
0
Fasting
Before Exercise 1
After Exercise 1
Before Exercise 2
After Exercise 2
Timepoint
FHA status
EUM
FHA

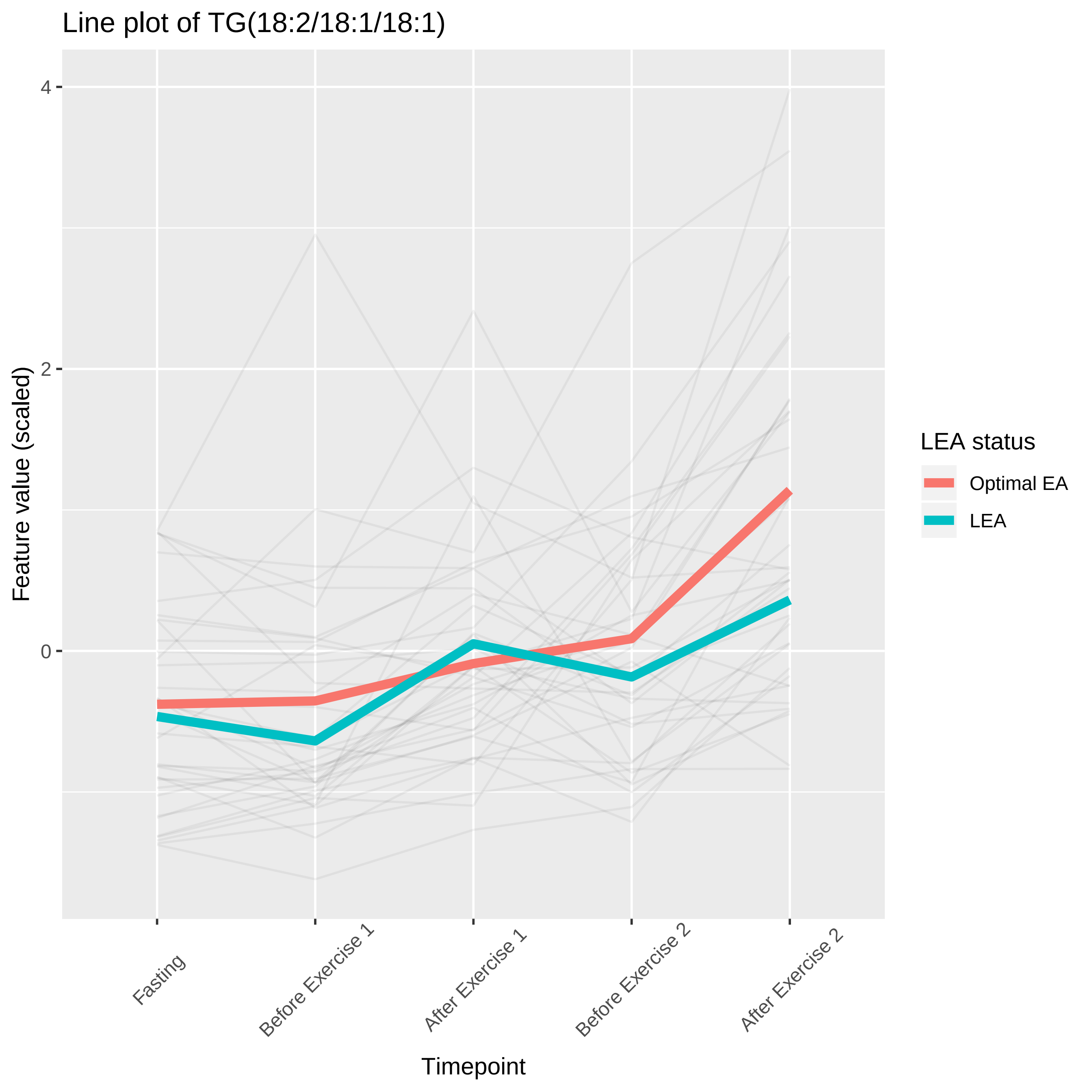

Line plot of TG(18:2/18:1/18:1)
Feature value (scaled)
4
2
0
Fasting
Before Exercise 1
After Exercise 1
Before Exercise 2
After Exercise 2
Timepoint
LEA status
Optimal EA
LEA

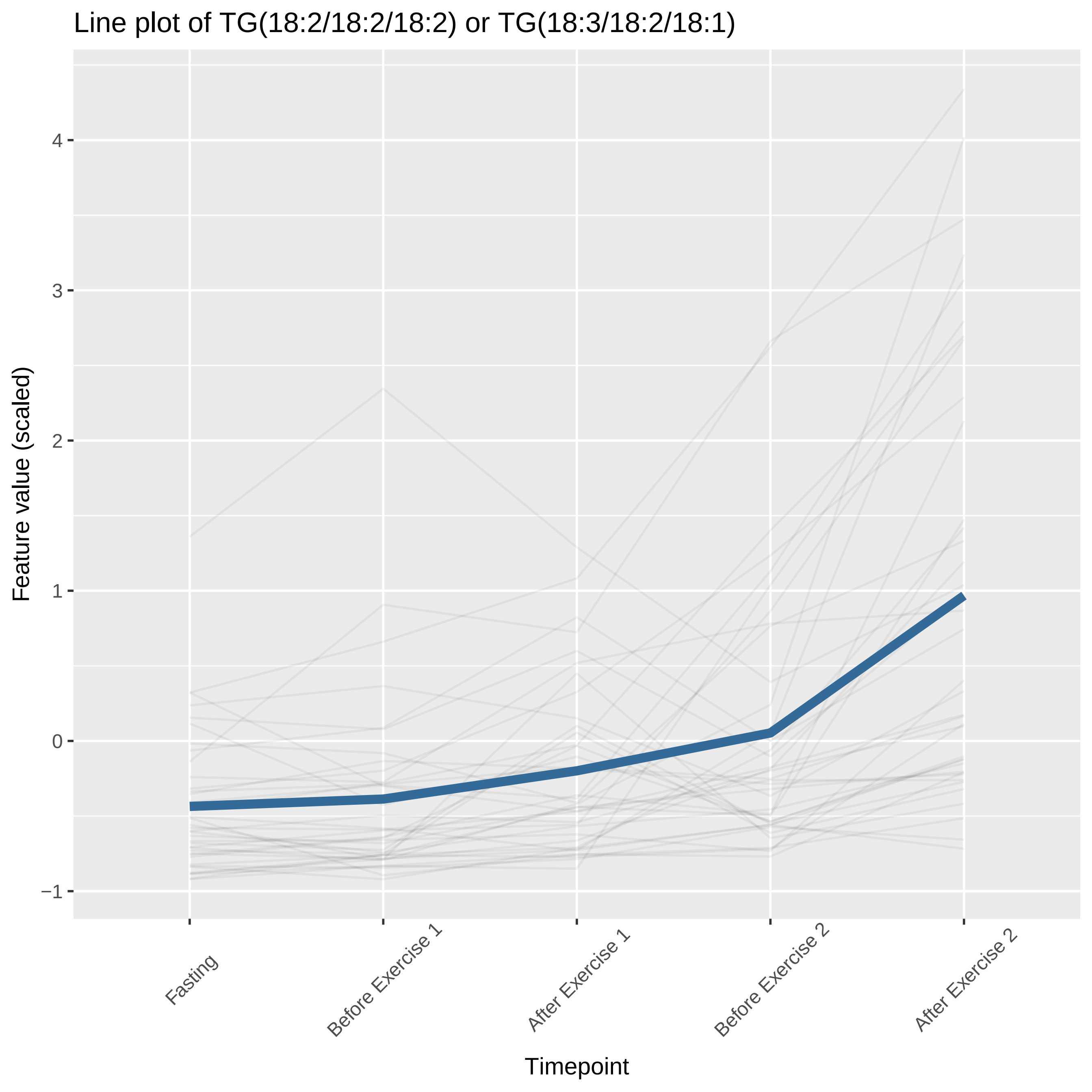

Line plot of TG(18:2/18:2/18:2) or TG(18:3/18:2/18:1)
Feature value (scaled)
4
3
2
1
0
−1
Fasting
Before Exercise 1
After Exercise 1
Before Exercise 2
After Exercise 2
Timepoint

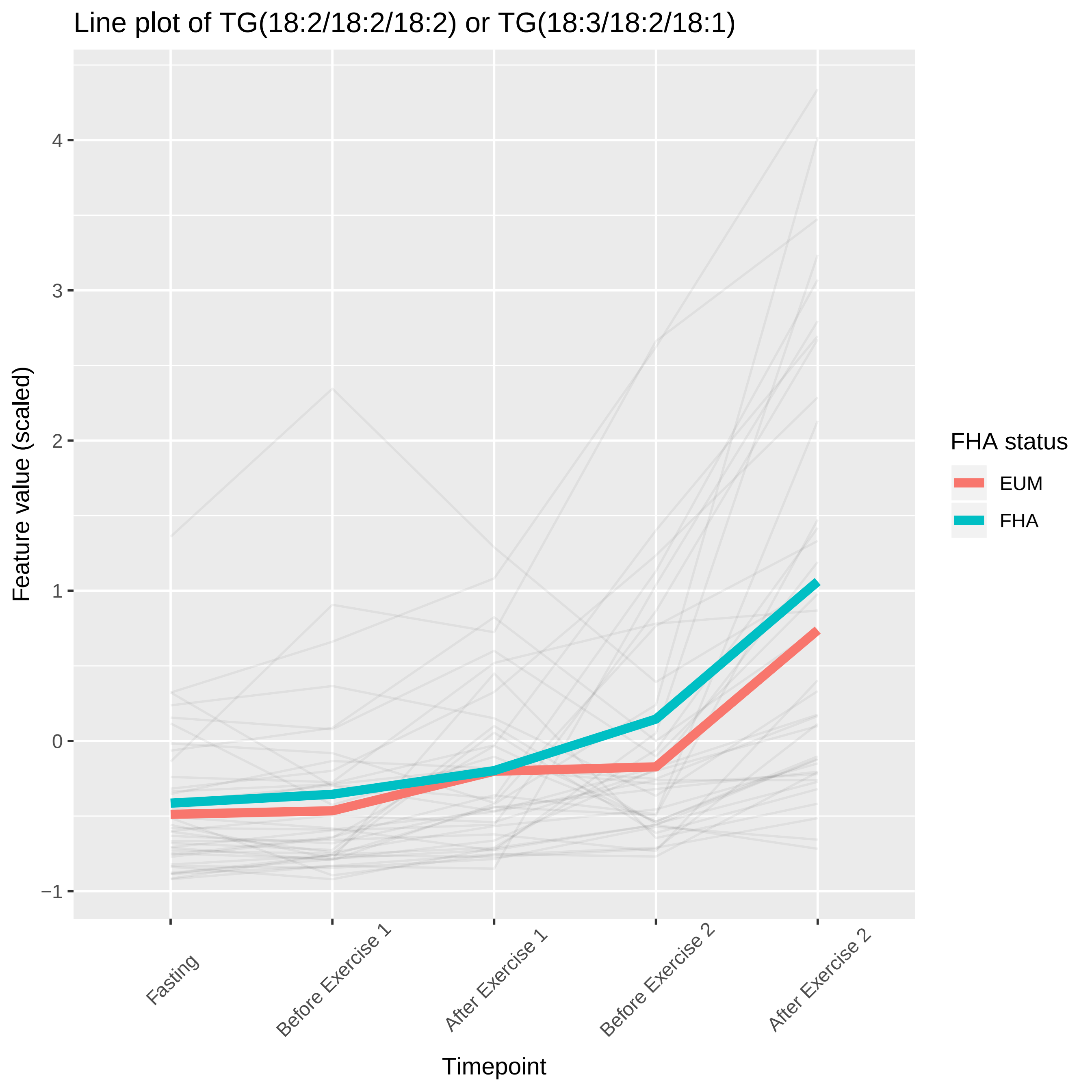
Line plot of TG(18:2/18:2/18:2) or TG(18:3/18:2/18:1)
Feature value (scaled)
4
3
2
1
0
−1
Fasting
Before Exercise 1
After Exercise 1
Before Exercise 2
After Exercise 2
Timepoint
FHA status
EUM
FHA

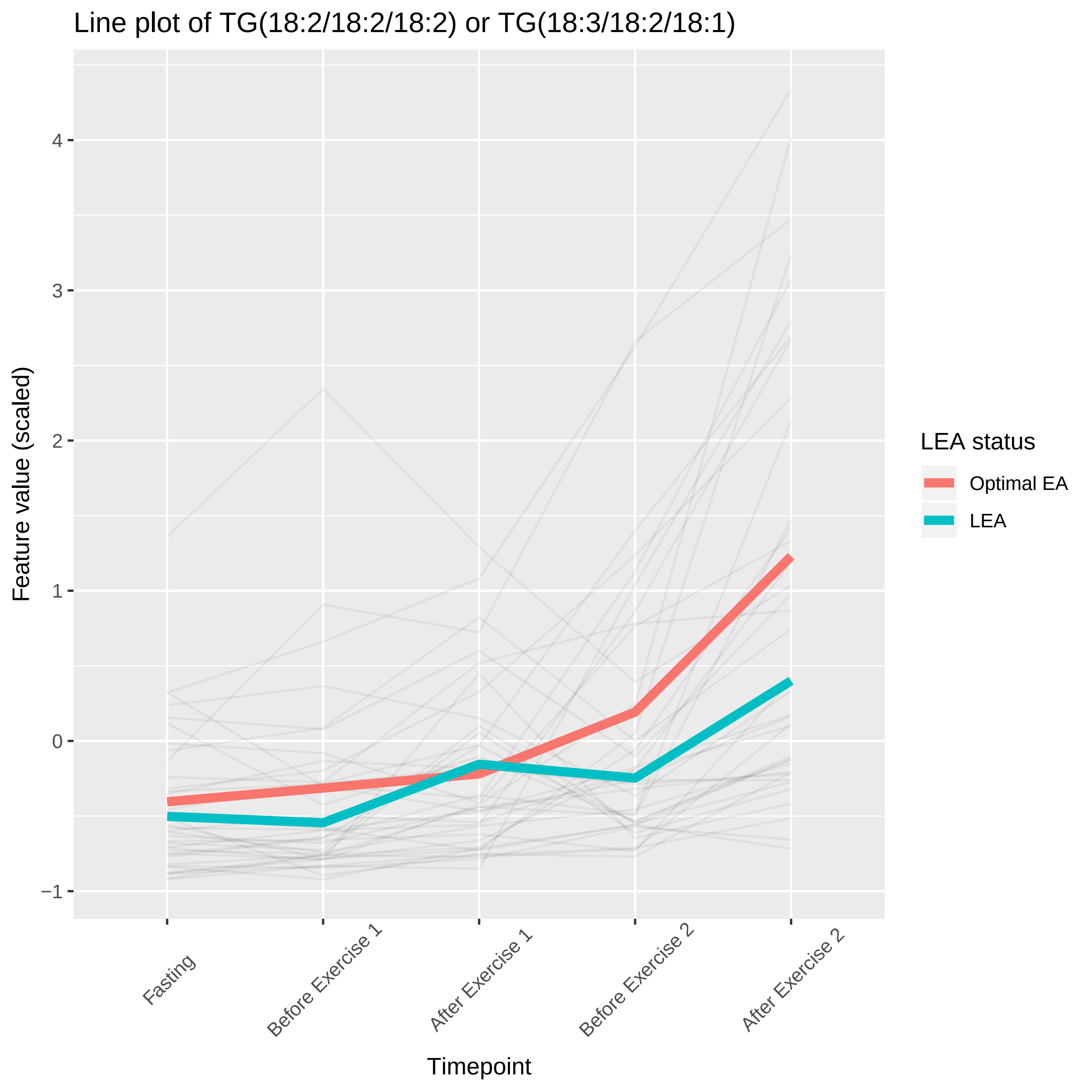

Line plot of TG(18:2/18:2/18:2) or TG(18:3/18:2/18:1)
Feature value (scaled)
4
3
2
1
0
−1
Fasting
Before Exercise 1
After Exercise 1
Before Exercise 2
After Exercise 2
Timepoint
LEA status
Optimal EA
LEA

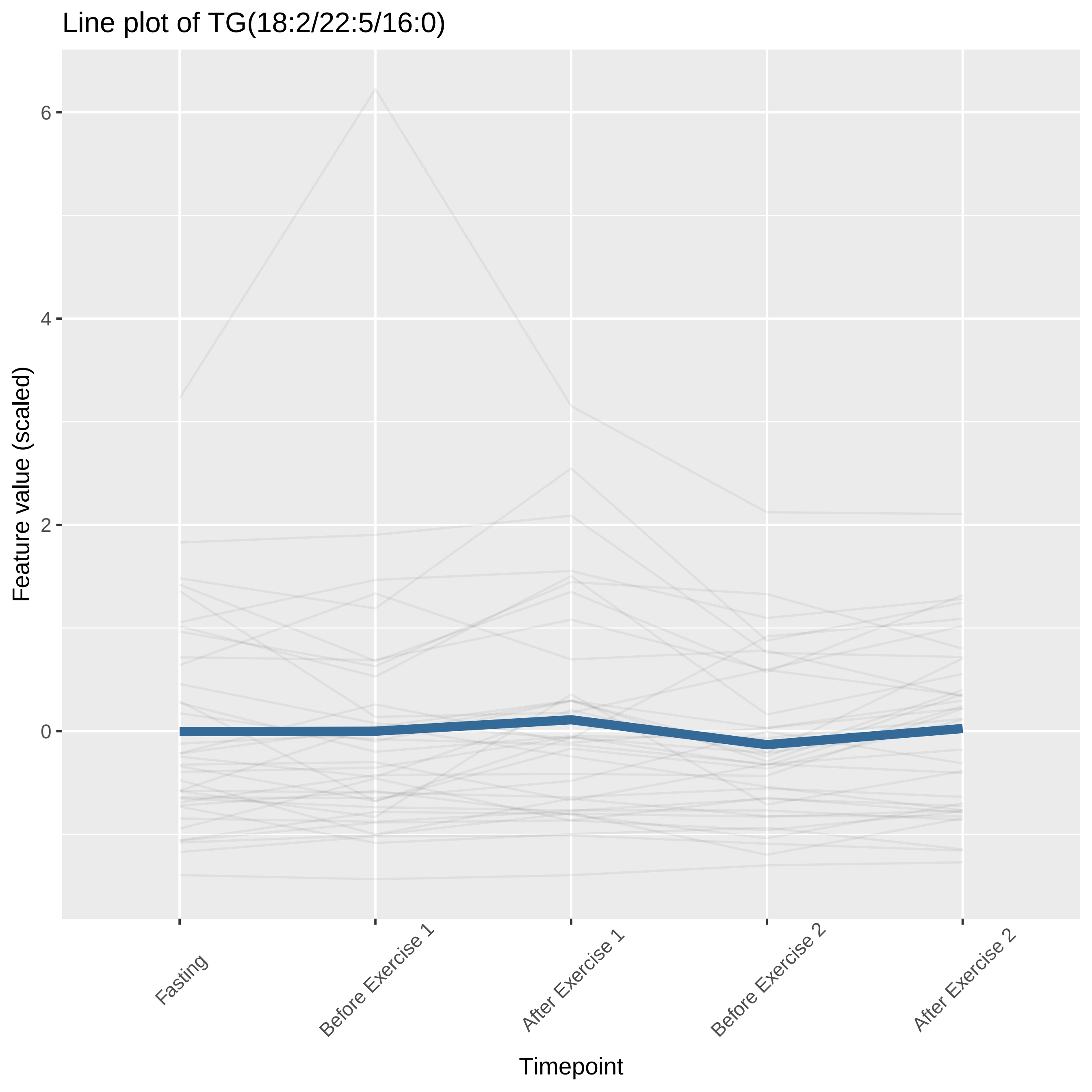
Line plot of TG(18:2/22:5/16:0)
Feature value (scaled)
6
4
2
0
Fasting
Before Exercise 1
After Exercise 1
Before Exercise 2
After Exercise 2
Timepoint

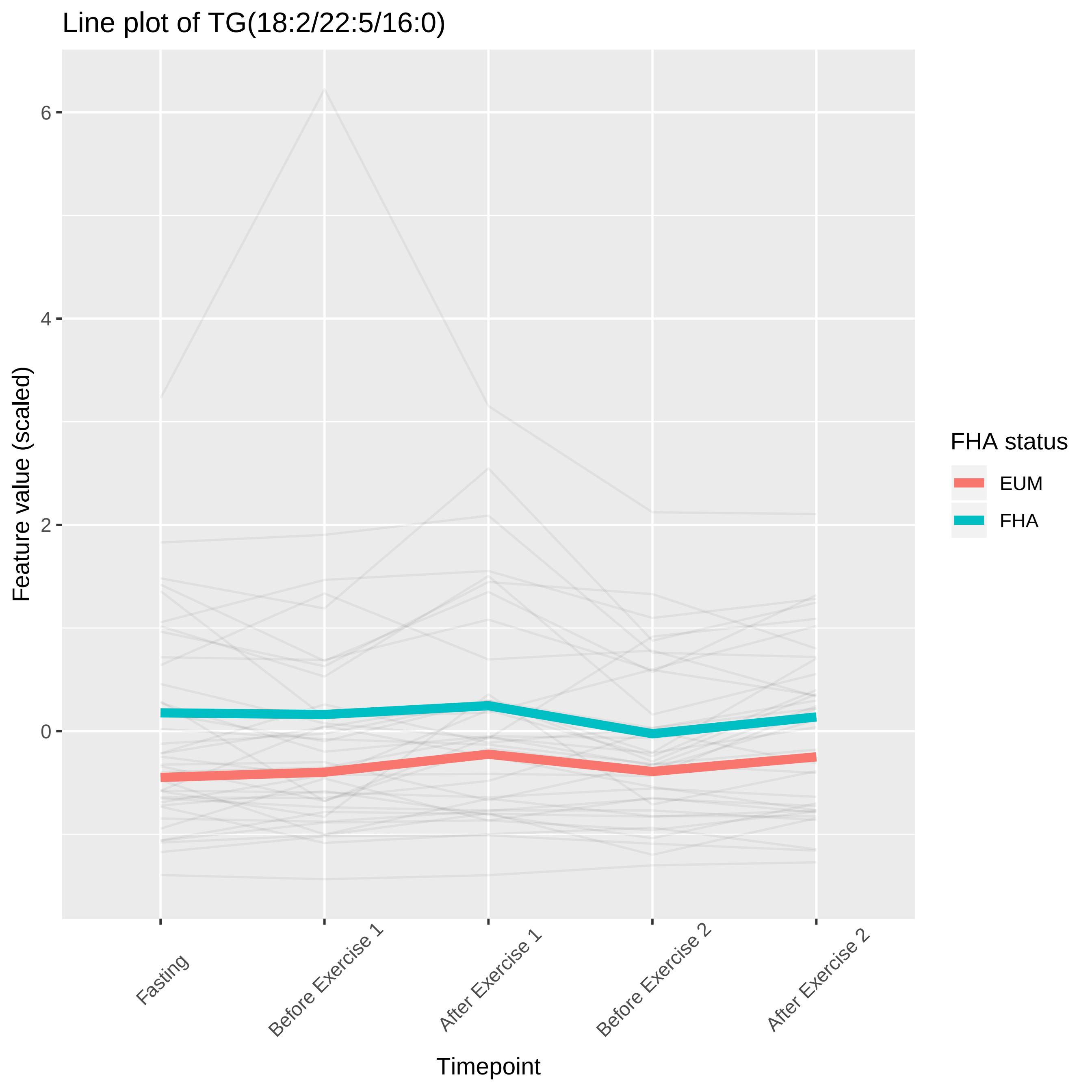

Line plot of TG(18:2/22:5/16:0)
Feature value (scaled)
6
4
2
0
Fasting
Before Exercise 1
After Exercise 1
Before Exercise 2
After Exercise 2
Timepoint
FHA status
EUM
FHA

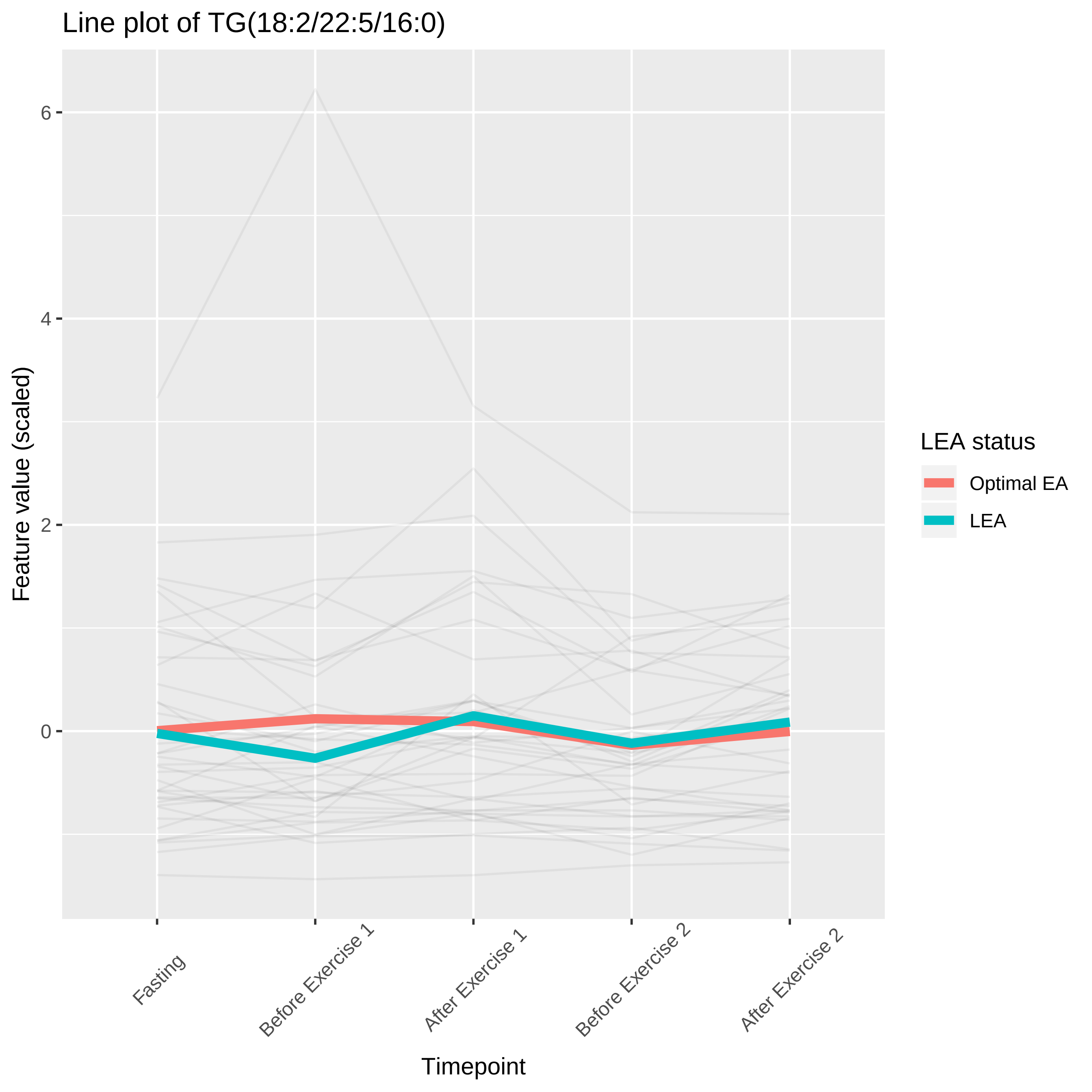

Line plot of TG(18:2/22:5/16:0)
Feature value (scaled)
6
4
2
0
Fasting
Before Exercise 1
After Exercise 1
Before Exercise 2
After Exercise 2
Timepoint
LEA status
Optimal EA
LEA

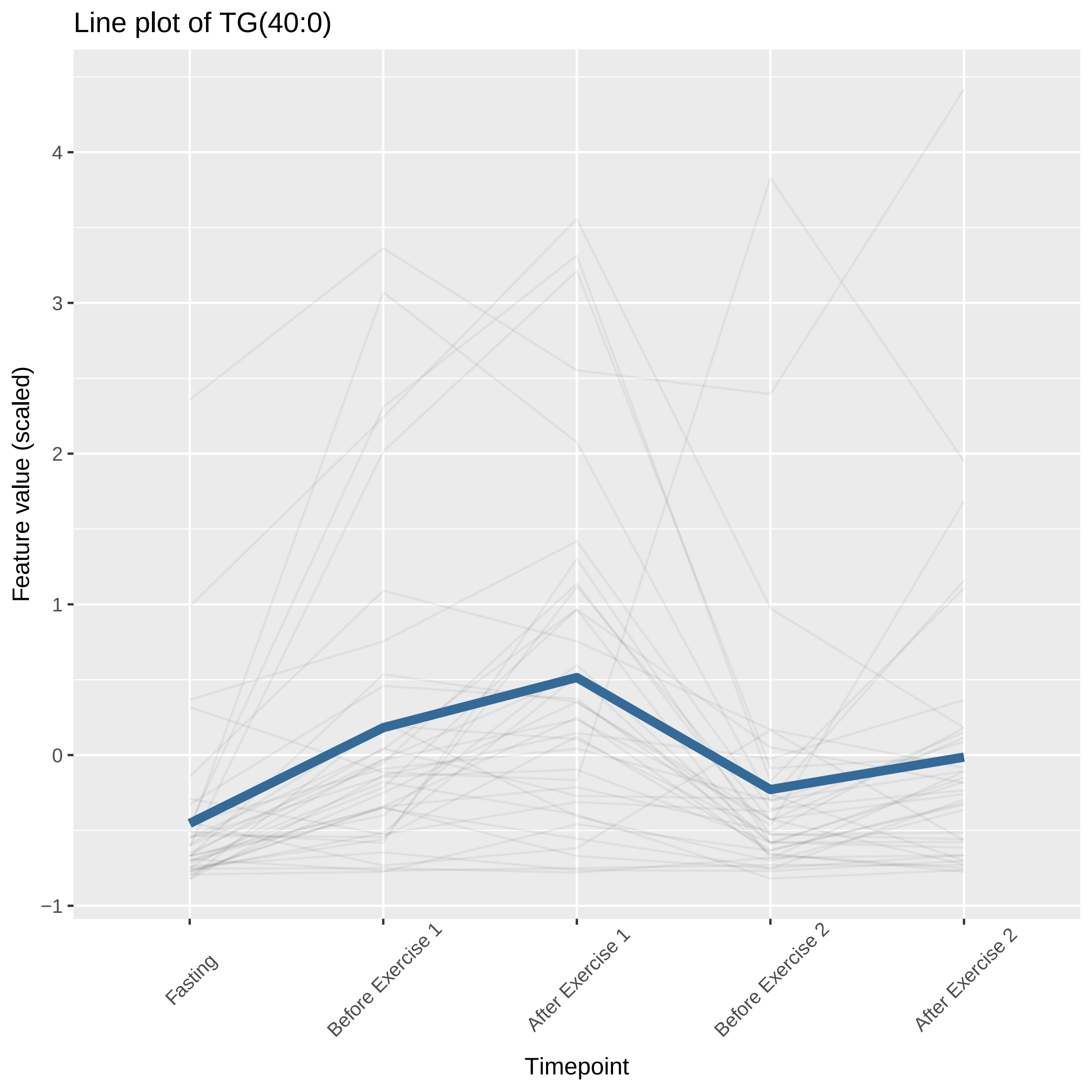

Line plot of TG(40:0)
Feature value (scaled)
4
3
2
1
0
−1
Fasting
Before Exercise 1
After Exercise 1
Before Exercise 2
After Exercise 2
Timepoint

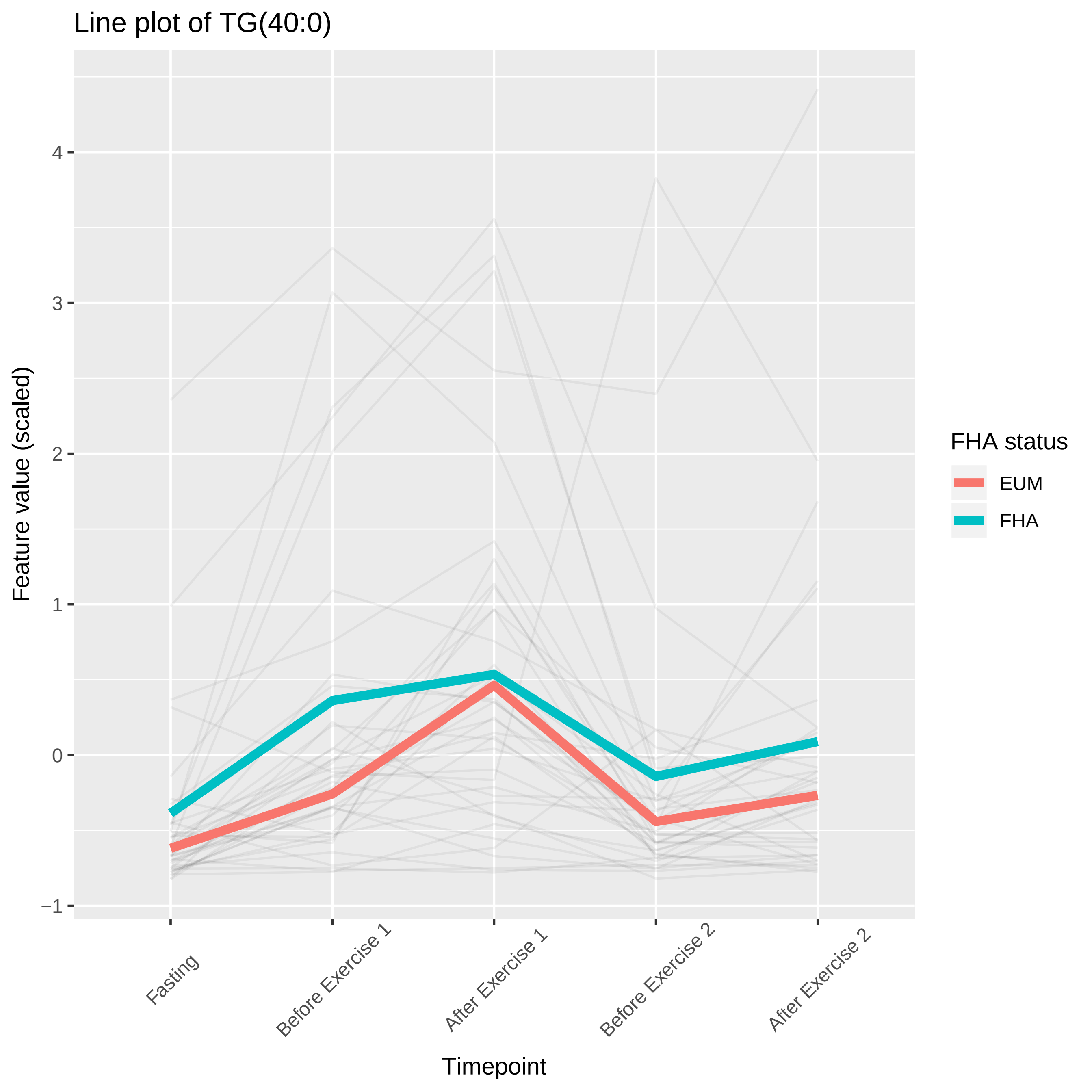

Line plot of TG(40:0)
Feature value (scaled)
4
3
2
1
0
−1
Fasting
Before Exercise 1
After Exercise 1
Before Exercise 2
After Exercise 2
Timepoint
FHA status
EUM
FHA

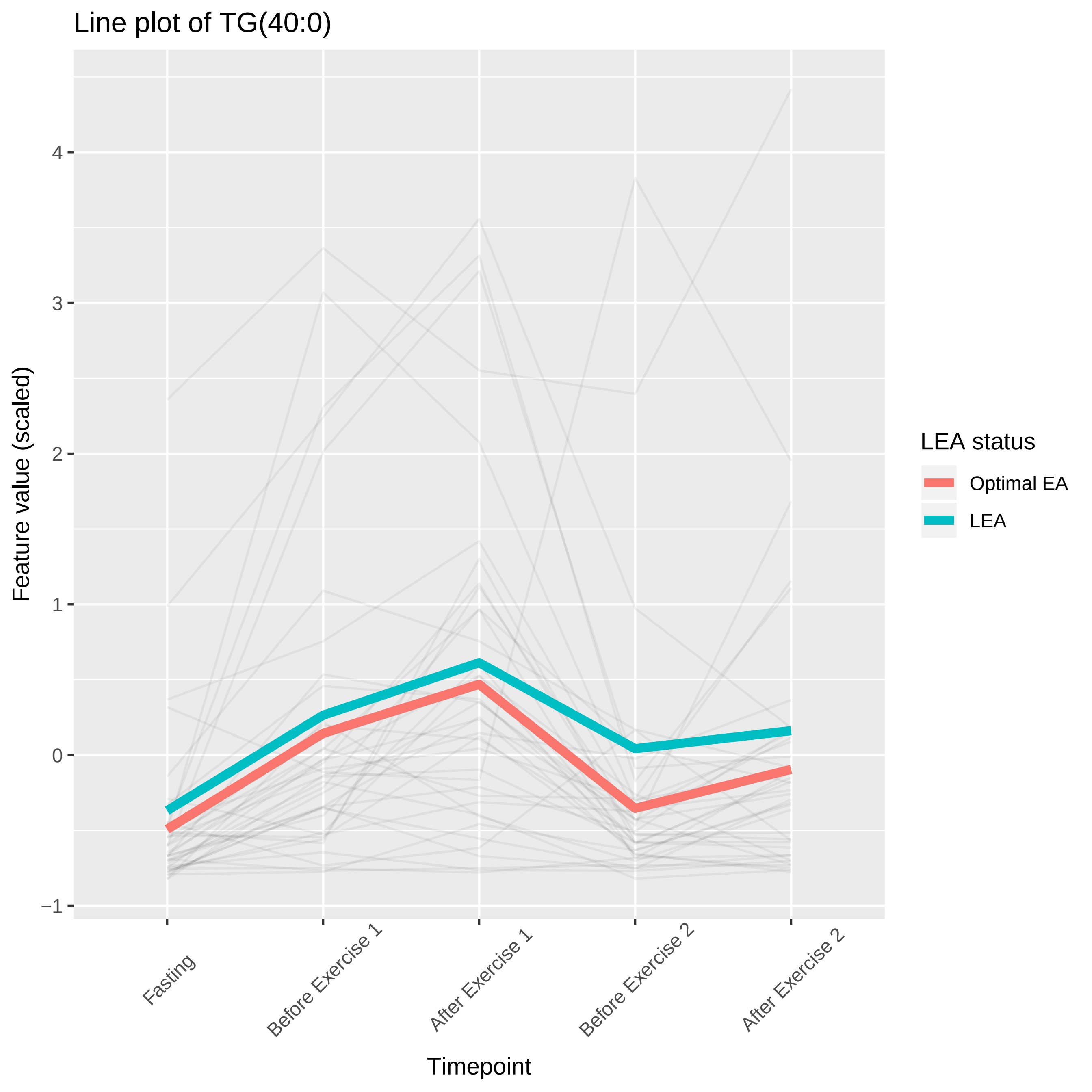

Line plot of TG(40:0)
Feature value (scaled)
4
3
2
1
0
−1
Fasting
Before Exercise 1
After Exercise 1
Before Exercise 2
After Exercise 2
Timepoint
LEA status
Optimal EA
LEA

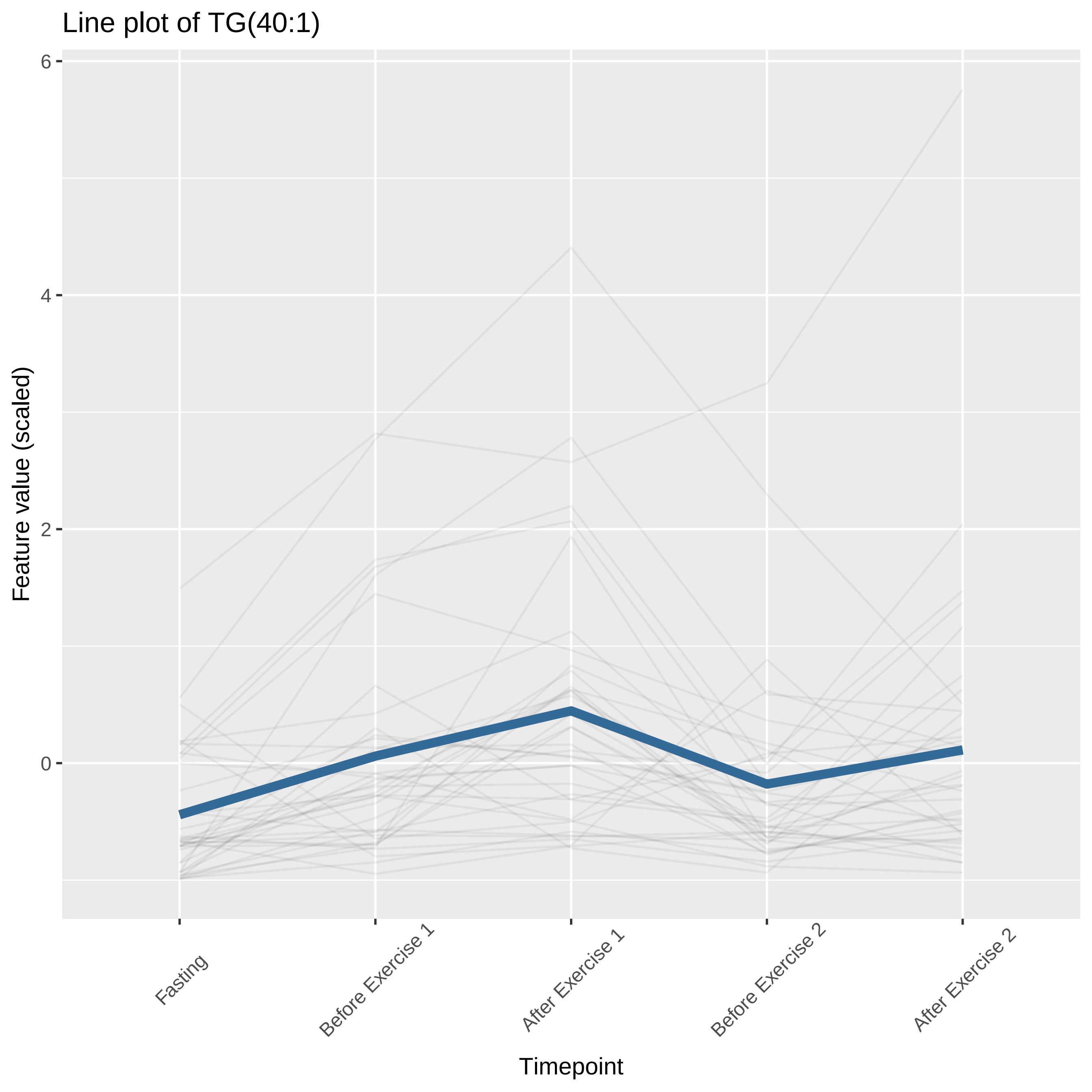

Line plot of TG(40:1)
Feature value (scaled)
6
4
2
0
Fasting
Before Exercise 1
After Exercise 1
Before Exercise 2
After Exercise 2
Timepoint

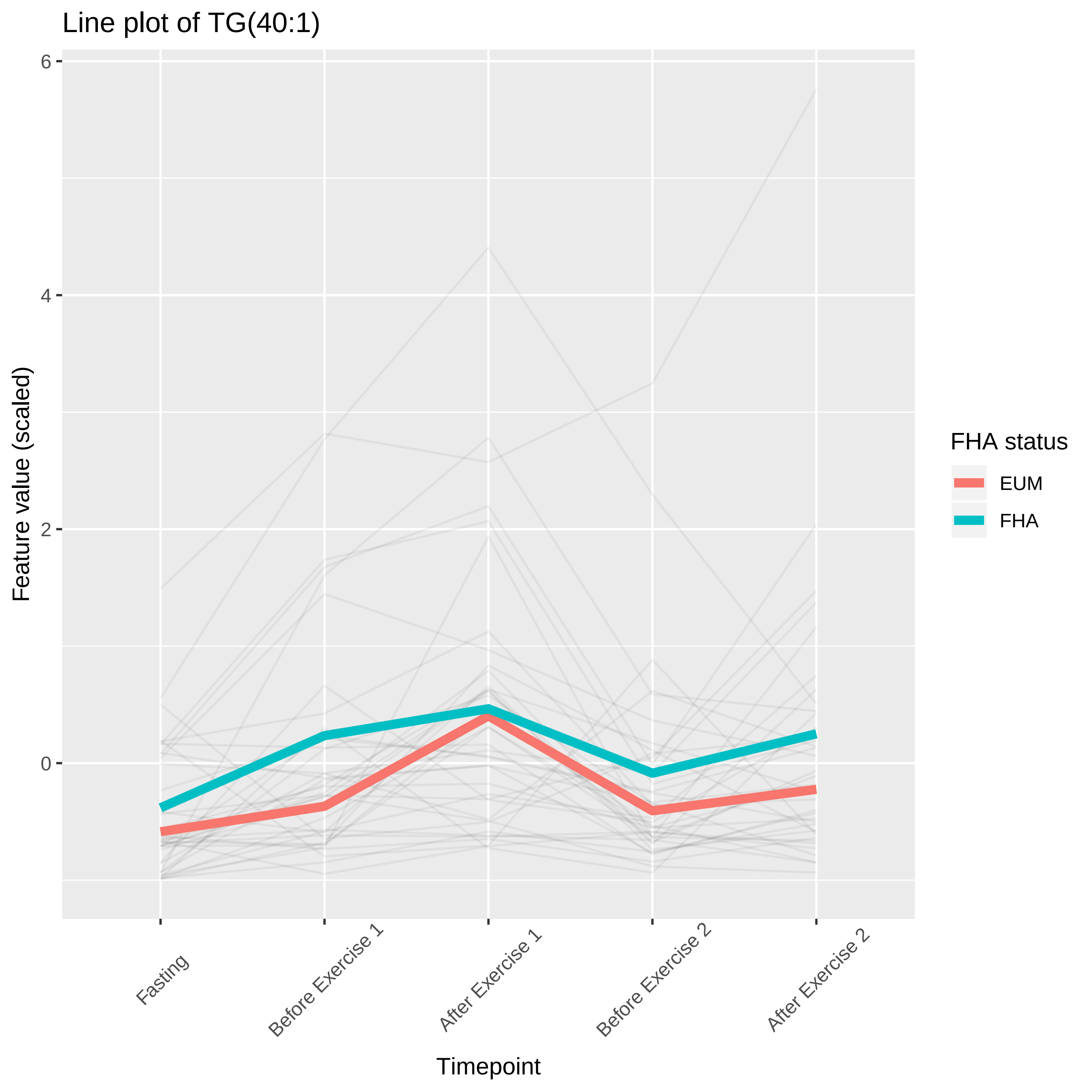
Line plot of TG(40:1)
Feature value (scaled)
6
4
2
0
Fasting
Before Exercise 1
After Exercise 1
Before Exercise 2
After Exercise 2
Timepoint
FHA status
EUM
FHA

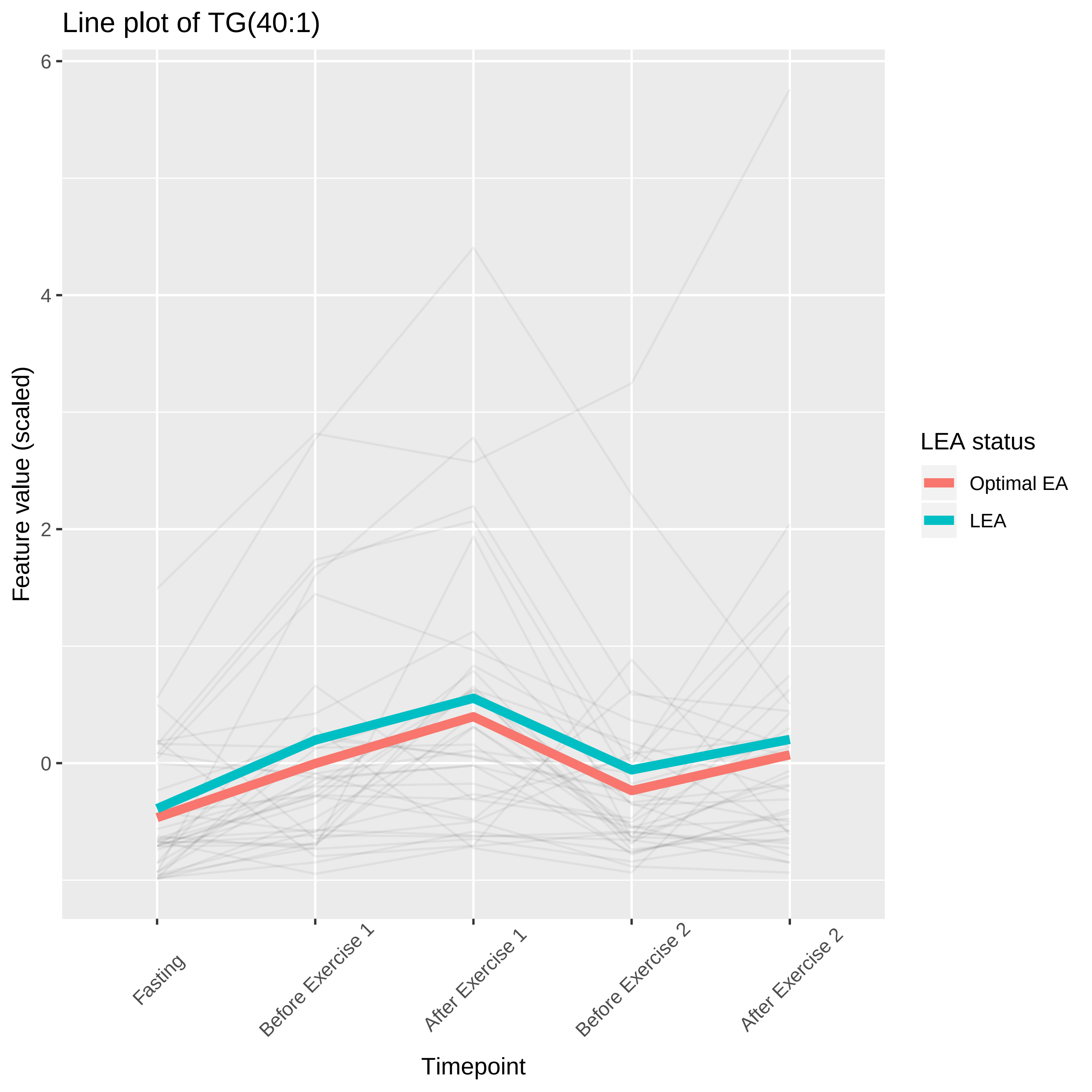

Line plot of TG(40:1)
Feature value (scaled)
6
4
2
0
Fasting
Before Exercise 1
After Exercise 1
Before Exercise 2
After Exercise 2
Timepoint
LEA status
Optimal EA
LEA

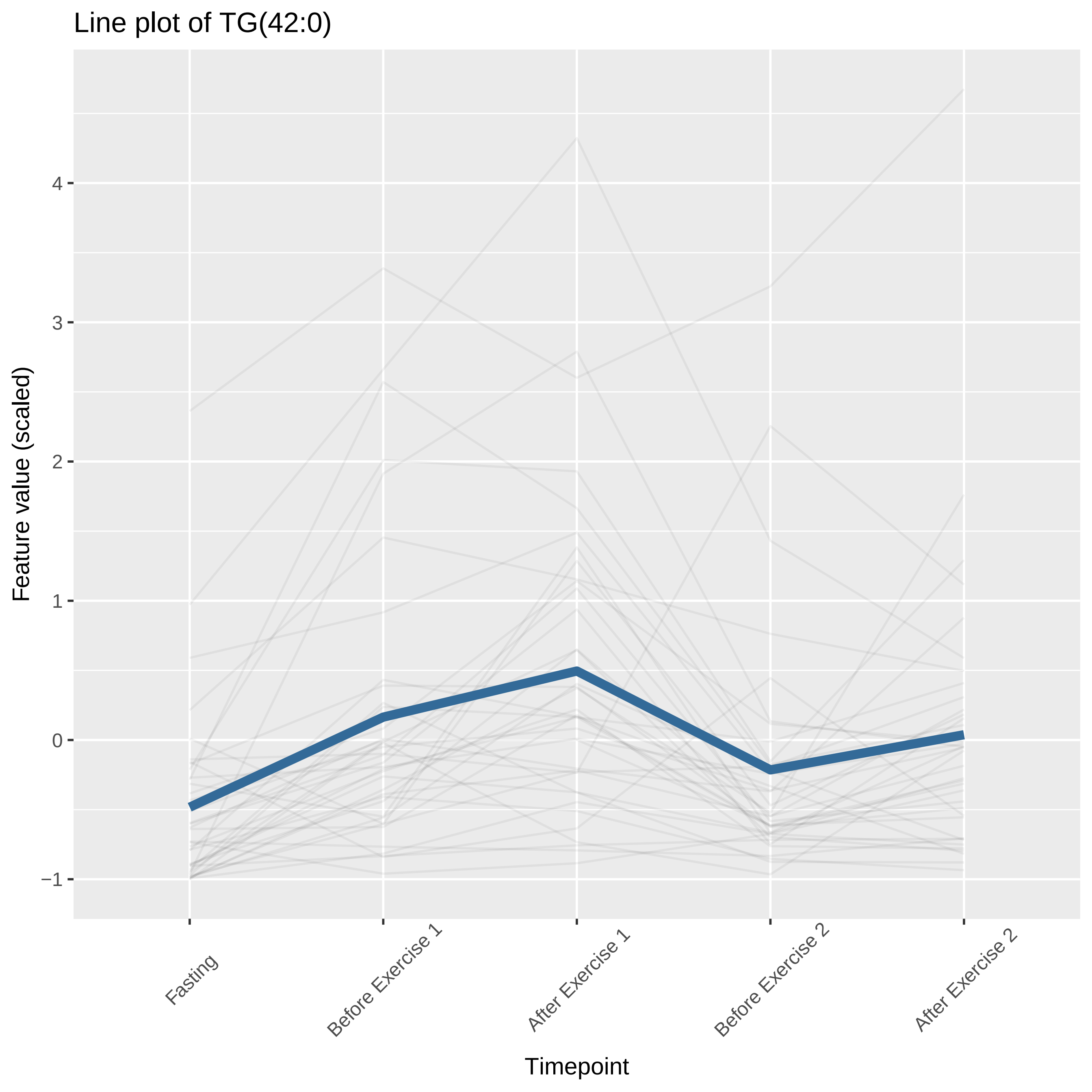
Line plot of TG(42:0)
Feature value (scaled)
4
3
2
1
0
−1
Fasting
Before Exercise 1
After Exercise 1
Before Exercise 2
After Exercise 2
Timepoint

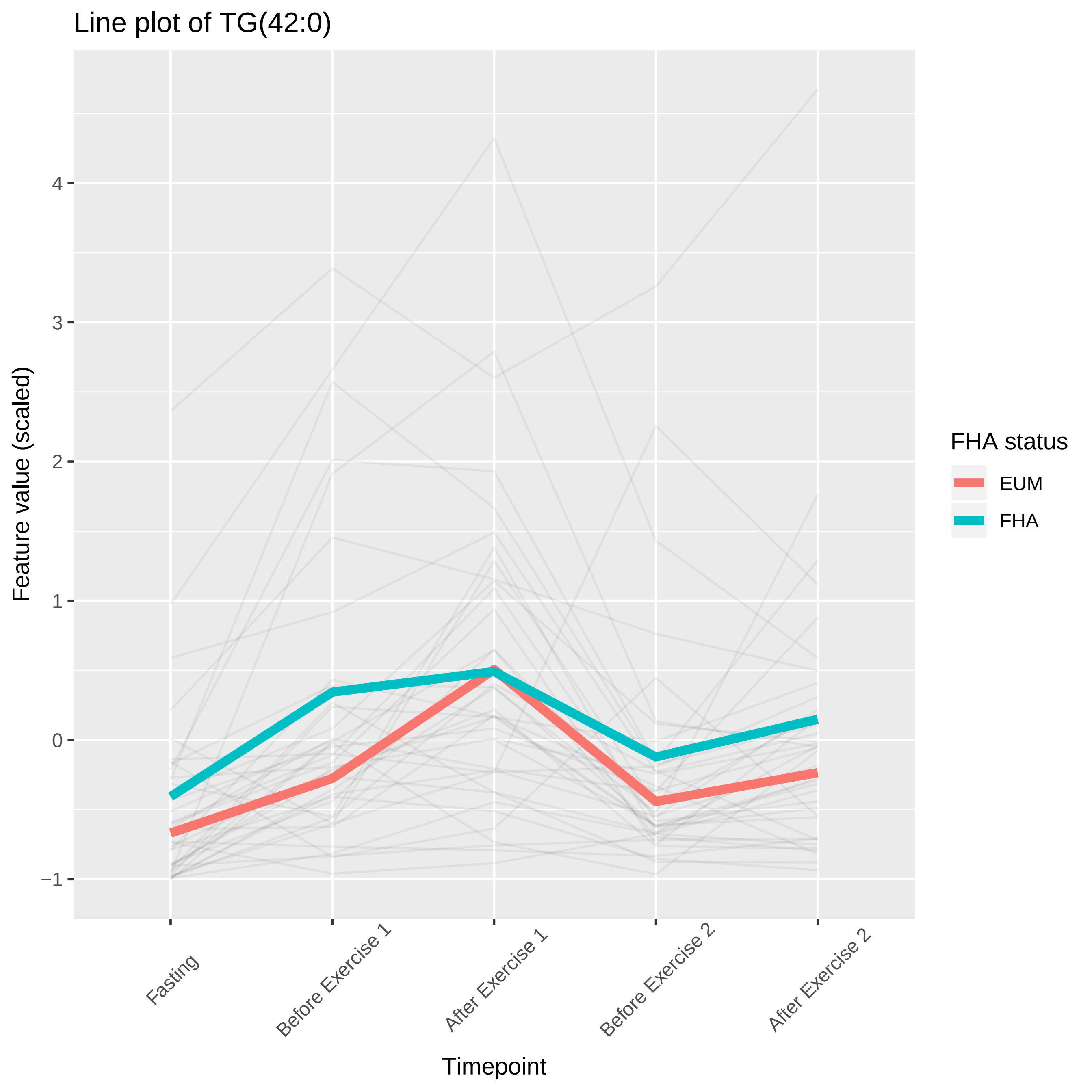
Line plot of TG(42:0)
Feature value (scaled)
4
3
2
1
0
−1
Fasting
Before Exercise 1
After Exercise 1
Before Exercise 2
After Exercise 2
Timepoint
FHA status
EUM
FHA

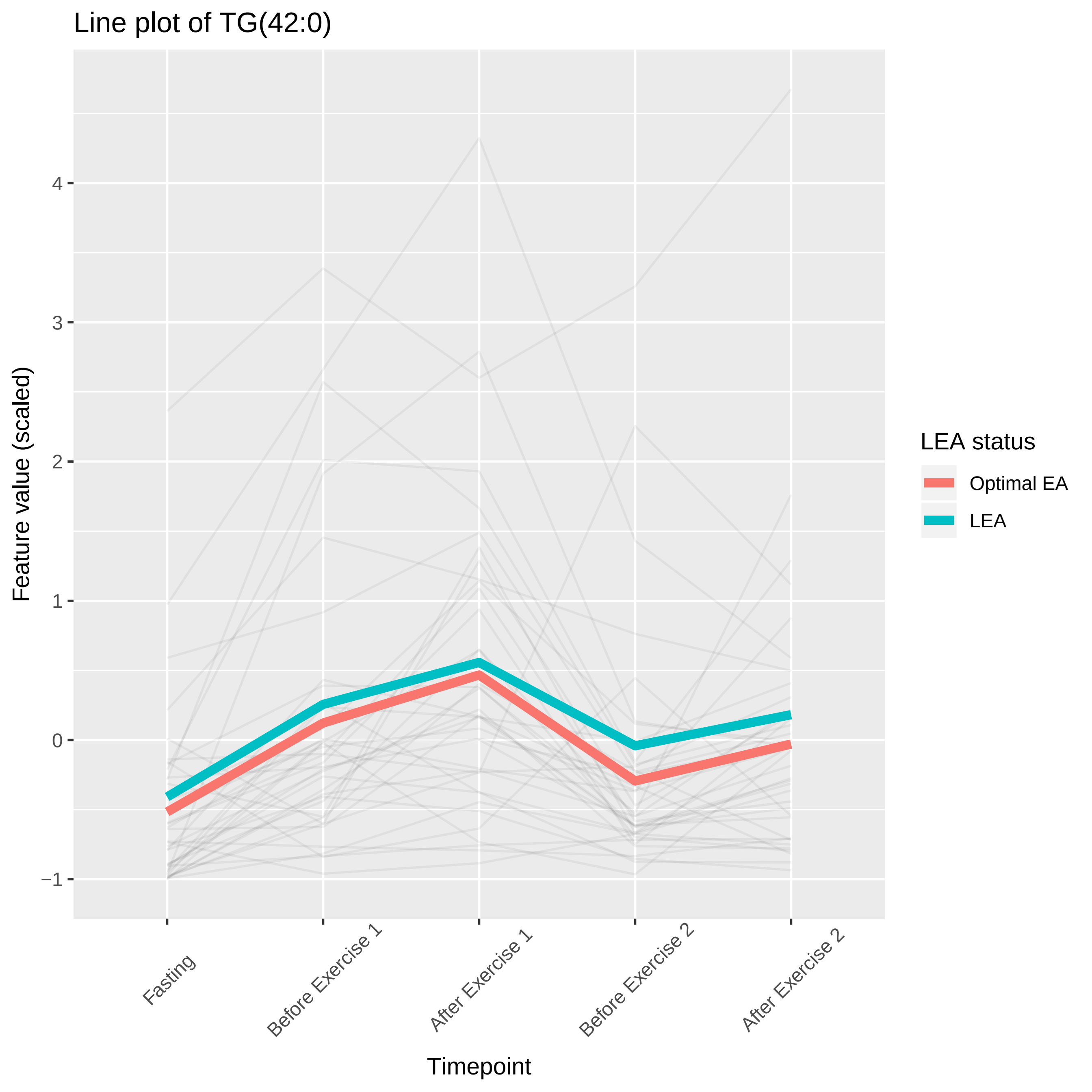
Line plot of TG(42:0)
Feature value (scaled)
4
3
2
1
0
−1
Fasting
Before Exercise 1
After Exercise 1
Before Exercise 2
After Exercise 2
Timepoint
LEA status
Optimal EA
LEA

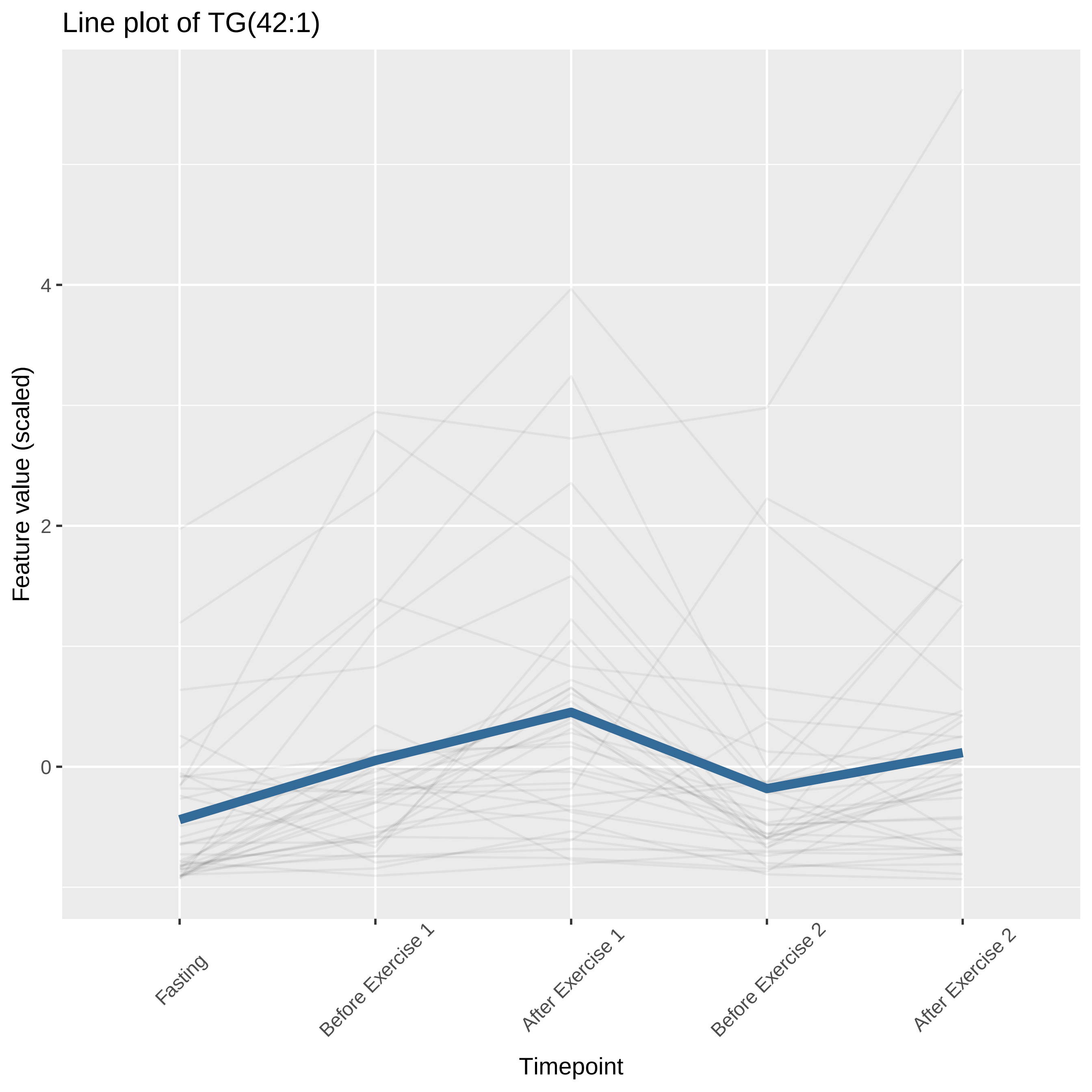
Line plot of TG(42:1)
Feature value (scaled)
4
2
0
Fasting
Before Exercise 1
After Exercise 1
Before Exercise 2
After Exercise 2
Timepoint

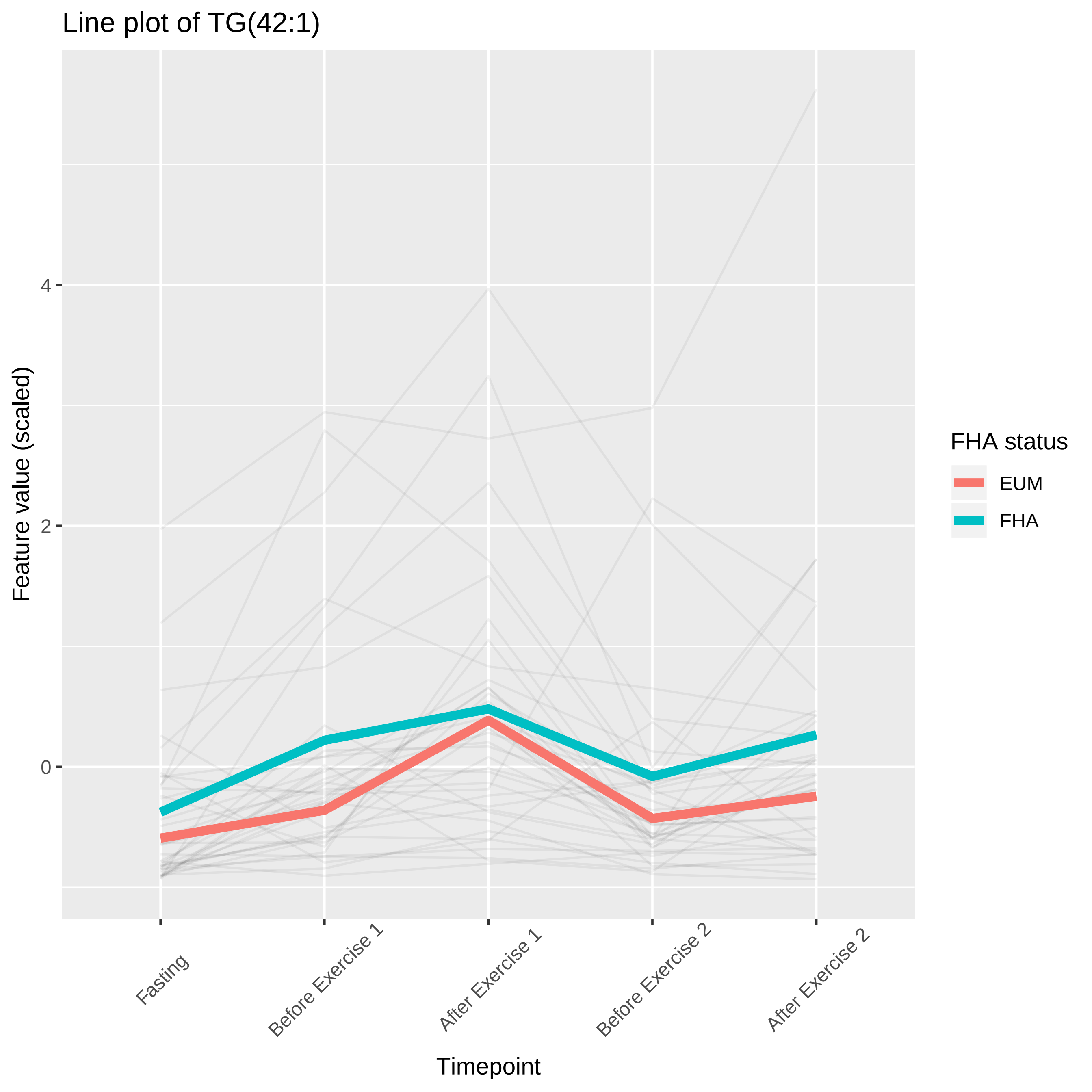

Line plot of TG(42:1)
Feature value (scaled)
4
2
0
Fasting
Before Exercise 1
After Exercise 1
Before Exercise 2
After Exercise 2
Timepoint
FHA status
EUM
FHA

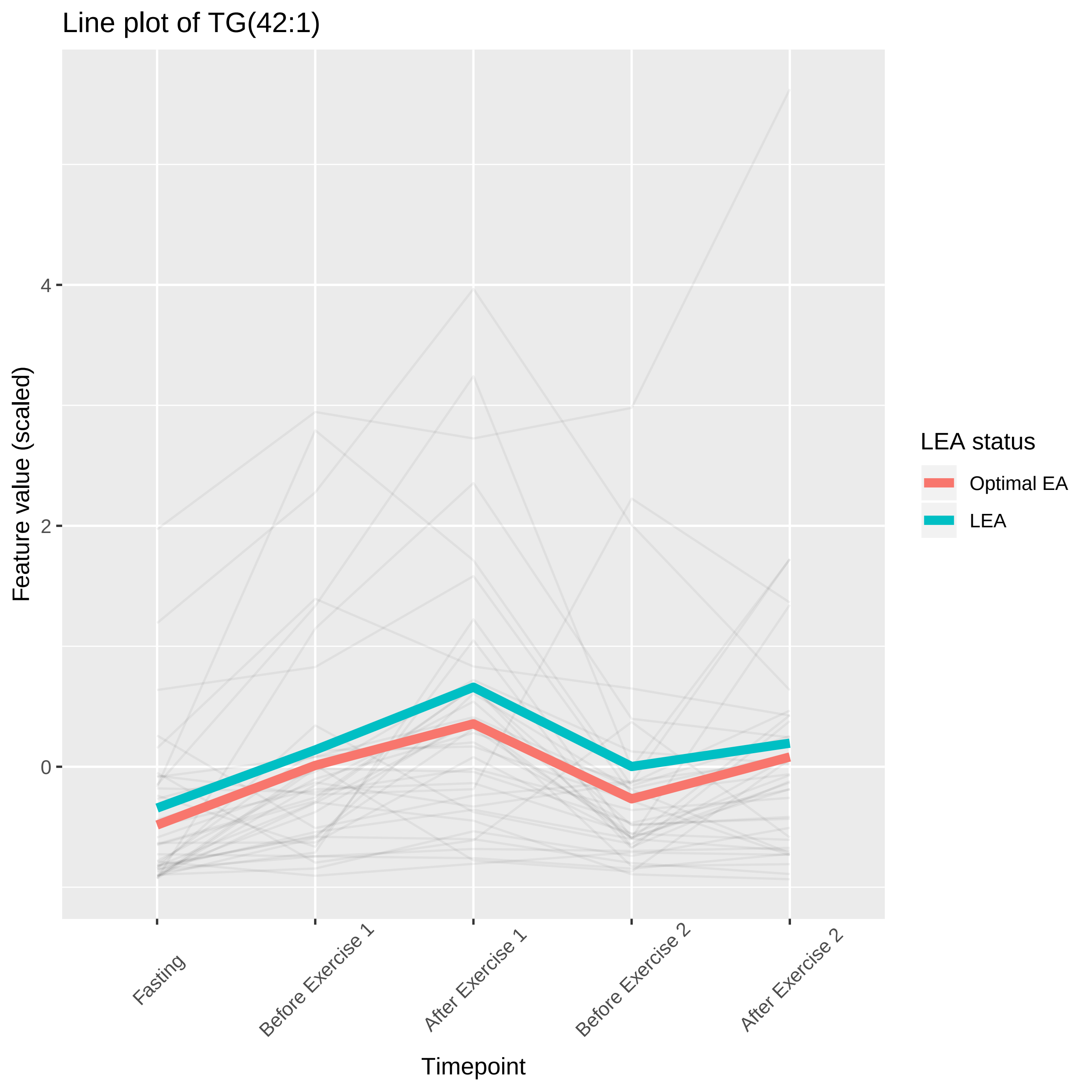
Line plot of TG(42:1)
Feature value (scaled)
4
2
0
Fasting
Before Exercise 1
After Exercise 1
Before Exercise 2
After Exercise 2
Timepoint
LEA status
Optimal EA
LEA

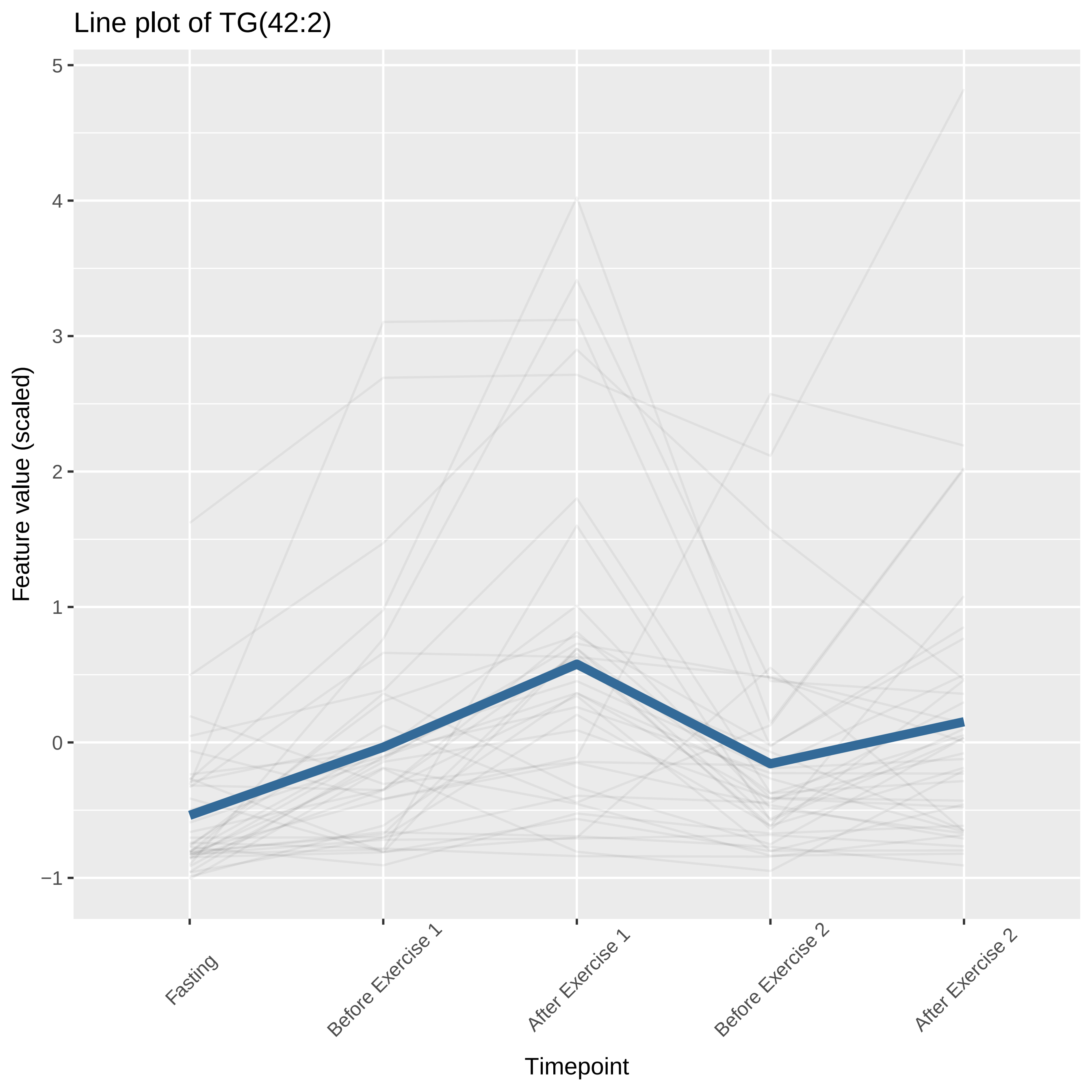
Line plot of TG(42:2)
Feature value (scaled)
5
4
3
2
1
0
−1
Fasting
Before Exercise 1
After Exercise 1
Before Exercise 2
After Exercise 2
Timepoint

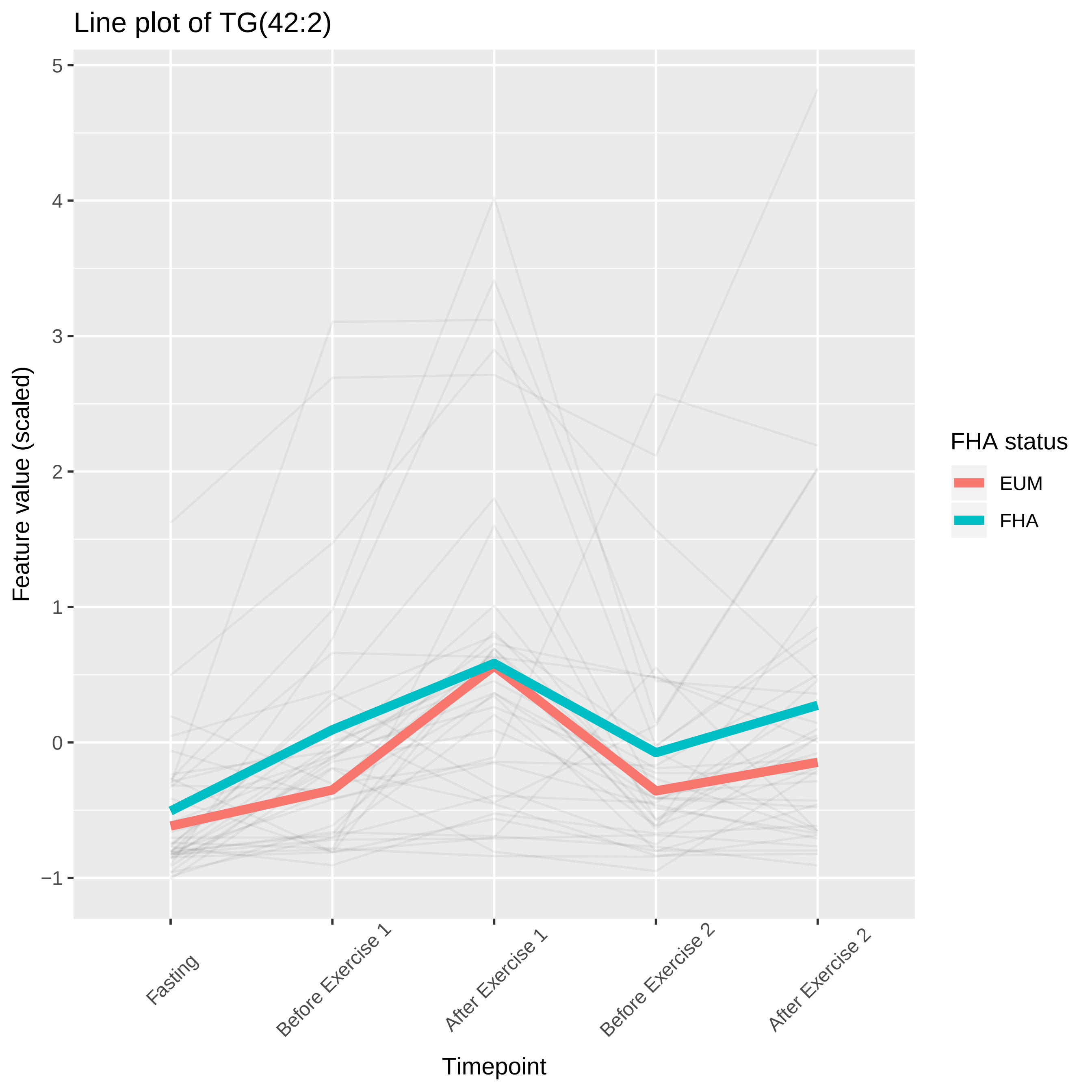

Line plot of TG(42:2)
Feature value (scaled)
5
4
3
2
1
0
−1
Fasting
Before Exercise 1
After Exercise 1
Before Exercise 2
After Exercise 2
Timepoint
FHA status
EUM
FHA

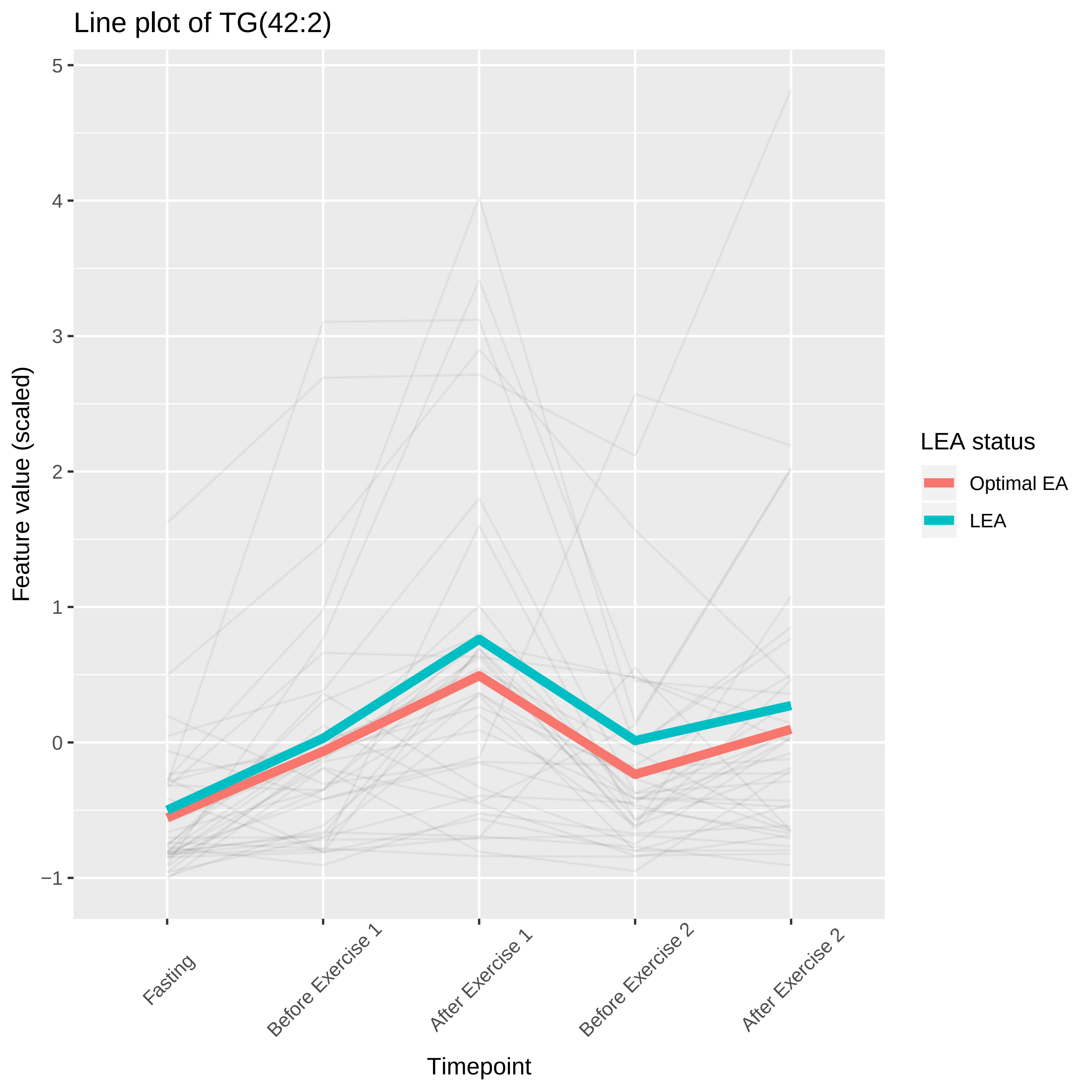

Line plot of TG(42:2)
Feature value (scaled)
5
4
3
2
1
0
−1
Fasting
Before Exercise 1
After Exercise 1
Before Exercise 2
After Exercise 2
Timepoint
LEA status
Optimal EA
LEA

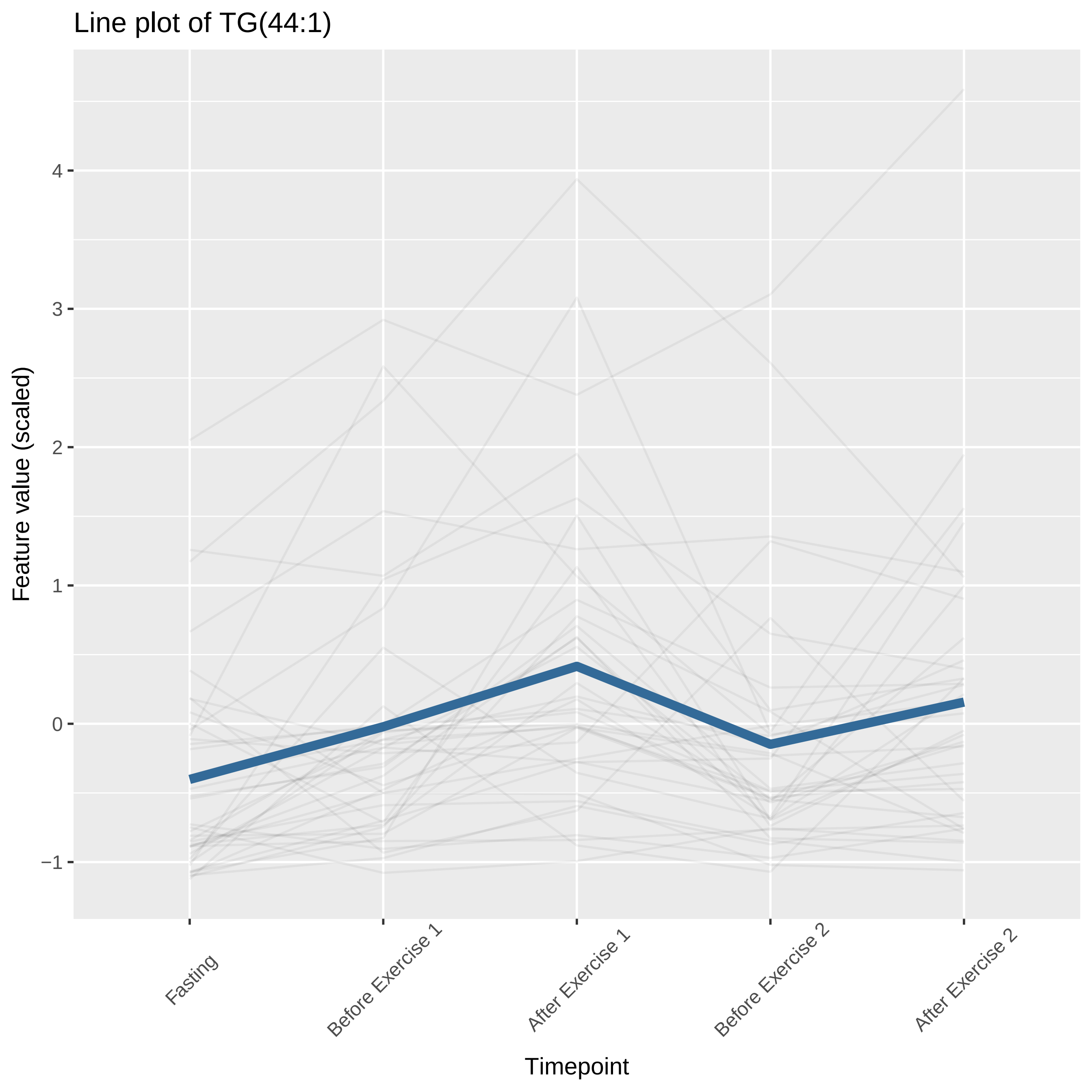

Line plot of TG(44:1)
Feature value (scaled)
4
3
2
1
0
−1
Fasting
Before Exercise 1
After Exercise 1
Before Exercise 2
After Exercise 2
Timepoint

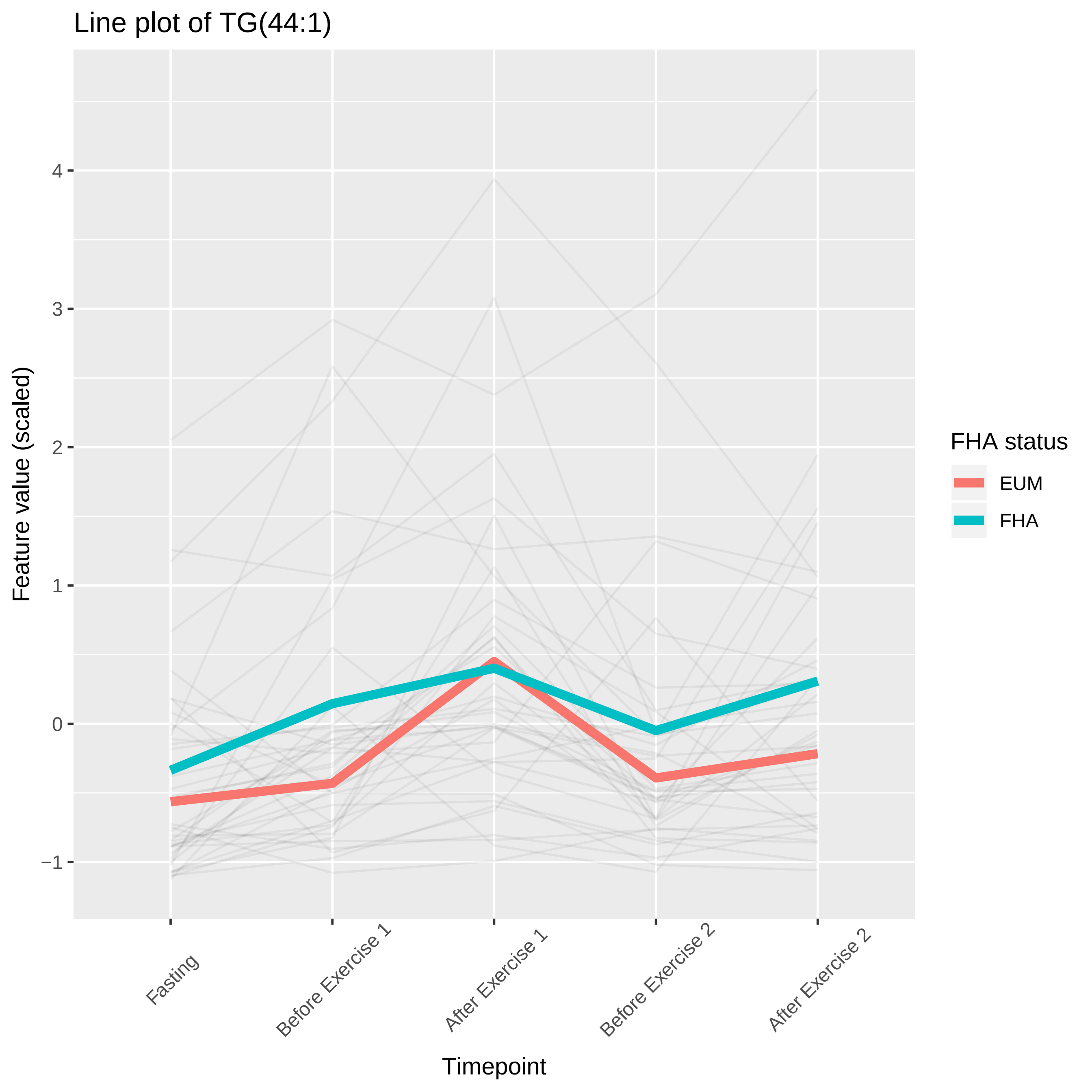

Line plot of TG(44:1)
Feature value (scaled)
4
3
2
1
0
−1
Fasting
Before Exercise 1
After Exercise 1
Before Exercise 2
After Exercise 2
Timepoint
FHA status
EUM
FHA

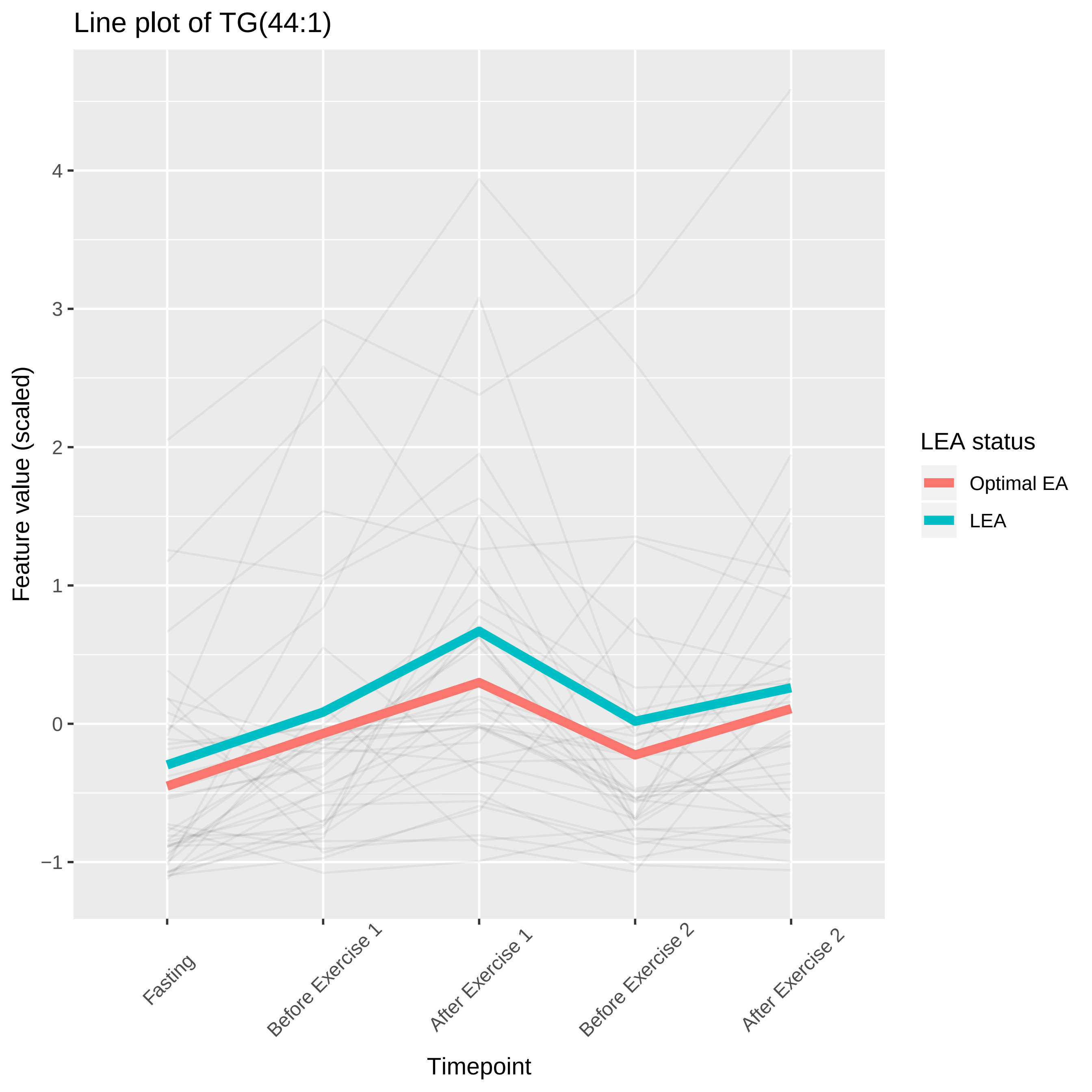

Line plot of TG(44:1)
Feature value (scaled)
4
3
2
1
0
−1
Fasting
Before Exercise 1
After Exercise 1
Before Exercise 2
After Exercise 2
Timepoint
LEA status
Optimal EA
LEA

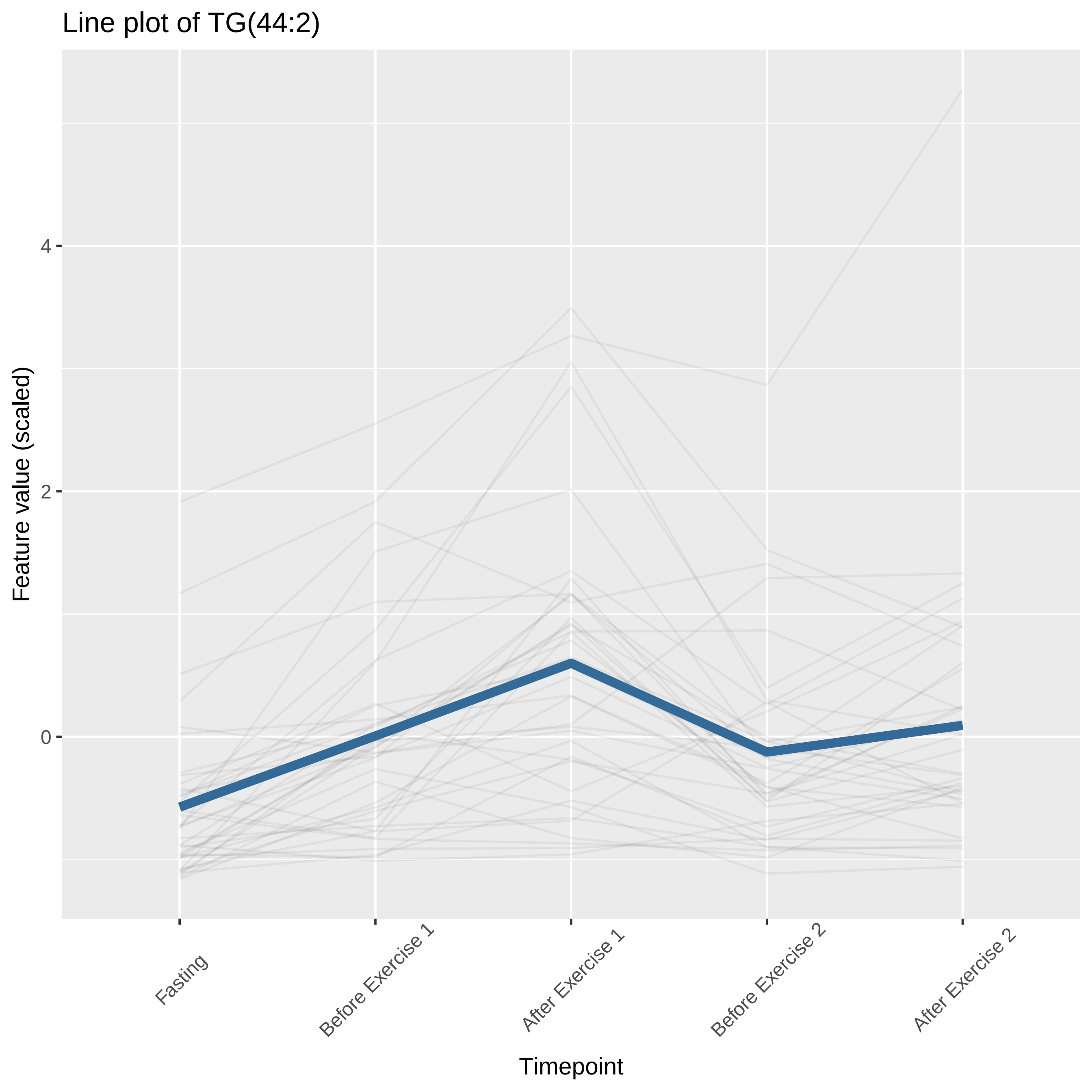

Line plot of TG(44:2)
Feature value (scaled)
4
2
0
Fasting
Before Exercise 1
After Exercise 1
Before Exercise 2
After Exercise 2
Timepoint

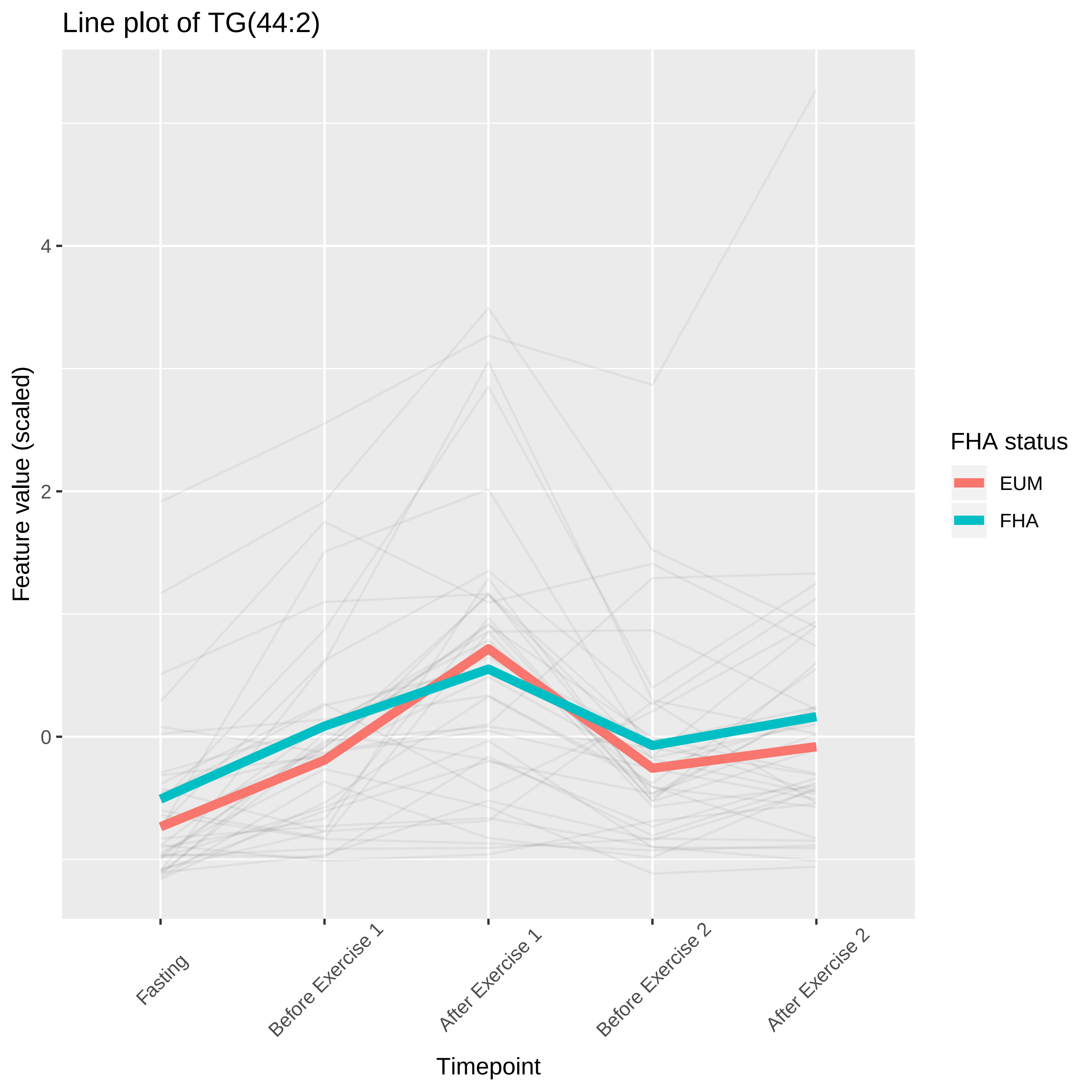

Line plot of TG(44:2)
Feature value (scaled)
4
2
0
Fasting
Before Exercise 1
After Exercise 1
Before Exercise 2
After Exercise 2
Timepoint
FHA status
EUM
FHA

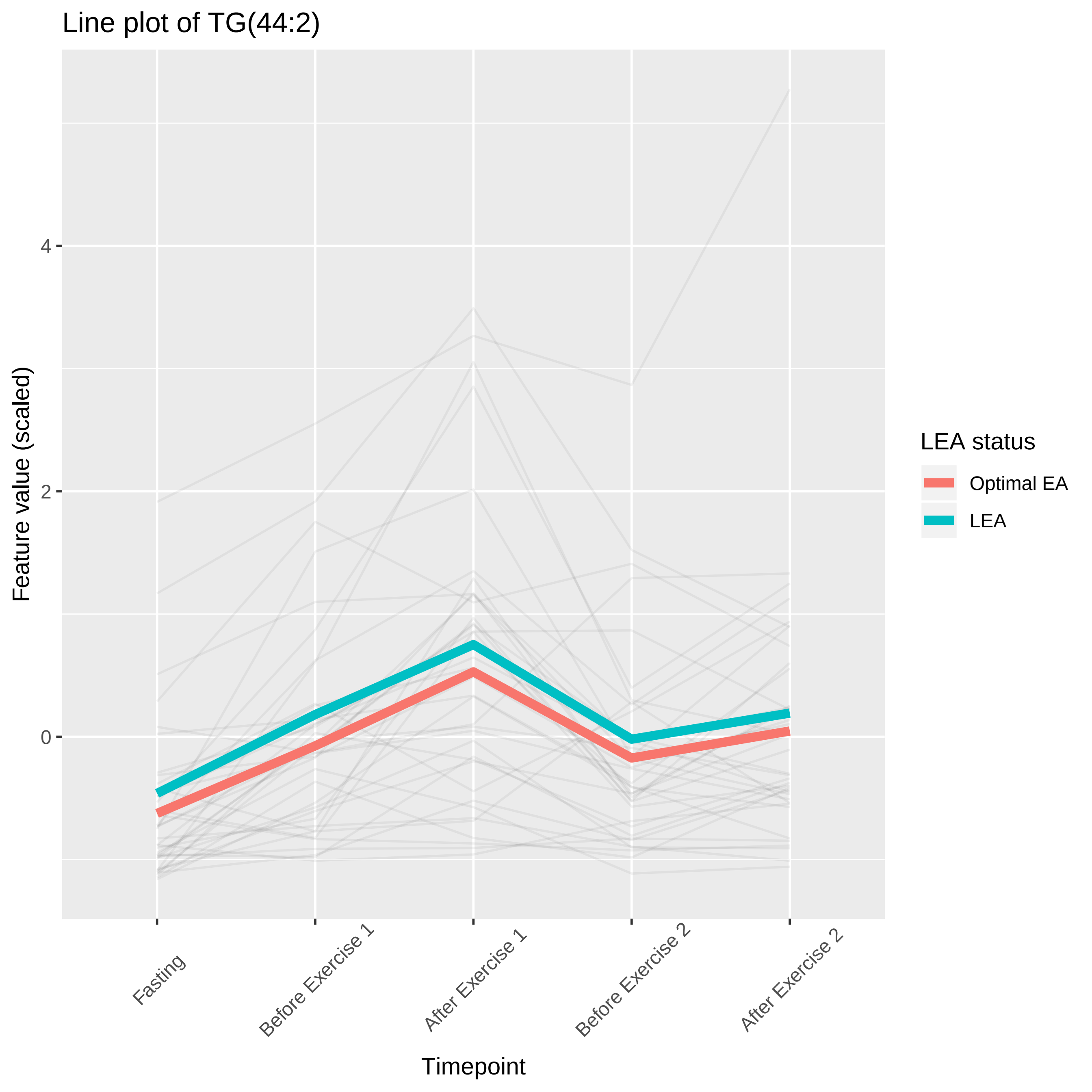
Line plot of TG(44:2)
Feature value (scaled)
4
2
0
Fasting
Before Exercise 1
After Exercise 1
Before Exercise 2
After Exercise 2
Timepoint
LEA status
Optimal EA
LEA

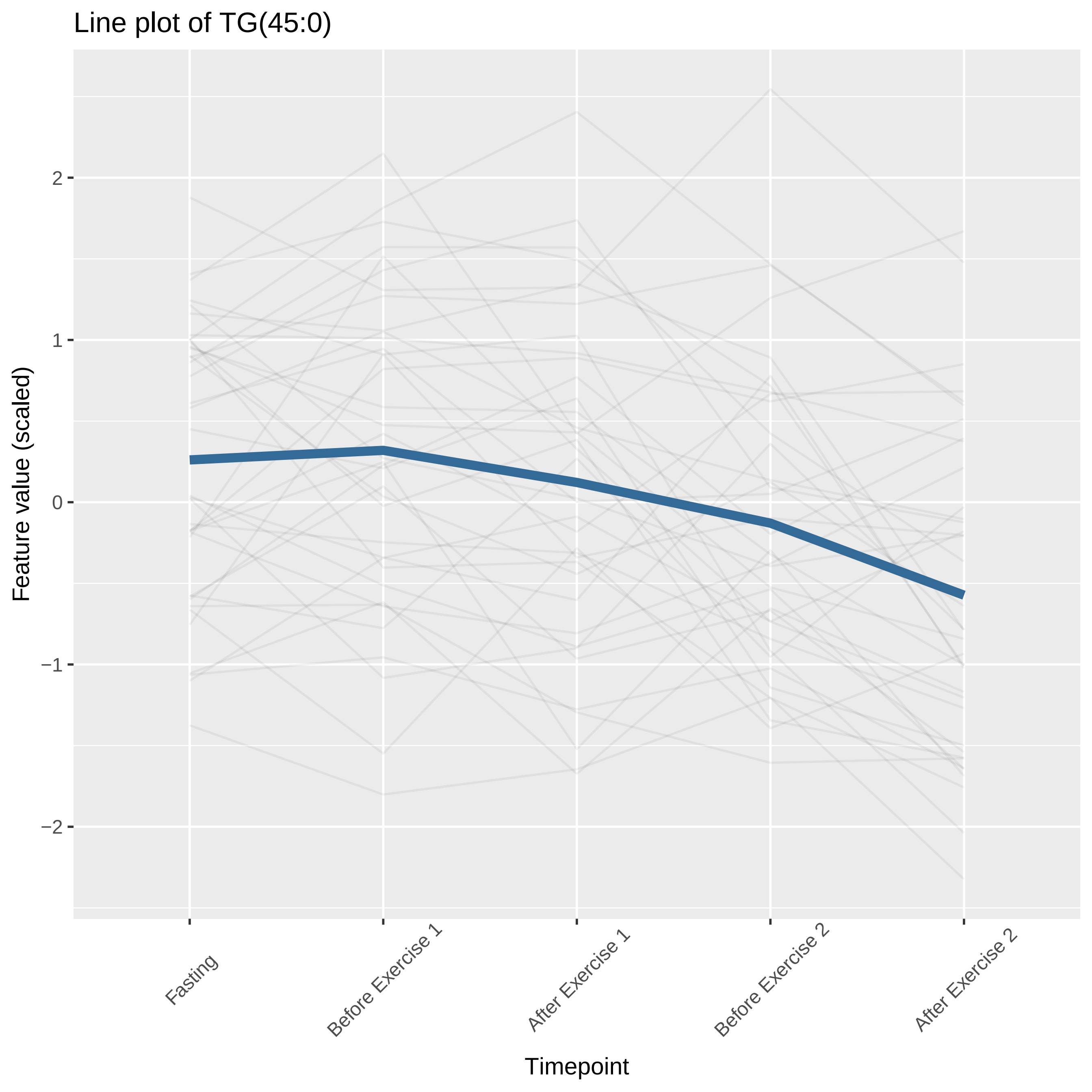

Line plot of TG(45:0)
Feature value (scaled)
2
1
0
−1
−2
Fasting
Before Exercise 1
After Exercise 1
Before Exercise 2
After Exercise 2
Timepoint

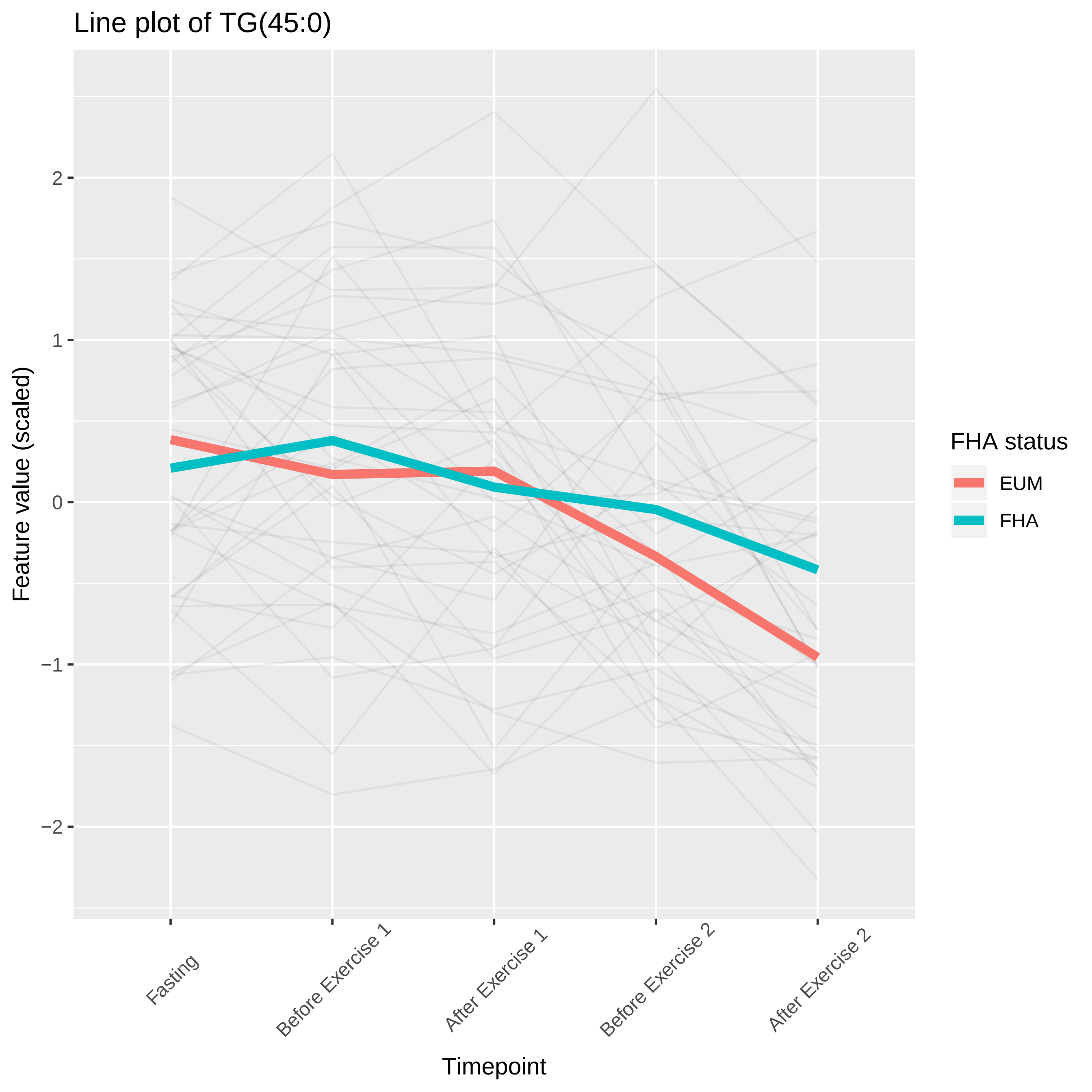

Line plot of TG(45:0)
Feature value (scaled)
2
1
0
−1
−2
Fasting
Before Exercise 1
After Exercise 1
Before Exercise 2
After Exercise 2
Timepoint
FHA status
EUM
FHA

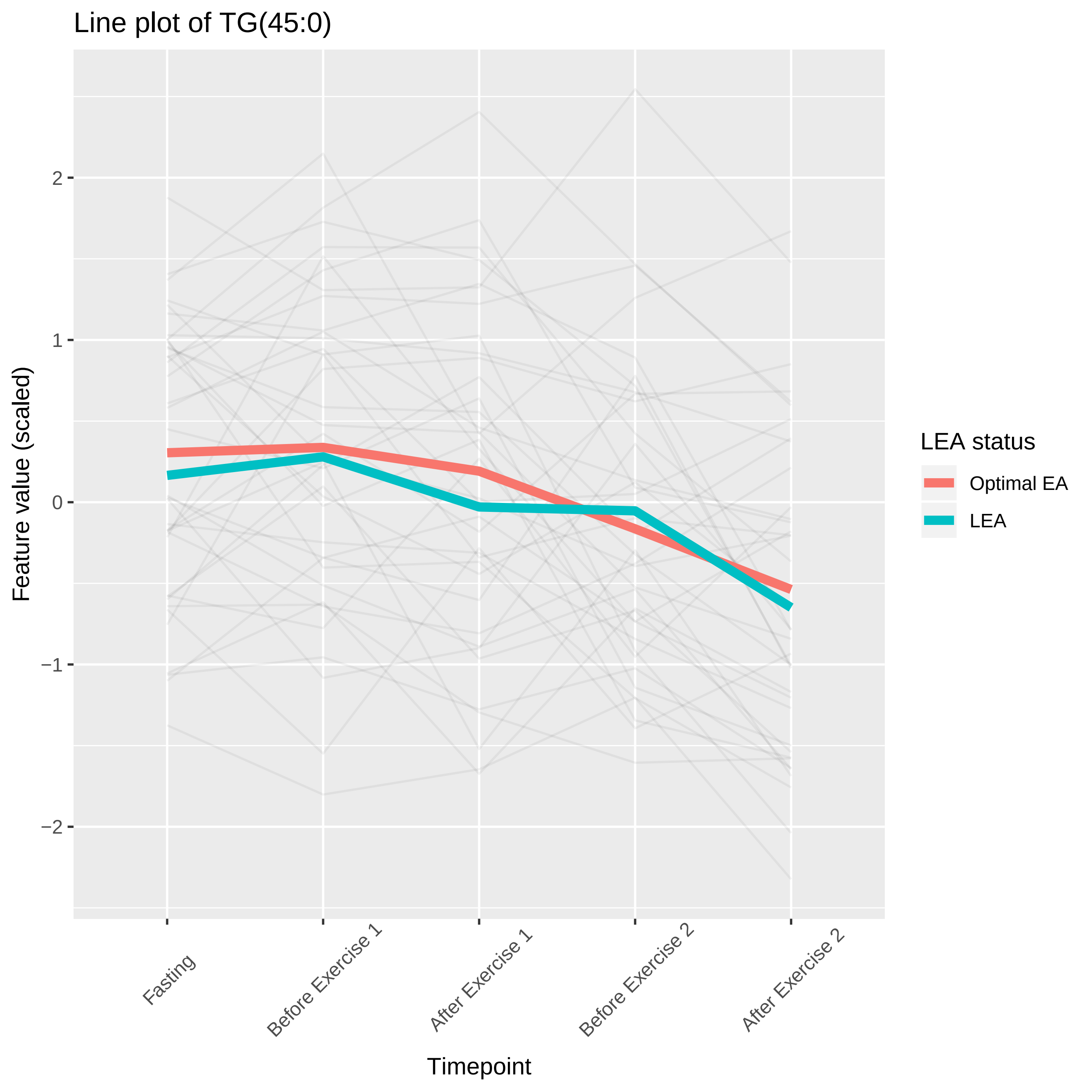

Line plot of TG(45:0)
Feature value (scaled)
2
1
0
−1
−2
Fasting
Before Exercise 1
After Exercise 1
Before Exercise 2
After Exercise 2
Timepoint
LEA status
Optimal EA
LEA

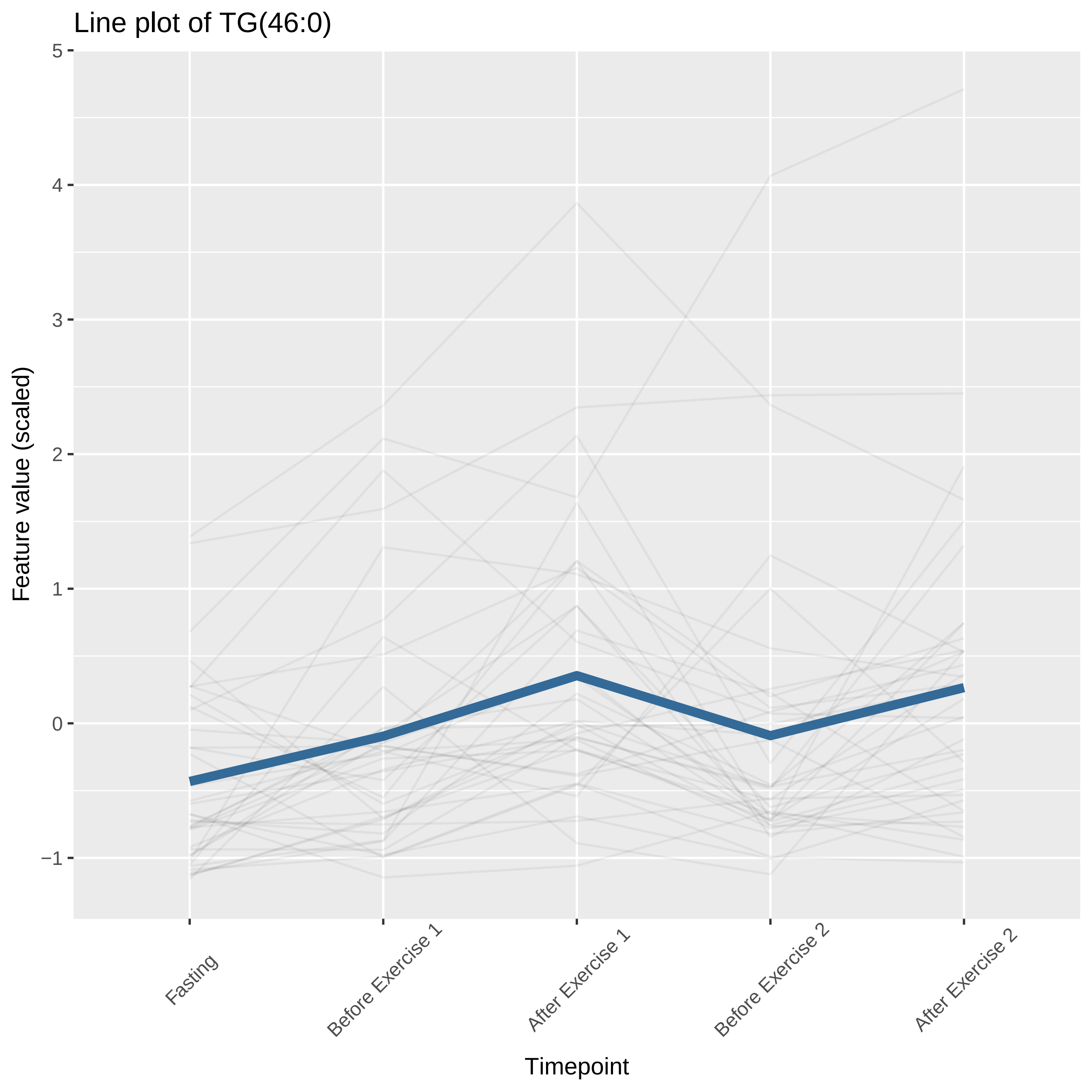
Line plot of TG(46:0)
Feature value (scaled)
5
4
3
2
1
0
−1
Fasting
Before Exercise 1
After Exercise 1
Before Exercise 2
After Exercise 2
Timepoint

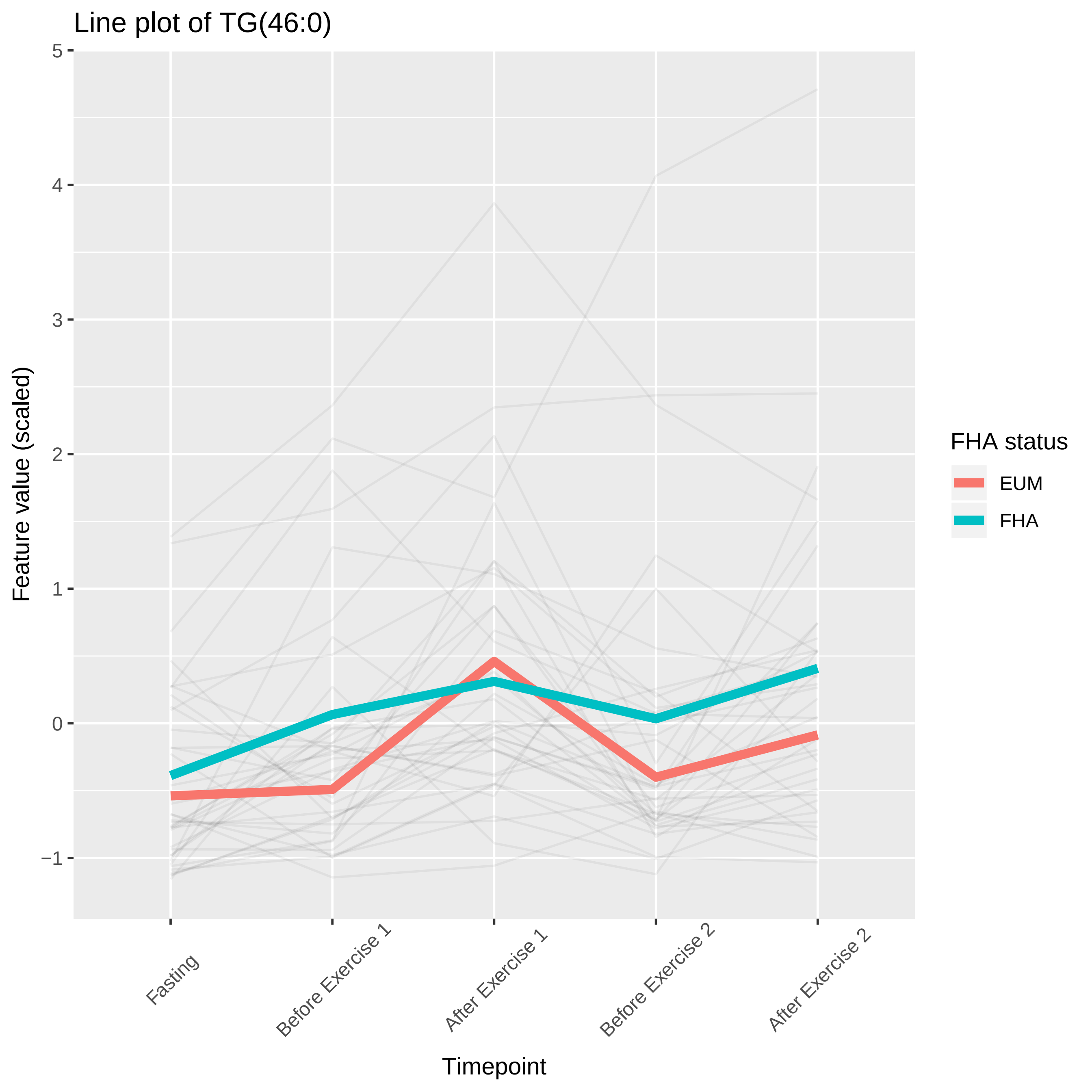
Line plot of TG(46:0)
Feature value (scaled)
5
4
3
2
1
0
−1
Fasting
Before Exercise 1
After Exercise 1
Before Exercise 2
After Exercise 2
Timepoint
FHA status
EUM
FHA

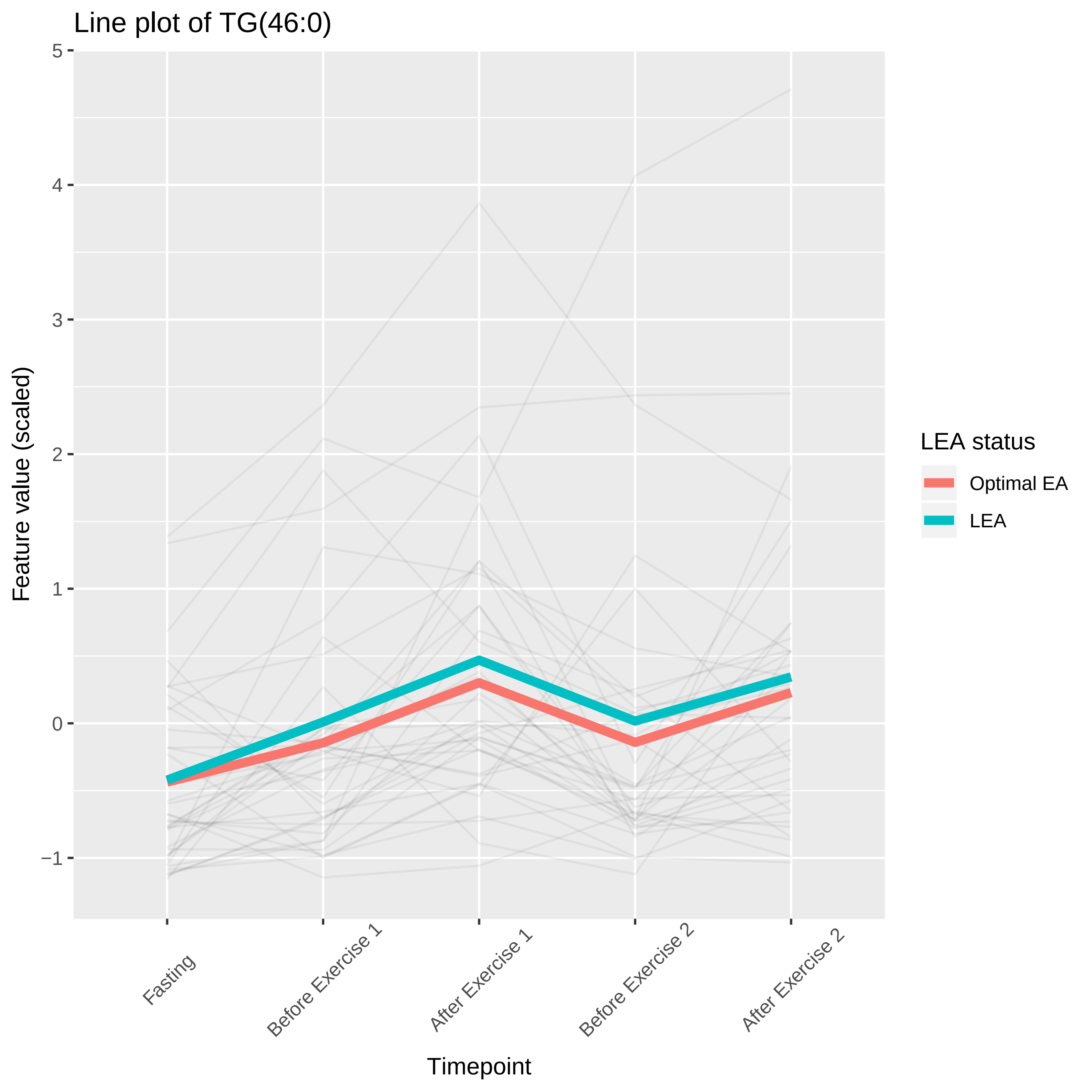

Line plot of TG(46:0)
Feature value (scaled)
5
4
3
2
1
0
−1
Fasting
Before Exercise 1
After Exercise 1
Before Exercise 2
After Exercise 2
Timepoint
LEA status
Optimal EA
LEA

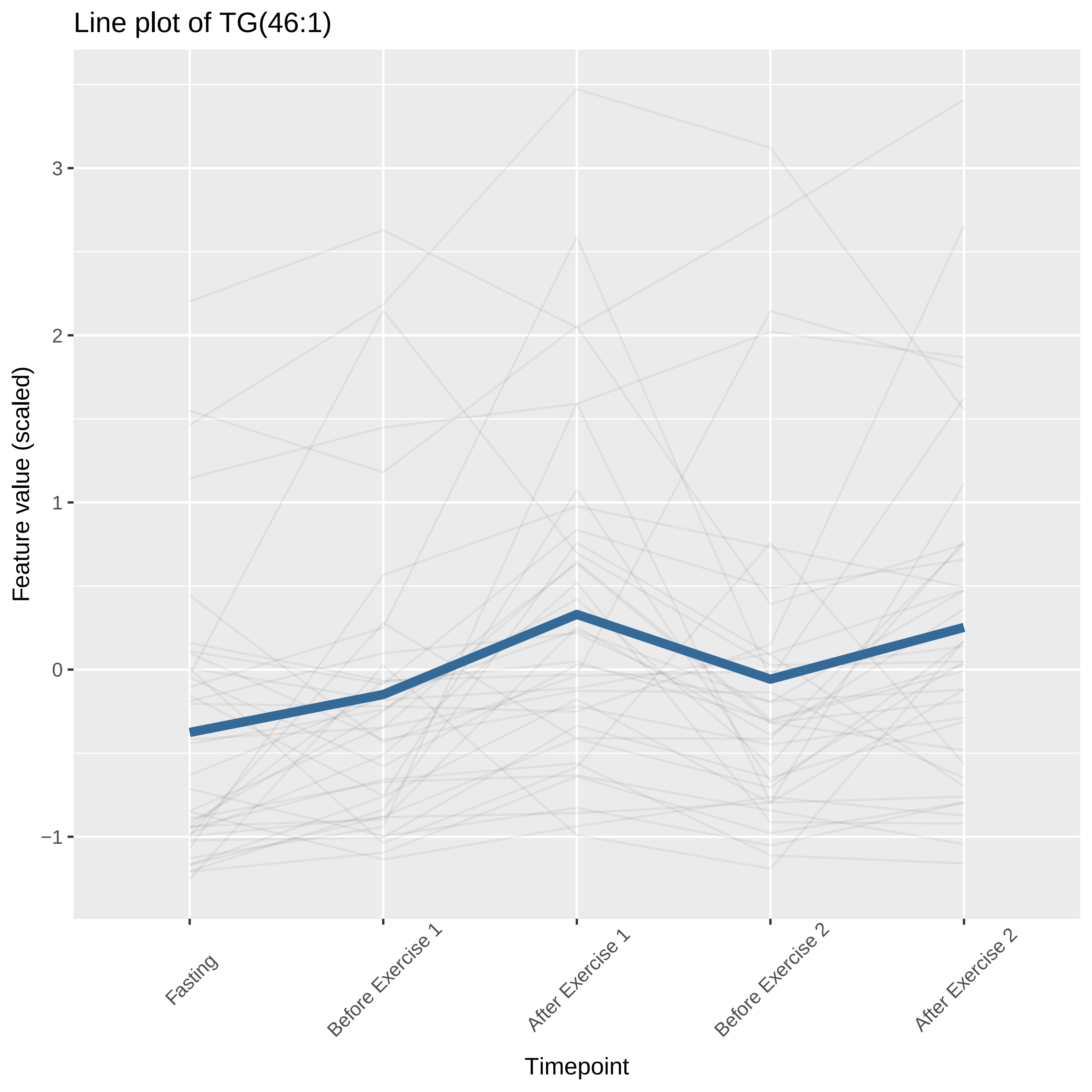

Line plot of TG(46:1)
Feature value (scaled)
3
2
1
0
−1
Fasting
Before Exercise 1
After Exercise 1
Before Exercise 2
After Exercise 2
Timepoint

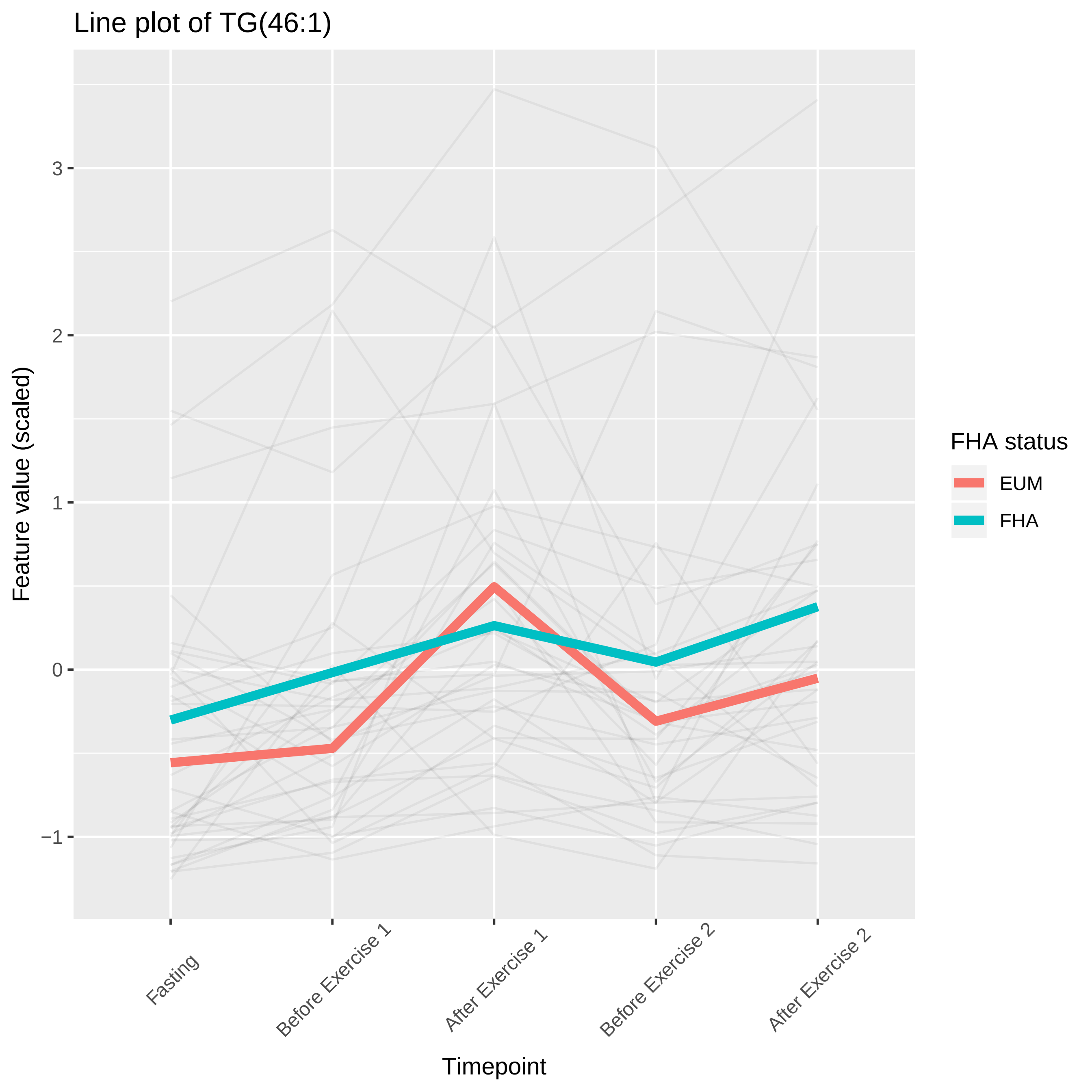

Line plot of TG(46:1)
Feature value (scaled)
3
2
1
0
−1
Fasting
Before Exercise 1
After Exercise 1
Before Exercise 2
After Exercise 2
Timepoint
FHA status
EUM
FHA

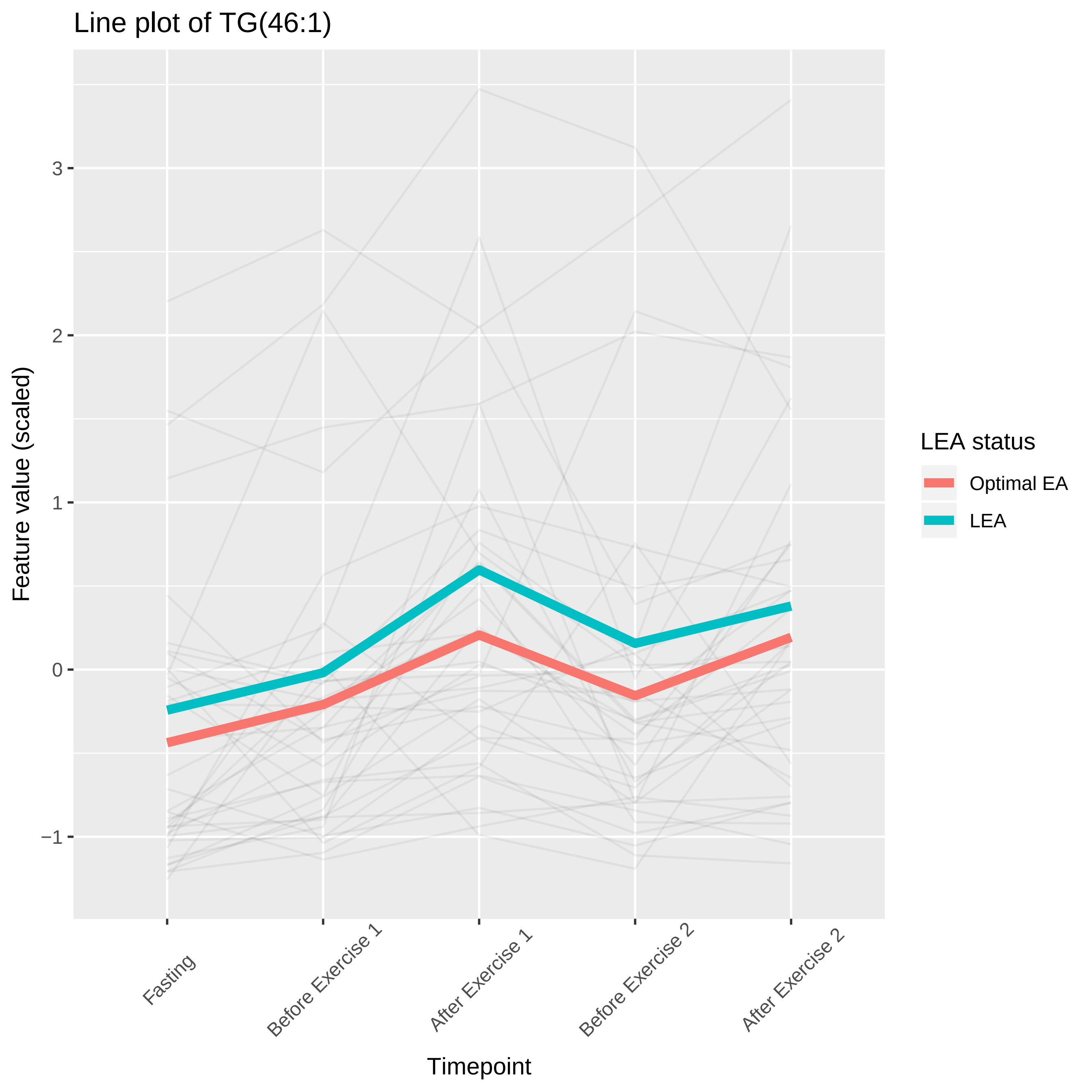

Line plot of TG(46:1)
Feature value (scaled)
3
2
1
0
−1
Fasting
Before Exercise 1
After Exercise 1
Before Exercise 2
After Exercise 2
Timepoint
LEA status
Optimal EA
LEA

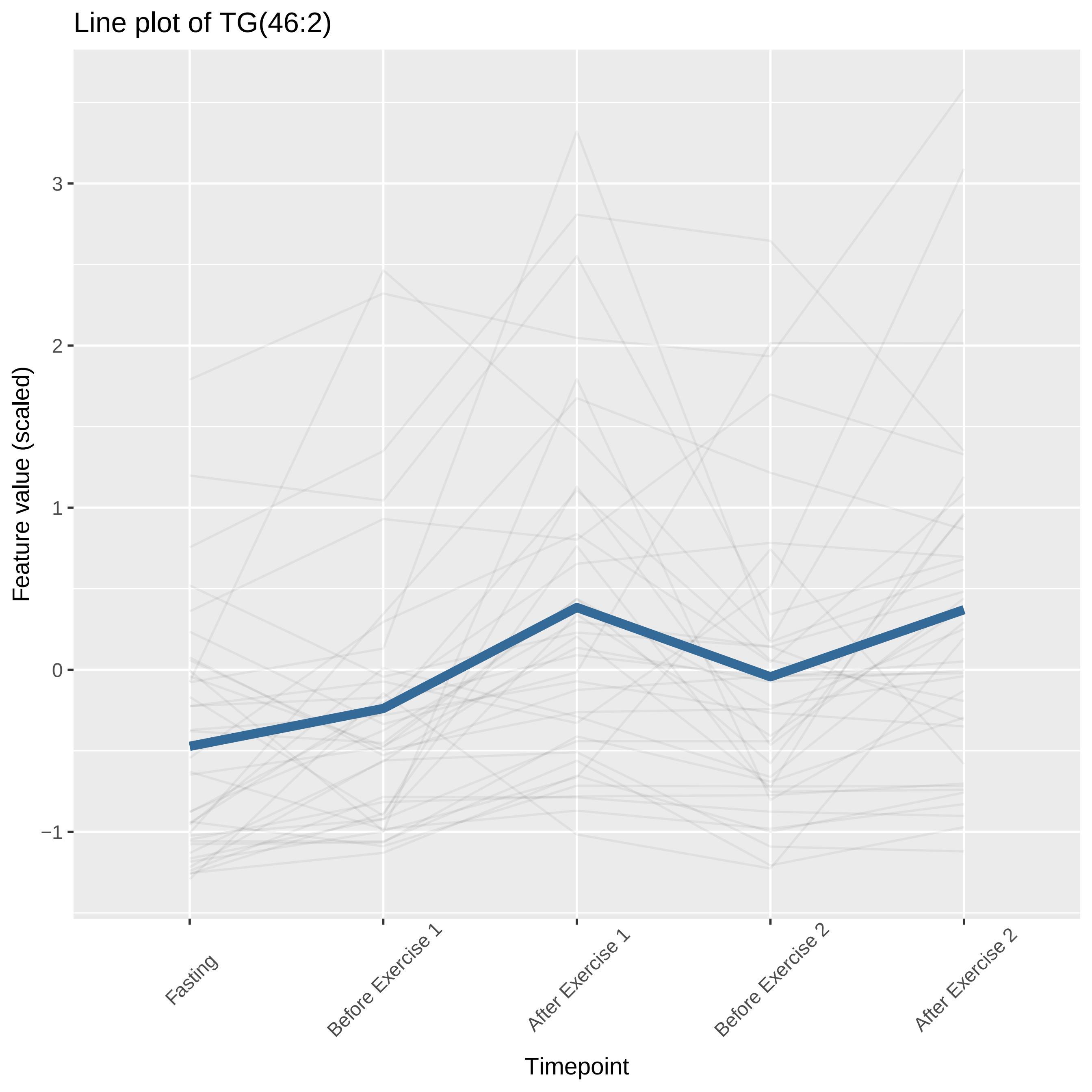

Line plot of TG(46:2)
Feature value (scaled)
3
2
1
0
−1
Fasting
Before Exercise 1
After Exercise 1
Before Exercise 2
After Exercise 2
Timepoint

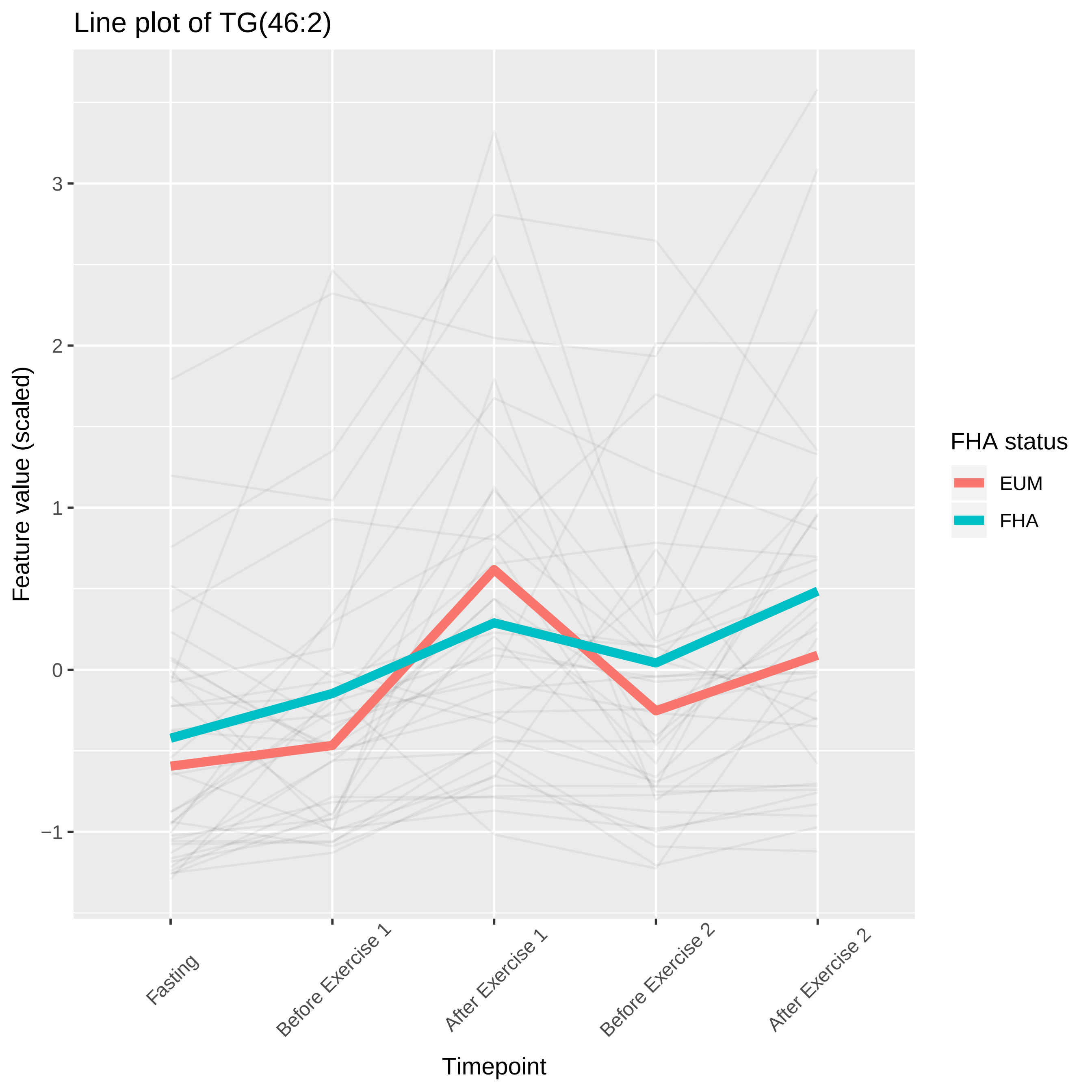

Line plot of TG(46:2)
Feature value (scaled)
3
2
1
0
−1
Fasting
Before Exercise 1
After Exercise 1
Before Exercise 2
After Exercise 2
Timepoint
FHA status
EUM
FHA

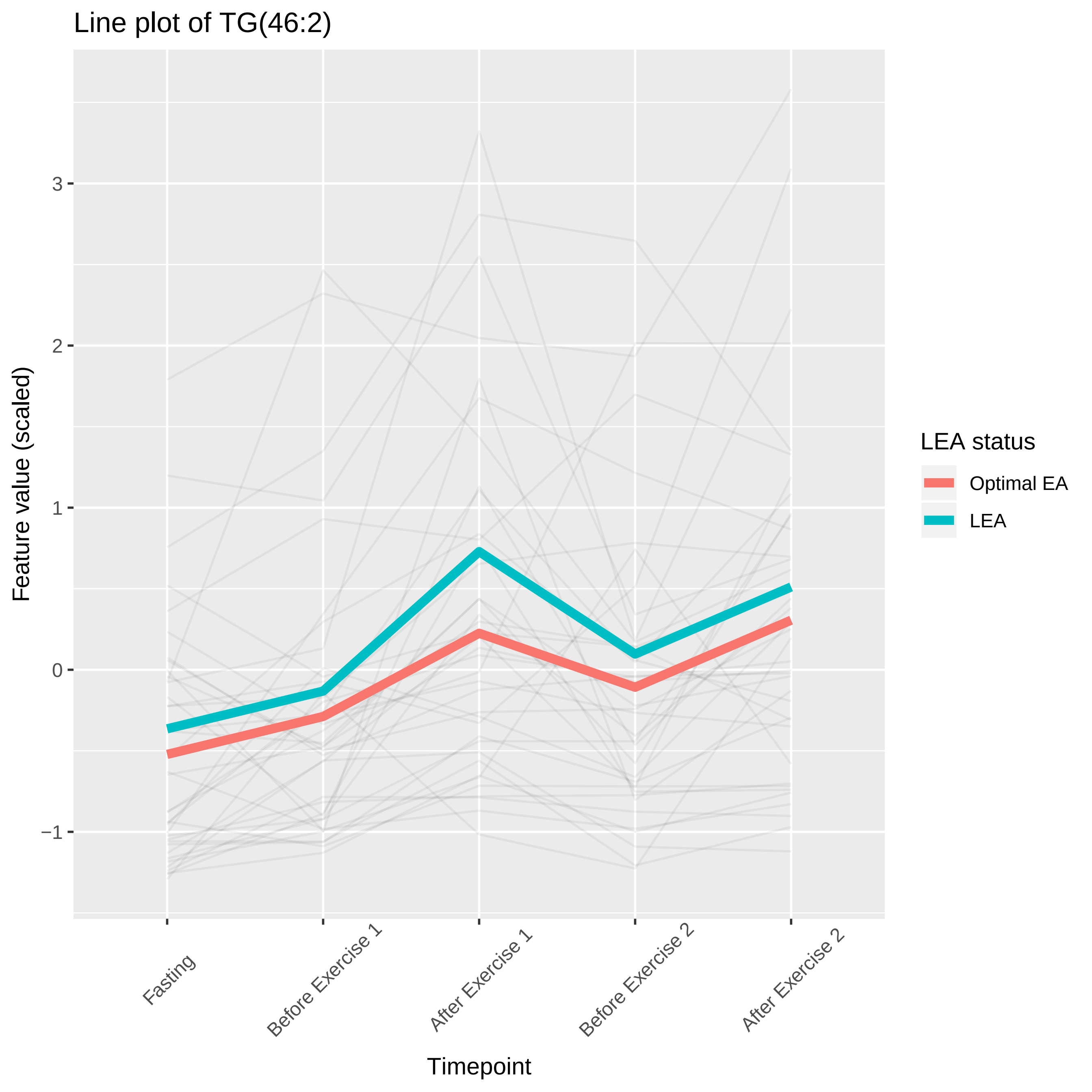
Line plot of TG(46:2)
Feature value (scaled)
3
2
1
0
−1
Fasting
Before Exercise 1
After Exercise 1
Before Exercise 2
After Exercise 2
Timepoint
LEA status
Optimal EA
LEA

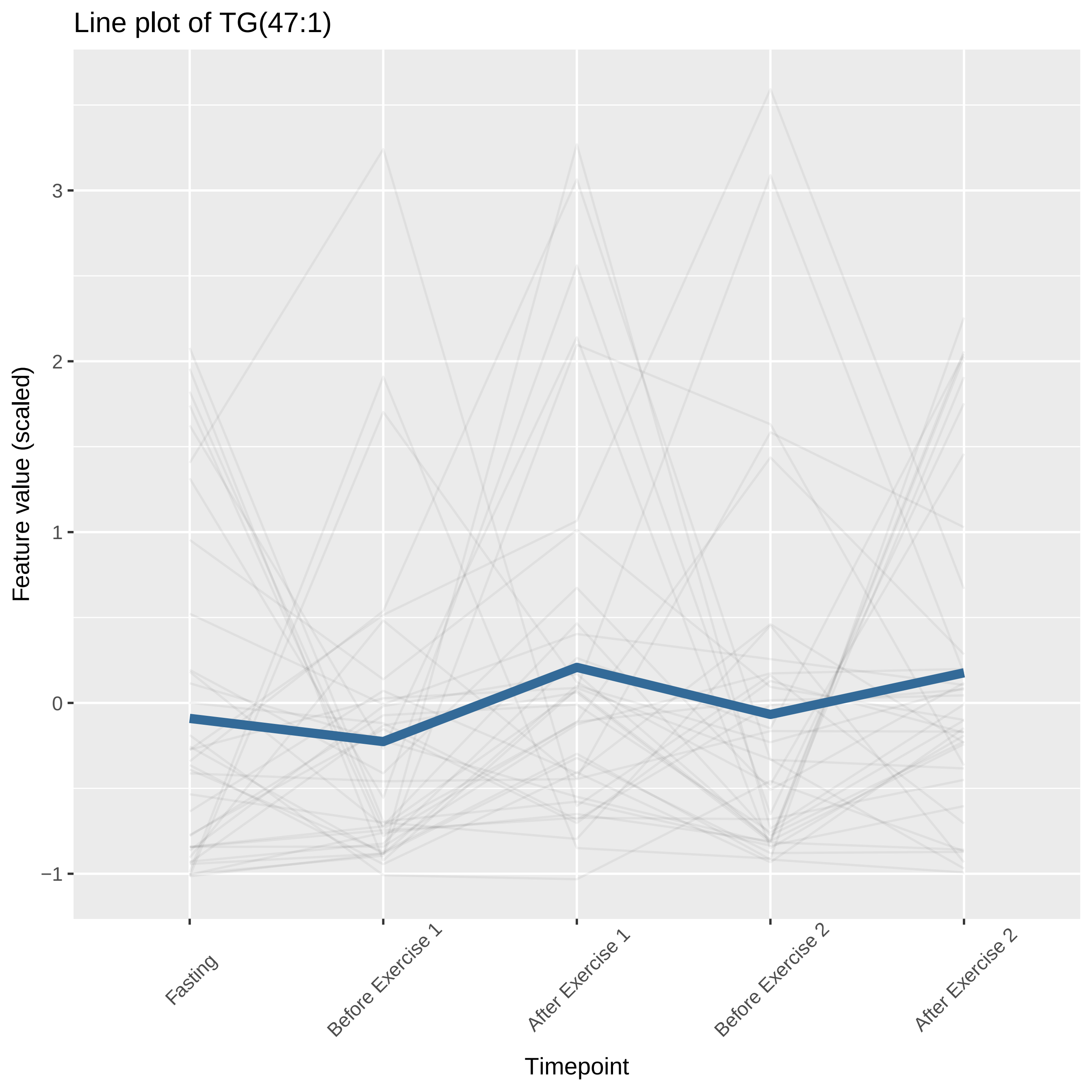

Line plot of TG(47:1)
Feature value (scaled)
3
2
1
0
−1
Fasting
Before Exercise 1
After Exercise 1
Before Exercise 2
After Exercise 2
Timepoint

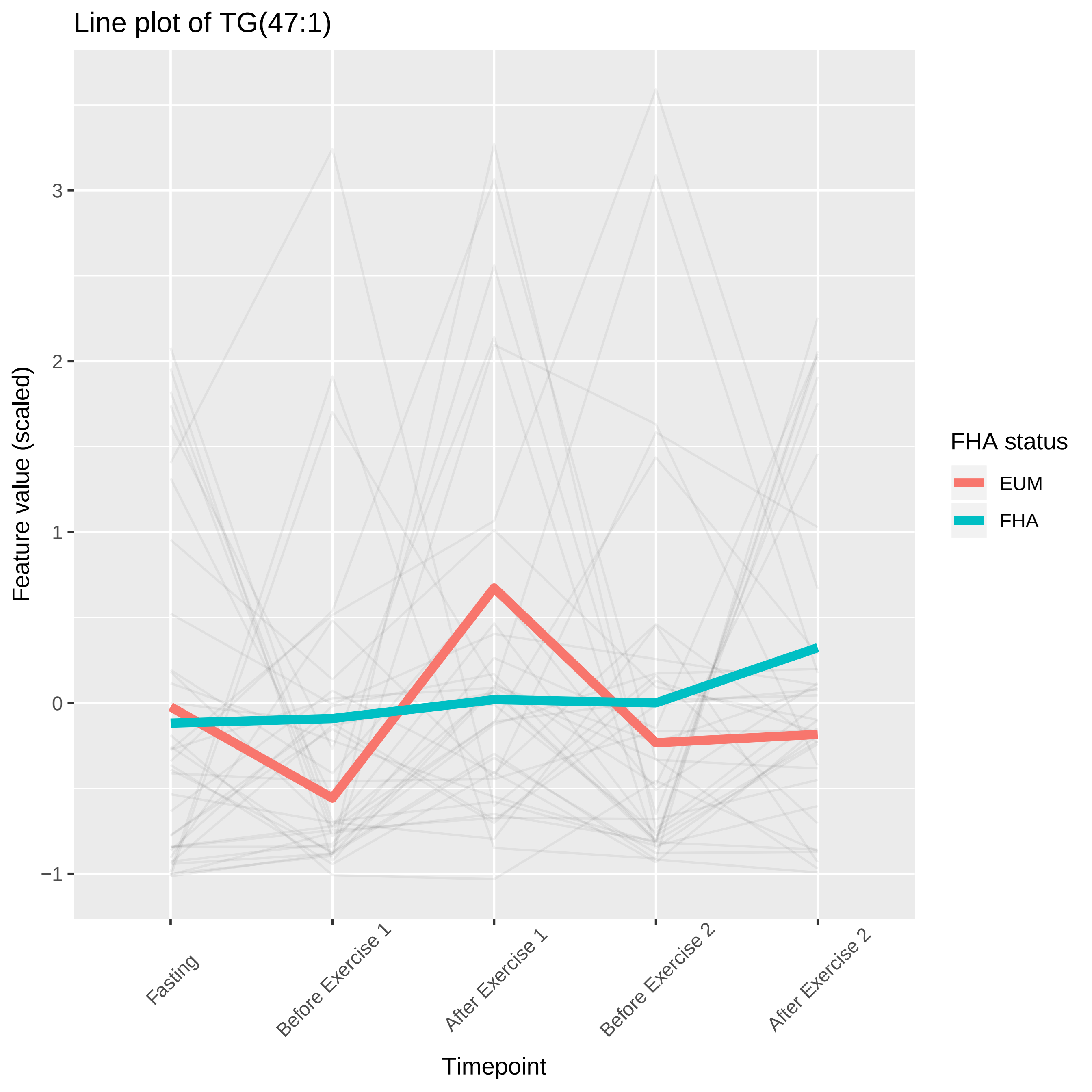

Line plot of TG(47:1)
Feature value (scaled)
3
2
1
0
−1
Fasting
Before Exercise 1
After Exercise 1
Before Exercise 2
After Exercise 2
Timepoint
FHA status
EUM
FHA

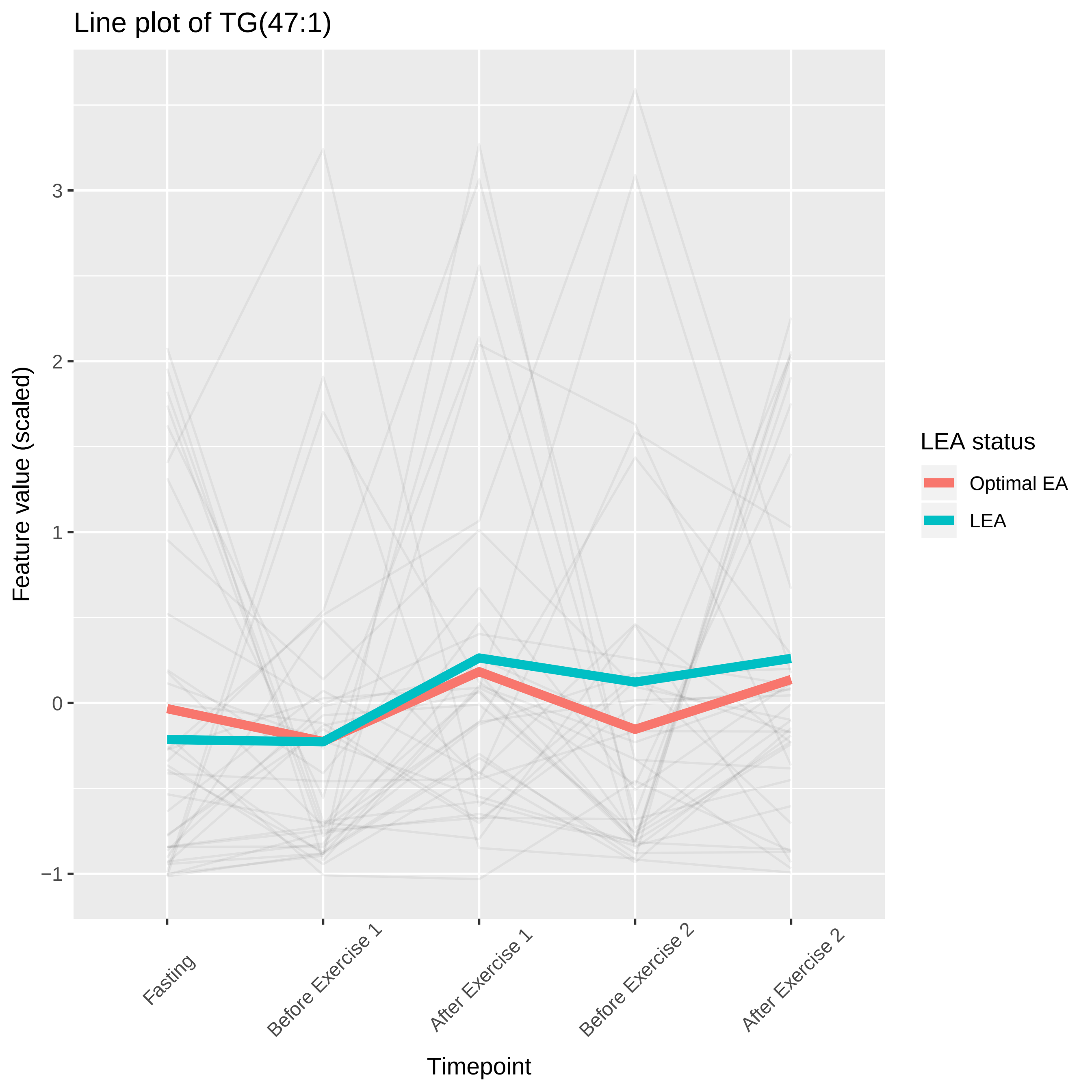

Line plot of TG(47:1)
Feature value (scaled)
3
2
1
0
−1
Fasting
Before Exercise 1
After Exercise 1
Before Exercise 2
After Exercise 2
Timepoint
LEA status
Optimal EA
LEA

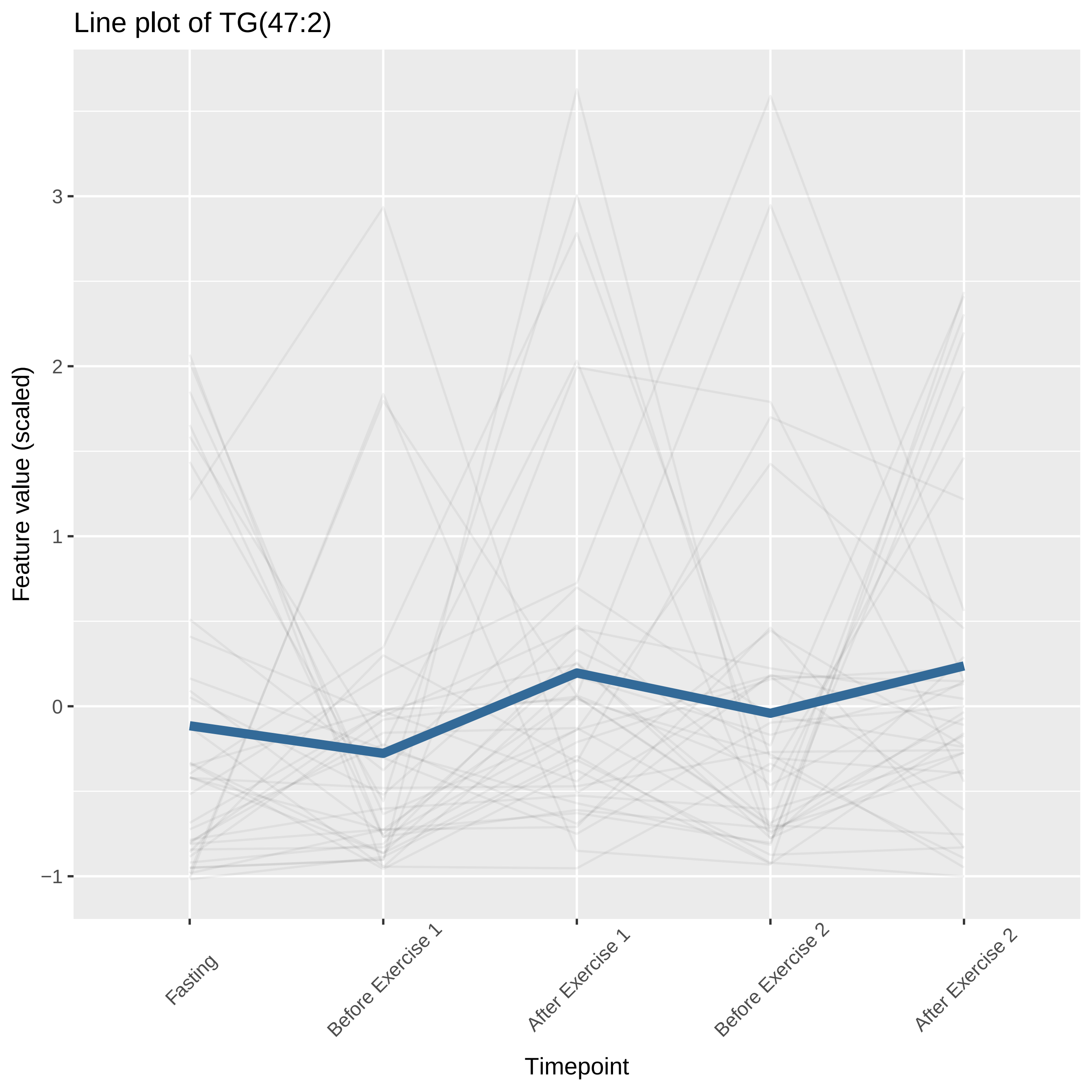

Line plot of TG(47:2)
Feature value (scaled)
3
2
1
0
−1
Fasting
Before Exercise 1
After Exercise 1
Before Exercise 2
After Exercise 2
Timepoint

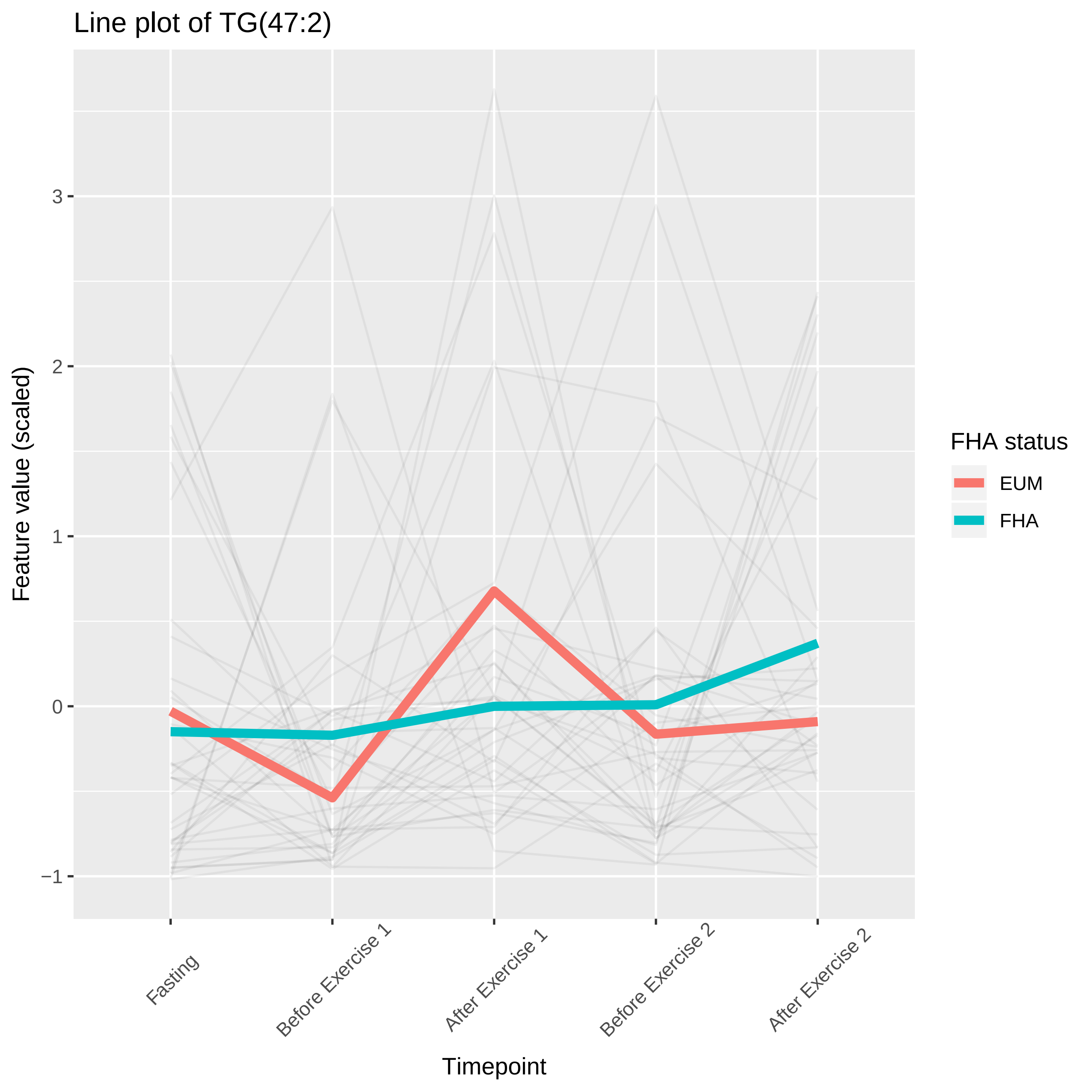
Line plot of TG(47:2)
Feature value (scaled)
3
2
1
0
−1
Fasting
Before Exercise 1
After Exercise 1
Before Exercise 2
After Exercise 2
Timepoint
FHA status
EUM
FHA

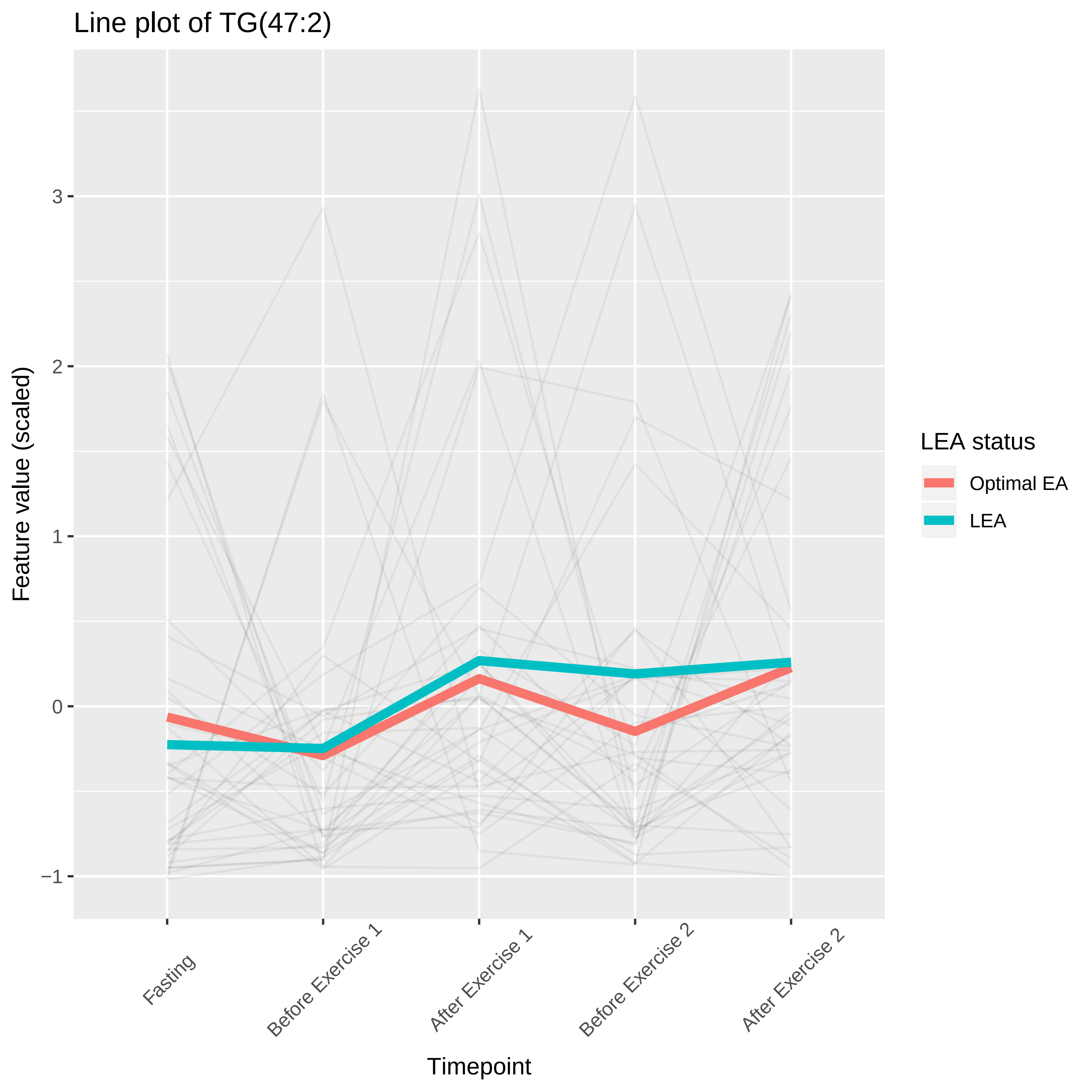

Line plot of TG(47:2)
Feature value (scaled)
3
2
1
0
−1
Fasting
Before Exercise 1
After Exercise 1
Before Exercise 2
After Exercise 2
Timepoint
LEA status
Optimal EA
LEA

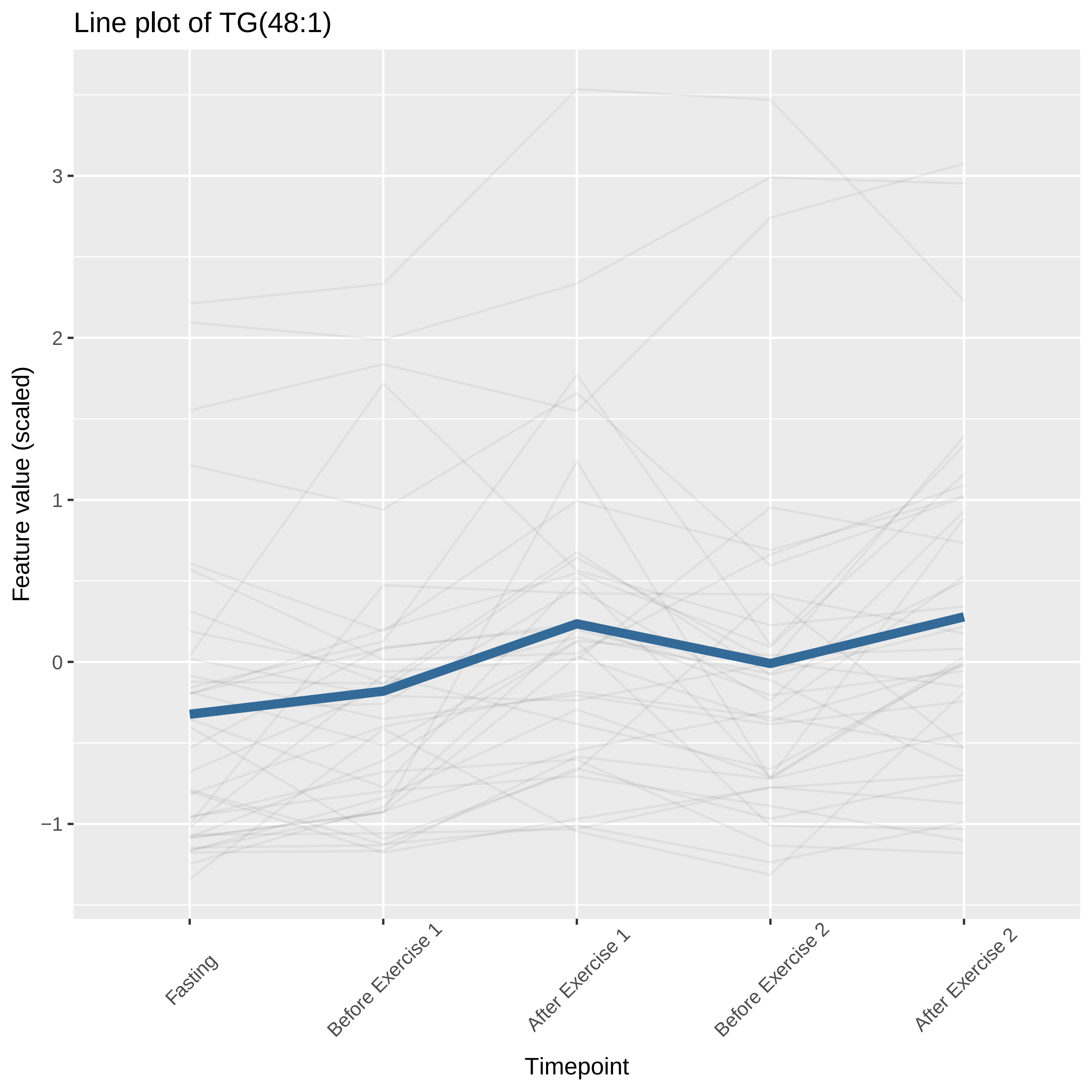

Line plot of TG(48:1)
Feature value (scaled)
3
2
1
0
−1
Fasting
Before Exercise 1
After Exercise 1
Before Exercise 2
After Exercise 2
Timepoint

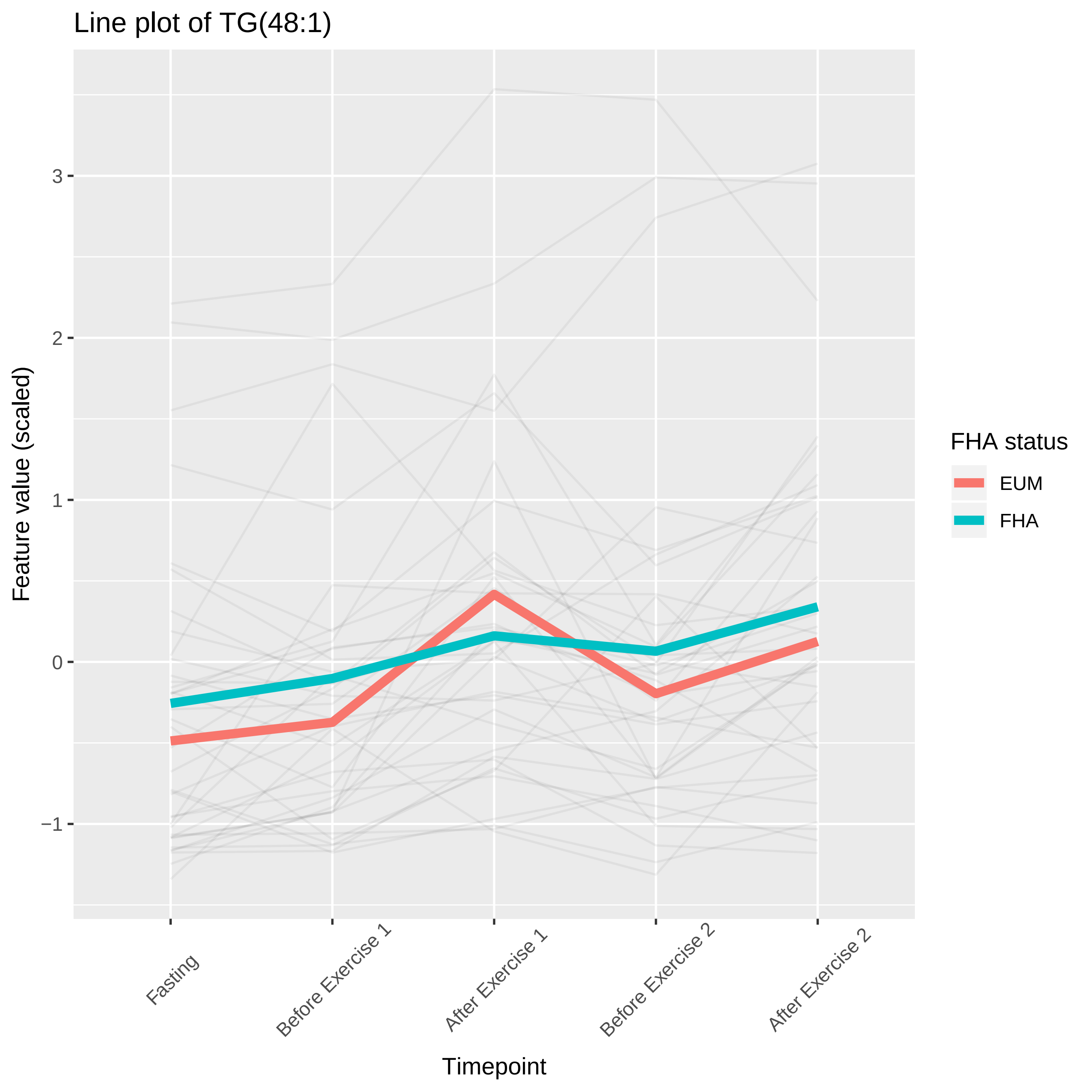

Line plot of TG(48:1)
Feature value (scaled)
3
2
1
0
−1
Fasting
Before Exercise 1
After Exercise 1
Before Exercise 2
After Exercise 2
Timepoint
FHA status
EUM
FHA

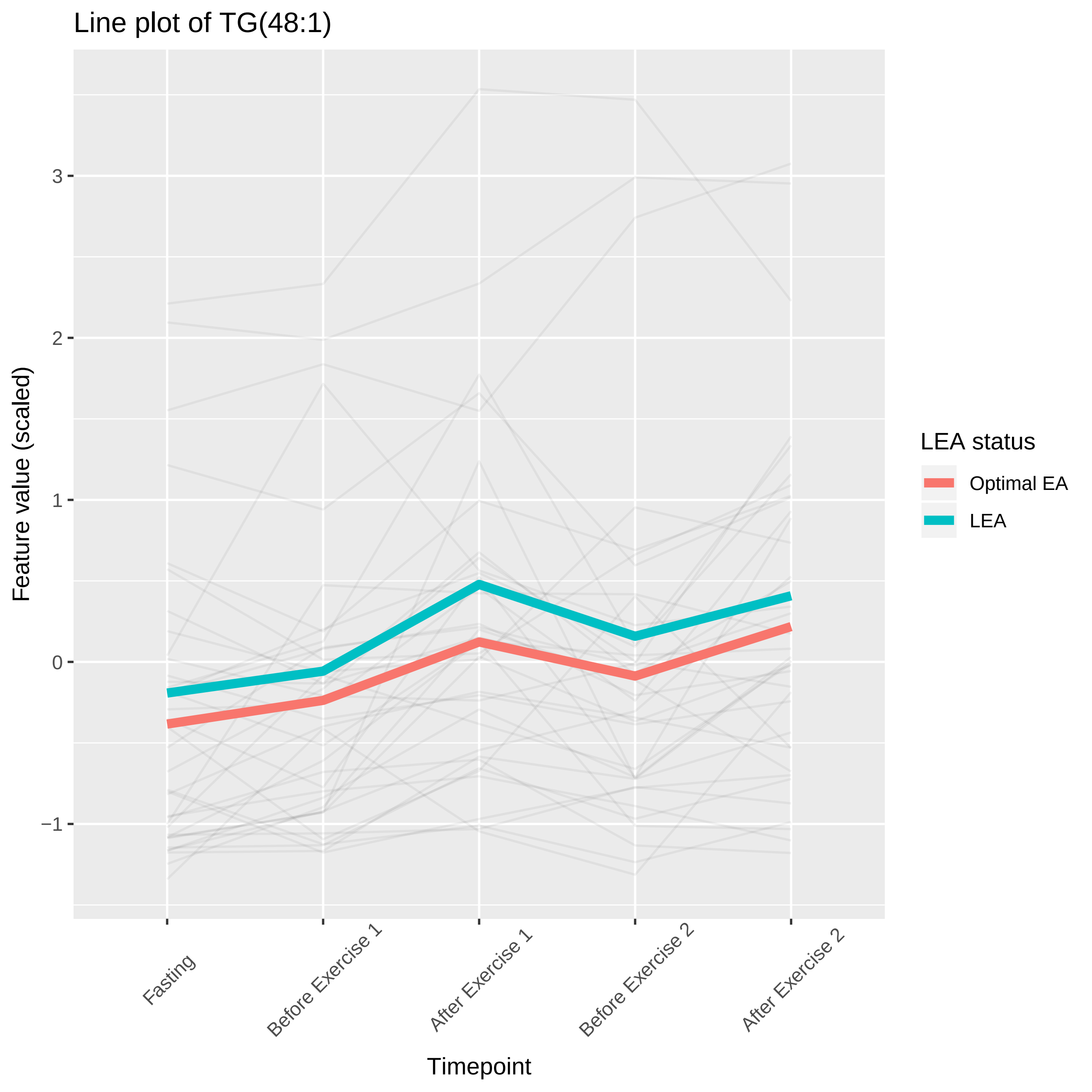
Line plot of TG(48:1)
Feature value (scaled)
3
2
1
0
−1
Fasting
Before Exercise 1
After Exercise 1
Before Exercise 2
After Exercise 2
Timepoint
LEA status
Optimal EA
LEA

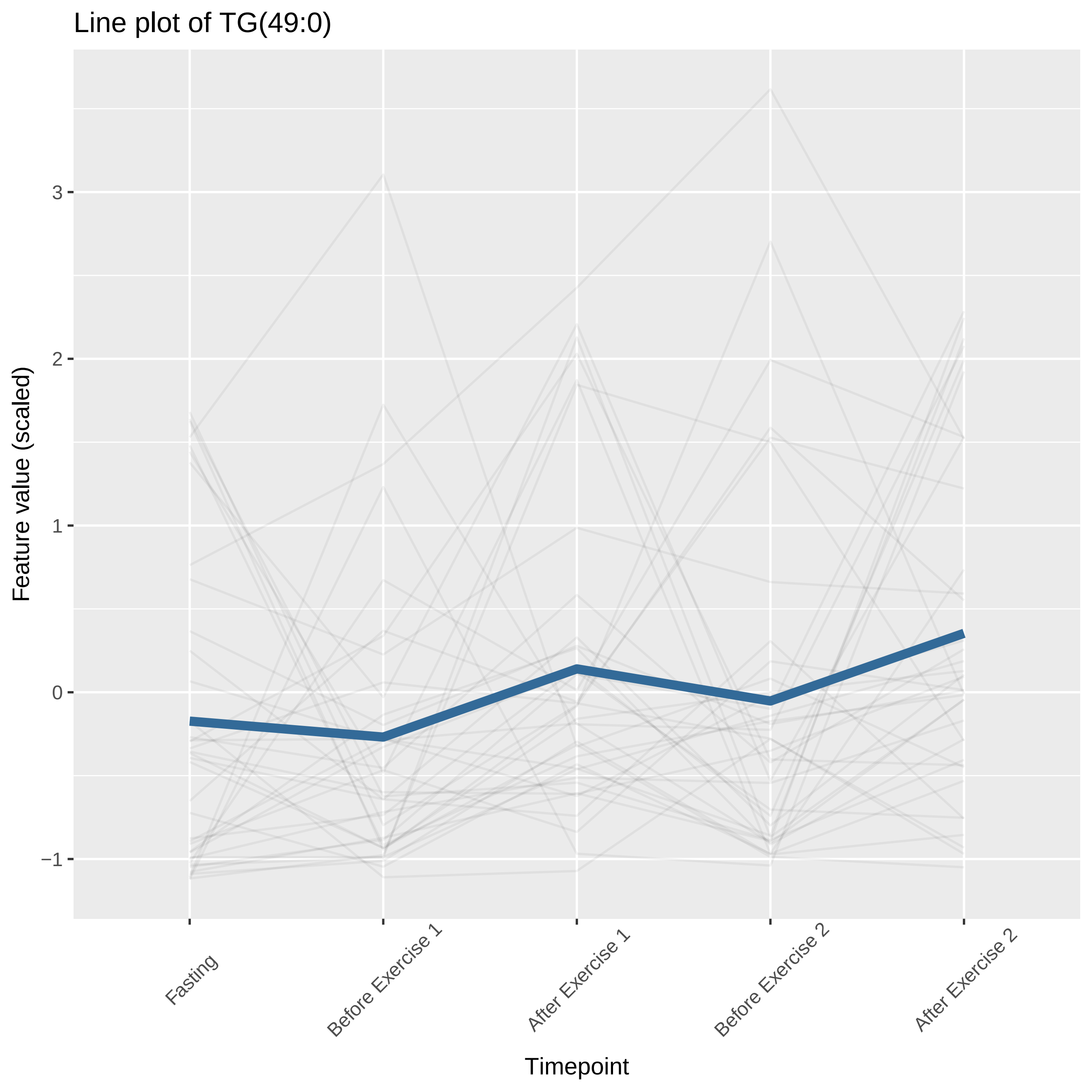

Line plot of TG(49:0)
Feature value (scaled)
3
2
1
0
−1
Fasting
Before Exercise 1
After Exercise 1
Before Exercise 2
After Exercise 2
Timepoint

Line plot of TG(49:0)

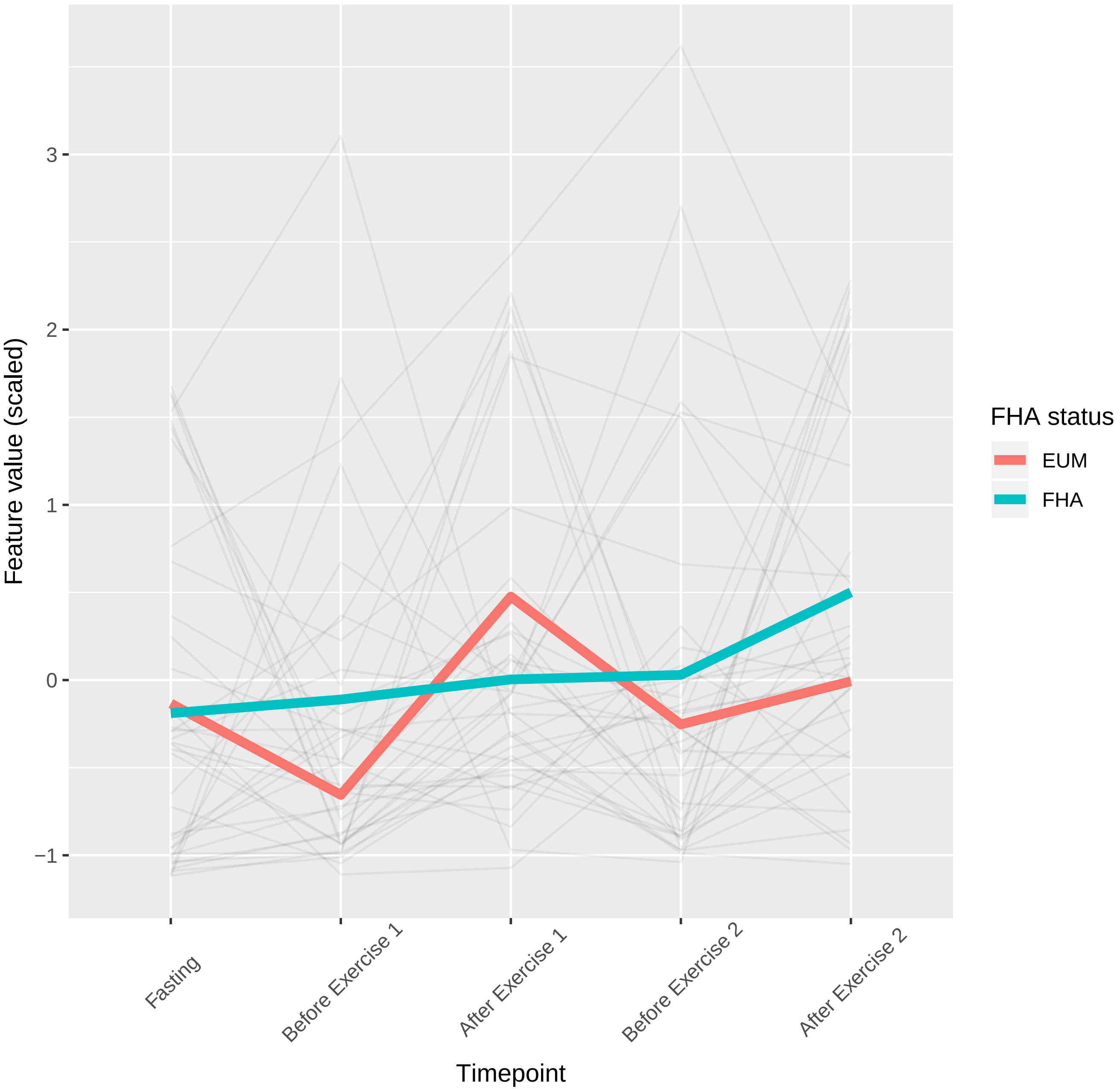

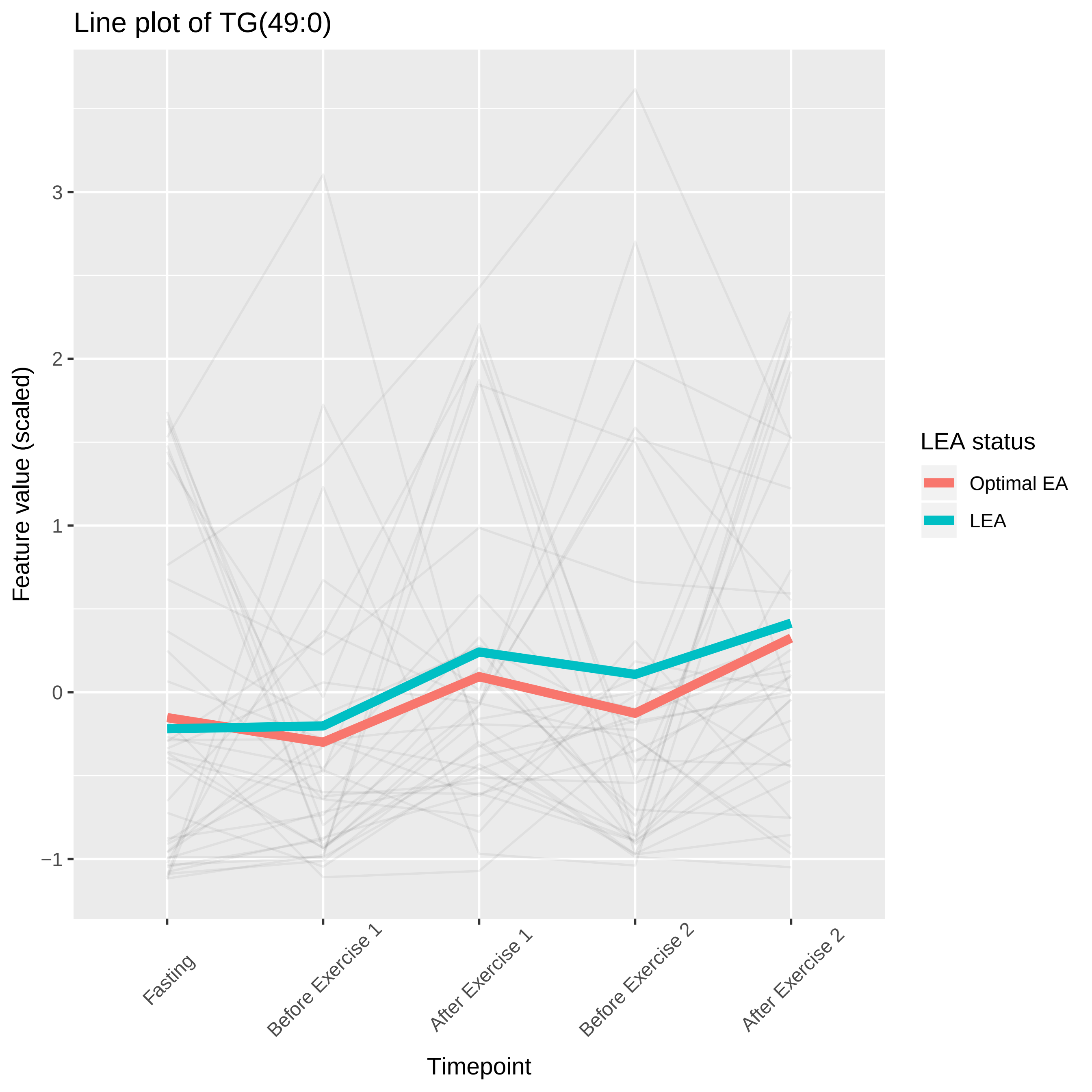
Line plot of TG(49:0)
Feature value (scaled)
3
2
1
0
−1
Fasting
Before Exercise 1
After Exercise 1
Before Exercise 2
After Exercise 2
Timepoint
LEA status
Optimal EA
LEA

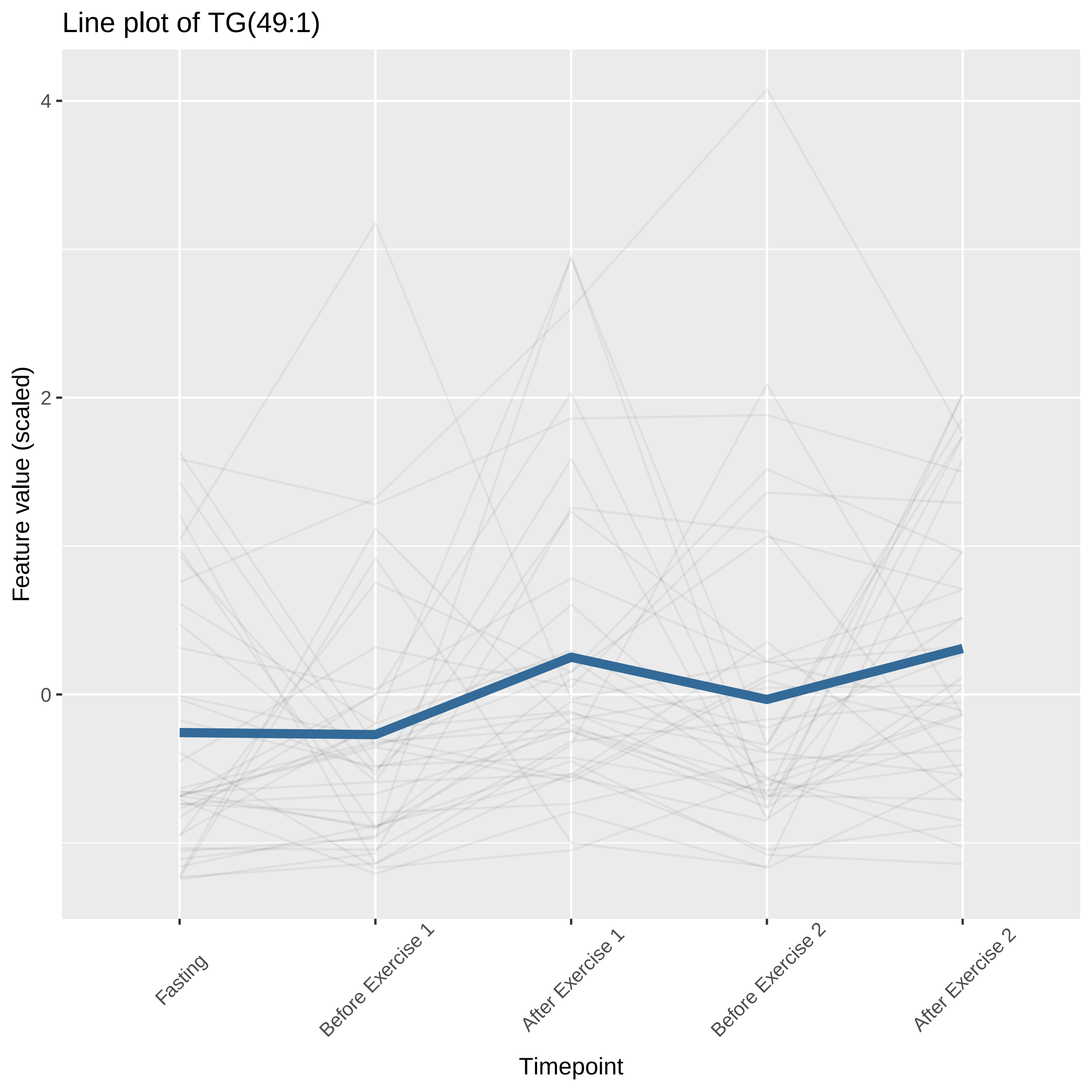
Line plot of TG(49:1)
Feature value (scaled)
4
2
0
Fasting
Before Exercise 1
After Exercise 1
Before Exercise 2
After Exercise 2
Timepoint

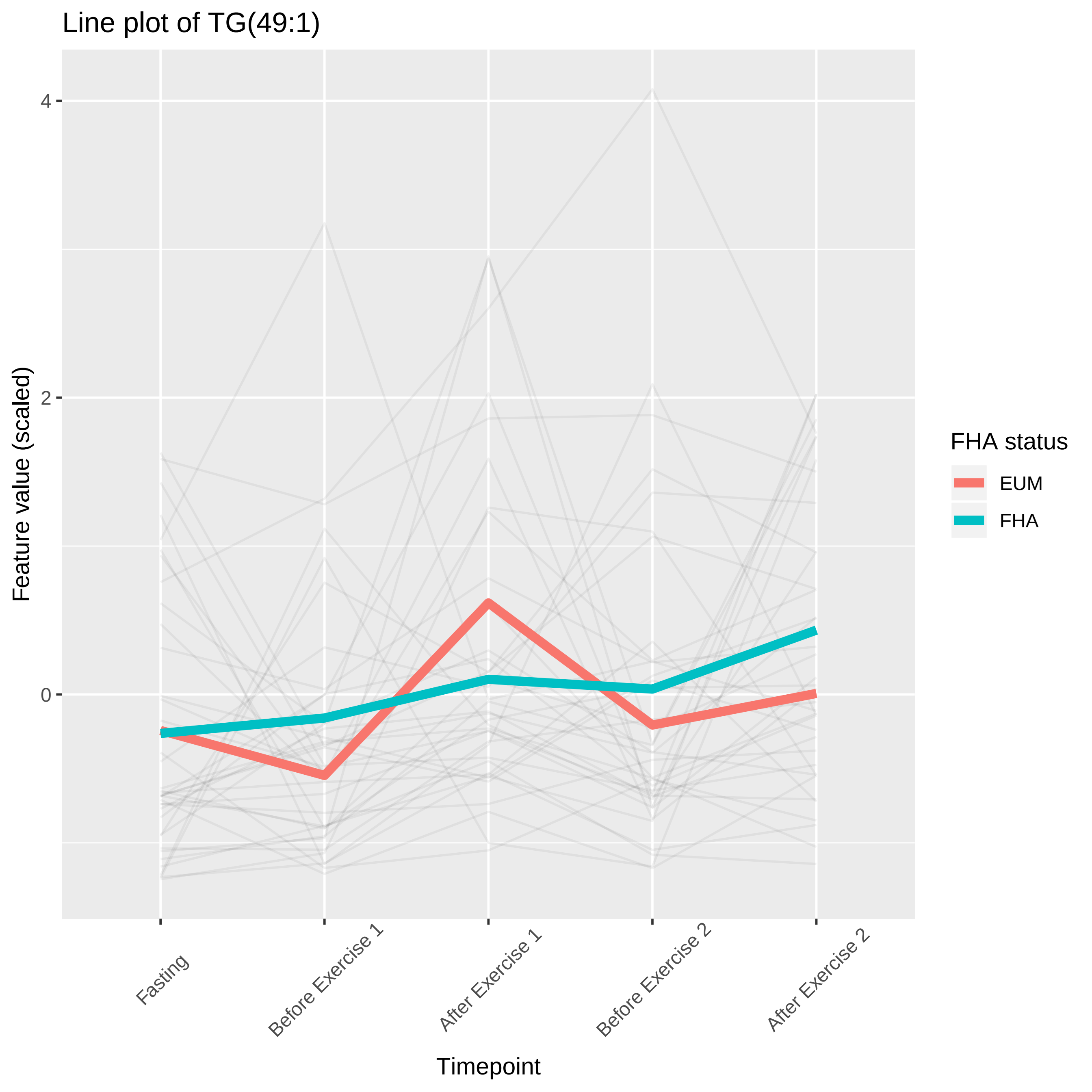

Line plot of TG(49:1)
Feature value (scaled)
4
2
0
Fasting
Before Exercise 1
After Exercise 1
Before Exercise 2
After Exercise 2
Timepoint
FHA status
EUM
FHA

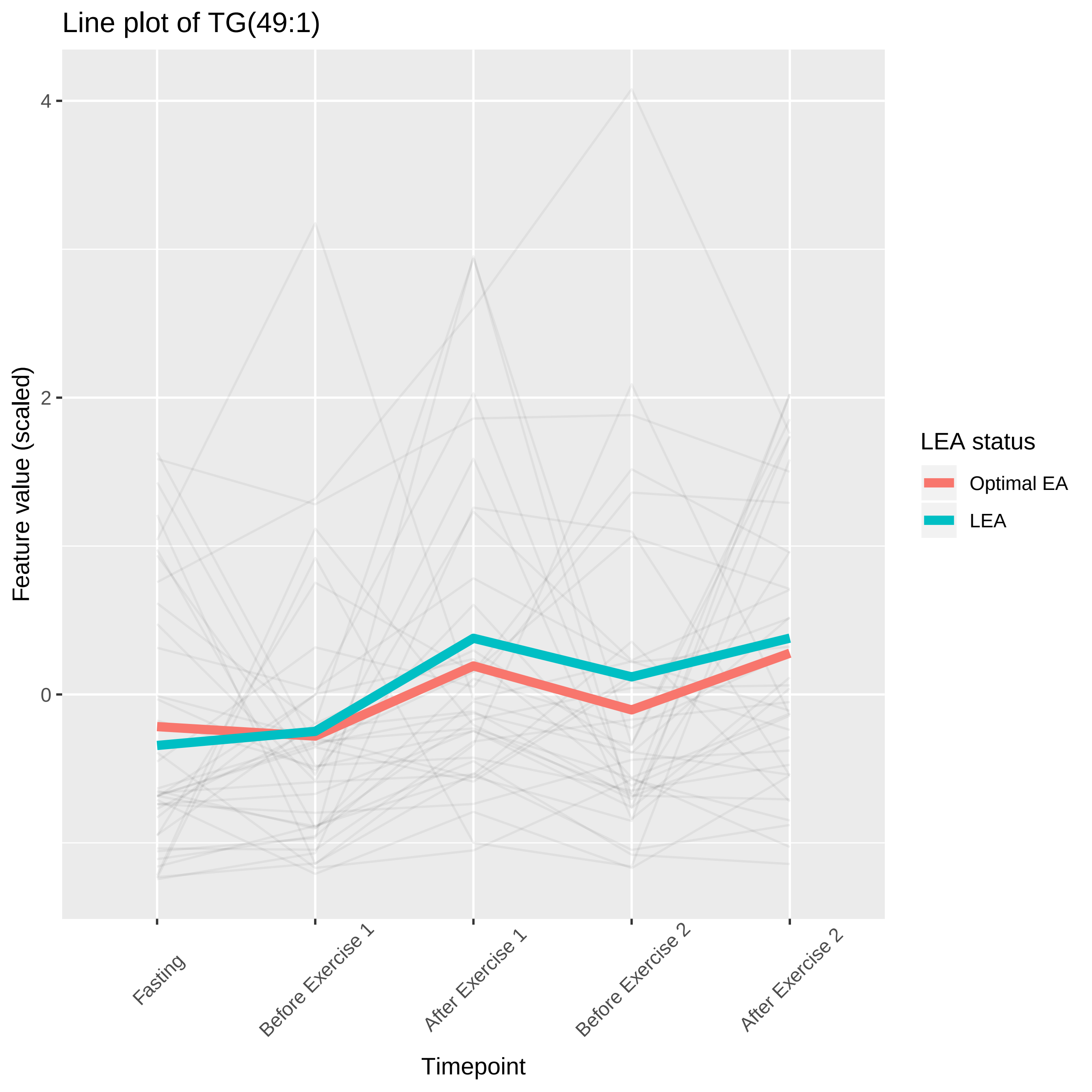

Line plot of TG(49:1)
Feature value (scaled)
4
2
0
Fasting
Before Exercise 1
After Exercise 1
Before Exercise 2
After Exercise 2
Timepoint
LEA status
Optimal EA
LEA

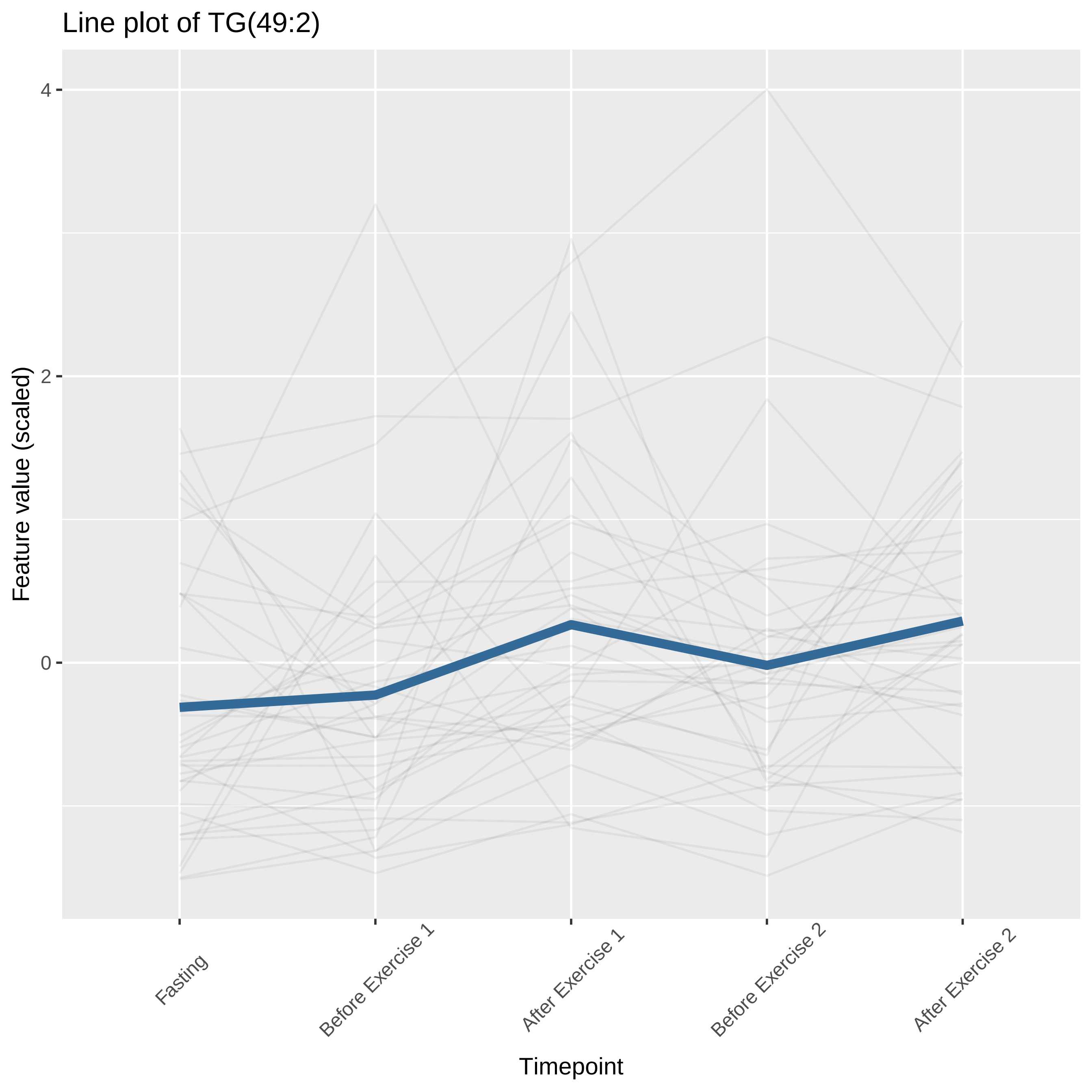
Line plot of TG(49:2)
Feature value (scaled)
4
2
0
Fasting
Before Exercise 1
After Exercise 1
Before Exercise 2
After Exercise 2
Timepoint

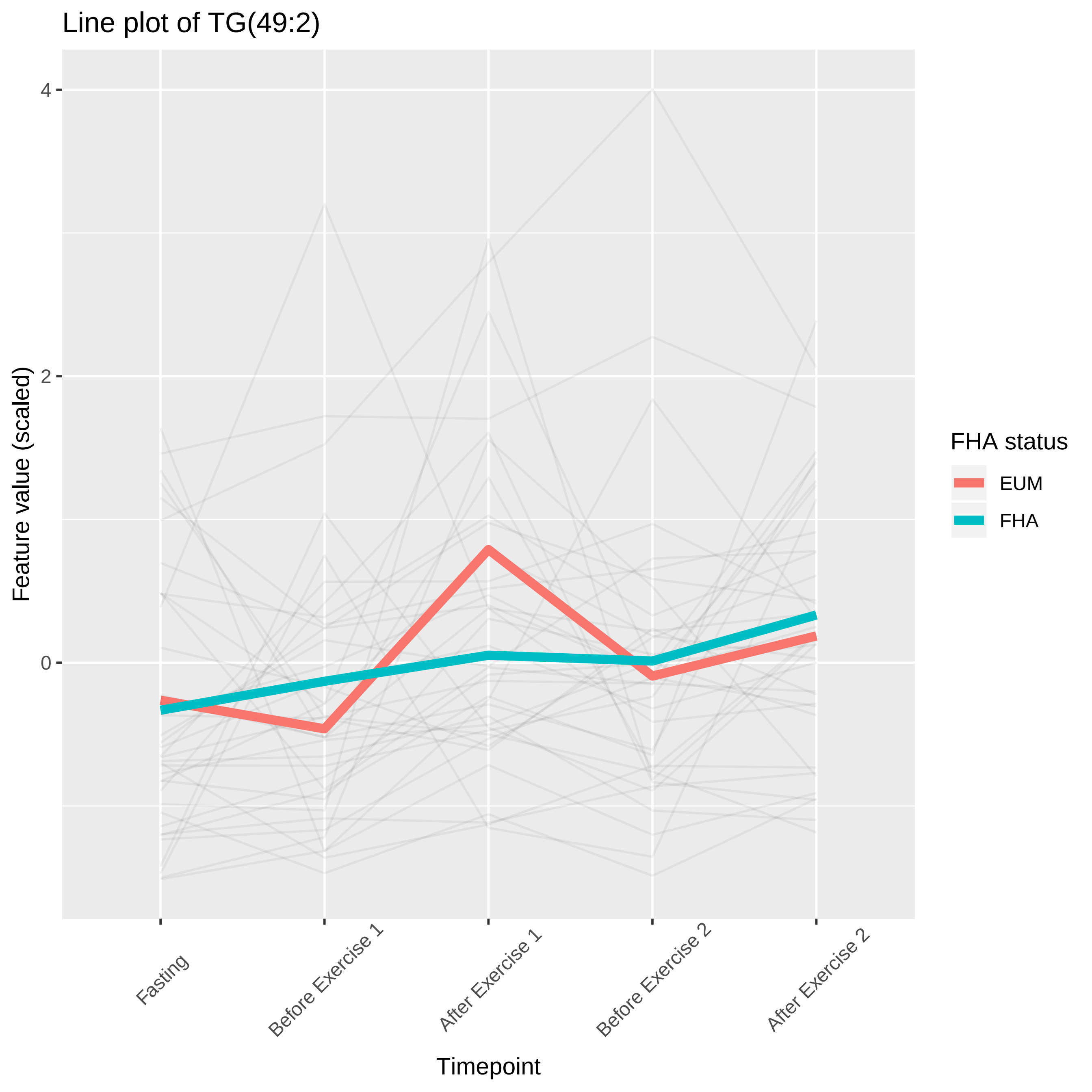

Line plot of TG(49:2)
Feature value (scaled)
4
2
0
Fasting
Before Exercise 1
After Exercise 1
Before Exercise 2
After Exercise 2
Timepoint
FHA status
EUM
FHA

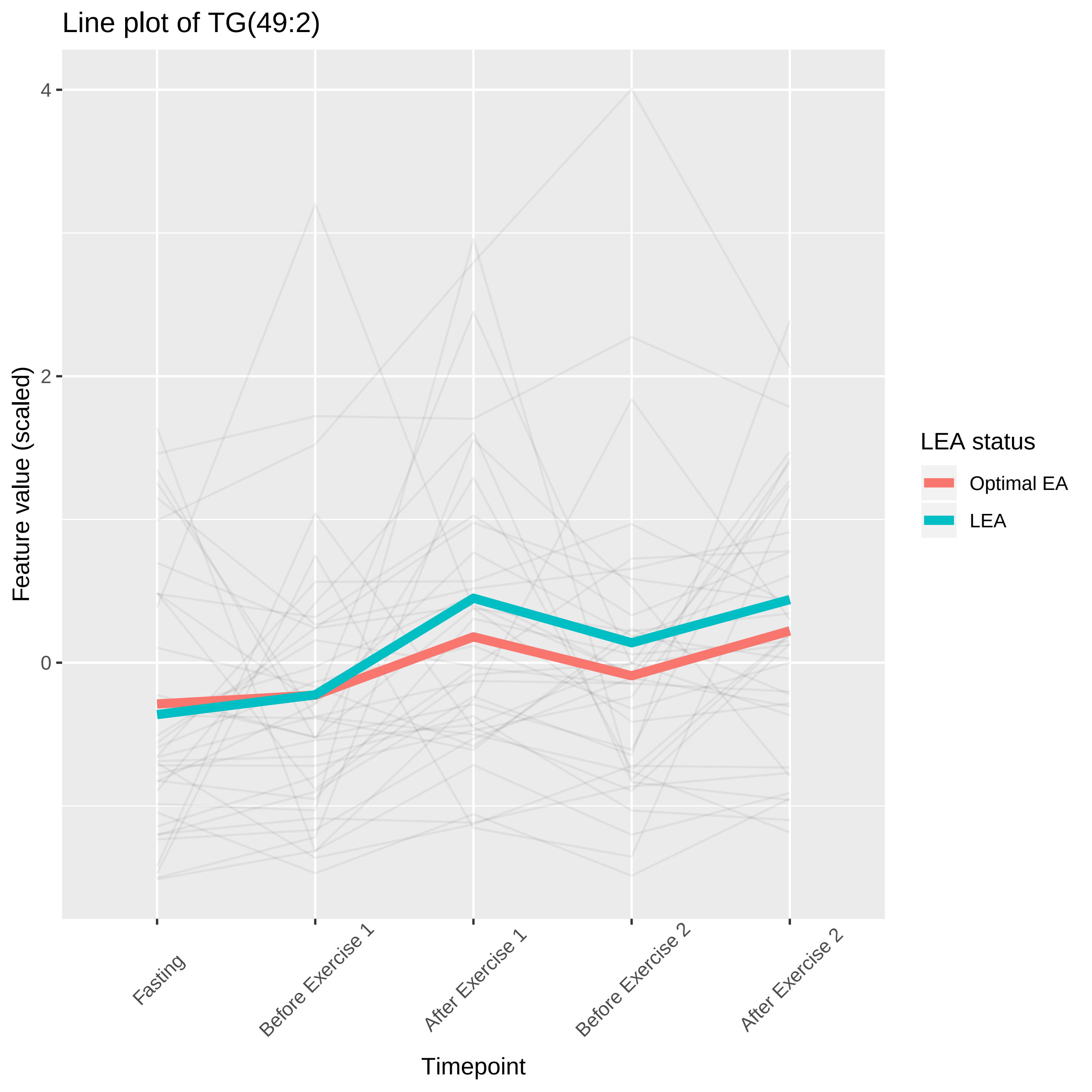
Line plot of TG(49:2)
Feature value (scaled)
4
2
0
Fasting
Before Exercise 1
After Exercise 1
Before Exercise 2
After Exercise 2
Timepoint
LEA status
Optimal EA
LEA

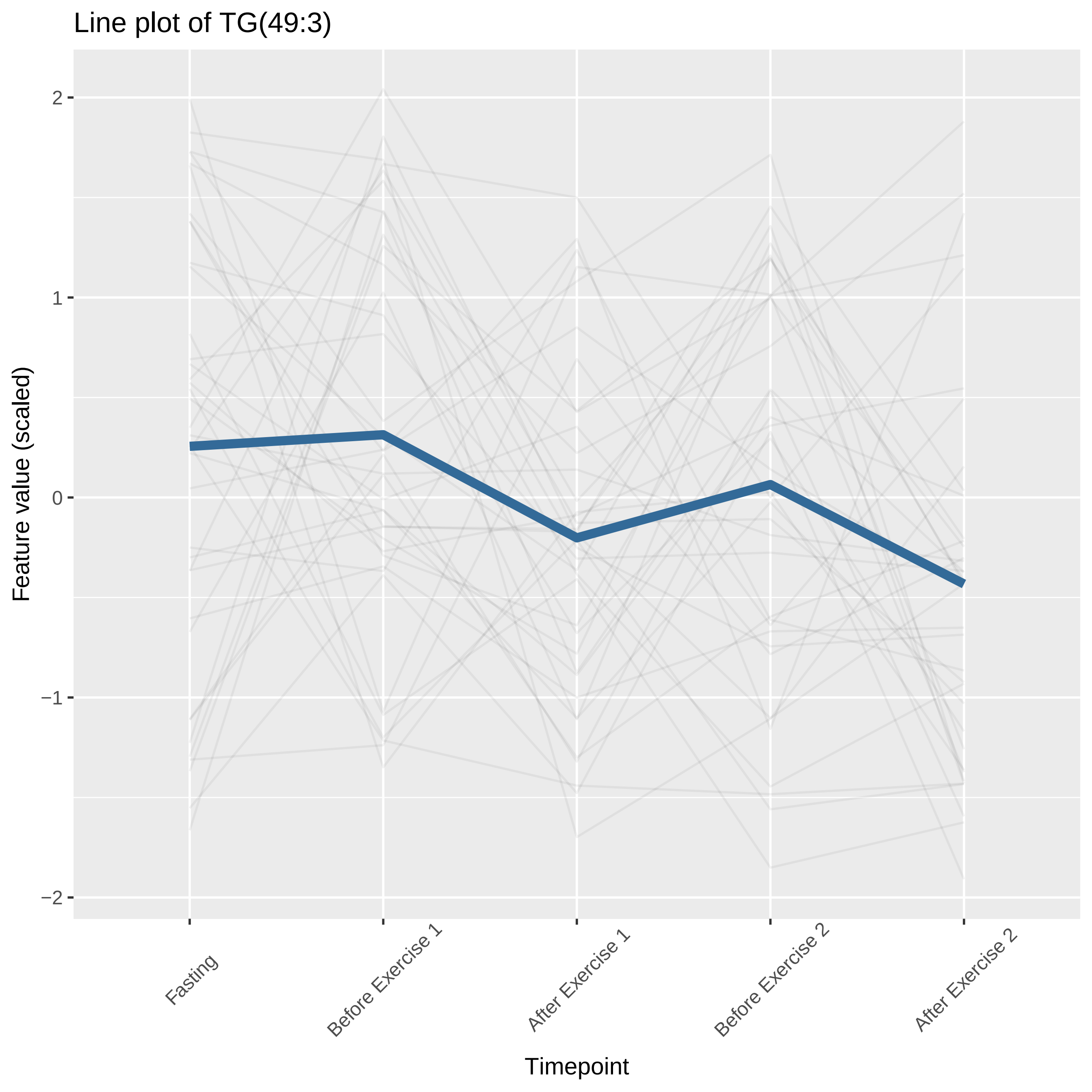

Line plot of TG(49:3)
Feature value (scaled)
2
1
0
−1
−2
Fasting
Before Exercise 1
After Exercise 1
Before Exercise 2
After Exercise 2
Timepoint

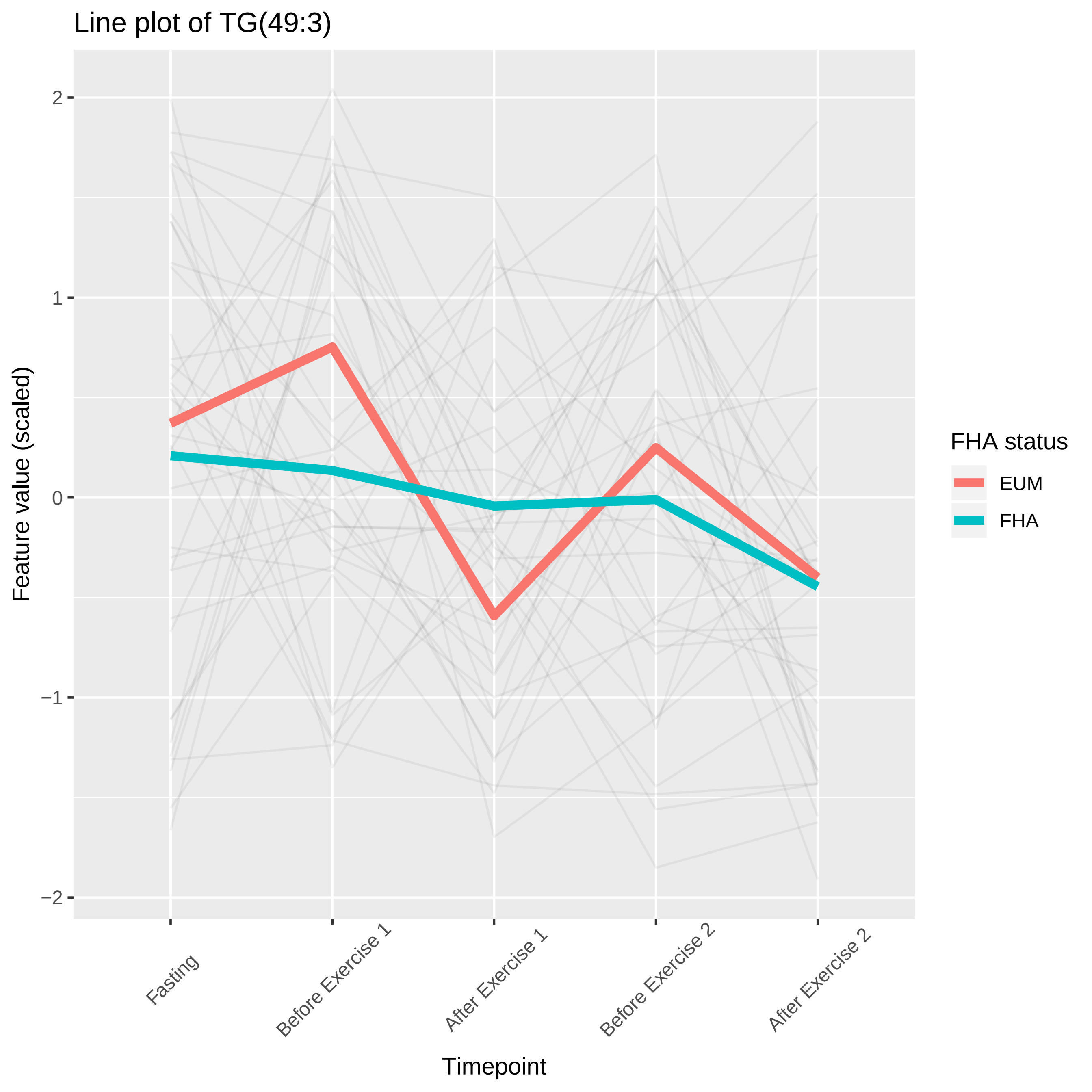

Line plot of TG(49:3)
Feature value (scaled)
2
1
0
−1
−2
Fasting
Before Exercise 1
After Exercise 1
Before Exercise 2
After Exercise 2
Timepoint
FHA status
EUM
FHA

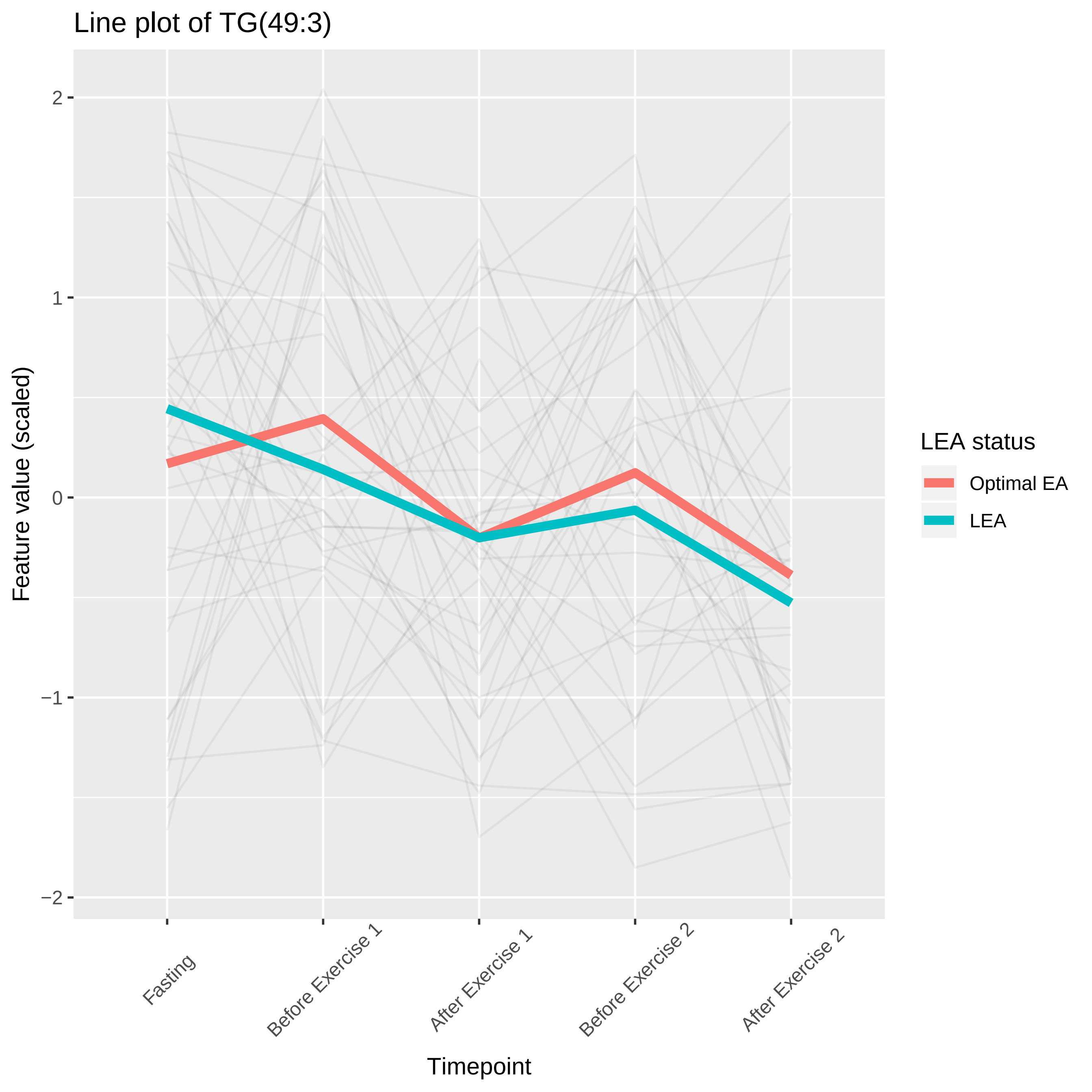

Line plot of TG(49:3)
Feature value (scaled)
2
1
0
−1
−2
Fasting
Before Exercise 1
After Exercise 1
Before Exercise 2
After Exercise 2
Timepoint
LEA status
Optimal EA
LEA

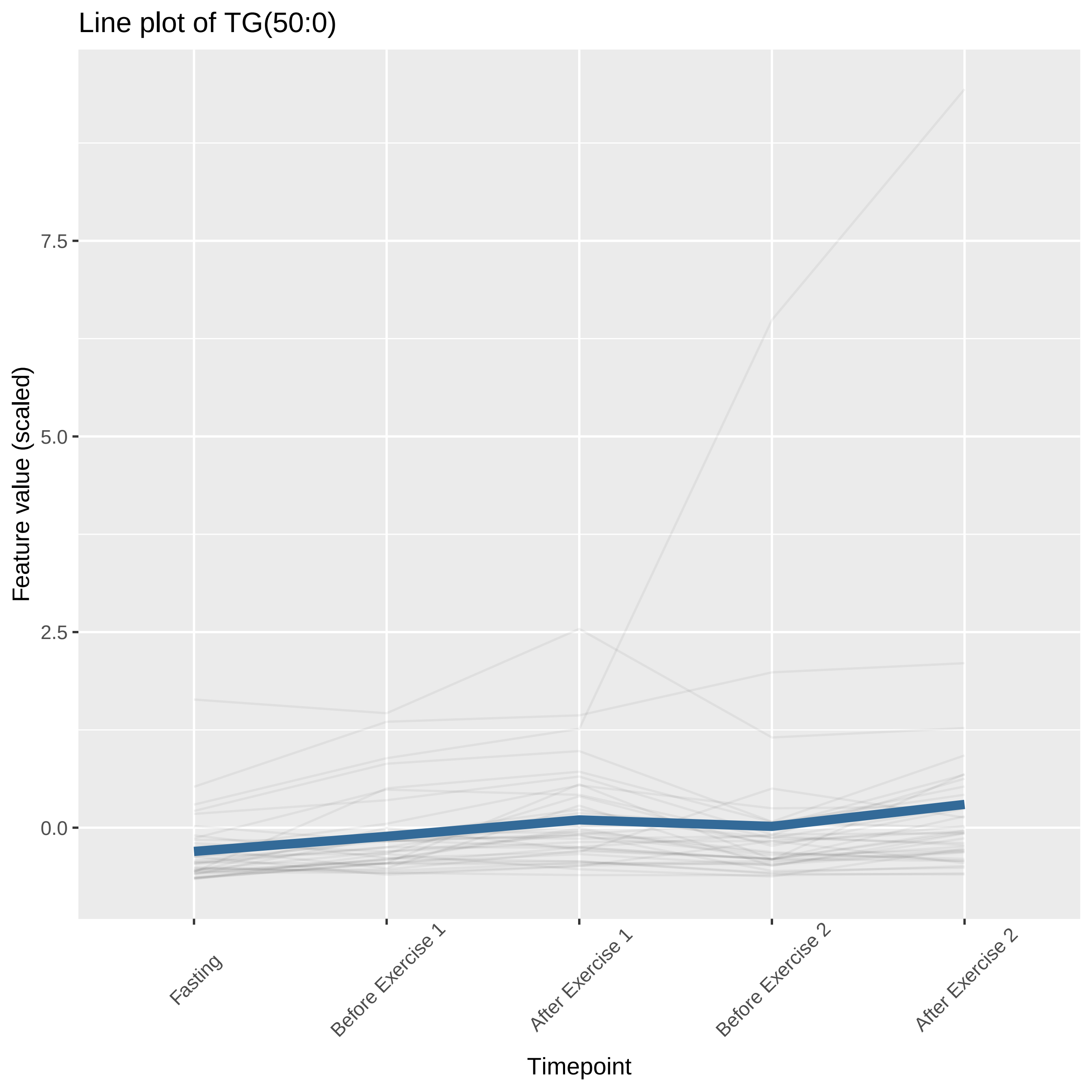

Line plot of TG(50:0)
Feature value (scaled)
7.5
5.0
2.5
0.0
Fasting
Before Exercise 1
After Exercise 1
Before Exercise 2
After Exercise 2
Timepoint

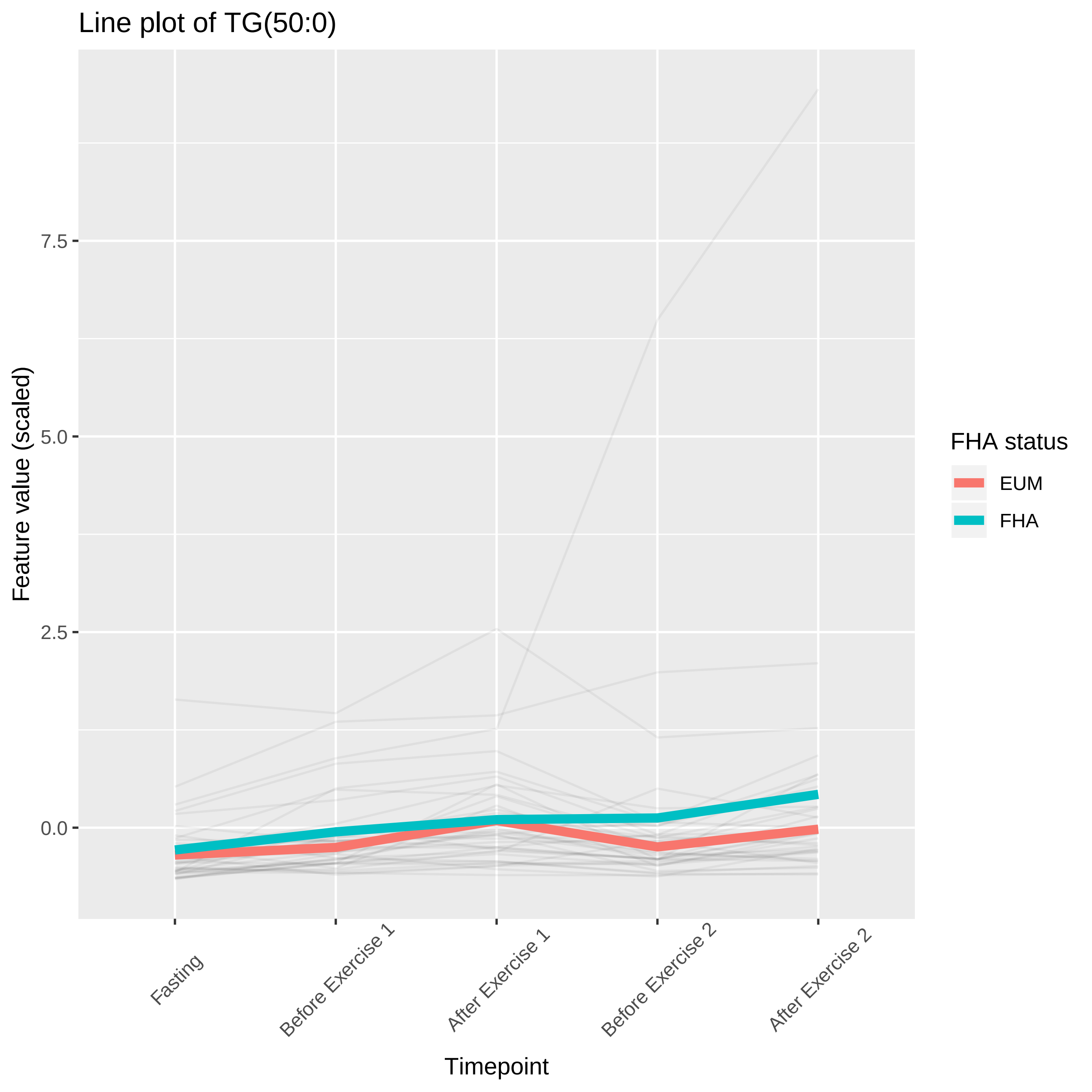

Line plot of TG(50:0)
Feature value (scaled)
7.5
5.0
2.5
0.0
Fasting
Before Exercise 1
After Exercise 1
Before Exercise 2
After Exercise 2
Timepoint
FHA status
EUM
FHA

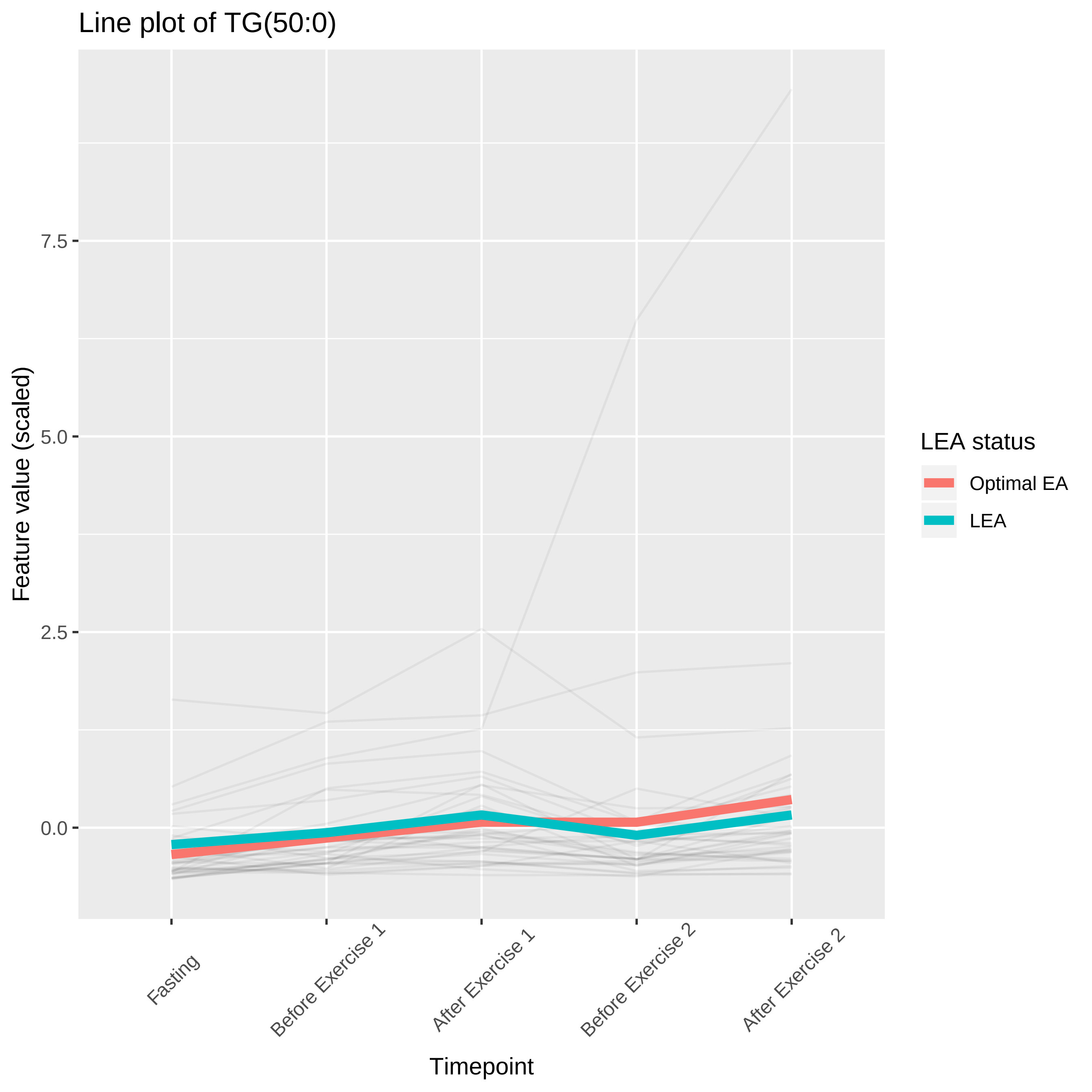

Line plot of TG(50:0)
Feature value (scaled)
7.5
5.0
2.5
0.0
Fasting
Before Exercise 1
After Exercise 1
Before Exercise 2
After Exercise 2
Timepoint
LEA status
Optimal EA
LEA

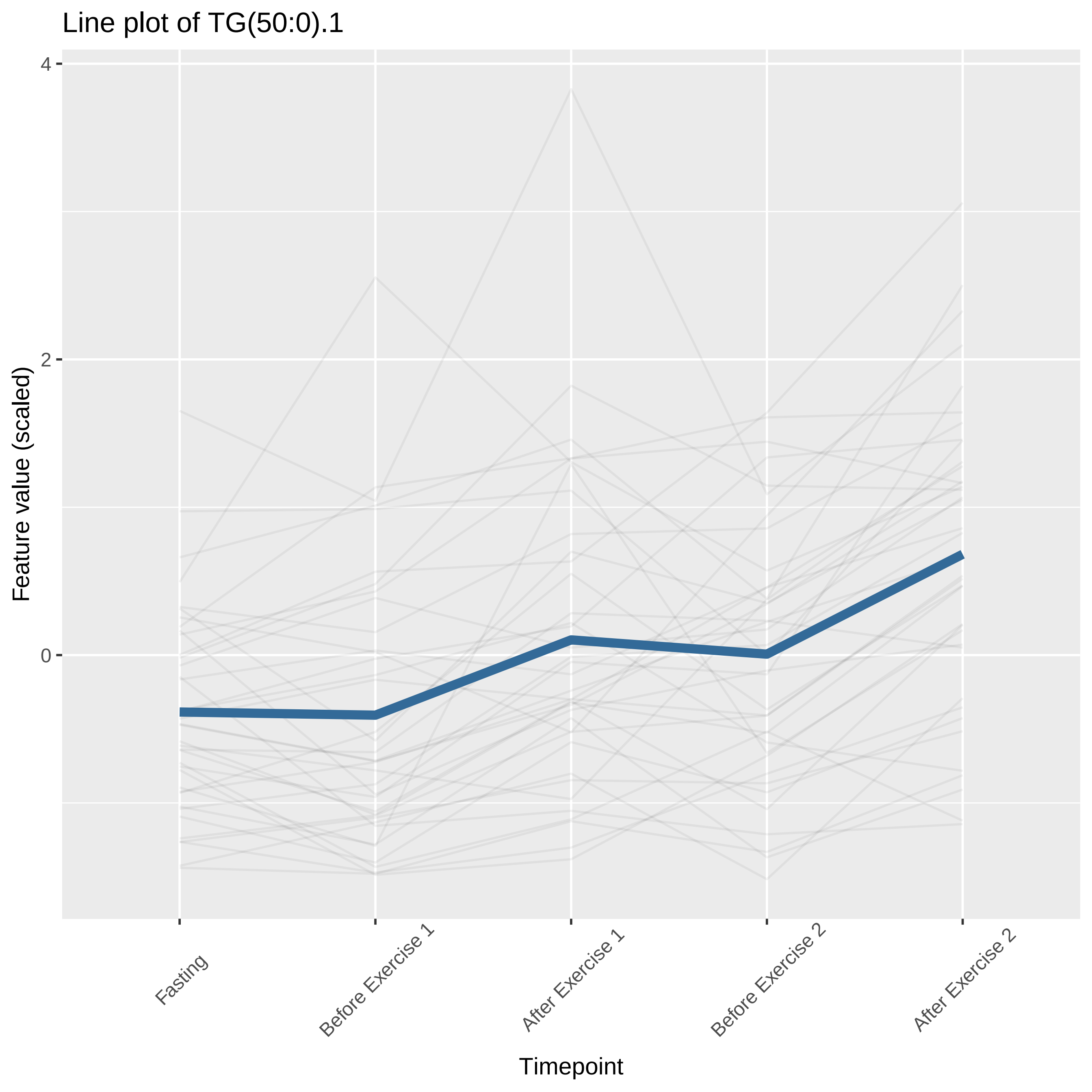

Line plot of TG(50:0).1
Feature value (scaled)
4
2
0
Fasting
Before Exercise 1
After Exercise 1
Before Exercise 2
After Exercise 2
Timepoint

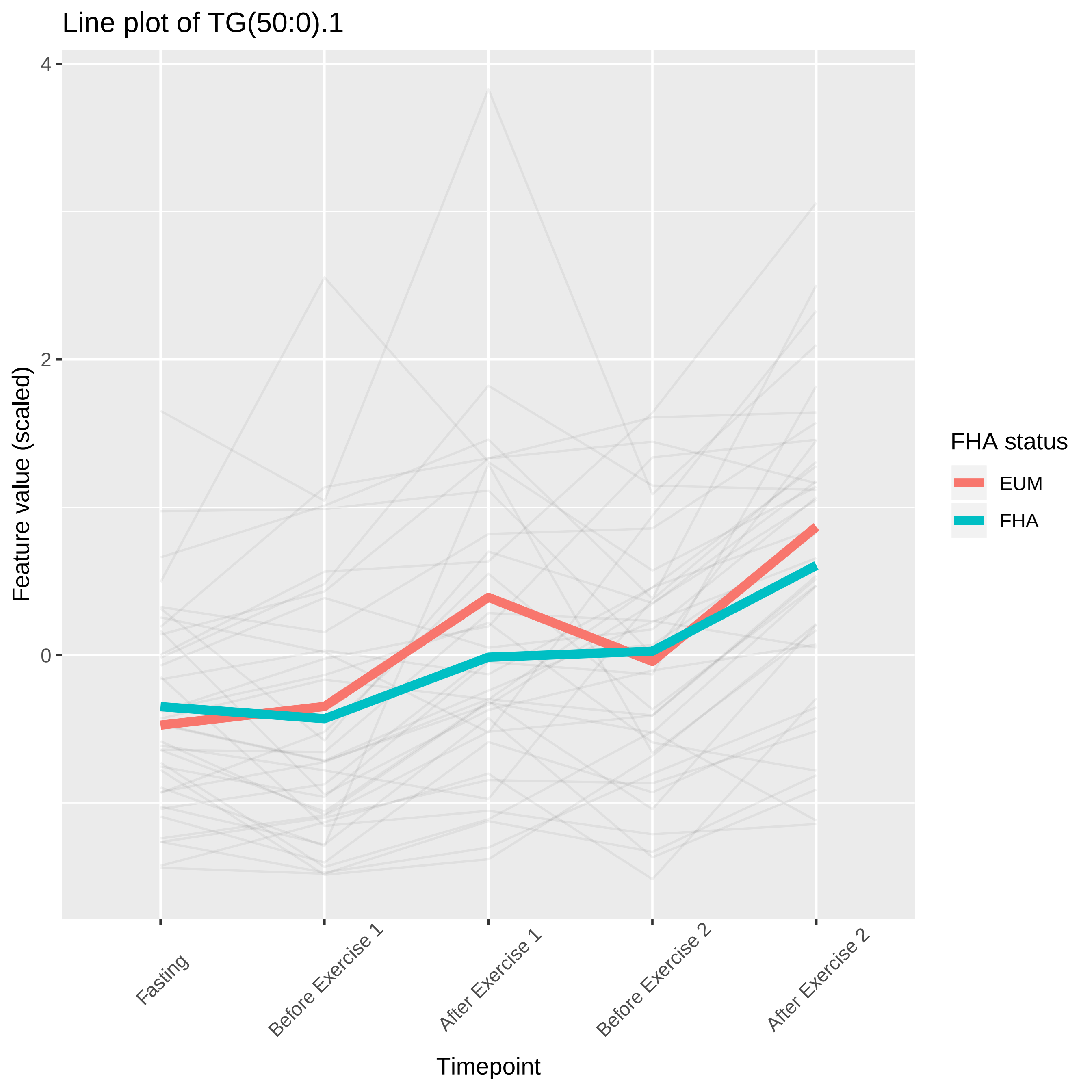

Line plot of TG(50:0).1
Feature value (scaled)
4
2
0
Fasting
Before Exercise 1
After Exercise 1
Before Exercise 2
After Exercise 2
Timepoint
FHA status
EUM
FHA

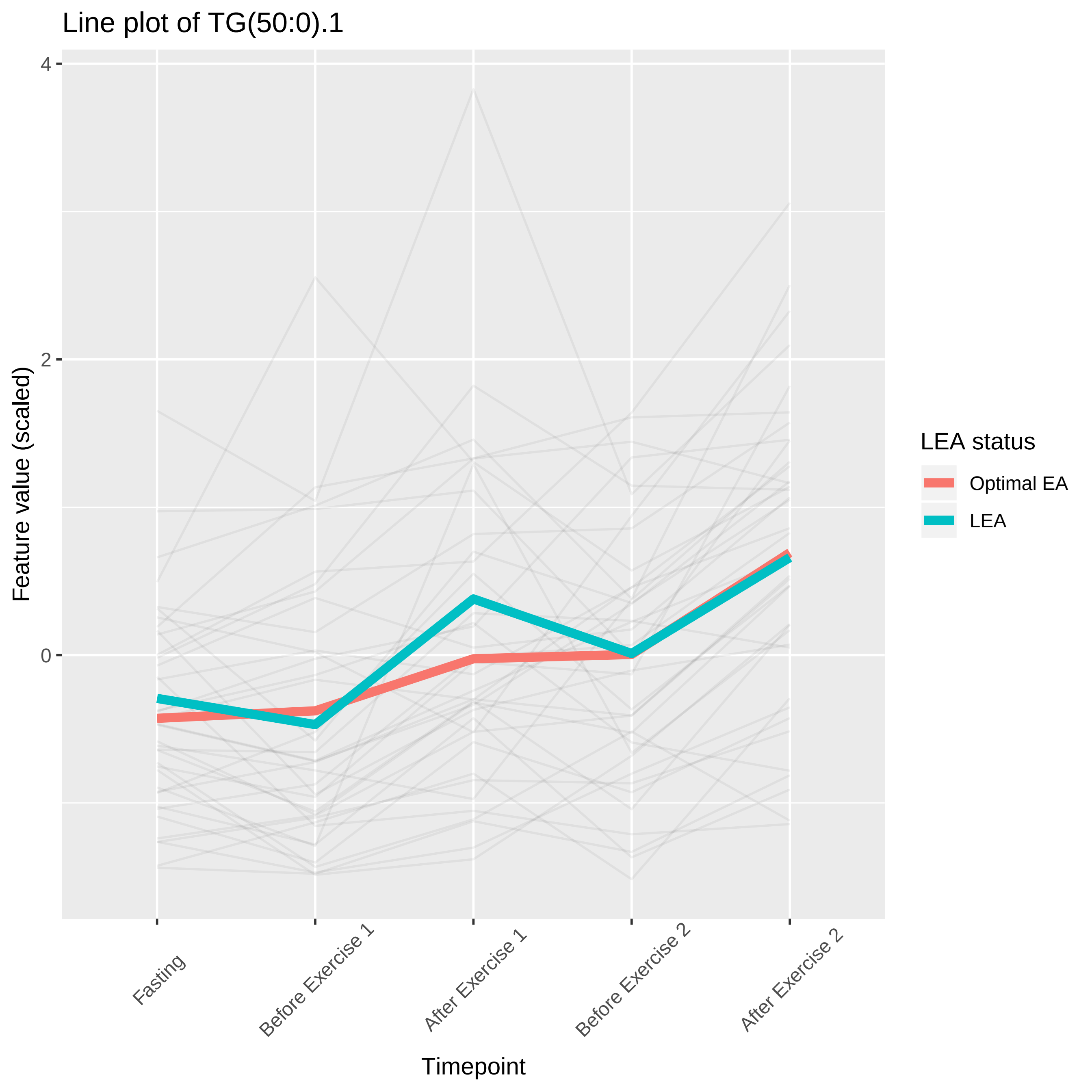

Line plot of TG(50:0).1
Feature value (scaled)
4
2
0
Fasting
Before Exercise 1
After Exercise 1
Before Exercise 2
After Exercise 2
Timepoint
LEA status
Optimal EA
LEA

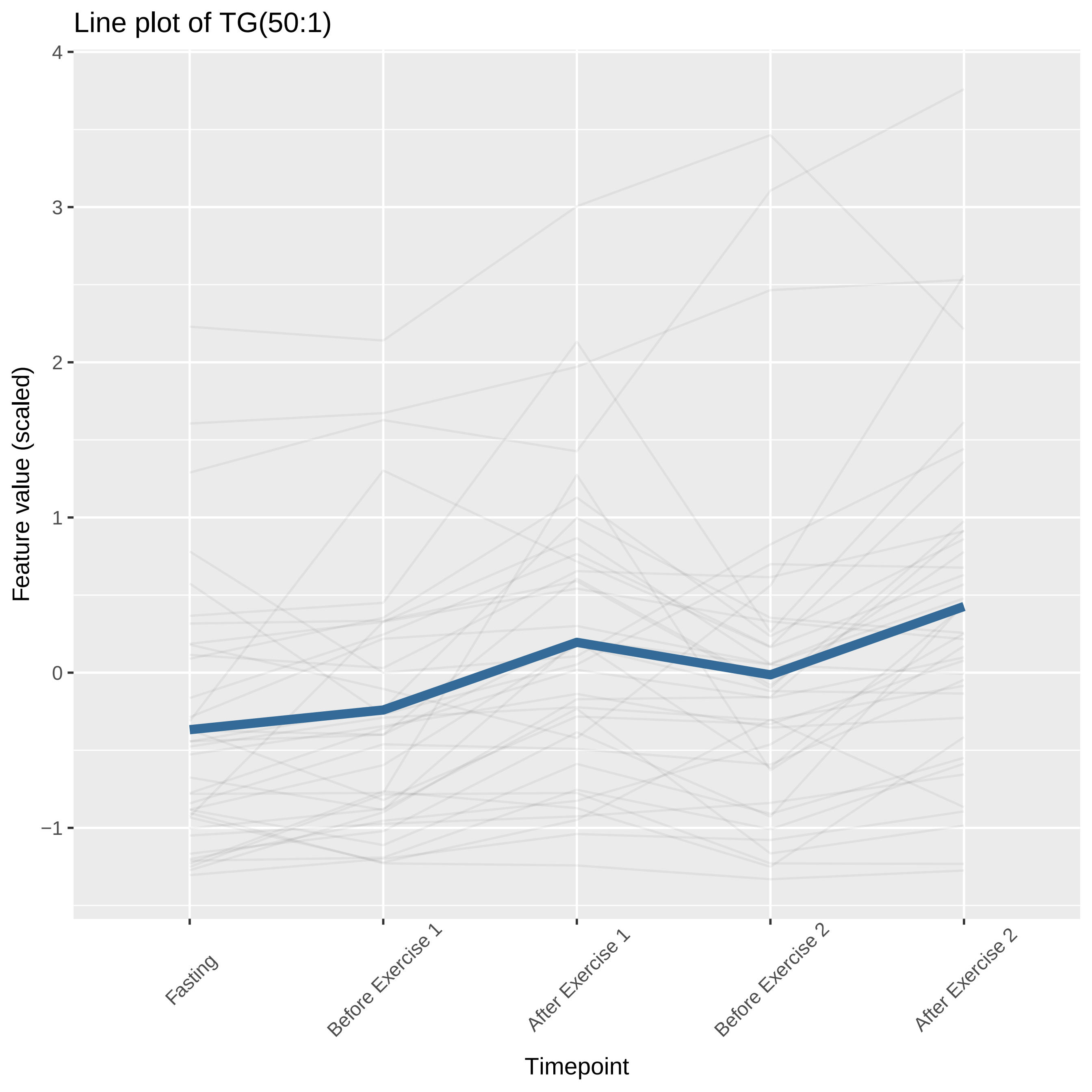
Line plot of TG(50:1)
Feature value (scaled)
4
3
2
1
0
−1
Fasting
Before Exercise 1
After Exercise 1
Before Exercise 2
After Exercise 2
Timepoint

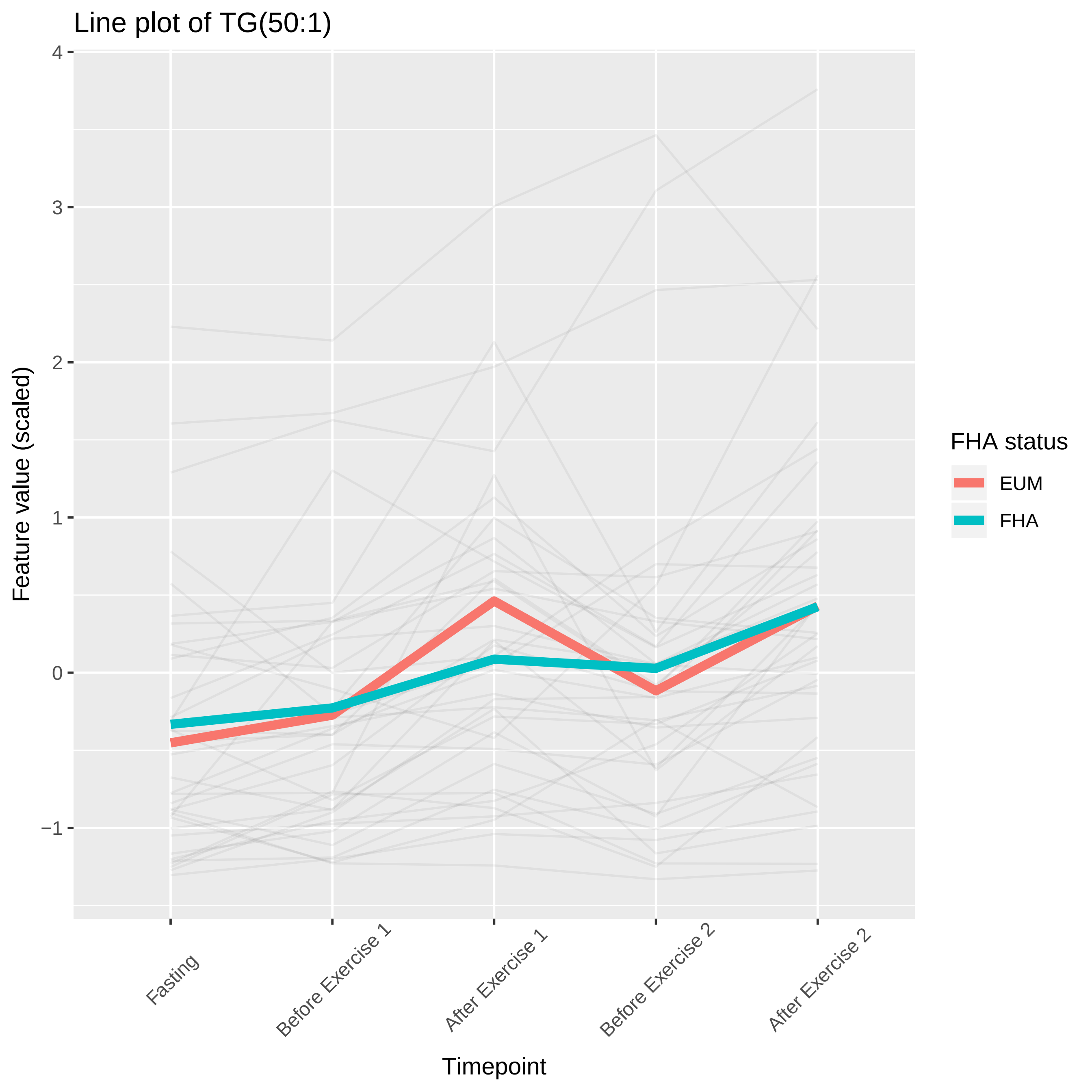

Line plot of TG(50:1)
Feature value (scaled)
4
3
2
1
0
−1
Fasting
Before Exercise 1
After Exercise 1
Before Exercise 2
After Exercise 2
Timepoint
FHA status
EUM
FHA

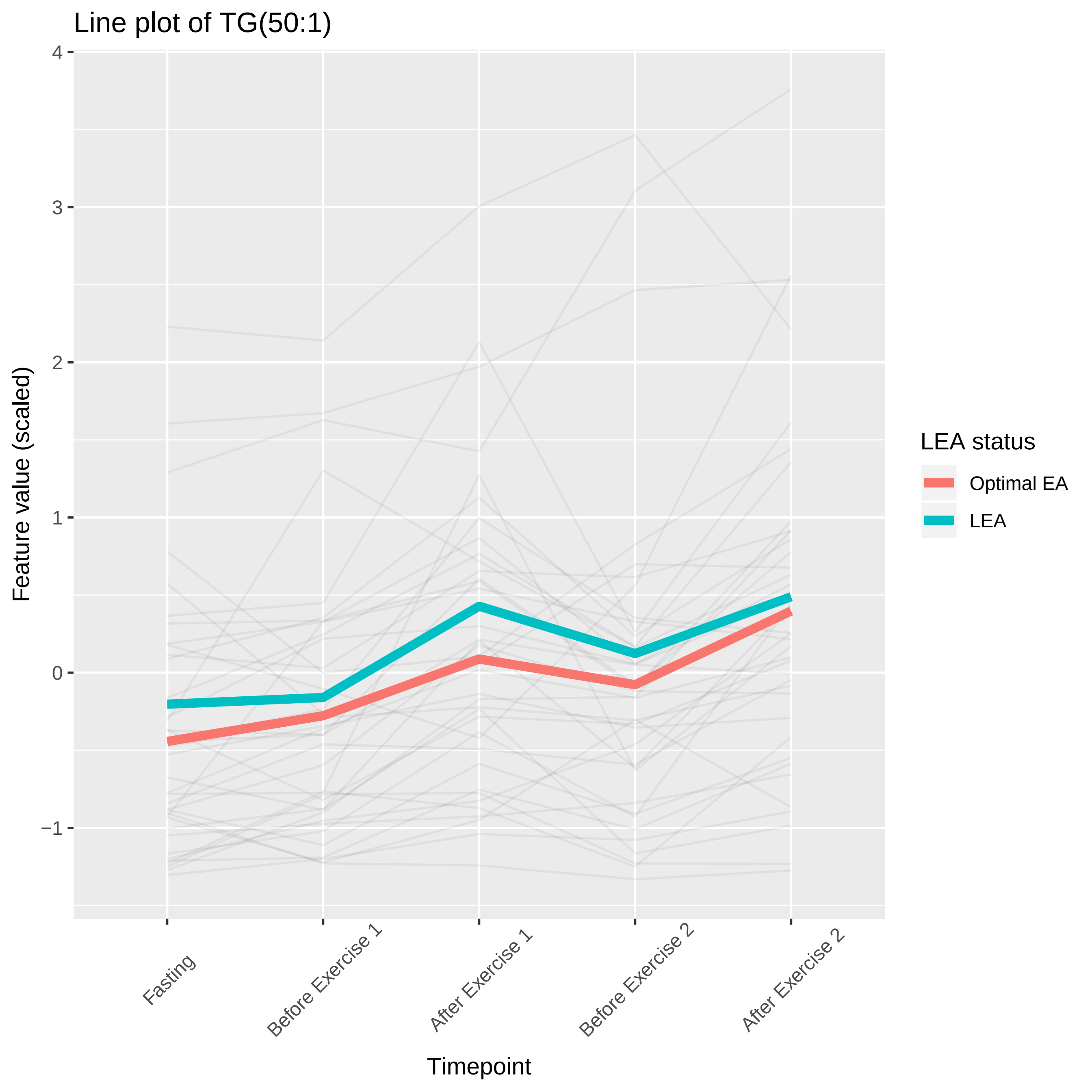

Line plot of TG(50:1)
Feature value (scaled)
4
3
2
1
0
−1
Fasting
Before Exercise 1
After Exercise 1
Before Exercise 2
After Exercise 2
Timepoint
LEA status
Optimal EA
LEA

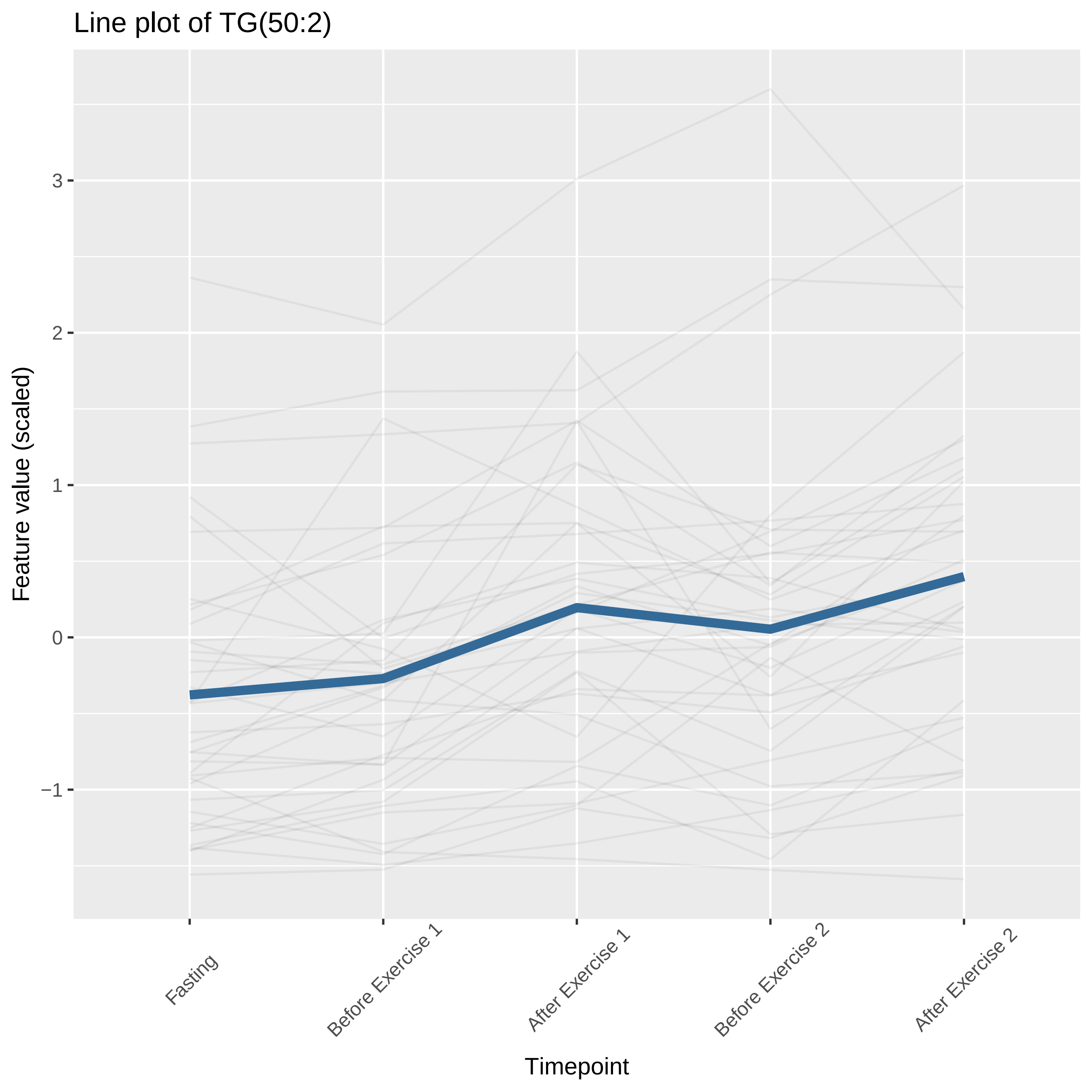

Line plot of TG(50:2)
Feature value (scaled)
3
2
1
0
−1
Fasting
Before Exercise 1
After Exercise 1
Before Exercise 2
After Exercise 2
Timepoint

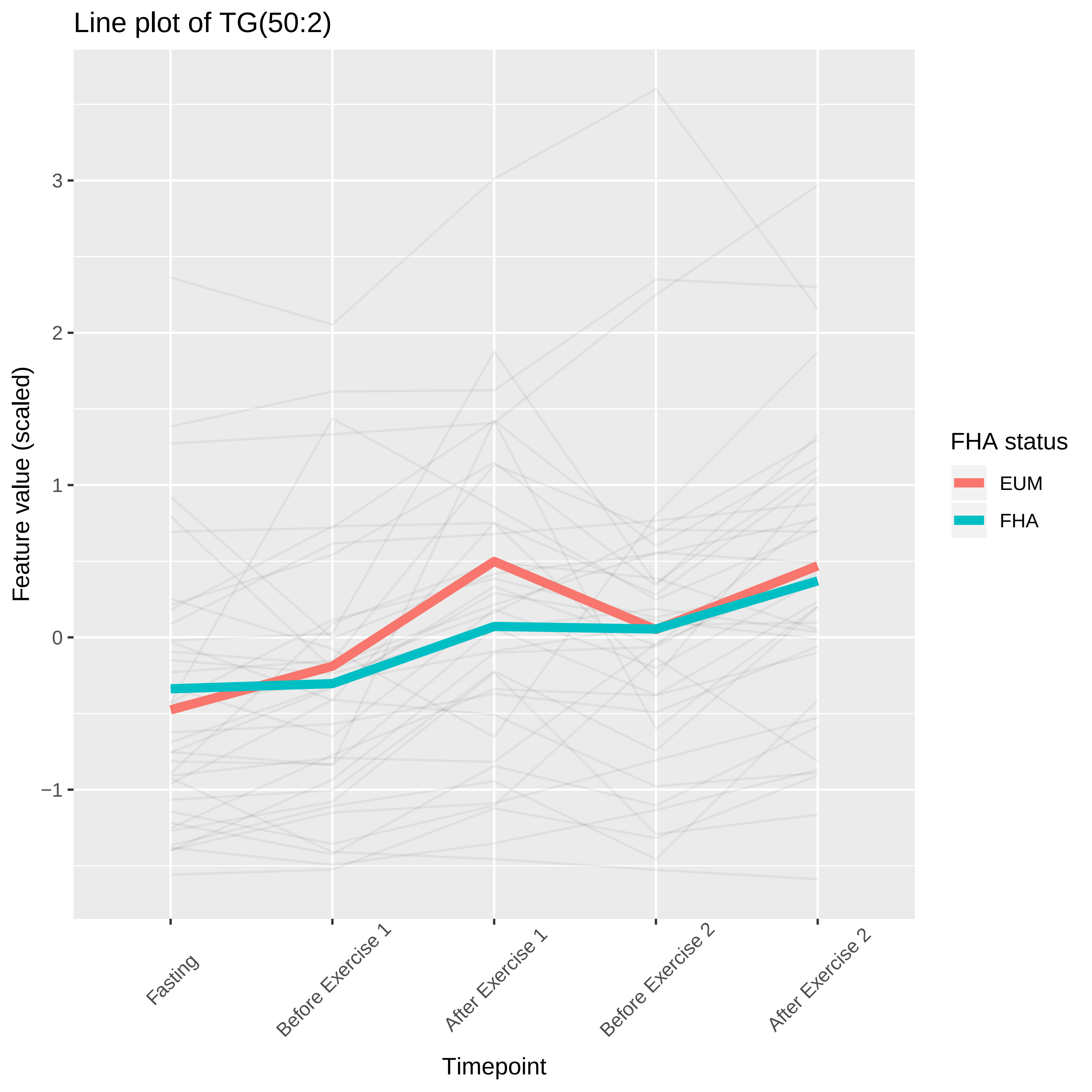
Line plot of TG(50:2)
Feature value (scaled)
3
2
1
0
−1
Fasting
Before Exercise 1
After Exercise 1
Before Exercise 2
After Exercise 2
Timepoint
FHA status
EUM
FHA

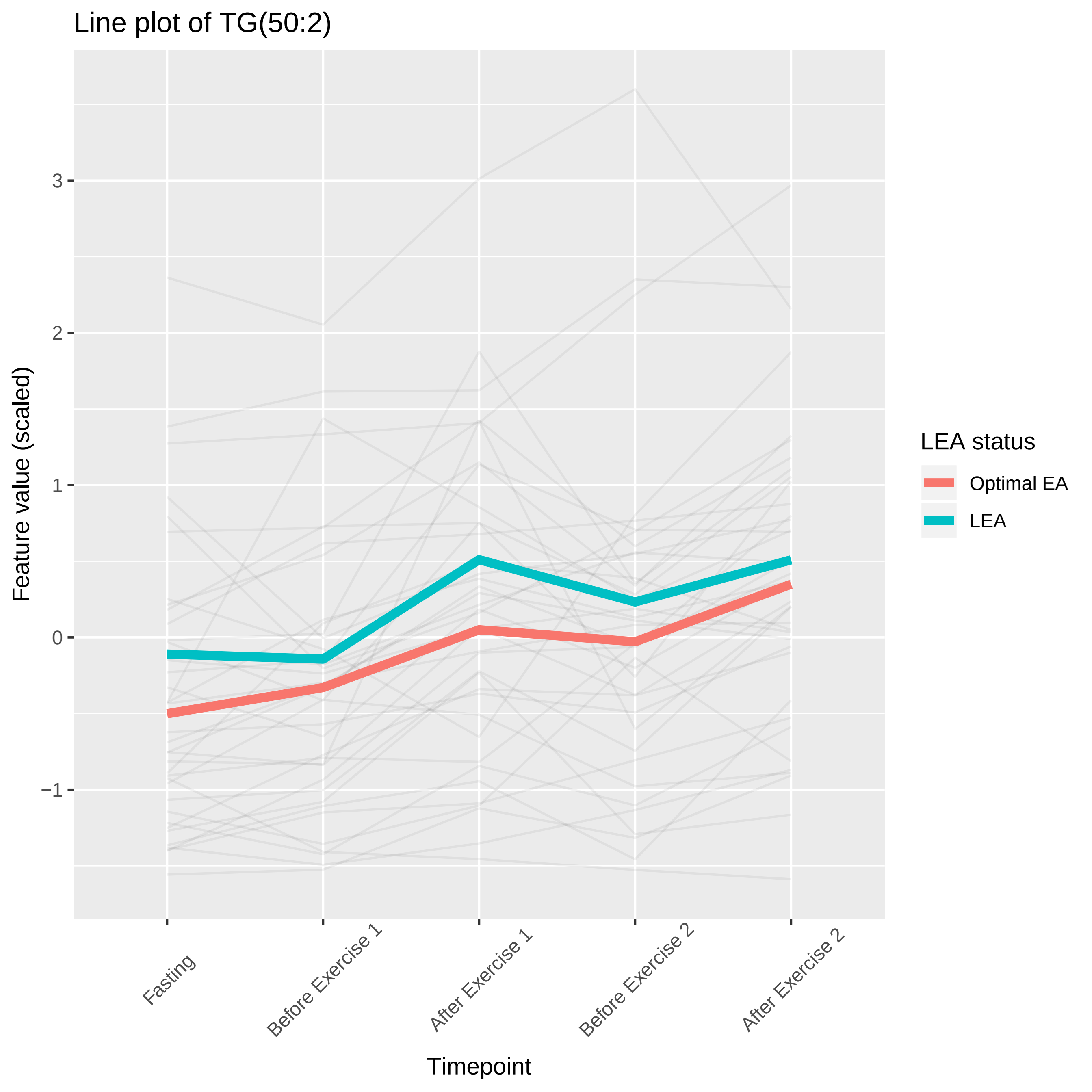

Line plot of TG(50:2)
Feature value (scaled)
3
2
1
0
−1
Fasting
Before Exercise 1
After Exercise 1
Before Exercise 2
After Exercise 2
Timepoint
LEA status
Optimal EA
LEA

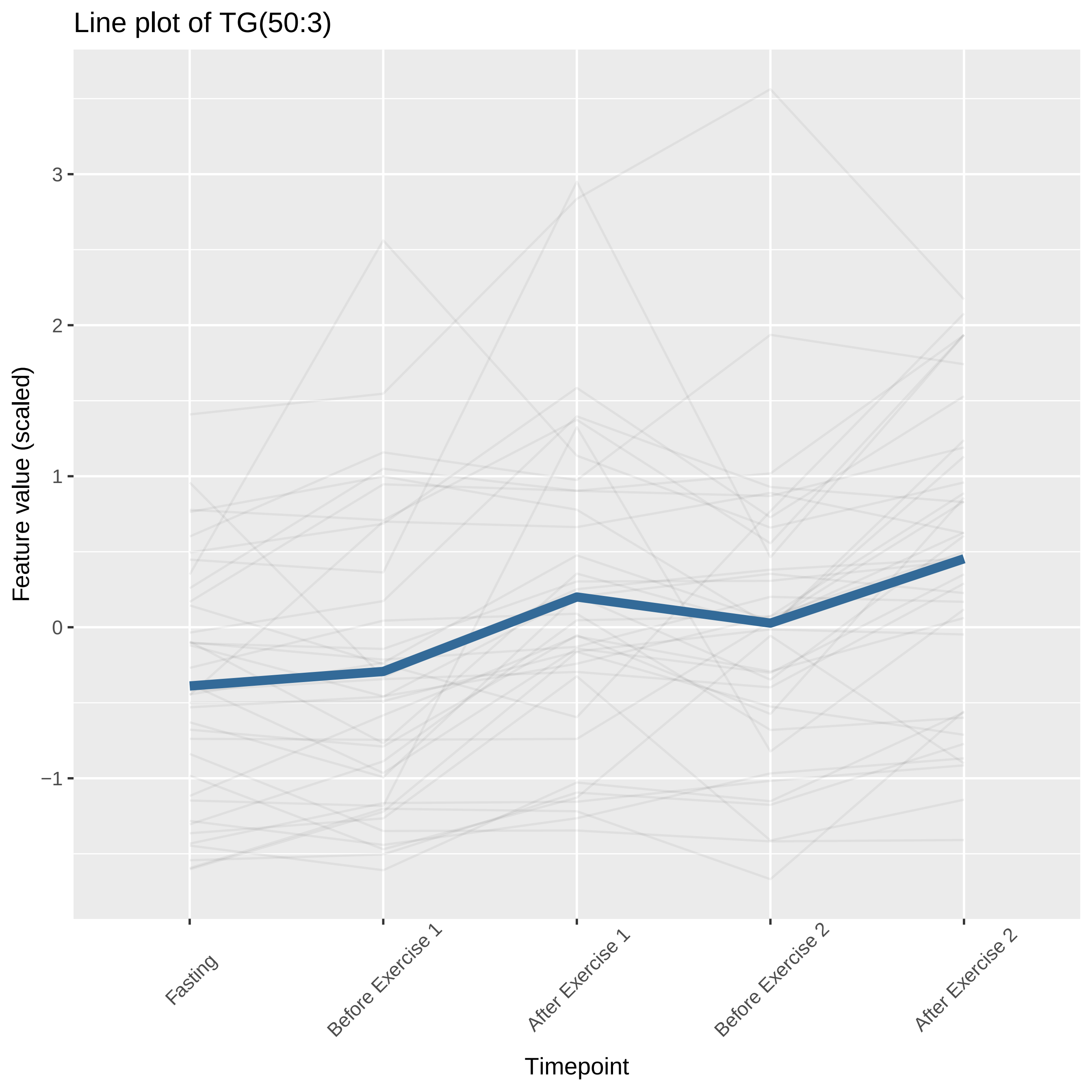

Line plot of TG(50:3)
Feature value (scaled)
3
2
1
0
−1
Fasting
Before Exercise 1
After Exercise 1
Before Exercise 2
After Exercise 2
Timepoint

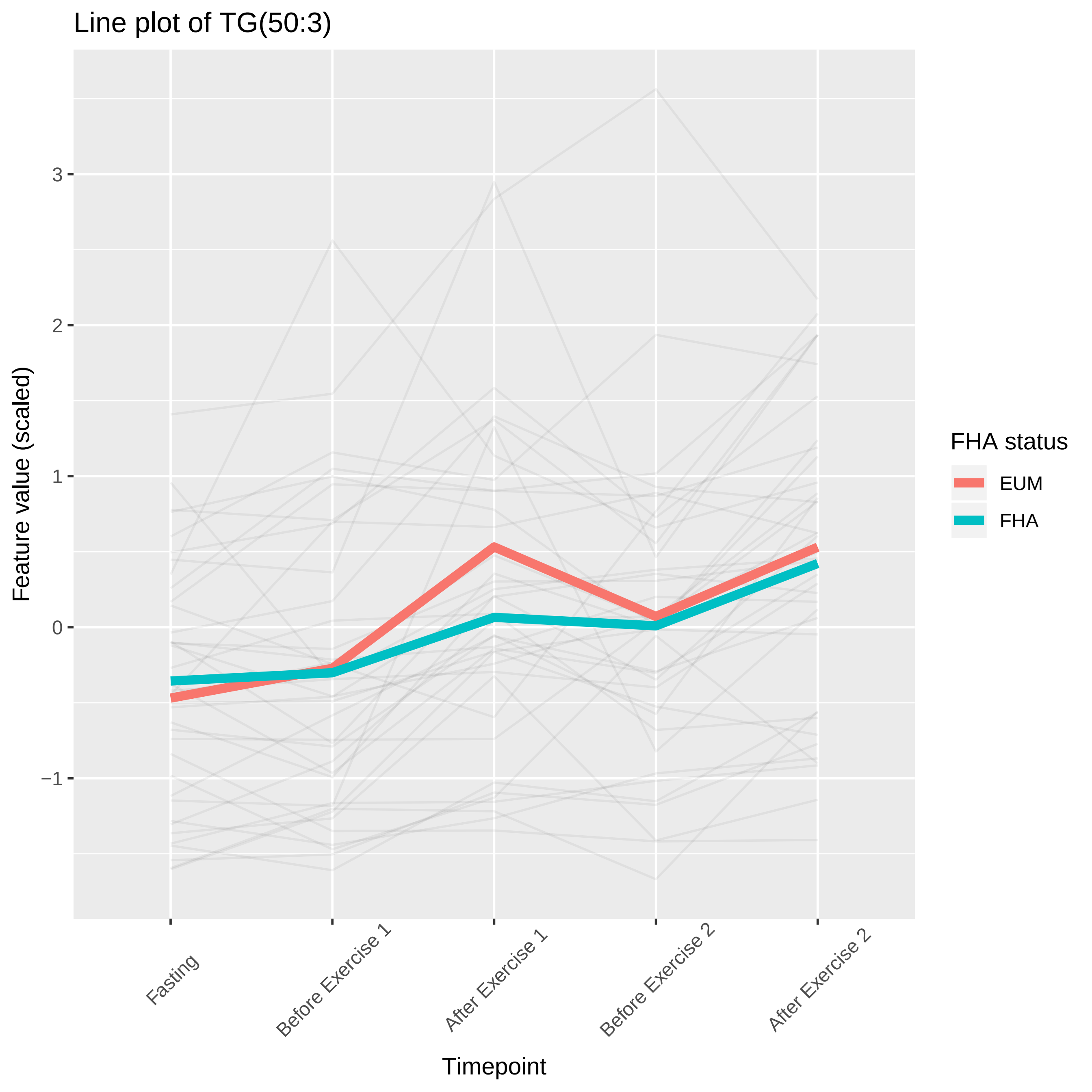
Line plot of TG(50:3)
Feature value (scaled)
3
2
1
0
−1
Fasting
Before Exercise 1
After Exercise 1
Before Exercise 2
After Exercise 2
Timepoint
FHA status
EUM
FHA

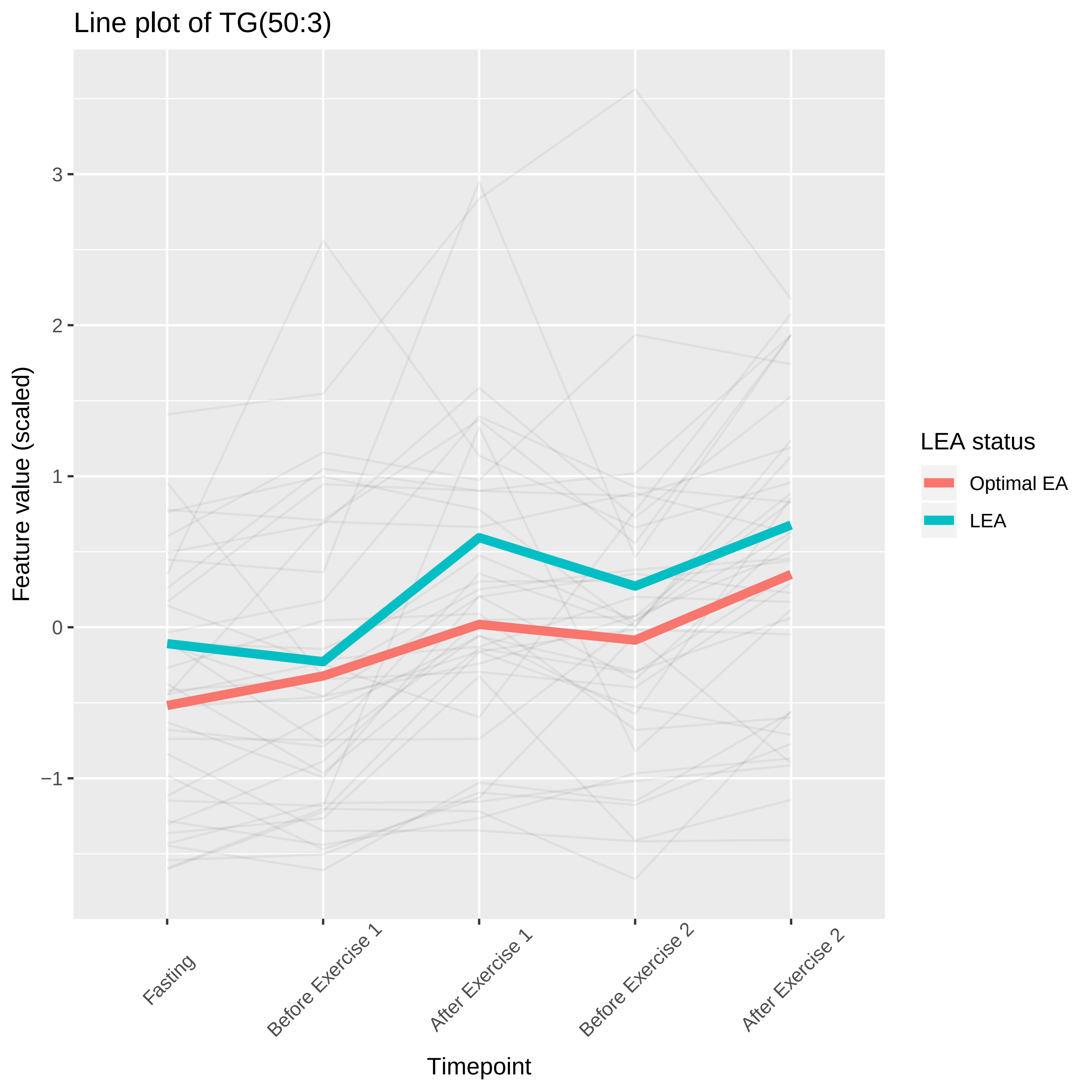

Line plot of TG(50:3)
Feature value (scaled)
3
2
1
0
−1
Fasting
Before Exercise 1
After Exercise 1
Before Exercise 2
After Exercise 2
Timepoint
LEA status
Optimal EA
LEA

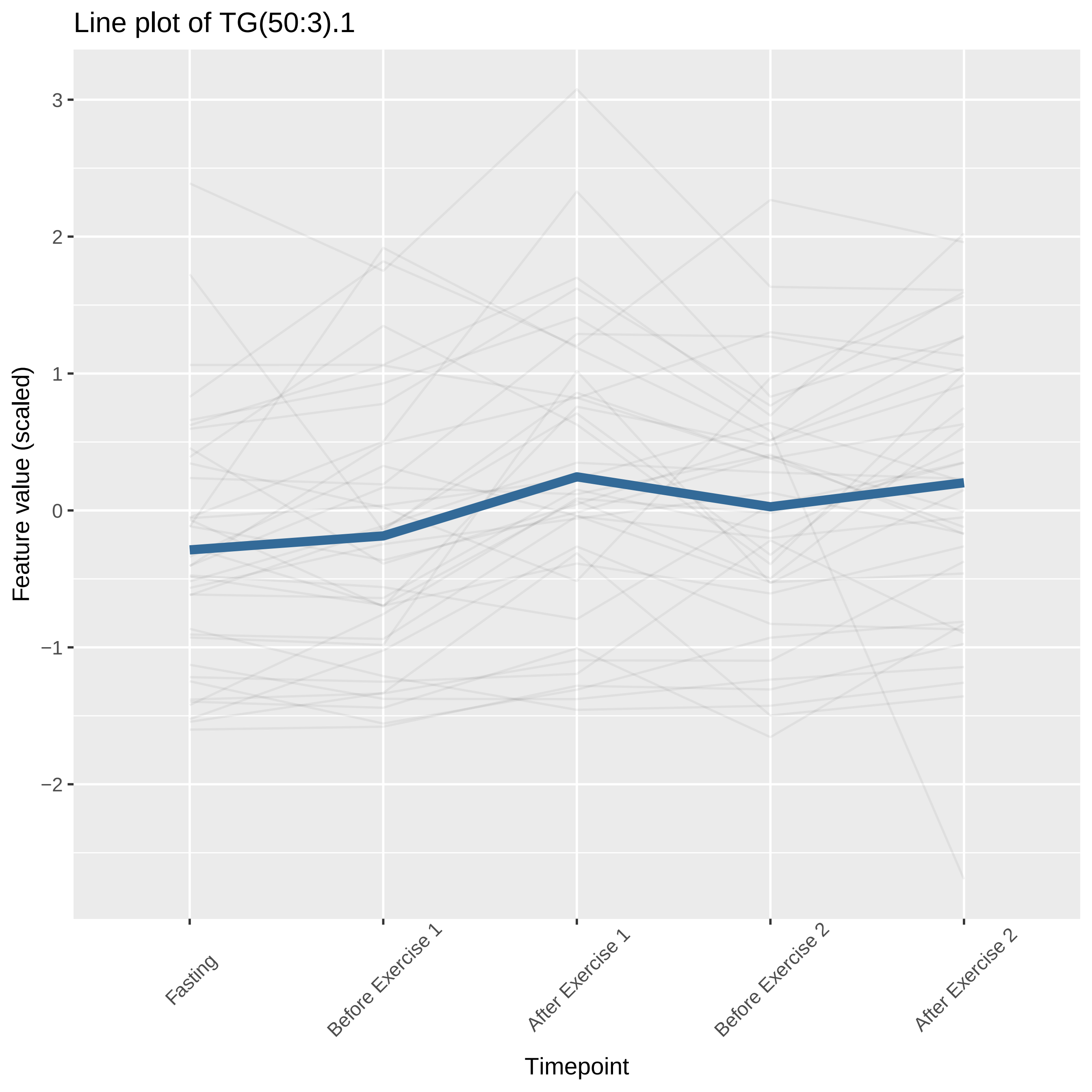

Line plot of TG(50:3).1
Feature value (scaled)
3
2
1
0
−1
−2
Fasting
Before Exercise 1
After Exercise 1
Before Exercise 2
After Exercise 2
Timepoint

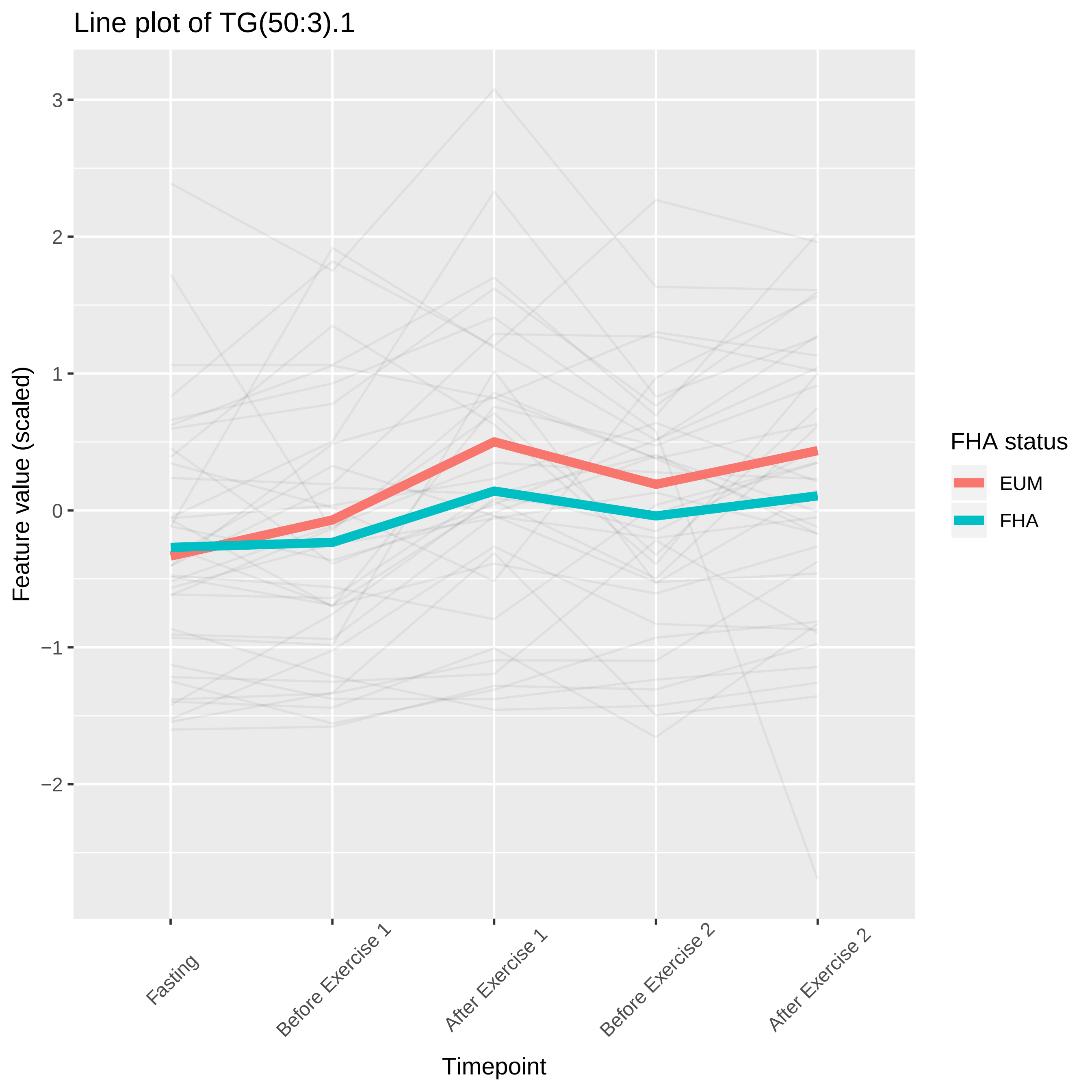

Line plot of TG(50:3).1
3
2
1
0
−1
−2
Feature value (scaled)
Fasting
Before Exercise 1
After Exercise 1
Before Exercise 2
After Exercise 2
Timepoint
FHA status
EUM
FHA

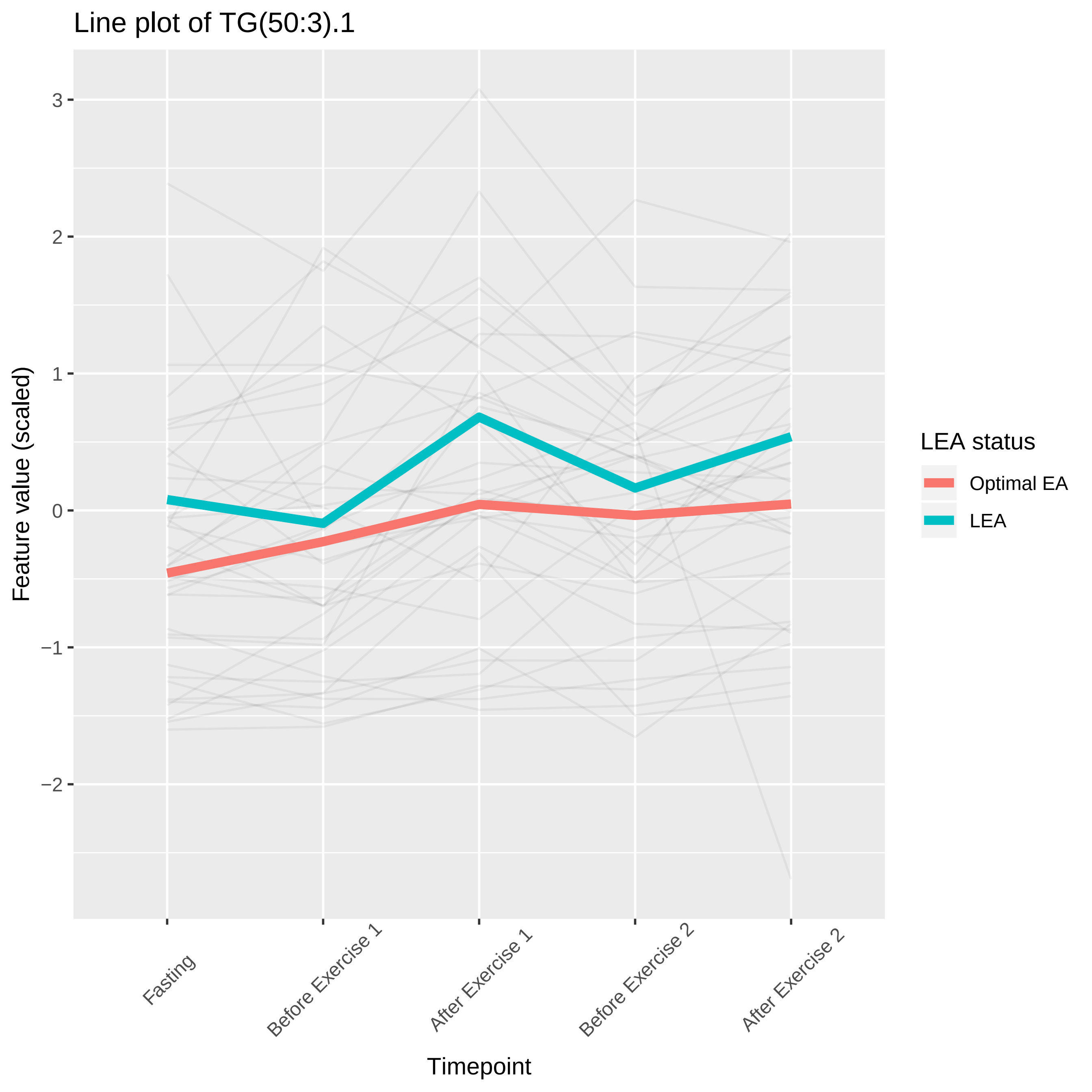

Line plot of TG(50:3).1
Feature value (scaled)
3
2
1
0
−1
−2
Fasting
Before Exercise 1
After Exercise 1
Before Exercise 2
After Exercise 2
Timepoint
LEA status
Optimal EA
LEA

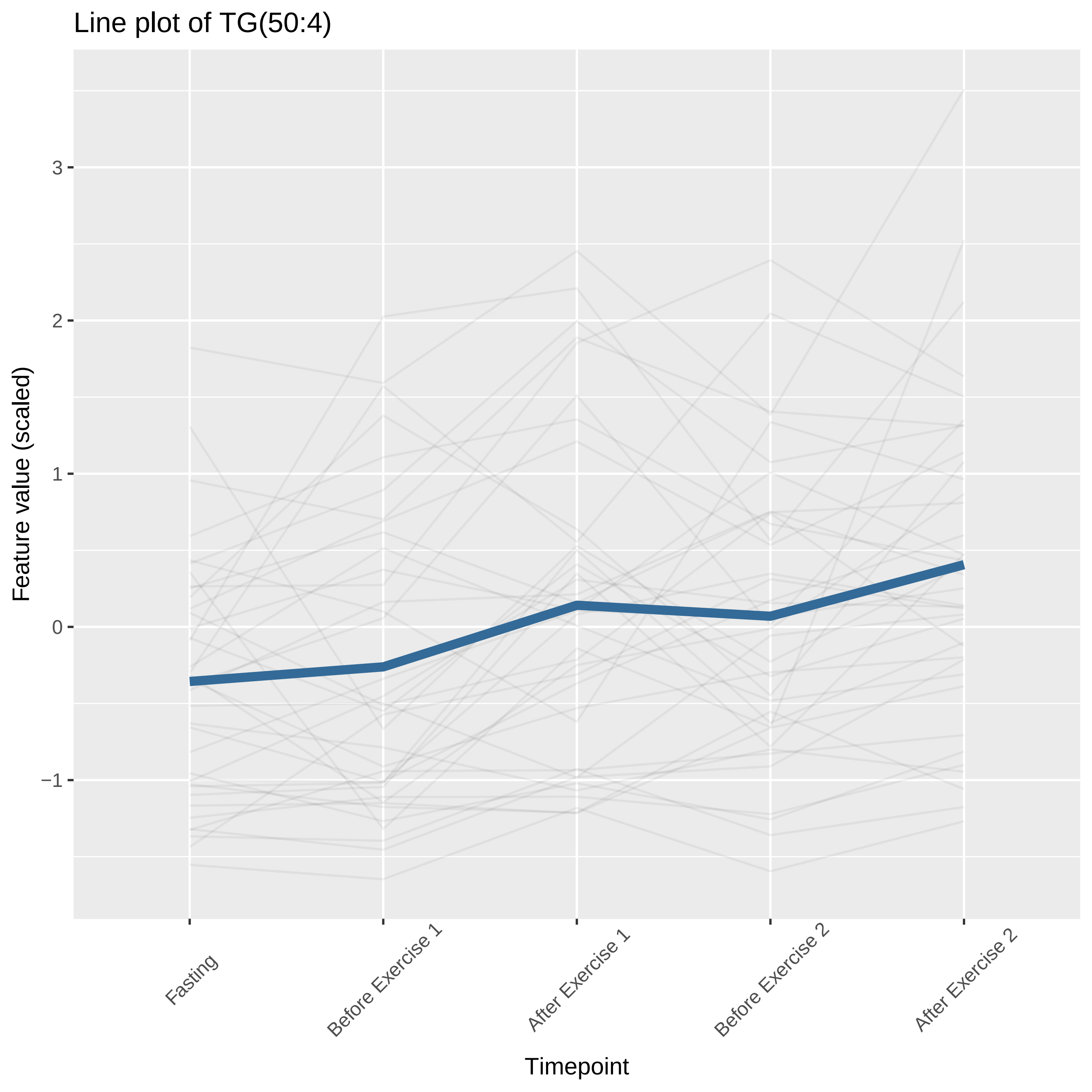

Line plot of TG(50:4)
Feature value (scaled)
3
2
1
0
−1
Fasting
Before Exercise 1
After Exercise 1
Before Exercise 2
After Exercise 2
Timepoint

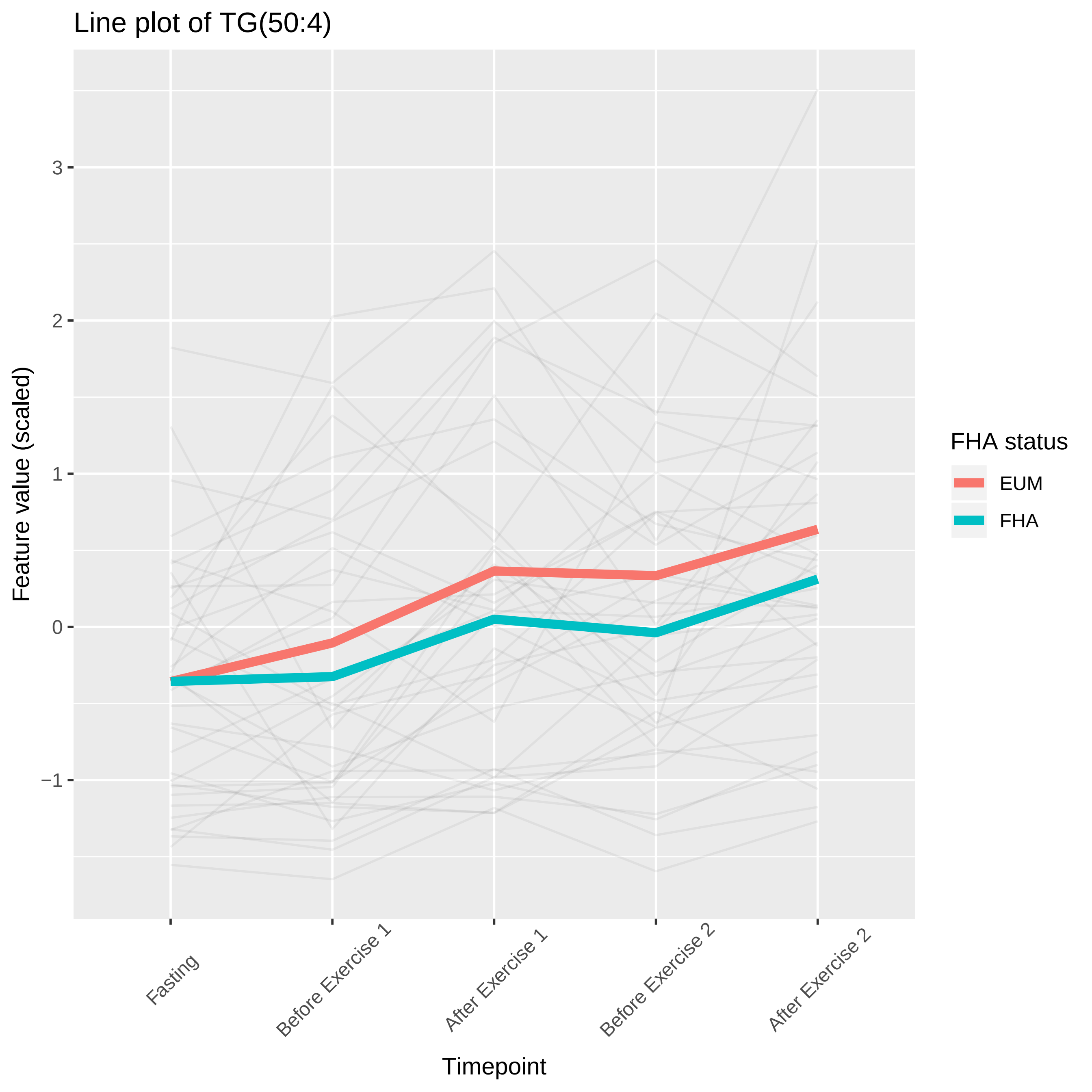

Line plot of TG(50:4)
Feature value (scaled)
3
2
1
0
−1
Fasting
Before Exercise 1
After Exercise 1
Before Exercise 2
After Exercise 2
Timepoint
FHA status
EUM
FHA

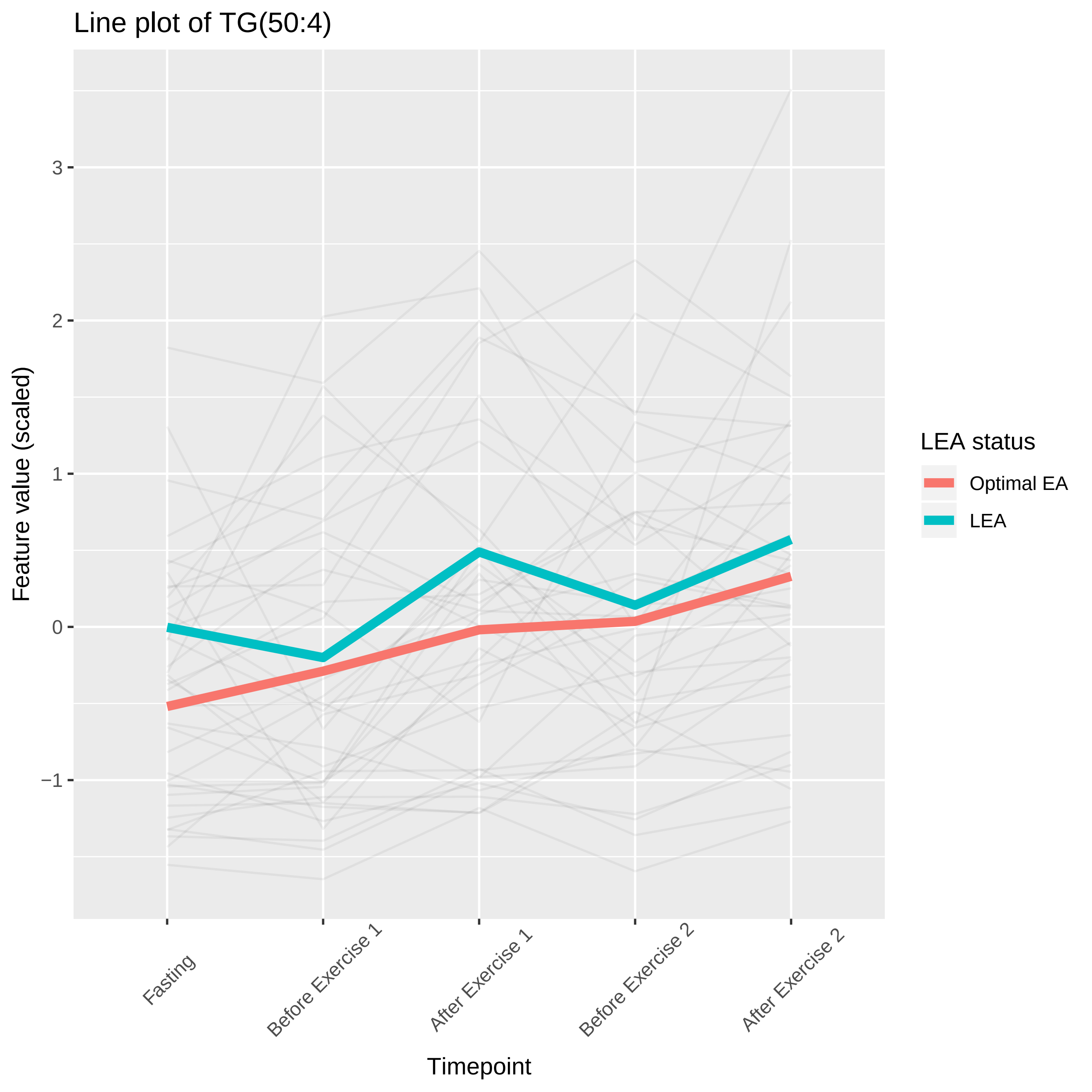
Line plot of TG(50:4)
Feature value (scaled)
3
2
1
0
−1
Fasting
Before Exercise 1
After Exercise 1
Before Exercise 2
After Exercise 2
Timepoint
LEA status
Optimal EA
LEA

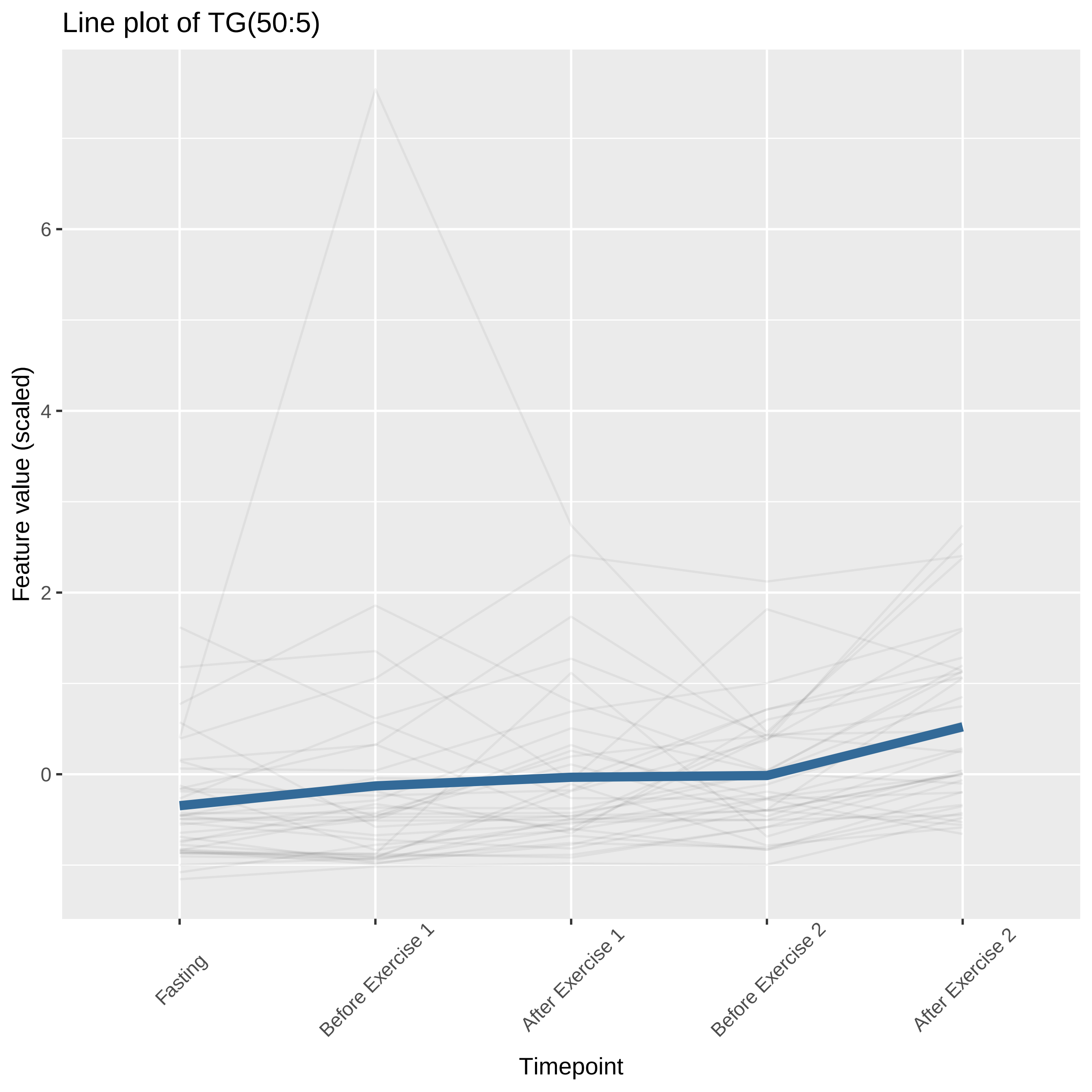

Line plot of TG(50:5)
Feature value (scaled)
6
4
2
0
Fasting
Before Exercise 1
After Exercise 1
Before Exercise 2
After Exercise 2
Timepoint

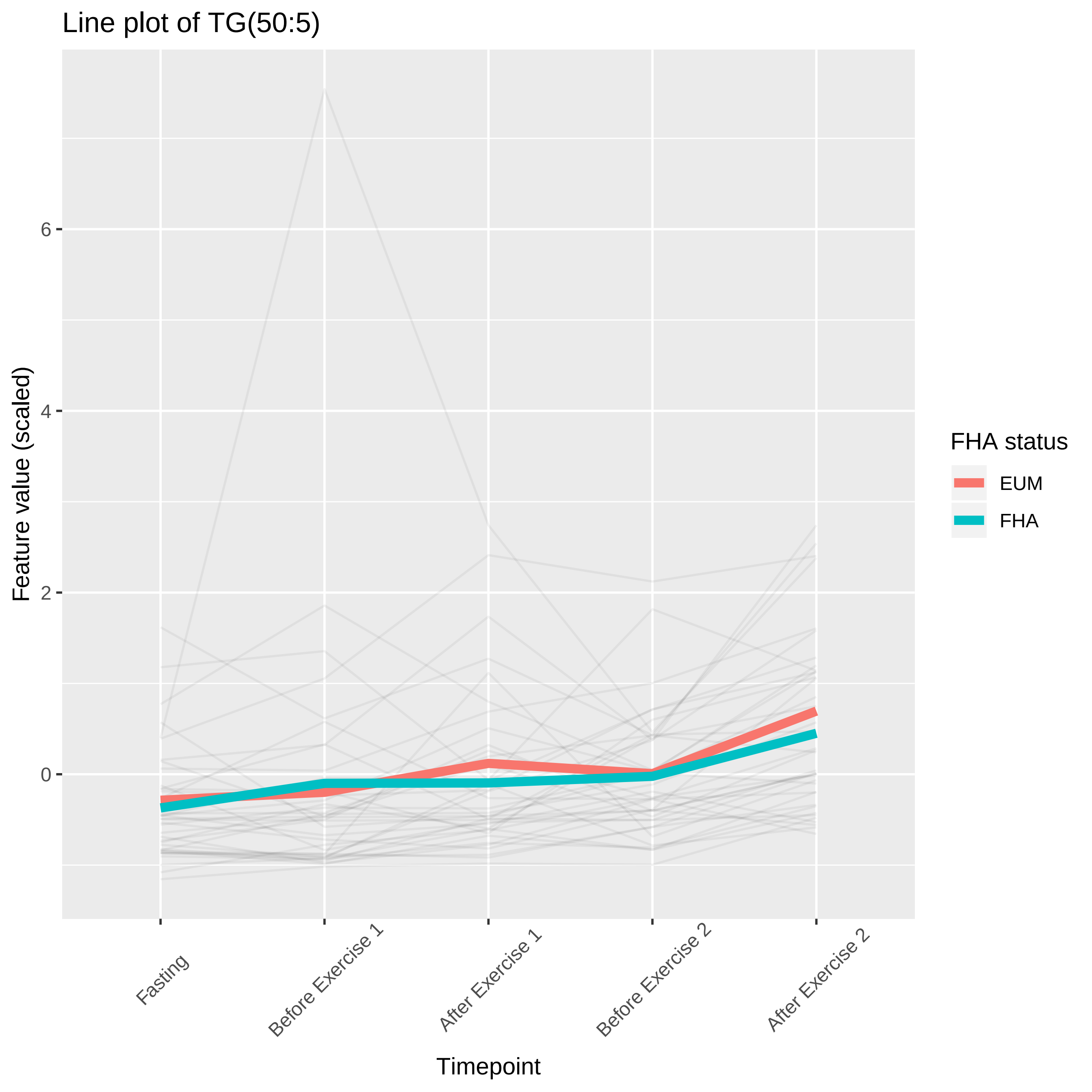

Line plot of TG(50:5)
Feature value (scaled)
6
4
2
0
Fasting
Before Exercise 1
After Exercise 1
Before Exercise 2
After Exercise 2
Timepoint
FHA status
EUM
FHA

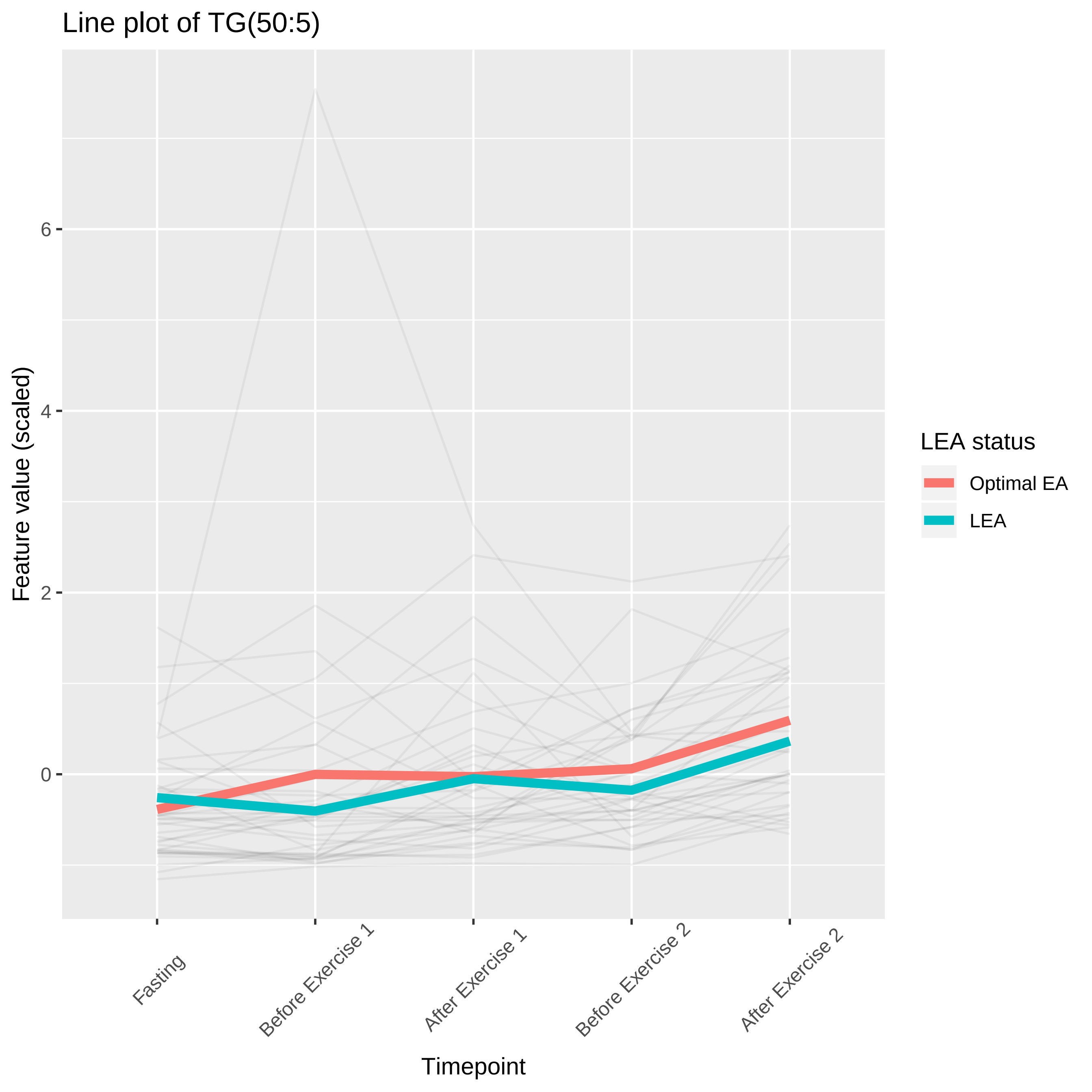

Line plot of TG(50:5)
Feature value (scaled)
6
4
2
0
Fasting
Before Exercise 1
After Exercise 1
Before Exercise 2
After Exercise 2
Timepoint
LEA status
Optimal EA
LEA

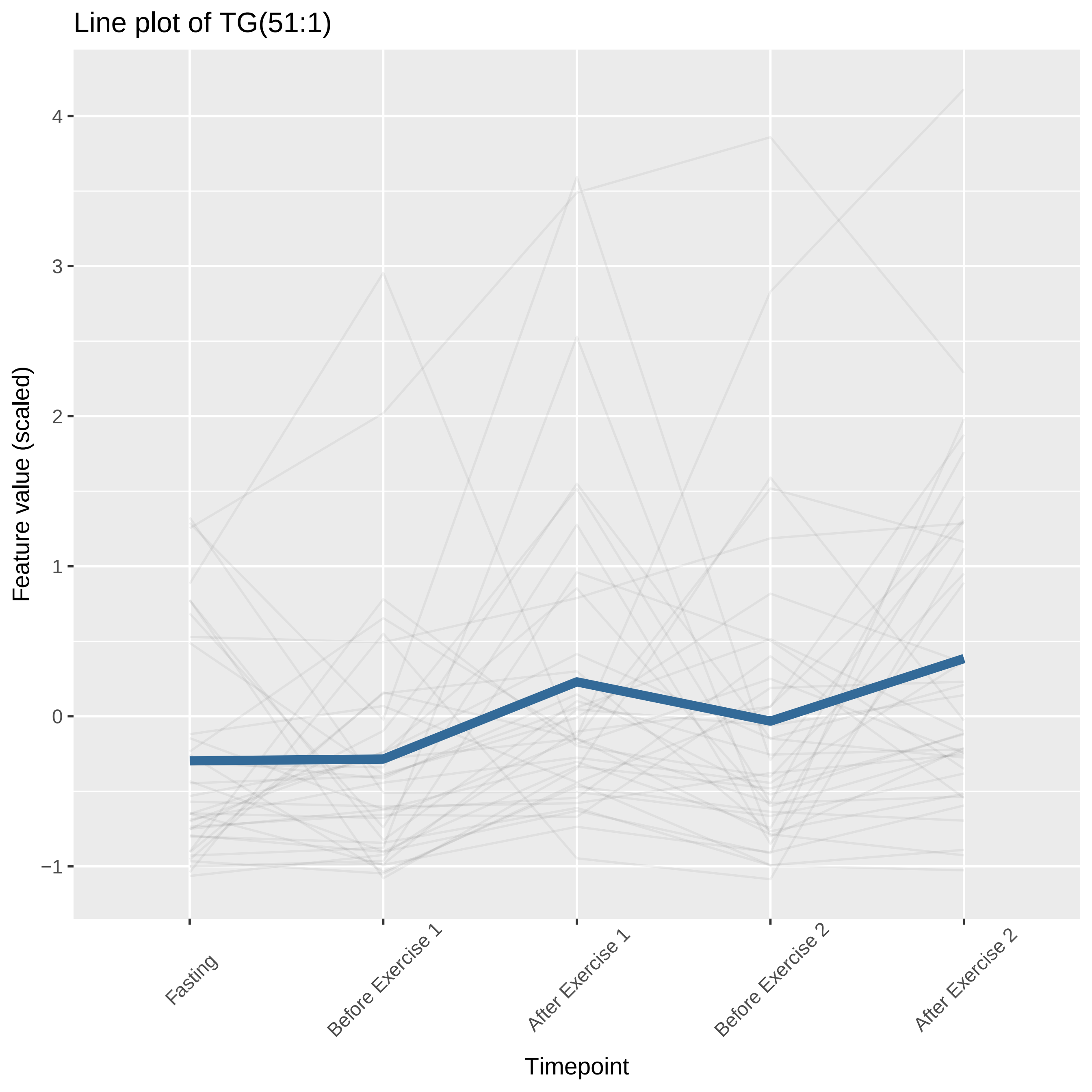
Line plot of TG(51:1)
Feature value (scaled)
4
3
2
1
0
−1
Fasting
Before Exercise 1
After Exercise 1
Before Exercise 2
After Exercise 2
Timepoint

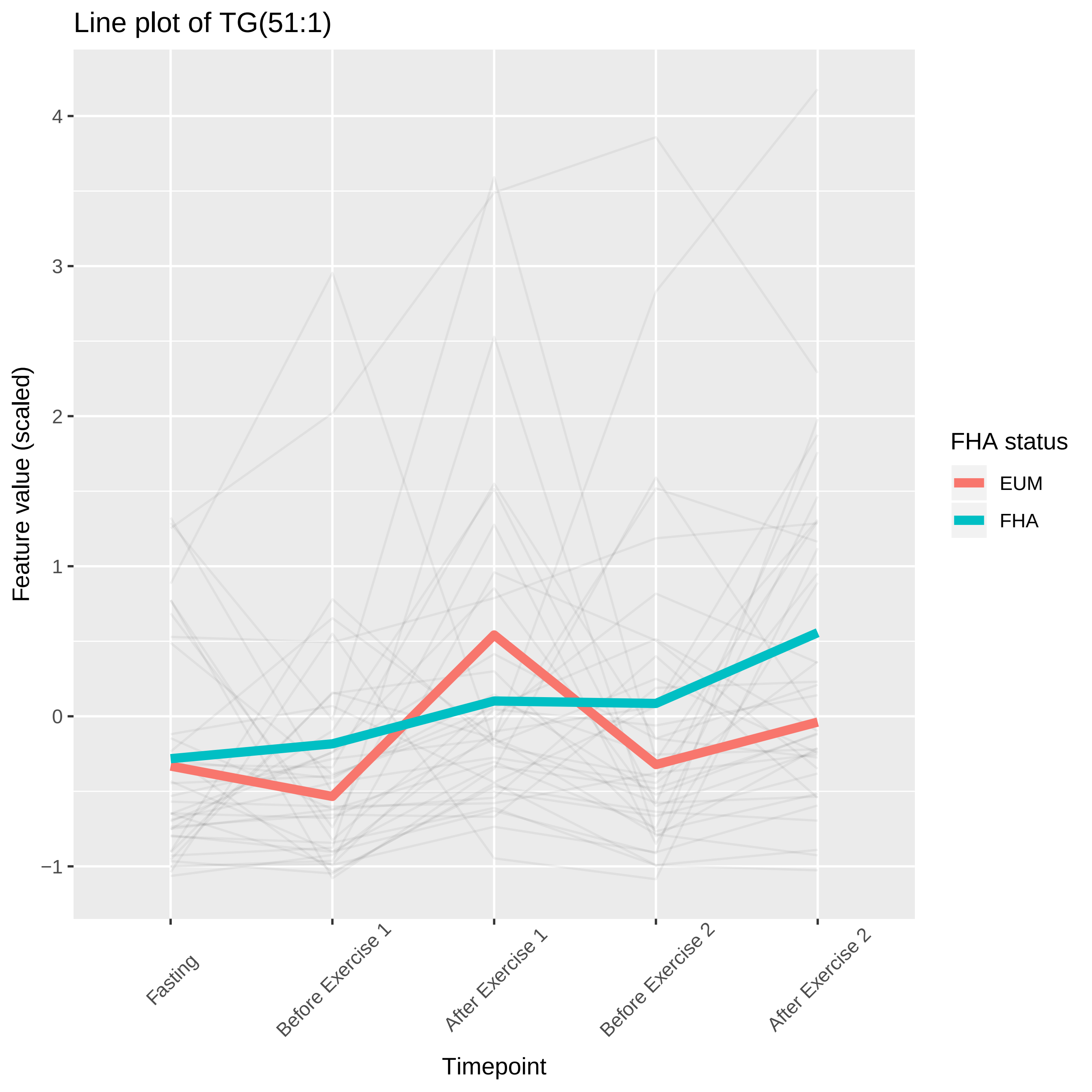

Line plot of TG(51:1)
Feature value (scaled)
4
3
2
1
0
−1
Fasting
Before Exercise 1
After Exercise 1
Before Exercise 2
After Exercise 2
Timepoint
FHA status
EUM
FHA

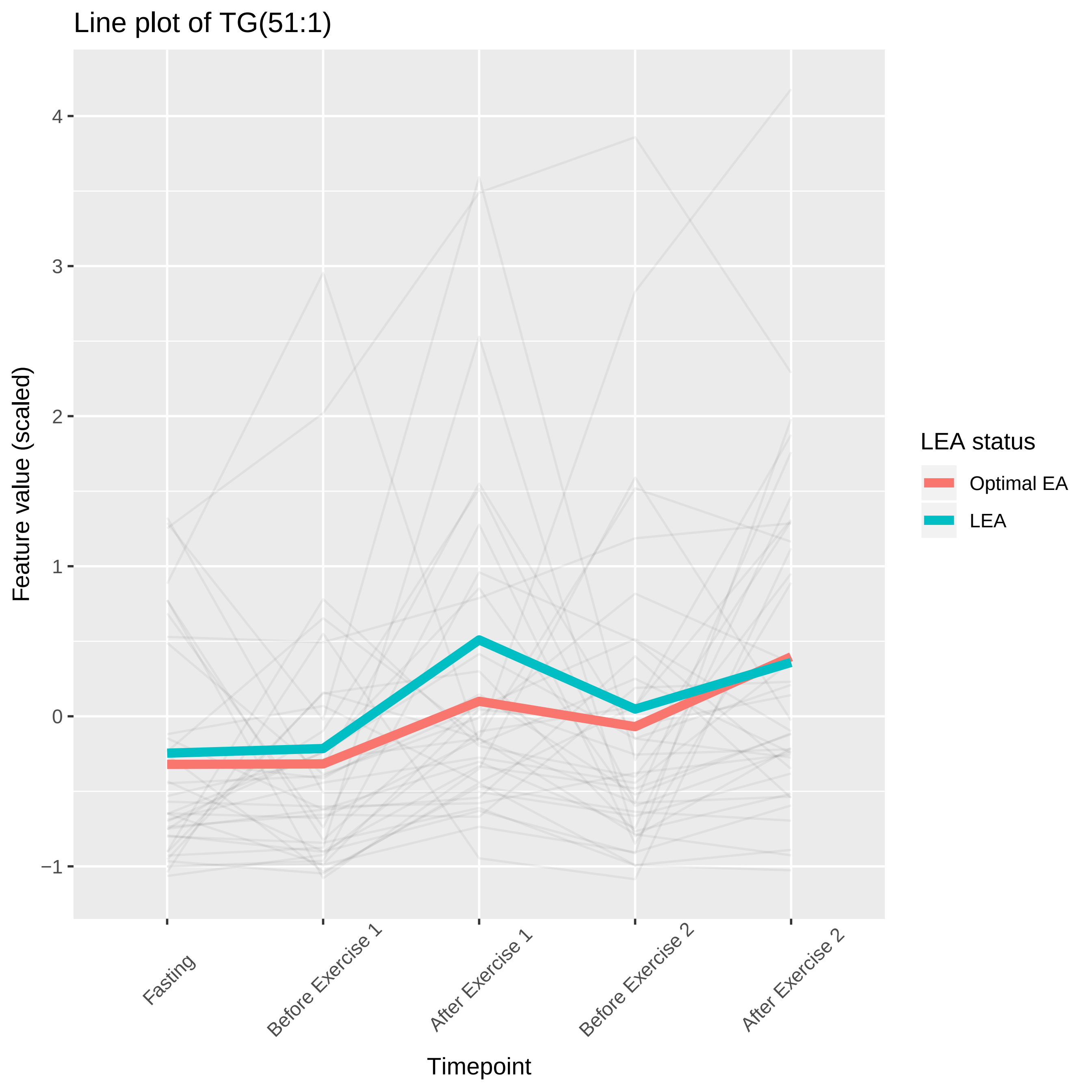

Line plot of TG(51:1)
Feature value (scaled)
4
3
2
1
0
−1
Fasting
Before Exercise 1
After Exercise 1
Before Exercise 2
After Exercise 2
Timepoint
LEA status
Optimal EA
LEA

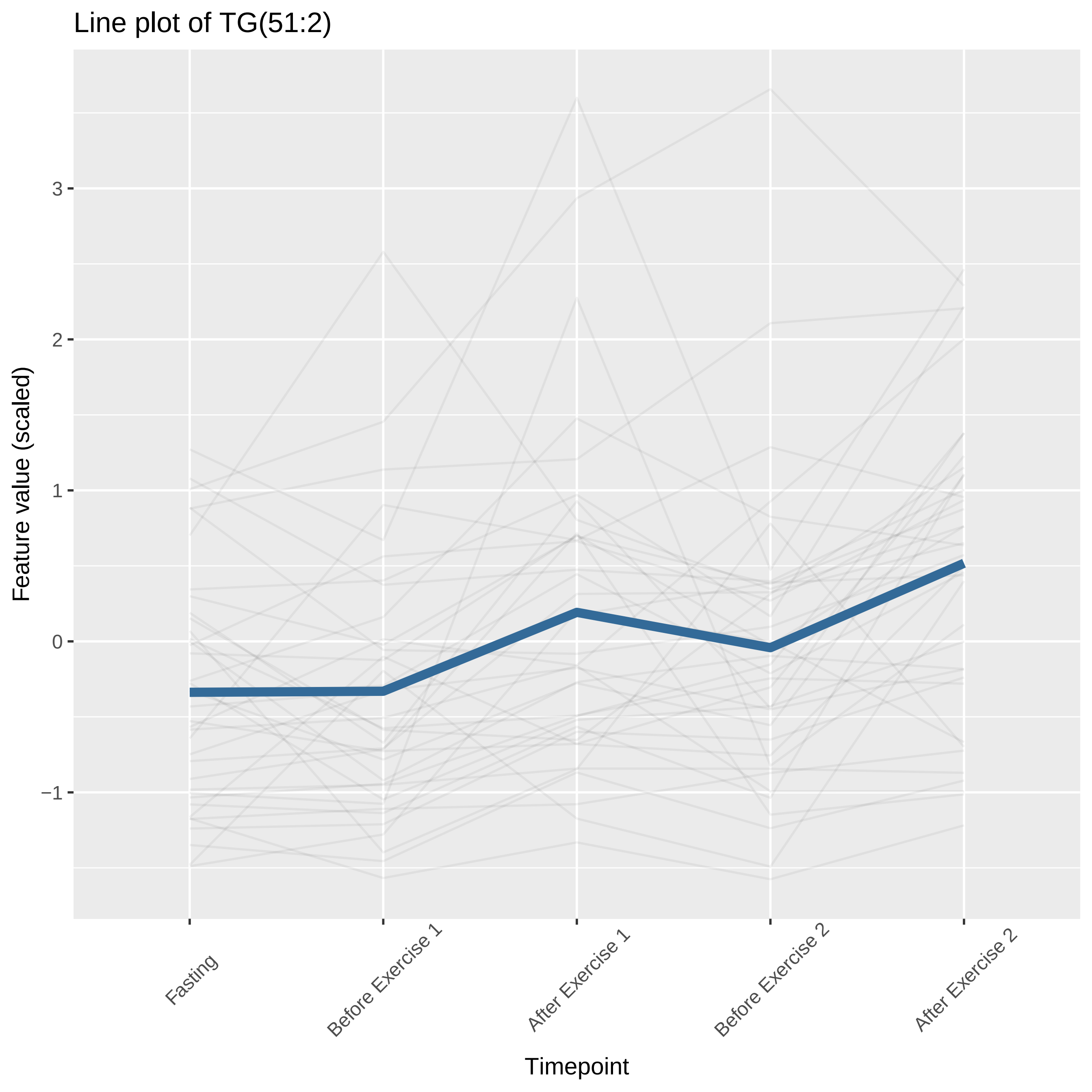

Line plot of TG(51:2)
Feature value (scaled)
3
2
1
0
−1
Fasting
Before Exercise 1
After Exercise 1
Before Exercise 2
After Exercise 2
Timepoint

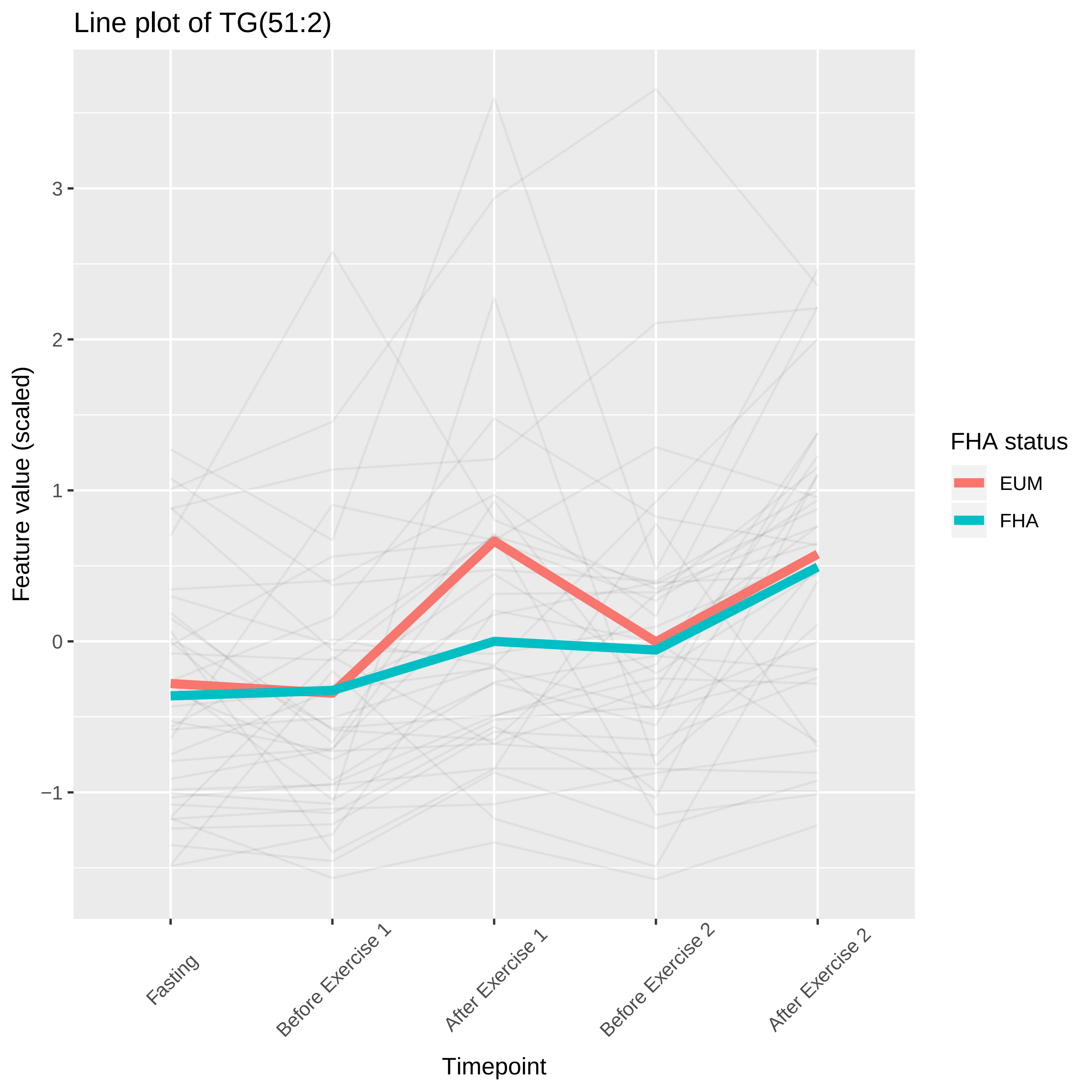

Line plot of TG(51:2)
Feature value (scaled)
3
2
1
0
−1
Fasting
Before Exercise 1
After Exercise 1
Before Exercise 2
After Exercise 2
Timepoint
FHA status
EUM
FHA

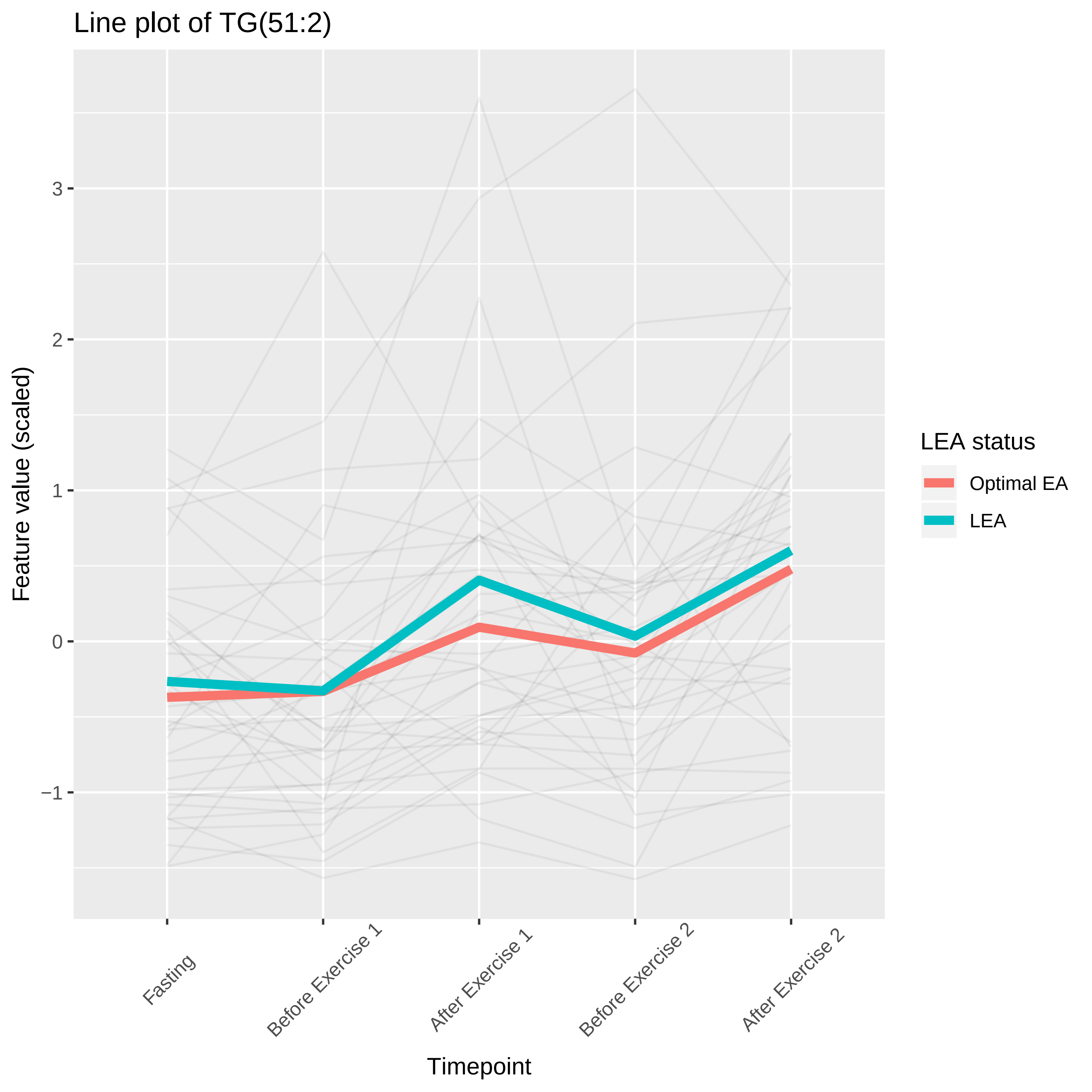

Line plot of TG(51:2)
Feature value (scaled)
3
2
1
0
−1
Fasting
Before Exercise 1
After Exercise 1
Before Exercise 2
After Exercise 2
Timepoint
LEA status
Optimal EA
LEA

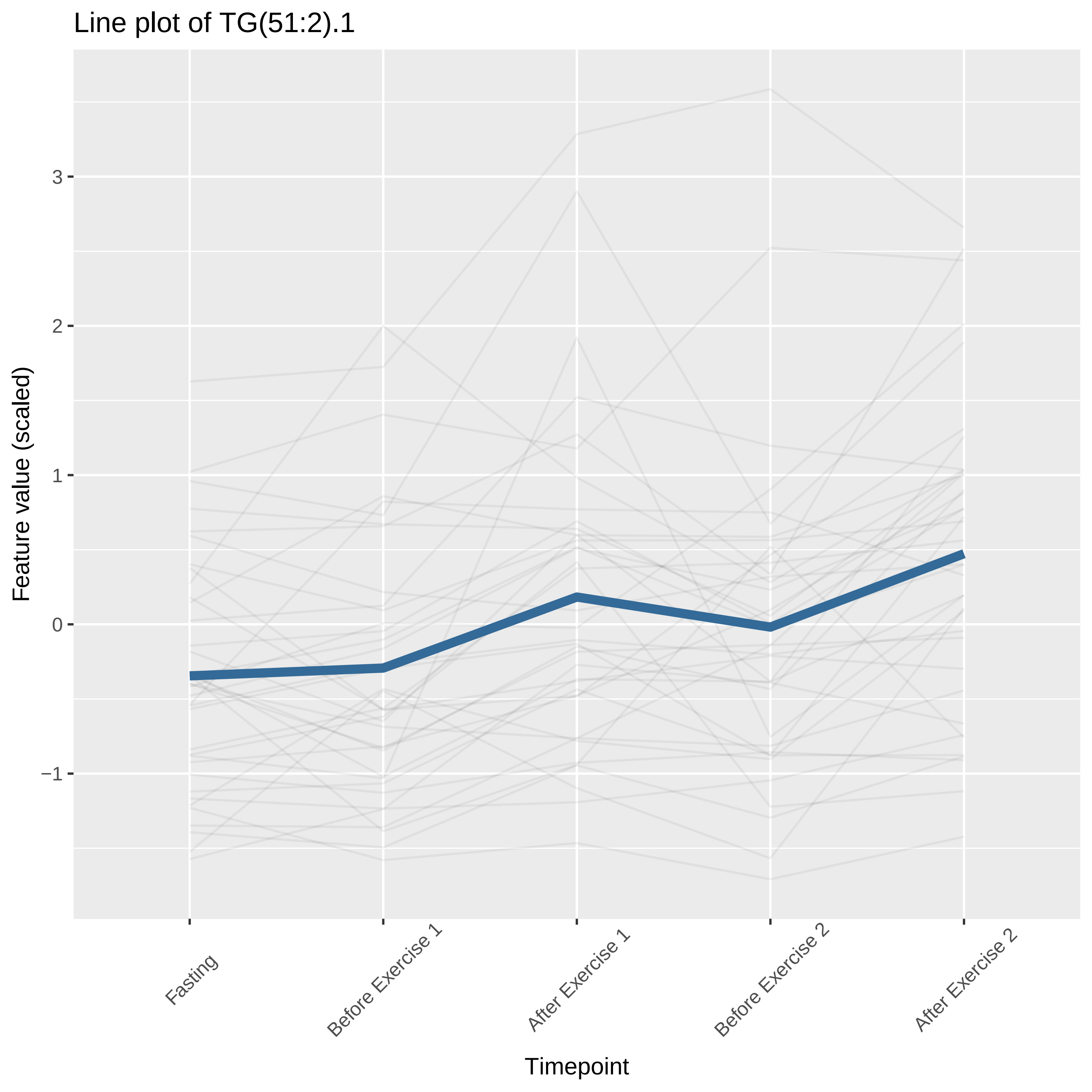

Line plot of TG(51:2).1
Feature value (scaled)
3
2
1
0
−1
Fasting
Before Exercise 1
After Exercise 1
Before Exercise 2
After Exercise 2
Timepoint

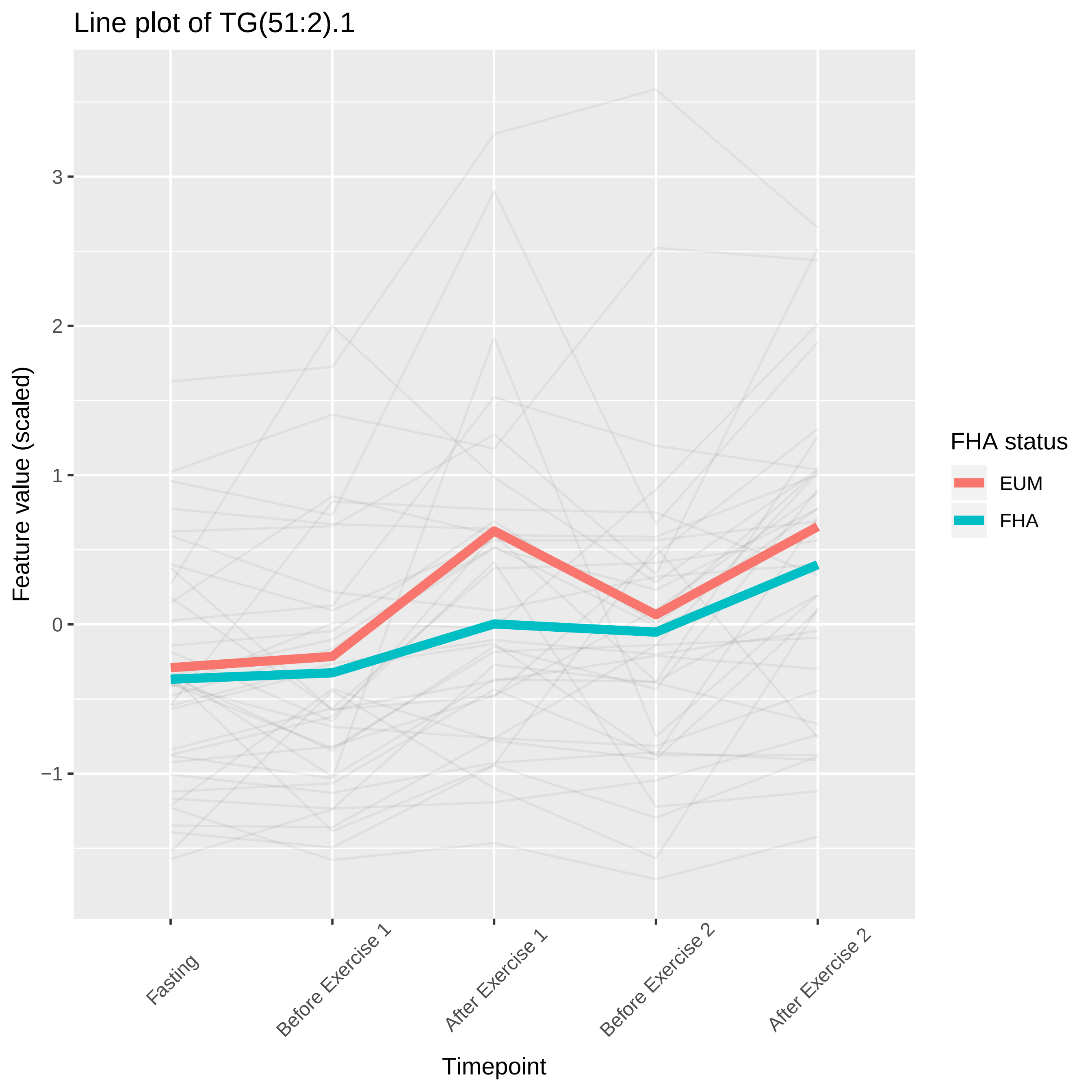

Line plot of TG(51:2).1
Feature value (scaled)
3
2
1
0
−1
Fasting
Before Exercise 1
After Exercise 1
Before Exercise 2
After Exercise 2
Timepoint
FHA status
EUM
FHA

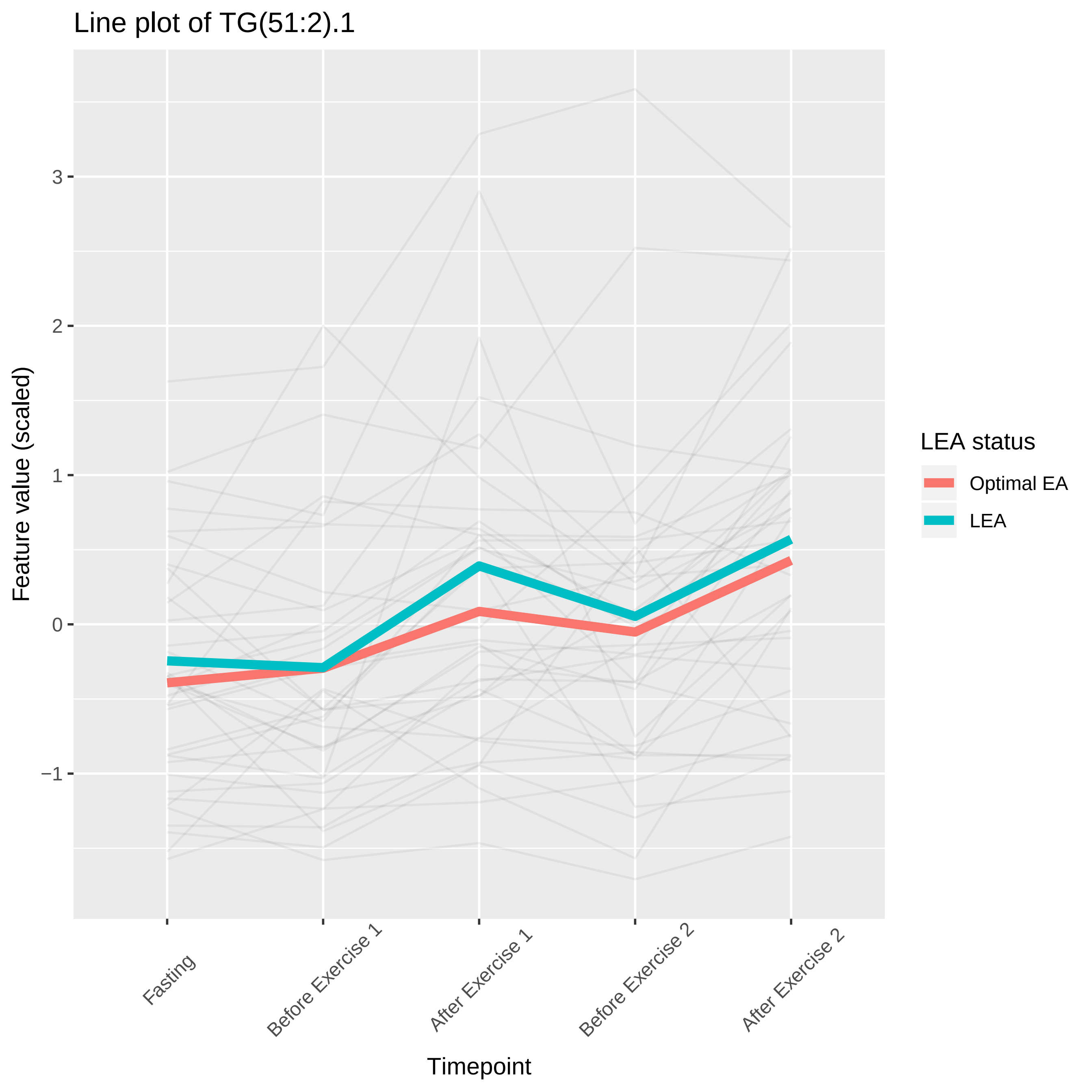
Line plot of TG(51:2).1
Feature value (scaled)
3
2
1
0
−1
Fasting
Before Exercise 1
After Exercise 1
Before Exercise 2
After Exercise 2
Timepoint
LEA status
Optimal EA
LEA

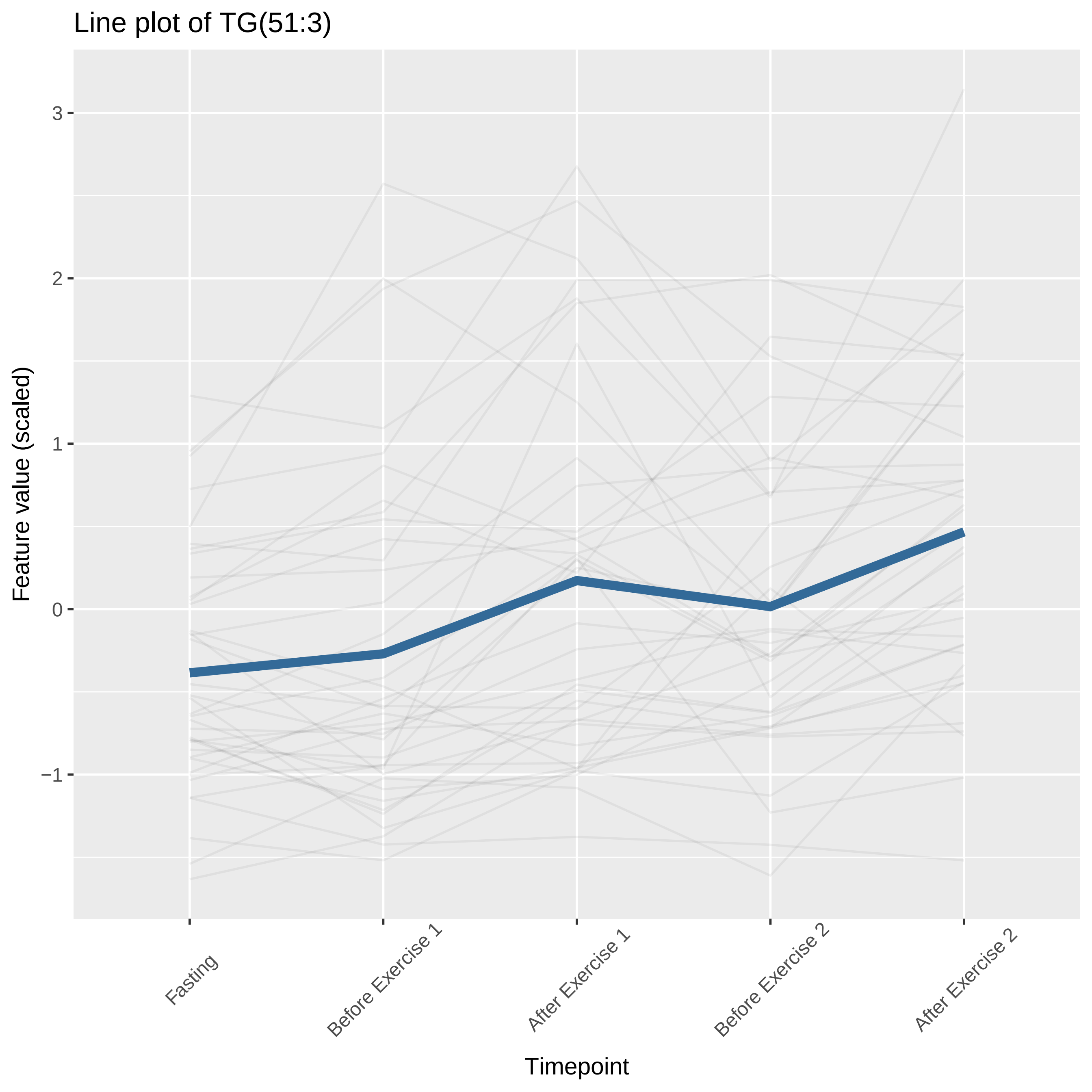

Line plot of TG(51:3)
Feature value (scaled)
3
2
1
0
−1
Fasting
Before Exercise 1
After Exercise 1
Before Exercise 2
After Exercise 2
Timepoint

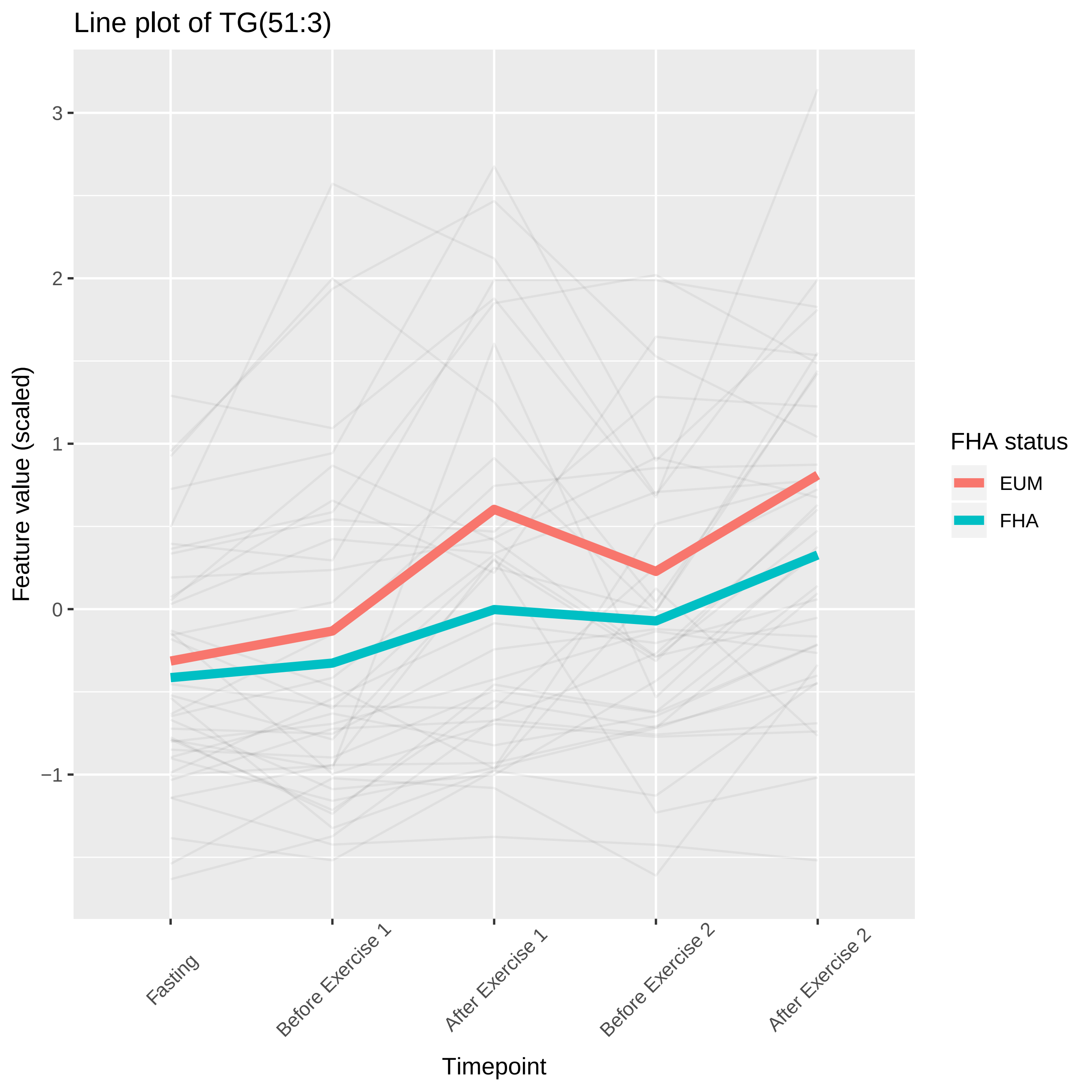

Line plot of TG(51:3)
Feature value (scaled)
3
2
1
0
−1
Fasting
Before Exercise 1
After Exercise 1
Before Exercise 2
After Exercise 2
Timepoint
FHA status
EUM
FHA

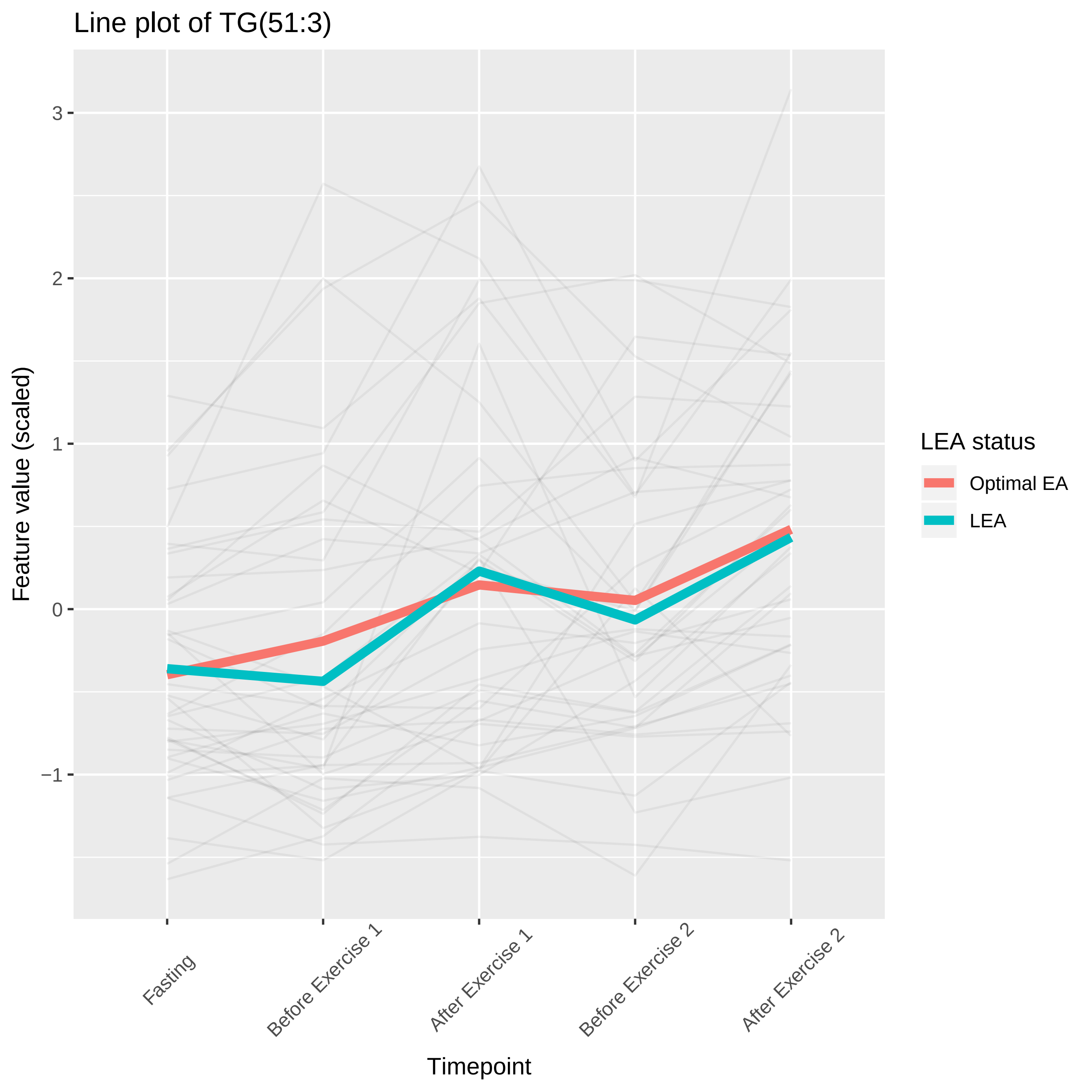

Line plot of TG(51:3)
Feature value (scaled)
3
2
1
0
−1
Fasting
Before Exercise 1
After Exercise 1
Before Exercise 2
After Exercise 2
Timepoint
LEA status
Optimal EA
LEA

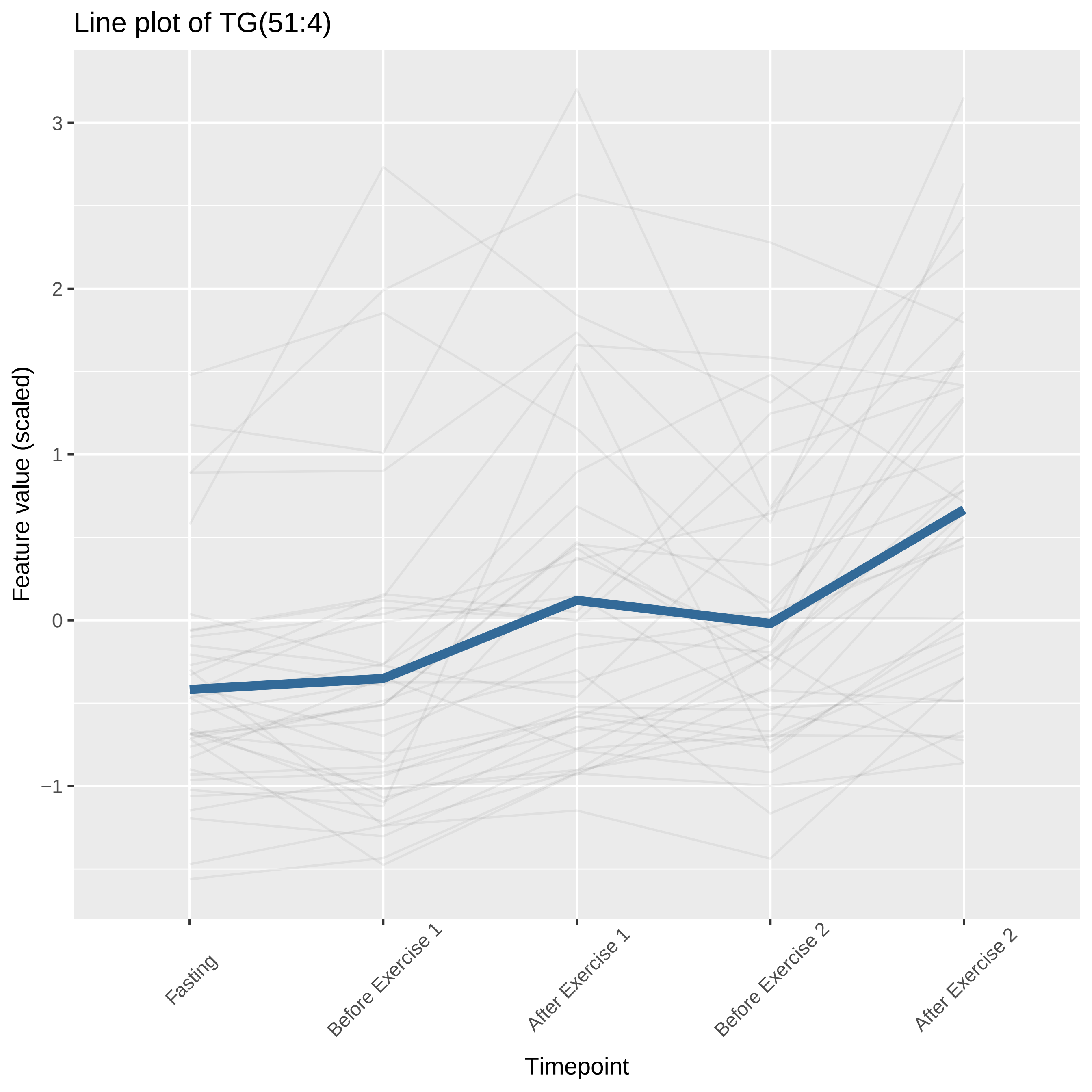

Line plot of TG(51:4)
Feature value (scaled)
3
2
1
0
−1
Fasting
Before Exercise 1
After Exercise 1
Before Exercise 2
After Exercise 2
Timepoint

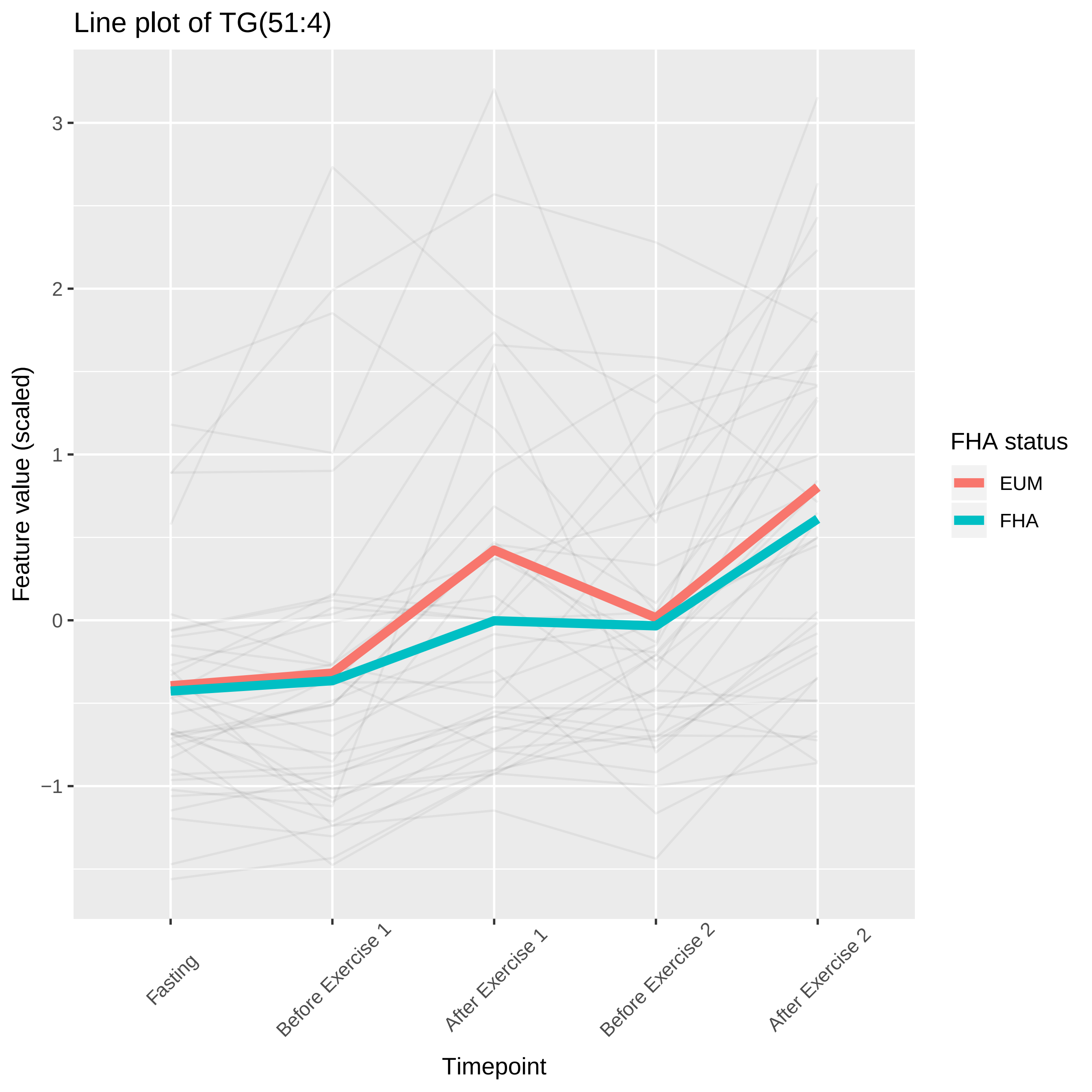

Line plot of TG(51:4)
Feature value (scaled)
3
2
1
0
−1
Fasting
Before Exercise 1
After Exercise 1
Before Exercise 2
After Exercise 2
Timepoint
FHA status
EUM
FHA

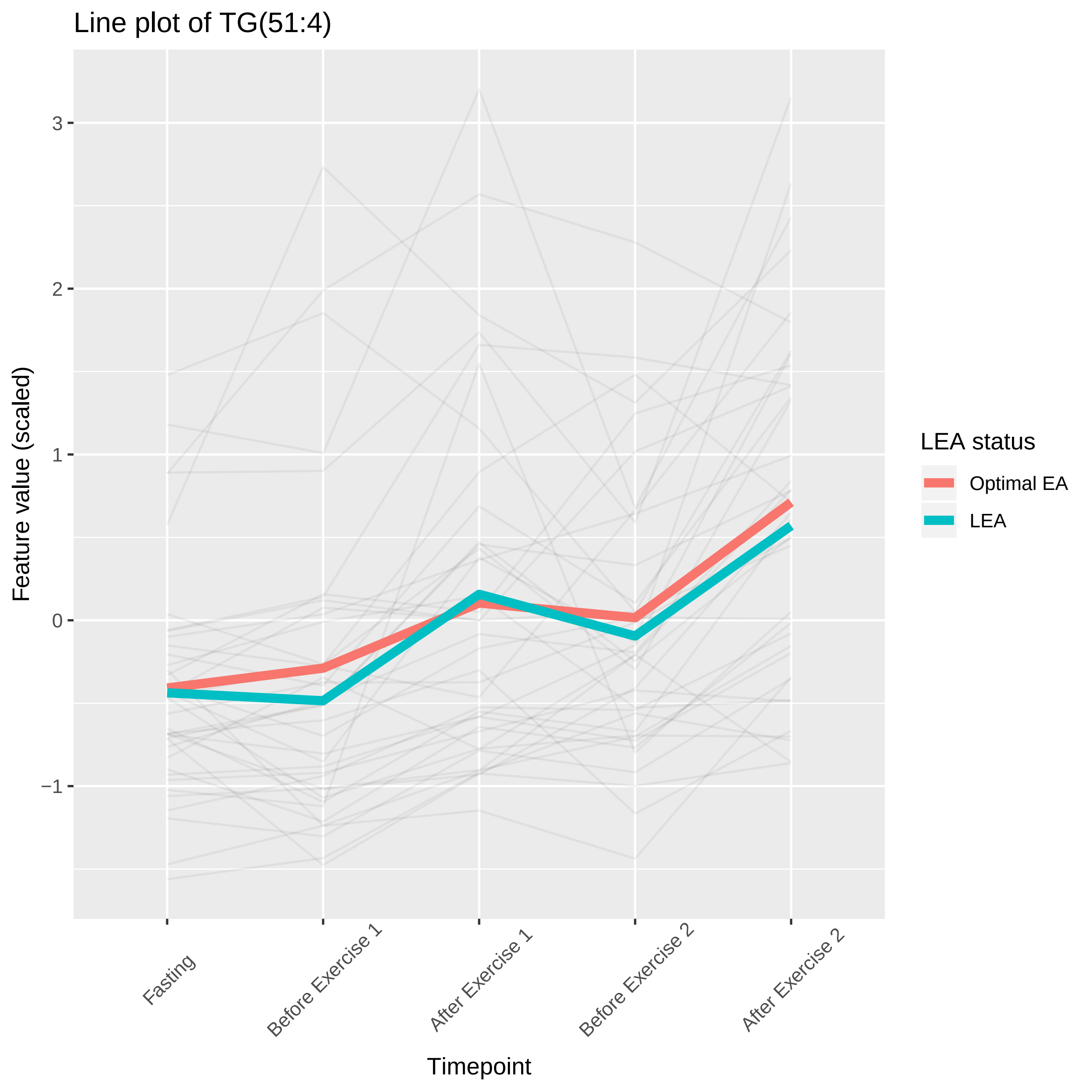

Line plot of TG(51:4)
Feature value (scaled)
3
2
1
0
−1
Fasting
Before Exercise 1
After Exercise 1
Before Exercise 2
After Exercise 2
Timepoint
LEA status
Optimal EA
LEA

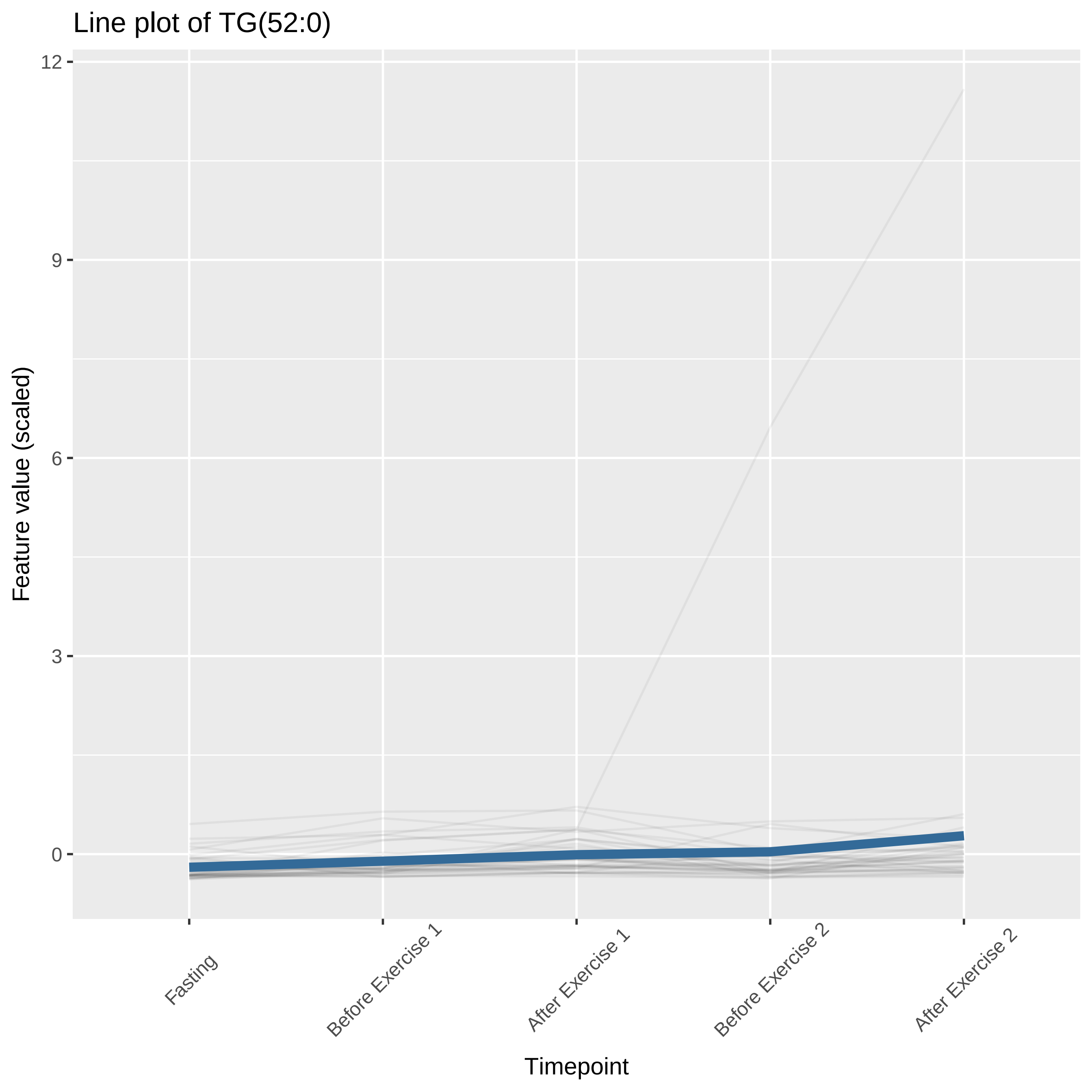
Line plot of TG(52:0)
Feature value (scaled)
12
9
6
3
0
Fasting
Before Exercise 1
After Exercise 1
Before Exercise 2
After Exercise 2
Timepoint

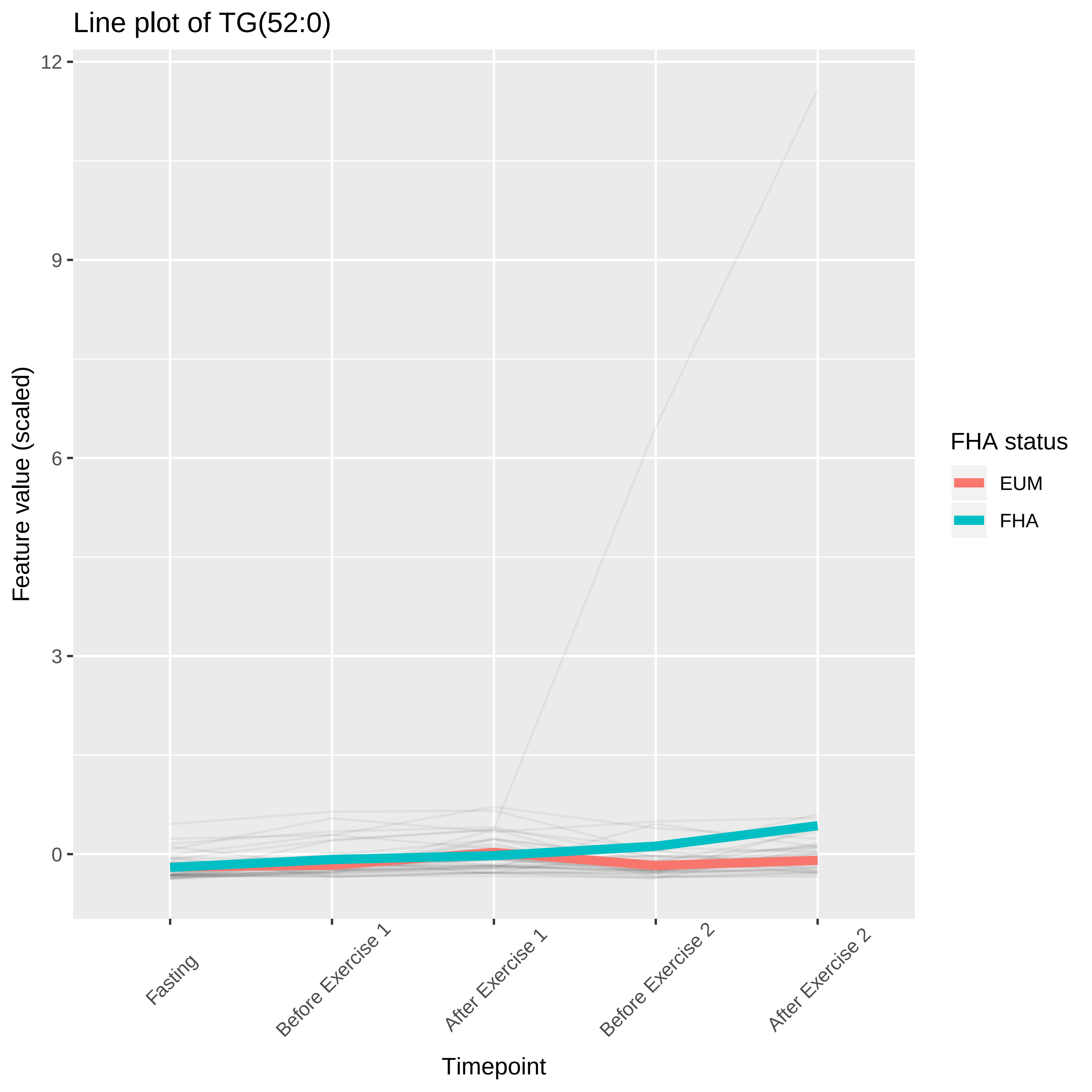
Line plot of TG(52:0)
12
9
6
3
0
Feature value (scaled)
Fasting
Before Exercise 1
After Exercise 1
Before Exercise 2
After Exercise 2
Timepoint
FHA status
EUM
FHA

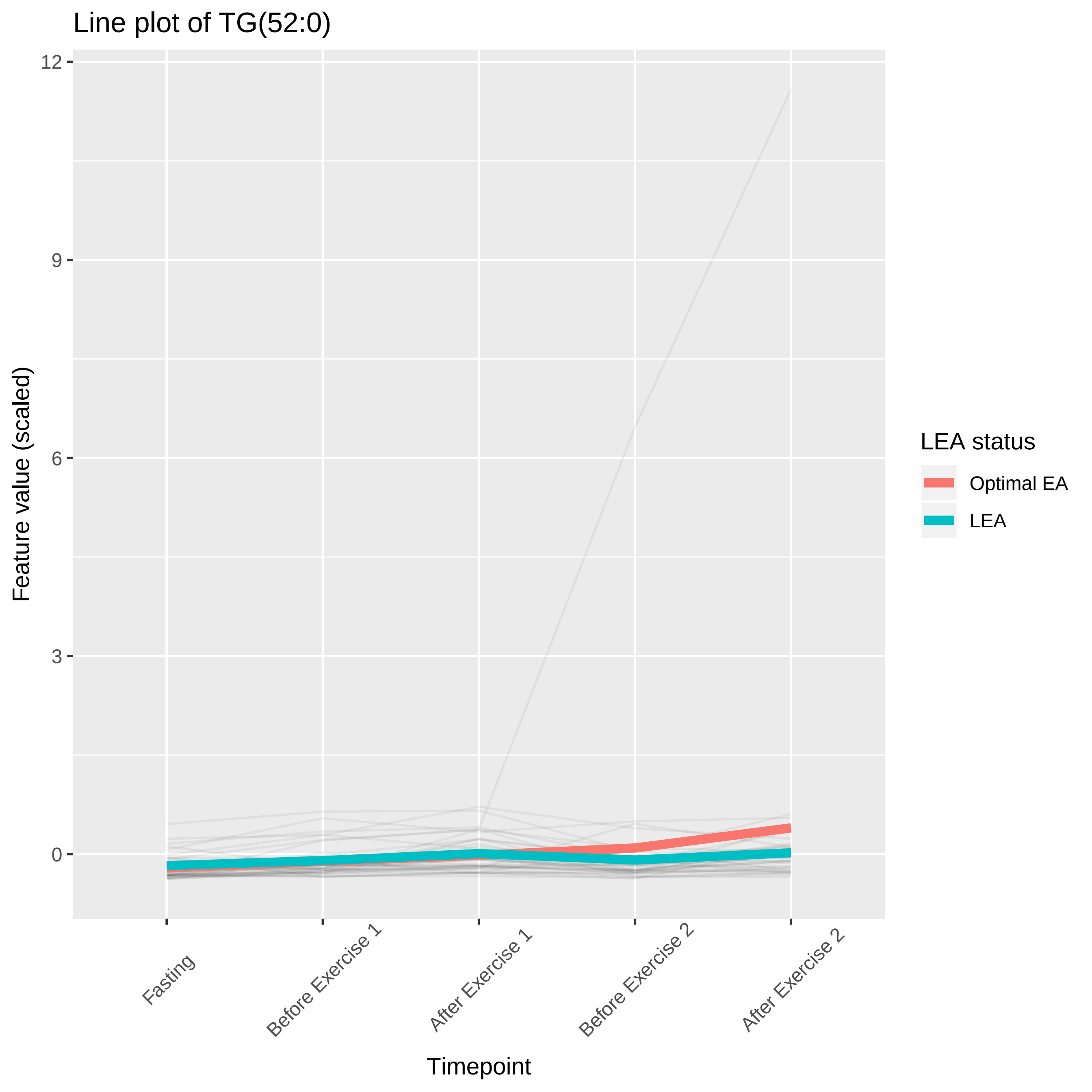

Line plot of TG(52:0)
Feature value (scaled)
12
9
6
3
0
Fasting
Before Exercise 1
After Exercise 1
Before Exercise 2
After Exercise 2
Timepoint
LEA status
Optimal EA
LEA

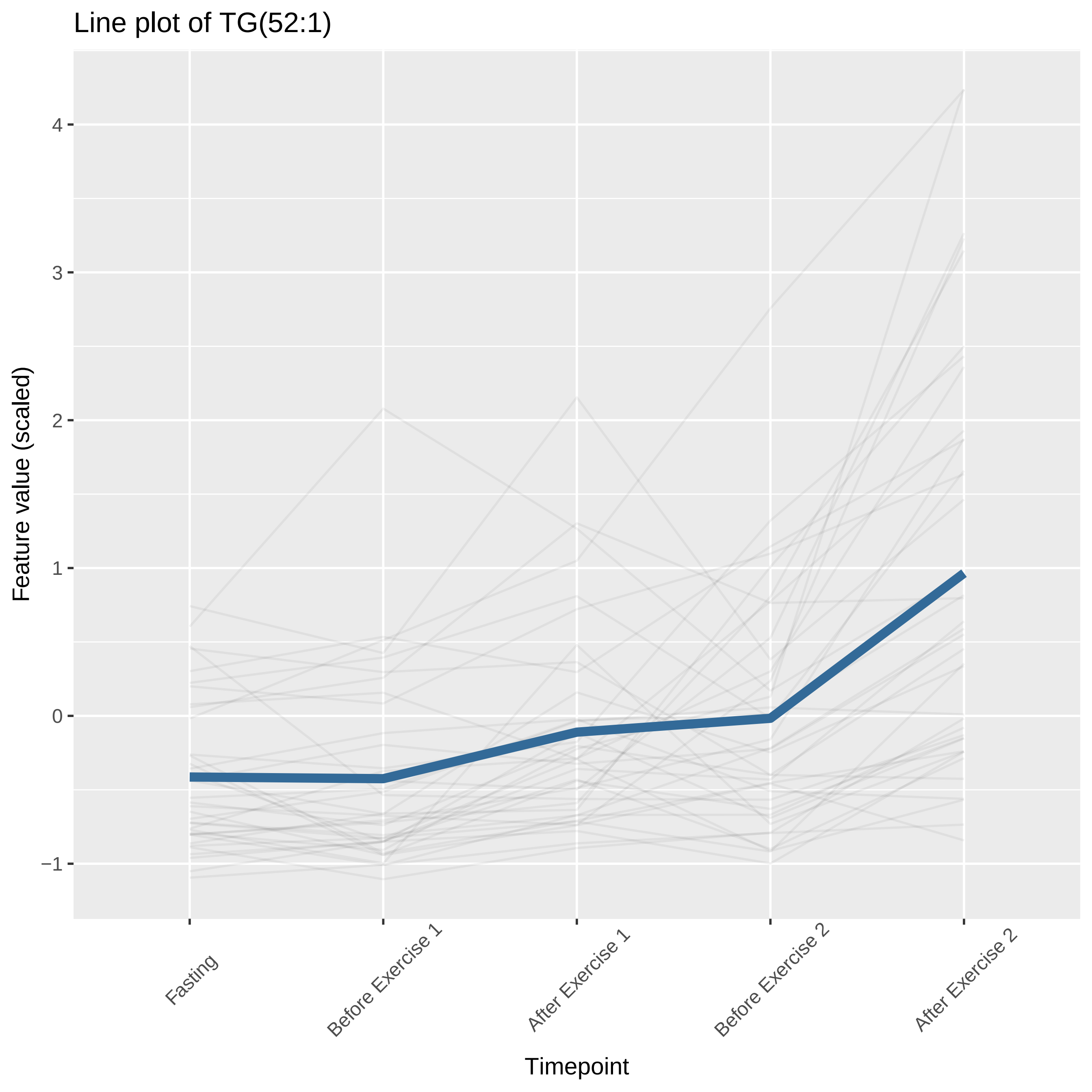

Line plot of TG(52:1)
Feature value (scaled)
4
3
2
1
0
−1
Fasting
Before Exercise 1
After Exercise 1
Before Exercise 2
After Exercise 2
Timepoint

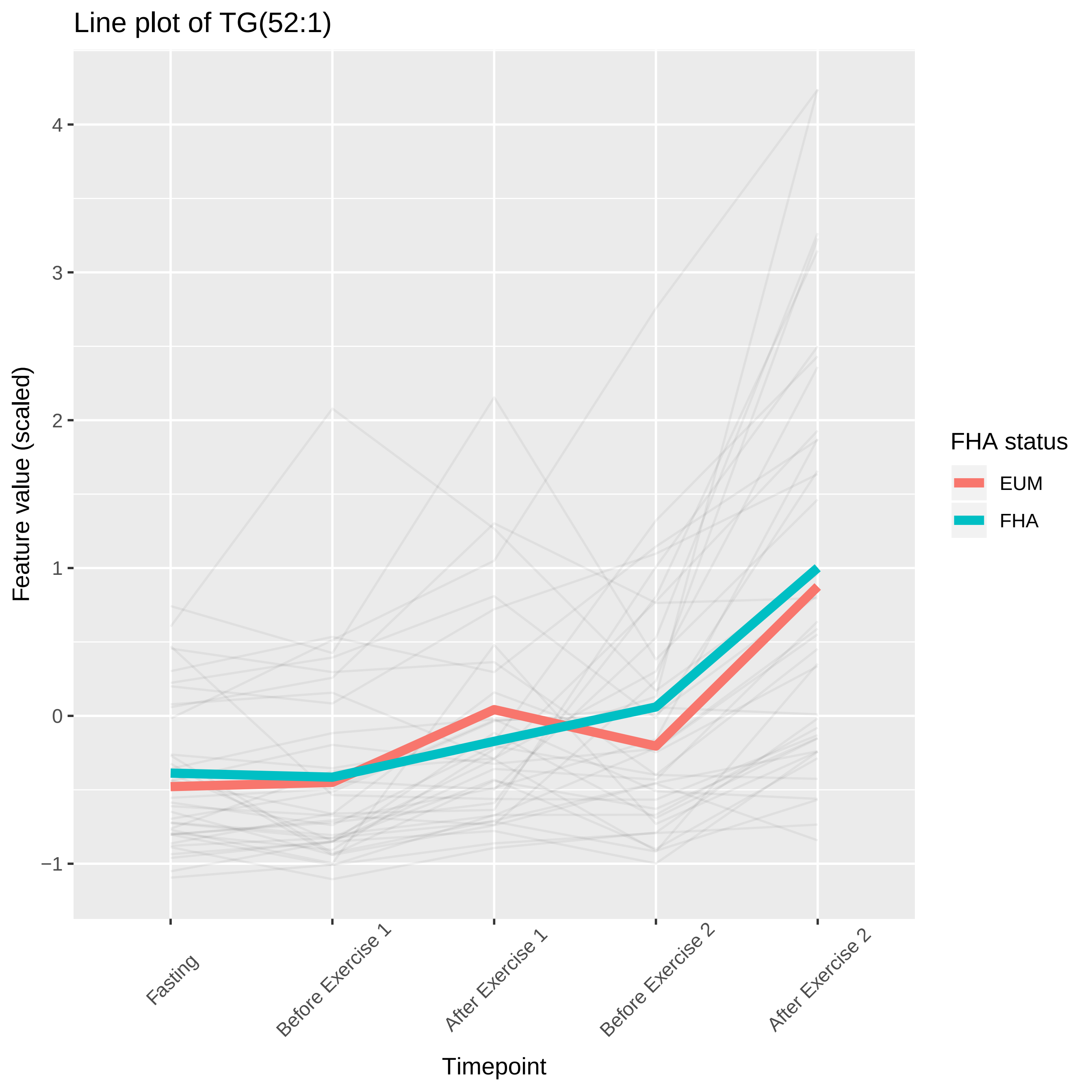

Line plot of TG(52:1)
Feature value (scaled)
4
3
2
1
0
−1
Fasting
Before Exercise 1
After Exercise 1
Before Exercise 2
After Exercise 2
Timepoint
FHA status
EUM
FHA

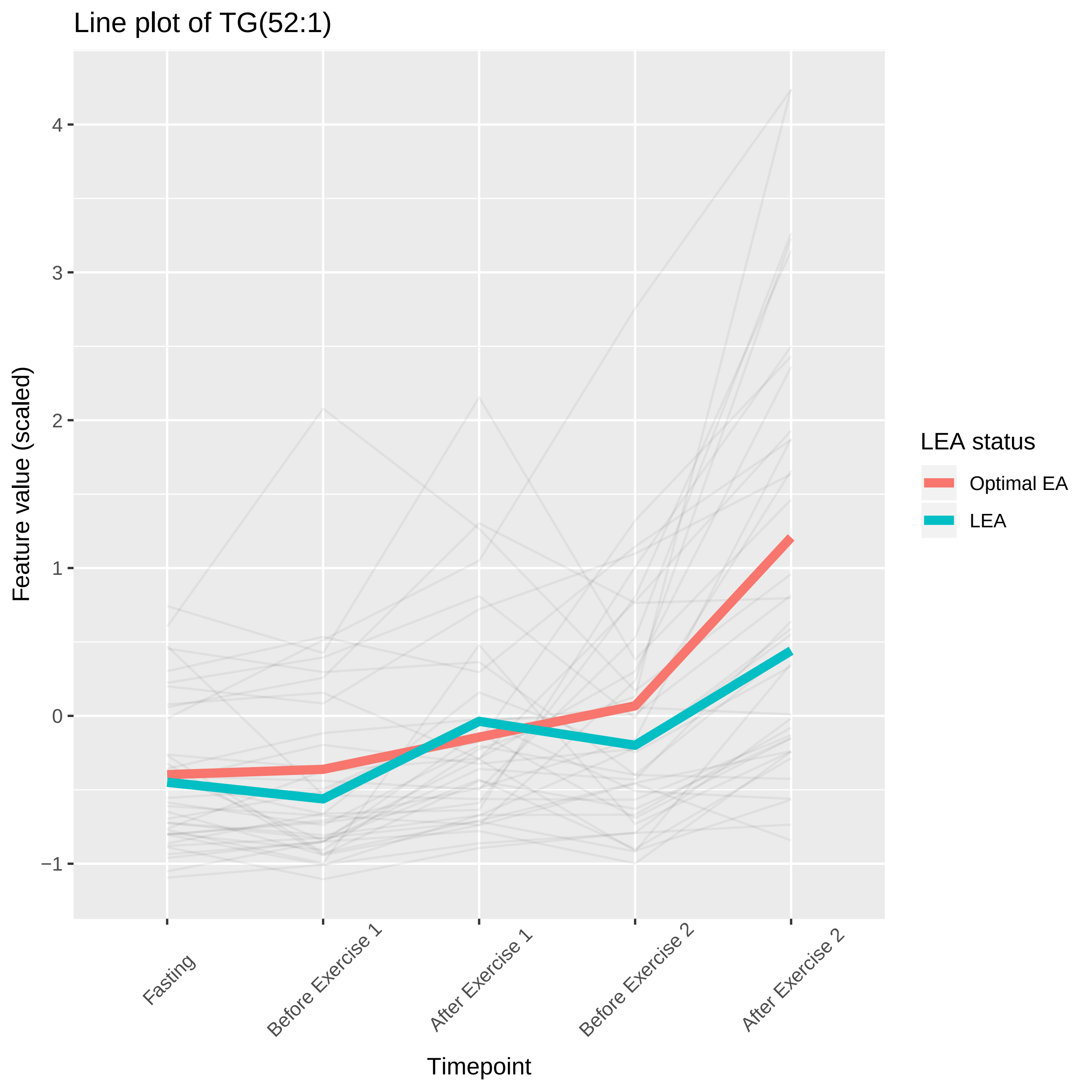

Line plot of TG(52:1)
Feature value (scaled)
4
3
2
1
0
−1
Fasting
Before Exercise 1
After Exercise 1
Before Exercise 2
After Exercise 2
Timepoint
LEA status
Optimal EA
LEA

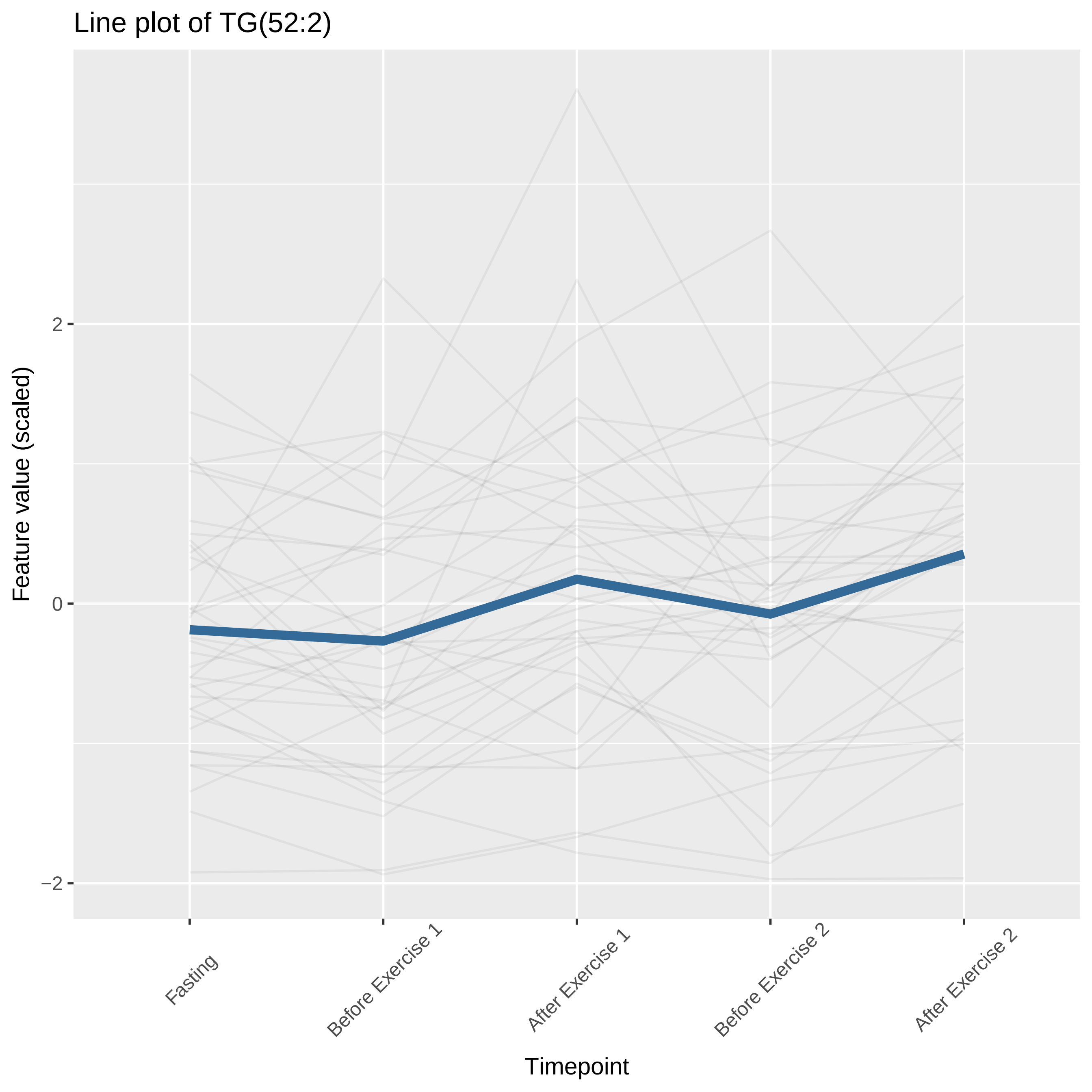

Line plot of TG(52:2)
Feature value (scaled)
2
0
−2
Fasting
Before Exercise 1
After Exercise 1
Before Exercise 2
After Exercise 2
Timepoint

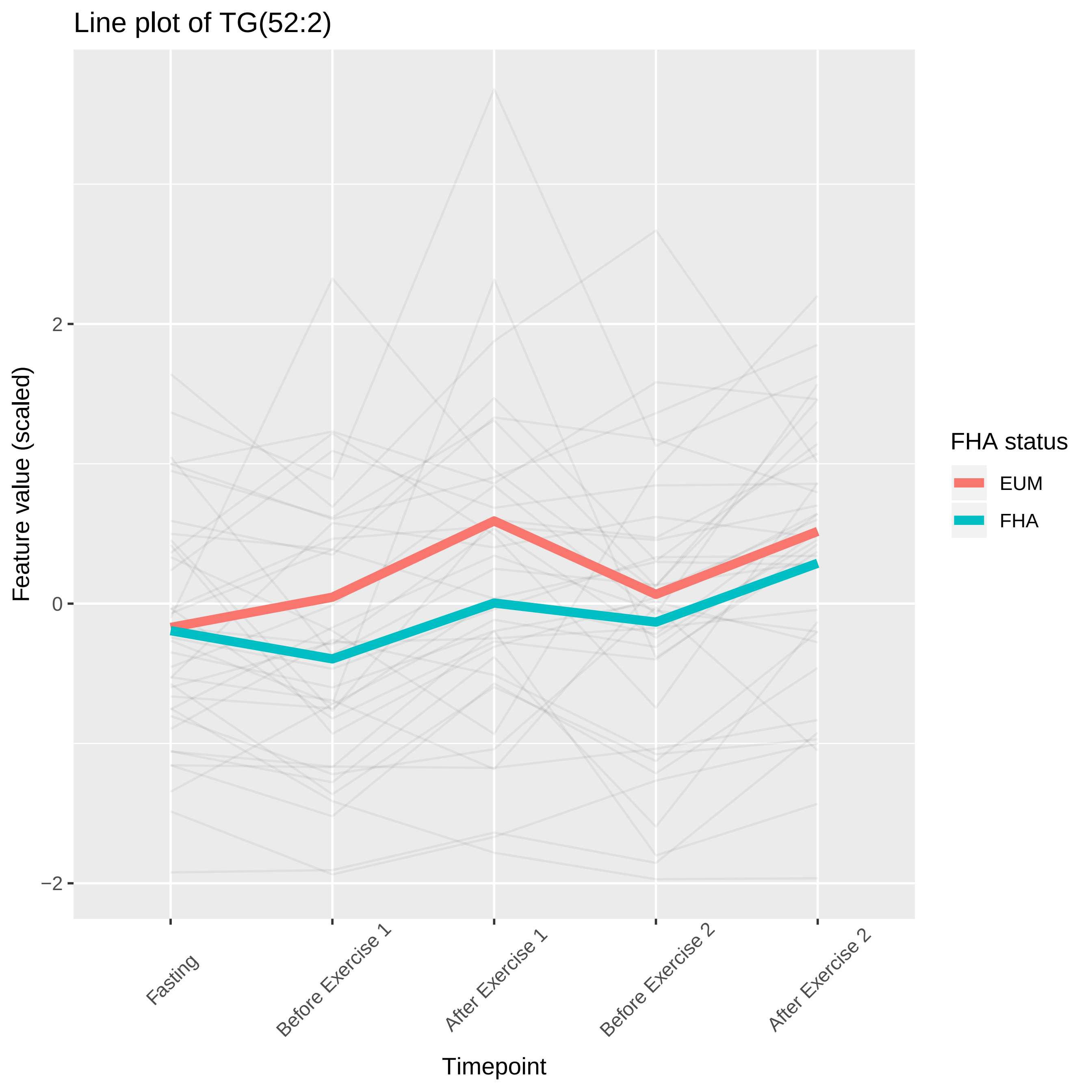

Line plot of TG(52:2)
Feature value (scaled)
2
0
−2
Fasting
Before Exercise 1
After Exercise 1
Before Exercise 2
After Exercise 2
Timepoint
FHA status
EUM
FHA

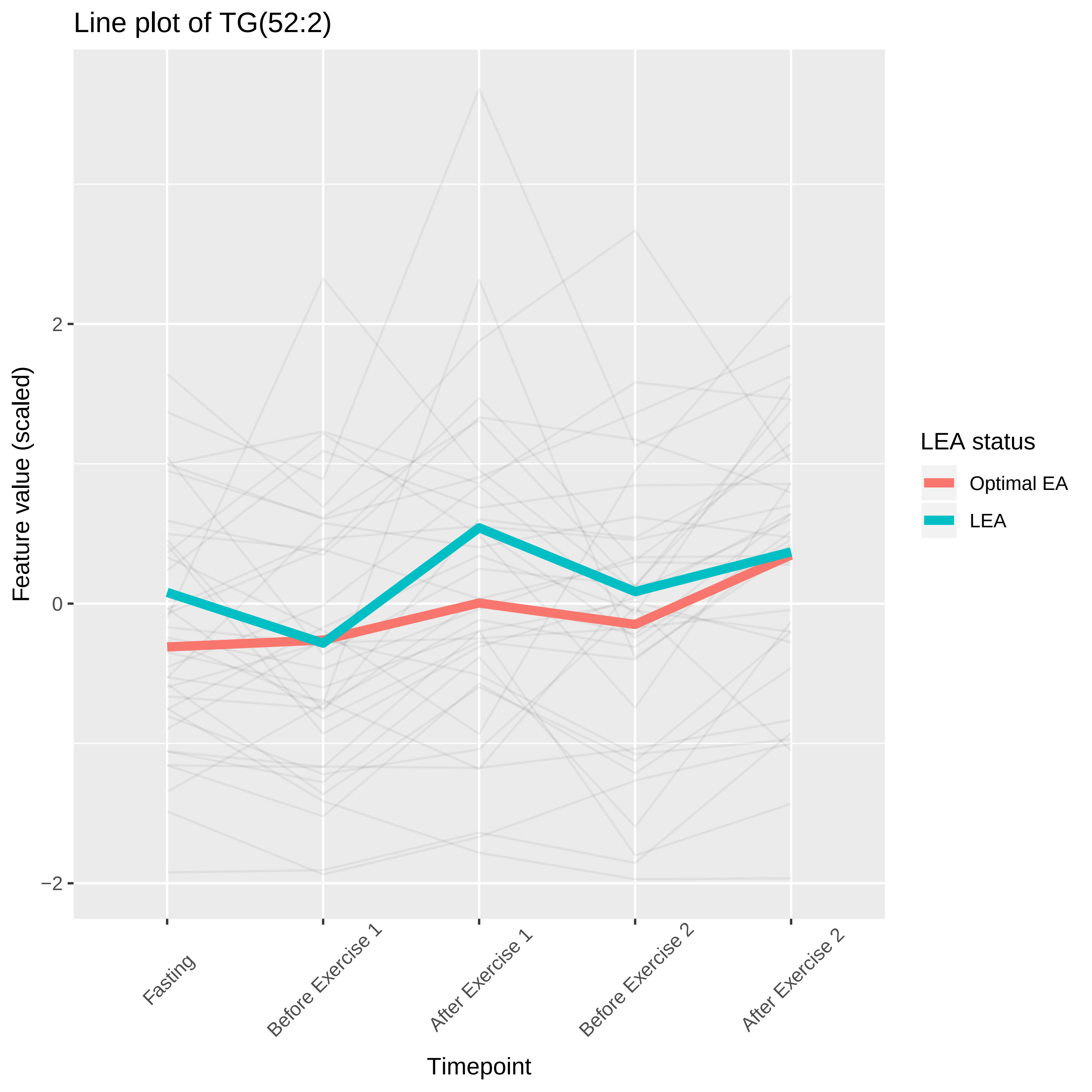

Line plot of TG(52:2)
Feature value (scaled)
2
0
−2
Fasting
Before Exercise 1
After Exercise 1
Before Exercise 2
After Exercise 2
Timepoint
LEA status
Optimal EA
LEA

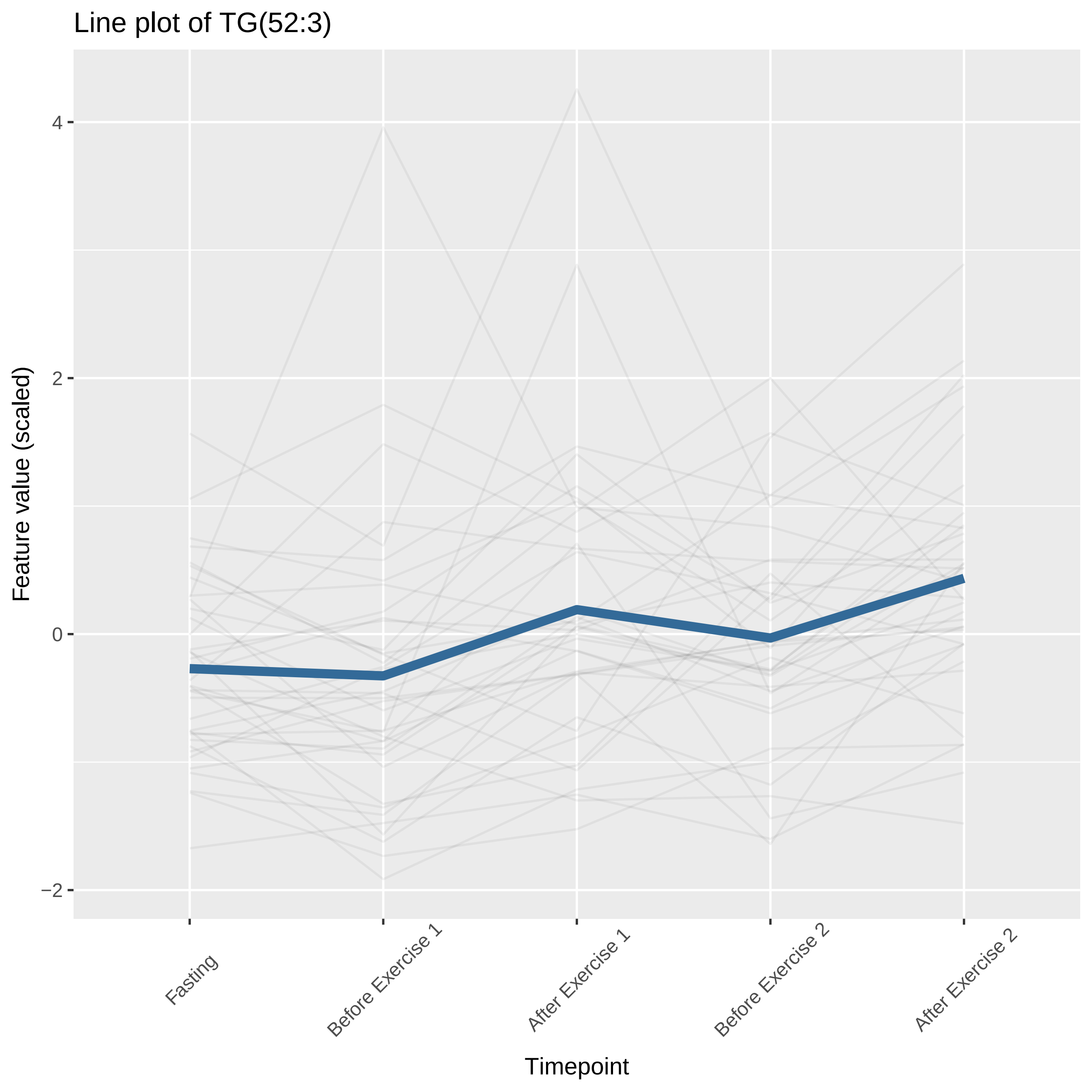

Line plot of TG(52:3)
Feature value (scaled)
4
2
0
−2
Fasting
Before Exercise 1
After Exercise 1
Before Exercise 2
After Exercise 2
Timepoint

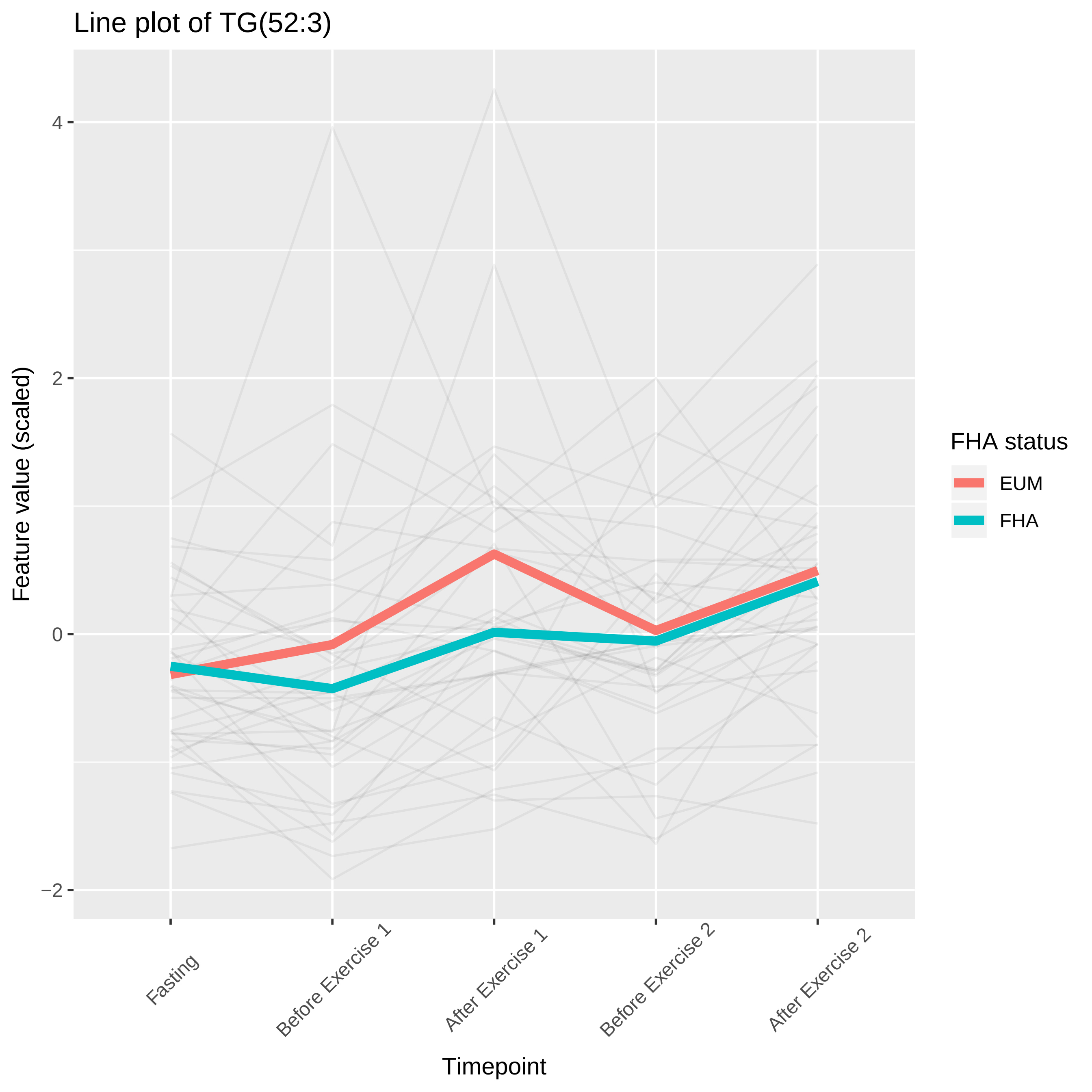

Line plot of TG(52:3)
Feature value (scaled)
4
2
0
−2
Fasting
Before Exercise 1
After Exercise 1
Before Exercise 2
After Exercise 2
Timepoint
FHA status
EUM
FHA

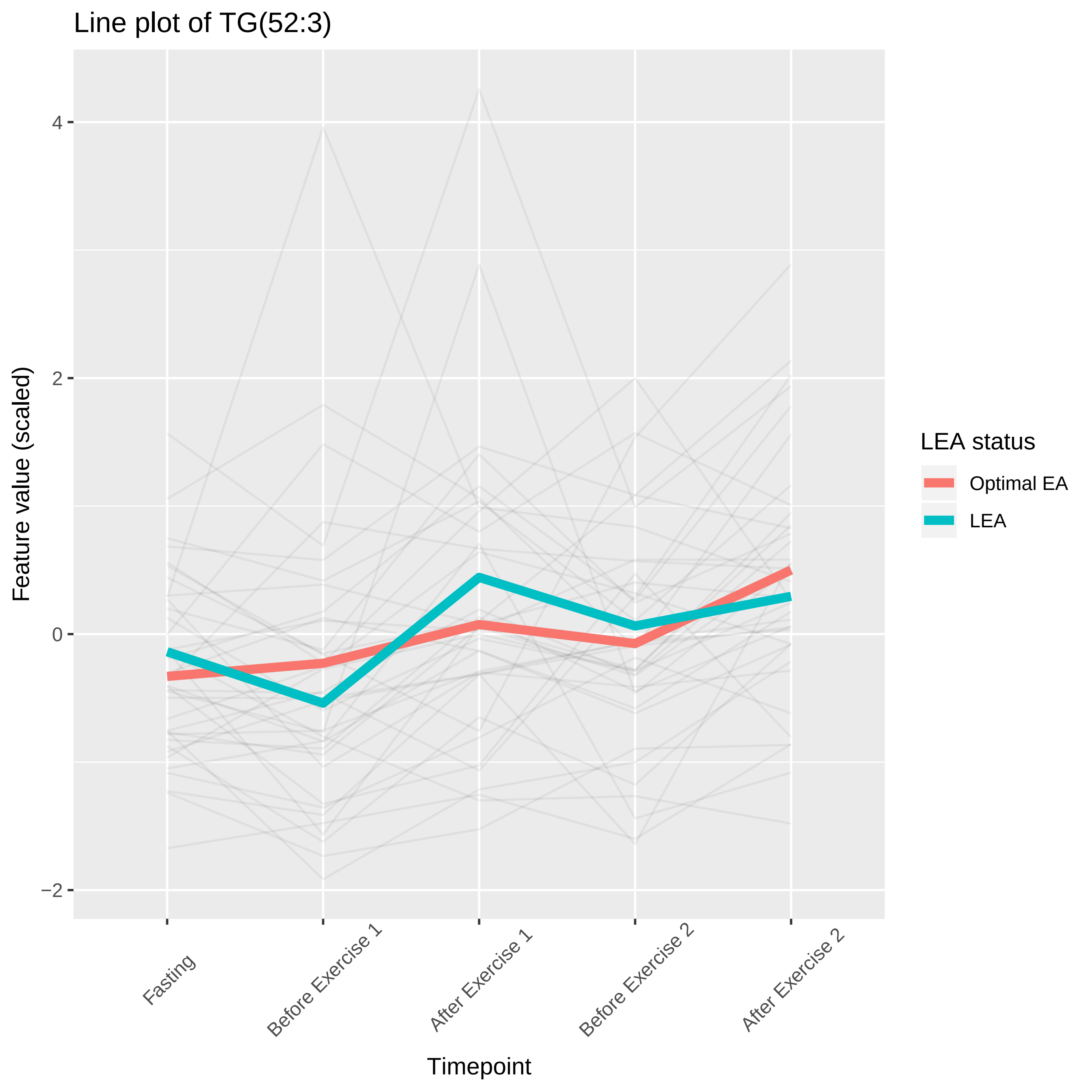

Line plot of TG(52:3)
Feature value (scaled)
4
2
0
−2
Fasting
Before Exercise 1
After Exercise 1
Before Exercise 2
After Exercise 2
Timepoint
LEA status
Optimal EA
LEA

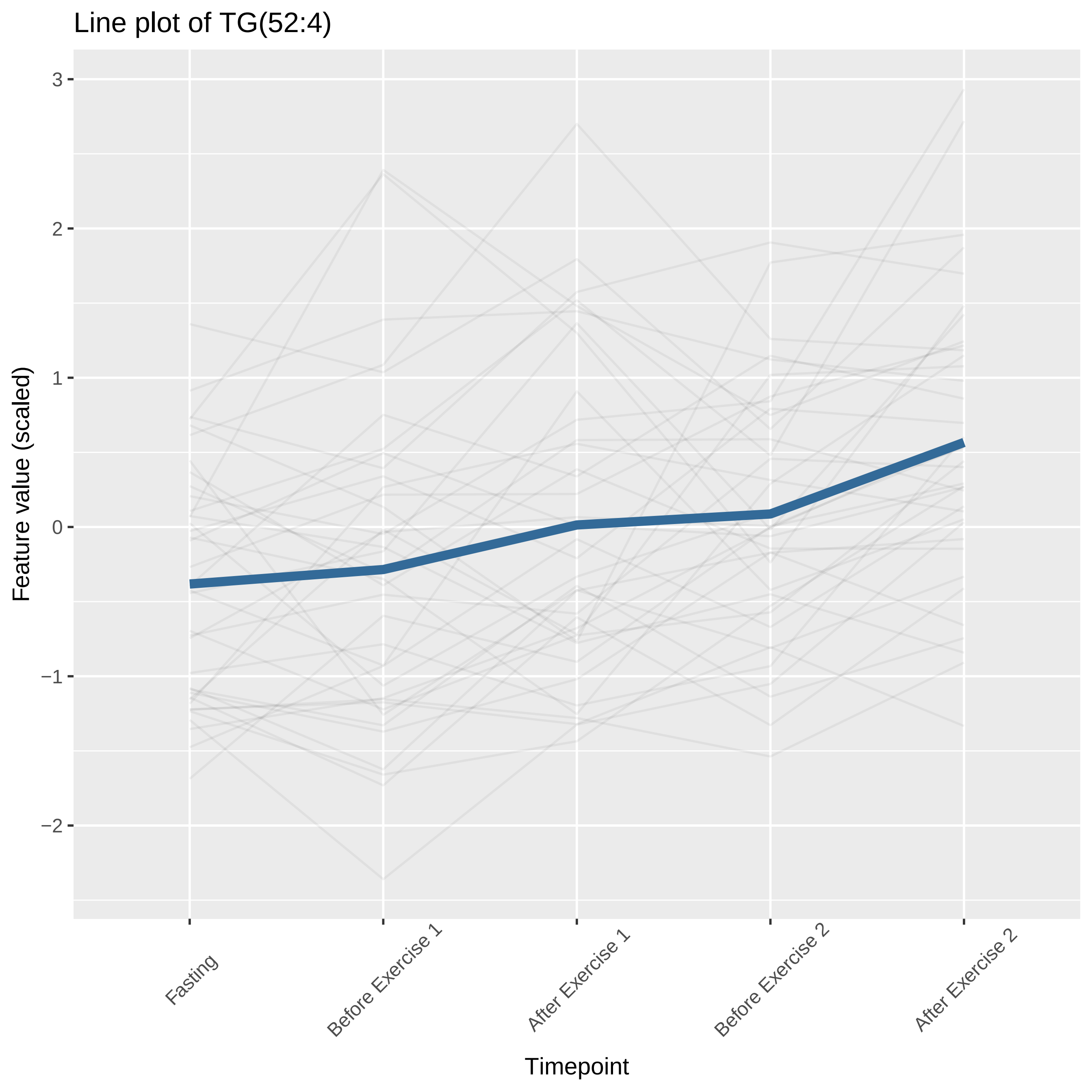

Line plot of TG(52:4)
Feature value (scaled)
3
2
1
0
−1
−2
Fasting
Before Exercise 1
After Exercise 1
Before Exercise 2
After Exercise 2
Timepoint

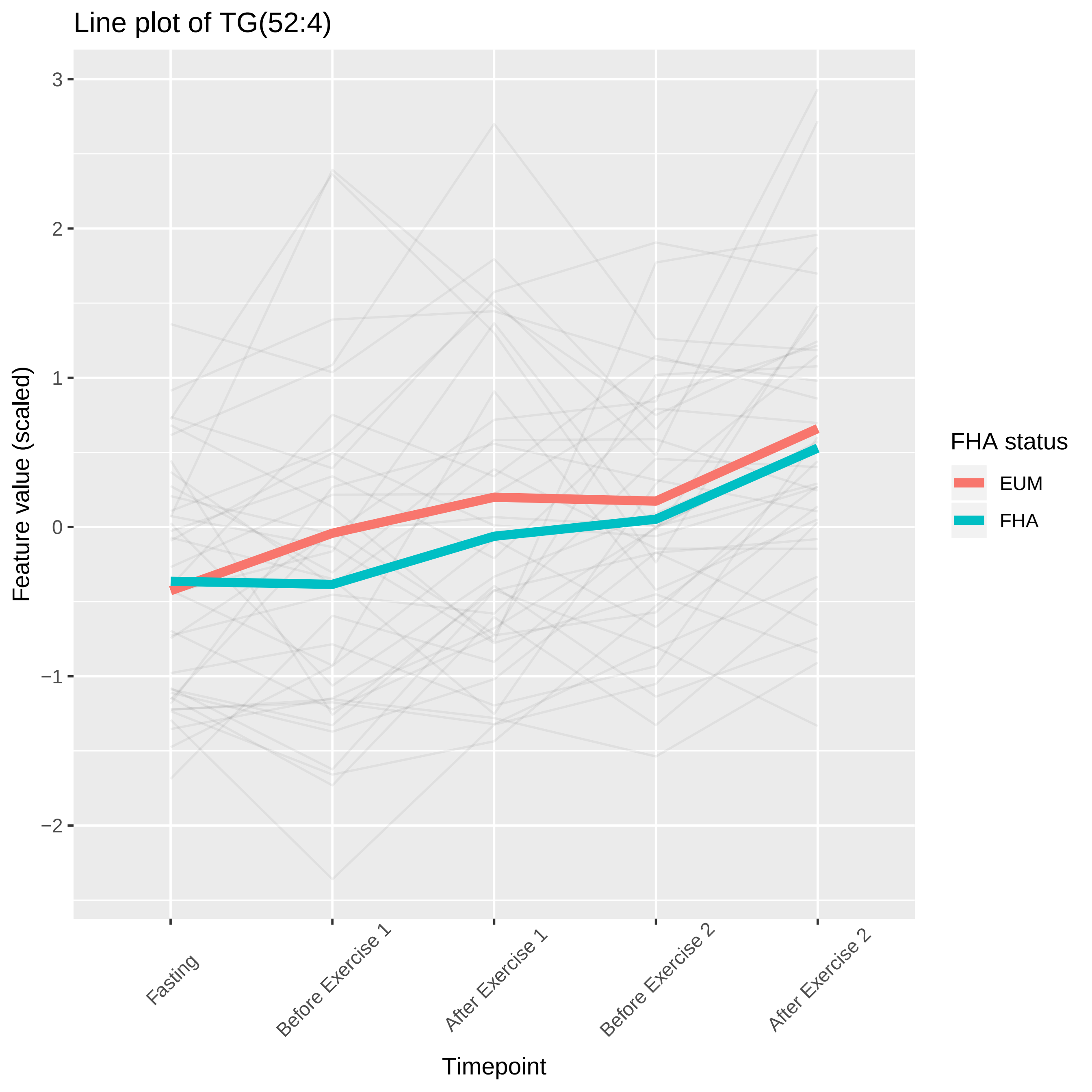

Line plot of TG(52:4)
Feature value (scaled)
3
2
1
0
−1
−2
Fasting
Before Exercise 1
After Exercise 1
Before Exercise 2
After Exercise 2
Timepoint
FHA status
EUM
FHA

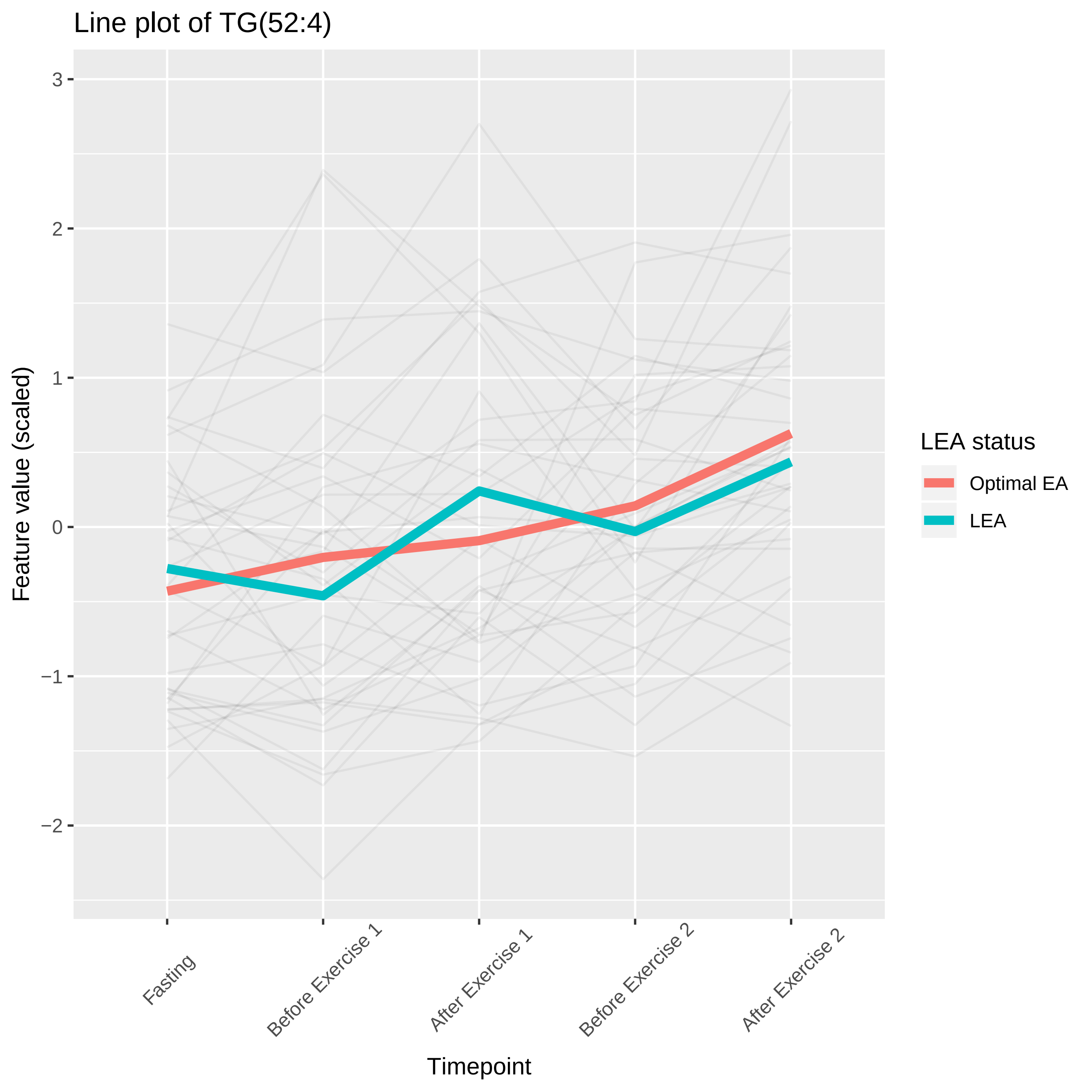

Line plot of TG(52:4)
Feature value (scaled)
3
2
1
0
−1
−2
Fasting
Before Exercise 1
After Exercise 1
Before Exercise 2
After Exercise 2
Timepoint
LEA status
Optimal EA
LEA

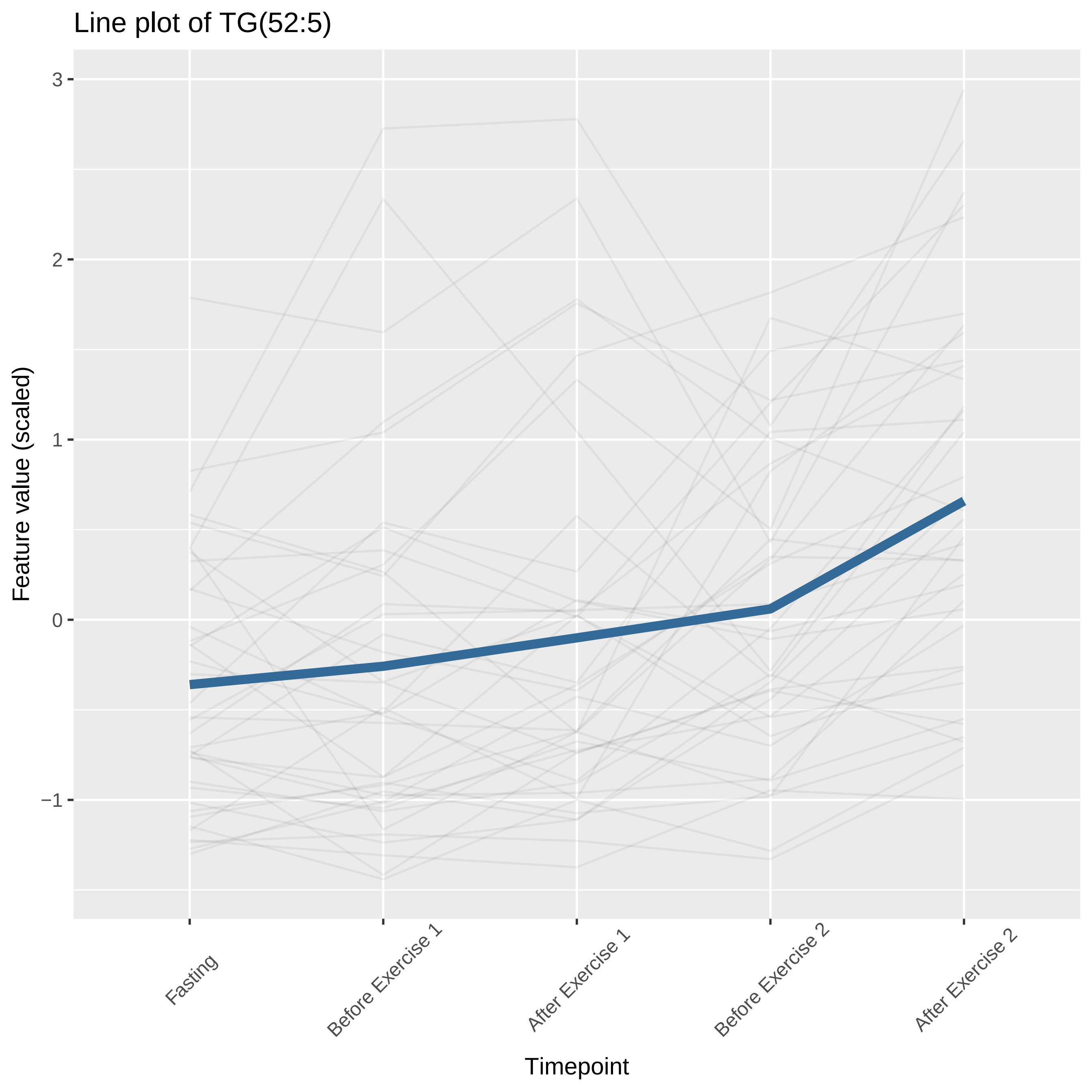

Line plot of TG(52:5)
Feature value (scaled)
3
2
1
0
−1
Fasting
Before Exercise 1
After Exercise 1
Before Exercise 2
After Exercise 2
Timepoint

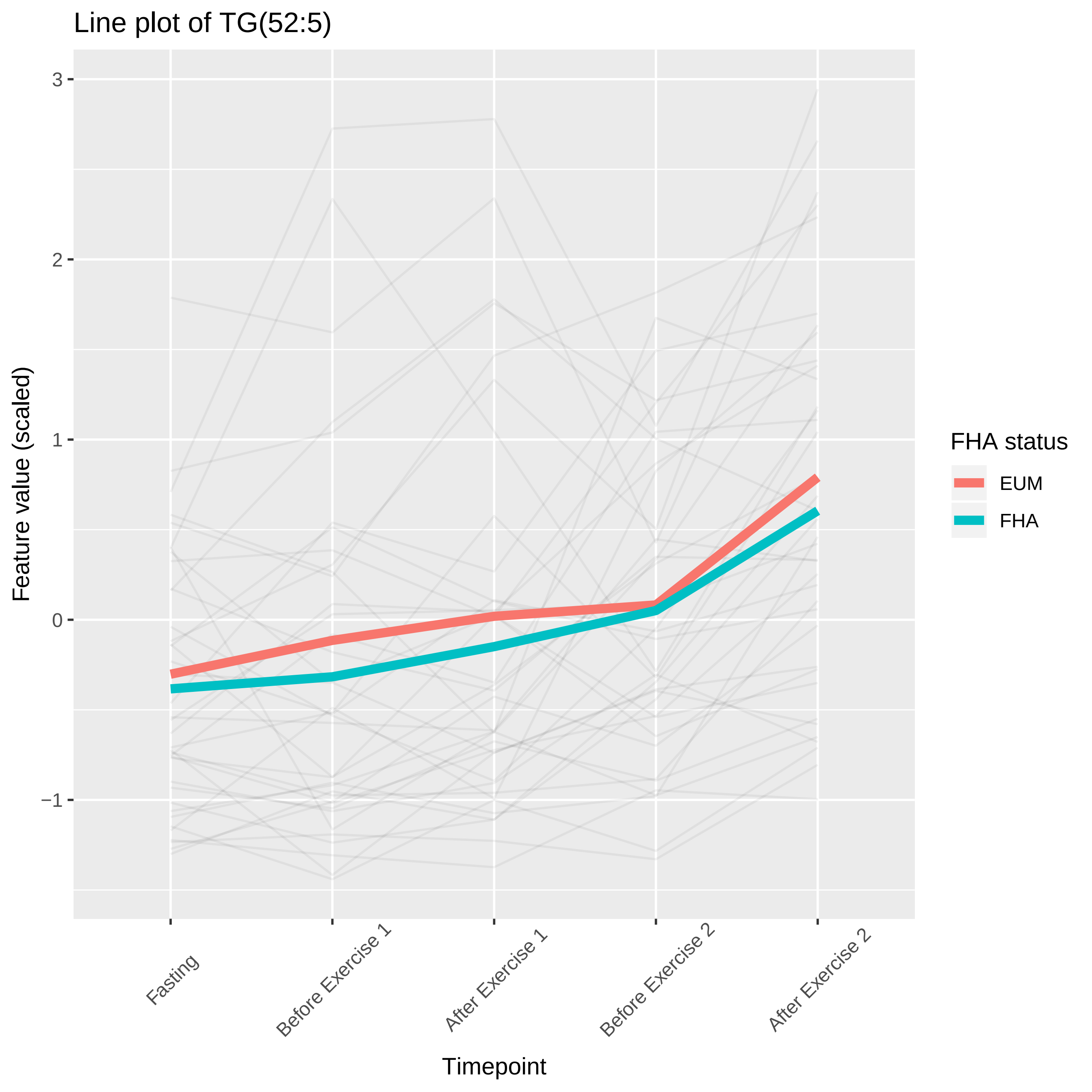

Line plot of TG(52:5)
Feature value (scaled)
3
2
1
0
−1
Fasting
Before Exercise 1
After Exercise 1
Before Exercise 2
After Exercise 2
Timepoint
FHA status
EUM
FHA

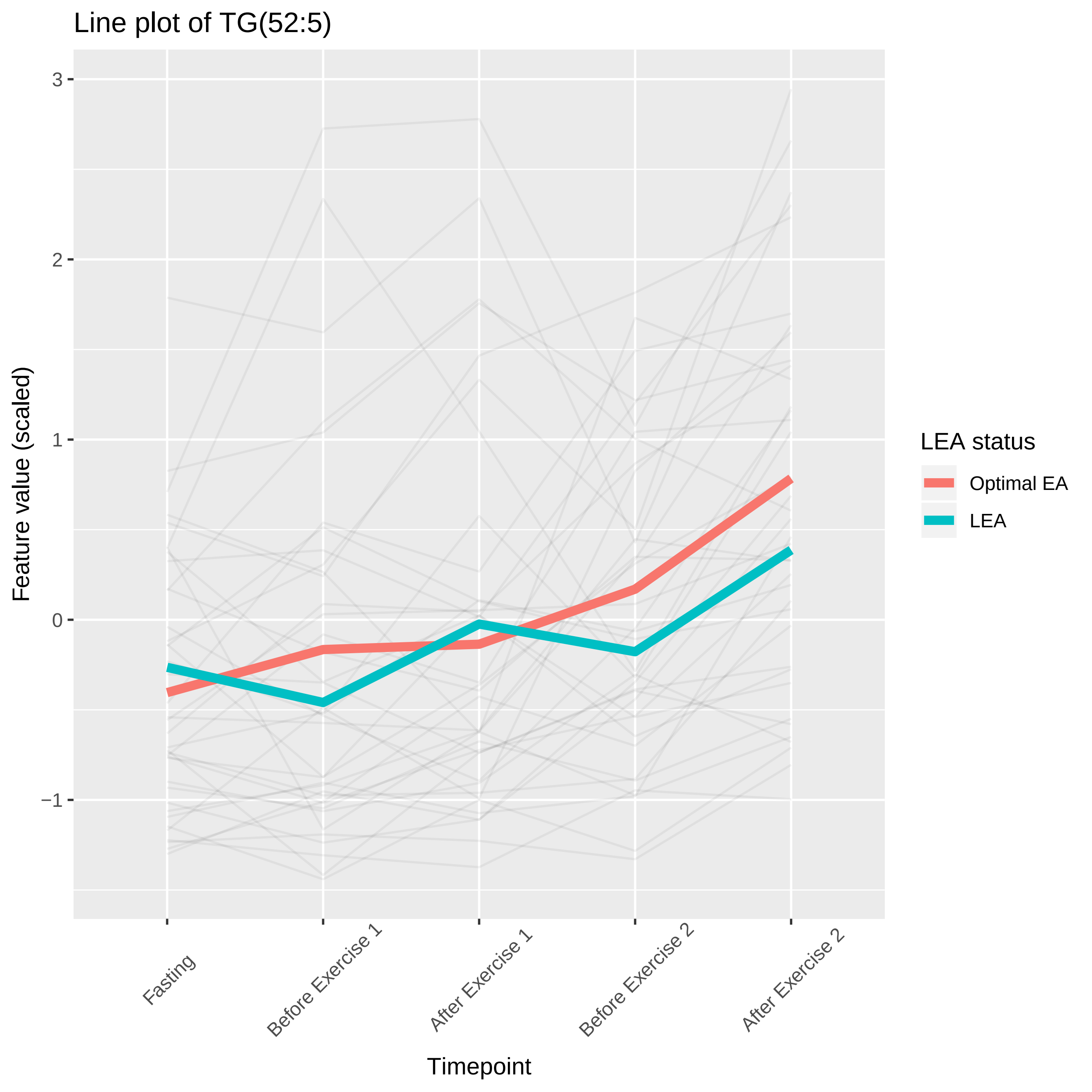

Line plot of TG(52:5)
Feature value (scaled)
3
2
1
0
−1
Fasting
Before Exercise 1
After Exercise 1
Before Exercise 2
After Exercise 2
Timepoint
LEA status
Optimal EA
LEA

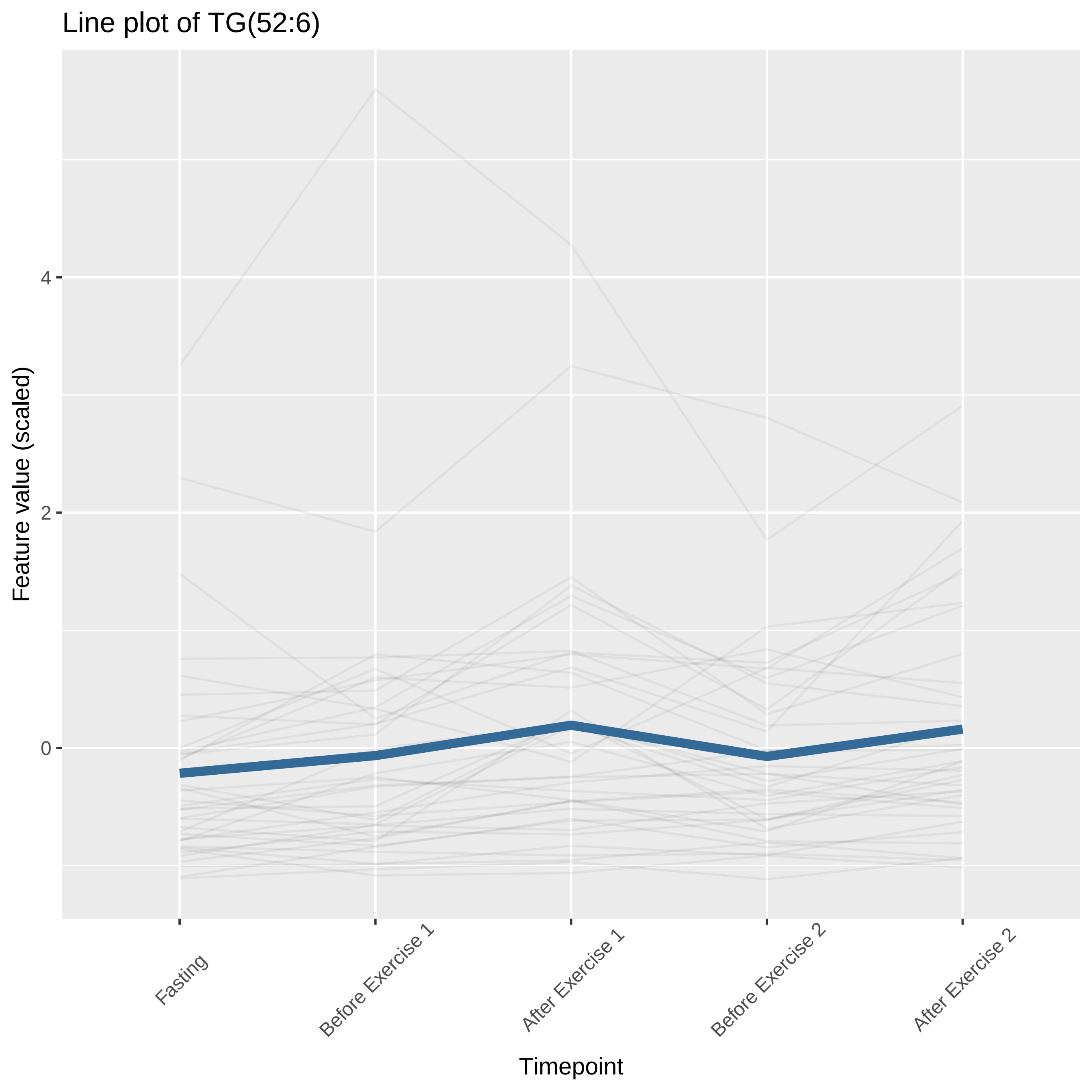

Line plot of TG(52:6)
Feature value (scaled)
4
2
0
Fasting
Before Exercise 1
After Exercise 1
Before Exercise 2
After Exercise 2
Timepoint

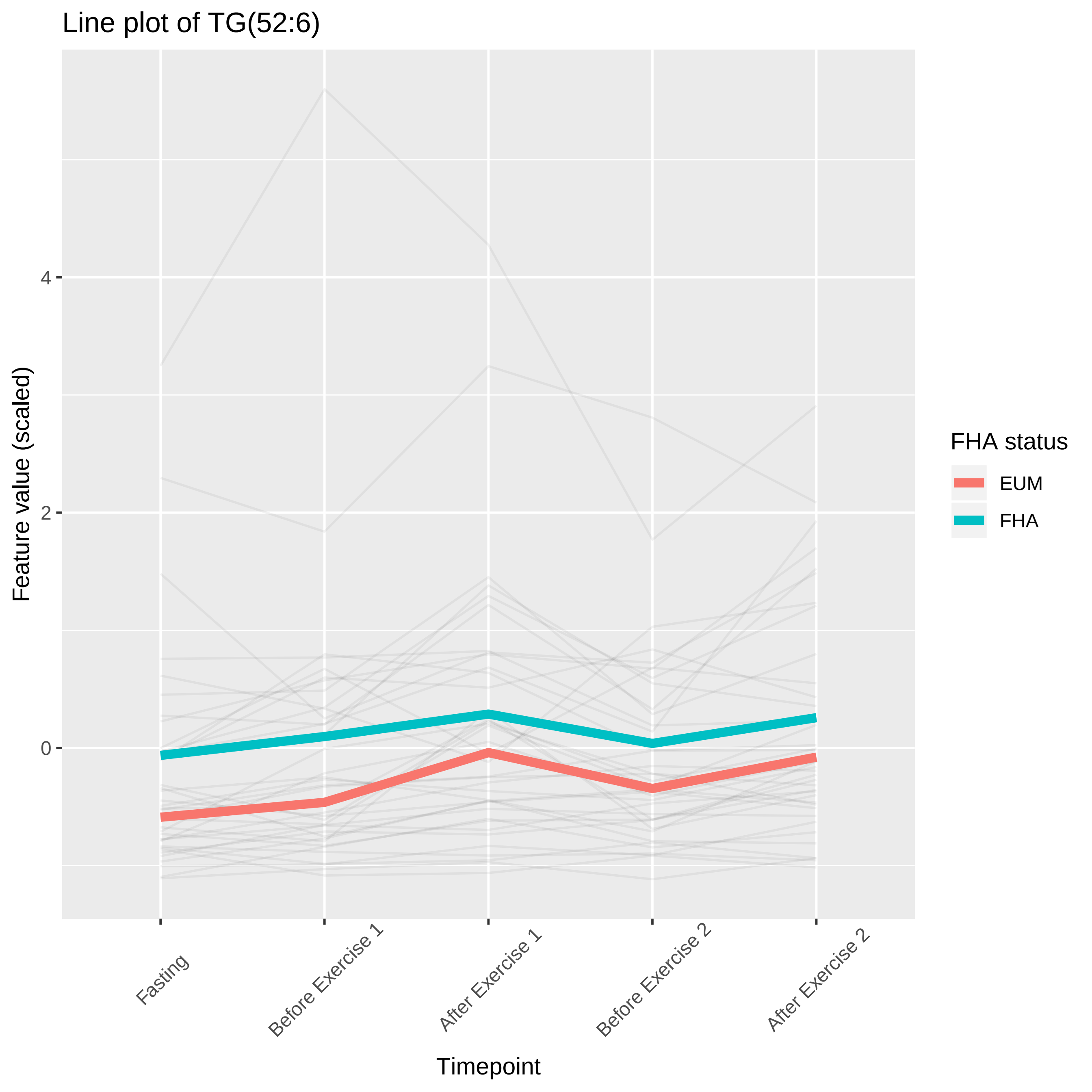

Line plot of TG(52:6)
Feature value (scaled)
4
2
0
Fasting
Before Exercise 1
After Exercise 1
Before Exercise 2
After Exercise 2
Timepoint
FHA status
EUM
FHA

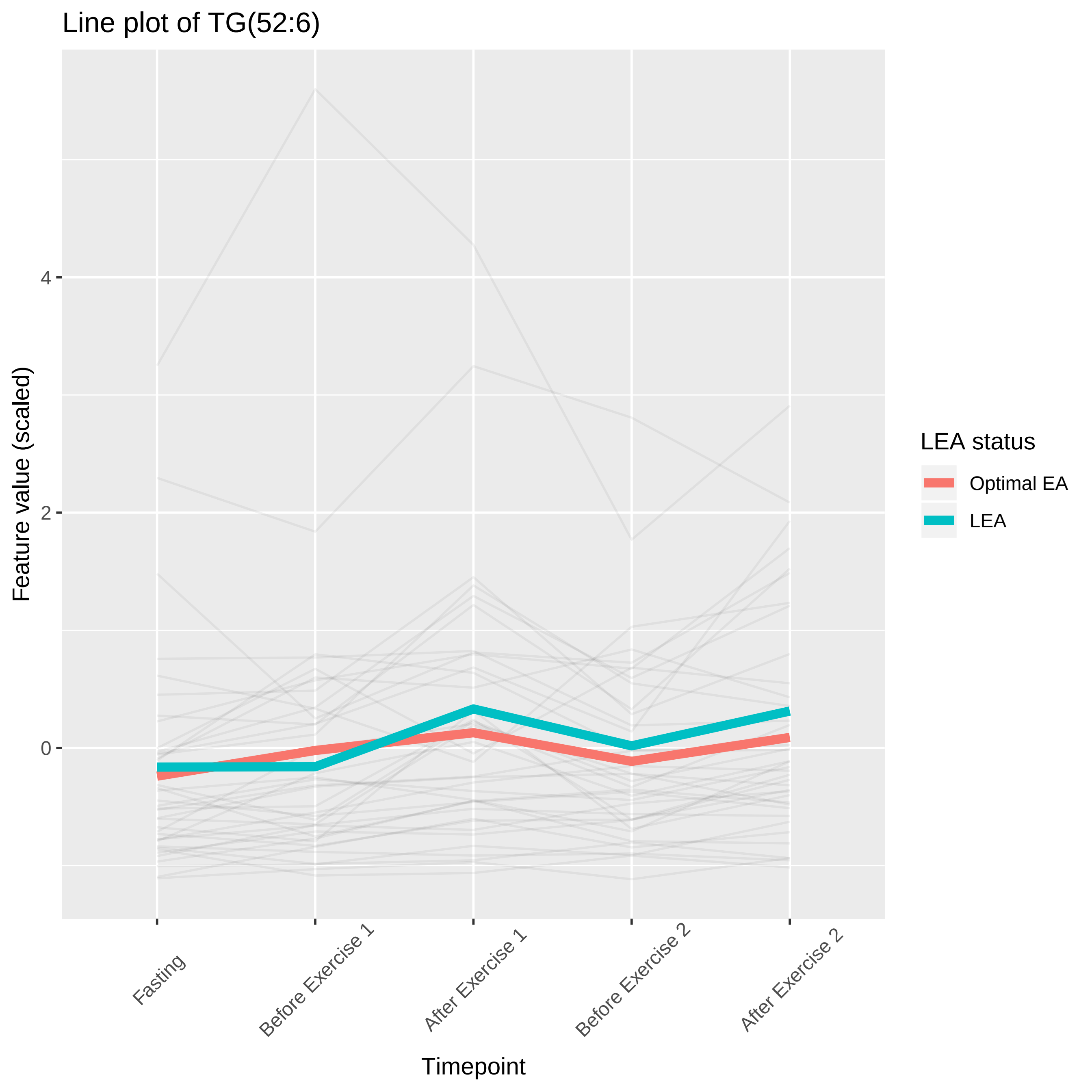
Line plot of TG(52:6)
Feature value (scaled)
4
2
0
Fasting
Before Exercise 1
After Exercise 1
Before Exercise 2
After Exercise 2
Timepoint
LEA status
Optimal EA
LEA

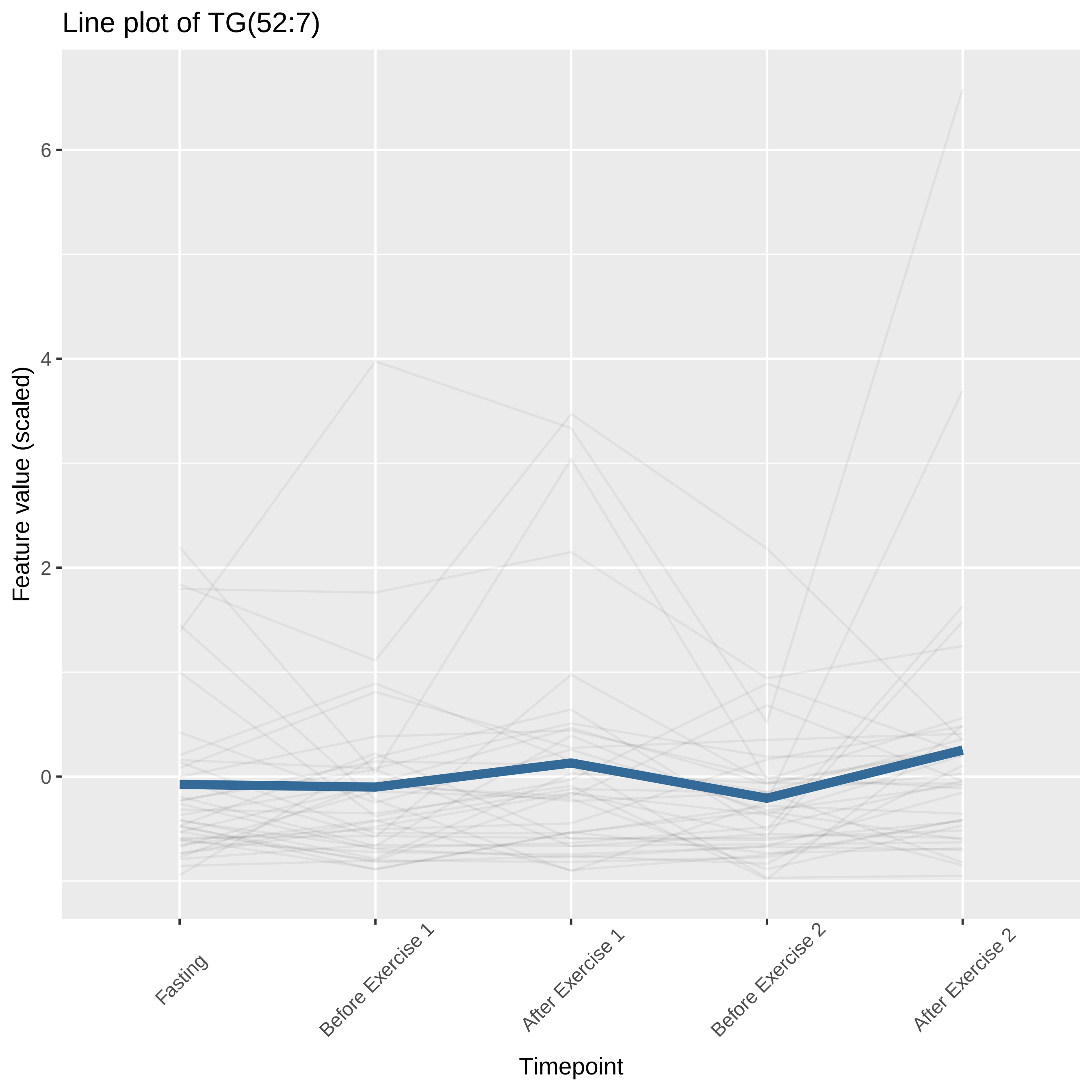

Line plot of TG(52:7)
Feature value (scaled)
6
4
2
0
Fasting
Before Exercise 1
After Exercise 1
Before Exercise 2
After Exercise 2
Timepoint

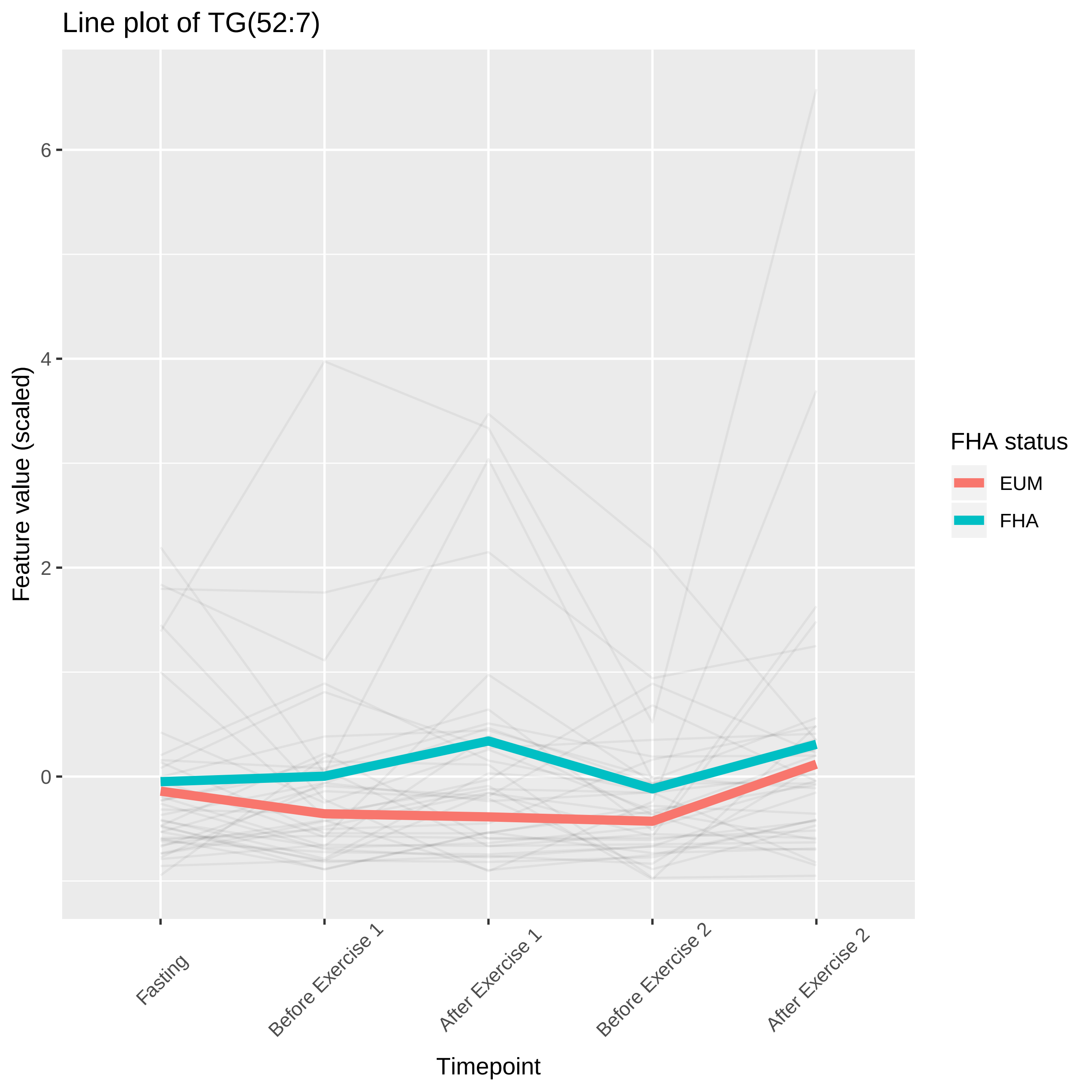

Line plot of TG(52:7)
Feature value (scaled)
6
4
2
0
Fasting
Before Exercise 1
After Exercise 1
Before Exercise 2
After Exercise 2
Timepoint
FHA status
EUM
FHA

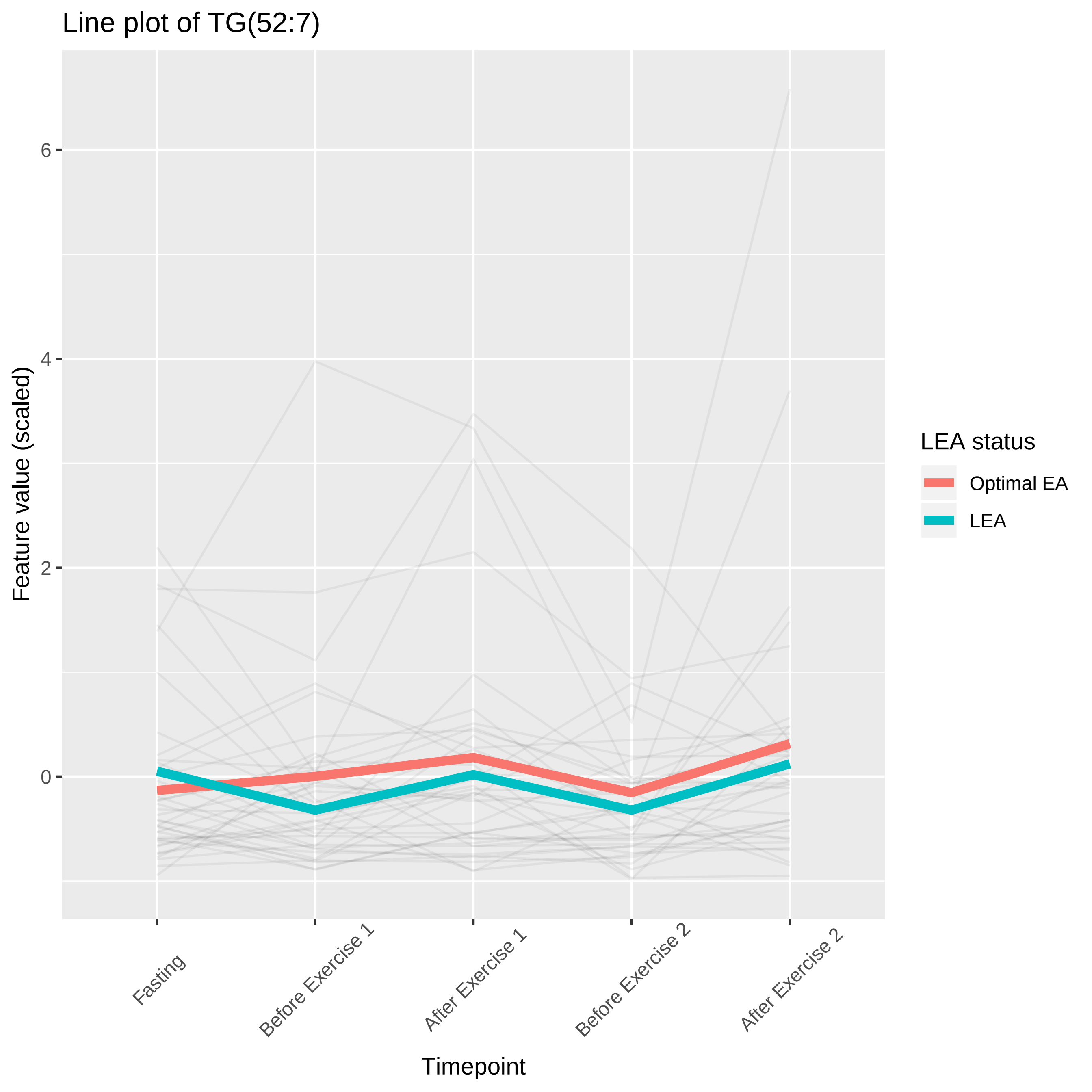
Line plot of TG(52:7)
Feature value (scaled)
0
2
4
6
Fasting
Before Exercise 1
After Exercise 1
Before Exercise 2
After Exercise 2
Timepoint
LEA status
Optimal EA
LEA

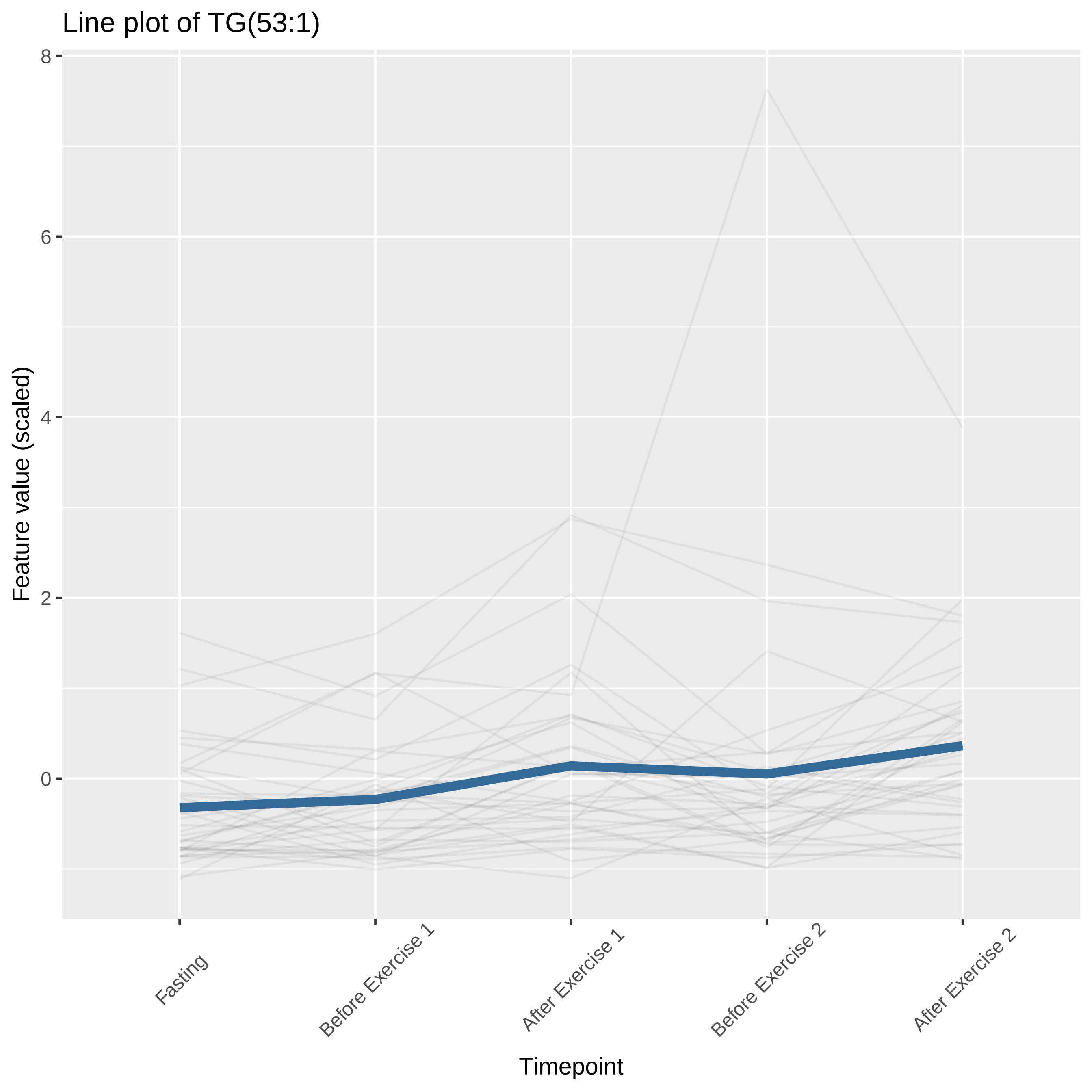
Line plot of TG(53:1)
Feature value (scaled)
0
2
4
6
8
Fasting
Before Exercise 1
After Exercise 1
Before Exercise 2
After Exercise 2
Timepoint

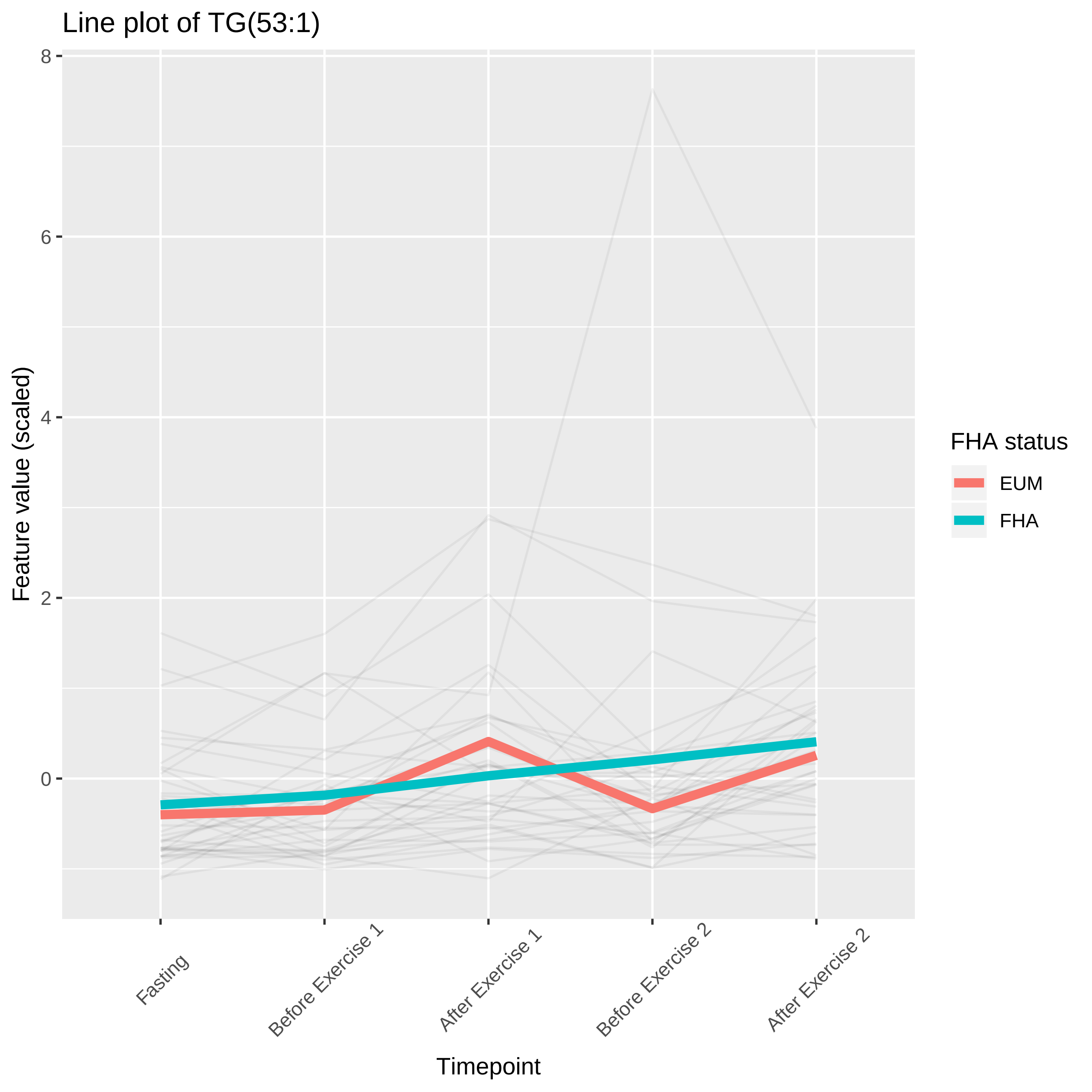

Line plot of TG(53:1)
Feature value (scaled)
8
6
4
2
0
Fasting
Before Exercise 1
After Exercise 1
Before Exercise 2
After Exercise 2
Timepoint
FHA status
EUM
FHA

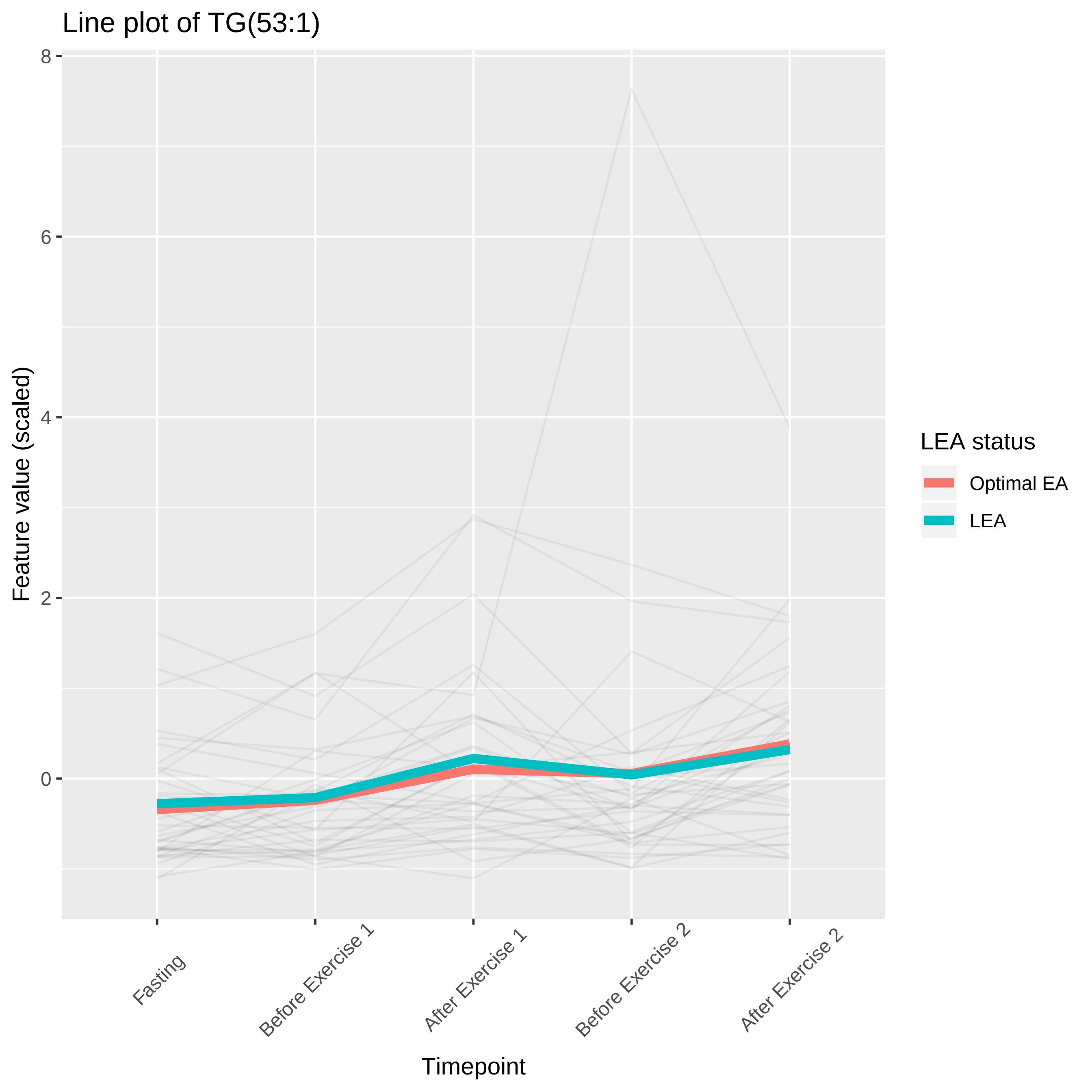
Line plot of TG(53:1)
Feature value (scaled)
8
6
4
2
0
Fasting
Before Exercise 1
After Exercise 1
Before Exercise 2
After Exercise 2
Timepoint
LEA status
Optimal EA
LEA

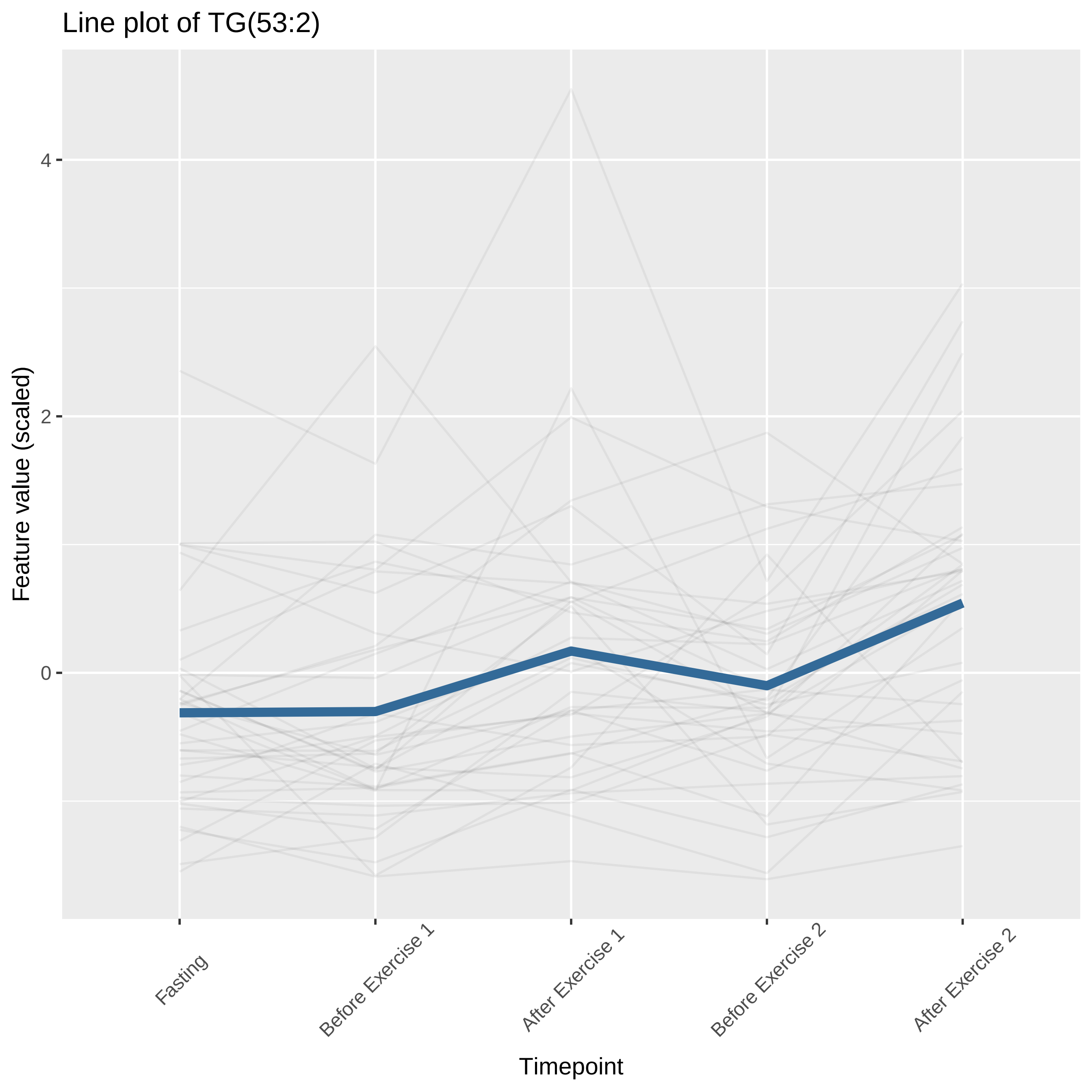

Line plot of TG(53:2)
Feature value (scaled)
4
2
0
Fasting
Before Exercise 1
After Exercise 1
Before Exercise 2
After Exercise 2
Timepoint

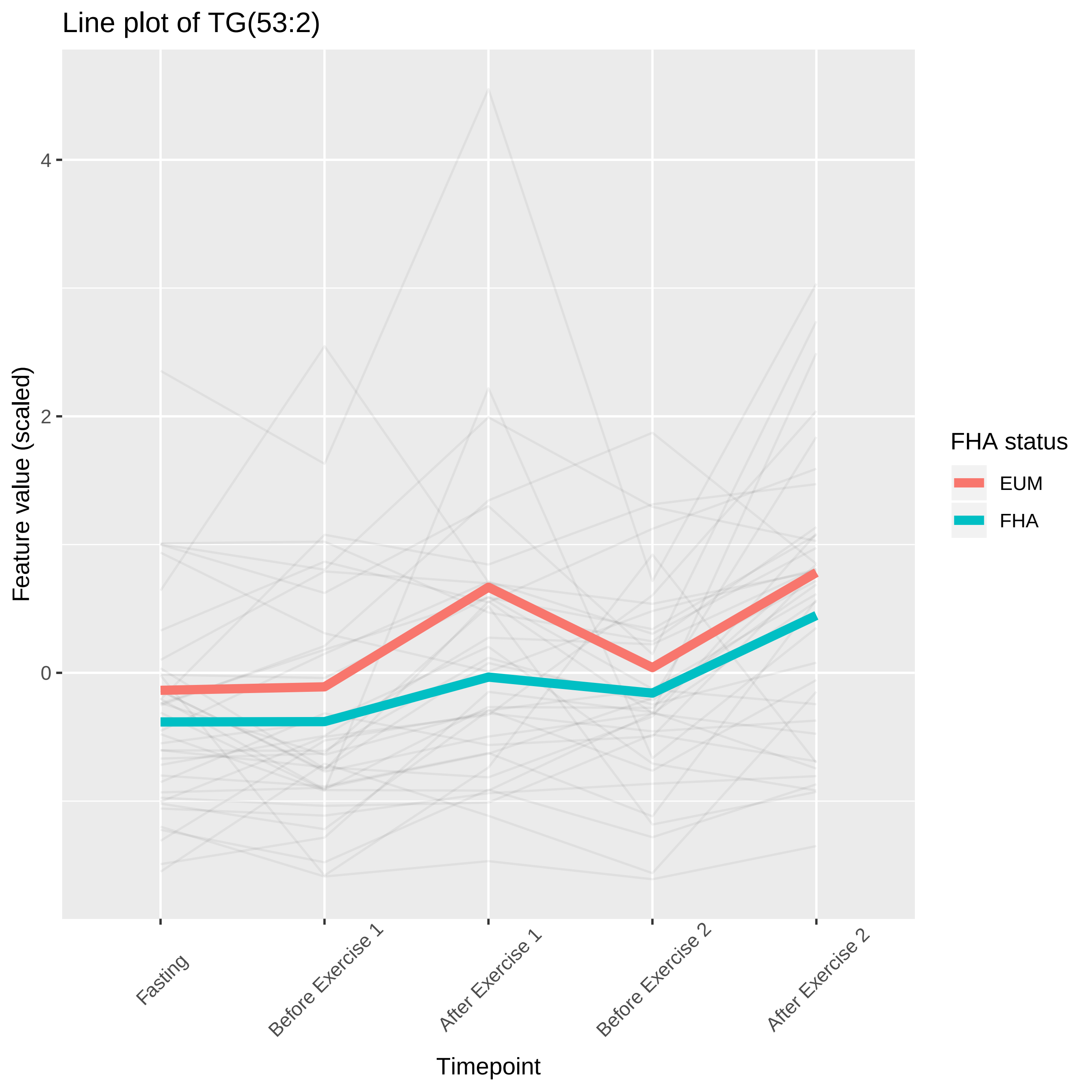

Line plot of TG(53:2)
Feature value (scaled)
4
2
0
Fasting
Before Exercise 1
After Exercise 1
Before Exercise 2
After Exercise 2
Timepoint
FHA status
EUM
FHA

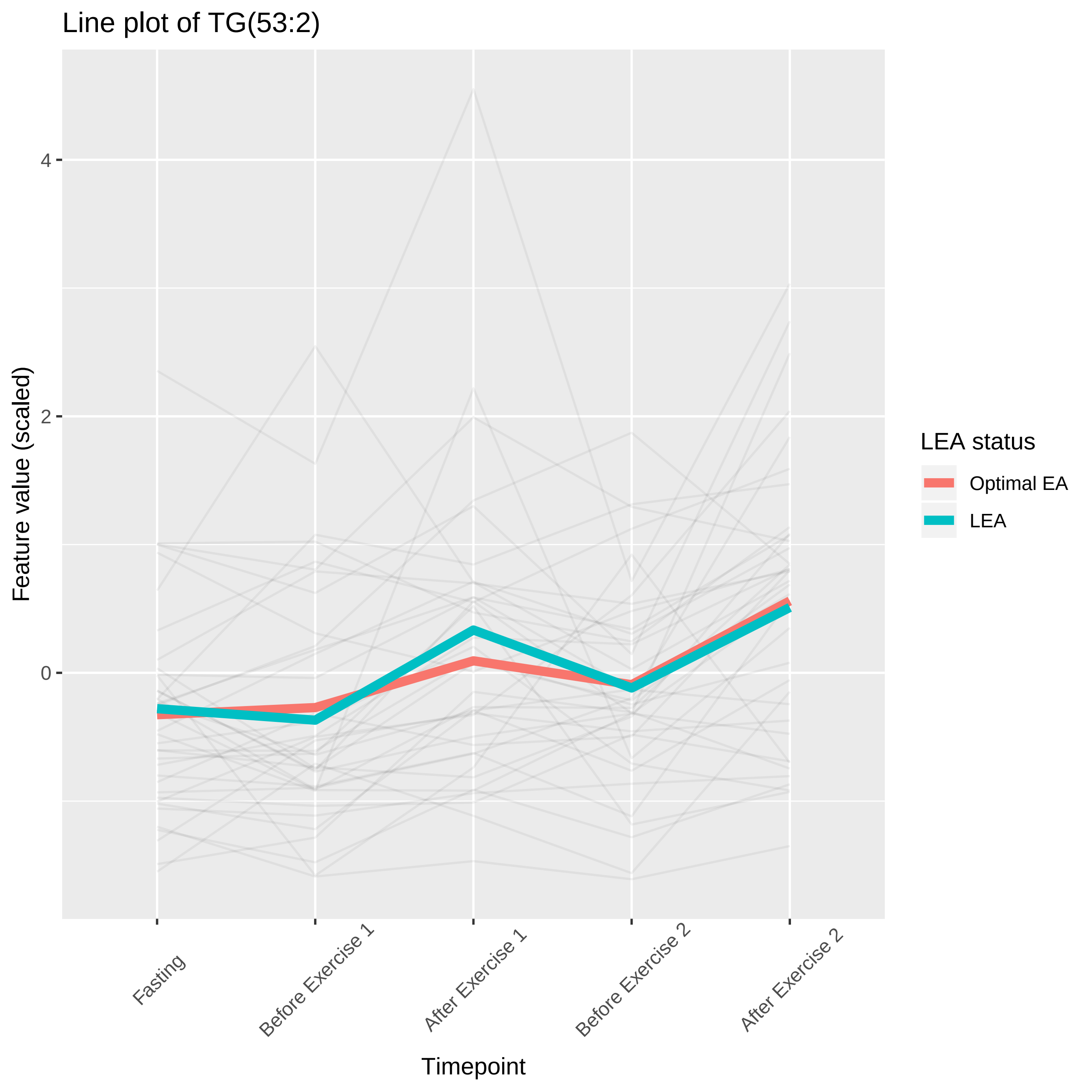

Line plot of TG(53:2)
Feature value (scaled)
4
2
0
Fasting
Before Exercise 1
After Exercise 1
Before Exercise 2
After Exercise 2
Timepoint
LEA status
Optimal EA
LEA

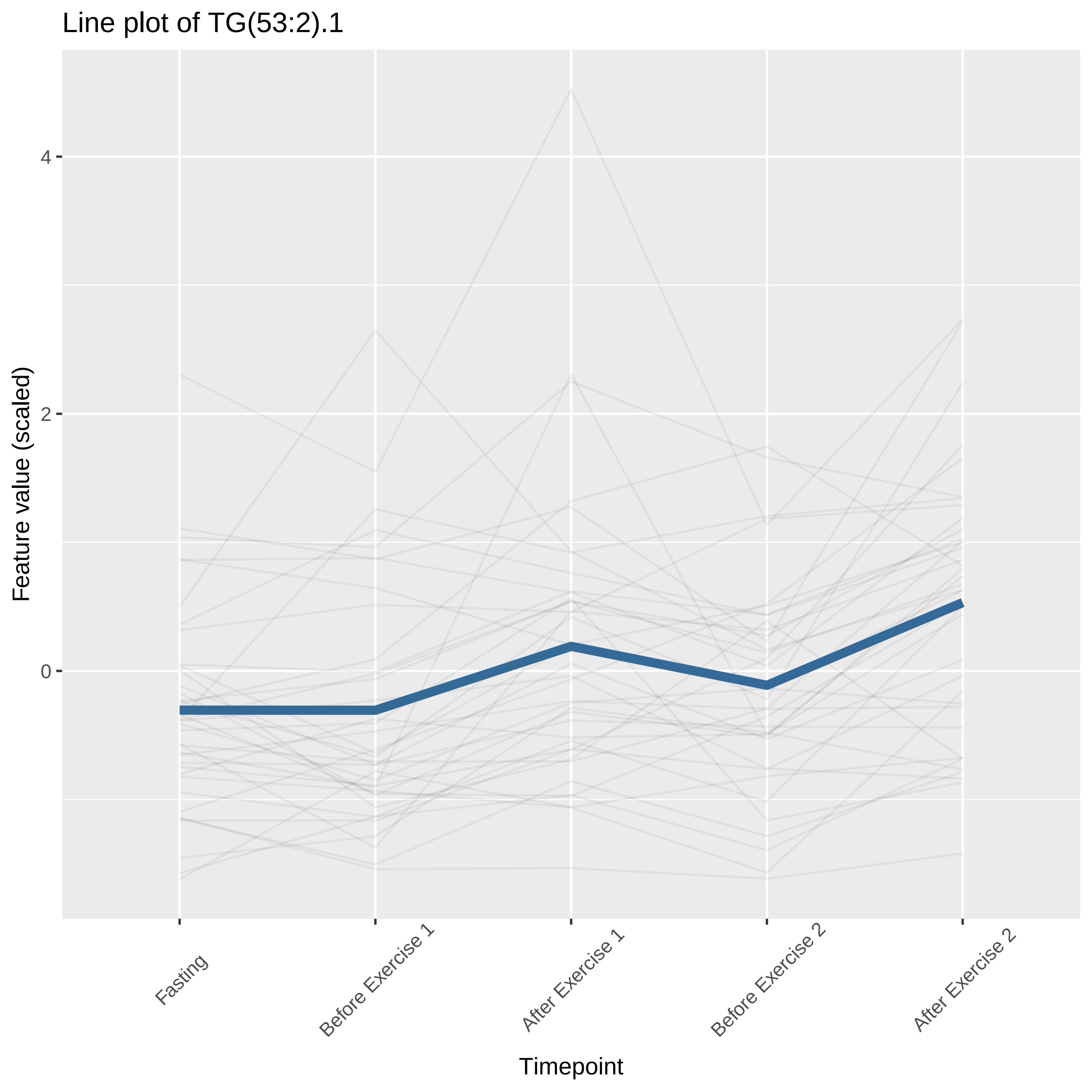

Line plot of TG(53:2).1
Feature value (scaled)
4
2
0
Fasting
Before Exercise 1
After Exercise 1
Before Exercise 2
After Exercise 2
Timepoint

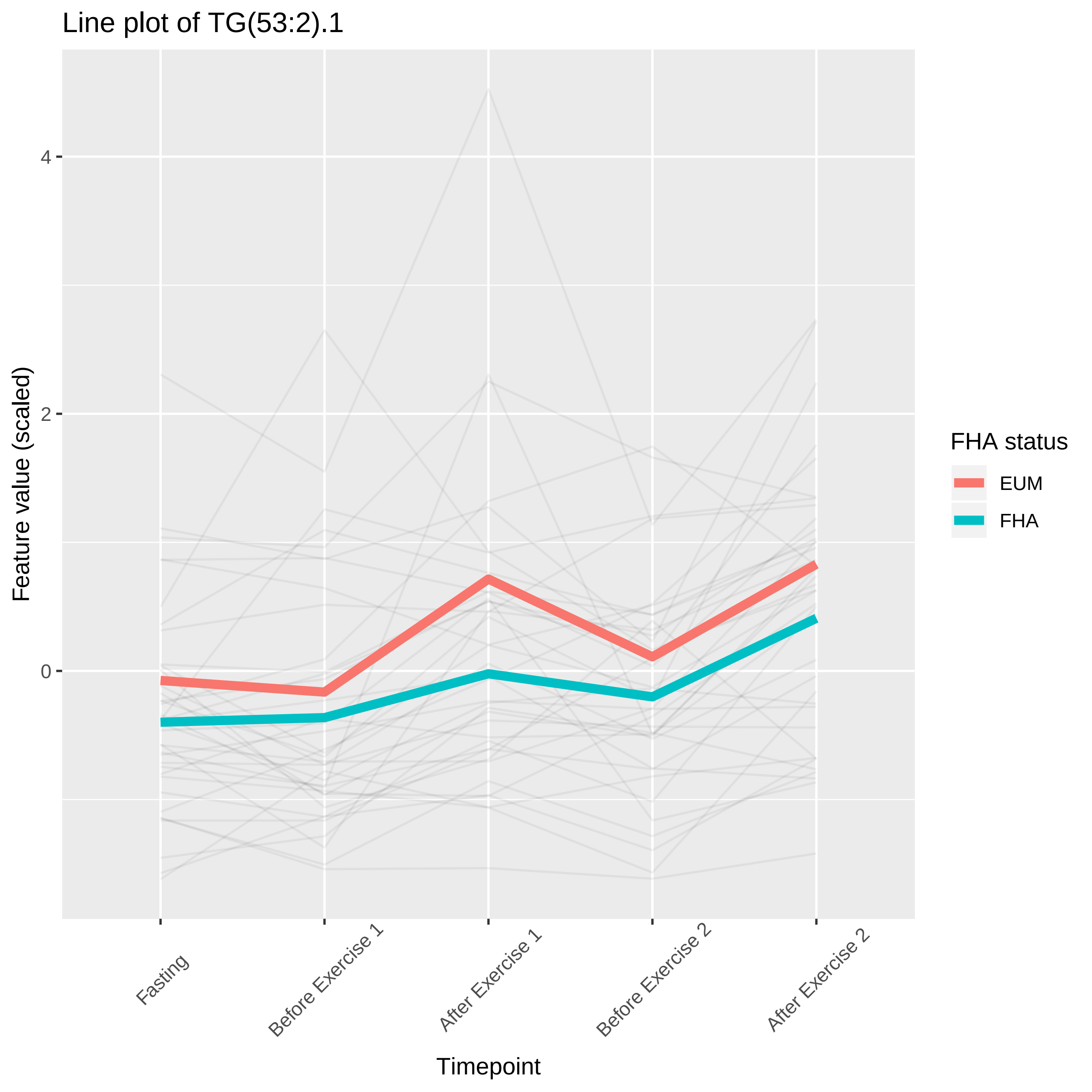

Line plot of TG(53:2).1
Feature value (scaled)
4
2
0
Fasting
Before Exercise 1
After Exercise 1
Before Exercise 2
After Exercise 2
Timepoint
FHA status
EUM
FHA

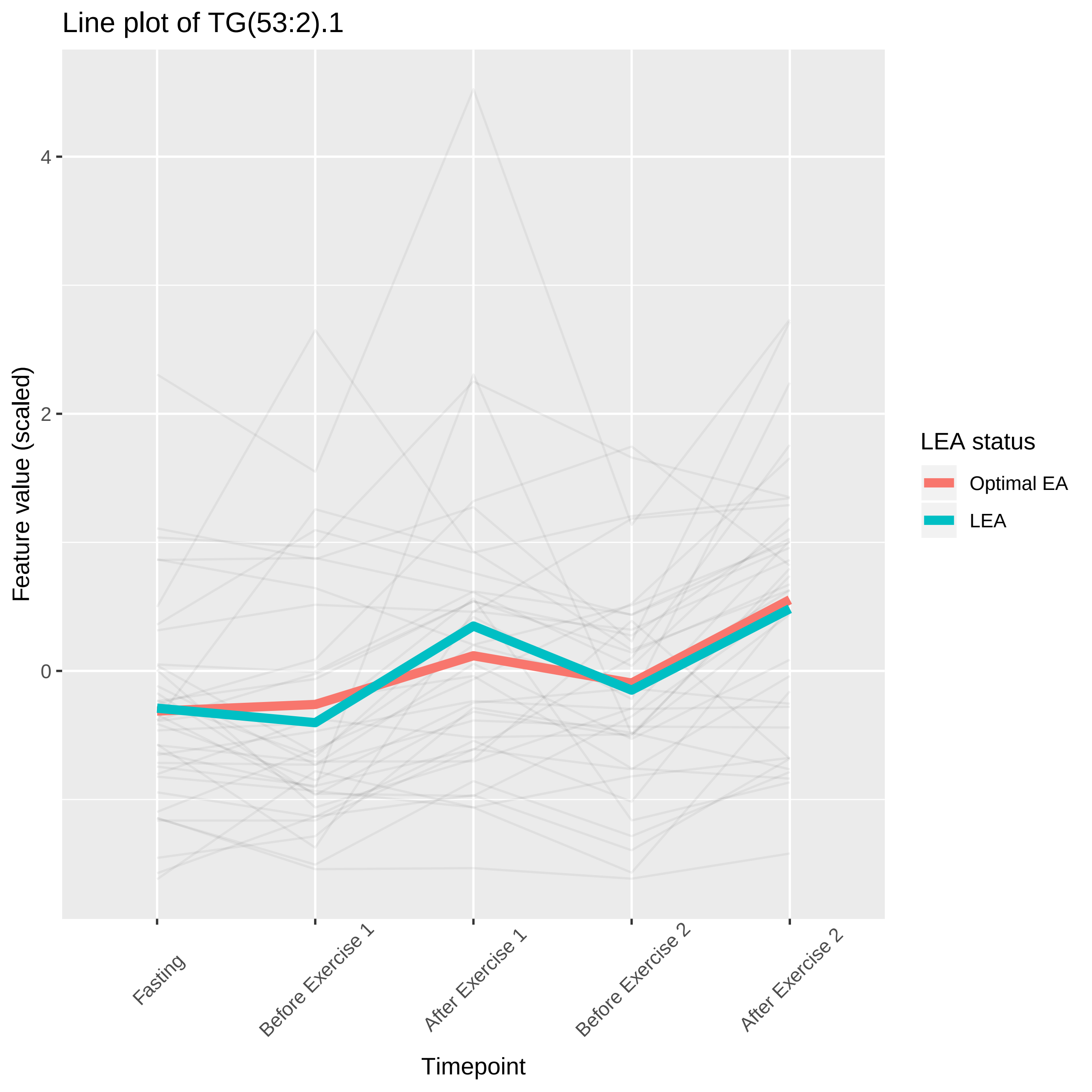

Line plot of TG(53:2).1
Feature value (scaled)
4
2
0
Fasting
Before Exercise 1
After Exercise 1
Before Exercise 2
After Exercise 2
Timepoint
LEA status
Optimal EA
LEA

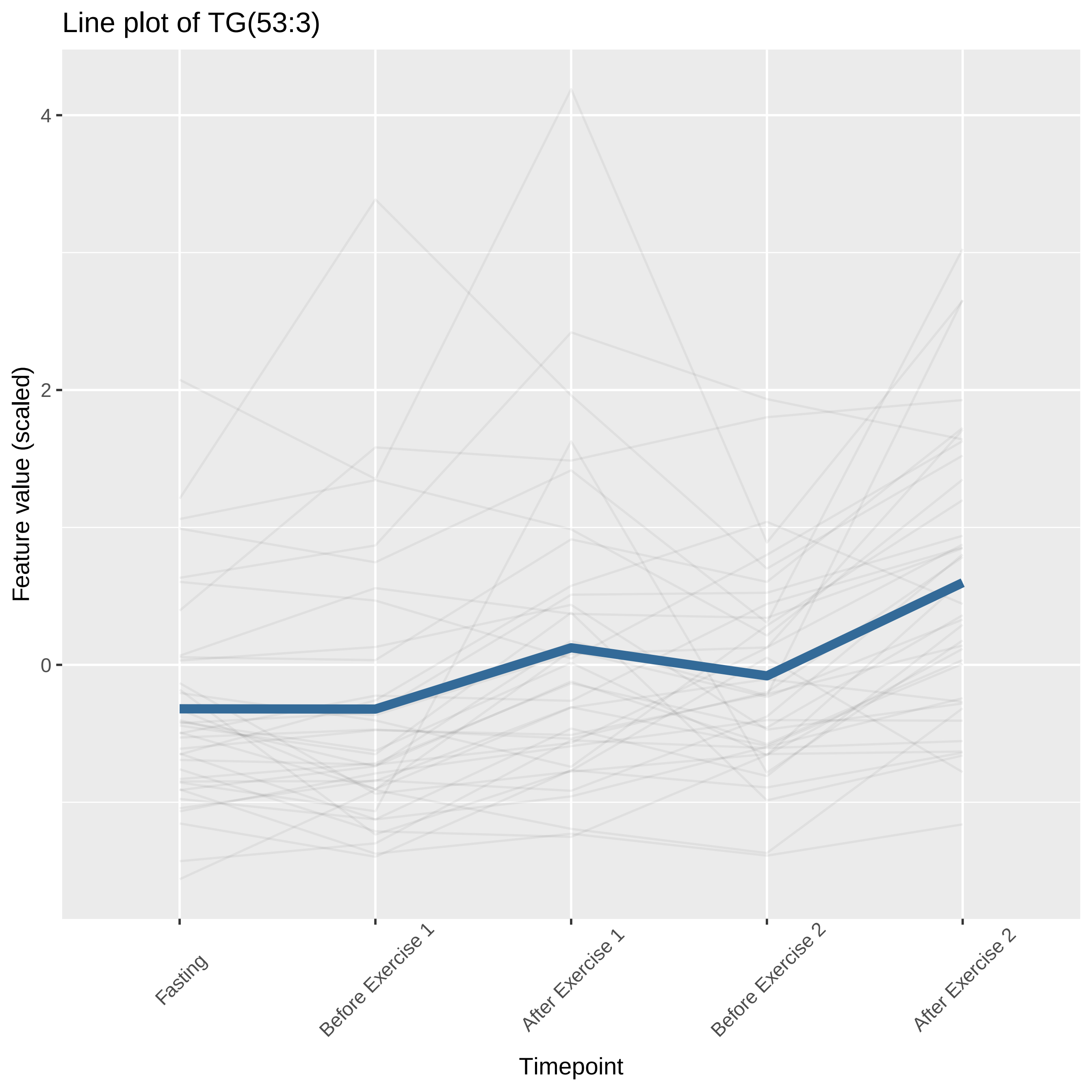

Line plot of TG(53:3)
Feature value (scaled)
4
2
0
Fasting
Before Exercise 1
After Exercise 1
Before Exercise 2
After Exercise 2
Timepoint

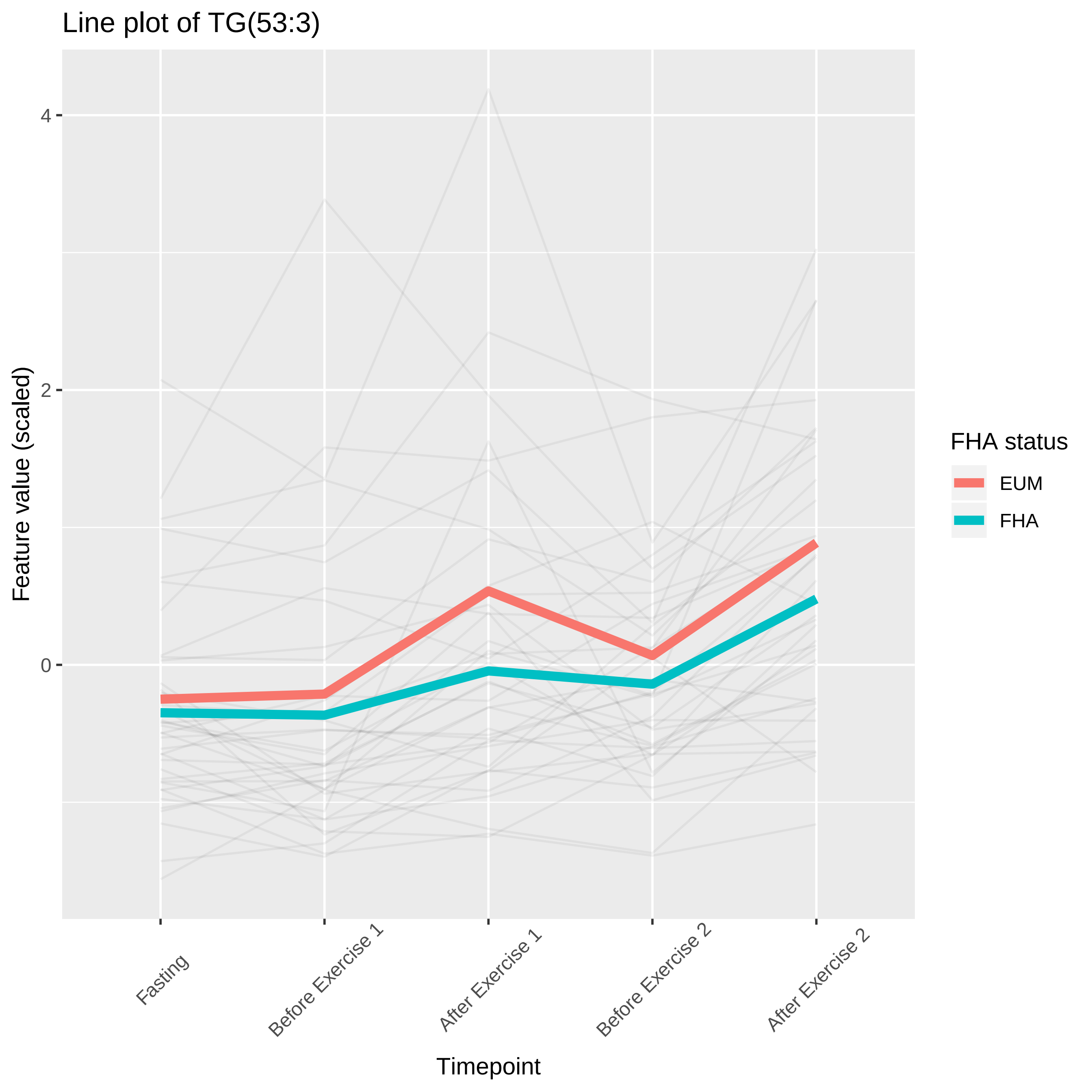

Line plot of TG(53:3)
Feature value (scaled)
4
2
0
Fasting
Before Exercise 1
After Exercise 1
Before Exercise 2
After Exercise 2
Timepoint
FHA status
EUM
FHA

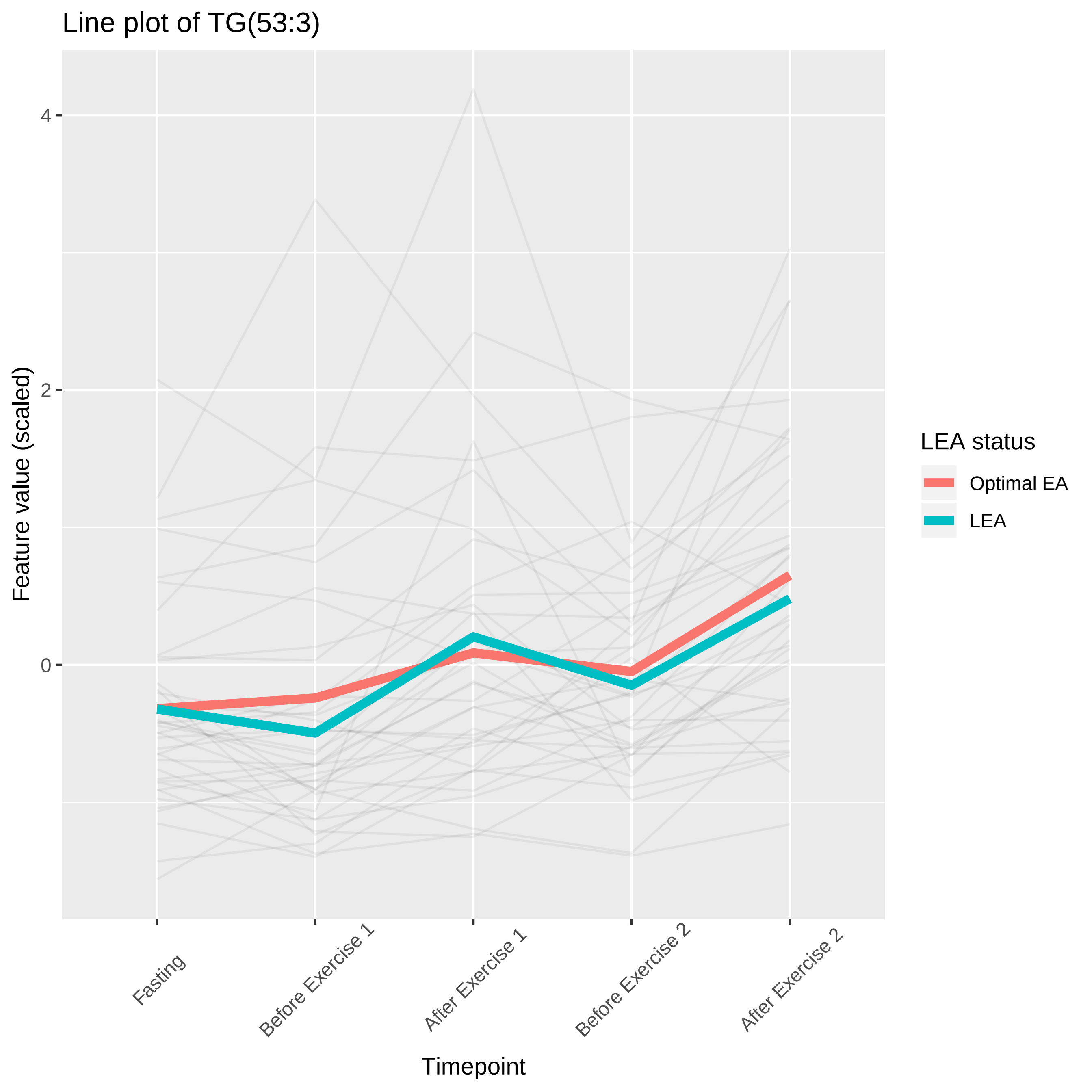

Line plot of TG(53:3)
Feature value (scaled)
4
2
0
Fasting
Before Exercise 1
After Exercise 1
Before Exercise 2
After Exercise 2
Timepoint
LEA status
Optimal EA
LEA

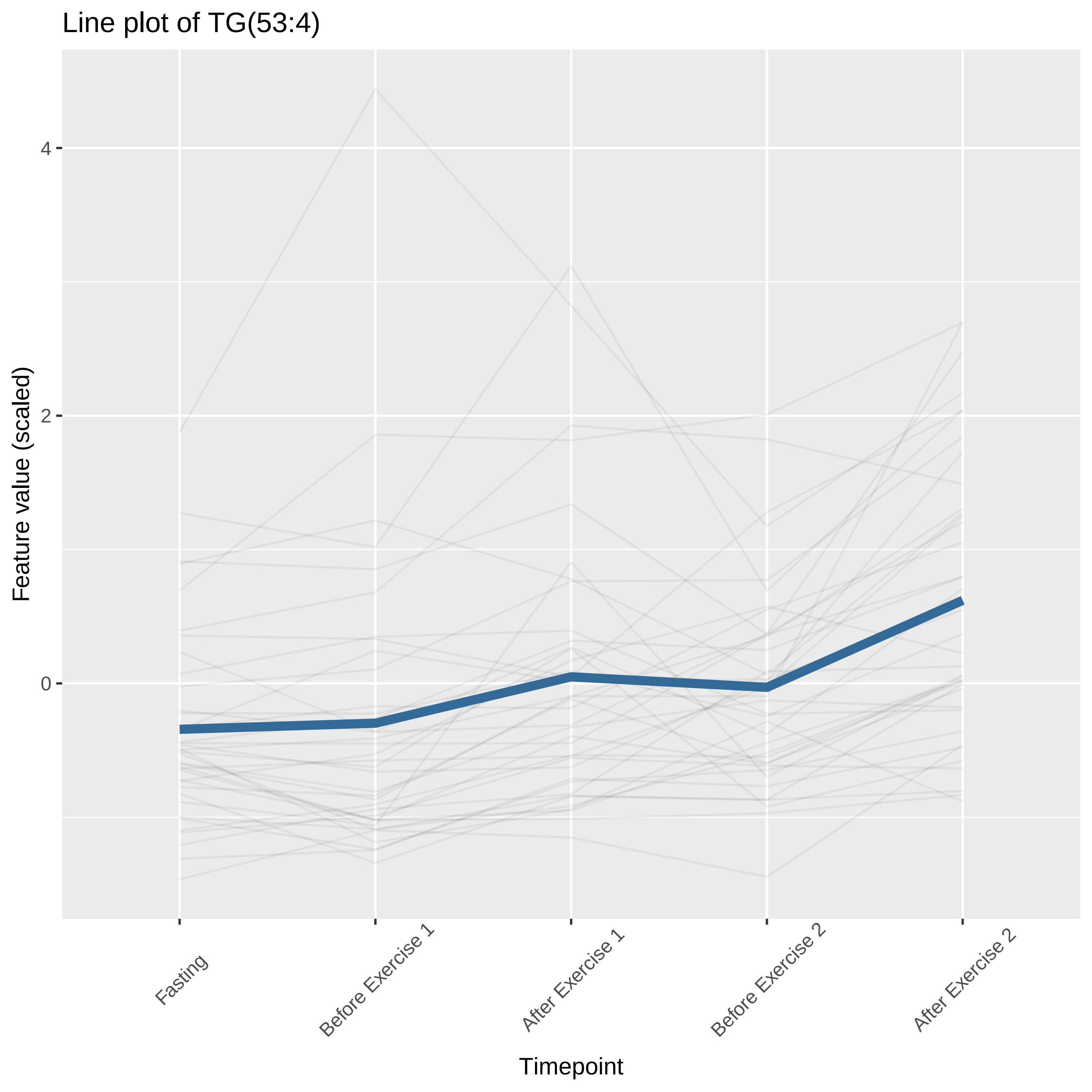

Line plot of TG(53:4)
Feature value (scaled)
4
2
0
Fasting
Before Exercise 1
After Exercise 1
Before Exercise 2
After Exercise 2
Timepoint

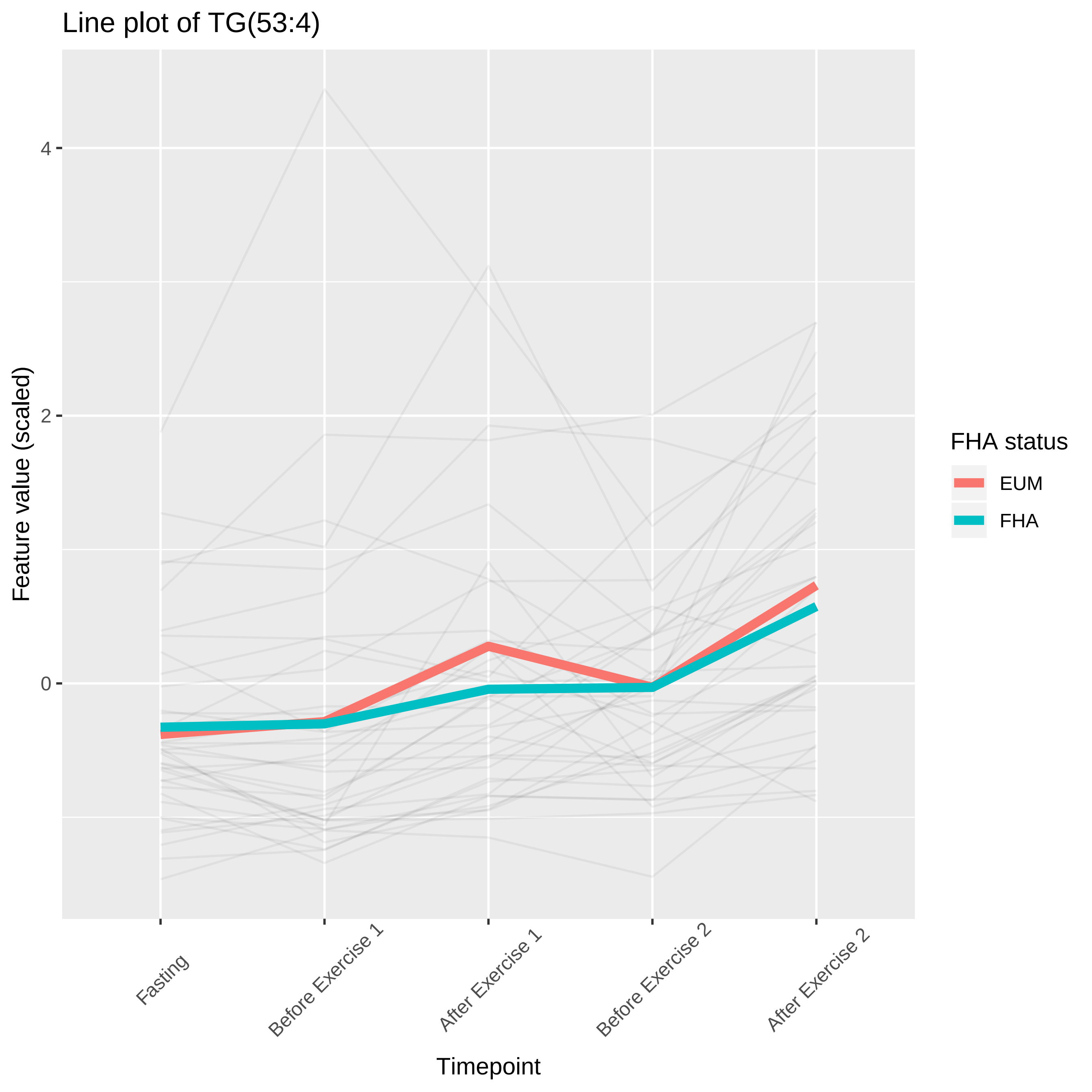

Line plot of TG(53:4)
Feature value (scaled)
4
2
0
Fasting
Before Exercise 1
After Exercise 1
Before Exercise 2
After Exercise 2
Timepoint
FHA status
EUM
FHA

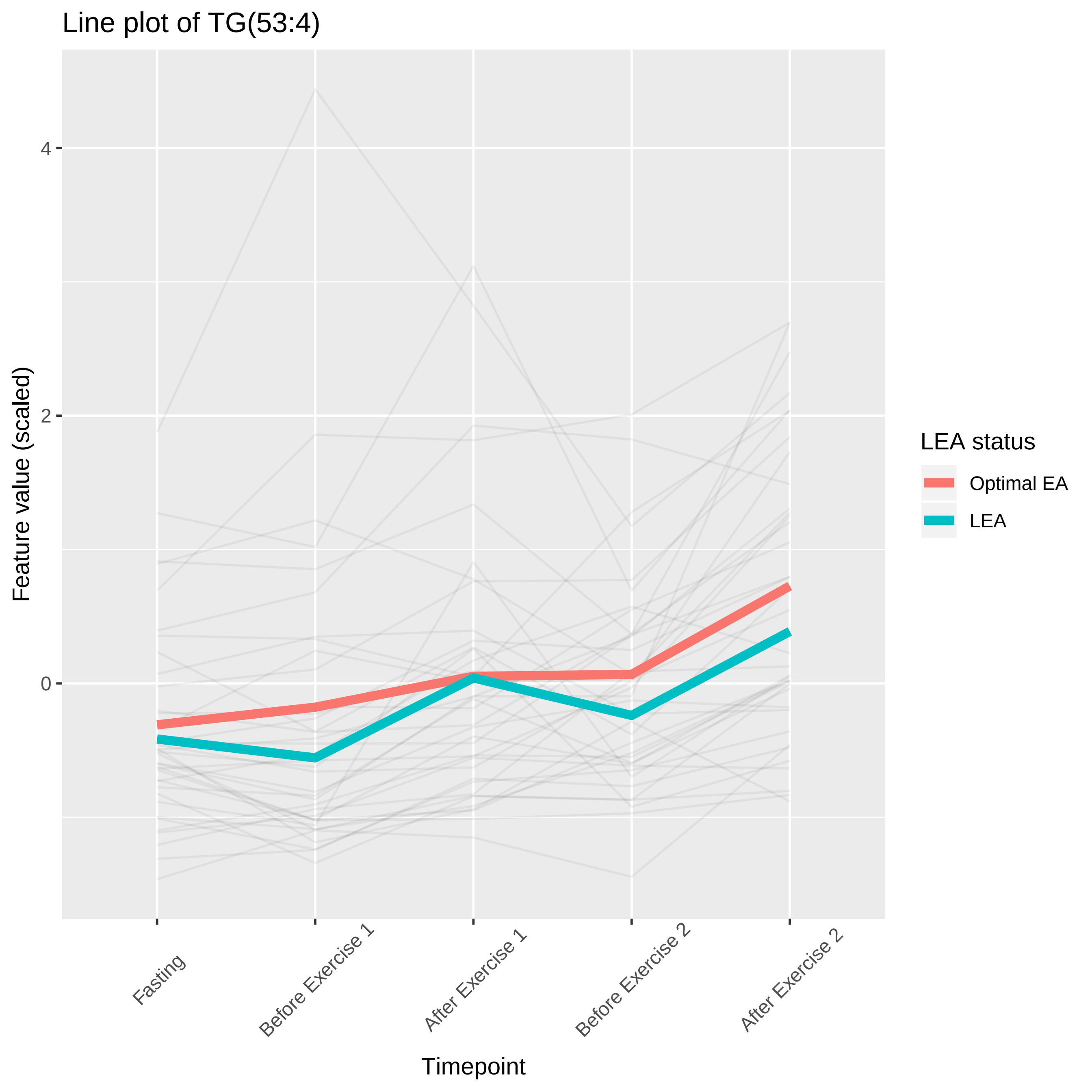
Line plot of TG(53:4)
Feature value (scaled)
4
2
0
Fasting
Before Exercise 1
After Exercise 1
Before Exercise 2
After Exercise 2
Timepoint
LEA status
Optimal EA
LEA

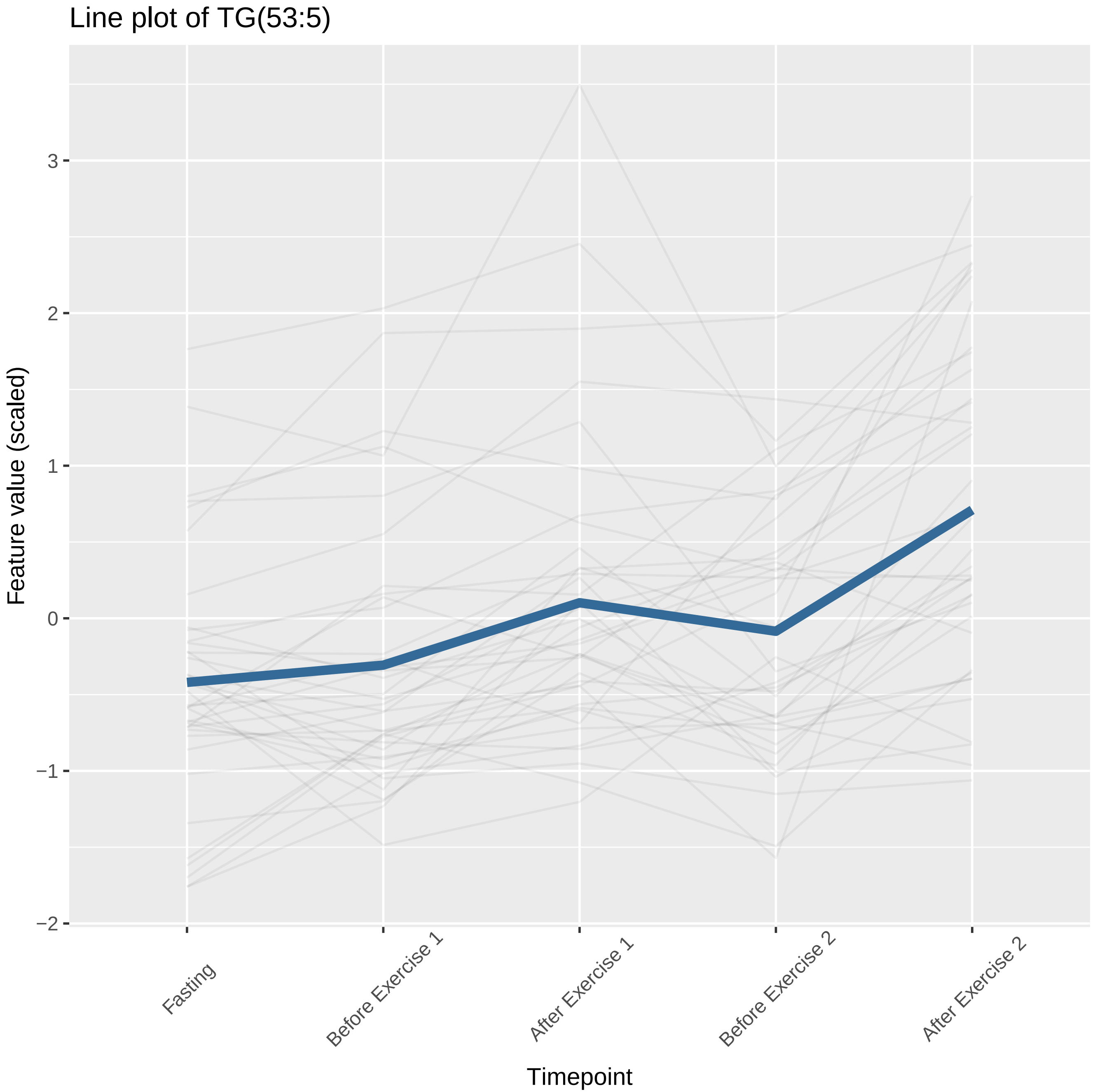

Line plot of TG(53:5)
Feature value (scaled)
3
2
1
0
−1
−2
Fasting
Before Exercise 1
After Exercise 1
Before Exercise 2
After Exercise 2
Timepoint

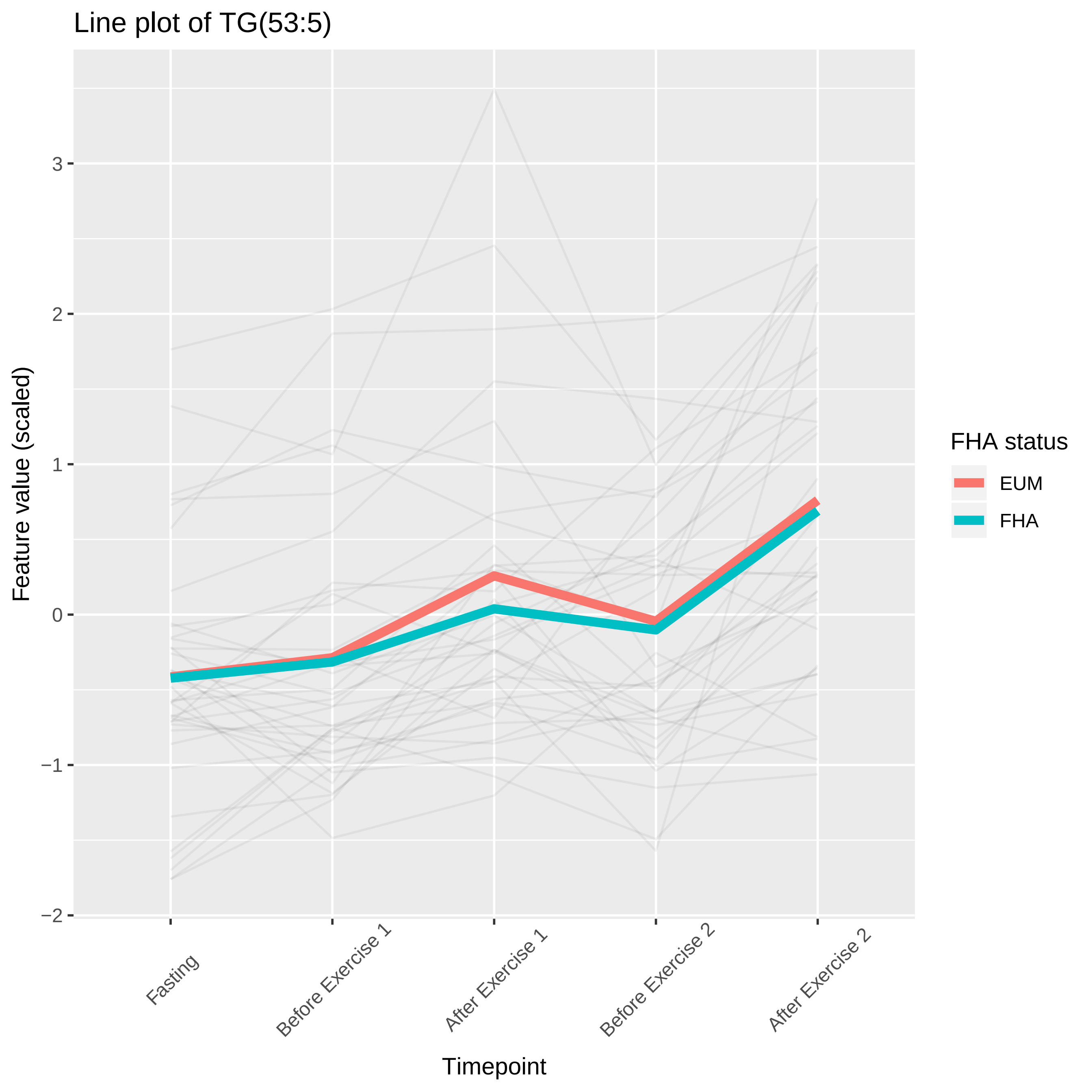

Line plot of TG(53:5)
Feature value (scaled)
3
2
1
0
−1
−2
Fasting
Before Exercise 1
After Exercise 1
Before Exercise 2
After Exercise 2
Timepoint
FHA status
EUM
FHA

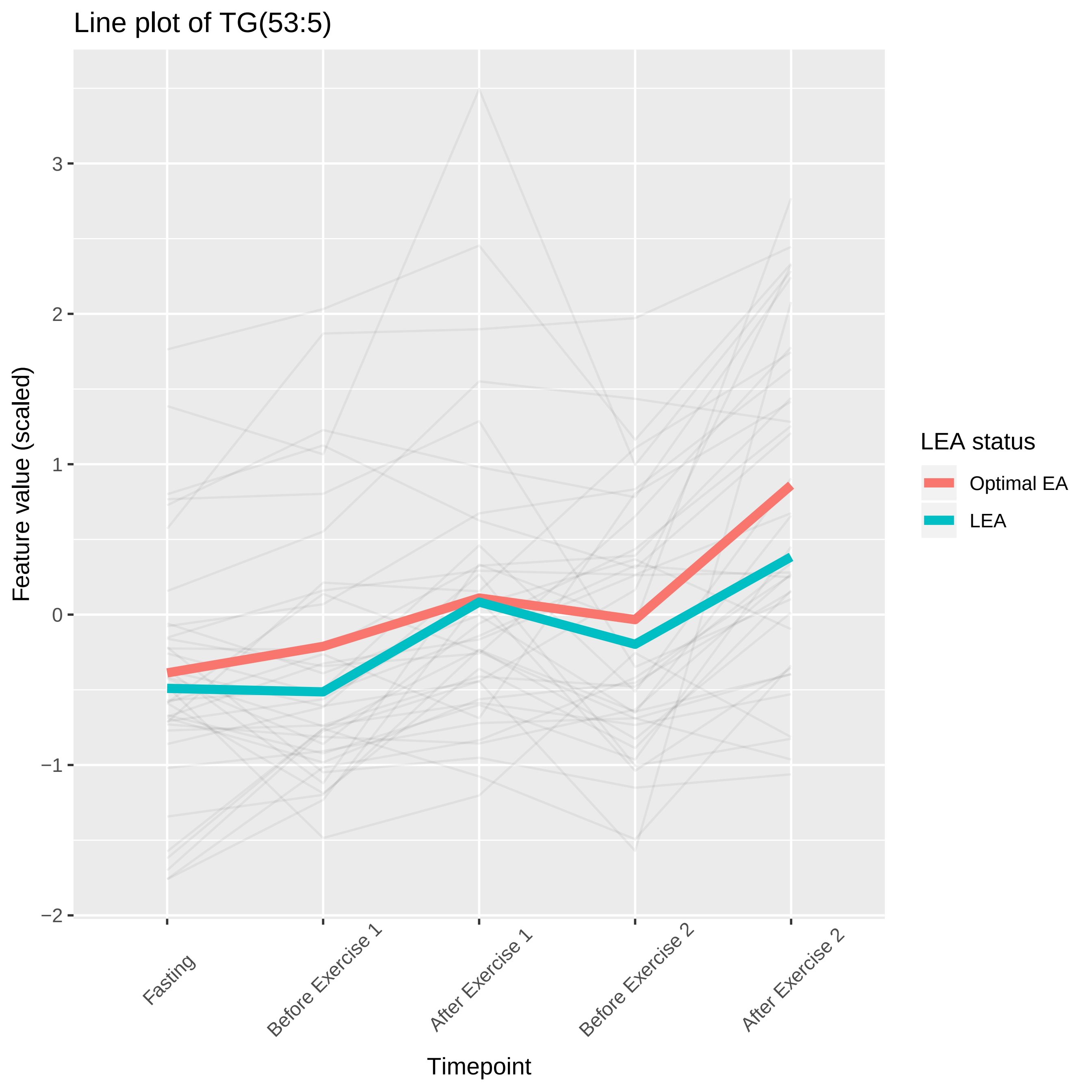
Line plot of TG(53:5)
Feature value (scaled)
3
2
1
0
−1
−2
Fasting
Before Exercise 1
After Exercise 1
Before Exercise 2
After Exercise 2
Timepoint
LEA status
Optimal EA
LEA

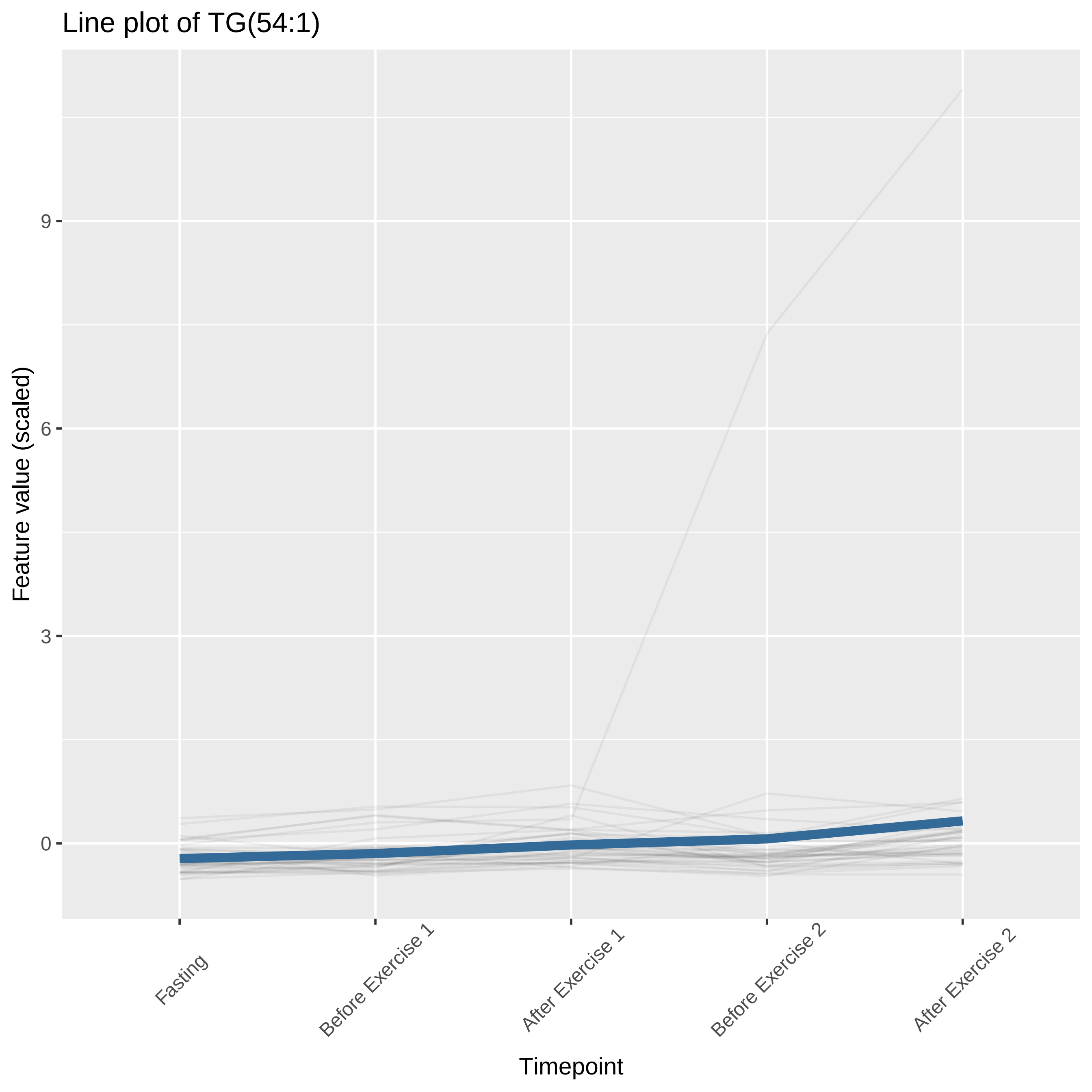

Line plot of TG(54:1)
Feature value (scaled)
9
6
3
0
Fasting
Before Exercise 1
After Exercise 1
Before Exercise 2
After Exercise 2
Timepoint

Line plot of TG(54:1)

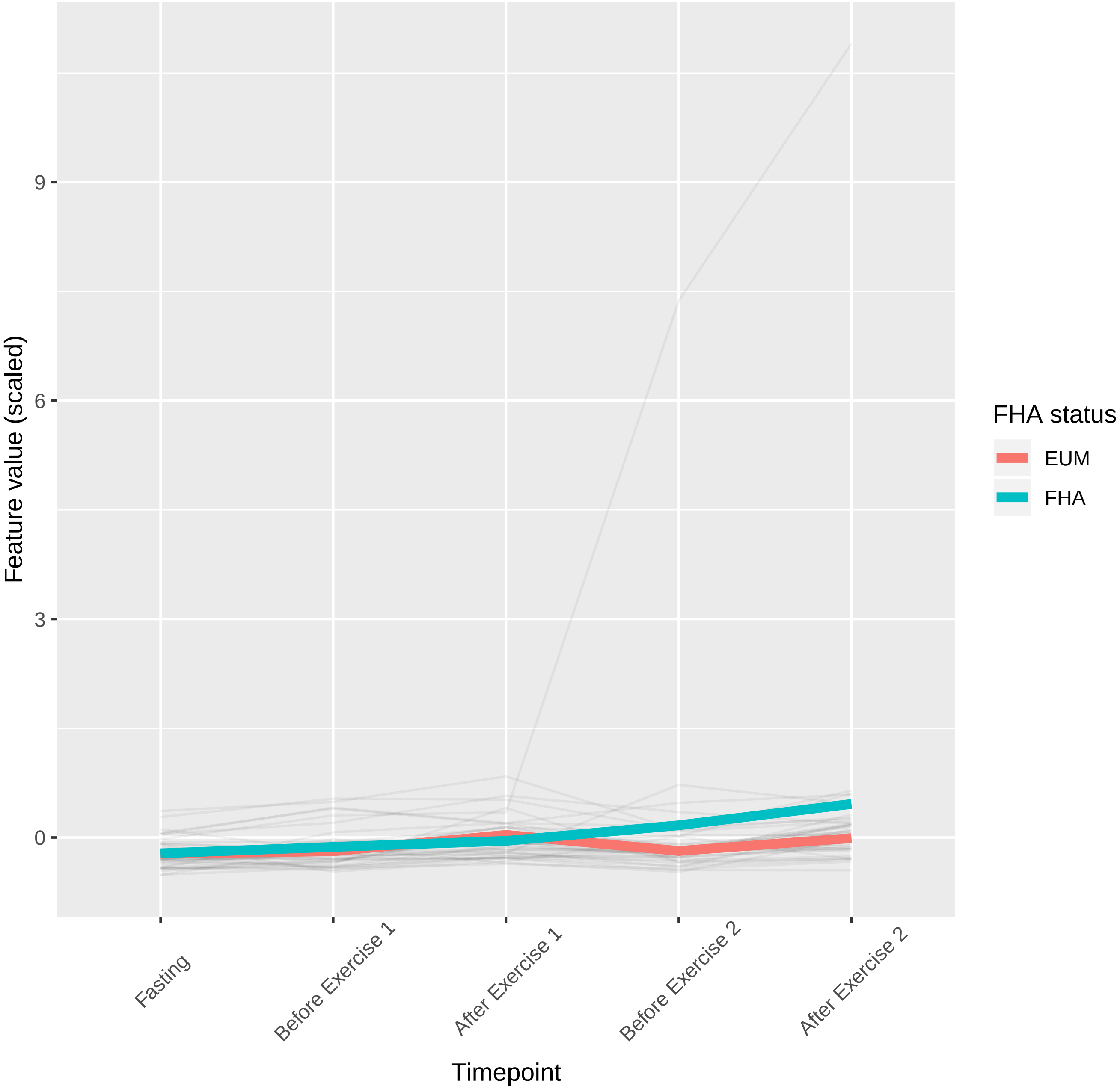

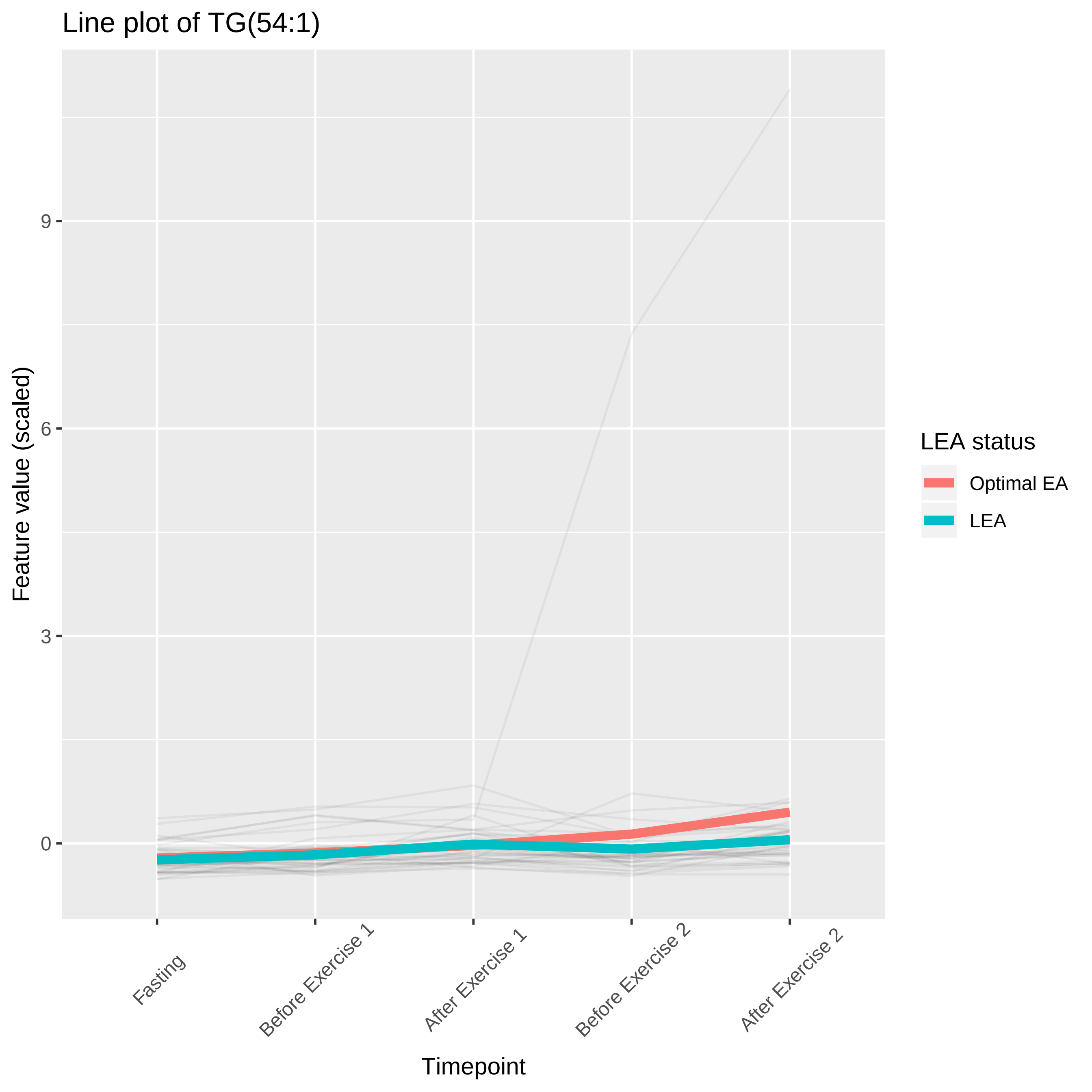

Line plot of TG(54:1)
Feature value (scaled)
9
6
3
0
Fasting
Before Exercise 1
After Exercise 1
Before Exercise 2
After Exercise 2
Timepoint
LEA status
Optimal EA
LEA

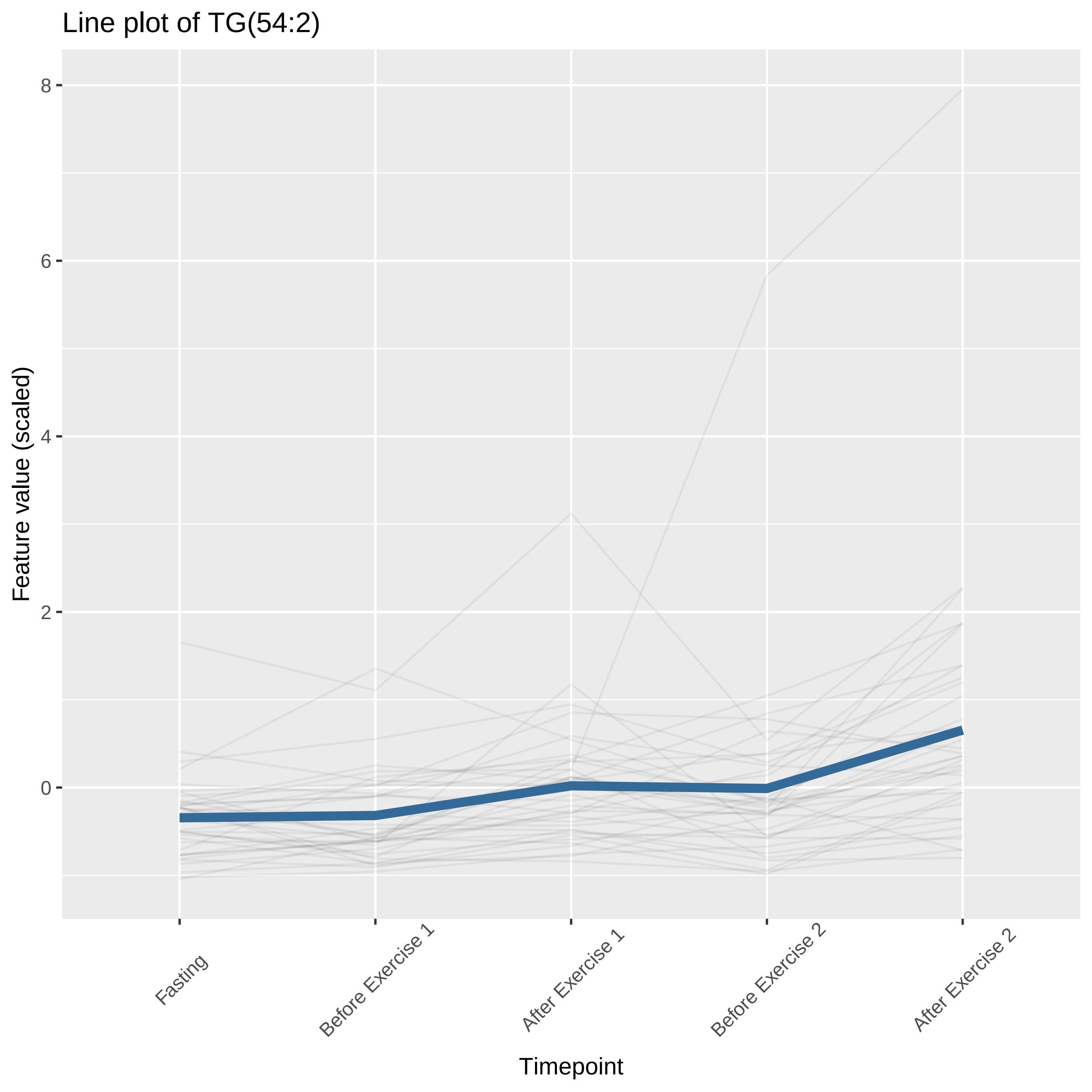

Line plot of TG(54:2)
Feature value (scaled)
8
6
4
2
0
Fasting
Before Exercise 1
After Exercise 1
Before Exercise 2
After Exercise 2
Timepoint

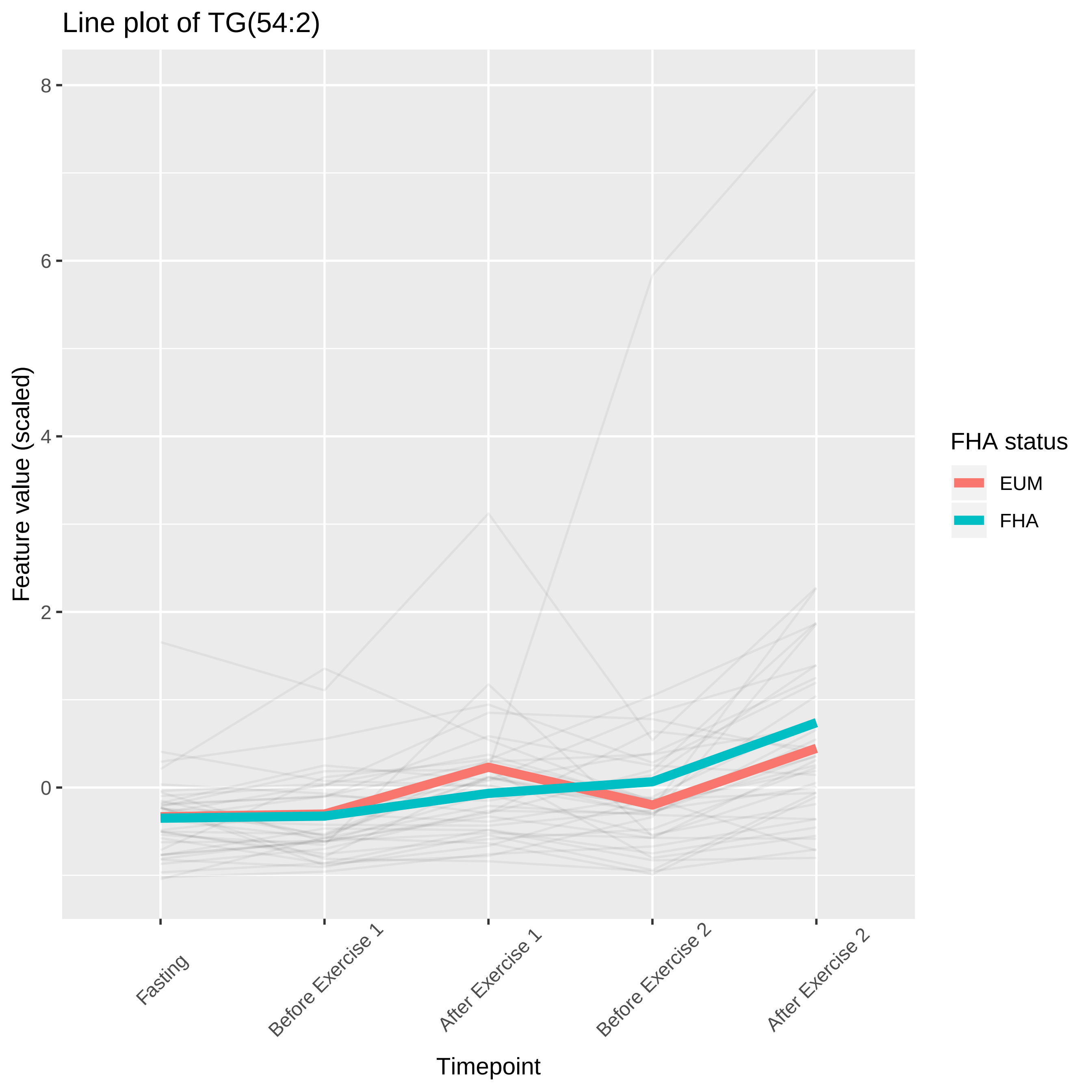
Line plot of TG(54:2)
Feature value (scaled)
8
6
4
2
0
Fasting
Before Exercise 1
After Exercise 1
Before Exercise 2
After Exercise 2
Timepoint
FHA status
EUM
FHA

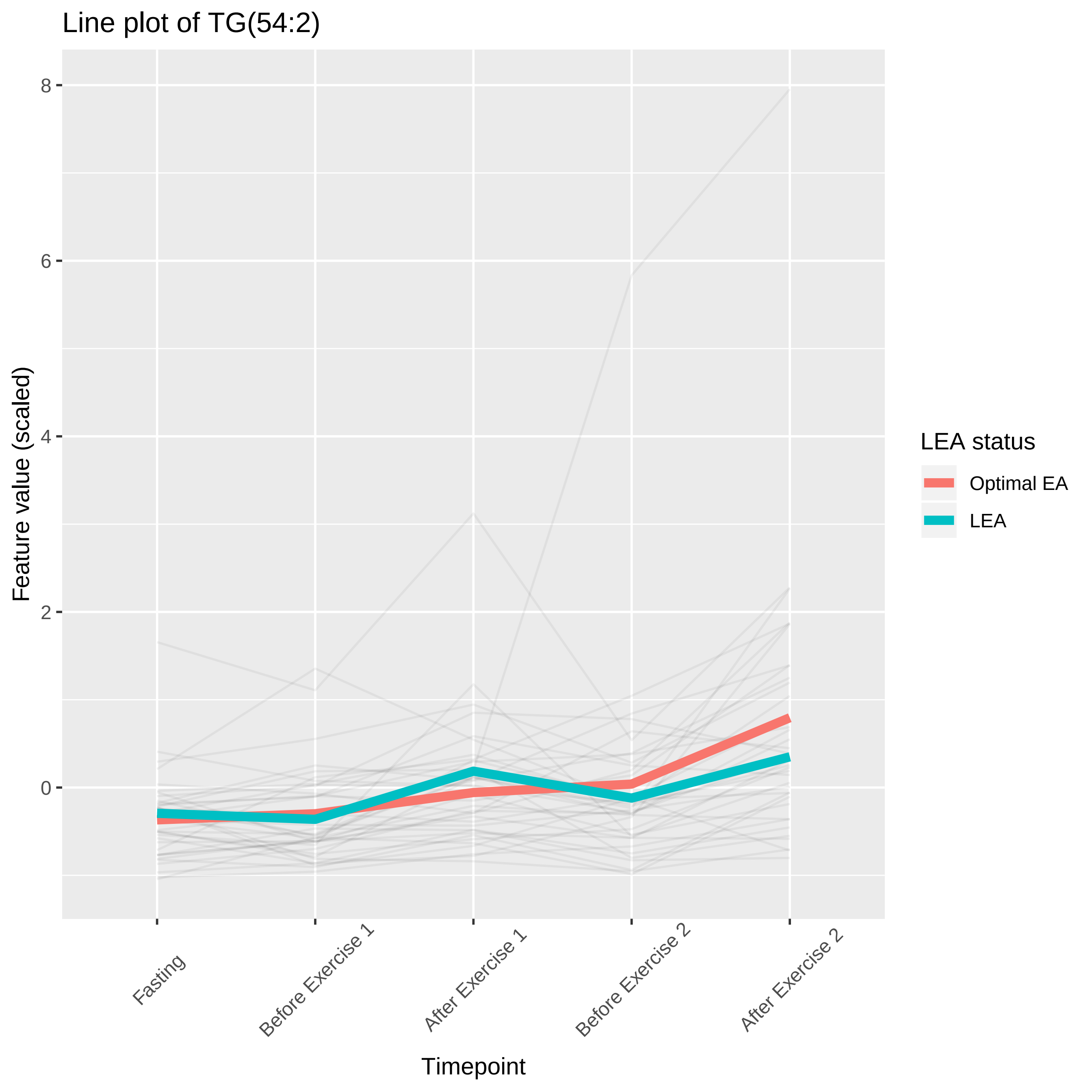
Line plot of TG(54:2)
Feature value (scaled)
8
6
4
2
0
Fasting
Before Exercise 1
After Exercise 1
Before Exercise 2
After Exercise 2
Timepoint
LEA status
Optimal EA
LEA

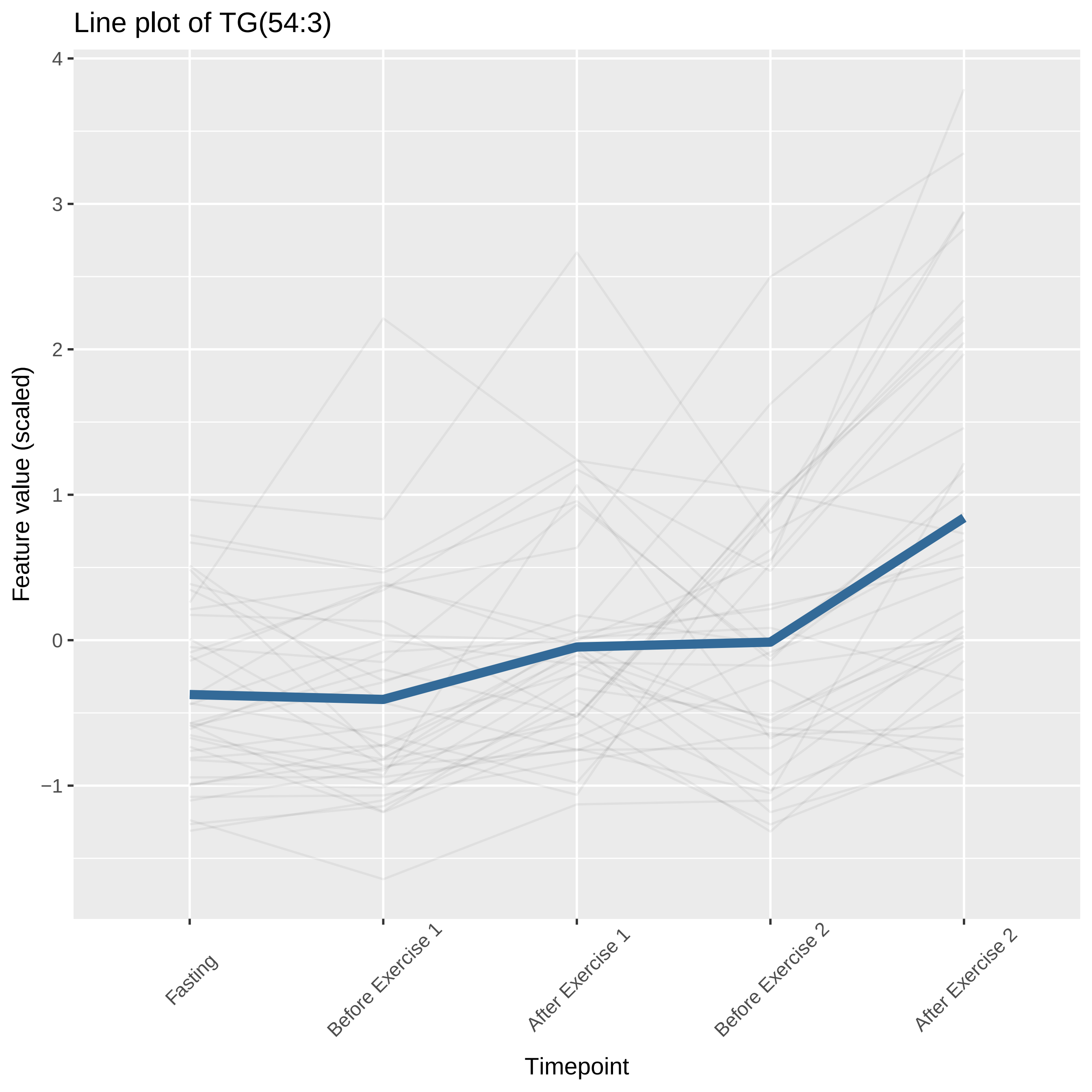

Line plot of TG(54:3)
Feature value (scaled)
4
3
2
1
0
−1
Fasting
Before Exercise 1
After Exercise 1
Before Exercise 2
After Exercise 2
Timepoint

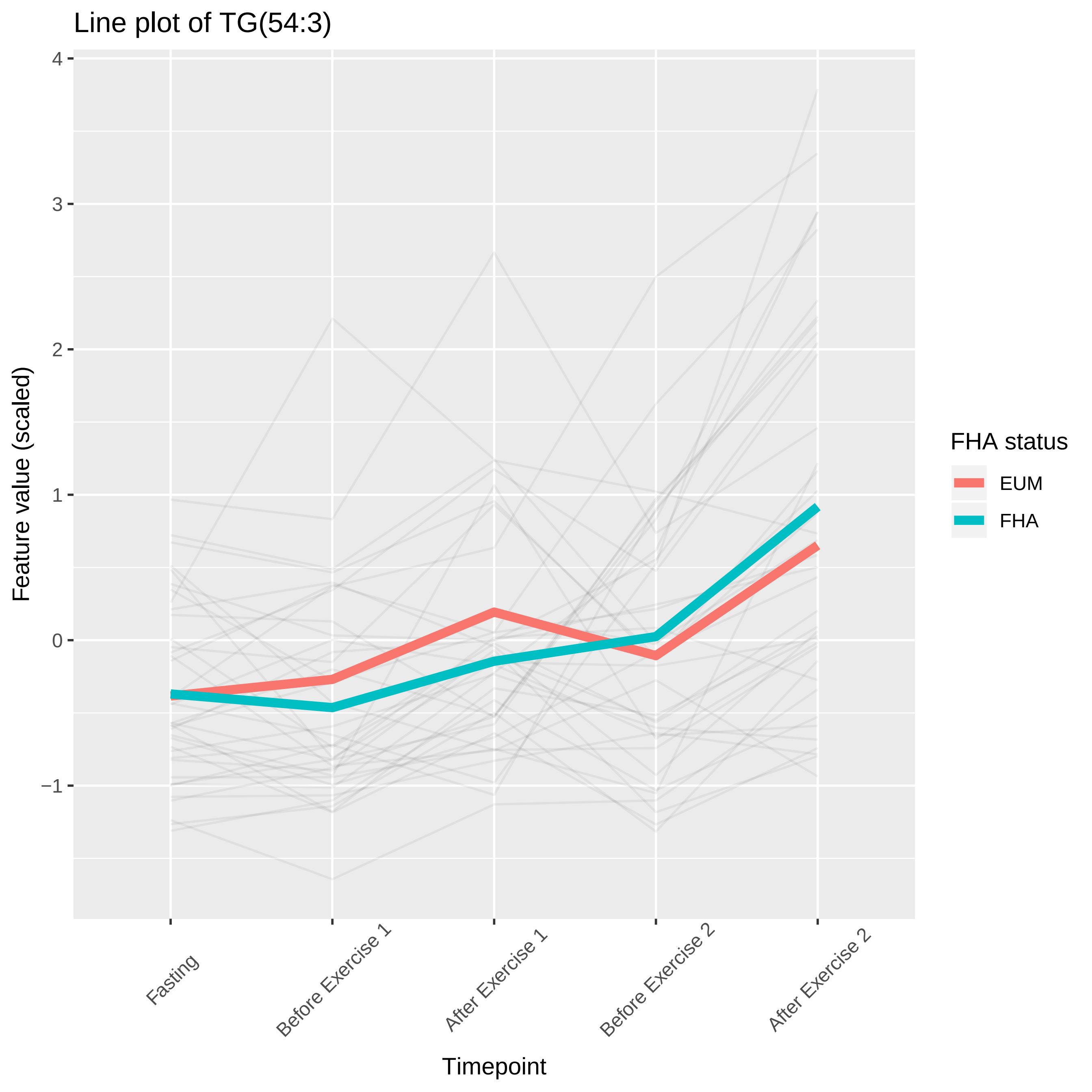

Line plot of TG(54:3)
Feature value (scaled)
4
3
2
1
0
−1
Fasting
Before Exercise 1
After Exercise 1
Before Exercise 2
After Exercise 2
Timepoint
FHA status
EUM
FHA

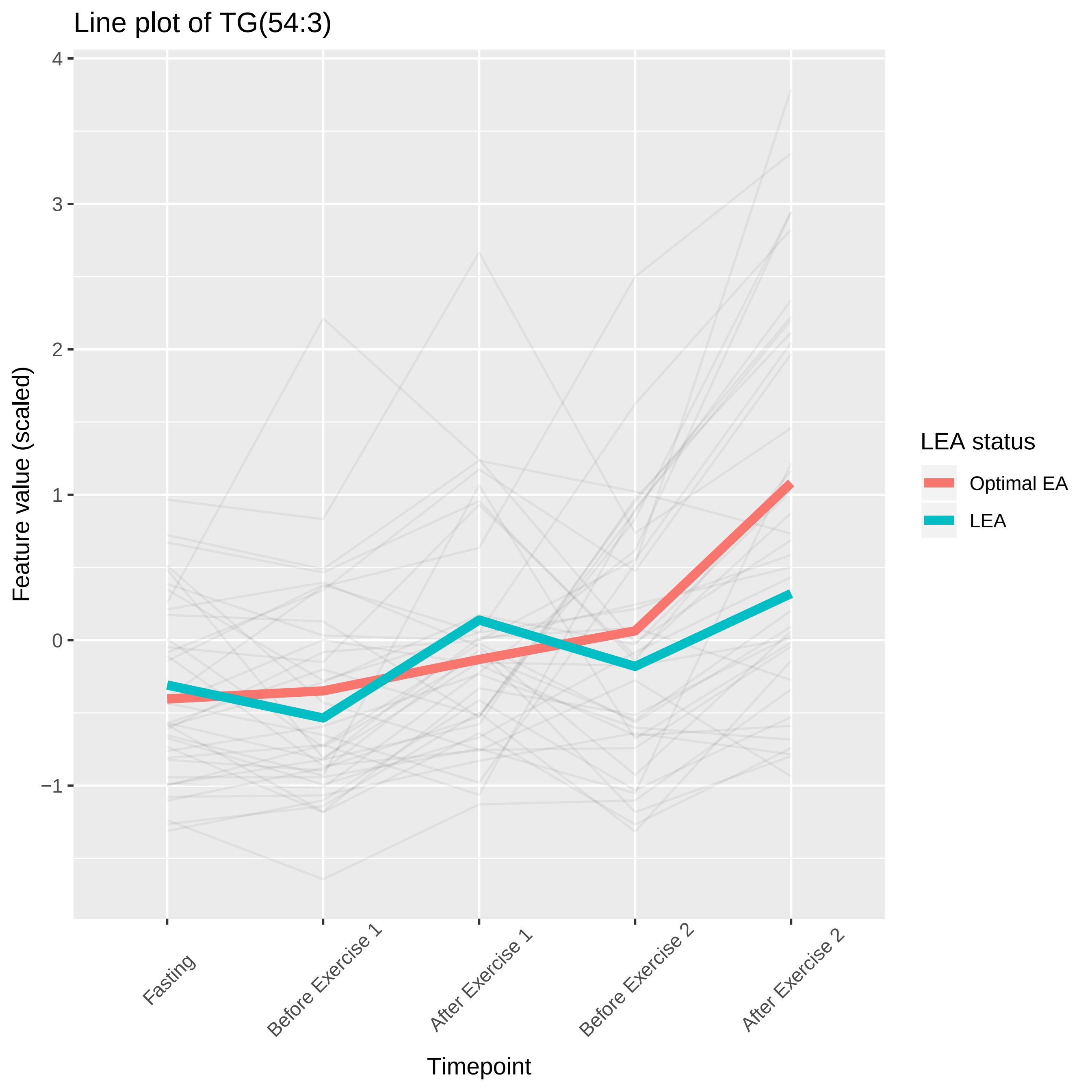

Line plot of TG(54:3)
Feature value (scaled)
4
3
2
1
0
−1
Fasting
Before Exercise 1
After Exercise 1
Before Exercise 2
After Exercise 2
Timepoint
LEA status
Optimal EA
LEA

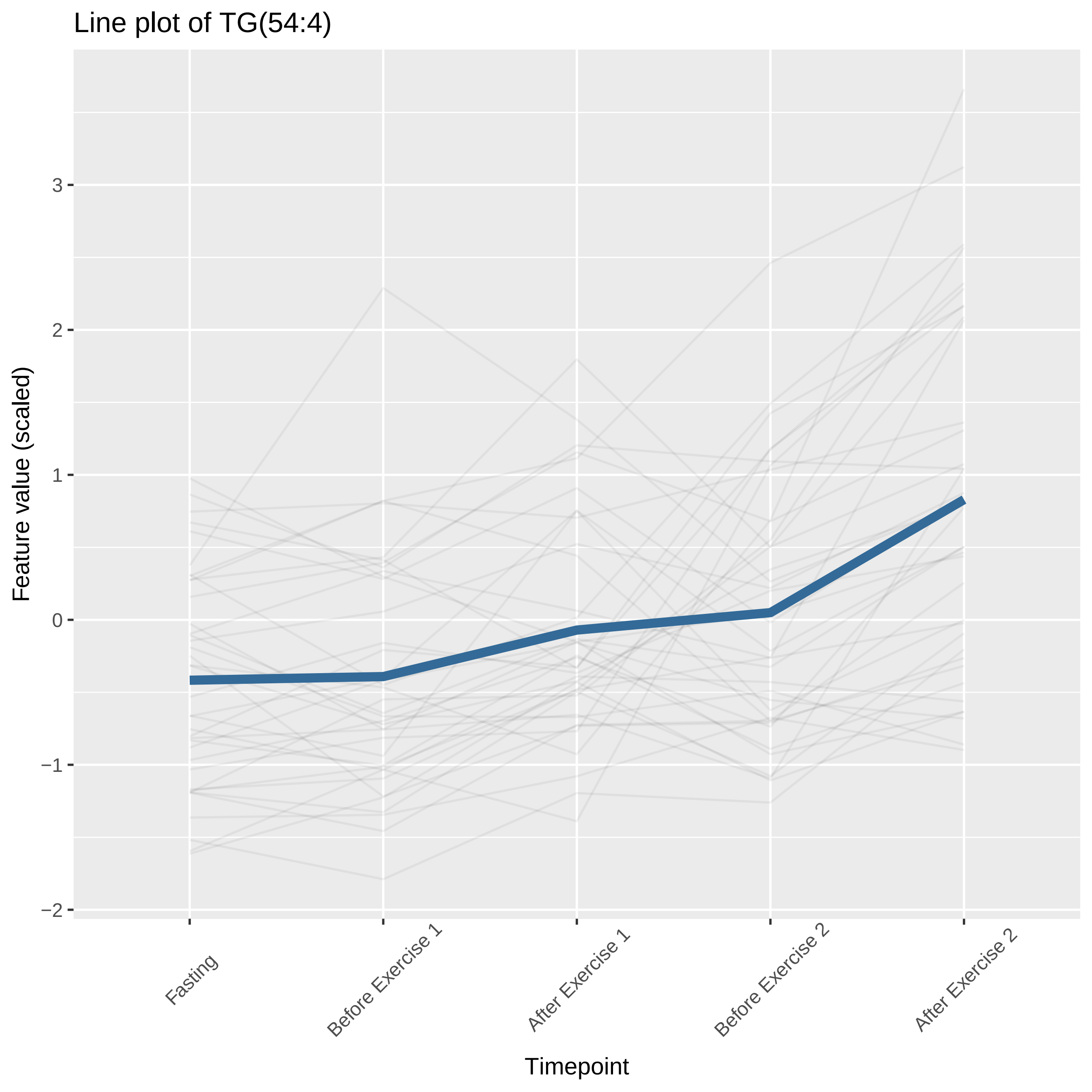
Line plot of TG(54:4)
Feature value (scaled)
3
2
1
0
−1
−2
Fasting
Before Exercise 1
After Exercise 1
Before Exercise 2
After Exercise 2
Timepoint

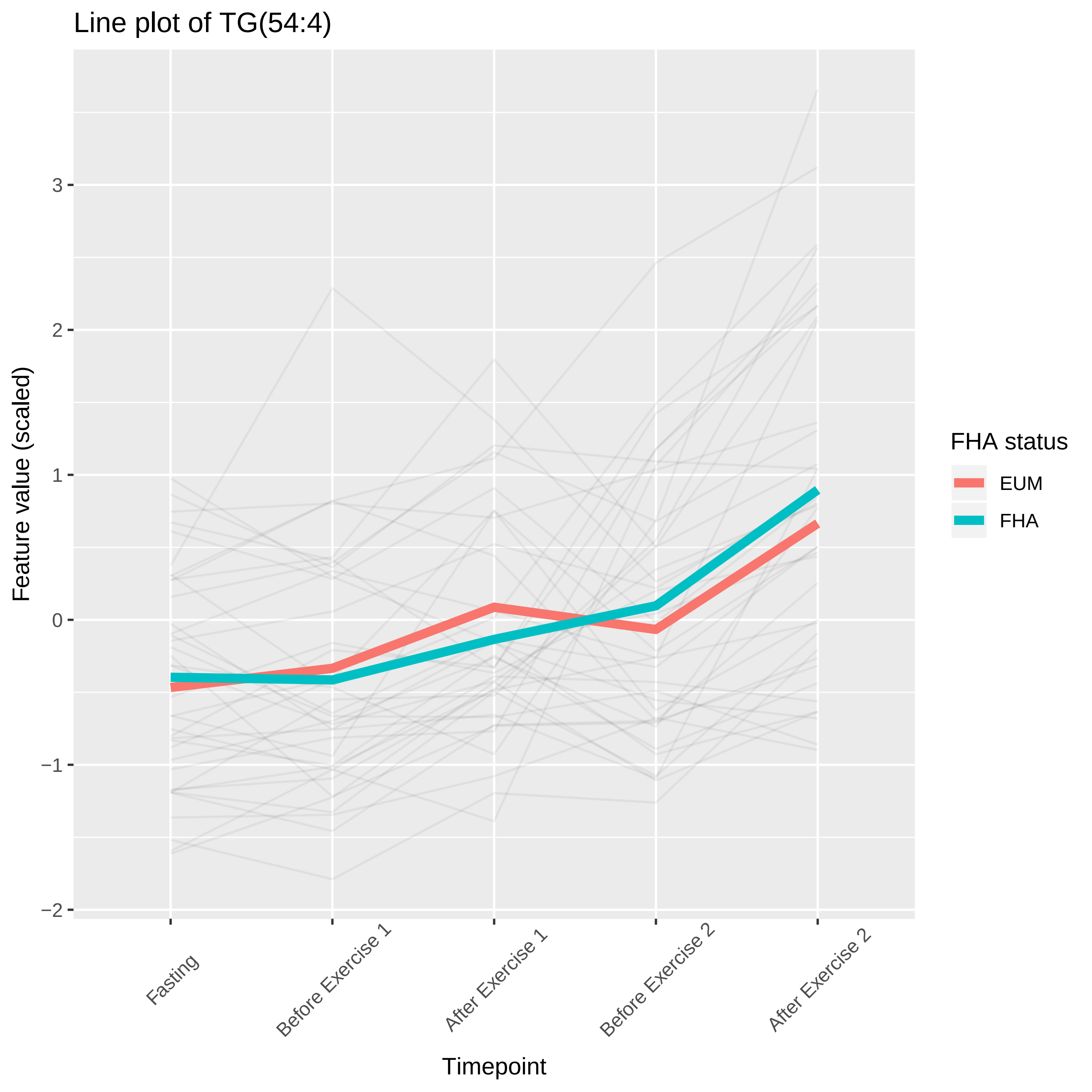
Line plot of TG(54:4)
Feature value (scaled)
3
2
1
0
−1
−2
Fasting
Before Exercise 1
After Exercise 1
Before Exercise 2
After Exercise 2
Timepoint
FHA status
EUM
FHA

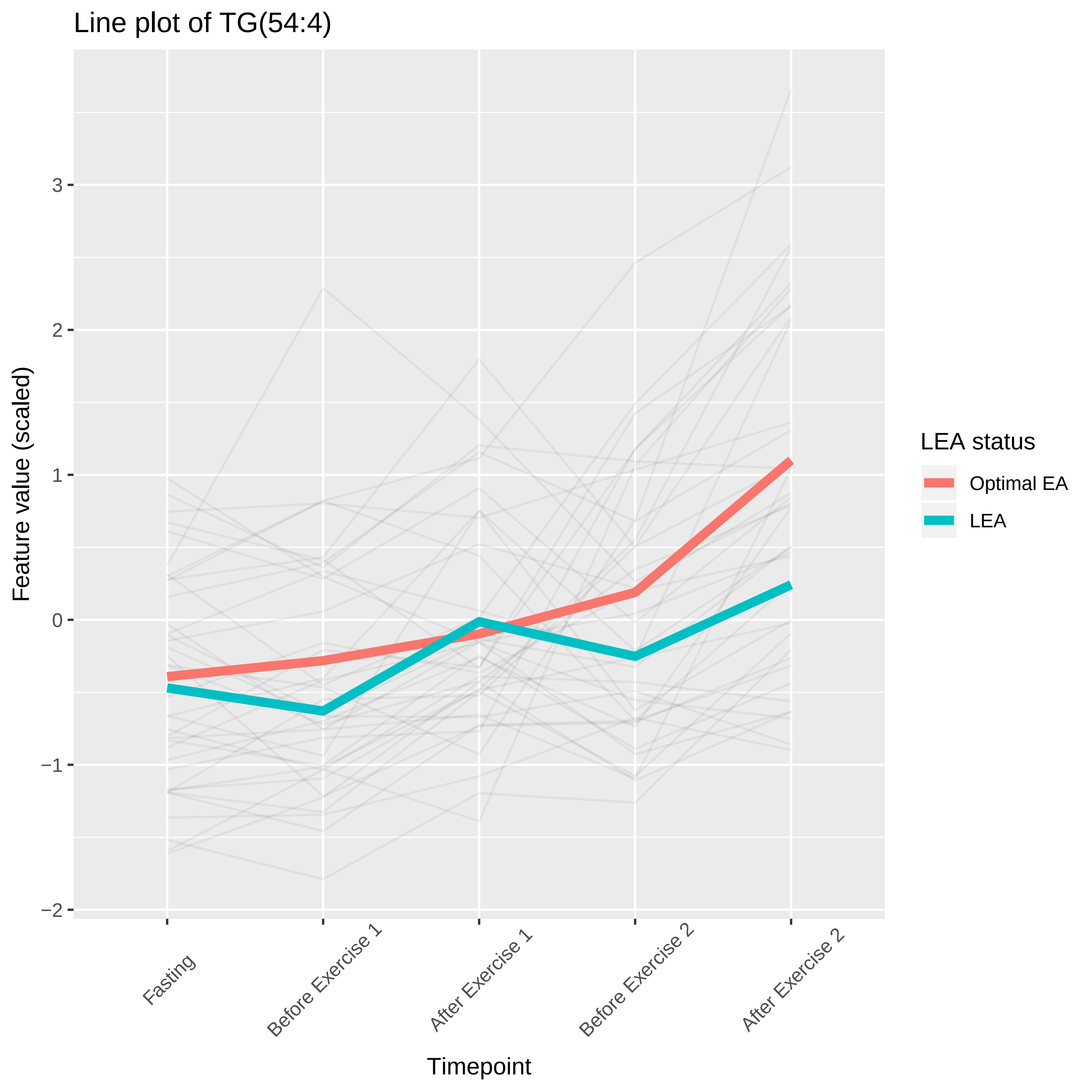

Line plot of TG(54:4)
Feature value (scaled)
3
2
1
0
−1
−2
Fasting
Before Exercise 1
After Exercise 1
Before Exercise 2
After Exercise 2
Timepoint
LEA status
Optimal EA
LEA

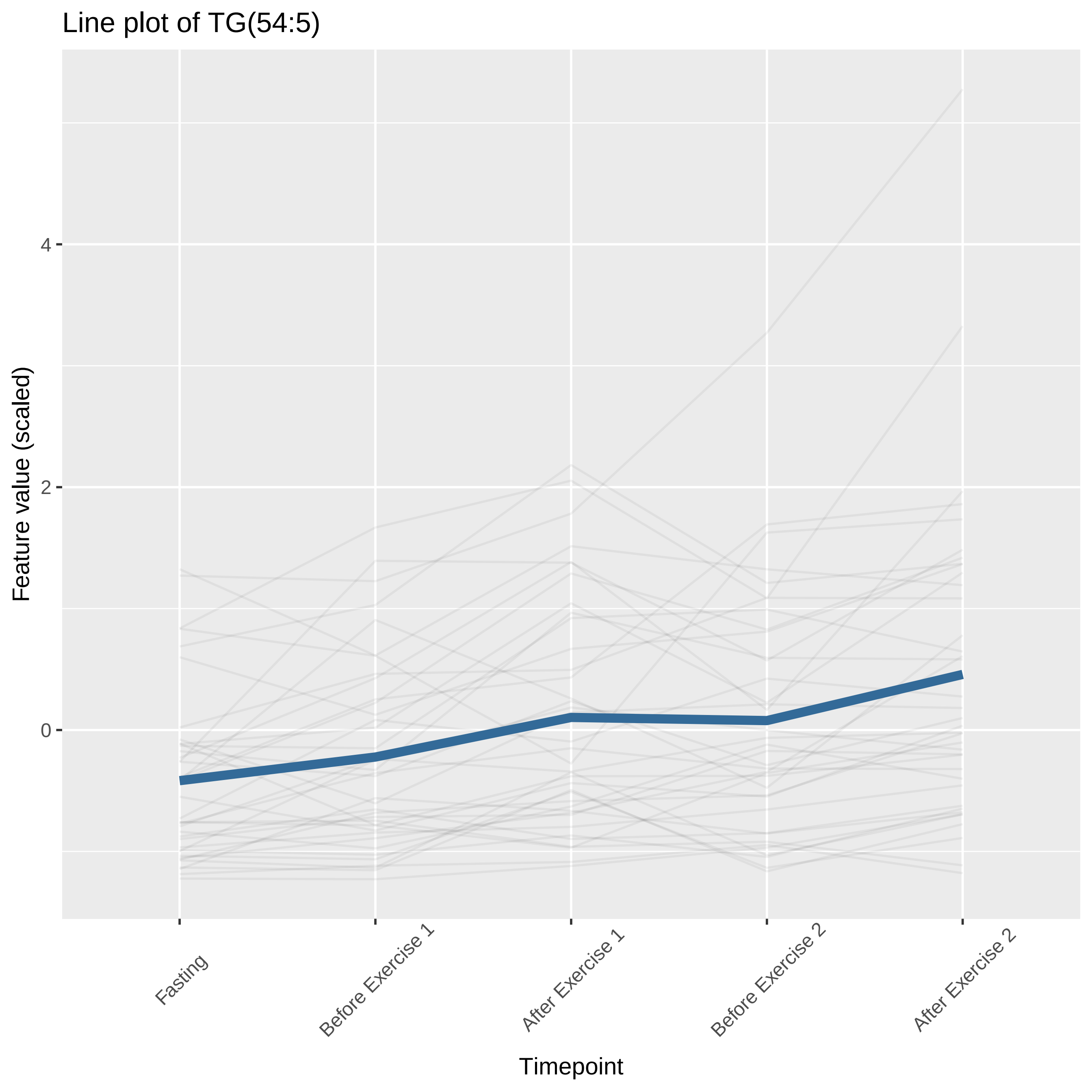

Line plot of TG(54:5)
Feature value (scaled)
4
2
0
Fasting
Before Exercise 1
After Exercise 1
Before Exercise 2
After Exercise 2
Timepoint

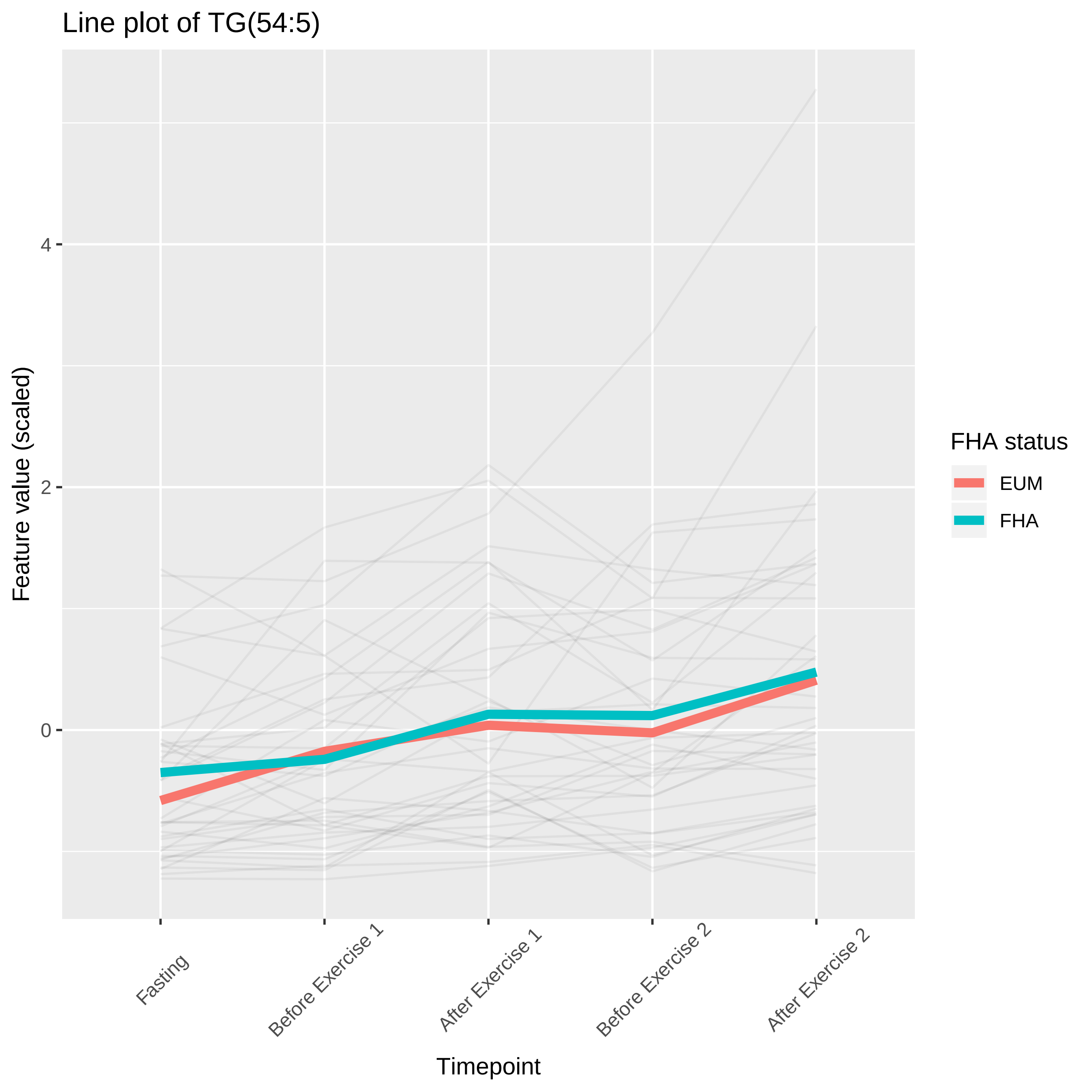

Line plot of TG(54:5)
Feature value (scaled)
4
2
0
Fasting
Before Exercise 1
After Exercise 1
Before Exercise 2
After Exercise 2
Timepoint
FHA status
EUM
FHA

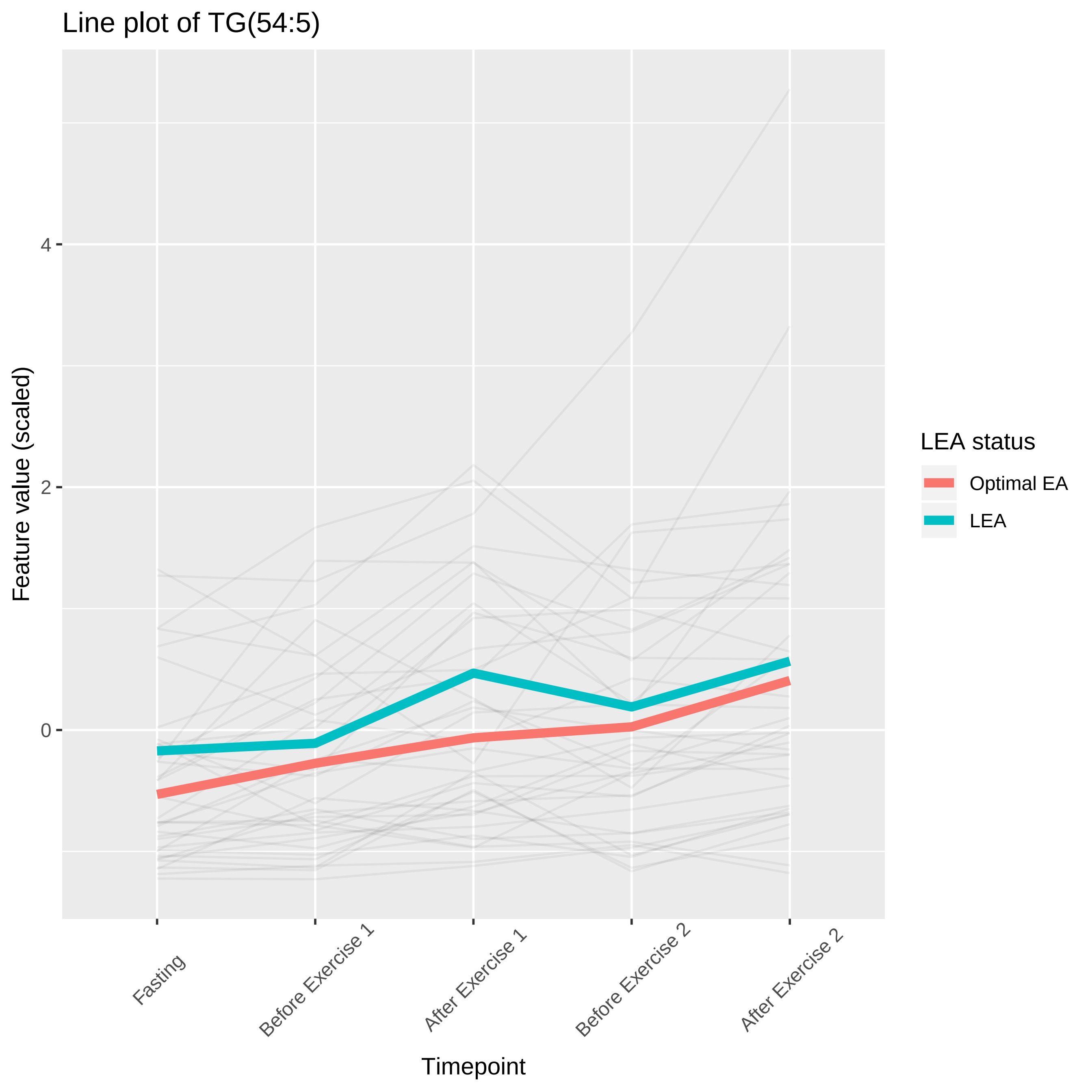

Line plot of TG(54:5)
Feature value (scaled)
4
2
0
Fasting
Before Exercise 1
After Exercise 1
Before Exercise 2
After Exercise 2
Timepoint
LEA status
Optimal EA
LEA

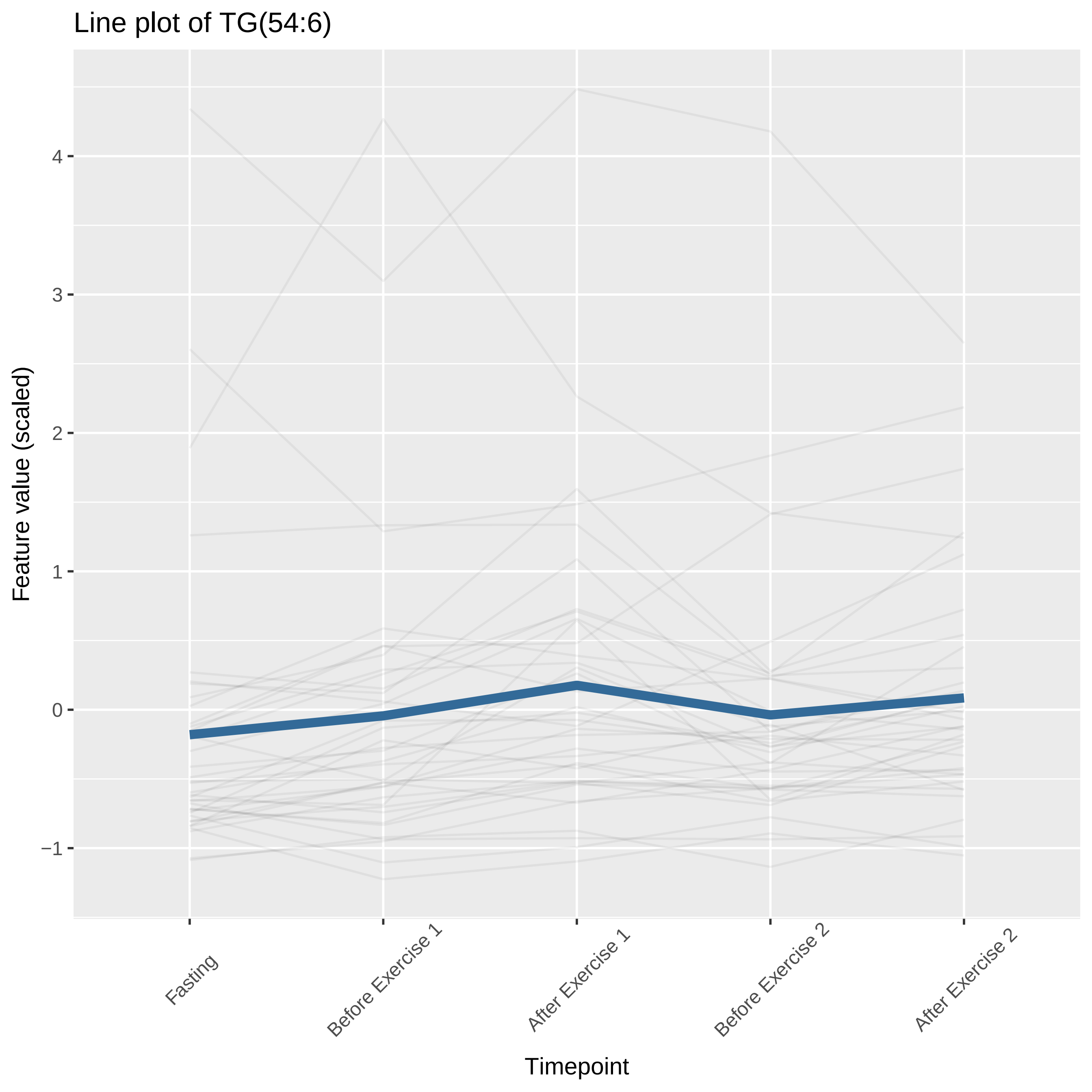

Line plot of TG(54:6)
Feature value (scaled)
4
3
2
1
0
−1
Fasting
Before Exercise 1
After Exercise 1
Before Exercise 2
After Exercise 2
Timepoint

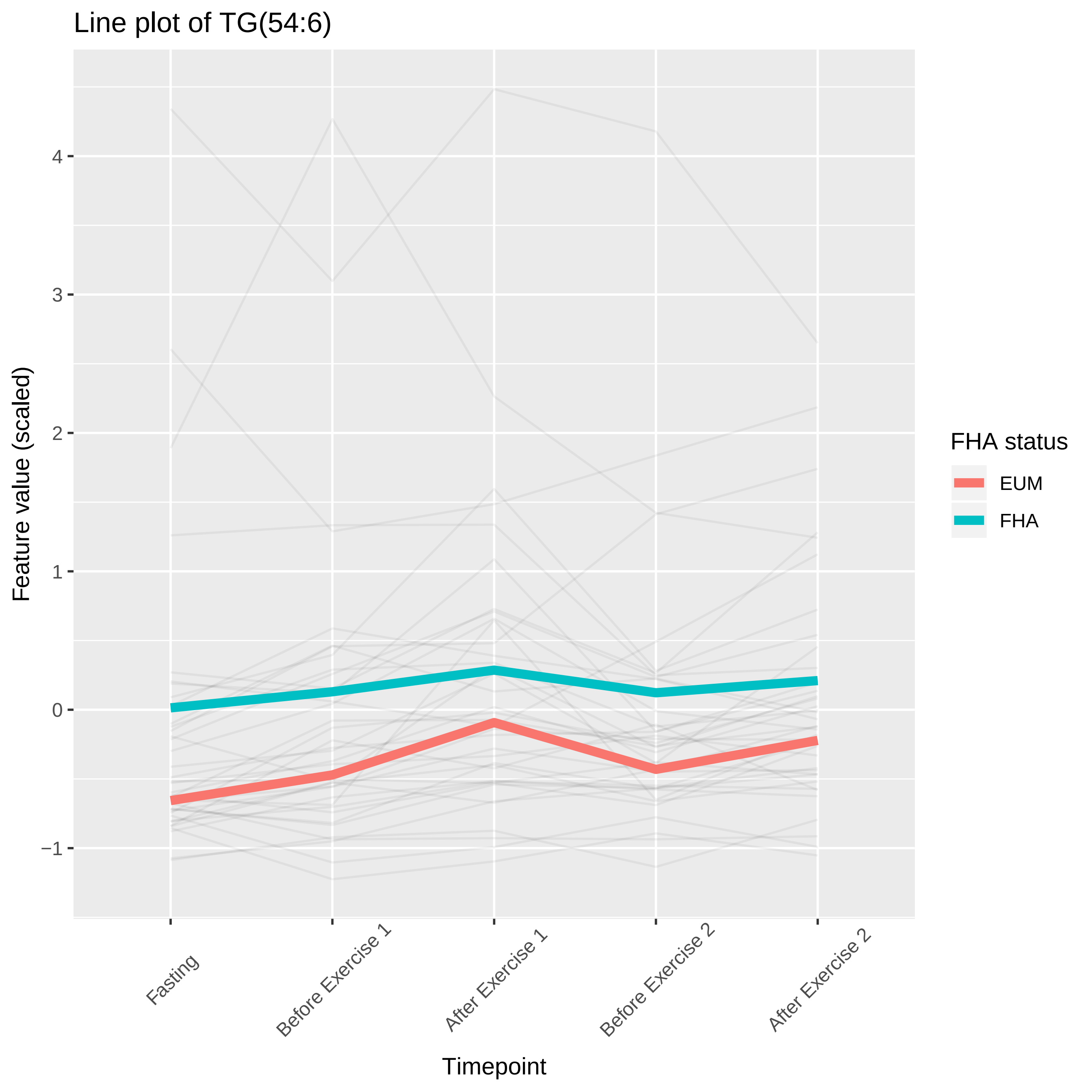

Line plot of TG(54:6)
Feature value (scaled)
4
3
2
1
0
−1
Fasting
Before Exercise 1
After Exercise 1
Before Exercise 2
After Exercise 2
Timepoint
FHA status
EUM
FHA

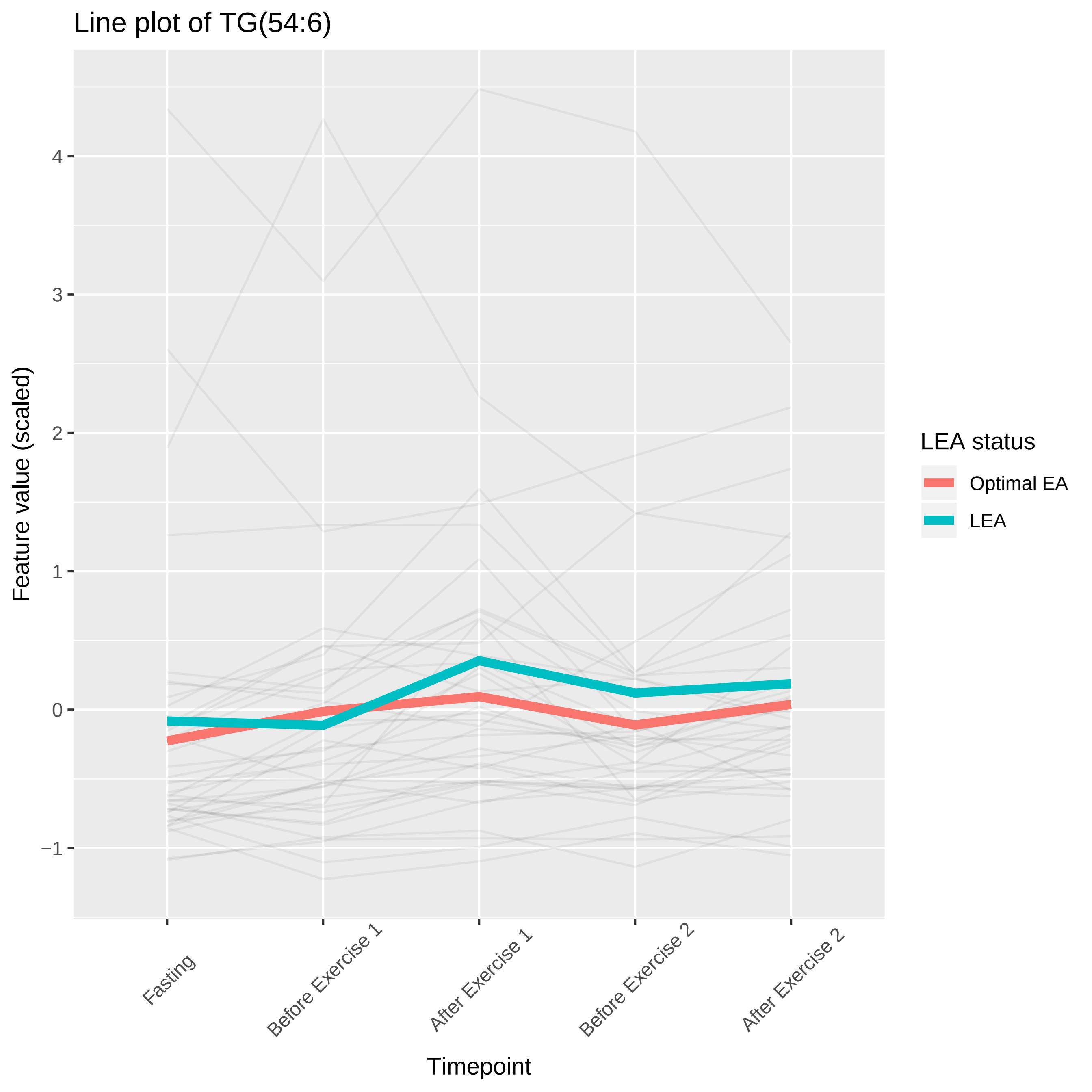

Line plot of TG(54:6)
Feature value (scaled)
4
3
2
1
0
−1
Fasting
Before Exercise 1
After Exercise 1
Before Exercise 2
After Exercise 2
Timepoint
LEA status
Optimal EA
LEA

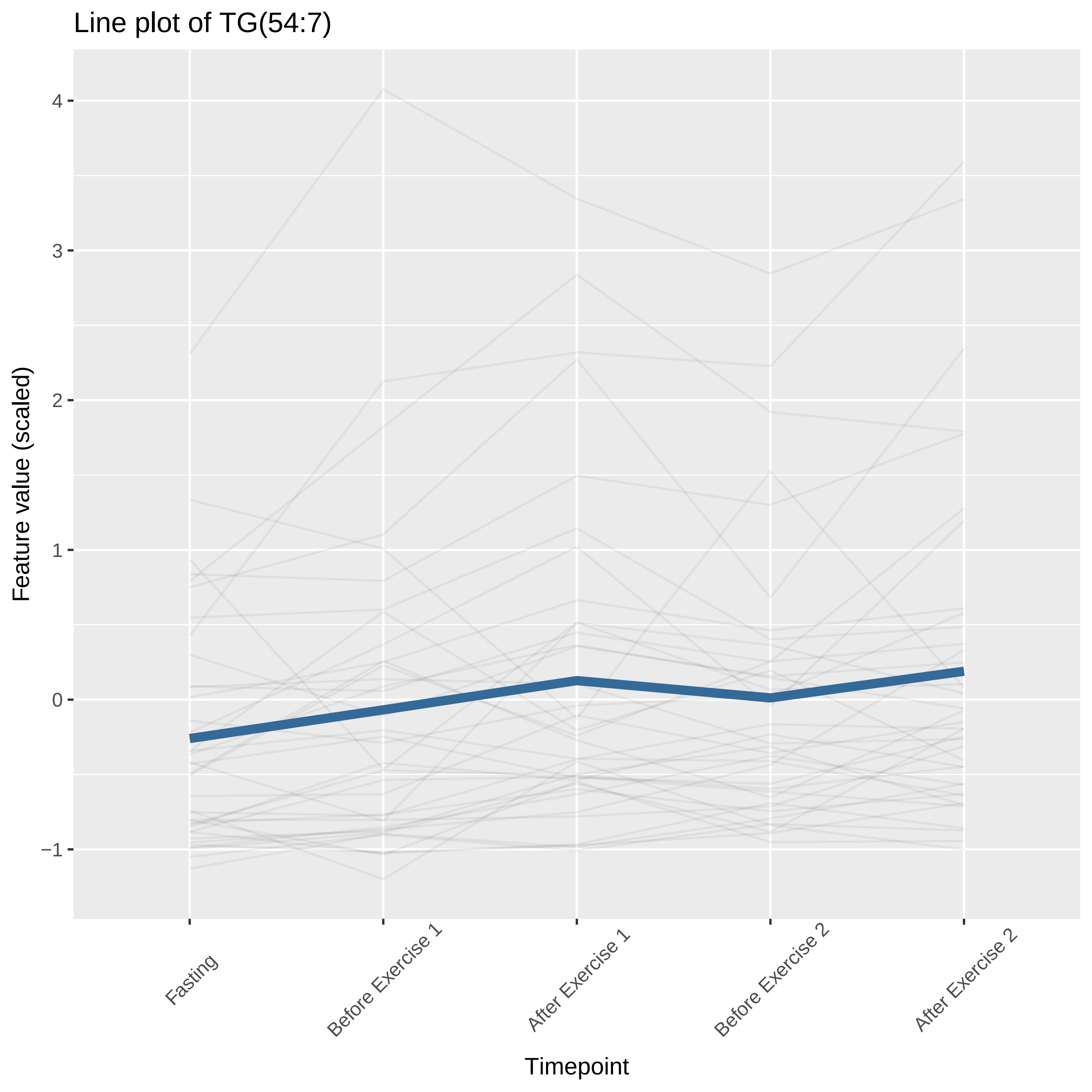

Line plot of TG(54:7)
Feature value (scaled)
4
3
2
1
0
−1
Fasting
Before Exercise 1
After Exercise 1
Before Exercise 2
After Exercise 2
Timepoint

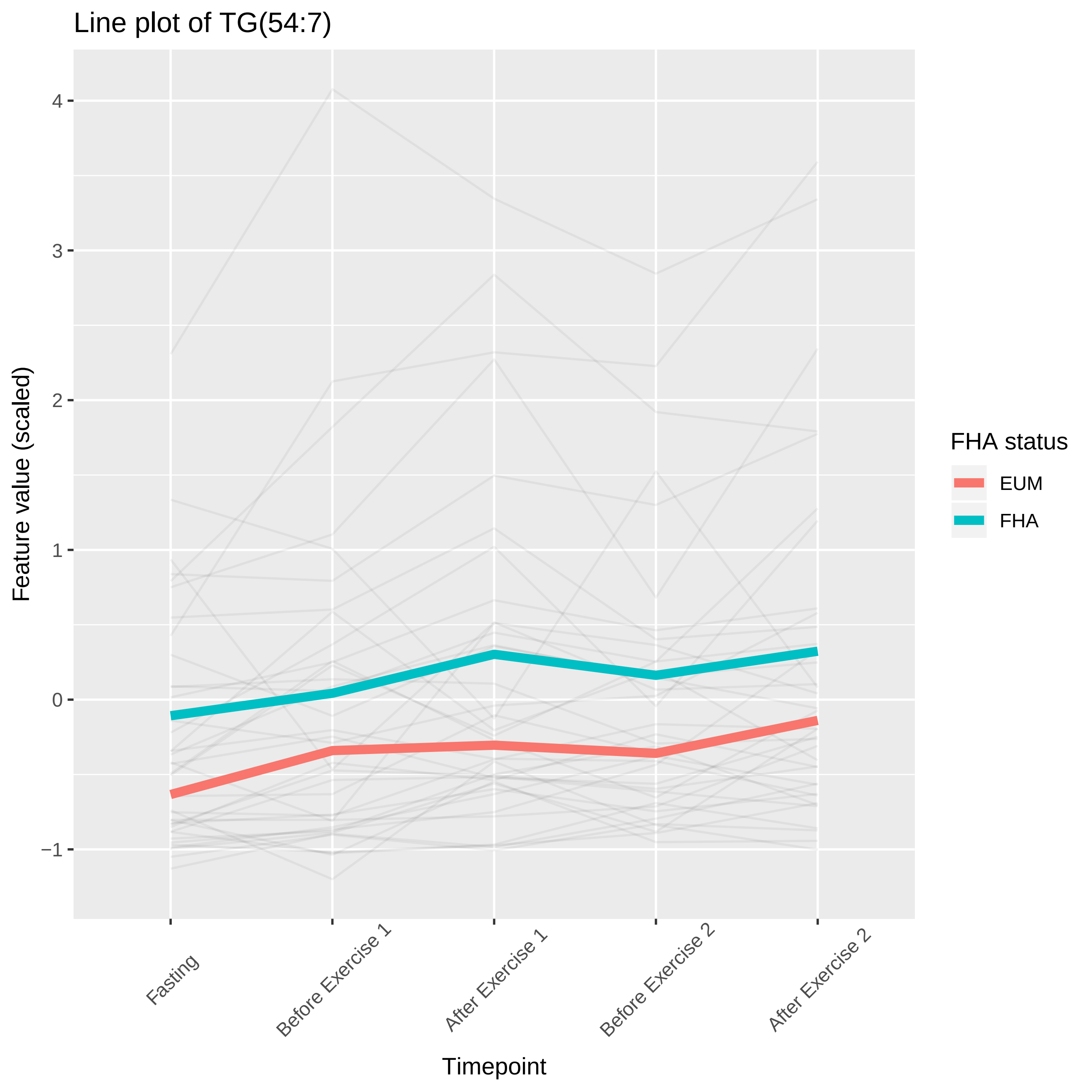

Line plot of TG(54:7)
Feature value (scaled)
4
3
2
1
0
−1
Fasting
Before Exercise 1
After Exercise 1
Before Exercise 2
After Exercise 2
Timepoint
FHA status
EUM
FHA

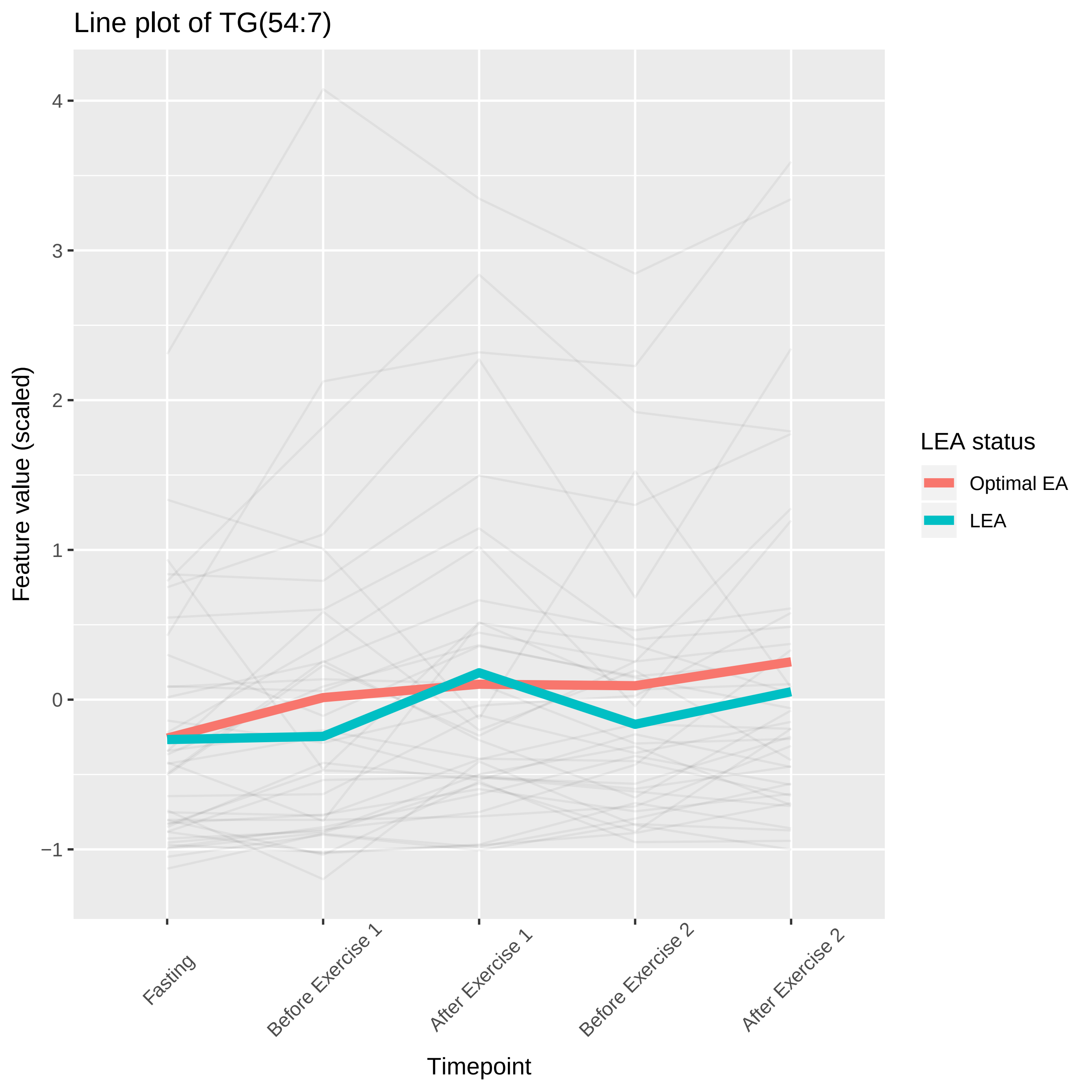

Line plot of TG(54:7)
Feature value (scaled)
4
3
2
1
0
−1
Fasting
Before Exercise 1
After Exercise 1
Before Exercise 2
After Exercise 2
Timepoint
LEA status
Optimal EA
LEA

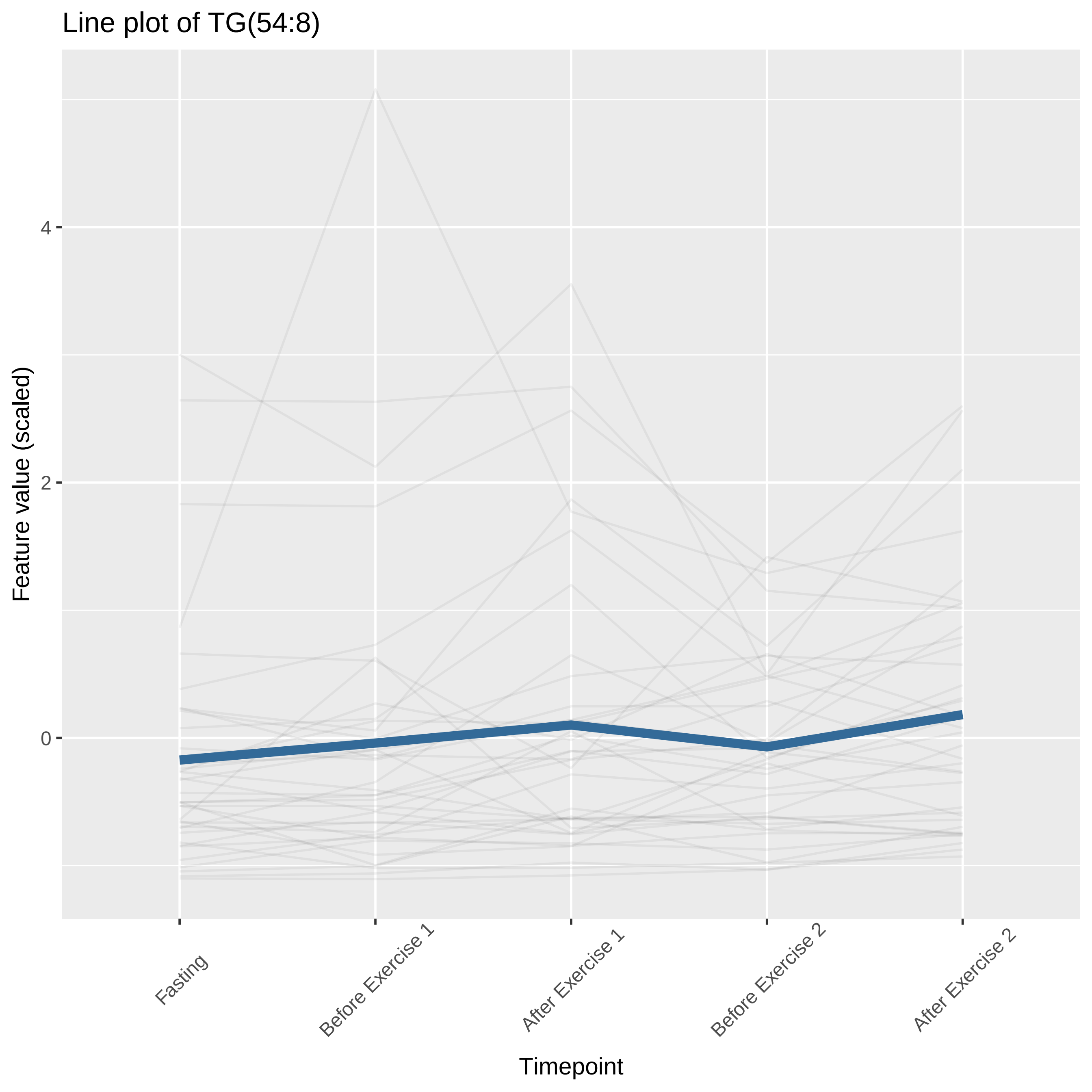

Line plot of TG(54:8)
Feature value (scaled)
4
2
0
Fasting
Before Exercise 1
After Exercise 1
Before Exercise 2
After Exercise 2
Timepoint

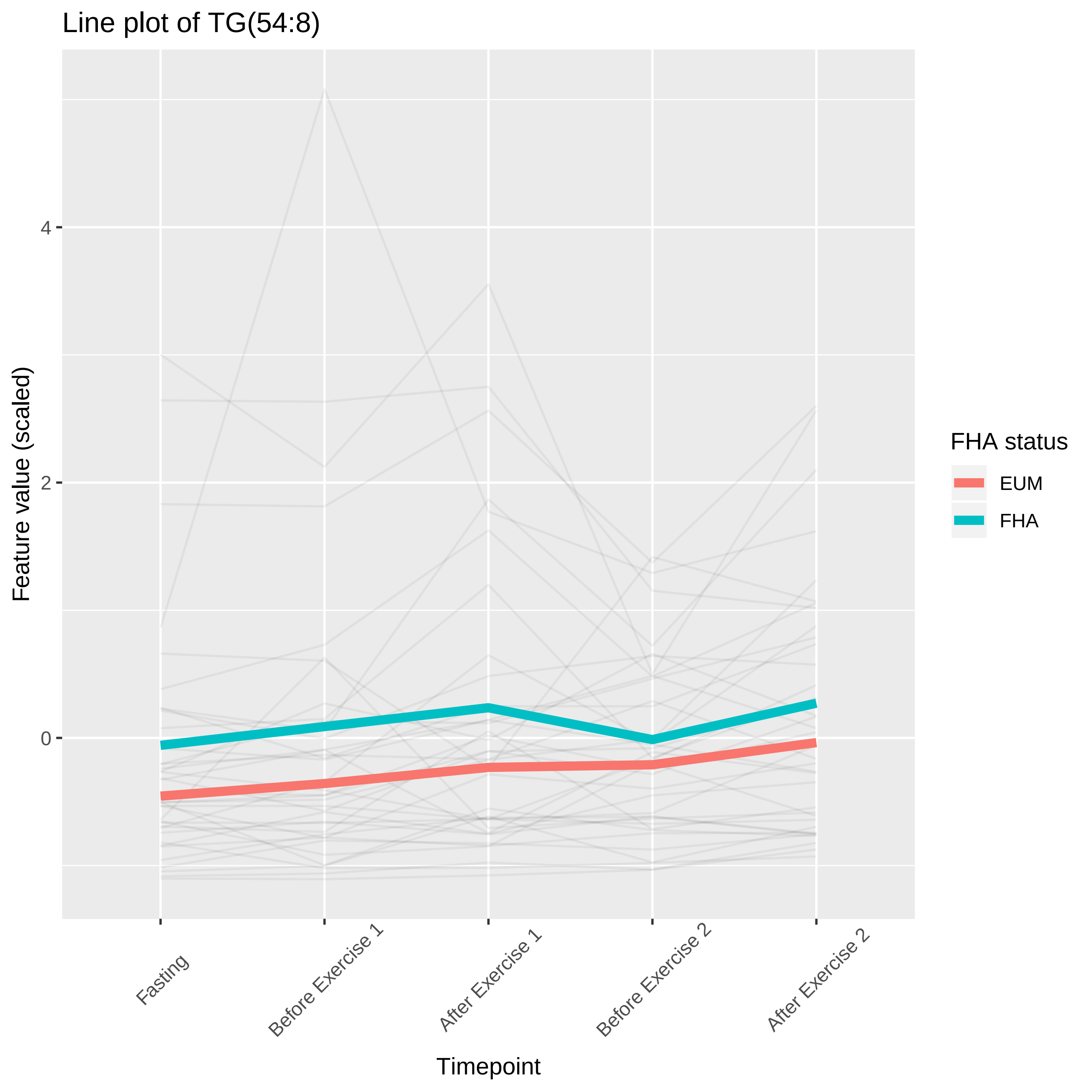

Line plot of TG(54:8)
Feature value (scaled)
4
2
0
Fasting
Before Exercise 1
After Exercise 1
Before Exercise 2
After Exercise 2
Timepoint
FHA status
EUM
FHA

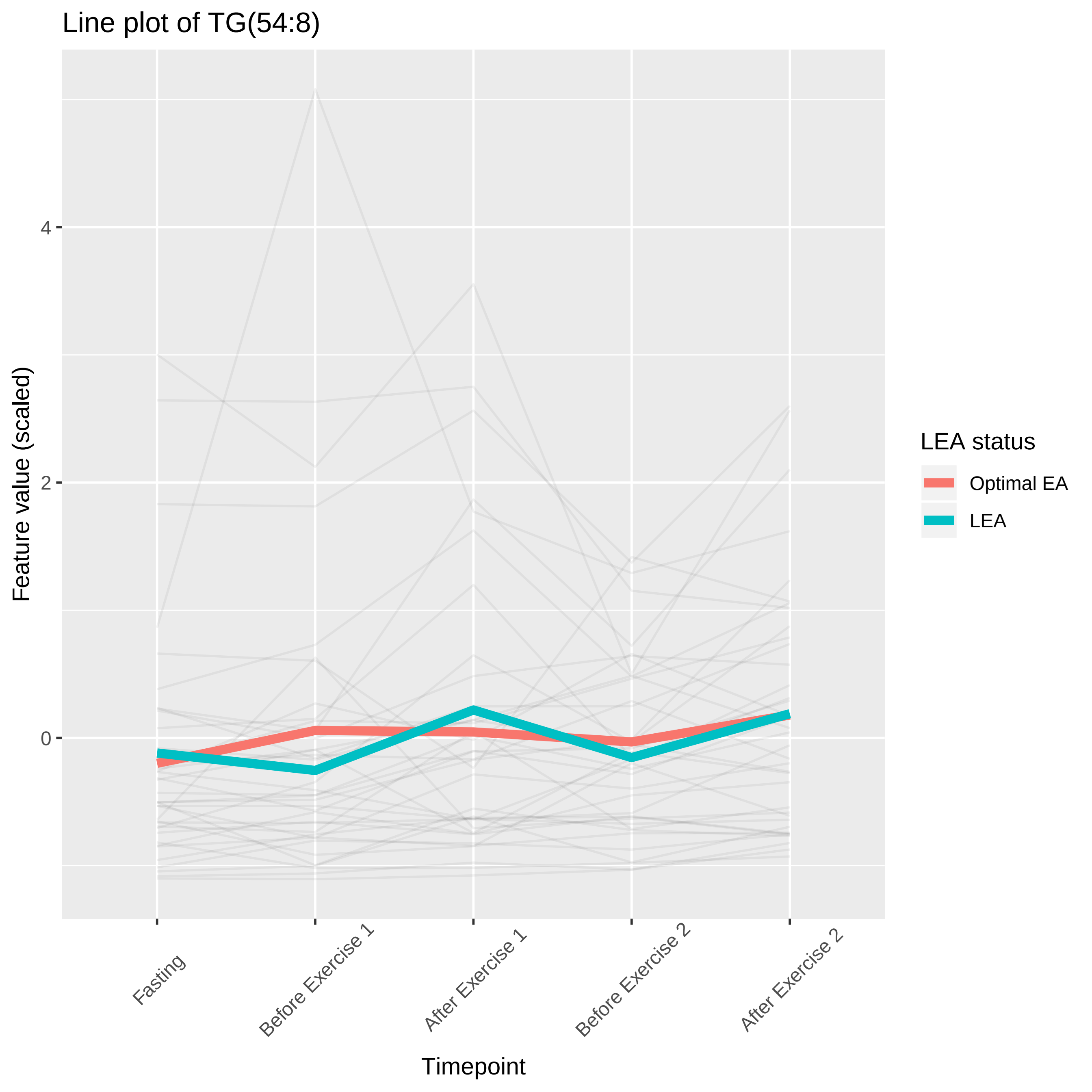
Line plot of TG(54:8)
Feature value (scaled)
4
2
0
Fasting
Before Exercise 1
After Exercise 1
Before Exercise 2
After Exercise 2
Timepoint
LEA status
Optimal EA
LEA

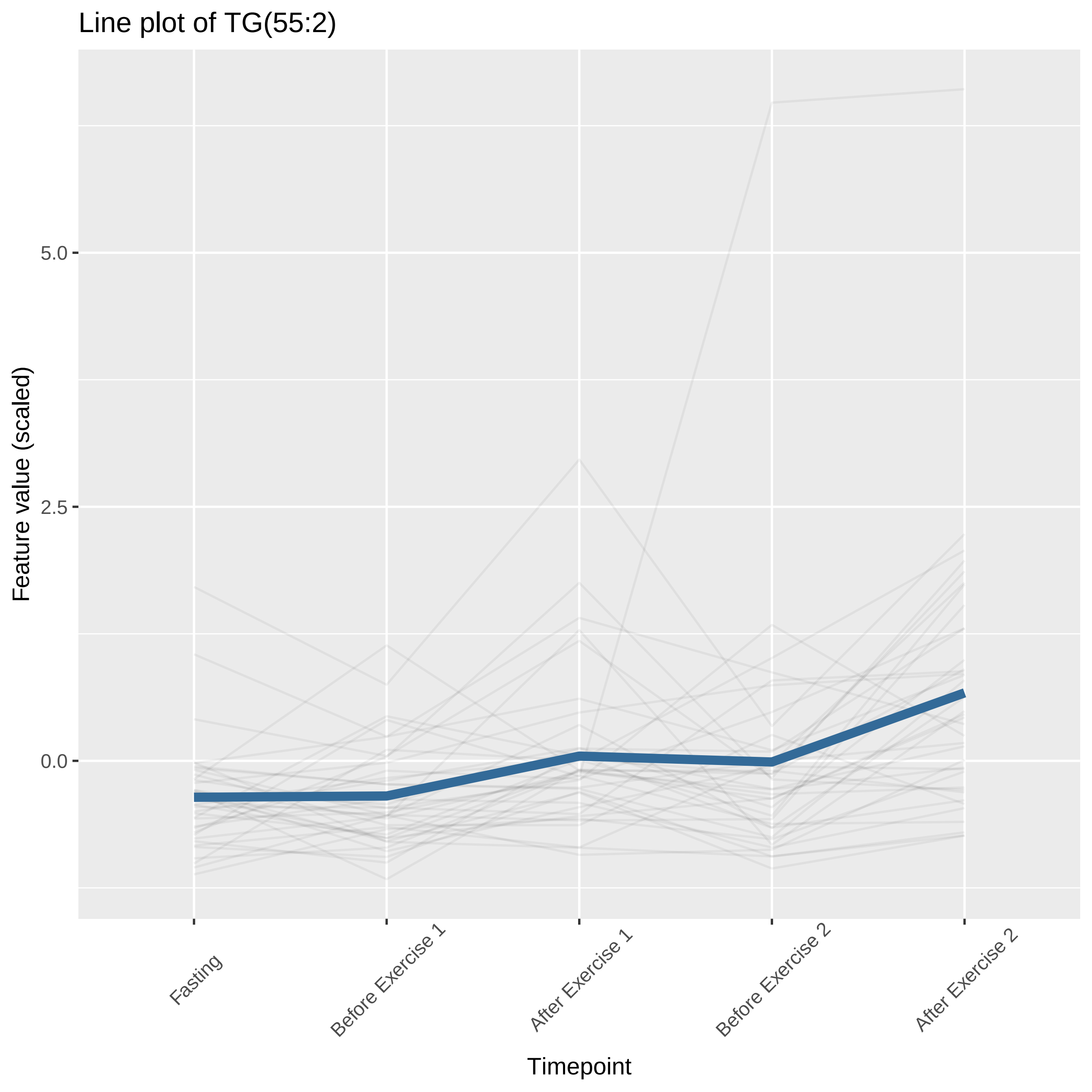

Line plot of TG(55:2)
Feature value (scaled)
5.0
2.5
0.0
Fasting
Before Exercise 1
After Exercise 1
Before Exercise 2
After Exercise 2
Timepoint

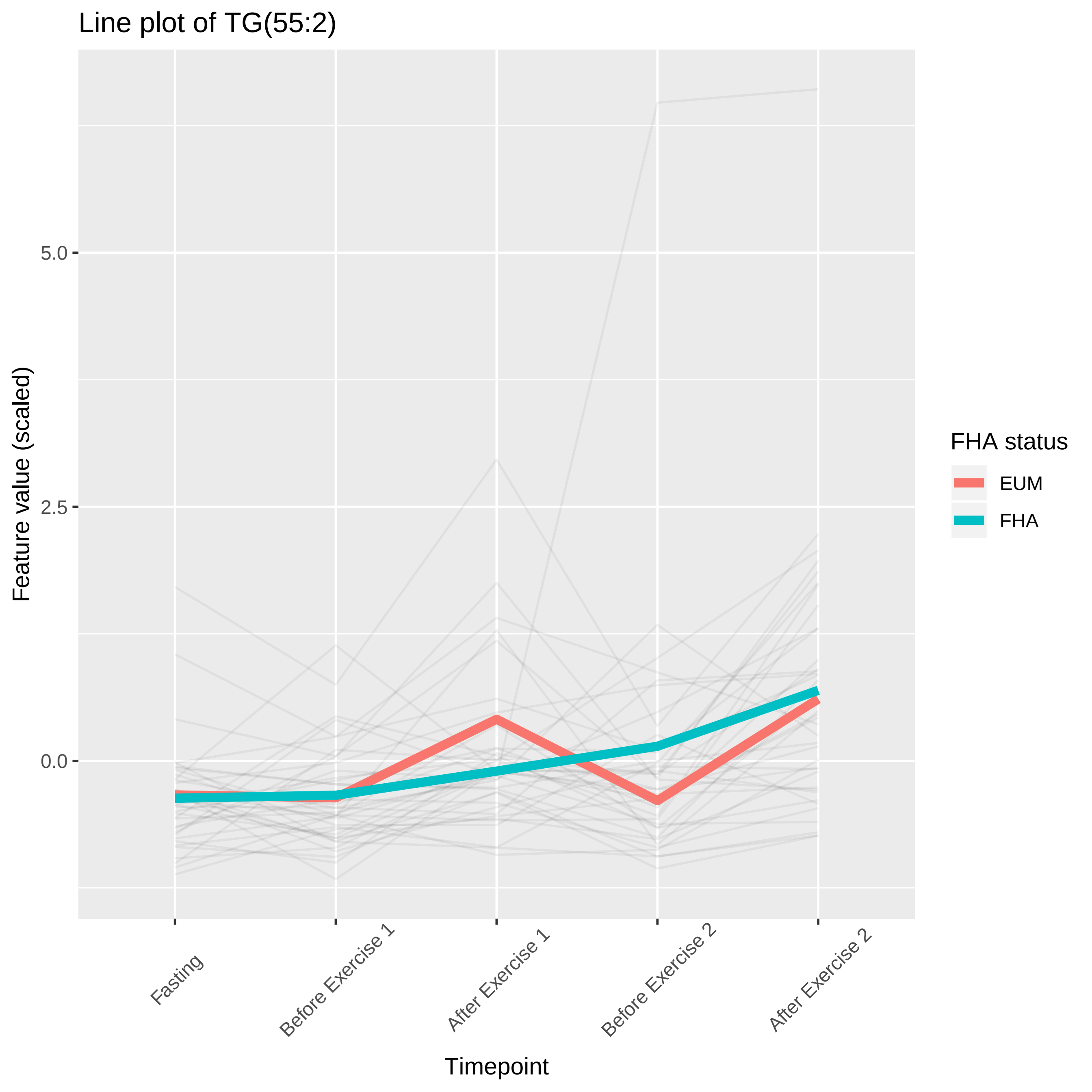

Line plot of TG(55:2)
Feature value (scaled)
5.0
2.5
0.0
Fasting
Before Exercise 1
After Exercise 1
Before Exercise 2
After Exercise 2
Timepoint
FHA status
EUM
FHA

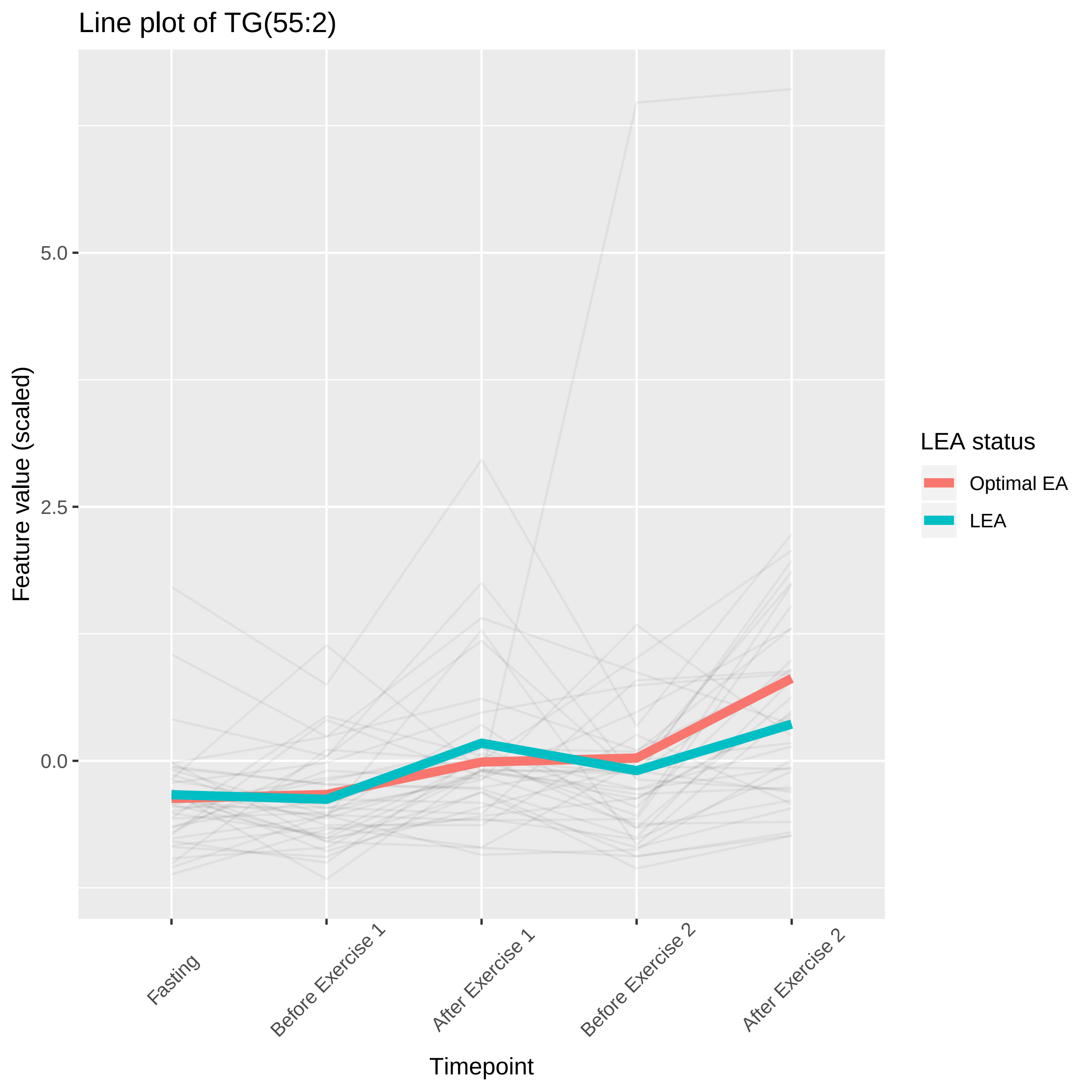

Line plot of TG(55:2)
Feature value (scaled)
5.0
2.5
0.0
Fasting
Before Exercise 1
After Exercise 1
Before Exercise 2
After Exercise 2
Timepoint
LEA status
Optimal EA
LEA

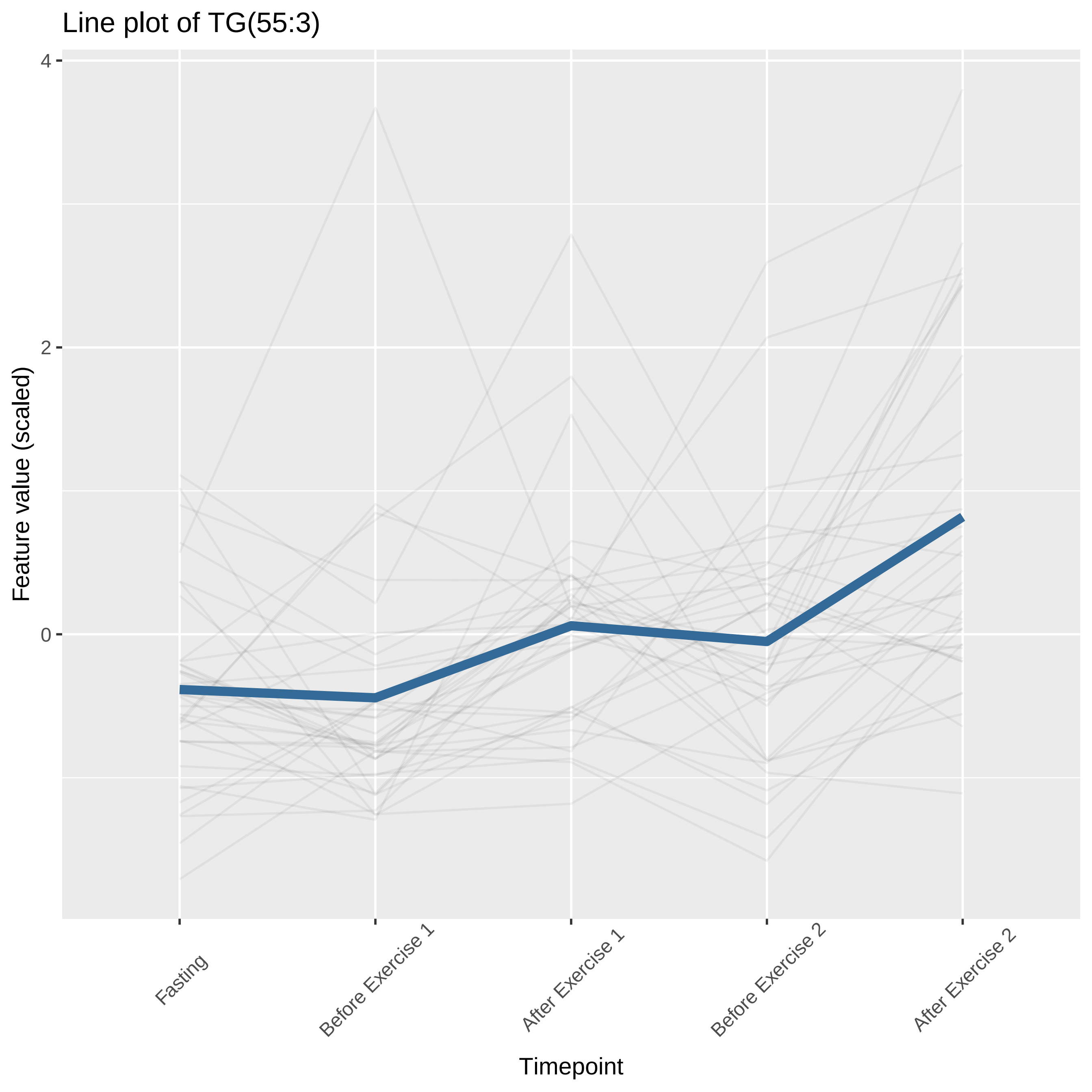

Line plot of TG(55:3)
Feature value (scaled)
4
2
0
Fasting
Before Exercise 1
After Exercise 1
Before Exercise 2
After Exercise 2
Timepoint

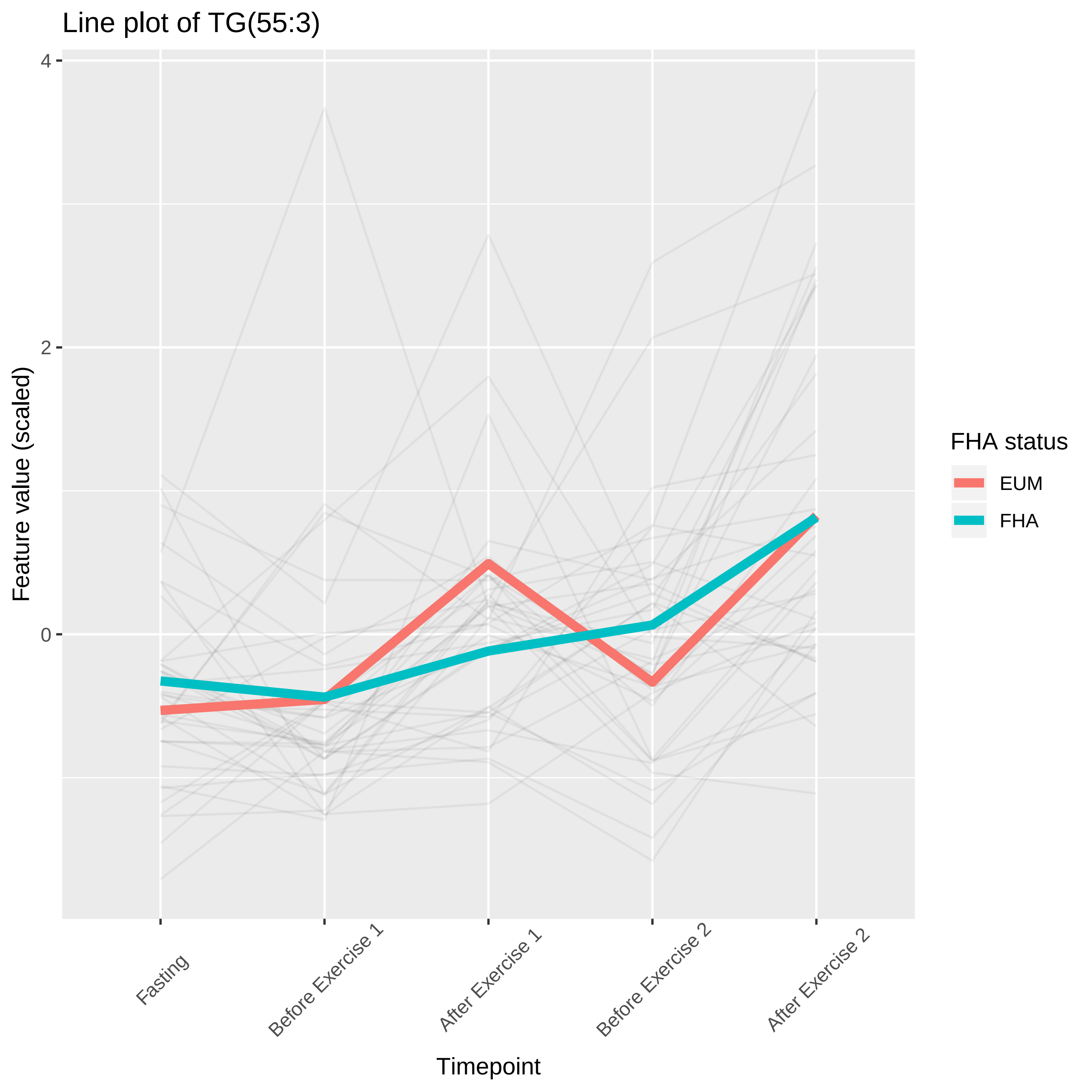

Line plot of TG(55:3)
4
2
0
Feature value (scaled)
Fasting
Before Exercise 1
After Exercise 1
Before Exercise 2
After Exercise 2
Timepoint
FHA status
EUM
FHA

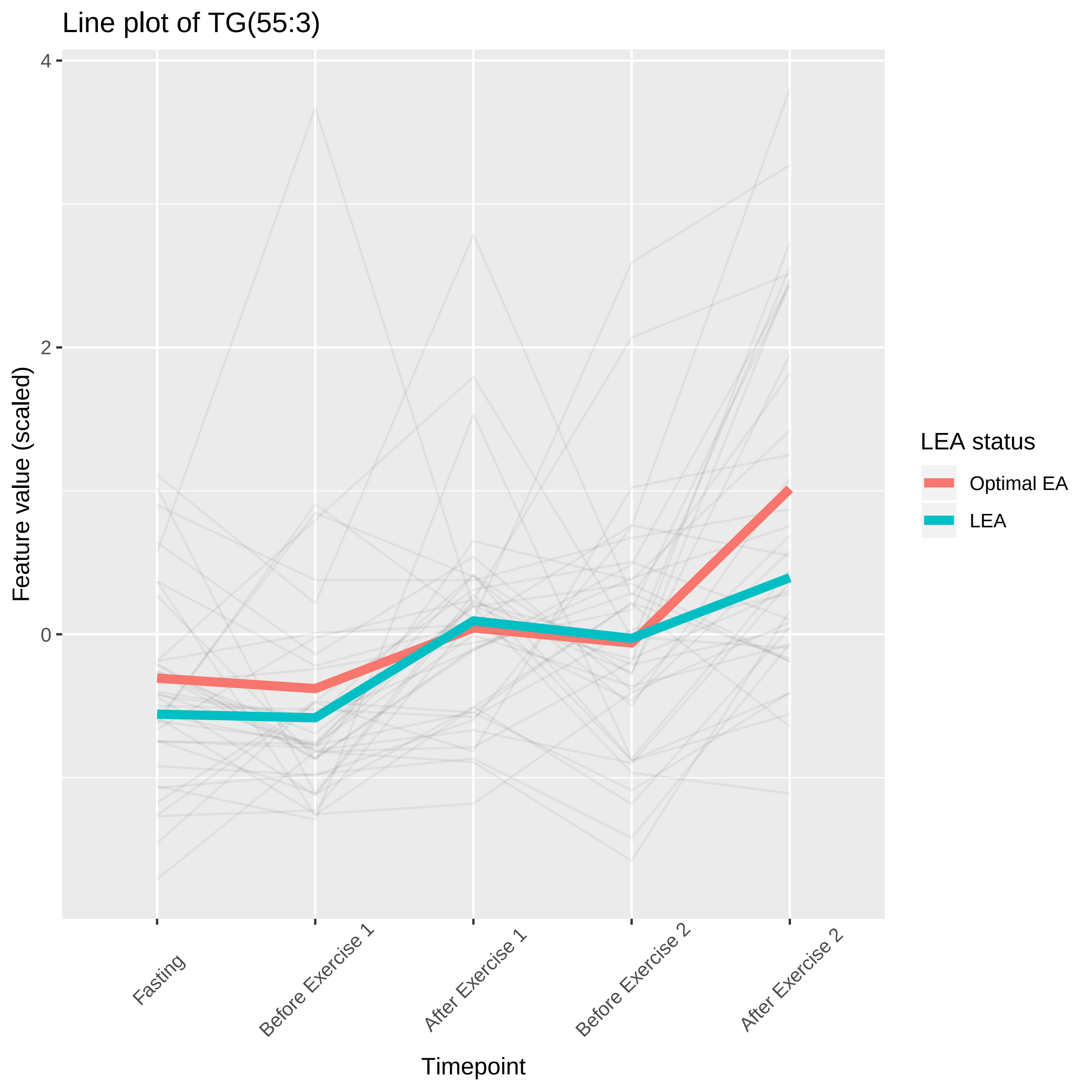

Line plot of TG(55:3)
Feature value (scaled)
4
2
0
Fasting
Before Exercise 1
After Exercise 1
Before Exercise 2
After Exercise 2
Timepoint
LEA status
Optimal EA
LEA

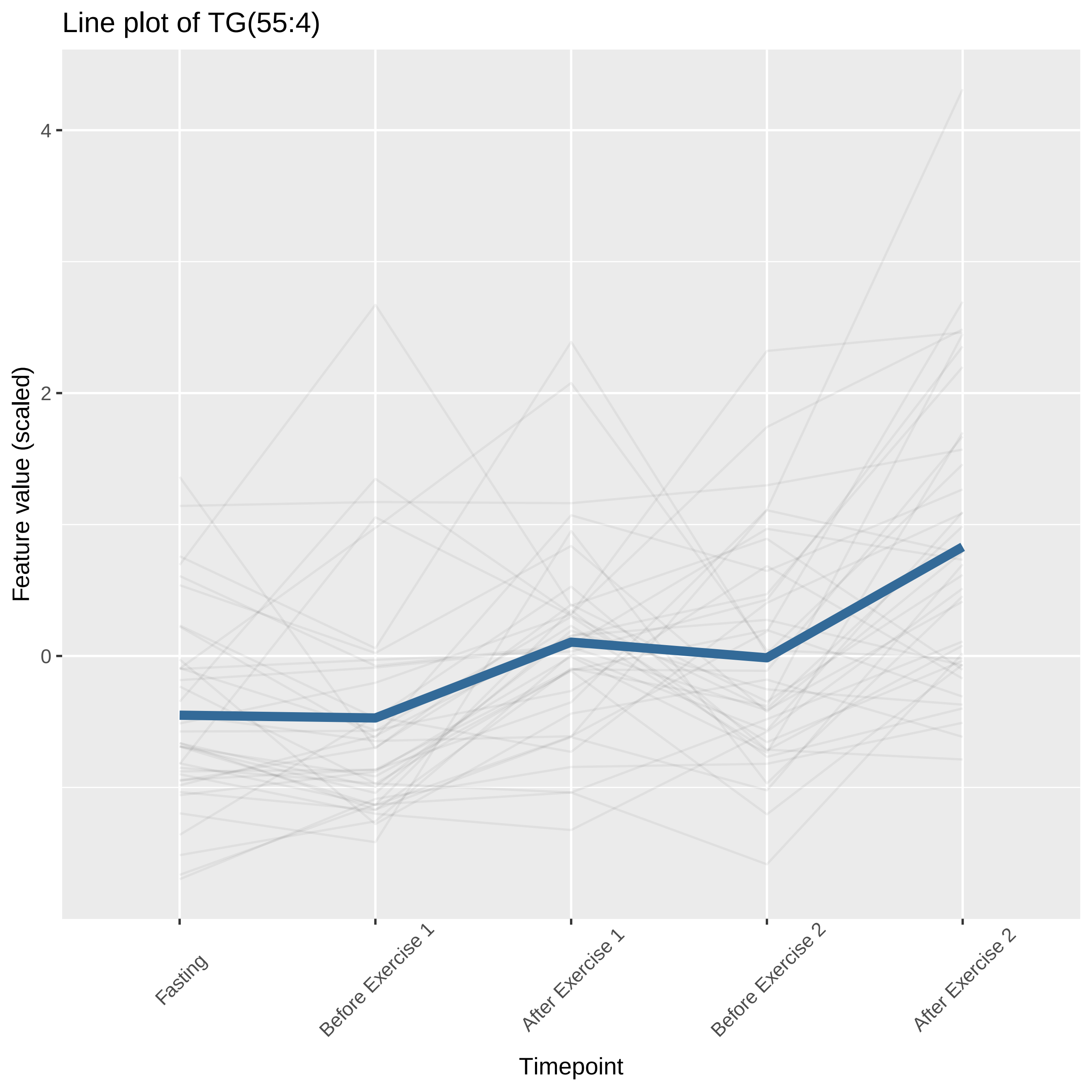

Line plot of TG(55:4)
Feature value (scaled)
4
2
0
Fasting
Before Exercise 1
After Exercise 1
Before Exercise 2
After Exercise 2
Timepoint

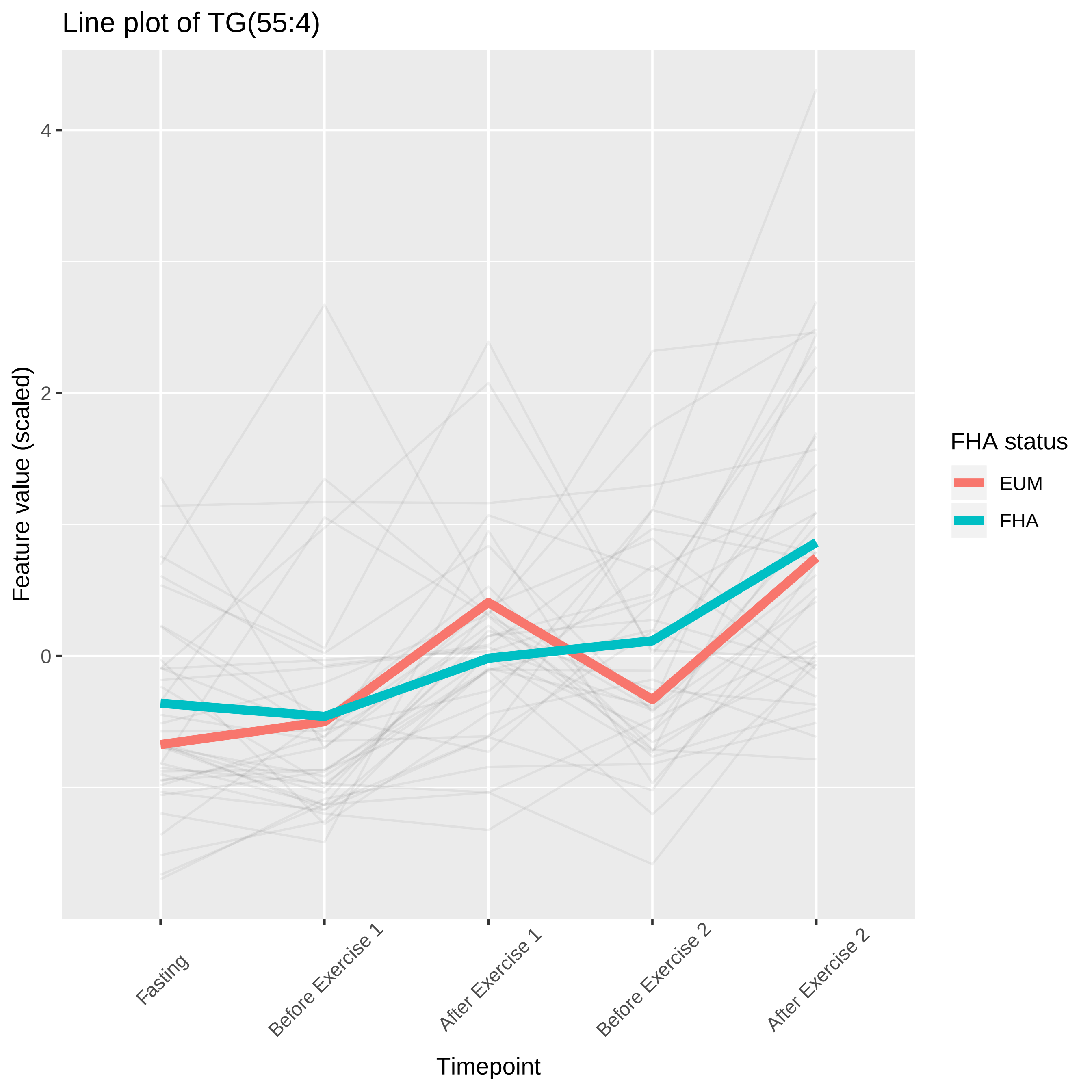
Line plot of TG(55:4)
Feature value (scaled)
4
2
0
Fasting
Before Exercise 1
After Exercise 1
Before Exercise 2
After Exercise 2
Timepoint
FHA status
EUM
FHA

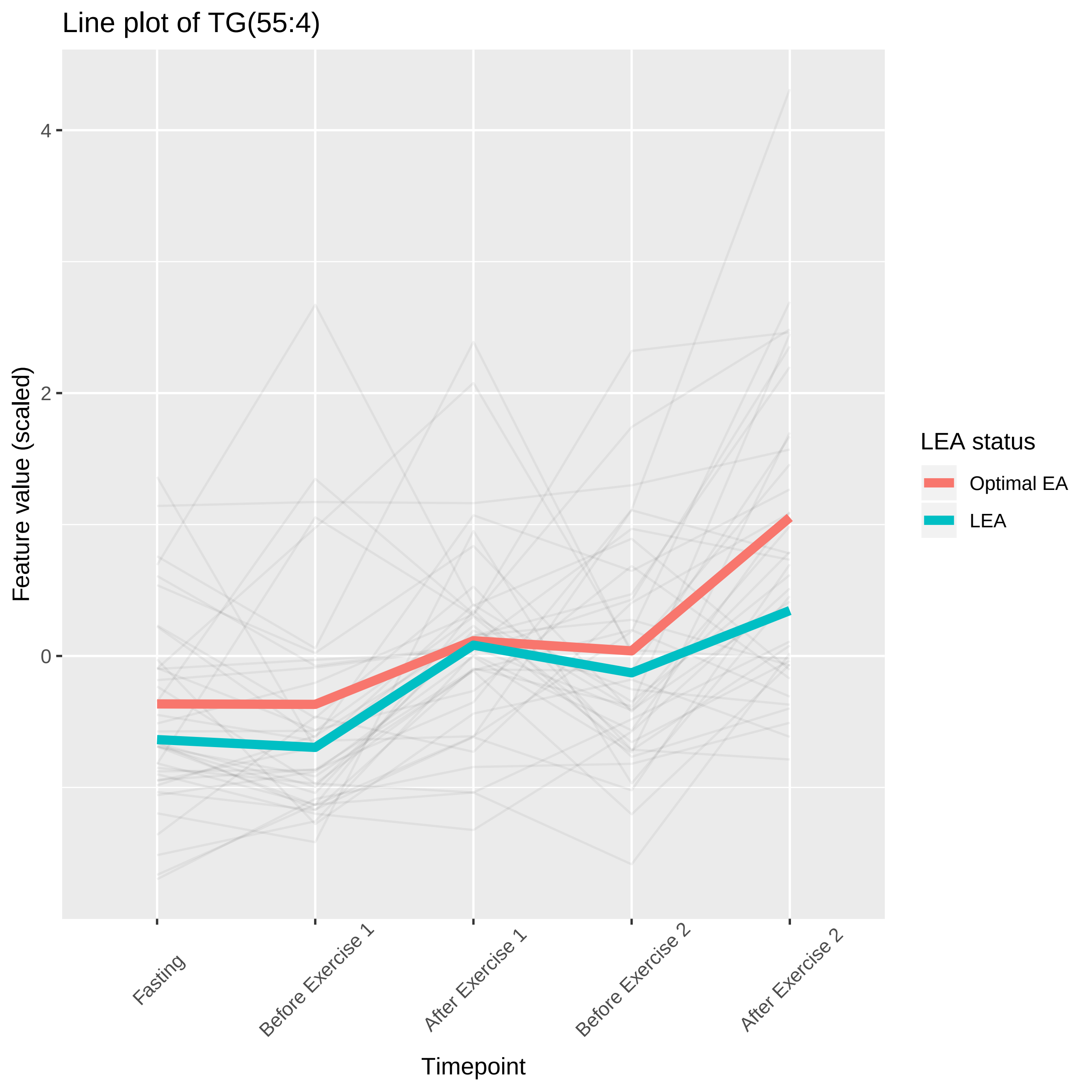

Line plot of TG(55:4)
Feature value (scaled)
4
2
0
Fasting
Before Exercise 1
After Exercise 1
Before Exercise 2
After Exercise 2
Timepoint
LEA status
Optimal EA
LEA

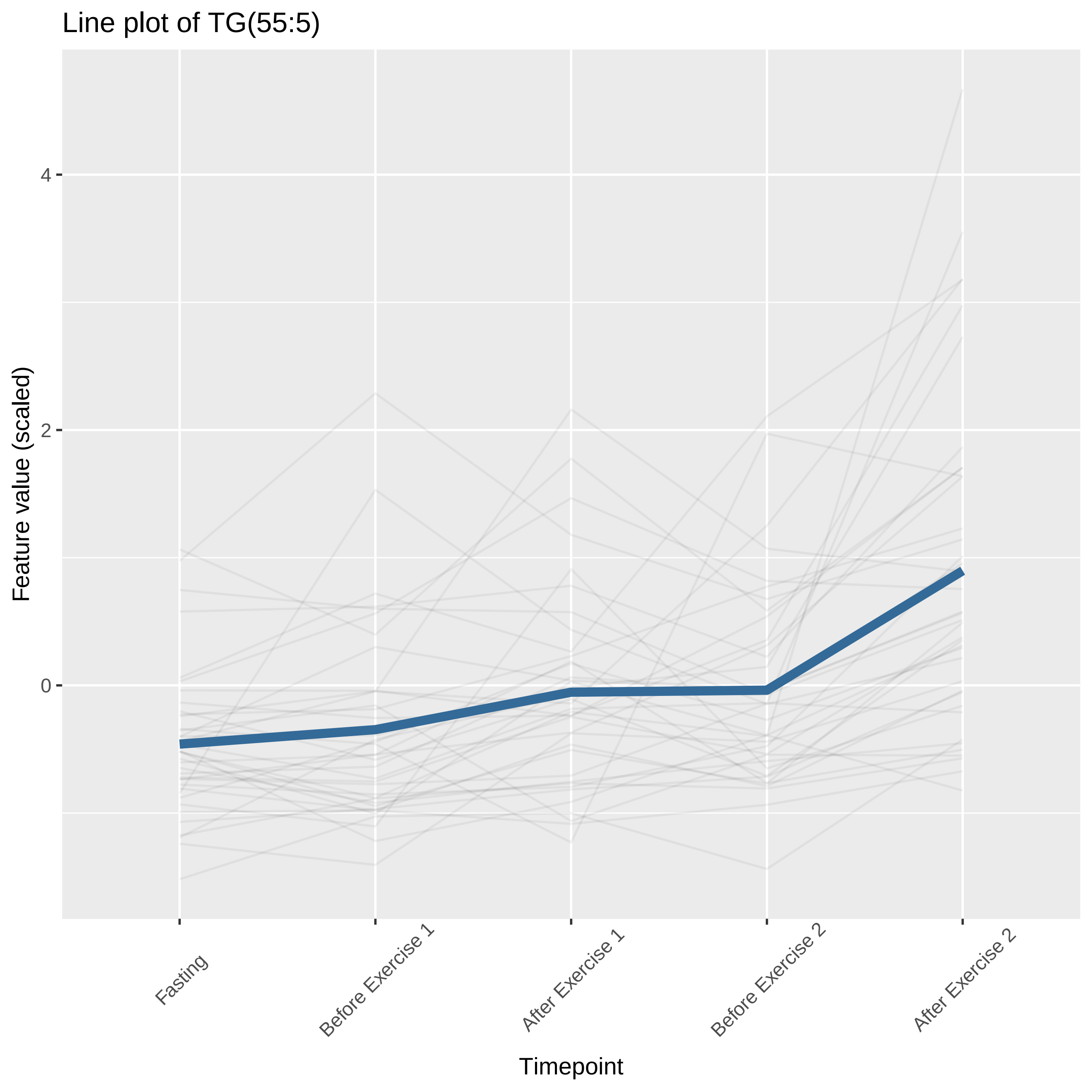

Line plot of TG(55:5)
Feature value (scaled)
4
2
0
Fasting
Before Exercise 1
After Exercise 1
Before Exercise 2
After Exercise 2
Timepoint

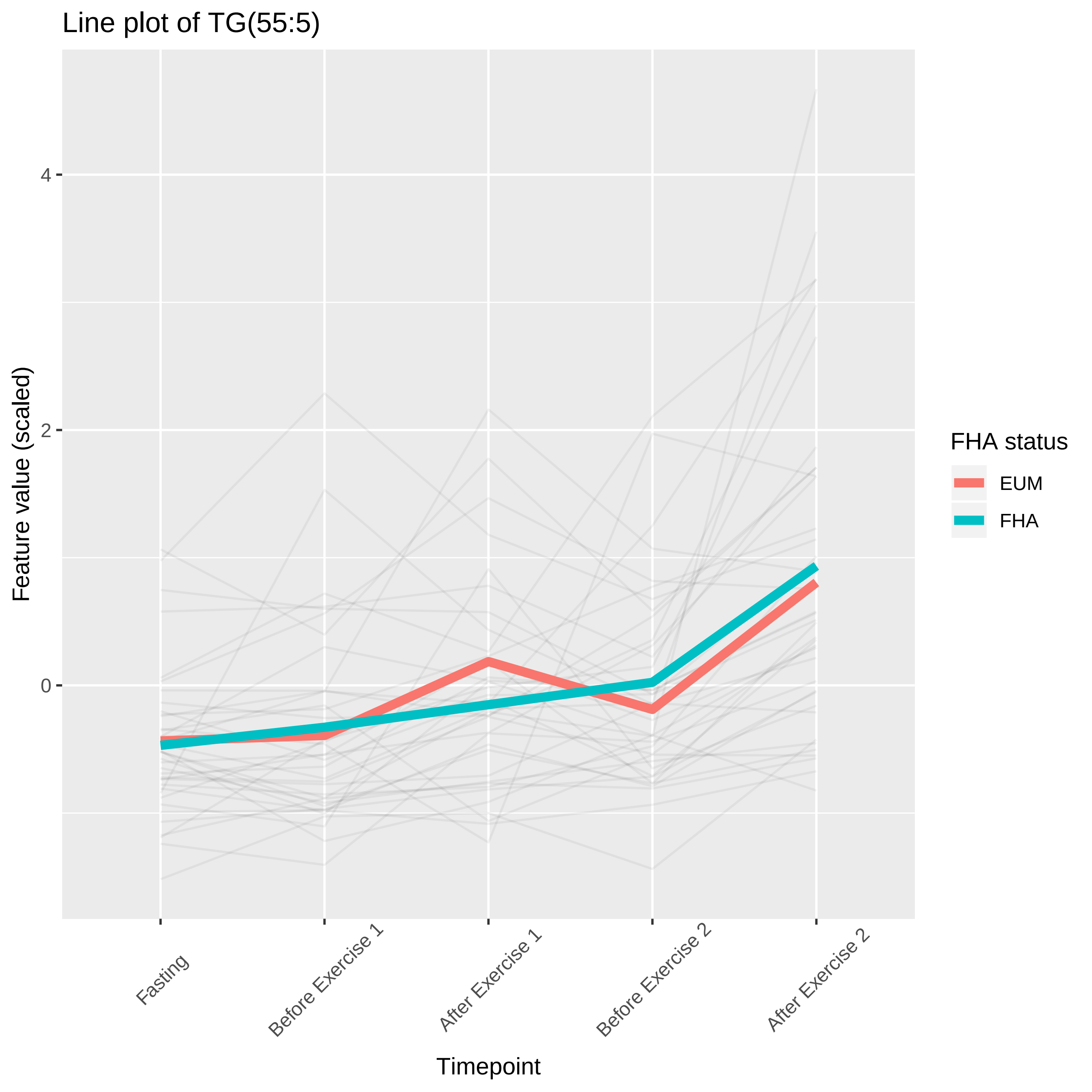

Line plot of TG(55:5)
Feature value (scaled)
4
2
0
Fasting
Before Exercise 1
After Exercise 1
Before Exercise 2
After Exercise 2
Timepoint
FHA status
EUM
FHA

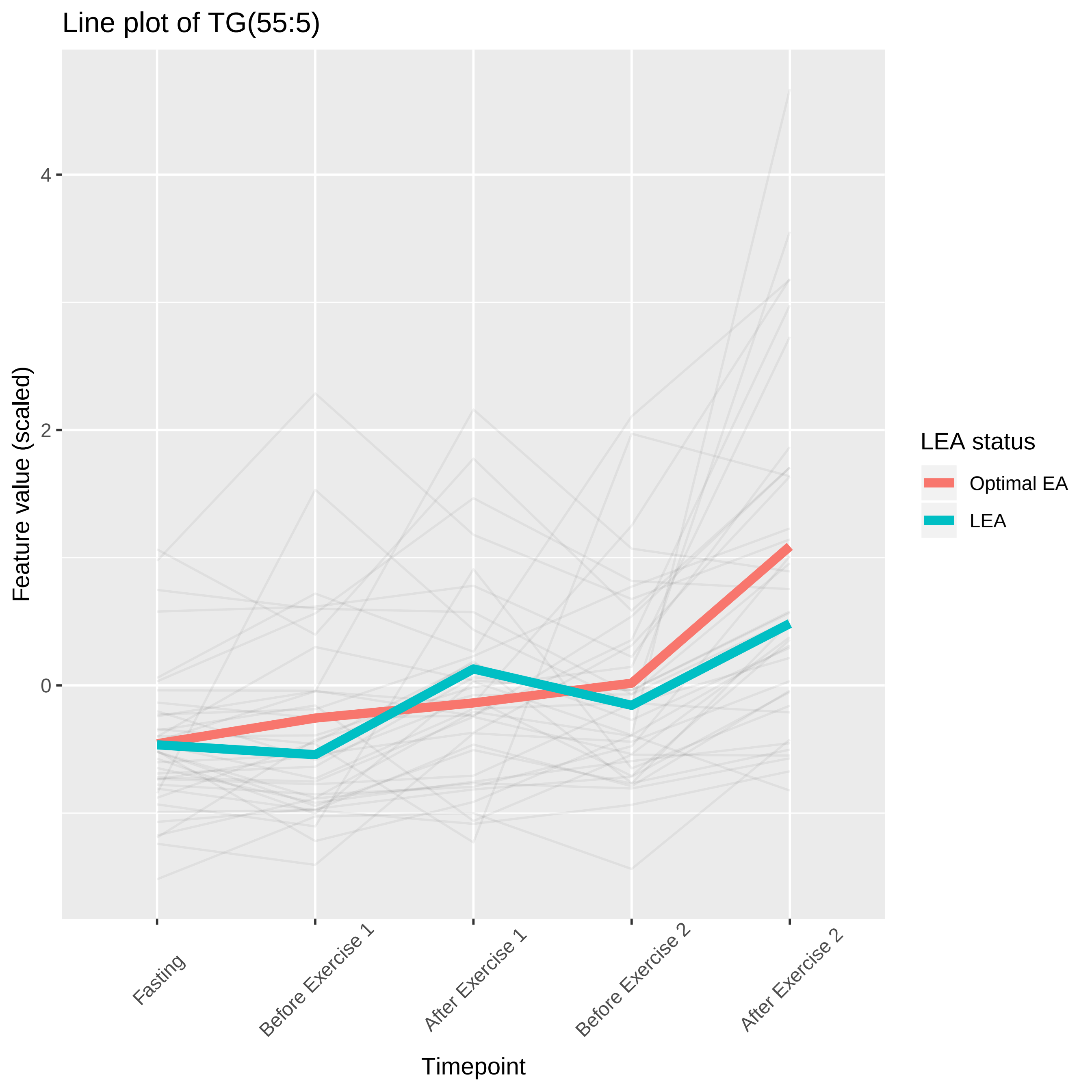

Line plot of TG(55:5)
Feature value (scaled)
4
2
0
Fasting
Before Exercise 1
After Exercise 1
Before Exercise 2
After Exercise 2
Timepoint
LEA status
Optimal EA
LEA

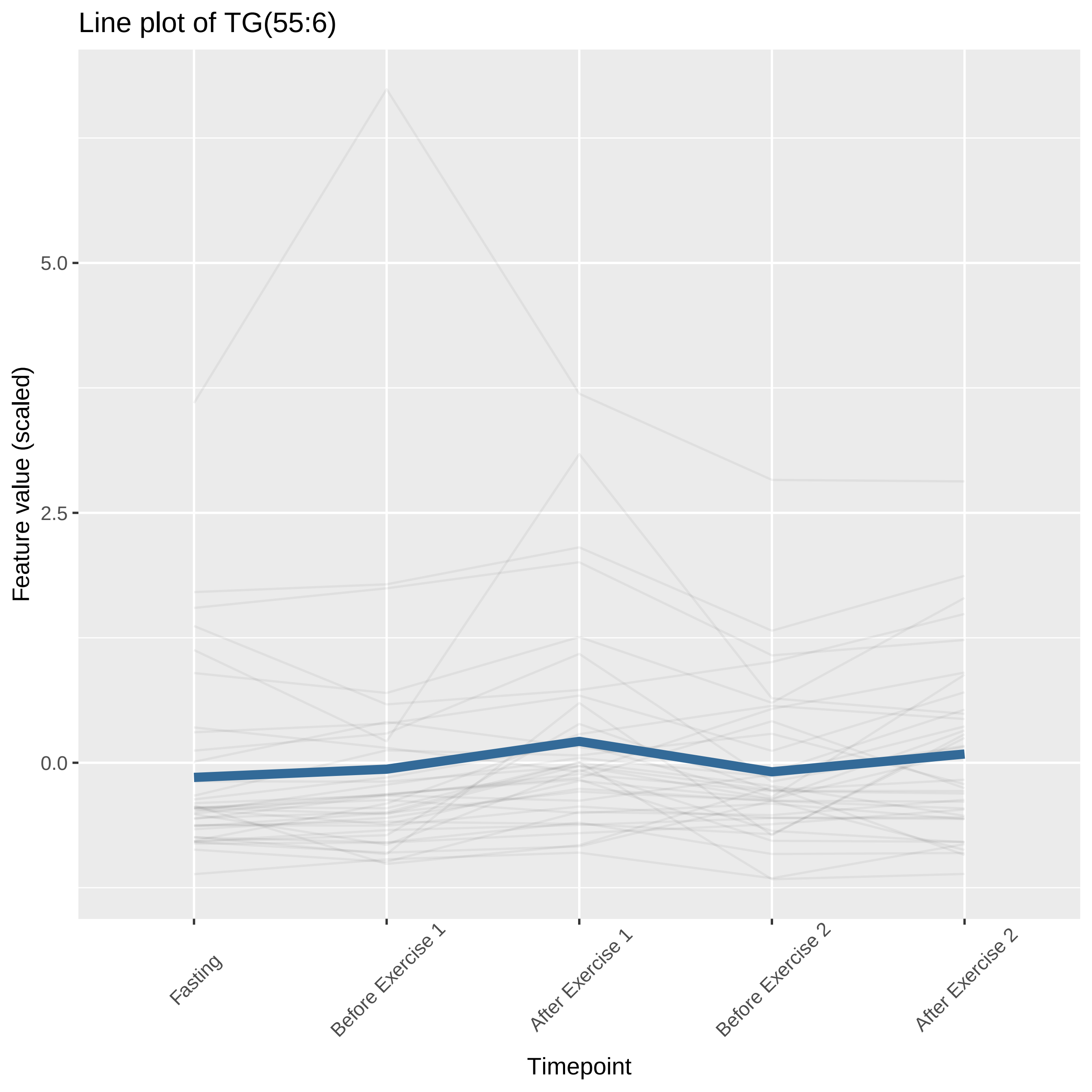
Line plot of TG(55:6)
Feature value (scaled)
5.0
2.5
0.0
Fasting
Before Exercise 1
After Exercise 1
Before Exercise 2
After Exercise 2
Timepoint

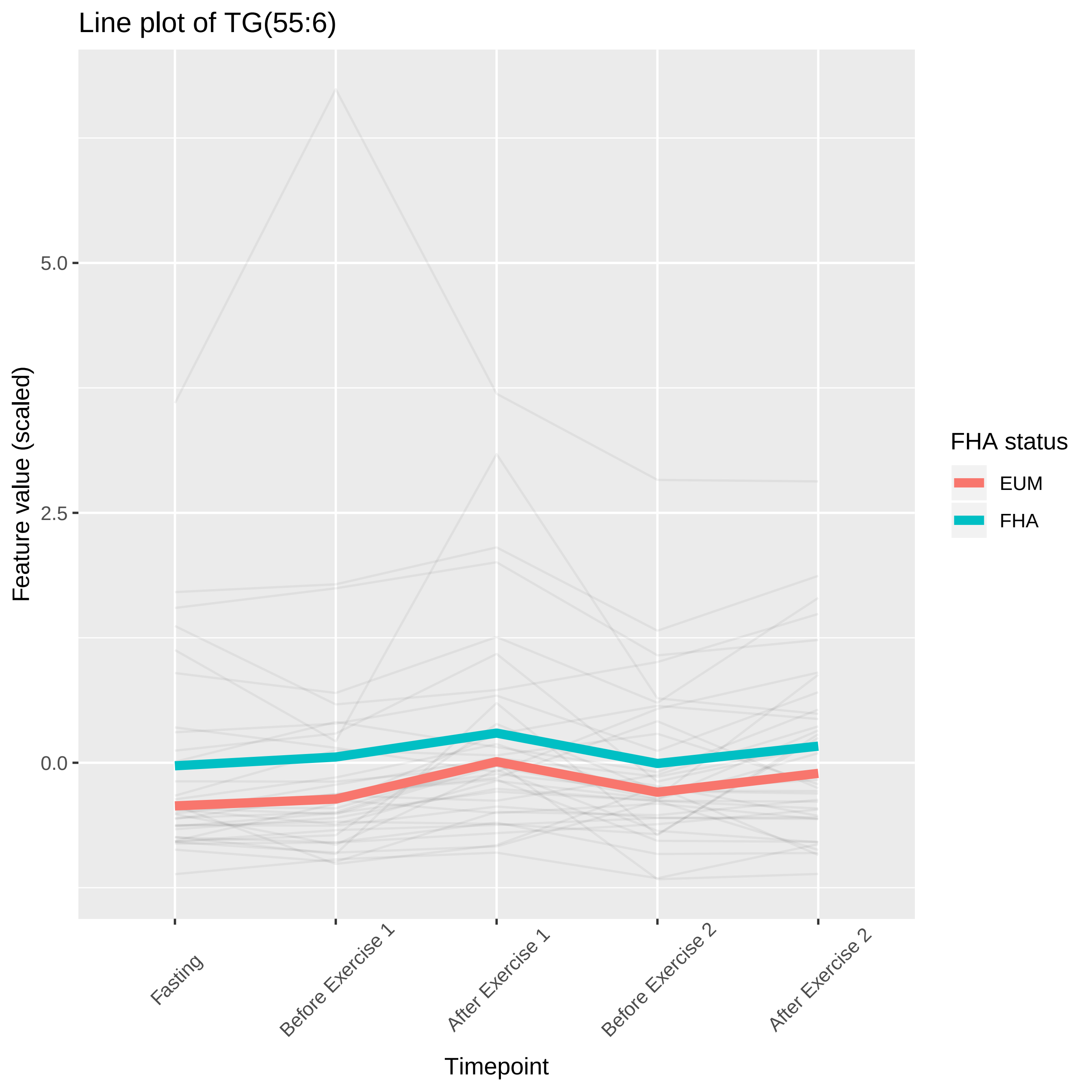

Line plot of TG(55:6)
Feature value (scaled)
5.0
2.5
0.0
Fasting
Before Exercise 1
After Exercise 1
Before Exercise 2
After Exercise 2
Timepoint
FHA status
EUM
FHA

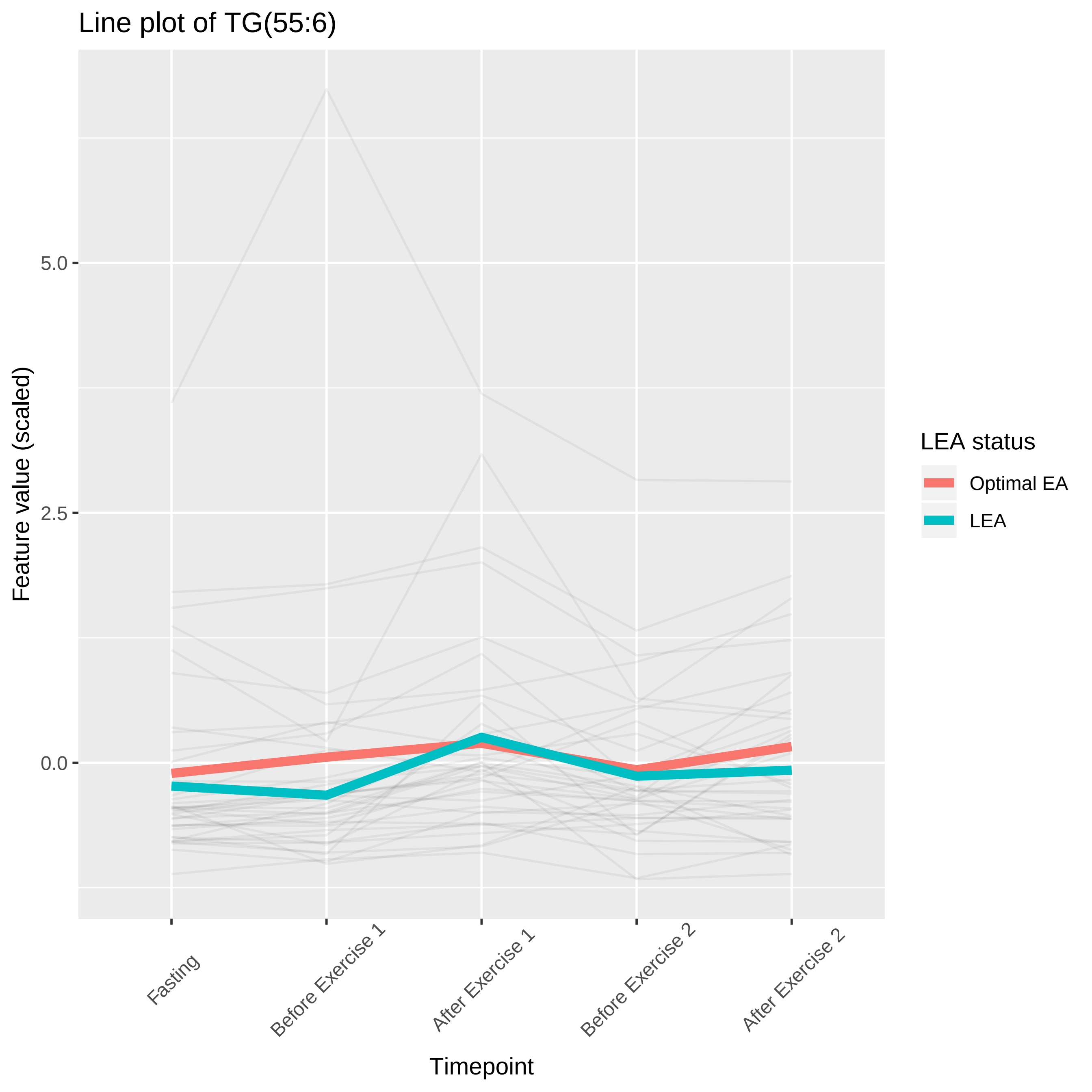

Line plot of TG(55:6)
Feature value (scaled)
5.0
2.5
0.0
Fasting
Before Exercise 1
After Exercise 1
Before Exercise 2
After Exercise 2
Timepoint
LEA status
Optimal EA
LEA

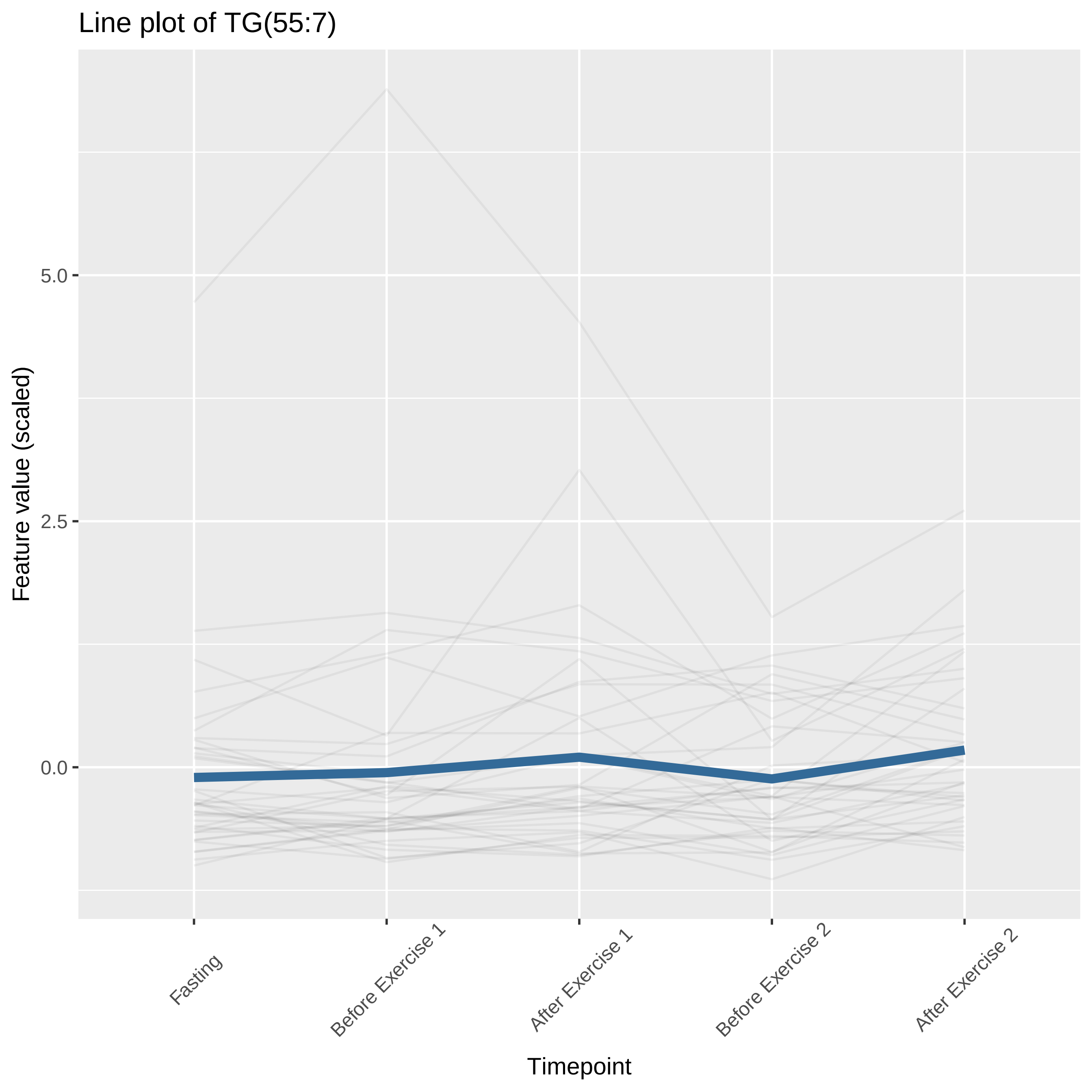

Line plot of TG(55:7)
Feature value (scaled)
5.0
2.5
0.0
Fasting
Before Exercise 1
After Exercise 1
Before Exercise 2
After Exercise 2
Timepoint

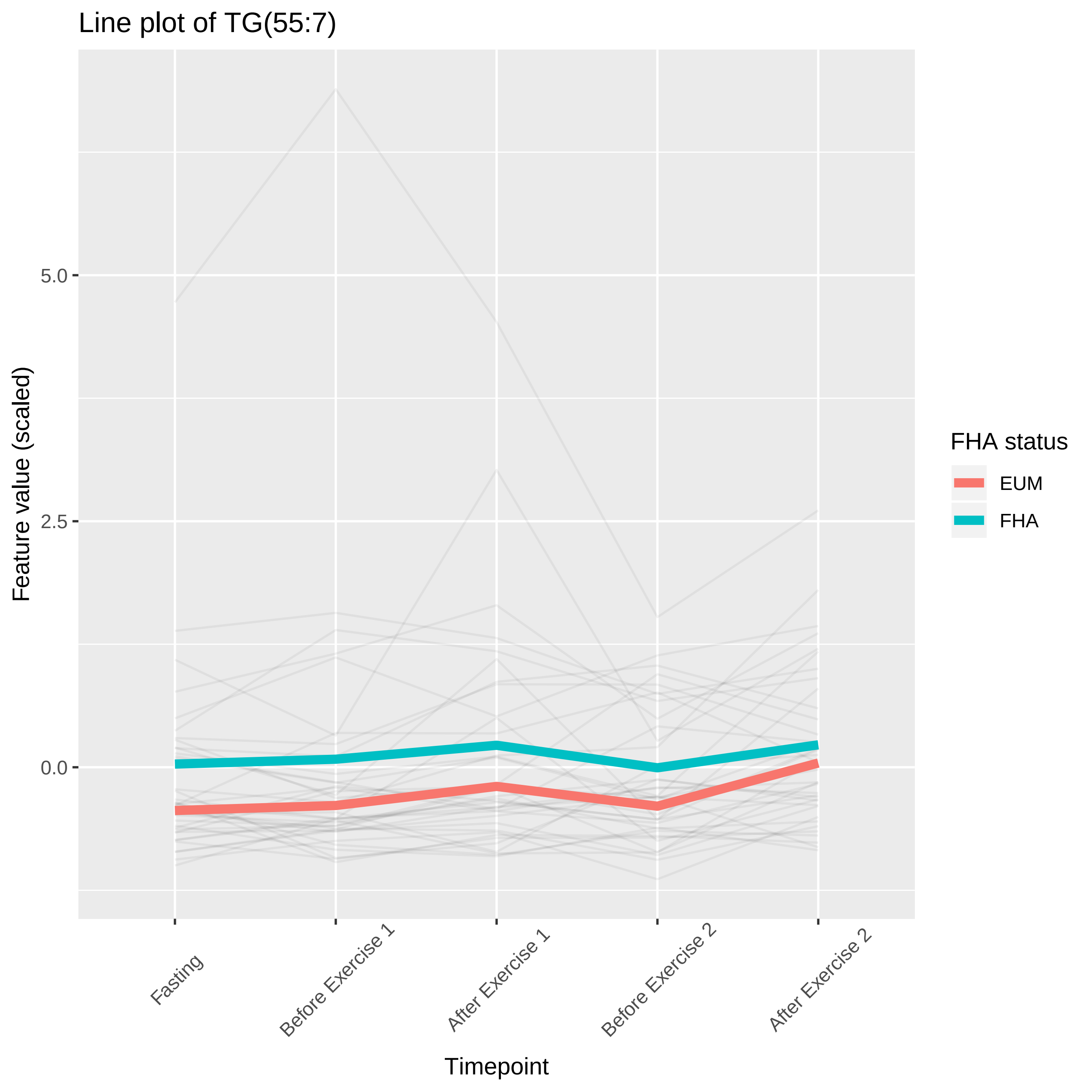

Line plot of TG(55:7)
Feature value (scaled)
5.0
2.5
0.0
Fasting
Before Exercise 1
After Exercise 1
Before Exercise 2
After Exercise 2
Timepoint
FHA status
EUM
FHA

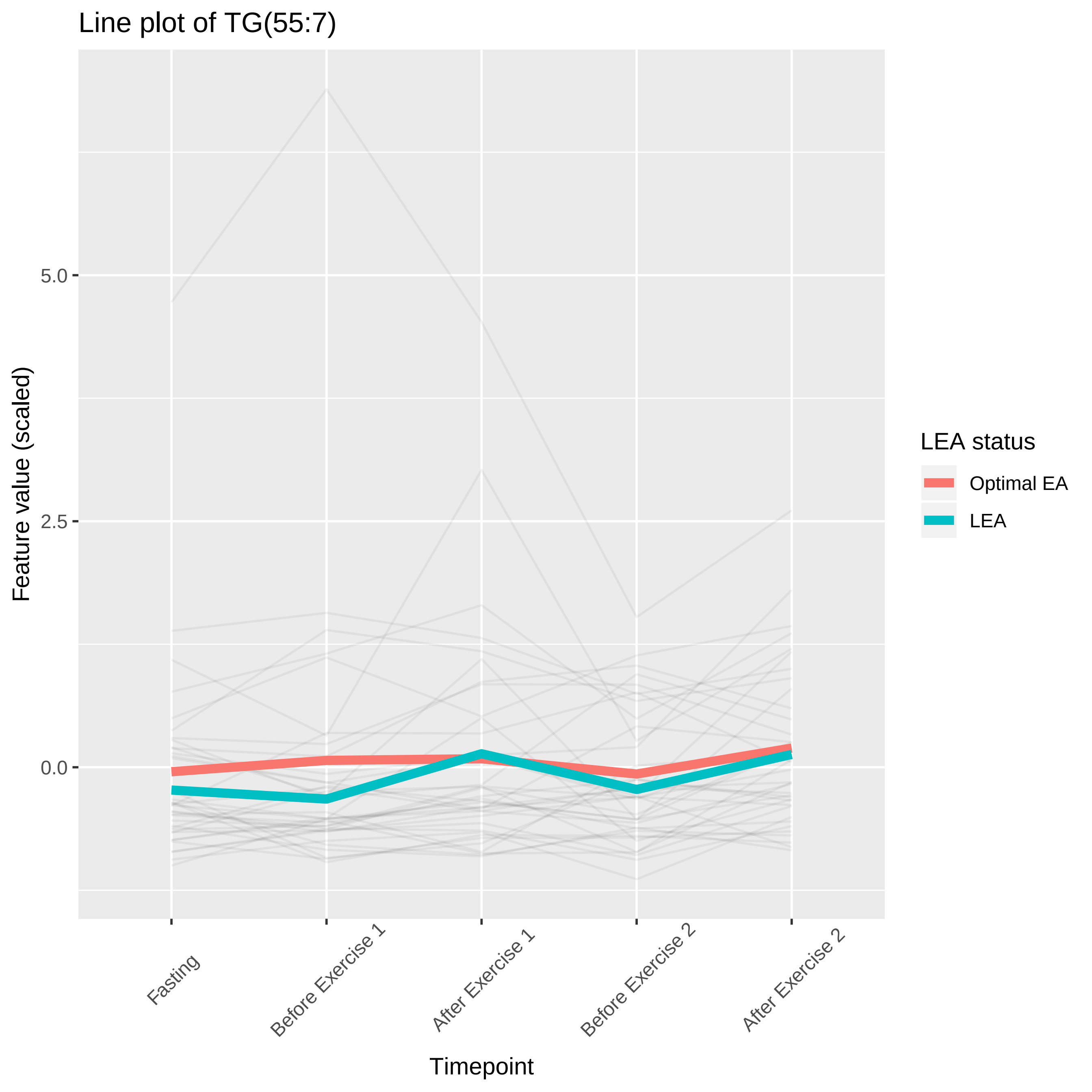

Line plot of TG(55:7)
Feature value (scaled)
5.0
2.5
0.0
Fasting
Before Exercise 1
After Exercise 1
Before Exercise 2
After Exercise 2
Timepoint
LEA status
Optimal EA
LEA

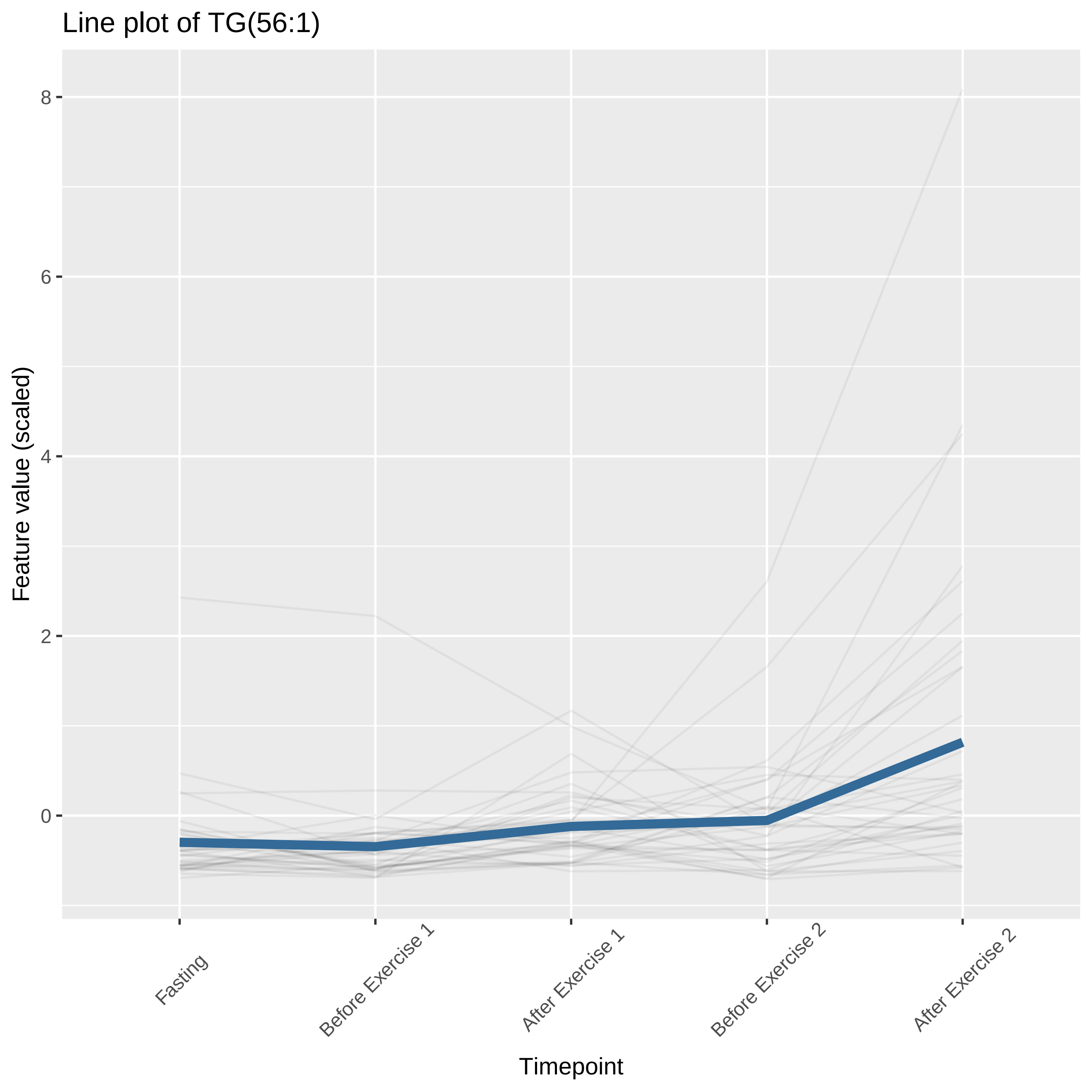

Line plot of TG(56:1)
Feature value (scaled)
8
6
4
2
0
Fasting
Before Exercise 1
After Exercise 1
Before Exercise 2
After Exercise 2
Timepoint

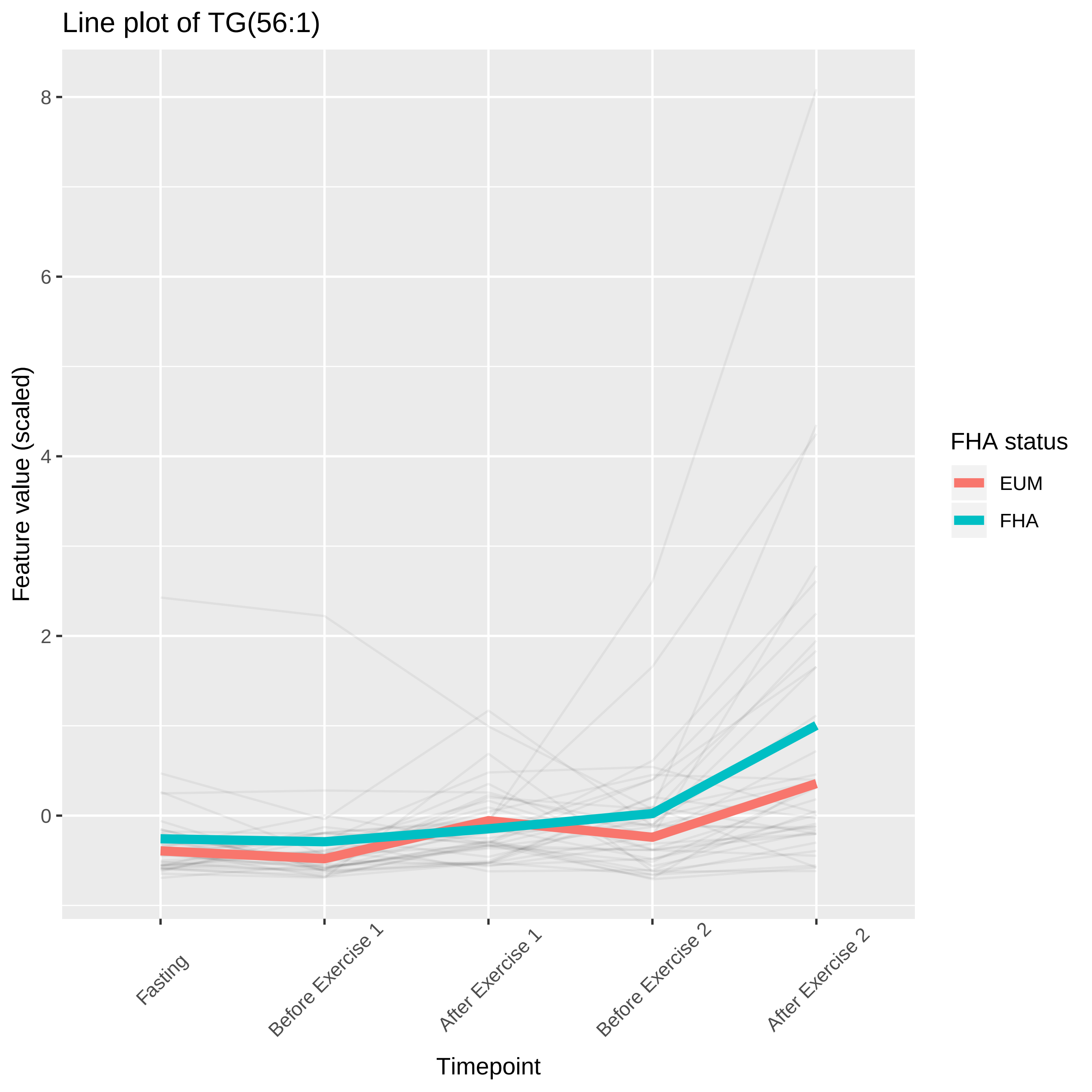

Line plot of TG(56:1)
Feature value (scaled)
8
6
4
2
0
Fasting
Before Exercise 1
After Exercise 1
Before Exercise 2
After Exercise 2
Timepoint
FHA status
EUM
FHA

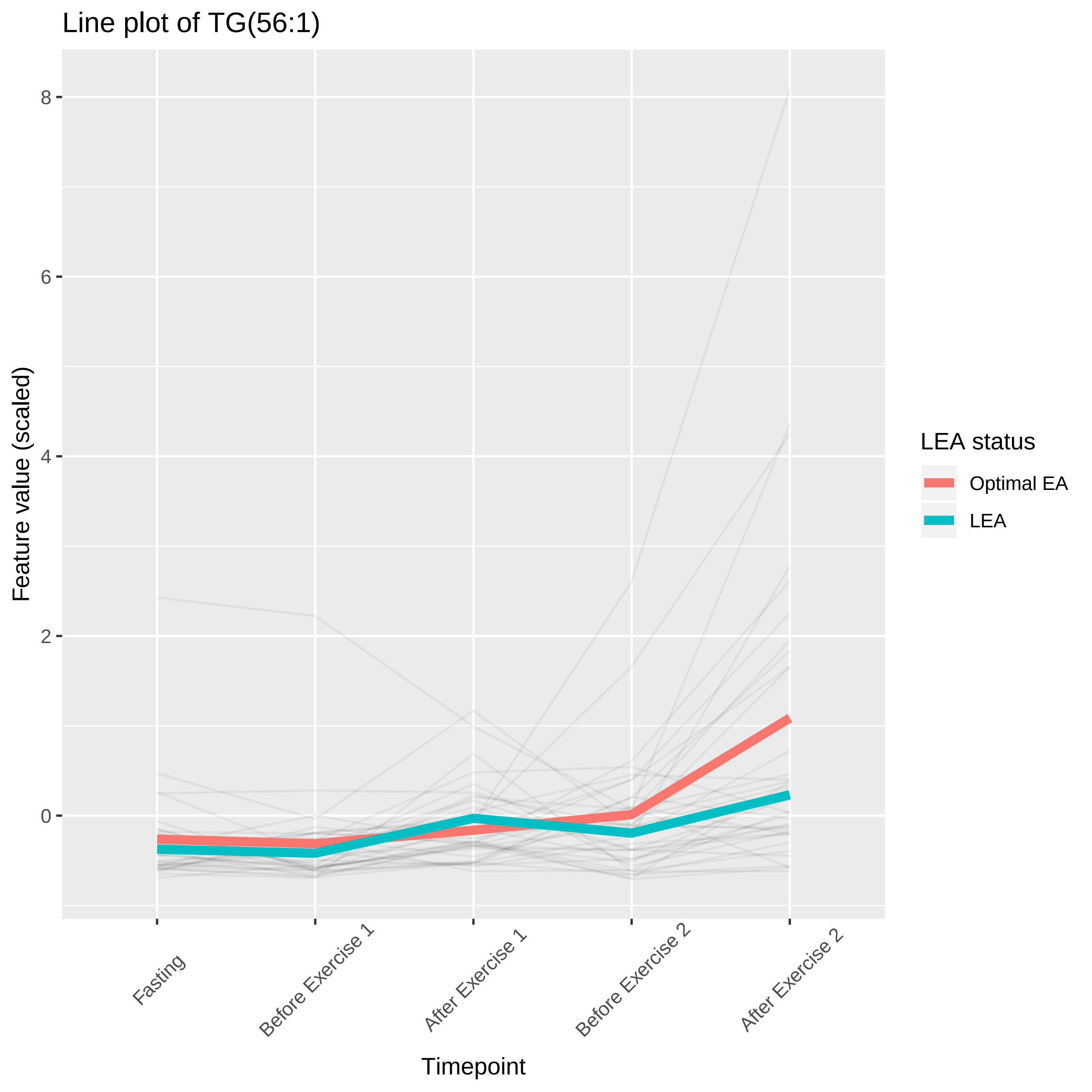
Line plot of TG(56:1)
Feature value (scaled)
8
6
4
2
0
Fasting
Before Exercise 1
After Exercise 1
Before Exercise 2
After Exercise 2
Timepoint
LEA status
Optimal EA
LEA

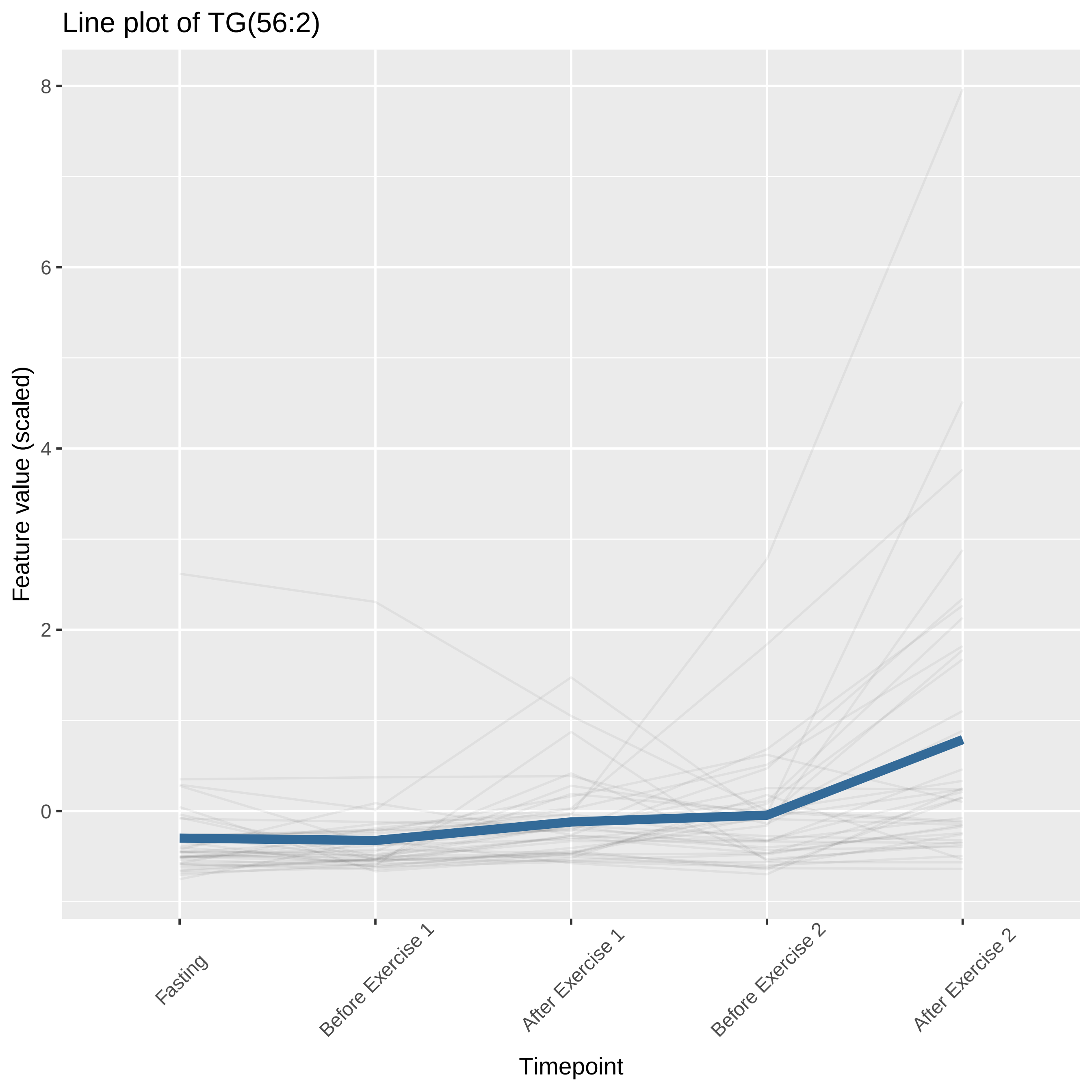
Line plot of TG(56:2)
Feature value (scaled)
8
6
4
2
0
Fasting
Before Exercise 1
After Exercise 1
Before Exercise 2
After Exercise 2
Timepoint

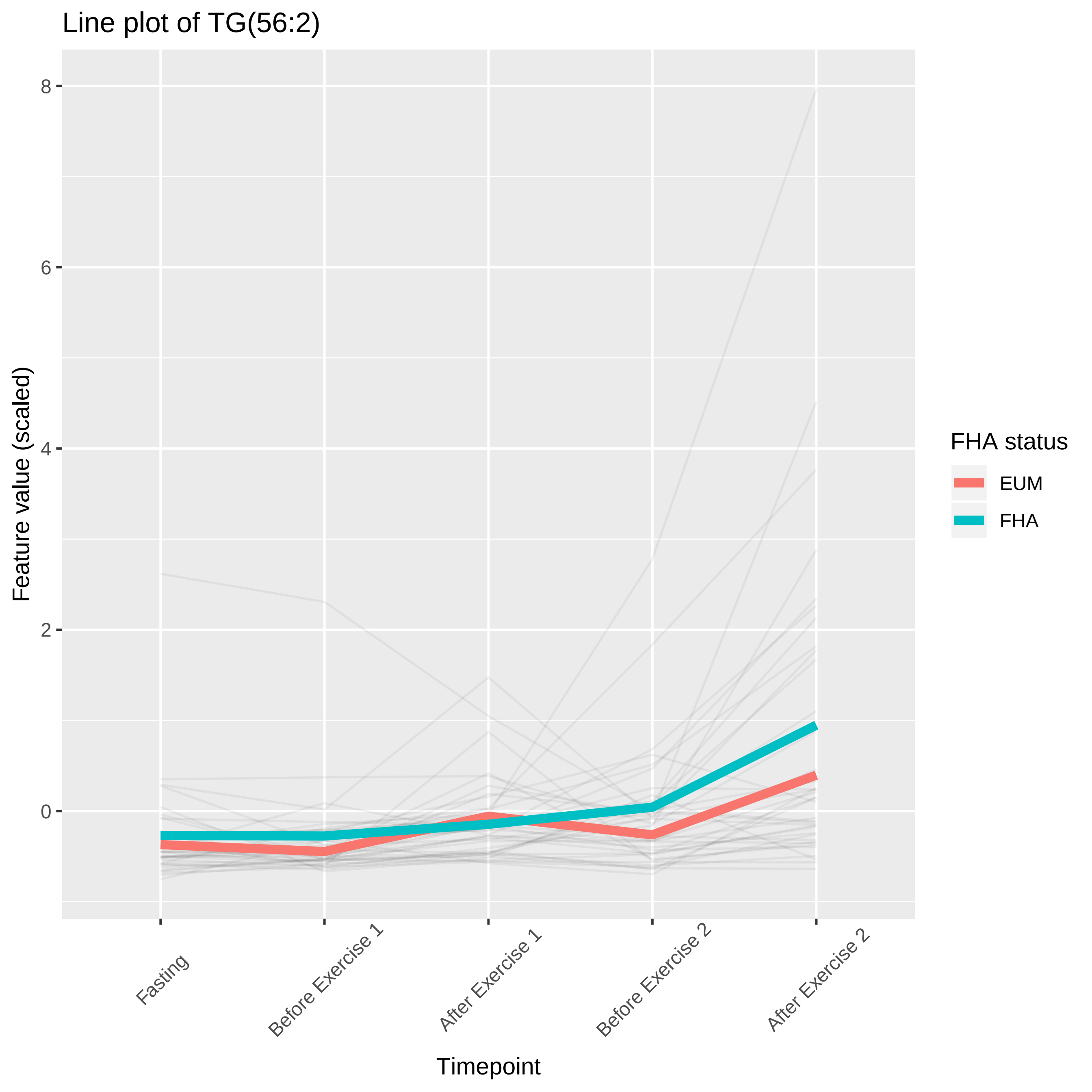

Line plot of TG(56:2)
Feature value (scaled)
8
6
4
2
0
Fasting
Before Exercise 1
After Exercise 1
Before Exercise 2
After Exercise 2
Timepoint
FHA status
EUM
FHA

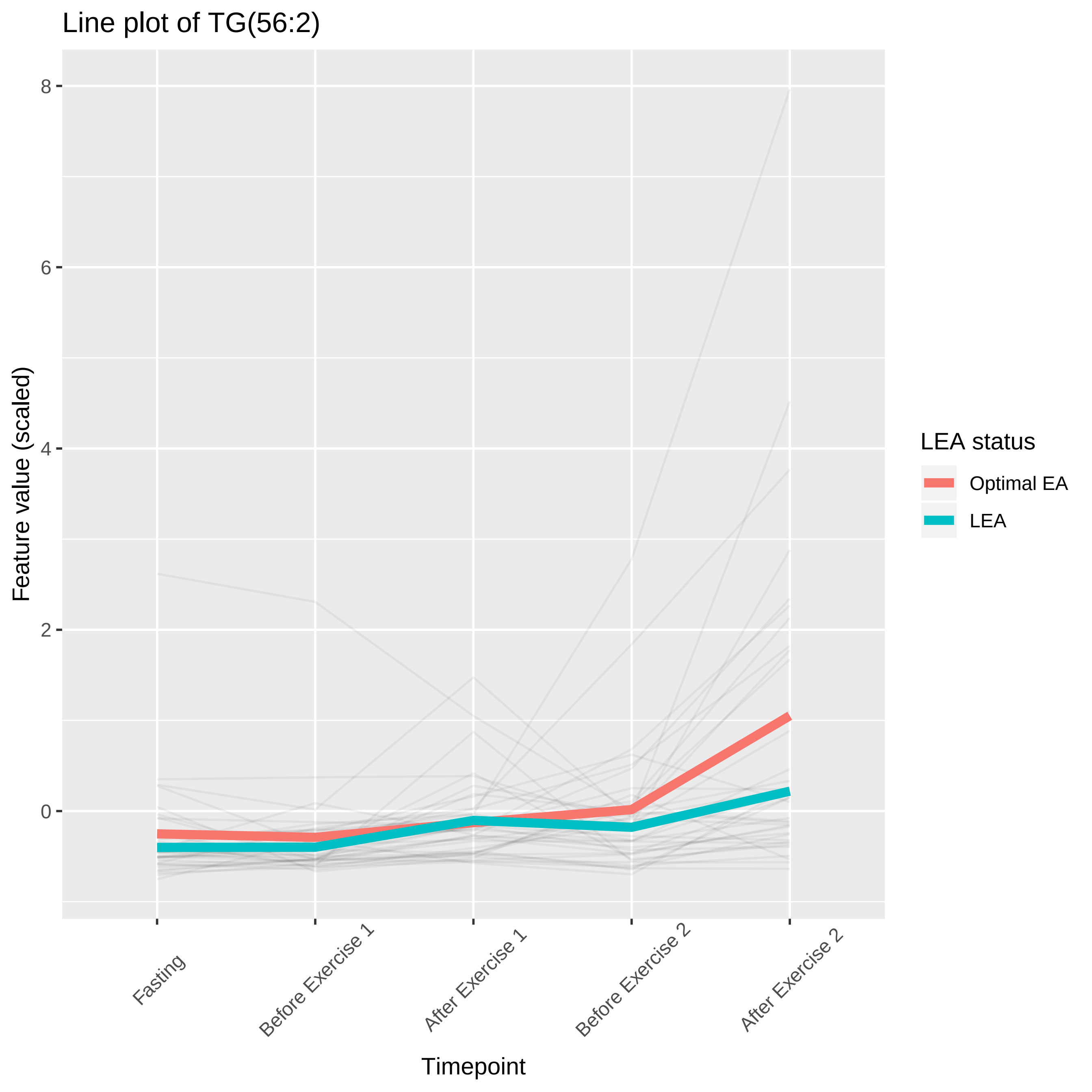

Line plot of TG(56:2)
Feature value (scaled)
8
6
4
2
0
Fasting
Before Exercise 1
After Exercise 1
Before Exercise 2
After Exercise 2
Timepoint
LEA status
Optimal EA
LEA

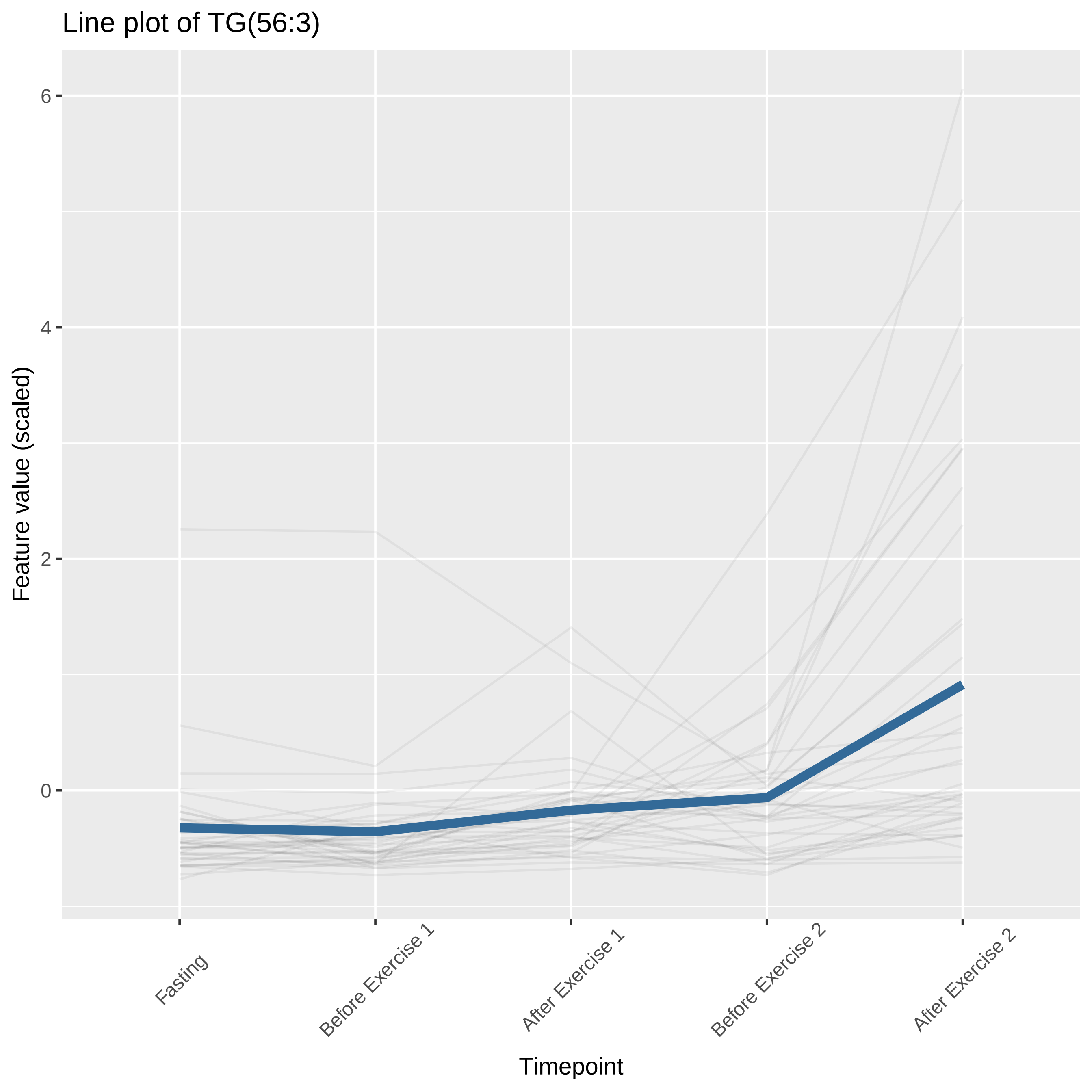

Line plot of TG(56:3)
Feature value (scaled)
6
4
2
0
Fasting
Before Exercise 1
After Exercise 1
Before Exercise 2
After Exercise 2
Timepoint

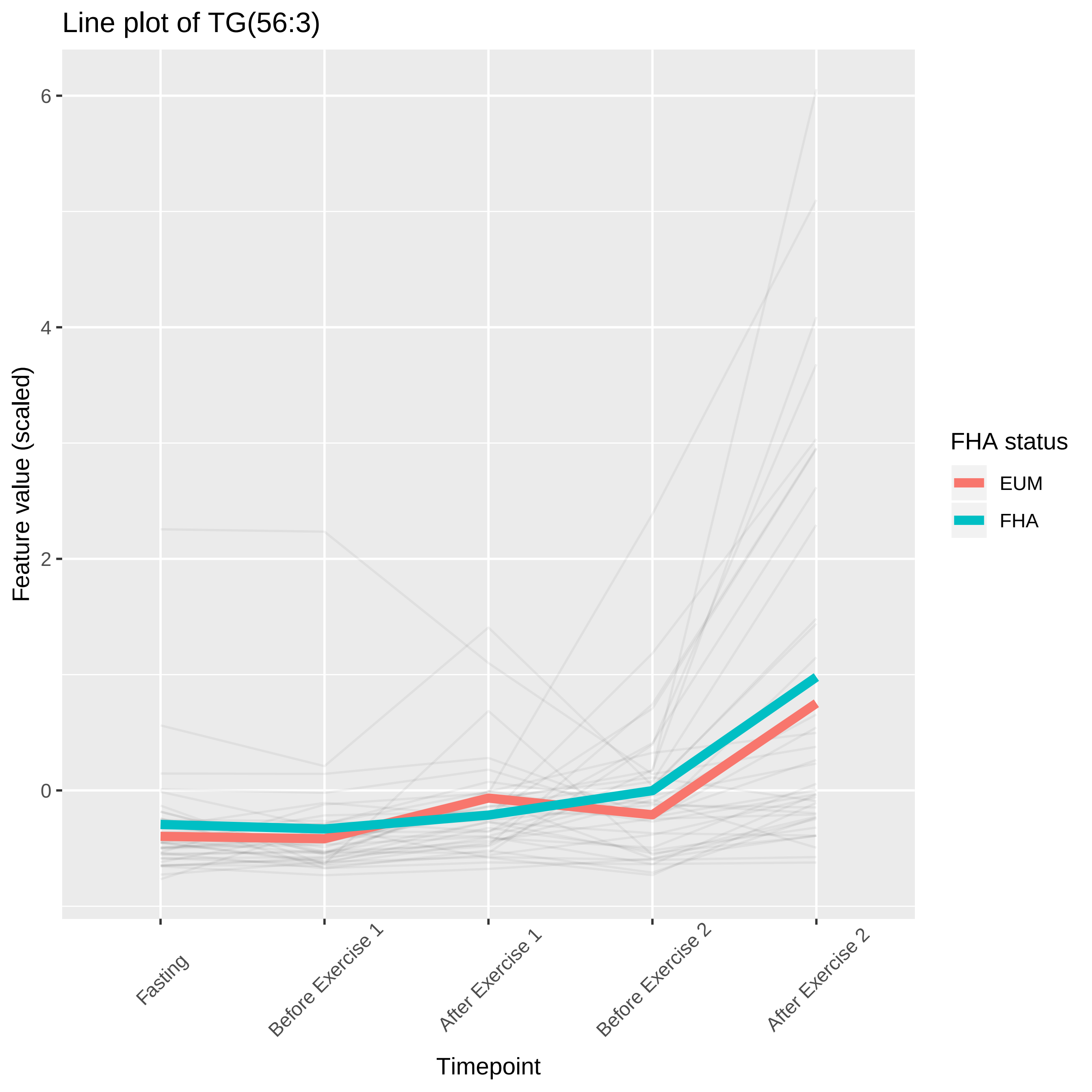

Line plot of TG(56:3)
Feature value (scaled)
6
4
2
0
Fasting
Before Exercise 1
After Exercise 1
Before Exercise 2
After Exercise 2
Timepoint
FHA status
EUM
FHA

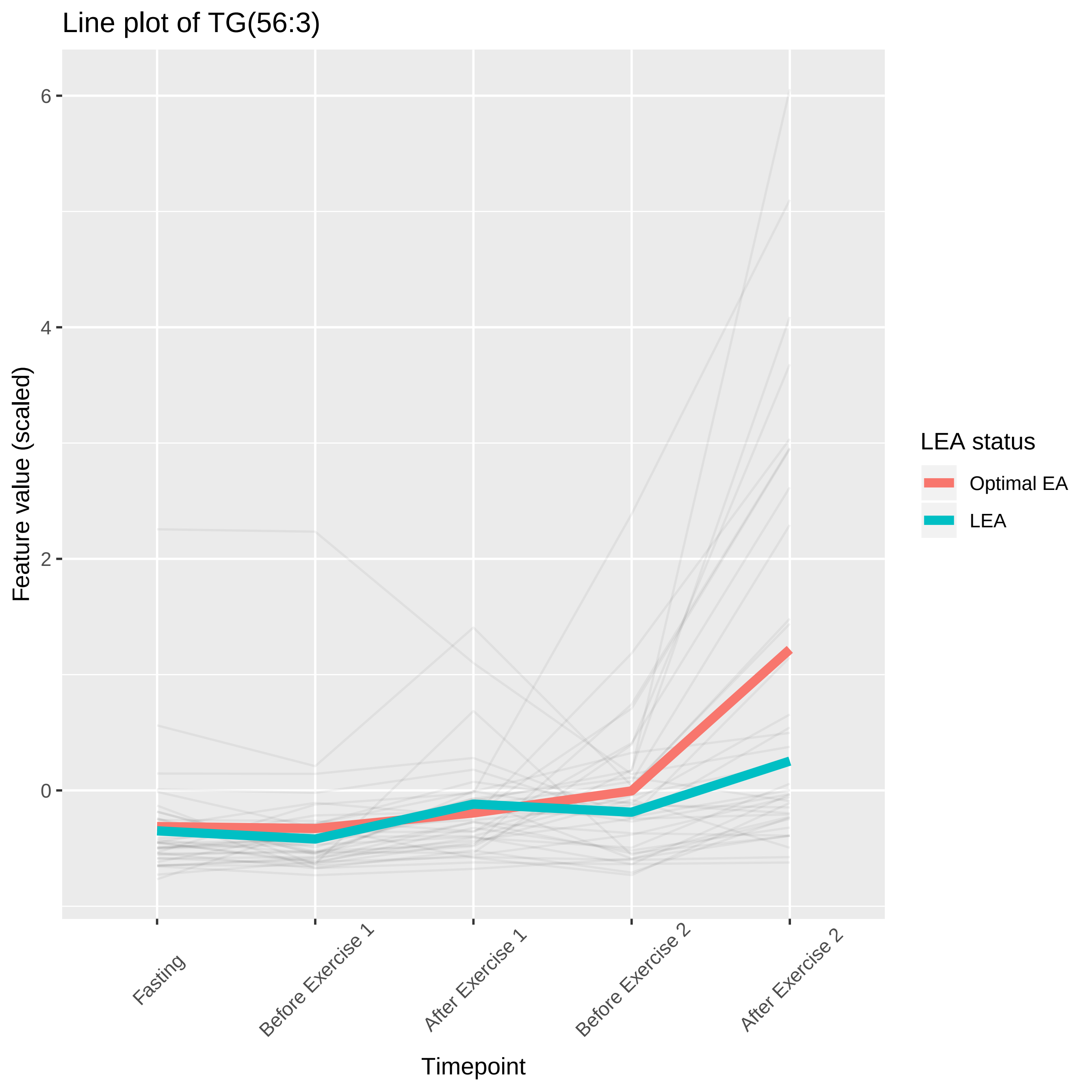

Line plot of TG(56:3)
Feature value (scaled)
6
4
2
0
Fasting
Before Exercise 1
After Exercise 1
Before Exercise 2
After Exercise 2
Timepoint
LEA status
Optimal EA
LEA

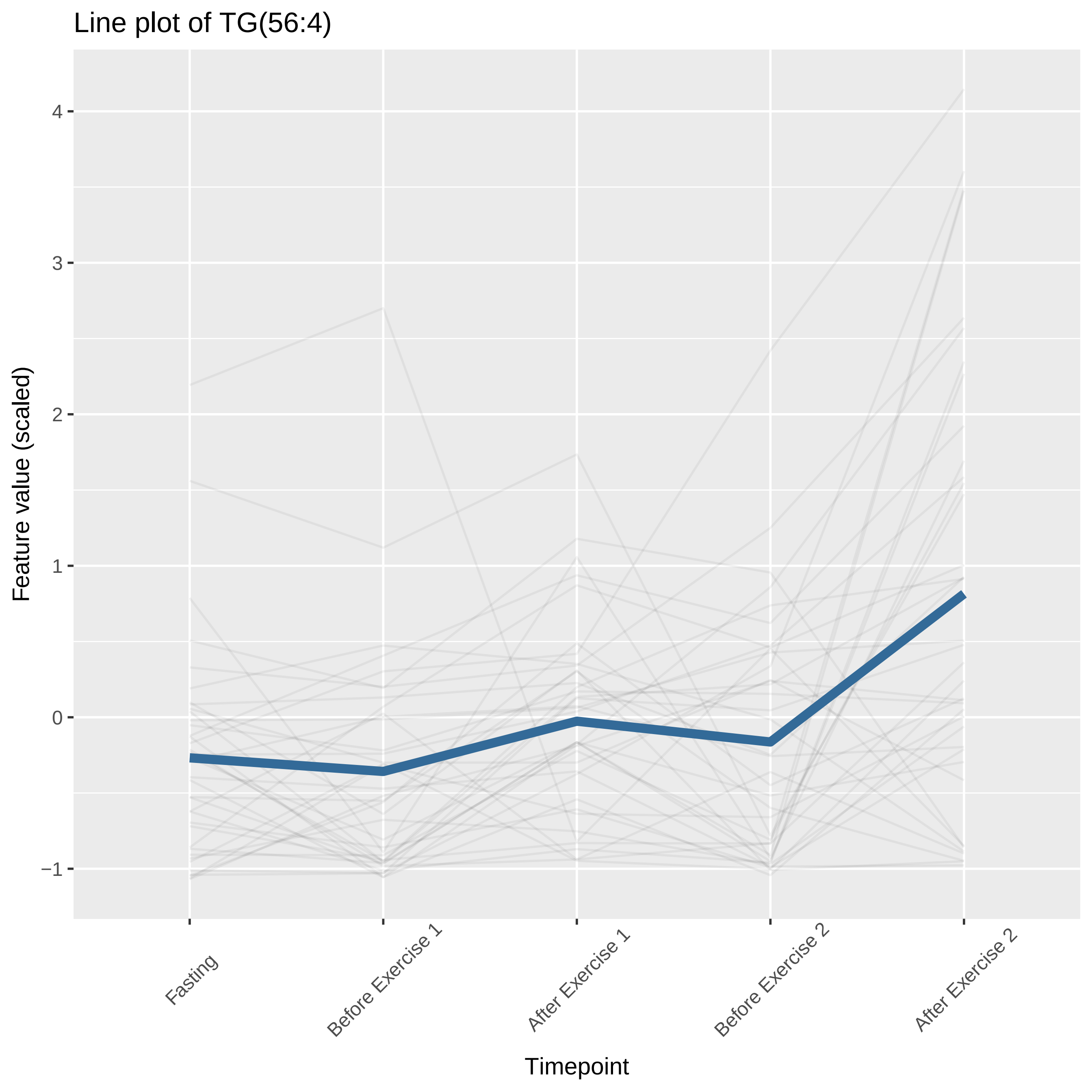

Line plot of TG(56:4)
Feature value (scaled)
4
3
2
1
0
−1
Fasting
Before Exercise 1
After Exercise 1
Before Exercise 2
After Exercise 2
Timepoint

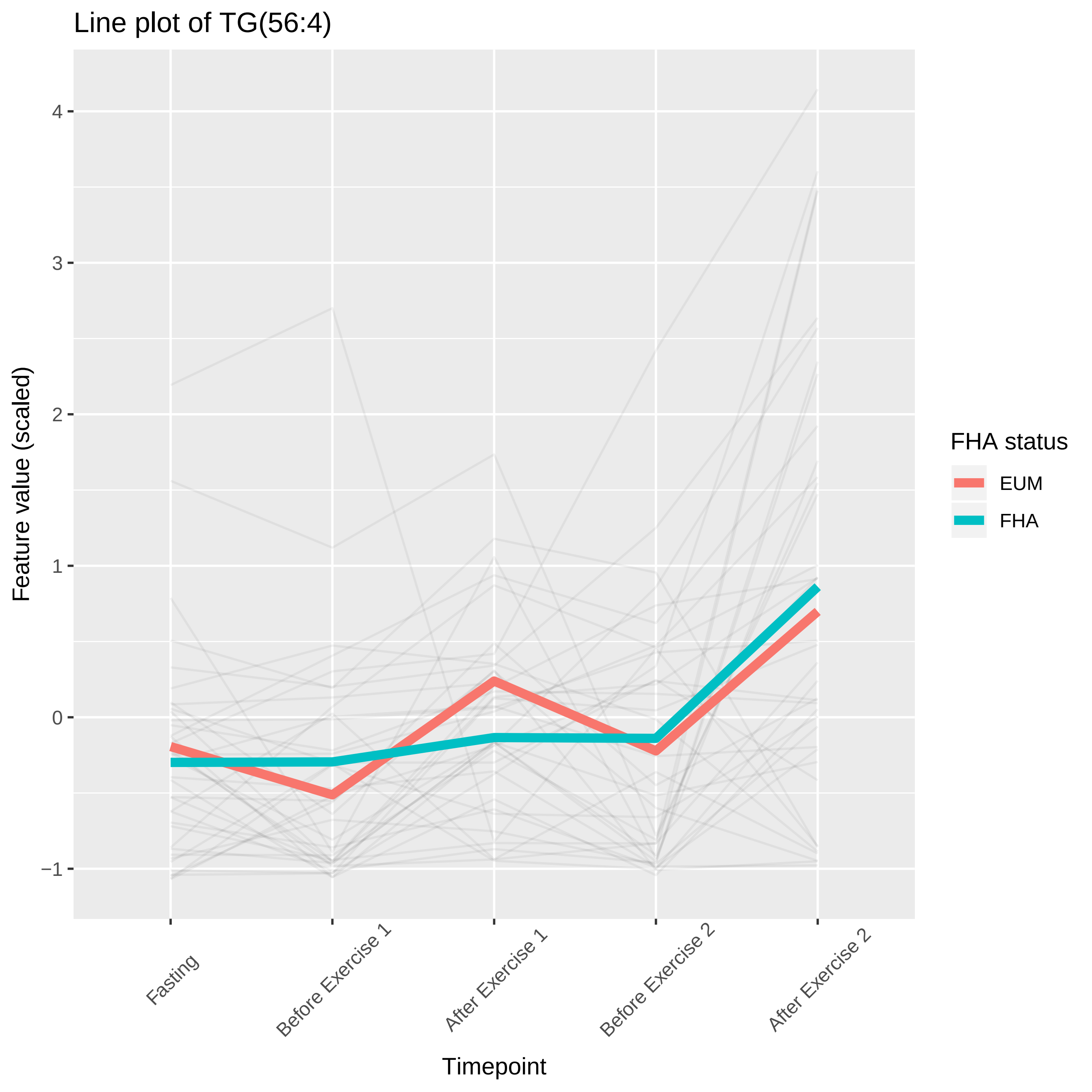

Line plot of TG(56:4)
Feature value (scaled)
4
3
2
1
0
−1
Fasting
Before Exercise 1
After Exercise 1
Before Exercise 2
After Exercise 2
Timepoint
FHA status
EUM
FHA

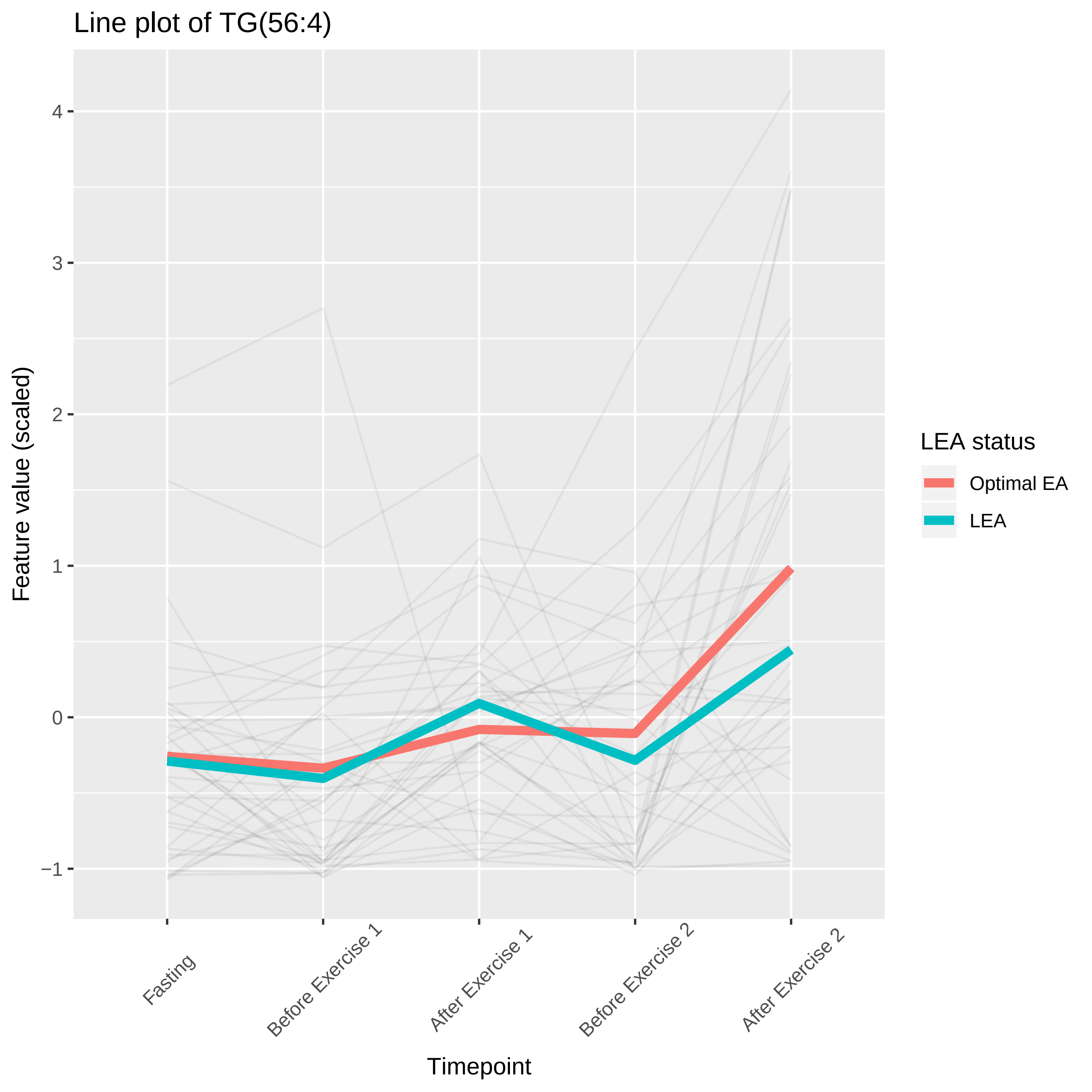

Line plot of TG(56:4)
Feature value (scaled)
4
3
2
1
0
−1
Fasting
Before Exercise 1
After Exercise 1
Before Exercise 2
After Exercise 2
Timepoint
LEA status
Optimal EA
LEA

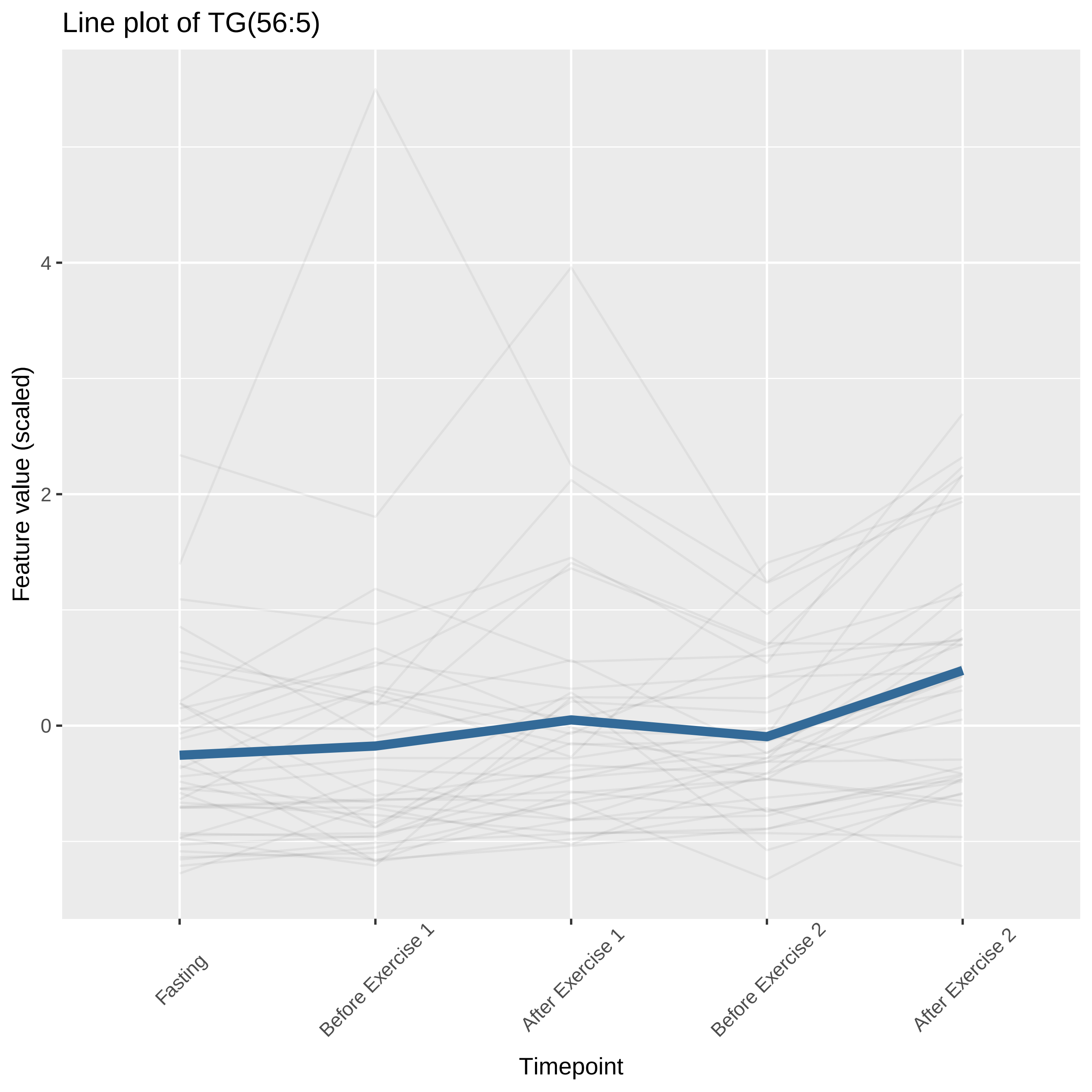

Line plot of TG(56:5)
Feature value (scaled)
4
2
0
Fasting
Before Exercise 1
After Exercise 1
Before Exercise 2
After Exercise 2
Timepoint

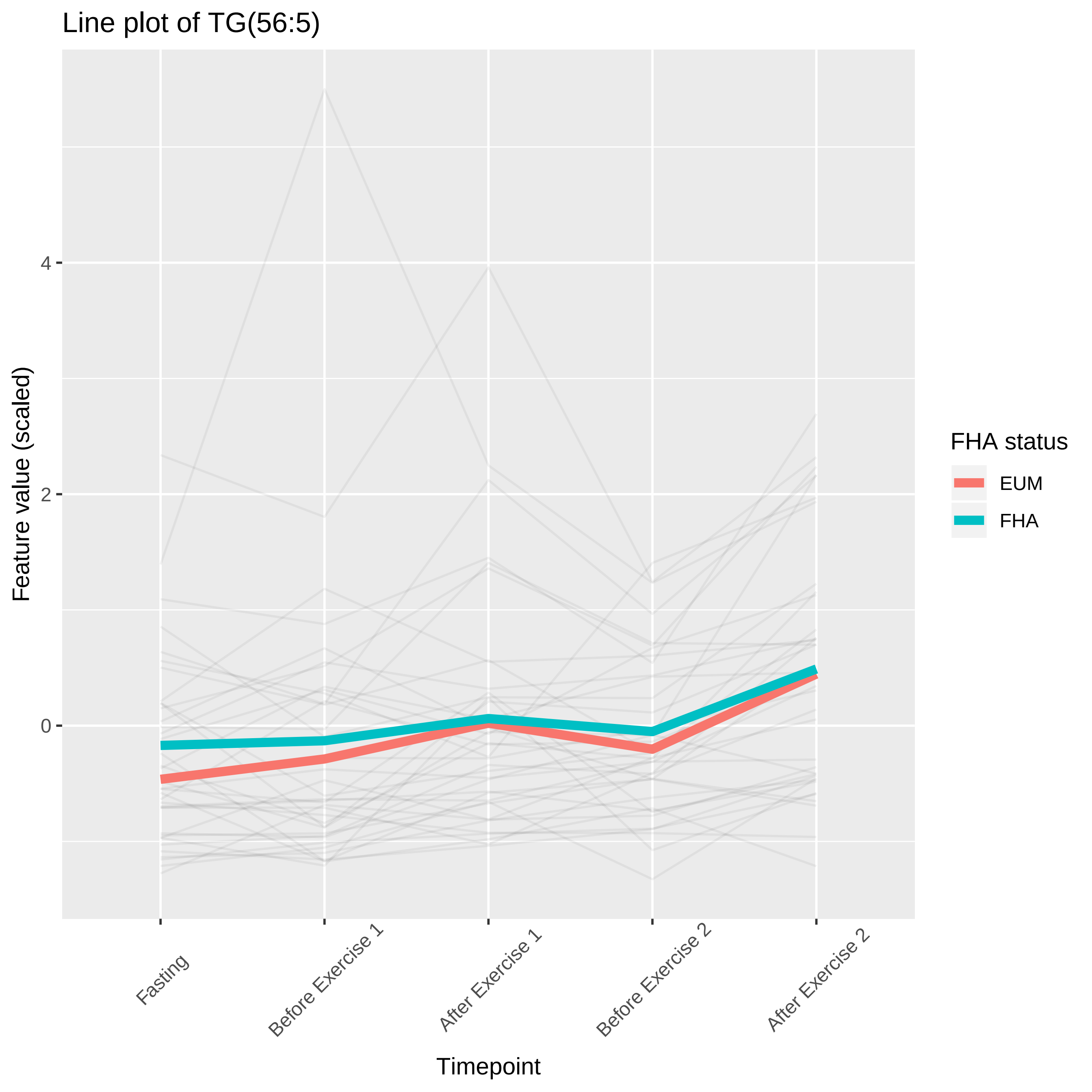
Line plot of TG(56:5)
Feature value (scaled)
4
2
0
Fasting
Before Exercise 1
After Exercise 1
Before Exercise 2
After Exercise 2
Timepoint
FHA status
EUM
FHA

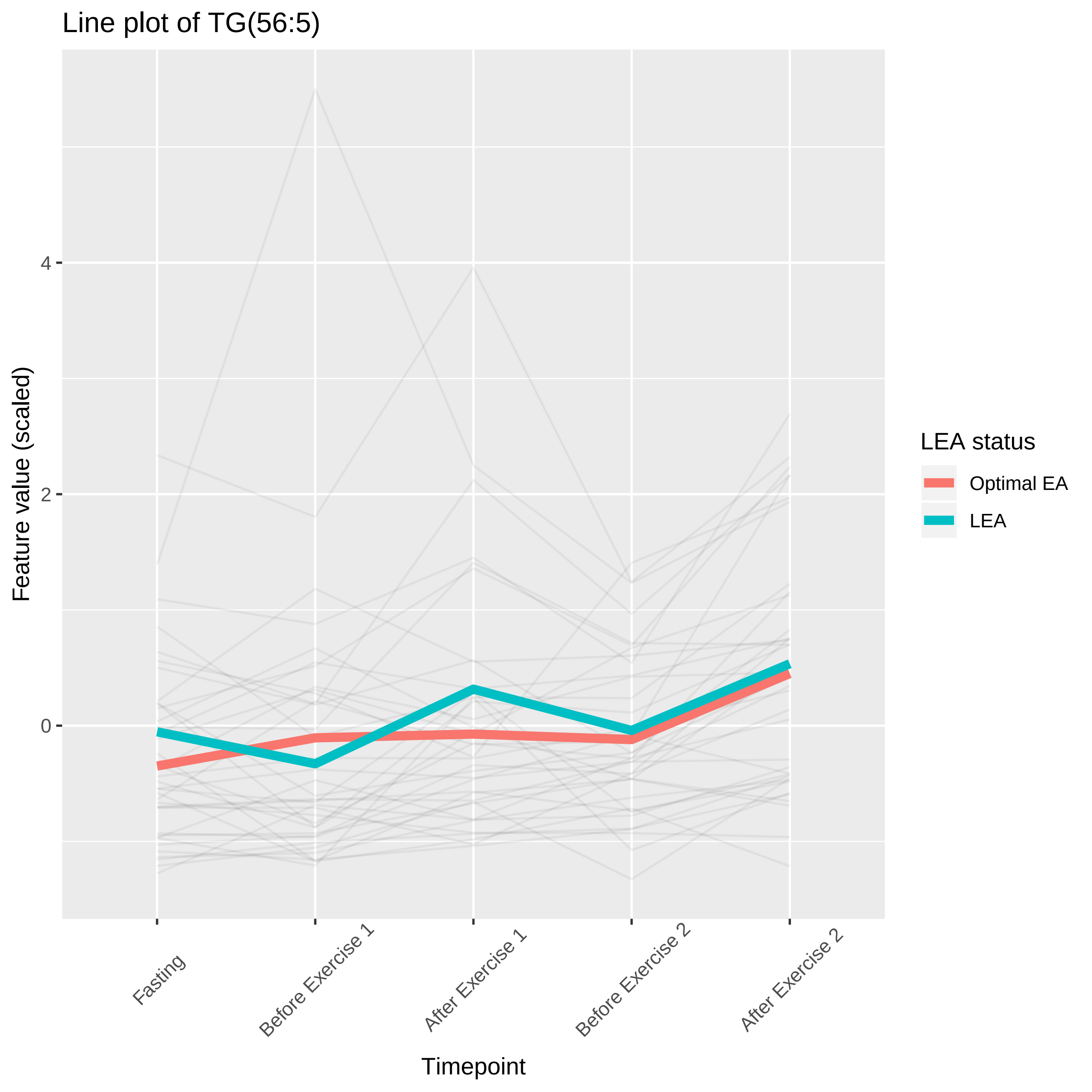

Line plot of TG(56:5)
Feature value (scaled)
4
2
0
Fasting
Before Exercise 1
After Exercise 1
Before Exercise 2
After Exercise 2
Timepoint
LEA status
Optimal EA
LEA

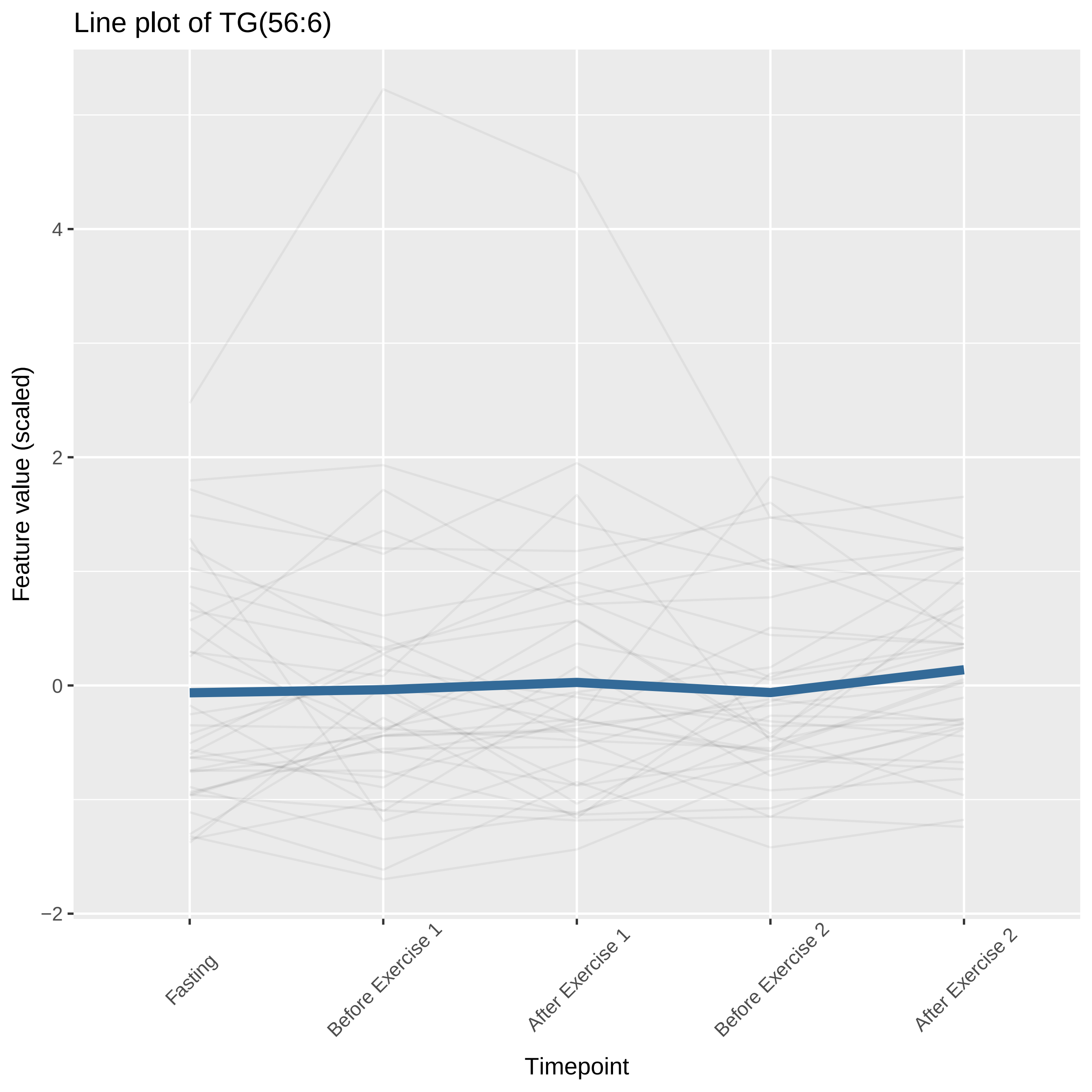

Line plot of TG(56:6)
Feature value (scaled)
4
2
0
−2
Fasting
Before Exercise 1
After Exercise 1
Before Exercise 2
After Exercise 2
Timepoint

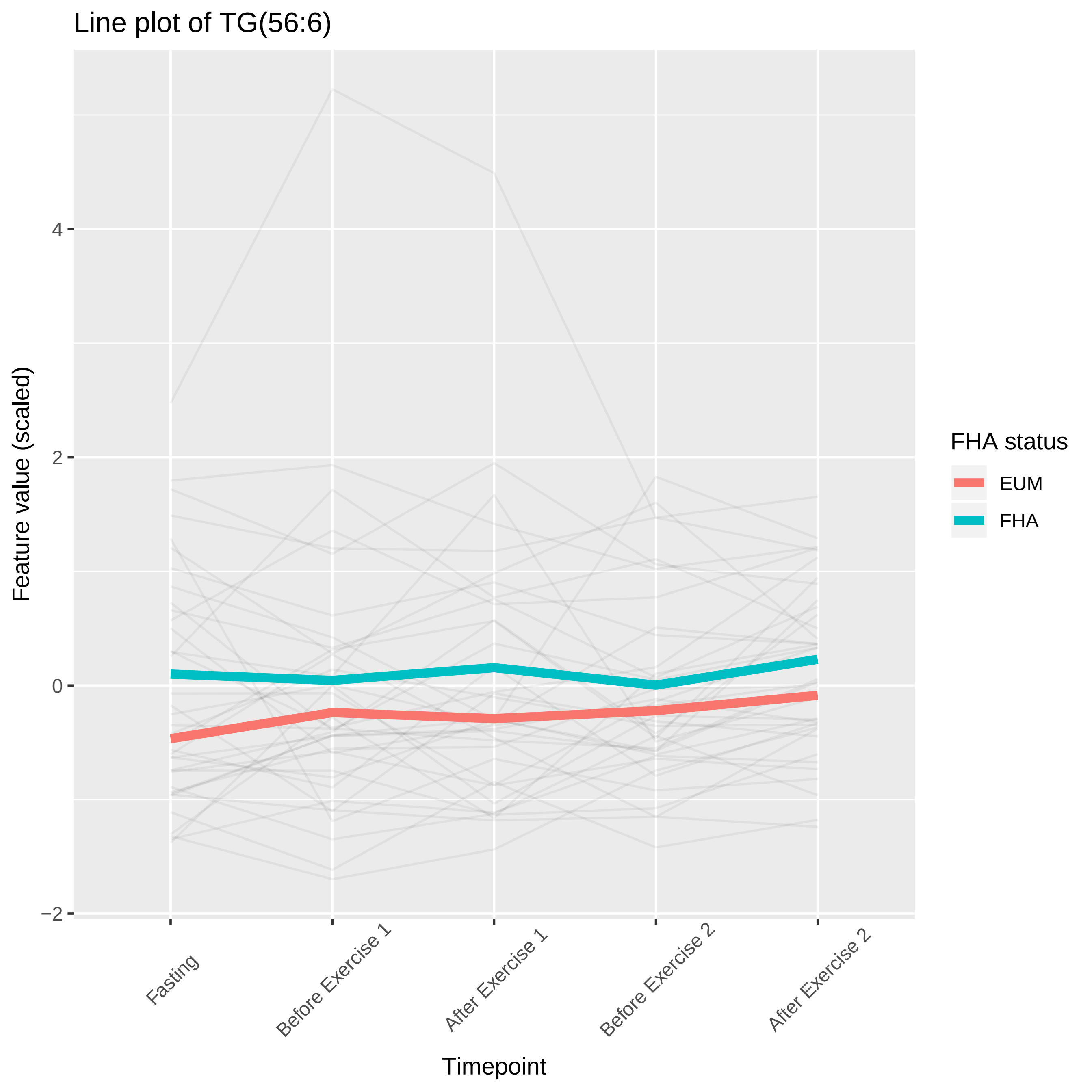

Line plot of TG(56:6)
Feature value (scaled)
4
2
0
−2
Fasting
Before Exercise 1
After Exercise 1
Before Exercise 2
After Exercise 2
Timepoint
FHA status
EUM
FHA

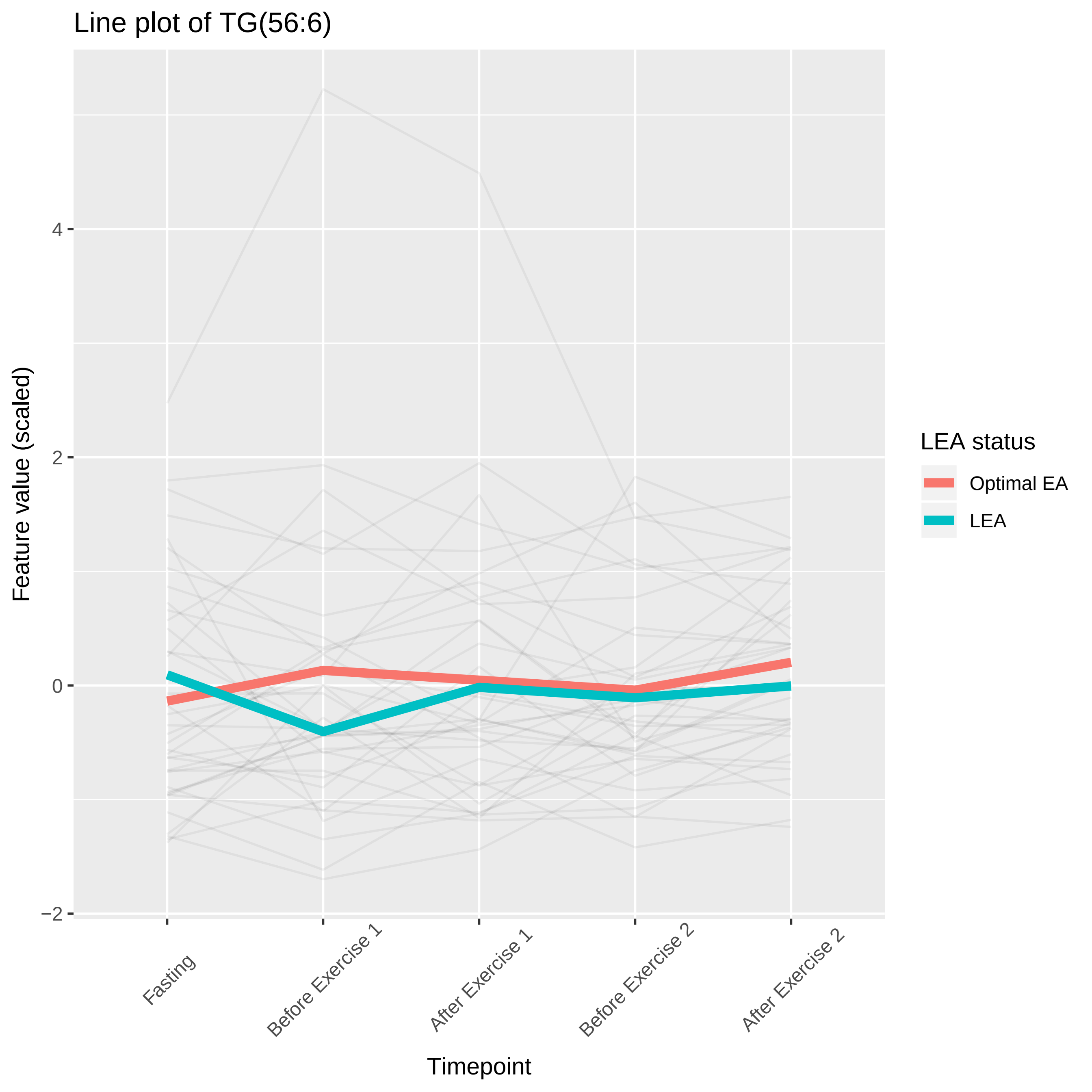

Line plot of TG(56:6)
Feature value (scaled)
4
2
0
−2
Fasting
Before Exercise 1
After Exercise 1
Before Exercise 2
After Exercise 2
Timepoint
LEA status
Optimal EA
LEA

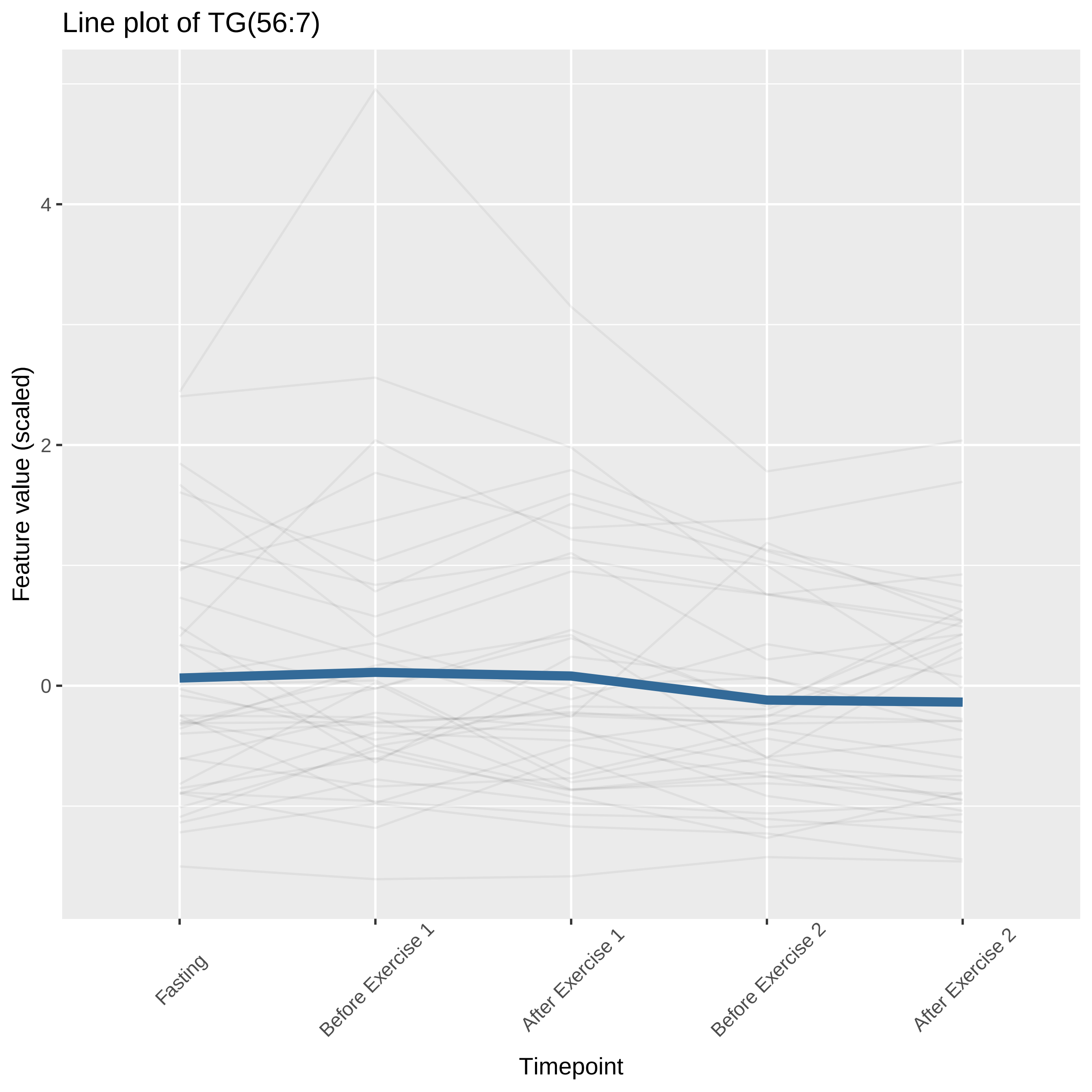
Line plot of TG(56:7)
Feature value (scaled)
4
2
0
Fasting
Before Exercise 1
After Exercise 1
Before Exercise 2
After Exercise 2
Timepoint

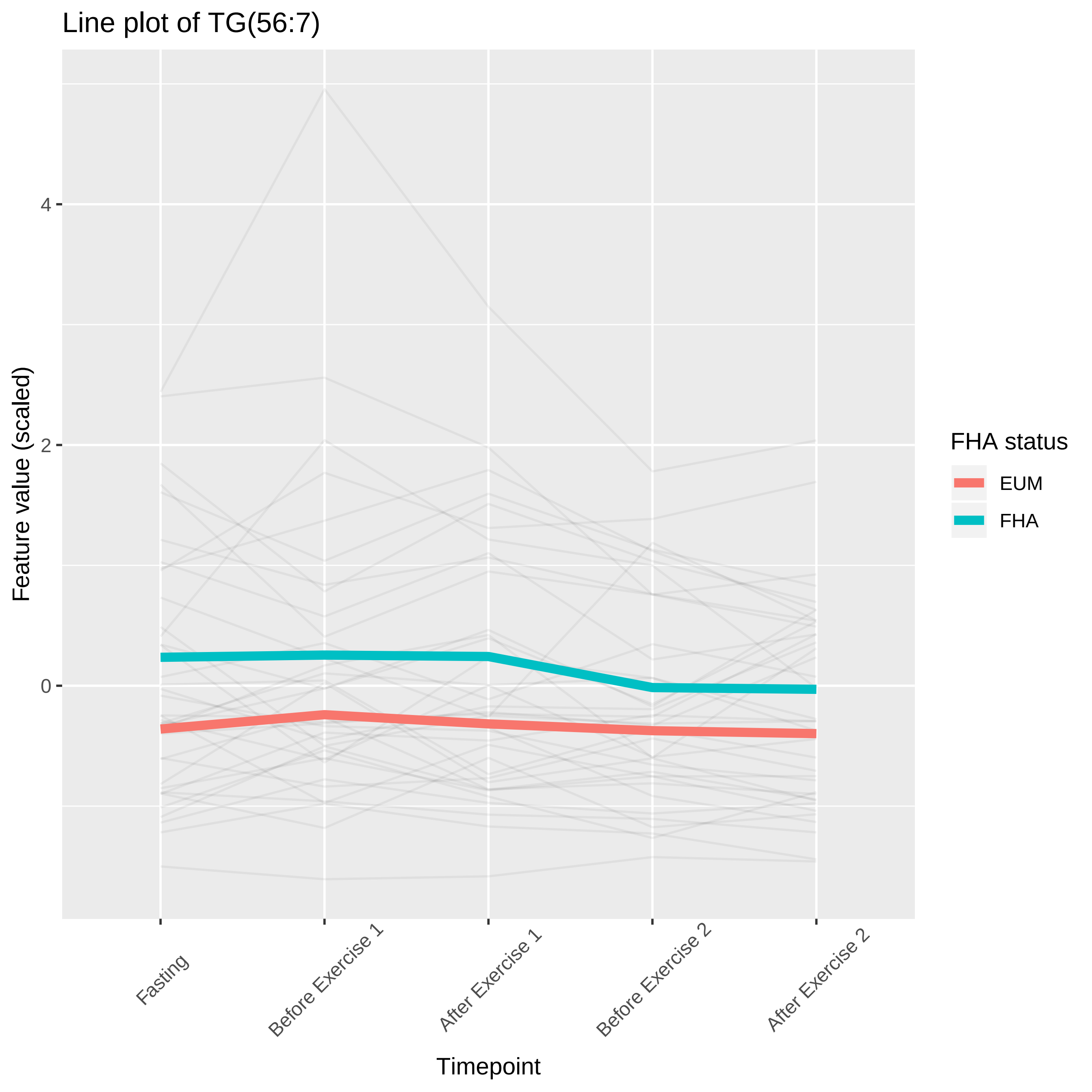

Line plot of TG(56:7)
Feature value (scaled)
4
2
0
Fasting
Before Exercise 1
After Exercise 1
Before Exercise 2
After Exercise 2
Timepoint
FHA status
EUM
FHA

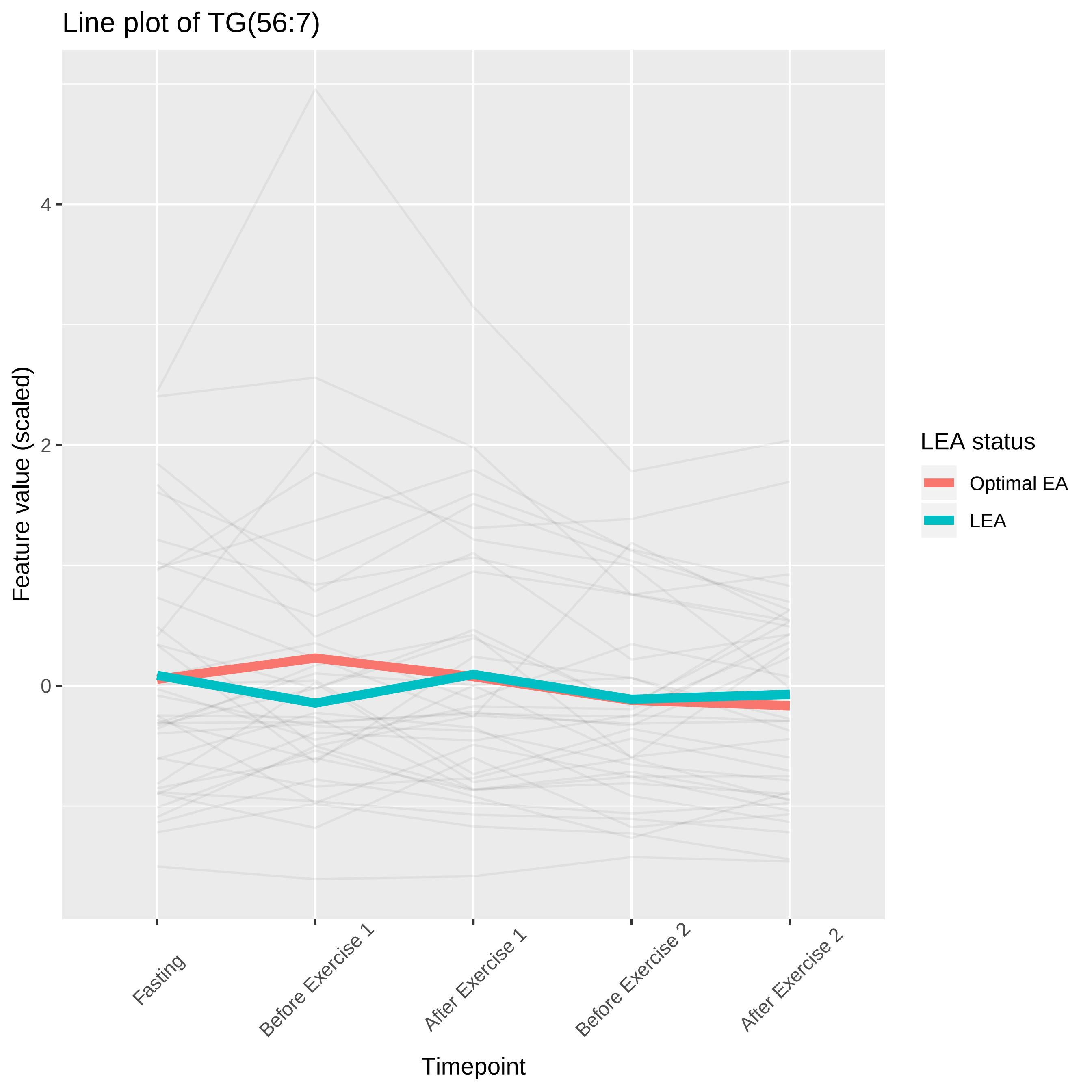

Line plot of TG(56:7)
Feature value (scaled)
4
2
0
Fasting
Before Exercise 1
After Exercise 1
Before Exercise 2
After Exercise 2
Timepoint
LEA status
Optimal EA
LEA

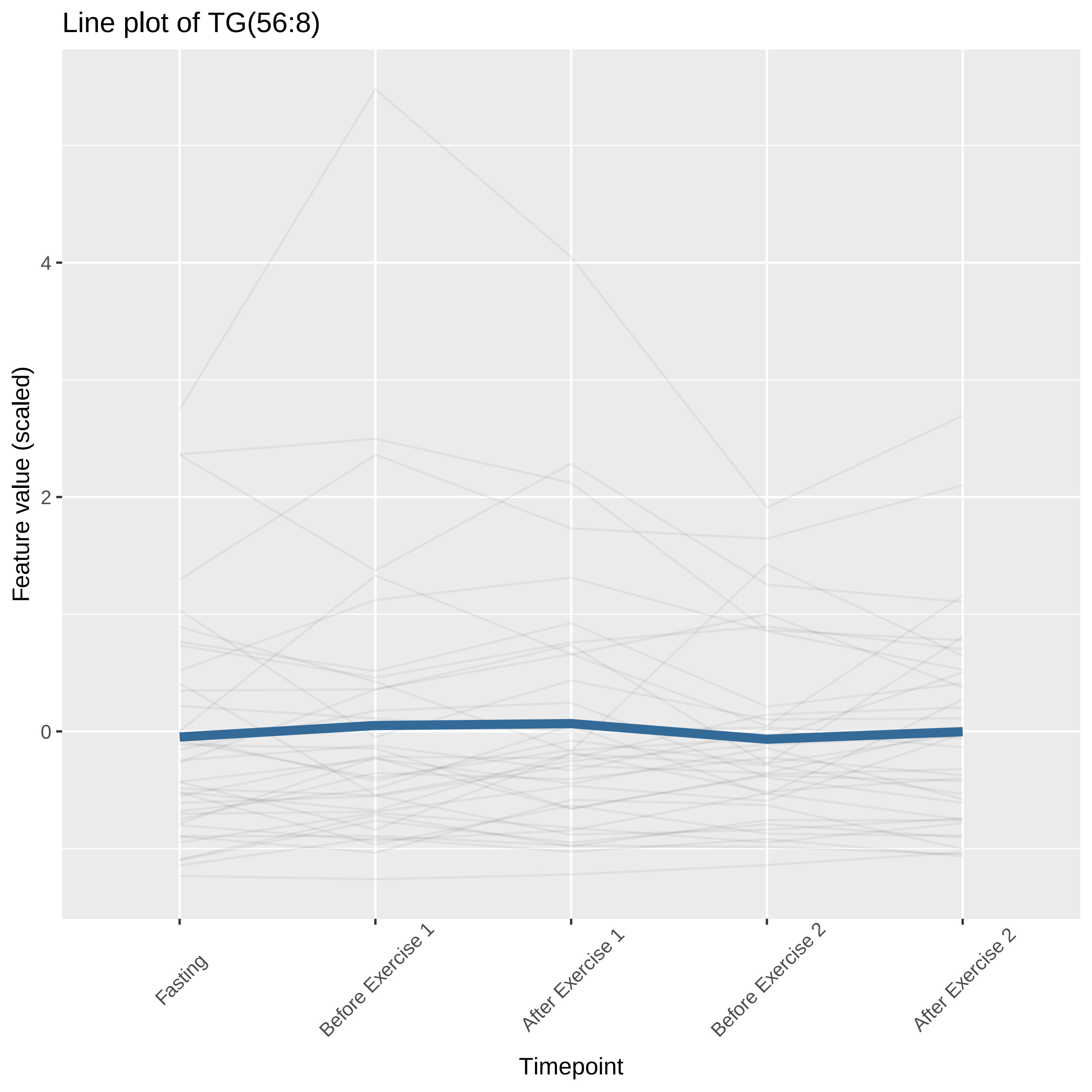
Line plot of TG(56:8)
Feature value (scaled)
4
2
0
Fasting
Before Exercise 1
After Exercise 1
Before Exercise 2
After Exercise 2
Timepoint

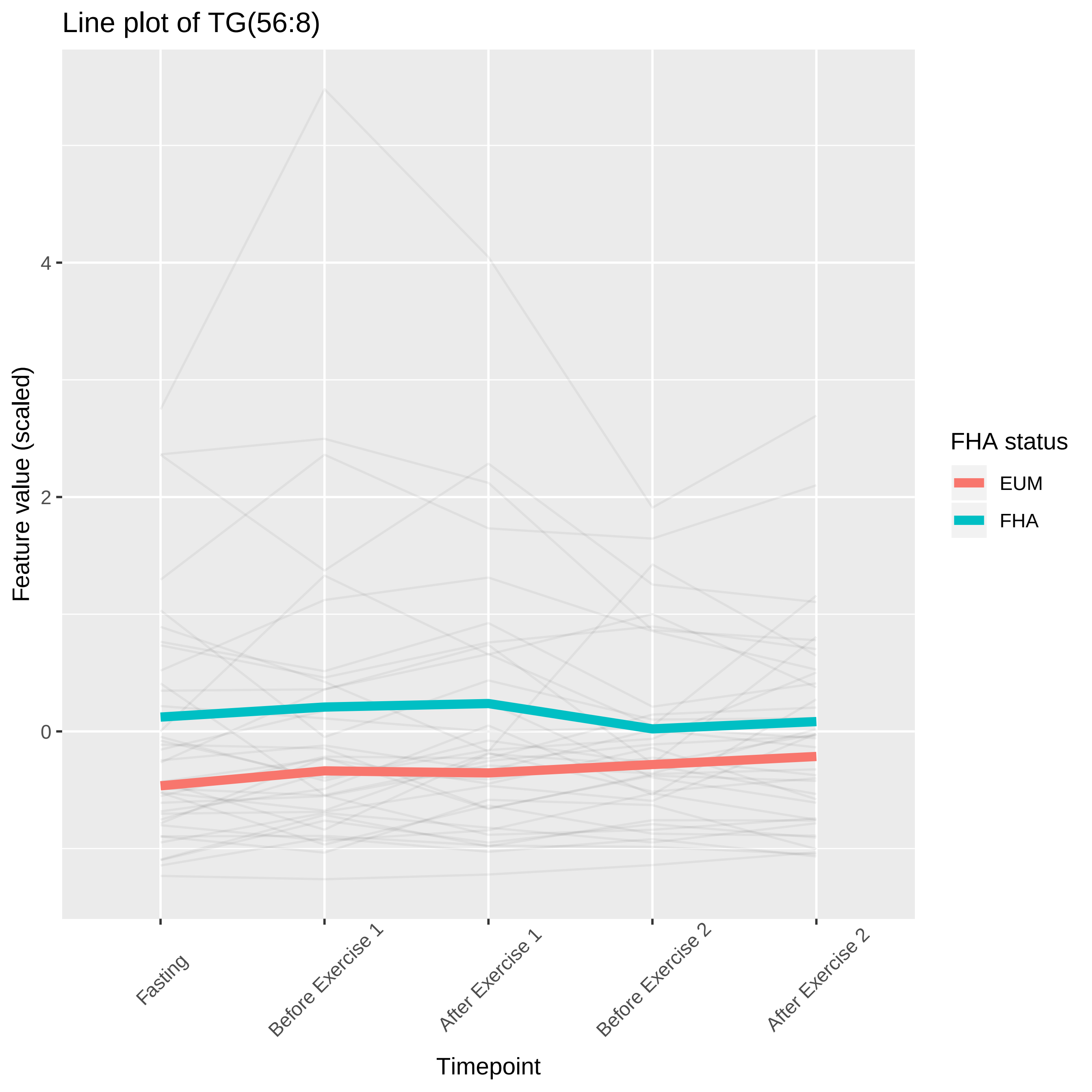

Line plot of TG(56:8)
Feature value (scaled)
4
2
0
Fasting
Before Exercise 1
After Exercise 1
Before Exercise 2
After Exercise 2
Timepoint
FHA status
EUM
FHA

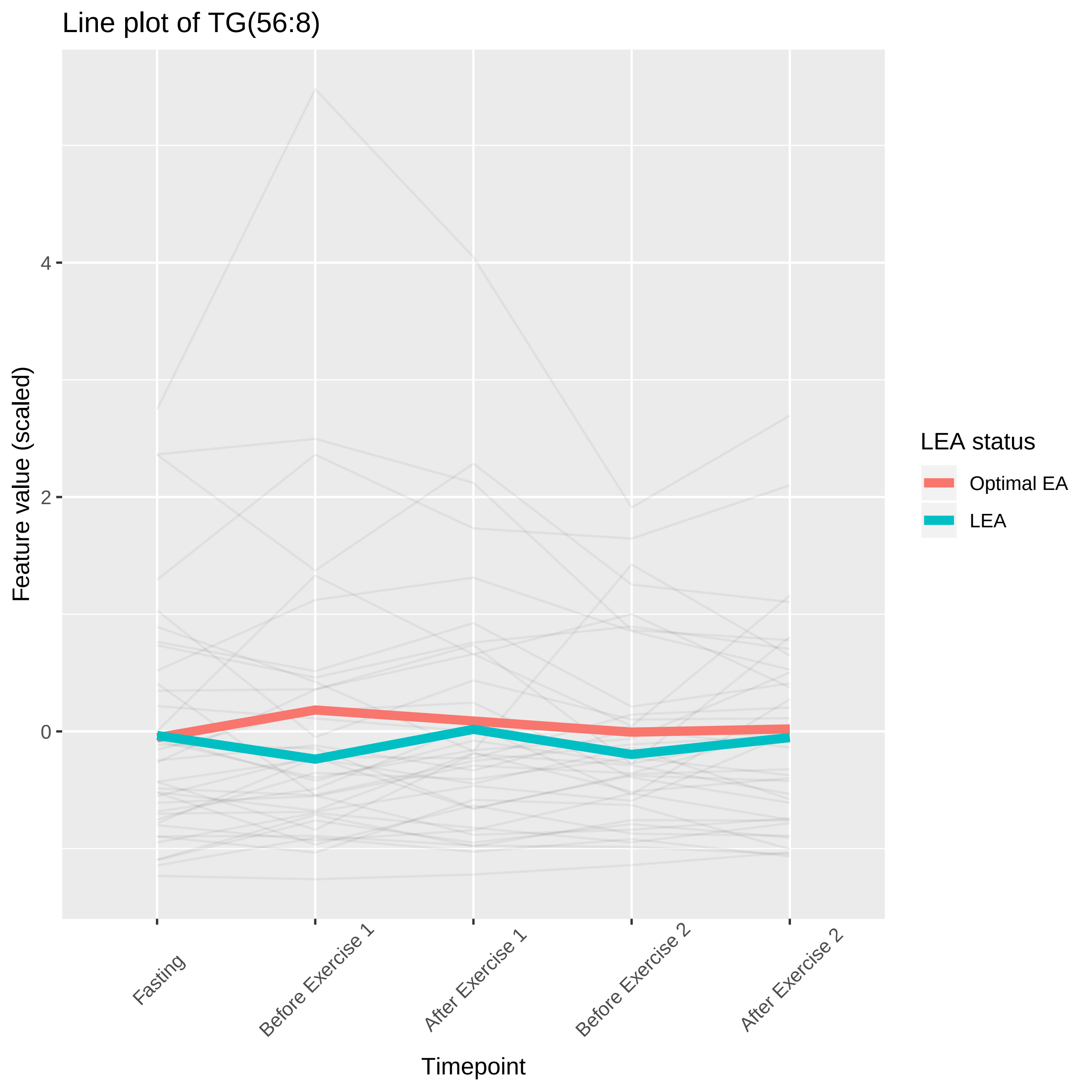
Line plot of TG(56:8)
Feature value (scaled)
4
2
0
Fasting
Before Exercise 1
After Exercise 1
Before Exercise 2
After Exercise 2
Timepoint
LEA status
Optimal EA
LEA

Line plot of TG(56:9)

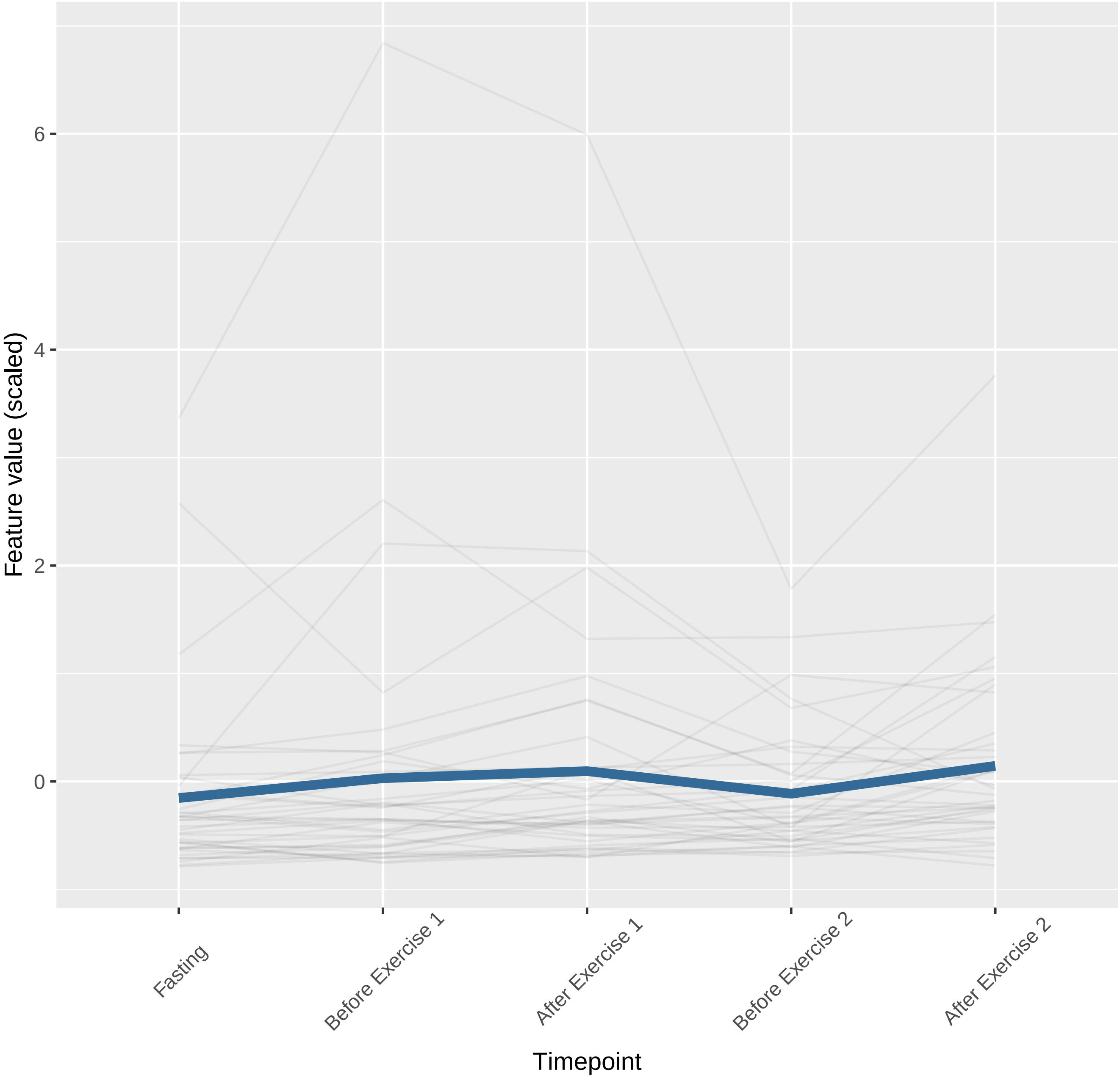

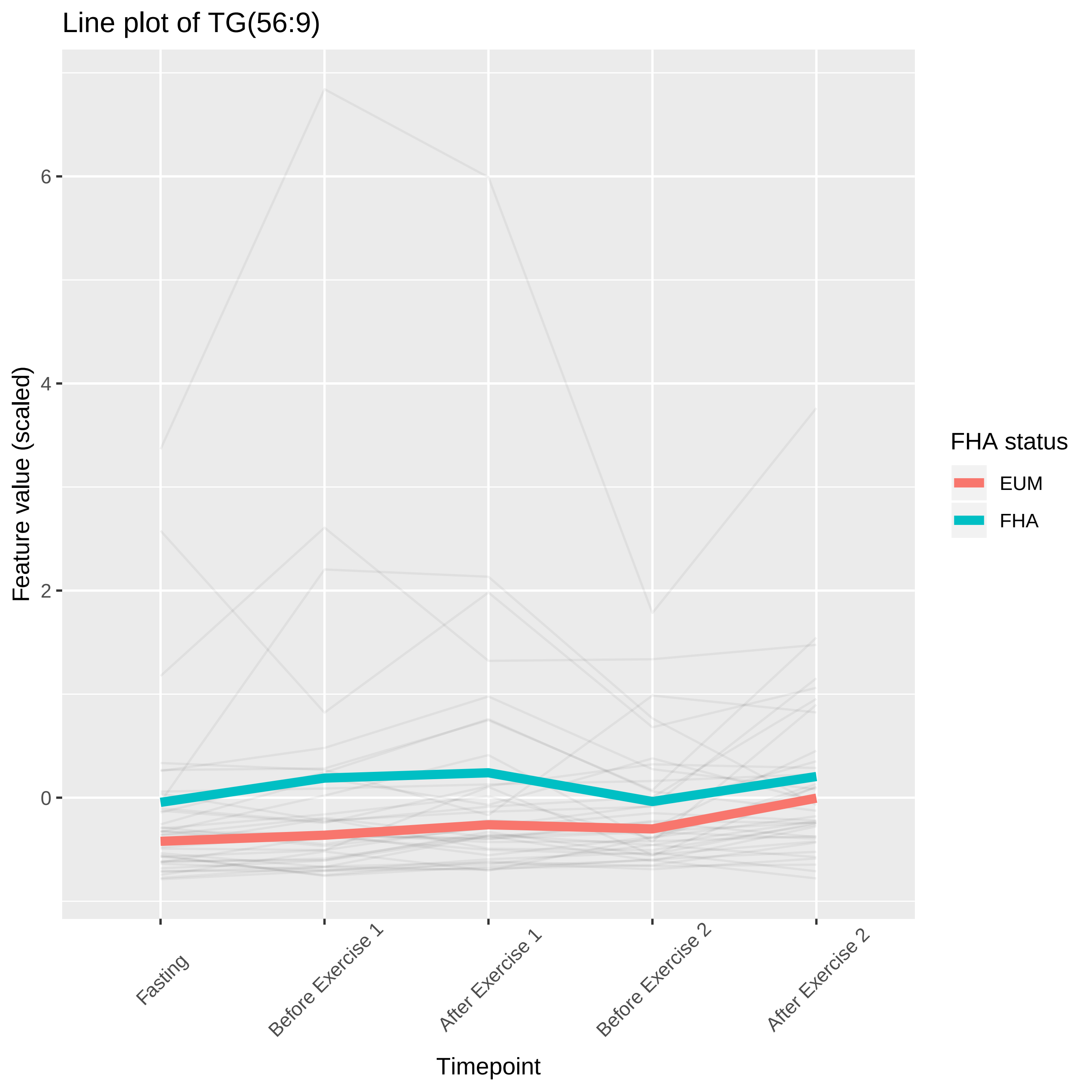

Line plot of TG(56:9)
Feature value (scaled)
6
4
2
0
Fasting
Before Exercise 1
After Exercise 1
Before Exercise 2
After Exercise 2
Timepoint
FHA status
EUM
FHA

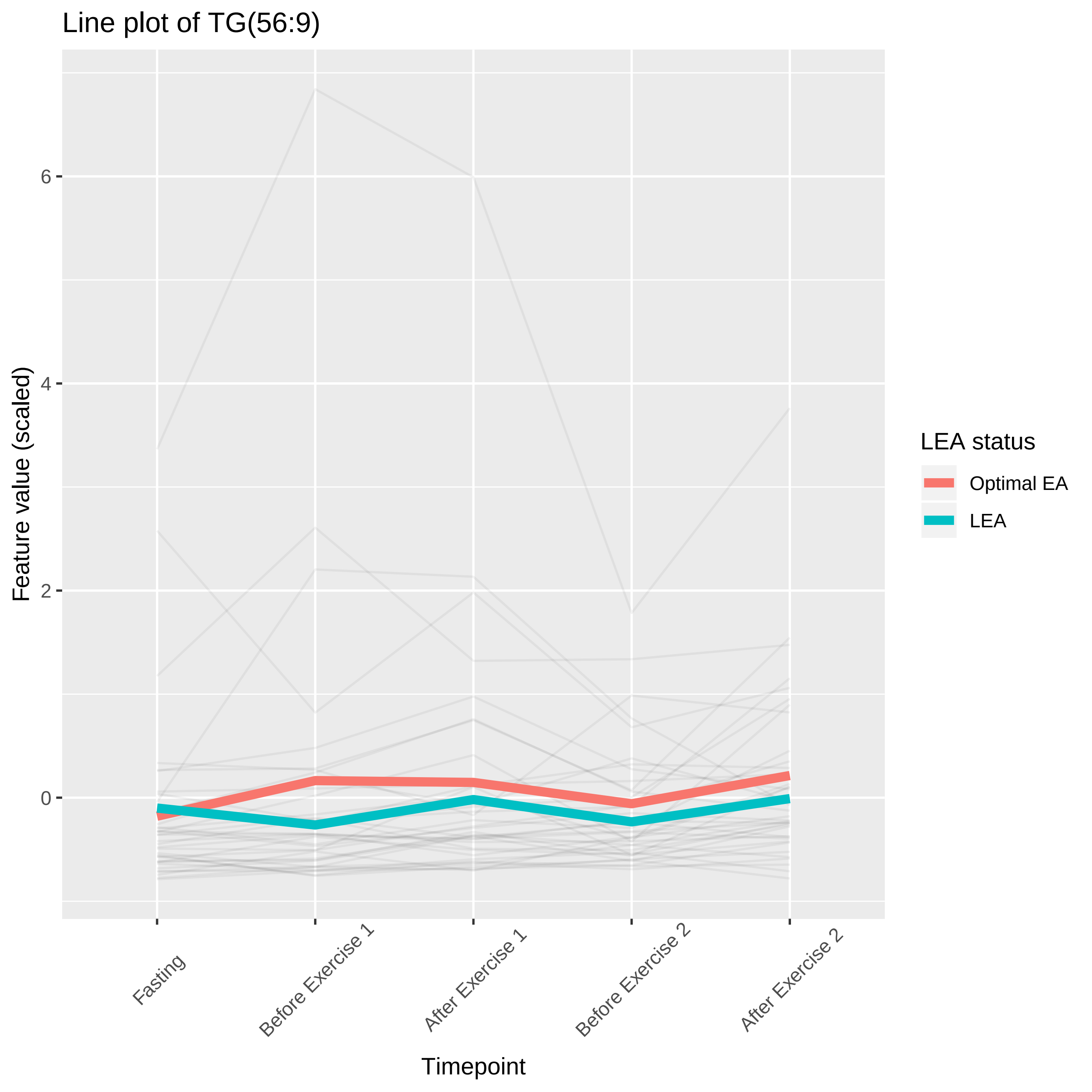

Line plot of TG(56:9)
Feature value (scaled)
0
2
4
6
Fasting
Before Exercise 1
After Exercise 1
Before Exercise 2
After Exercise 2
Timepoint
LEA status
Optimal EA
LEA

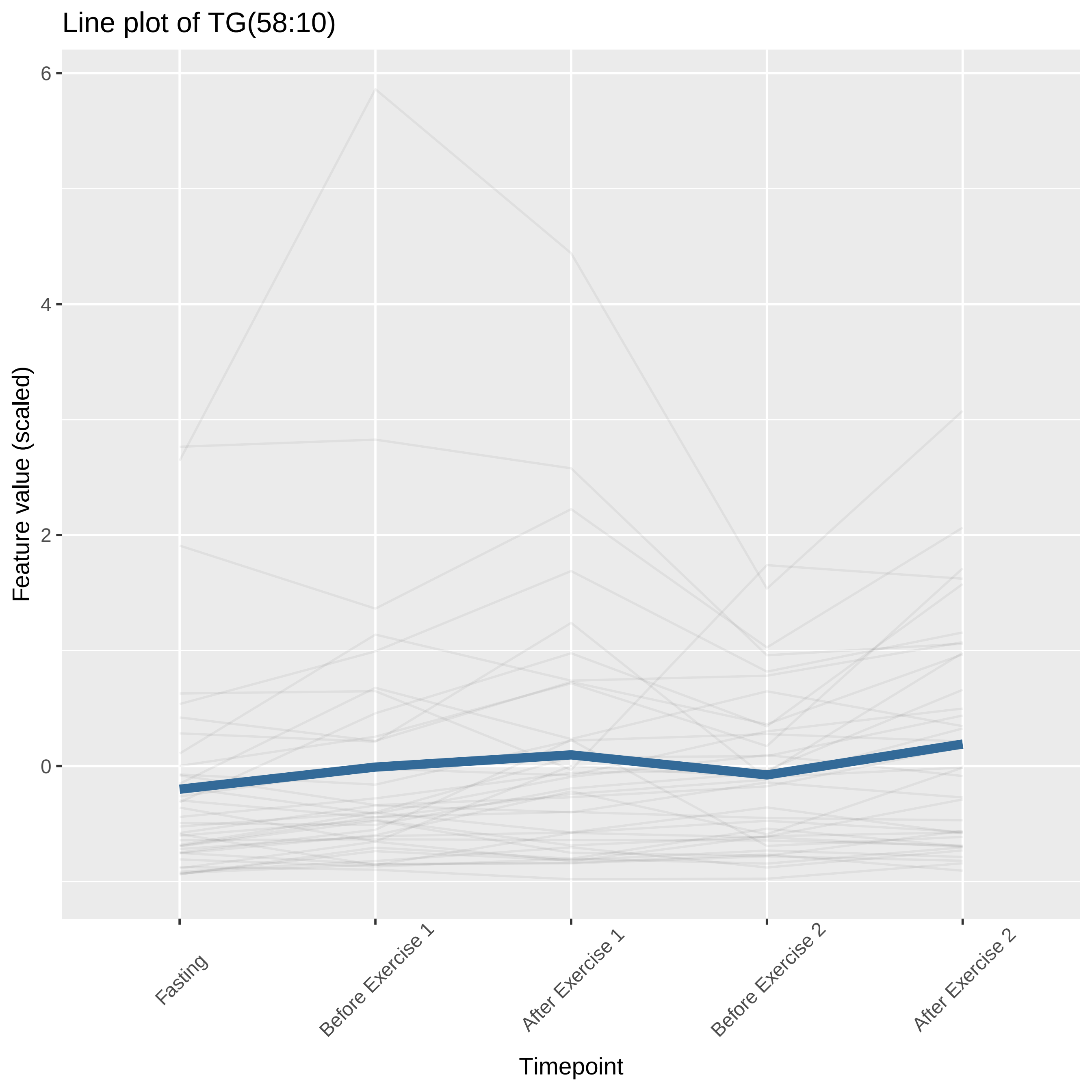

Line plot of TG(58:10)
Feature value (scaled)
6
4
2
0
Fasting
Before Exercise 1
After Exercise 1
Before Exercise 2
After Exercise 2
Timepoint

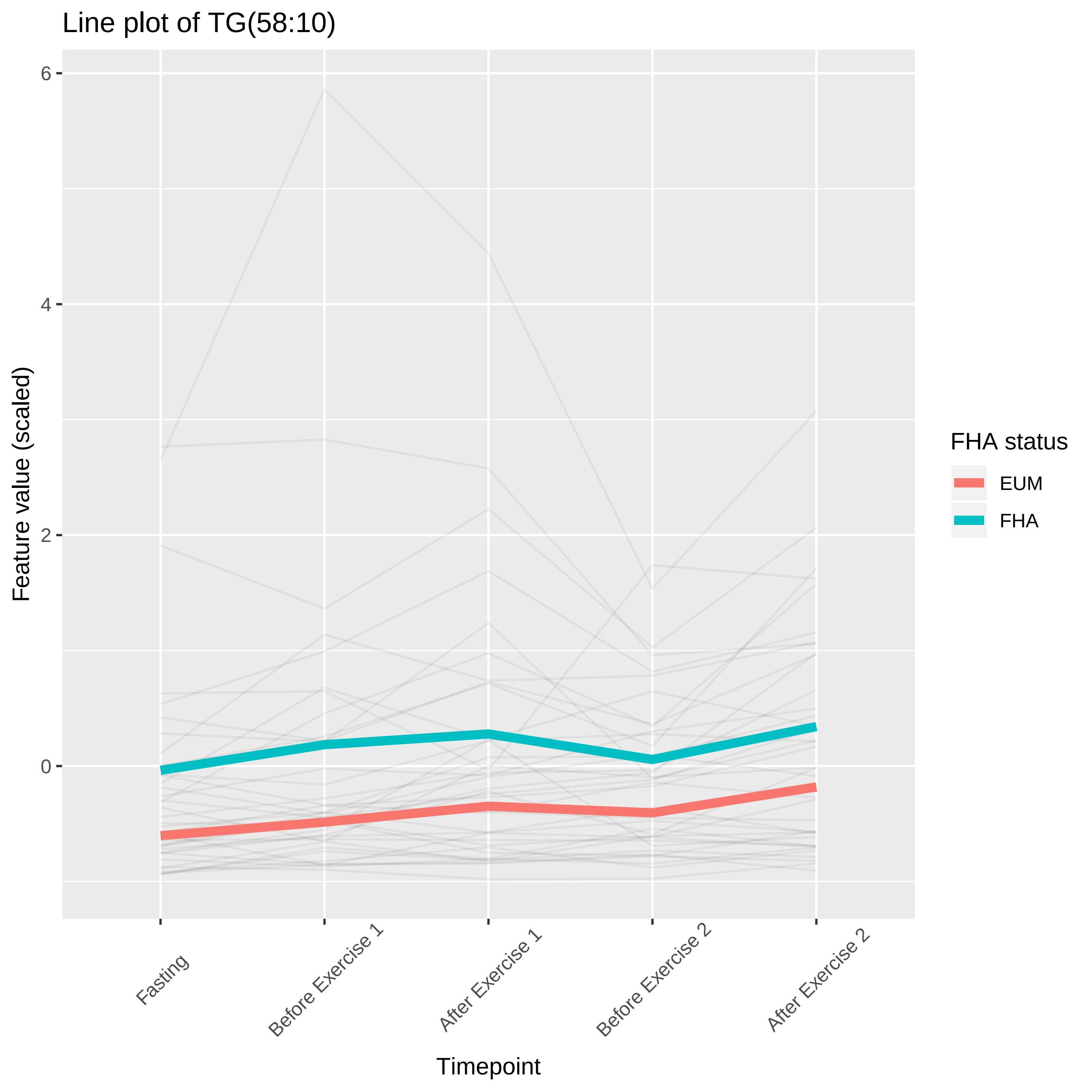

Line plot of TG(58:10)
Feature value (scaled)
6
4
2
0
Fasting
Before Exercise 1
After Exercise 1
Before Exercise 2
After Exercise 2
Timepoint
FHA status
EUM
FHA

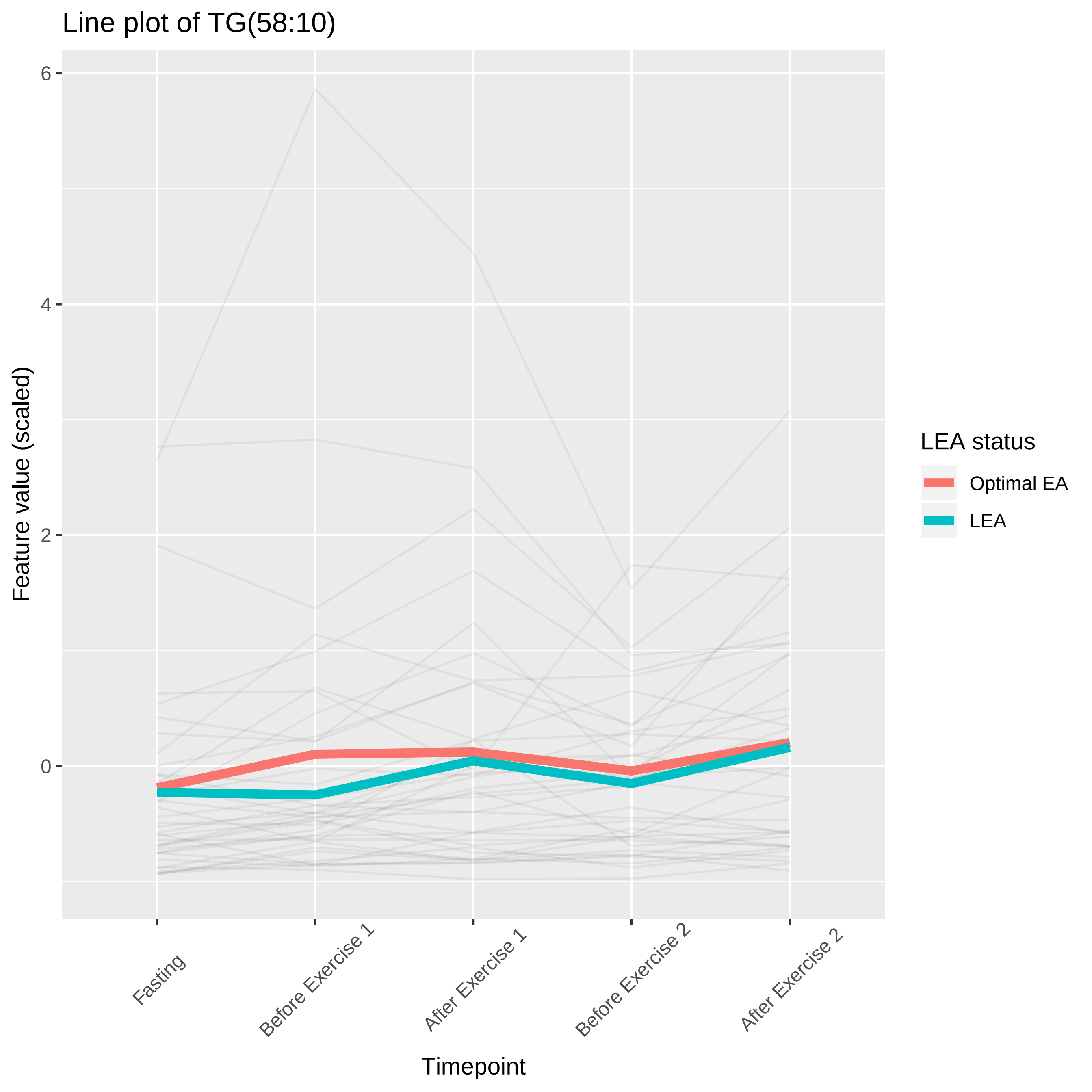

Line plot of TG(58:10)
Feature value (scaled)
6
4
2
0
Fasting
Before Exercise 1
After Exercise 1
Before Exercise 2
After Exercise 2
Timepoint
LEA status
Optimal EA
LEA

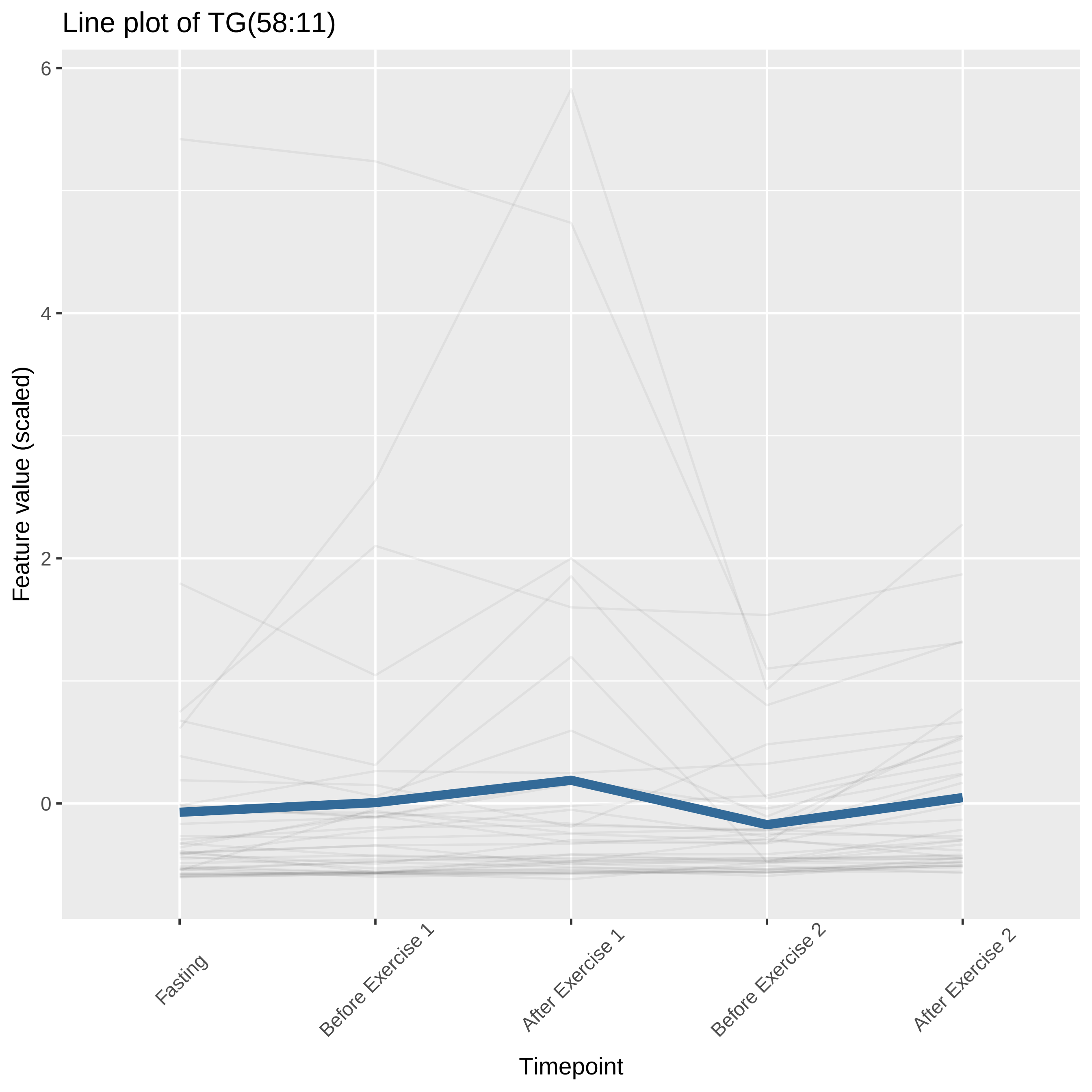

Line plot of TG(58:11)
Feature value (scaled)
6
4
2
0
Fasting
Before Exercise 1
After Exercise 1
Before Exercise 2
After Exercise 2
Timepoint

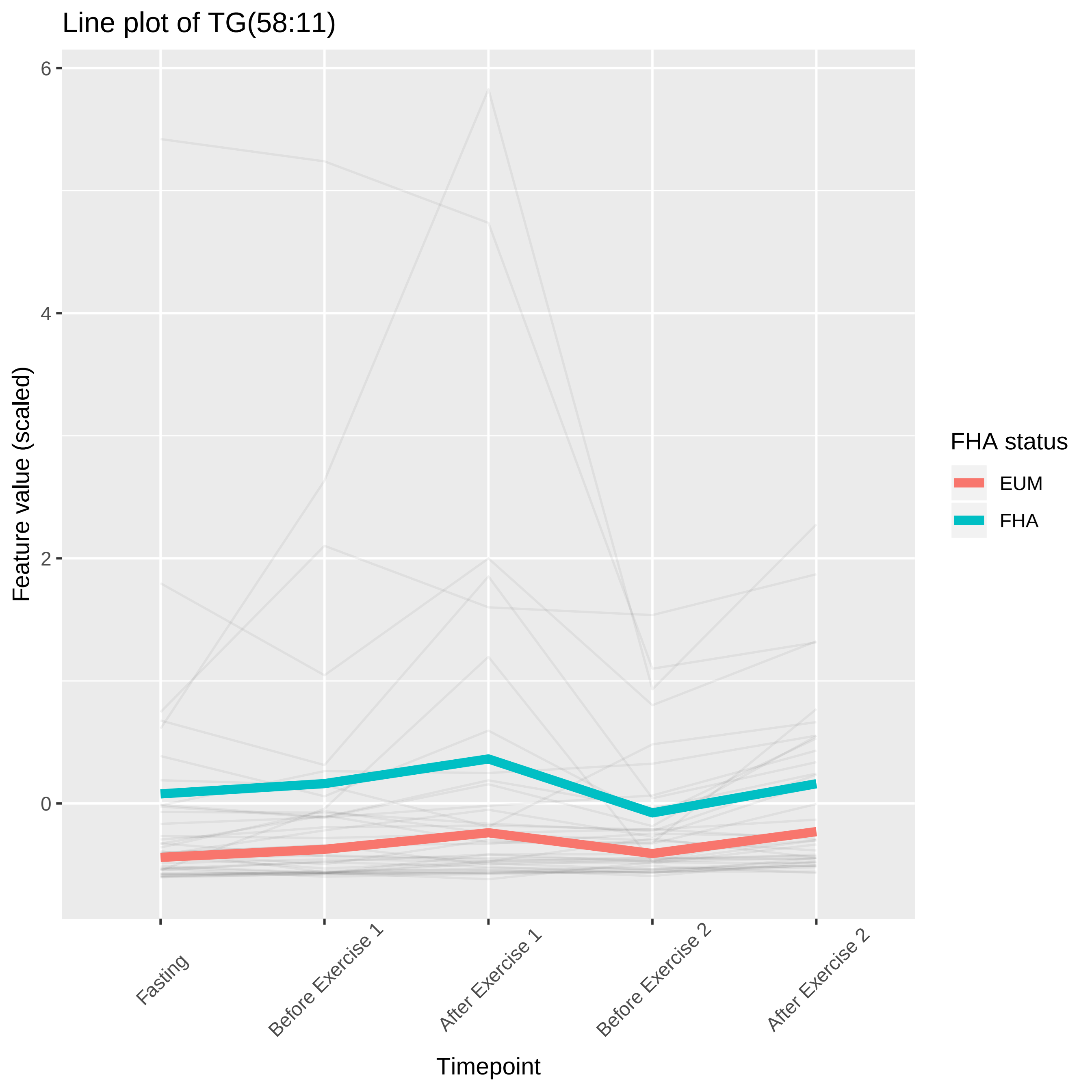

Line plot of TG(58:11)
Feature value (scaled)
6
4
2
0
Fasting
Before Exercise 1
After Exercise 1
Before Exercise 2
After Exercise 2
Timepoint
FHA status
EUM
FHA

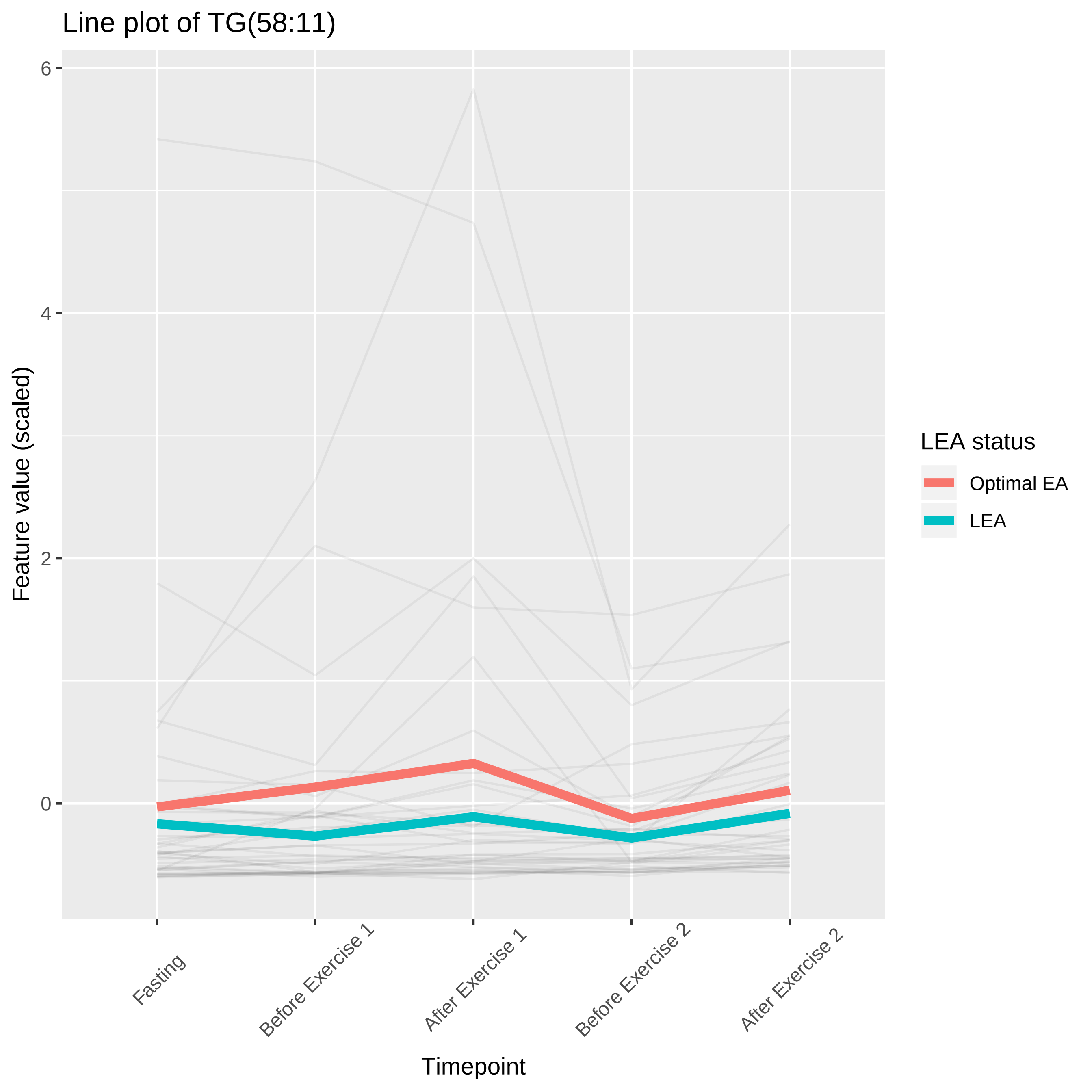

Line plot of TG(58:11)
Feature value (scaled)
6
4
2
0
Fasting
Before Exercise 1
After Exercise 1
Before Exercise 2
After Exercise 2
Timepoint
LEA status
Optimal EA
LEA

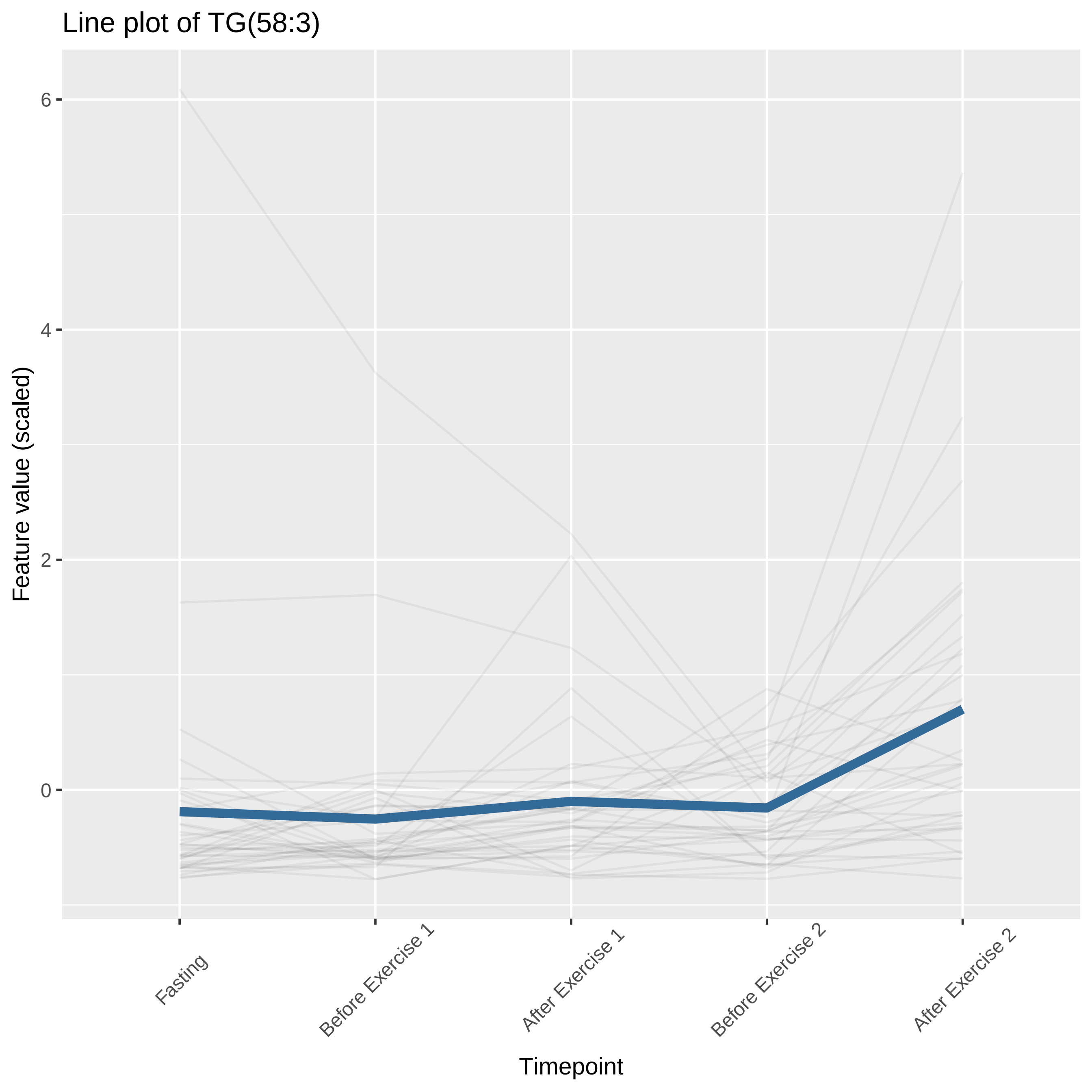

Line plot of TG(58:3)
Feature value (scaled)
6
4
2
0
Fasting
Before Exercise 1
After Exercise 1
Before Exercise 2
After Exercise 2
Timepoint

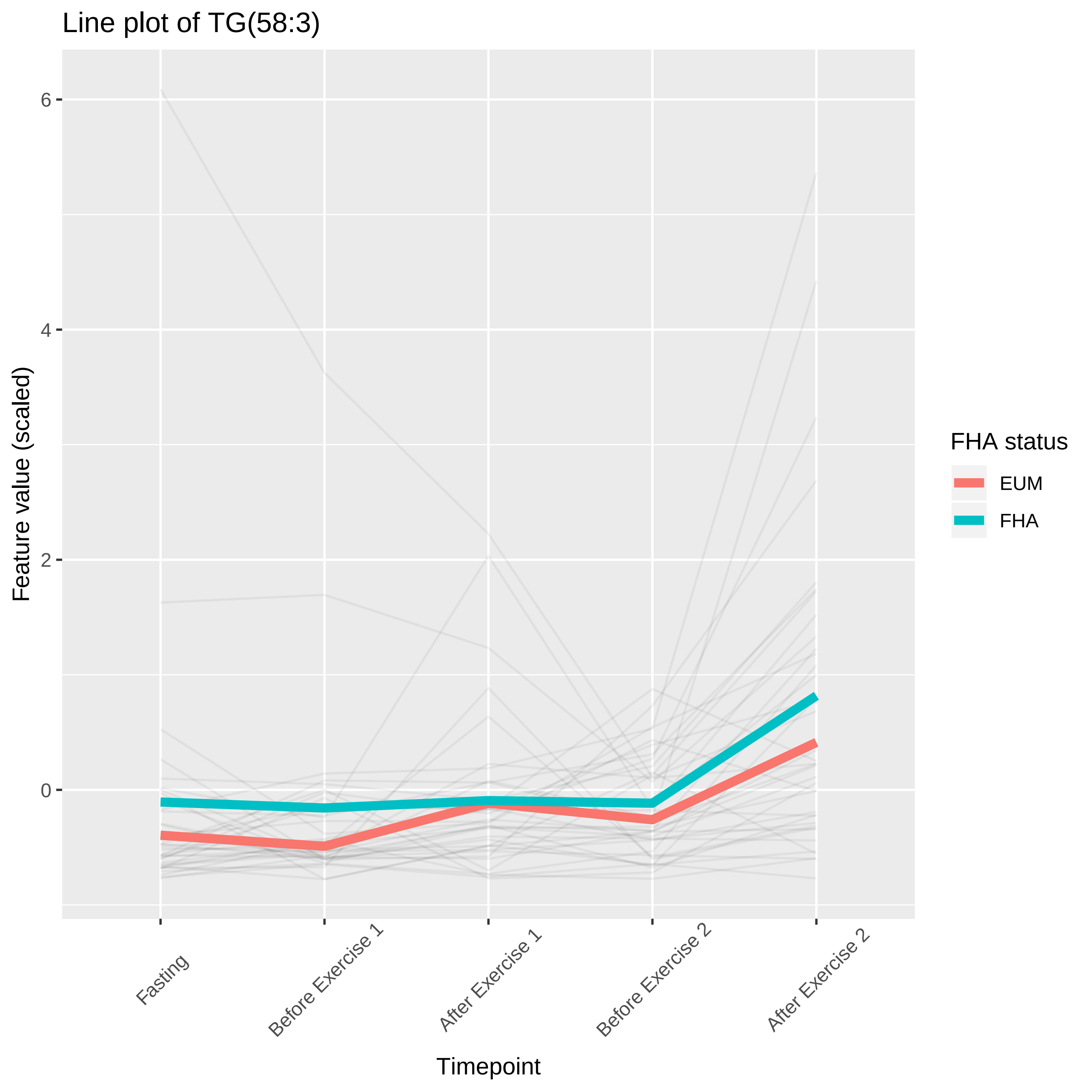

Line plot of TG(58:3)
Feature value (scaled)
6
4
2
0
Fasting
Before Exercise 1
After Exercise 1
Before Exercise 2
After Exercise 2
Timepoint
FHA status
EUM
FHA

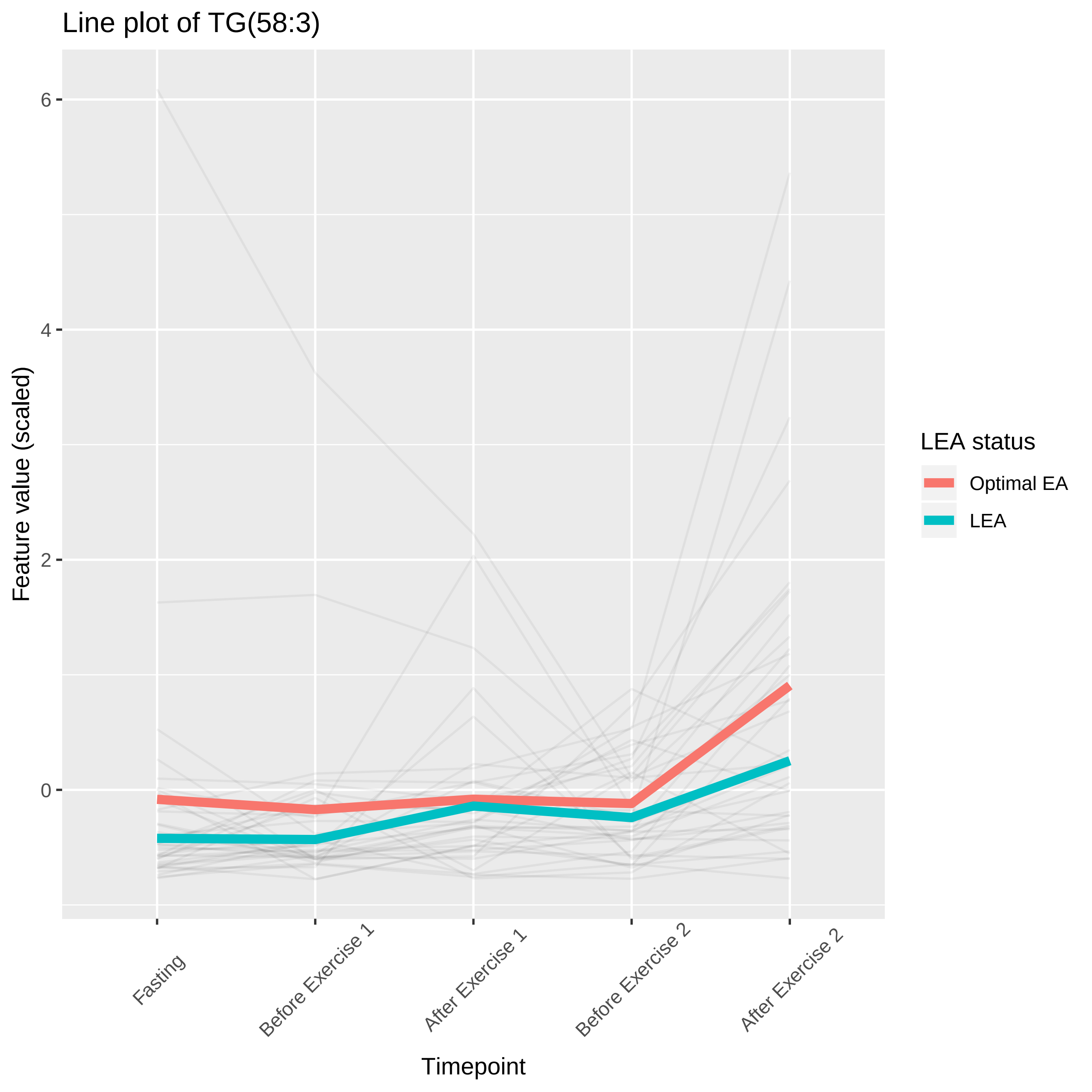

Line plot of TG(58:3)
Feature value (scaled)
6
4
2
0
Fasting
Before Exercise 1
After Exercise 1
Before Exercise 2
After Exercise 2
Timepoint
LEA status
Optimal EA
LEA

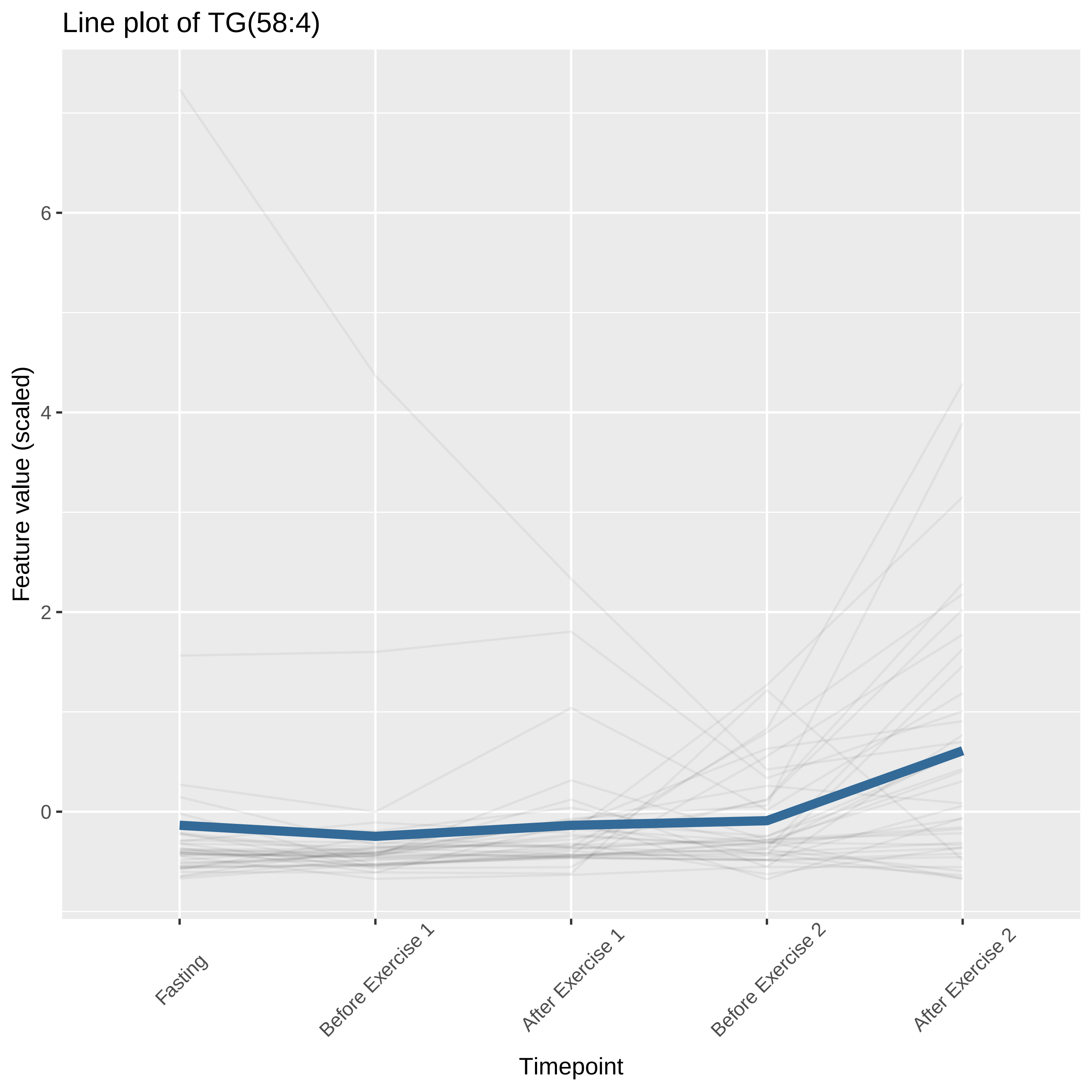
Line plot of TG(58:4)
Feature value (scaled)
6
4
2
0
Fasting
Before Exercise 1
After Exercise 1
Before Exercise 2
After Exercise 2
Timepoint

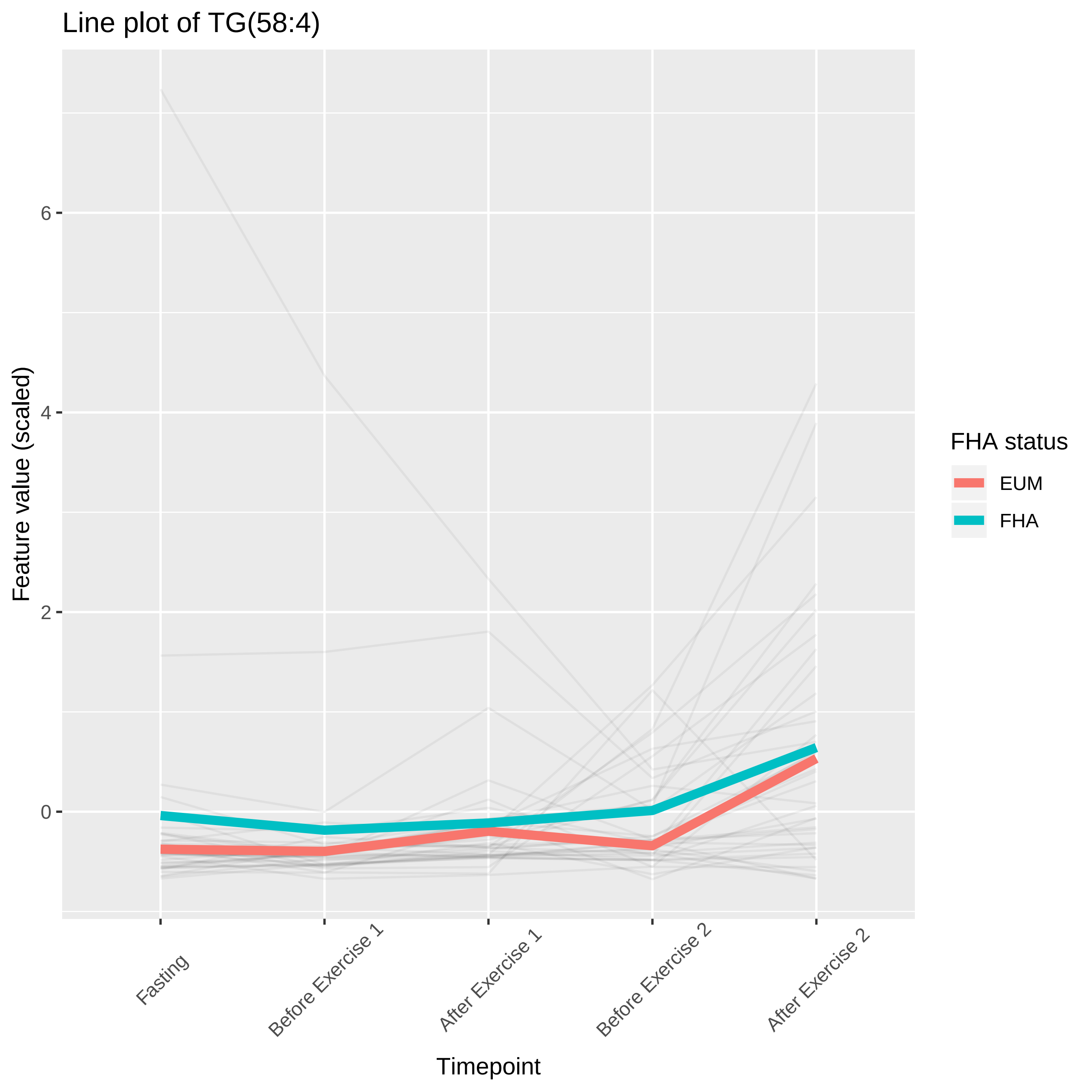

Line plot of TG(58:4)
Feature value (scaled)
6
4
2
0
Fasting
Before Exercise 1
After Exercise 1
Before Exercise 2
After Exercise 2
Timepoint
FHA status
EUM
FHA

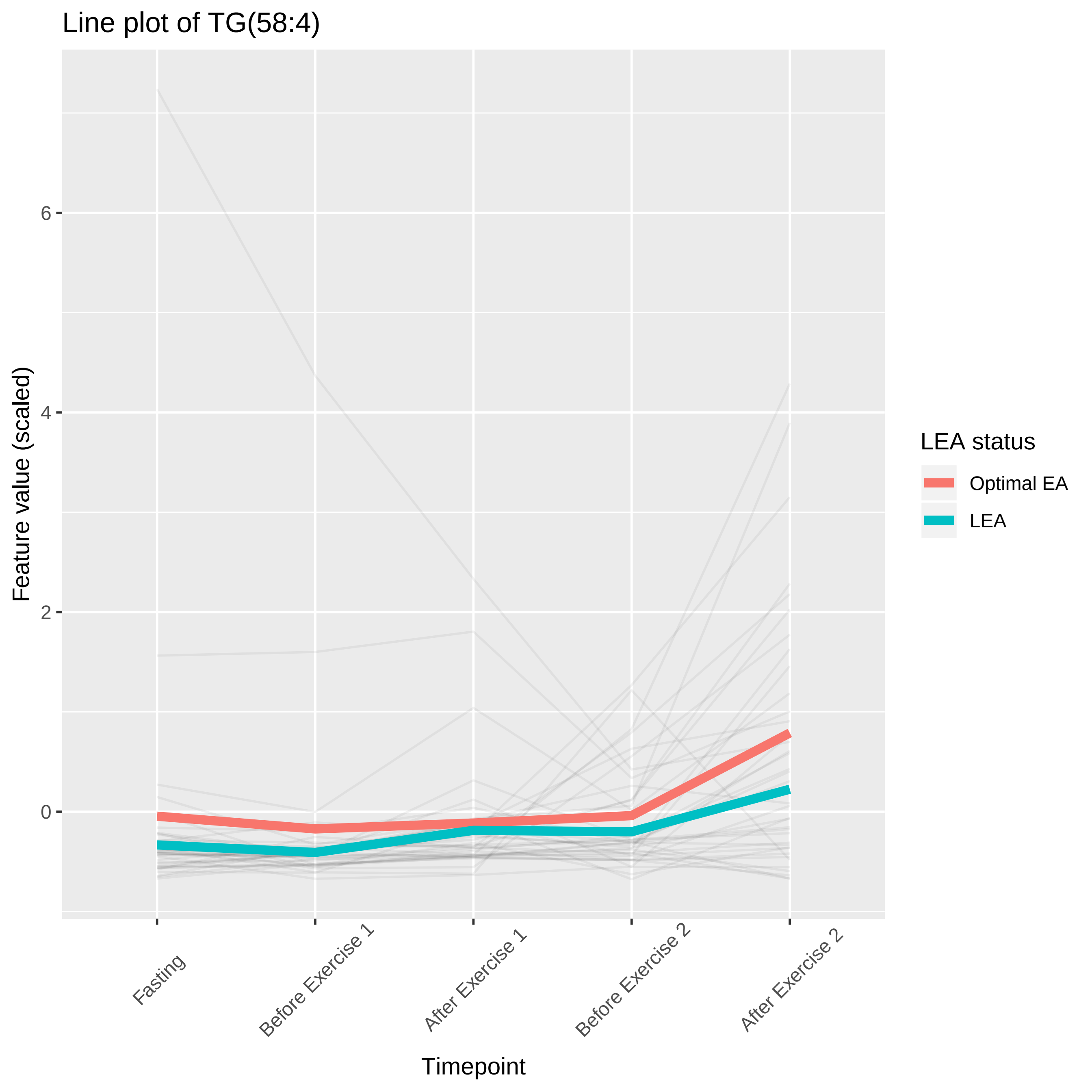

Line plot of TG(58:4)
Feature value (scaled)
6
4
2
0
Fasting
Before Exercise 1
After Exercise 1
Before Exercise 2
After Exercise 2
Timepoint
LEA status
Optimal EA
LEA

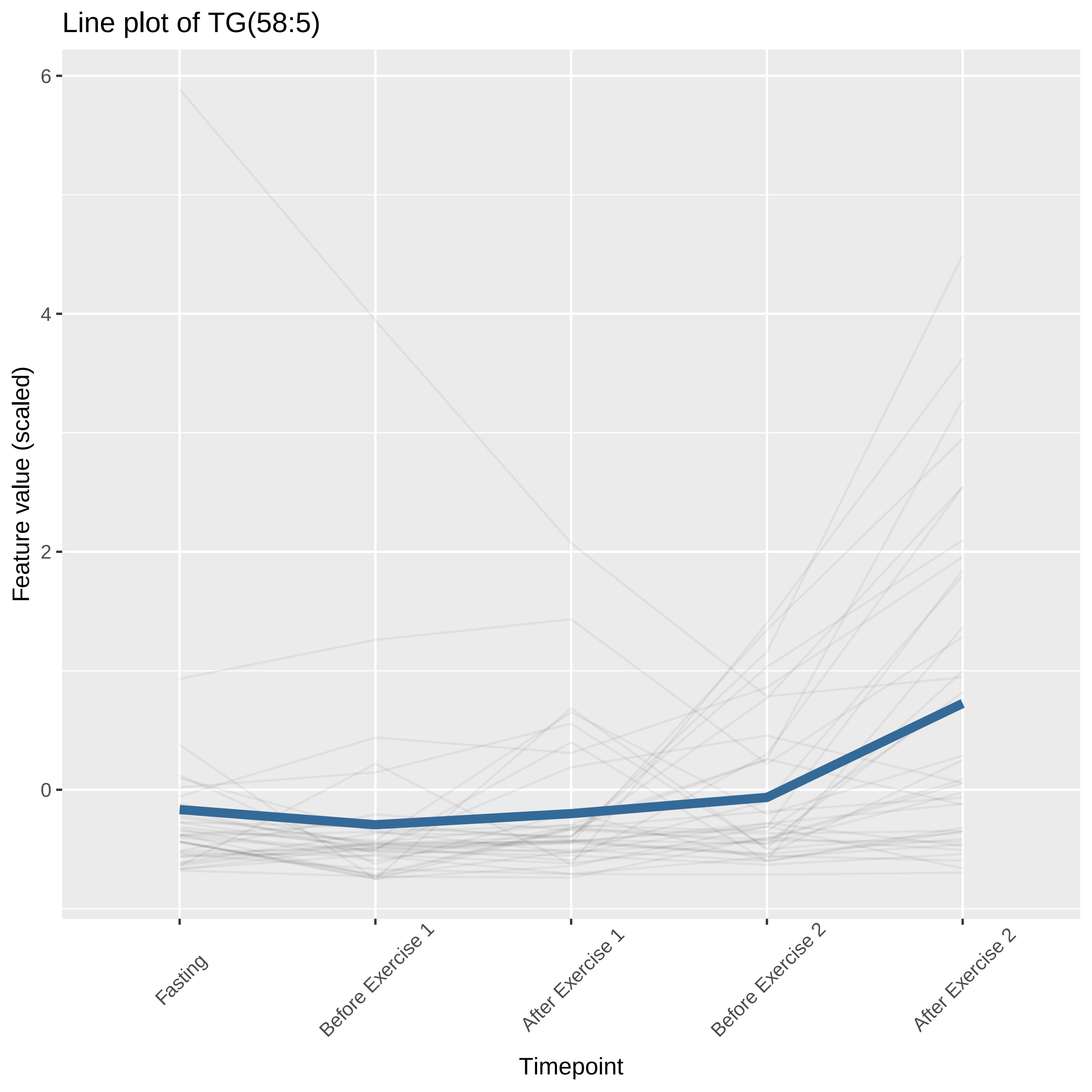
Line plot of TG(58:5)
Feature value (scaled)
6
4
2
0
Fasting
Before Exercise 1
After Exercise 1
Before Exercise 2
After Exercise 2
Timepoint

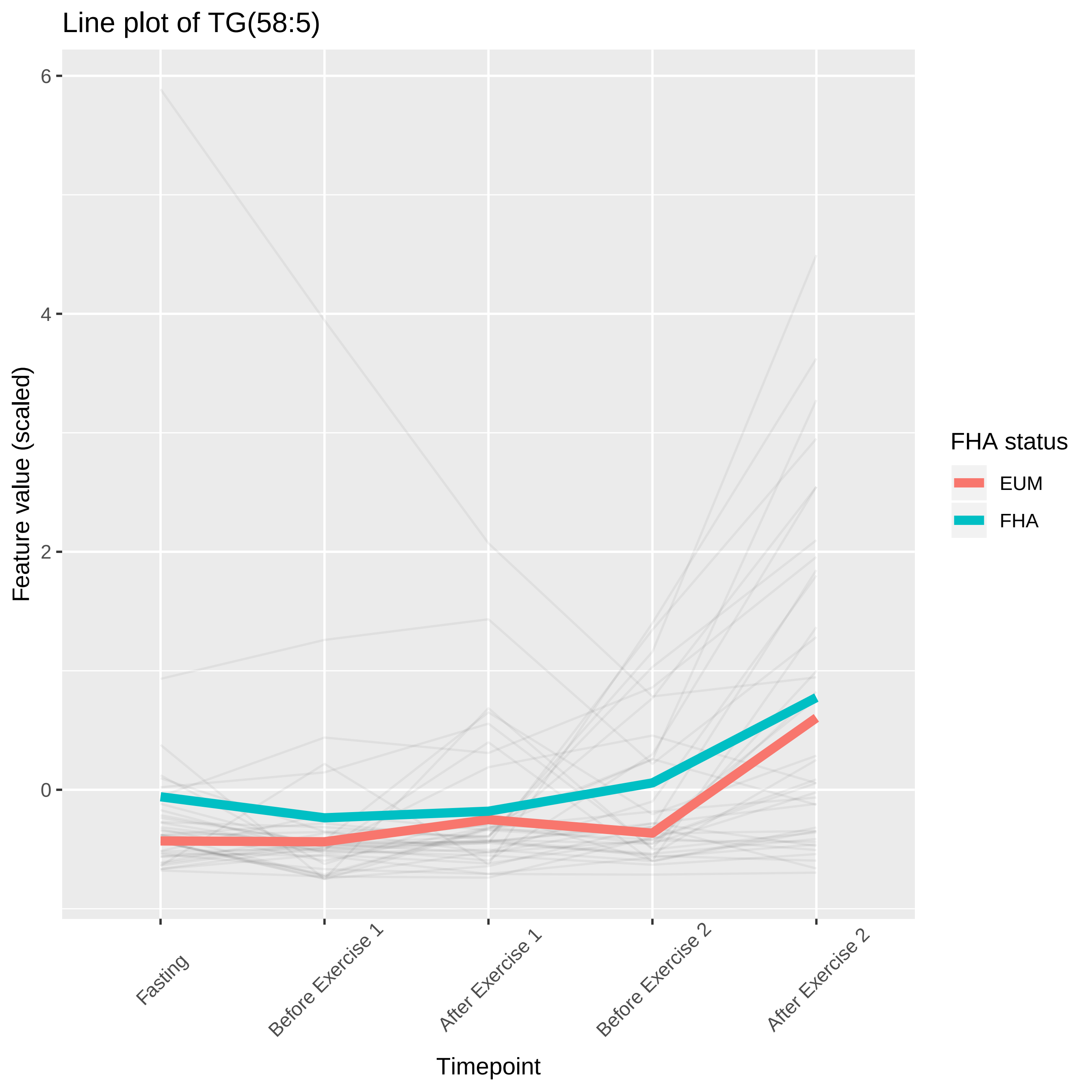

Line plot of TG(58:5)
Feature value (scaled)
6
4
2
0
Fasting
Before Exercise 1
After Exercise 1
Before Exercise 2
After Exercise 2
Timepoint
FHA status
EUM
FHA

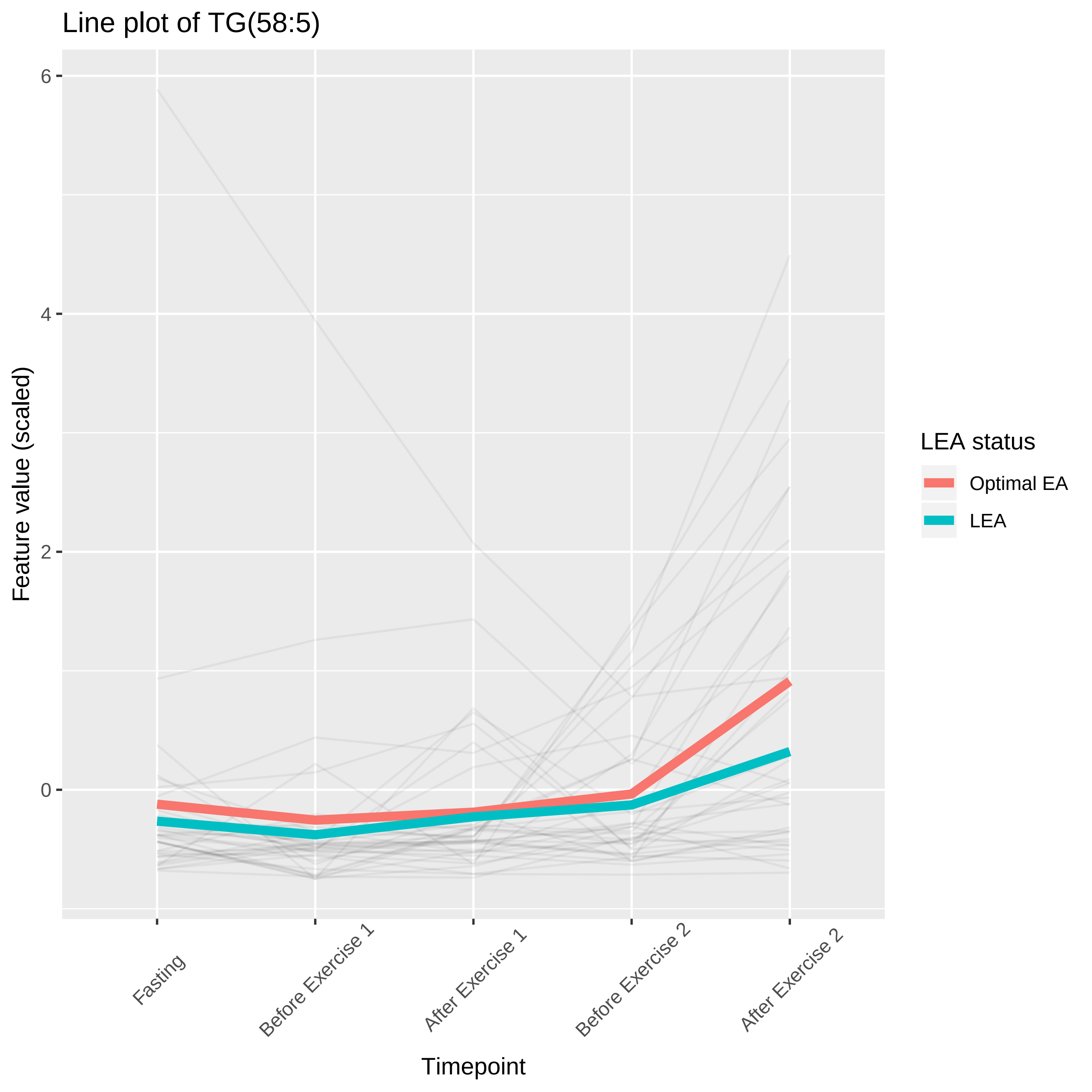

Line plot of TG(58:5)
Feature value (scaled)
6
4
2
0
Fasting
Before Exercise 1
After Exercise 1
Before Exercise 2
After Exercise 2
Timepoint
LEA status
Optimal EA
LEA

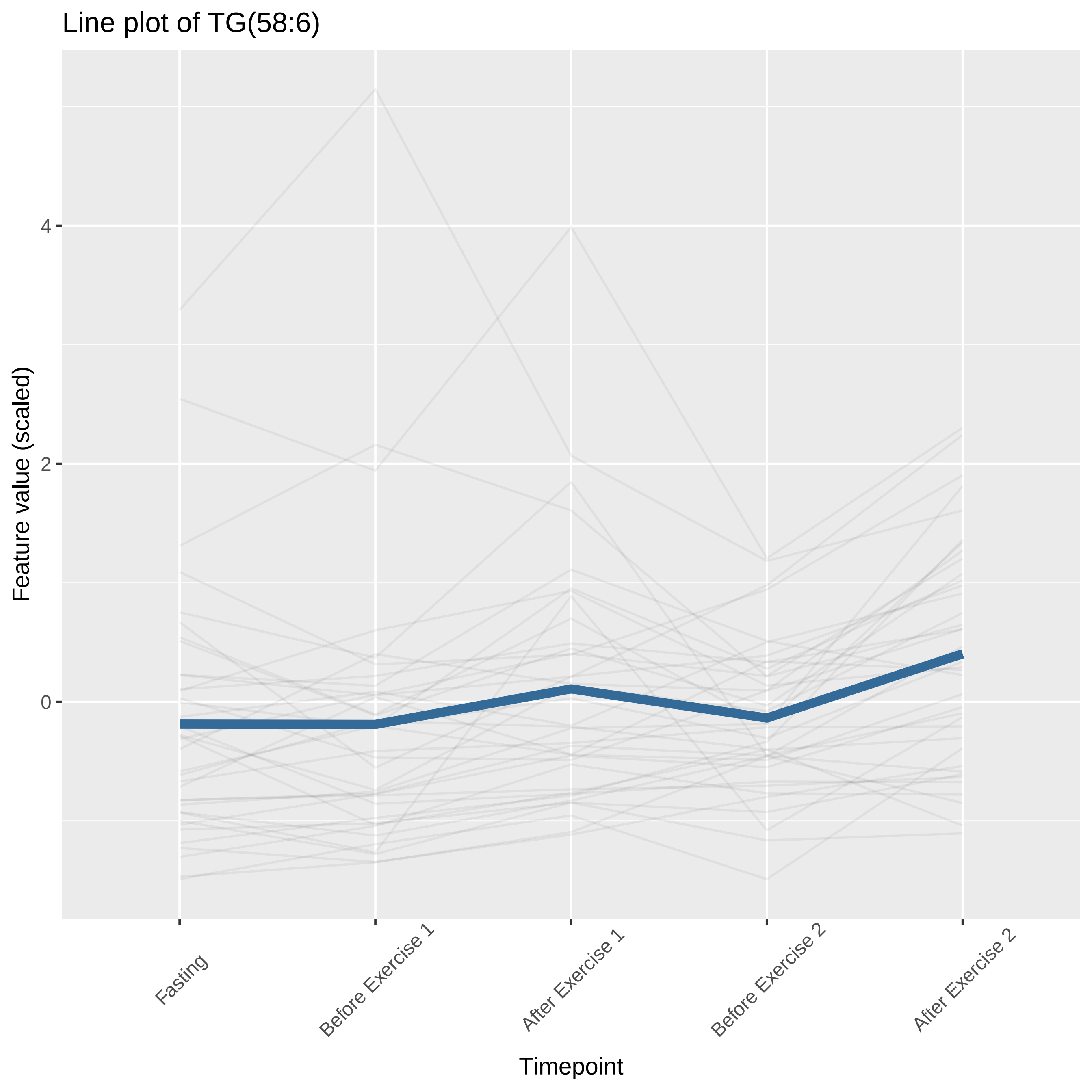

Line plot of TG(58:6)
Feature value (scaled)
4
2
0
Fasting
Before Exercise 1
After Exercise 1
Before Exercise 2
After Exercise 2
Timepoint

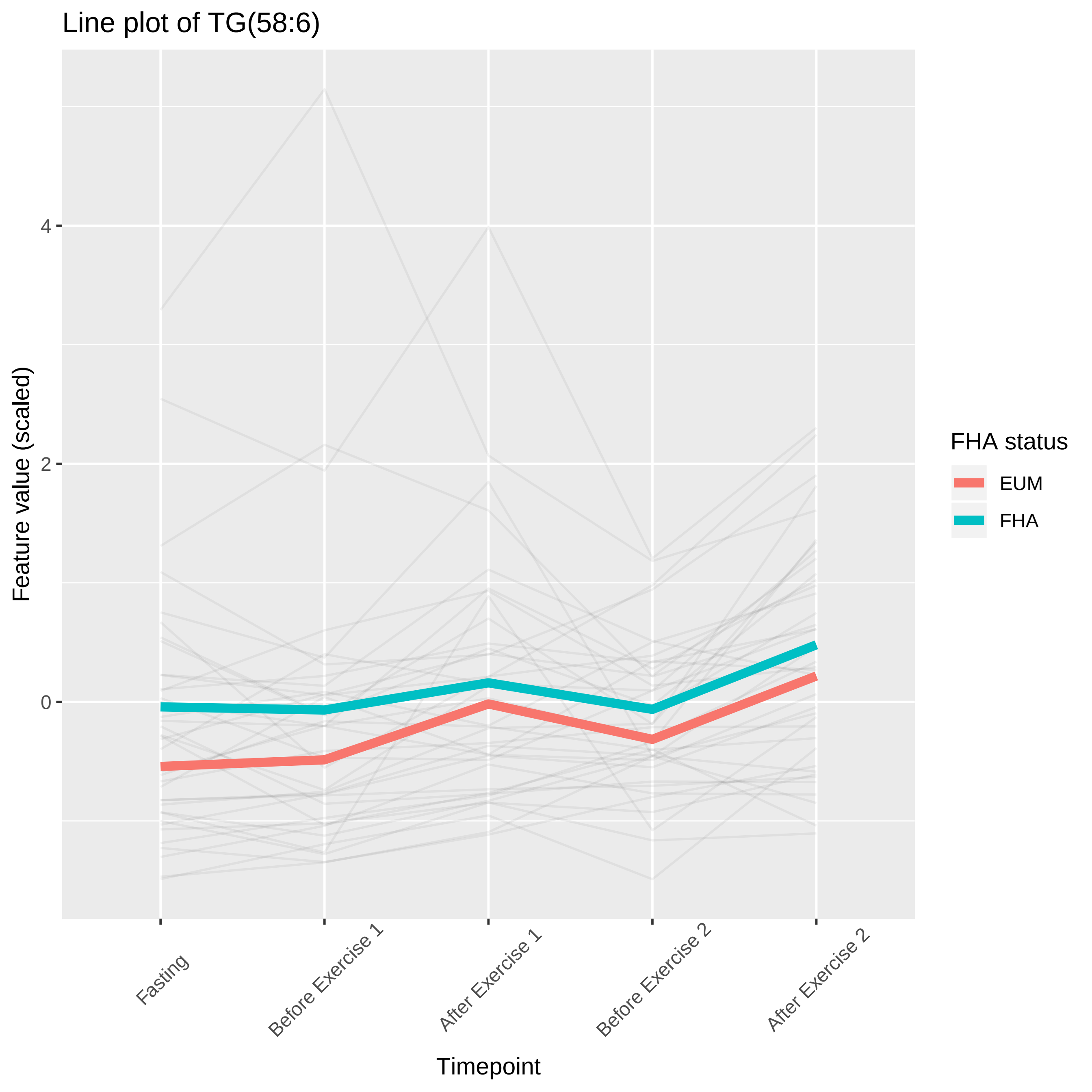

Line plot of TG(58:6)
Feature value (scaled)
4
2
0
Fasting
Before Exercise 1
After Exercise 1
Before Exercise 2
After Exercise 2
Timepoint
FHA status
EUM
FHA

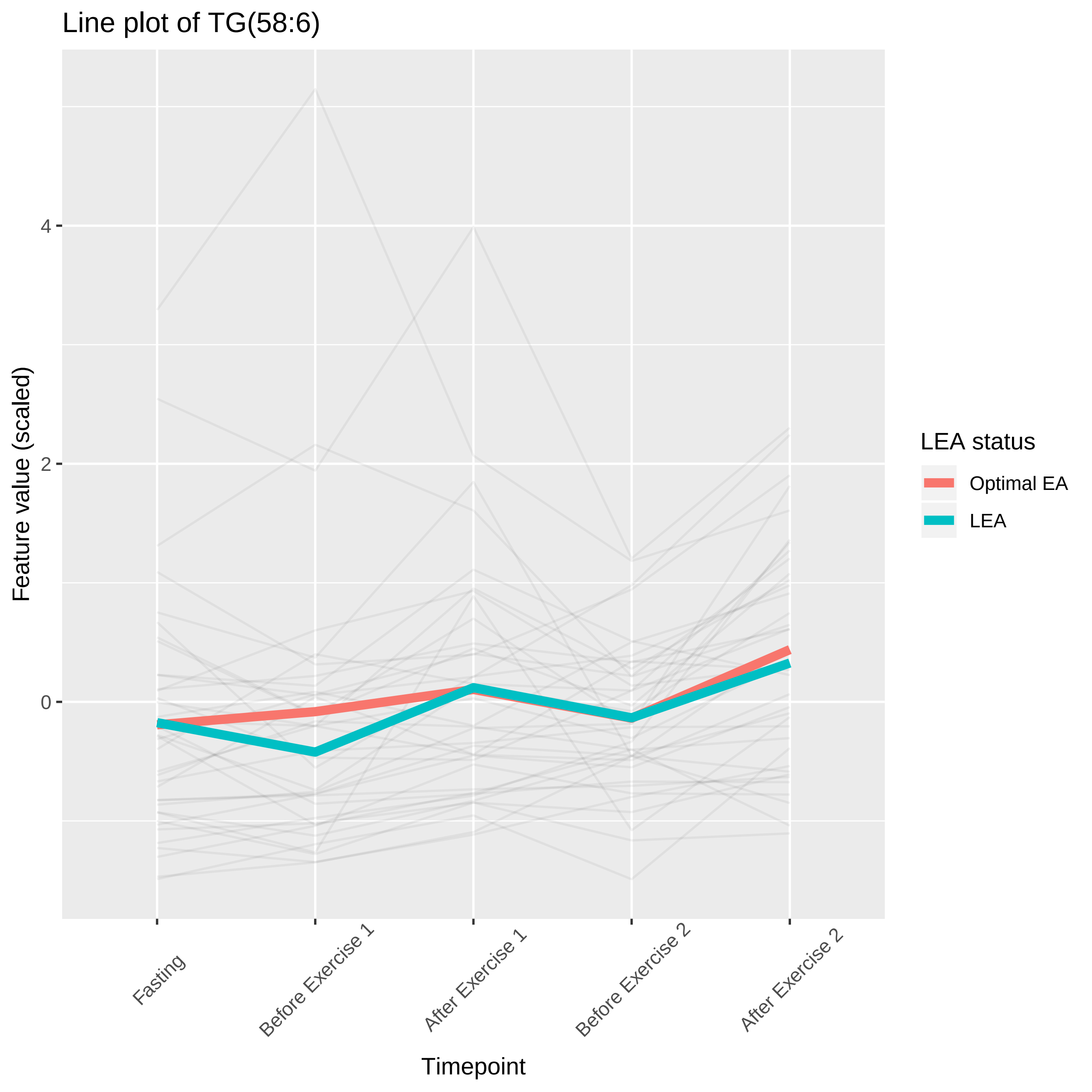
Line plot of TG(58:6)
Feature value (scaled)
4
2
0
Fasting
Before Exercise 1
After Exercise 1
Before Exercise 2
After Exercise 2
Timepoint
LEA status
Optimal EA
LEA

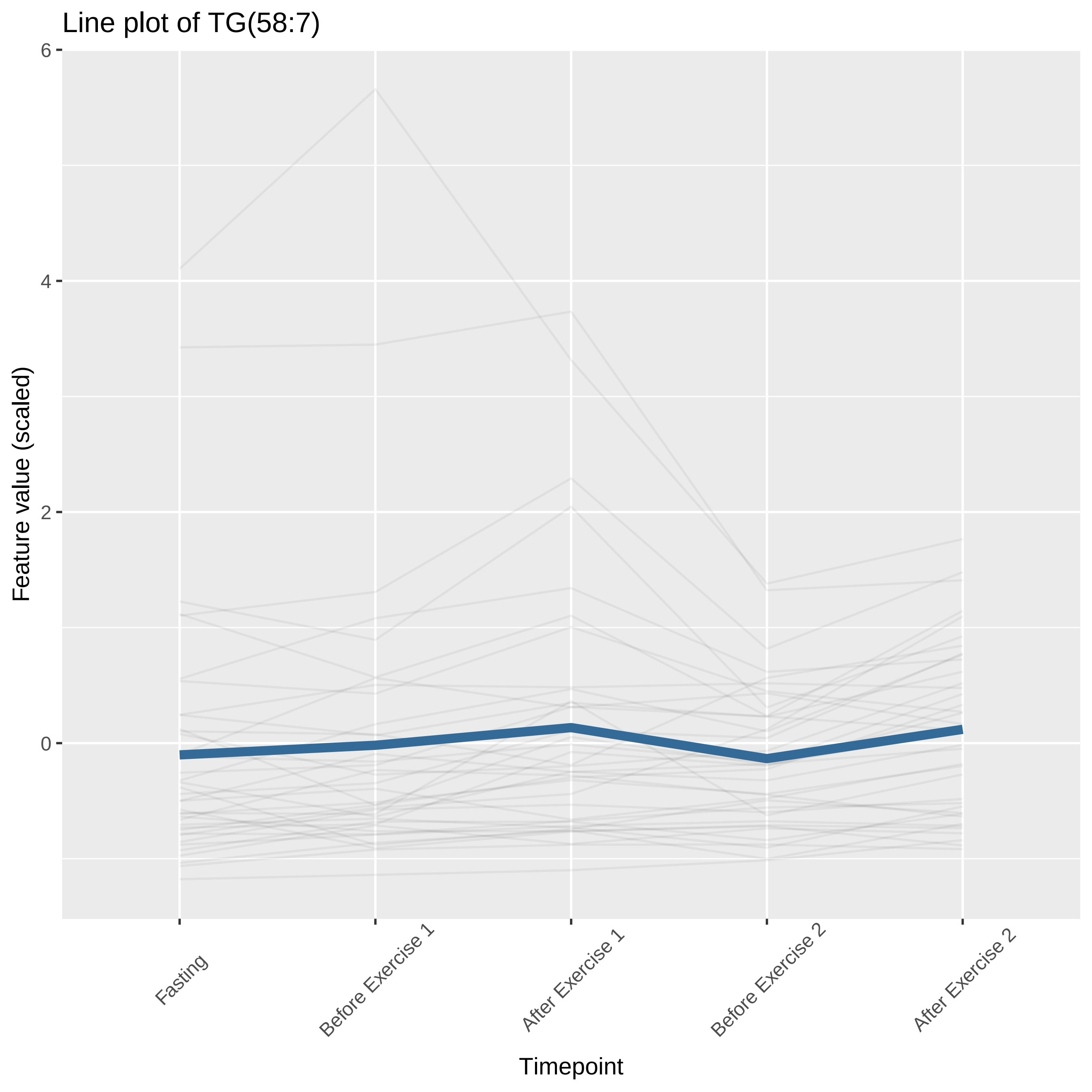

Line plot of TG(58:7)
6
4
2
0
Feature value (scaled)
Fasting
Before Exercise 1
After Exercise 1
Before Exercise 2
After Exercise 2
Timepoint

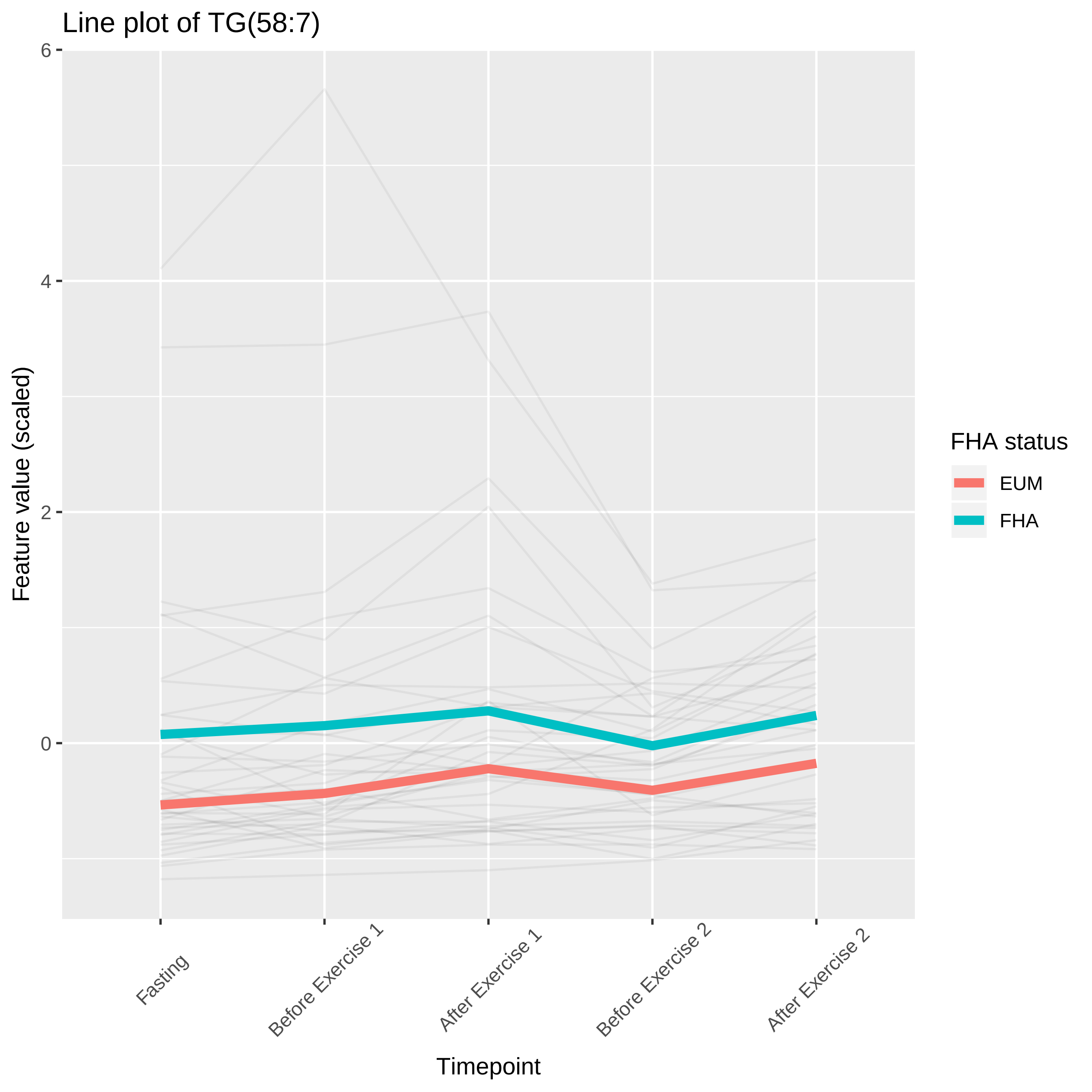

Line plot of TG(58:7)
Feature value (scaled)
6
4
2
0
Fasting
Before Exercise 1
After Exercise 1
Before Exercise 2
After Exercise 2
Timepoint
FHA status
EUM
FHA

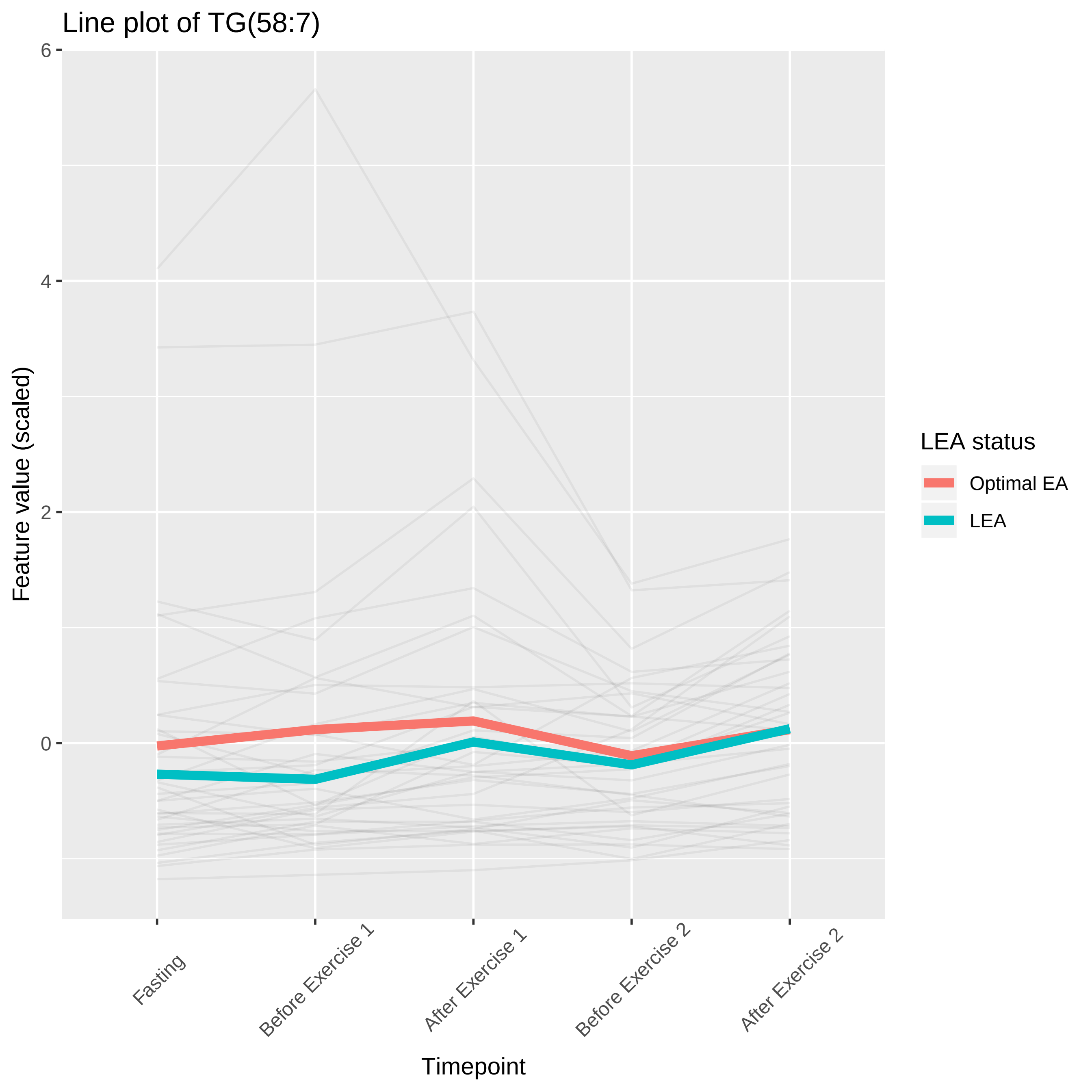
Line plot of TG(58:7)
Feature value (scaled)
6
4
2
0
Fasting
Before Exercise 1
After Exercise 1
Before Exercise 2
After Exercise 2
Timepoint
LEA status
Optimal EA
LEA

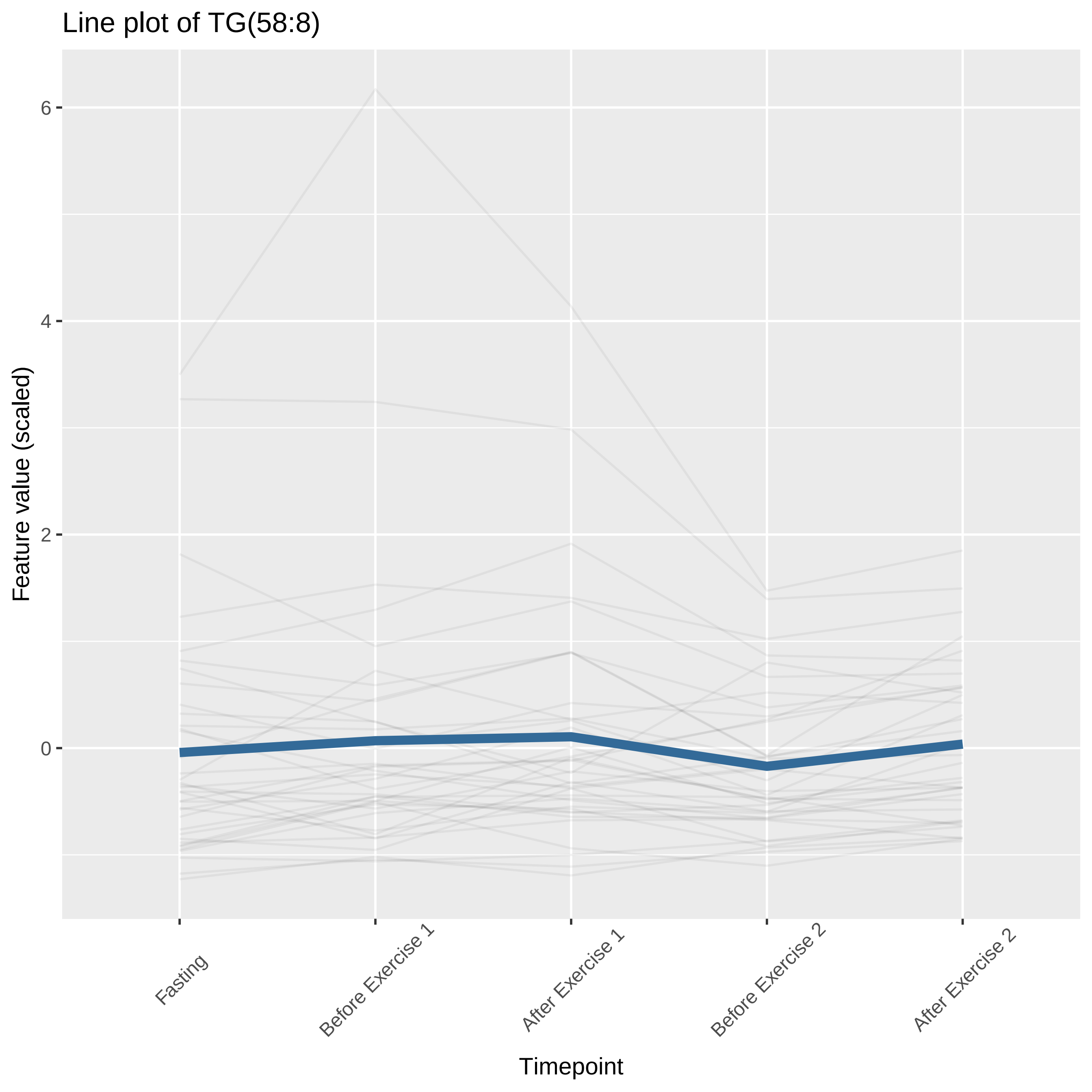

Line plot of TG(58:8)
Feature value (scaled)
6
4
2
0
Fasting
Before Exercise 1
After Exercise 1
Before Exercise 2
After Exercise 2
Timepoint

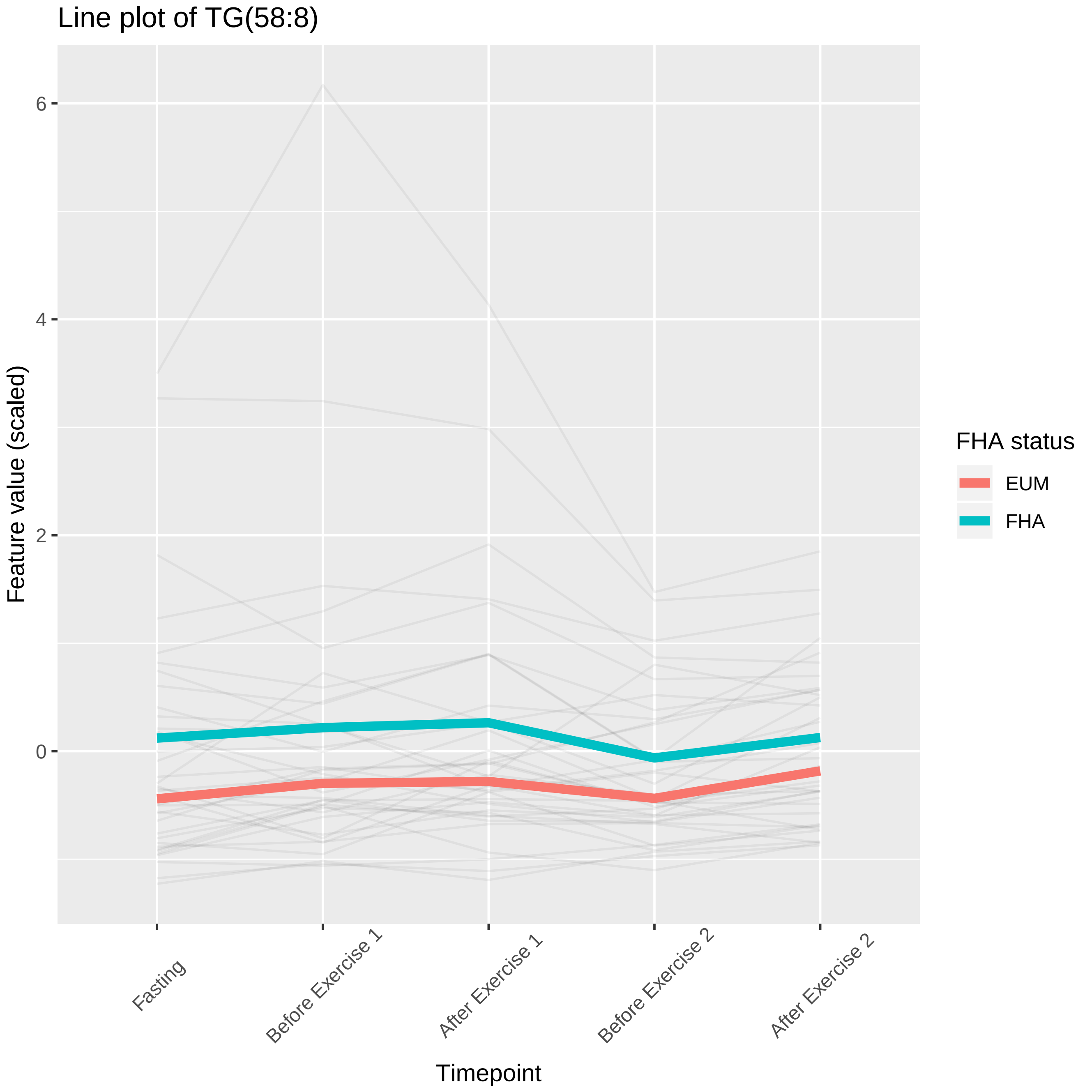

Line plot of TG(58:8)
Feature value (scaled)
6
4
2
0
Fasting
Before Exercise 1
After Exercise 1
Before Exercise 2
After Exercise 2
Timepoint
FHA status
EUM
FHA

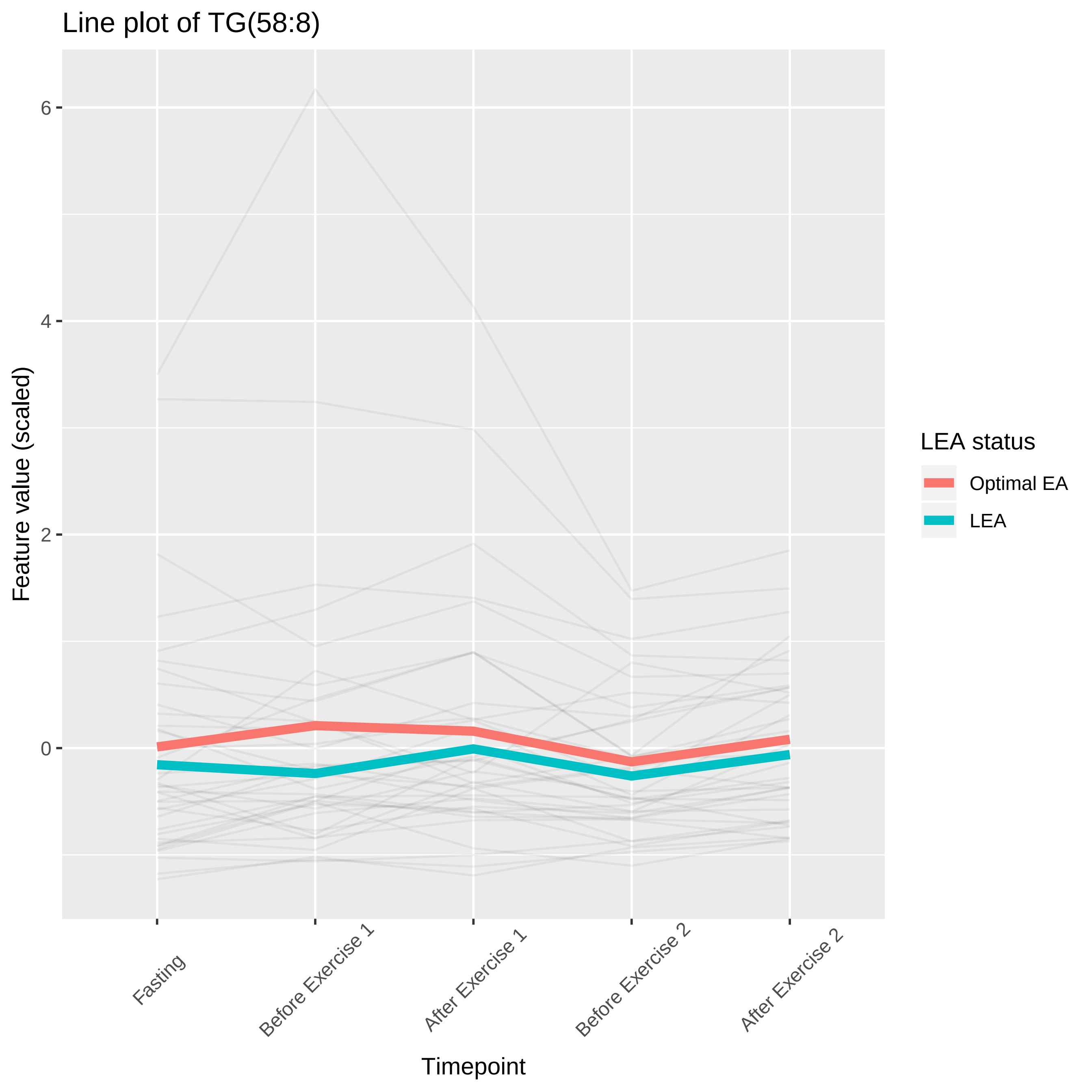

Line plot of TG(58:8)
Feature value (scaled)
6
4
2
0
Fasting
Before Exercise 1
After Exercise 1
Before Exercise 2
After Exercise 2
Timepoint
LEA status
Optimal EA
LEA

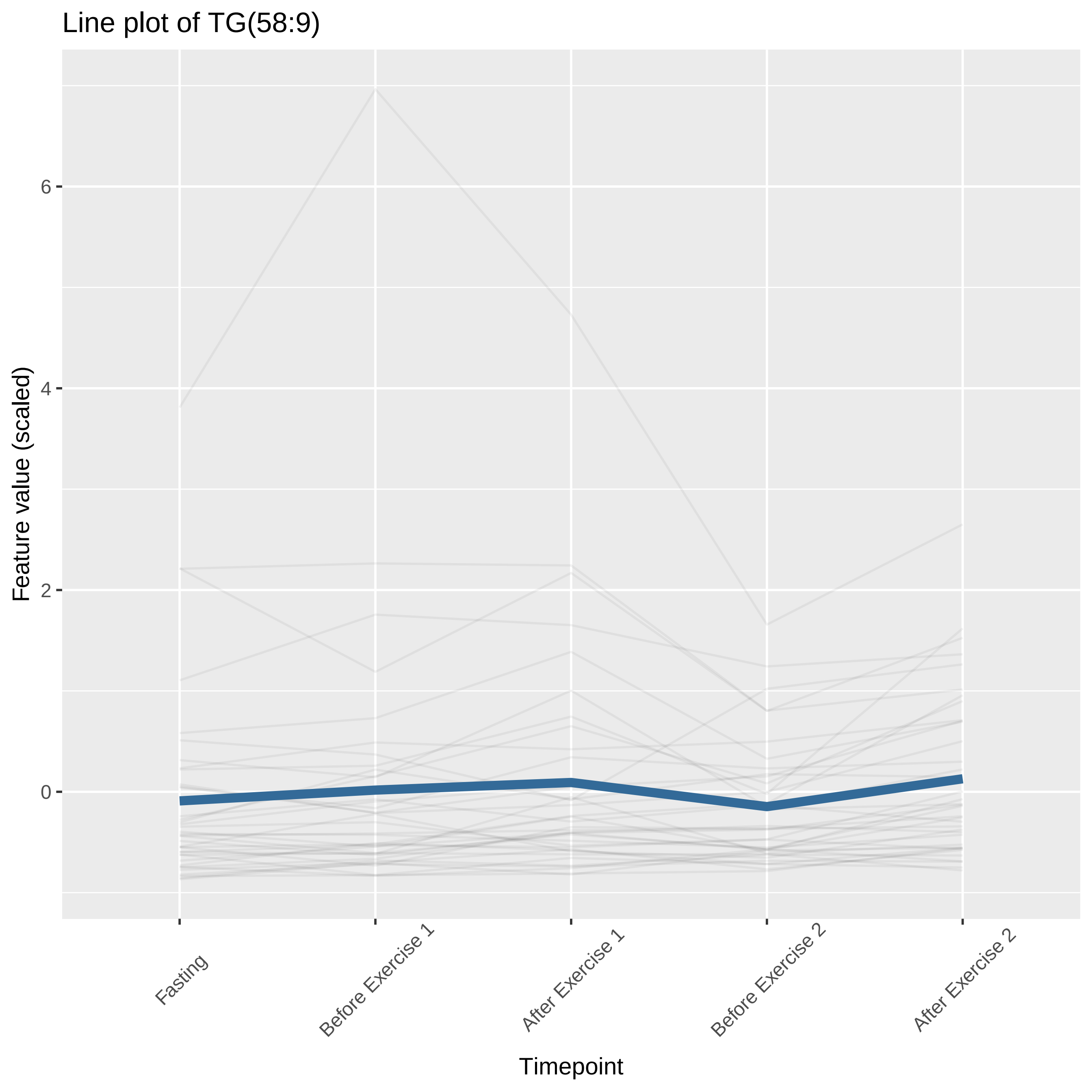

Line plot of TG(58:9)
Feature value (scaled)
6
4
2
0
Fasting
Before Exercise 1
After Exercise 1
Before Exercise 2
After Exercise 2
Timepoint

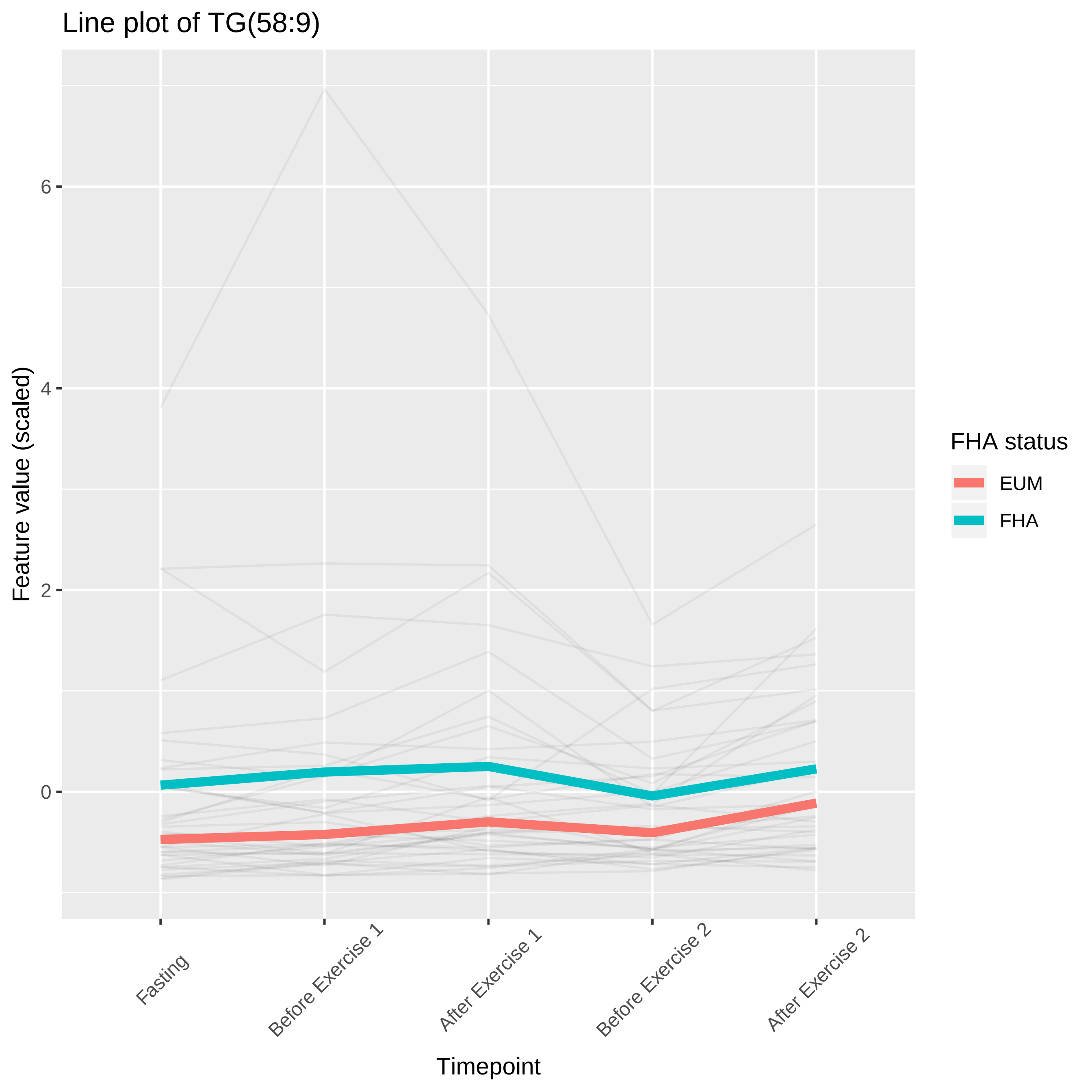

Line plot of TG(58:9)
Feature value (scaled)
0
2
4
6
Fasting
Before Exercise 1
After Exercise 1
Before Exercise 2
After Exercise 2
Timepoint
FHA status
EUM
FHA

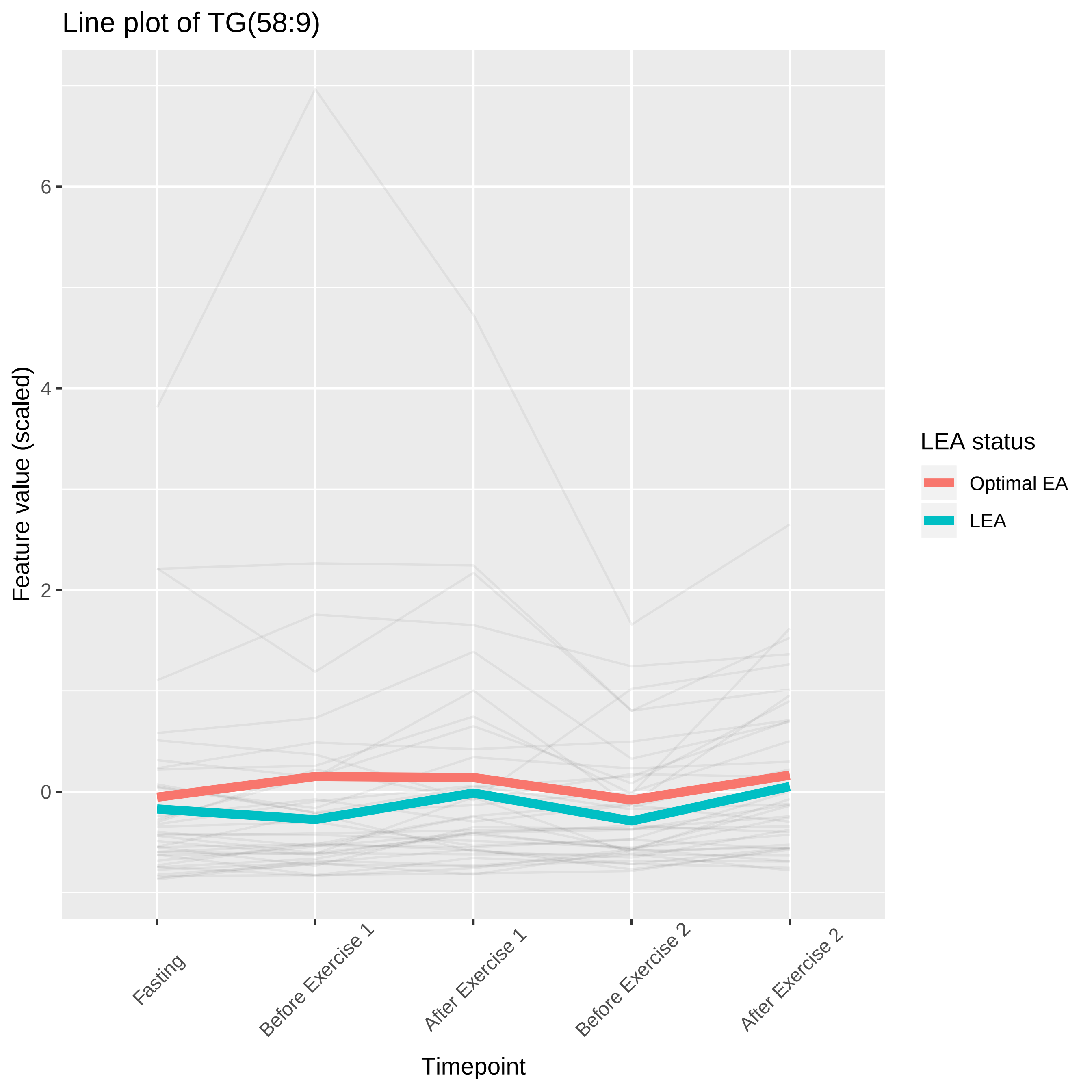

Line plot of TG(58:9)
Feature value (scaled)
6
4
2
0
Fasting
Before Exercise 1
After Exercise 1
Before Exercise 2
After Exercise 2
Timepoint
LEA status
Optimal EA
LEA

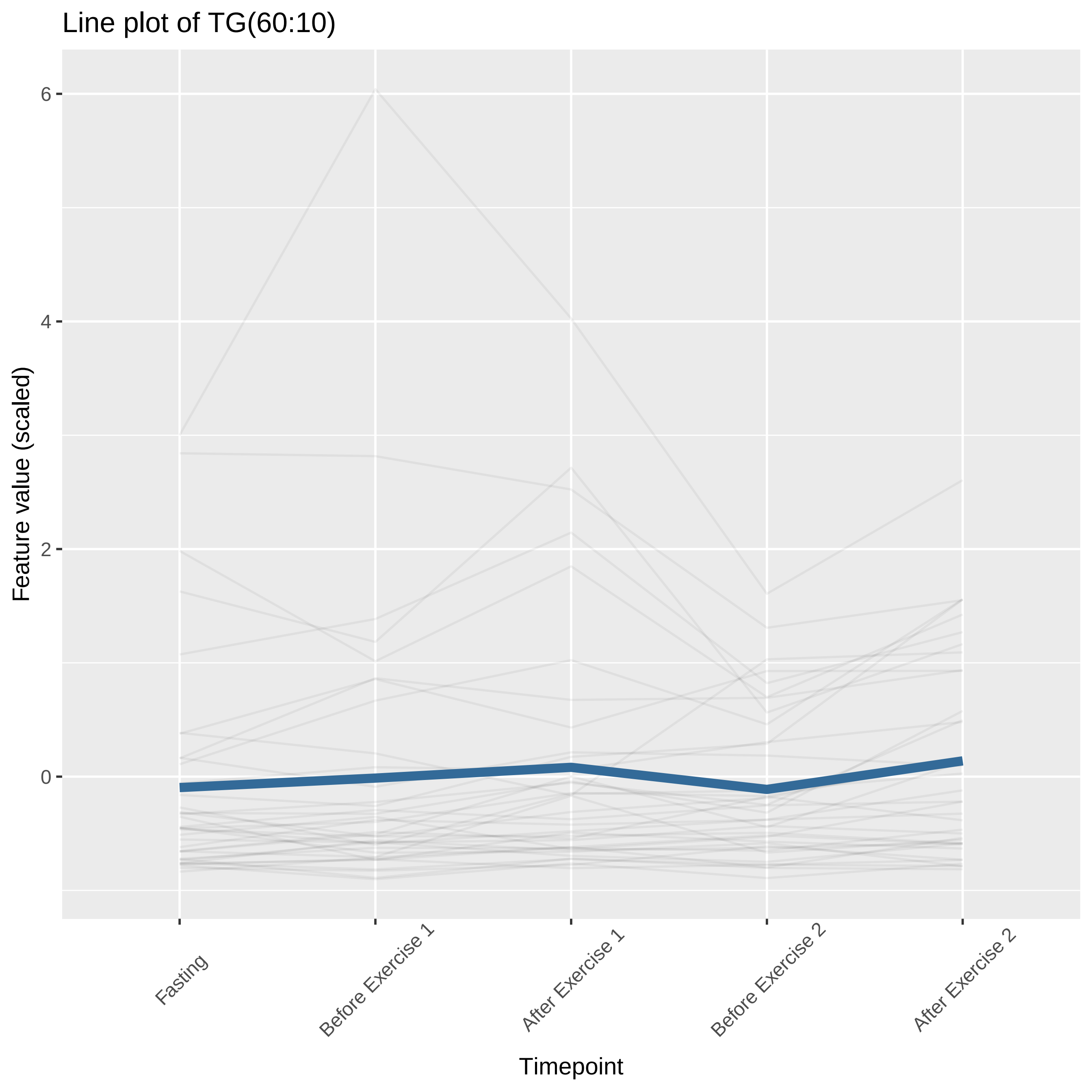

Line plot of TG(60:10)
Feature value (scaled)
6
4
2
0
Fasting
Before Exercise 1
After Exercise 1
Before Exercise 2
After Exercise 2
Timepoint

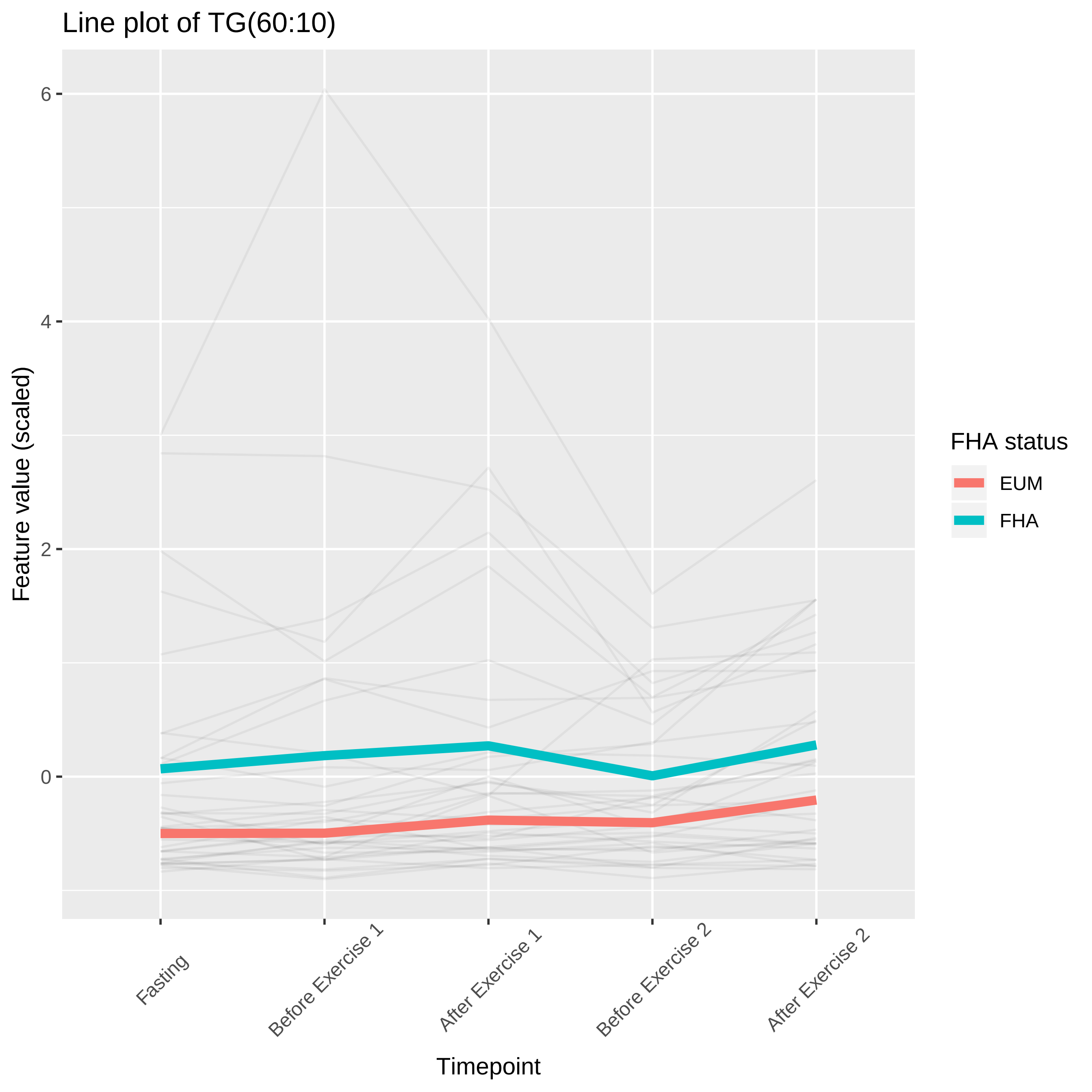

Line plot of TG(60:10)
Feature value (scaled)
6
4
2
0
Fasting
Before Exercise 1
After Exercise 1
Before Exercise 2
After Exercise 2
Timepoint
FHA status
EUM
FHA

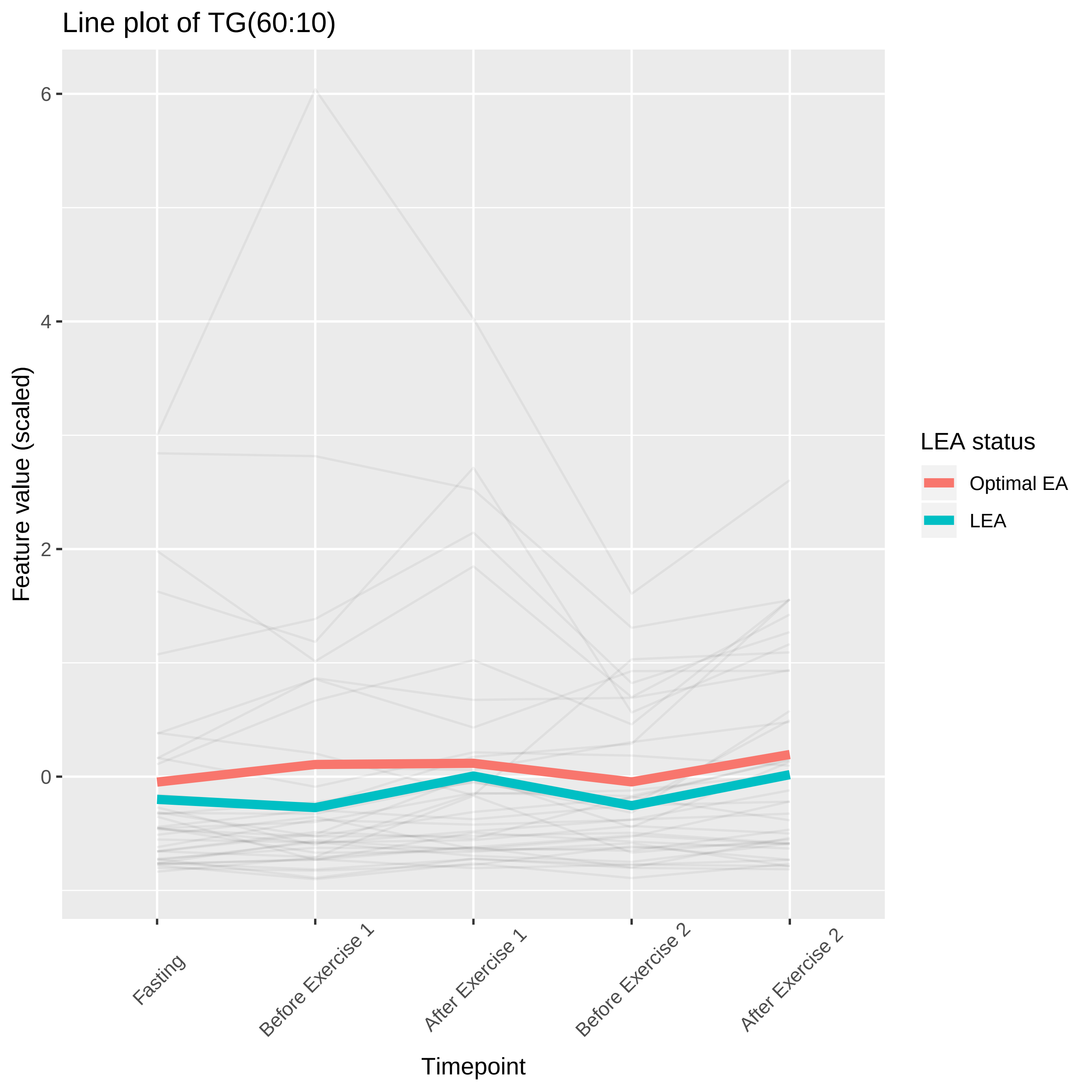

Line plot of TG(60:10)
Feature value (scaled)
6
4
2
0
Fasting
Before Exercise 1
After Exercise 1
Before Exercise 2
After Exercise 2
Timepoint
LEA status
Optimal EA
LEA

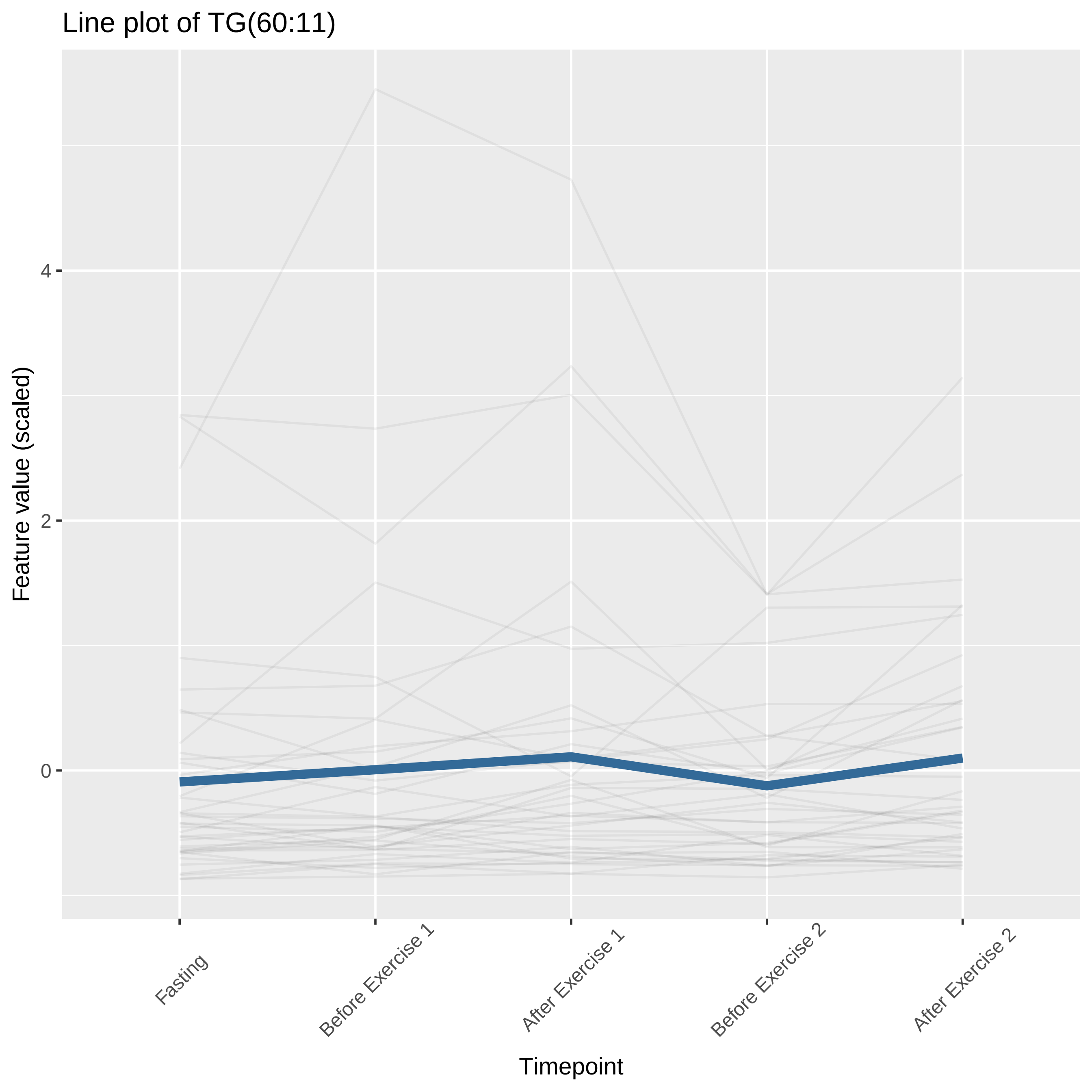

Line plot of TG(60:11)
Feature value (scaled)
4
2
0
Fasting
Before Exercise 1
After Exercise 1
Before Exercise 2
After Exercise 2
Timepoint

Line plot of TG(60:11)

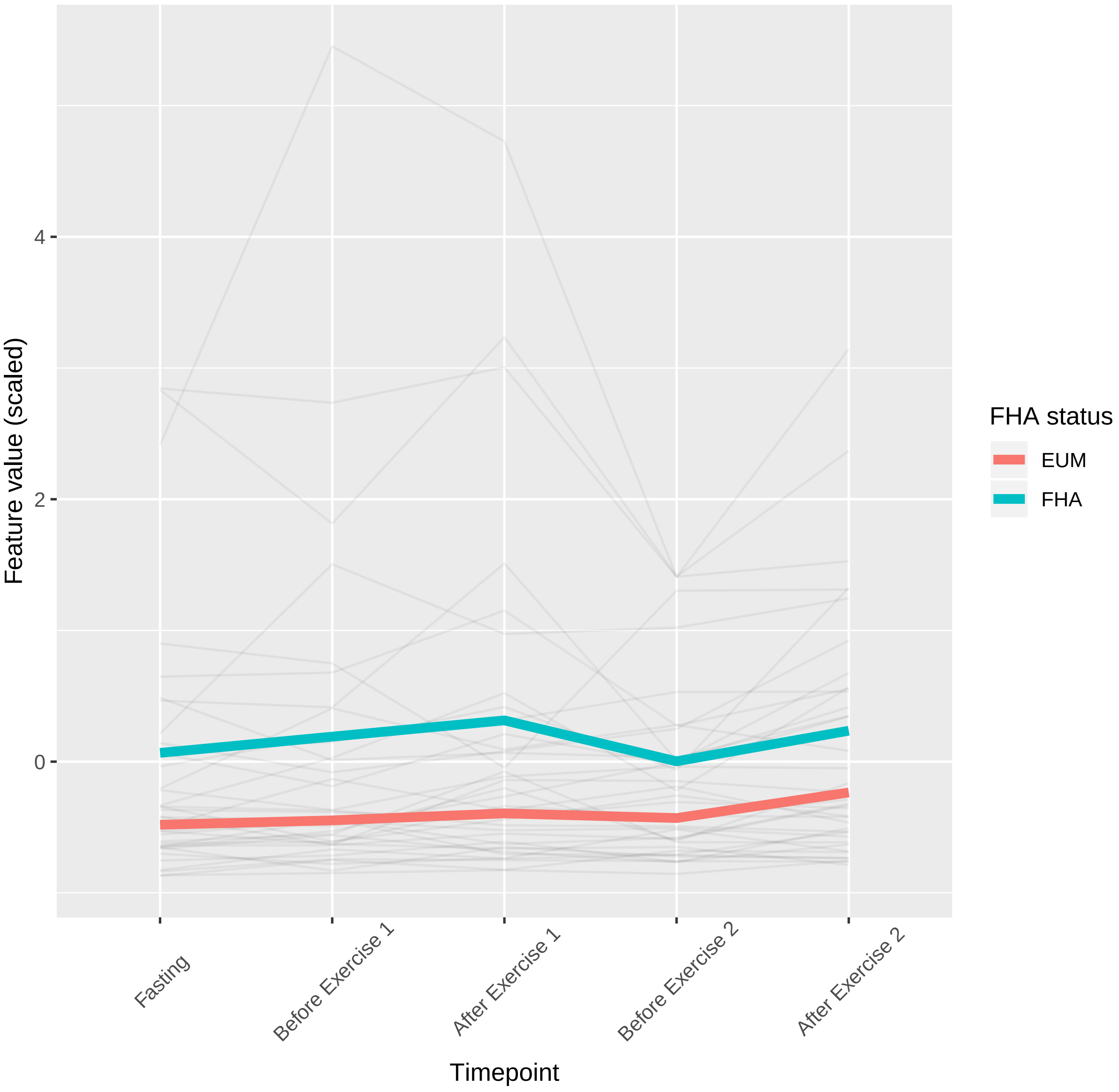

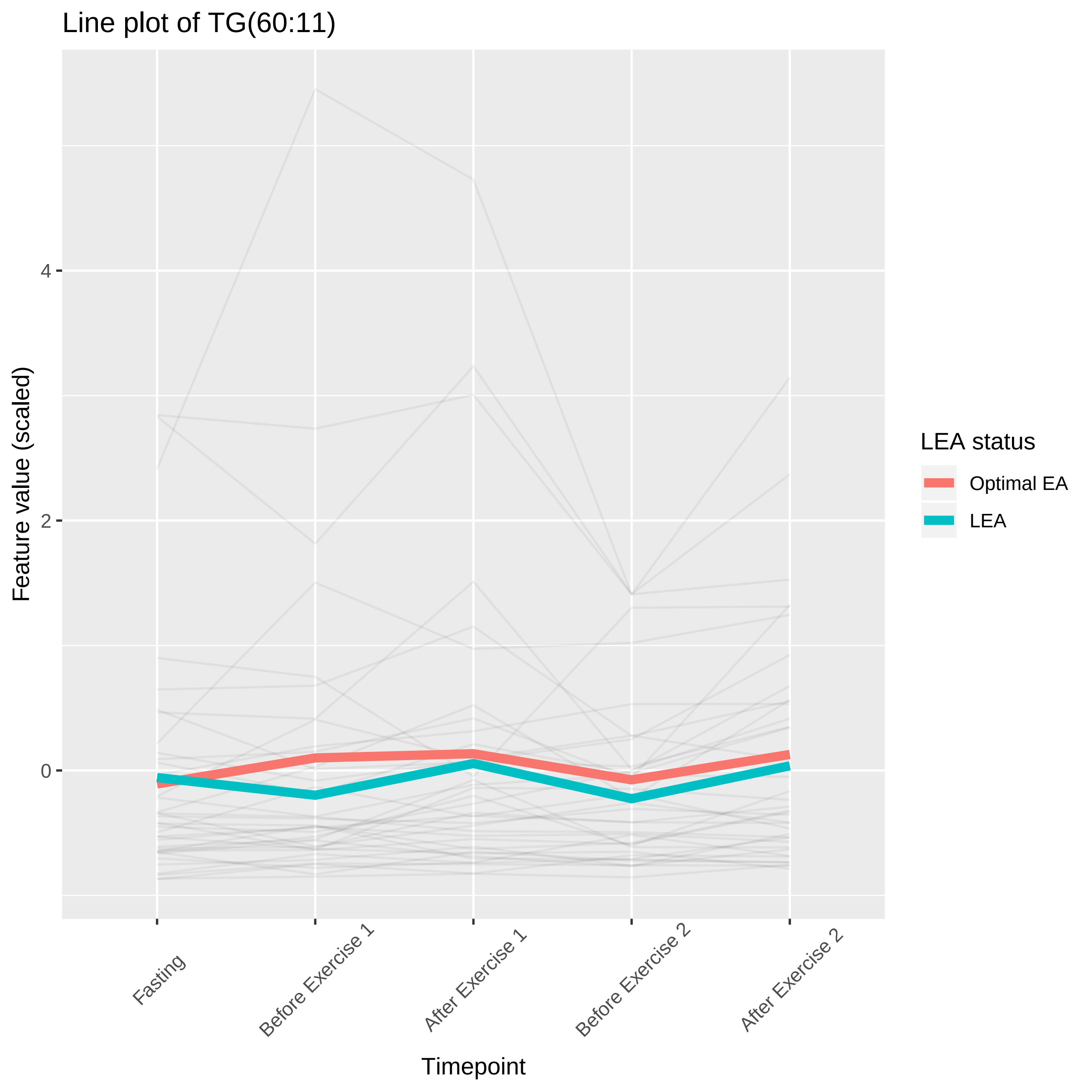

Line plot of TG(60:11)
Feature value (scaled)
4
2
0
Fasting
Before Exercise 1
After Exercise 1
Before Exercise 2
After Exercise 2
Timepoint
LEA status
Optimal EA
LEA

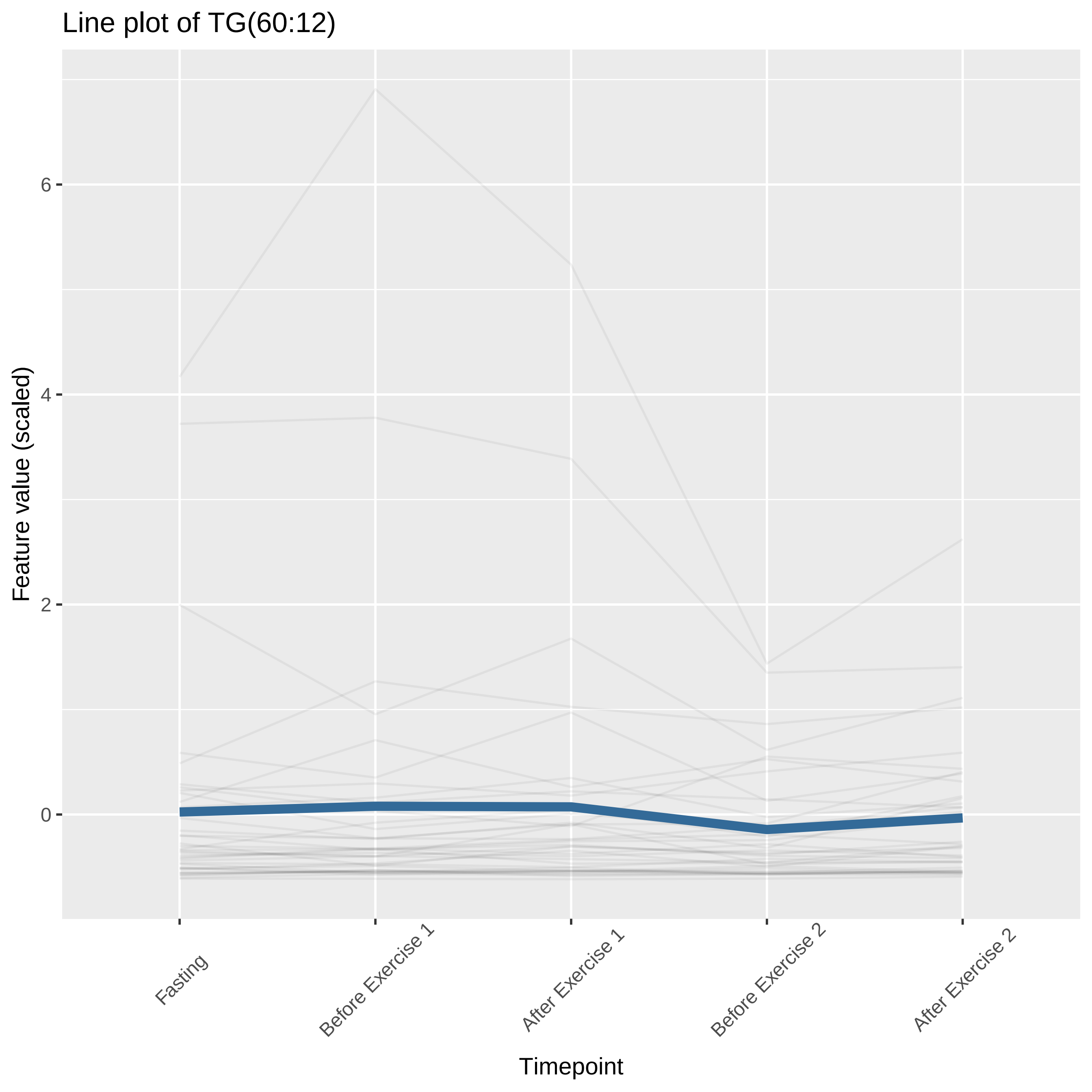
Line plot of TG(60:12)
Feature value (scaled)
0
2
4
6
Fasting
Before Exercise 1
After Exercise 1
Before Exercise 2
After Exercise 2
Timepoint

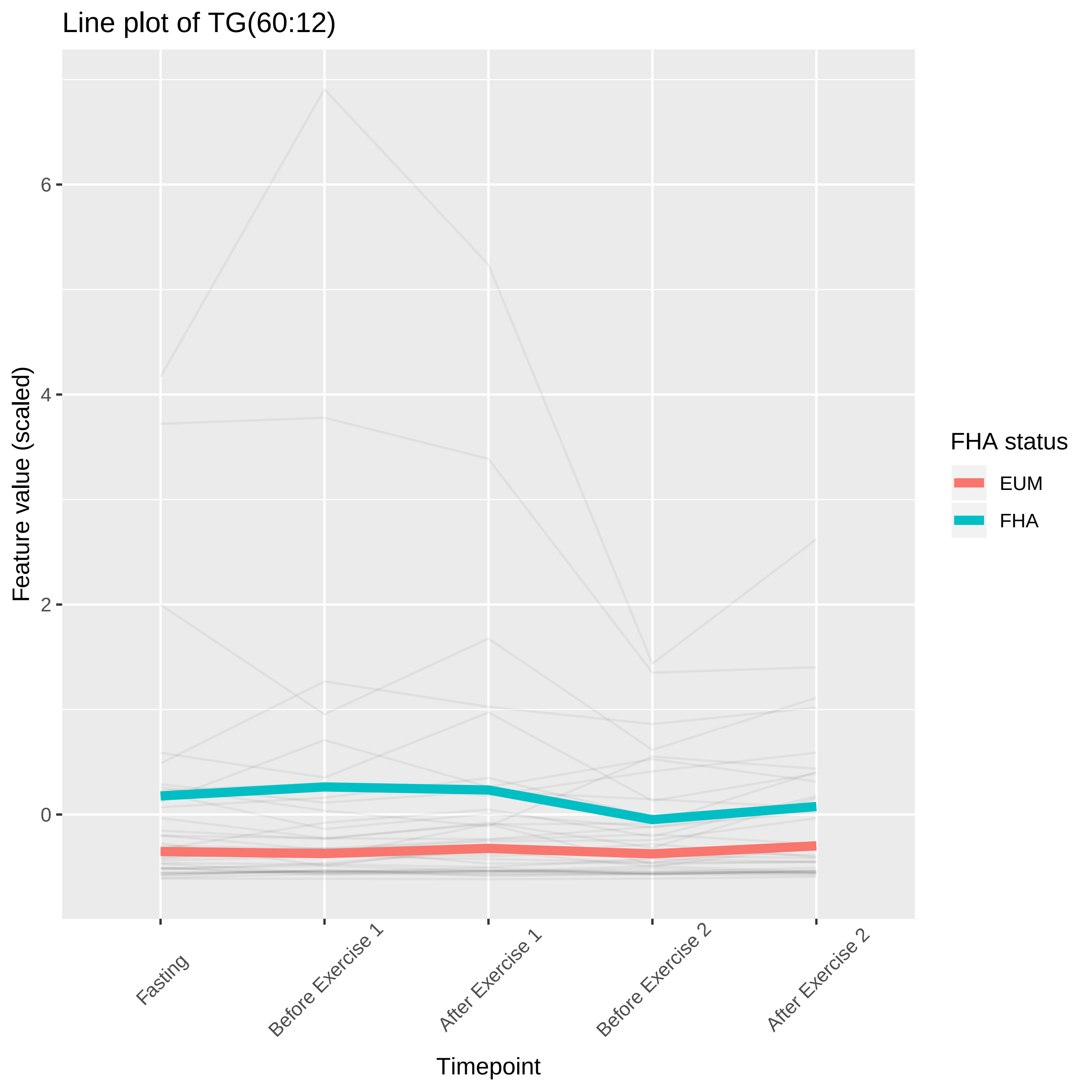

Line plot of TG(60:12)
Feature value (scaled)
0
2
4
6
Fasting
Before Exercise 1
After Exercise 1
Before Exercise 2
After Exercise 2
Timepoint
FHA status
EUM
FHA

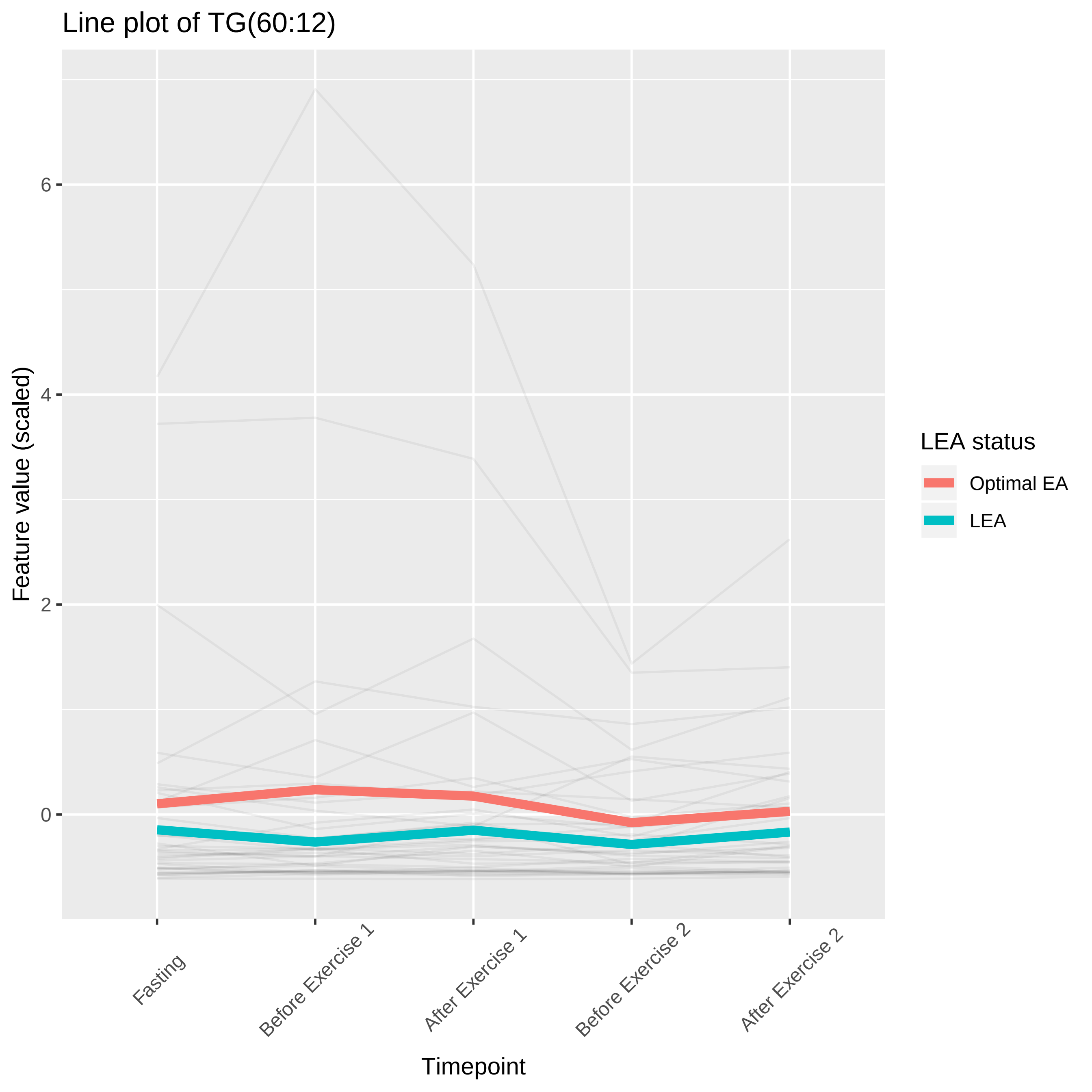

Line plot of TG(60:12)
Feature value (scaled)
6
4
2
0
Fasting
Before Exercise 1
After Exercise 1
Before Exercise 2
After Exercise 2
Timepoint
LEA status
Optimal EA
LEA

Line plot of TG(60:7)

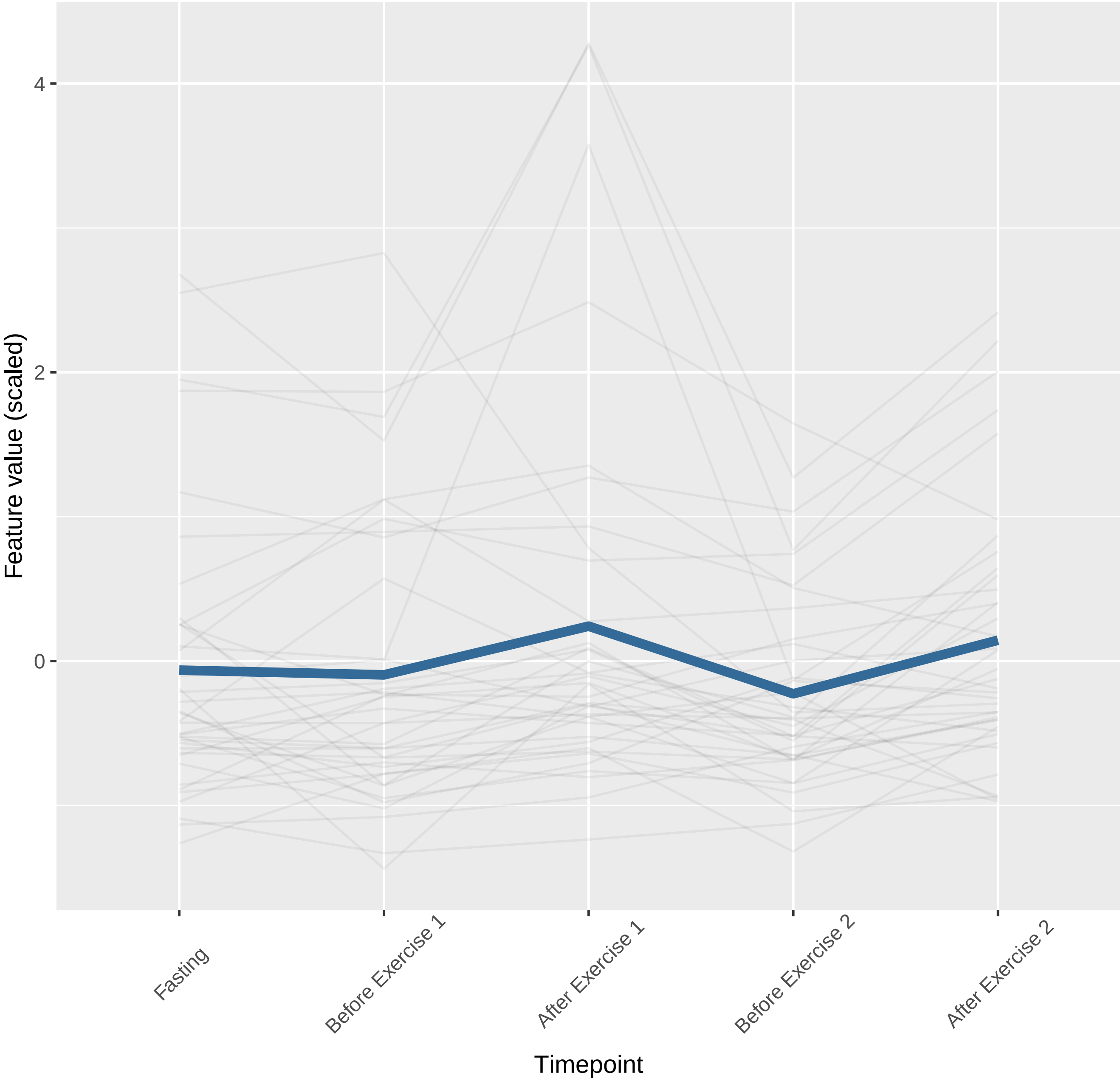

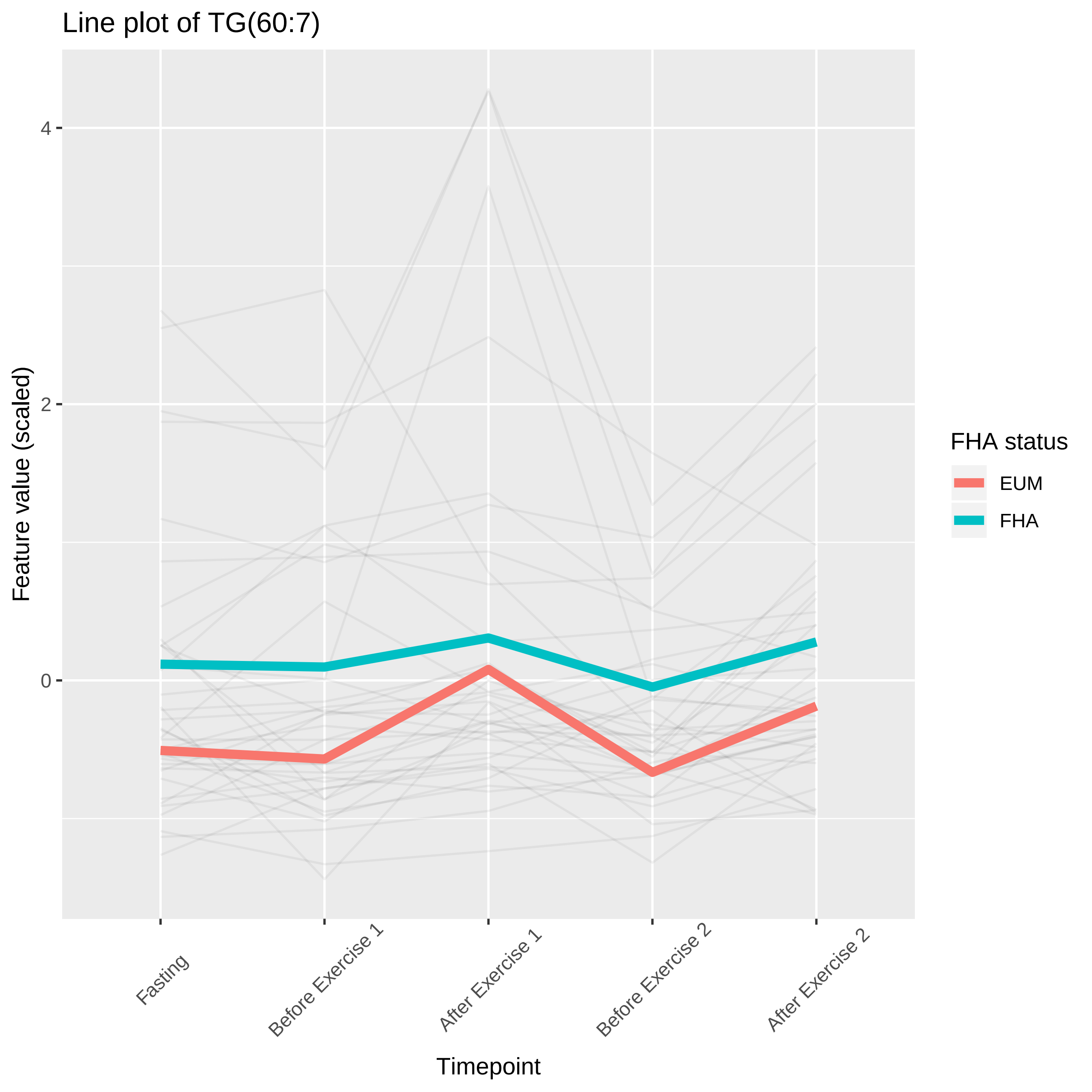
Line plot of TG(60:7)
Feature value (scaled)
4
2
0
Fasting
Before Exercise 1
After Exercise 1
Before Exercise 2
After Exercise 2
Timepoint
FHA status
EUM
FHA

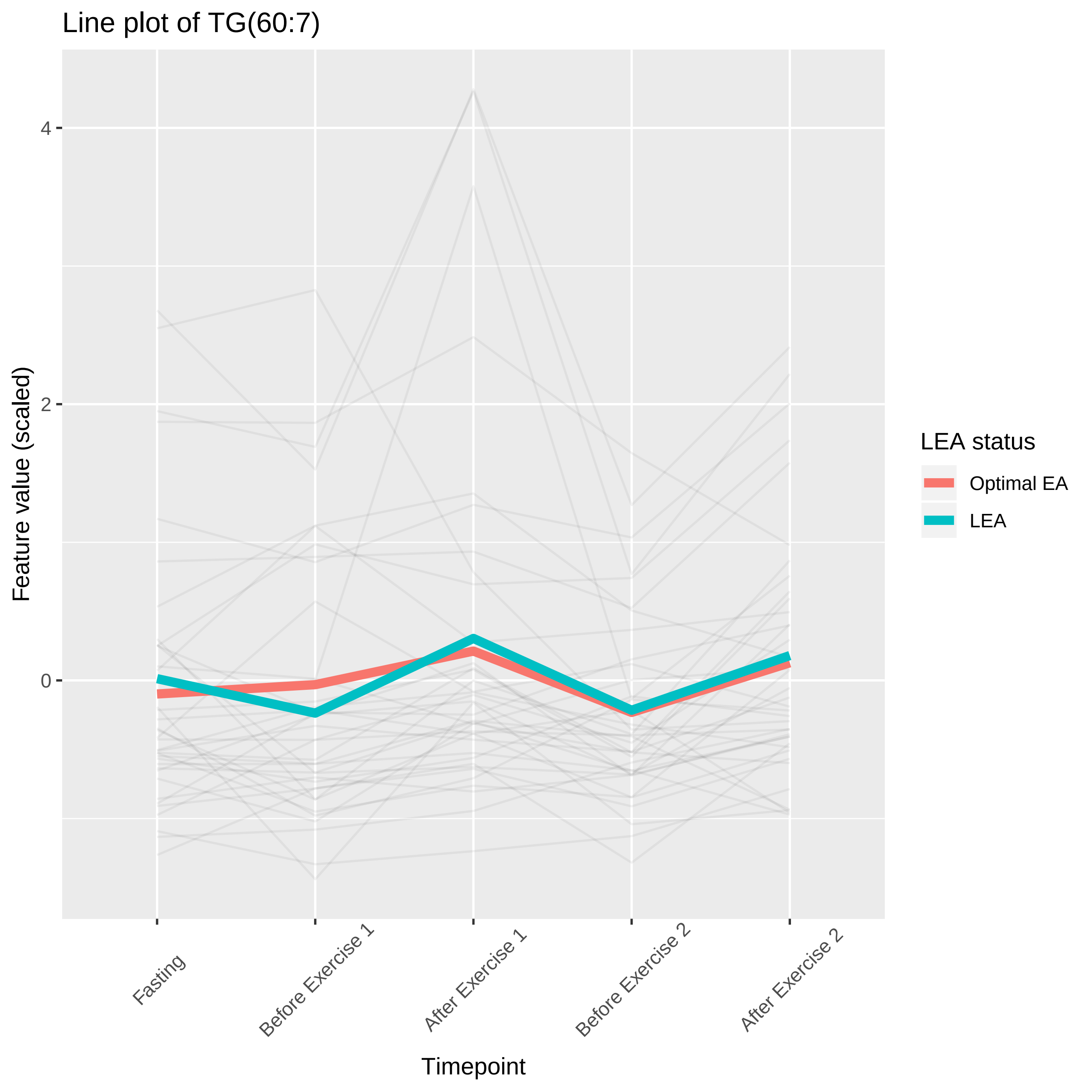

Line plot of TG(60:7)
Feature value (scaled)
4
2
0
Fasting
Before Exercise 1
After Exercise 1
Before Exercise 2
After Exercise 2
Timepoint
LEA status
Optimal EA
LEA

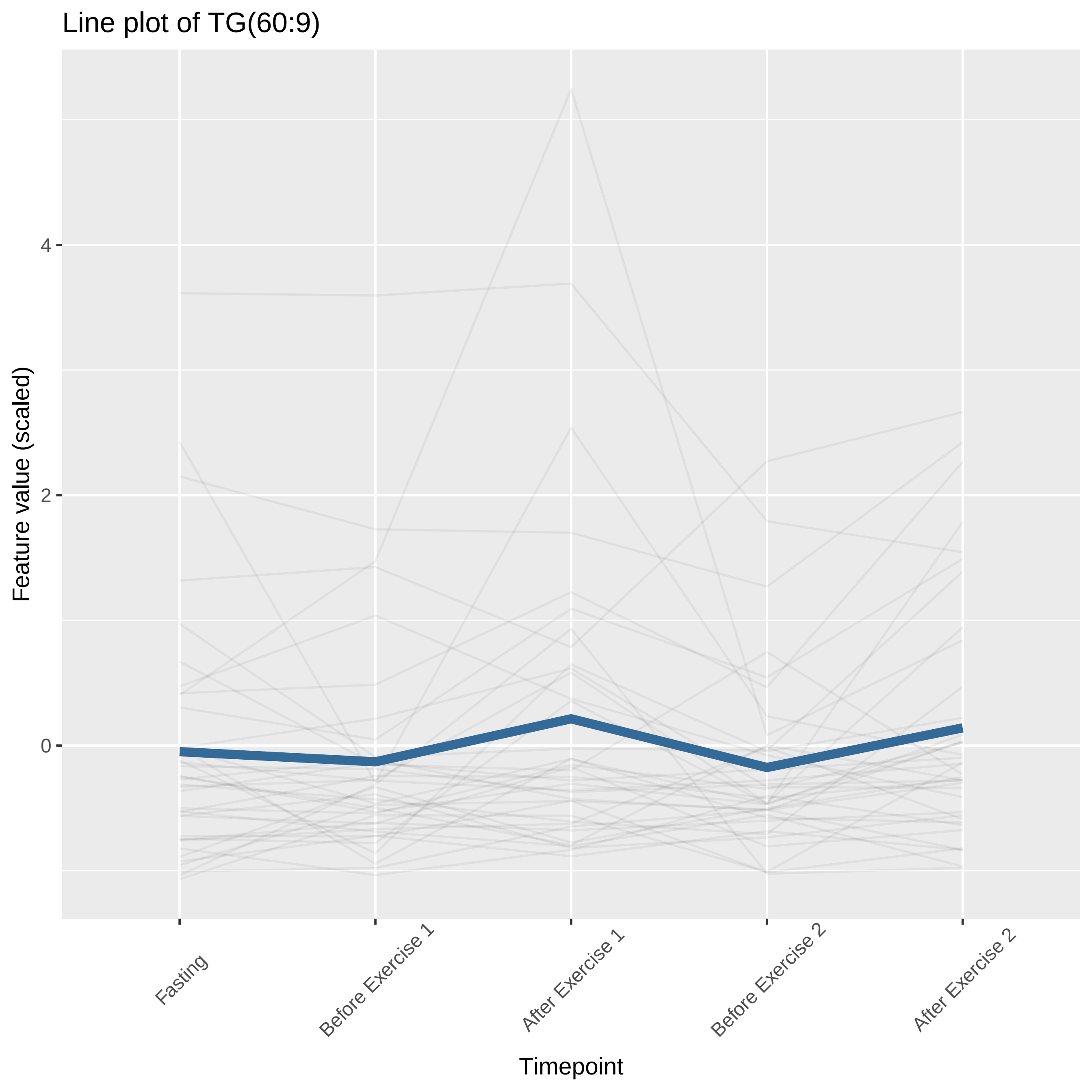

Line plot of TG(60:9)
Feature value (scaled)
4
2
0
Fasting
Before Exercise 1
After Exercise 1
Before Exercise 2
After Exercise 2
Timepoint

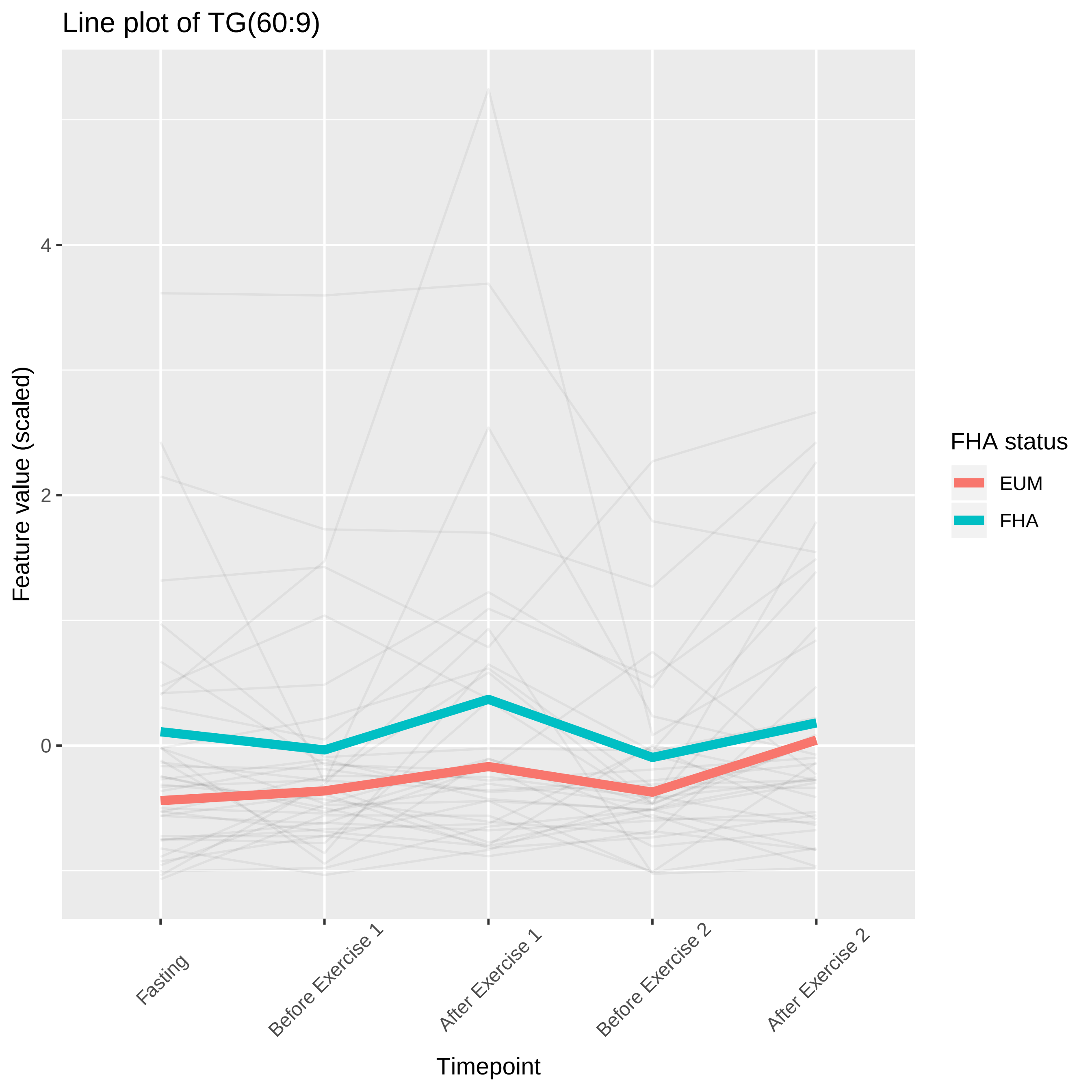

Line plot of TG(60:9)
Feature value (scaled)
4
2
0
Fasting
Before Exercise 1
After Exercise 1
Before Exercise 2
After Exercise 2
Timepoint
FHA status
EUM
FHA

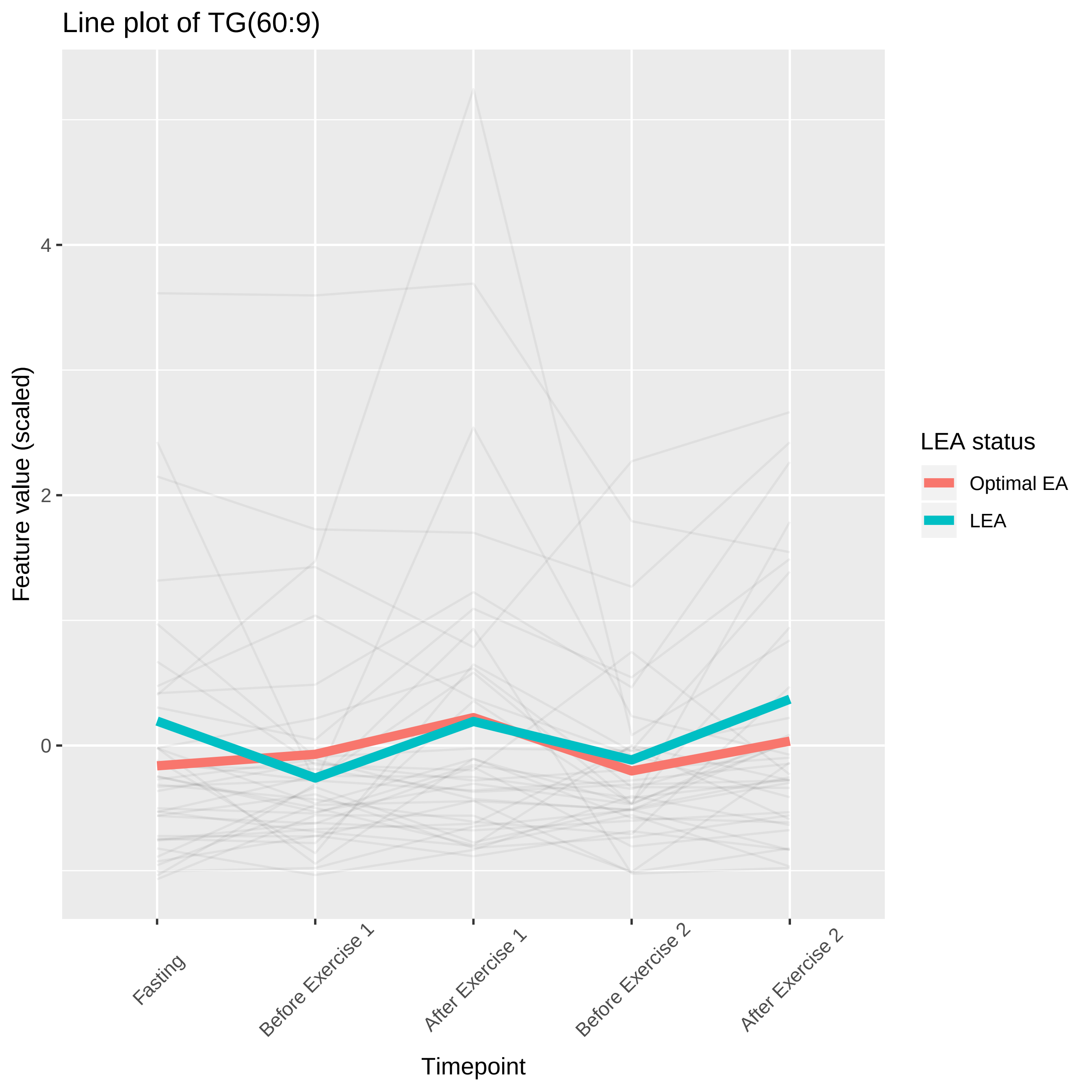

Line plot of TG(60:9)
Feature value (scaled)
4
2
0
Fasting
Before Exercise 1
After Exercise 1
Before Exercise 2
After Exercise 2
Timepoint
LEA status
Optimal EA
LEA

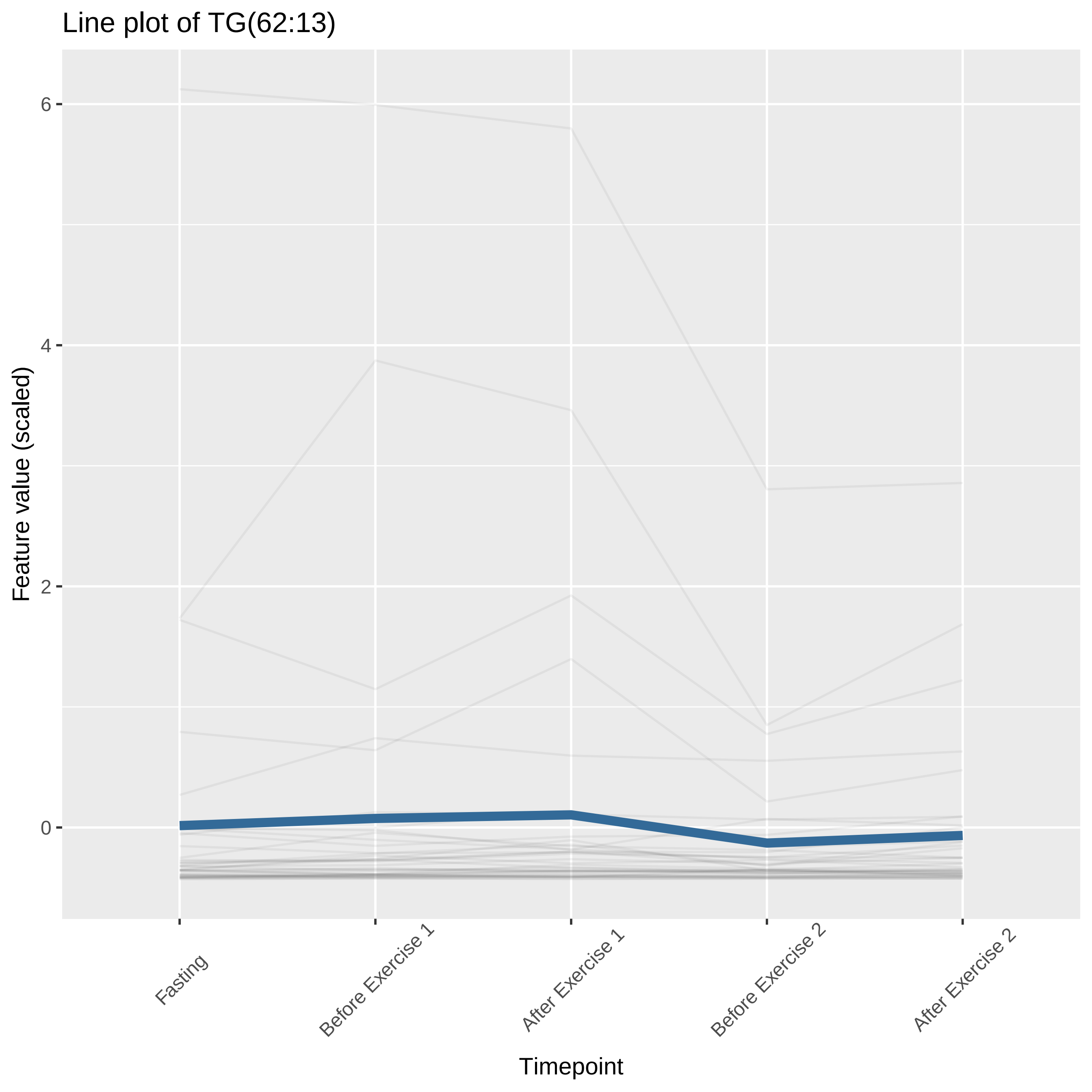

Line plot of TG(62:13)
Feature value (scaled)
6
4
2
0
Fasting
Before Exercise 1
After Exercise 1
Before Exercise 2
After Exercise 2
Timepoint

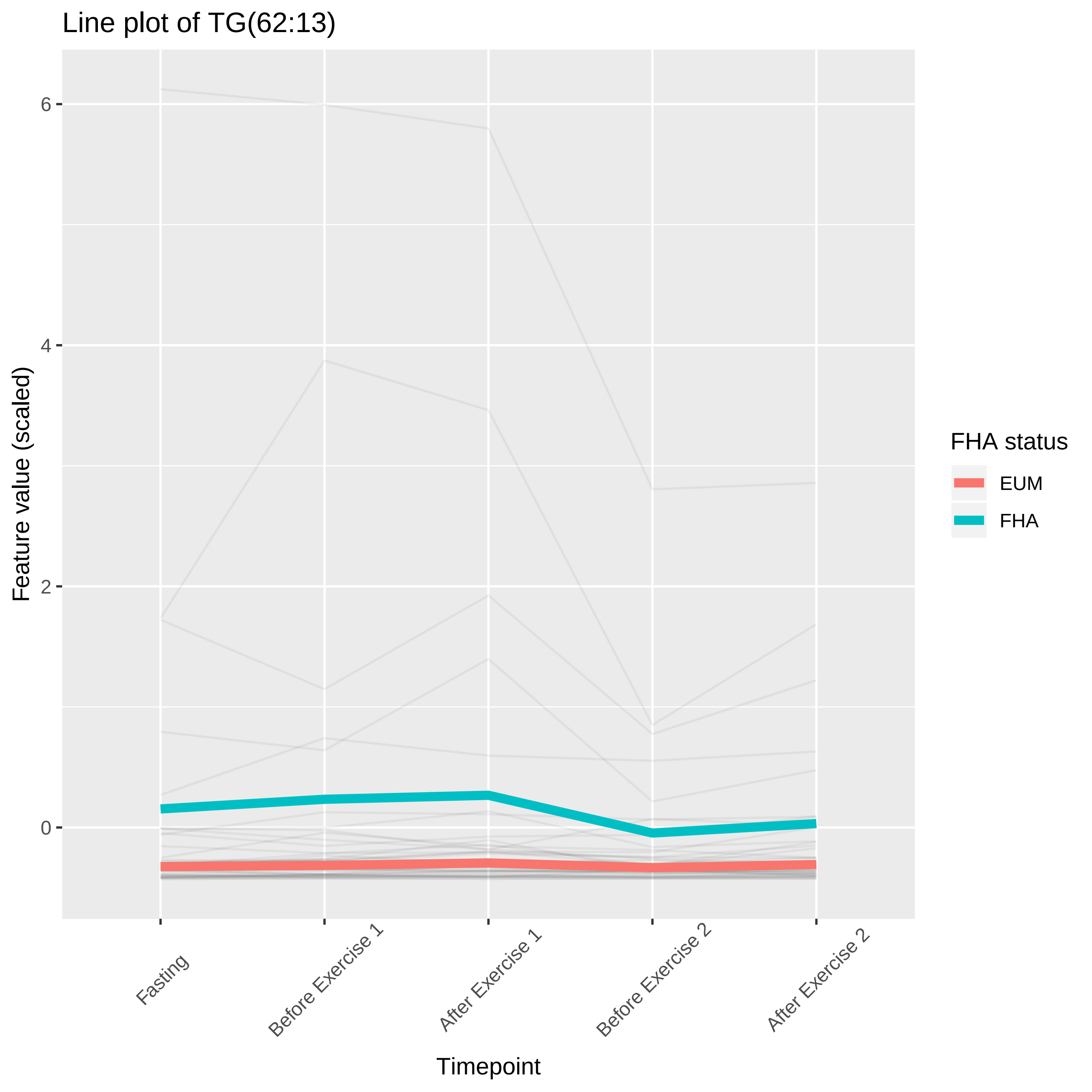

Line plot of TG(62:13)
Feature value (scaled)
6
4
2
0
Fasting
Before Exercise 1
After Exercise 1
Before Exercise 2
After Exercise 2
Timepoint
FHA status
EUM
FHA

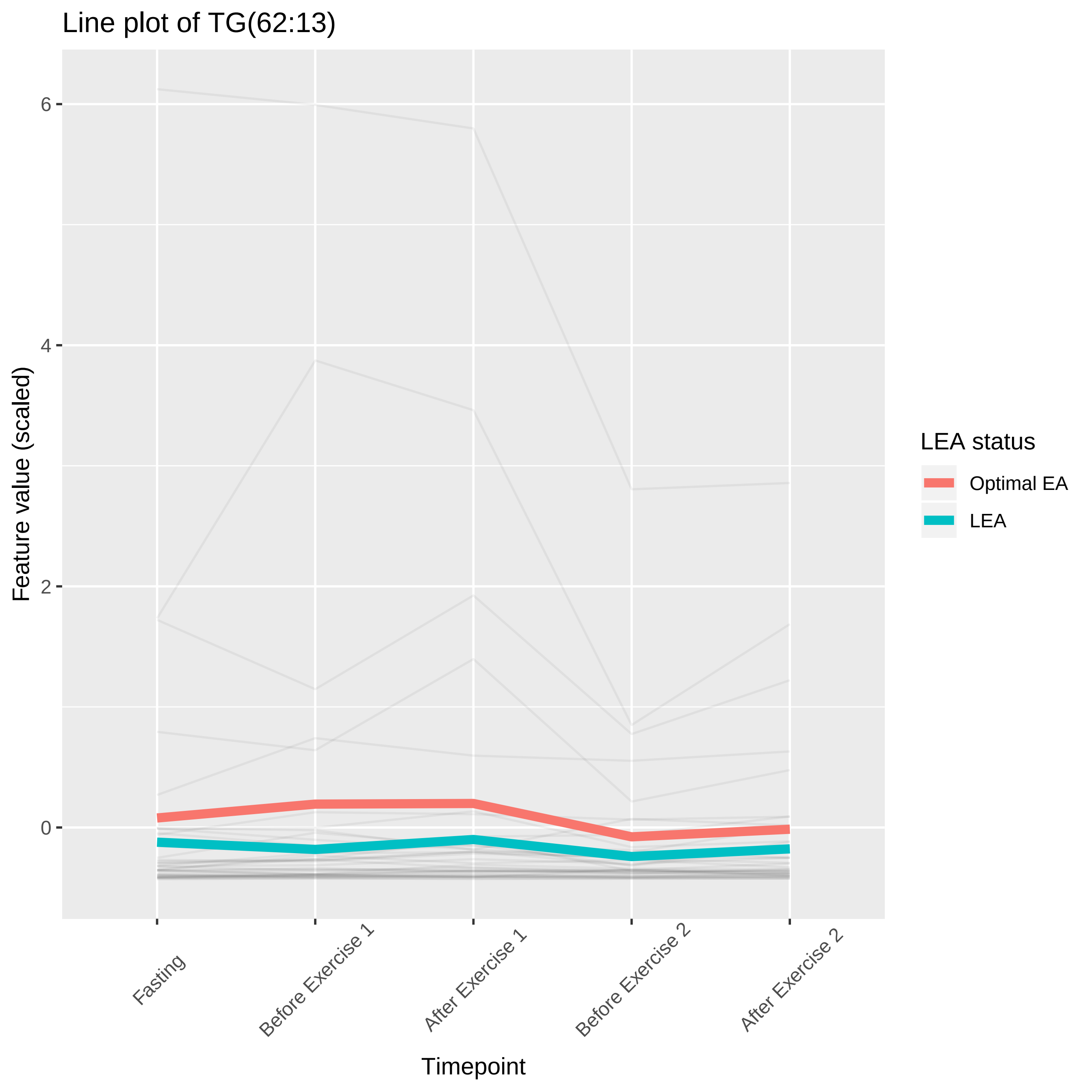
Line plot of TG(62:13)
Feature value (scaled)
6
4
2
0
Fasting
Before Exercise 1
After Exercise 1
Before Exercise 2
After Exercise 2
Timepoint
LEA status
Optimal EA
LEA

GLYCEROPHOSPHOLIPIDS

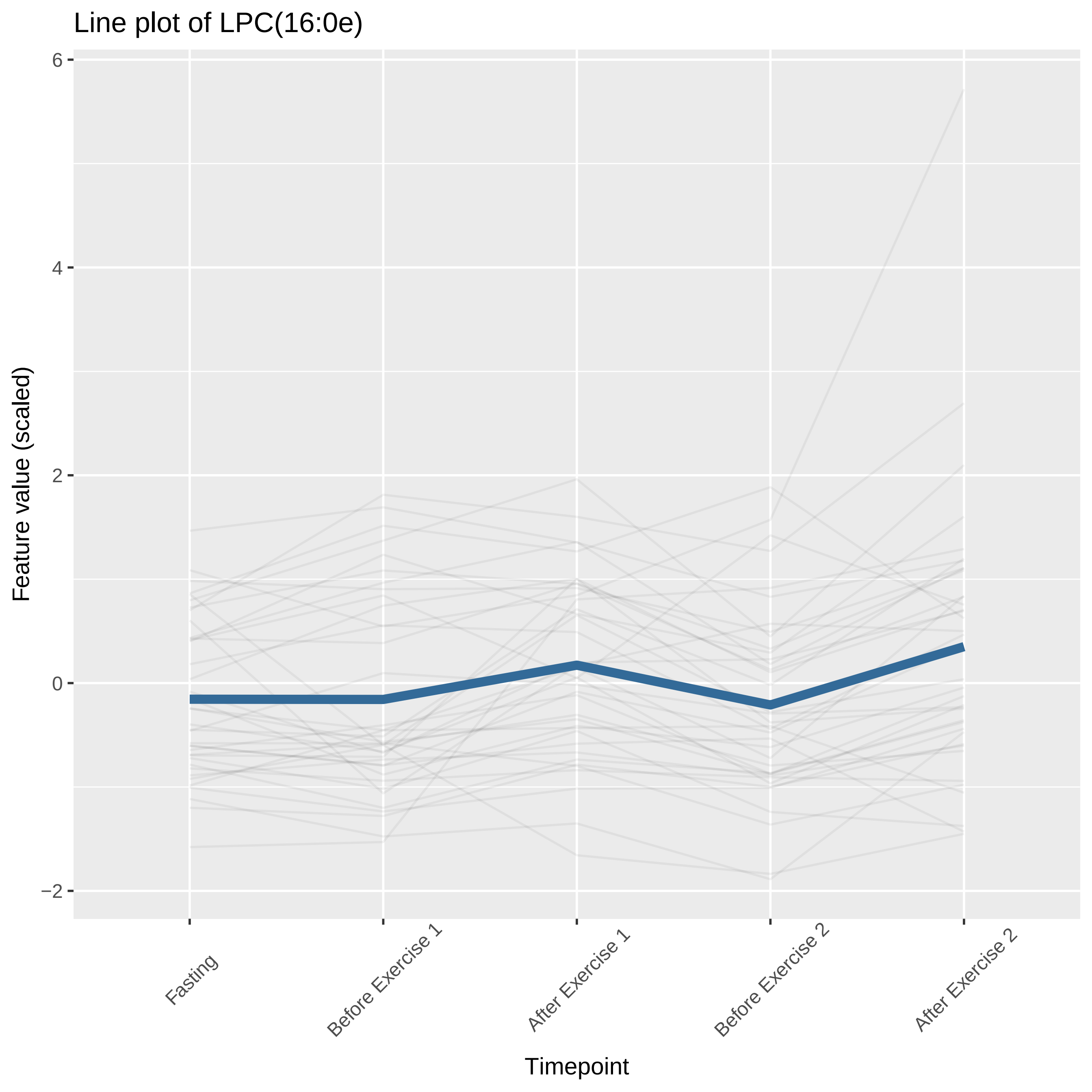

Line plot of LPC(16:0e)
Feature value (scaled)
6
4
2
0
−2
Fasting
Before Exercise 1
After Exercise 1
Before Exercise 2
After Exercise 2
Timepoint

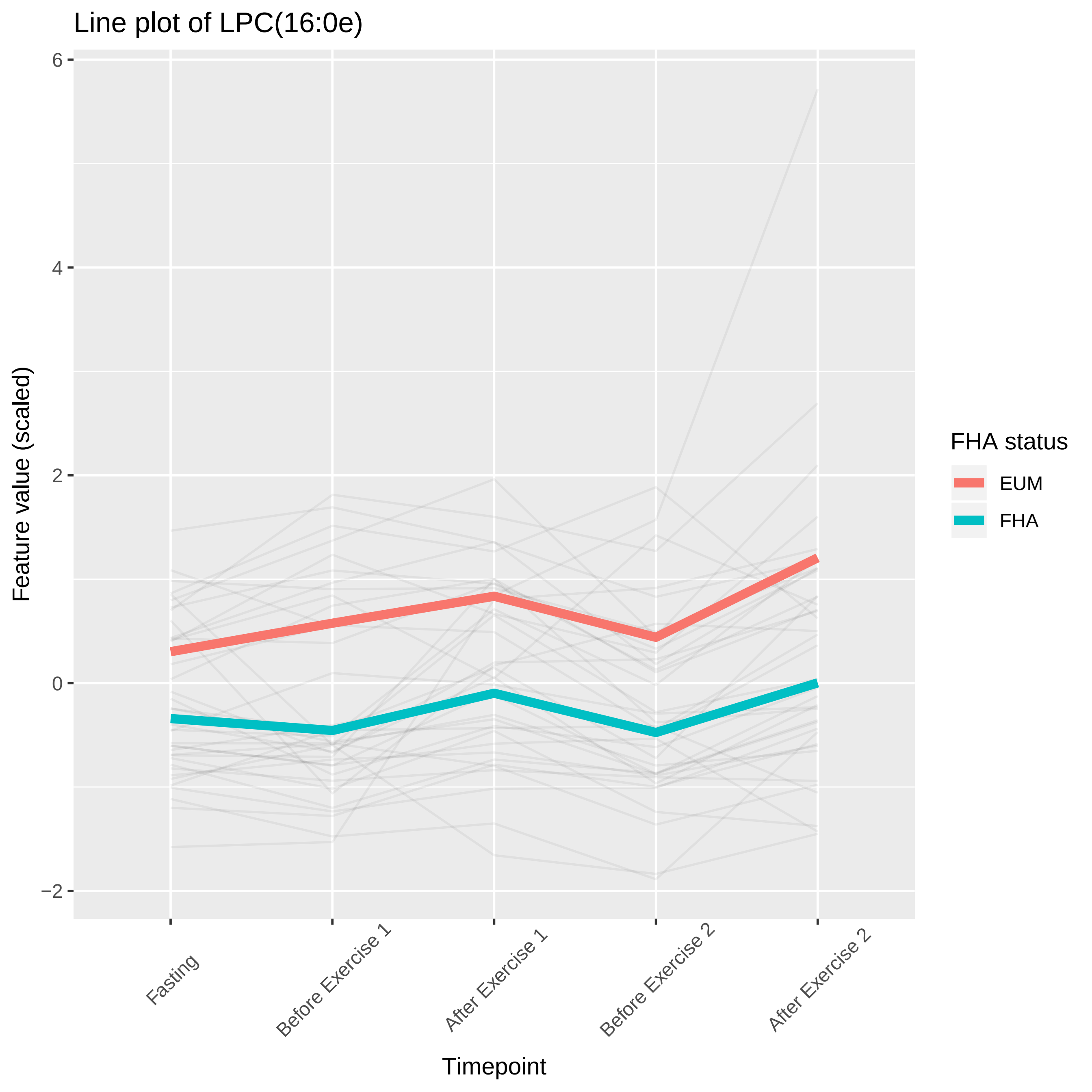

Line plot of LPC(16:0e)
Feature value (scaled)
6
4
2
0
−2
Fasting
Before Exercise 1
After Exercise 1
Before Exercise 2
After Exercise 2
Timepoint
FHA status
EUM
FHA

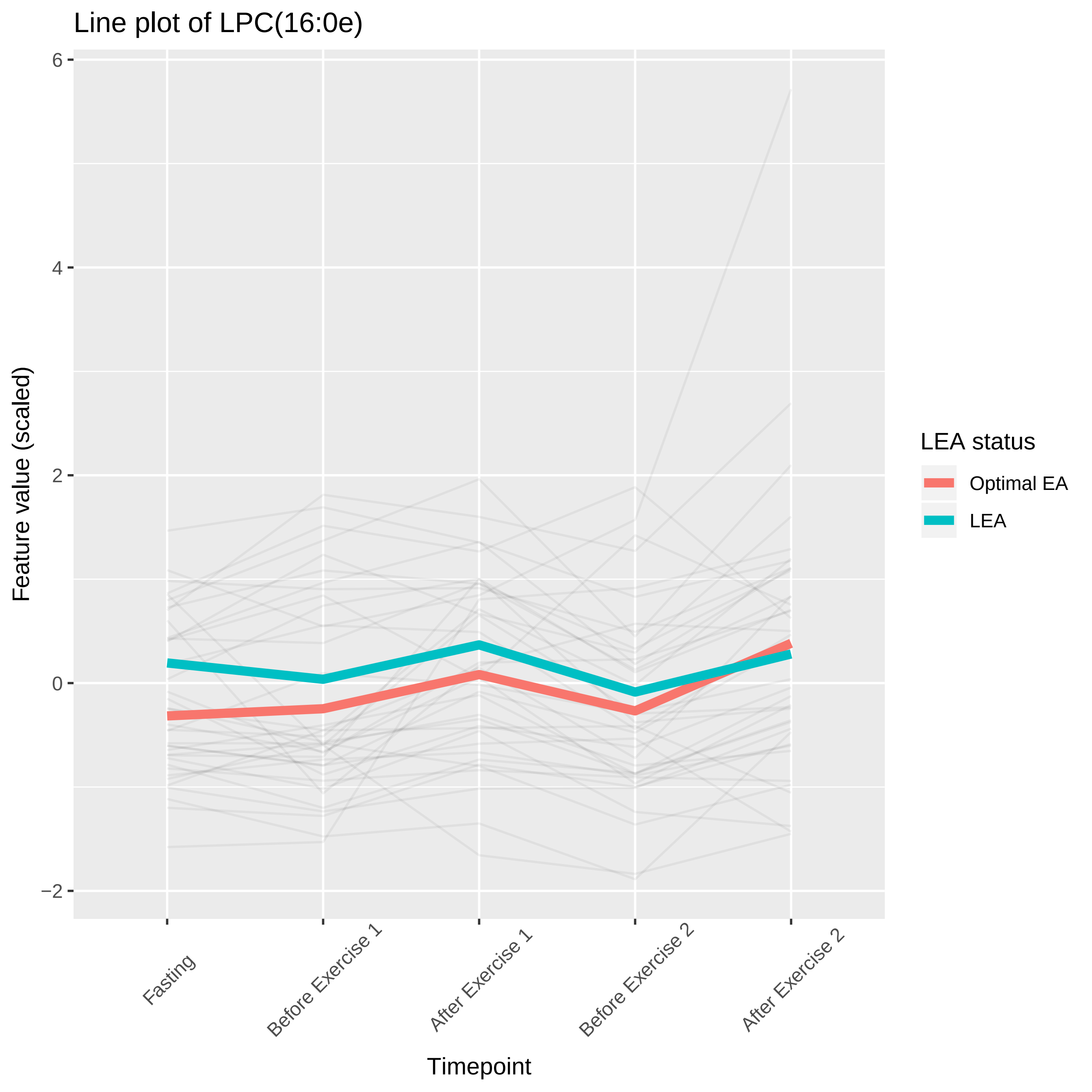
Line plot of LPC(16:0e)
Feature value (scaled)
6
4
2
0
−2
Fasting
Before Exercise 1
After Exercise 1
Before Exercise 2
After Exercise 2
Timepoint
LEA status
Optimal EA
LEA

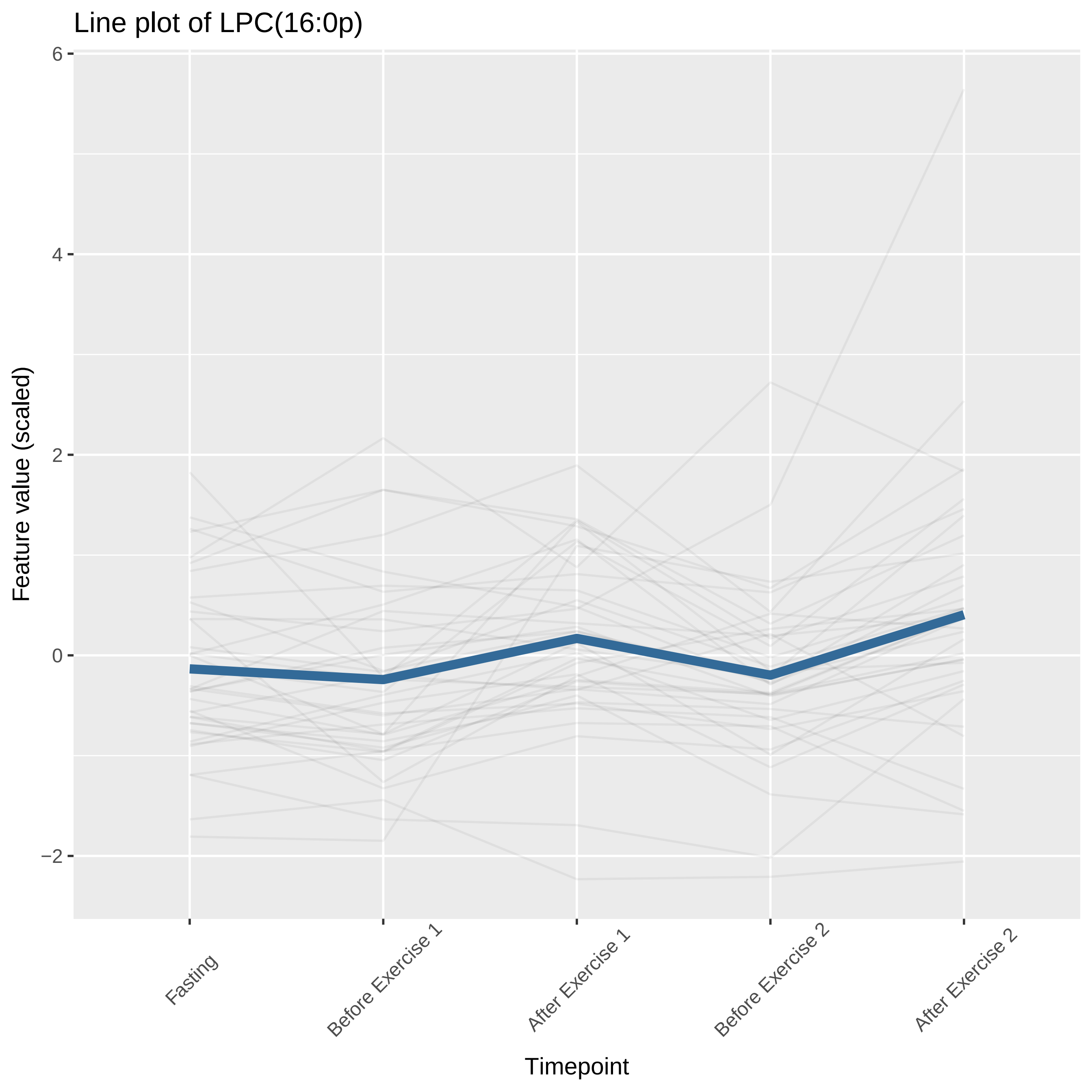

Line plot of LPC(16:0p)
Feature value (scaled)
6
4
2
0
−2
Fasting
Before Exercise 1
After Exercise 1
Before Exercise 2
After Exercise 2
Timepoint

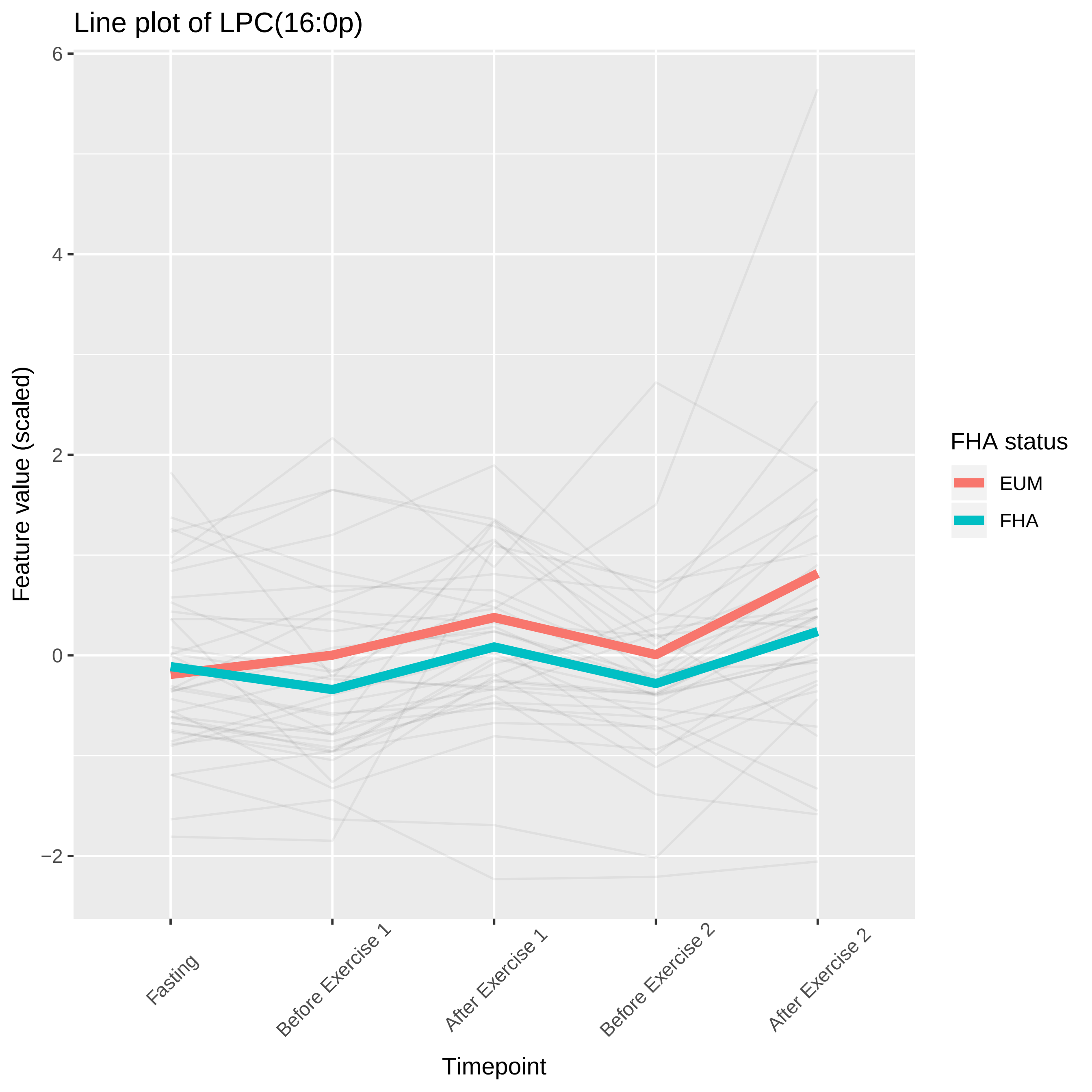

Line plot of LPC(16:0p)
Feature value (scaled)
6
4
2
0
−2
Fasting
Before Exercise 1
After Exercise 1
Before Exercise 2
After Exercise 2
Timepoint
FHA status
EUM
FHA

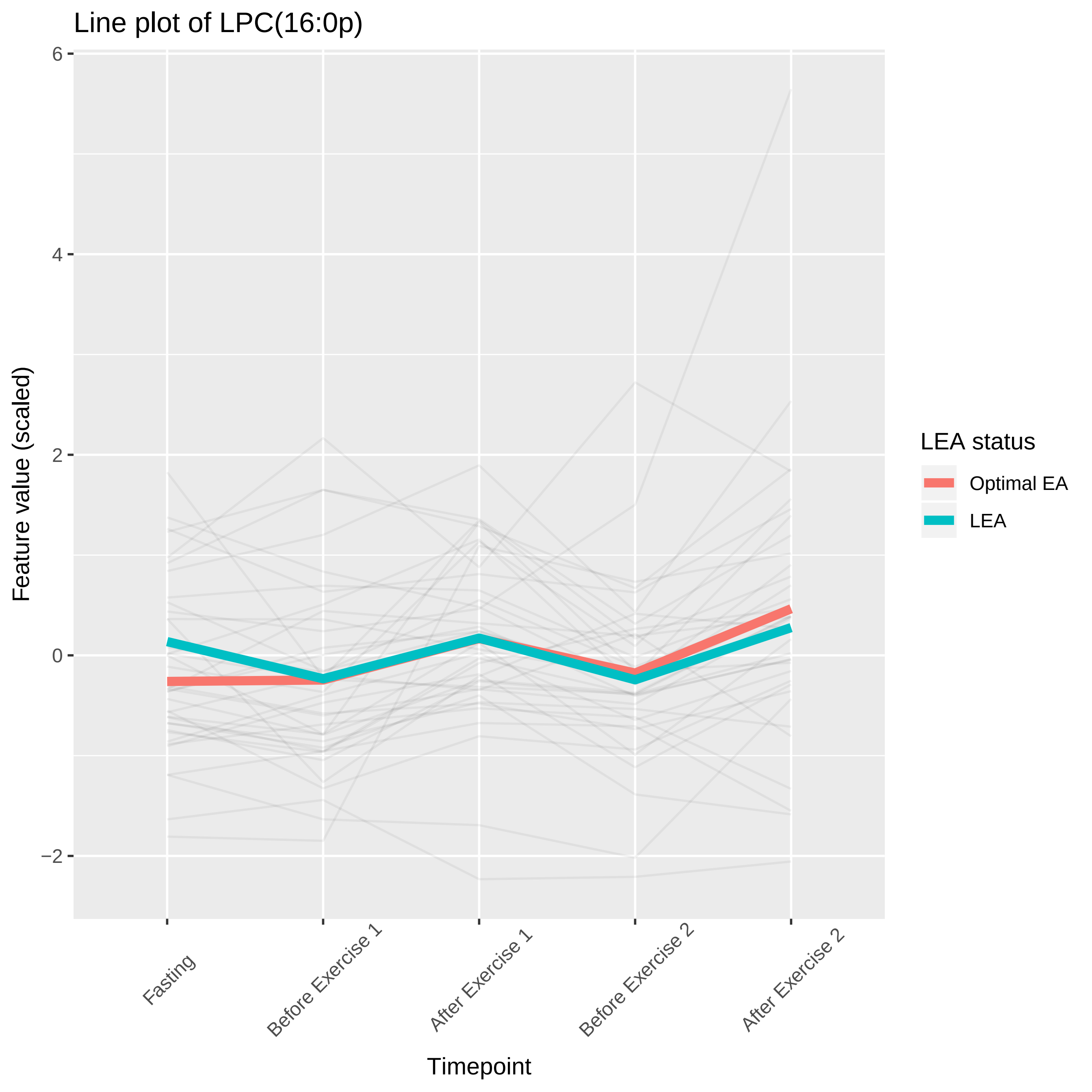

Line plot of LPC(16:0p)
Feature value (scaled)
6
4
2
0
−2
Fasting
Before Exercise 1
After Exercise 1
Before Exercise 2
After Exercise 2
Timepoint
LEA status
Optimal EA
LEA

Line plot of LPC(16:1)

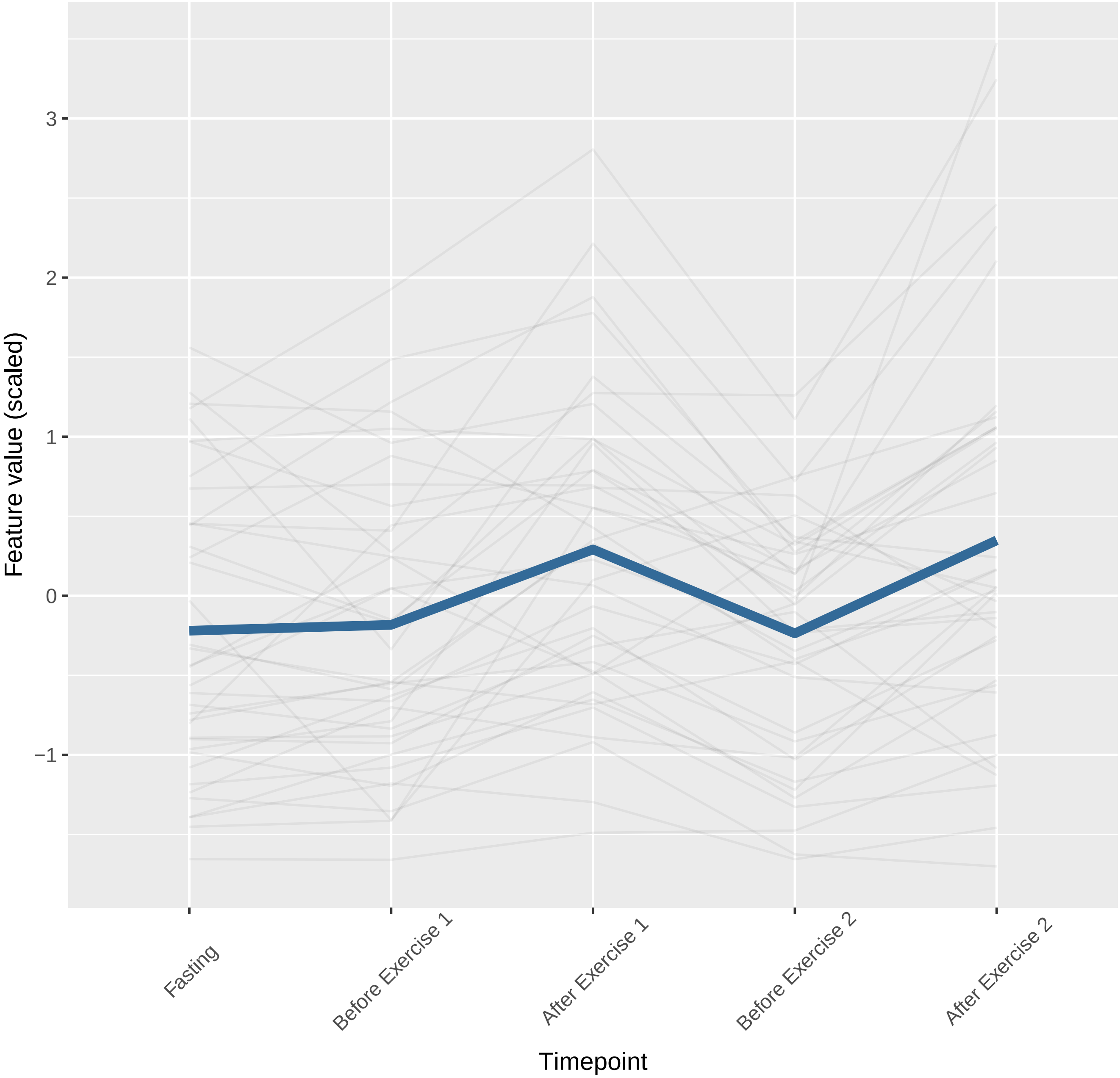

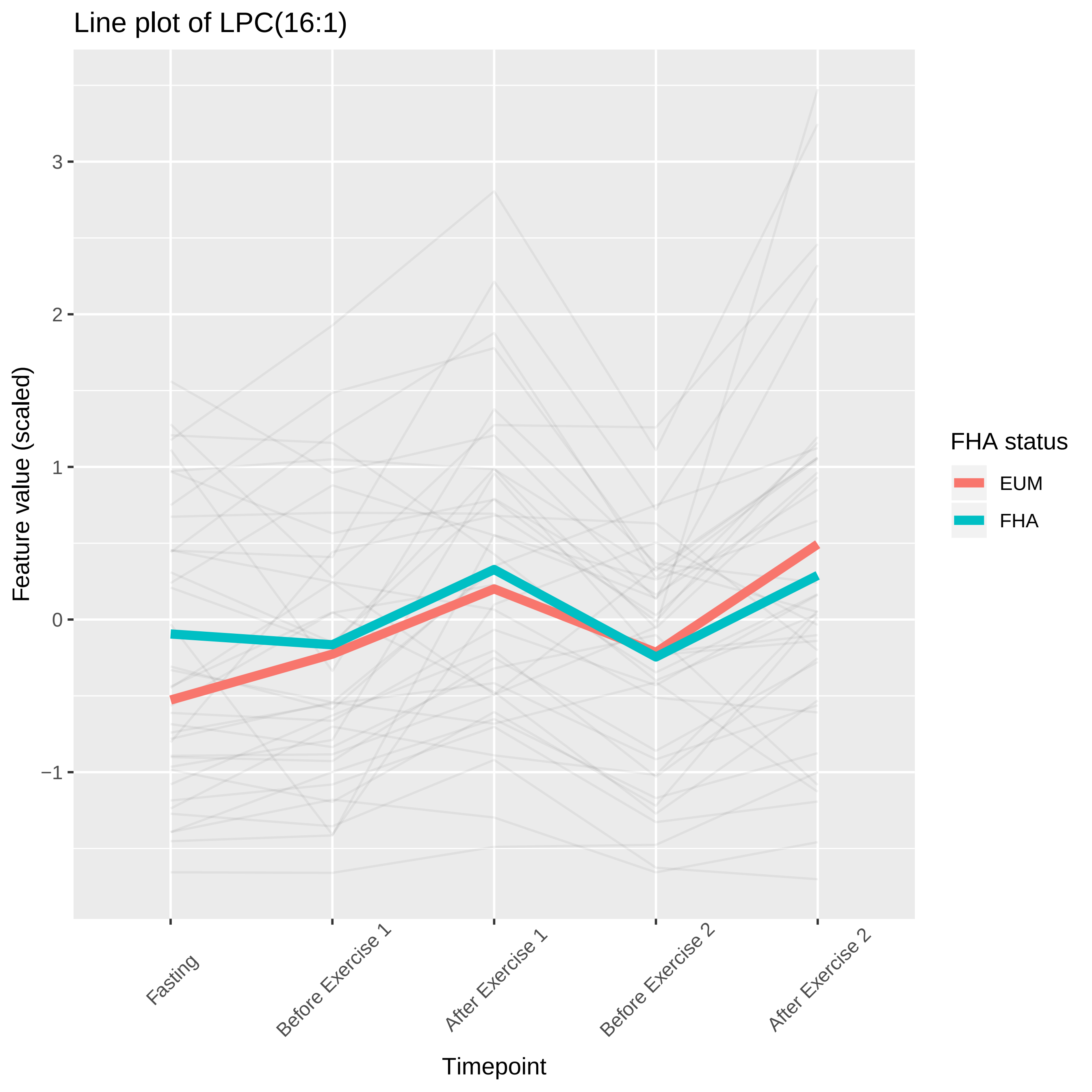

Line plot of LPC(16:1)
Feature value (scaled)
3
2
1
0
−1
Fasting
Before Exercise 1
After Exercise 1
Before Exercise 2
After Exercise 2
Timepoint
FHA status
EUM
FHA

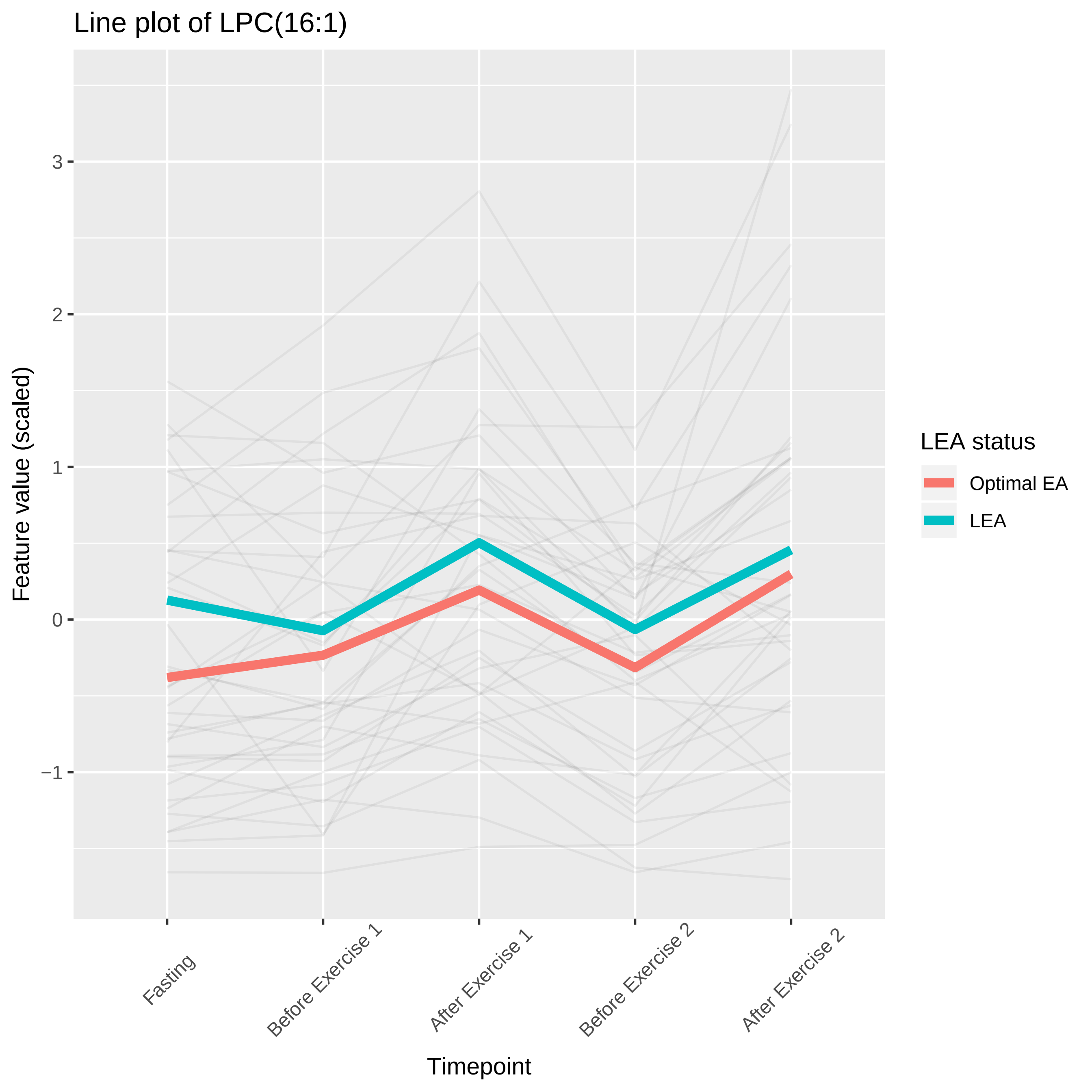
Line plot of LPC(16:1)
Feature value (scaled)
3
2
1
0
−1
Fasting
Before Exercise 1
After Exercise 1
Before Exercise 2
After Exercise 2
Timepoint
LEA status
Optimal EA
LEA

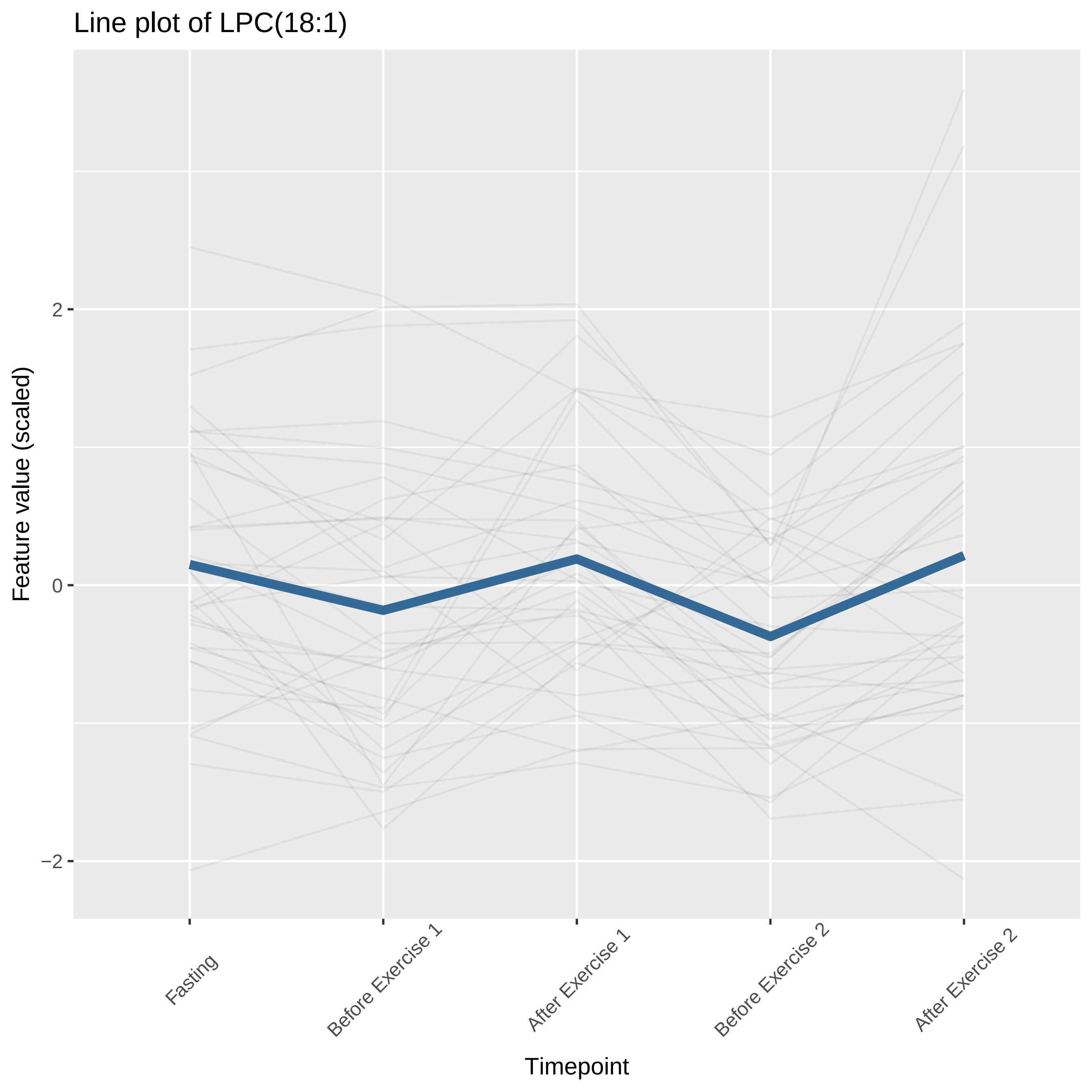

Line plot of LPC(18:1)
Feature value (scaled)
2
0
−2
Fasting
Before Exercise 1
After Exercise 1
Before Exercise 2
After Exercise 2
Timepoint

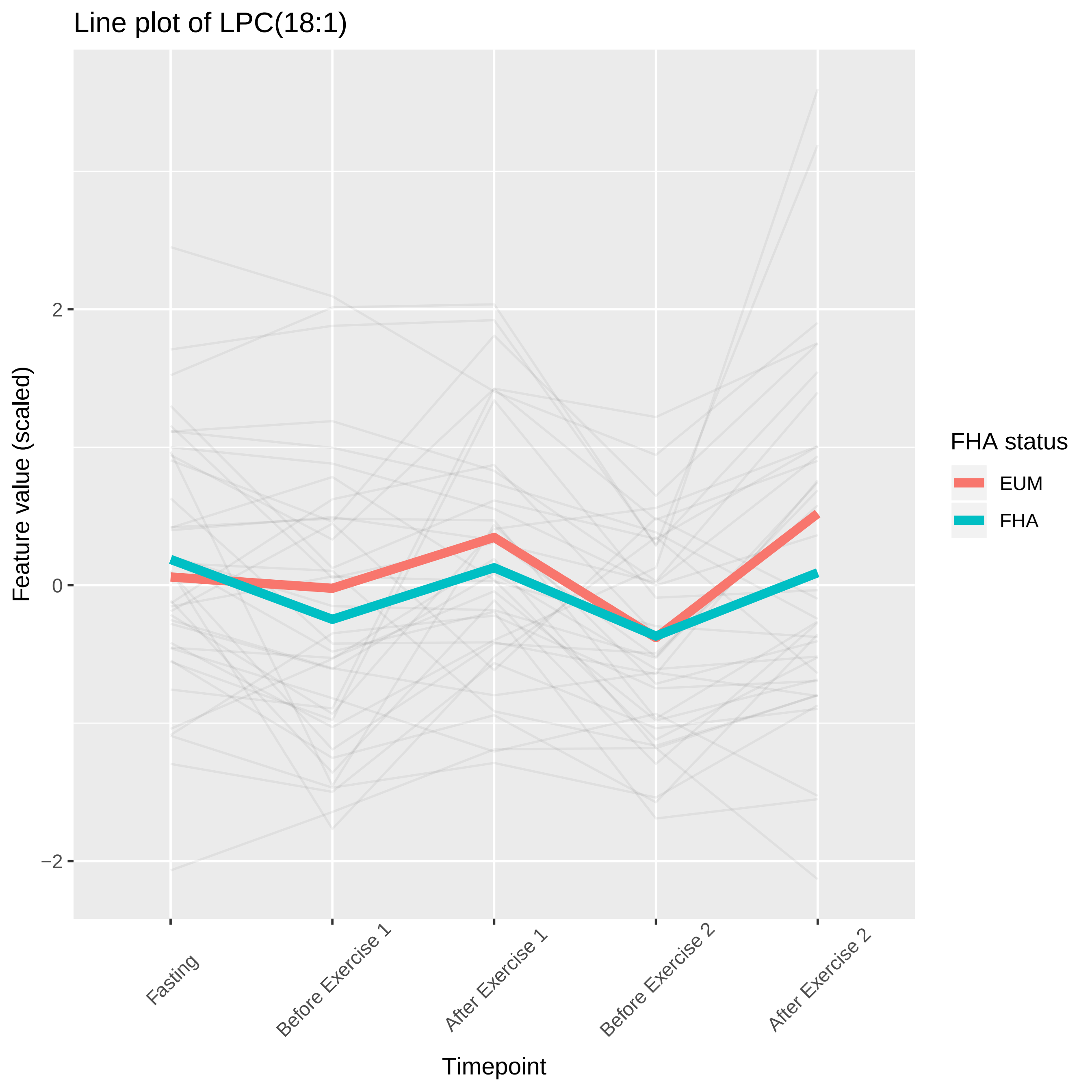
Line plot of LPC(18:1)
Feature value (scaled)
2
0
−2
Fasting
Before Exercise 1
After Exercise 1
Before Exercise 2
After Exercise 2
Timepoint
FHA status
EUM
FHA

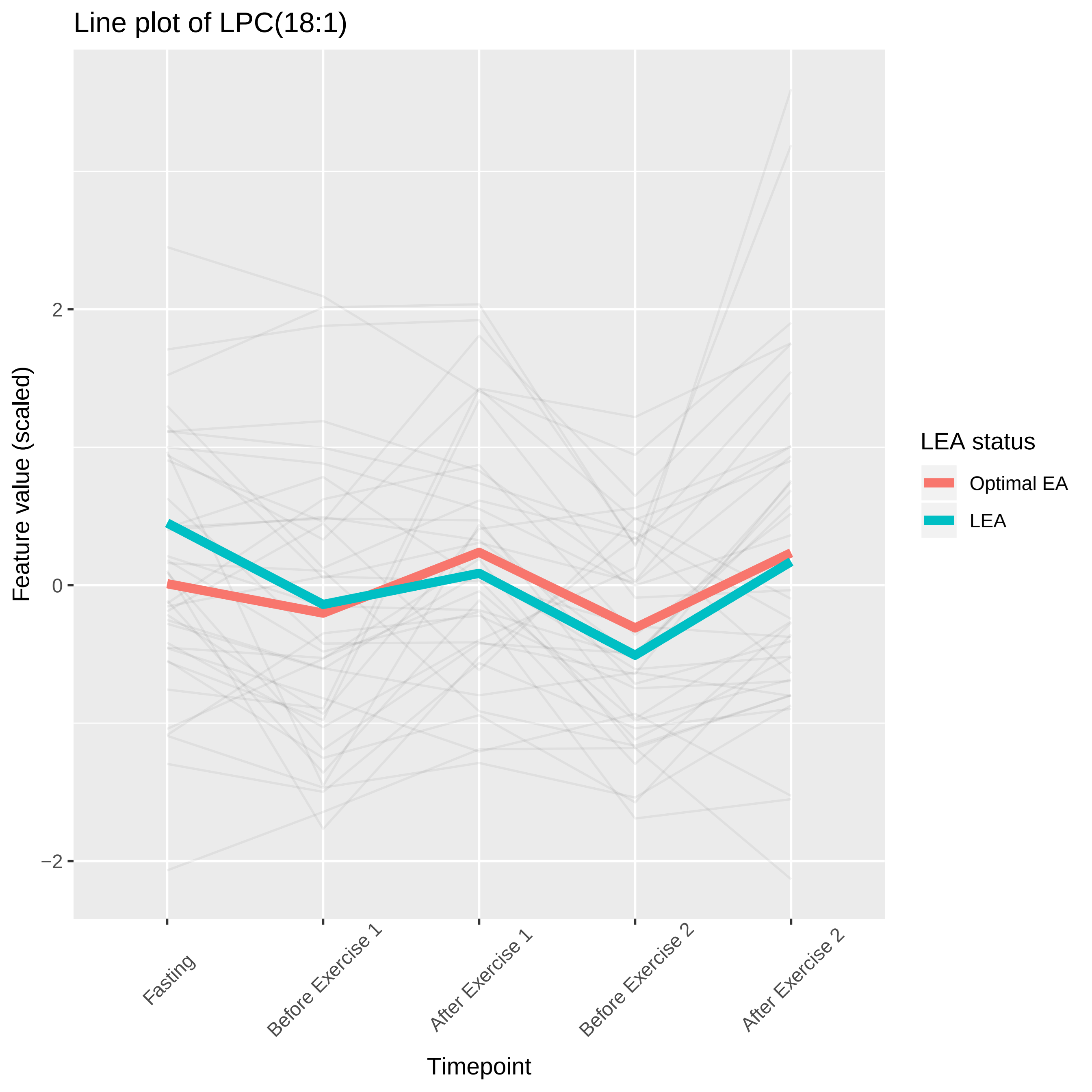

Line plot of LPC(18:1)
Feature value (scaled)
2
0
−2
Fasting
Before Exercise 1
After Exercise 1
Before Exercise 2
After Exercise 2
Timepoint
LEA status
Optimal EA
LEA

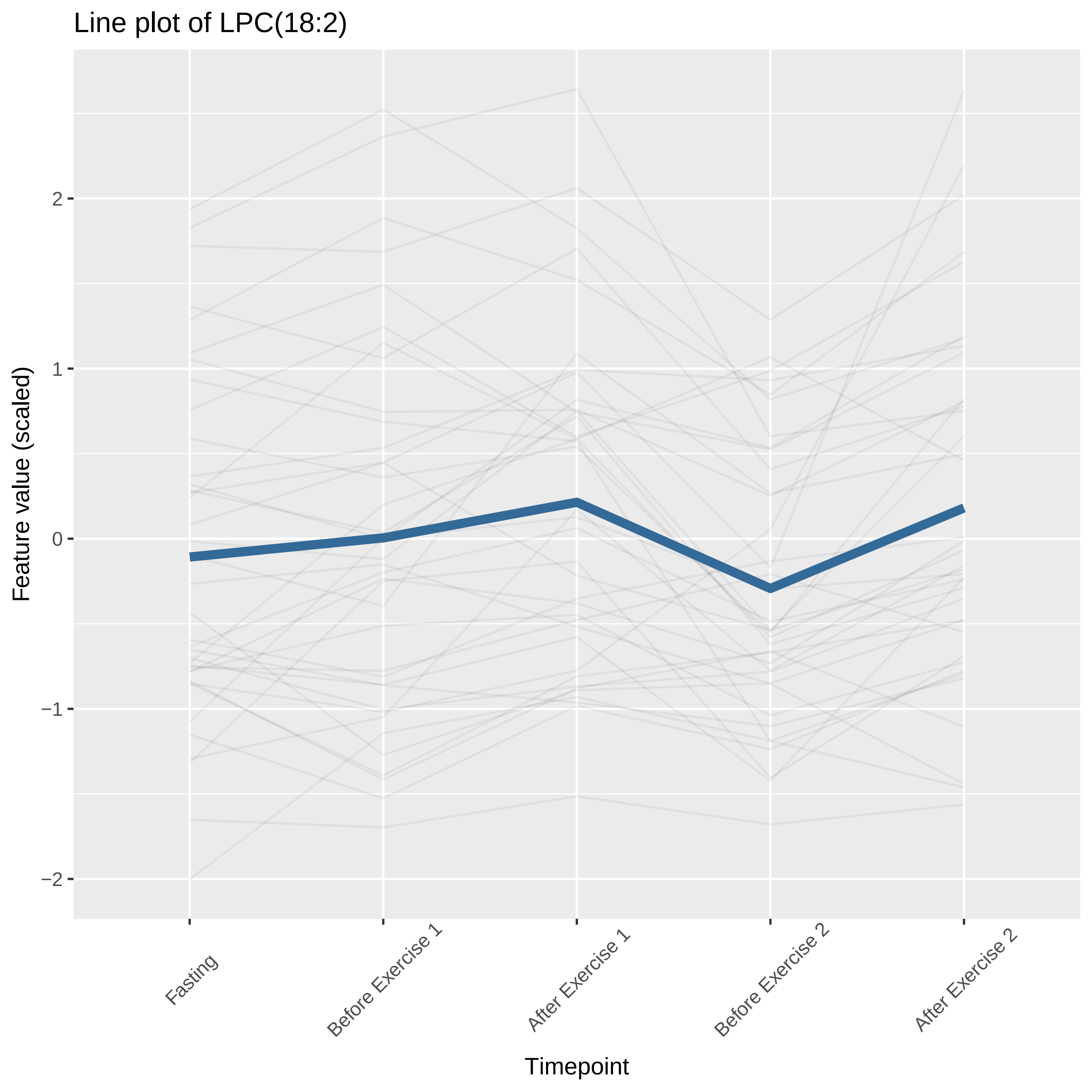
Line plot of LPC(18:2)
Feature value (scaled)
2
1
0
−1
−2
Fasting
Before Exercise 1
After Exercise 1
Before Exercise 2
After Exercise 2
Timepoint

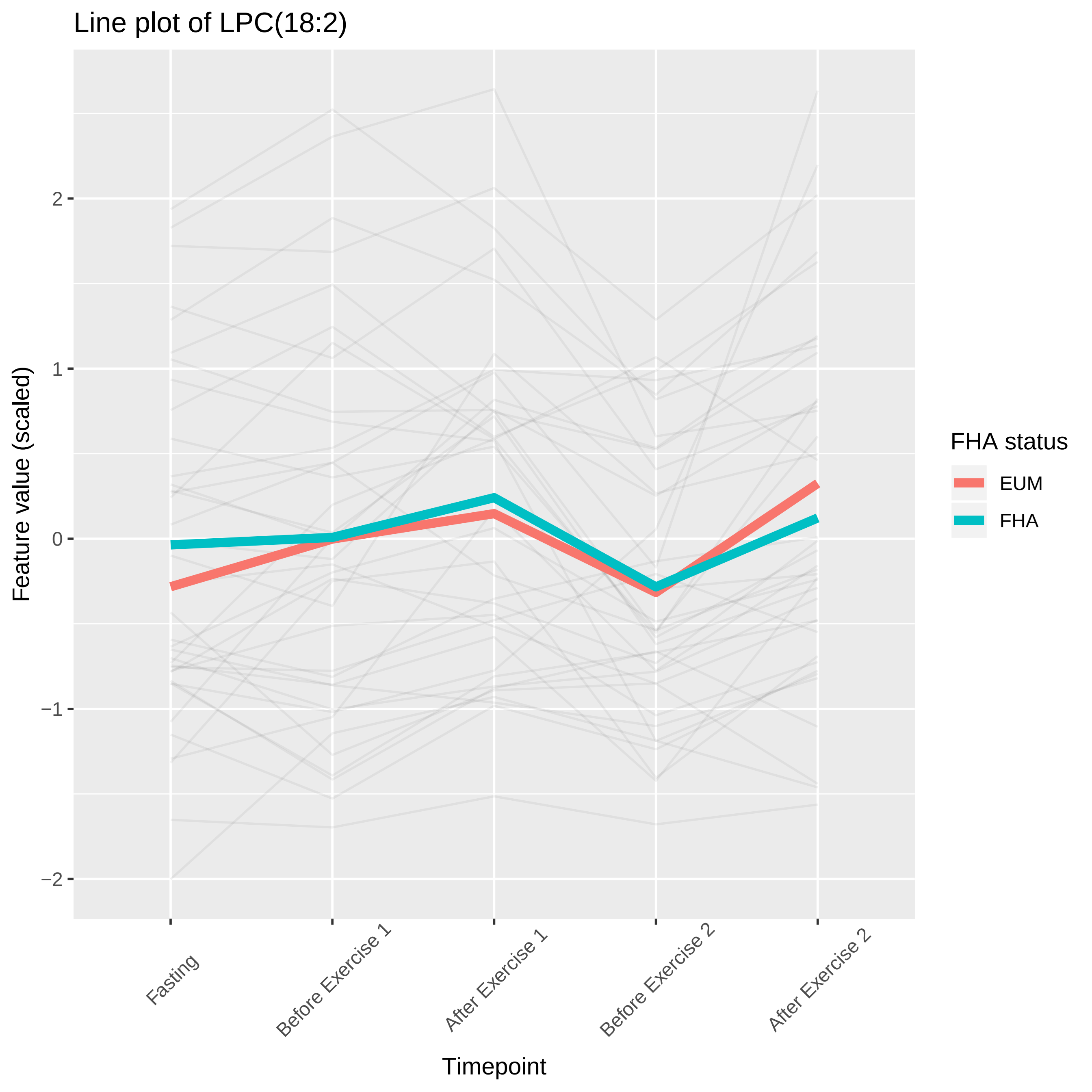

Line plot of LPC(18:2)
Feature value (scaled)
2
1
0
−1
−2
Fasting
Before Exercise 1
After Exercise 1
Before Exercise 2
After Exercise 2
Timepoint
FHA status
EUM
FHA

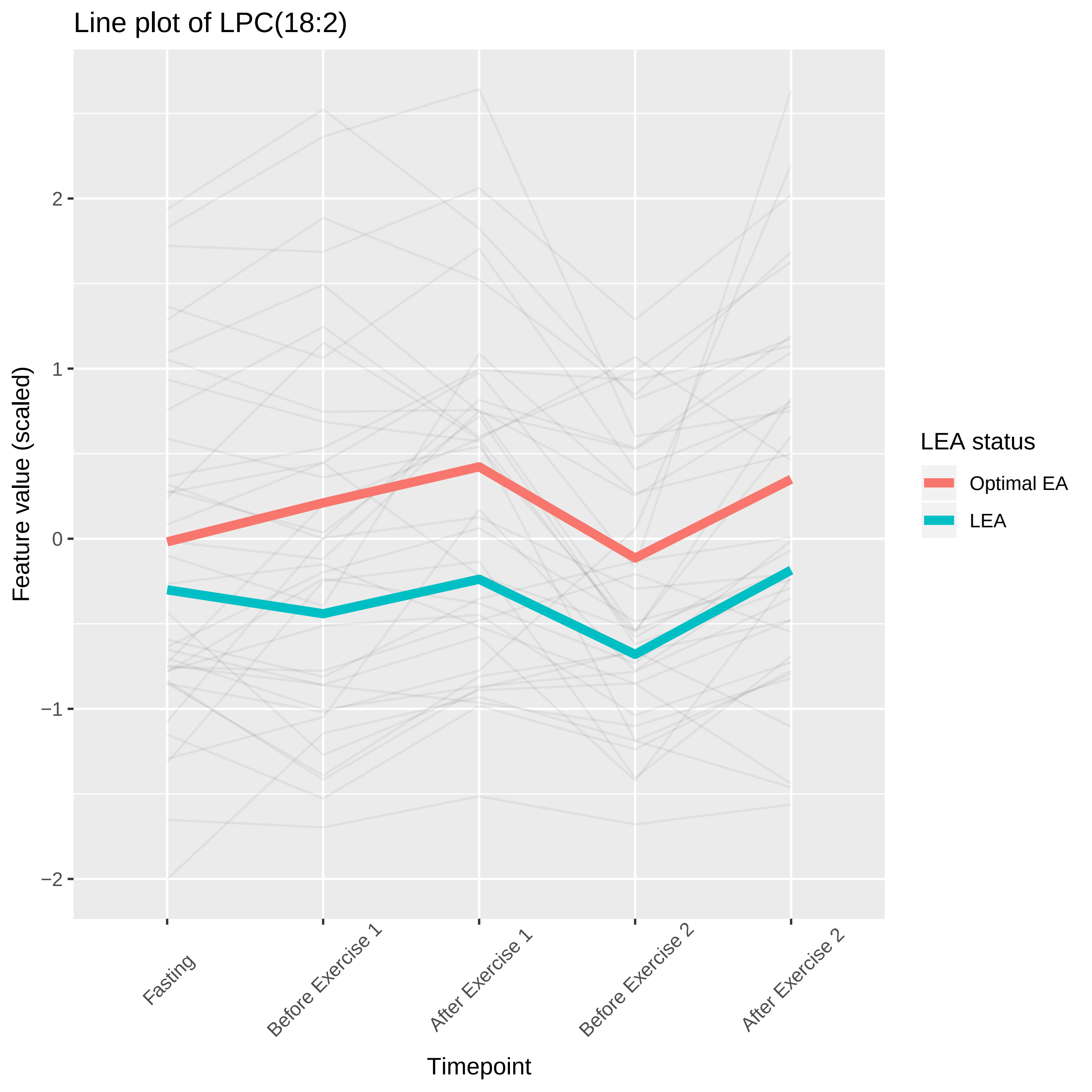

Line plot of LPC(18:2)
Feature value (scaled)
2
1
0
−1
−2
Fasting
Before Exercise 1
After Exercise 1
Before Exercise 2
After Exercise 2
Timepoint
LEA status
Optimal EA
LEA

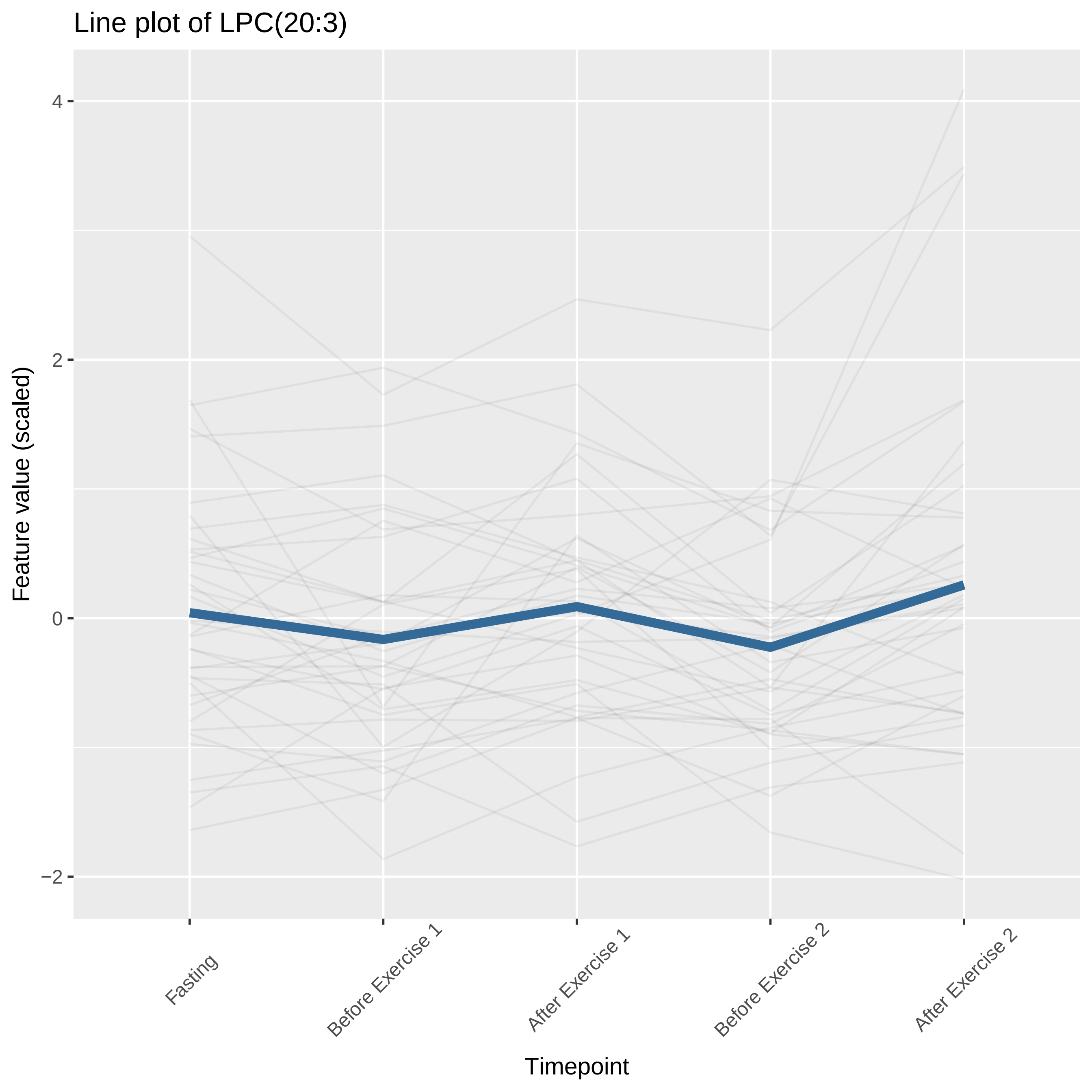
Line plot of LPC(20:3)
Feature value (scaled)
4
2
0
−2
Fasting
Before Exercise 1
After Exercise 1
Before Exercise 2
After Exercise 2
Timepoint

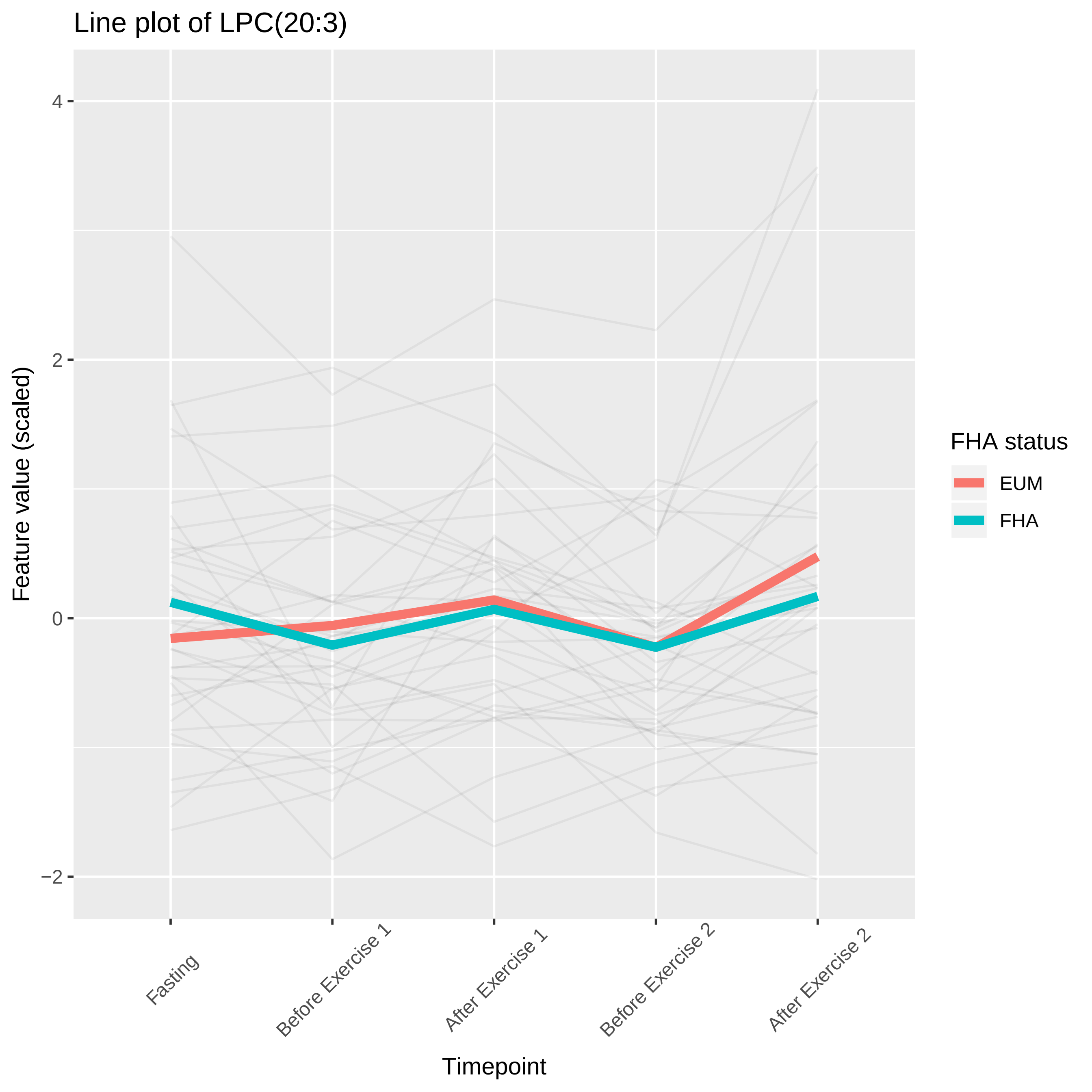

Line plot of LPC(20:3)
Feature value (scaled)
4
2
0
−2
Fasting
Before Exercise 1
After Exercise 1
Before Exercise 2
After Exercise 2
Timepoint
FHA status
EUM
FHA

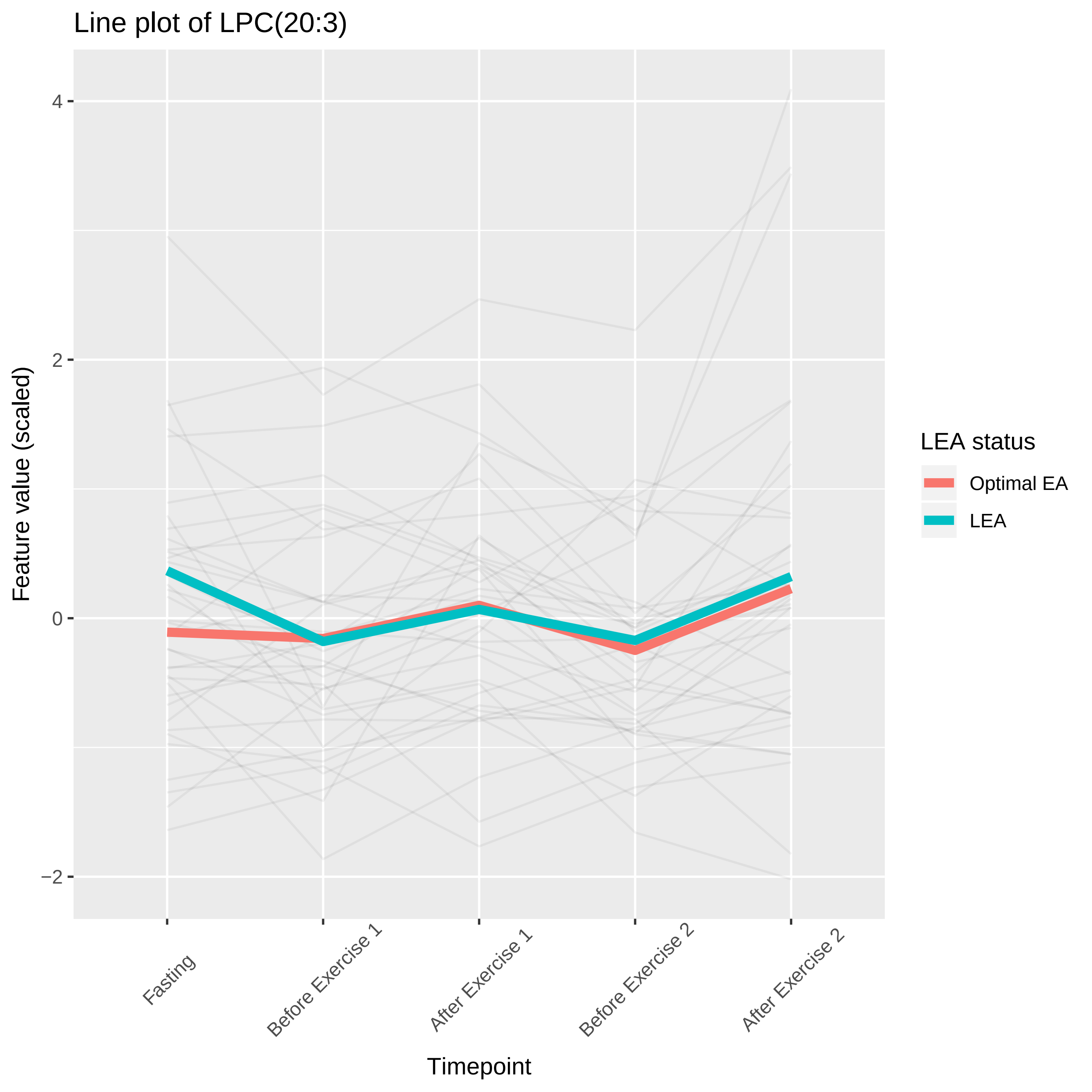

Line plot of LPC(20:3)
Feature value (scaled)
4
2
0
−2
Fasting
Before Exercise 1
After Exercise 1
Before Exercise 2
After Exercise 2
Timepoint
LEA status
Optimal EA
LEA

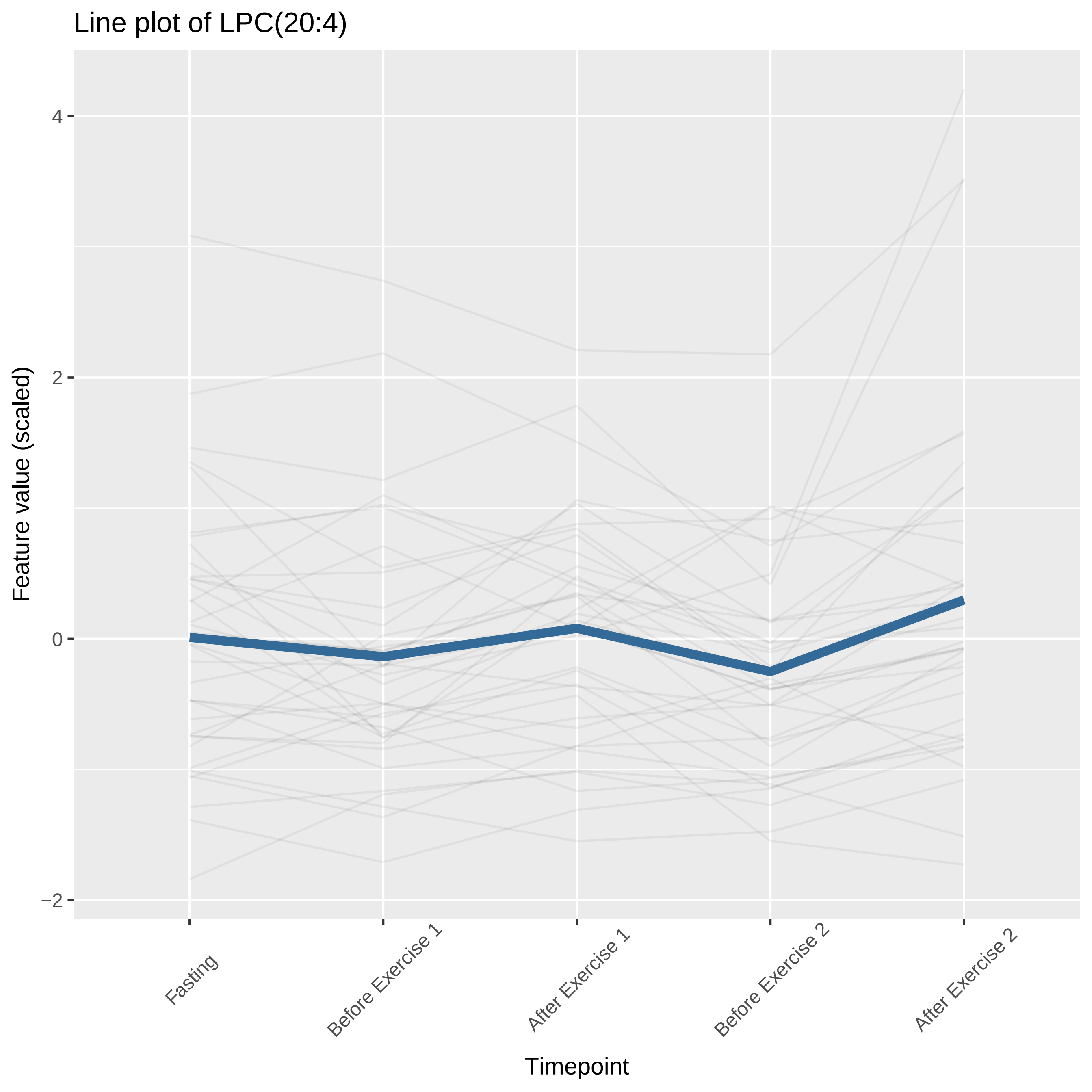
Line plot of LPC(20:4)
Feature value (scaled)
4
2
0
−2
Fasting
Before Exercise 1
After Exercise 1
Before Exercise 2
After Exercise 2
Timepoint

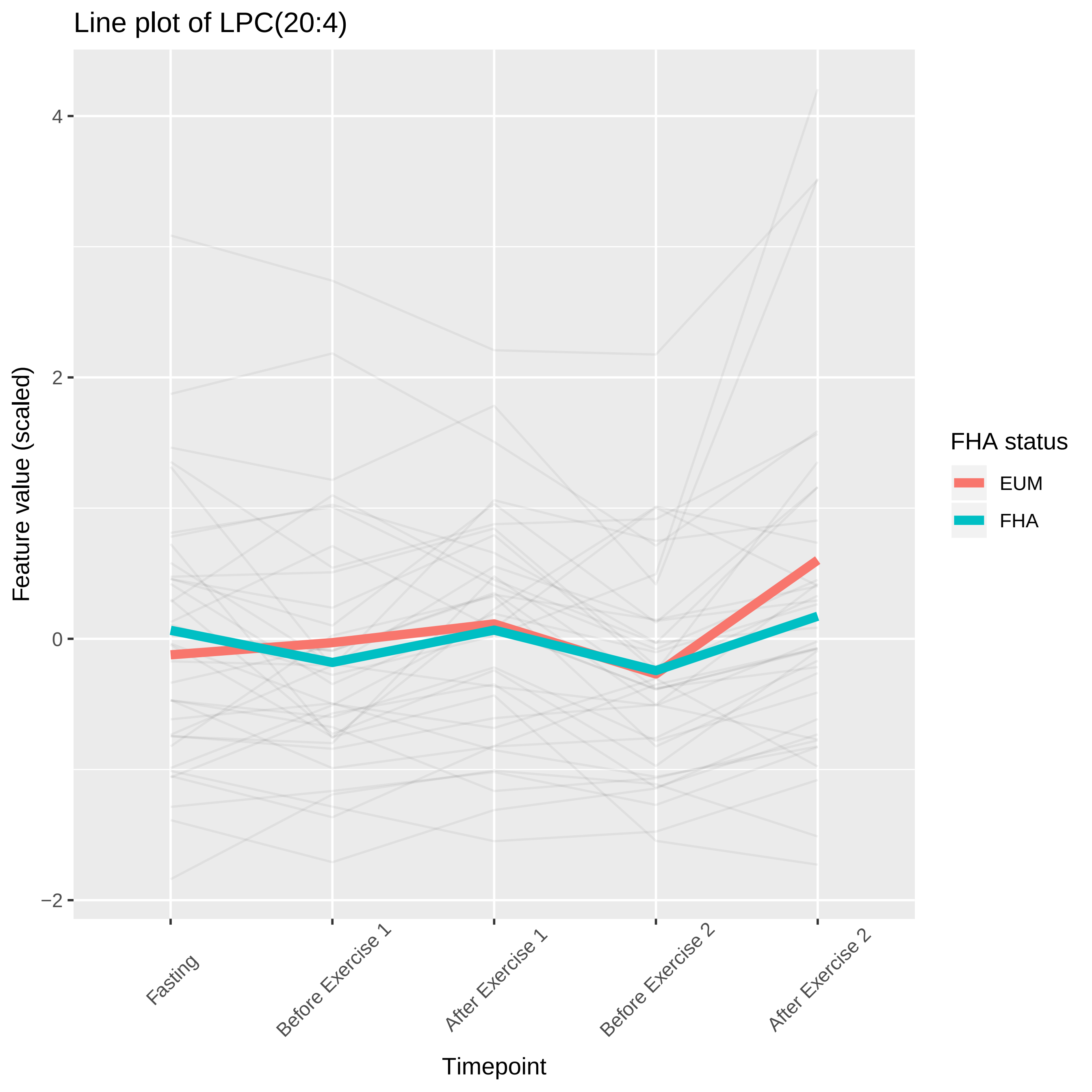

Line plot of LPC(20:4)
4
2
0
−2
Feature value (scaled)
Fasting
Before Exercise 1
After Exercise 1
Before Exercise 2
After Exercise 2
Timepoint
FHA status
EUM
FHA

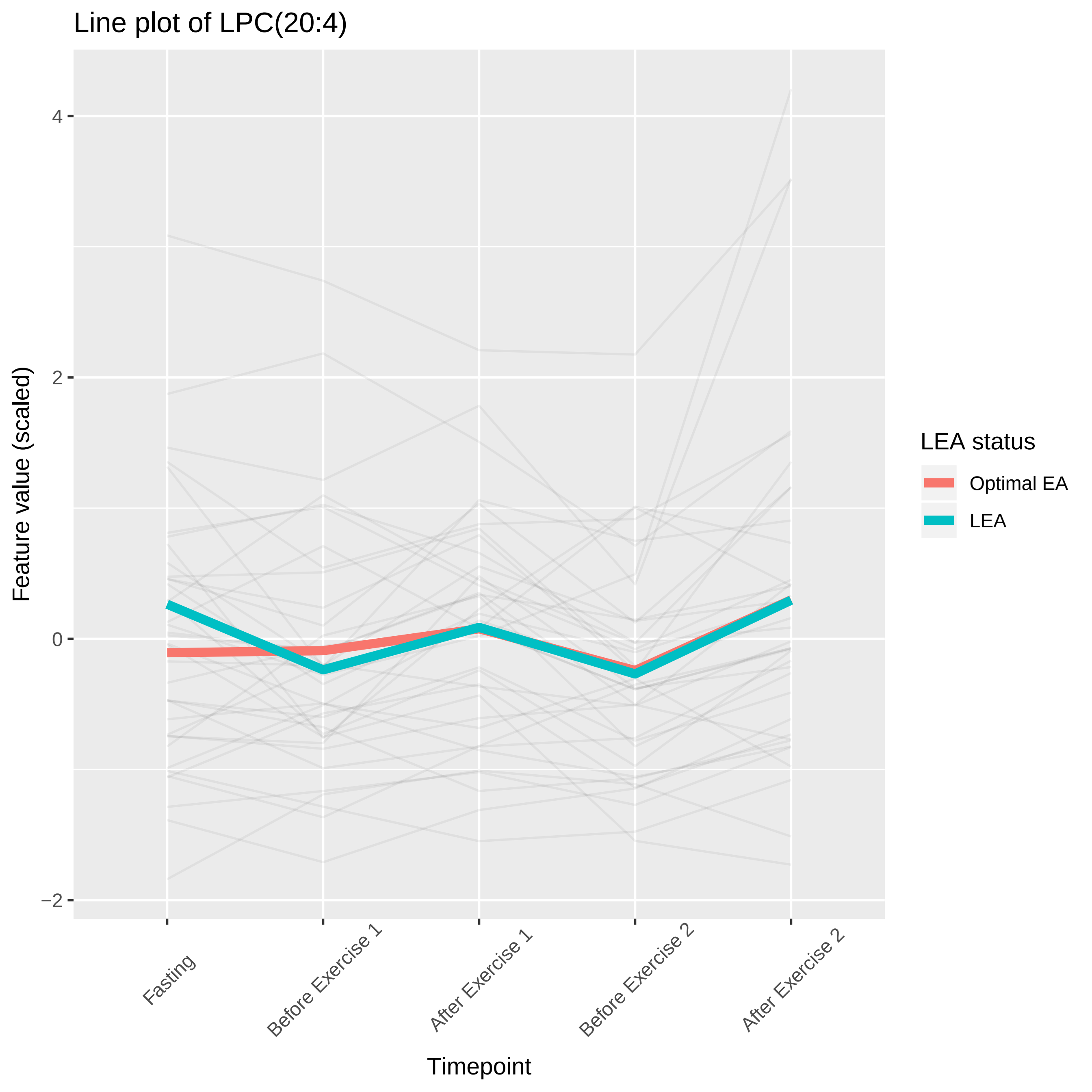

Line plot of LPC(20:4)
Feature value (scaled)
4
2
0
−2
Fasting
Before Exercise 1
After Exercise 1
Before Exercise 2
After Exercise 2
Timepoint
LEA status
Optimal EA
LEA

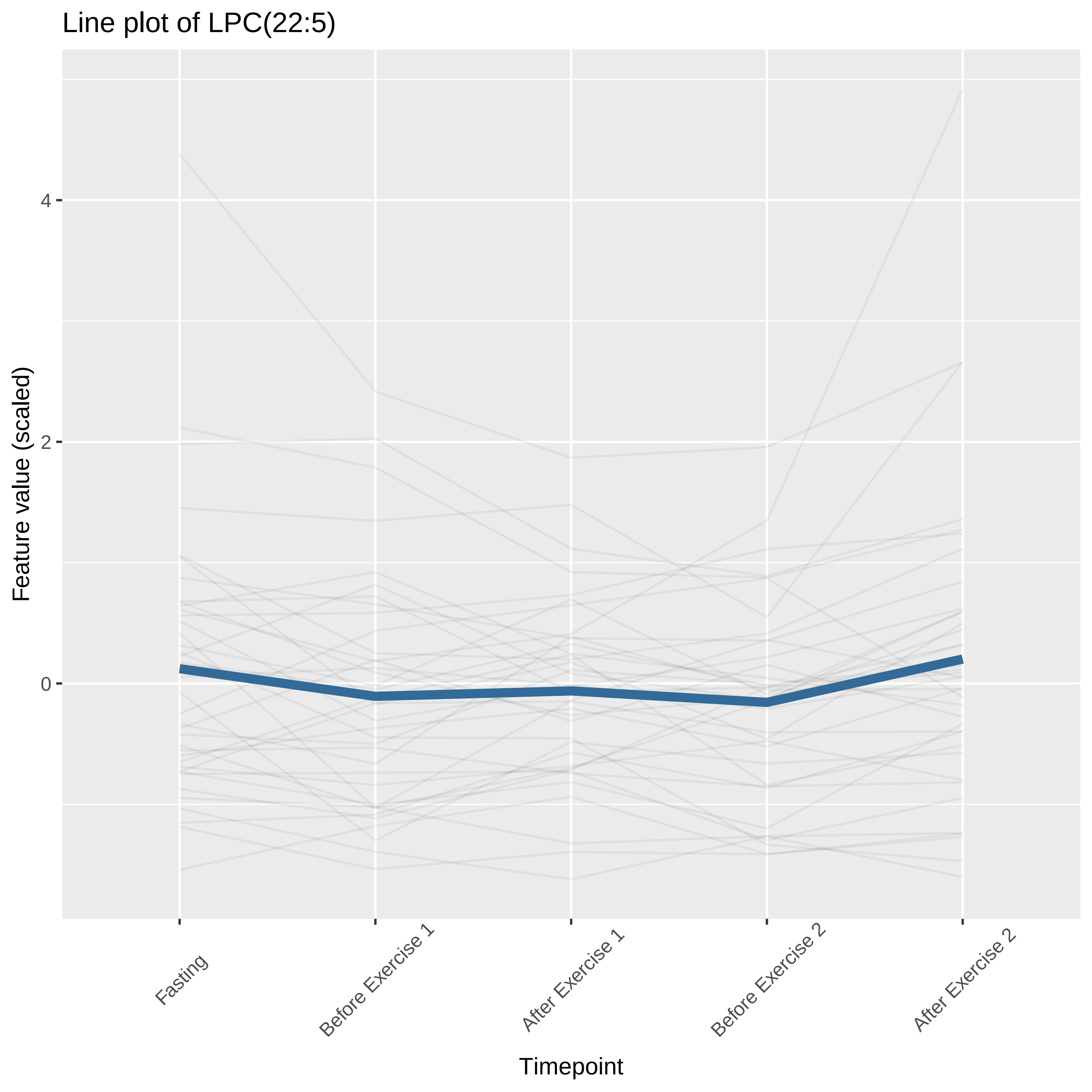
Line plot of LPC(22:5)
Feature value (scaled)
4
2
0
Fasting
Before Exercise 1
After Exercise 1
Before Exercise 2
After Exercise 2
Timepoint

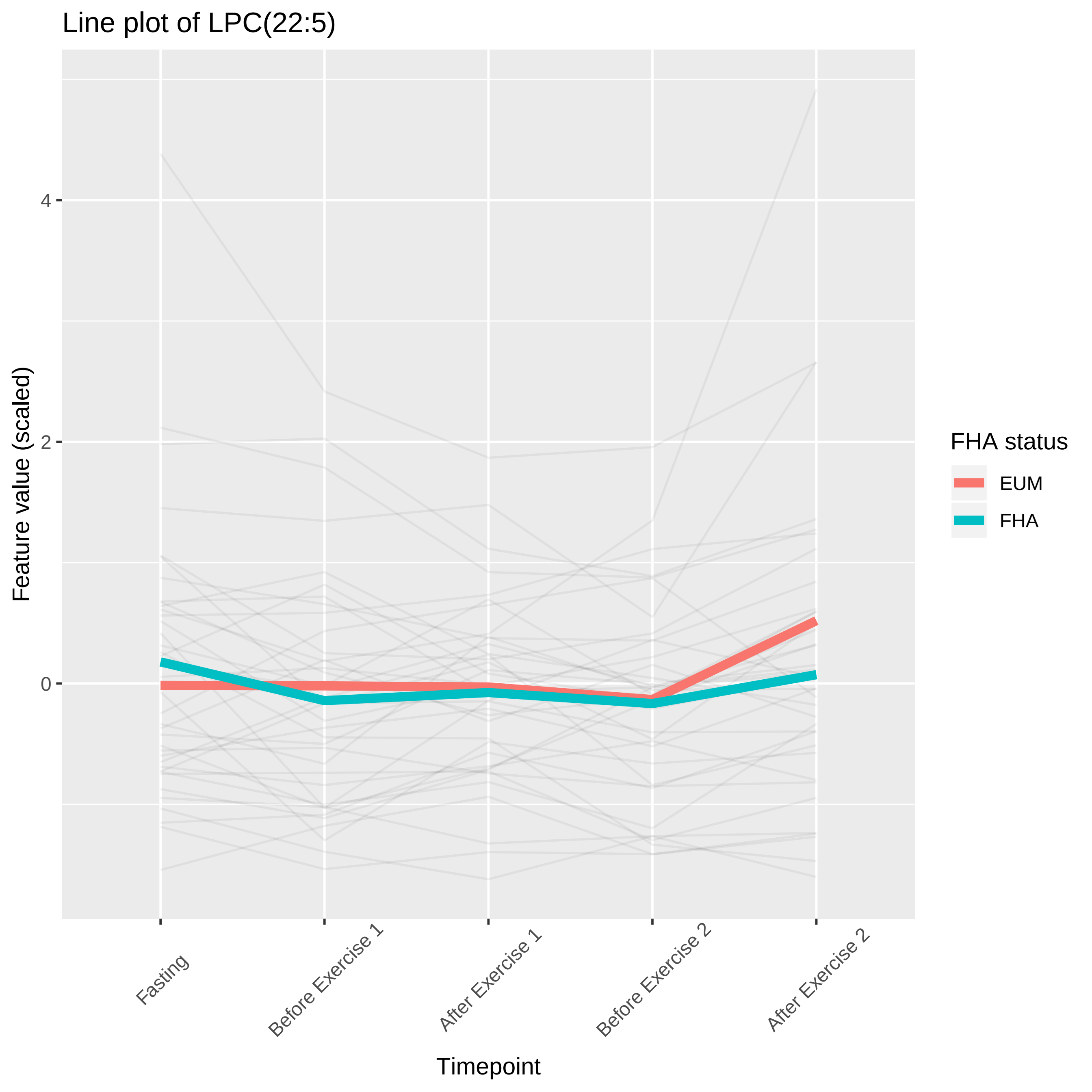

Line plot of LPC(22:5)
Feature value (scaled)
4
2
0
Fasting
Before Exercise 1
After Exercise 1
Before Exercise 2
After Exercise 2
Timepoint
FHA status
EUM
FHA

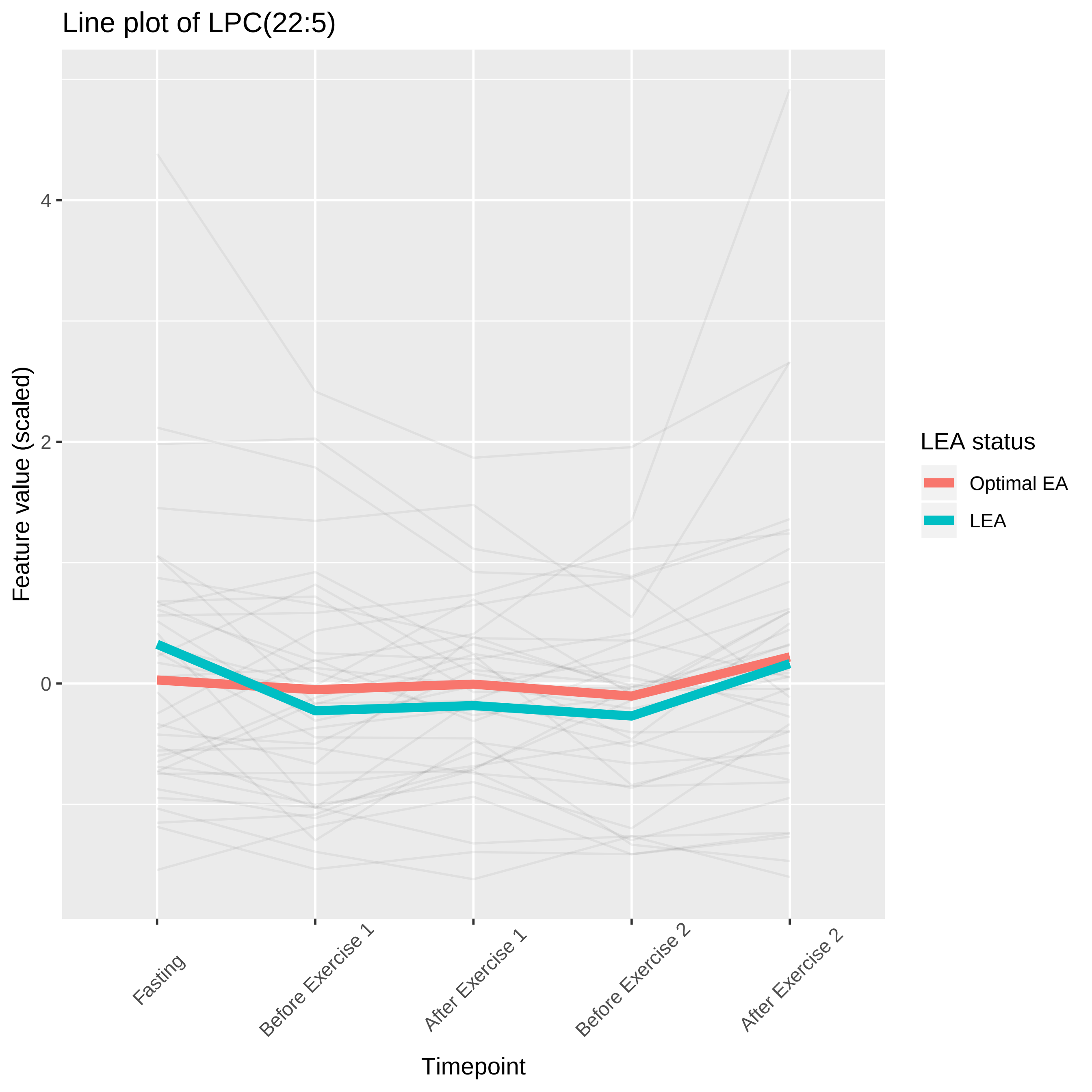

Line plot of LPC(22:5)
Feature value (scaled)
4
2
0
Fasting
Before Exercise 1
After Exercise 1
Before Exercise 2
After Exercise 2
Timepoint
LEA status
Optimal EA
LEA

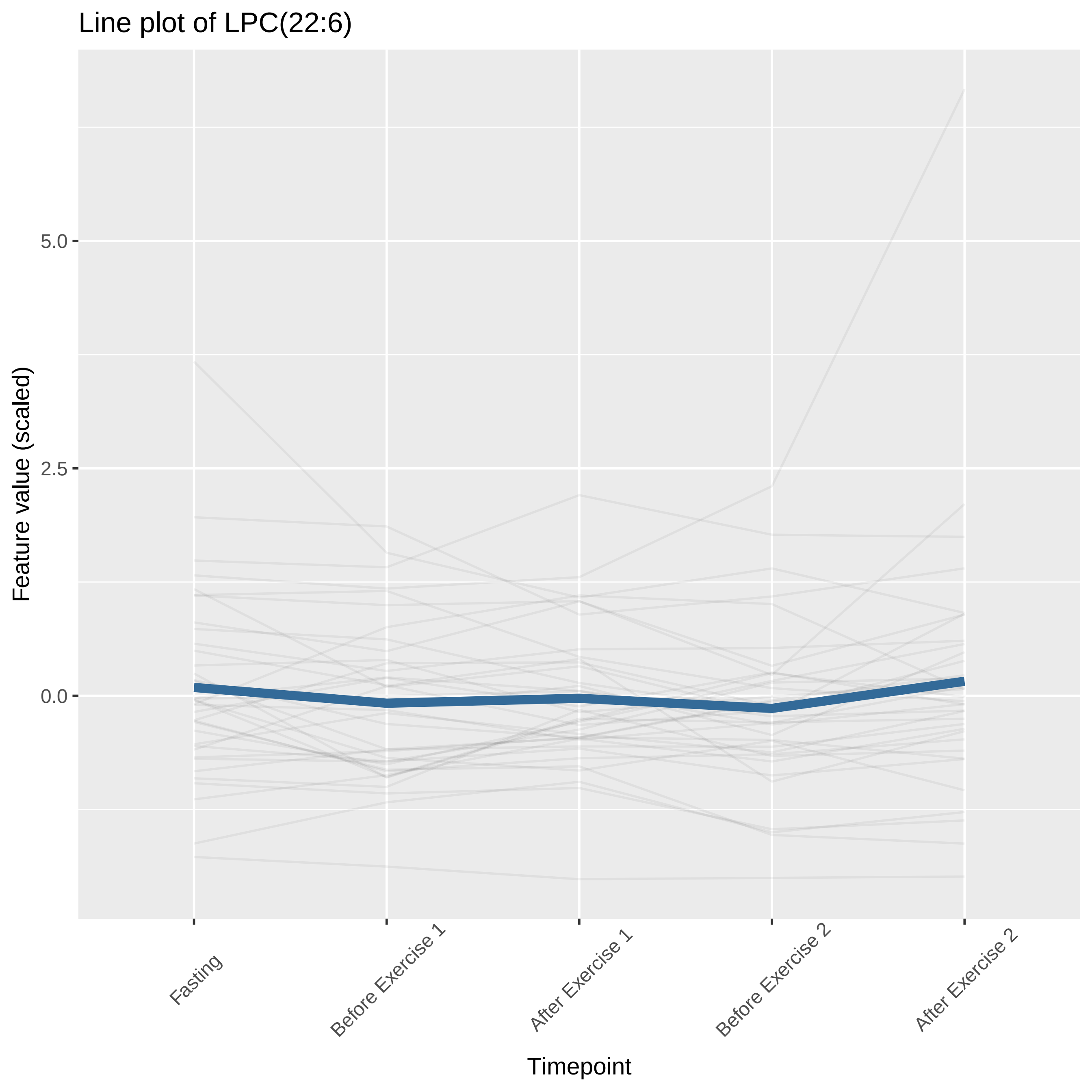

Line plot of LPC(22:6)
Feature value (scaled)
5.0
2.5
0.0
Fasting
Before Exercise 1
After Exercise 1
Before Exercise 2
After Exercise 2
Timepoint

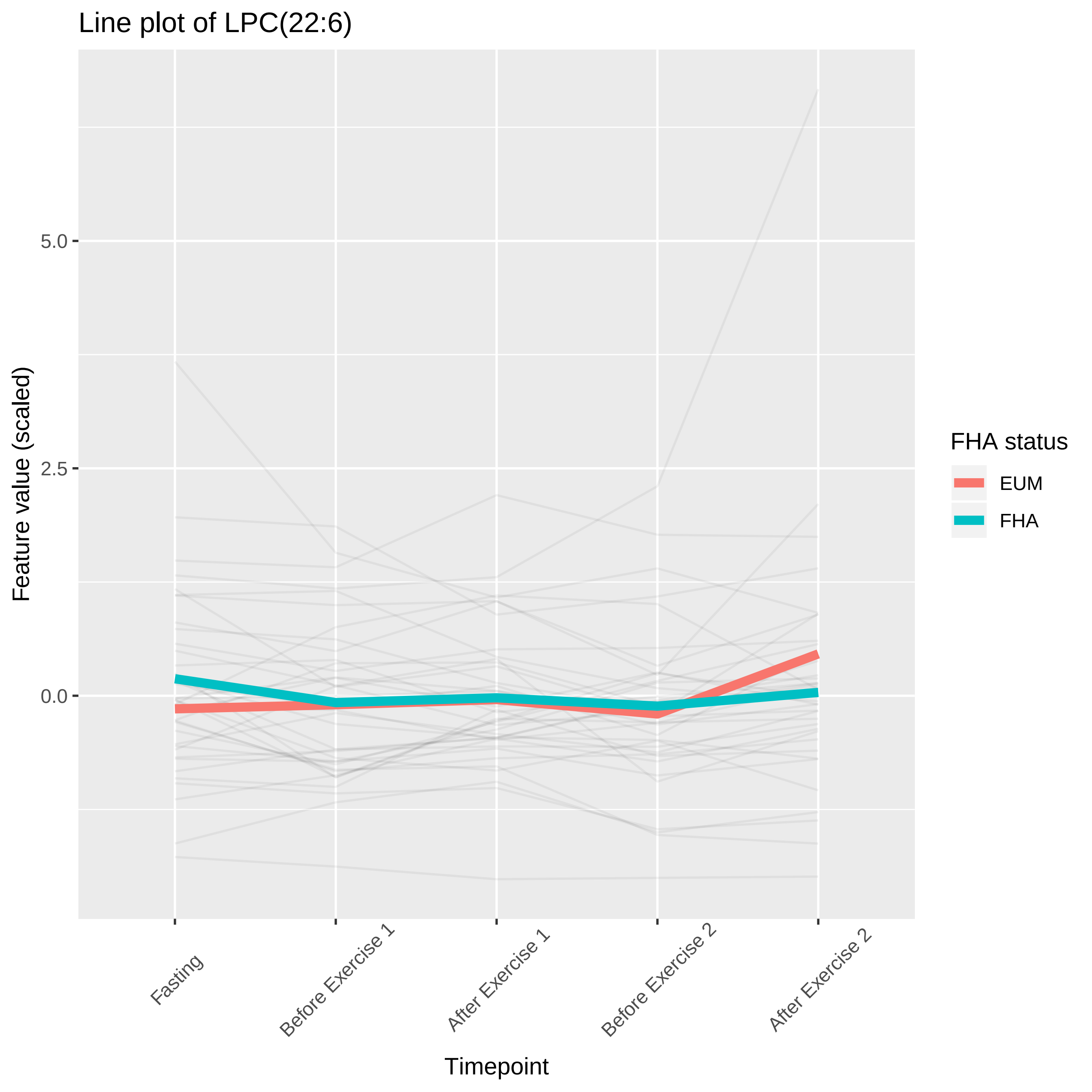

Line plot of LPC(22:6)
Feature value (scaled)
5.0
2.5
0.0
Fasting
Before Exercise 1
After Exercise 1
Before Exercise 2
After Exercise 2
Timepoint
FHA status
EUM
FHA

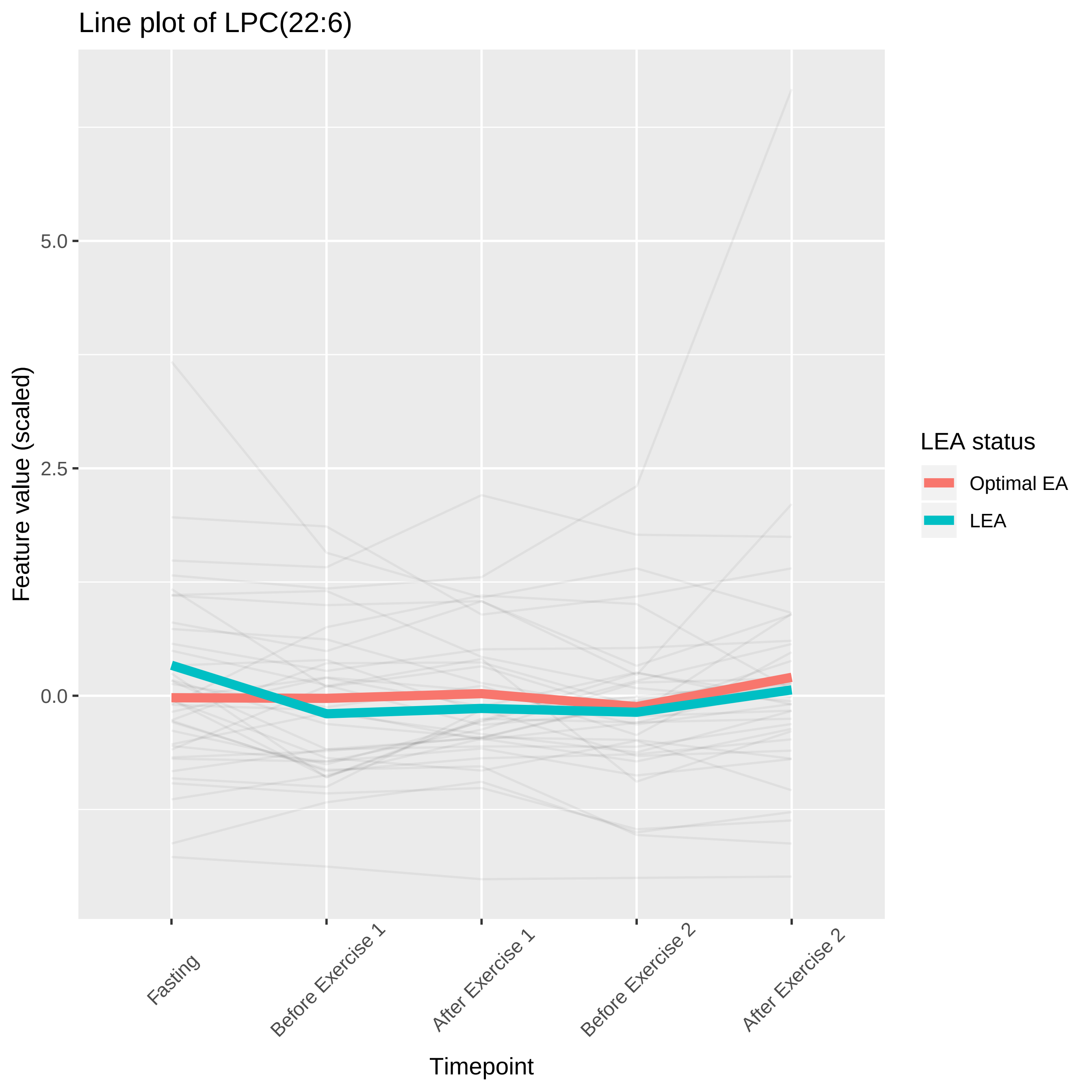

Line plot of LPC(22:6)
Feature value (scaled)
5.0
2.5
0.0
Fasting
Before Exercise 1
After Exercise 1
Before Exercise 2
After Exercise 2
Timepoint
LEA status
Optimal EA
LEA

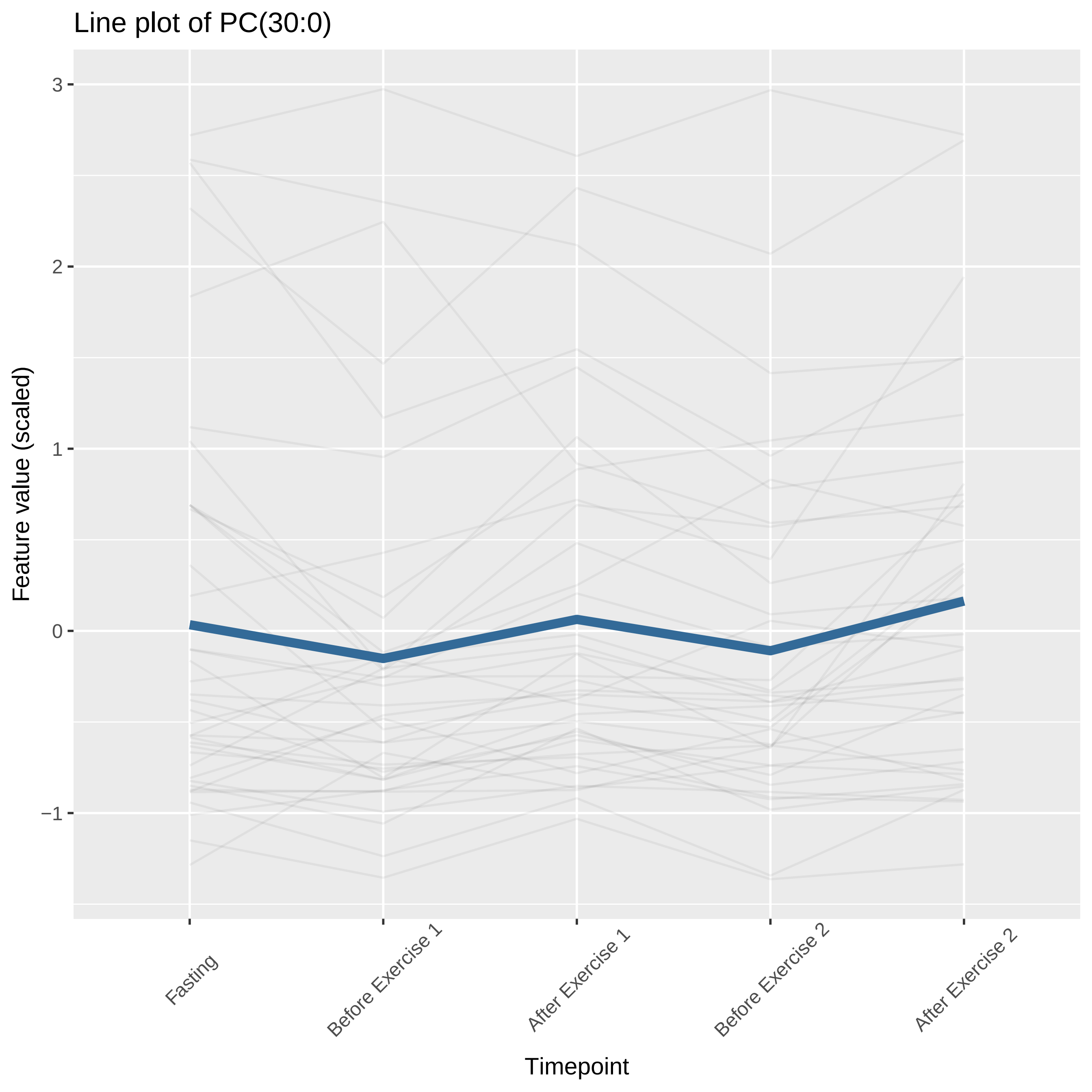

Line plot of PC(30:0)
Feature value (scaled)
3
2
1
0
−1
Fasting
Before Exercise 1
After Exercise 1
Before Exercise 2
After Exercise 2
Timepoint

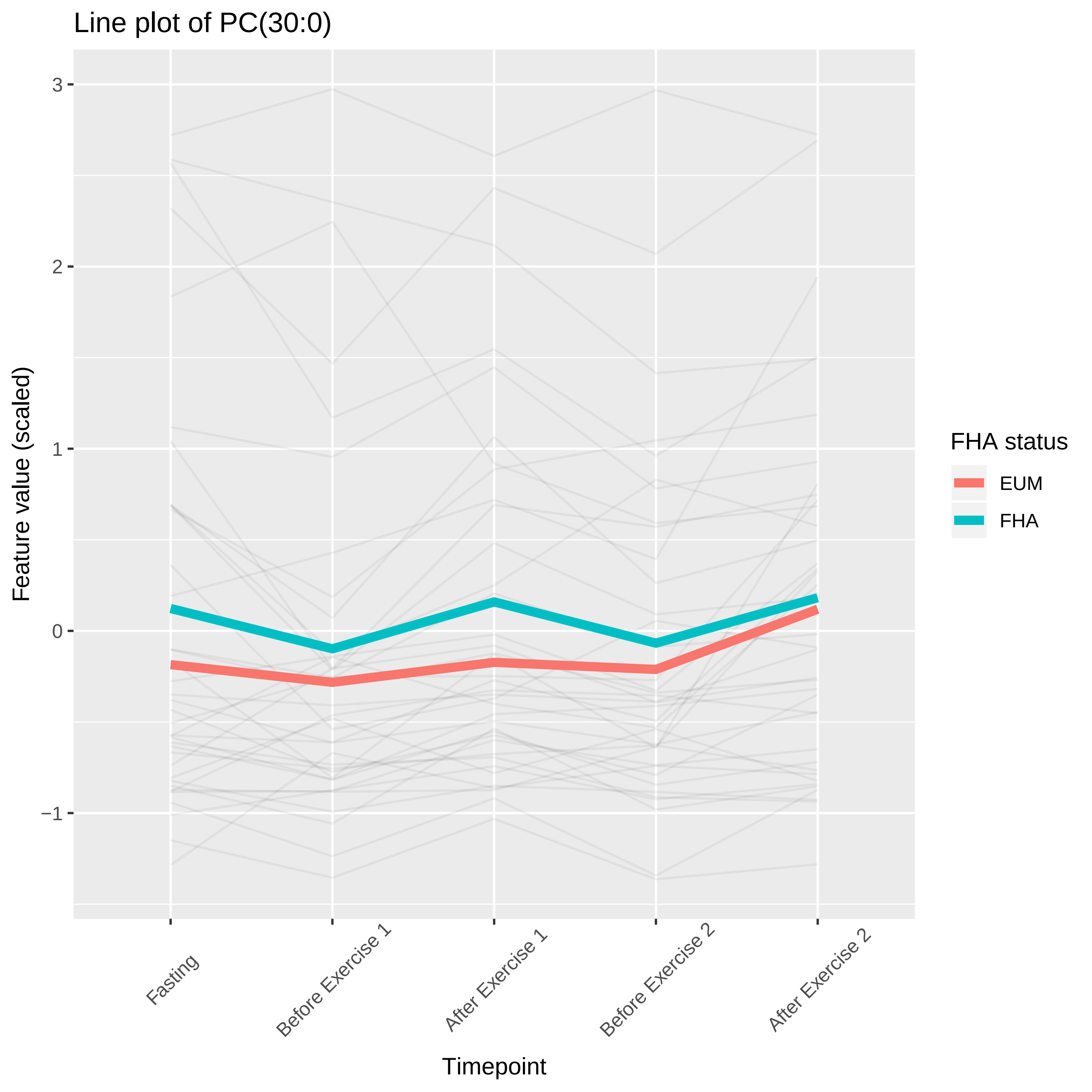

Line plot of PC(30:0)
Feature value (scaled)
3
2
1
0
−1
Fasting
Before Exercise 1
After Exercise 1
Before Exercise 2
After Exercise 2
Timepoint
FHA status
EUM
FHA

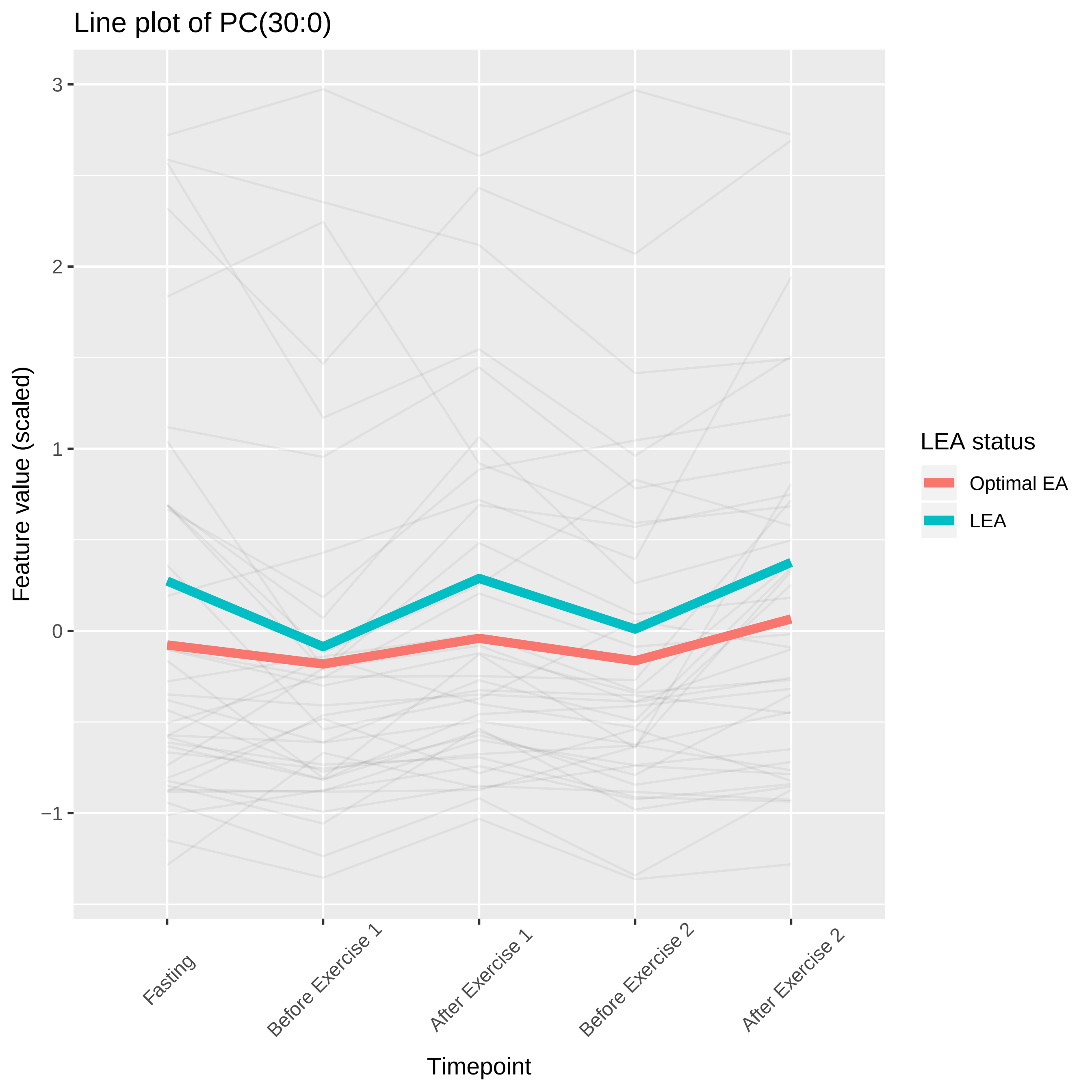

Line plot of PC(30:0)
Feature value (scaled)
3
2
1
0
−1
Fasting
Before Exercise 1
After Exercise 1
Before Exercise 2
After Exercise 2
Timepoint
LEA status
Optimal EA
LEA

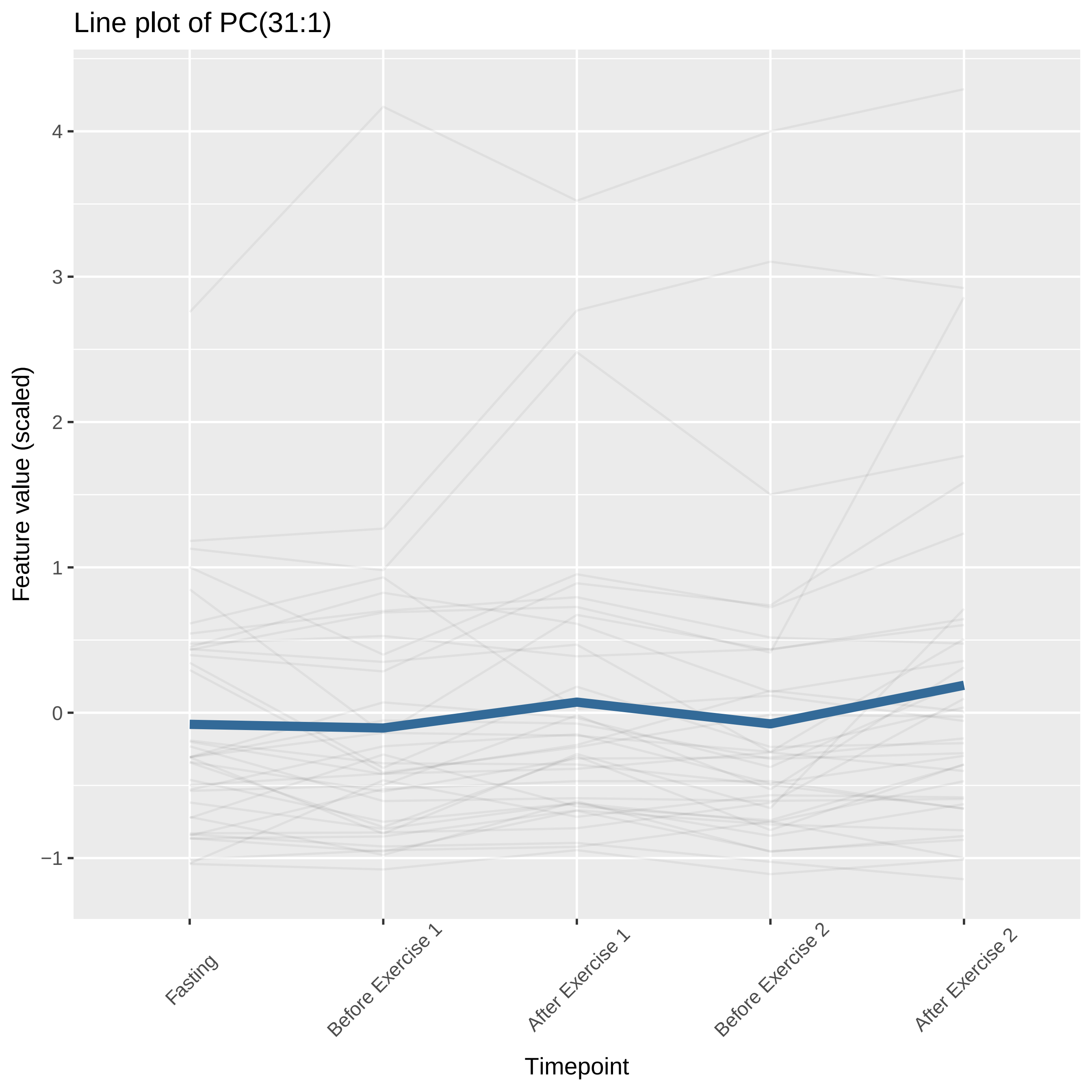
Line plot of PC(31:1)
Feature value (scaled)
4
3
2
1
0
−1
Fasting
Before Exercise 1
After Exercise 1
Before Exercise 2
After Exercise 2
Timepoint

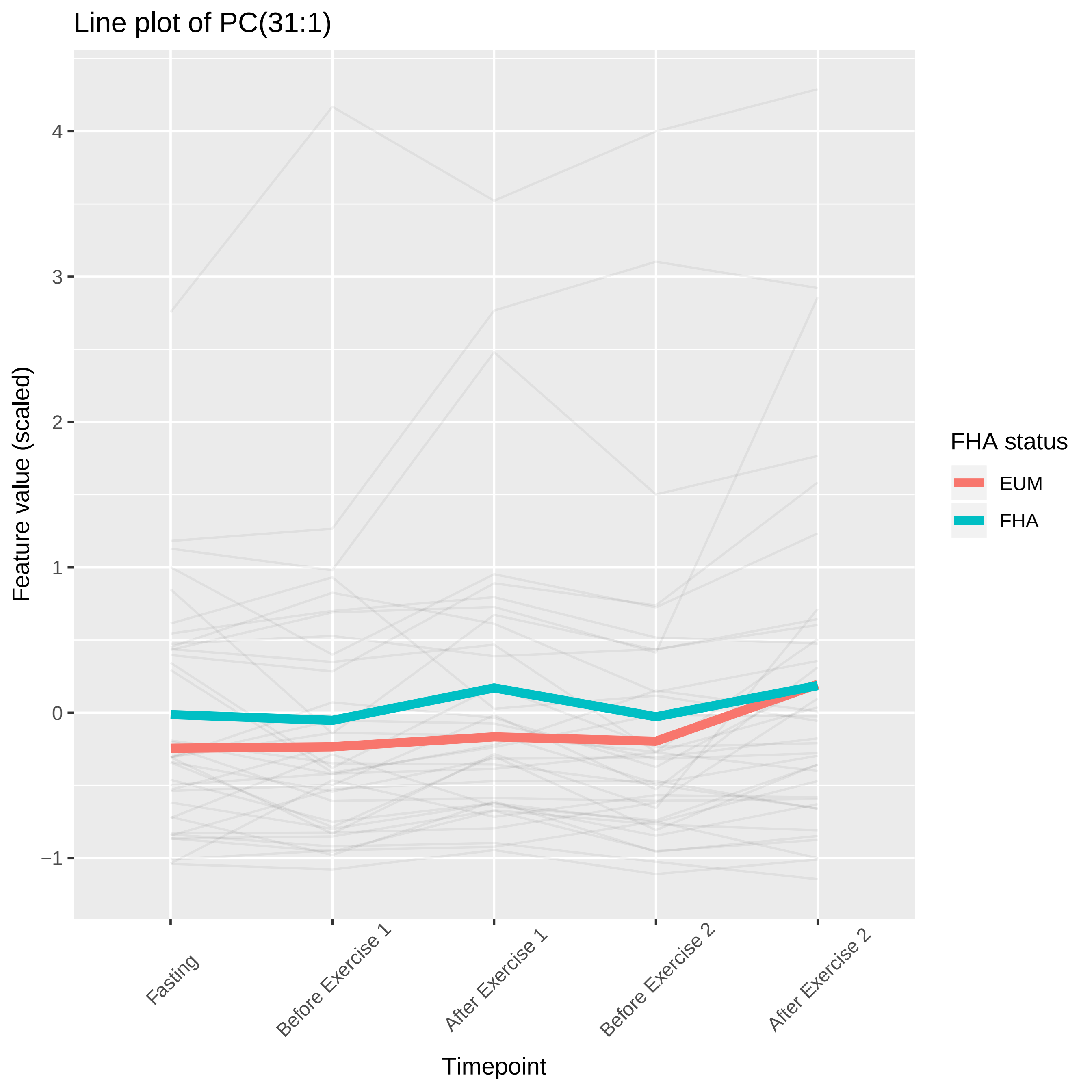

Line plot of PC(31:1)
Feature value (scaled)
4
3
2
1
0
−1
Fasting
Before Exercise 1
After Exercise 1
Before Exercise 2
After Exercise 2
Timepoint
FHA status
EUM
FHA

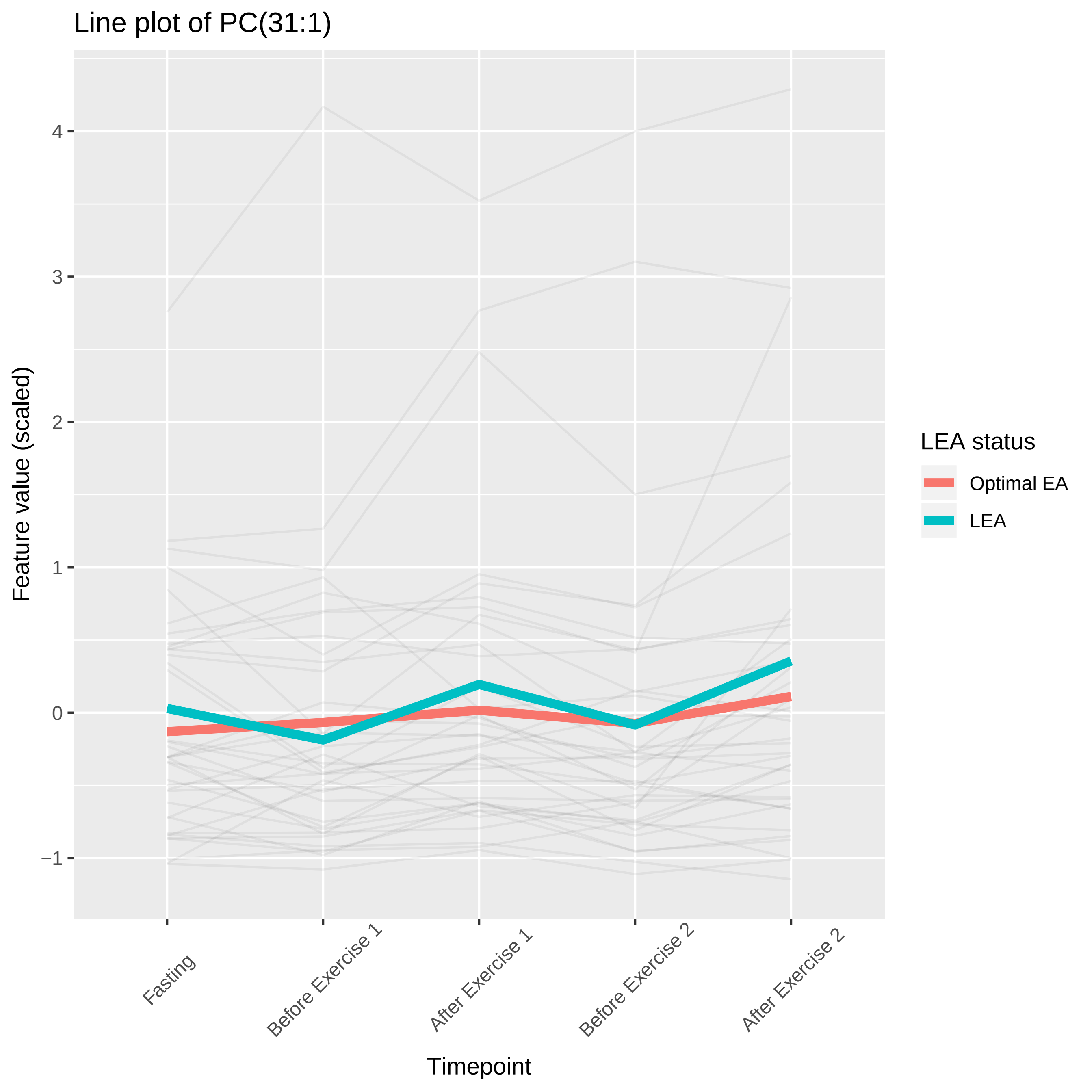
Line plot of PC(31:1)
Feature value (scaled)
4
3
2
1
0
−1
Fasting
Before Exercise 1
After Exercise 1
Before Exercise 2
After Exercise 2
Timepoint
LEA status
Optimal EA
LEA

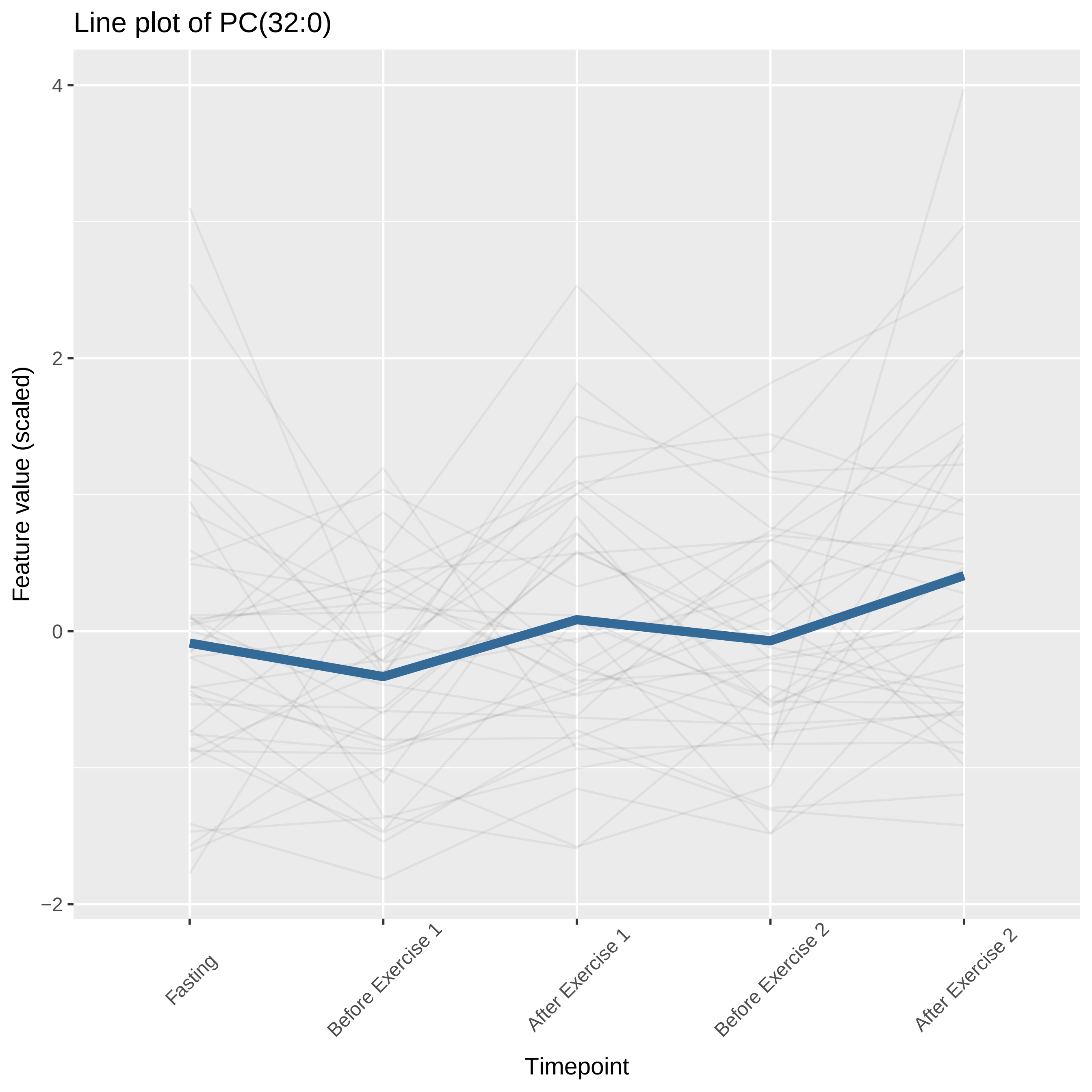

Line plot of PC(32:0)
Feature value (scaled)
4
2
0
−2
Fasting
Before Exercise 1
After Exercise 1
Before Exercise 2
After Exercise 2
Timepoint

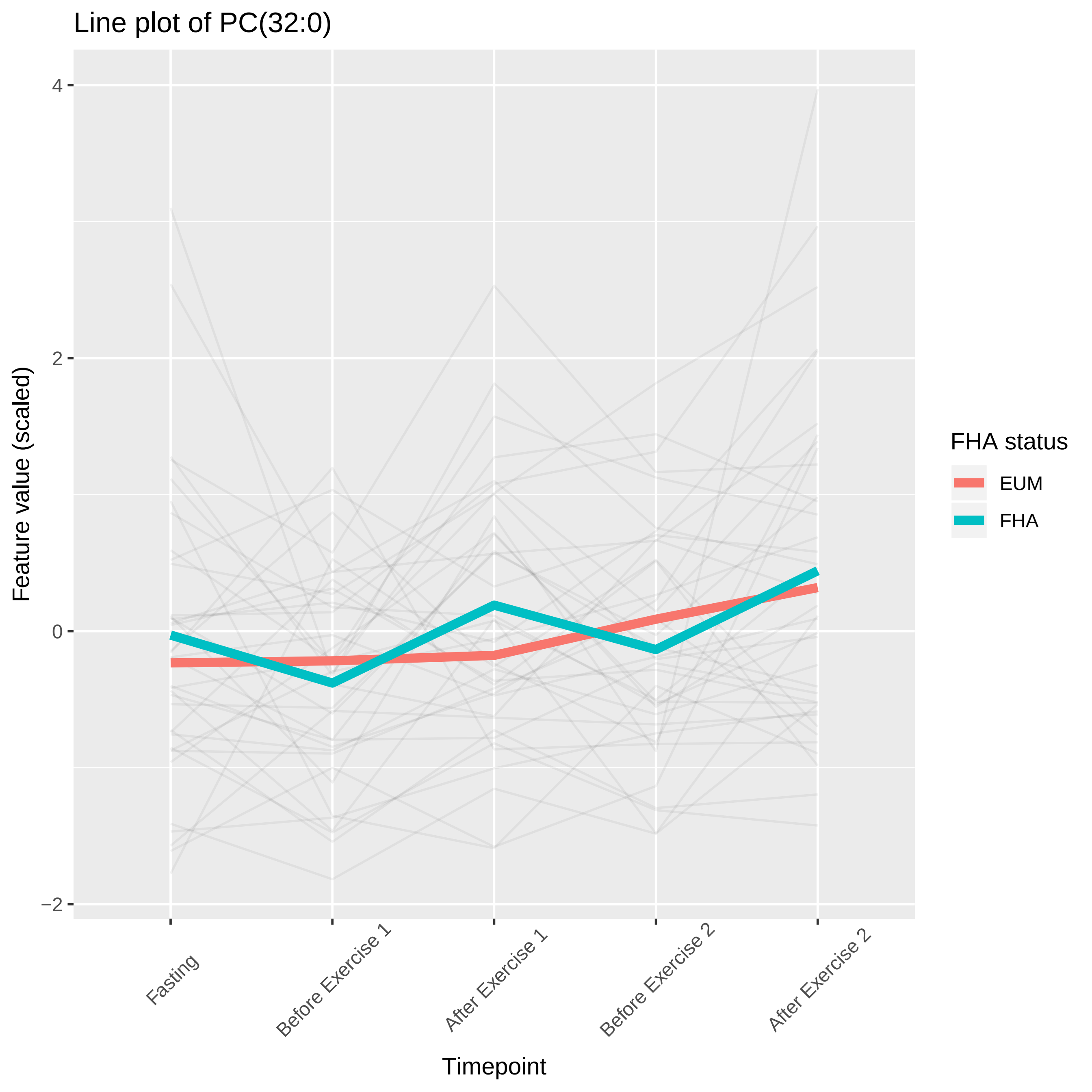

Line plot of PC(32:0)
4
2
0
−2
Feature value (scaled)
Fasting
Before Exercise 1
After Exercise 1
Before Exercise 2
After Exercise 2
Timepoint
FHA status
EUM
FHA

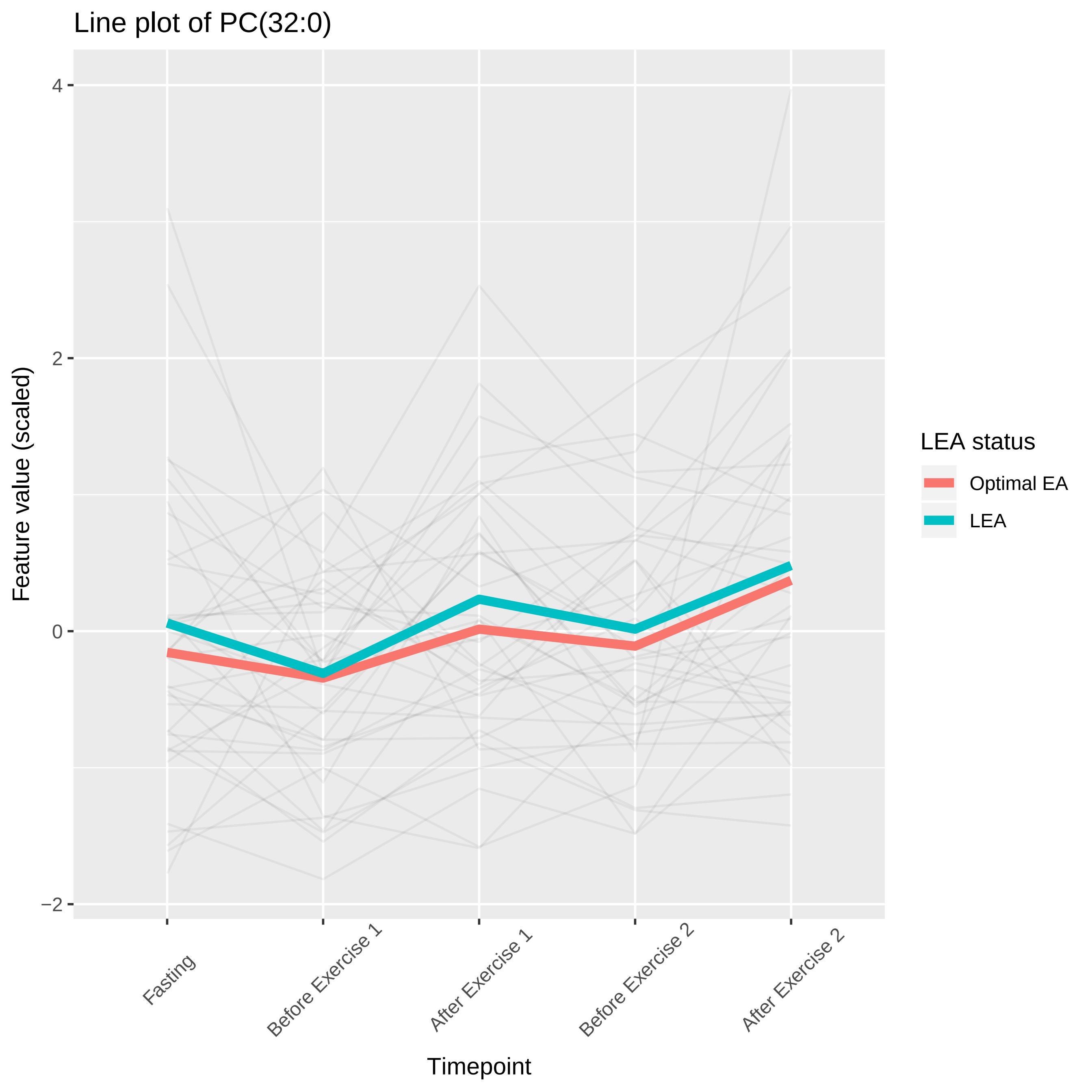

Line plot of PC(32:0)
Feature value (scaled)
4
2
0
−2
Fasting
Before Exercise 1
After Exercise 1
Before Exercise 2
After Exercise 2
Timepoint
LEA status
Optimal EA
LEA

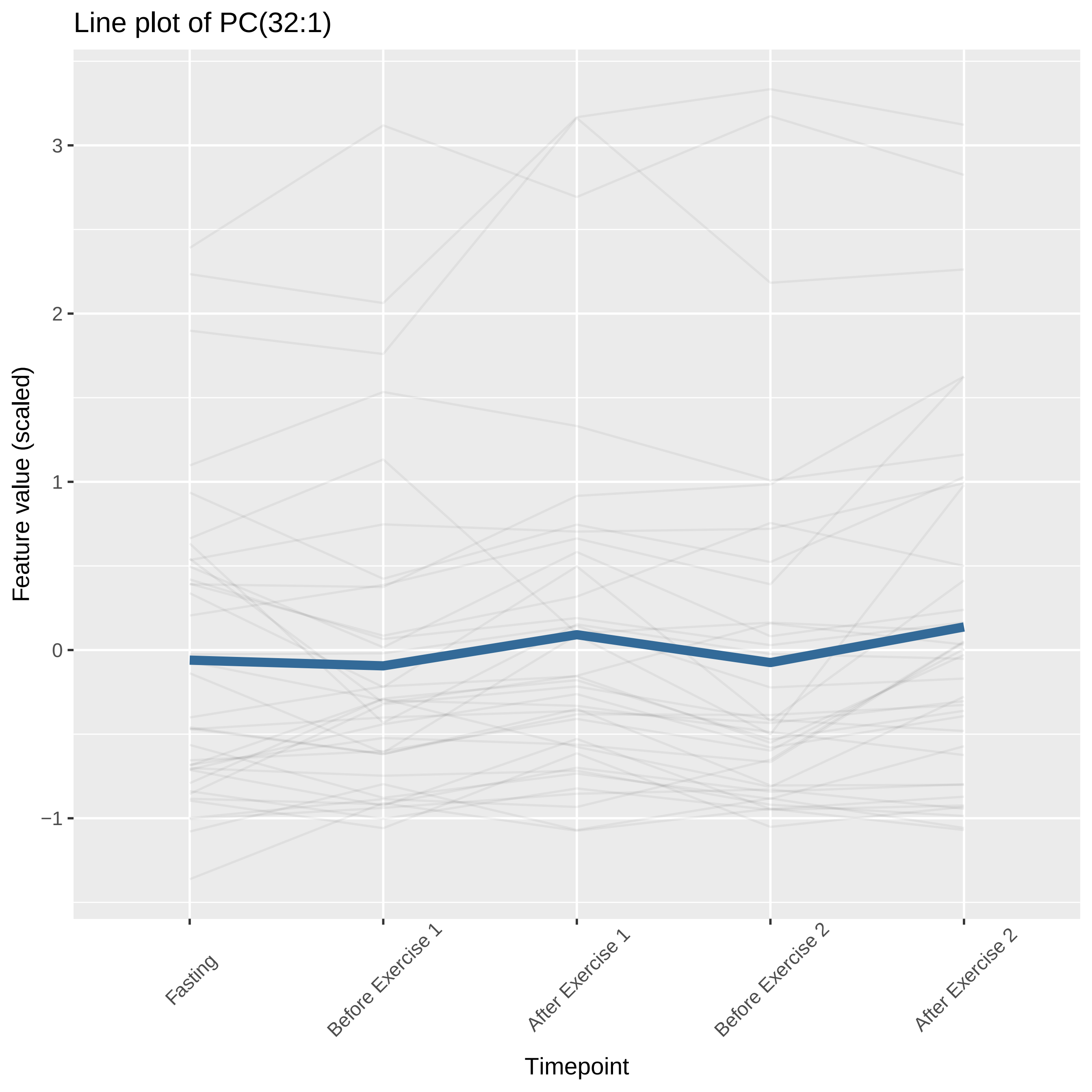

Line plot of PC(32:1)
Feature value (scaled)
3
2
1
0
−1
Fasting
Before Exercise 1
After Exercise 1
Before Exercise 2
After Exercise 2
Timepoint

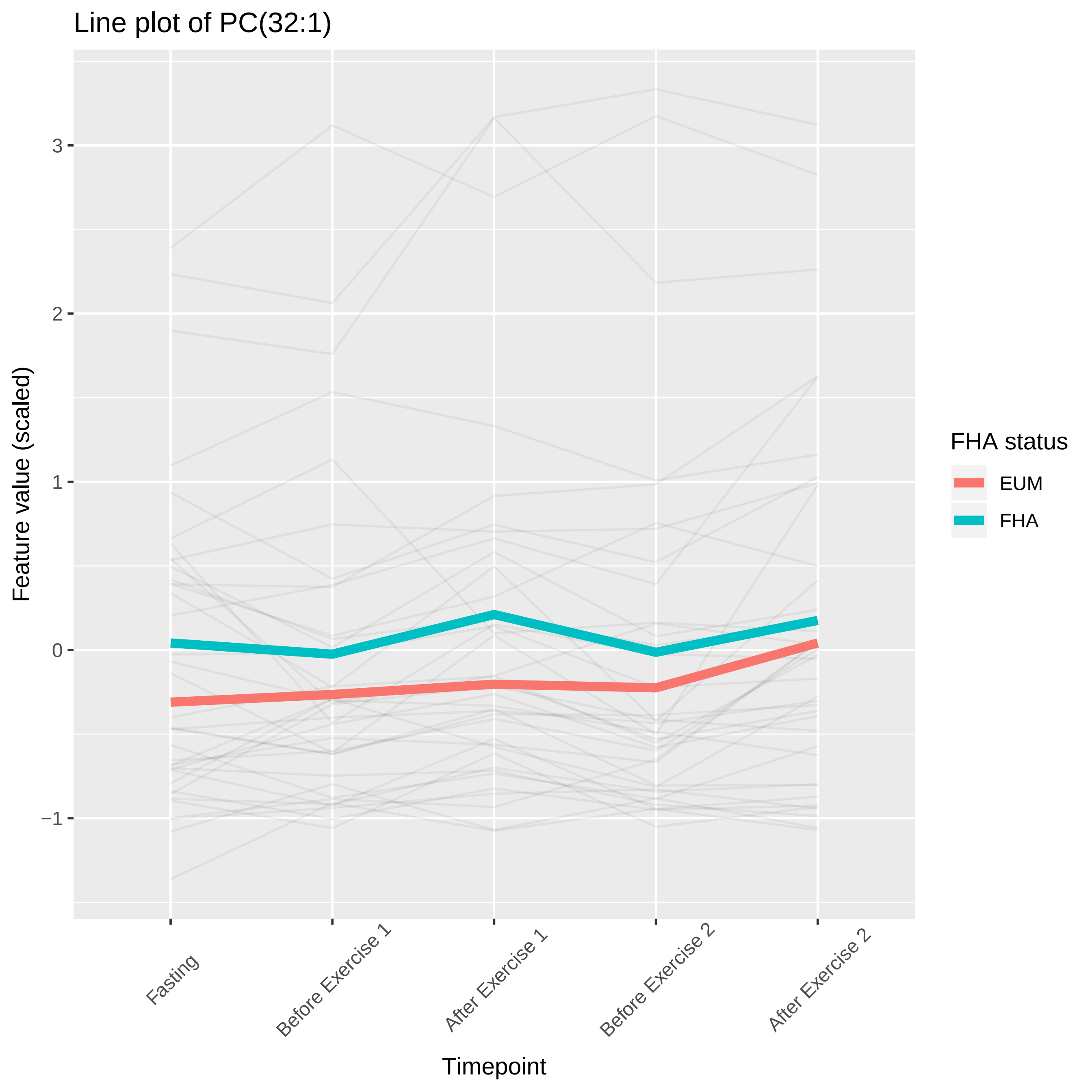

Line plot of PC(32:1)
Feature value (scaled)
3
2
1
0
−1
Fasting
Before Exercise 1
After Exercise 1
Before Exercise 2
After Exercise 2
Timepoint
FHA status
EUM
FHA

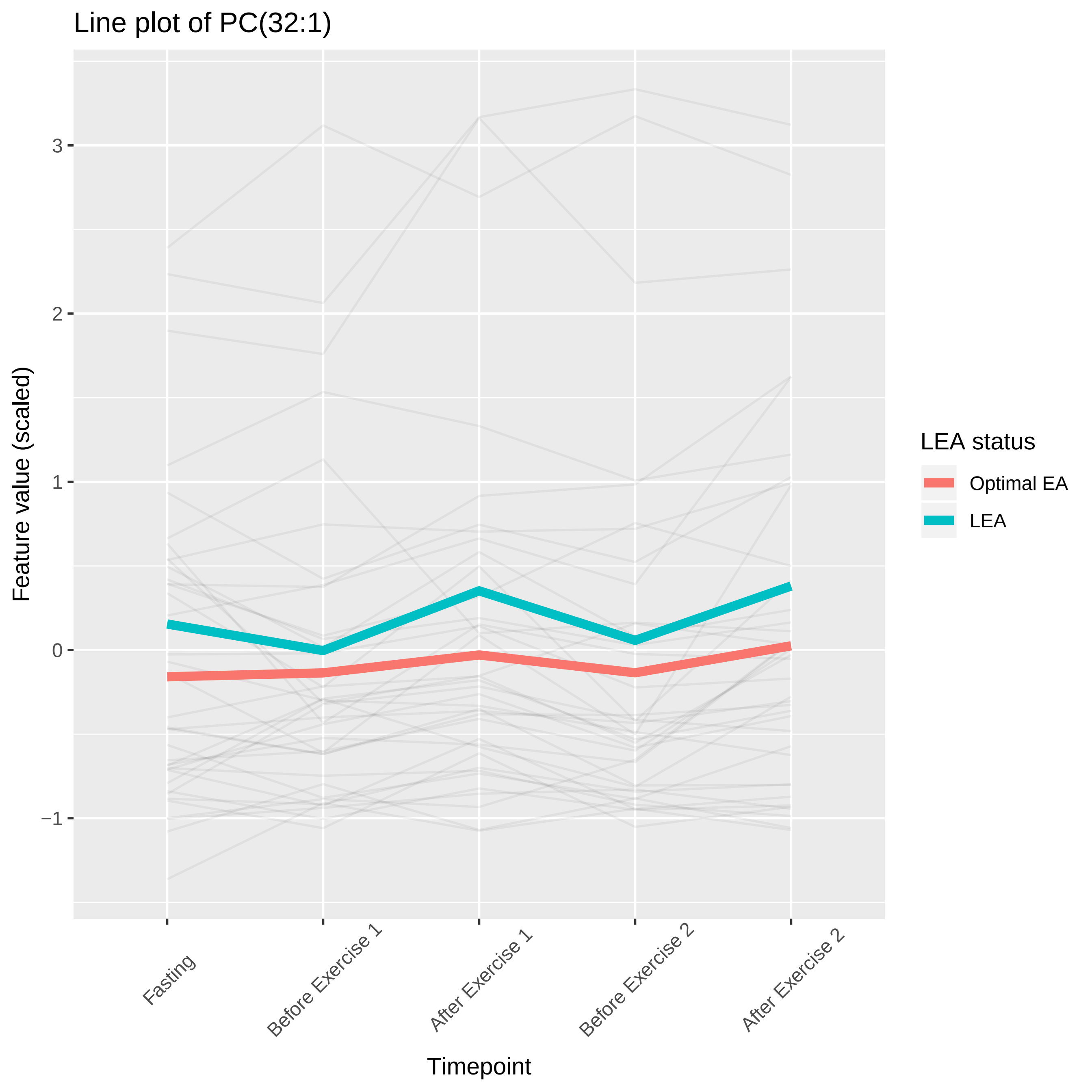

Line plot of PC(32:1)
Feature value (scaled)
3
2
1
0
−1
Fasting
Before Exercise 1
After Exercise 1
Before Exercise 2
After Exercise 2
Timepoint
LEA status
Optimal EA
LEA

Line plot of PC(32:2)

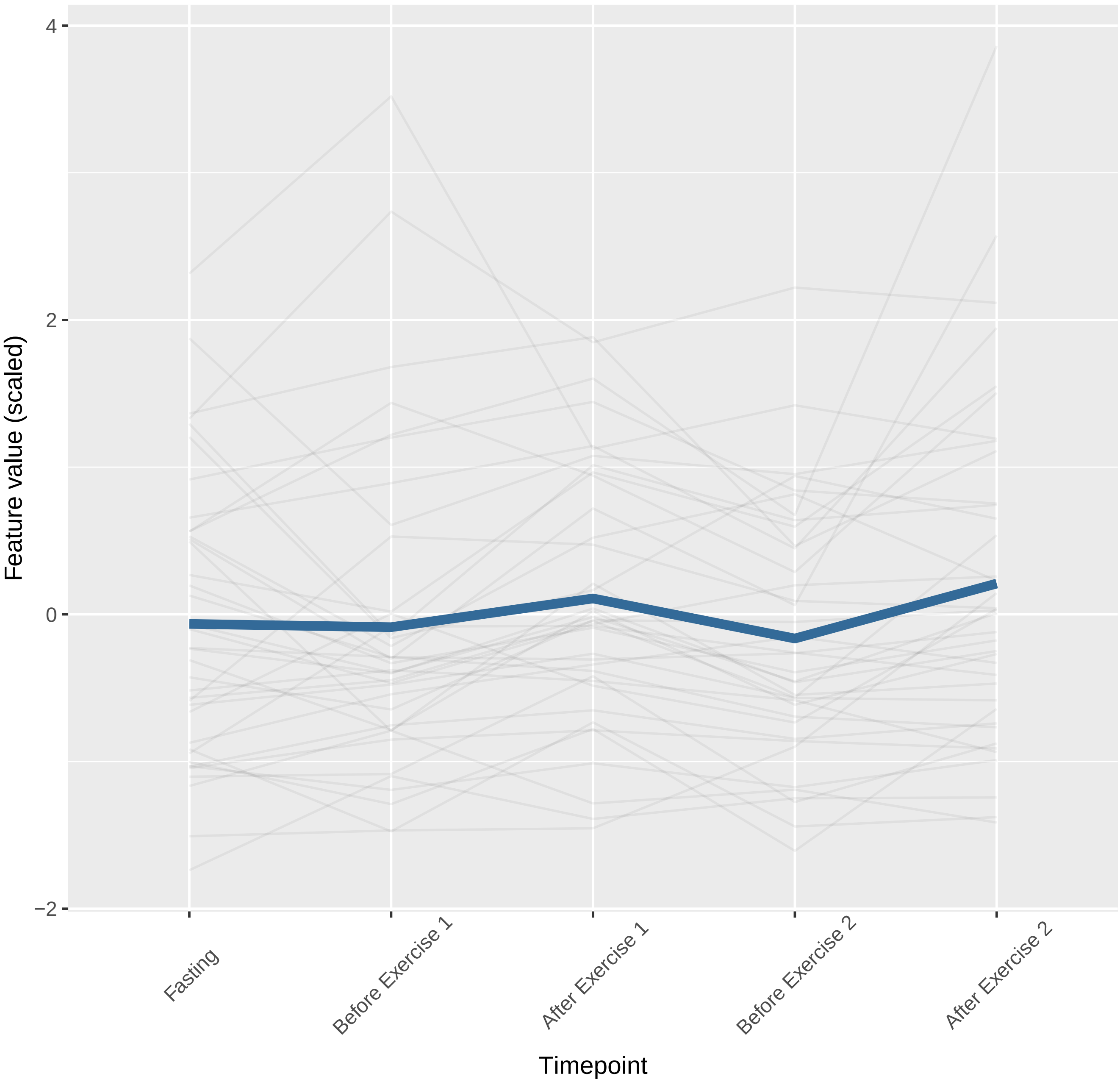

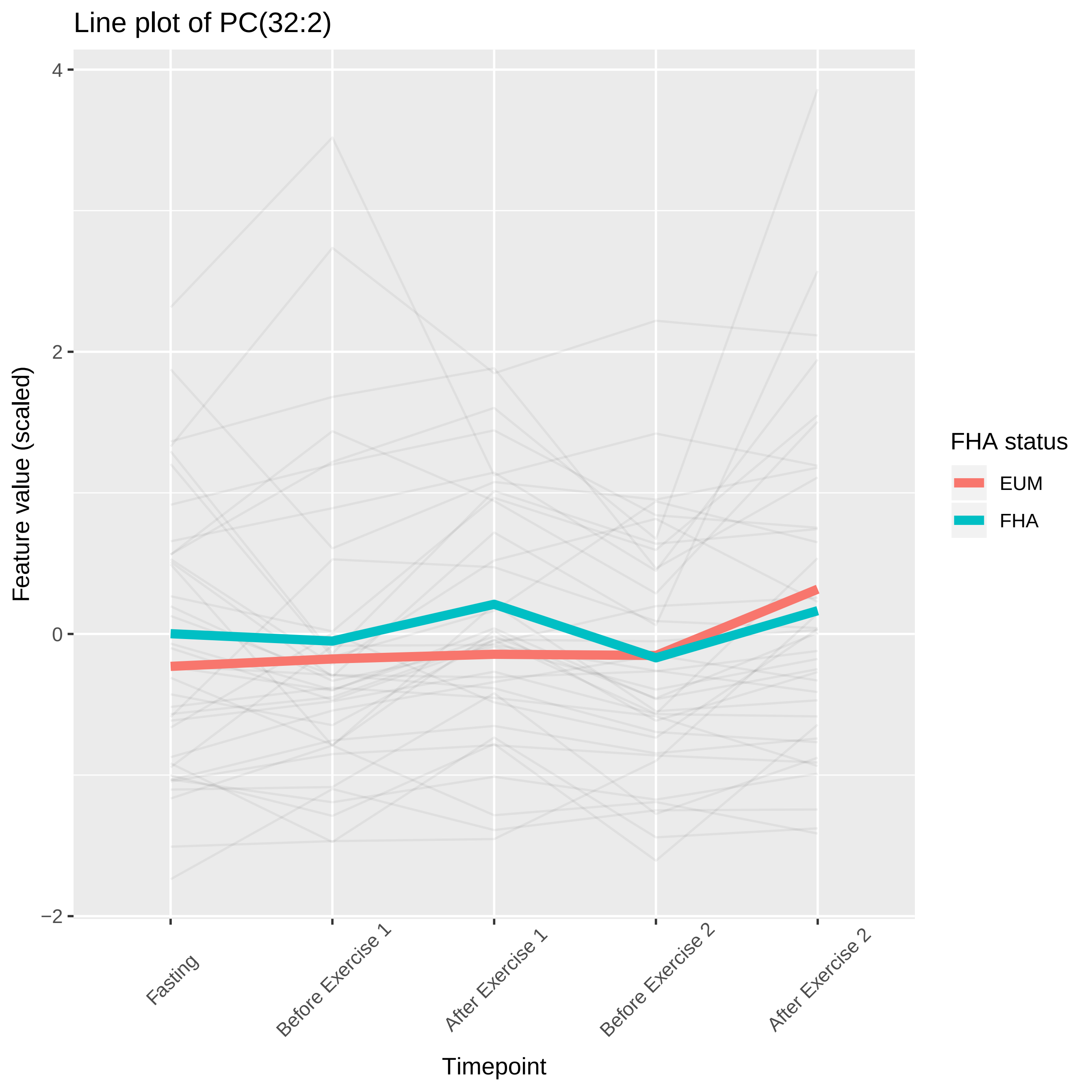
Line plot of PC(32:2)
Feature value (scaled)
4
2
0
−2
Fasting
Before Exercise 1
After Exercise 1
Before Exercise 2
After Exercise 2
Timepoint
FHA status
EUM
FHA

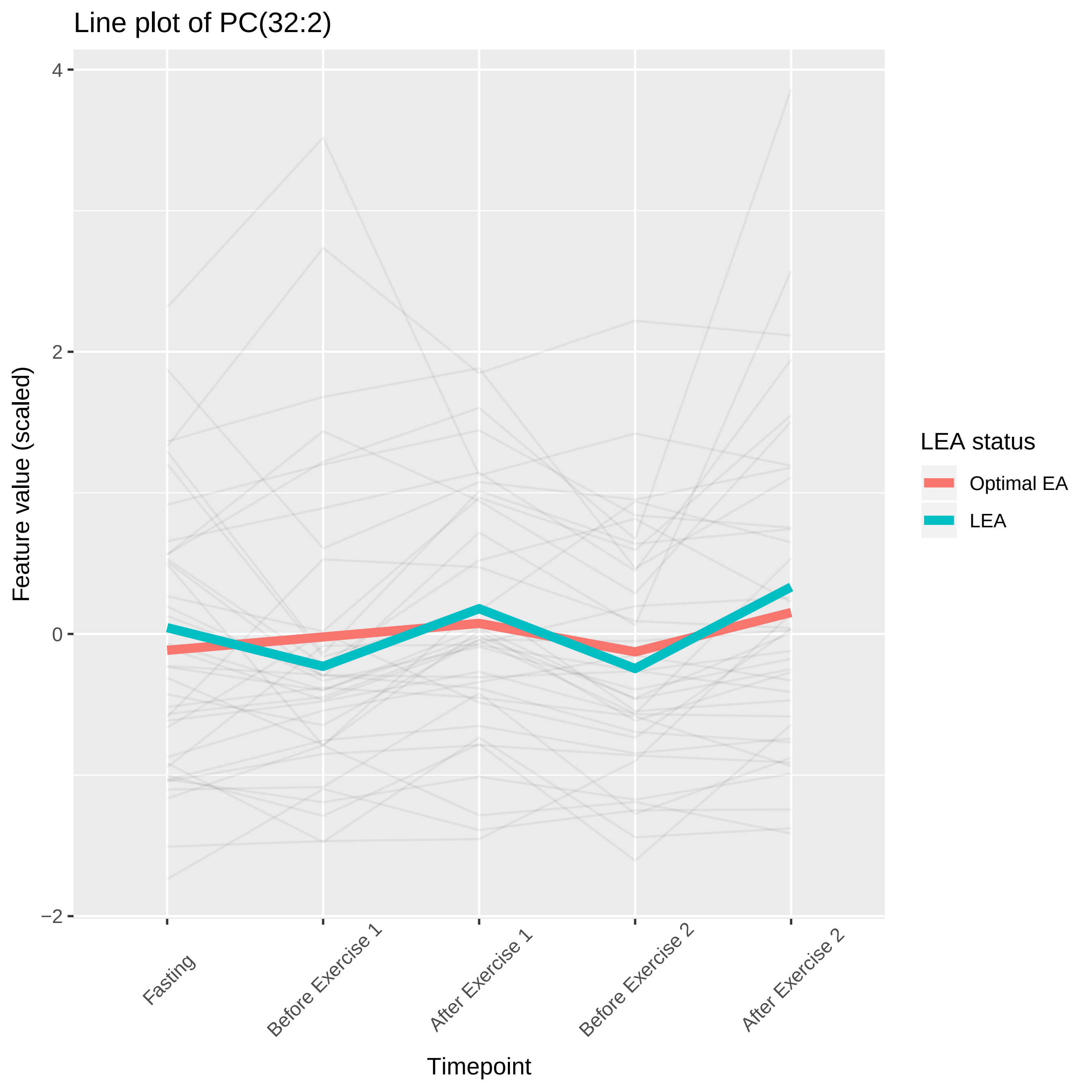

Line plot of PC(32:2)
4
2
0
−2
Feature value (scaled)
Fasting
Before Exercise 1
After Exercise 1
Before Exercise 2
After Exercise 2
Timepoint
LEA status
Optimal EA
LEA

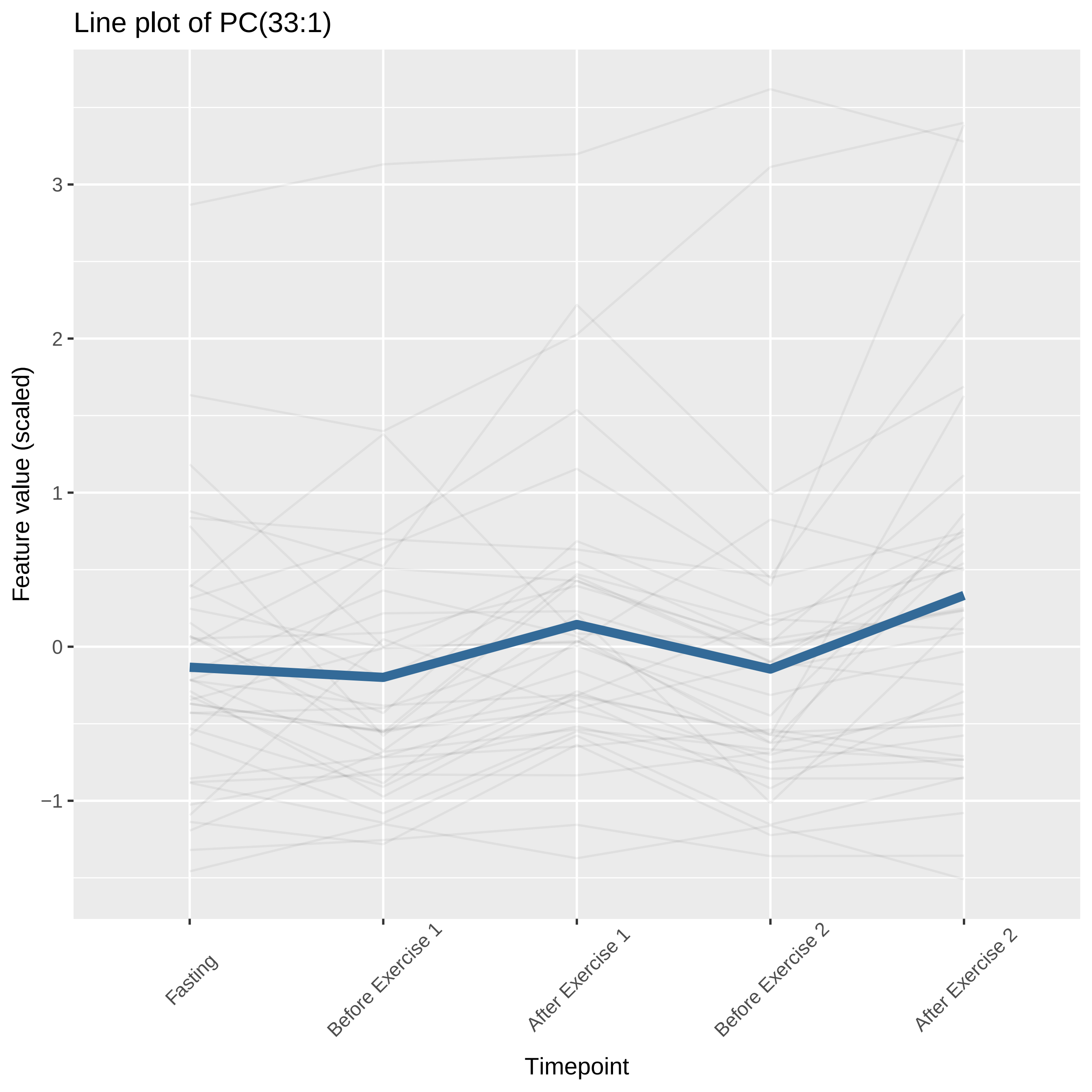

Line plot of PC(33:1)
Feature value (scaled)
3
2
1
0
−1
Fasting
Before Exercise 1
After Exercise 1
Before Exercise 2
After Exercise 2
Timepoint

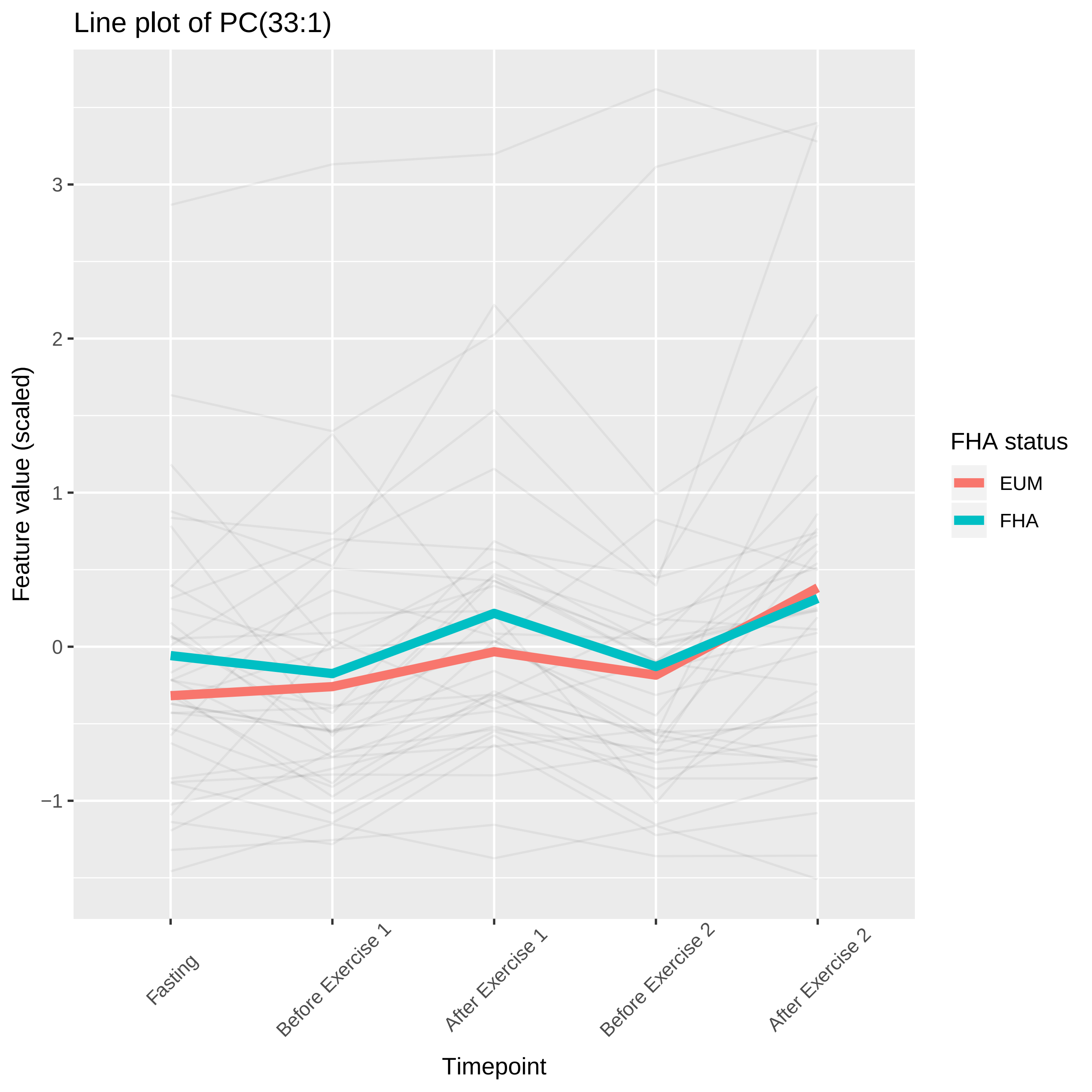

Line plot of PC(33:1)
Feature value (scaled)
3
2
1
0
−1
Fasting
Before Exercise 1
After Exercise 1
Before Exercise 2
After Exercise 2
Timepoint
FHA status
EUM
FHA

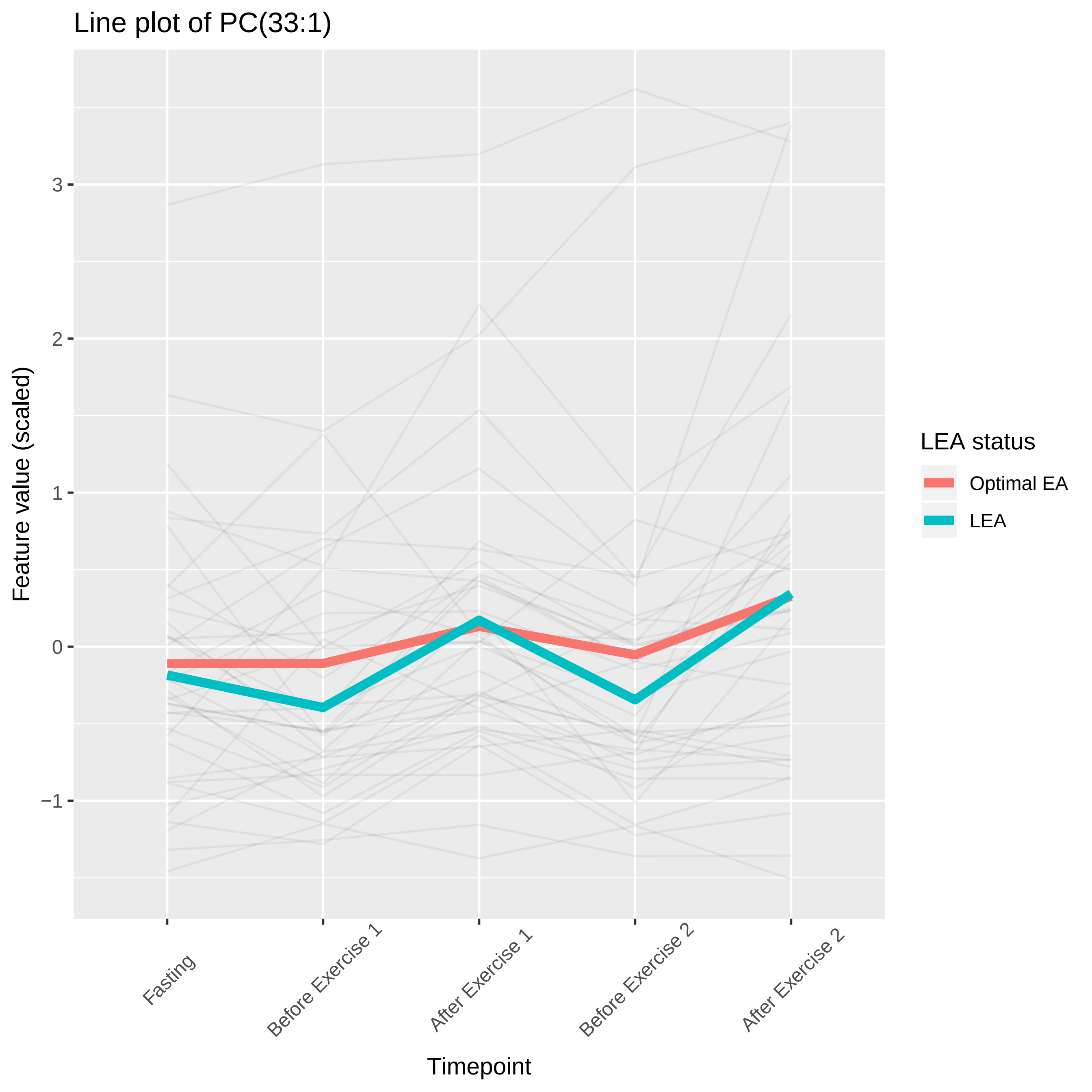

Line plot of PC(33:1)
Feature value (scaled)
3
2
1
0
−1
Fasting
Before Exercise 1
After Exercise 1
Before Exercise 2
After Exercise 2
Timepoint
LEA status
Optimal EA
LEA

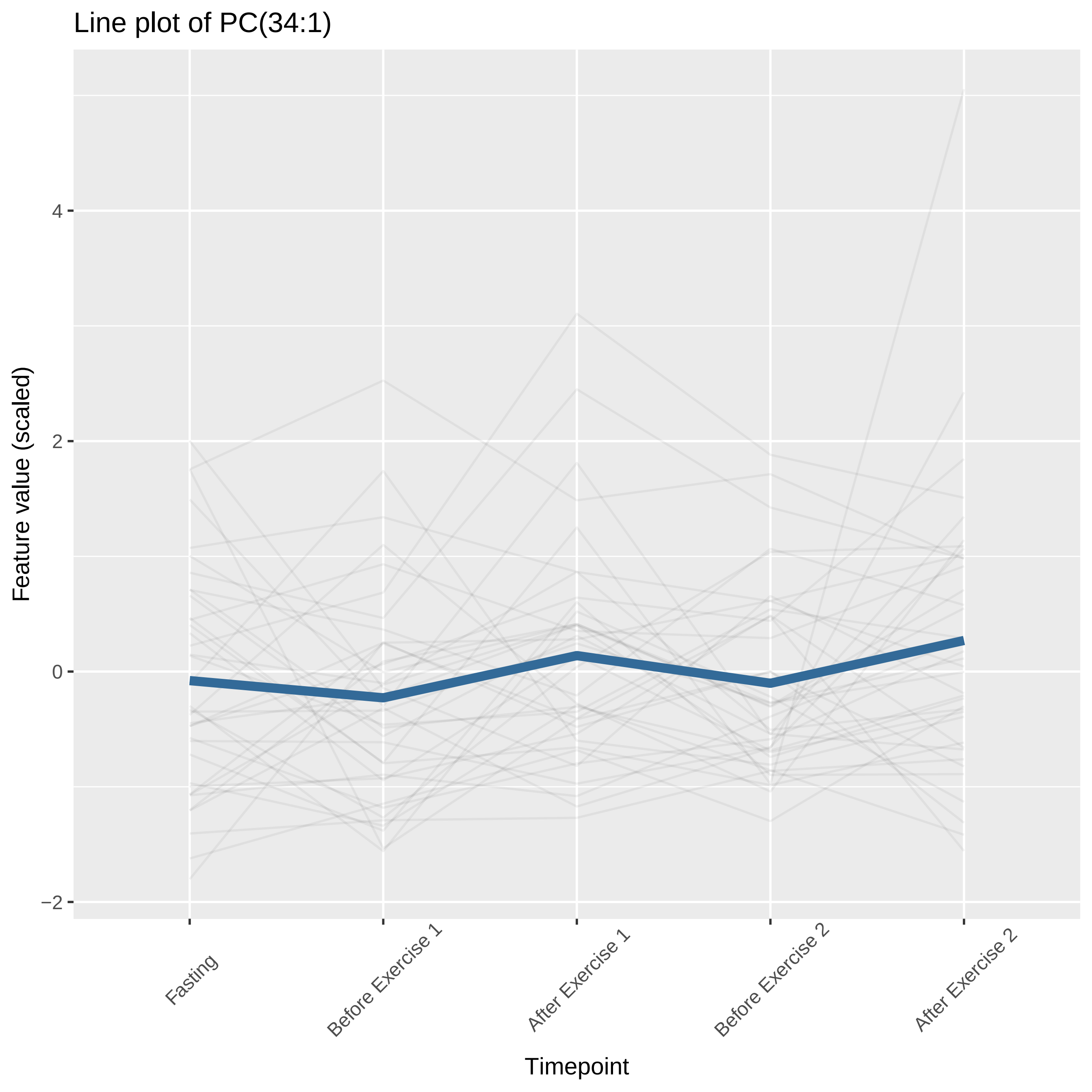

Line plot of PC(34:1)
Feature value (scaled)
4
2
0
−2
Fasting
Before Exercise 1
After Exercise 1
Before Exercise 2
After Exercise 2
Timepoint

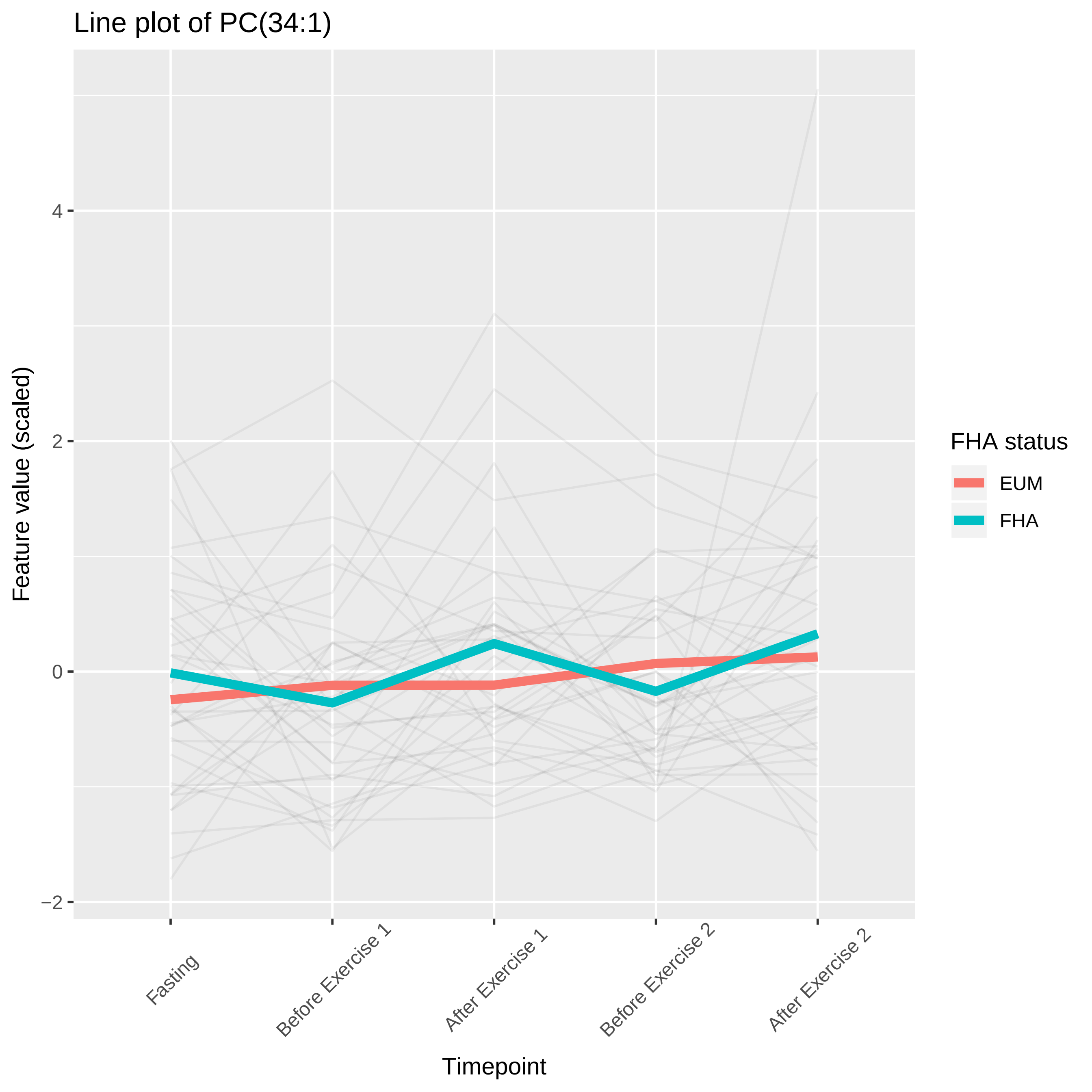
Line plot of PC(34:1)
Feature value (scaled)
4
2
0
−2
Fasting
Before Exercise 1
After Exercise 1
Before Exercise 2
After Exercise 2
Timepoint
FHA status
EUM
FHA

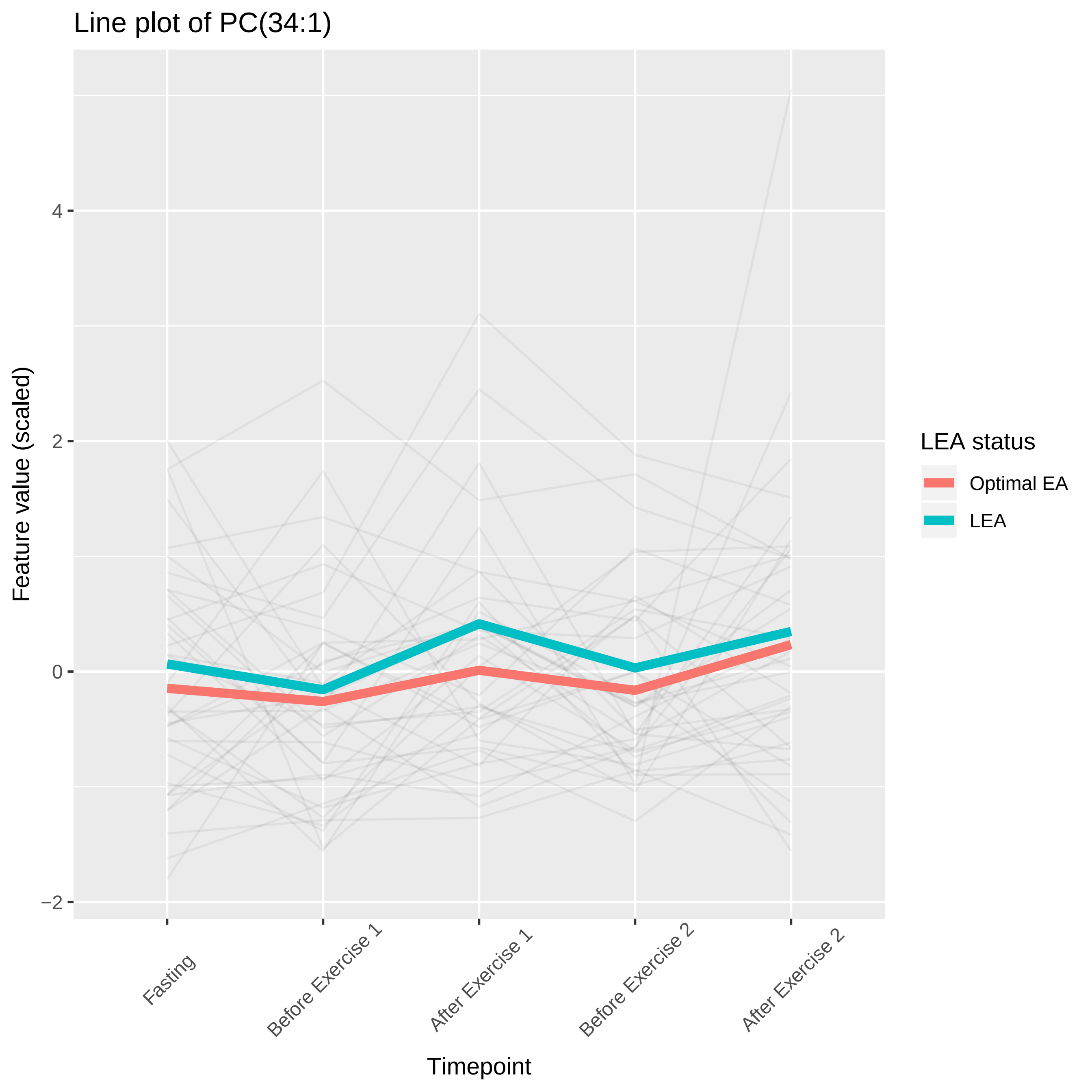
Line plot of PC(34:1)
Feature value (scaled)
4
2
0
−2
Fasting
Before Exercise 1
After Exercise 1
Before Exercise 2
After Exercise 2
Timepoint
LEA status
Optimal EA
LEA

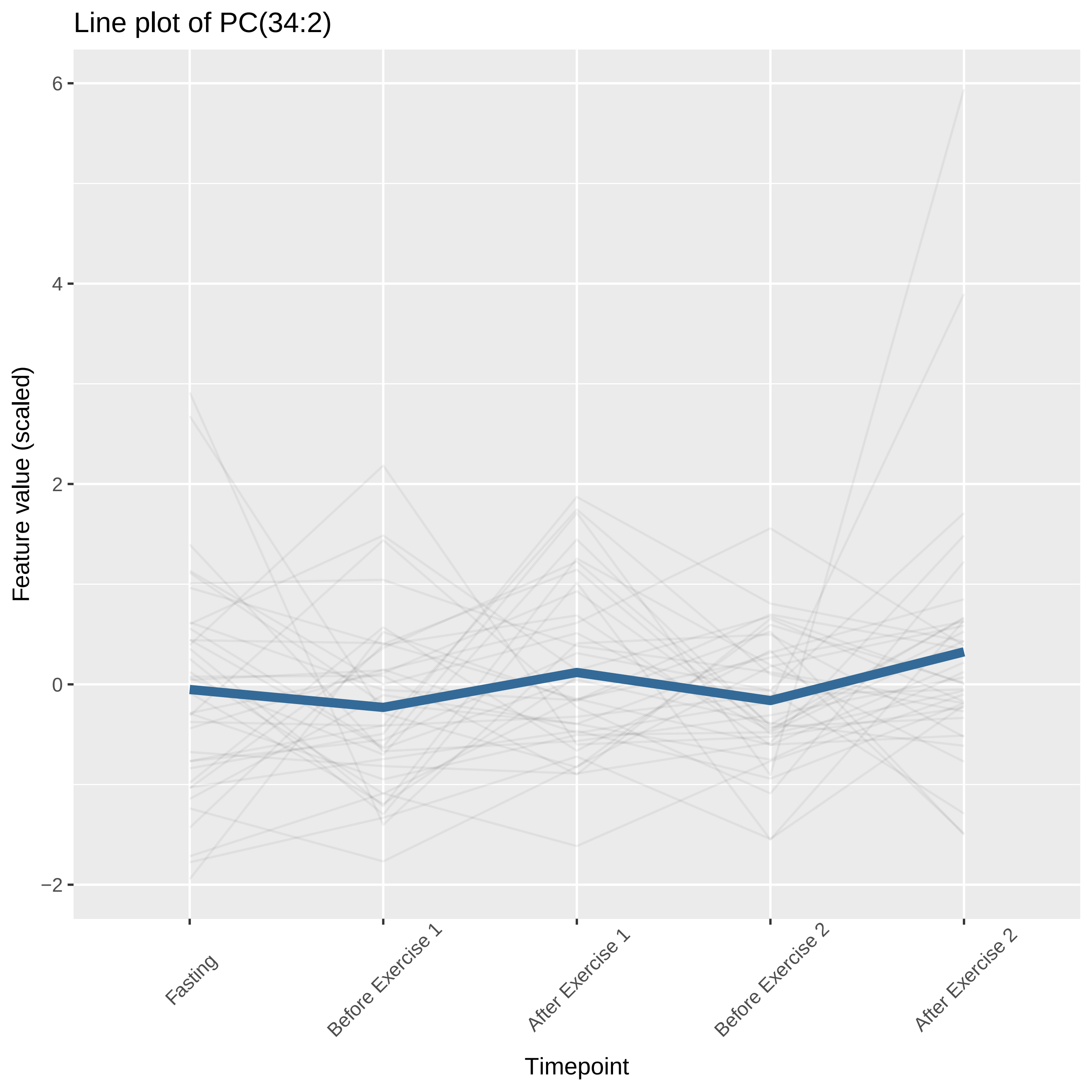

Line plot of PC(34:2)
Feature value (scaled)
6
4
2
0
−2
Fasting
Before Exercise 1
After Exercise 1
Before Exercise 2
After Exercise 2
Timepoint

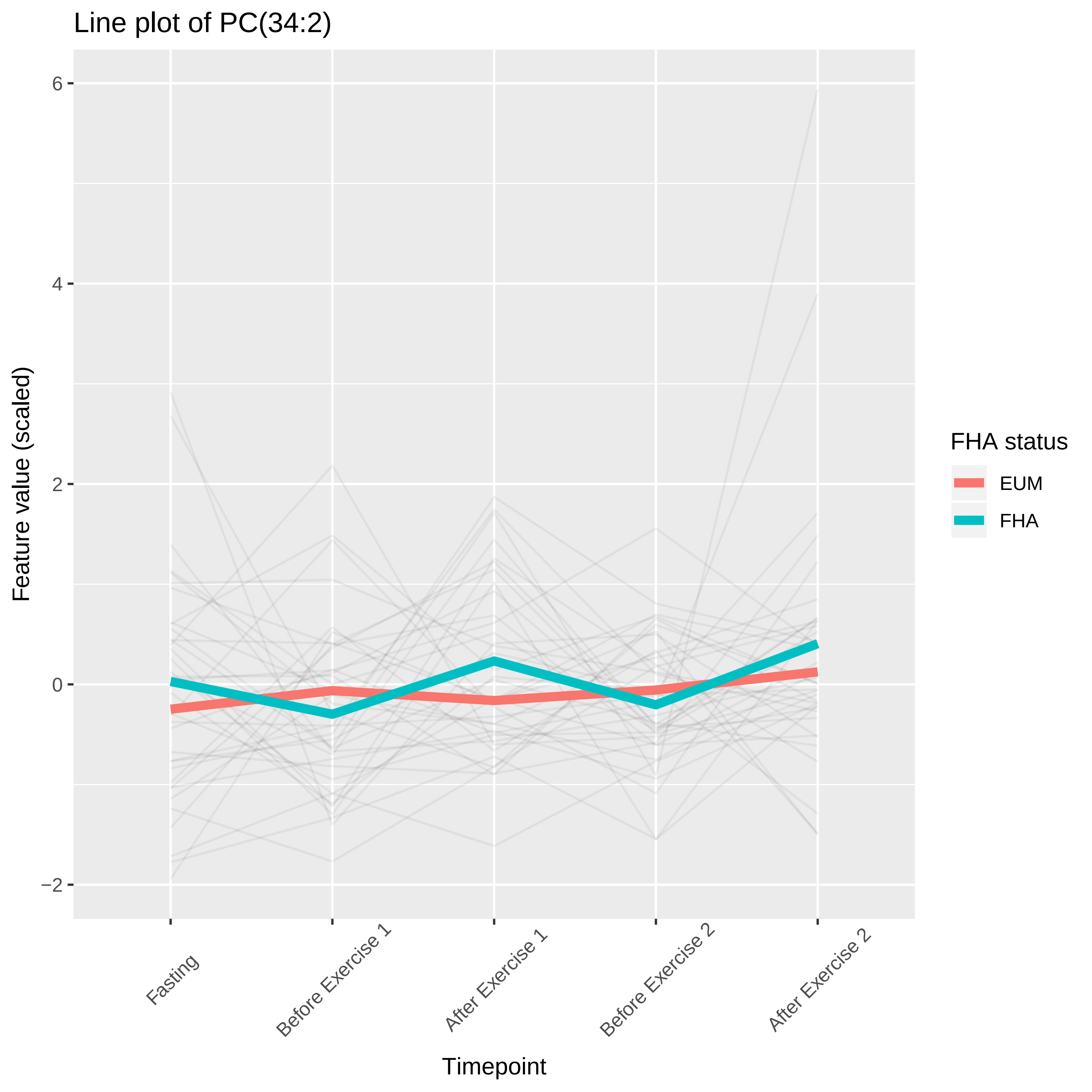
Line plot of PC(34:2)
Feature value (scaled)
6
4
2
0
−2
Fasting
Before Exercise 1
After Exercise 1
Before Exercise 2
After Exercise 2
Timepoint
FHA status
EUM
FHA

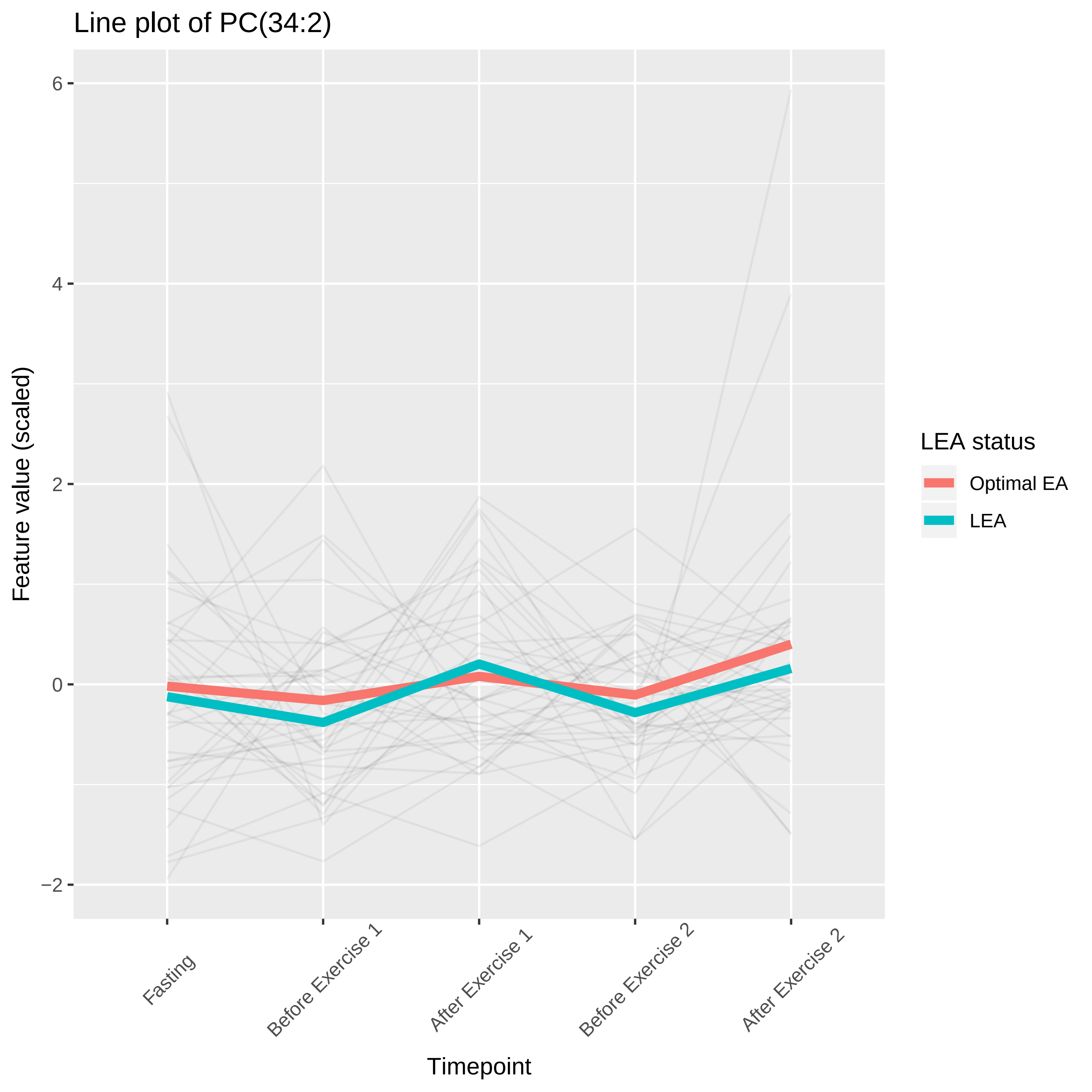

Line plot of PC(34:2)
Feature value (scaled)
6
4
2
0
−2
Fasting
Before Exercise 1
After Exercise 1
Before Exercise 2
After Exercise 2
Timepoint
LEA status
Optimal EA
LEA

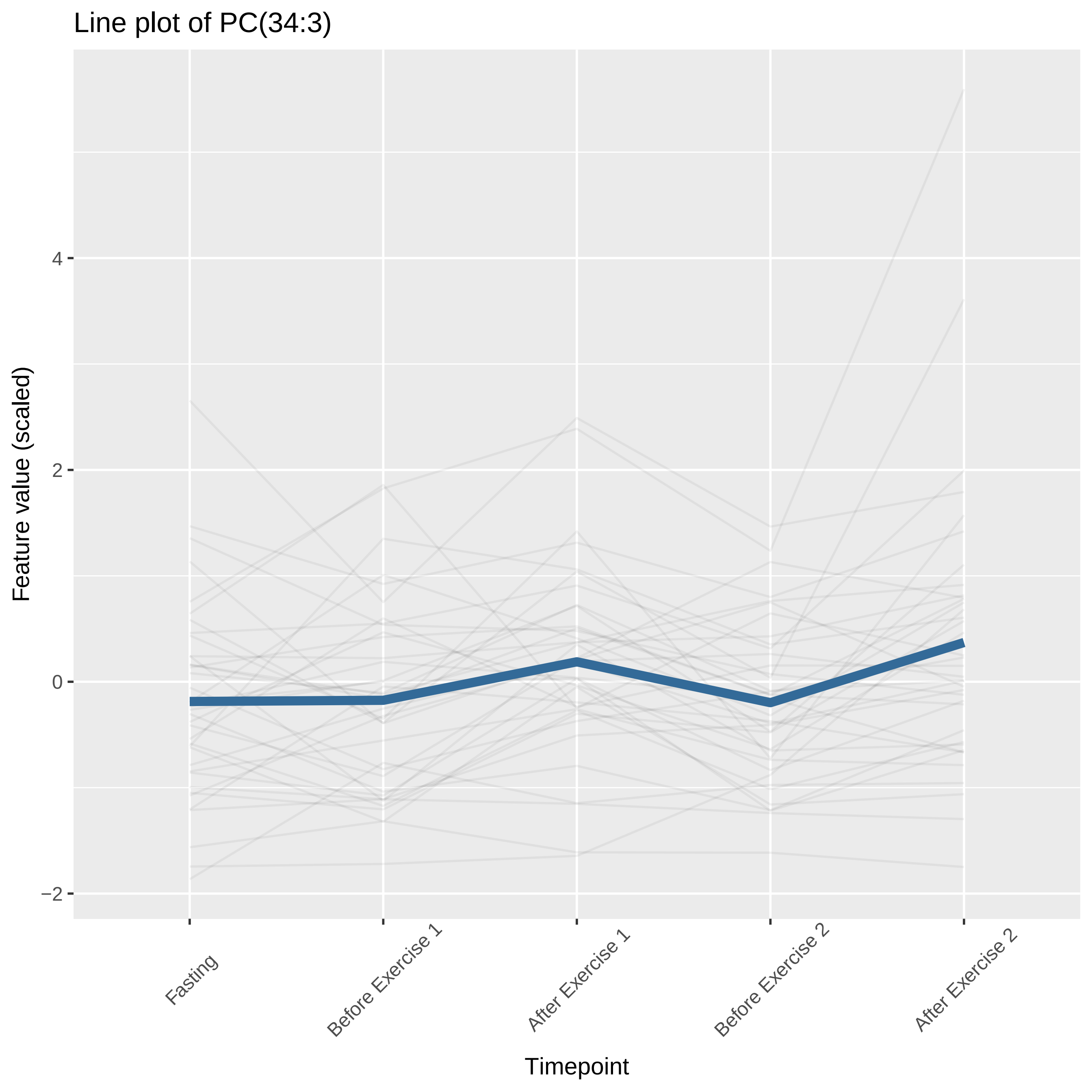

Line plot of PC(34:3)
Feature value (scaled)
4
2
0
−2
Fasting
Before Exercise 1
After Exercise 1
Before Exercise 2
After Exercise 2
Timepoint

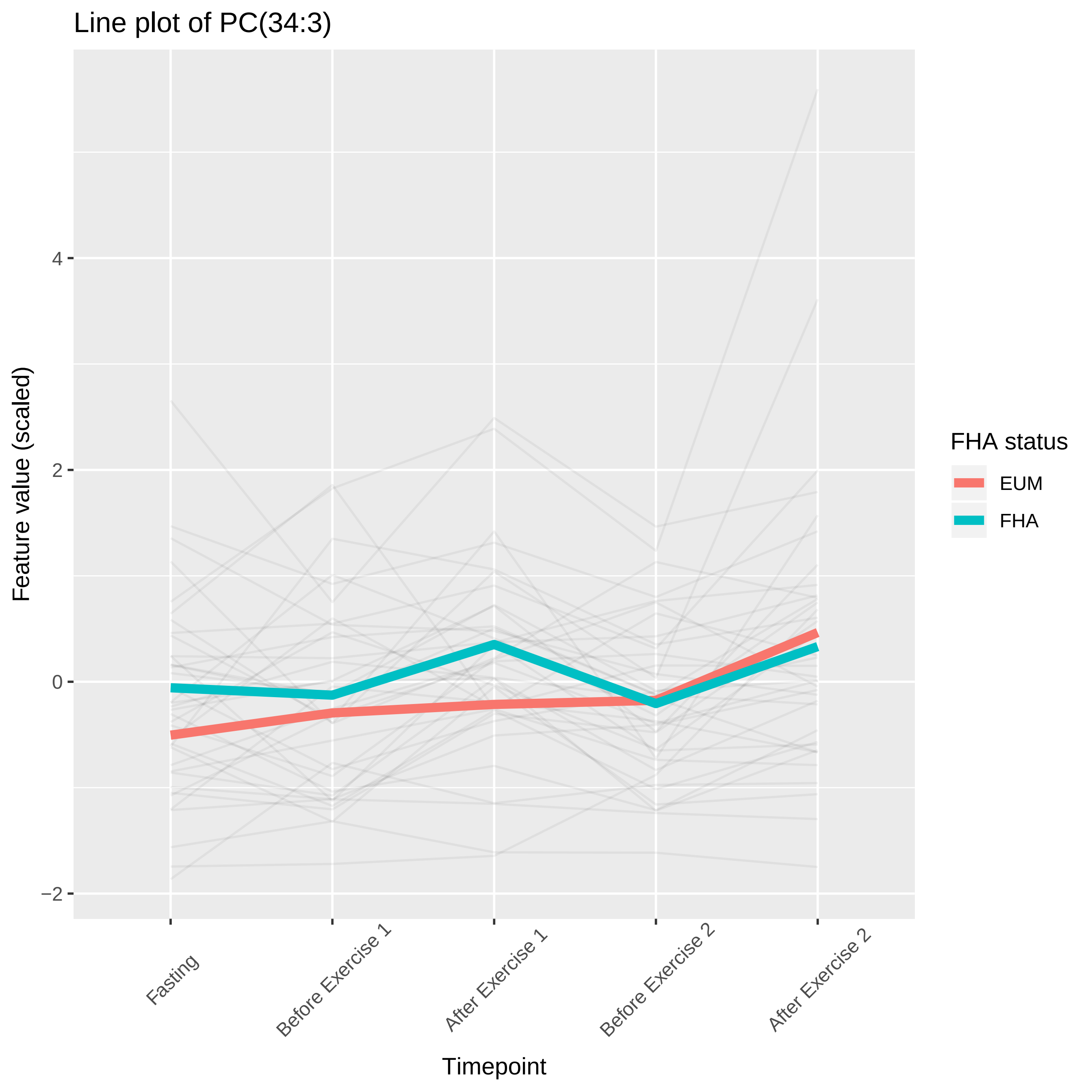

Line plot of PC(34:3)
Feature value (scaled)
4
2
0
−2
Fasting
Before Exercise 1
After Exercise 1
Before Exercise 2
After Exercise 2
Timepoint
FHA status
EUM
FHA

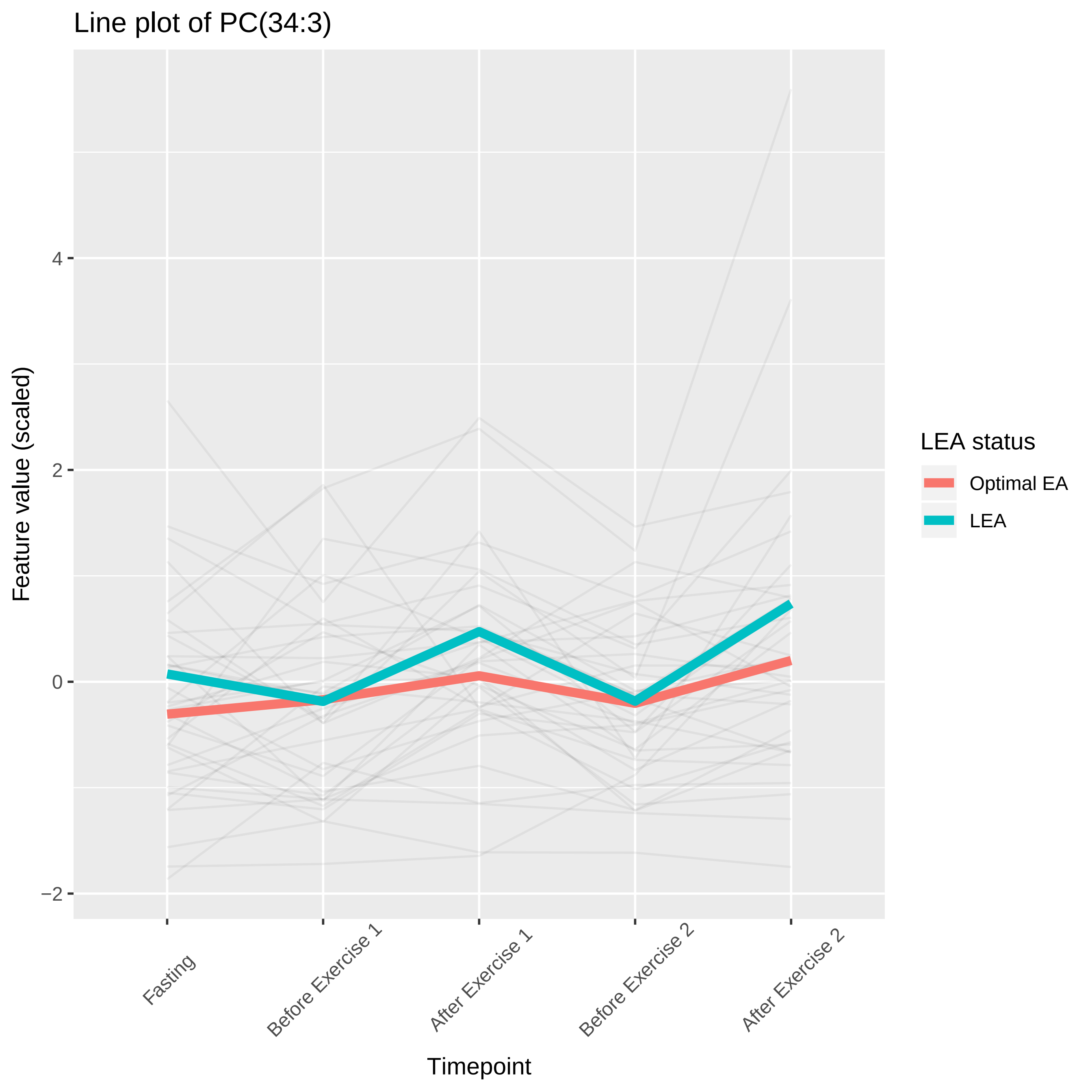

Line plot of PC(34:3)
Feature value (scaled)
4
2
0
−2
Fasting
Before Exercise 1
After Exercise 1
Before Exercise 2
After Exercise 2
Timepoint
LEA status
Optimal EA
LEA

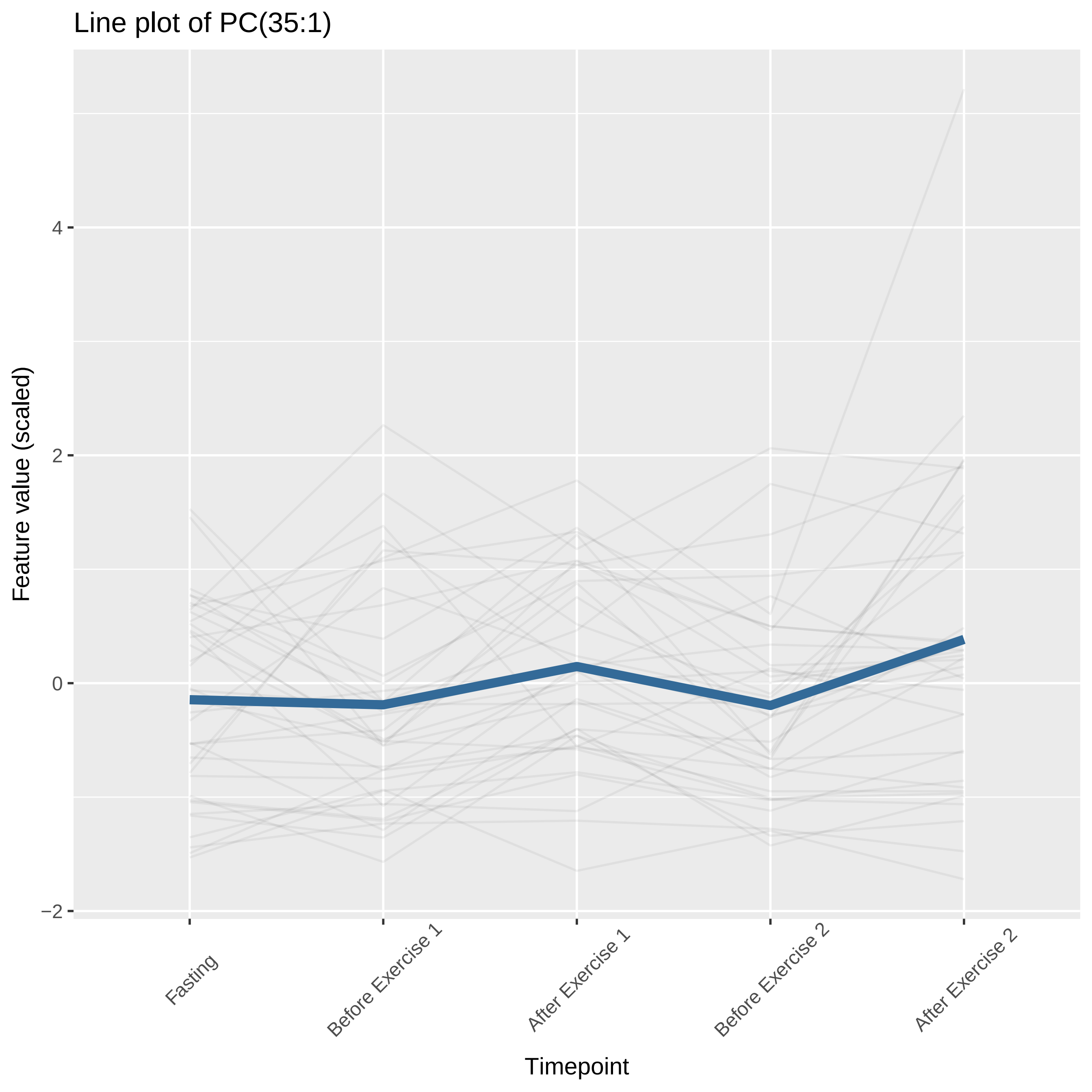

Line plot of PC(35:1)
Feature value (scaled)
4
2
0
−2
Fasting
Before Exercise 1
After Exercise 1
Before Exercise 2
After Exercise 2
Timepoint

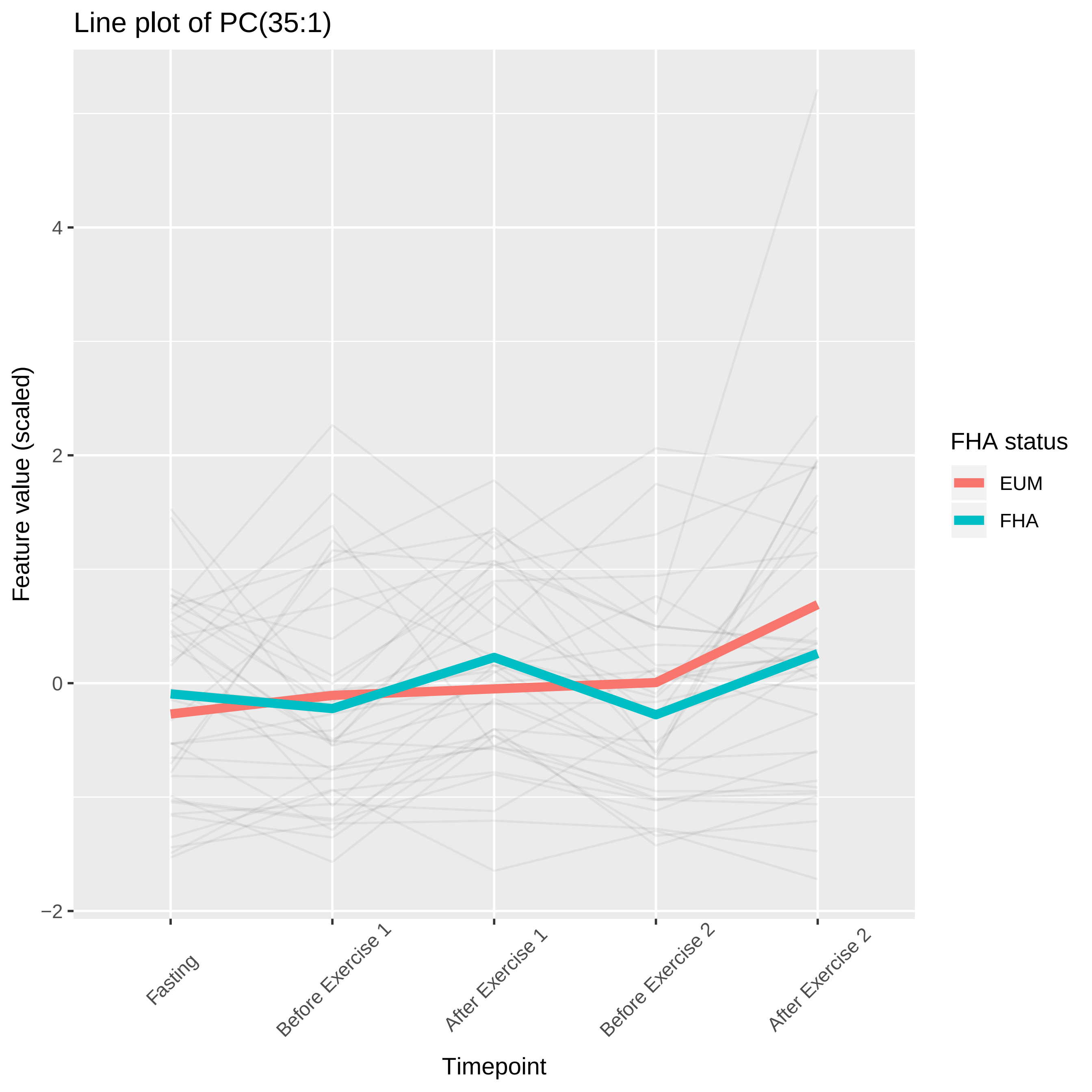

Line plot of PC(35:1)
Feature value (scaled)
4
2
0
−2
Fasting
Before Exercise 1
After Exercise 1
Before Exercise 2
After Exercise 2
Timepoint
FHA status
EUM
FHA

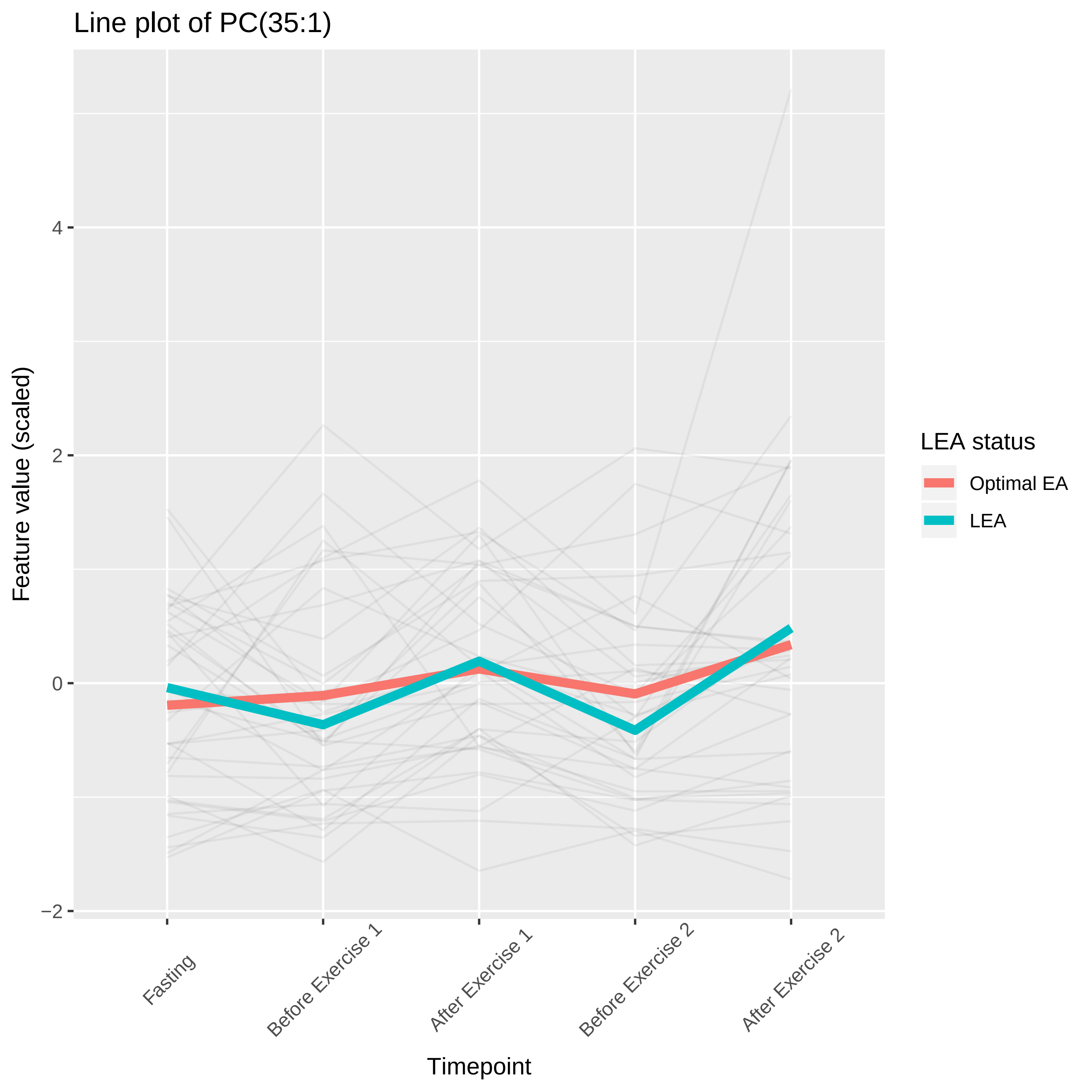

Line plot of PC(35:1)
Feature value (scaled)
4
2
0
−2
Fasting
Before Exercise 1
After Exercise 1
Before Exercise 2
After Exercise 2
Timepoint
LEA status
Optimal EA
LEA

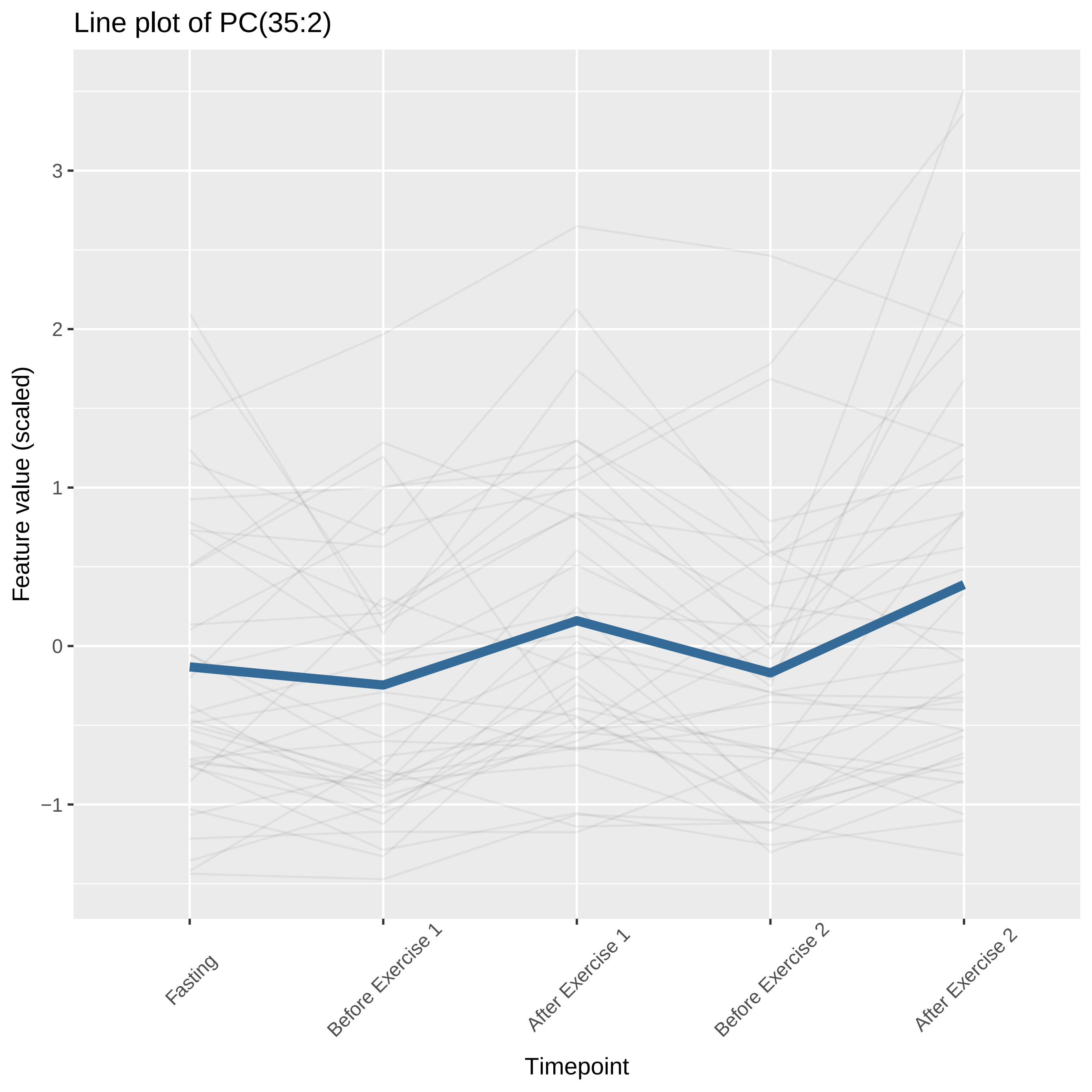
Line plot of PC(35:2)
Feature value (scaled)
3
2
1
0
−1
Fasting
Before Exercise 1
After Exercise 1
Before Exercise 2
After Exercise 2
Timepoint

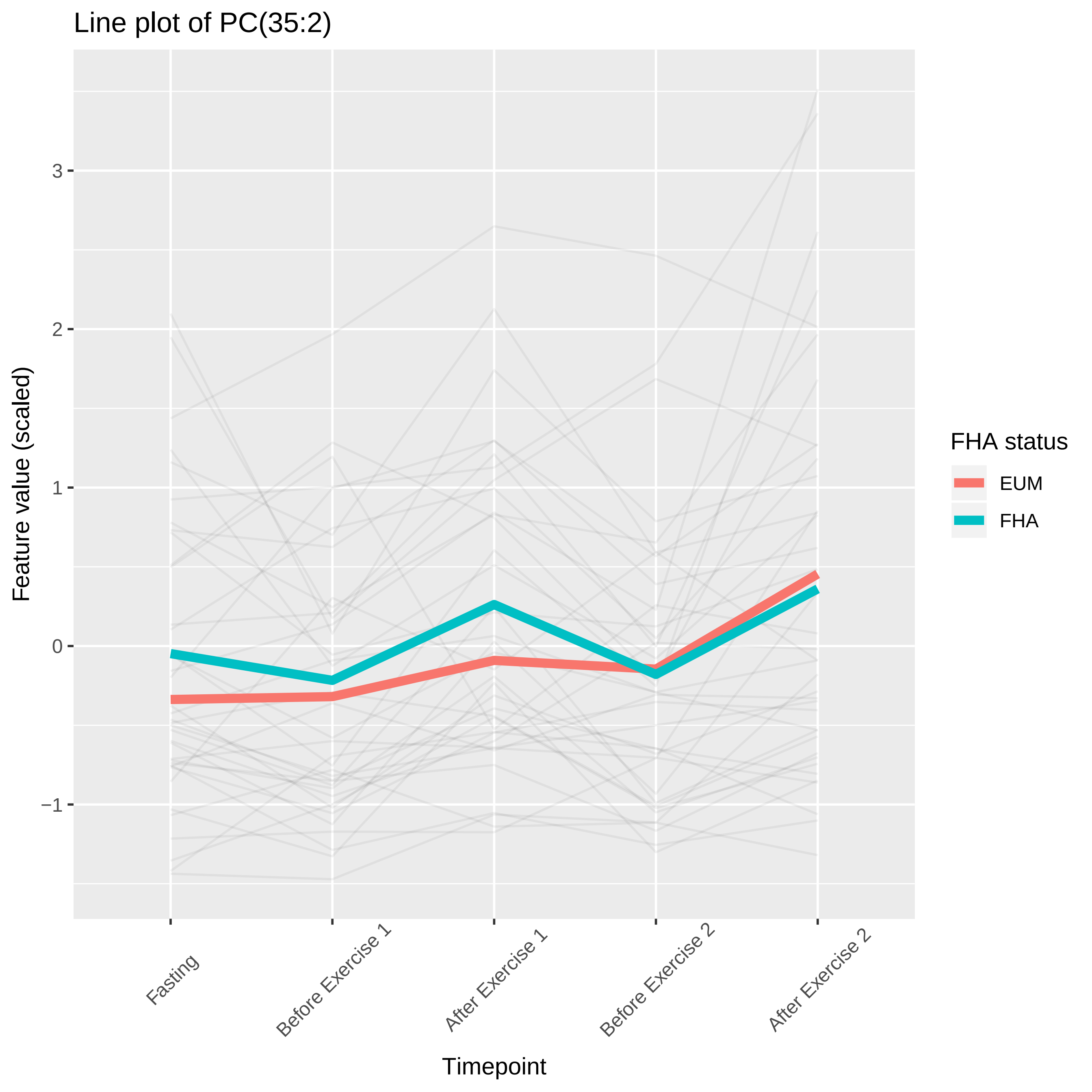

Line plot of PC(35:2)
Feature value (scaled)
3
2
1
0
−1
Fasting
Before Exercise 1
After Exercise 1
Before Exercise 2
After Exercise 2
Timepoint
FHA status
EUM
FHA

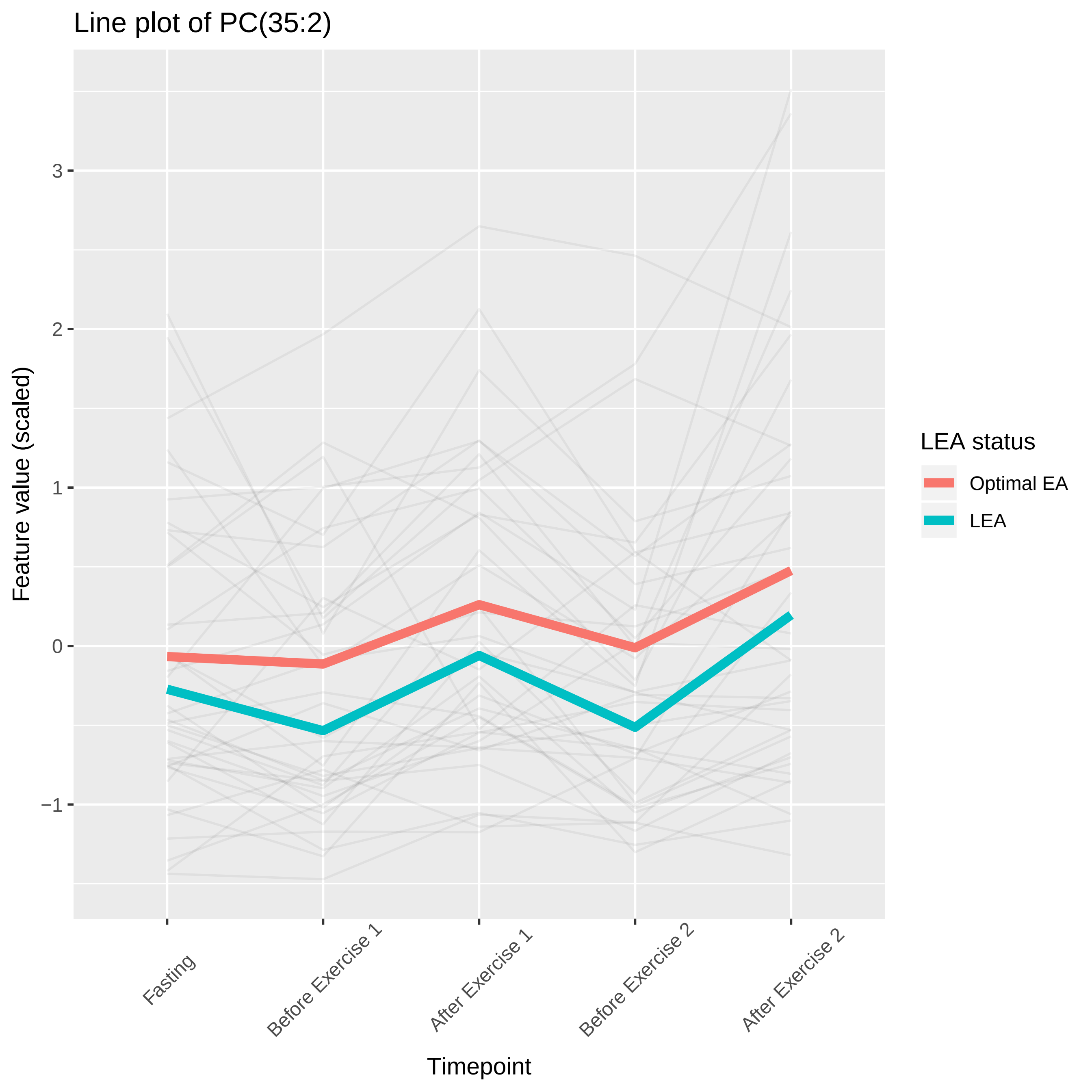

Line plot of PC(35:2)
Feature value (scaled)
3
2
1
0
−1
Fasting
Before Exercise 1
After Exercise 1
Before Exercise 2
After Exercise 2
Timepoint
LEA status
Optimal EA
LEA

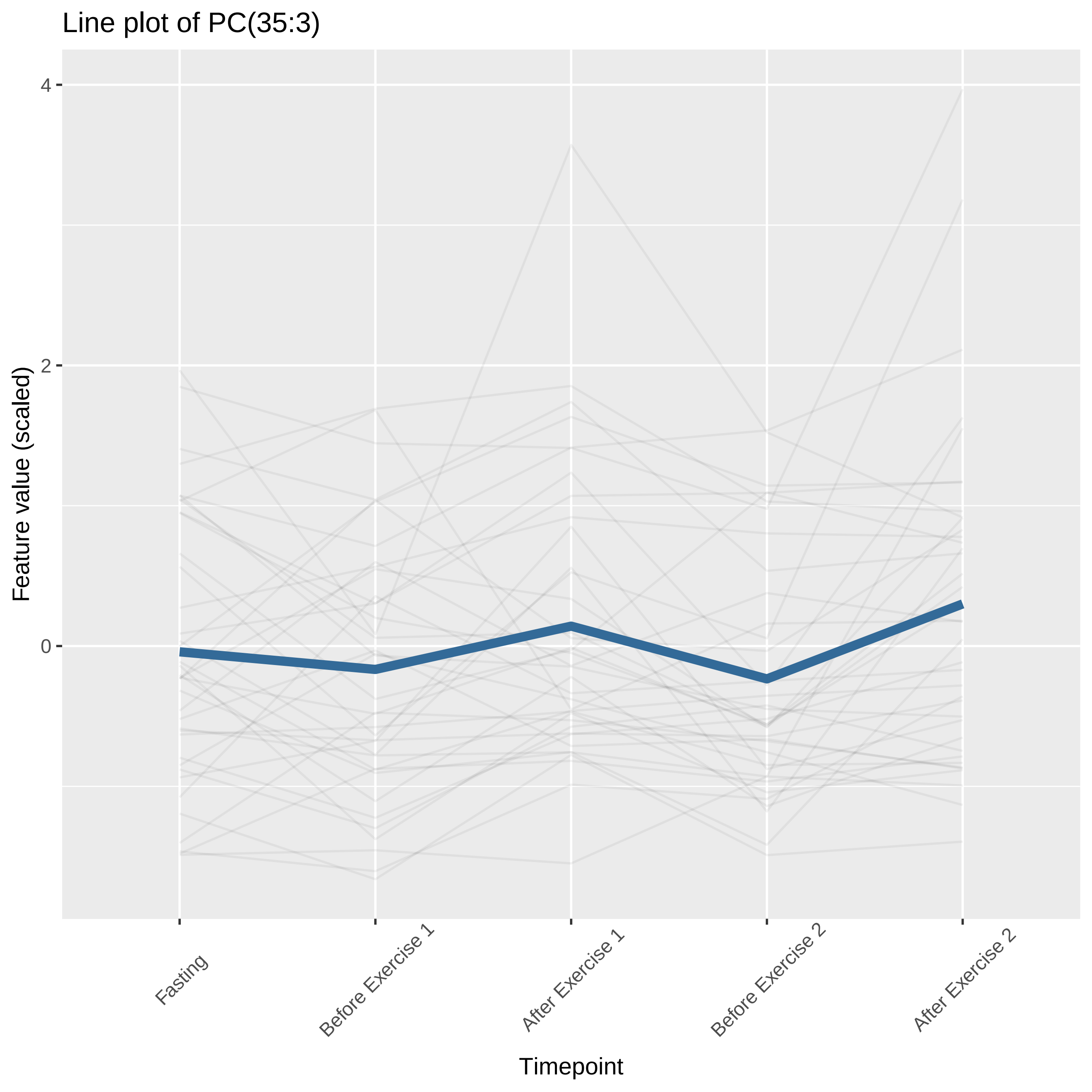

Line plot of PC(35:3)
Feature value (scaled)
4
2
0
Fasting
Before Exercise 1
After Exercise 1
Before Exercise 2
After Exercise 2
Timepoint

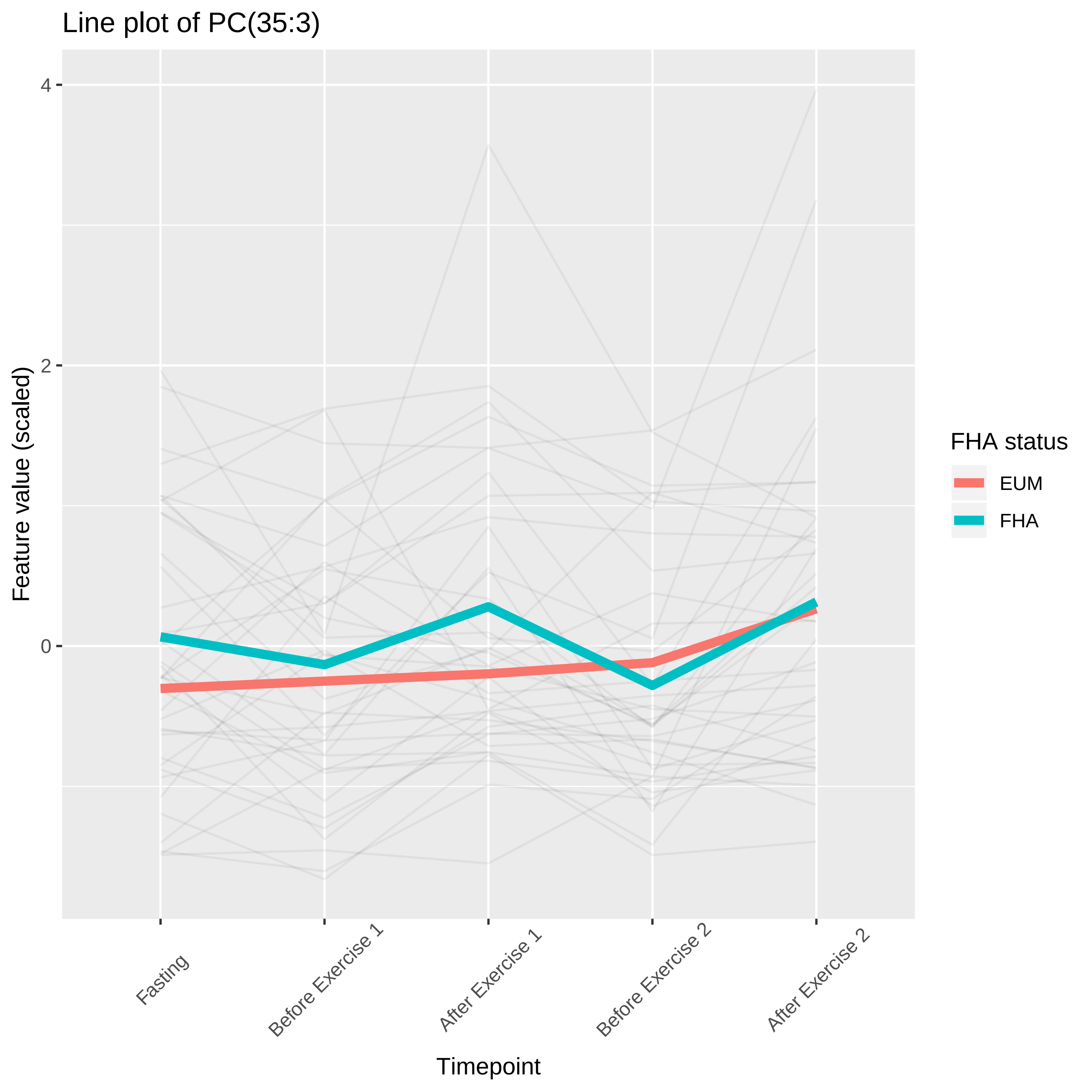
Line plot of PC(35:3)
Feature value (scaled)
4
2
0
Fasting
Before Exercise 1
After Exercise 1
Before Exercise 2
After Exercise 2
Timepoint
FHA status
EUM
FHA

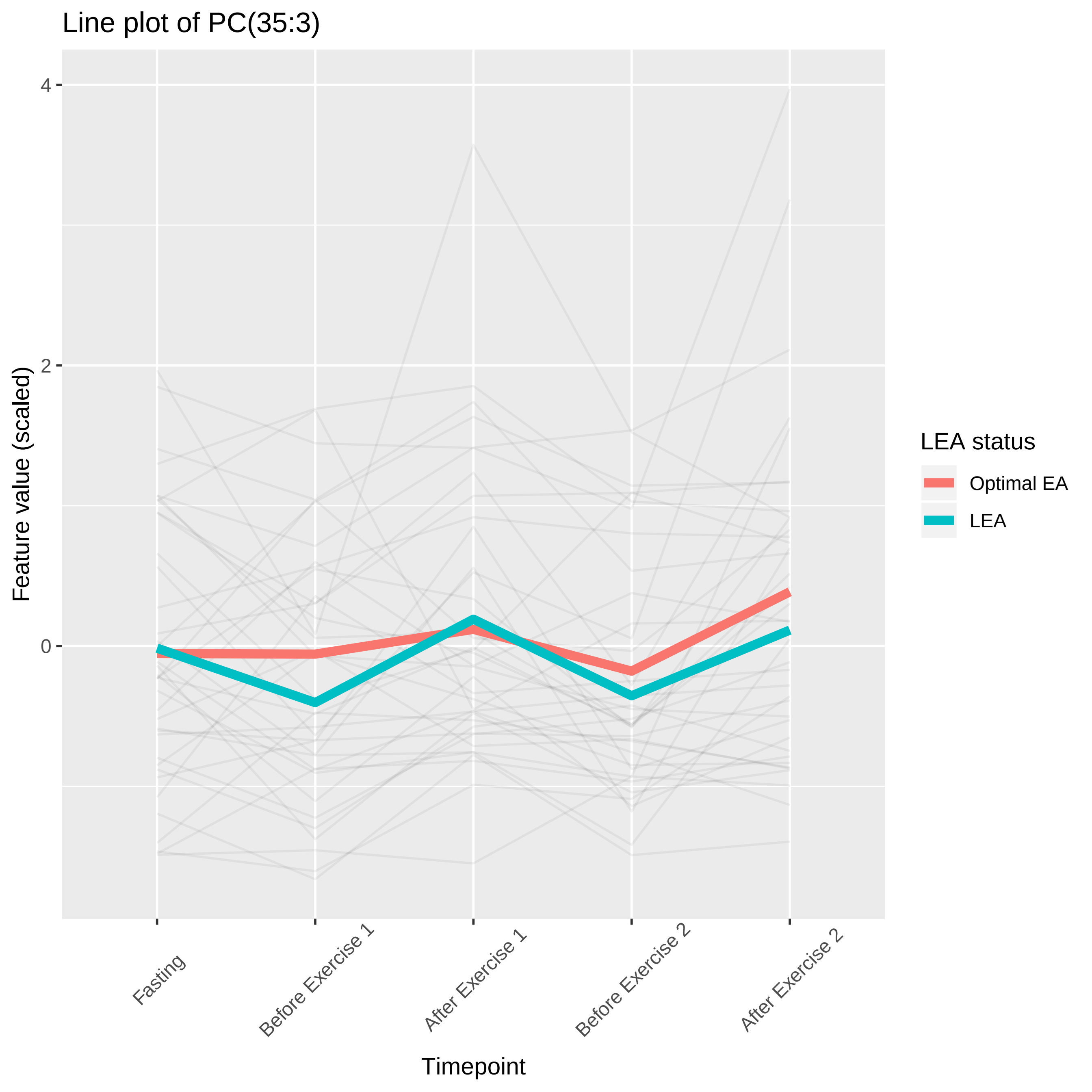

Line plot of PC(35:3)
Feature value (scaled)
4
2
0
Fasting
Before Exercise 1
After Exercise 1
Before Exercise 2
After Exercise 2
Timepoint
LEA status
Optimal EA
LEA

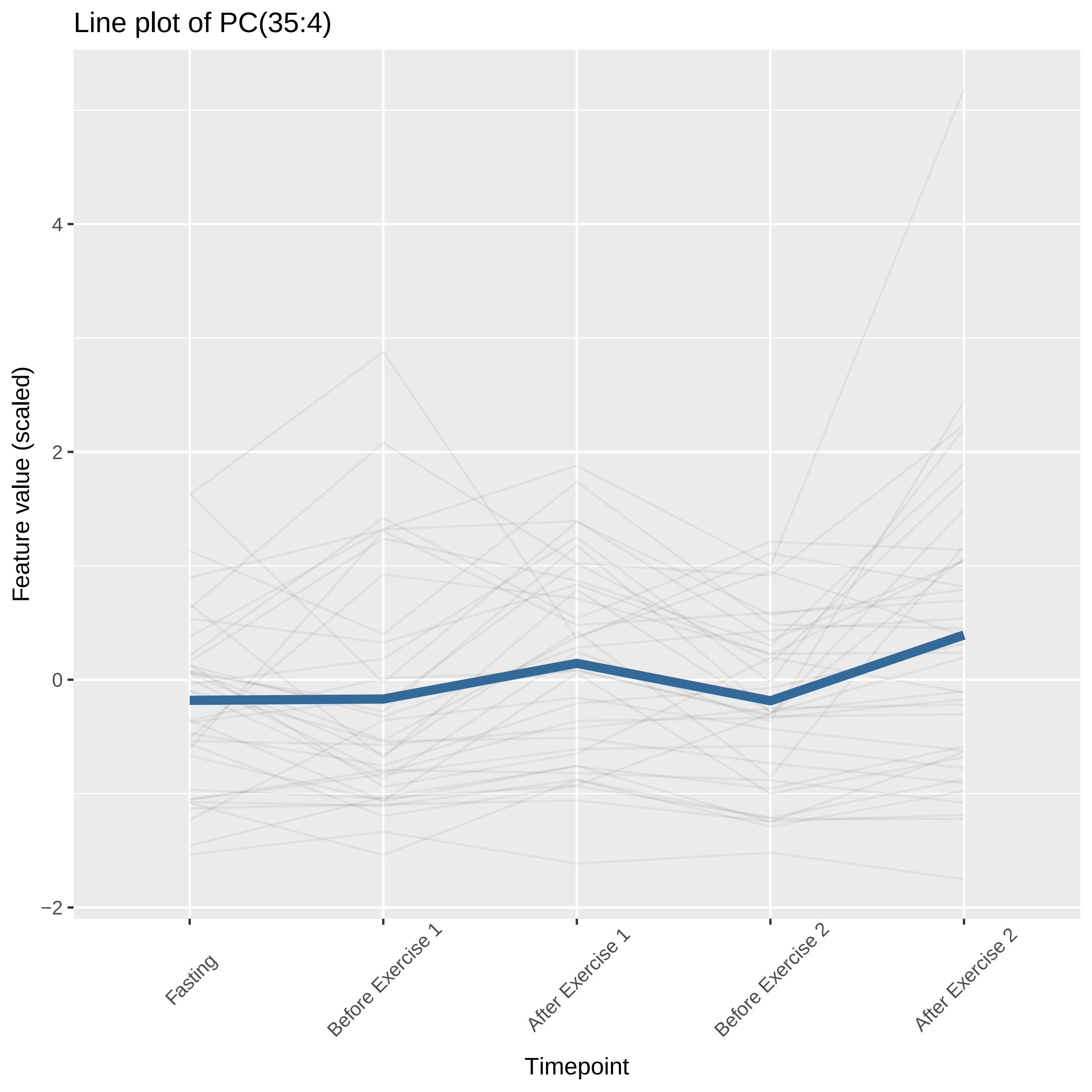
Line plot of PC(35:4)
Feature value (scaled)
4
2
0
−2
Fasting
Before Exercise 1
After Exercise 1
Before Exercise 2
After Exercise 2
Timepoint

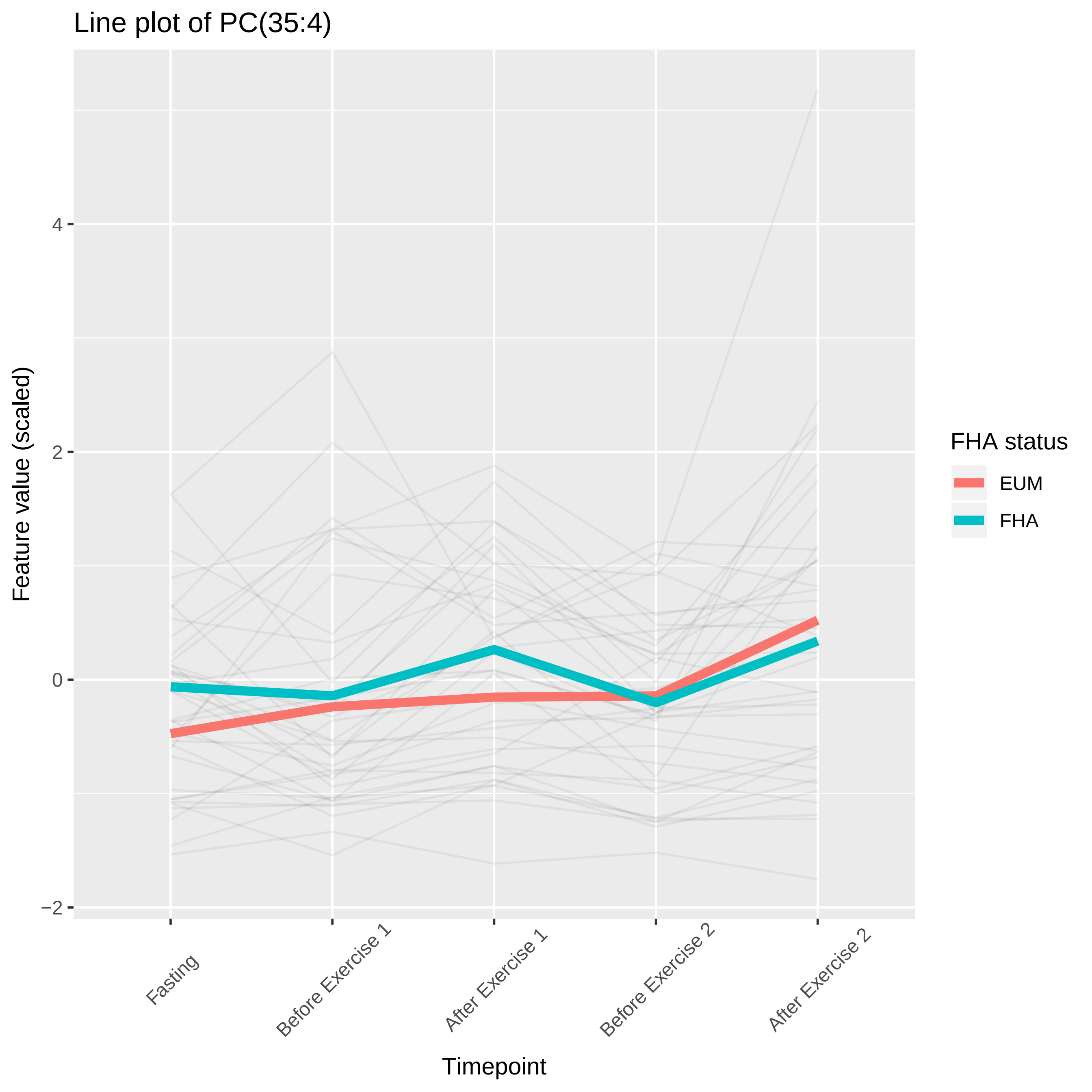

Line plot of PC(35:4)
Feature value (scaled)
4
2
0
−2
Fasting
Before Exercise 1
After Exercise 1
Before Exercise 2
After Exercise 2
Timepoint
FHA status
EUM
FHA

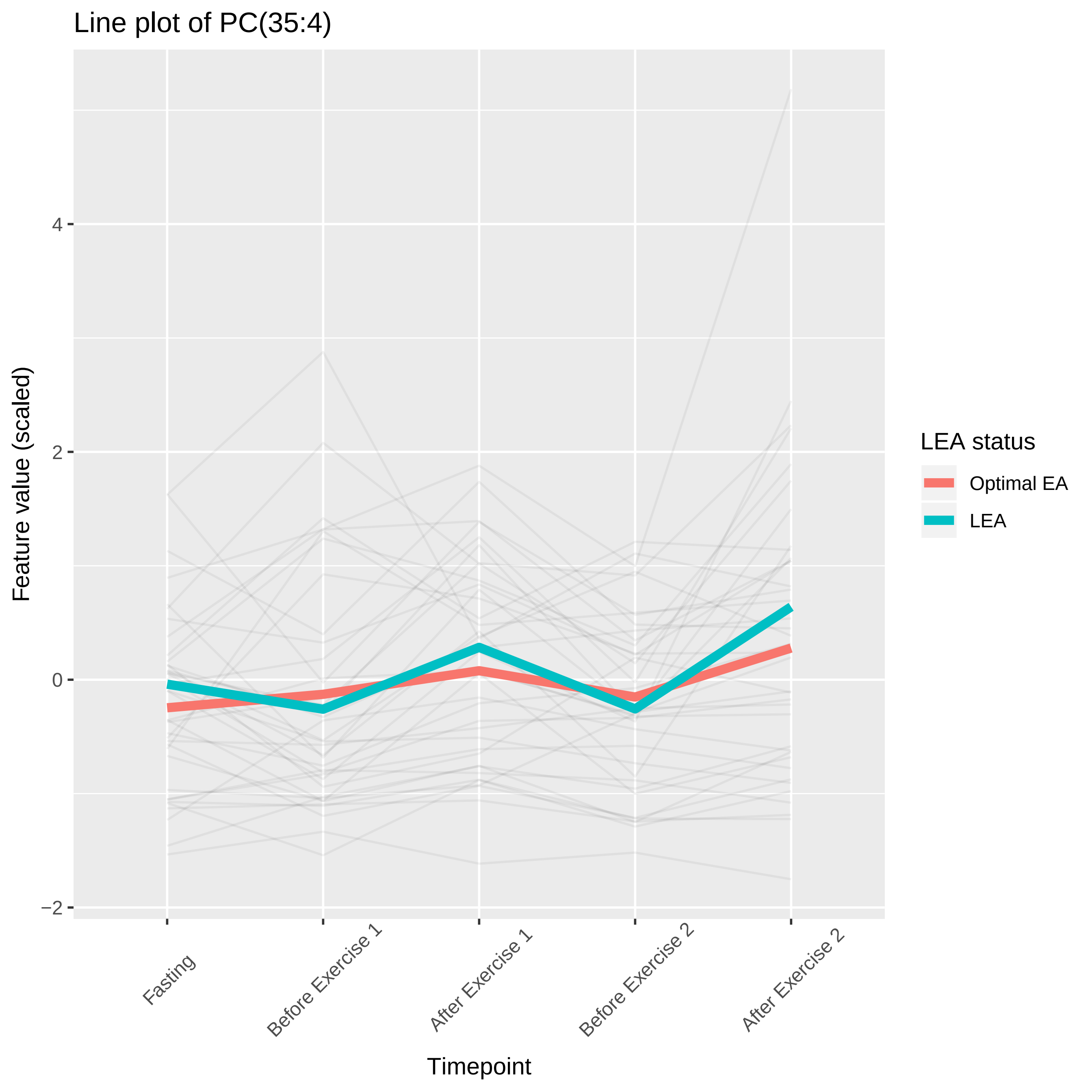

Line plot of PC(35:4)
Feature value (scaled)
4
2
0
−2
Fasting
Before Exercise 1
After Exercise 1
Before Exercise 2
After Exercise 2
Timepoint
LEA status
Optimal EA
LEA

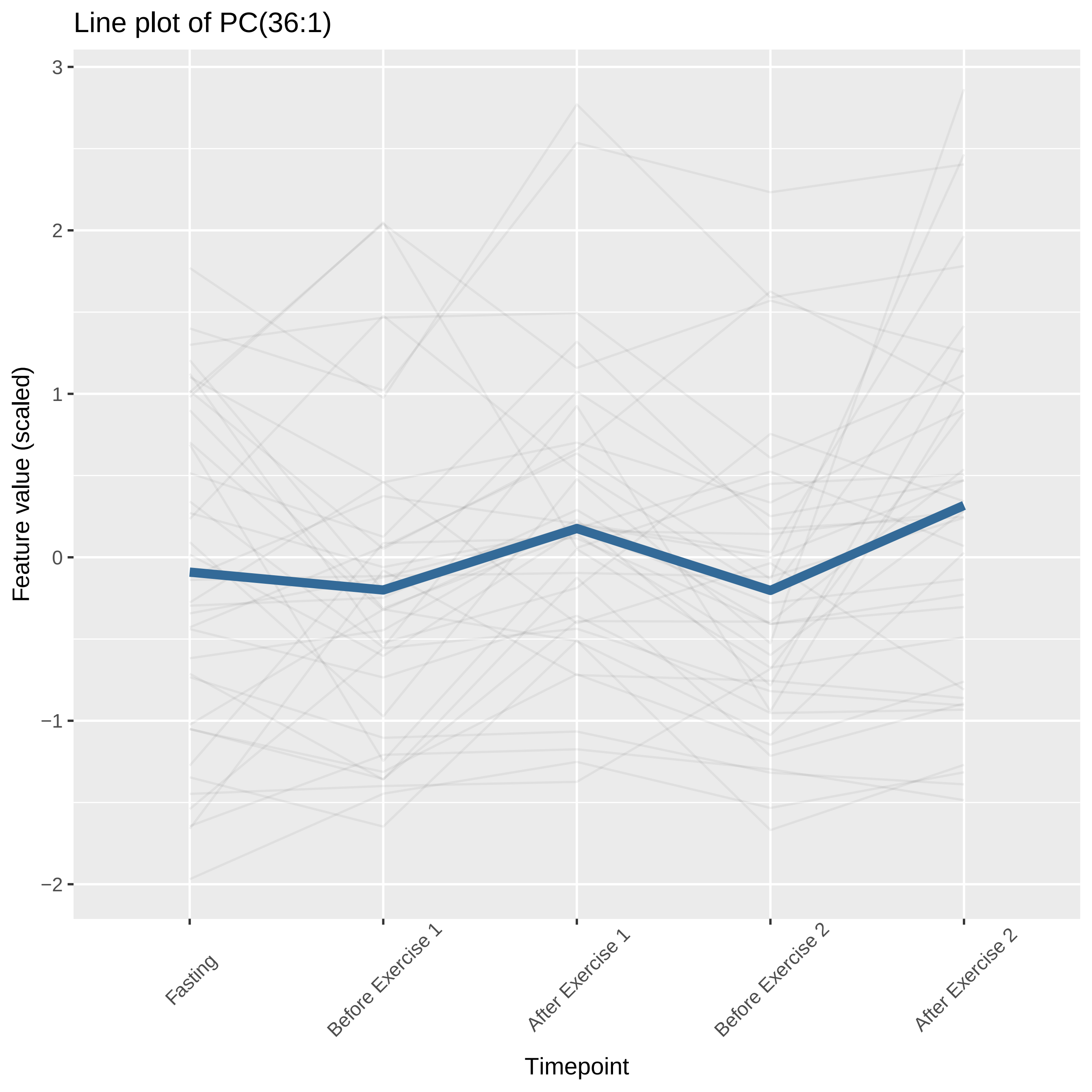
Line plot of PC(36:1)
Feature value (scaled)
3
2
1
0
−1
−2
Fasting
Before Exercise 1
After Exercise 1
Before Exercise 2
After Exercise 2
Timepoint

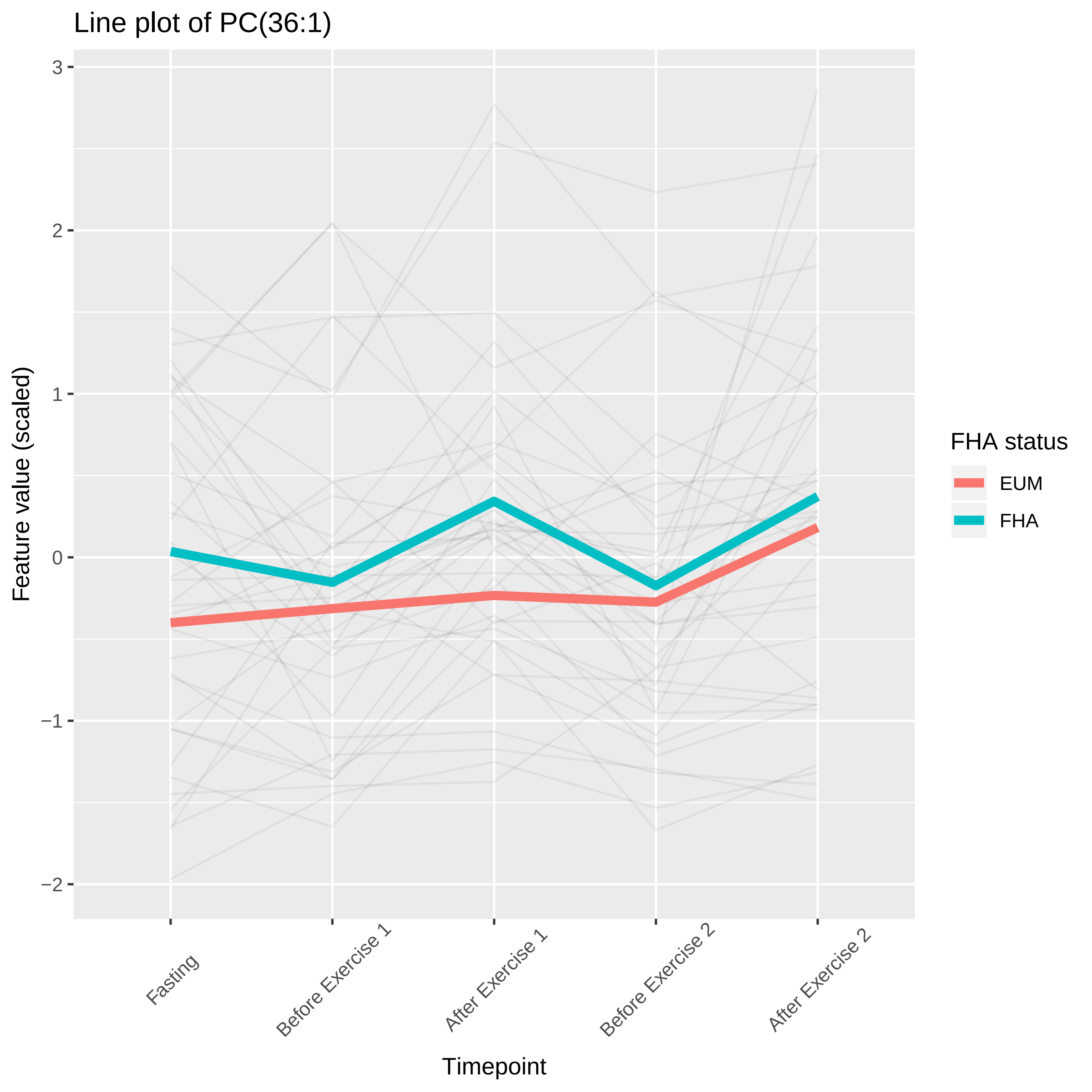
Line plot of PC(36:1)
Feature value (scaled)
3
2
1
0
−1
−2
Fasting
Before Exercise 1
After Exercise 1
Before Exercise 2
After Exercise 2
Timepoint
FHA status
EUM
FHA

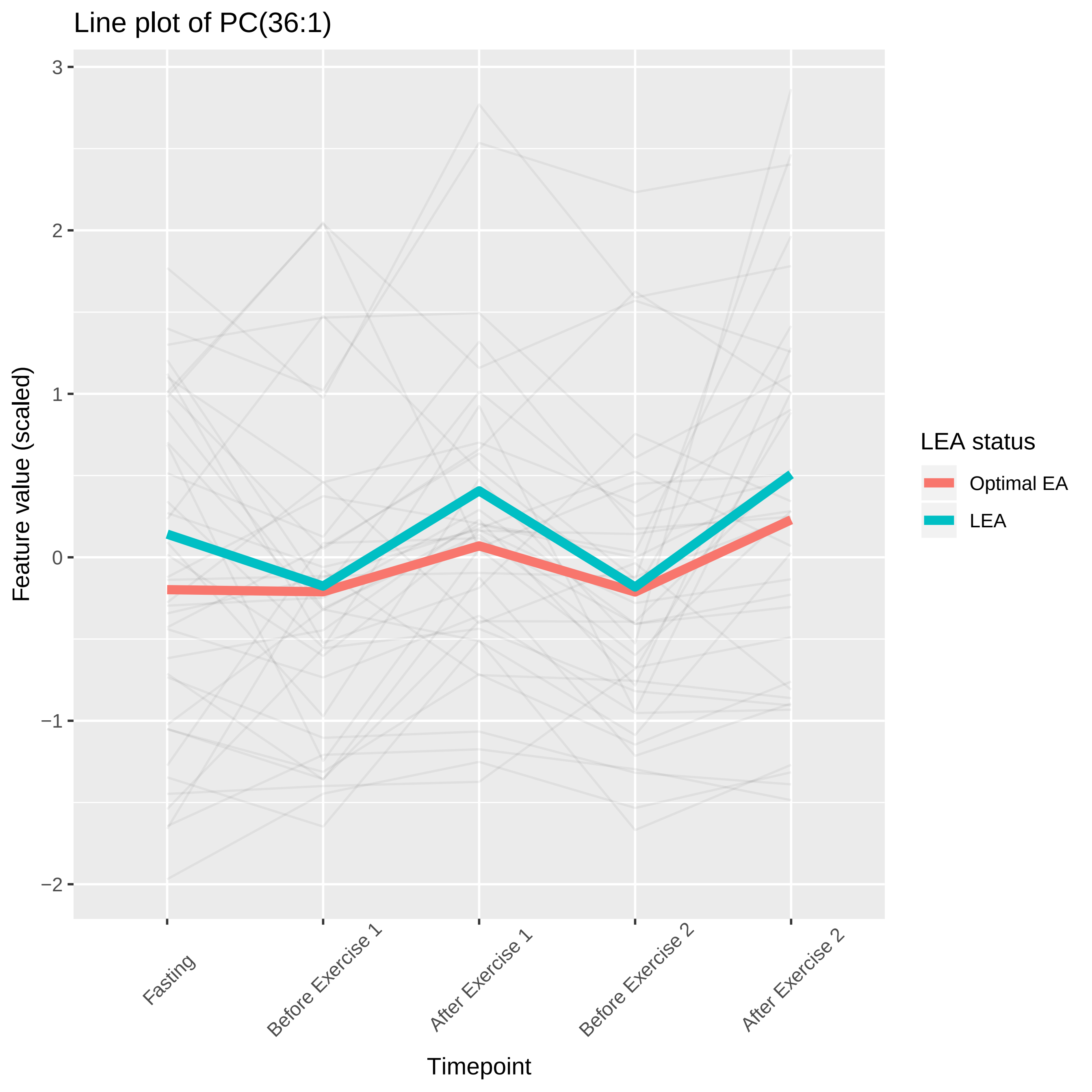
Line plot of PC(36:1)
Feature value (scaled)
3
2
1
0
−1
−2
Fasting
Before Exercise 1
After Exercise 1
Before Exercise 2
After Exercise 2
Timepoint
LEA status
Optimal EA
LEA

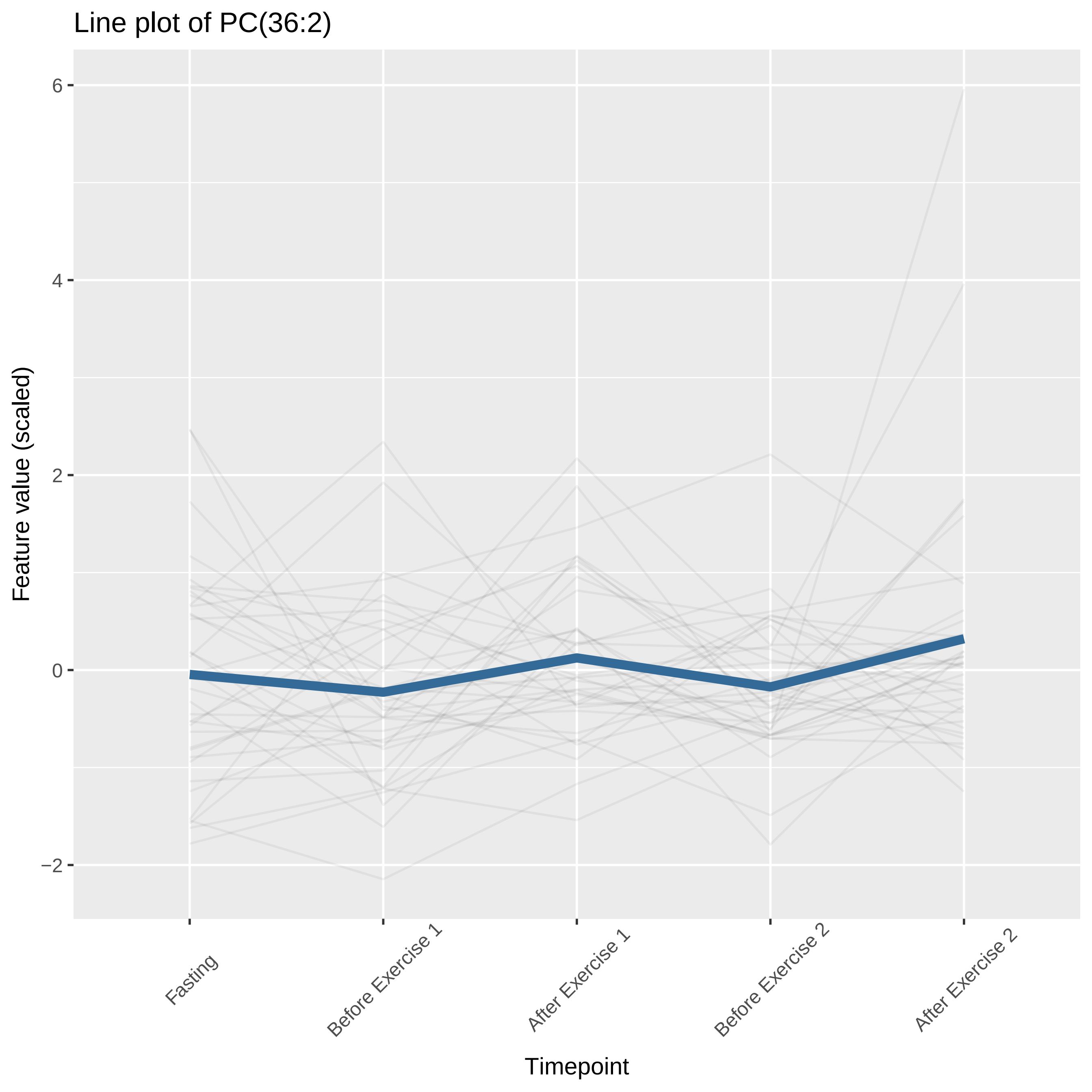

Line plot of PC(36:2)
Feature value (scaled)
6
4
2
0
−2
Fasting
Before Exercise 1
After Exercise 1
Before Exercise 2
After Exercise 2
Timepoint

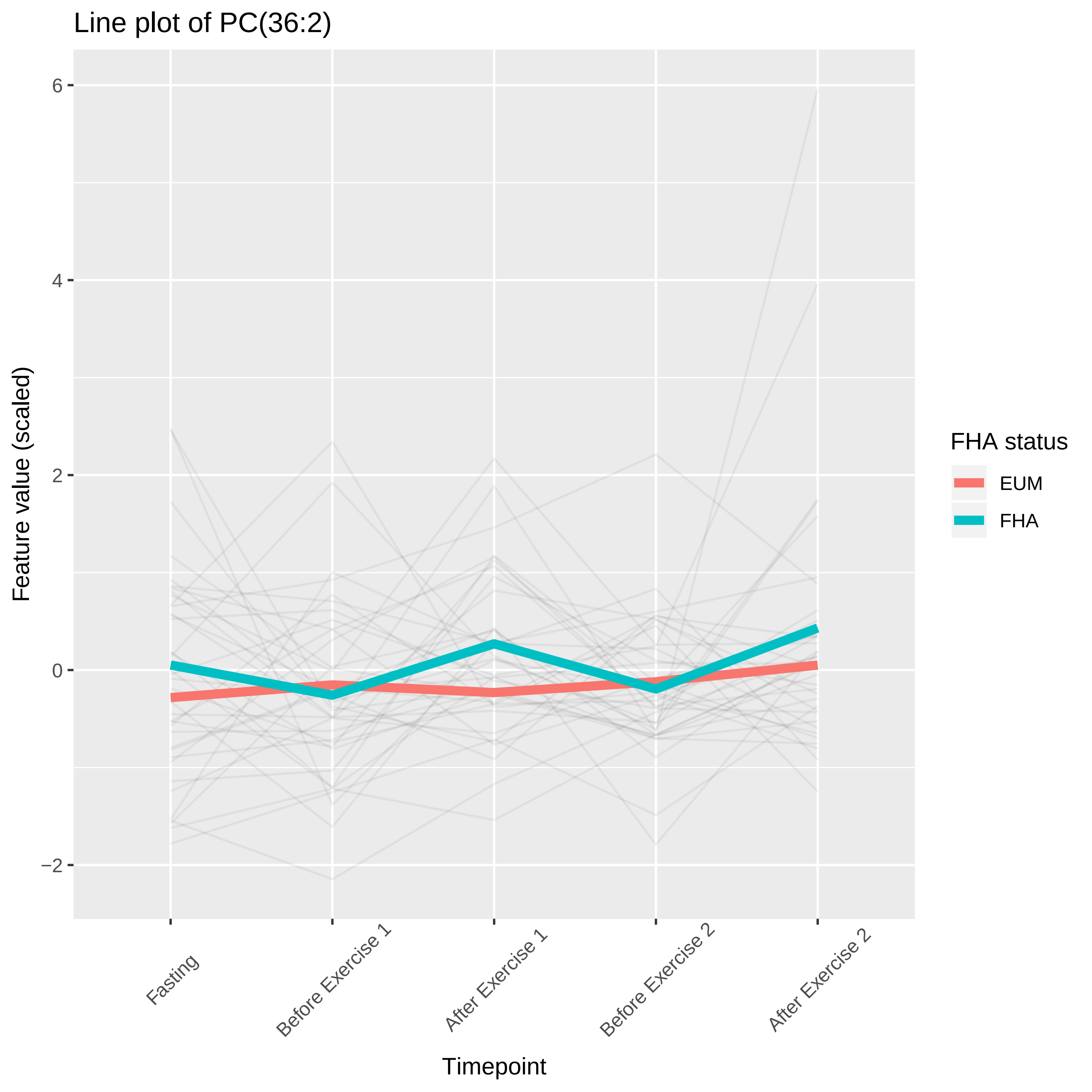

Line plot of PC(36:2)
Feature value (scaled)
6
4
2
0
−2
Fasting
Before Exercise 1
After Exercise 1
Before Exercise 2
After Exercise 2
Timepoint
FHA status
EUM
FHA

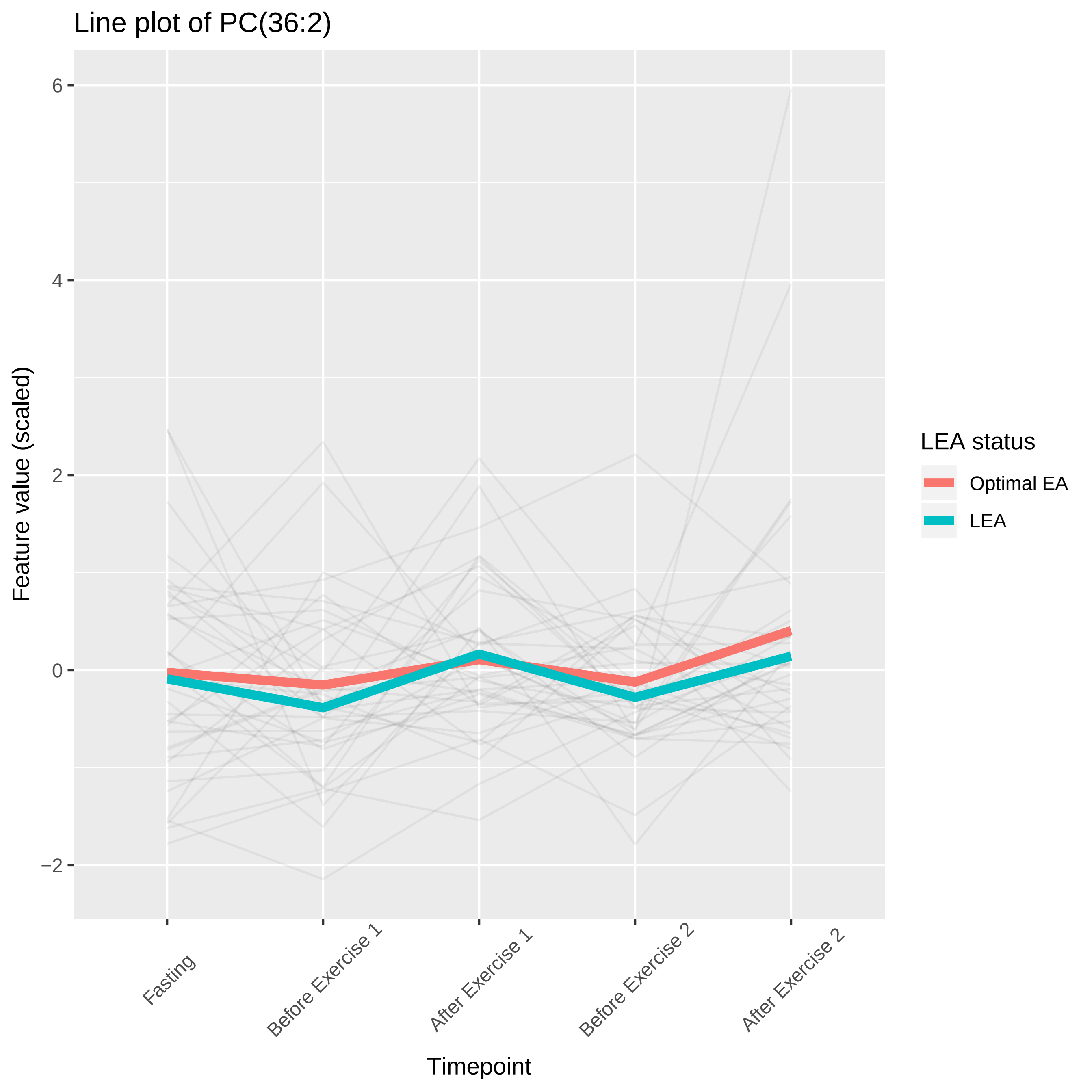

Line plot of PC(36:2)
Feature value (scaled)
6
4
2
0
−2
Fasting
Before Exercise 1
After Exercise 1
Before Exercise 2
After Exercise 2
Timepoint
LEA status
Optimal EA
LEA

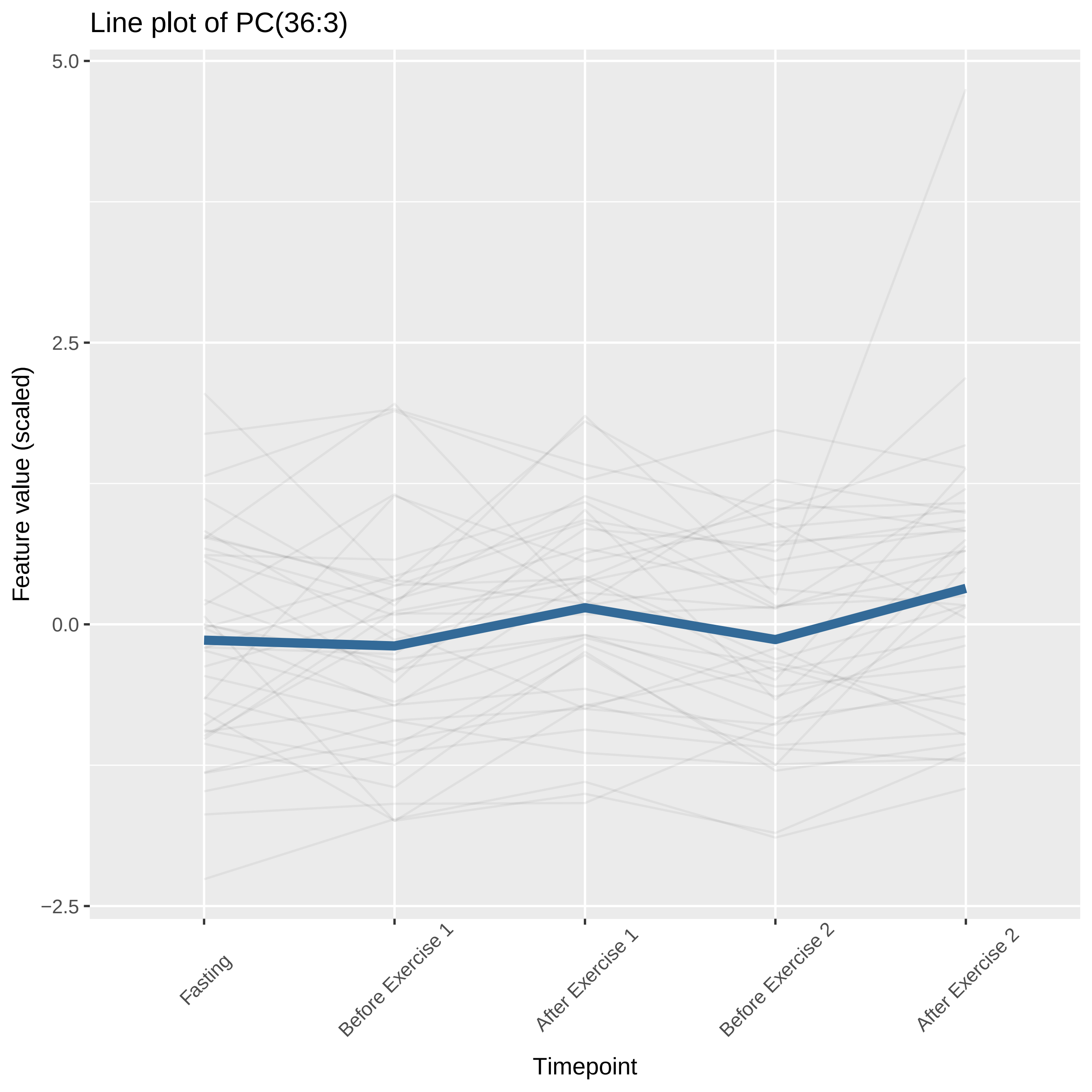

Line plot of PC(36:3)
Feature value (scaled)
5.0
2.5
0.0
−2.5
Fasting
Before Exercise 1
After Exercise 1
Before Exercise 2
After Exercise 2
Timepoint

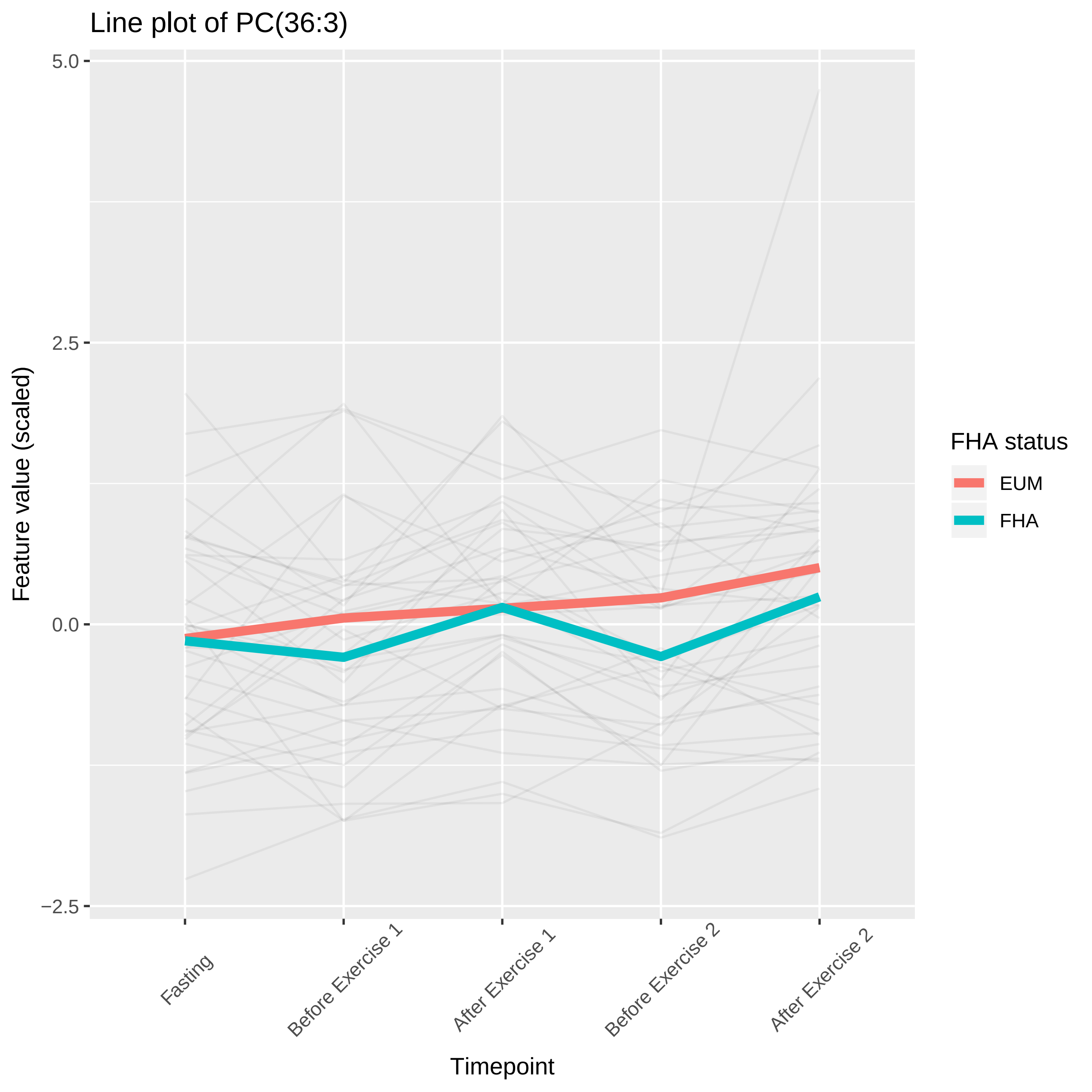

Line plot of PC(36:3)
Feature value (scaled)
5.0
2.5
0.0
−2.5
Fasting
Before Exercise 1
After Exercise 1
Before Exercise 2
After Exercise 2
Timepoint
FHA status
EUM
FHA

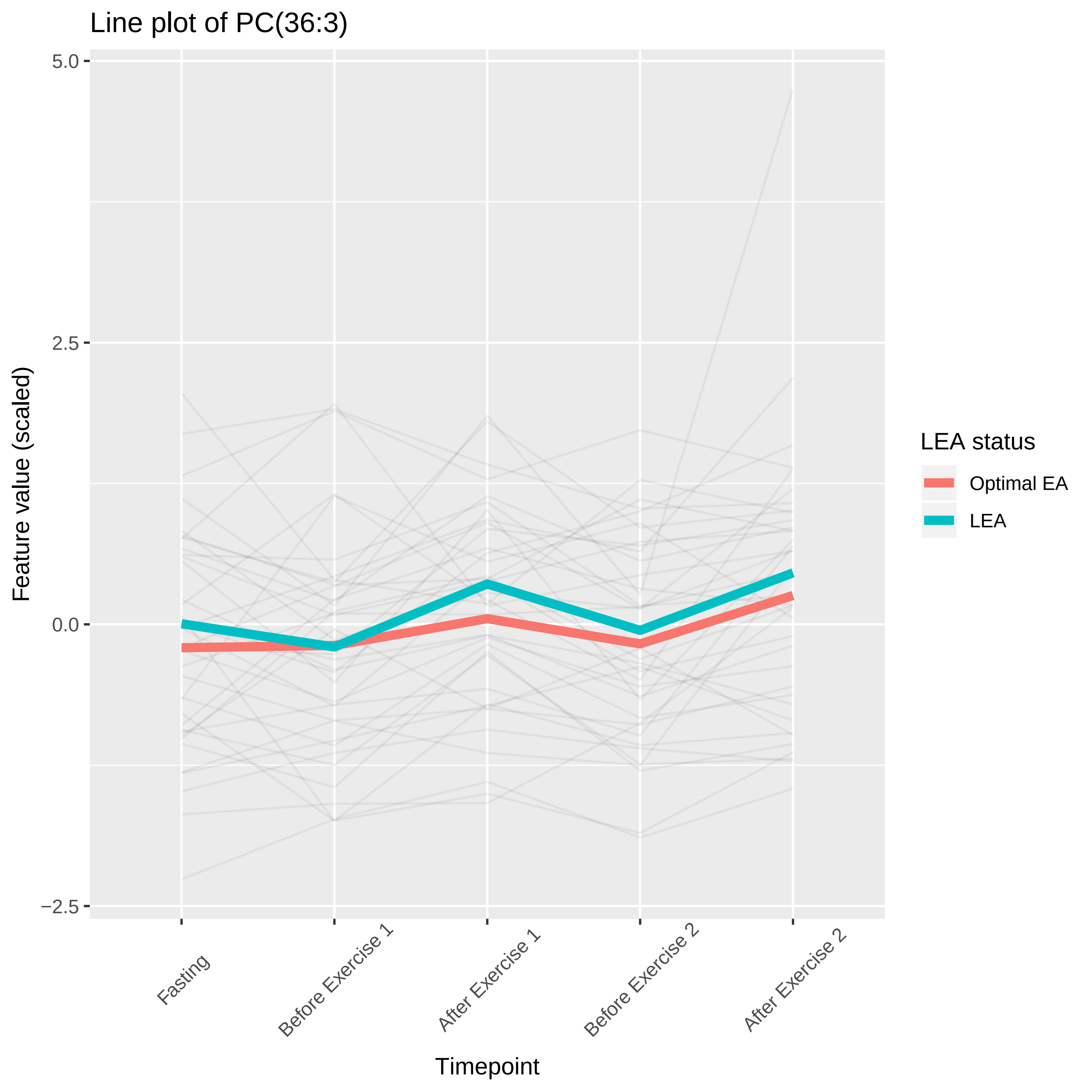

Line plot of PC(36:3)
5.0
2.5
0.0
−2.5
Feature value (scaled)
Fasting
Before Exercise 1
After Exercise 1
Before Exercise 2
After Exercise 2
Timepoint
LEA status
Optimal EA
LEA

Line plot of PC(36:4)

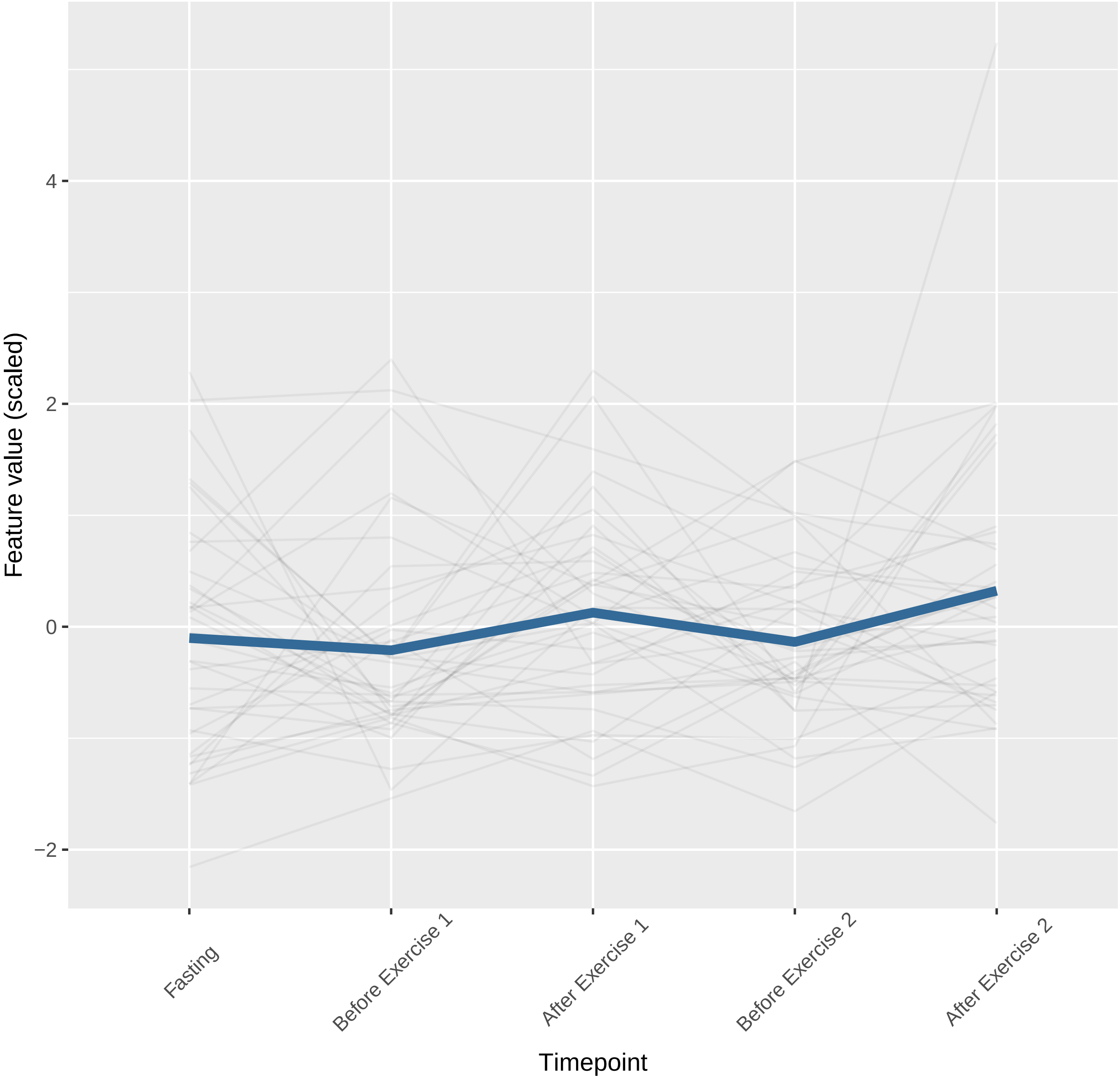

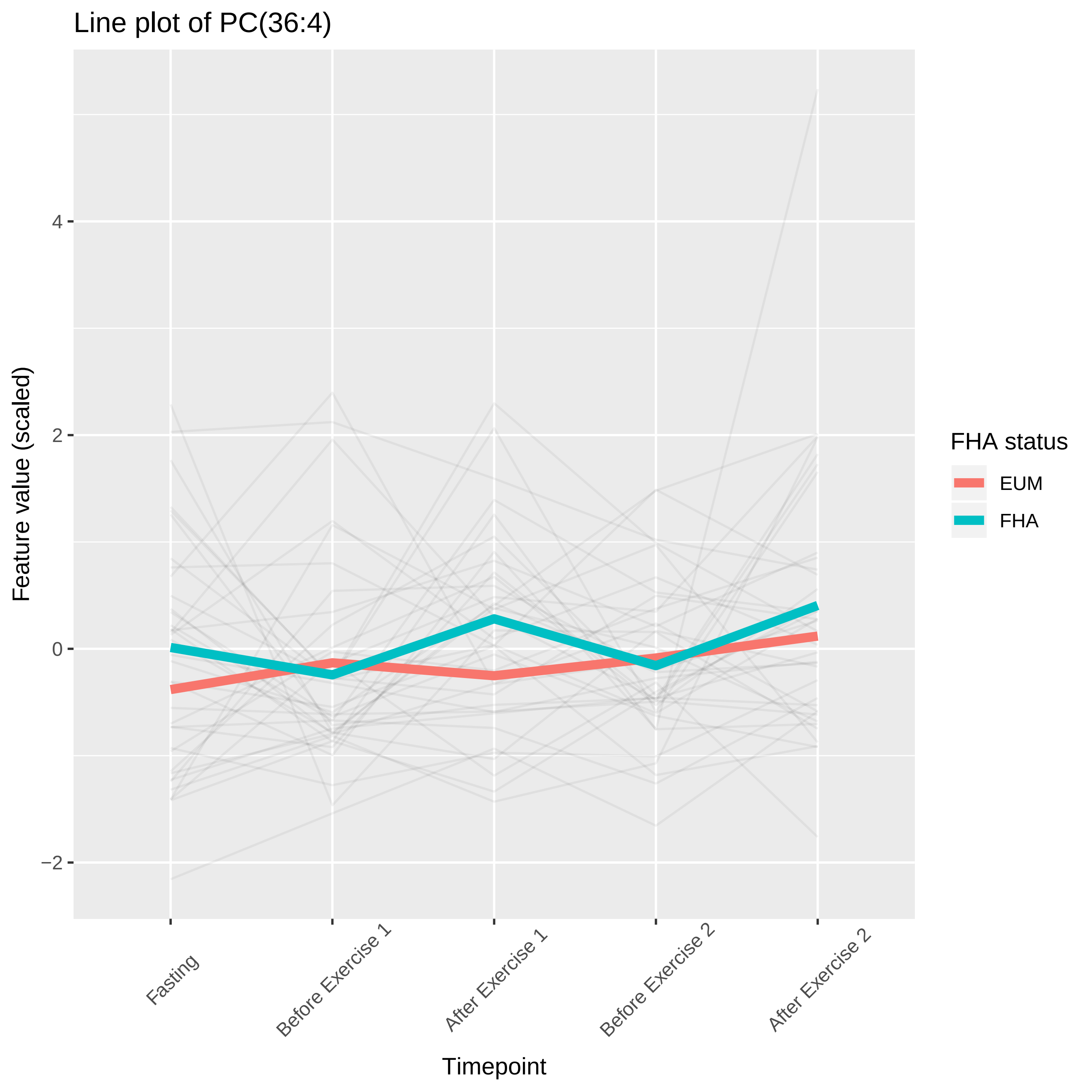

Line plot of PC(36:4)
Feature value (scaled)
4
2
0
−2
Fasting
Before Exercise 1
After Exercise 1
Before Exercise 2
After Exercise 2
Timepoint
FHA status
EUM
FHA

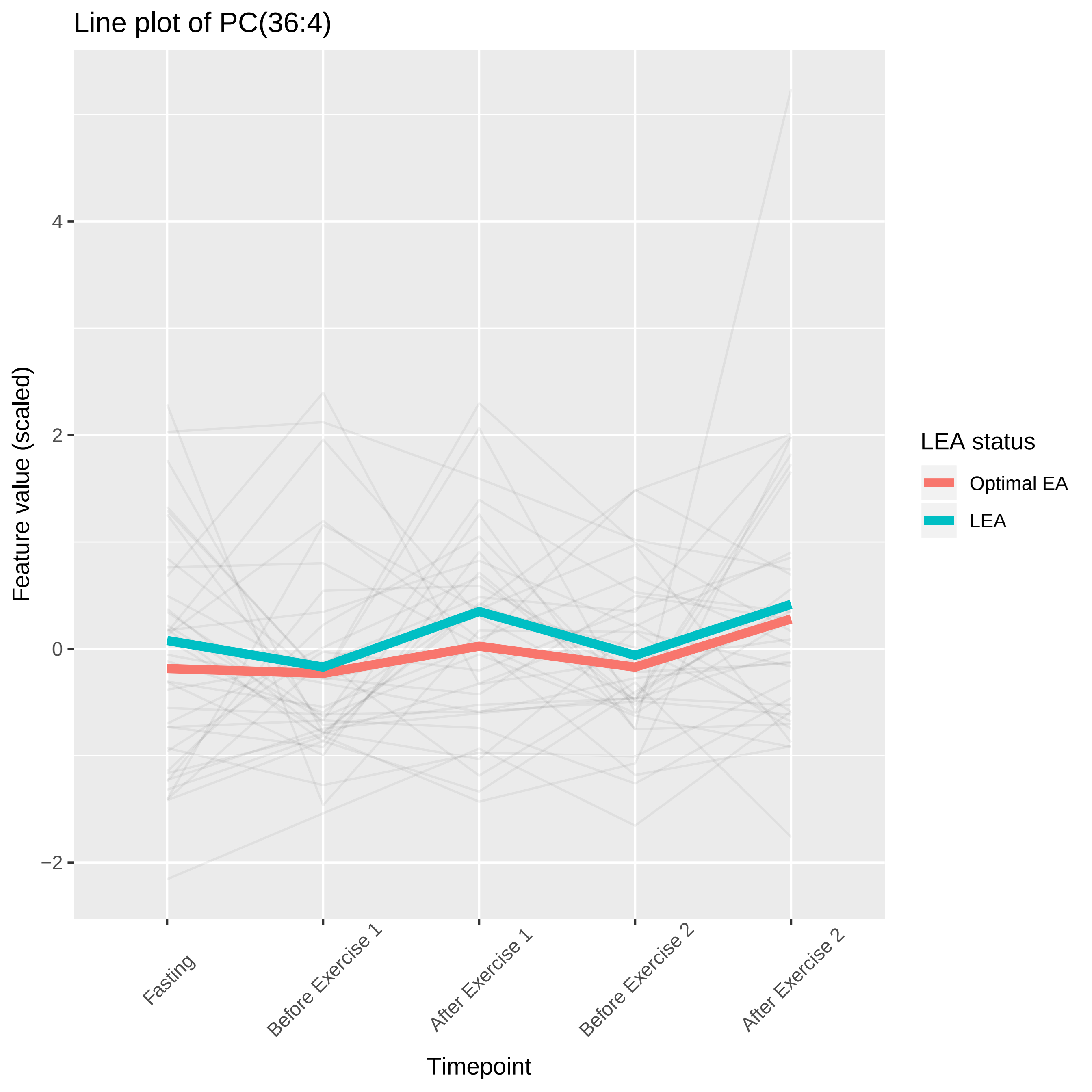

Line plot of PC(36:4)
Feature value (scaled)
4
2
0
−2
Fasting
Before Exercise 1
After Exercise 1
Before Exercise 2
After Exercise 2
Timepoint
LEA status
Optimal EA
LEA

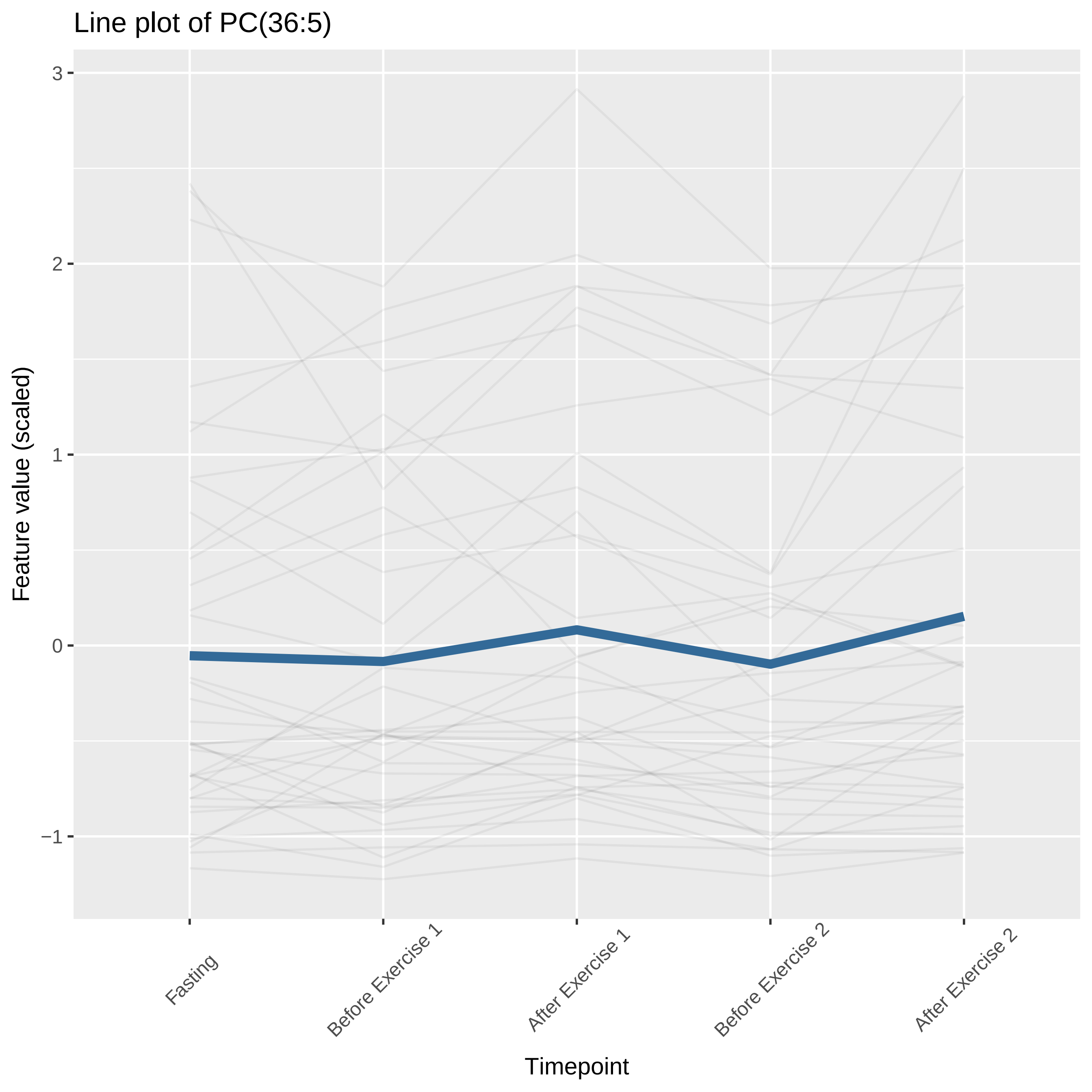

Line plot of PC(36:5)
Feature value (scaled)
3
2
1
0
−1
Fasting
Before Exercise 1
After Exercise 1
Before Exercise 2
After Exercise 2
Timepoint

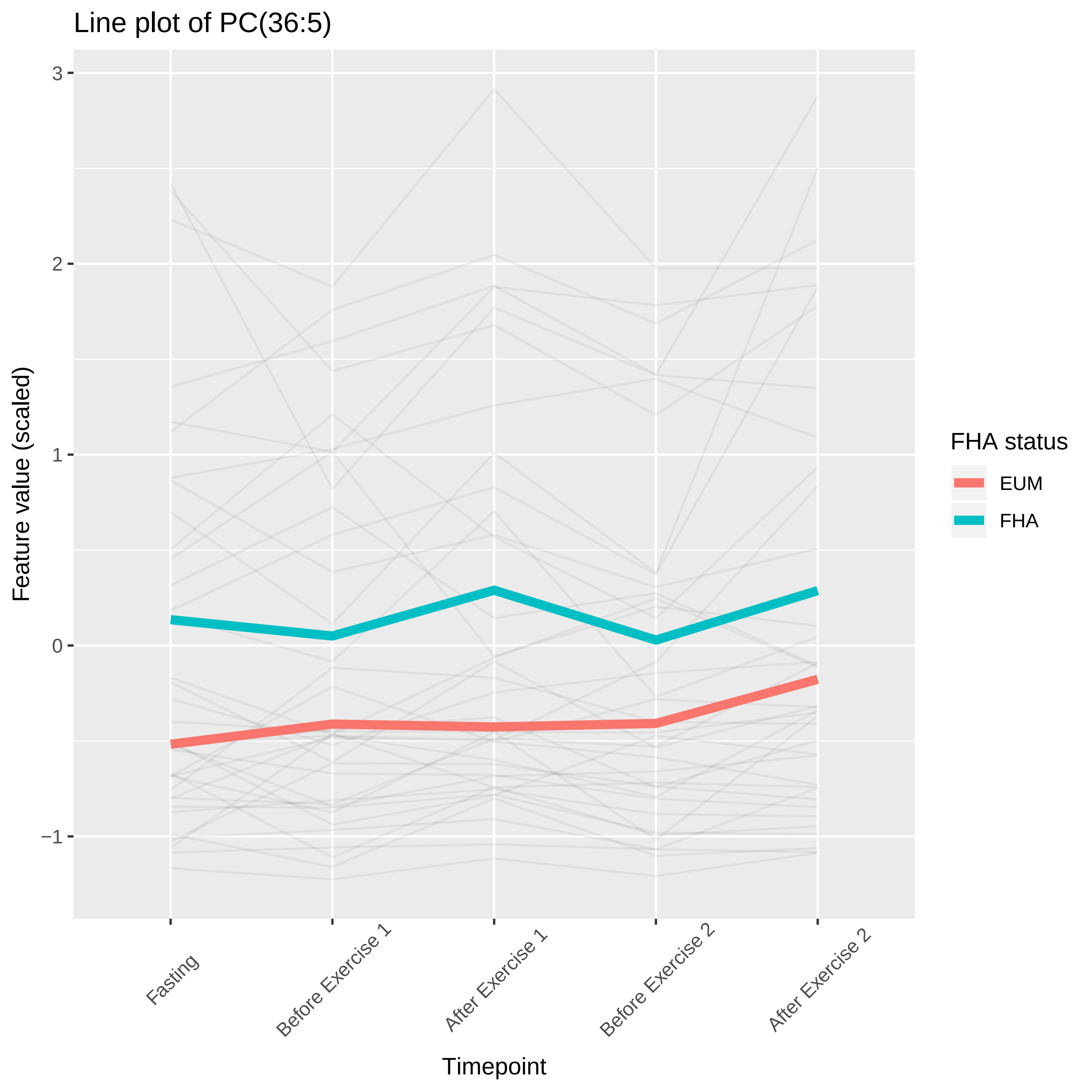
Line plot of PC(36:5)
Feature value (scaled)
3
2
1
0
−1
Fasting
Before Exercise 1
After Exercise 1
Before Exercise 2
After Exercise 2
Timepoint
FHA status
EUM
FHA

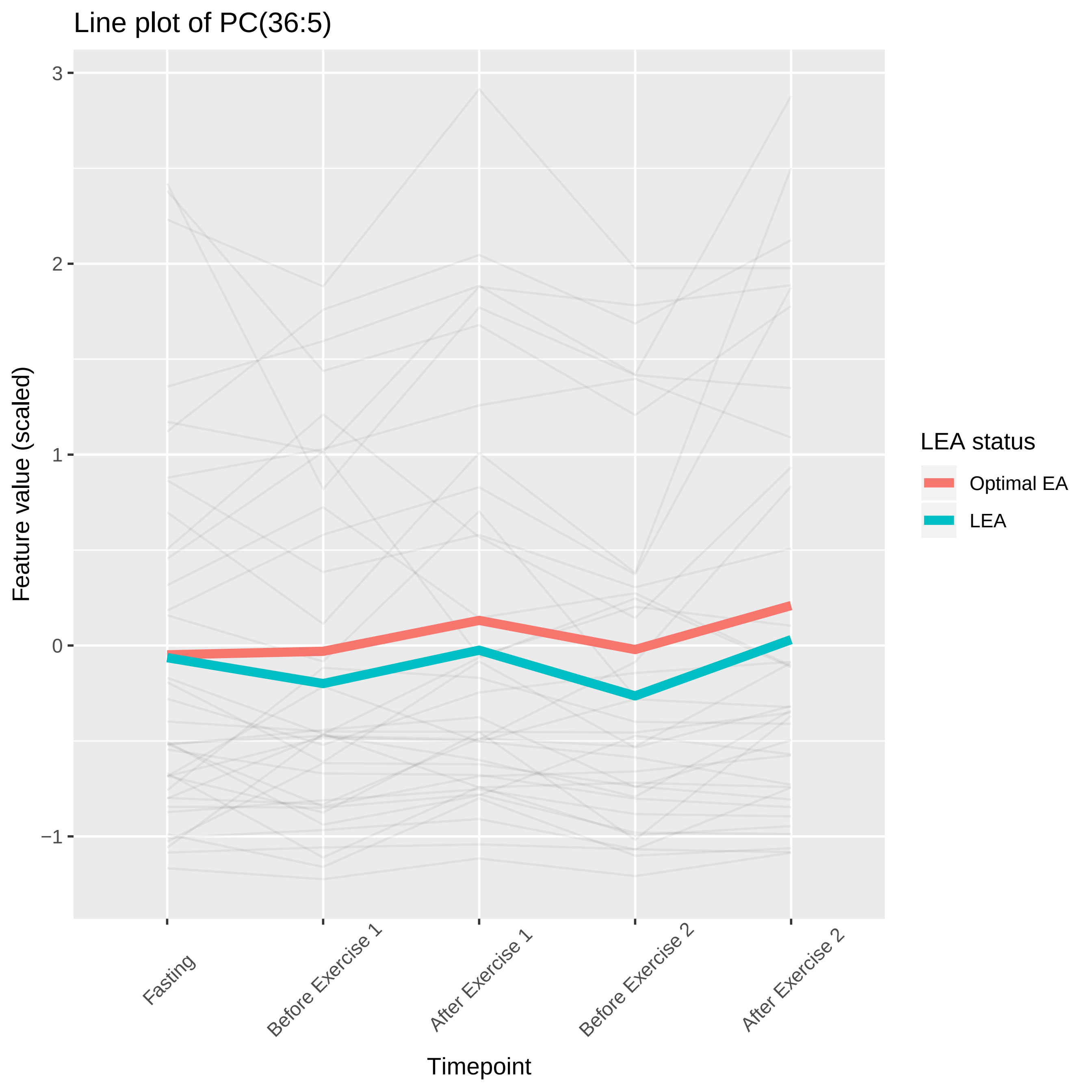

Line plot of PC(36:5)
Feature value (scaled)
3
2
1
0
−1
Fasting
Before Exercise 1
After Exercise 1
Before Exercise 2
After Exercise 2
Timepoint
LEA status
Optimal EA
LEA

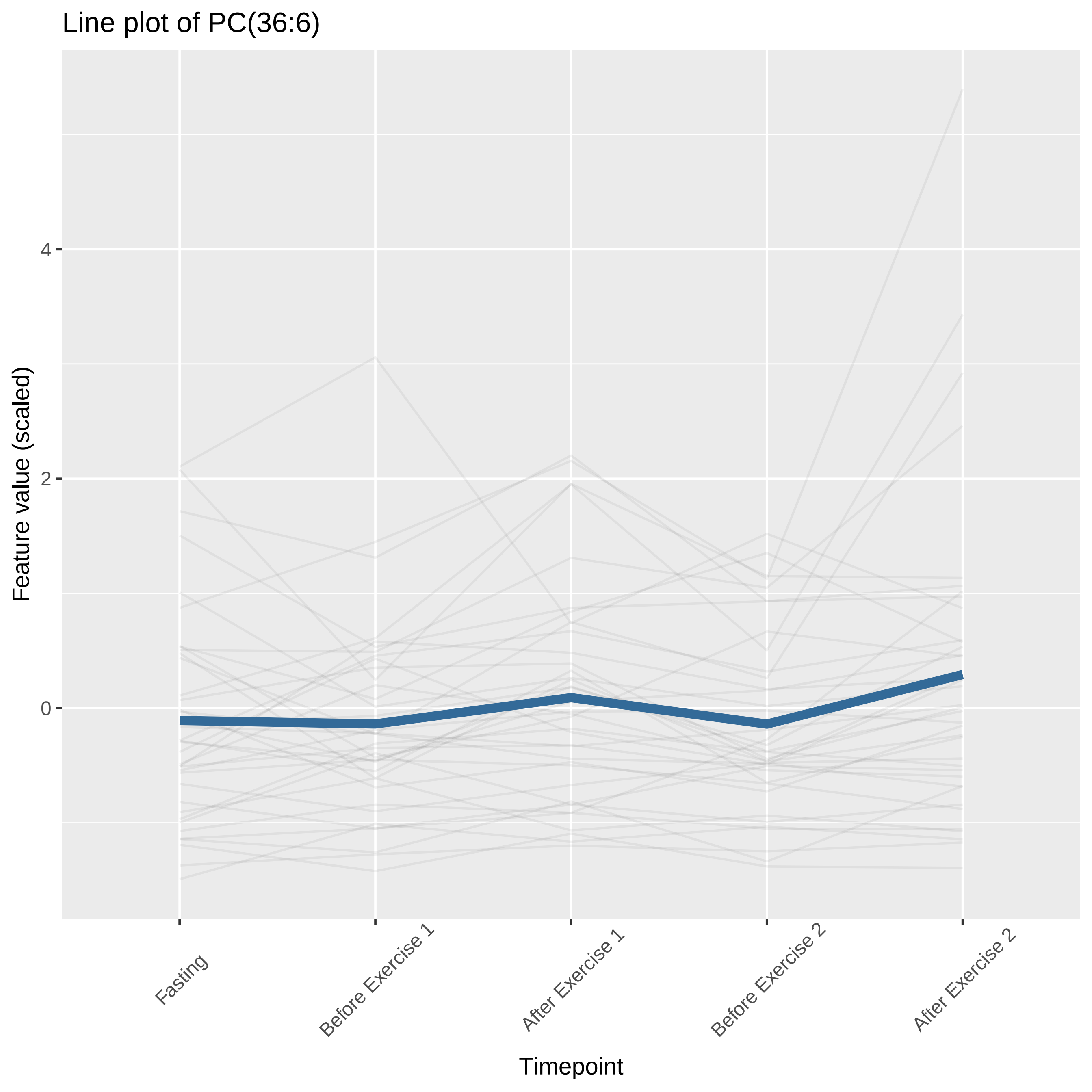
Line plot of PC(36:6)
Feature value (scaled)
4
2
0
Fasting
Before Exercise 1
After Exercise 1
Before Exercise 2
After Exercise 2
Timepoint

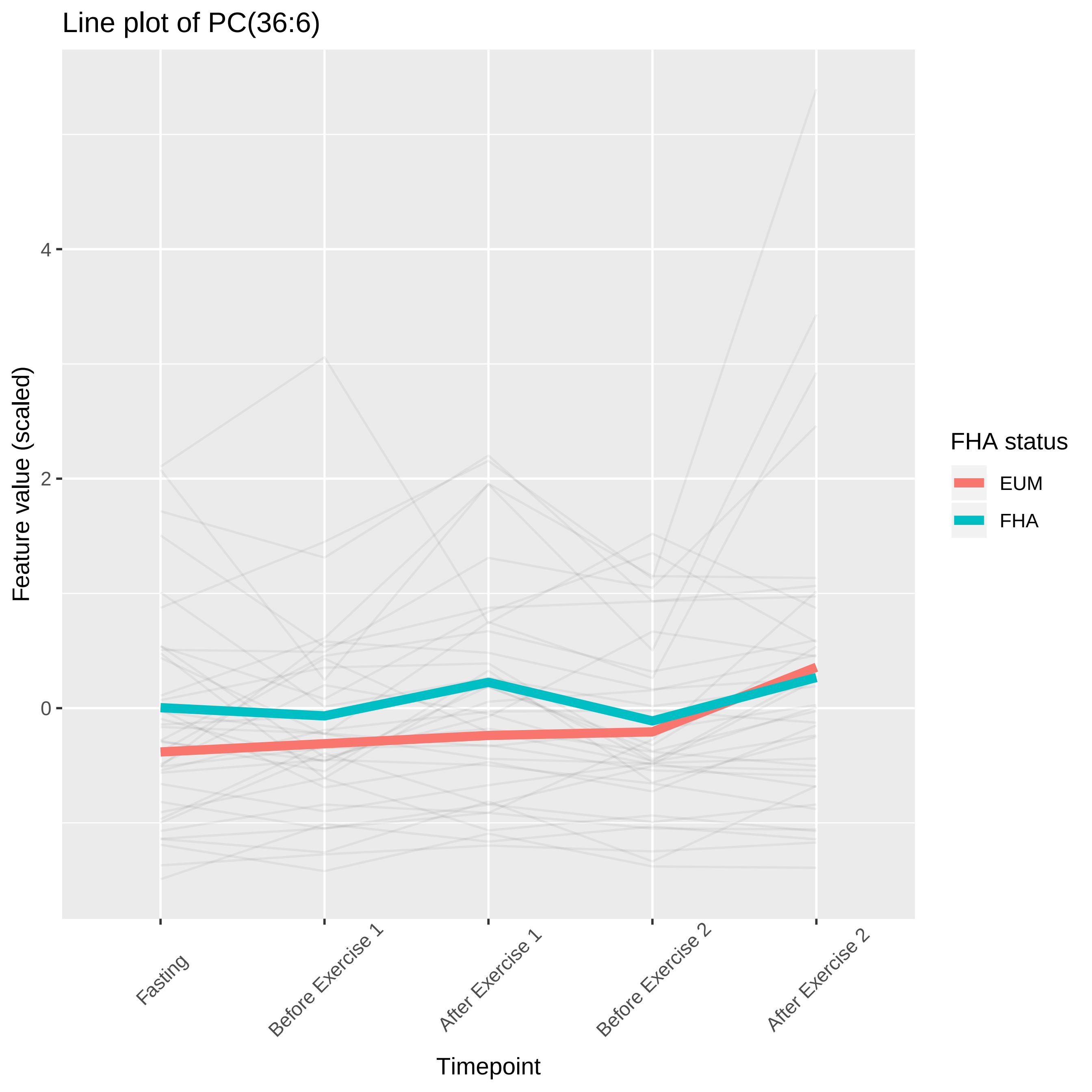

Line plot of PC(36:6)
Feature value (scaled)
4
2
0
Fasting
Before Exercise 1
After Exercise 1
Before Exercise 2
After Exercise 2
Timepoint
FHA status
EUM
FHA

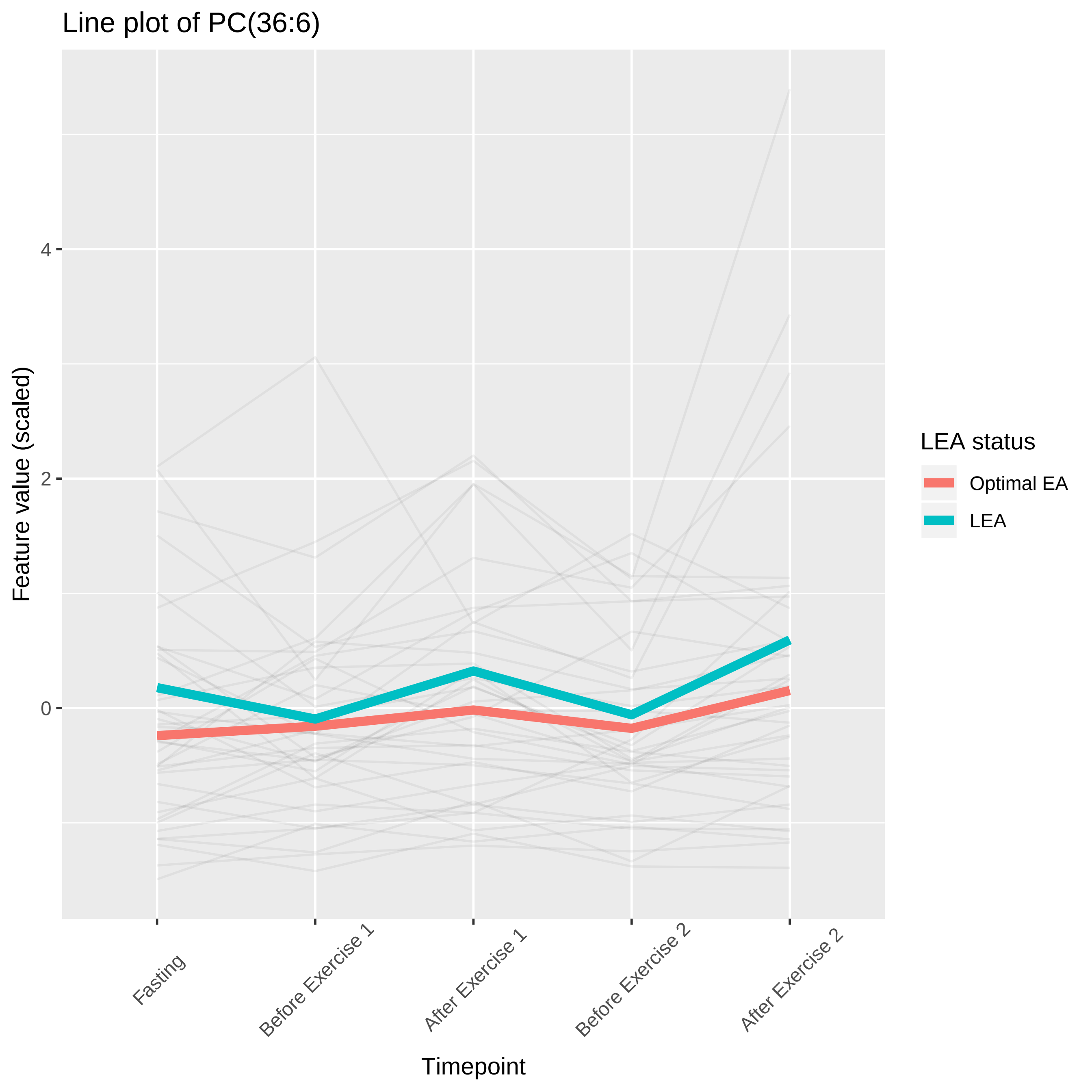
Line plot of PC(36:6)
Feature value (scaled)
4
2
0
Fasting
Before Exercise 1
After Exercise 1
Before Exercise 2
After Exercise 2
Timepoint
LEA status
Optimal EA
LEA

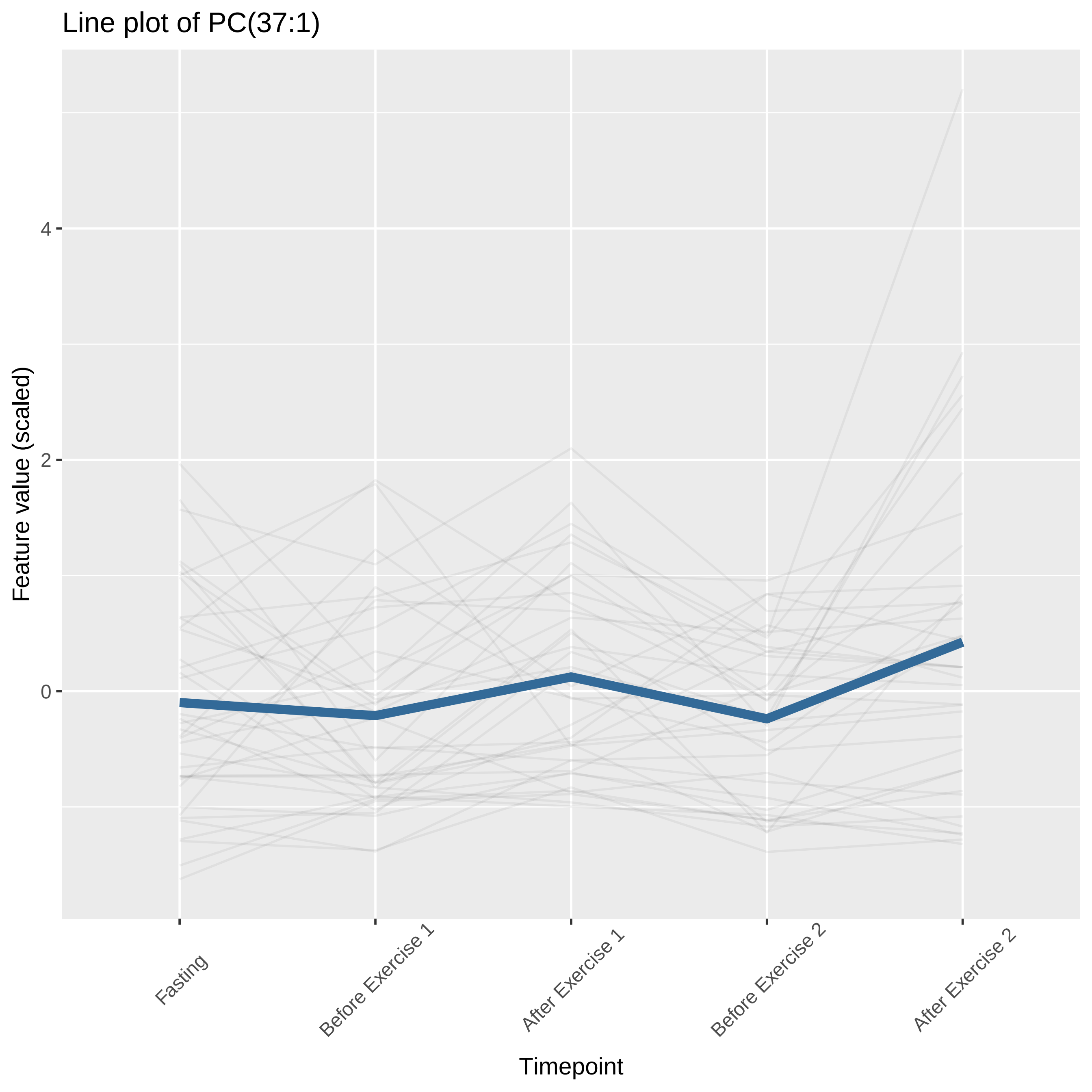

Line plot of PC(37:1)
Feature value (scaled)
4
2
0
Fasting
Before Exercise 1
After Exercise 1
Before Exercise 2
After Exercise 2
Timepoint

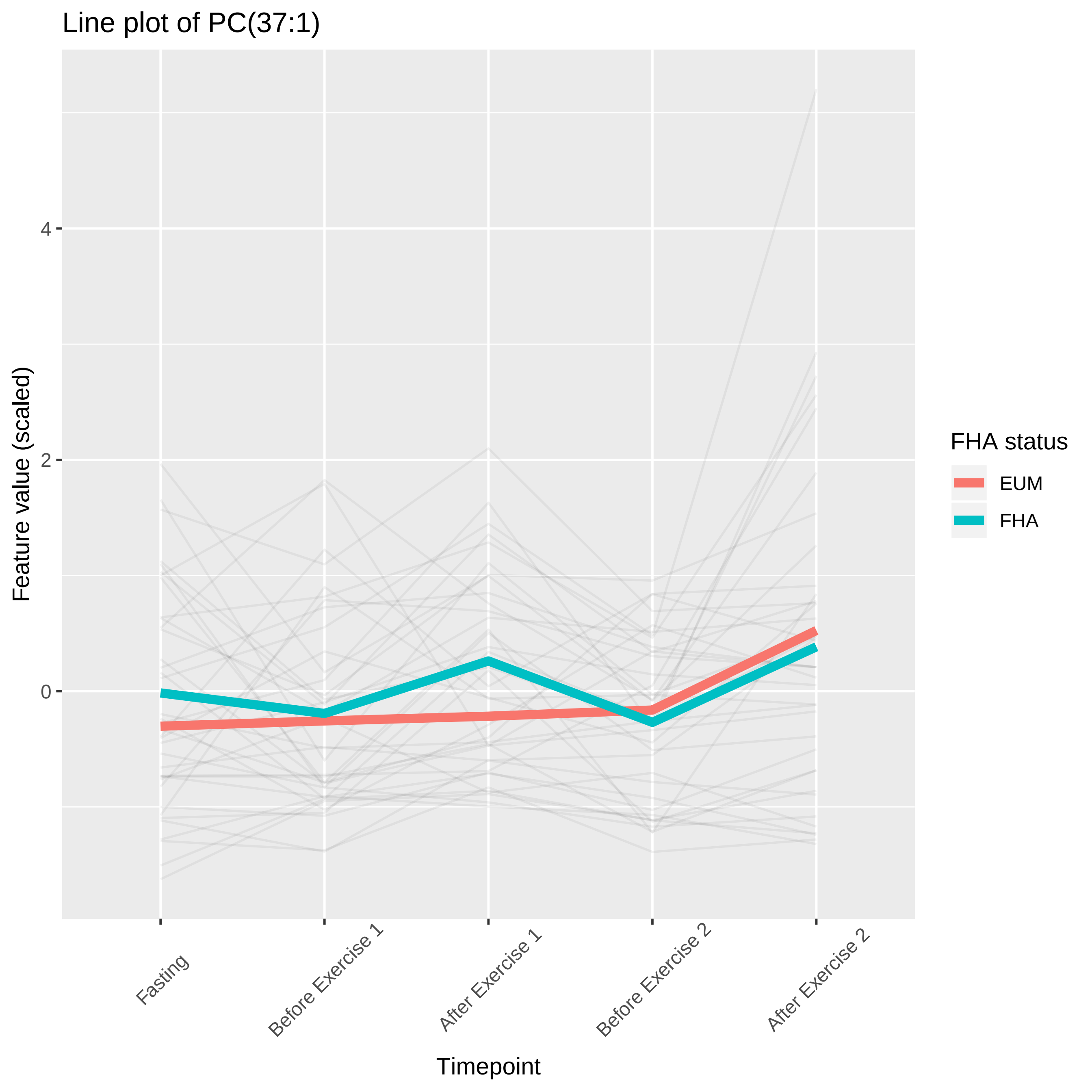
Line plot of PC(37:1)
Feature value (scaled)
4
2
0
Fasting
Before Exercise 1
After Exercise 1
Before Exercise 2
After Exercise 2
Timepoint
FHA status
EUM
FHA

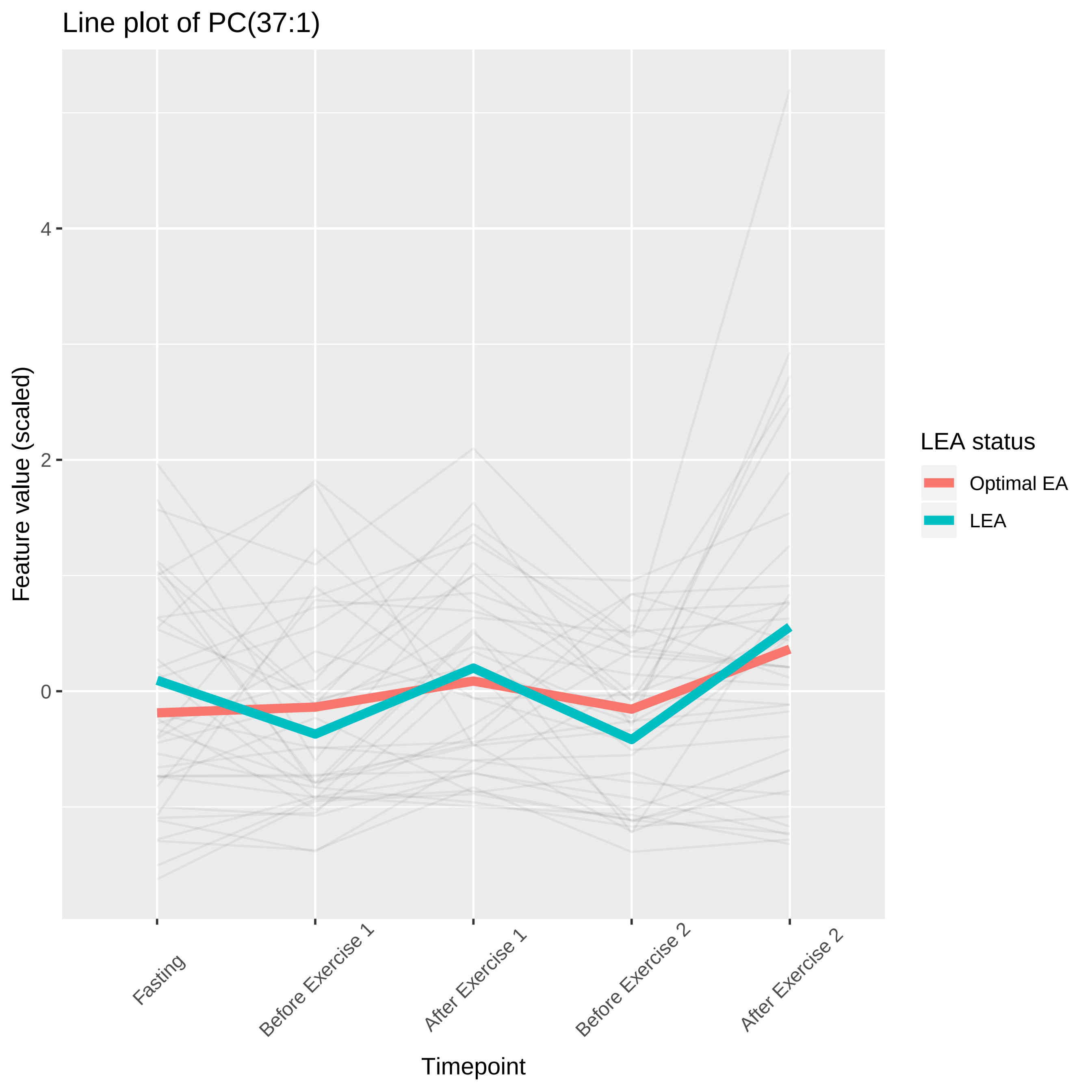

Line plot of PC(37:1)
Feature value (scaled)
4
2
0
Fasting
Before Exercise 1
After Exercise 1
Before Exercise 2
After Exercise 2
Timepoint
LEA status
Optimal EA
LEA

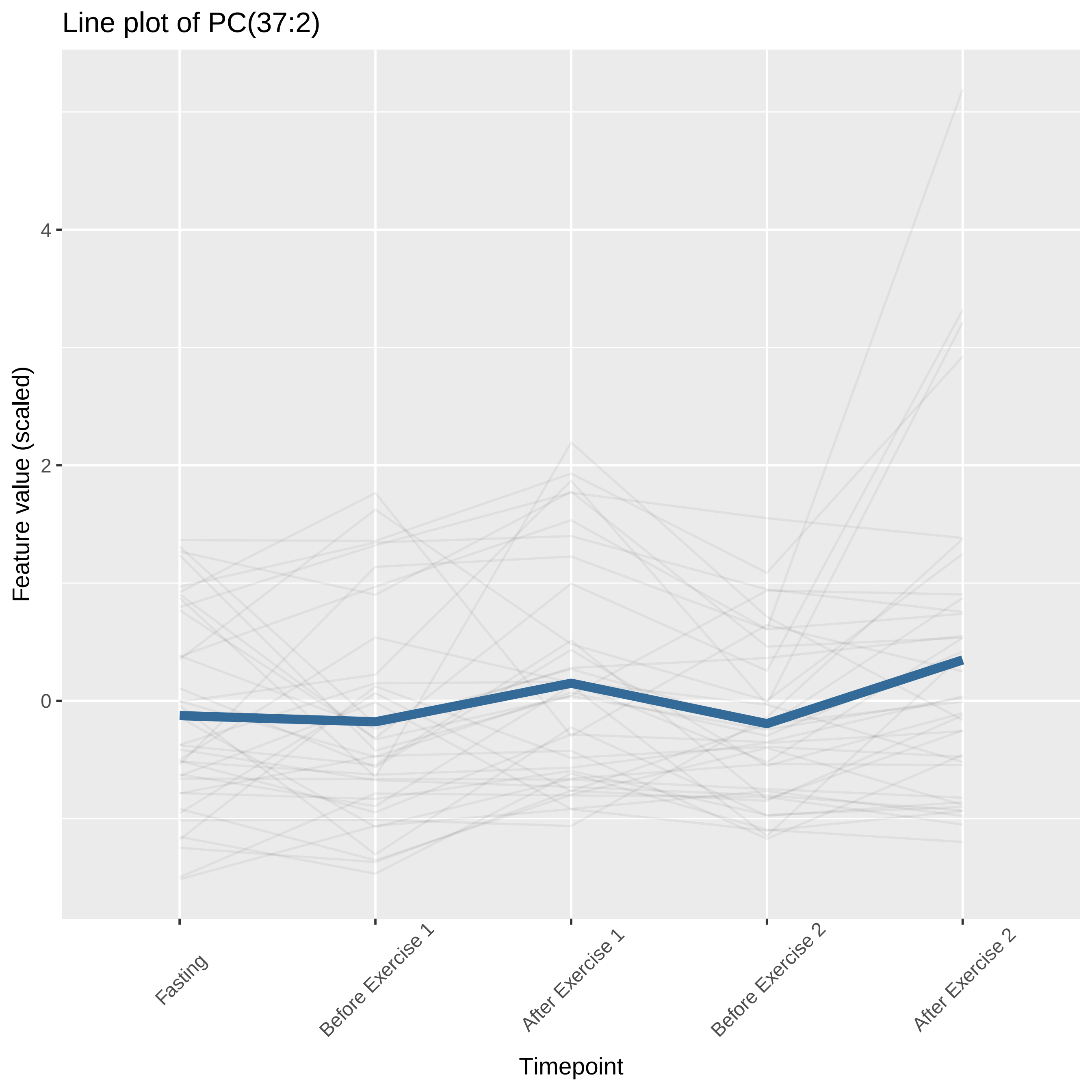

Line plot of PC(37:2)
Feature value (scaled)
4
2
0
Fasting
Before Exercise 1
After Exercise 1
Before Exercise 2
After Exercise 2
Timepoint

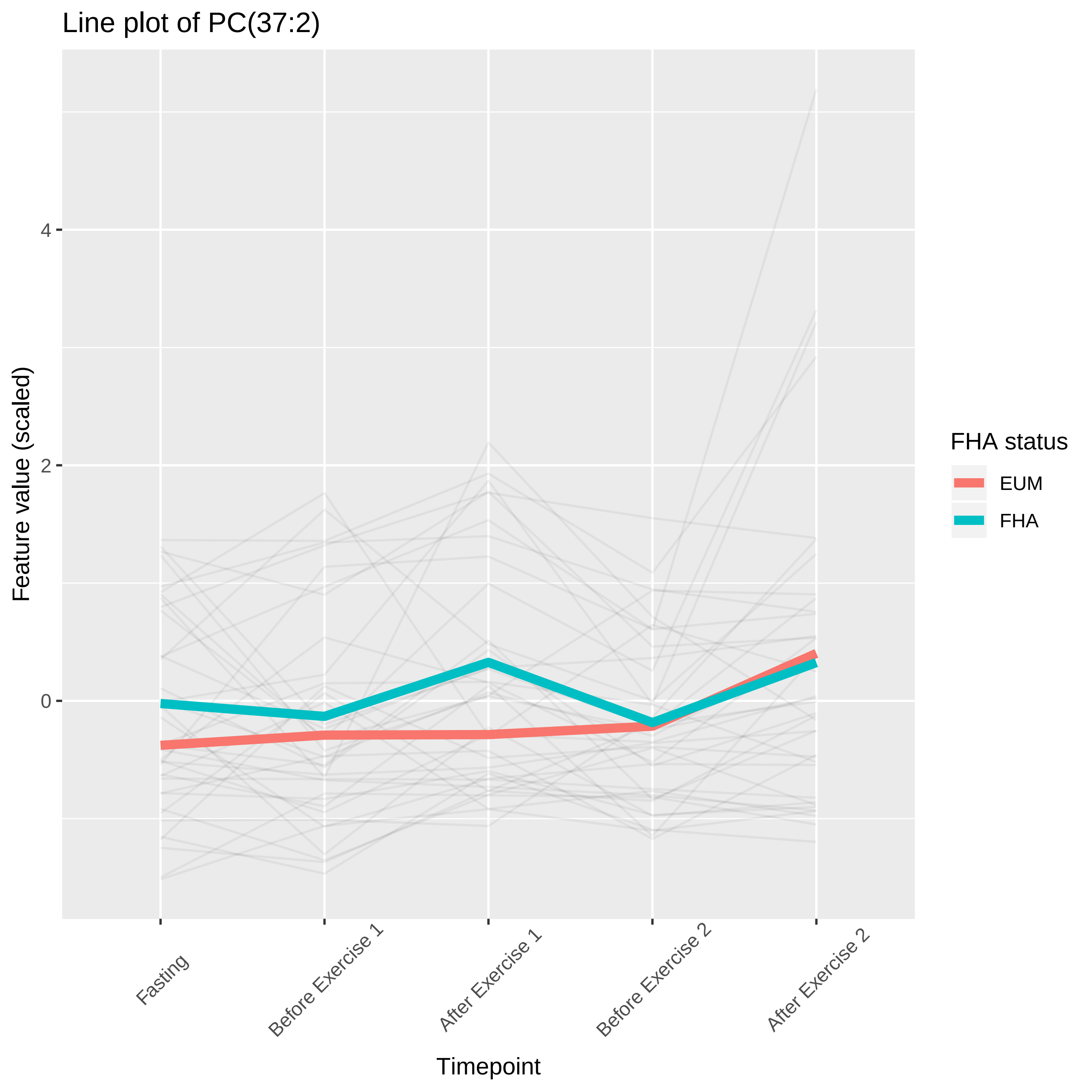
Line plot of PC(37:2)
Feature value (scaled)
4
2
0
Fasting
Before Exercise 1
After Exercise 1
Before Exercise 2
After Exercise 2
Timepoint
FHA status
EUM
FHA

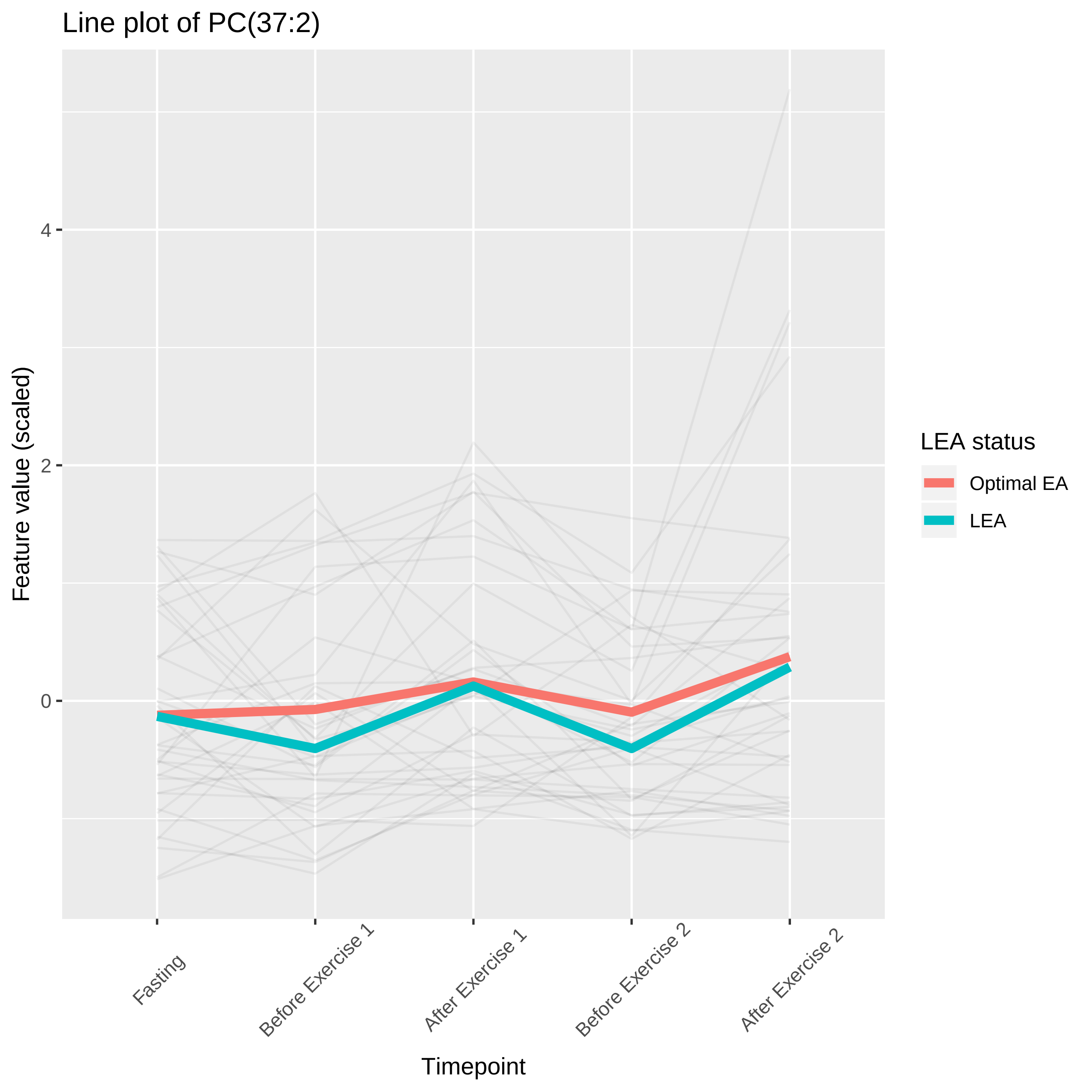

Line plot of PC(37:2)
Feature value (scaled)
4
2
0
Fasting
Before Exercise 1
After Exercise 1
Before Exercise 2
After Exercise 2
Timepoint
LEA status
Optimal EA
LEA

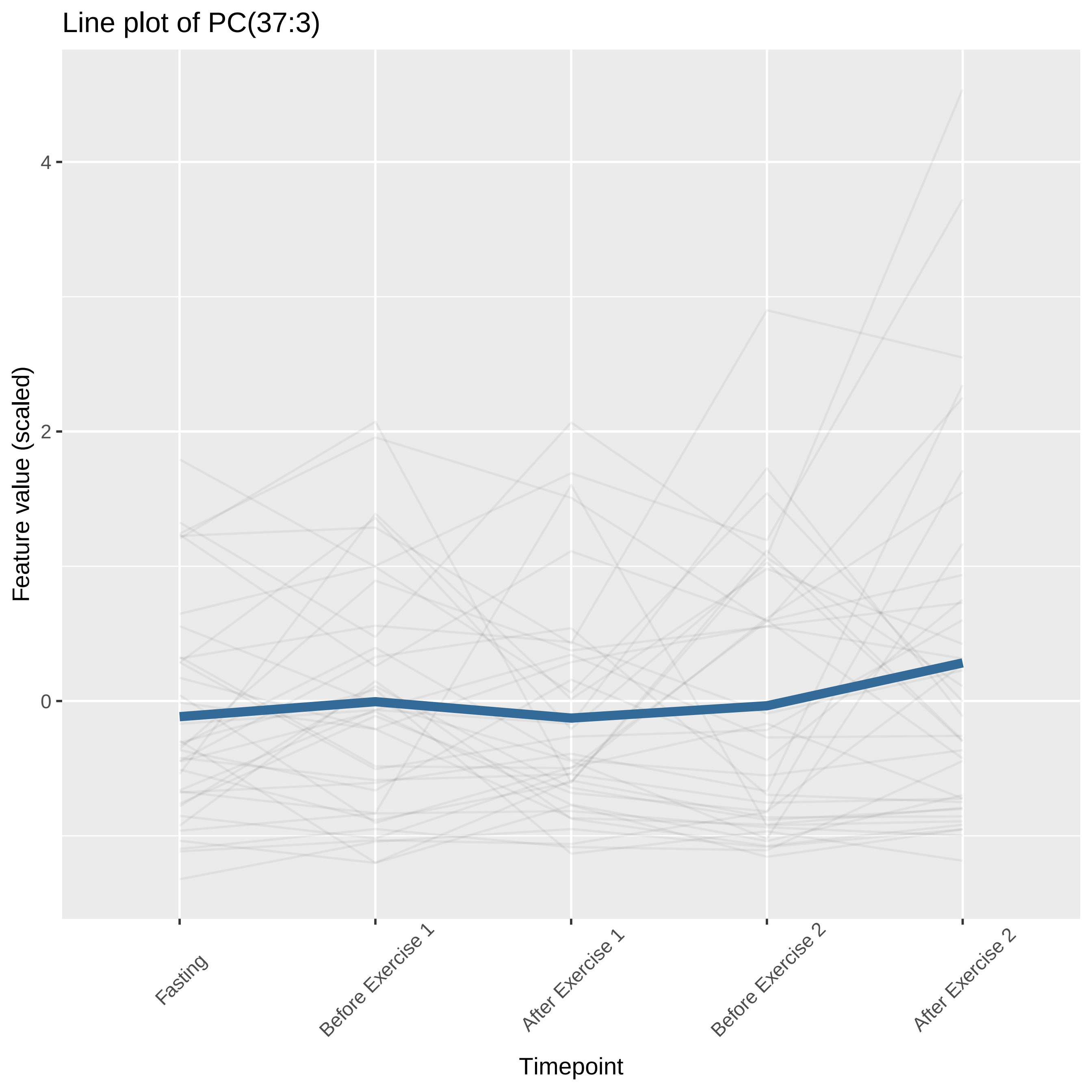

Line plot of PC(37:3)
Feature value (scaled)
4
2
0
Fasting
Before Exercise 1
After Exercise 1
Before Exercise 2
After Exercise 2
Timepoint

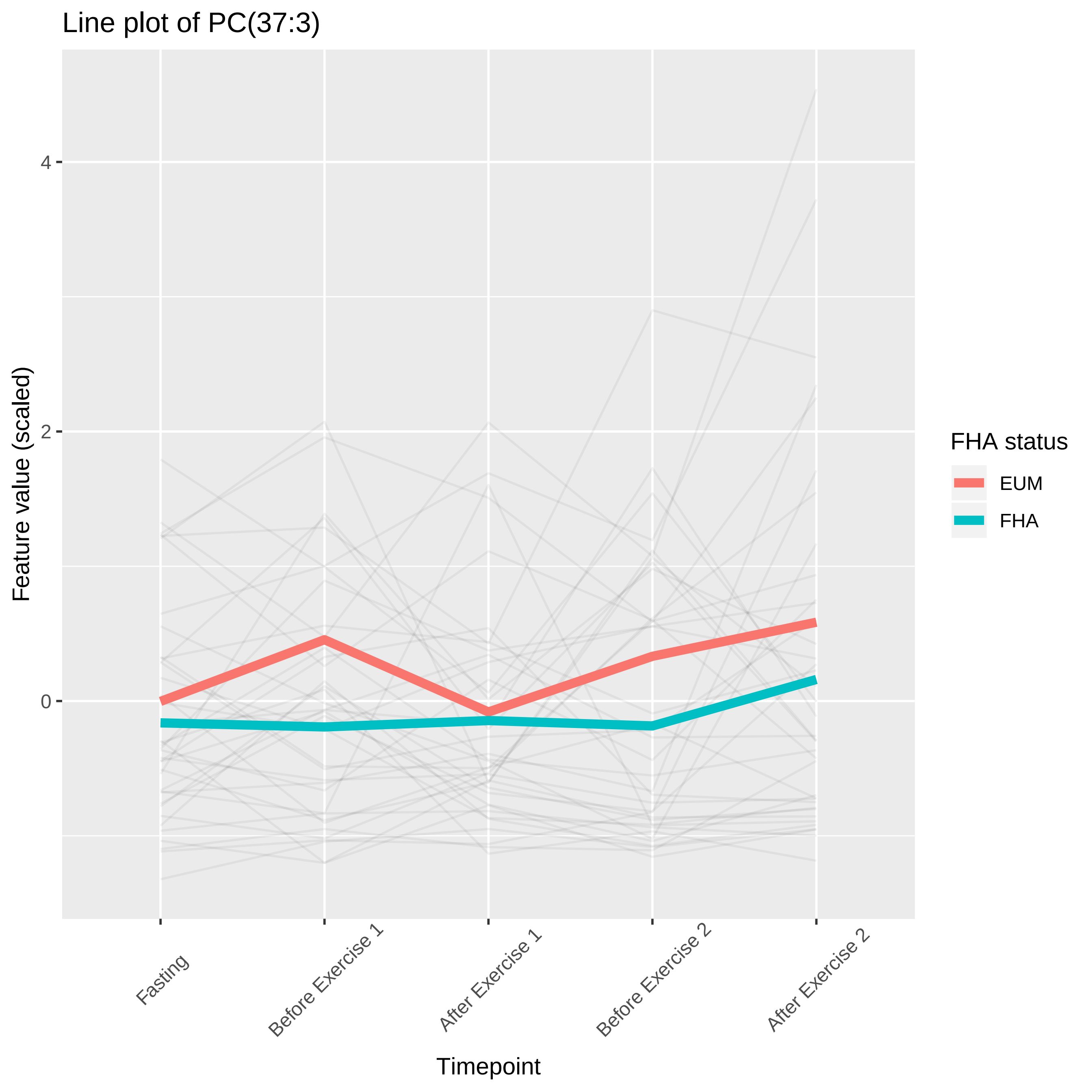
Line plot of PC(37:3)
Feature value (scaled)
4
2
0
Fasting
Before Exercise 1
After Exercise 1
Before Exercise 2
After Exercise 2
Timepoint
FHA status
EUM
FHA

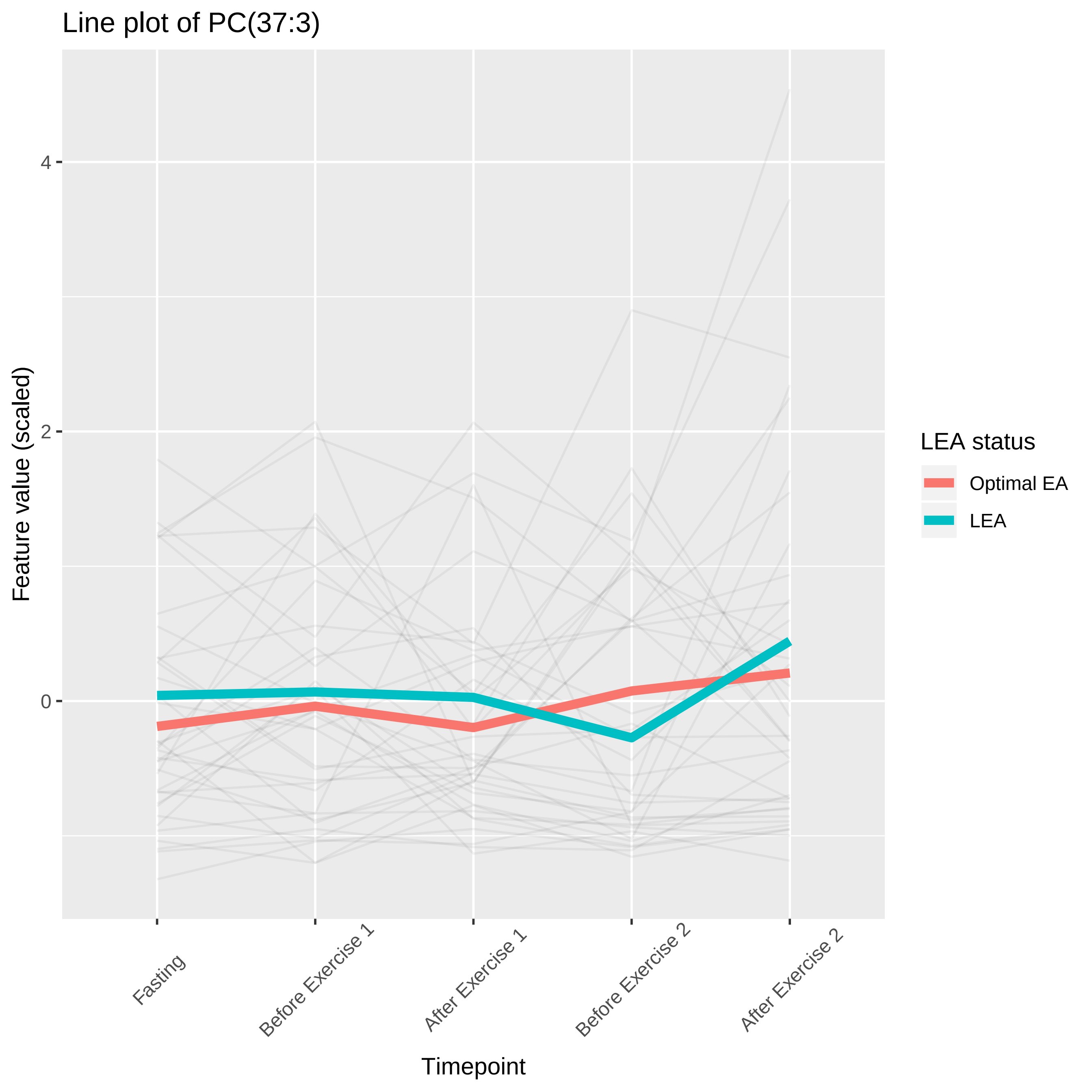

Line plot of PC(37:3)
Feature value (scaled)
4
2
0
Fasting
Before Exercise 1
After Exercise 1
Before Exercise 2
After Exercise 2
Timepoint
LEA status
Optimal EA
LEA

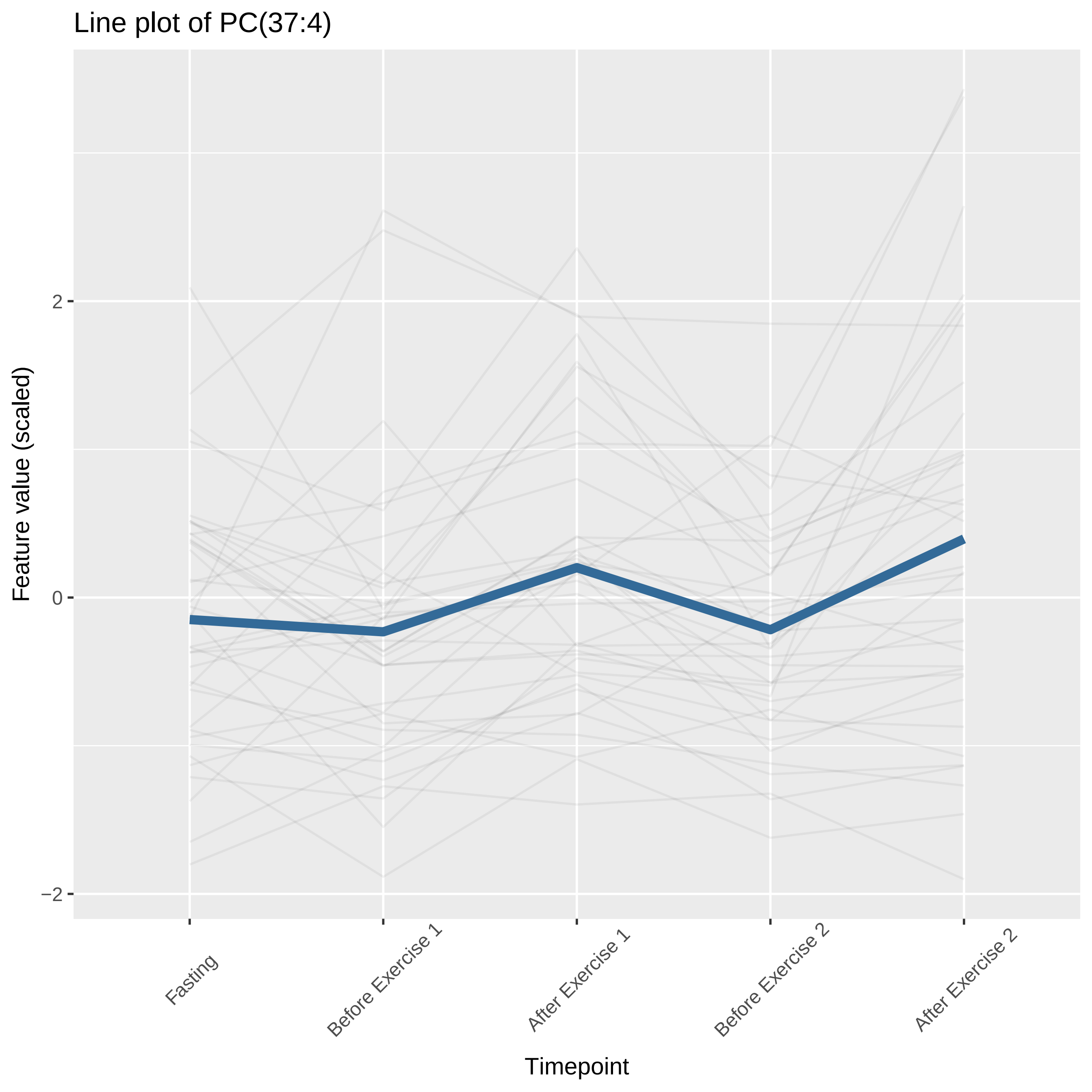

Line plot of PC(37:4)
Feature value (scaled)
2
0
−2
Fasting
Before Exercise 1
After Exercise 1
Before Exercise 2
After Exercise 2
Timepoint

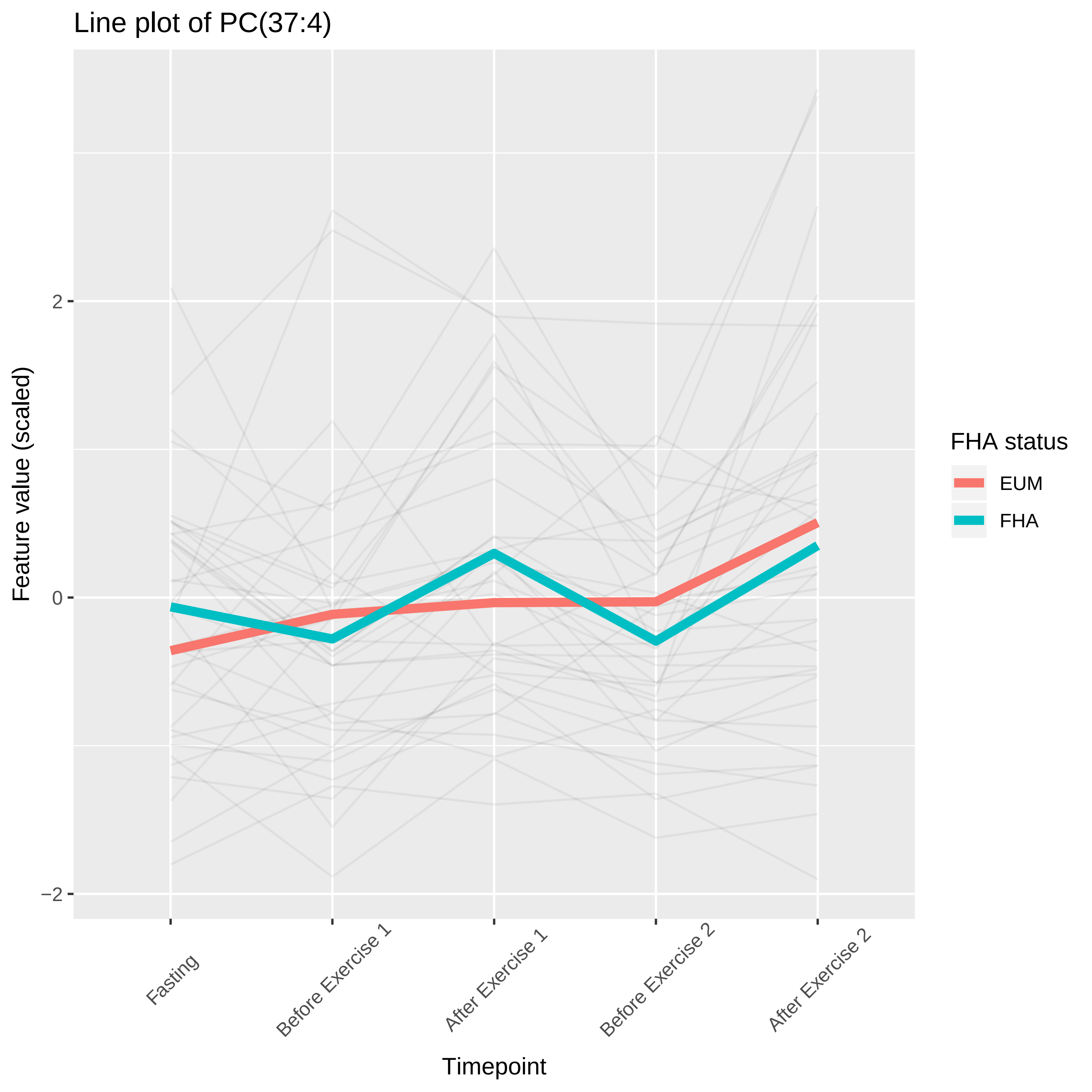

Line plot of PC(37:4)
Feature value (scaled)
2
0
−2
Fasting
Before Exercise 1
After Exercise 1
Before Exercise 2
After Exercise 2
Timepoint
FHA status
EUM
FHA

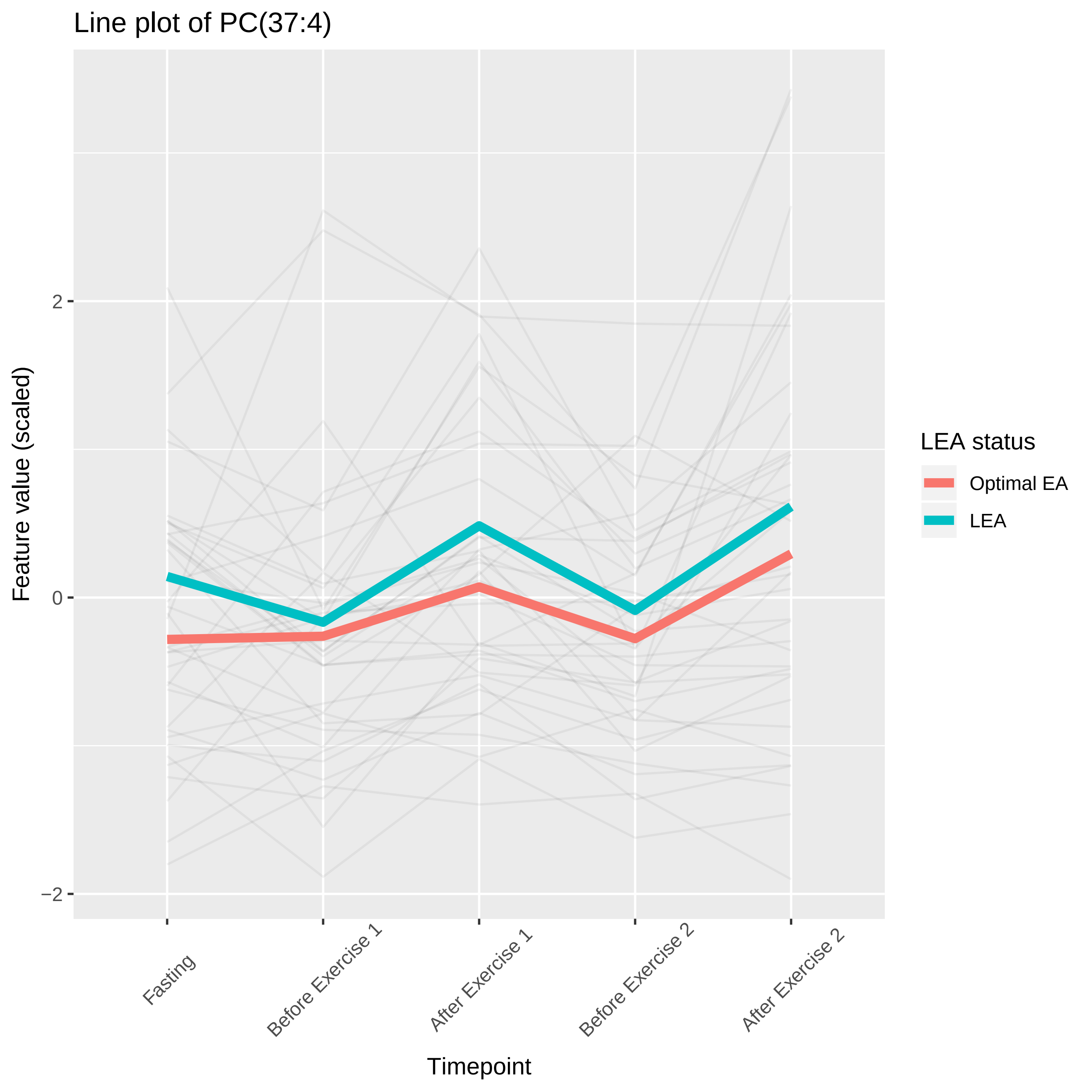

Line plot of PC(37:4)
Feature value (scaled)
2
0
−2
Fasting
Before Exercise 1
After Exercise 1
Before Exercise 2
After Exercise 2
Timepoint
LEA status
Optimal EA
LEA

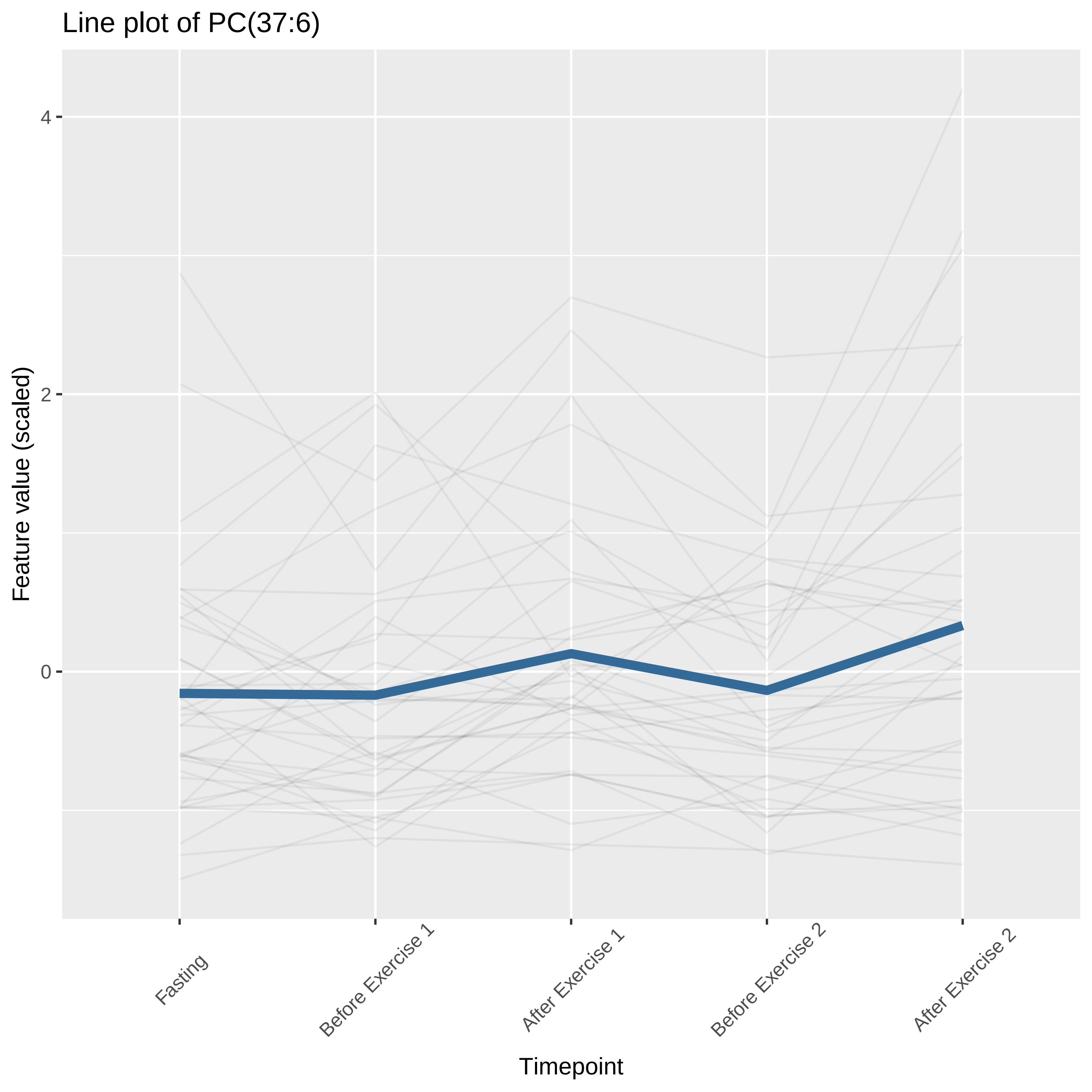

Line plot of PC(37:6)
Feature value (scaled)
4
2
0
Fasting
Before Exercise 1
After Exercise 1
Before Exercise 2
After Exercise 2
Timepoint

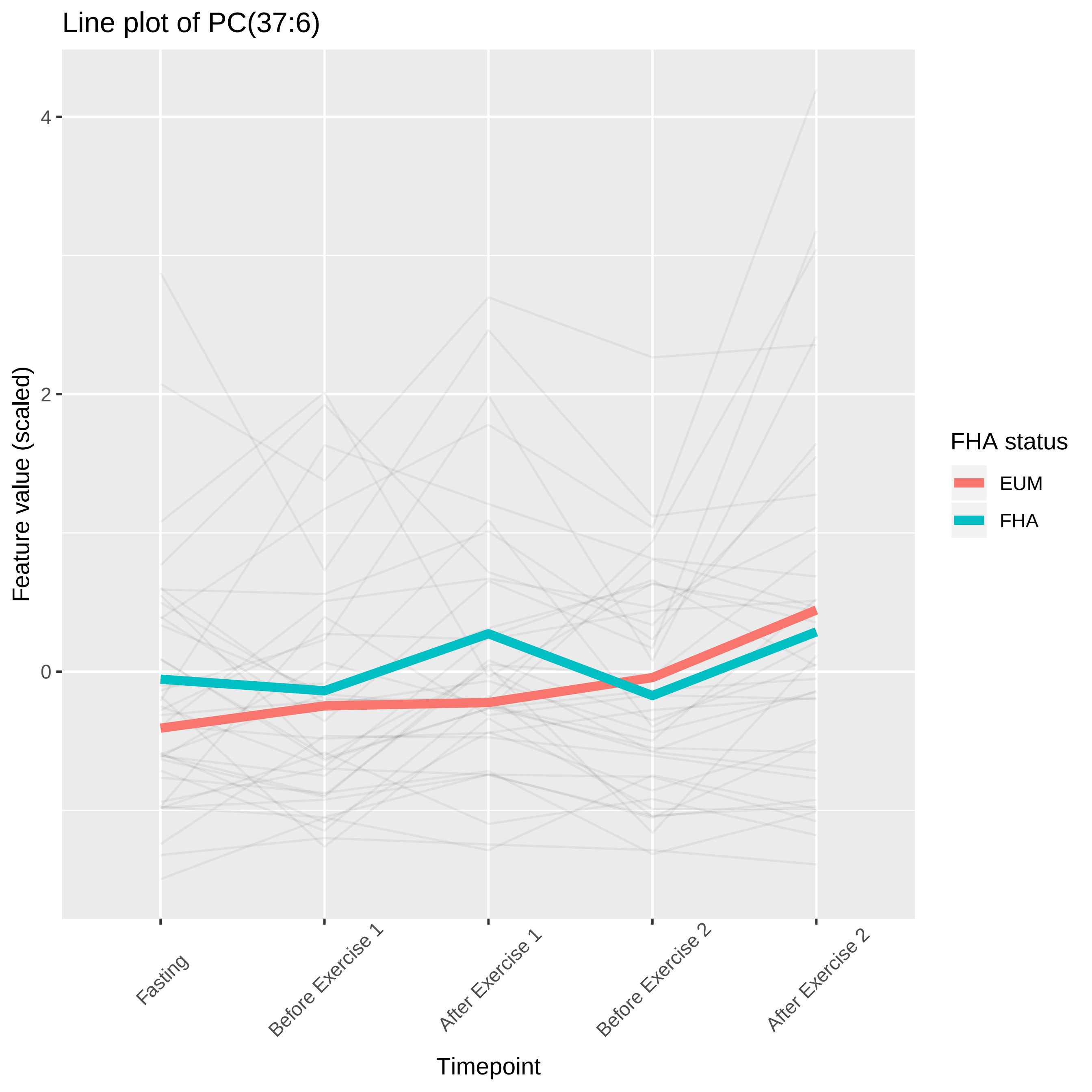

Line plot of PC(37:6)
Feature value (scaled)
4
2
0
Fasting
Before Exercise 1
After Exercise 1
Before Exercise 2
After Exercise 2
Timepoint
FHA status
EUM
FHA

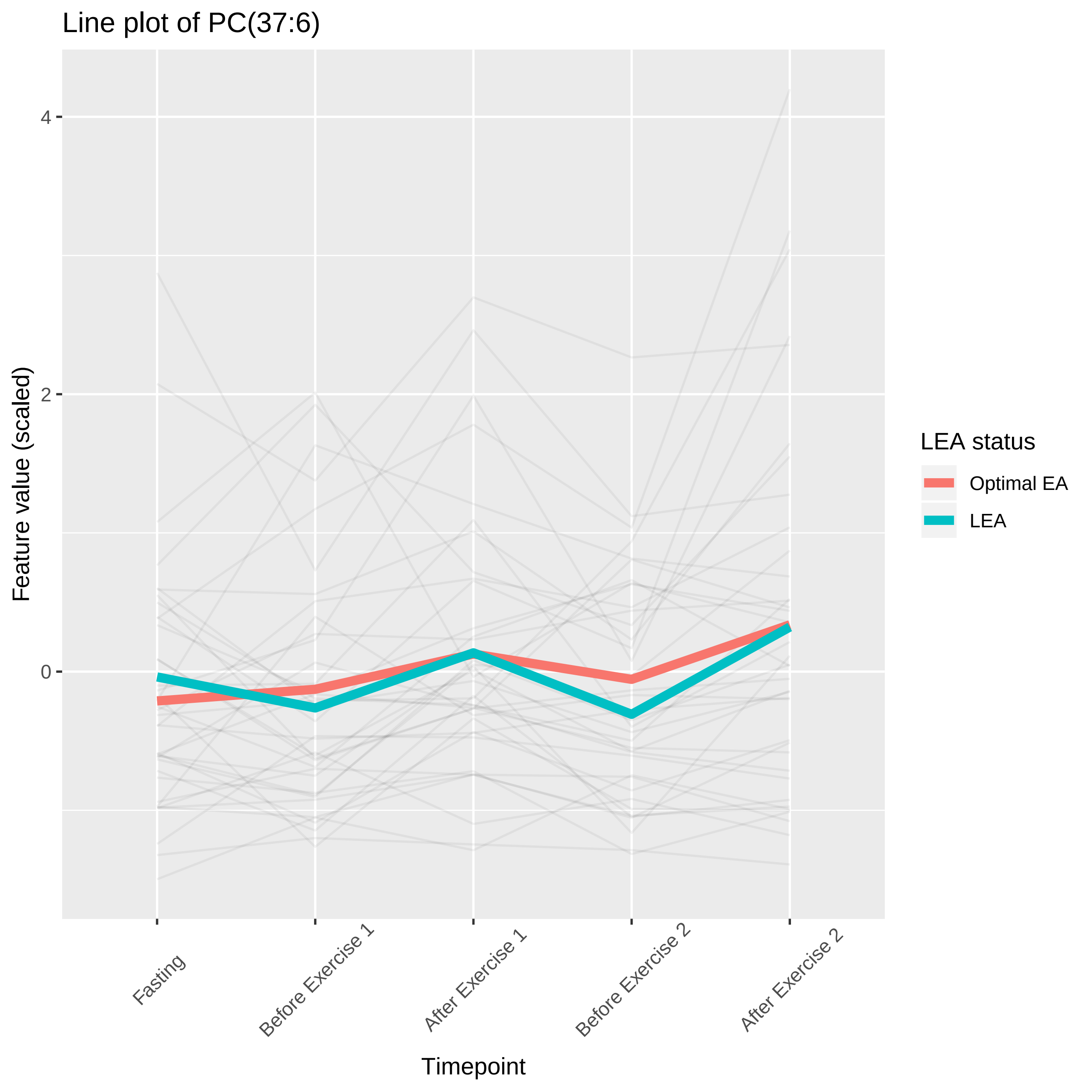

Line plot of PC(37:6)
Feature value (scaled)
4
2
0
Fasting
Before Exercise 1
After Exercise 1
Before Exercise 2
After Exercise 2
Timepoint
LEA status
Optimal EA
LEA

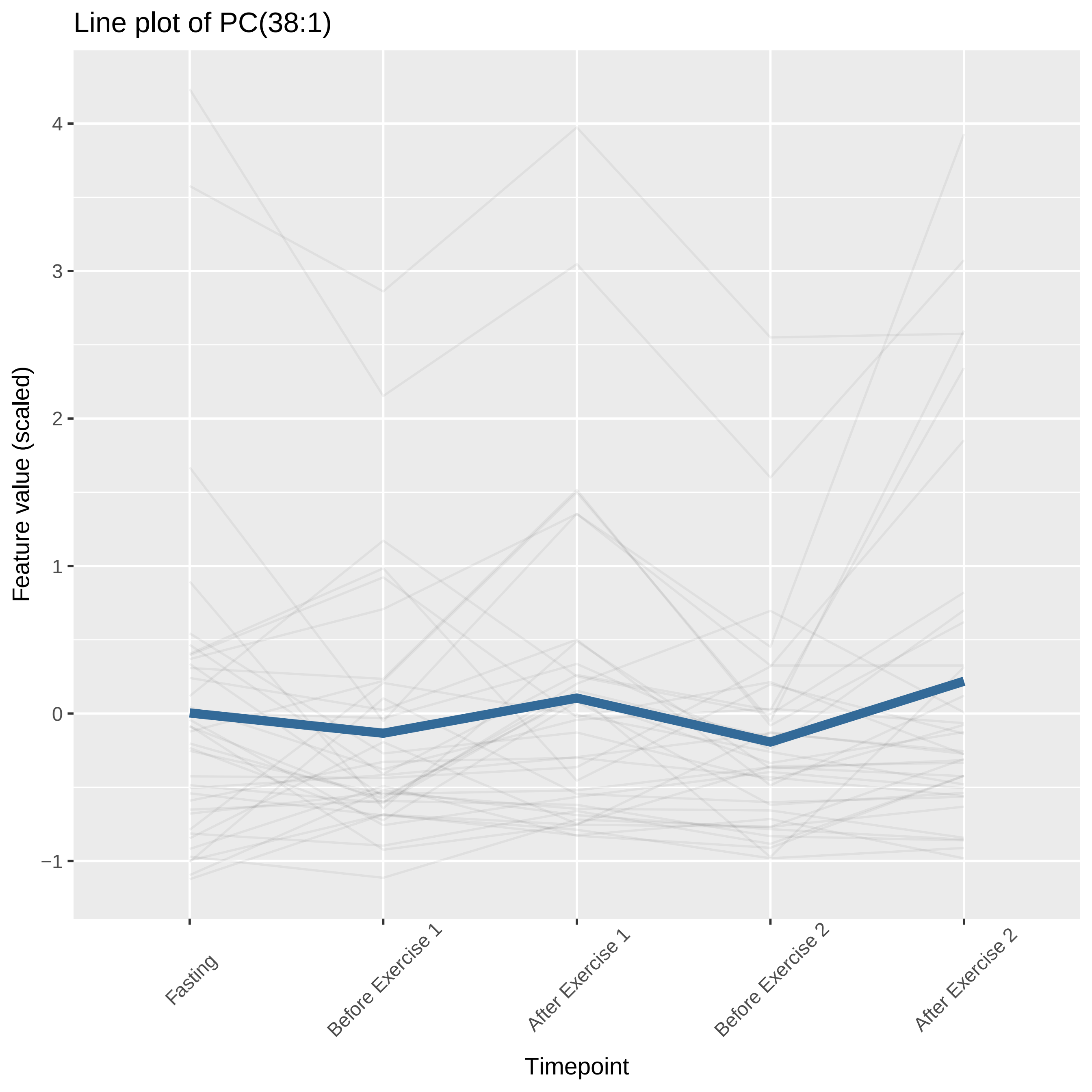

Line plot of PC(38:1)
Feature value (scaled)
4
3
2
1
0
−1
Fasting
Before Exercise 1
After Exercise 1
Before Exercise 2
After Exercise 2
Timepoint

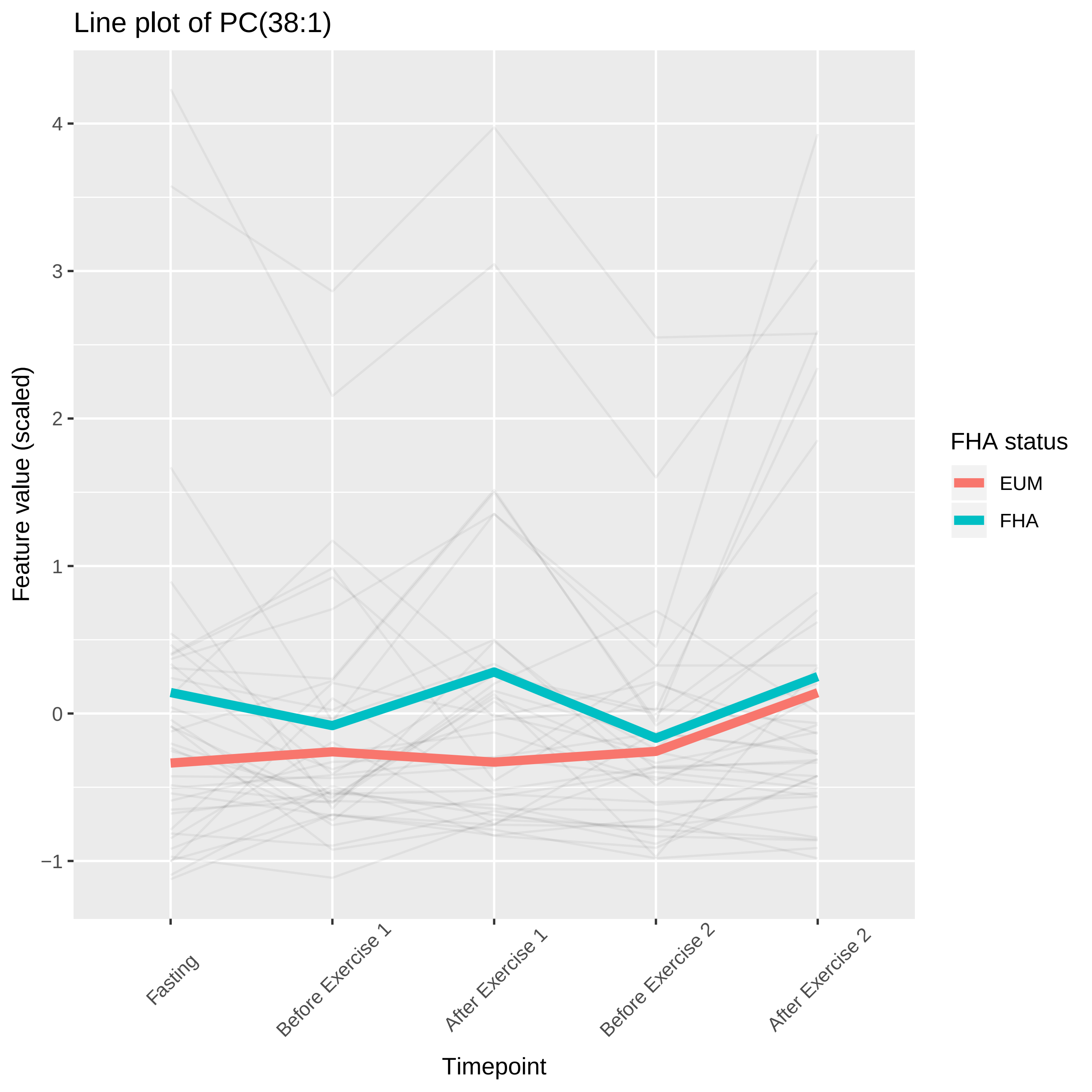

Line plot of PC(38:1)
Feature value (scaled)
4
3
2
1
0
−1
Fasting
Before Exercise 1
After Exercise 1
Before Exercise 2
After Exercise 2
Timepoint
FHA status
EUM
FHA

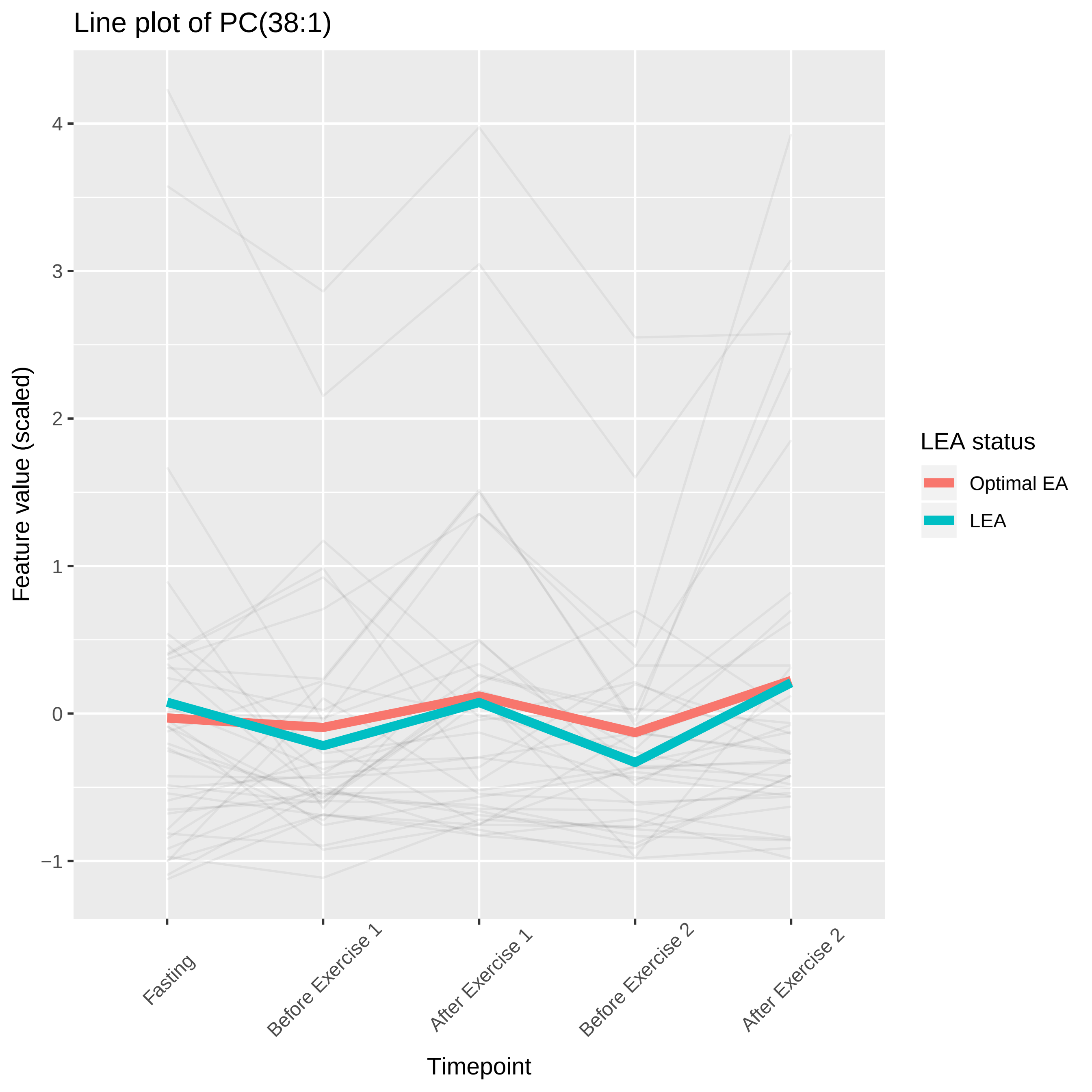
Line plot of PC(38:1)
Feature value (scaled)
4
3
2
1
0
−1
Fasting
Before Exercise 1
After Exercise 1
Before Exercise 2
After Exercise 2
Timepoint
LEA status
Optimal EA
LEA

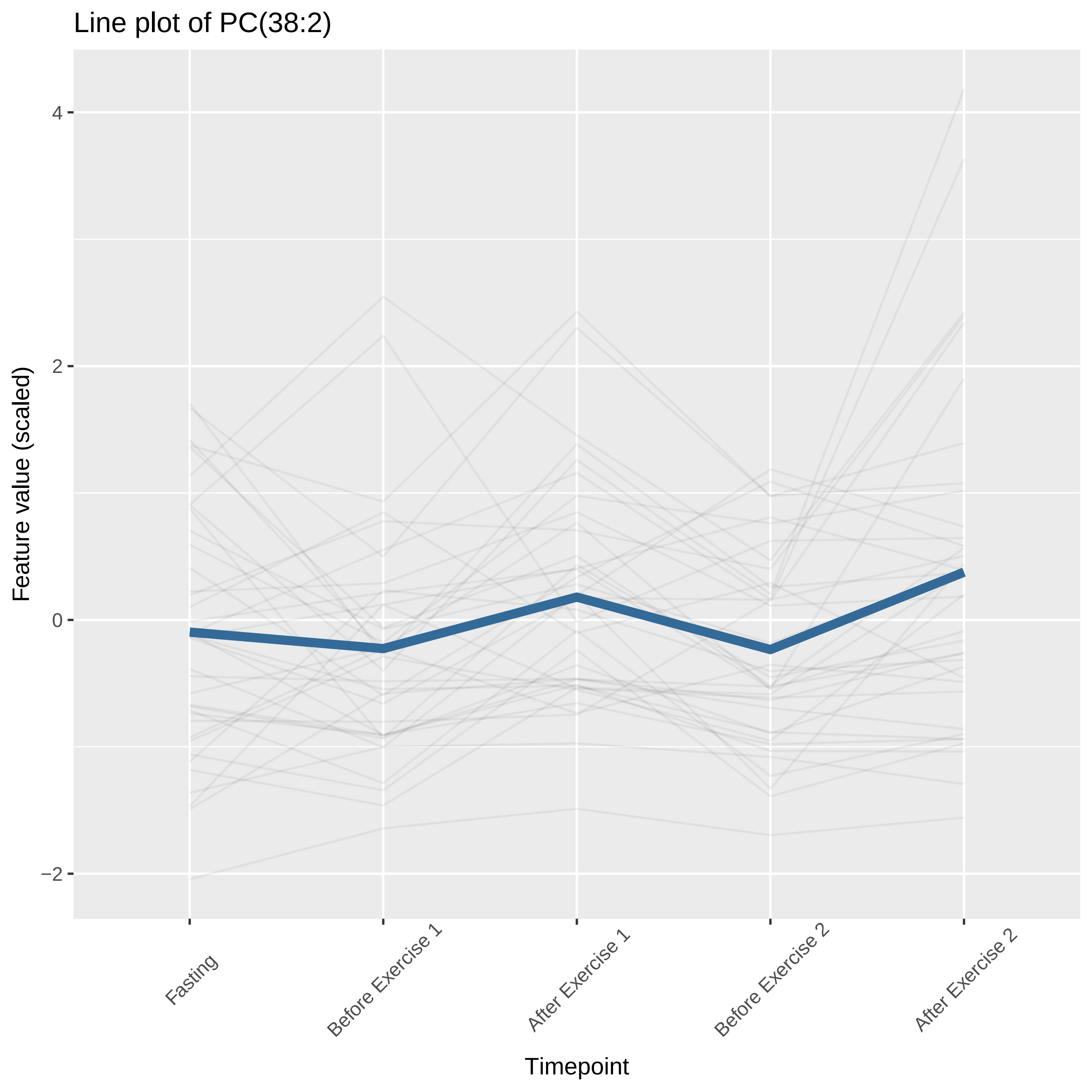

Line plot of PC(38:2)
Feature value (scaled)
4
2
0
−2
Fasting
Before Exercise 1
After Exercise 1
Before Exercise 2
After Exercise 2
Timepoint

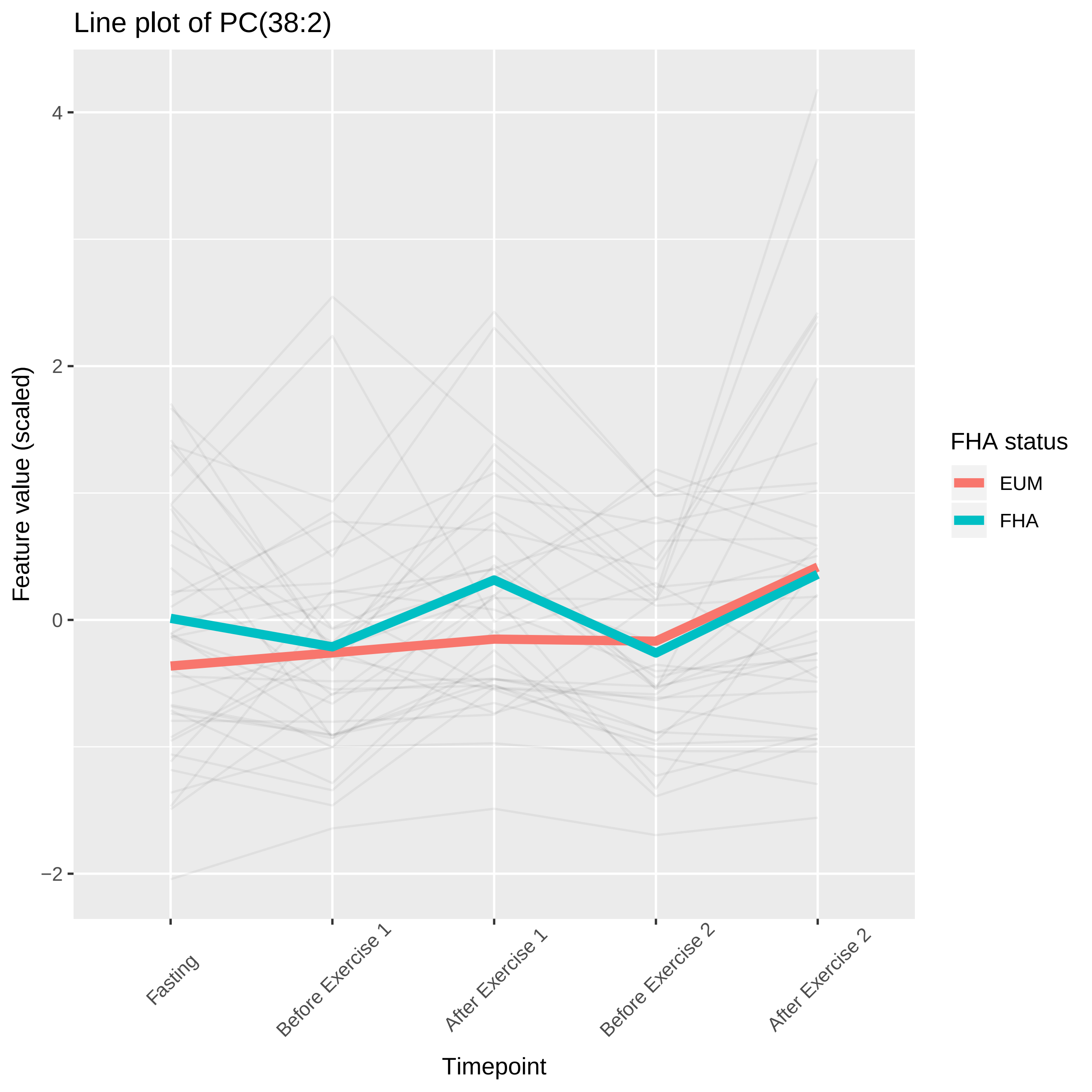

Line plot of PC(38:2)
Feature value (scaled)
4
2
0
−2
Fasting
Before Exercise 1
After Exercise 1
Before Exercise 2
After Exercise 2
Timepoint
FHA status
EUM
FHA

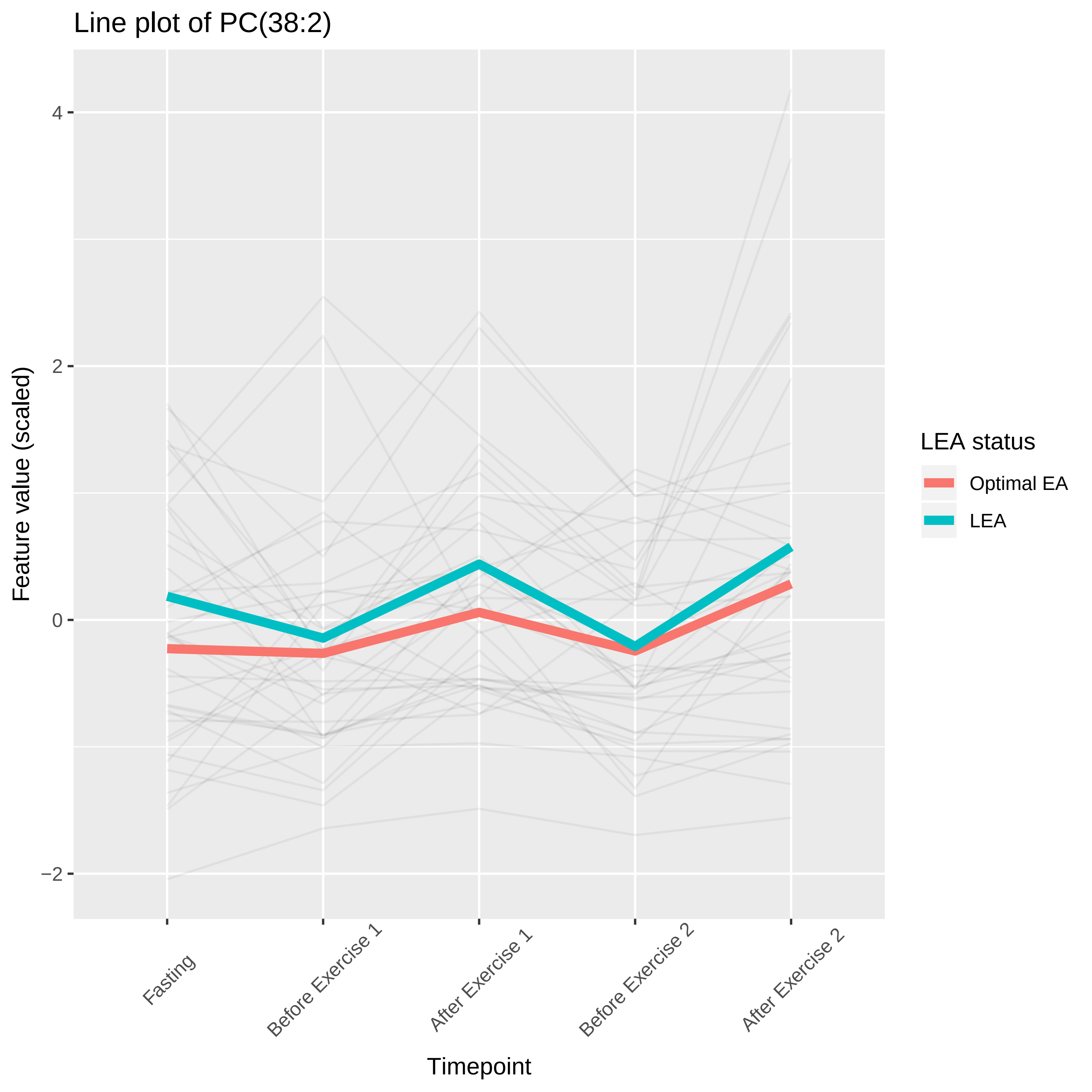

Line plot of PC(38:2)
Feature value (scaled)
4
2
0
−2
Fasting
Before Exercise 1
After Exercise 1
Before Exercise 2
After Exercise 2
Timepoint
LEA status
Optimal EA
LEA

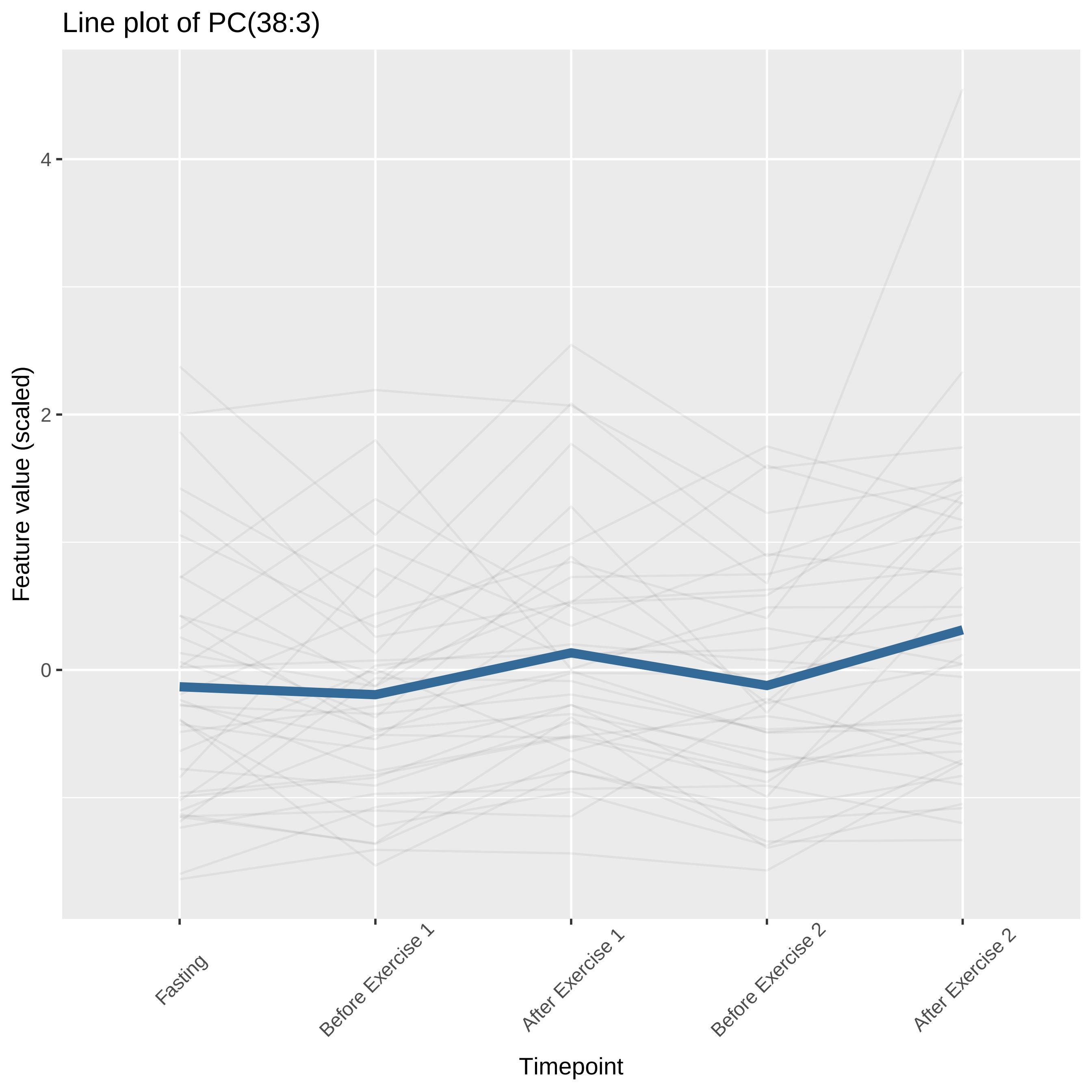

Line plot of PC(38:3)
Feature value (scaled)
4
2
0
Fasting
Before Exercise 1
After Exercise 1
Before Exercise 2
After Exercise 2
Timepoint

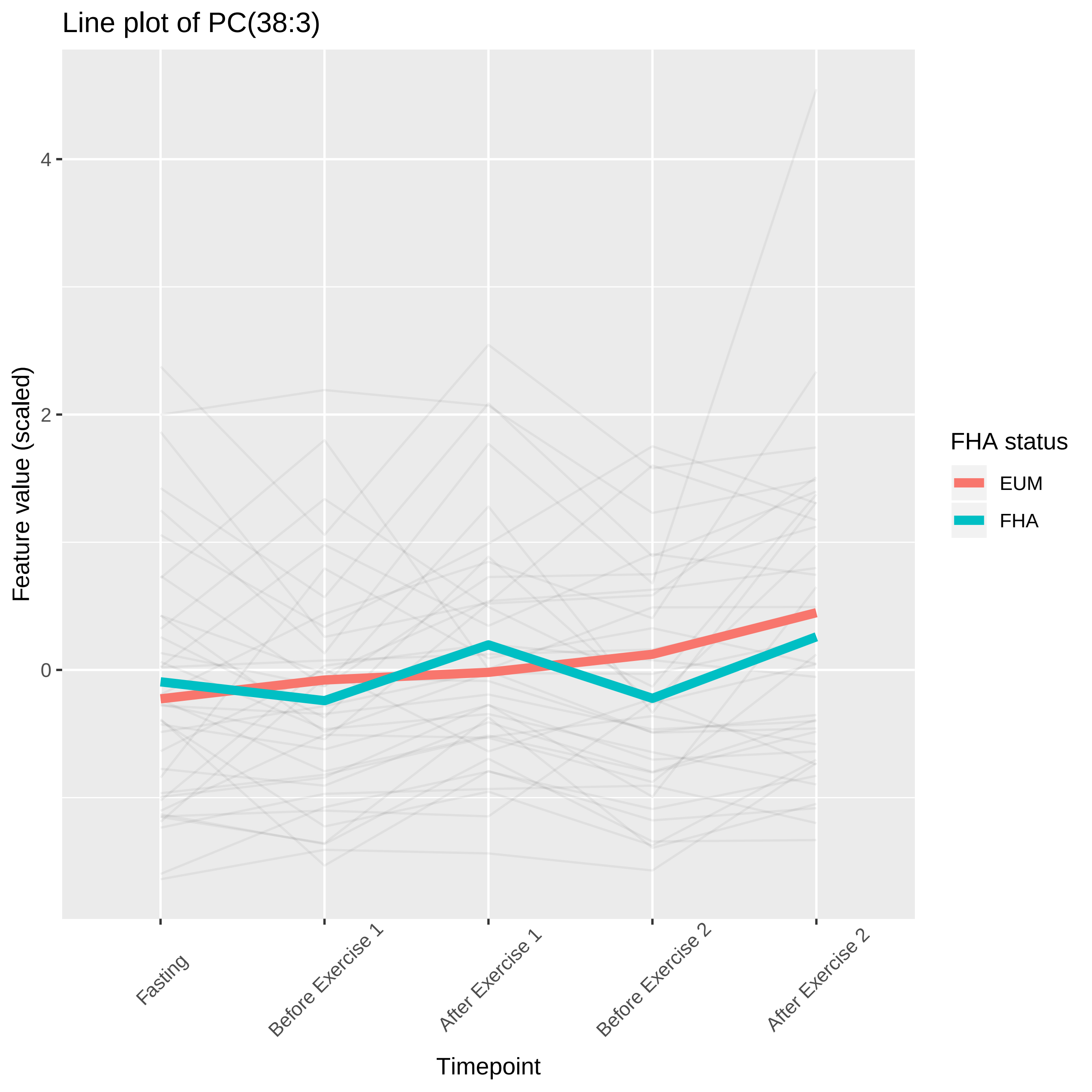

Line plot of PC(38:3)
Feature value (scaled)
4
2
0
Fasting
Before Exercise 1
After Exercise 1
Before Exercise 2
After Exercise 2
Timepoint
FHA status
EUM
FHA

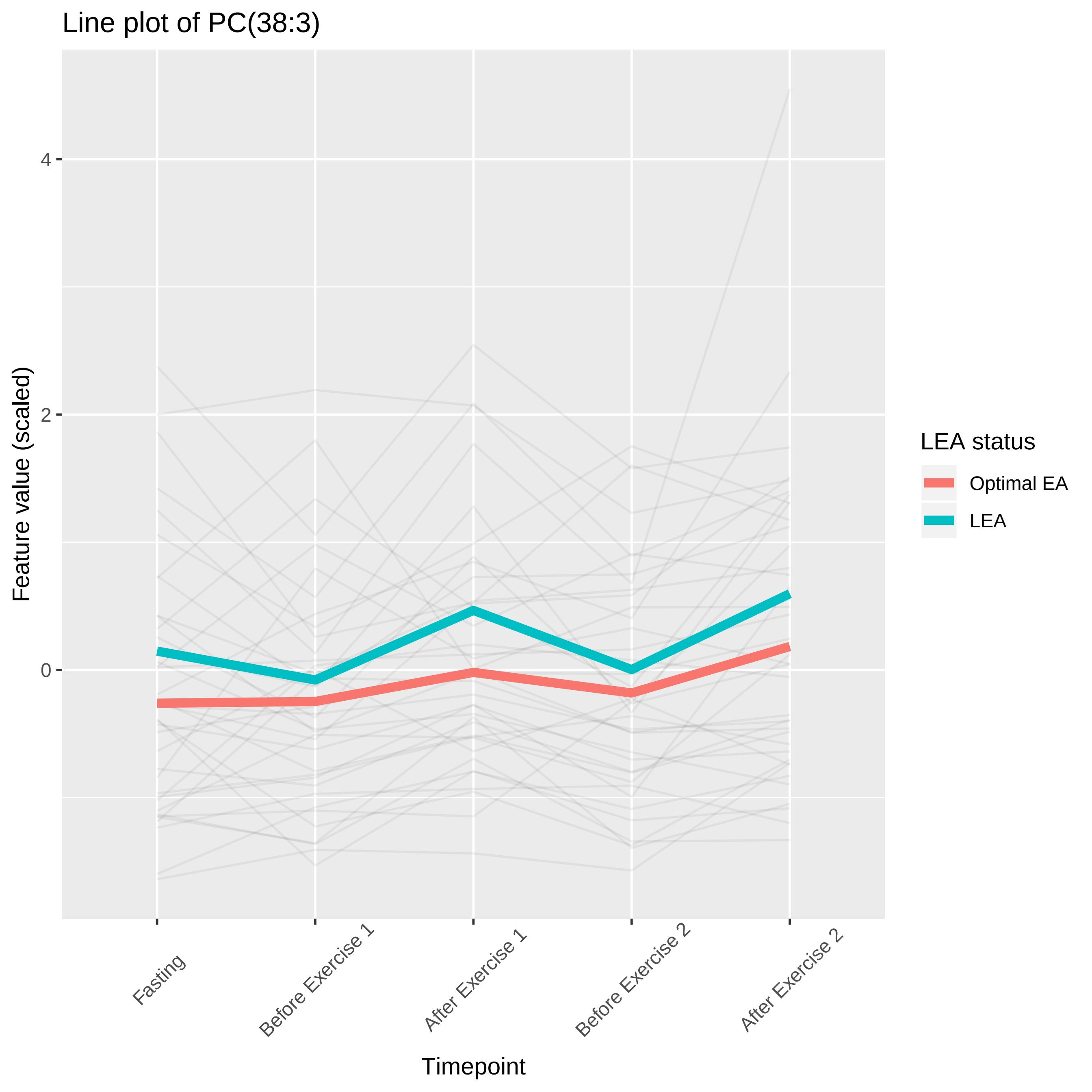

Line plot of PC(38:3)
Feature value (scaled)
4
2
0
Fasting
Before Exercise 1
After Exercise 1
Before Exercise 2
After Exercise 2
Timepoint
LEA status
Optimal EA
LEA

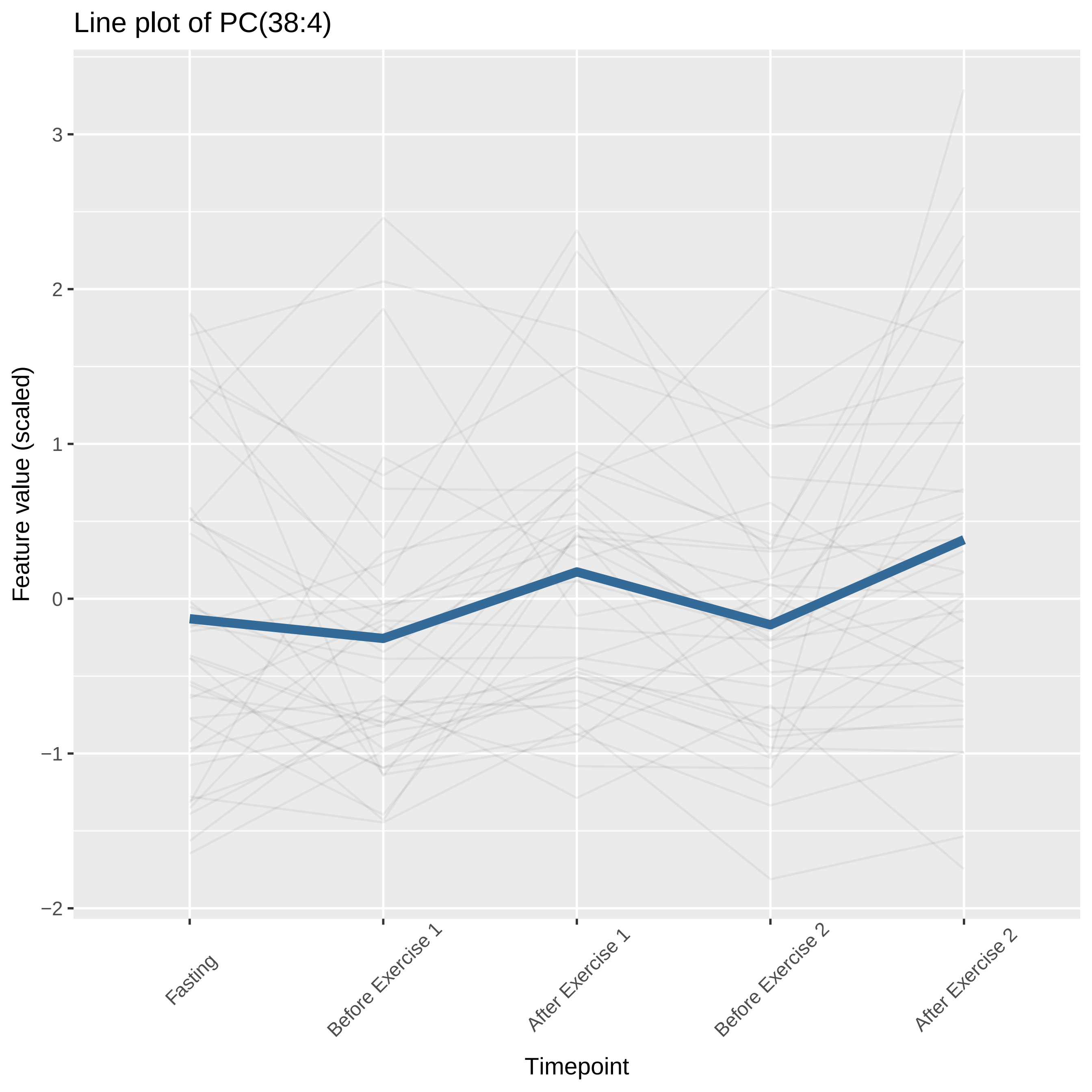

Line plot of PC(38:4)
Feature value (scaled)
3
2
1
0
−1
−2
Fasting
Before Exercise 1
After Exercise 1
Before Exercise 2
After Exercise 2
Timepoint

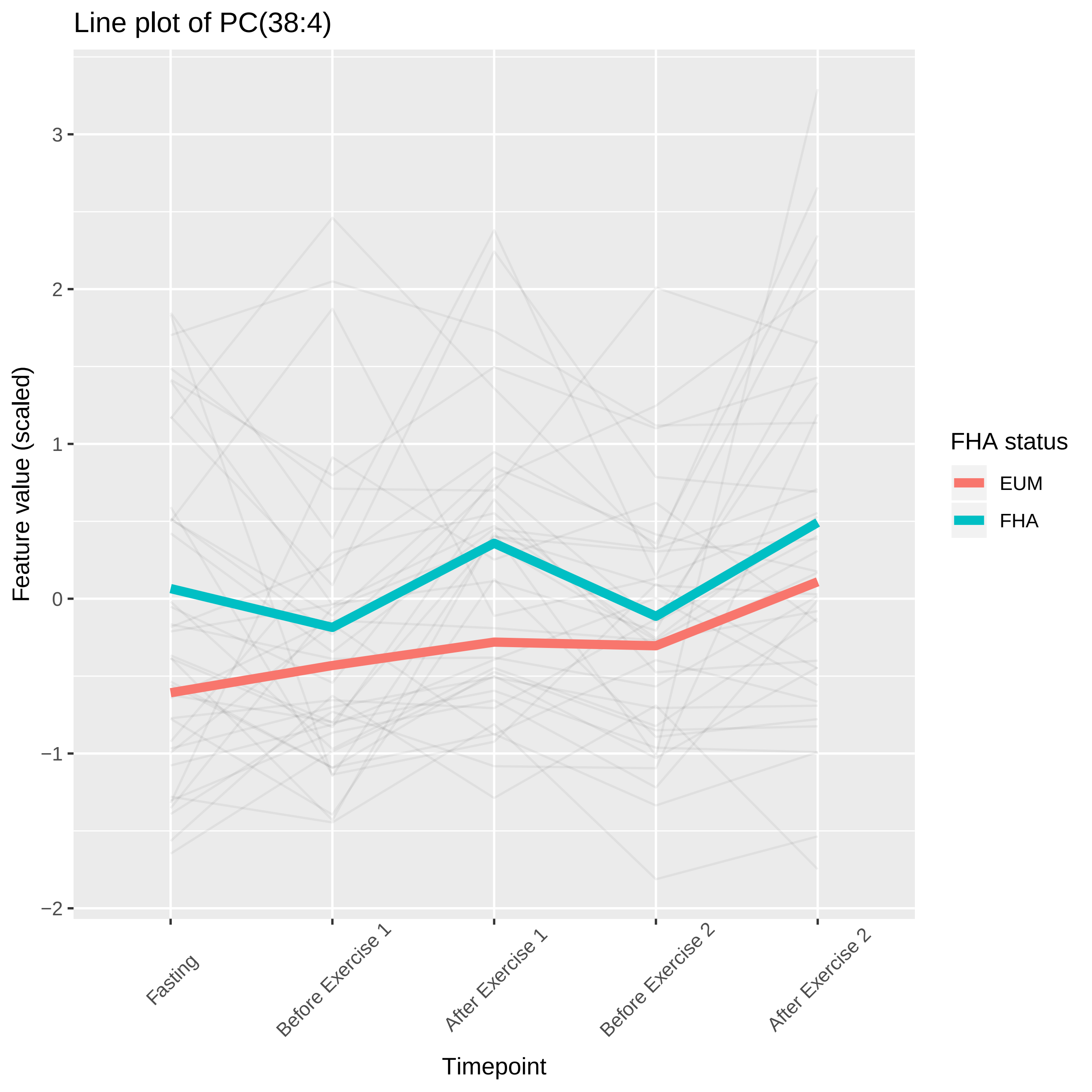

Line plot of PC(38:4)
Feature value (scaled)
3
2
1
0
−1
−2
Fasting
Before Exercise 1
After Exercise 1
Before Exercise 2
After Exercise 2
Timepoint
FHA status
EUM
FHA

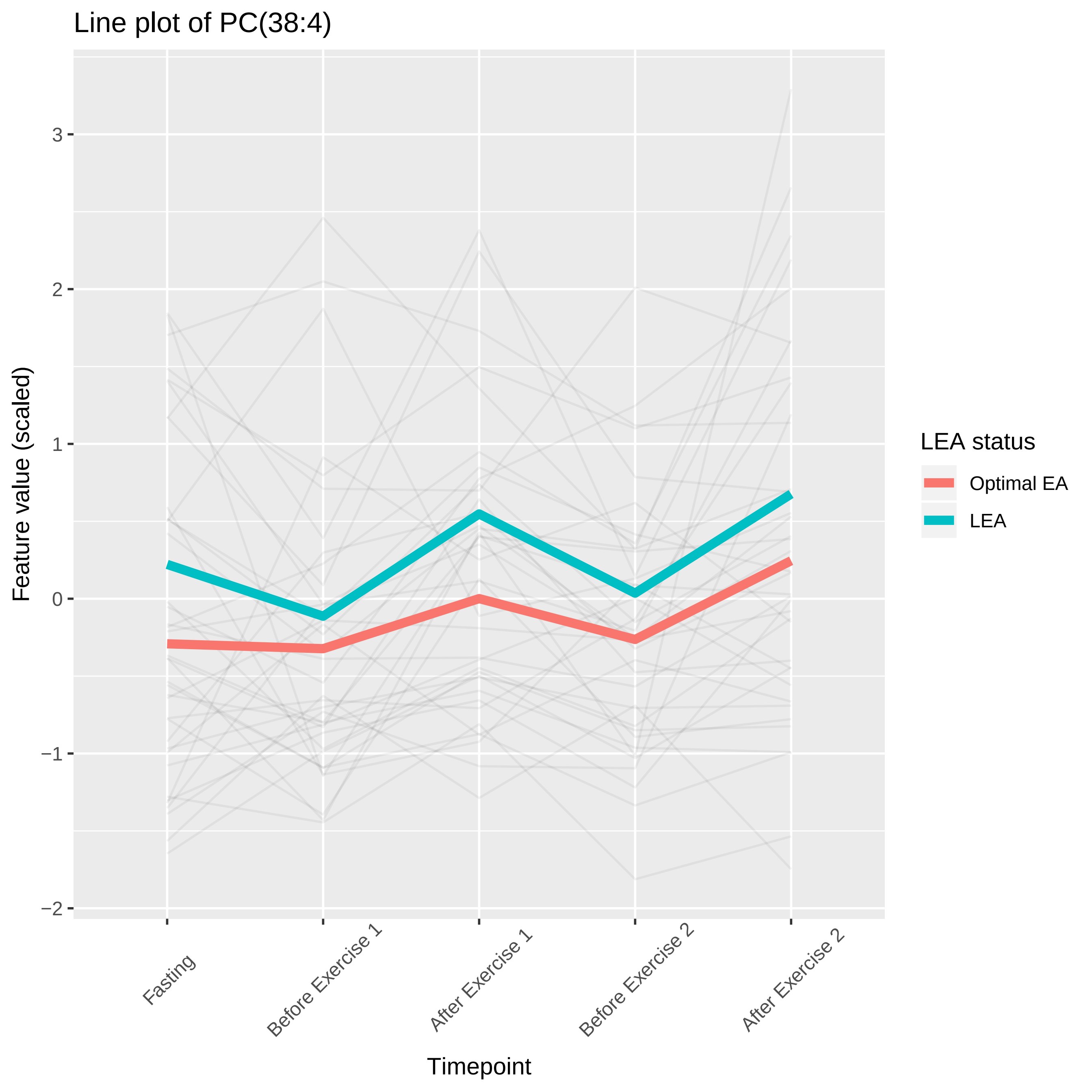

Line plot of PC(38:4)
Feature value (scaled)
3
2
1
0
−1
−2
Fasting
Before Exercise 1
After Exercise 1
Before Exercise 2
After Exercise 2
Timepoint
LEA status
Optimal EA
LEA

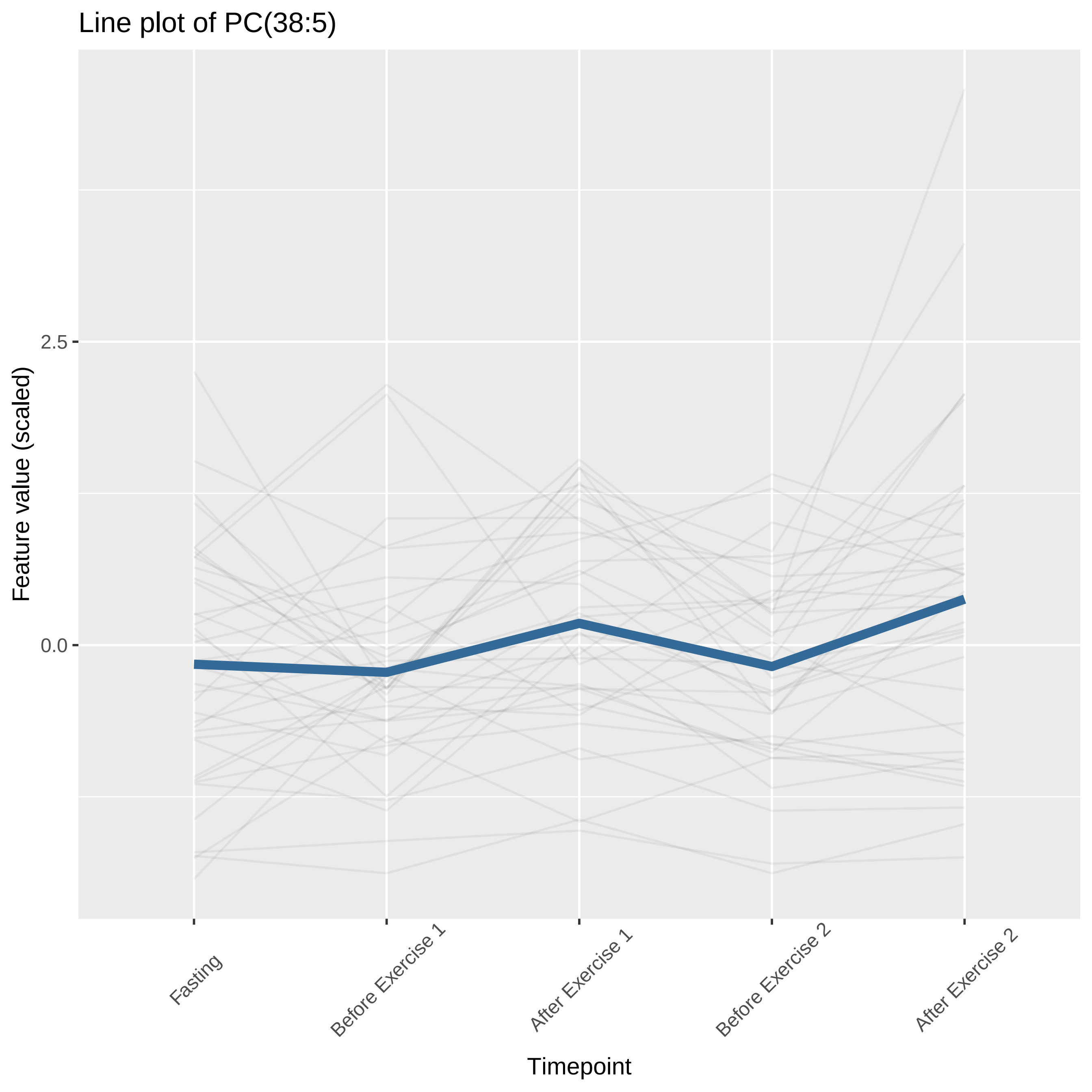
Line plot of PC(38:5)
Feature value (scaled)
2.5
0.0
Fasting
Before Exercise 1
After Exercise 1
Before Exercise 2
After Exercise 2
Timepoint

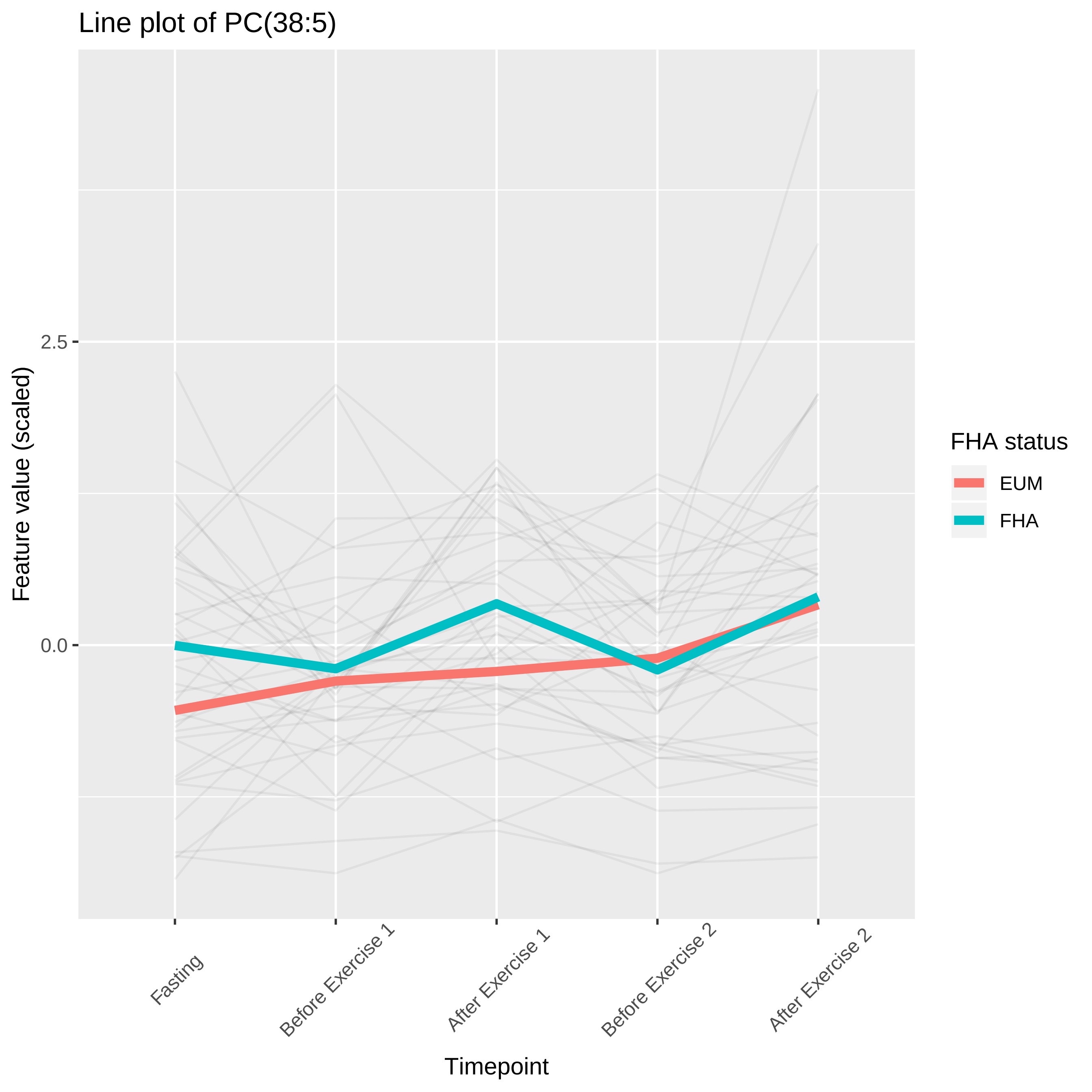
Line plot of PC(38:5)
Feature value (scaled)
2.5
0.0
Fasting
Before Exercise 1
After Exercise 1
Before Exercise 2
After Exercise 2
Timepoint
FHA status
EUM
FHA

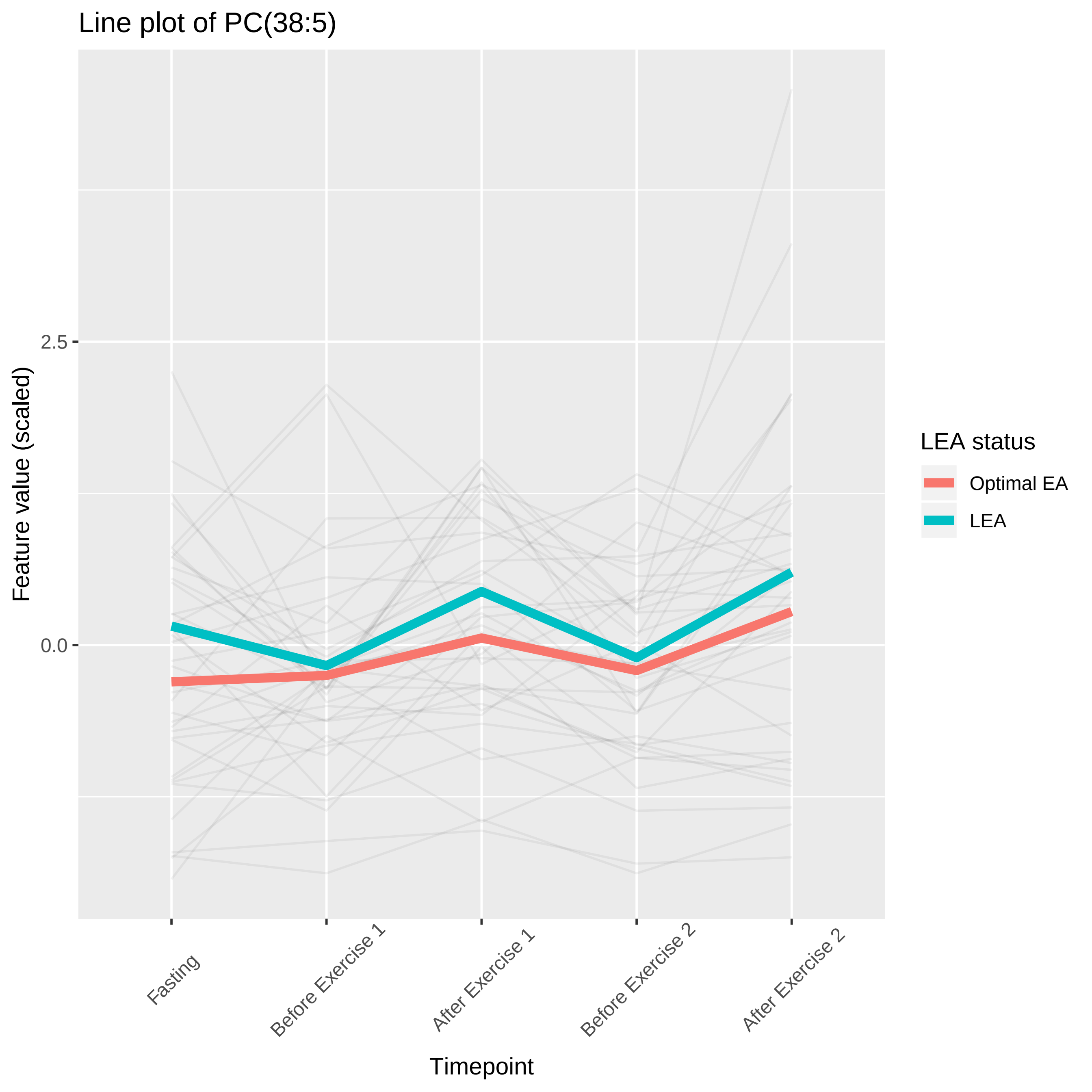

Line plot of PC(38:5)
Feature value (scaled)
2.5
0.0
Fasting
Before Exercise 1
After Exercise 1
Before Exercise 2
After Exercise 2
Timepoint
LEA status
Optimal EA
LEA

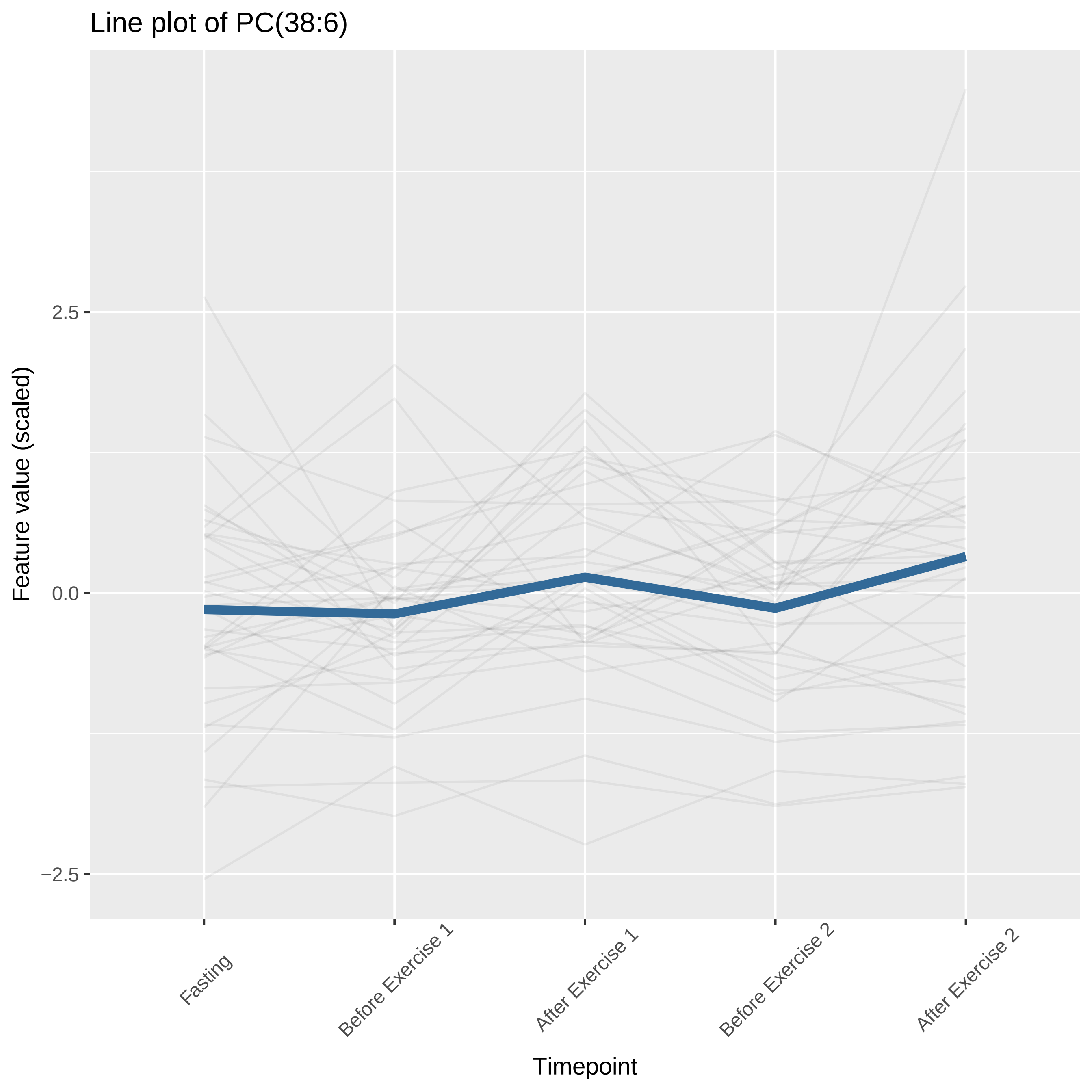

Line plot of PC(38:6)
2.5
0.0
−2.5
Feature value (scaled)
Fasting
Before Exercise 1
After Exercise 1
Before Exercise 2
After Exercise 2
Timepoint

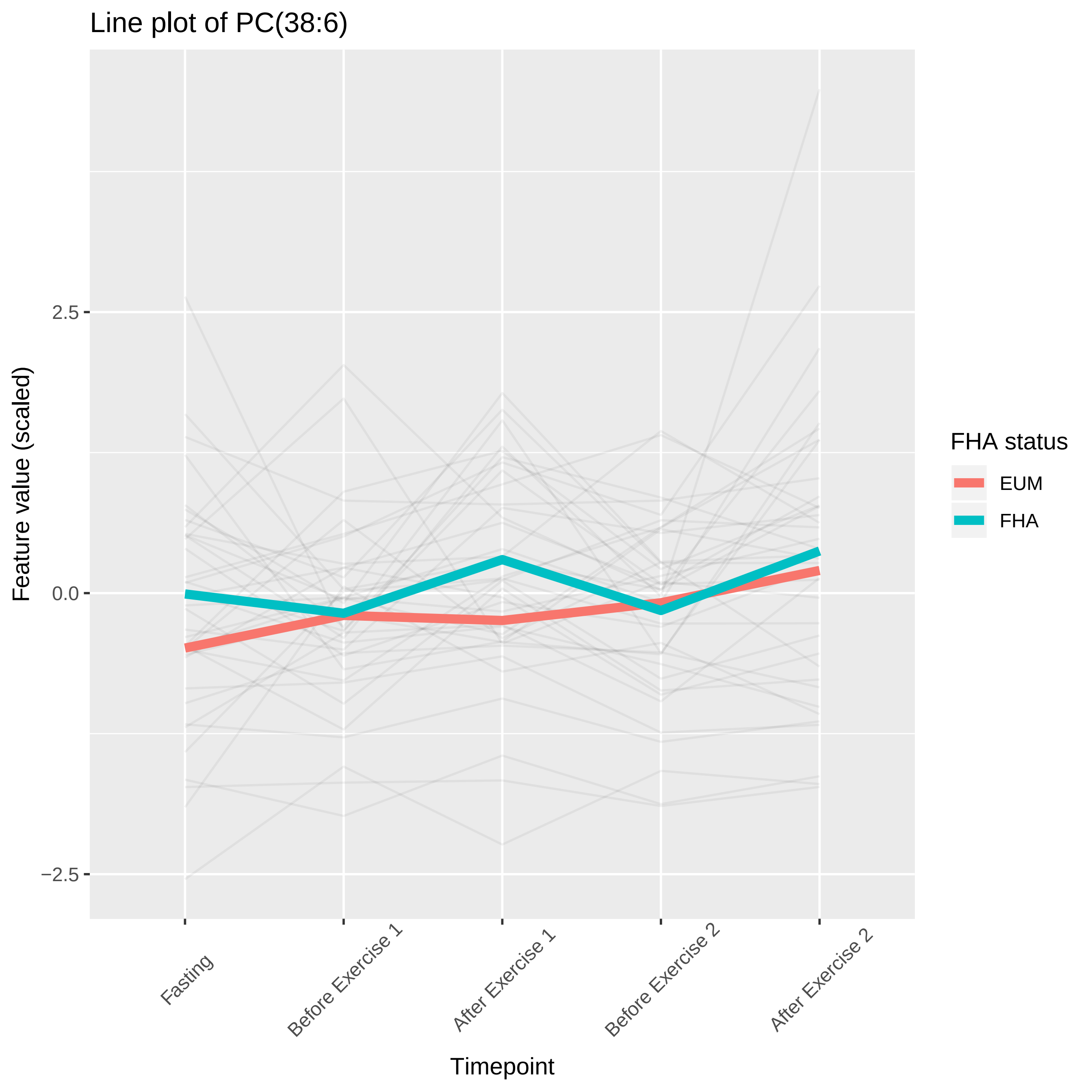
Line plot of PC(38:6)
Feature value (scaled)
2.5
0.0
−2.5
Fasting
Before Exercise 1
After Exercise 1
Before Exercise 2
After Exercise 2
Timepoint
FHA status
EUM
FHA

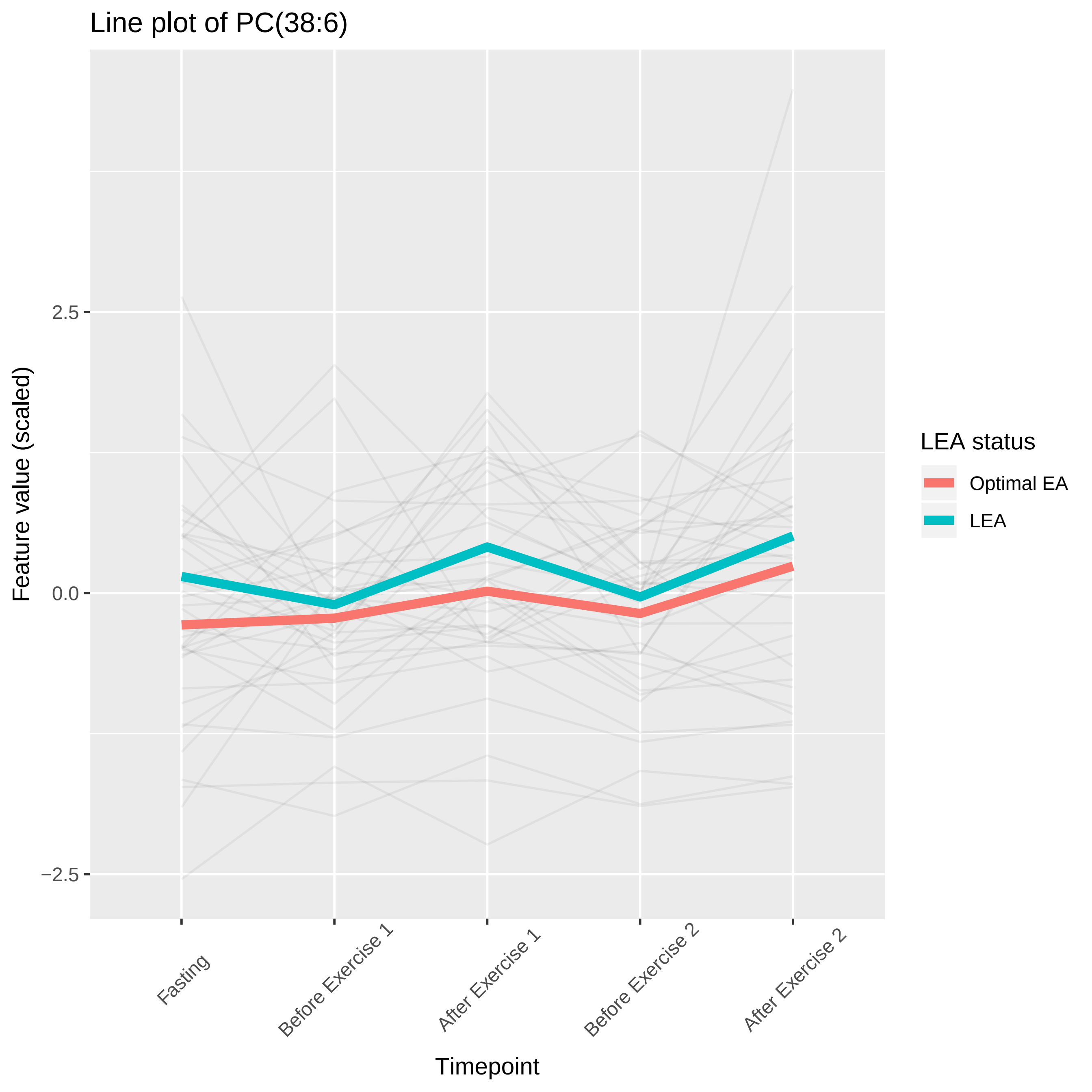

Line plot of PC(38:6)
Feature value (scaled)
2.5
0.0
−2.5
Fasting
Before Exercise 1
After Exercise 1
Before Exercise 2
After Exercise 2
Timepoint
LEA status
Optimal EA
LEA

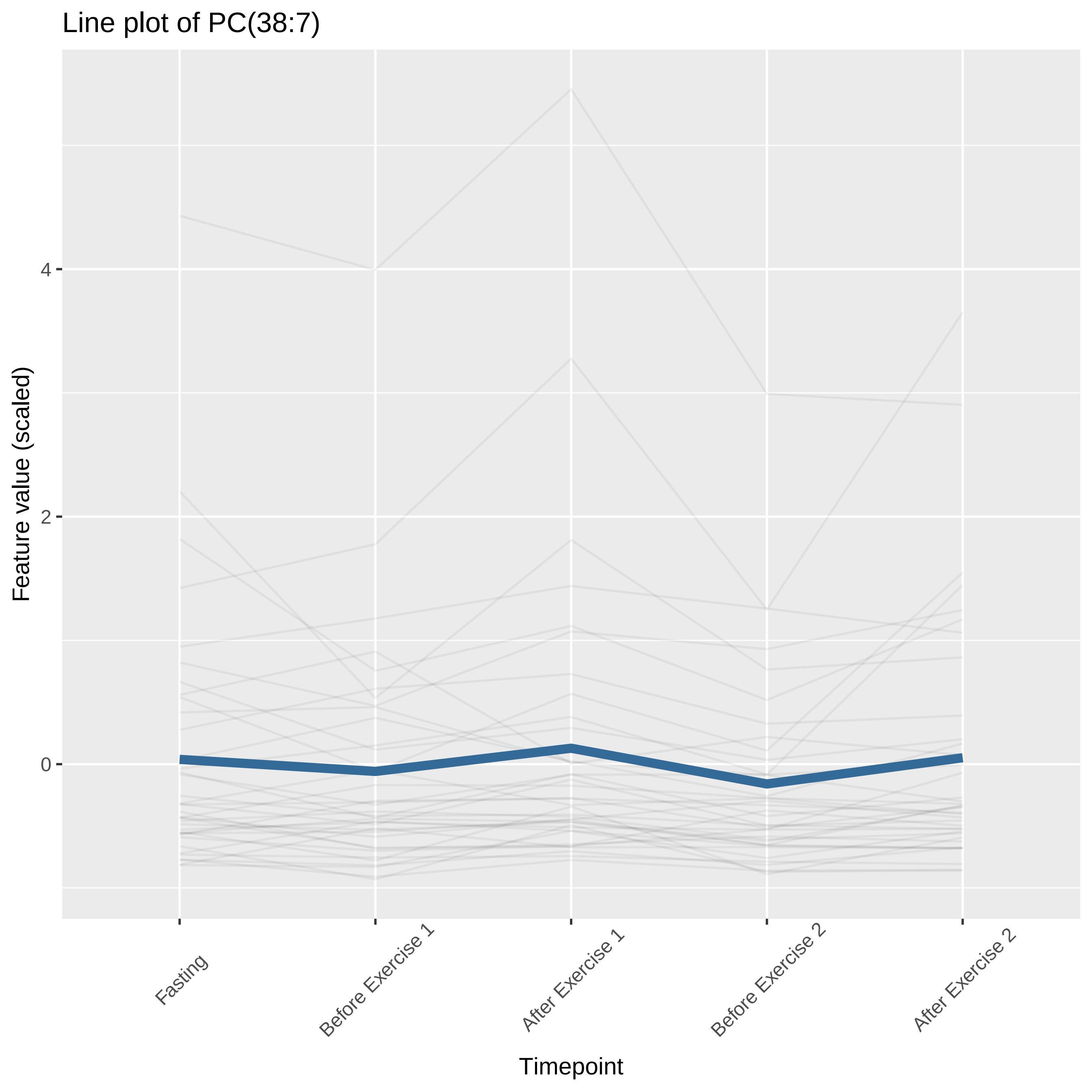

Line plot of PC(38:7)
Feature value (scaled)
4
2
0
Fasting
Before Exercise 1
After Exercise 1
Before Exercise 2
After Exercise 2
Timepoint

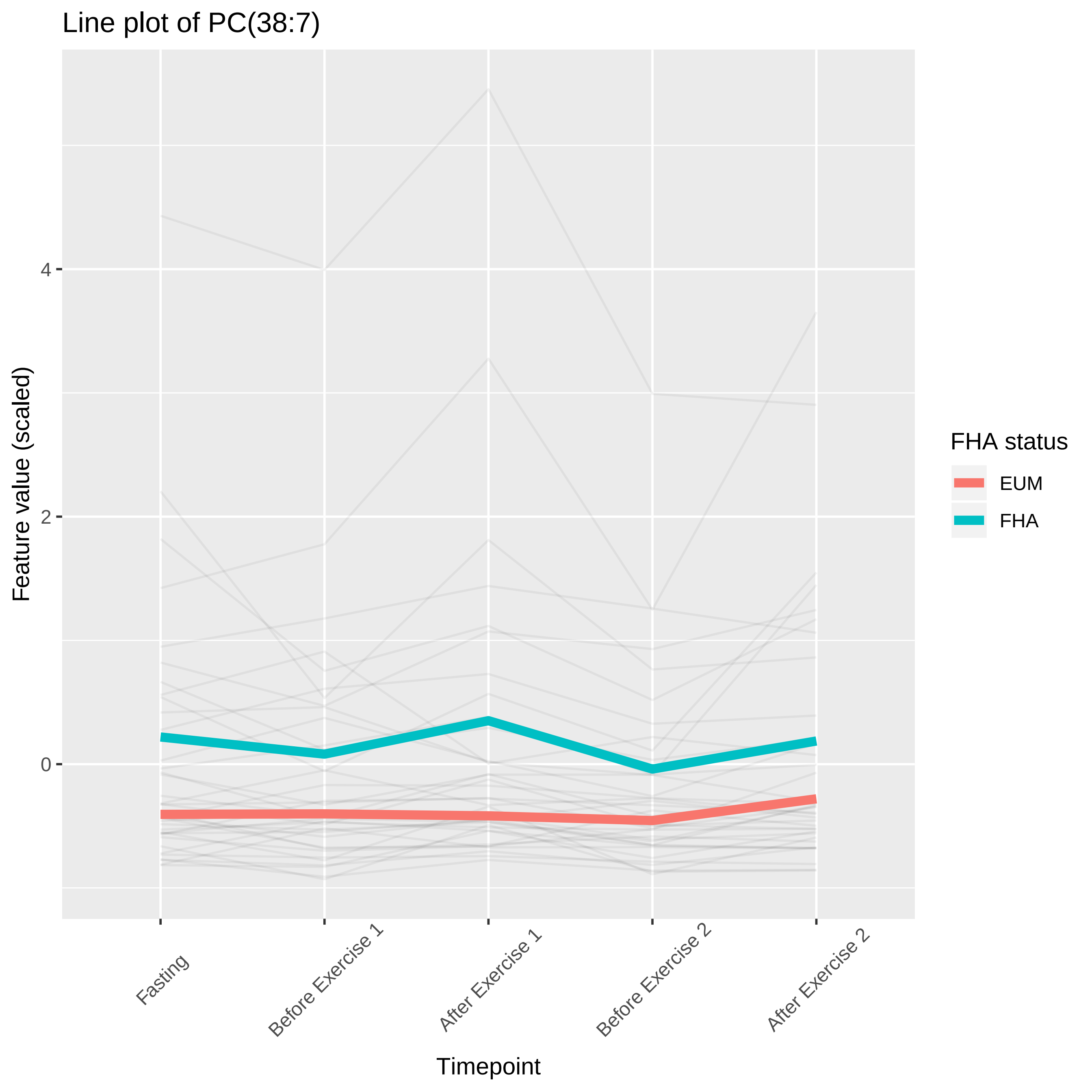

Line plot of PC(38:7)
Feature value (scaled)
4
2
0
Fasting
Before Exercise 1
After Exercise 1
Before Exercise 2
After Exercise 2
Timepoint
FHA status
EUM
FHA

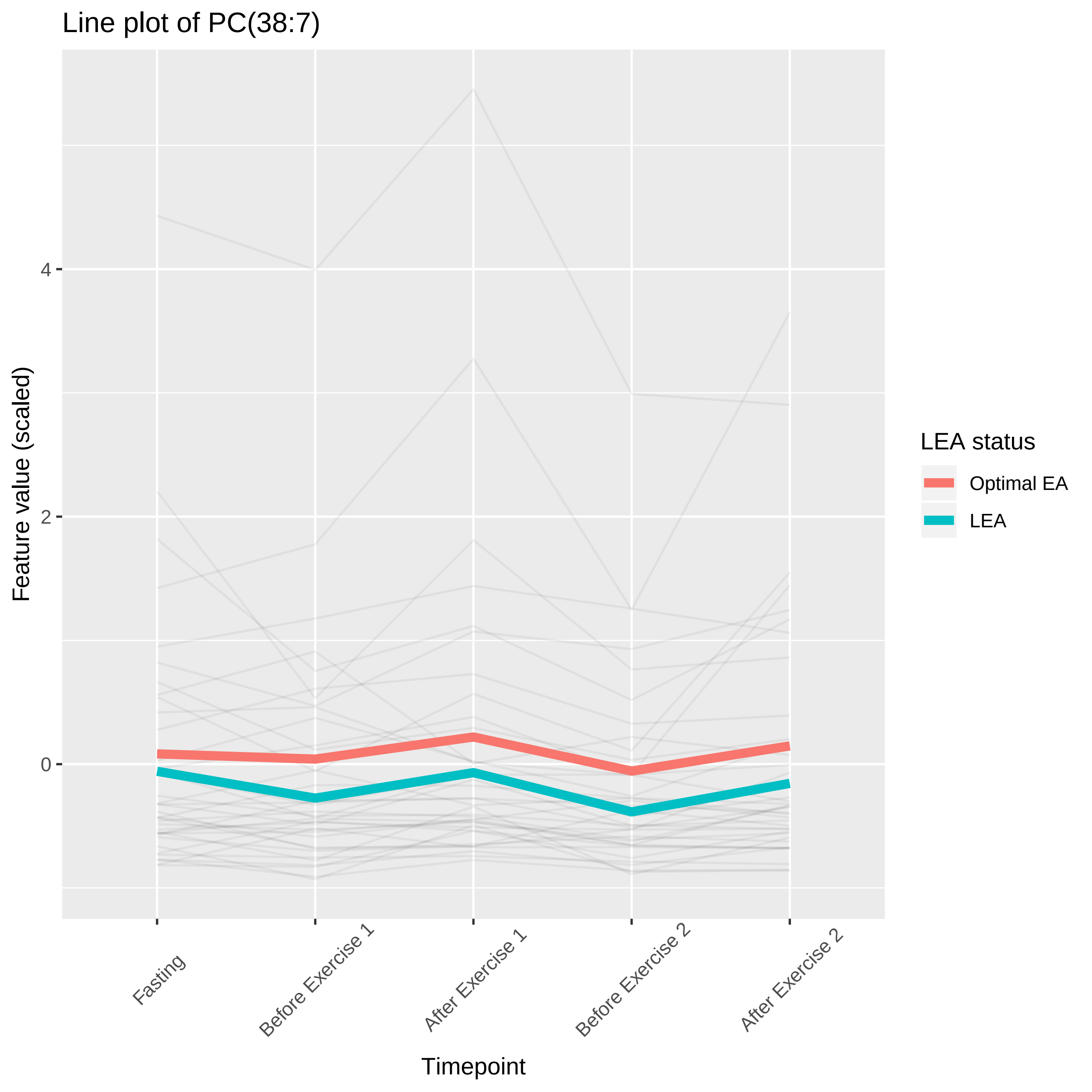

Line plot of PC(38:7)
Feature value (scaled)
4
2
0
Fasting
Before Exercise 1
After Exercise 1
Before Exercise 2
After Exercise 2
Timepoint
LEA status
Optimal EA
LEA

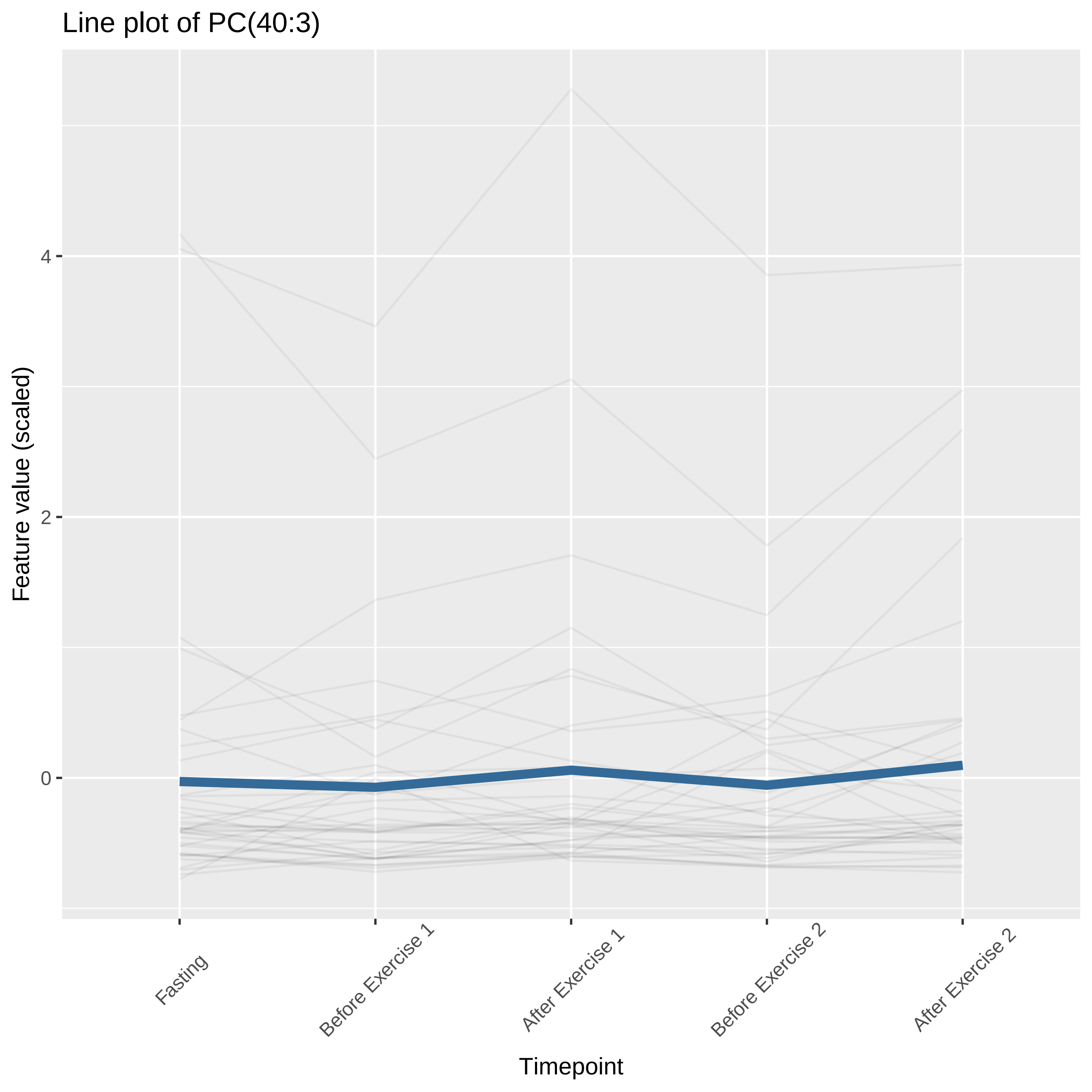
Line plot of PC(40:3)
Feature value (scaled)
4
2
0
Fasting
Before Exercise 1
After Exercise 1
Before Exercise 2
After Exercise 2
Timepoint

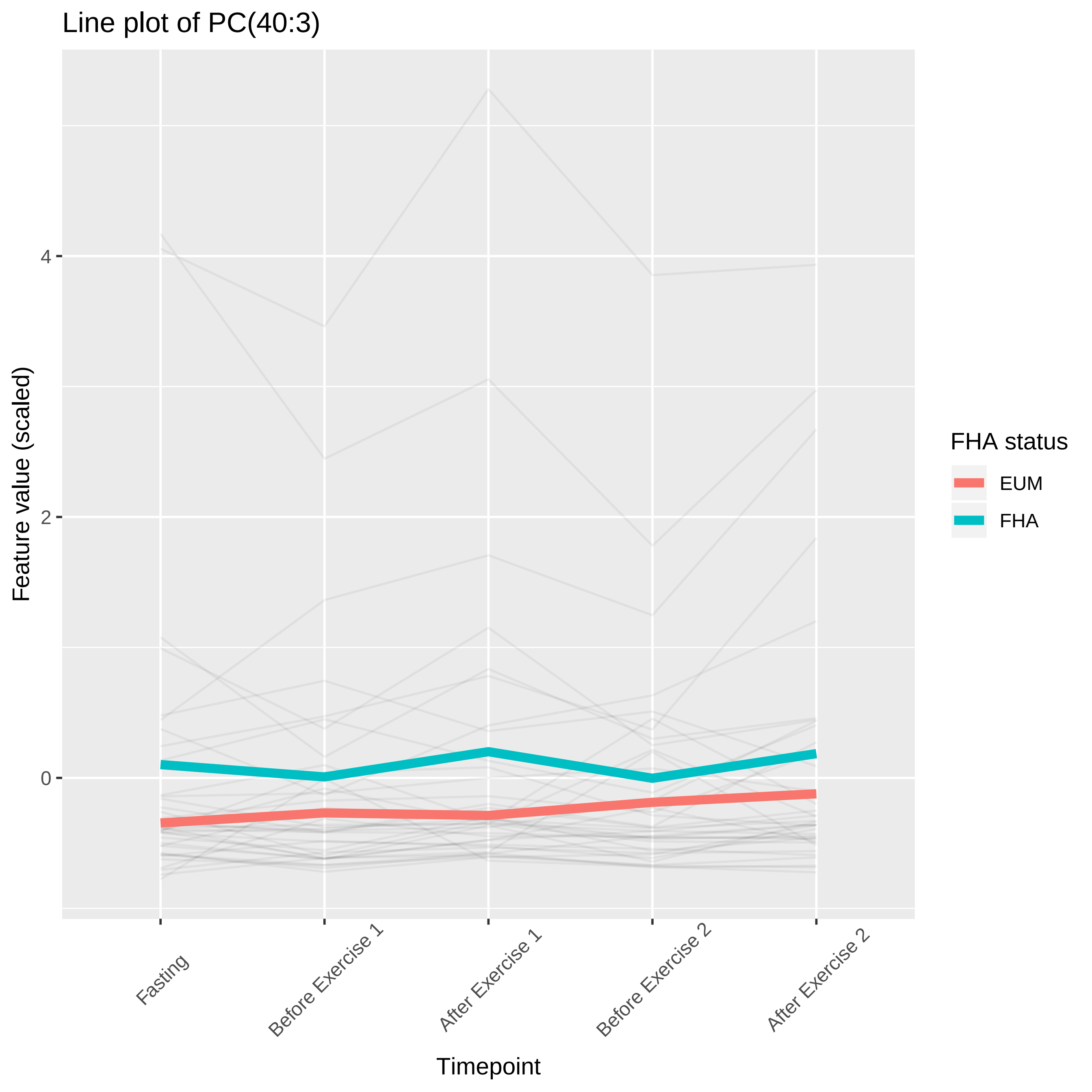

Line plot of PC(40:3)
Feature value (scaled)
4
2
0
Fasting
Before Exercise 1
After Exercise 1
Before Exercise 2
After Exercise 2
Timepoint
FHA status
EUM
FHA

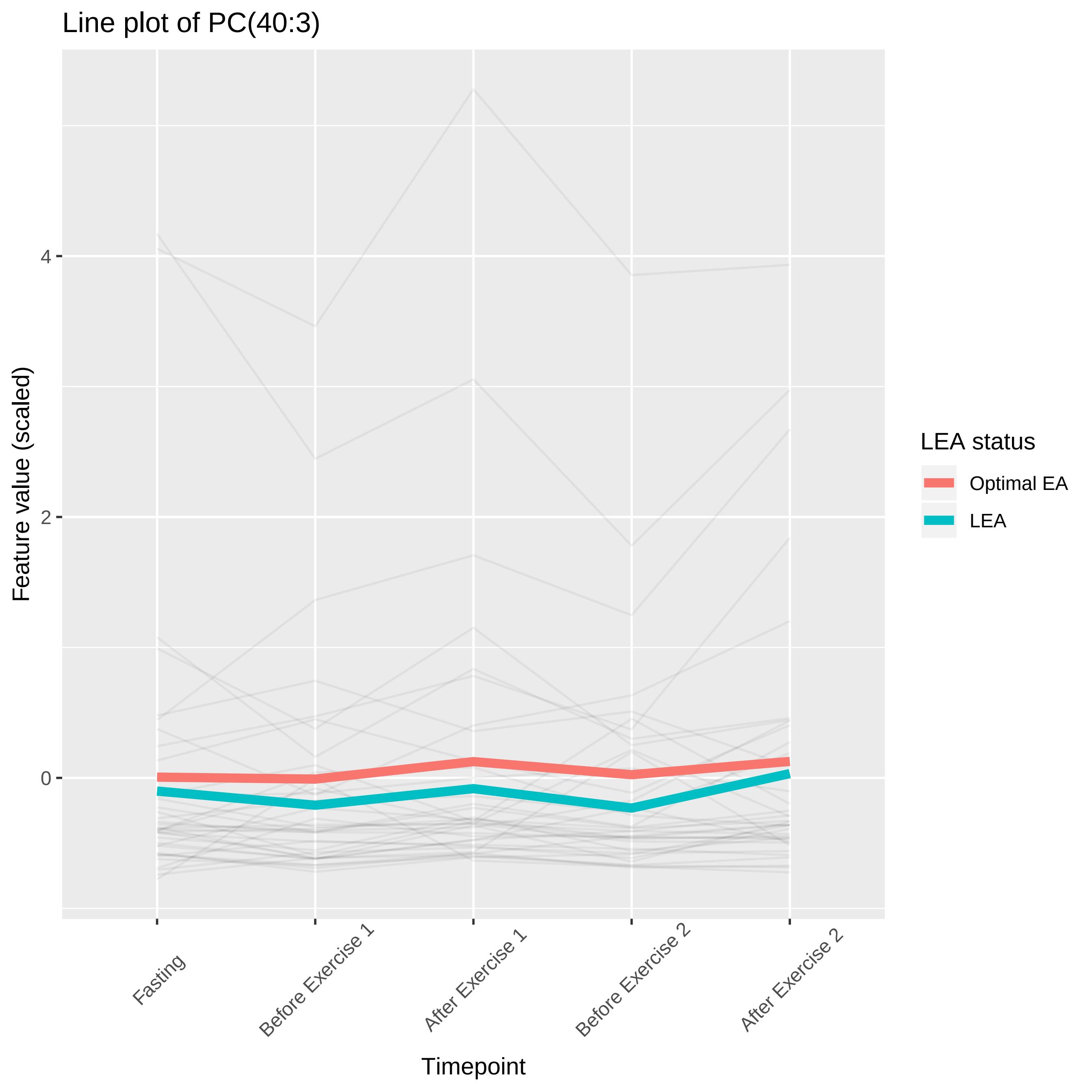

Line plot of PC(40:3)
Feature value (scaled)
4
2
0
Fasting
Before Exercise 1
After Exercise 1
Before Exercise 2
After Exercise 2
Timepoint
LEA status
Optimal EA
LEA

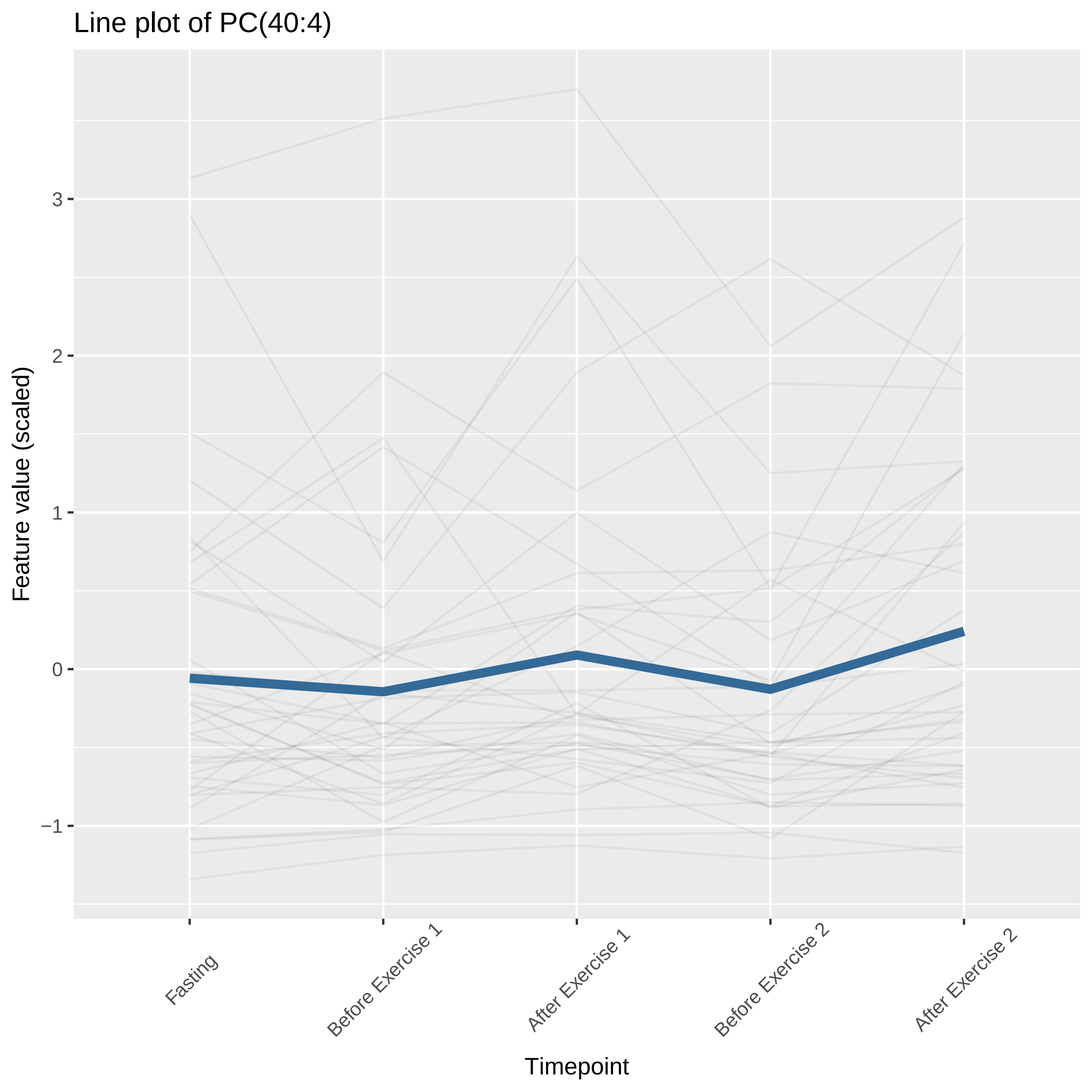

Line plot of PC(40:4)
Feature value (scaled)
3
2
1
0
−1
Fasting
Before Exercise 1
After Exercise 1
Before Exercise 2
After Exercise 2
Timepoint

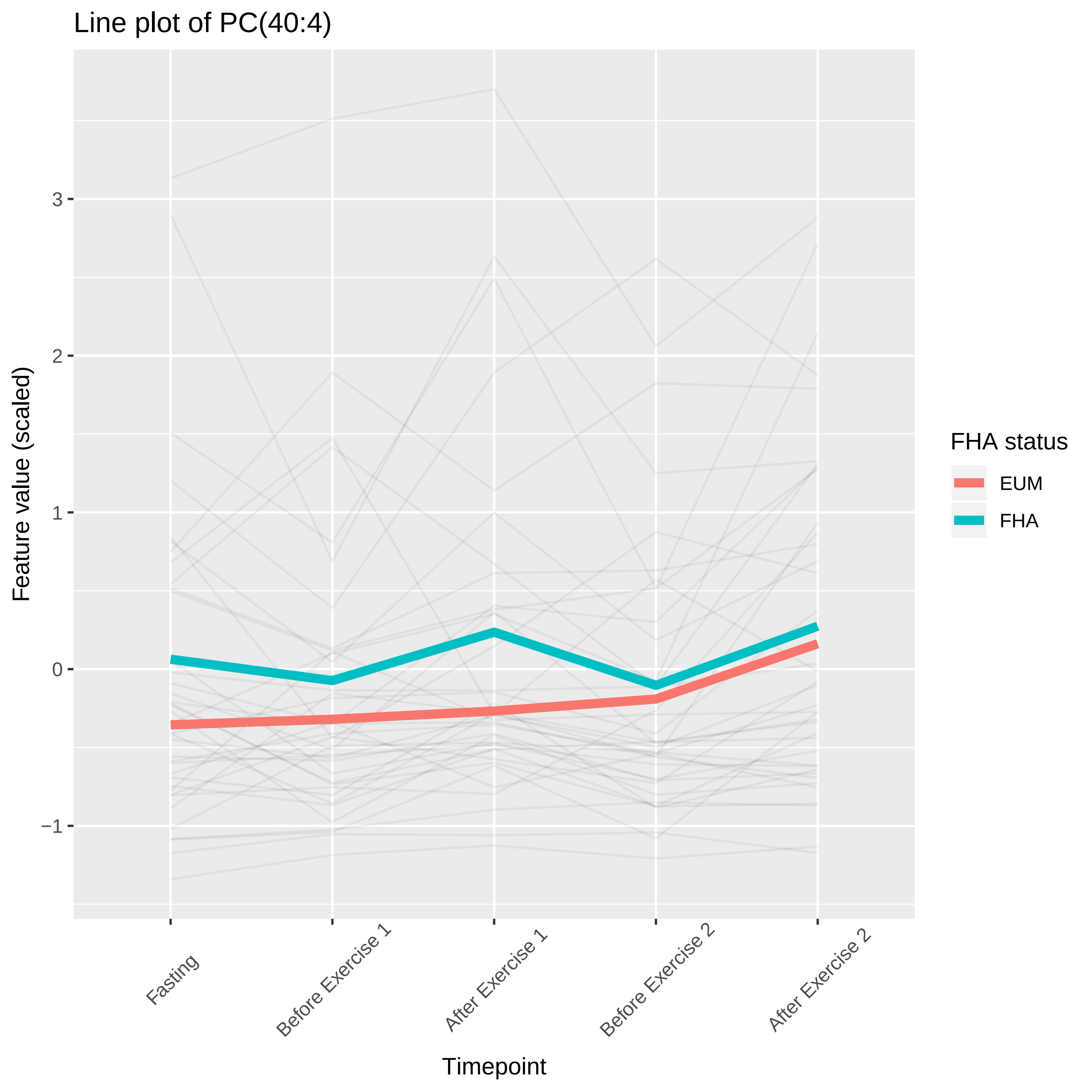

Line plot of PC(40:4)
Feature value (scaled)
3
2
1
0
−1
Fasting
Before Exercise 1
After Exercise 1
Before Exercise 2
After Exercise 2
Timepoint
FHA status
EUM
FHA

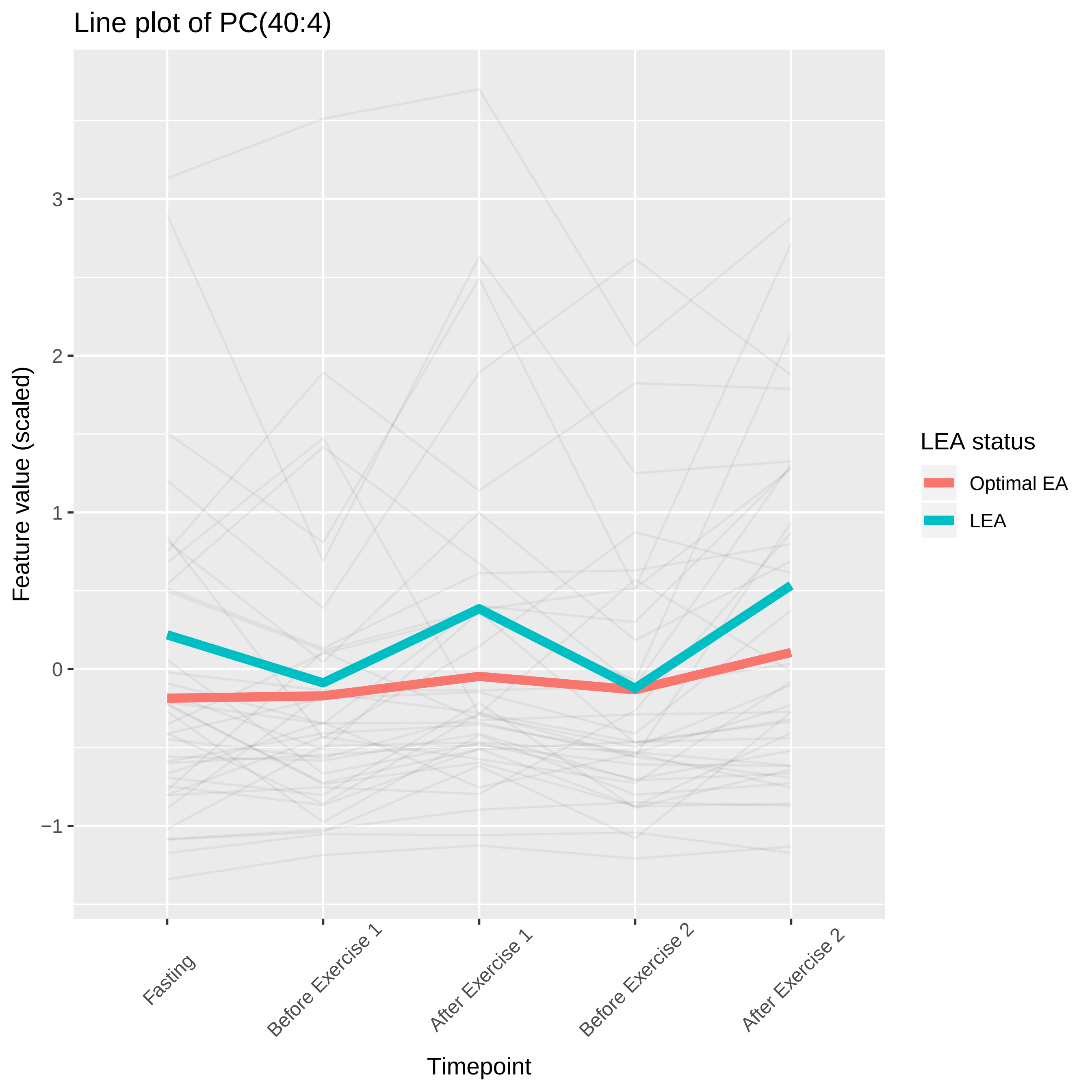

Line plot of PC(40:4)
Feature value (scaled)
3
2
1
0
−1
Fasting
Before Exercise 1
After Exercise 1
Before Exercise 2
After Exercise 2
Timepoint
LEA status
Optimal EA
LEA

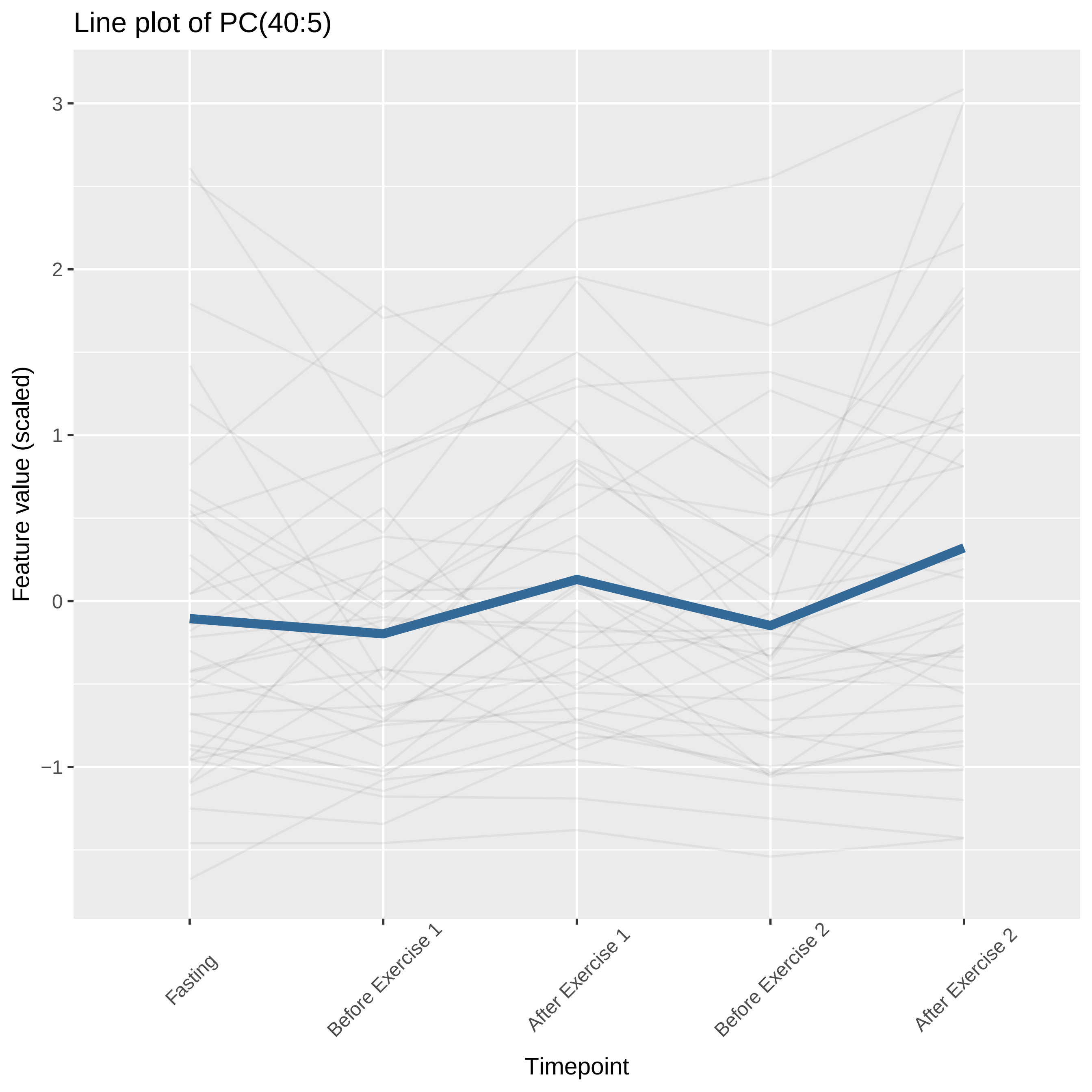

Line plot of PC(40:5)
Feature value (scaled)
3
2
1
0
−1
Fasting
Before Exercise 1
After Exercise 1
Before Exercise 2
After Exercise 2
Timepoint

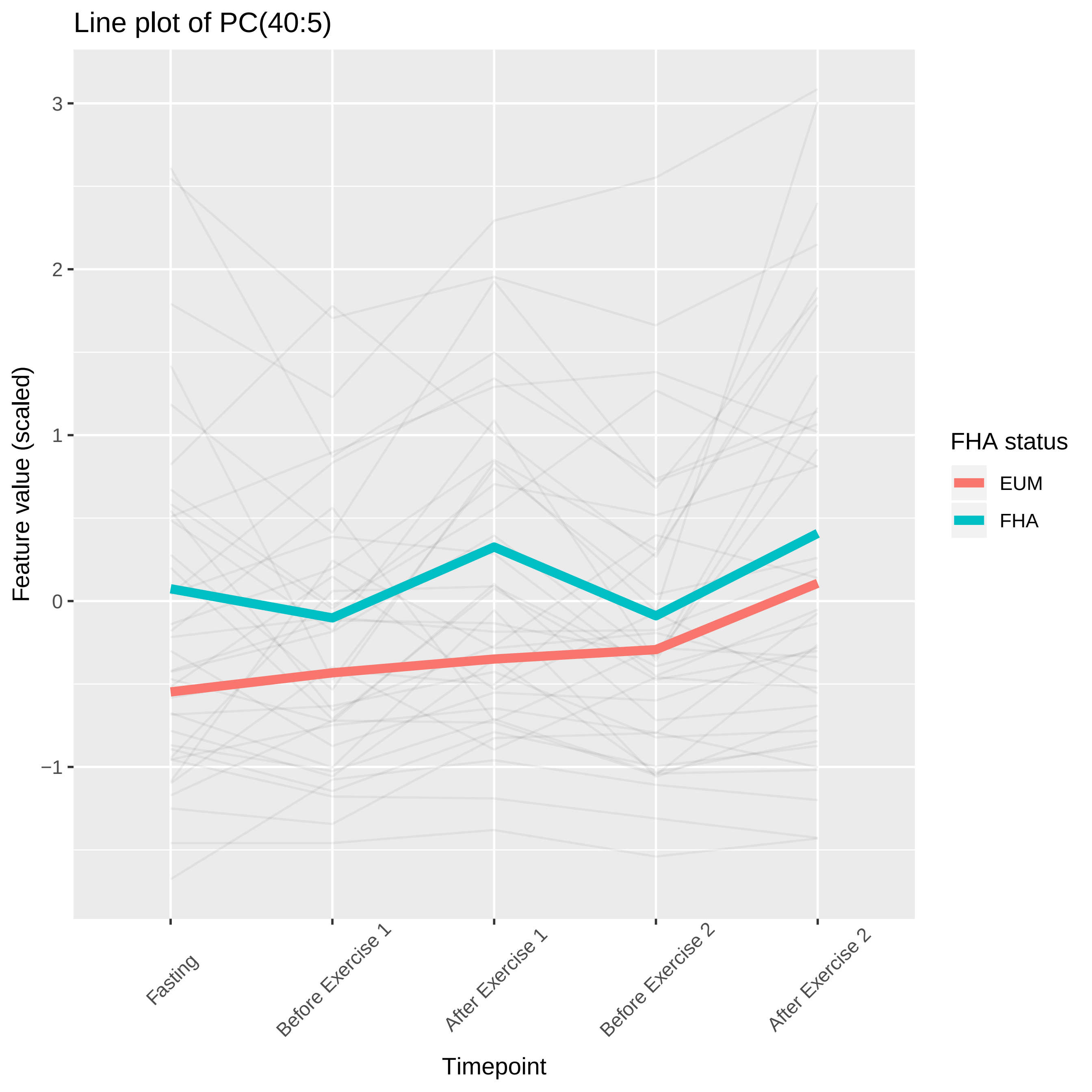
Line plot of PC(40:5)
Feature value (scaled)
3
2
1
0
−1
Fasting
Before Exercise 1
After Exercise 1
Before Exercise 2
After Exercise 2
Timepoint
FHA status
EUM
FHA

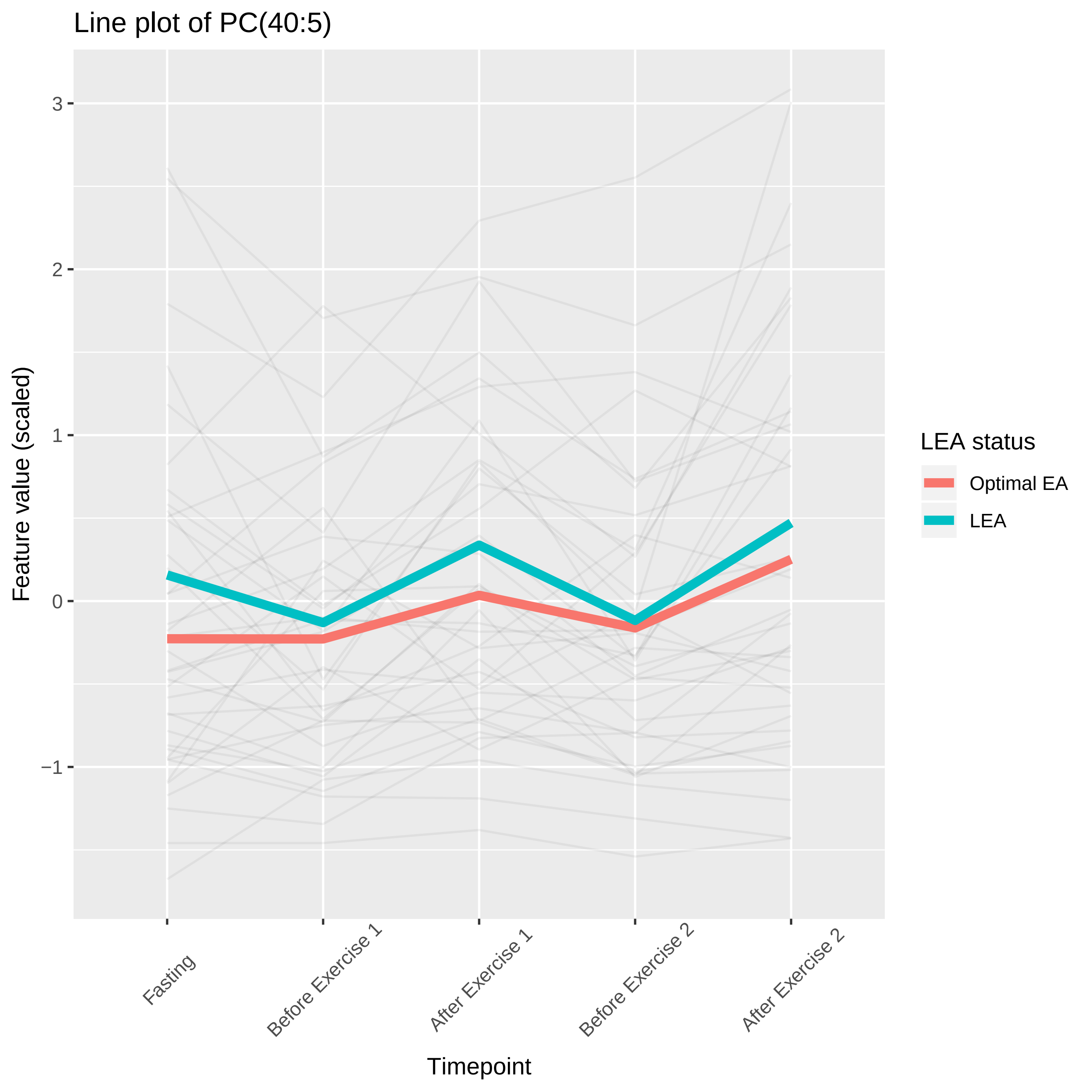

Line plot of PC(40:5)
Feature value (scaled)
3
2
1
0
−1
Fasting
Before Exercise 1
After Exercise 1
Before Exercise 2
After Exercise 2
Timepoint
LEA status
Optimal EA
LEA

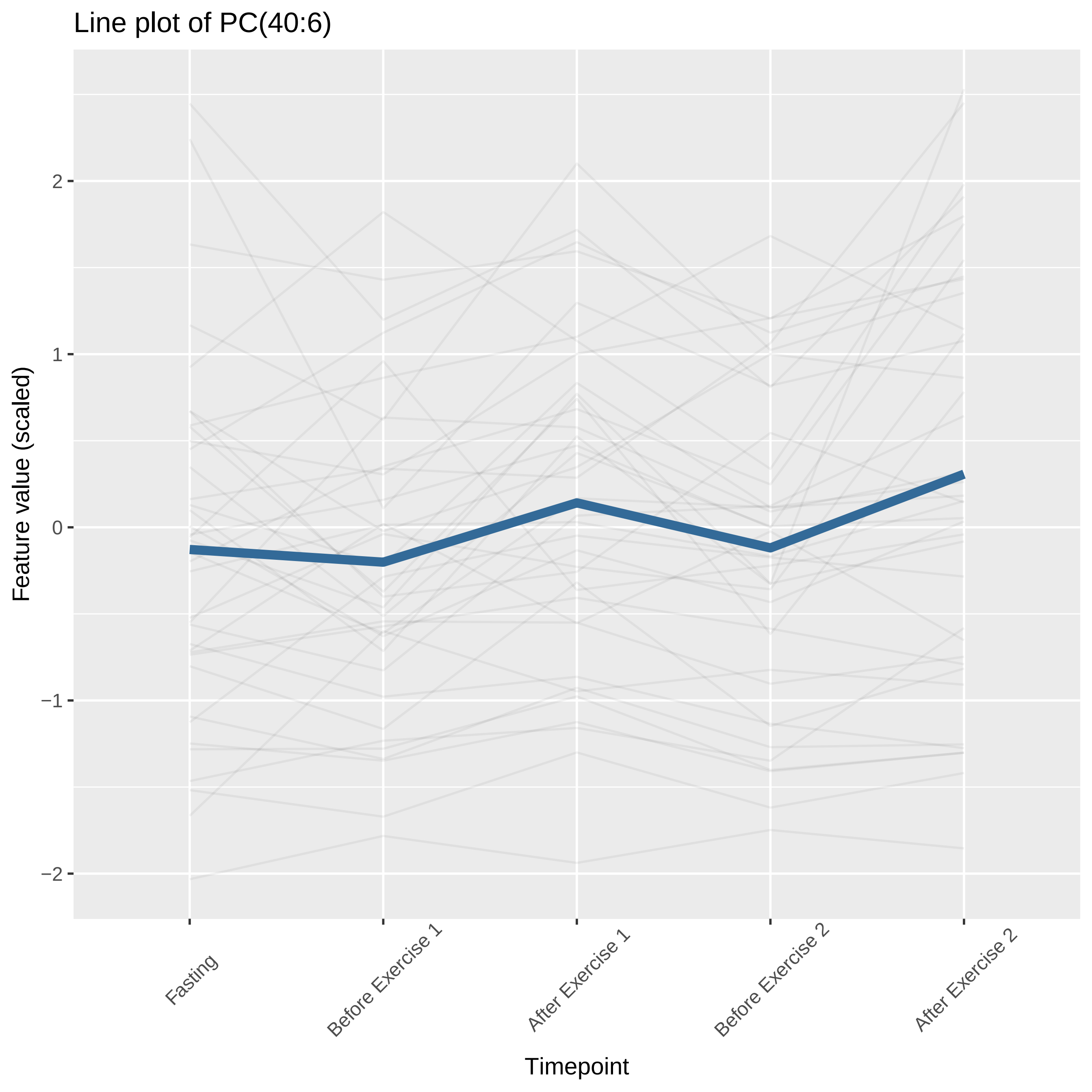

Line plot of PC(40:6)
Feature value (scaled)
2
1
0
−1
−2
Fasting
Before Exercise 1
After Exercise 1
Before Exercise 2
After Exercise 2
Timepoint

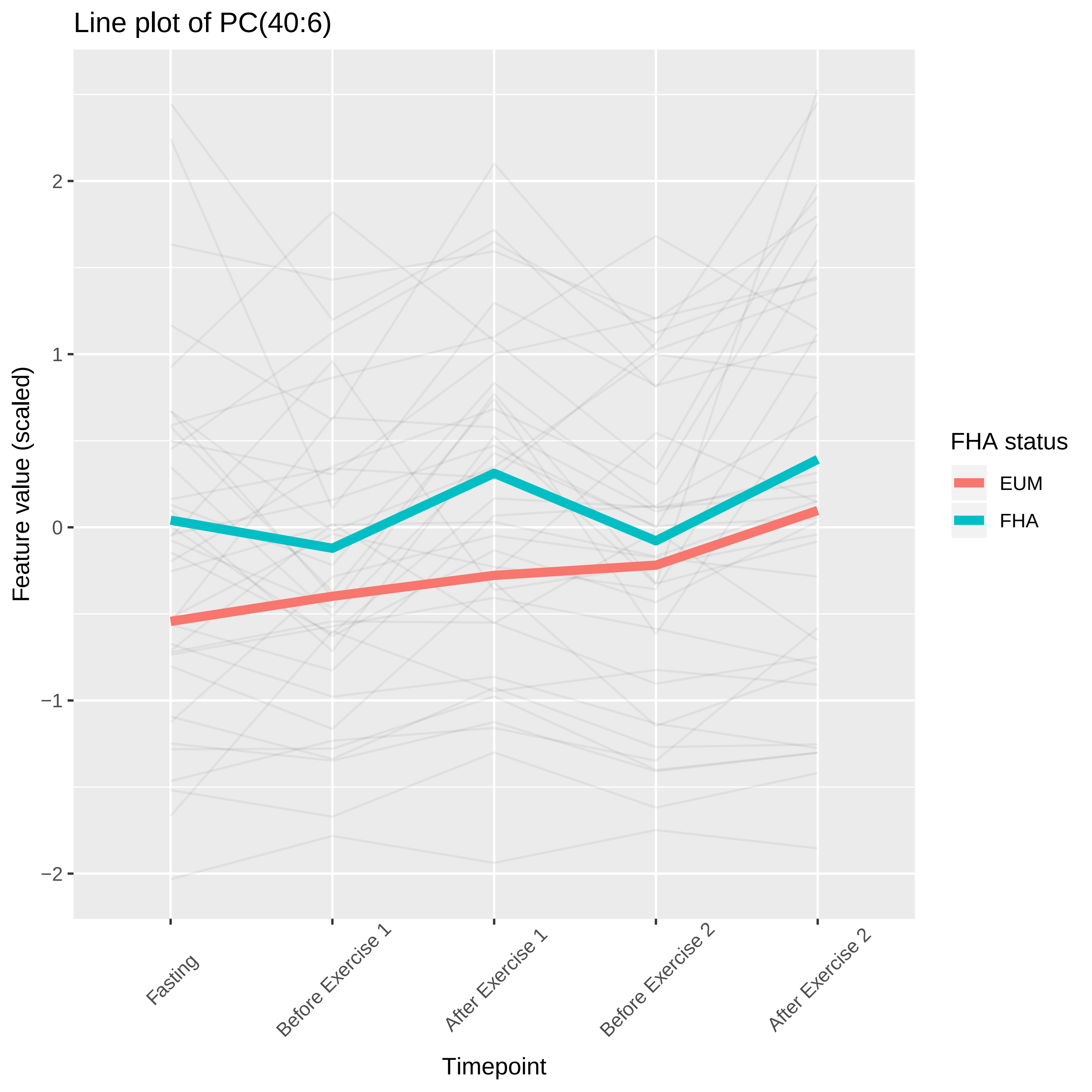

Line plot of PC(40:6)
Feature value (scaled)
2
1
0
−1
−2
Fasting
Before Exercise 1
After Exercise 1
Before Exercise 2
After Exercise 2
Timepoint
FHA status
EUM
FHA

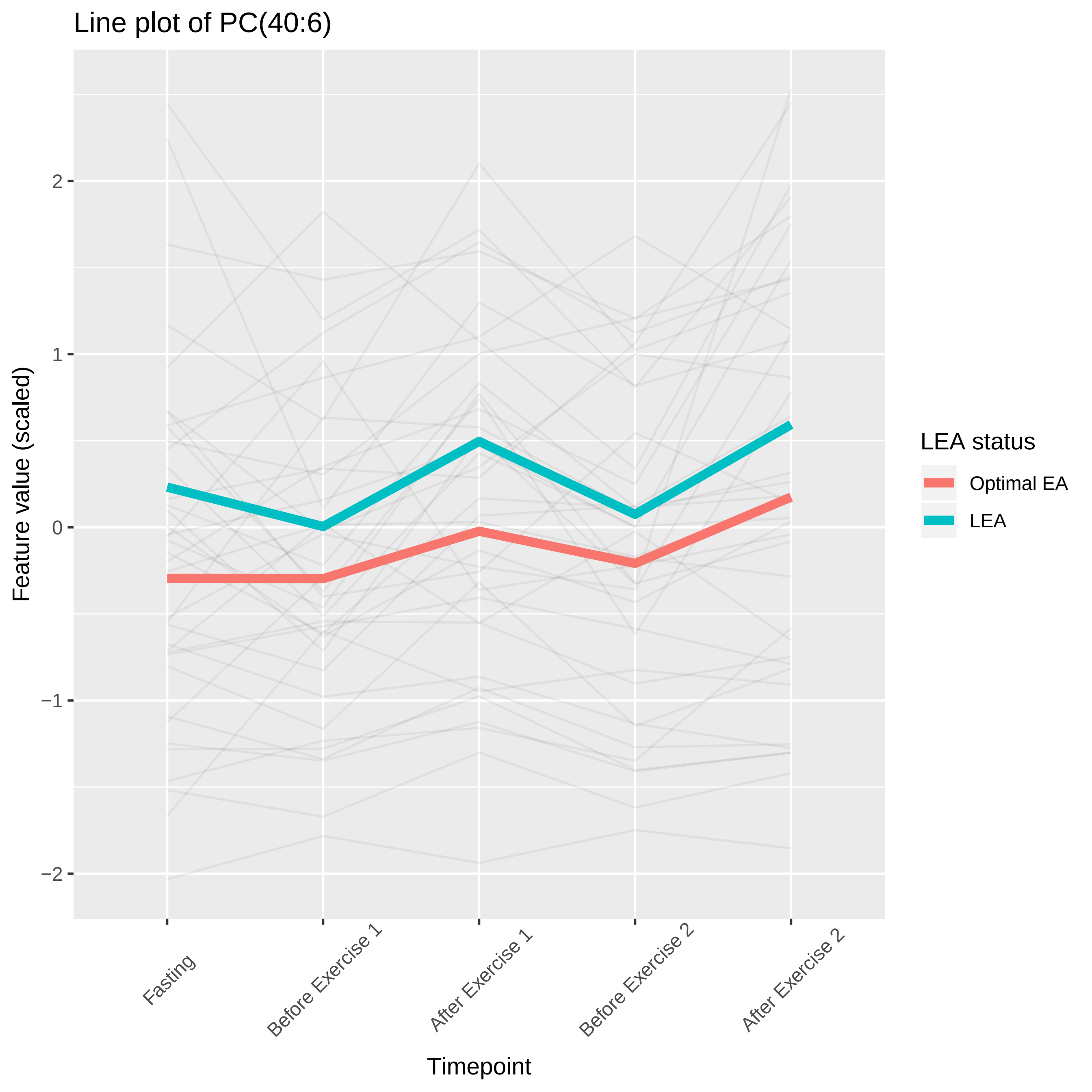

Line plot of PC(40:6)
Feature value (scaled)
2
1
0
−1
−2
Fasting
Before Exercise 1
After Exercise 1
Before Exercise 2
After Exercise 2
Timepoint
LEA status
Optimal EA
LEA

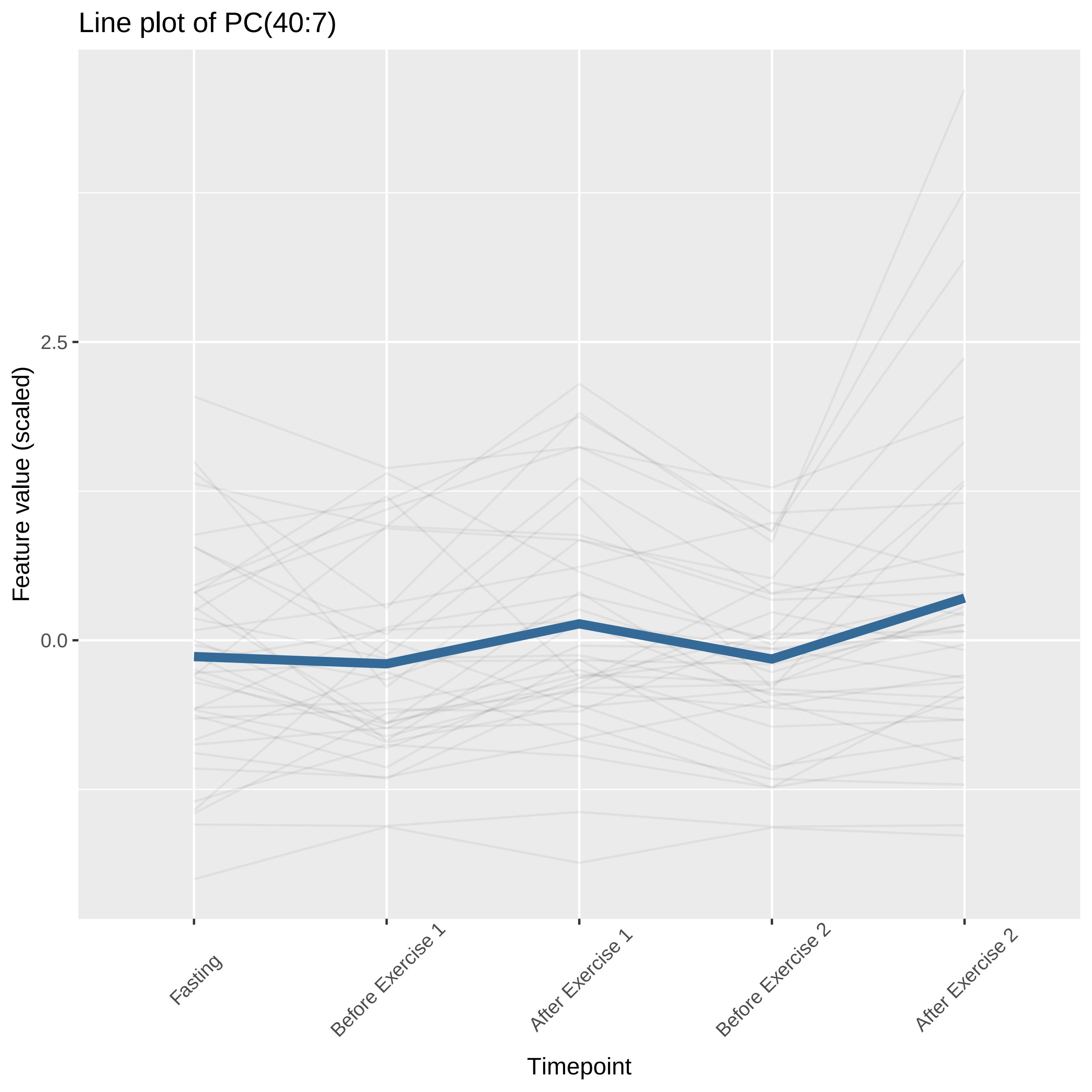

Line plot of PC(40:7)
Feature value (scaled)
2.5
0.0
Fasting
Before Exercise 1
After Exercise 1
Before Exercise 2
After Exercise 2
Timepoint

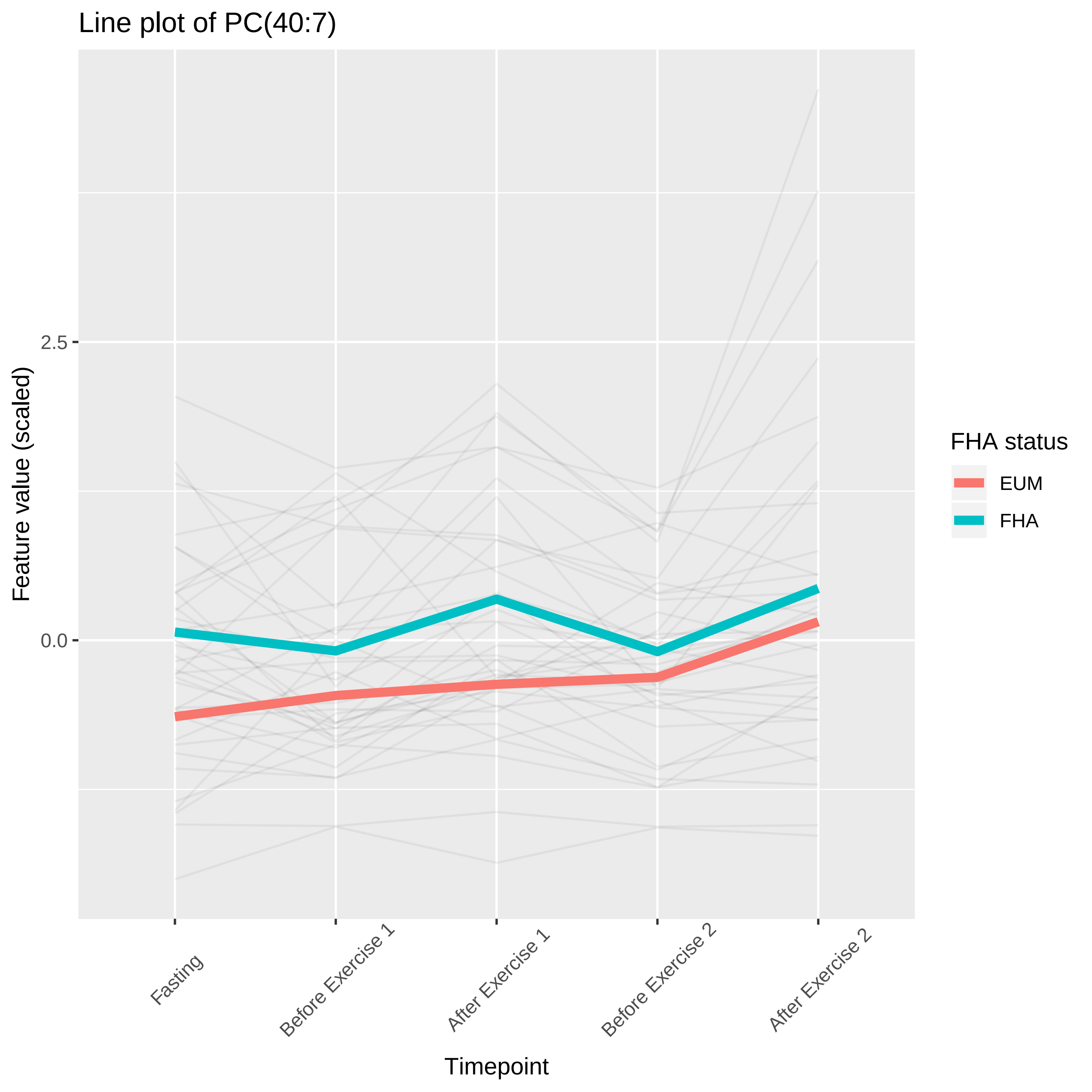
Line plot of PC(40:7)
Feature value (scaled)
2.5
0.0
Fasting
Before Exercise 1
After Exercise 1
Before Exercise 2
After Exercise 2
Timepoint
FHA status
EUM
FHA

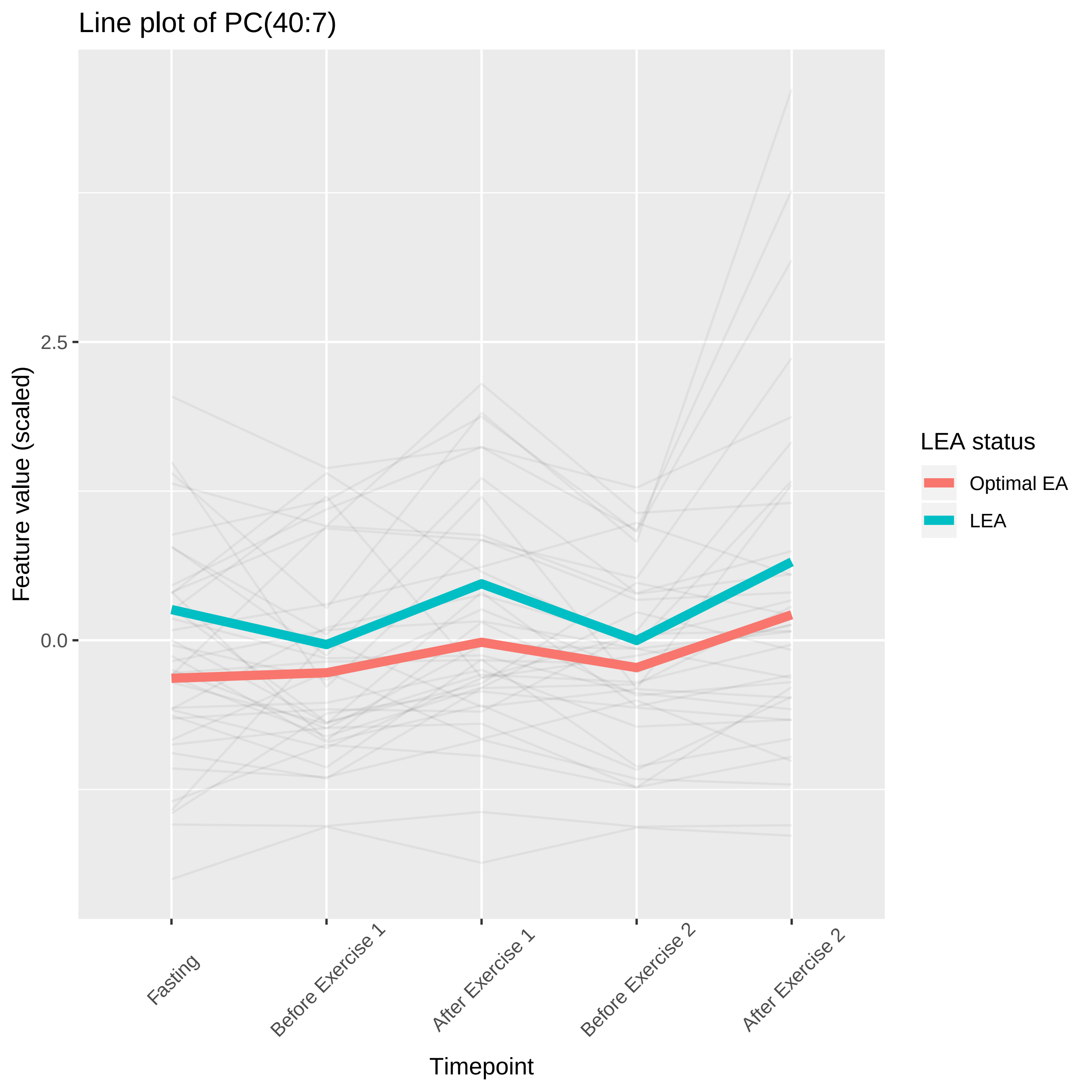

Line plot of PC(40:7)
Feature value (scaled)
2.5
0.0
Fasting
Before Exercise 1
After Exercise 1
Before Exercise 2
After Exercise 2
Timepoint
LEA status
Optimal EA
LEA

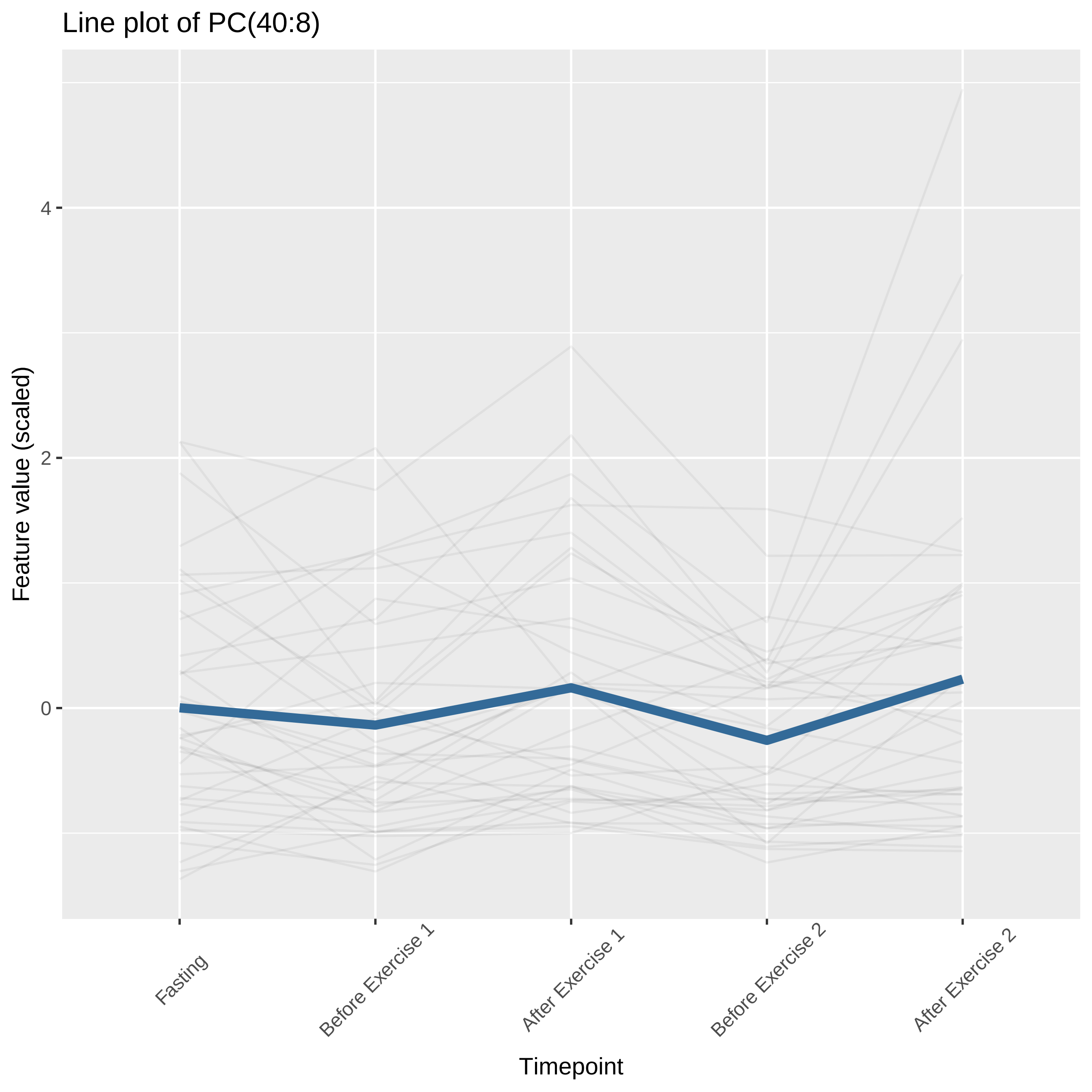

Line plot of PC(40:8)
Feature value (scaled)
4
2
0
Fasting
Before Exercise 1
After Exercise 1
Before Exercise 2
After Exercise 2
Timepoint

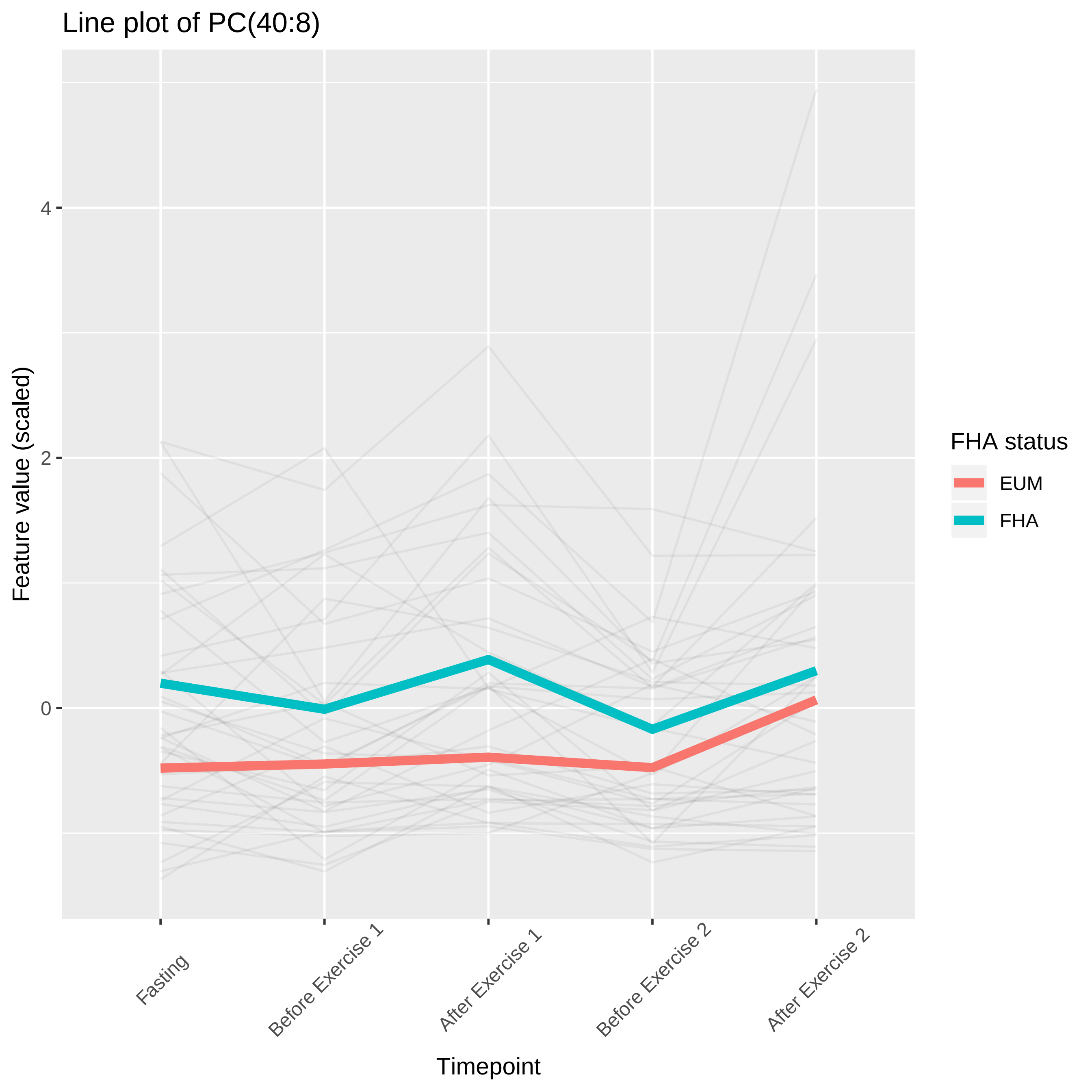

Line plot of PC(40:8)
Feature value (scaled)
4
2
0
Fasting
Before Exercise 1
After Exercise 1
Before Exercise 2
After Exercise 2
Timepoint
FHA status
EUM
FHA

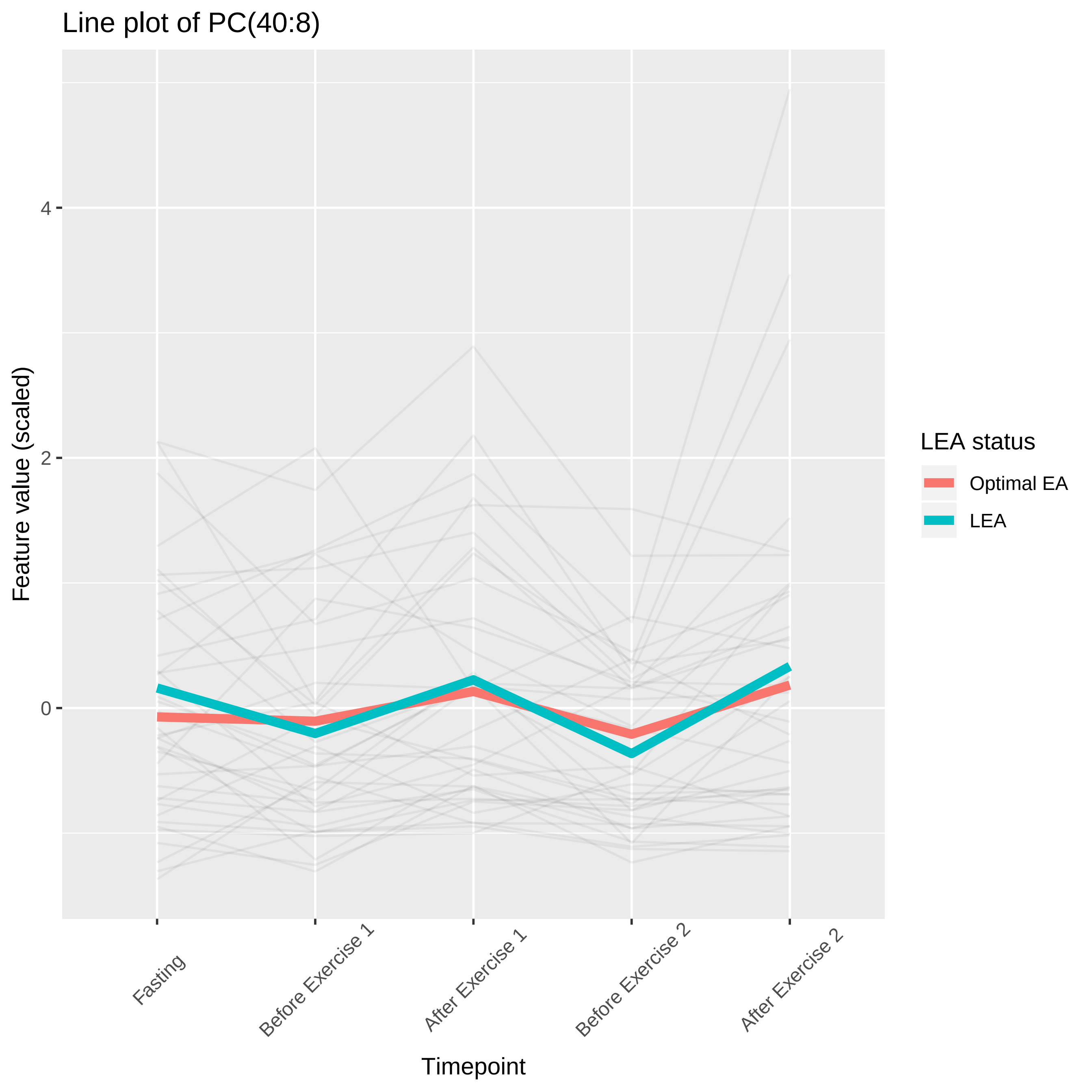

Line plot of PC(40:8)
Feature value (scaled)
4
2
0
Fasting
Before Exercise 1
After Exercise 1
Before Exercise 2
After Exercise 2
Timepoint
LEA status
Optimal EA
LEA

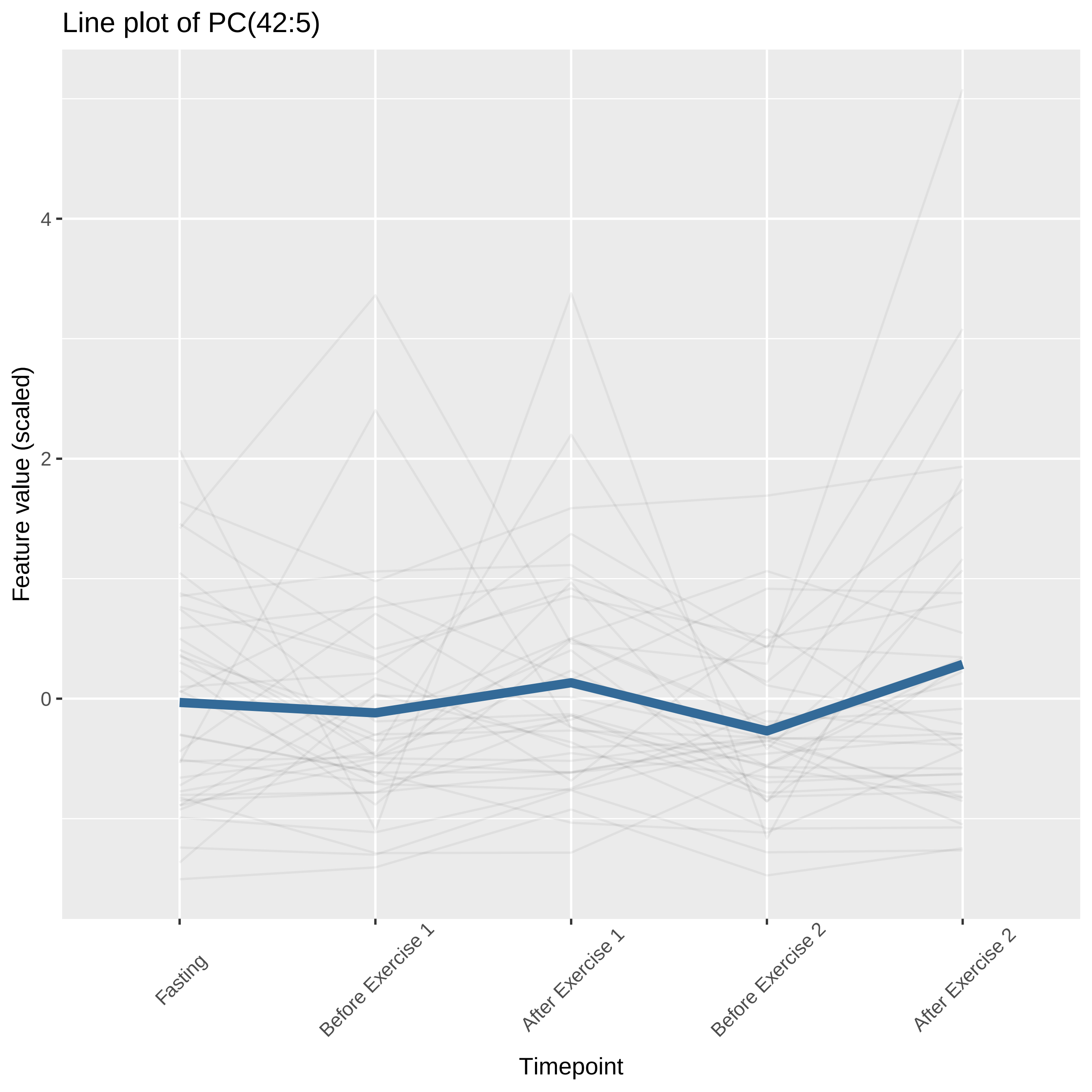

Line plot of PC(42:5)
Feature value (scaled)
4
2
0
Fasting
Before Exercise 1
After Exercise 1
Before Exercise 2
After Exercise 2
Timepoint

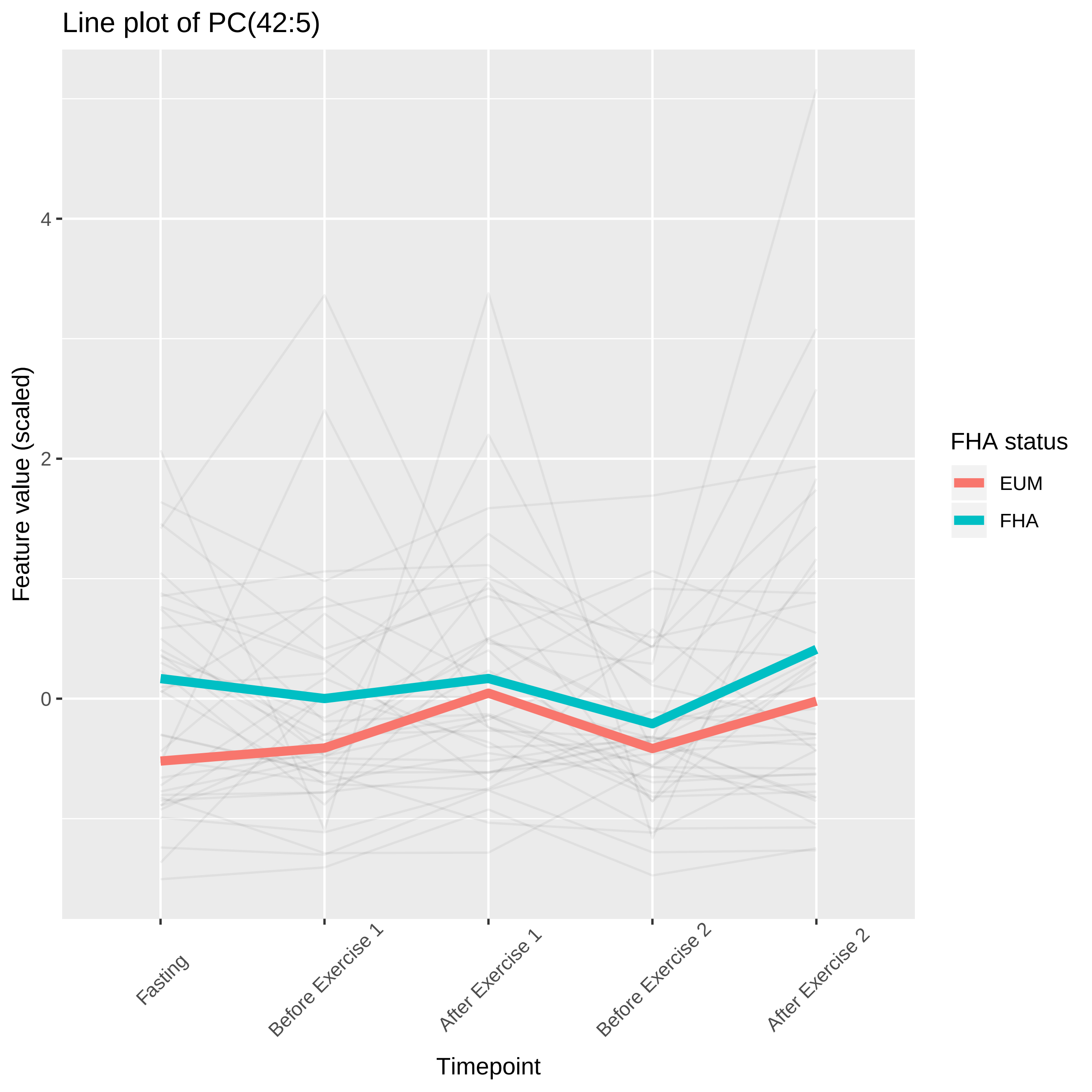

Line plot of PC(42:5)
Feature value (scaled)
0
2
4
Fasting
Before Exercise 1
After Exercise 1
Before Exercise 2
After Exercise 2
Timepoint
FHA status
EUM
FHA

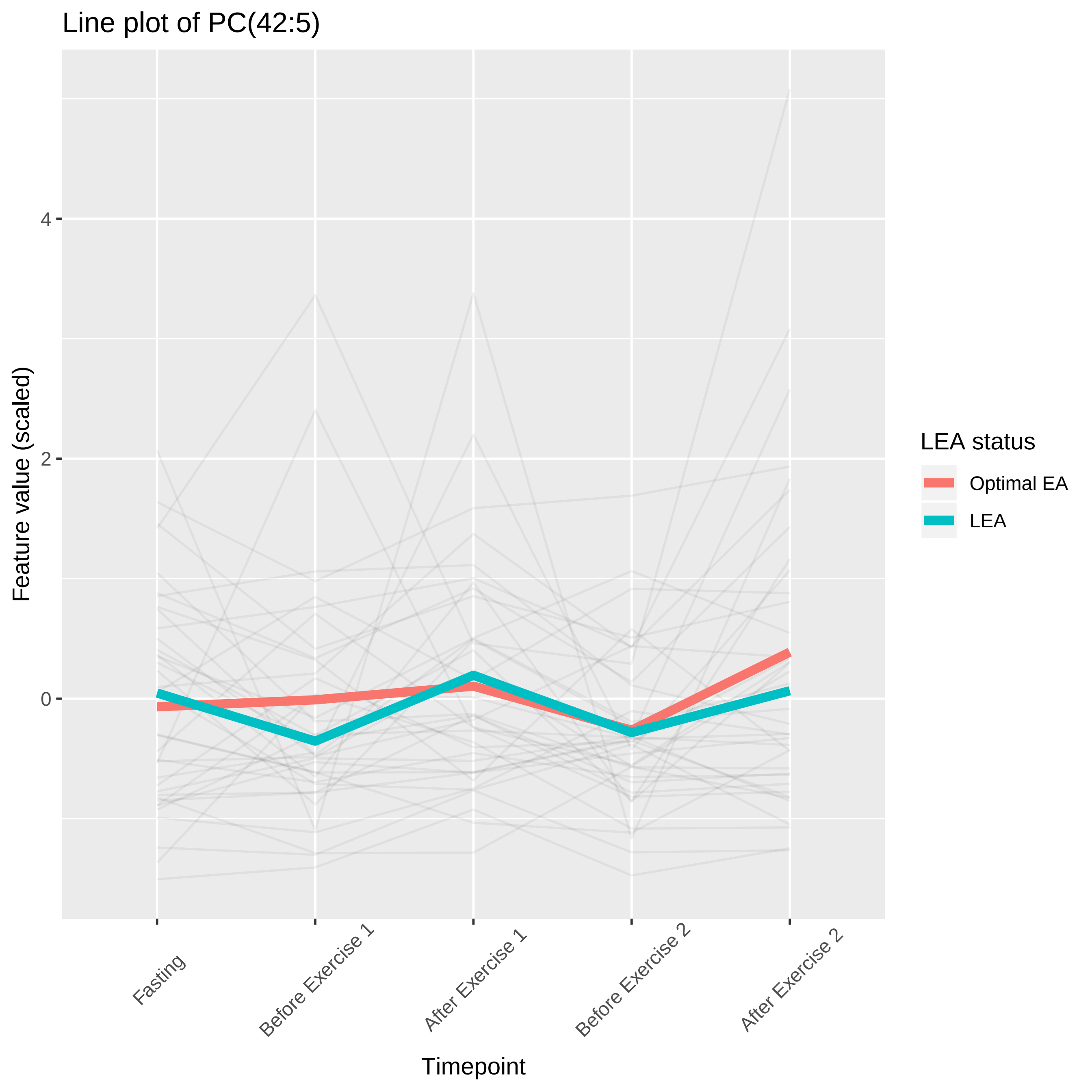

Line plot of PC(42:5)
Feature value (scaled)
4
2
0
Fasting
Before Exercise 1
After Exercise 1
Before Exercise 2
After Exercise 2
Timepoint
LEA status
Optimal EA
LEA

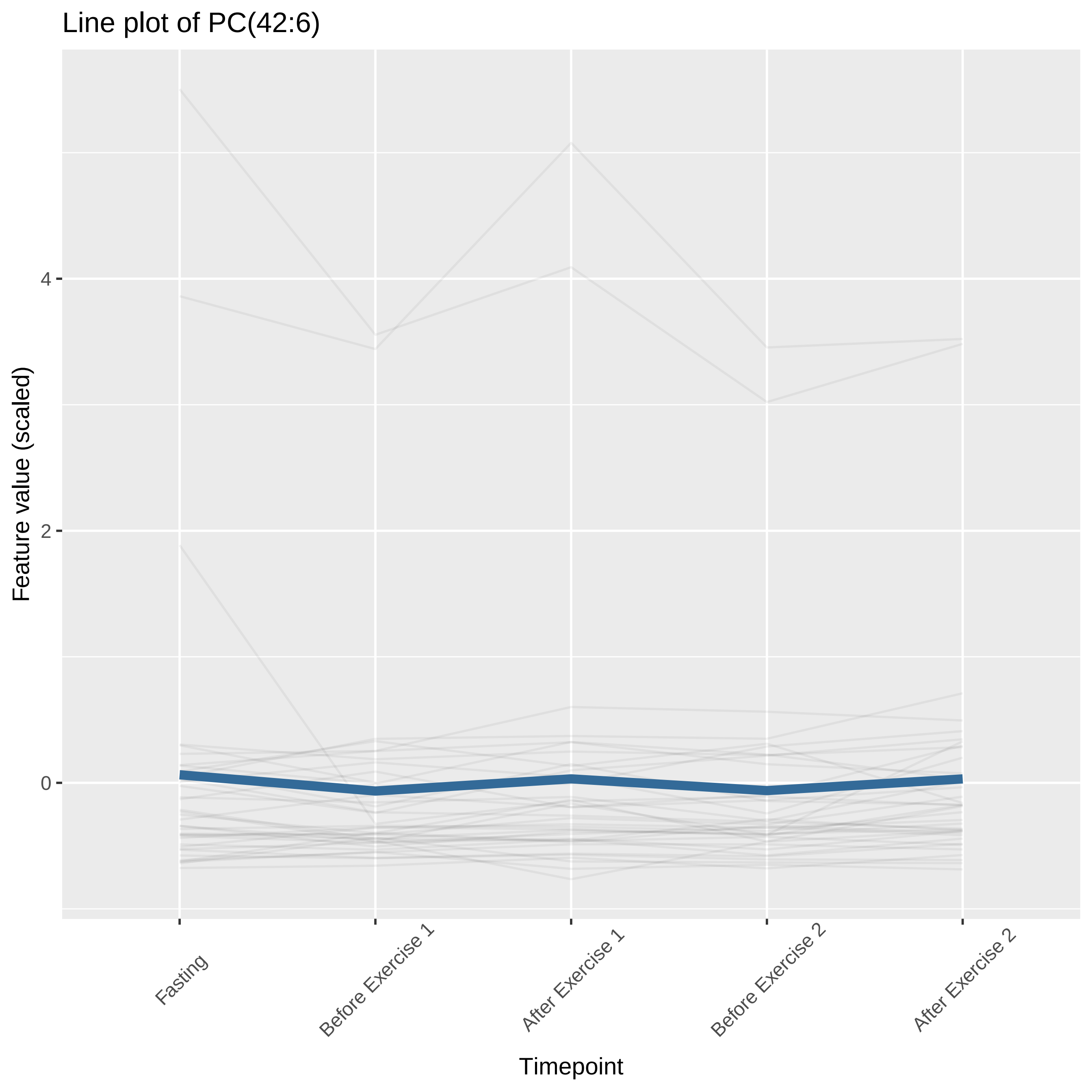

Line plot of PC(42:6)
Feature value (scaled)
4
2
0
Fasting
Before Exercise 1
After Exercise 1
Before Exercise 2
After Exercise 2
Timepoint

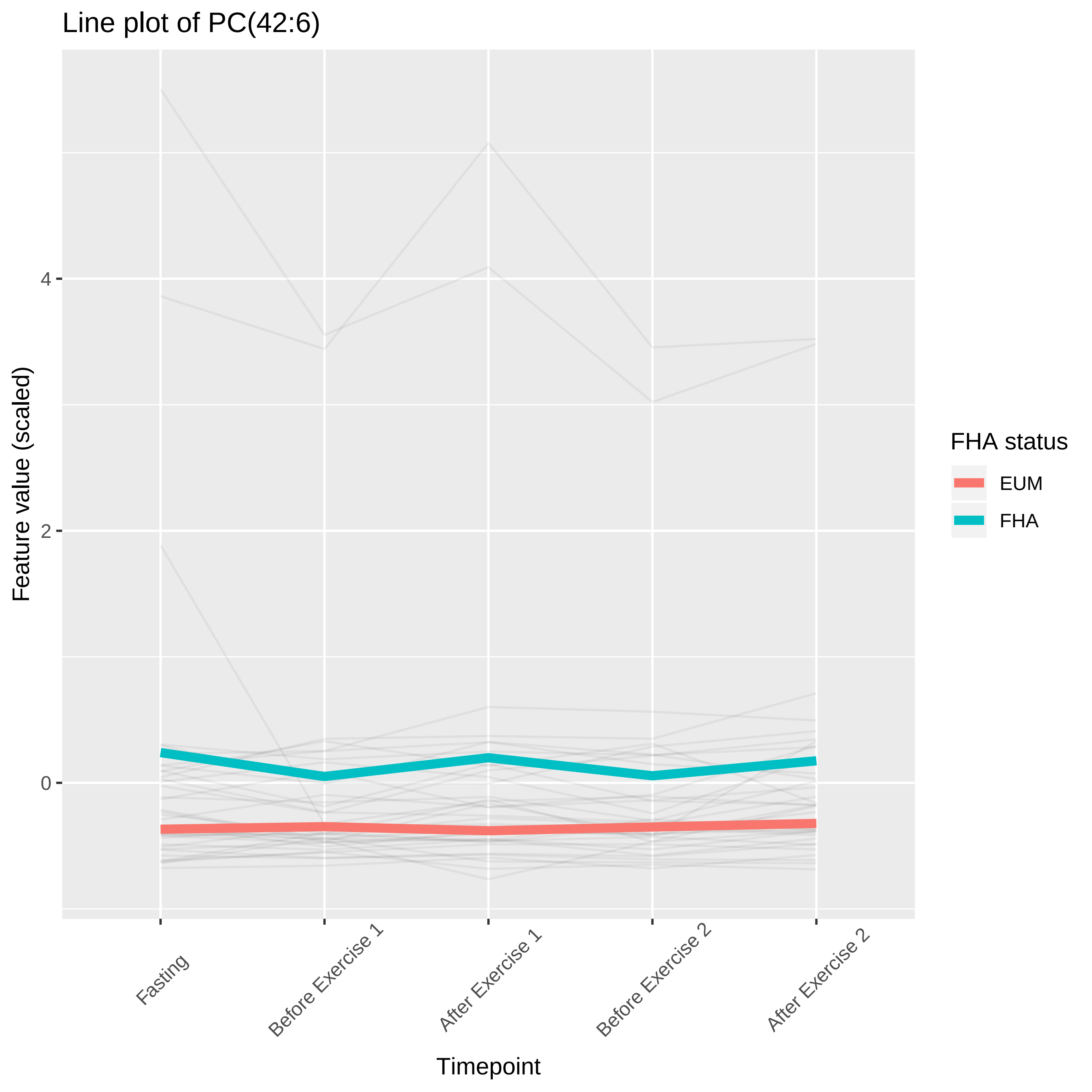

Line plot of PC(42:6)
Feature value (scaled)
4
2
0
Fasting
Before Exercise 1
After Exercise 1
Before Exercise 2
After Exercise 2
Timepoint
FHA status
EUM
FHA

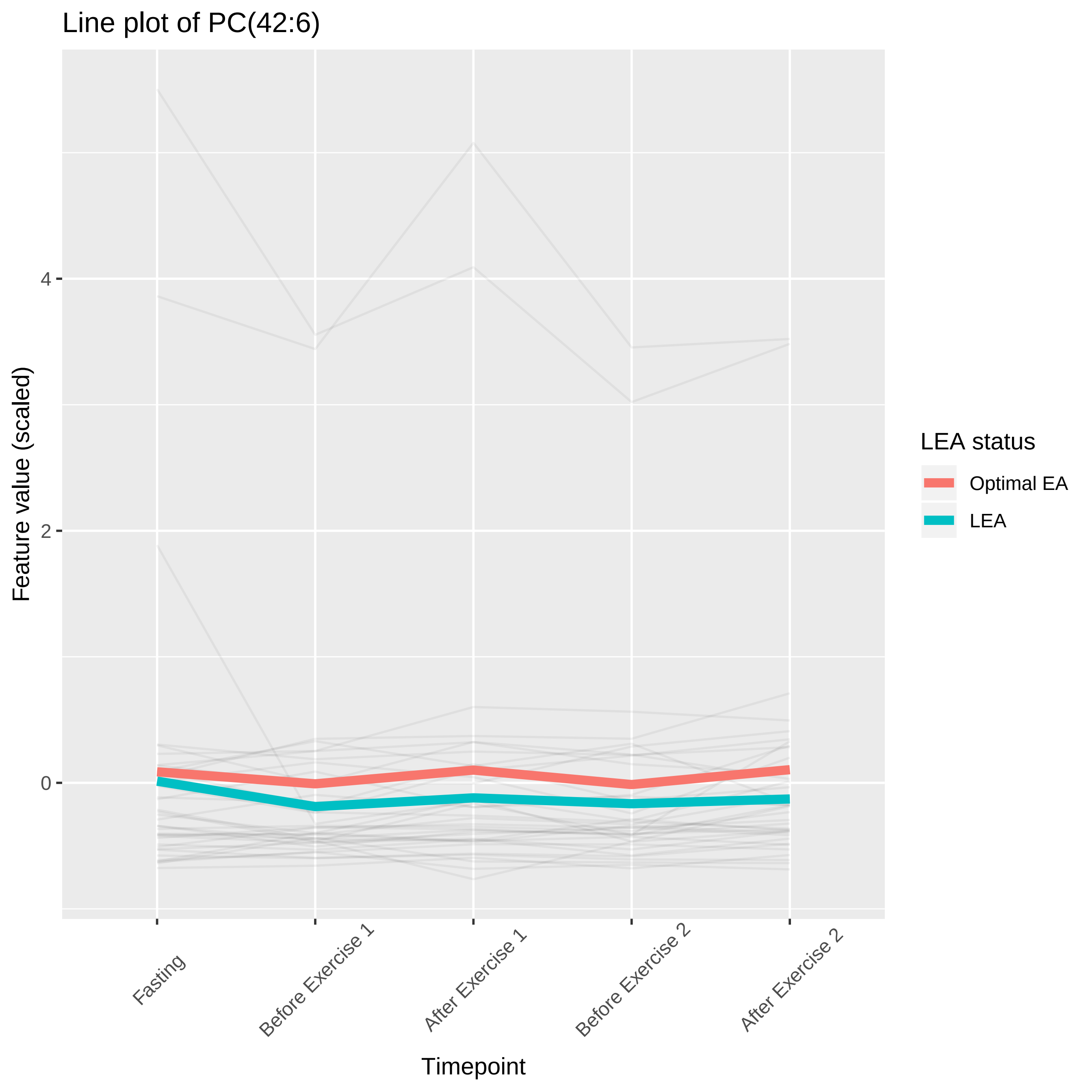

Line plot of PC(42:6)
Feature value (scaled)
4
2
0
Fasting
Before Exercise 1
After Exercise 1
Before Exercise 2
After Exercise 2
Timepoint
LEA status
Optimal EA
LEA

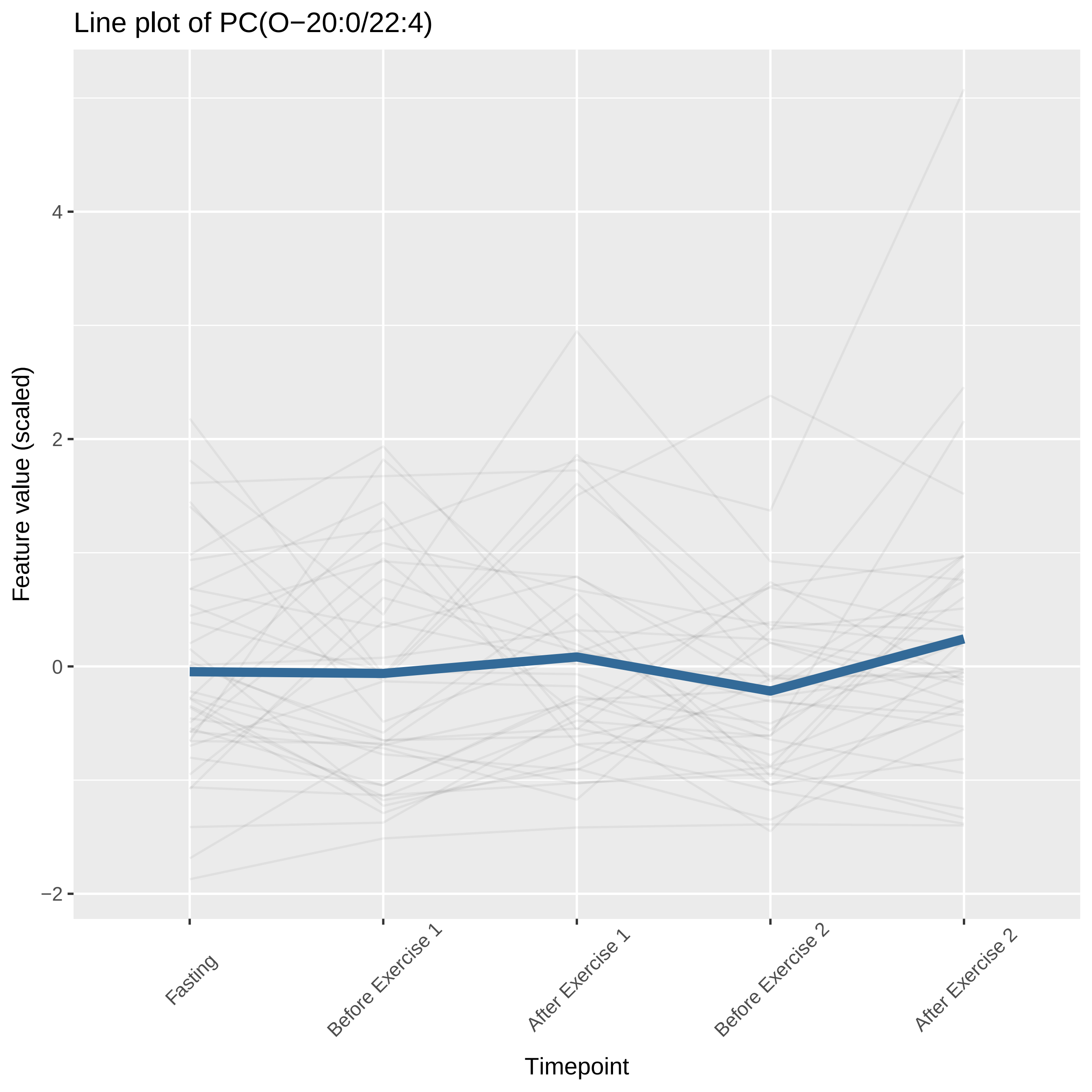

Line plot of PC(O−20:0/22:4)
Feature value (scaled)
4
2
0
−2
Fasting
Before Exercise 1
After Exercise 1
Before Exercise 2
After Exercise 2
Timepoint

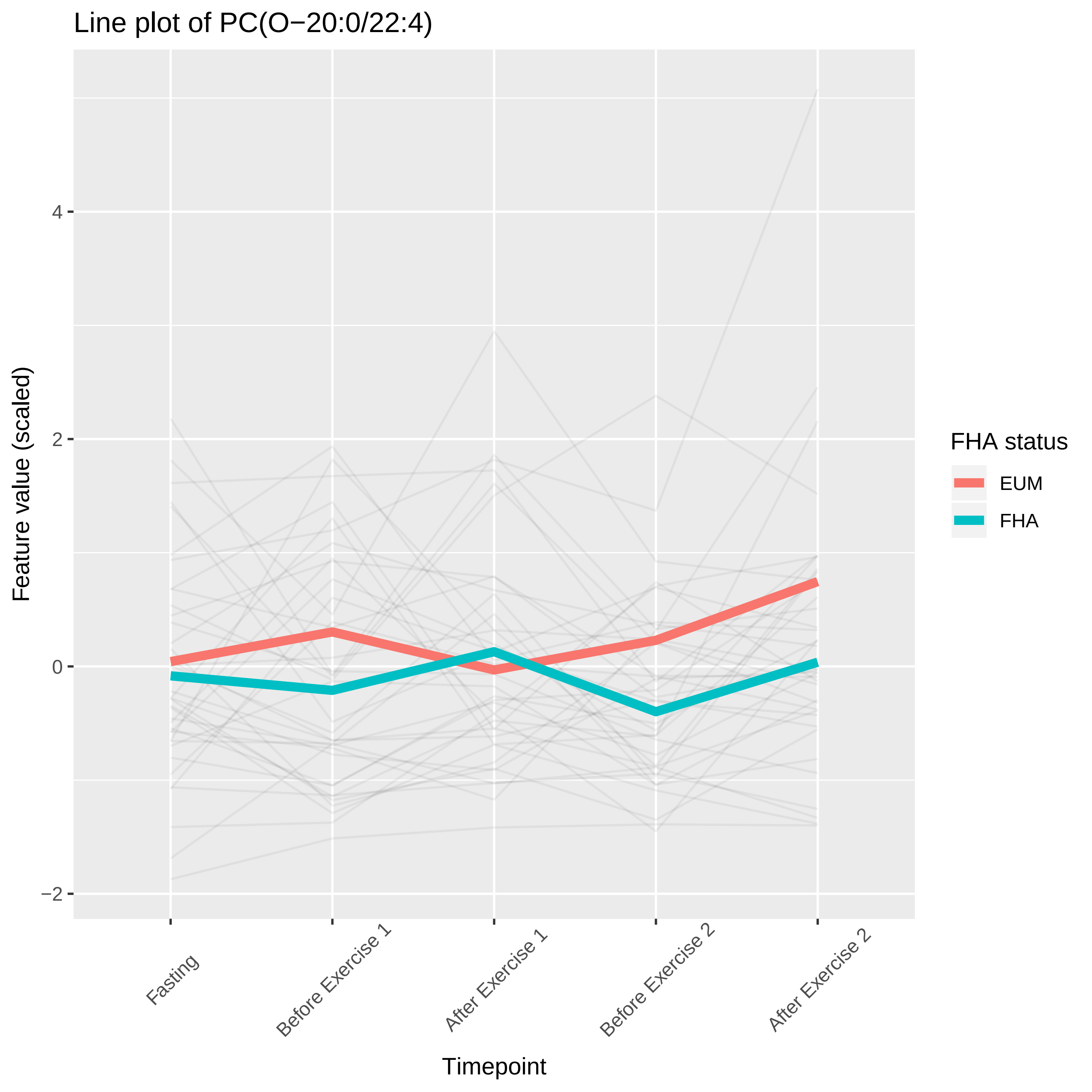
Line plot of PC(O−20:0/22:4)
Feature value (scaled)
4
2
0
−2
Fasting
Before Exercise 1
After Exercise 1
Before Exercise 2
After Exercise 2
Timepoint
FHA status
EUM
FHA

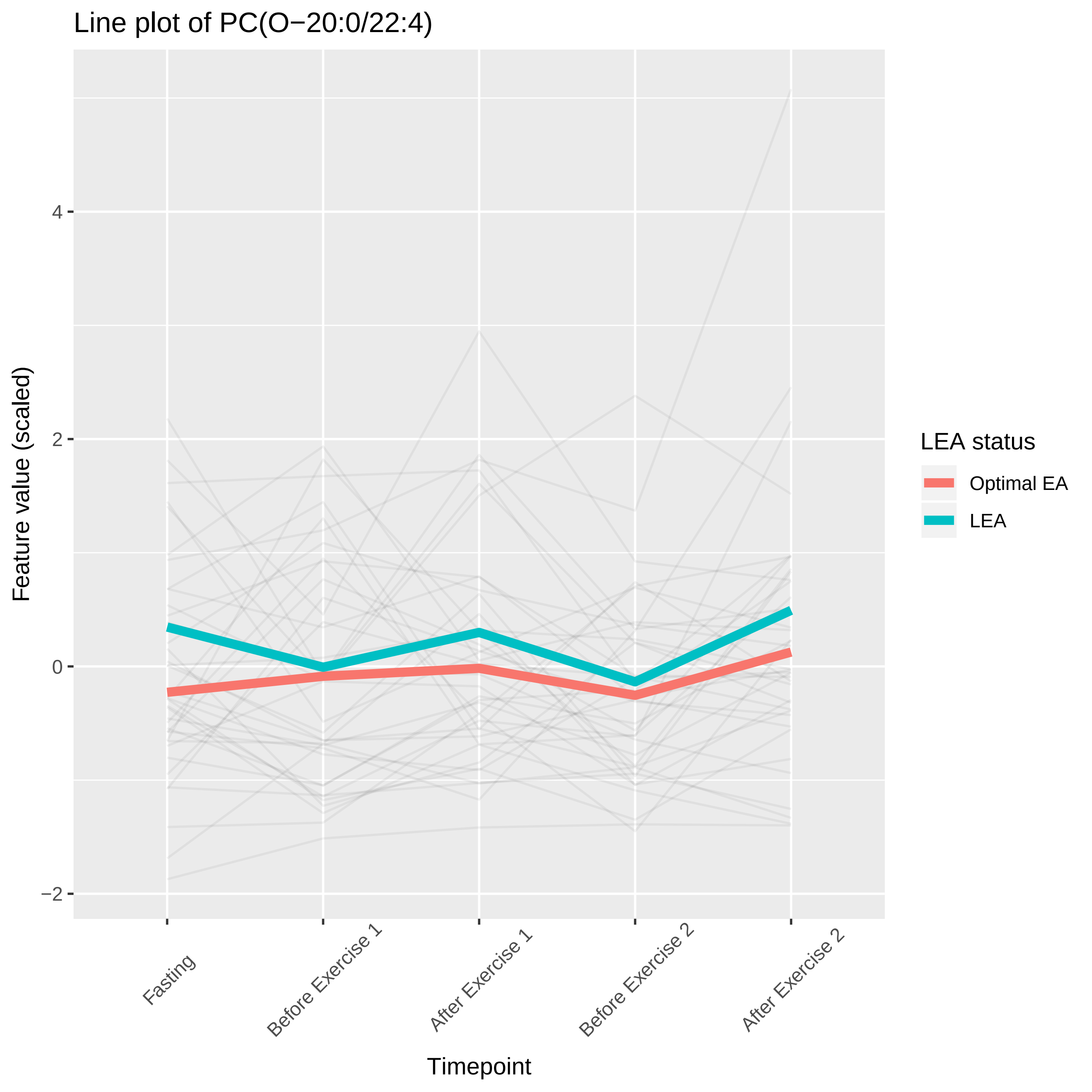

Line plot of PC(O−20:0/22:4)
Feature value (scaled)
4
2
0
−2
Fasting
Before Exercise 1
After Exercise 1
Before Exercise 2
After Exercise 2
Timepoint
LEA status
Optimal EA
LEA

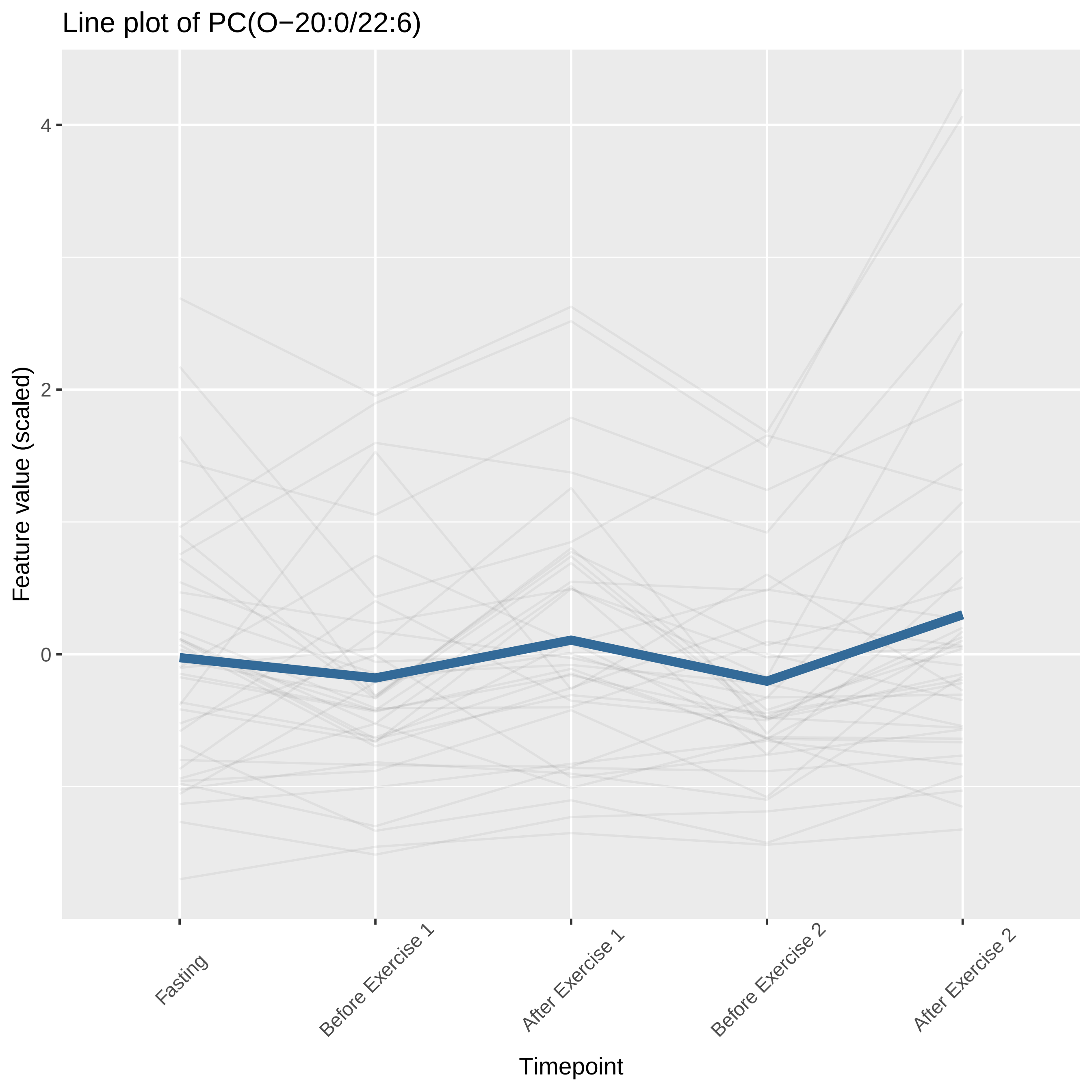
Line plot of PC(O−20:0/22:6)
Feature value (scaled)
4
2
0
Fasting
Before Exercise 1
After Exercise 1
Before Exercise 2
After Exercise 2
Timepoint

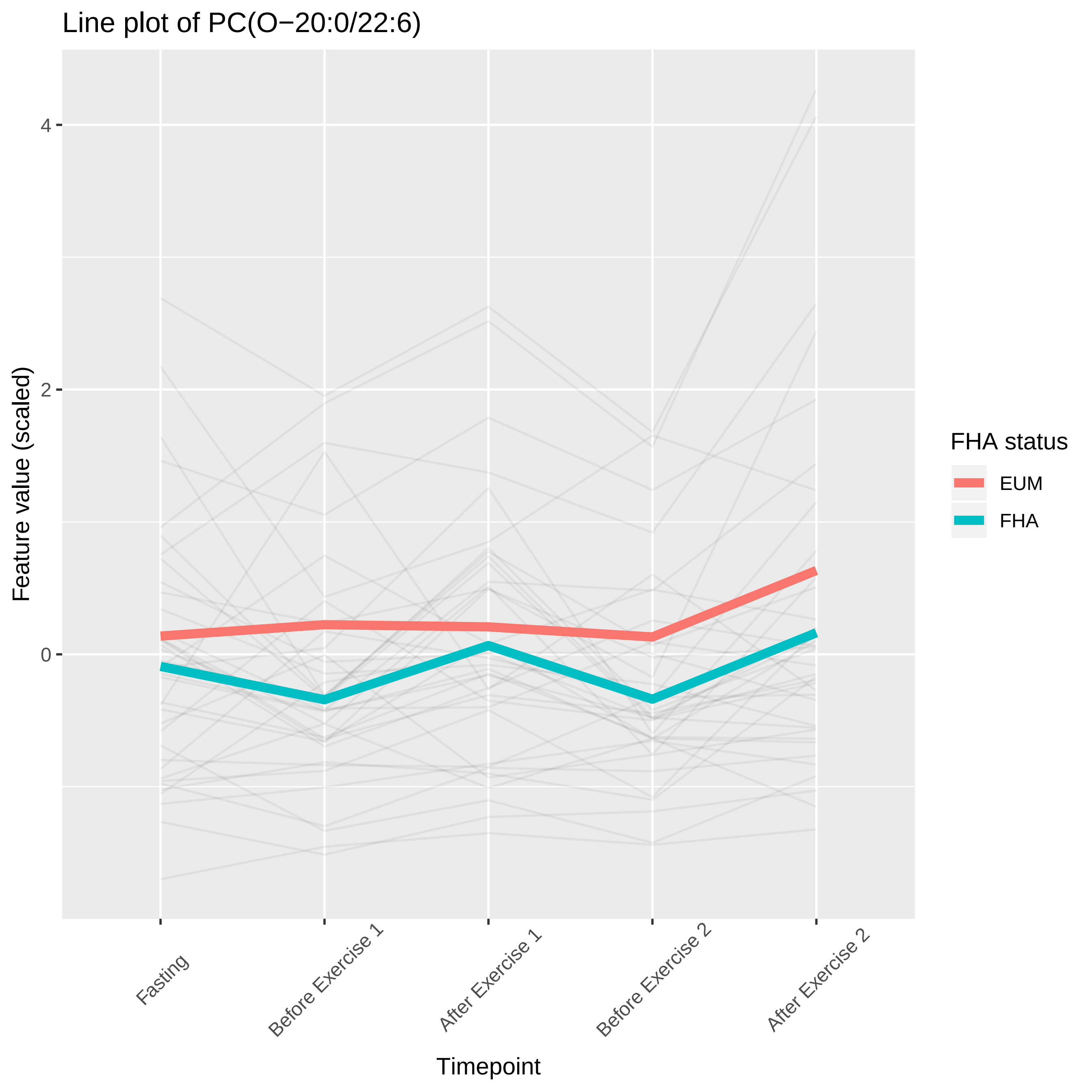

Line plot of PC(O−20:0/22:6)
Feature value (scaled)
4
2
0
Fasting
Before Exercise 1
After Exercise 1
Before Exercise 2
After Exercise 2
Timepoint
FHA status
EUM
FHA

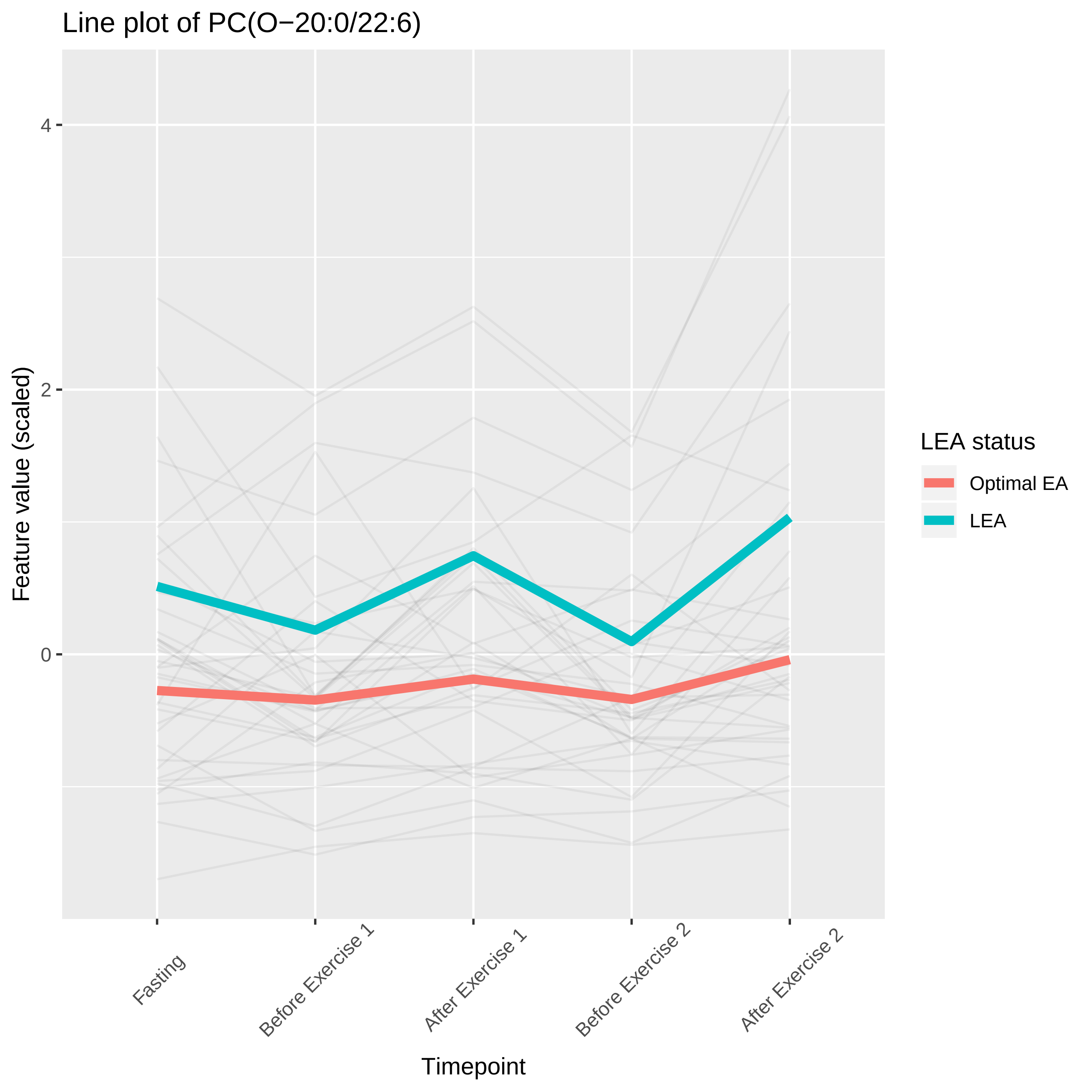

Line plot of PC(O−20:0/22:6)
Feature value (scaled)
4
2
0
Fasting
Before Exercise 1
After Exercise 1
Before Exercise 2
After Exercise 2
Timepoint
LEA status
Optimal EA
LEA

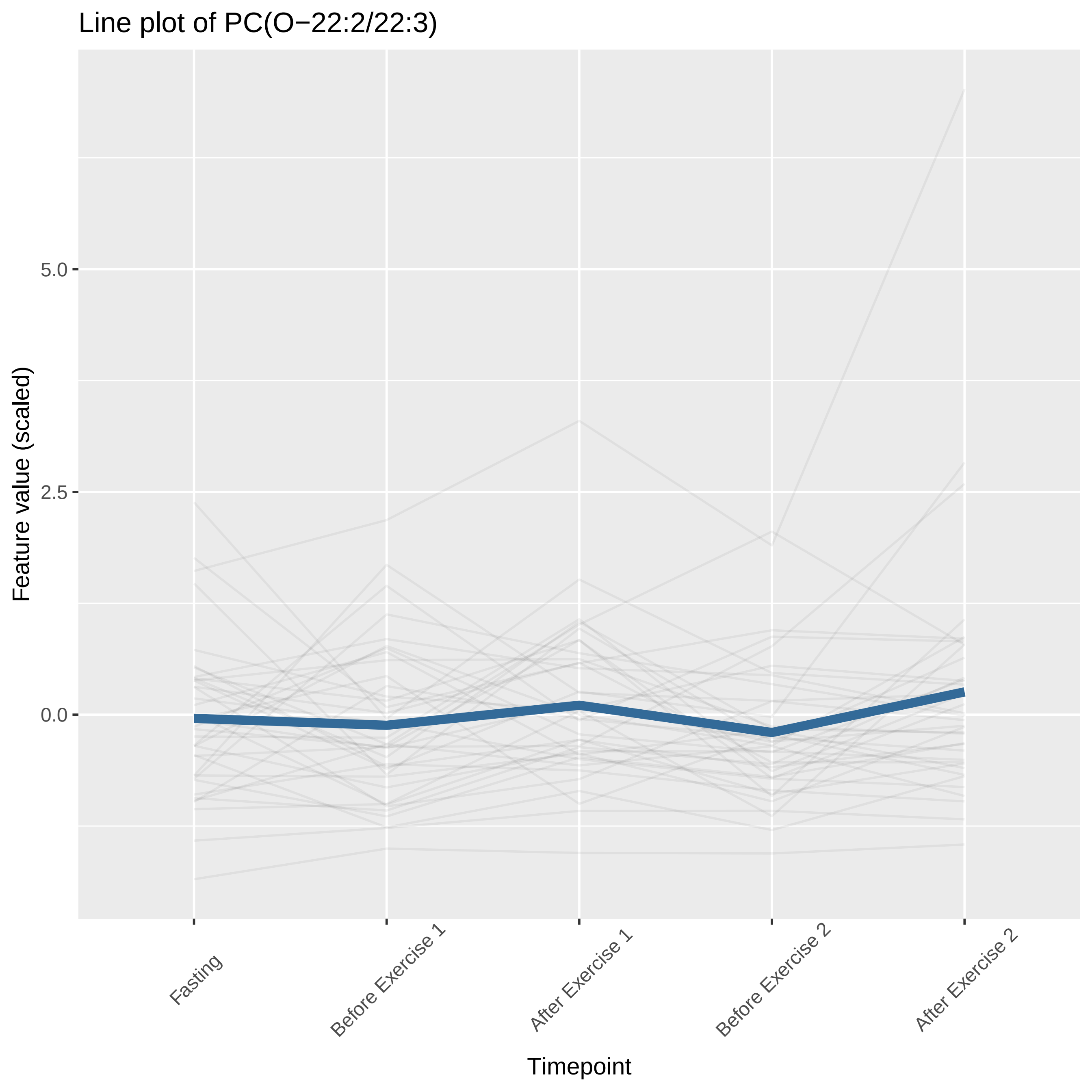

Line plot of PC(O−22:2/22:3)
Feature value (scaled)
5.0
2.5
0.0
Fasting
Before Exercise 1
After Exercise 1
Before Exercise 2
After Exercise 2
Timepoint

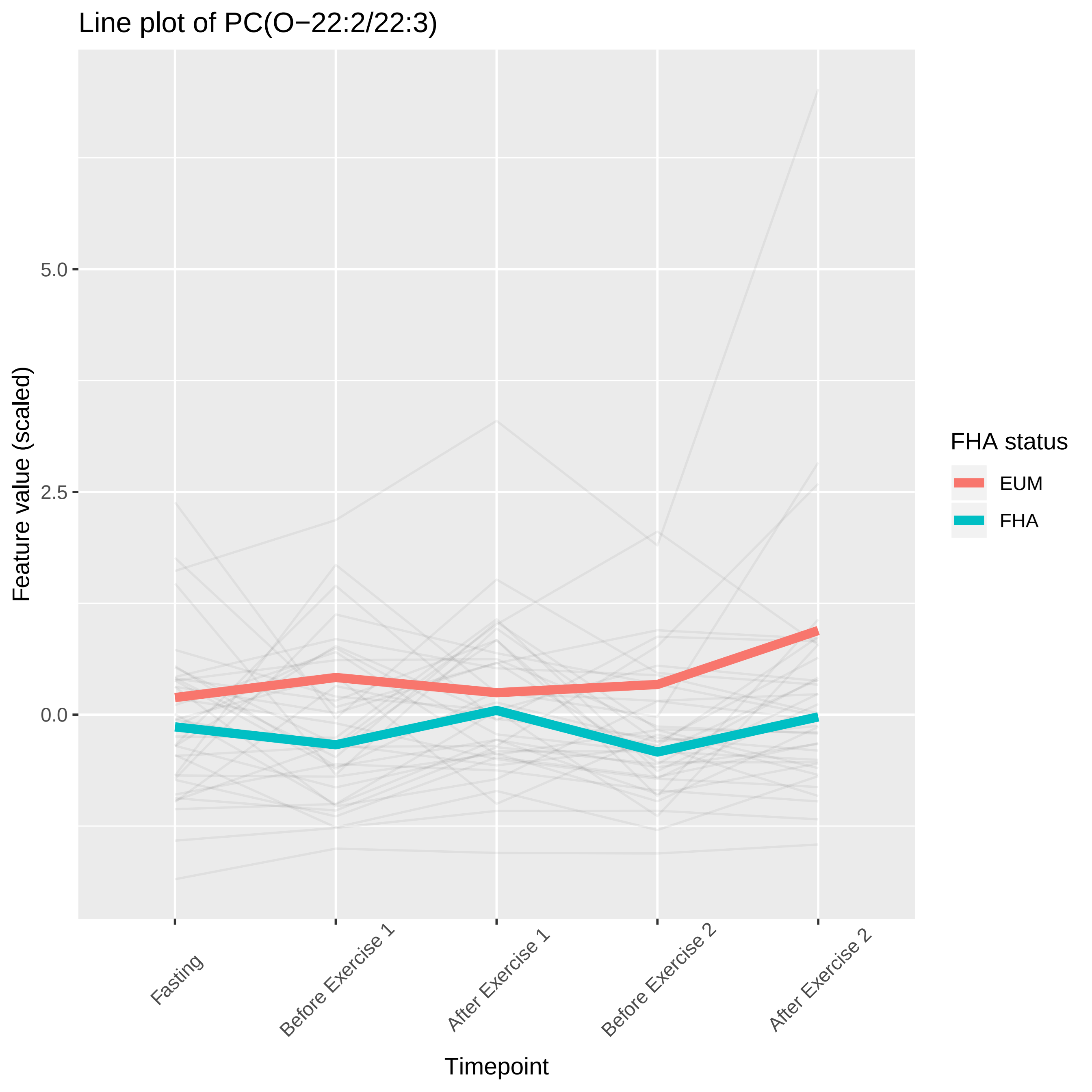

Line plot of PC(O−22:2/22:3)
Feature value (scaled)
5.0
2.5
0.0
Fasting
Before Exercise 1
After Exercise 1
Before Exercise 2
After Exercise 2
Timepoint
FHA status
EUM
FHA

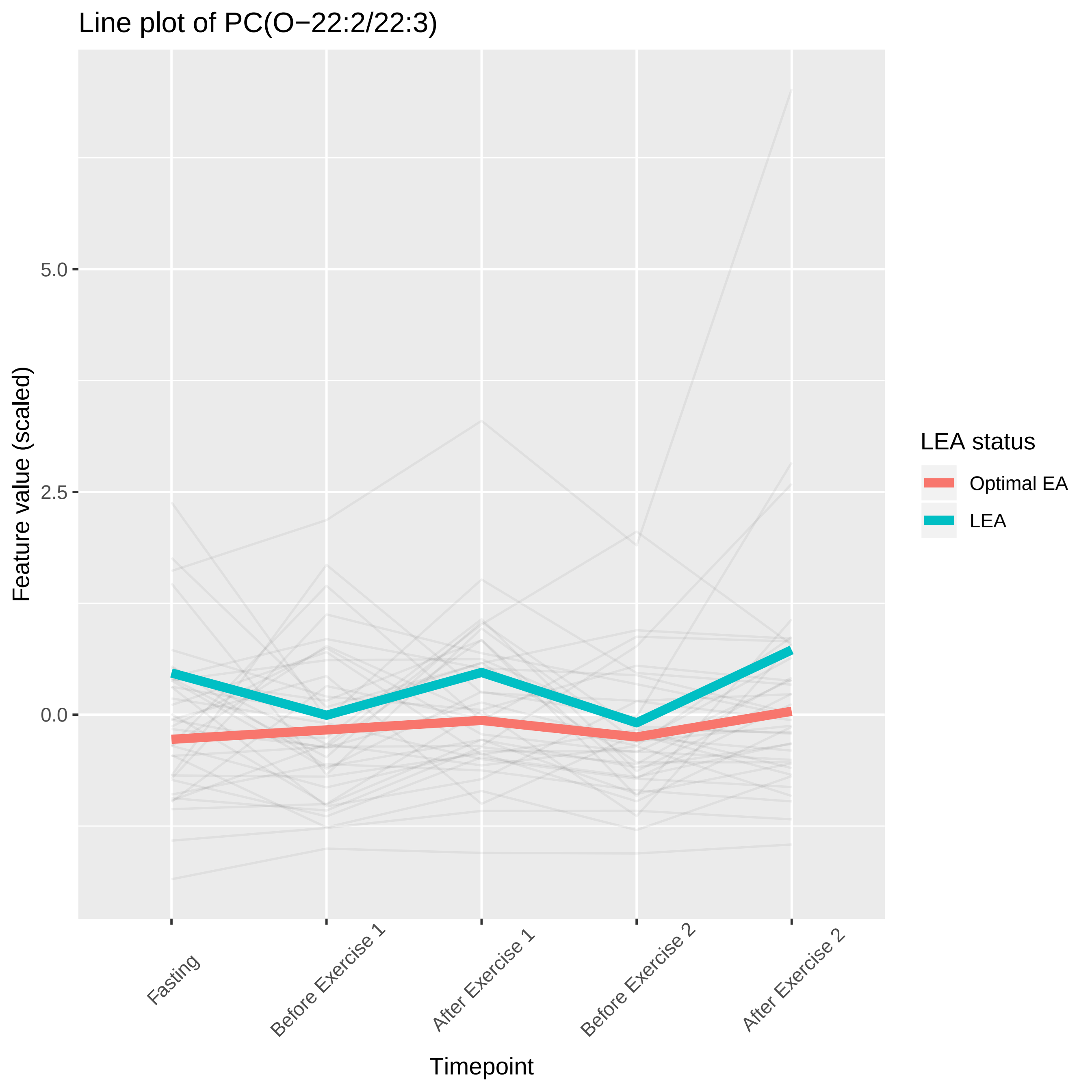
Line plot of PC(O−22:2/22:3)
Feature value (scaled)
5.0
2.5
0.0
Fasting
Before Exercise 1
After Exercise 1
Before Exercise 2
After Exercise 2
Timepoint
LEA status
Optimal EA
LEA

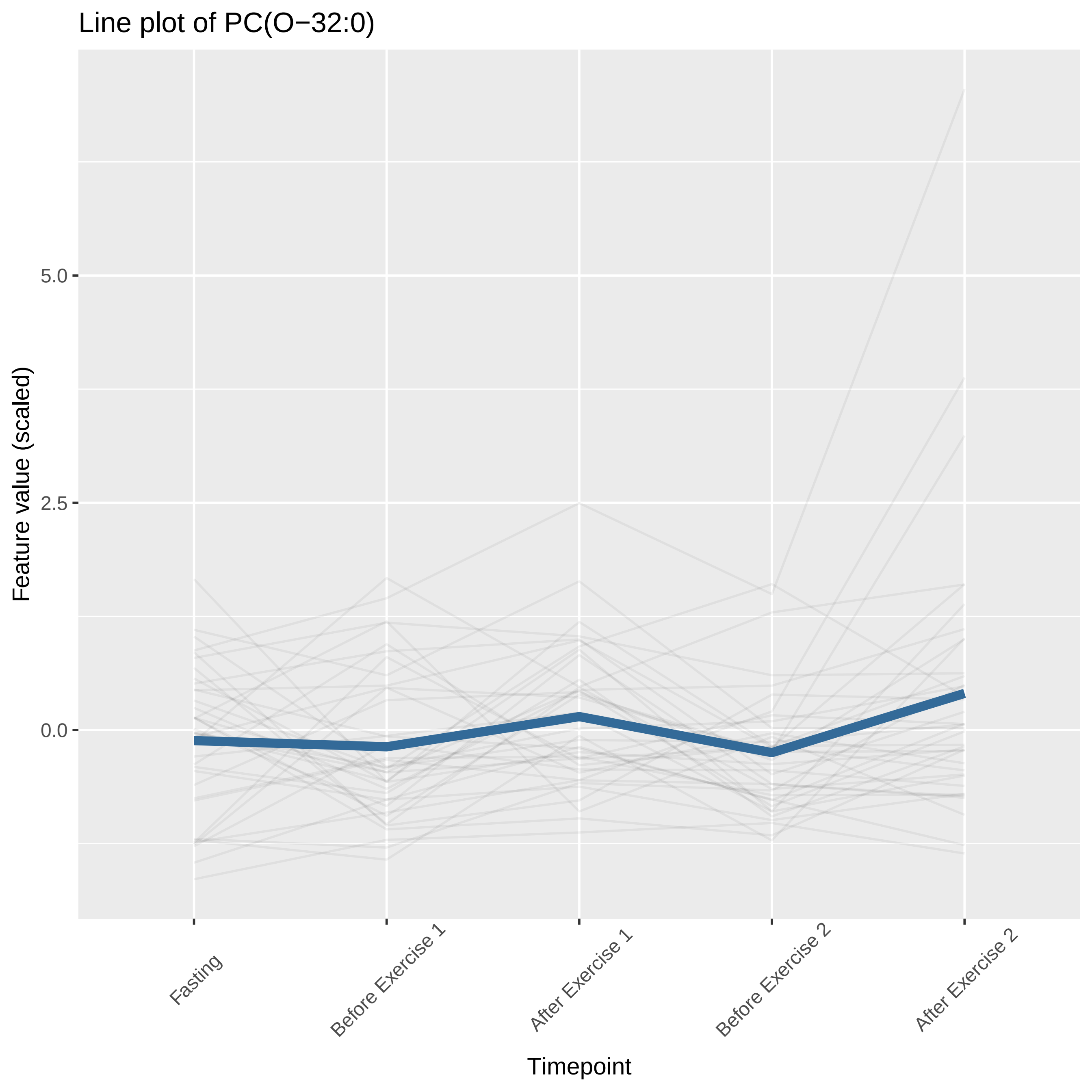

Line plot of PC(O−32:0)
Feature value (scaled)
5.0
2.5
0.0
Fasting
Before Exercise 1
After Exercise 1
Before Exercise 2
After Exercise 2
Timepoint

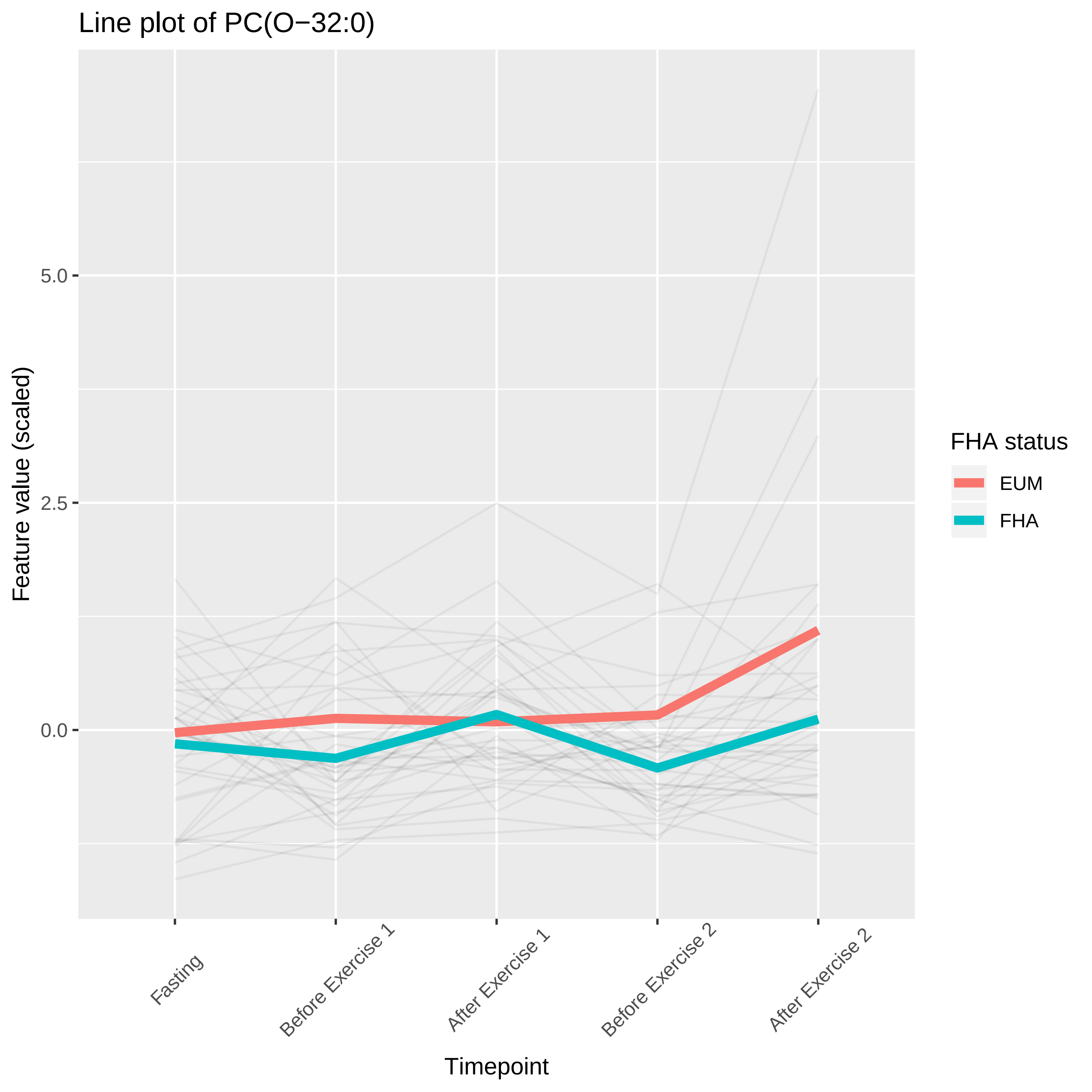

Line plot of PC(O−32:0)
Feature value (scaled)
5.0
2.5
0.0
Fasting
Before Exercise 1
After Exercise 1
Before Exercise 2
After Exercise 2
Timepoint
FHA status
EUM
FHA

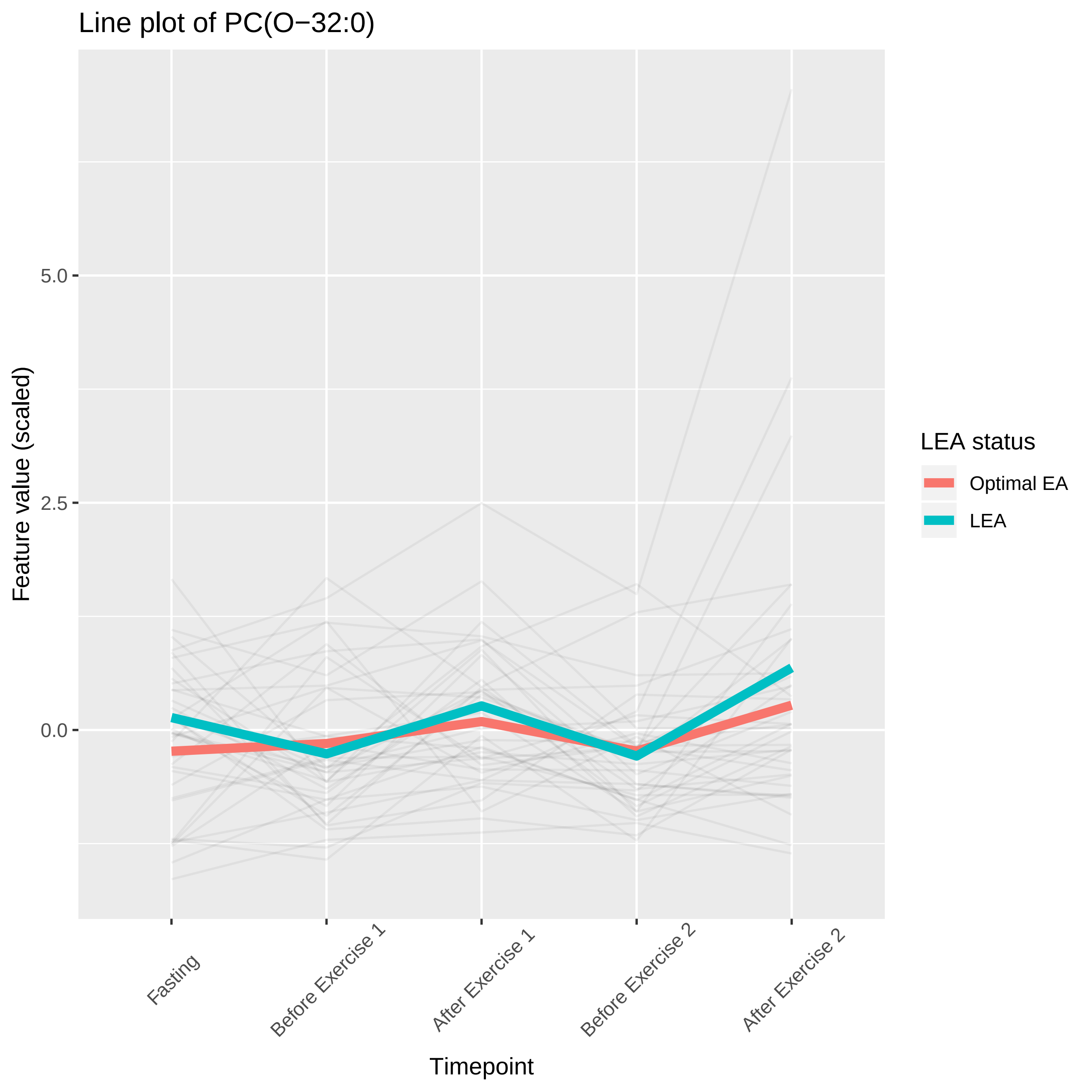
Line plot of PC(O−32:0)
Feature value (scaled)
5.0
2.5
0.0
Fasting
Before Exercise 1
After Exercise 1
Before Exercise 2
After Exercise 2
Timepoint
LEA status
Optimal EA
LEA

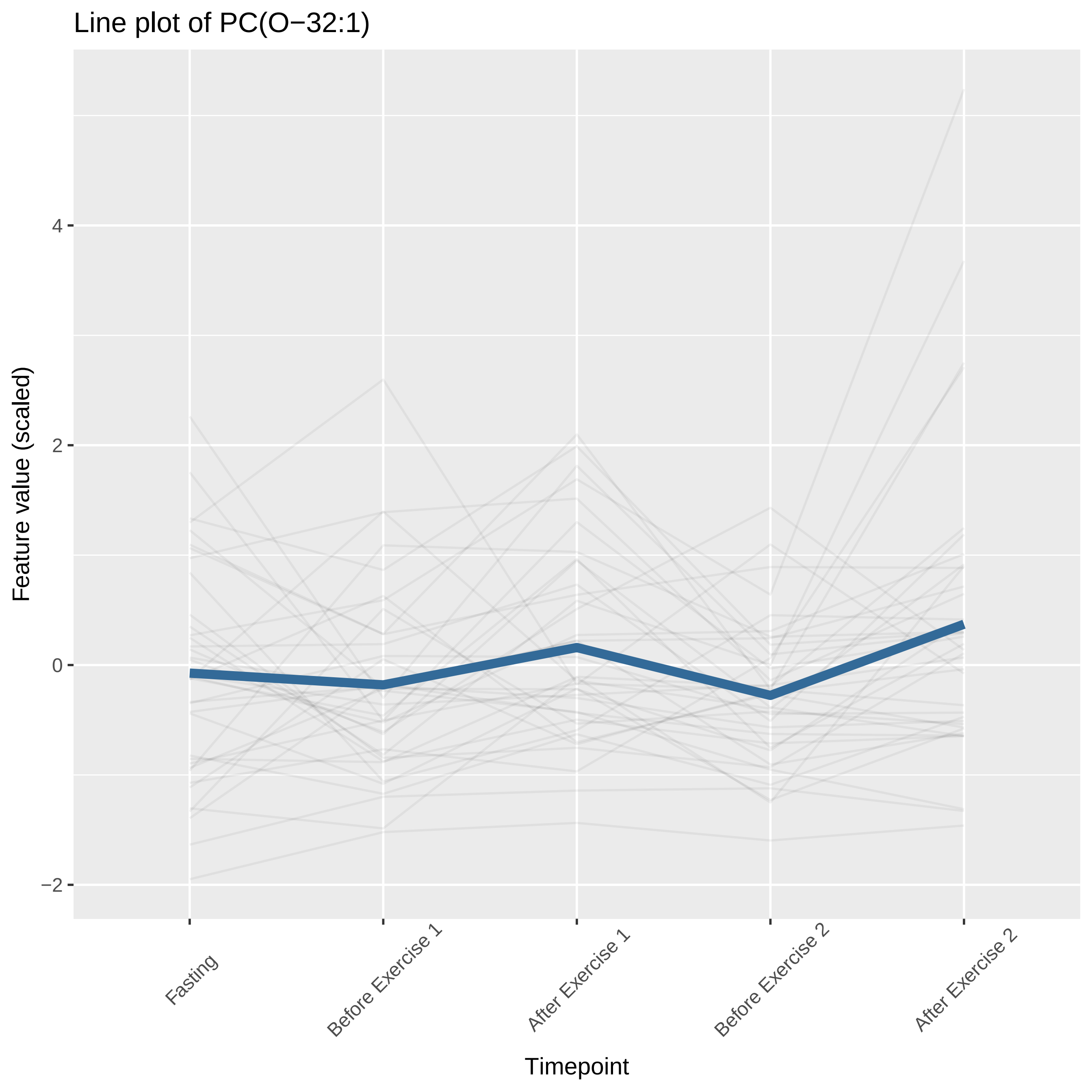

Line plot of PC(O−32:1)
Feature value (scaled)
4
2
0
−2
Fasting
Before Exercise 1
After Exercise 1
Before Exercise 2
After Exercise 2
Timepoint

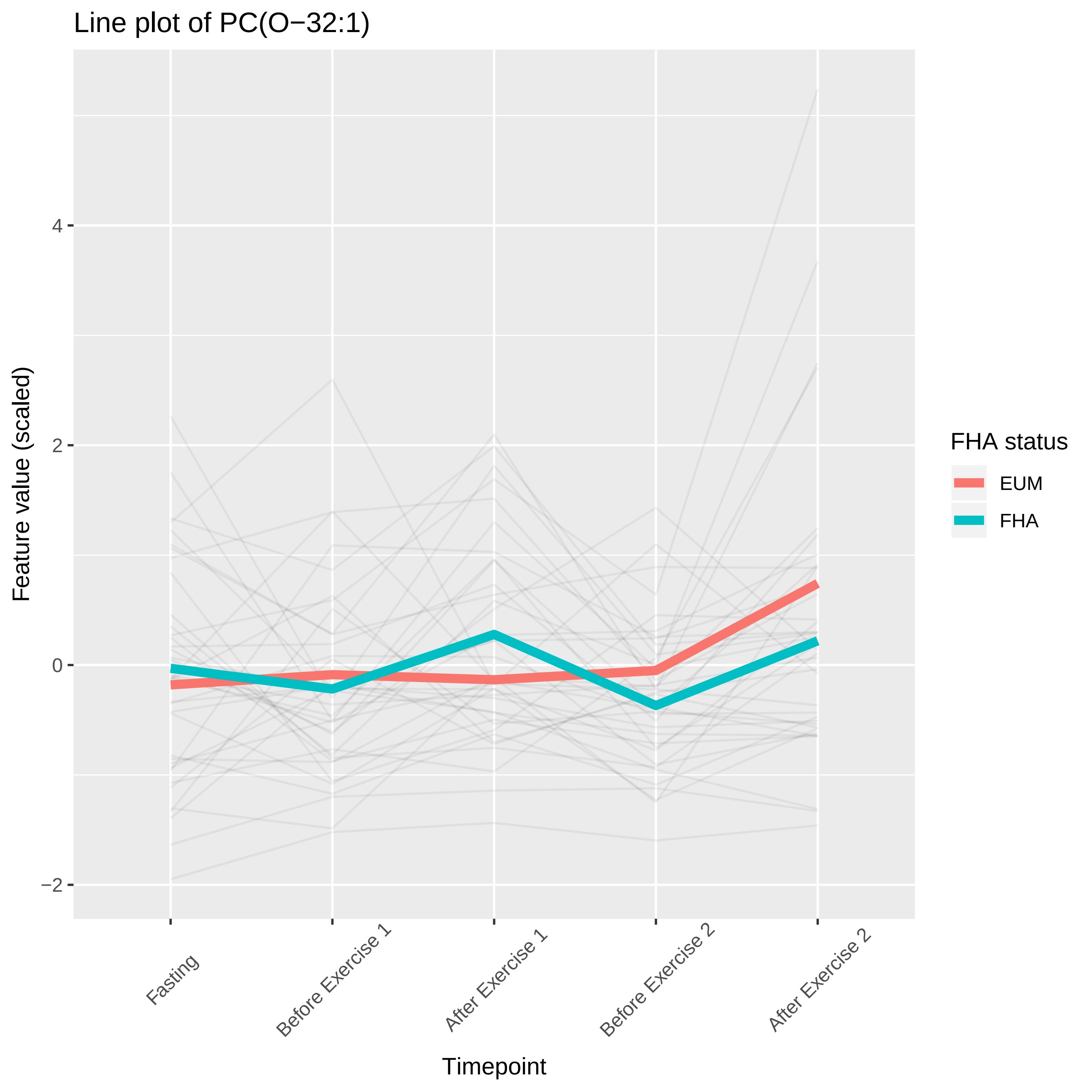
Line plot of PC(O−32:1)
Feature value (scaled)
4
2
0
−2
Fasting
Before Exercise 1
After Exercise 1
Before Exercise 2
After Exercise 2
Timepoint
FHA status
EUM
FHA

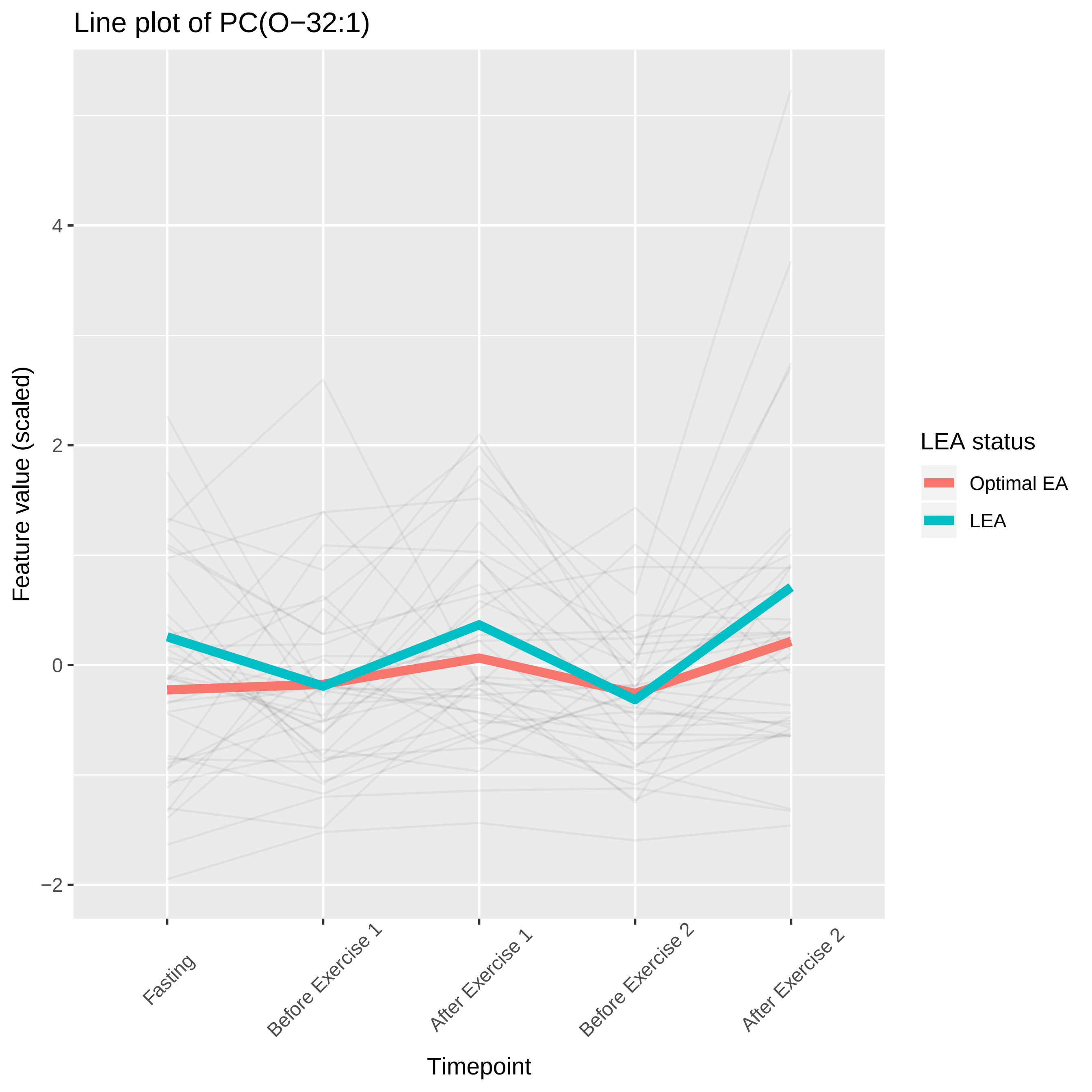
Line plot of PC(O−32:1)
Feature value (scaled)
4
2
0
−2
Fasting
Before Exercise 1
After Exercise 1
Before Exercise 2
After Exercise 2
Timepoint
LEA status
Optimal EA
LEA

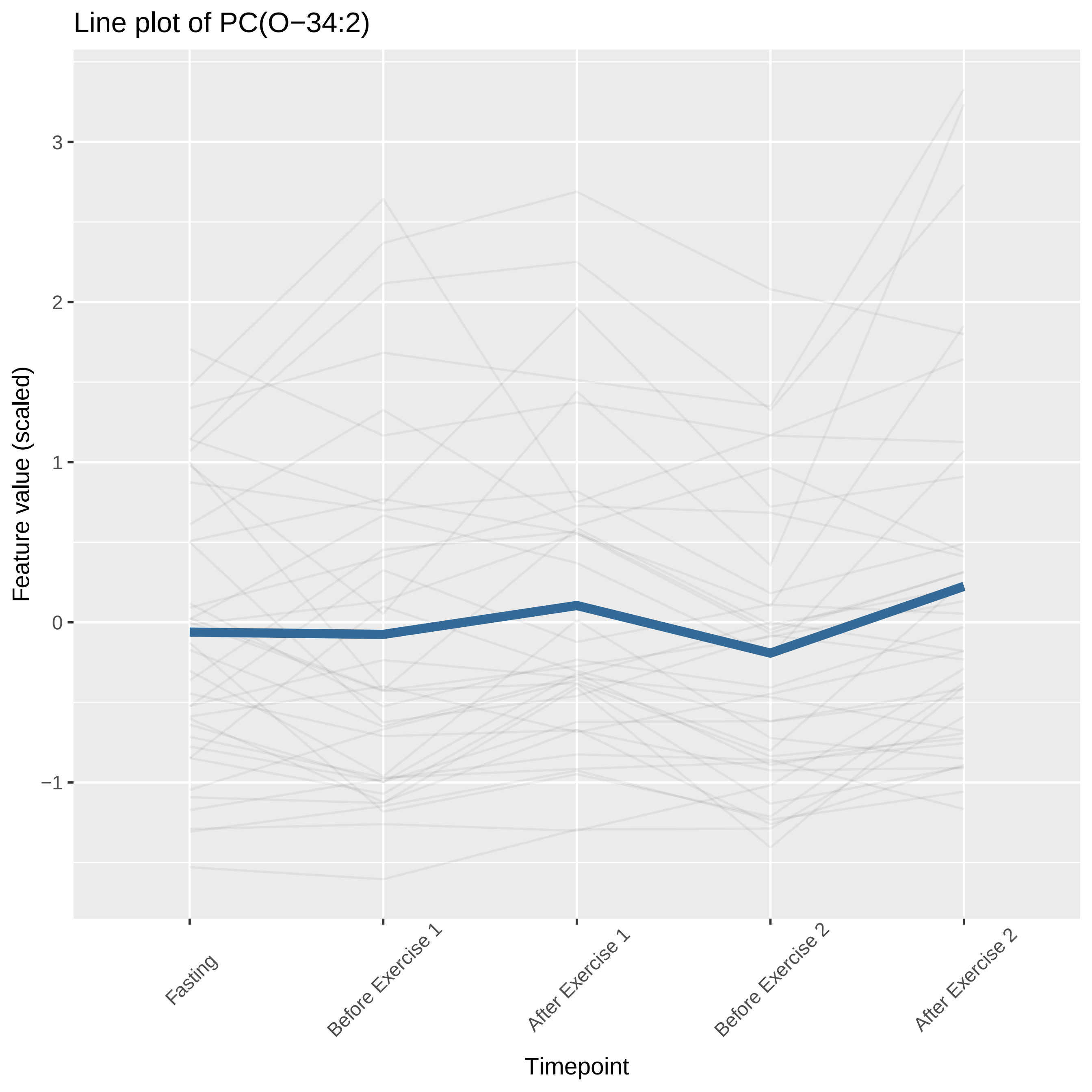
Line plot of PC(O−34:2)
Feature value (scaled)
3
2
1
0
−1
Fasting
Before Exercise 1
After Exercise 1
Before Exercise 2
After Exercise 2
Timepoint

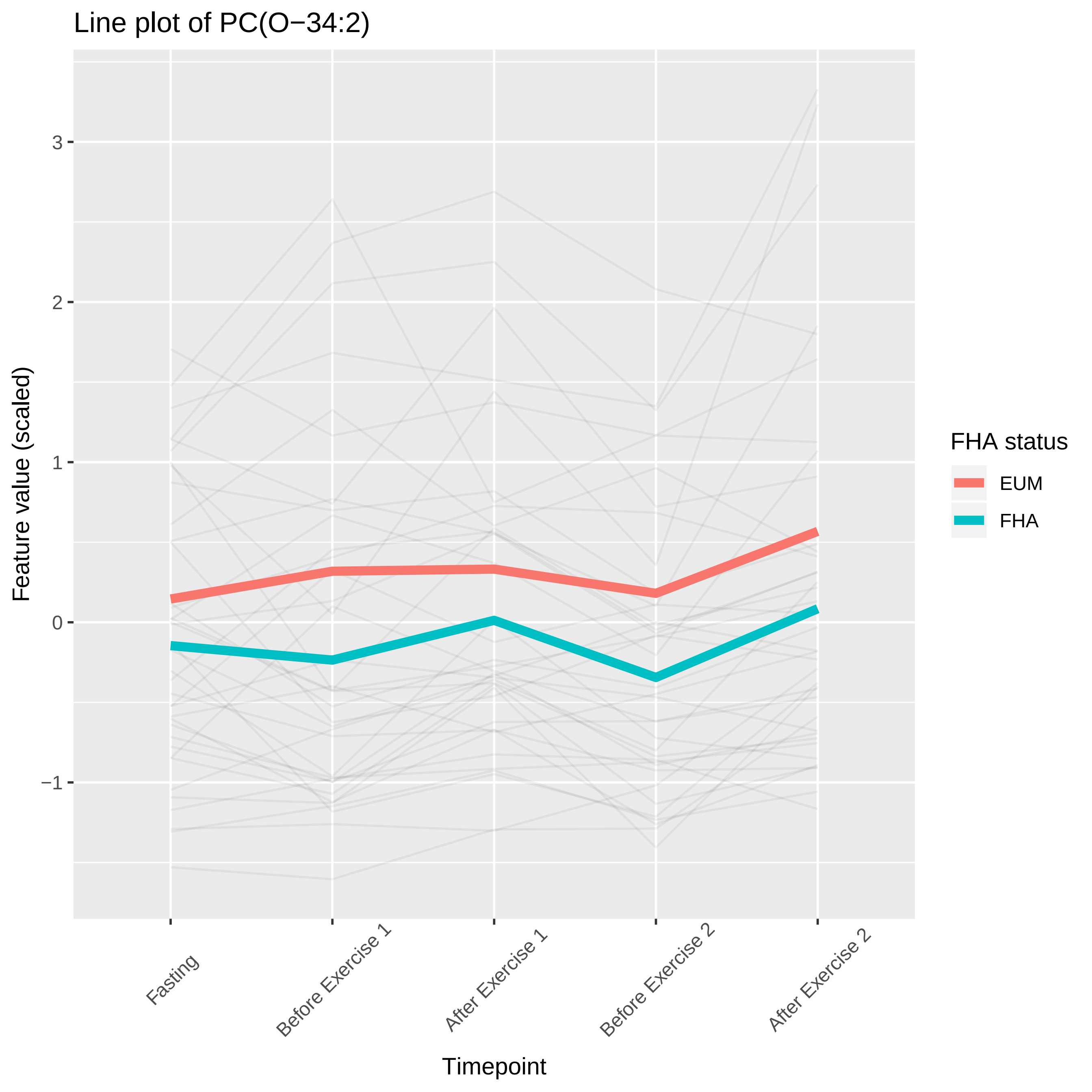

Line plot of PC(O−34:2)
Feature value (scaled)
3
2
1
0
−1
Fasting
Before Exercise 1
After Exercise 1
Before Exercise 2
After Exercise 2
Timepoint
FHA status
EUM
FHA

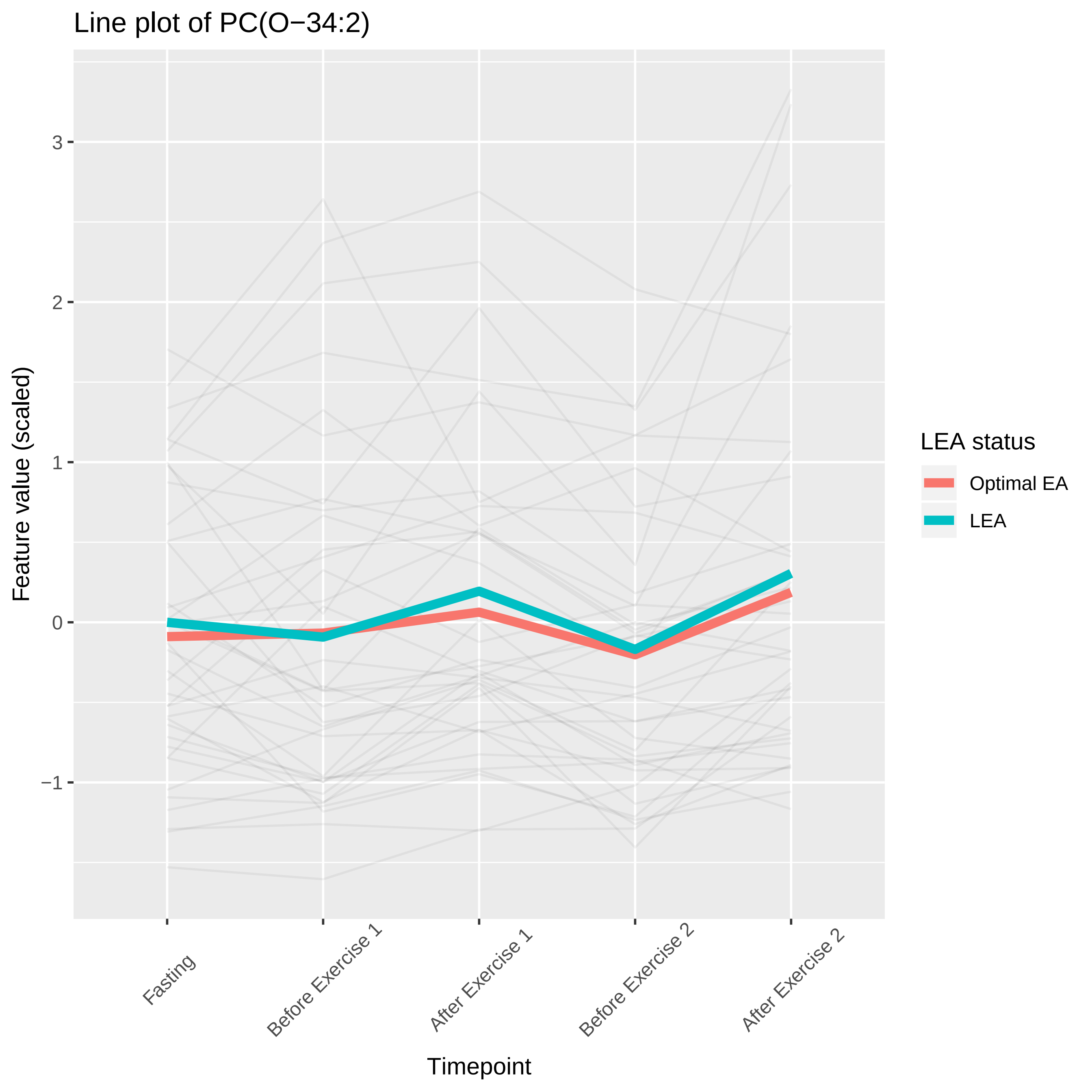

Line plot of PC(O−34:2)
Feature value (scaled)
3
2
1
0
−1
Fasting
Before Exercise 1
After Exercise 1
Before Exercise 2
After Exercise 2
Timepoint
LEA status
Optimal EA
LEA

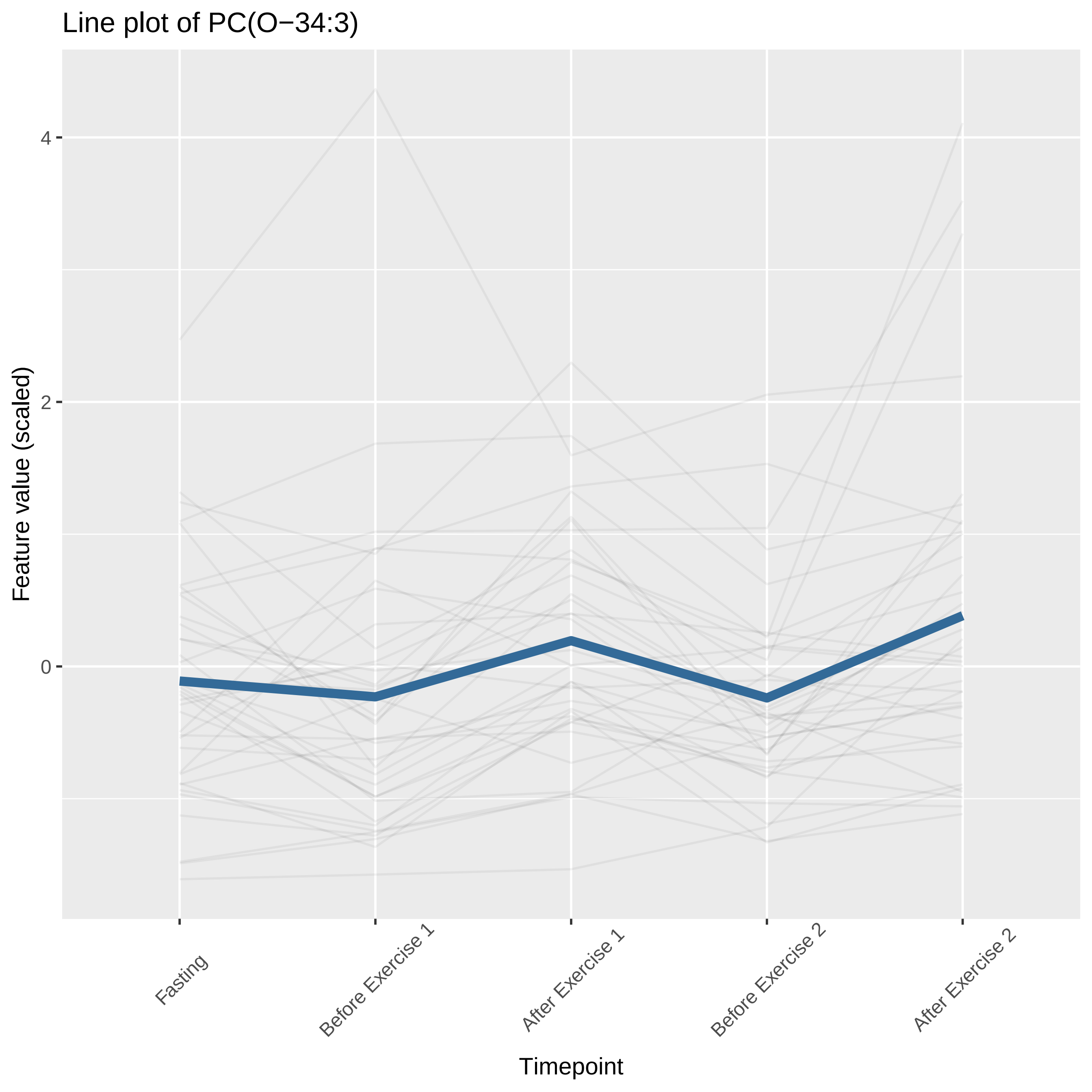

Line plot of PC(O−34:3)
Feature value (scaled)
4
2
0
Fasting
Before Exercise 1
After Exercise 1
Before Exercise 2
After Exercise 2
Timepoint

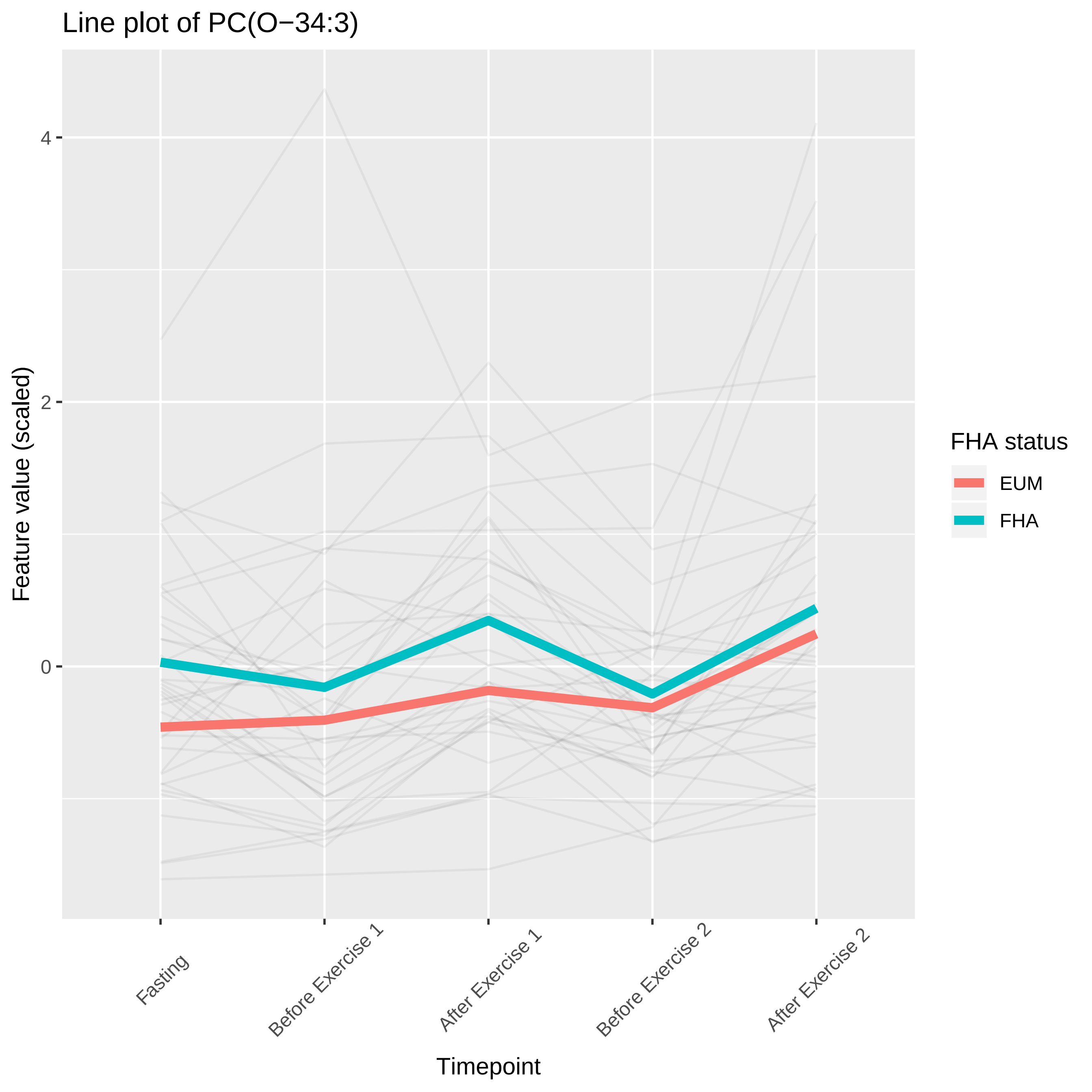

Line plot of PC(O−34:3)
Feature value (scaled)
4
2
0
Fasting
Before Exercise 1
After Exercise 1
Before Exercise 2
After Exercise 2
Timepoint
FHA status
EUM
FHA

Line plot of PC(O−34:3)

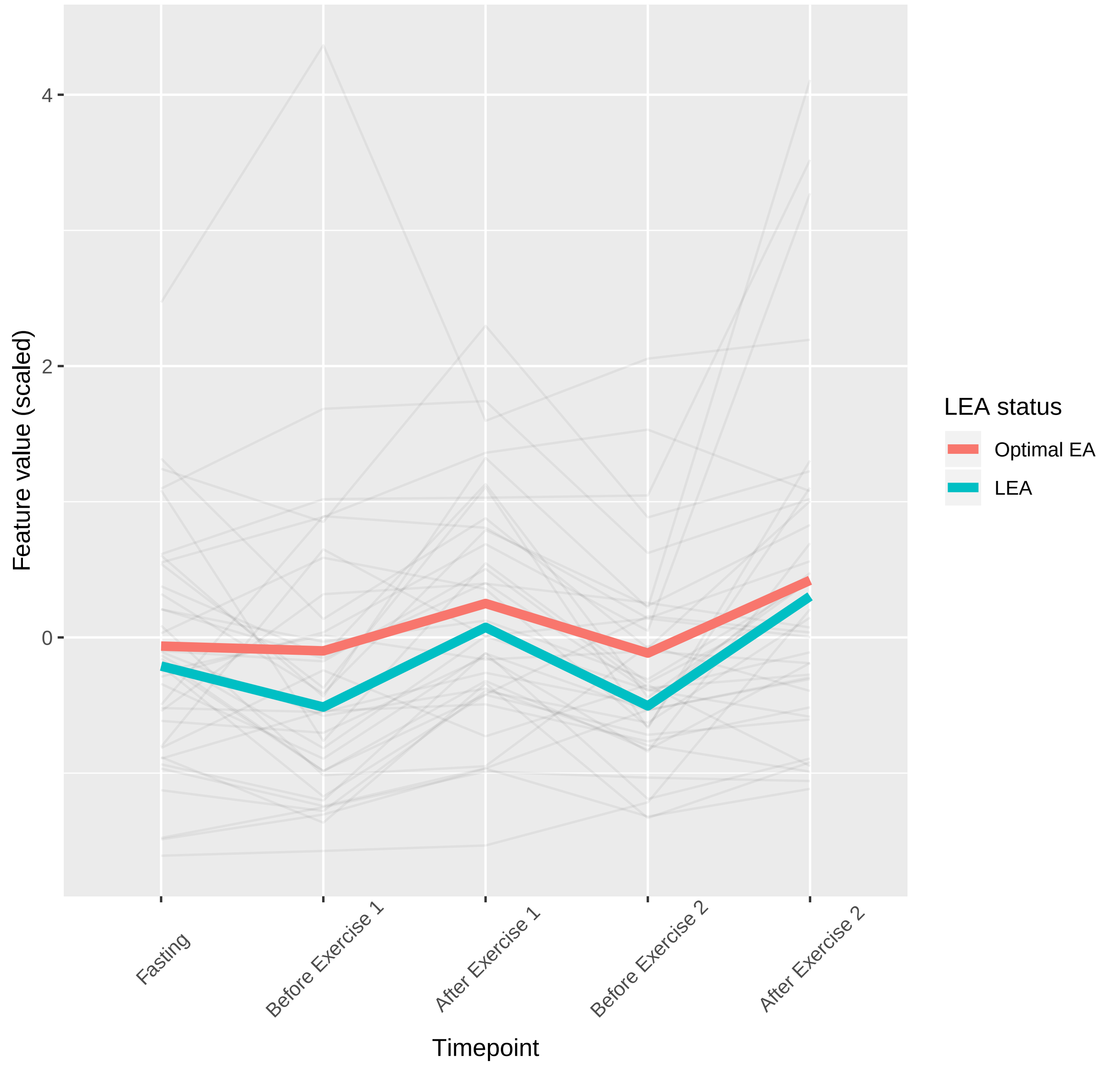

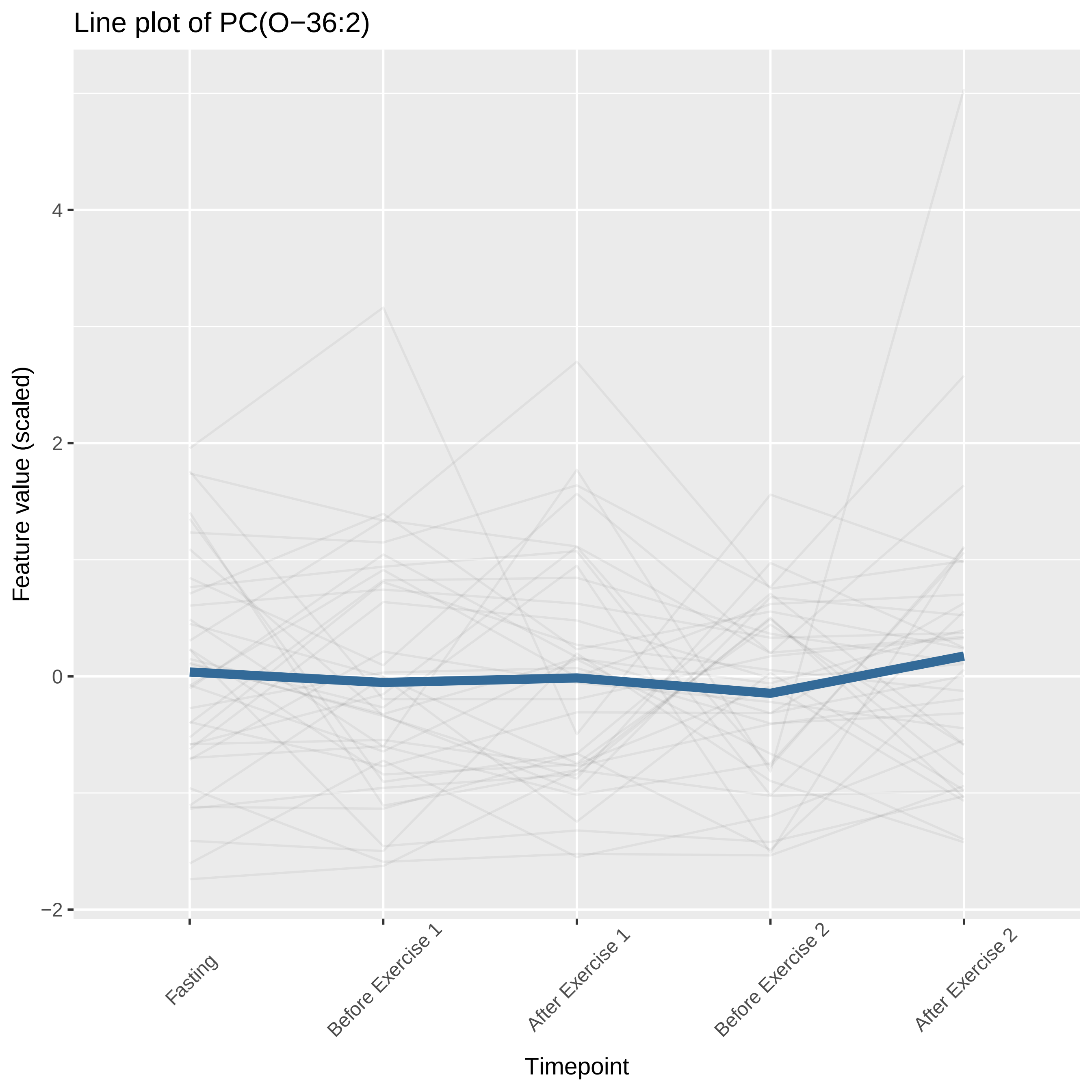

Line plot of PC(O−36:2)
Feature value (scaled)
4
2
0
−2
Fasting
Before Exercise 1
After Exercise 1
Before Exercise 2
After Exercise 2
Timepoint

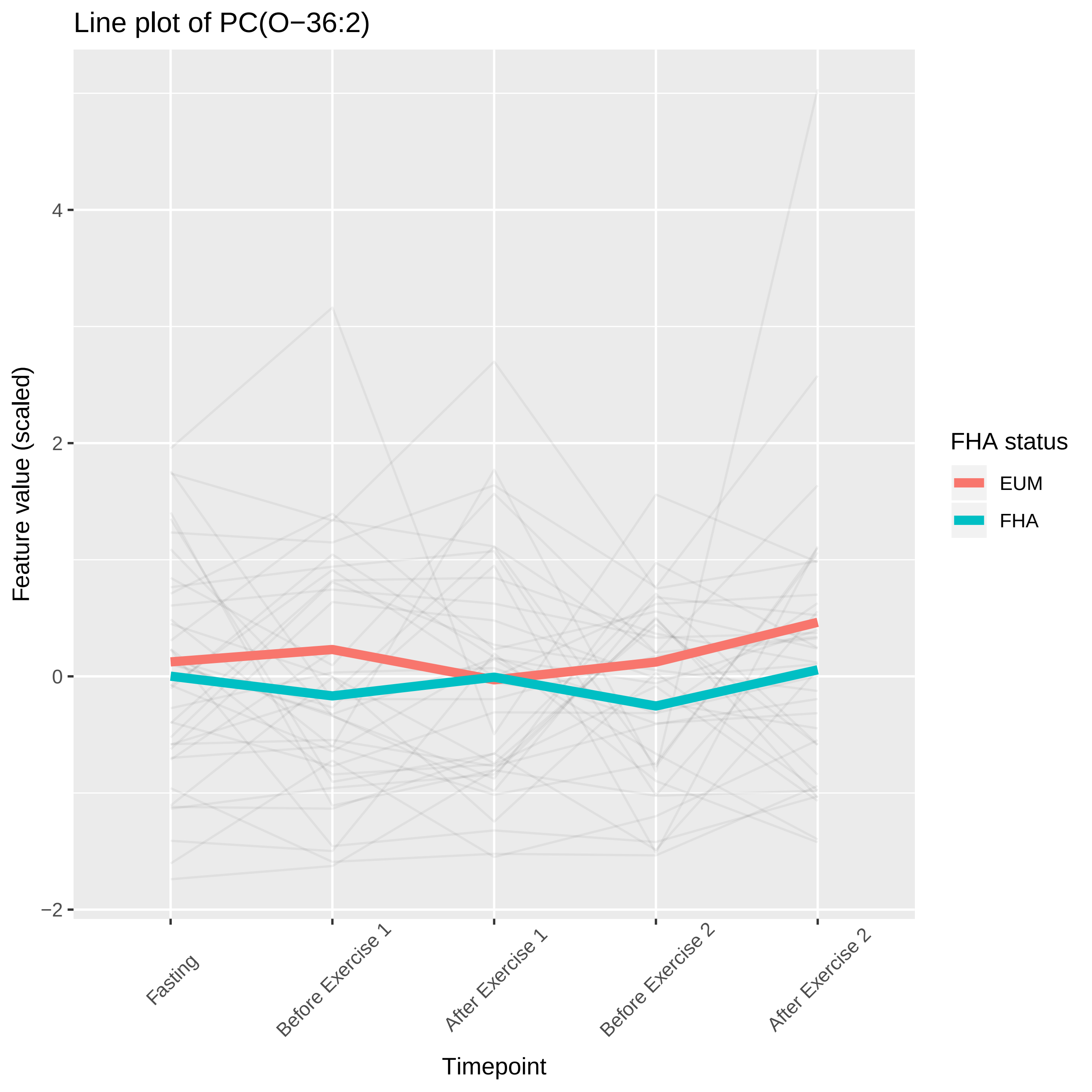
Line plot of PC(O−36:2)
Feature value (scaled)
4
2
0
−2
Fasting
Before Exercise 1
After Exercise 1
Before Exercise 2
After Exercise 2
Timepoint
FHA status
EUM
FHA

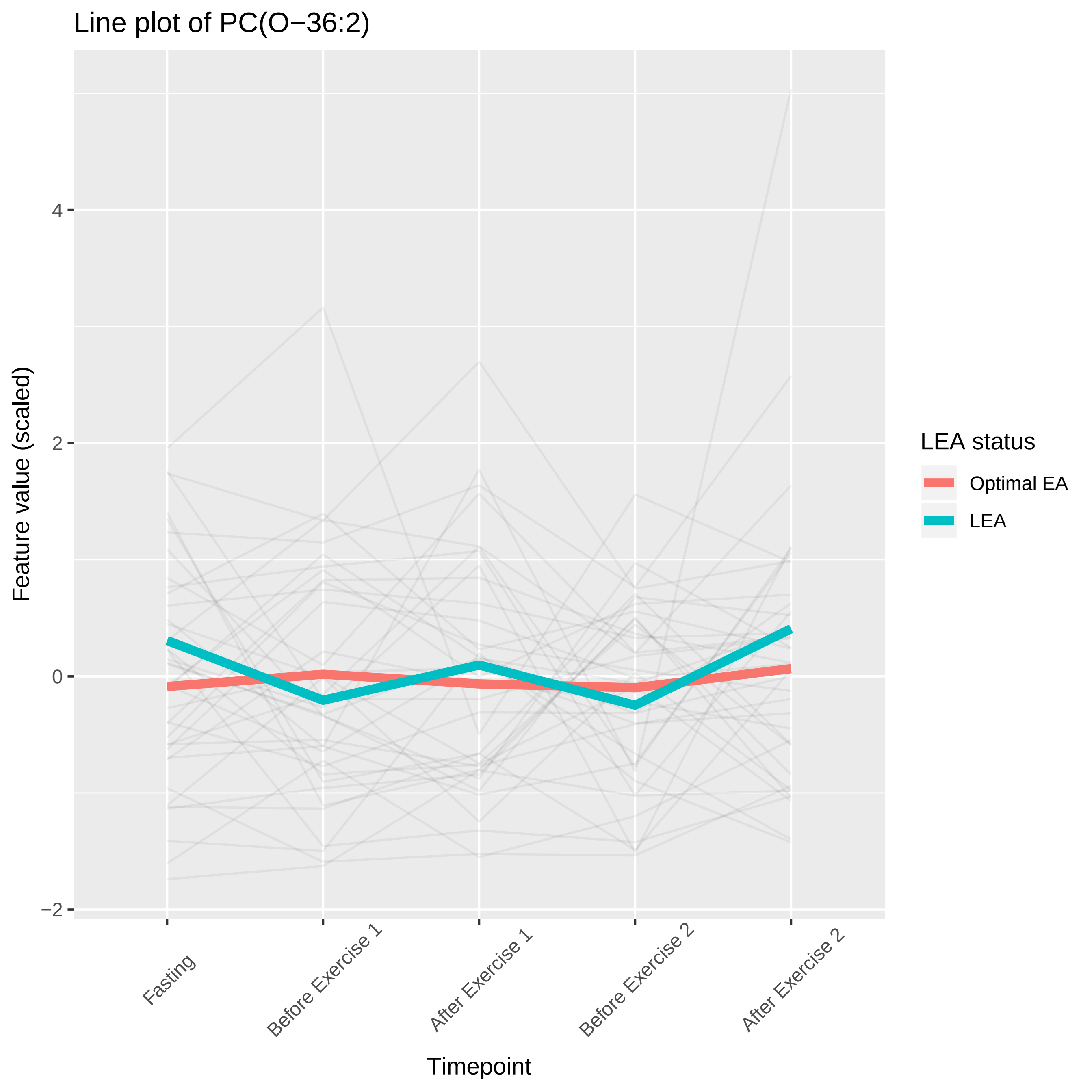

Line plot of PC(O−36:2)
Feature value (scaled)
4
2
0
−2
Fasting
Before Exercise 1
After Exercise 1
Before Exercise 2
After Exercise 2
Timepoint
LEA status
Optimal EA
LEA

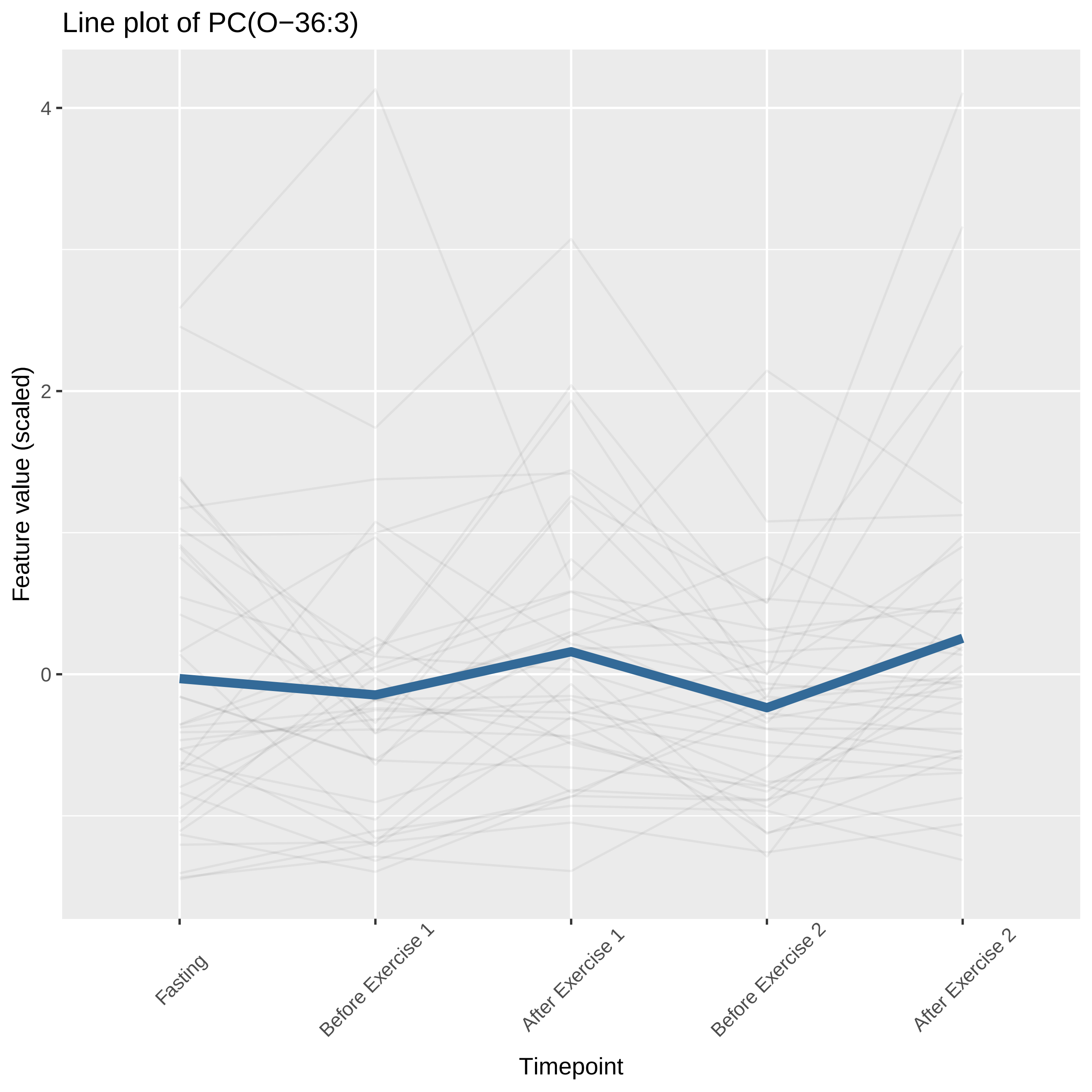

Line plot of PC(O−36:3)
Feature value (scaled)
4
2
0
Fasting
Before Exercise 1
After Exercise 1
Before Exercise 2
After Exercise 2
Timepoint

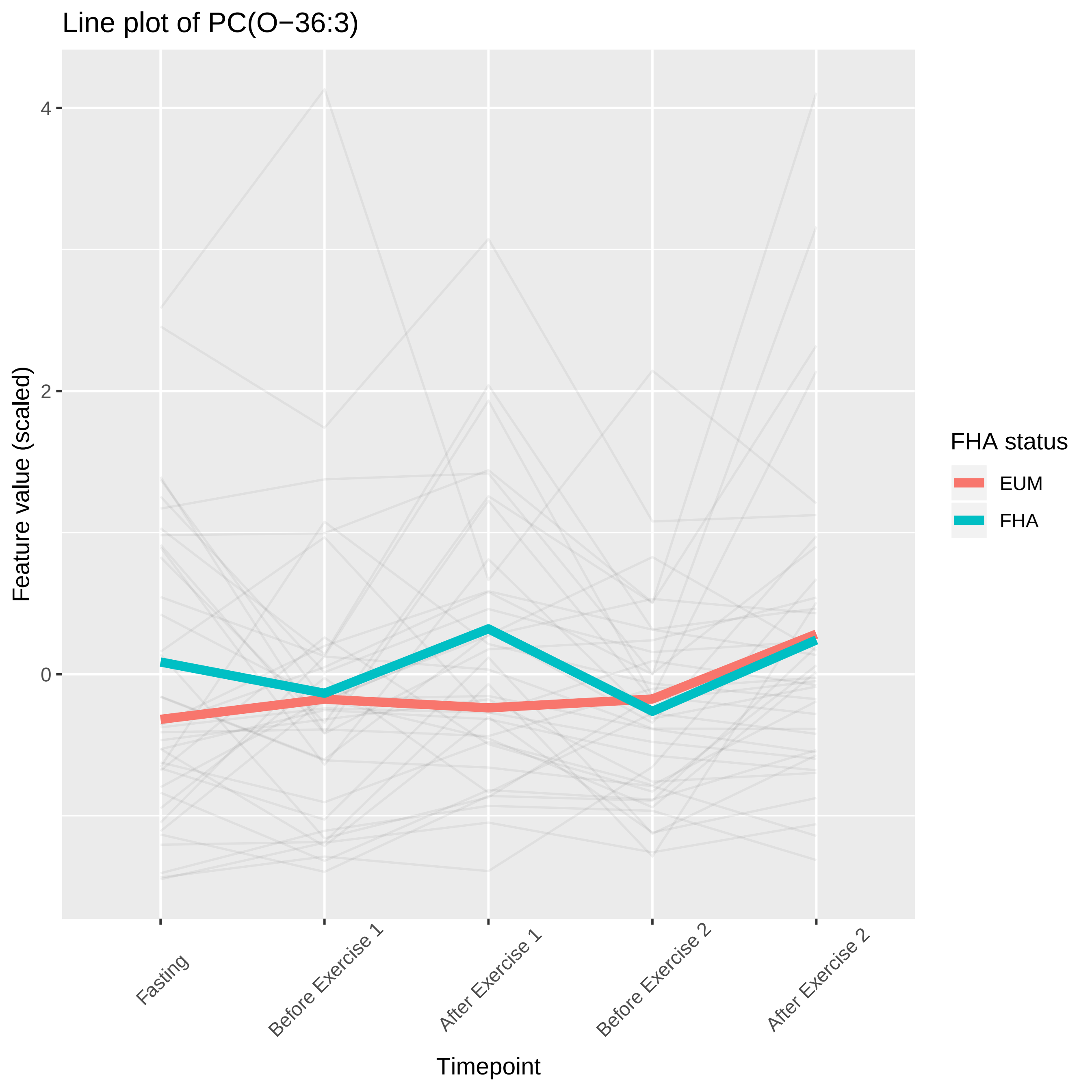

Line plot of PC(O−36:3)
Feature value (scaled)
4
2
0
Fasting
Before Exercise 1
After Exercise 1
Before Exercise 2
After Exercise 2
Timepoint
FHA status
EUM
FHA

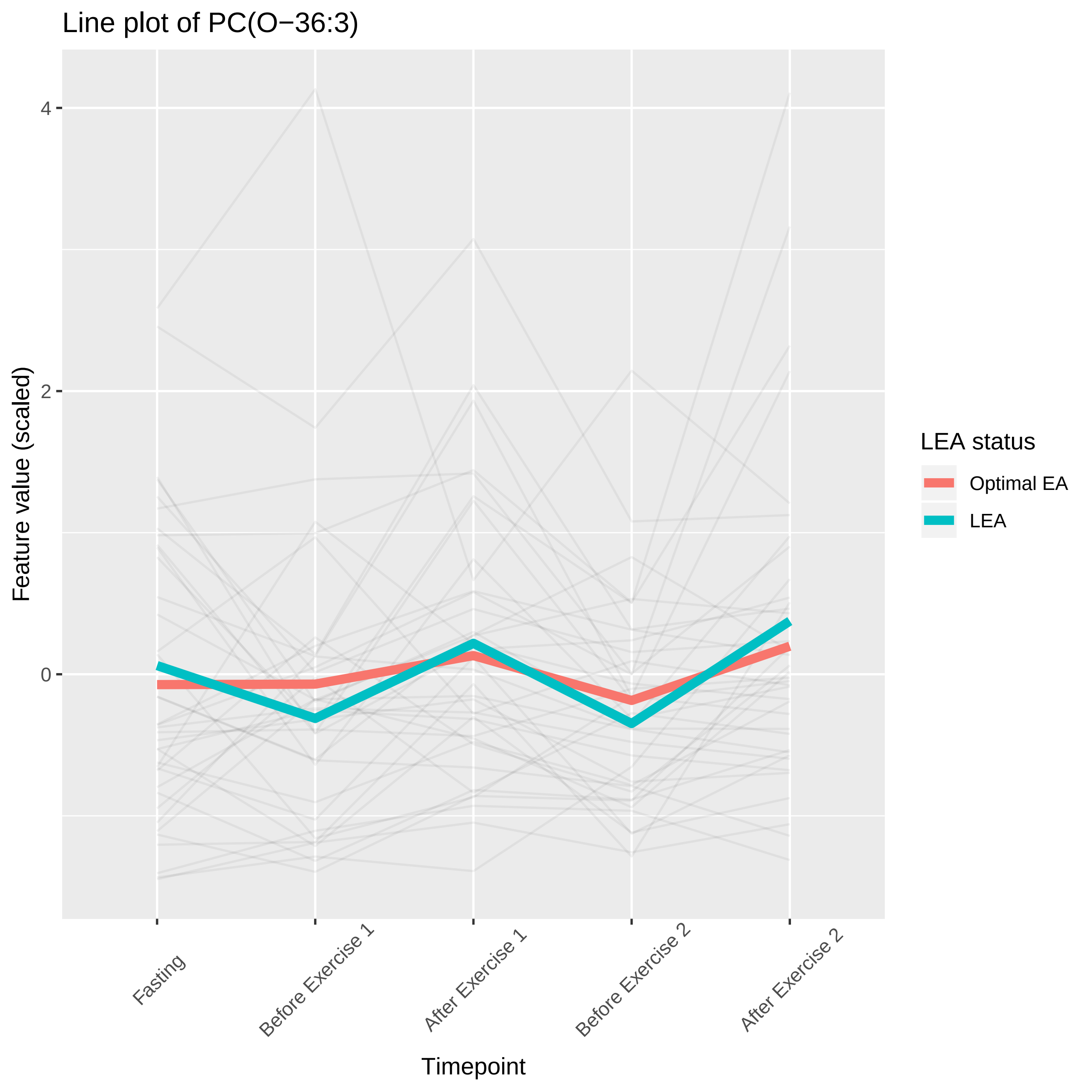

Line plot of PC(O−36:3)
Feature value (scaled)
4
2
0
Fasting
Before Exercise 1
After Exercise 1
Before Exercise 2
After Exercise 2
Timepoint
LEA status
Optimal EA
LEA

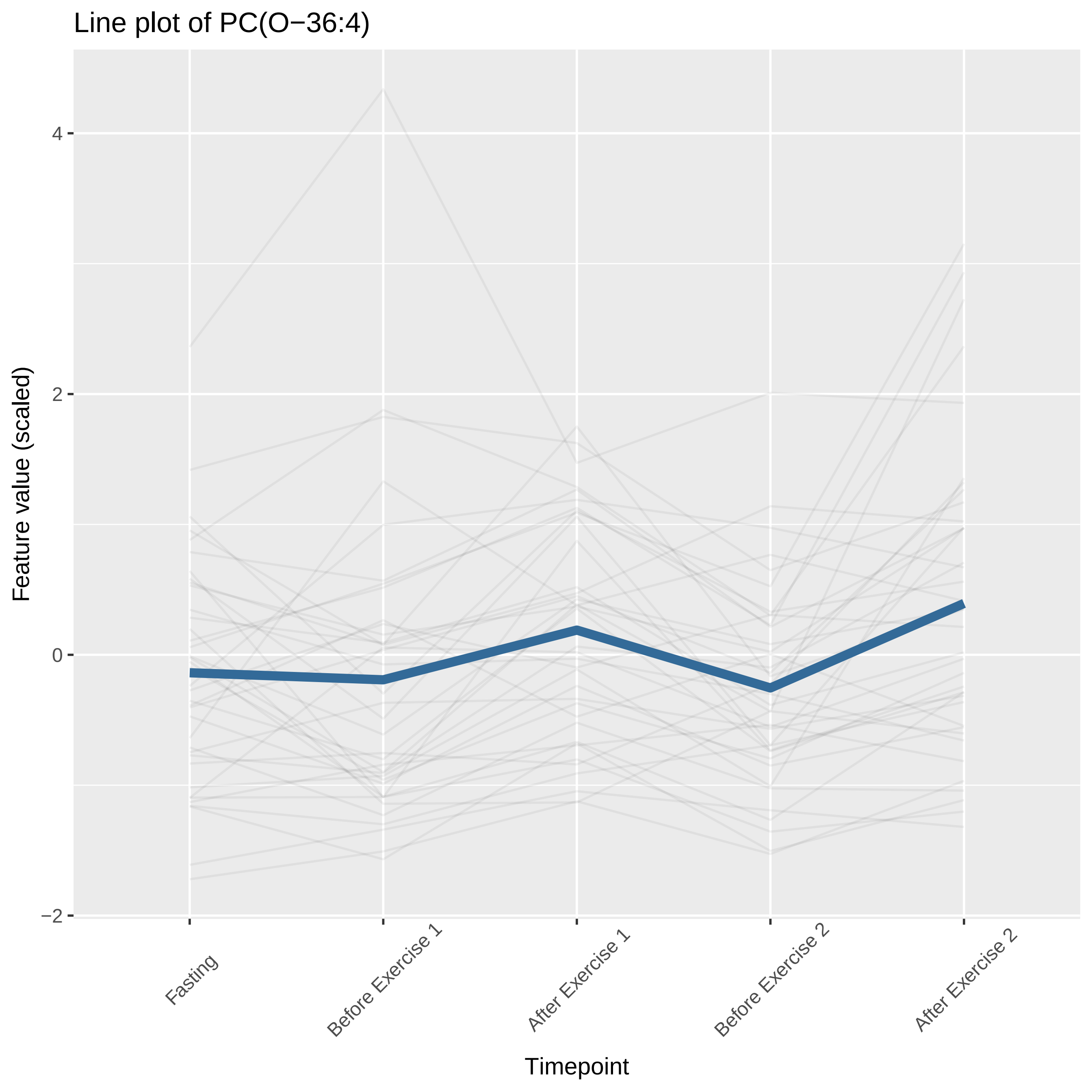

Line plot of PC(O−36:4)
Feature value (scaled)
4
2
0
−2
Fasting
Before Exercise 1
After Exercise 1
Before Exercise 2
After Exercise 2
Timepoint

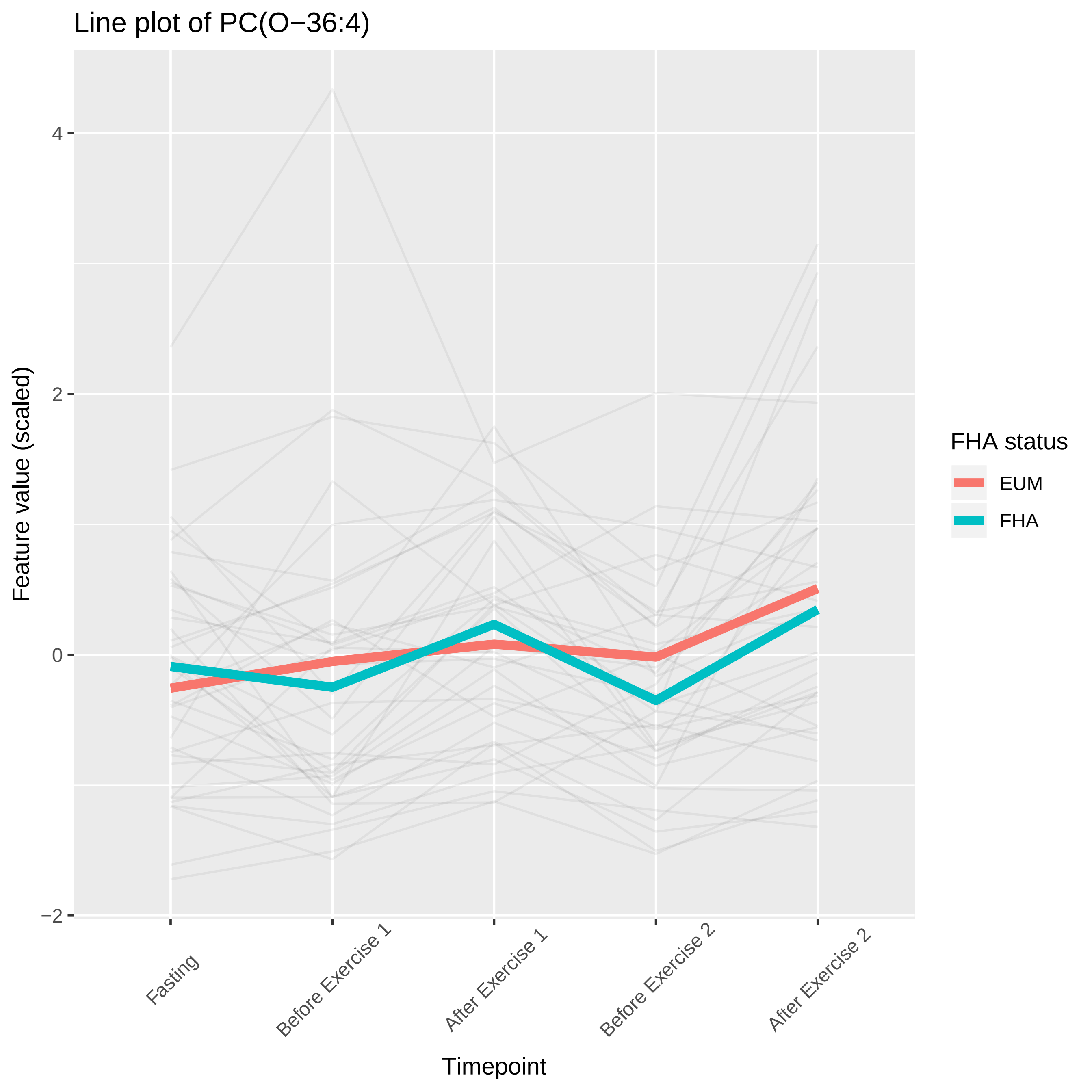

Line plot of PC(O−36:4)
Feature value (scaled)
4
2
0
−2
Fasting
Before Exercise 1
After Exercise 1
Before Exercise 2
After Exercise 2
Timepoint
FHA status
EUM
FHA

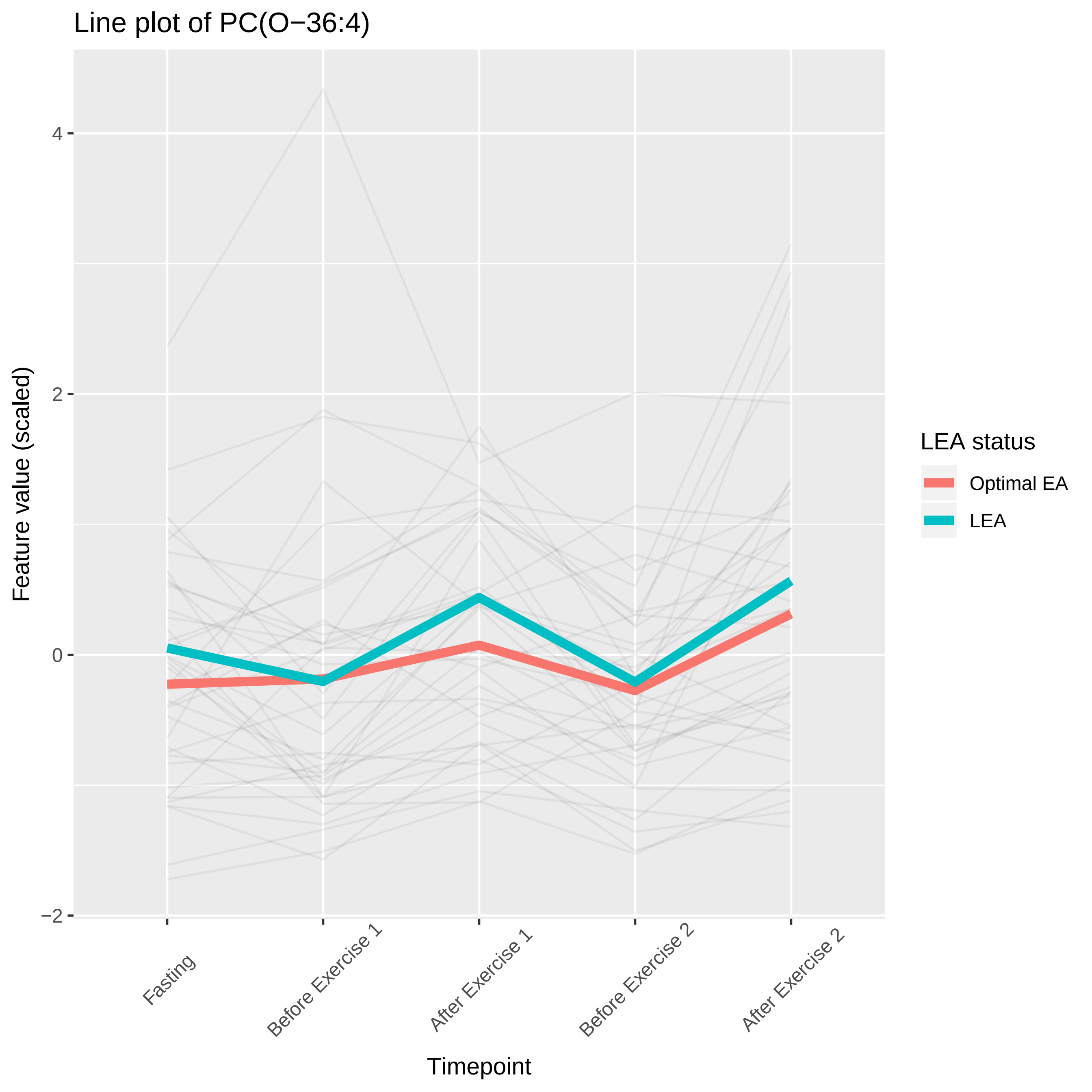
Line plot of PC(O−36:4)
Feature value (scaled)
4
2
0
−2
Fasting
Before Exercise 1
After Exercise 1
Before Exercise 2
After Exercise 2
Timepoint
LEA status
Optimal EA
LEA

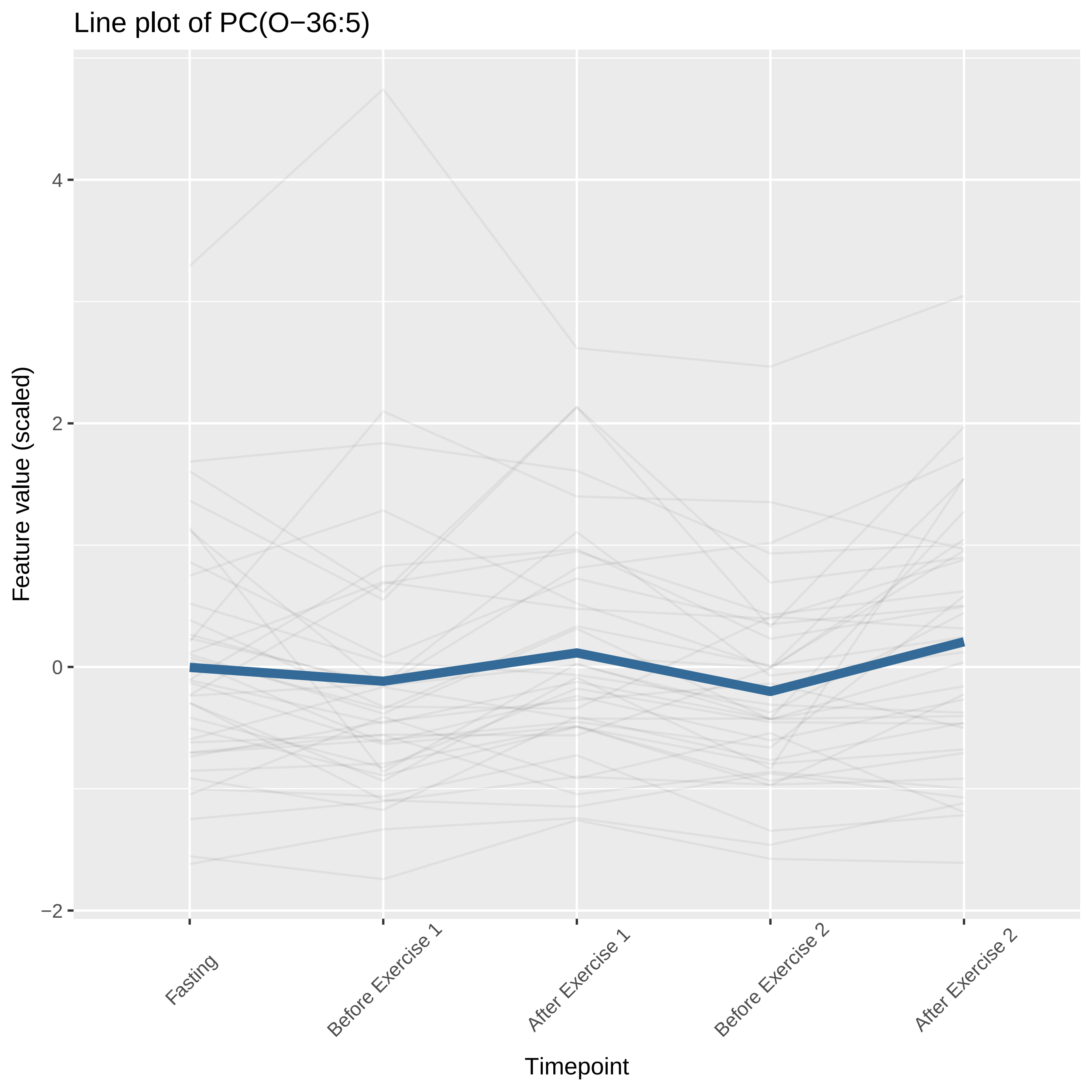

Line plot of PC(O−36:5)
Feature value (scaled)
4
2
0
−2
Fasting
Before Exercise 1
After Exercise 1
Before Exercise 2
After Exercise 2
Timepoint

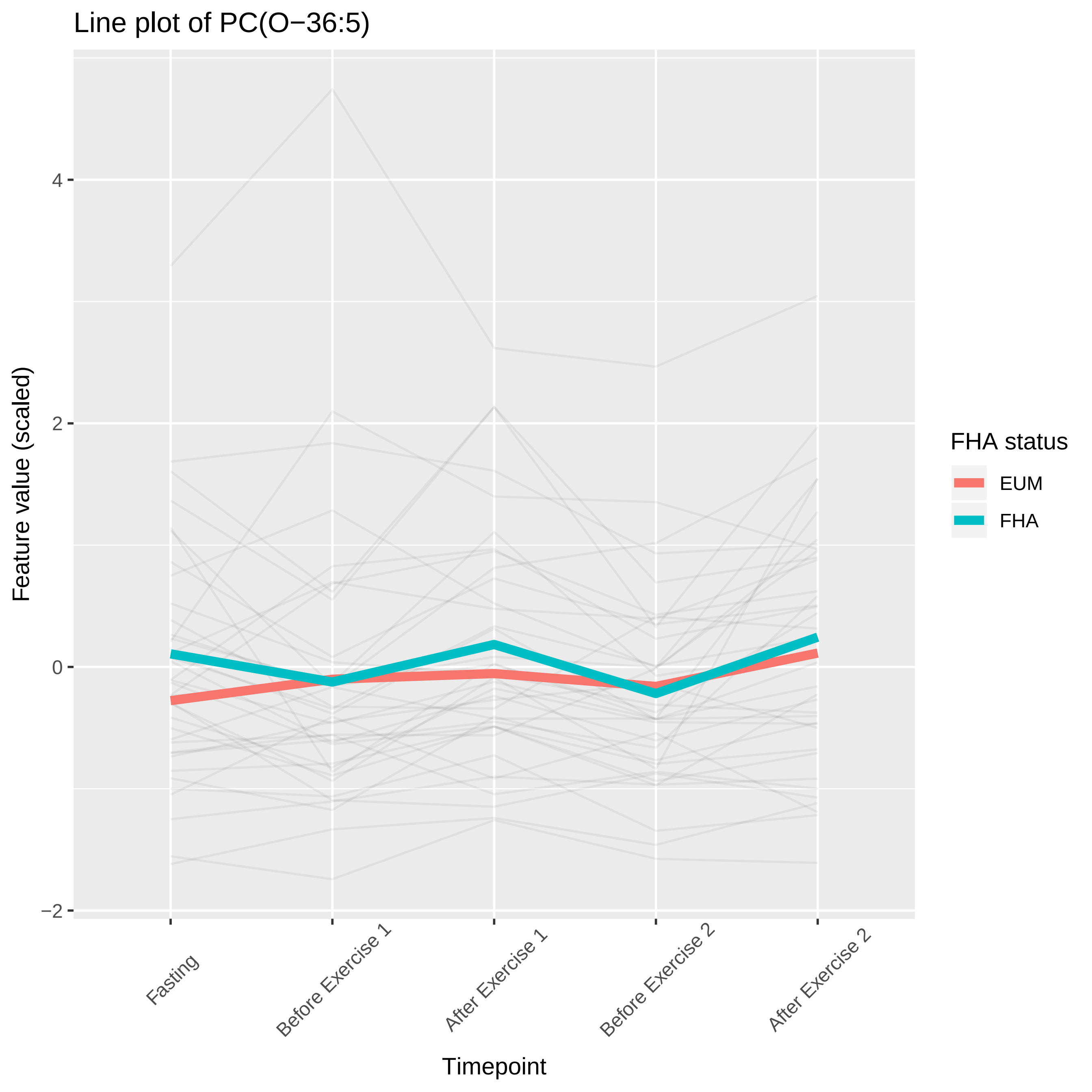

Line plot of PC(O−36:5)
Feature value (scaled)
4
2
0
−2
Fasting
Before Exercise 1
After Exercise 1
Before Exercise 2
After Exercise 2
Timepoint
FHA status
EUM
FHA

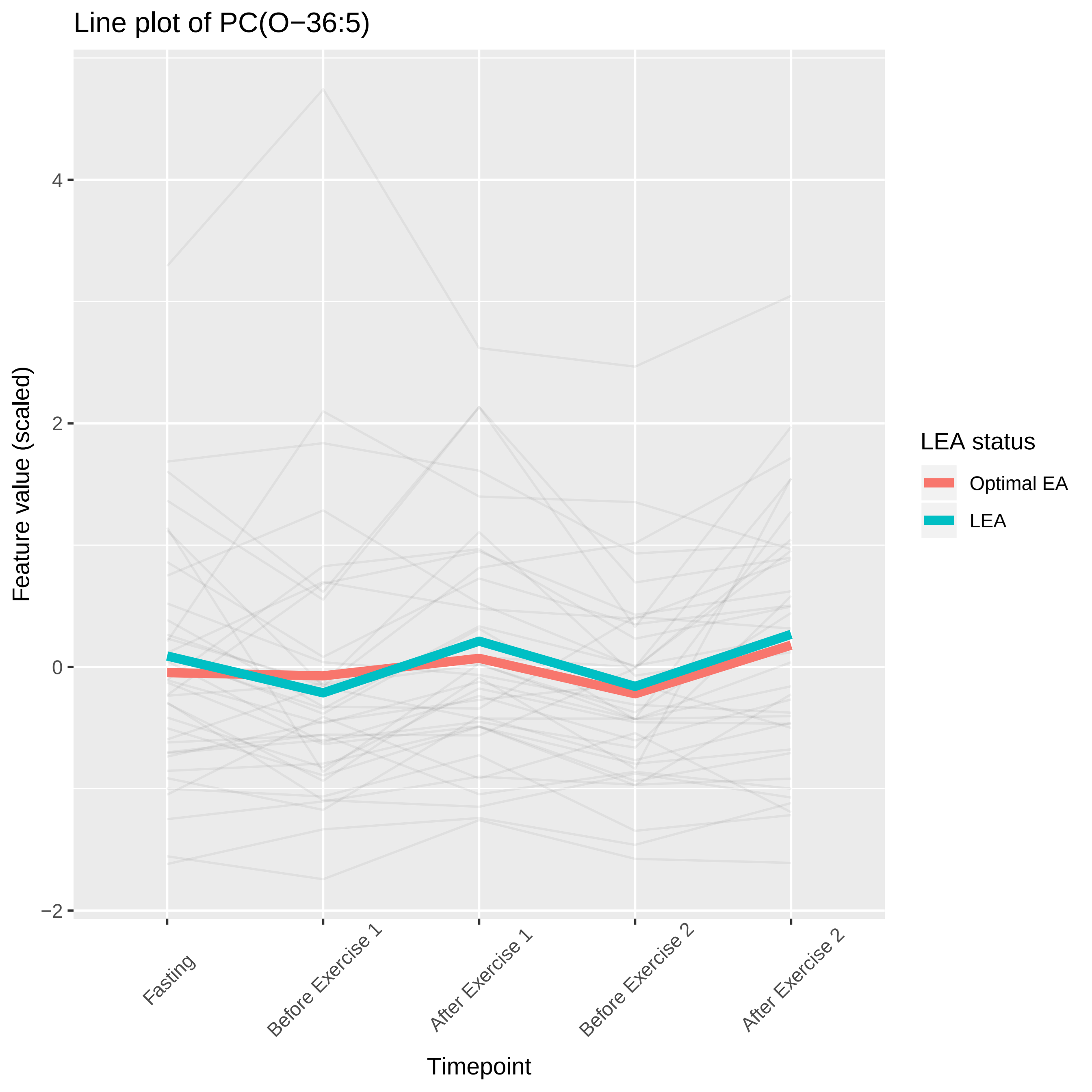

Line plot of PC(O−36:5)
Feature value (scaled)
4
2
0
−2
Fasting
Before Exercise 1
After Exercise 1
Before Exercise 2
After Exercise 2
Timepoint
LEA status
Optimal EA
LEA

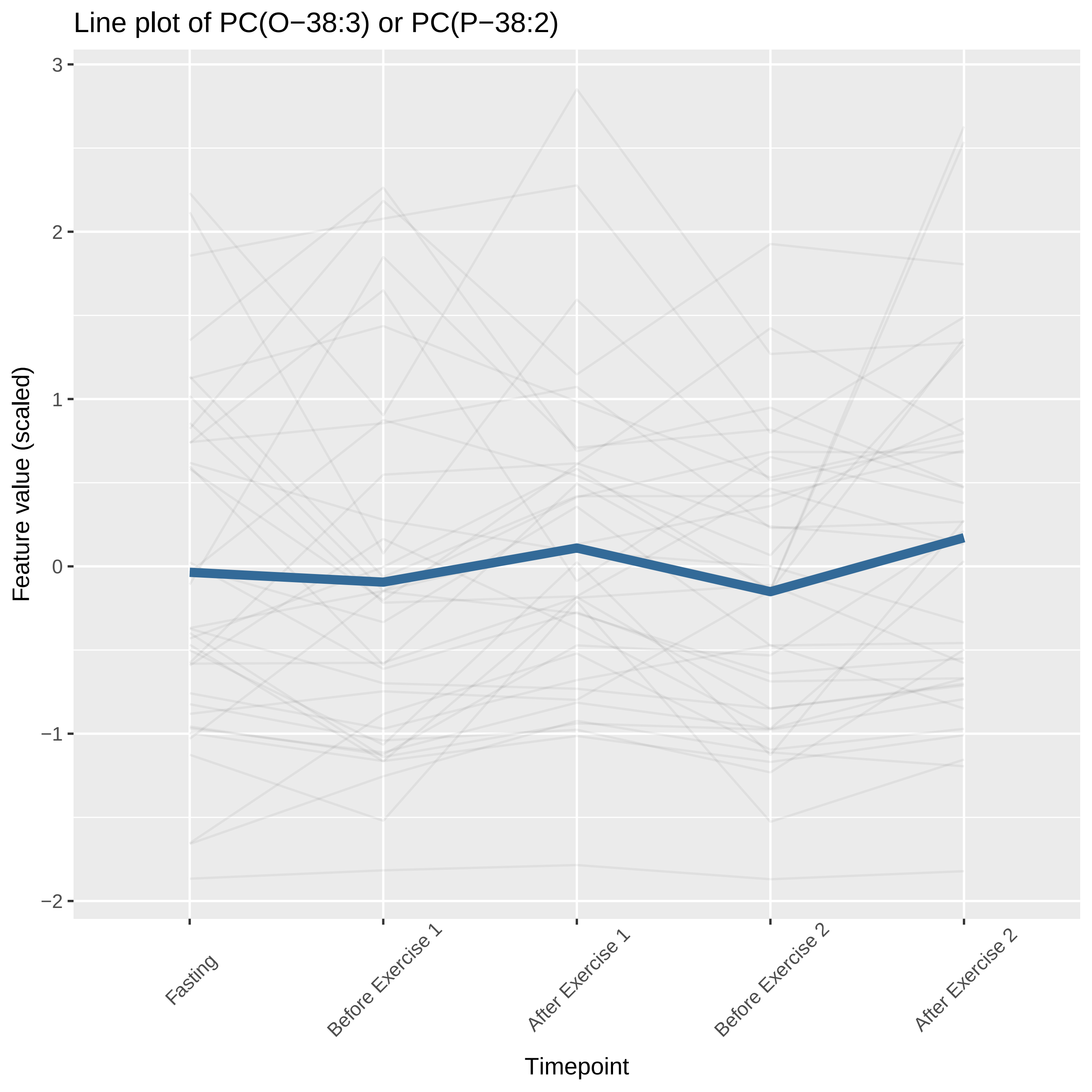
Line plot of PC(O−38:3) or PC(P−38:2)
Feature value (scaled)
3
2
1
0
−1
−2
Fasting
Before Exercise 1
After Exercise 1
Before Exercise 2
After Exercise 2
Timepoint

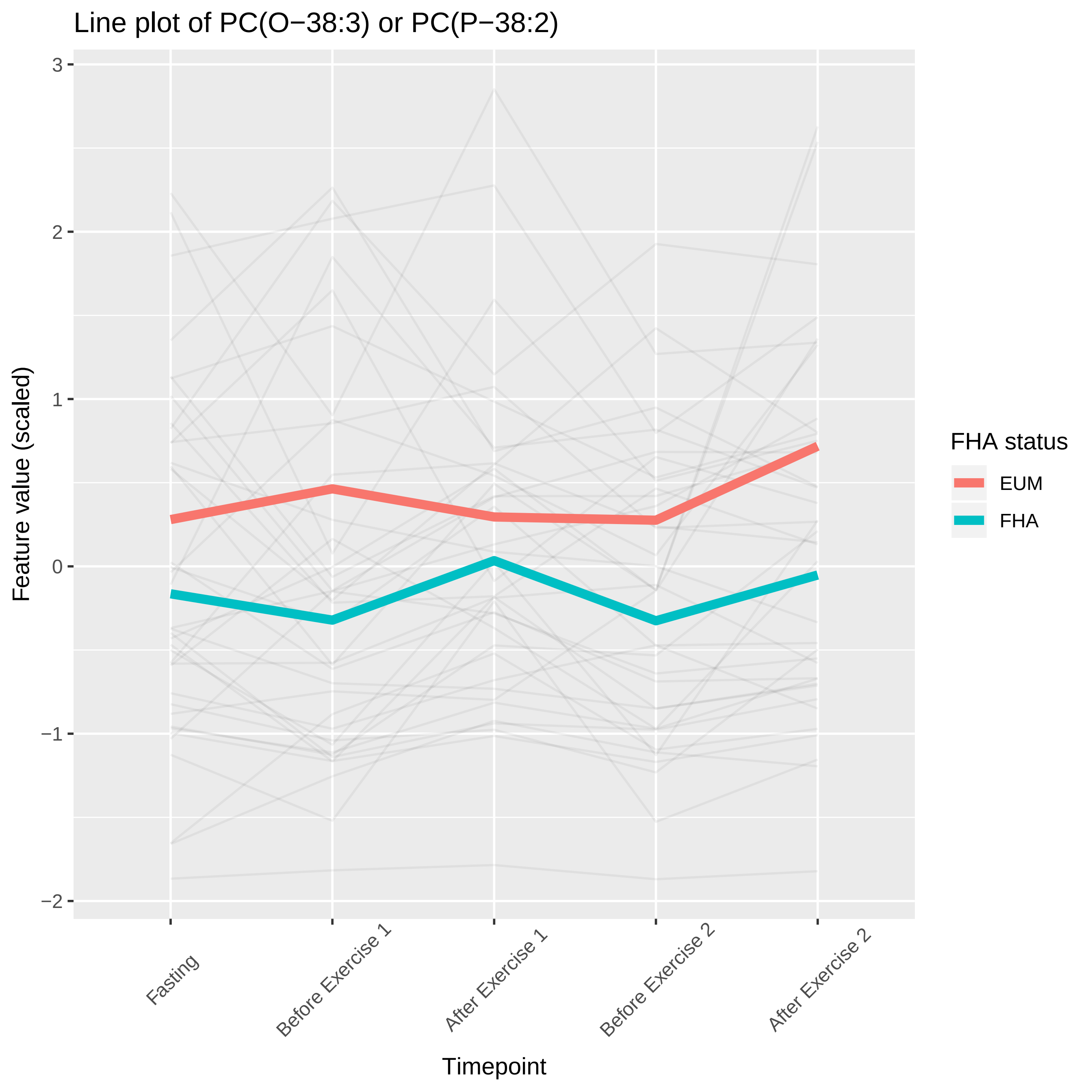

Line plot of PC(O−38:3) or PC(P−38:2)
Feature value (scaled)
3
2
1
0
−1
−2
Fasting
Before Exercise 1
After Exercise 1
Before Exercise 2
After Exercise 2
Timepoint
FHA status
EUM
FHA

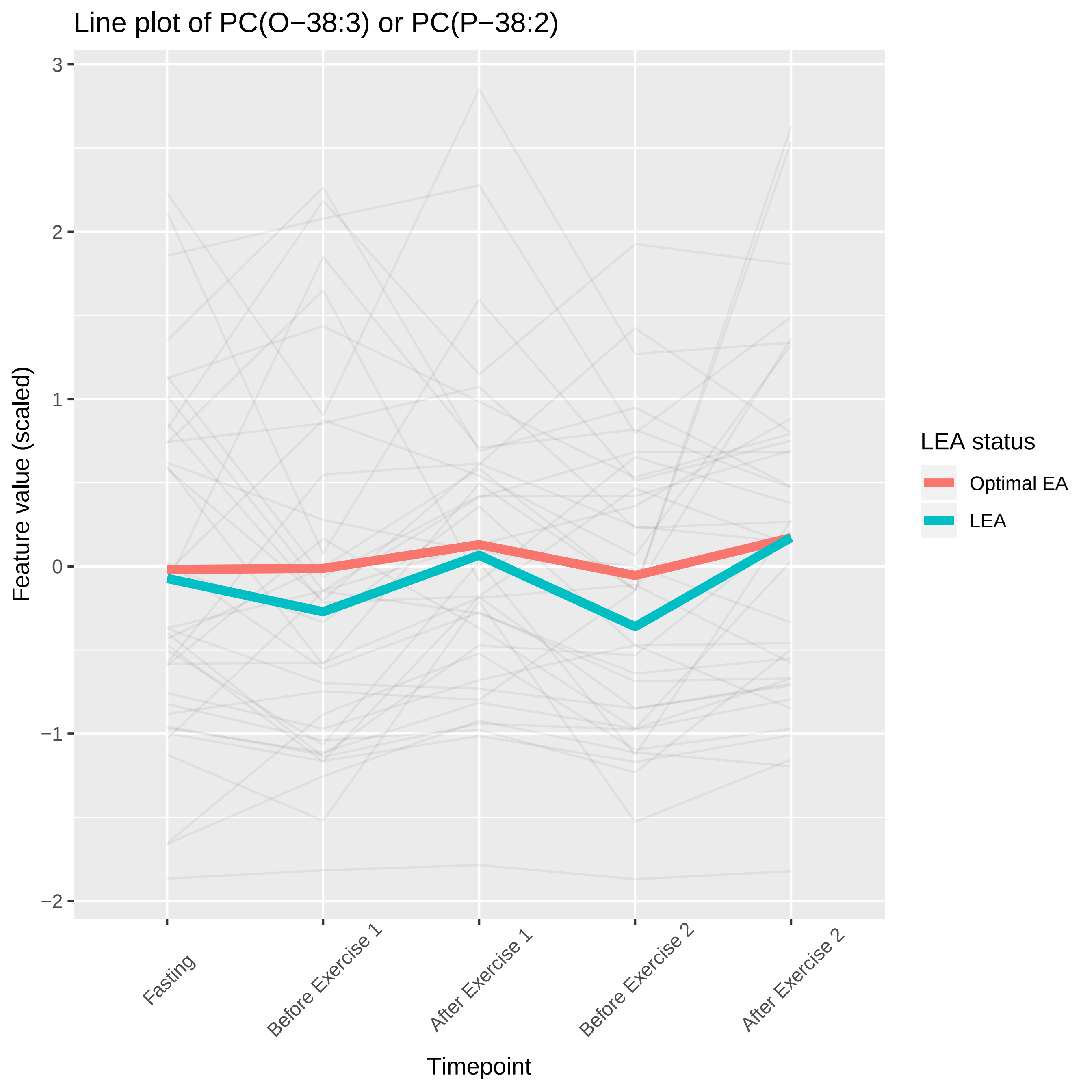

Line plot of PC(O−38:3) or PC(P−38:2)
Feature value (scaled)
3
2
1
0
−1
−2
Fasting
Before Exercise 1
After Exercise 1
Before Exercise 2
After Exercise 2
Timepoint
LEA status
Optimal EA
LEA

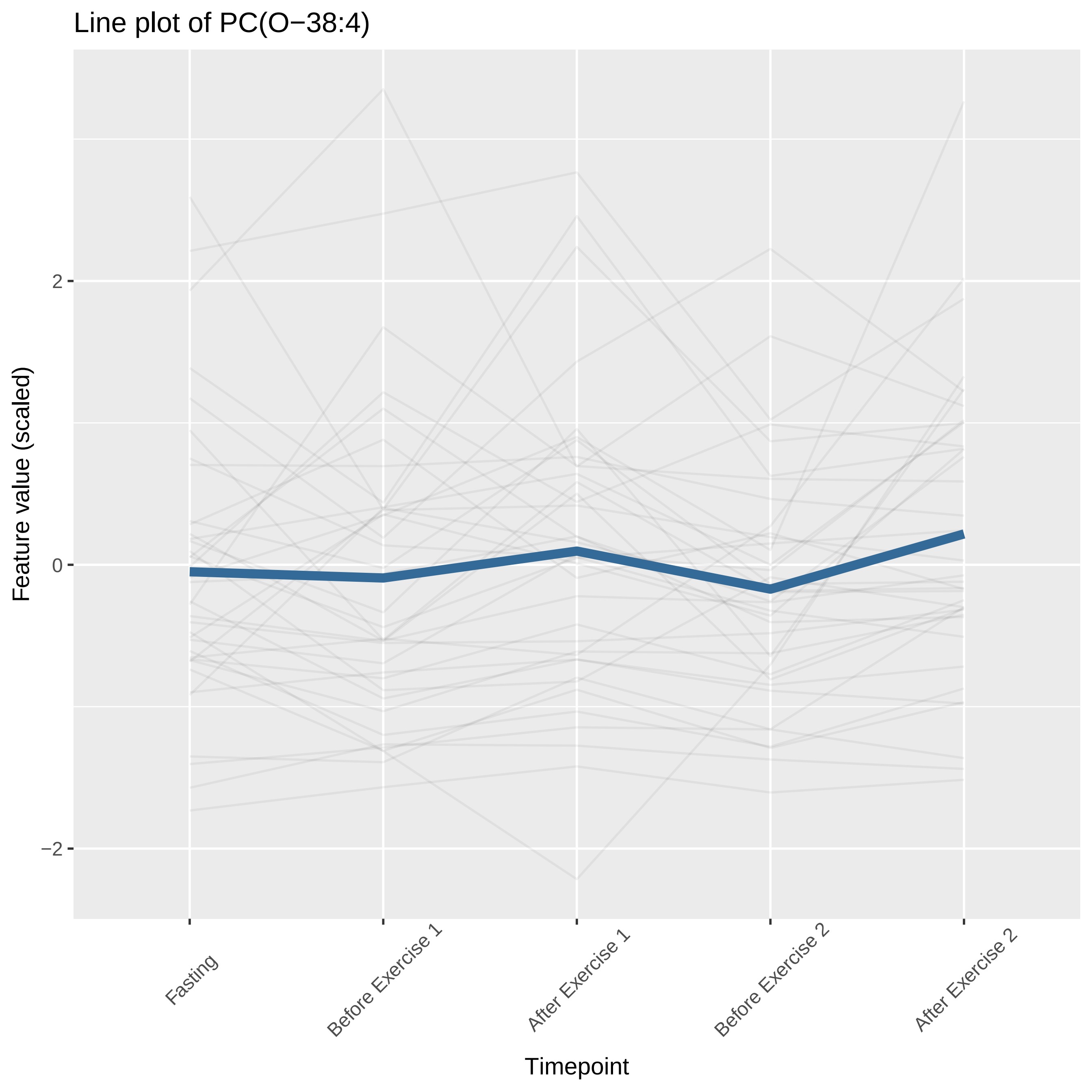

Line plot of PC(O−38:4)
Feature value (scaled)
2
0
−2
Fasting
Before Exercise 1
After Exercise 1
Before Exercise 2
After Exercise 2
Timepoint

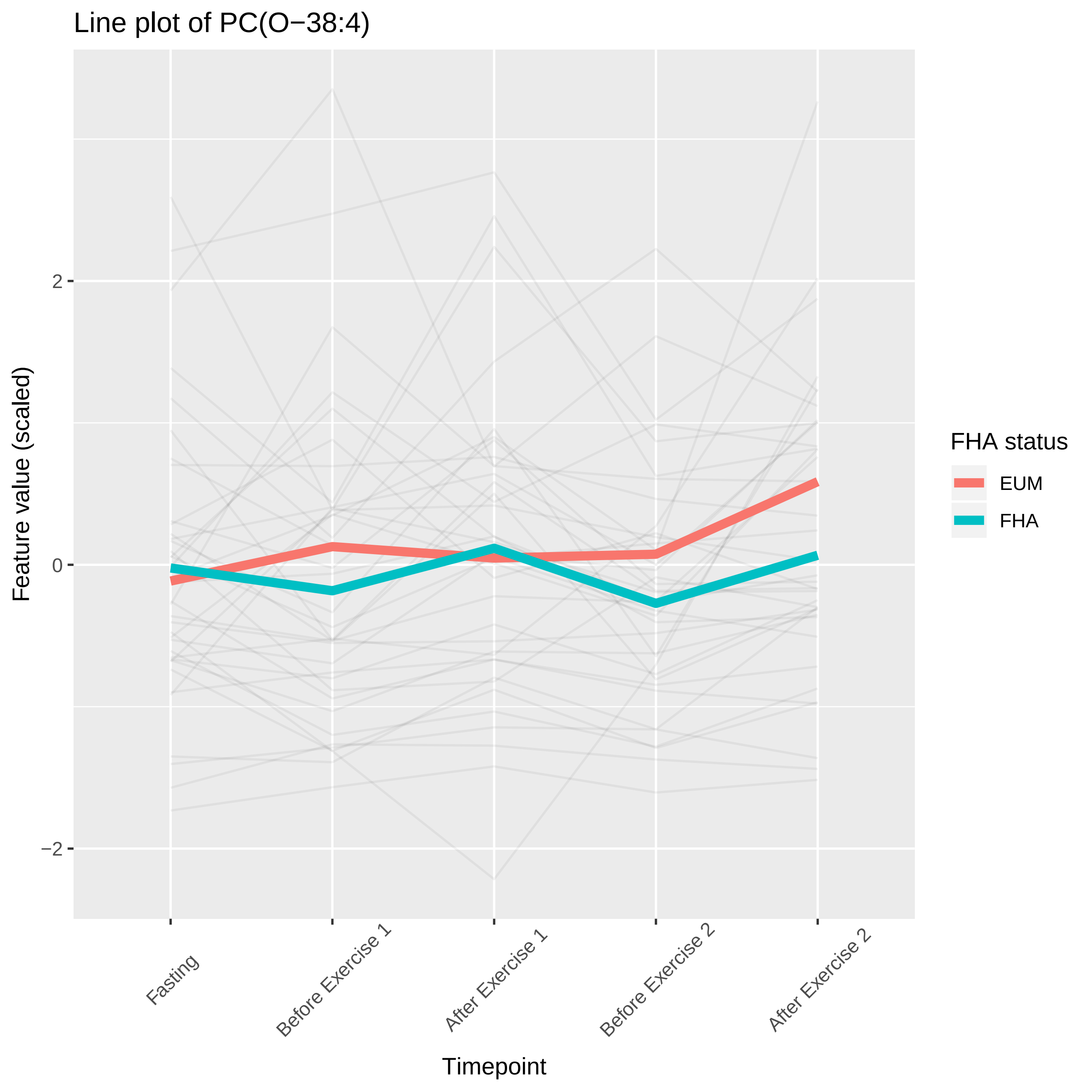

Line plot of PC(O−38:4)
Feature value (scaled)
2
0
−2
Fasting
Before Exercise 1
After Exercise 1
Before Exercise 2
After Exercise 2
Timepoint
FHA status
EUM
FHA

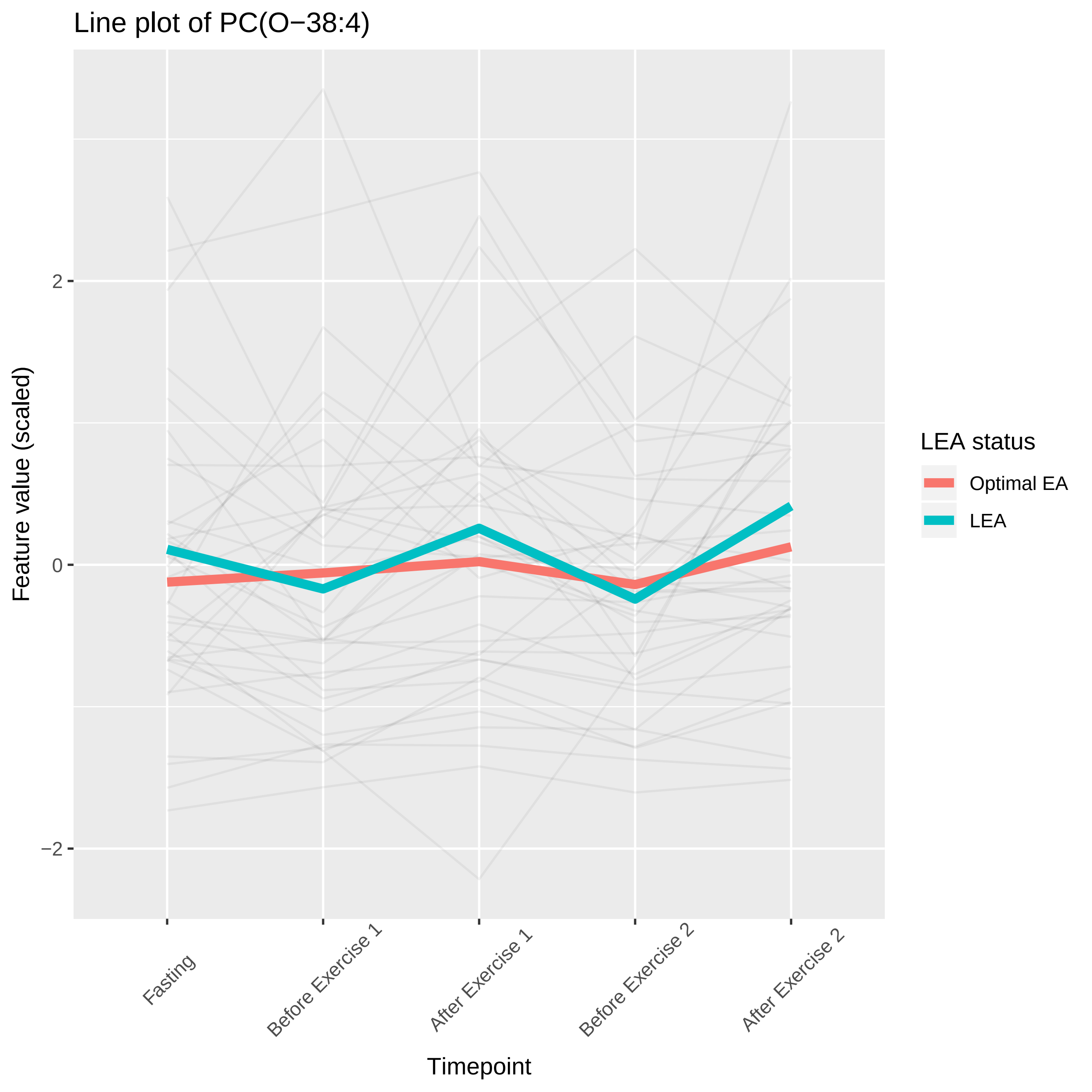
Line plot of PC(O−38:4)
Feature value (scaled)
2
0
−2
Fasting
Before Exercise 1
After Exercise 1
Before Exercise 2
After Exercise 2
Timepoint
LEA status
Optimal EA
LEA

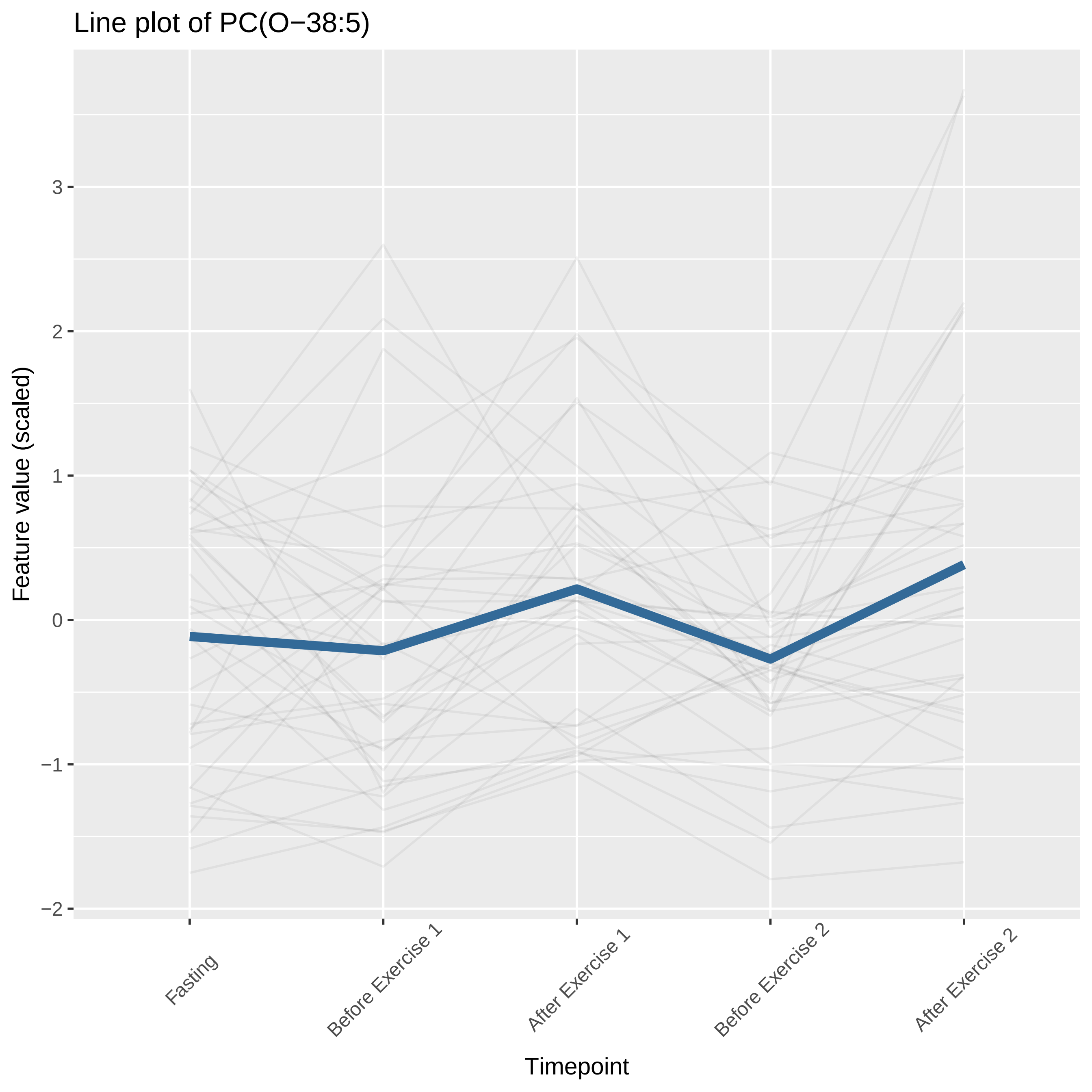

Line plot of PC(O−38:5)
Feature value (scaled)
3
2
1
0
−1
−2
Fasting
Before Exercise 1
After Exercise 1
Before Exercise 2
After Exercise 2
Timepoint

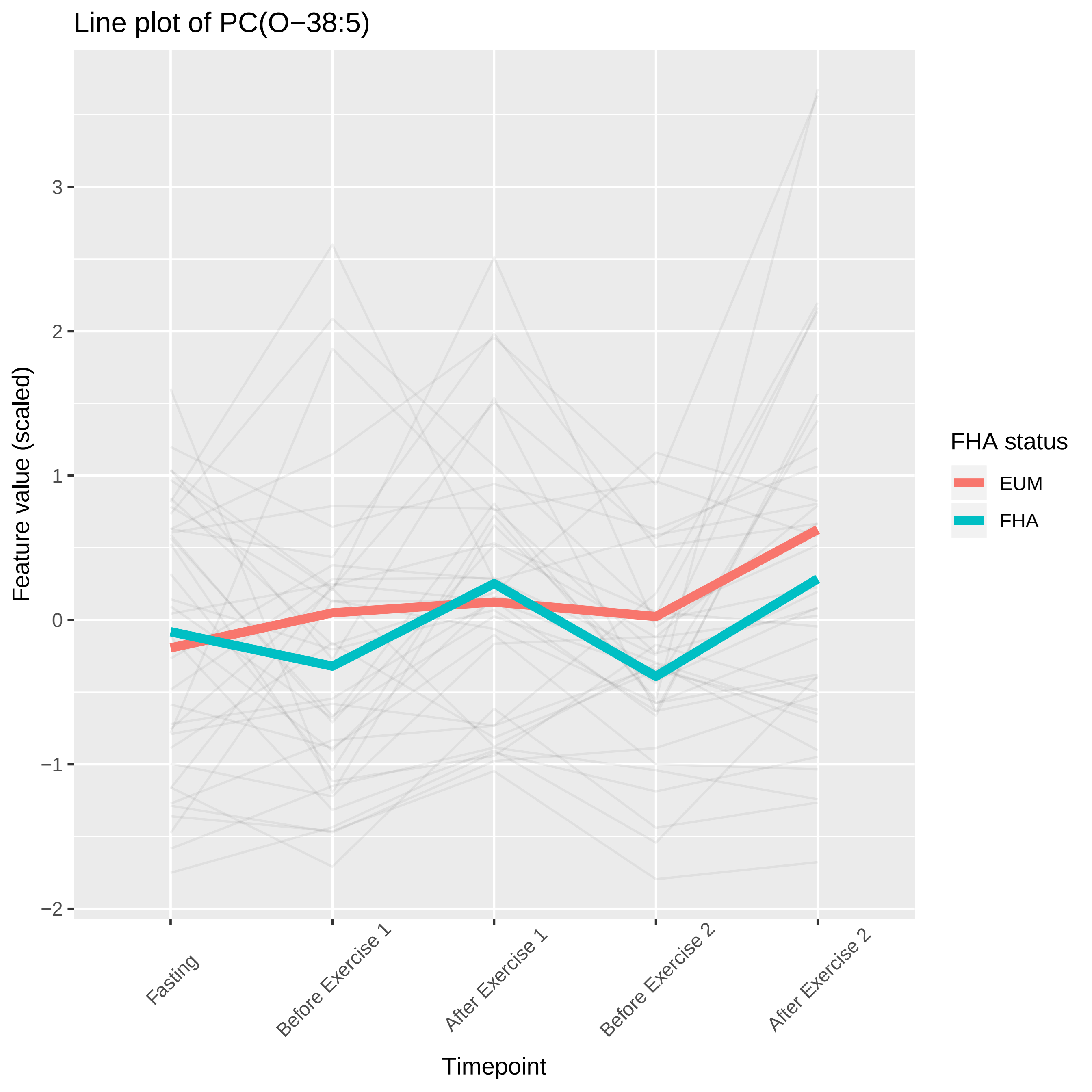

Line plot of PC(O−38:5)
Feature value (scaled)
3
2
1
0
−1
−2
Fasting
Before Exercise 1
After Exercise 1
Before Exercise 2
After Exercise 2
Timepoint
FHA status
EUM
FHA

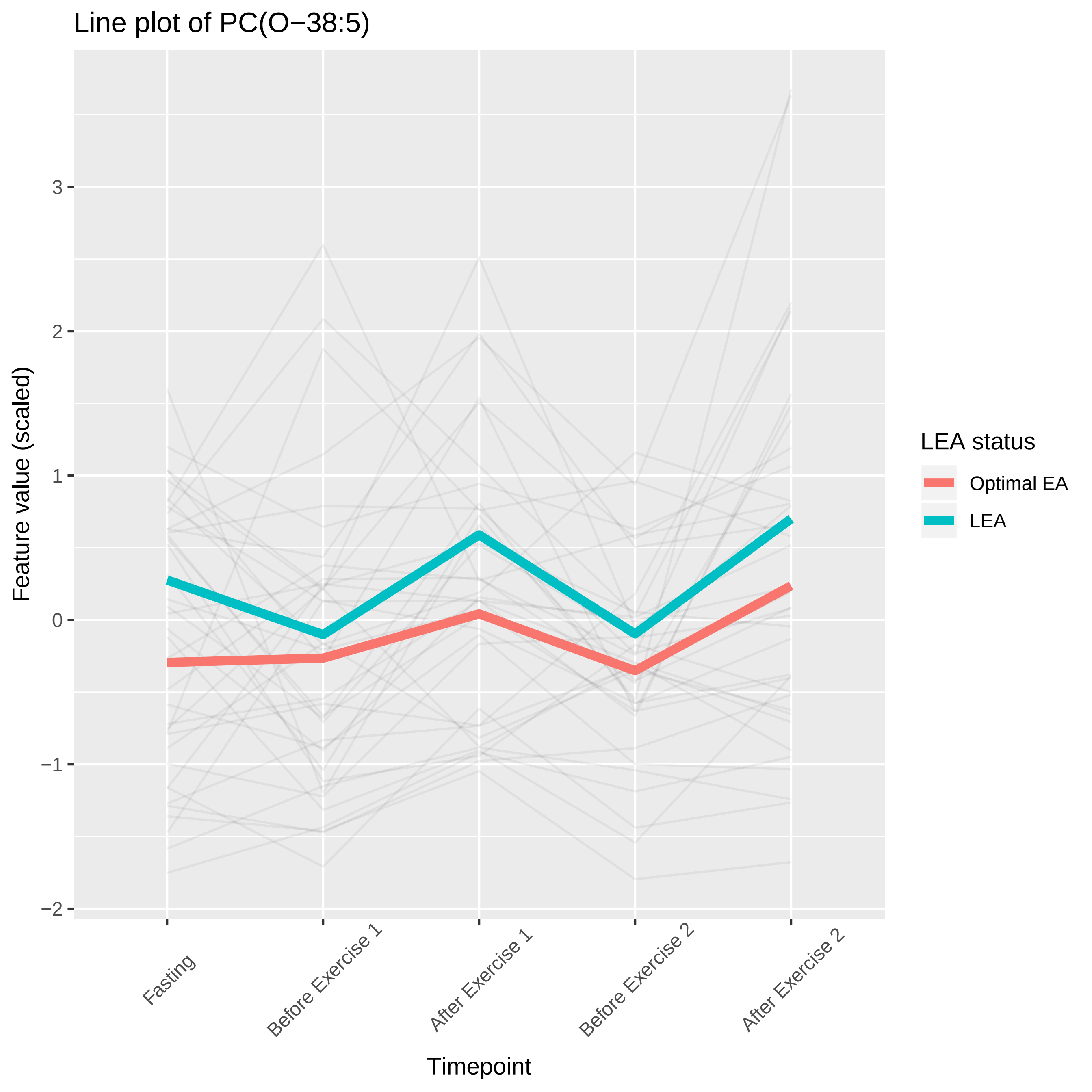

Line plot of PC(O−38:5)
Feature value (scaled)
3
2
1
0
−1
−2
Fasting
Before Exercise 1
After Exercise 1
Before Exercise 2
After Exercise 2
Timepoint
LEA status
Optimal EA
LEA

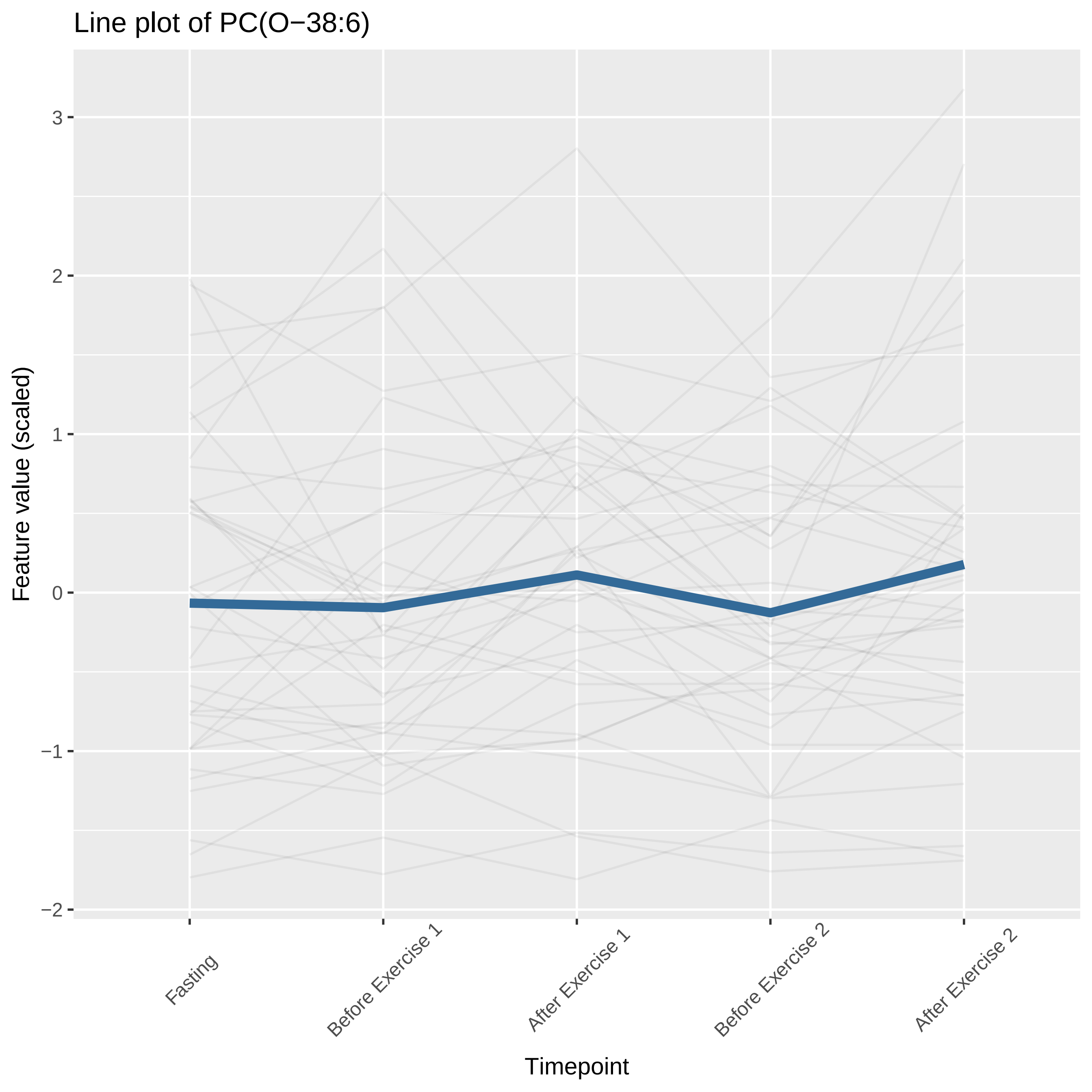
Line plot of PC(O−38:6)
Feature value (scaled)
3
2
1
0
−1
−2
Fasting
Before Exercise 1
After Exercise 1
Before Exercise 2
After Exercise 2
Timepoint

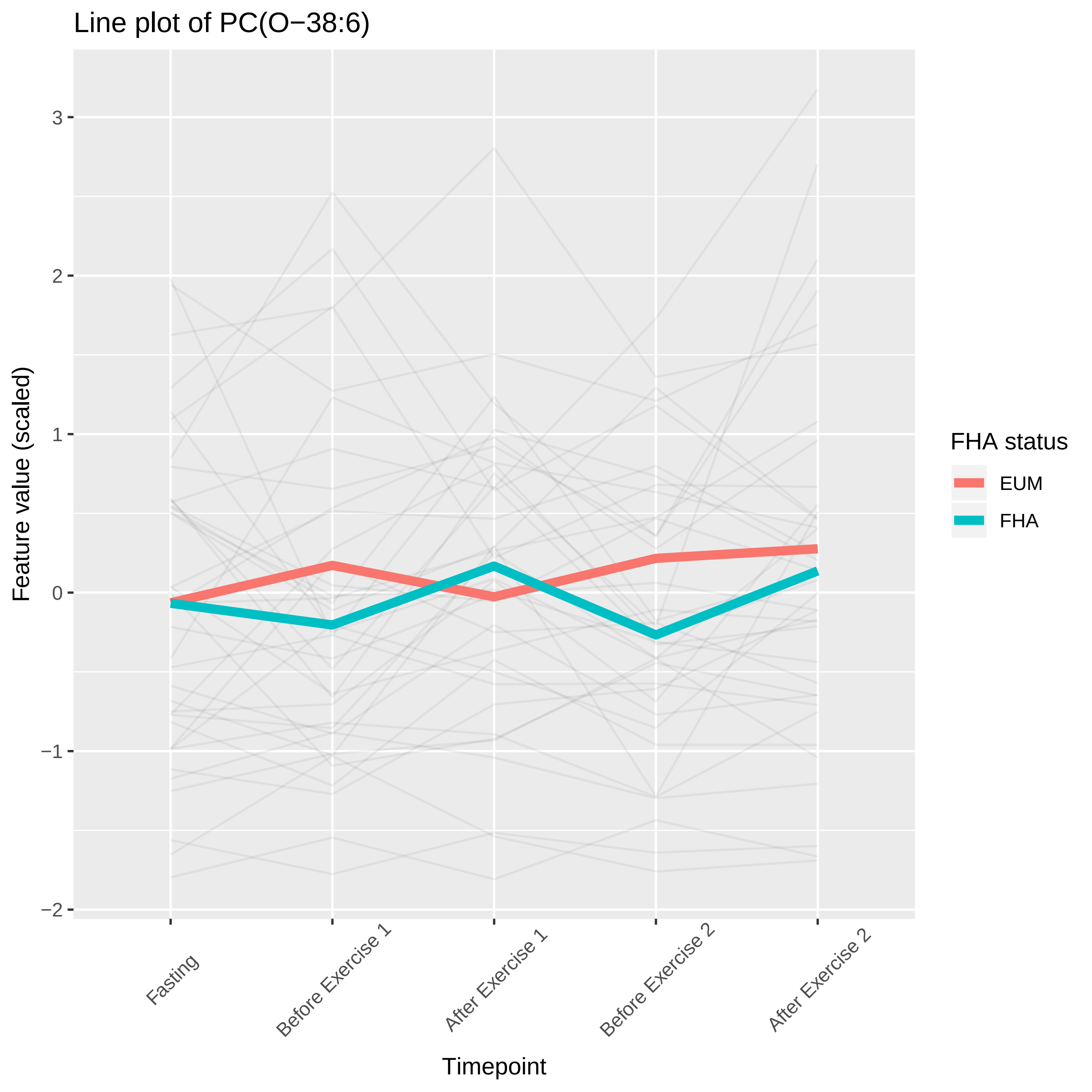

Line plot of PC(O−38:6)
Feature value (scaled)
3
2
1
0
−1
−2
Fasting
Before Exercise 1
After Exercise 1
Before Exercise 2
After Exercise 2
Timepoint
FHA status
EUM
FHA

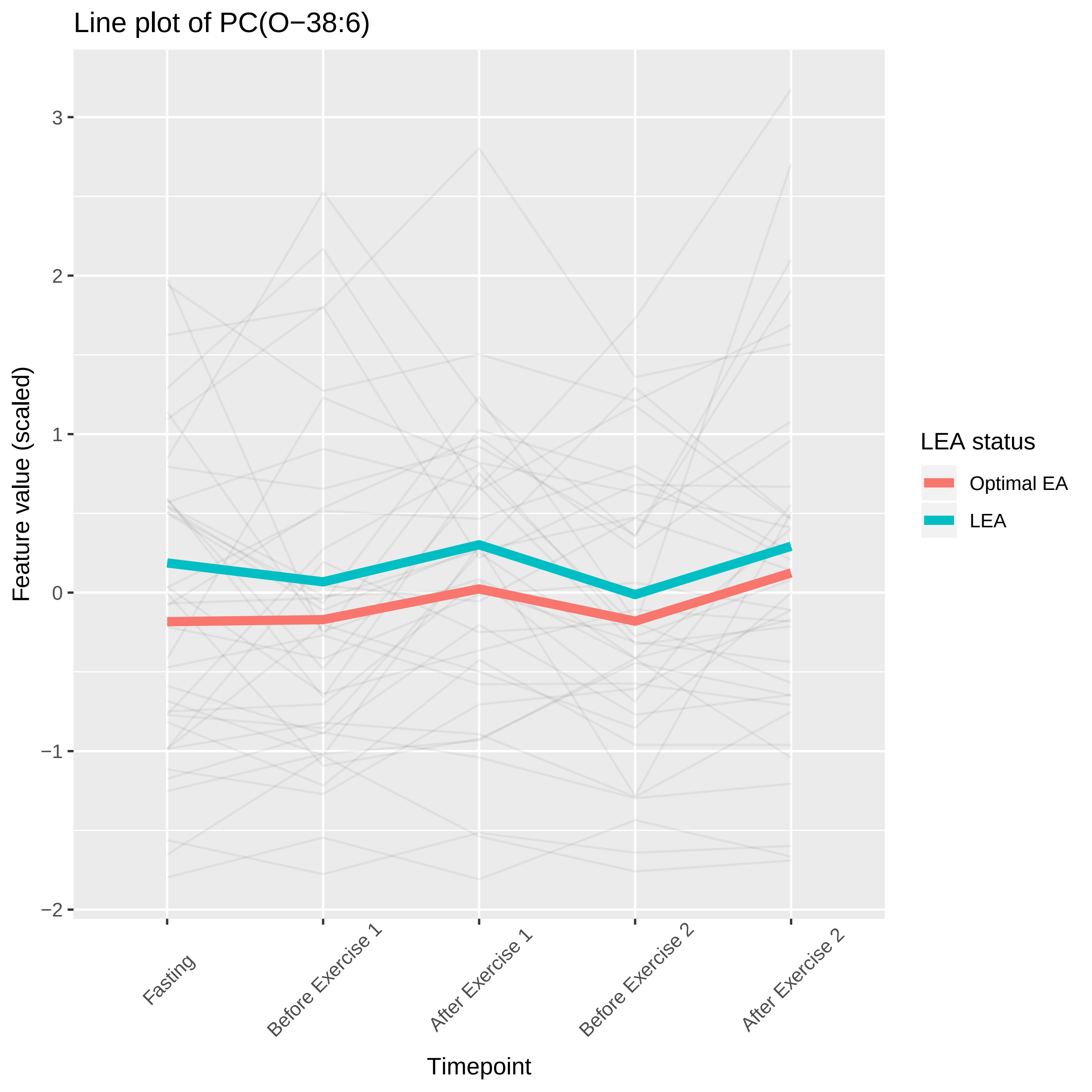

Line plot of PC(O−38:6)
Feature value (scaled)
3
2
1
0
−1
−2
Fasting
Before Exercise 1
After Exercise 1
Before Exercise 2
After Exercise 2
Timepoint
LEA status
Optimal EA
LEA

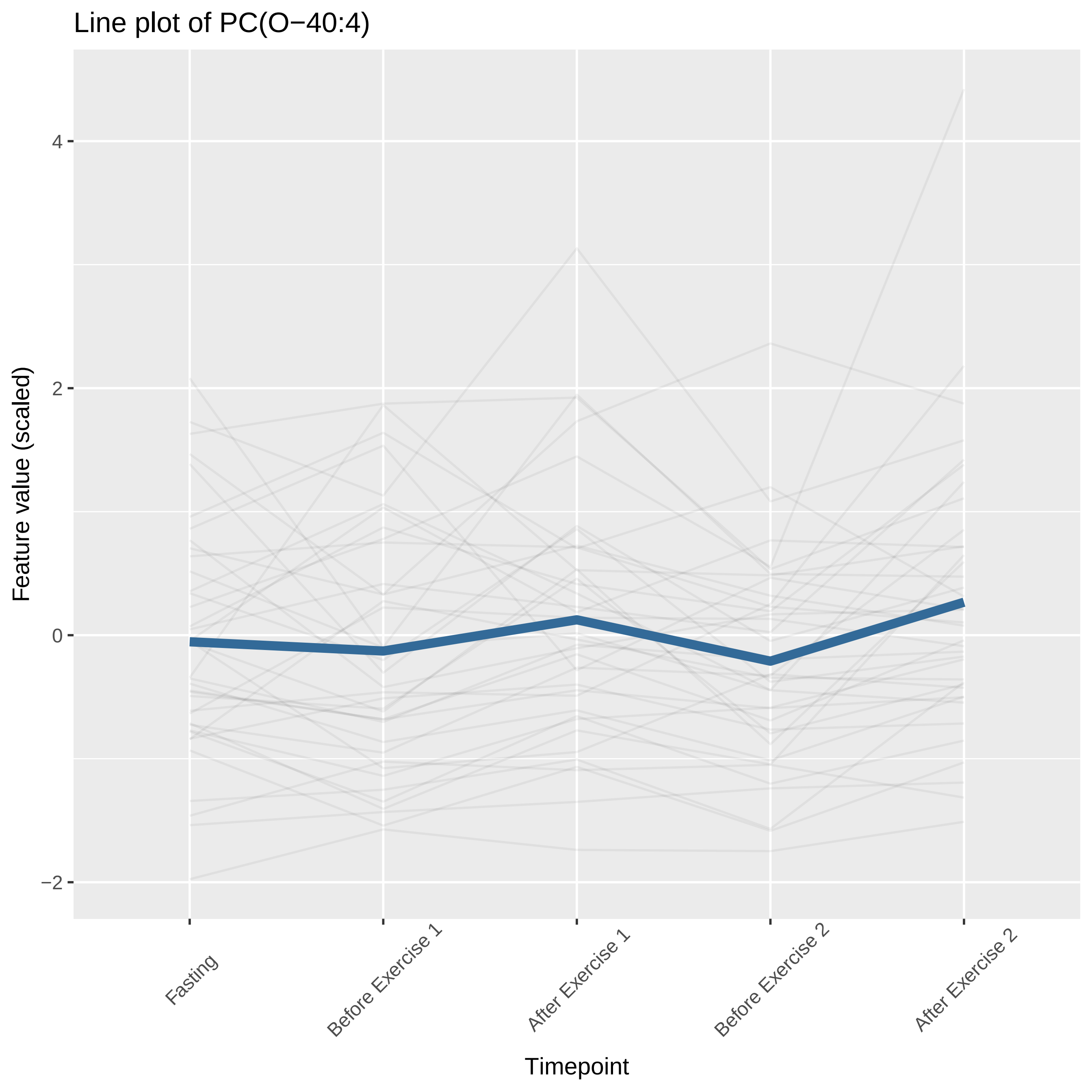

Line plot of PC(O−40:4)
Feature value (scaled)
4
2
0
−2
Fasting
Before Exercise 1
After Exercise 1
Before Exercise 2
After Exercise 2
Timepoint

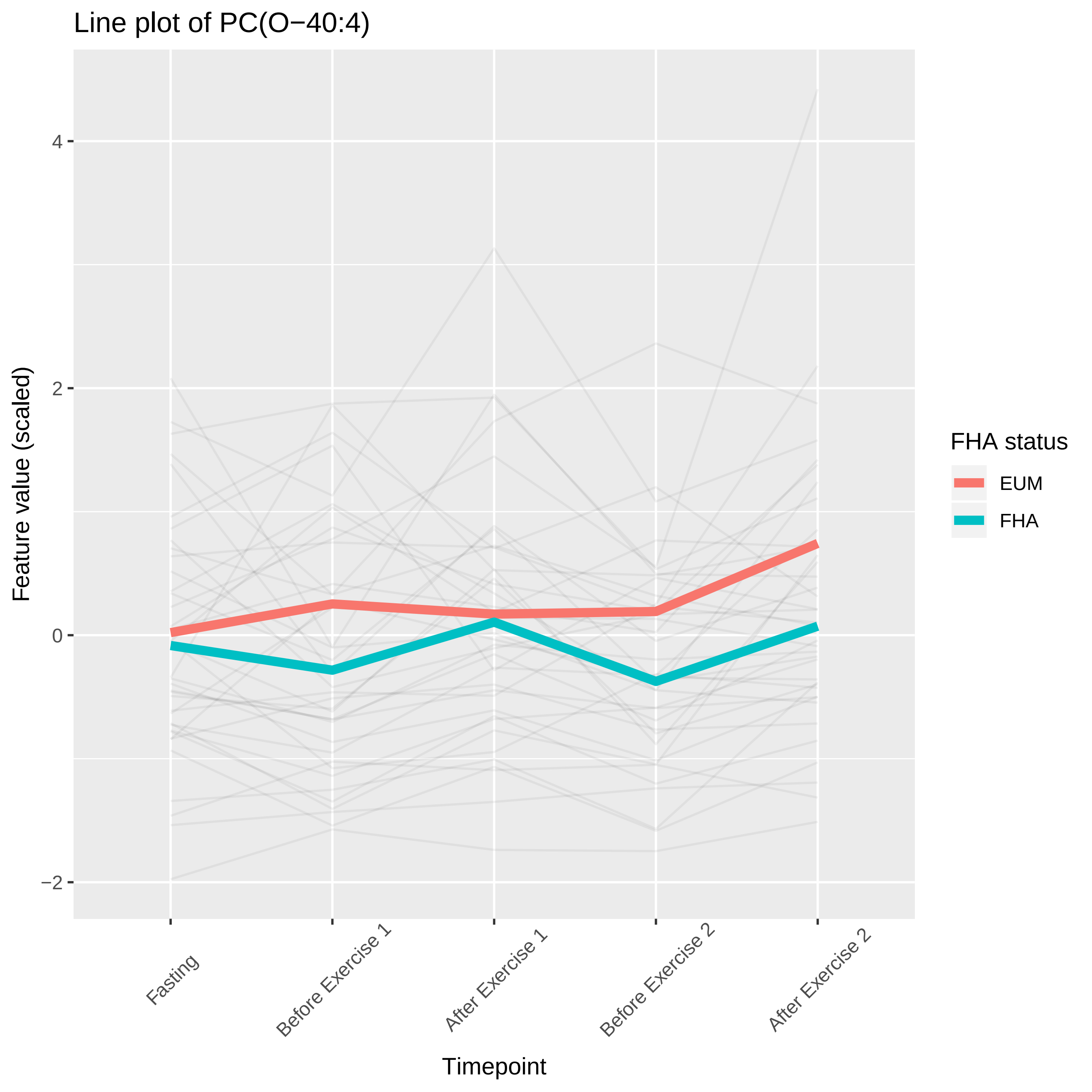
Line plot of PC(O−40:4)
Feature value (scaled)
4
2
0
−2
Fasting
Before Exercise 1
After Exercise 1
Before Exercise 2
After Exercise 2
Timepoint
FHA status
EUM
FHA

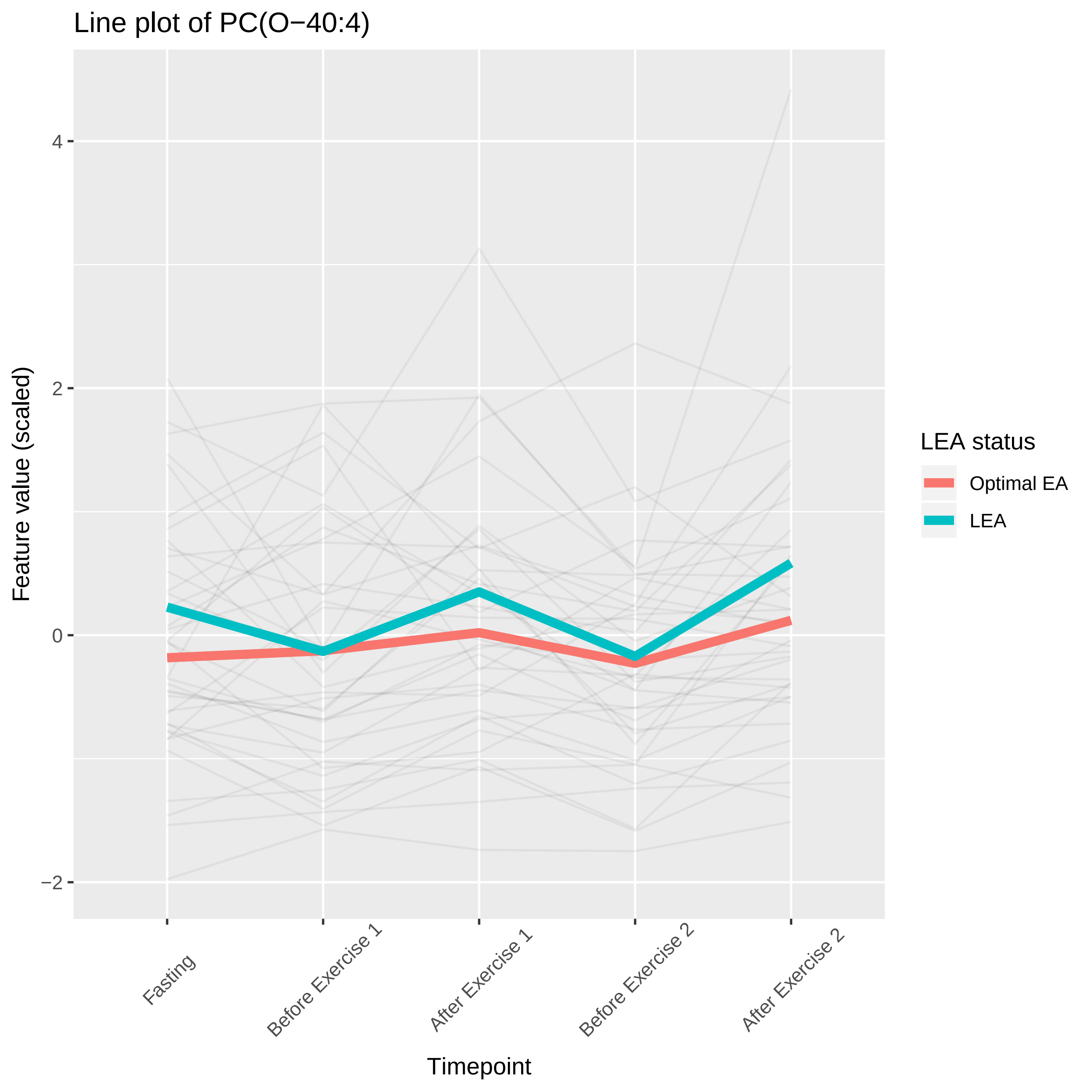
Line plot of PC(O−40:4)
Feature value (scaled)
4
2
0
−2
Fasting
Before Exercise 1
After Exercise 1
Before Exercise 2
After Exercise 2
Timepoint
LEA status
Optimal EA
LEA

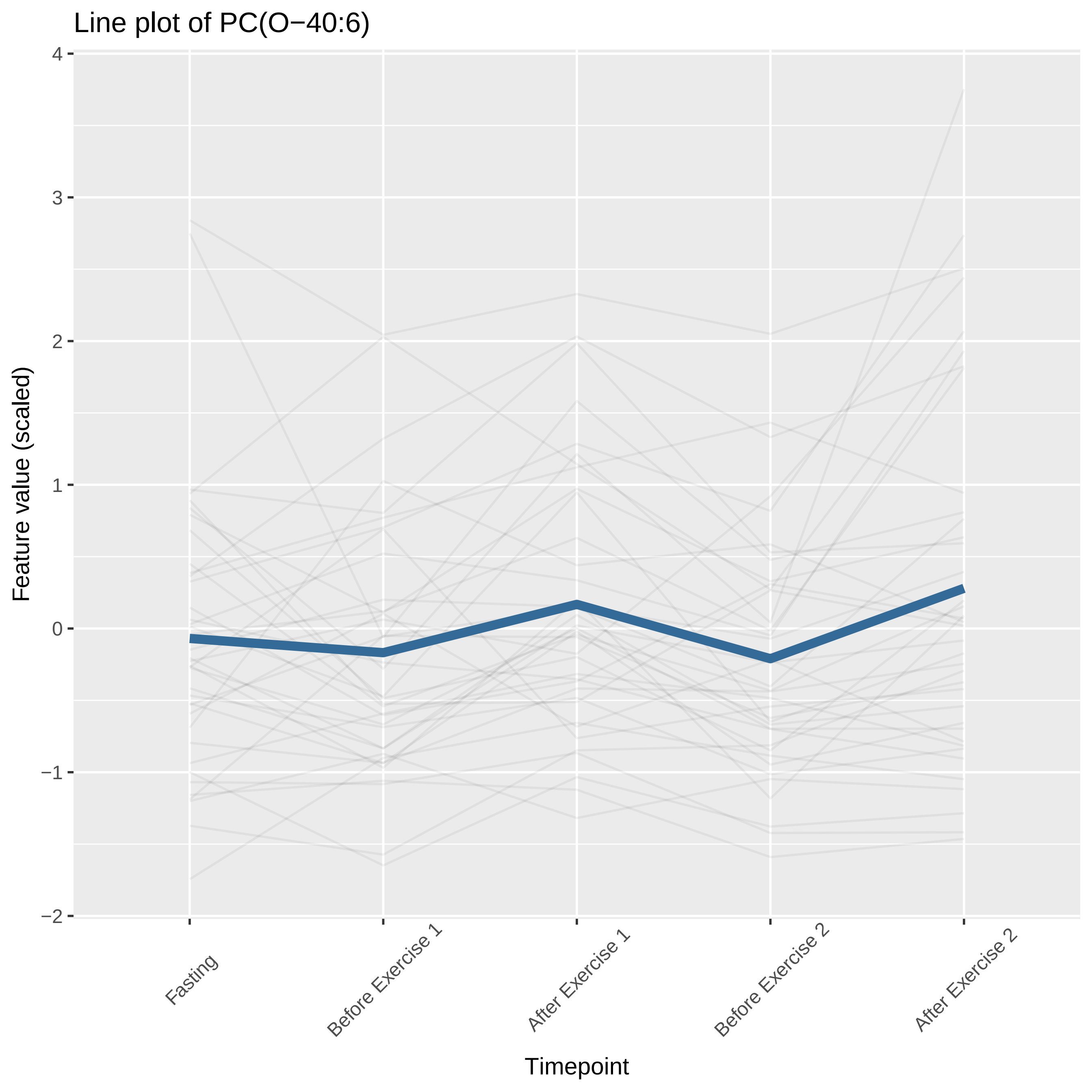

Line plot of PC(O−40:6)
4
3
2
1
0
−1
−2
Feature value (scaled)
Fasting
Before Exercise 1
After Exercise 1
Before Exercise 2
After Exercise 2
Timepoint

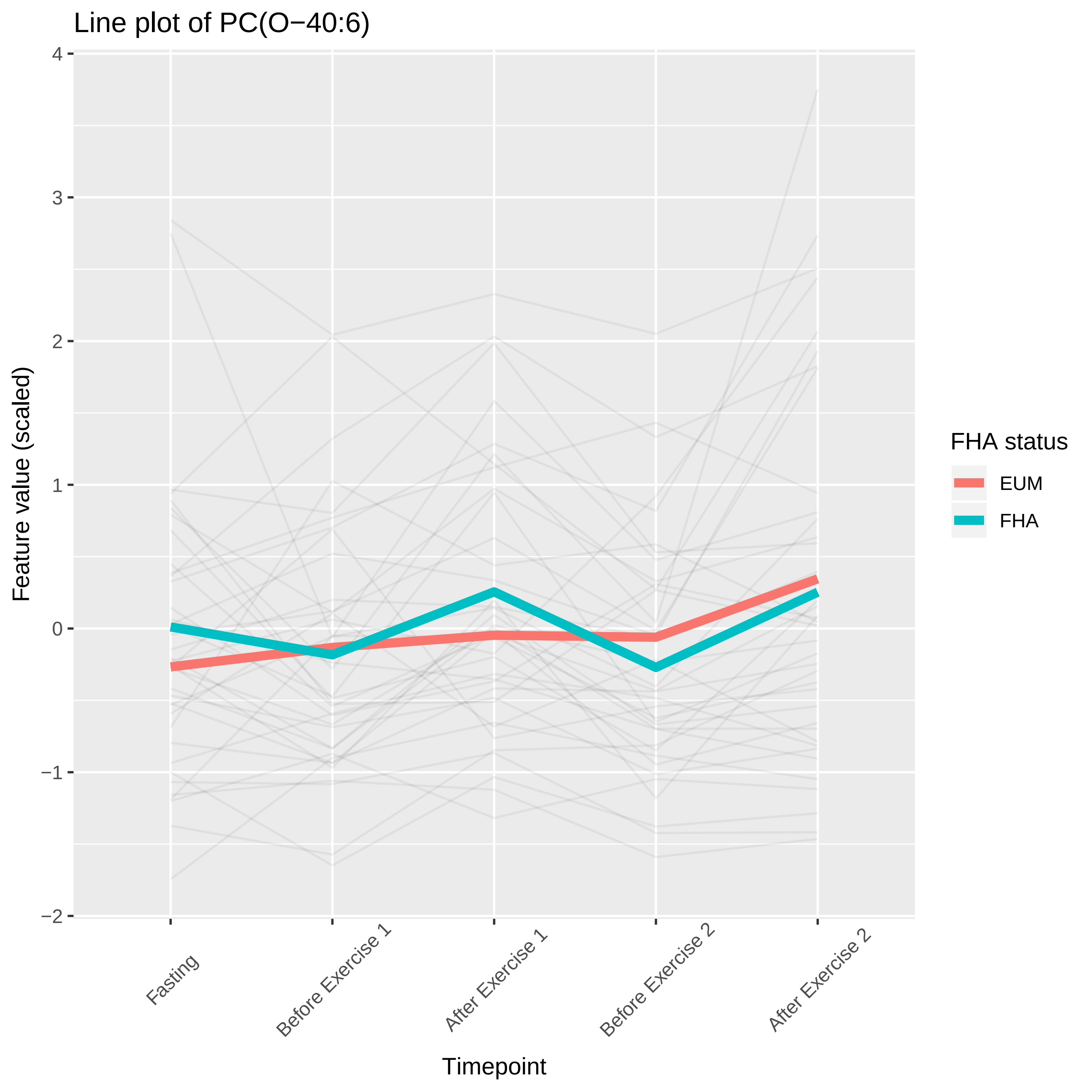
Line plot of PC(O−40:6)
Feature value (scaled)
4
3
2
1
0
−1
−2
Fasting
Before Exercise 1
After Exercise 1
Before Exercise 2
After Exercise 2
Timepoint
FHA status
EUM
FHA

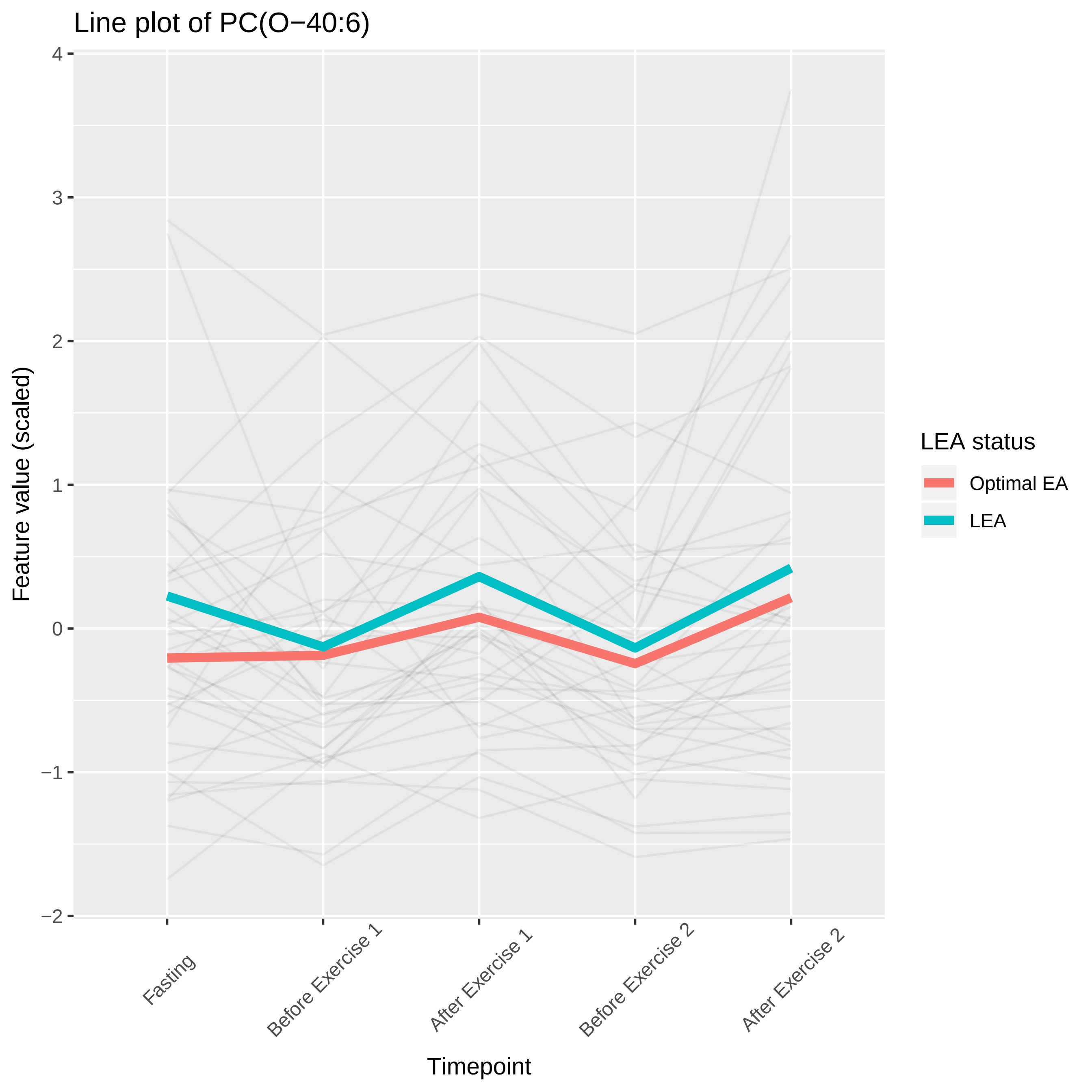

Line plot of PC(O−40:6)
Feature value (scaled)
4
3
2
1
0
−1
−2
Fasting
Before Exercise 1
After Exercise 1
Before Exercise 2
After Exercise 2
Timepoint
LEA status
Optimal EA
LEA

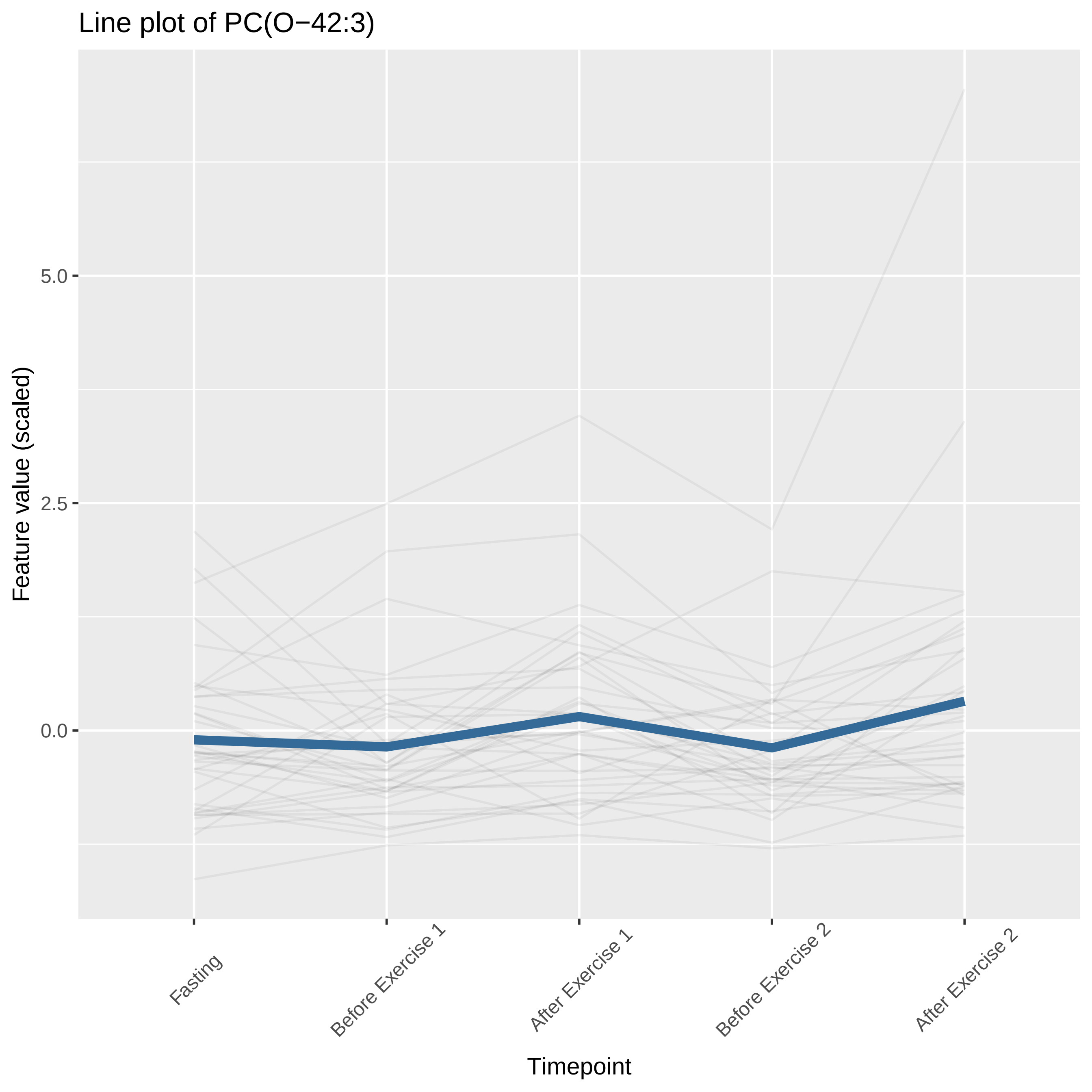

Line plot of PC(O−42:3)
Feature value (scaled)
5.0
2.5
0.0
Fasting
Before Exercise 1
After Exercise 1
Before Exercise 2
After Exercise 2
Timepoint

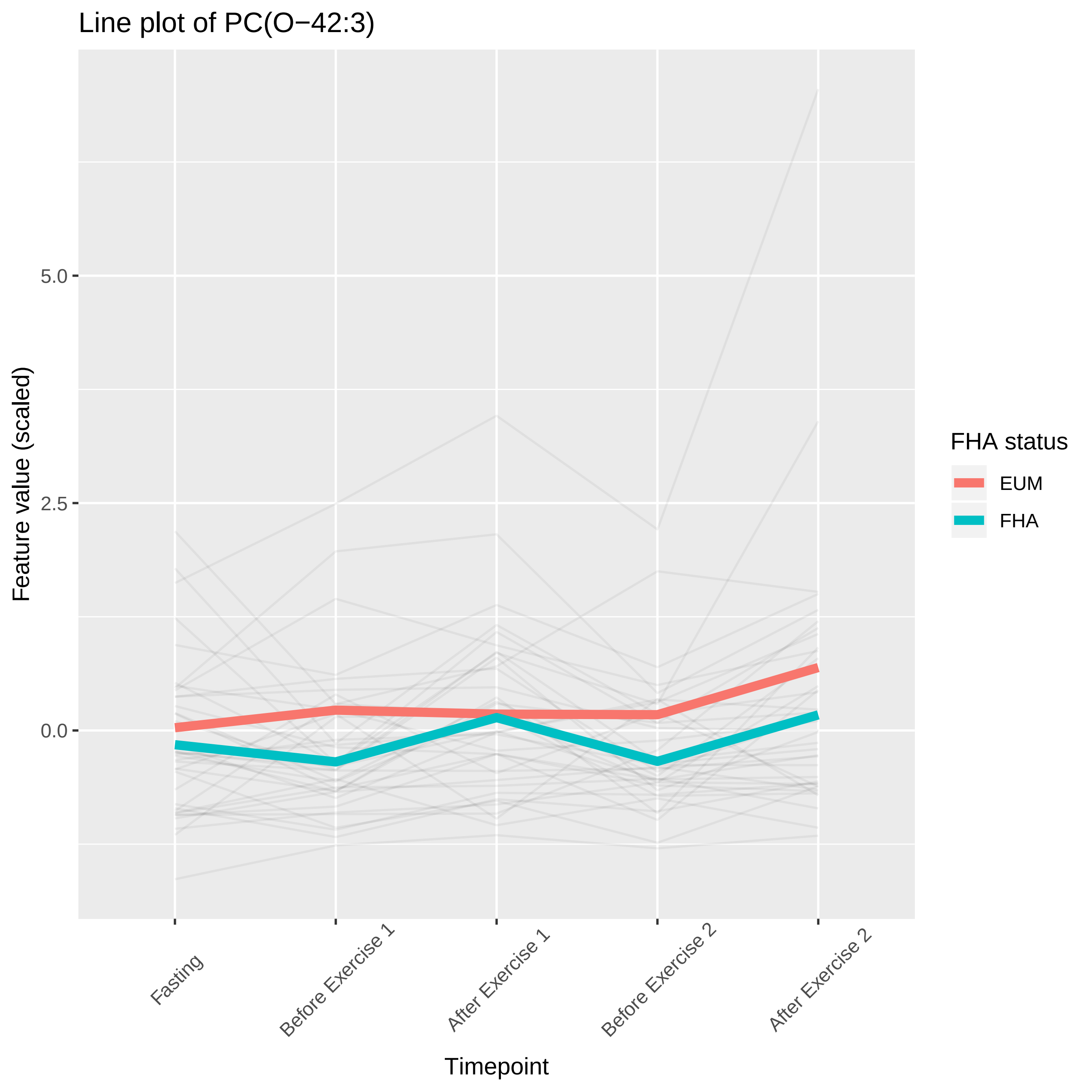

Line plot of PC(O−42:3)
Feature value (scaled)
5.0
2.5
0.0
Fasting
Before Exercise 1
After Exercise 1
Before Exercise 2
After Exercise 2
Timepoint
FHA status
EUM
FHA

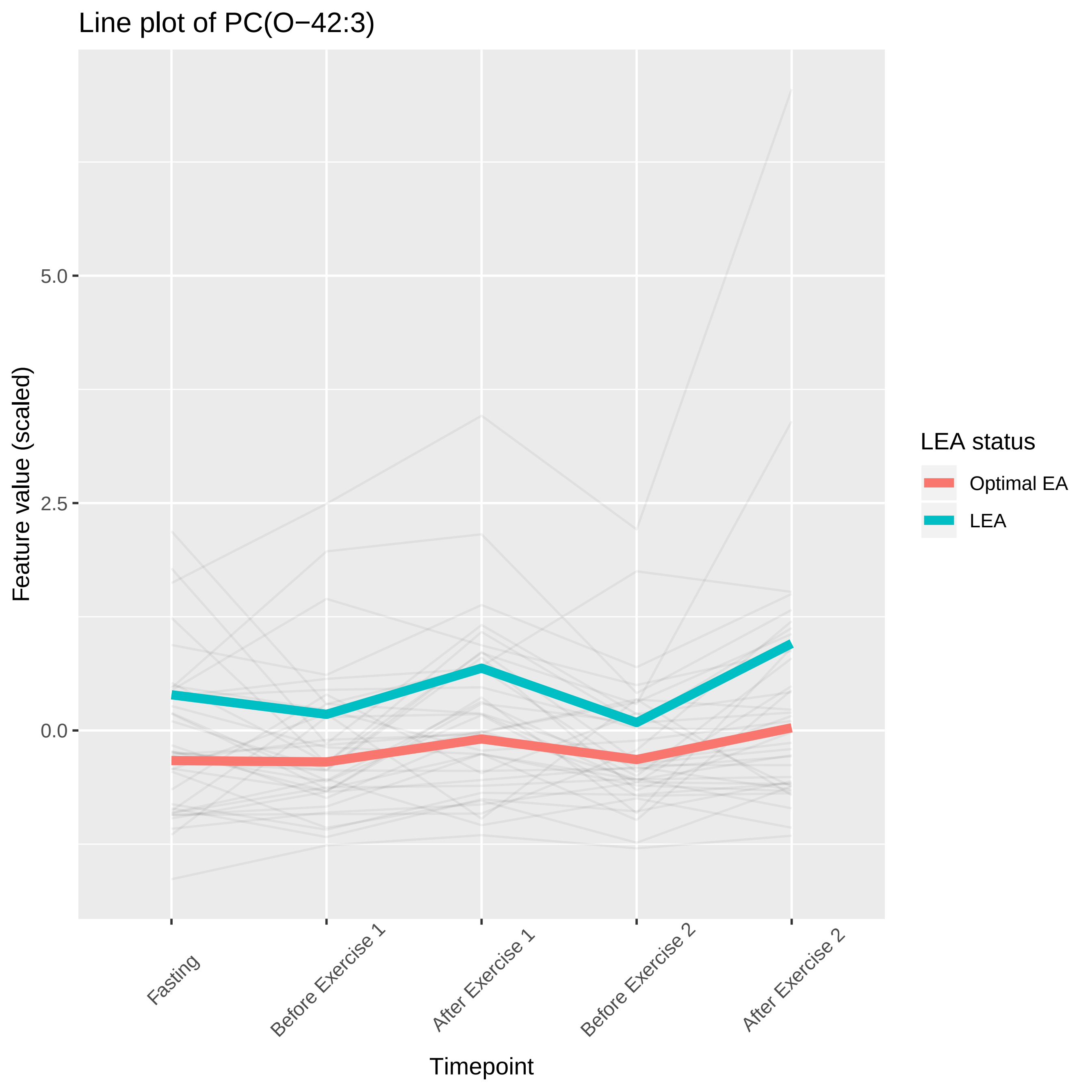

Line plot of PC(O−42:3)
Feature value (scaled)
5.0
2.5
0.0
Fasting
Before Exercise 1
After Exercise 1
Before Exercise 2
After Exercise 2
Timepoint
LEA status
Optimal EA
LEA

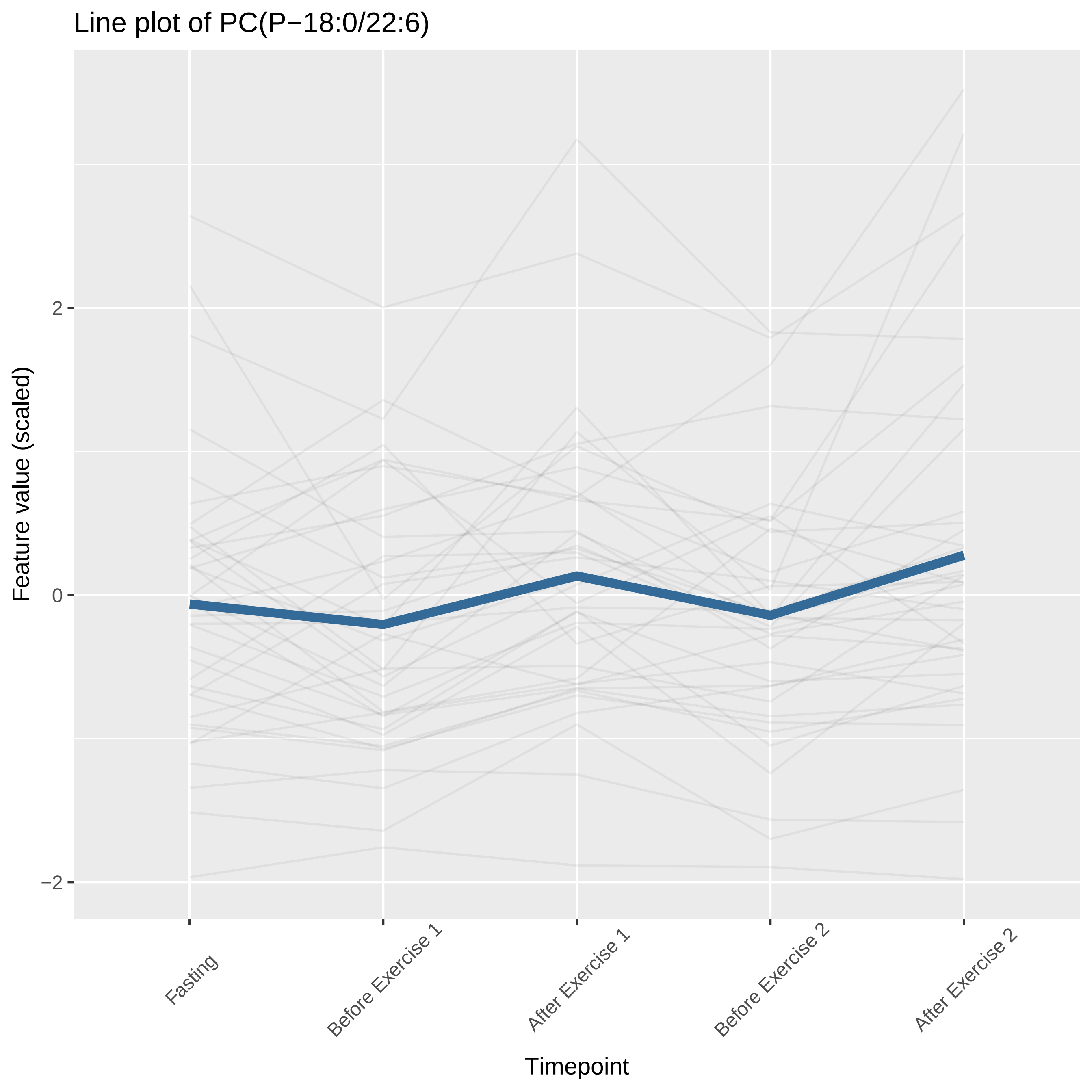
Line plot of PC(P−18:0/22:6)
Feature value (scaled)
2
0
−2
Fasting
Before Exercise 1
After Exercise 1
Before Exercise 2
After Exercise 2
Timepoint

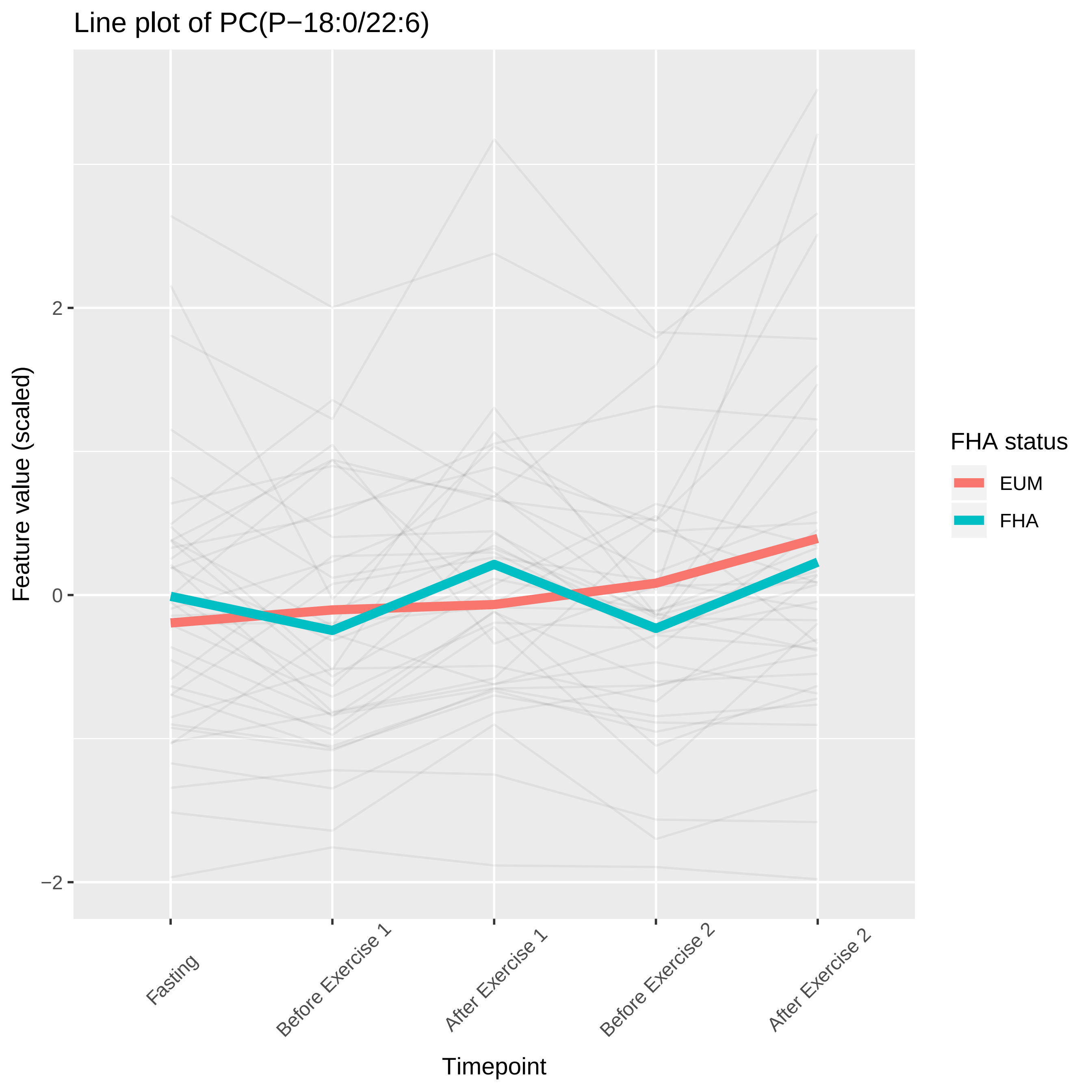

Line plot of PC(P−18:0/22:6)
Feature value (scaled)
2
0
−2
Fasting
Before Exercise 1
After Exercise 1
Before Exercise 2
After Exercise 2
Timepoint
FHA status
EUM
FHA

Line plot of PC(P−18:0/22:6)

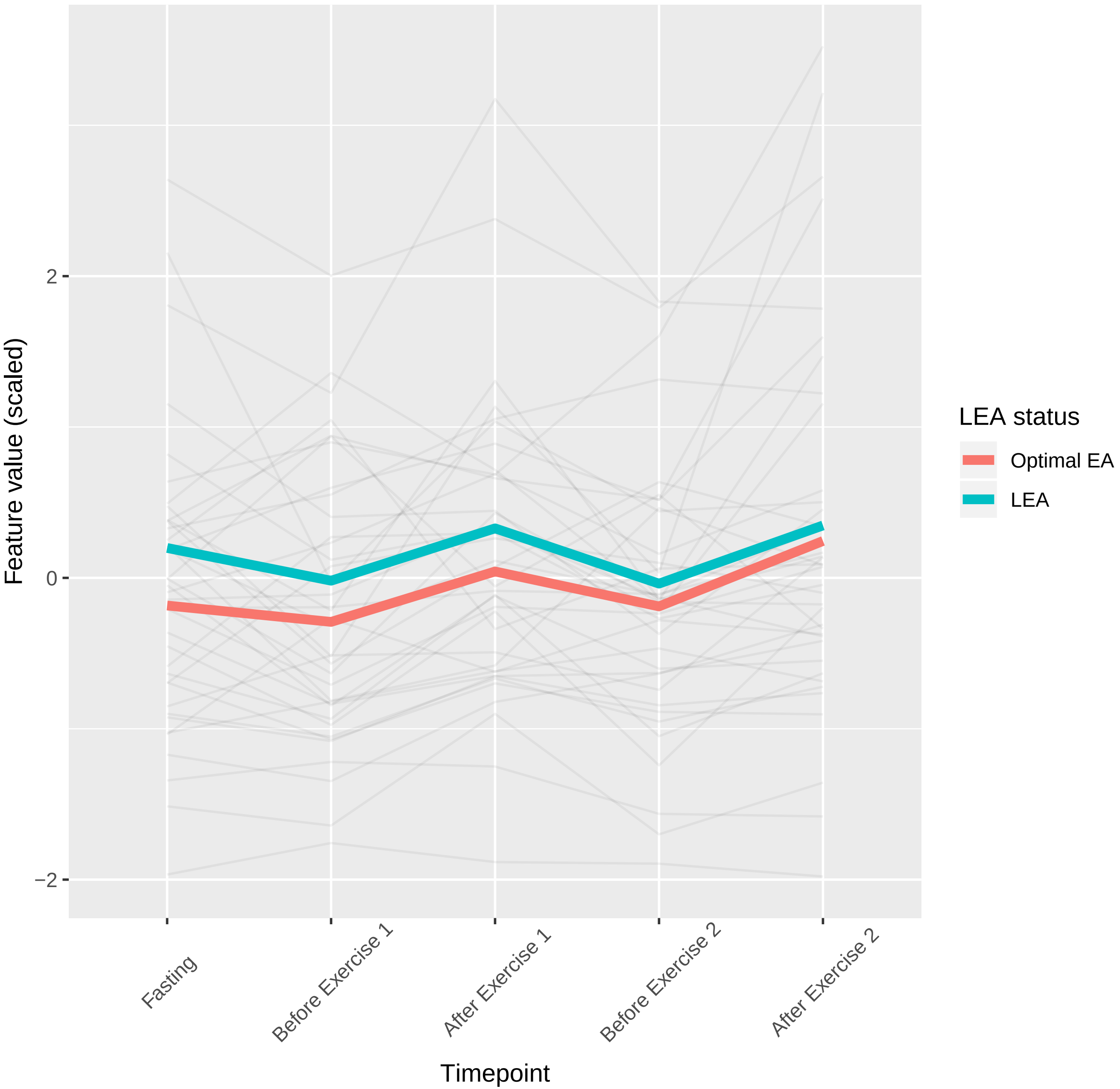

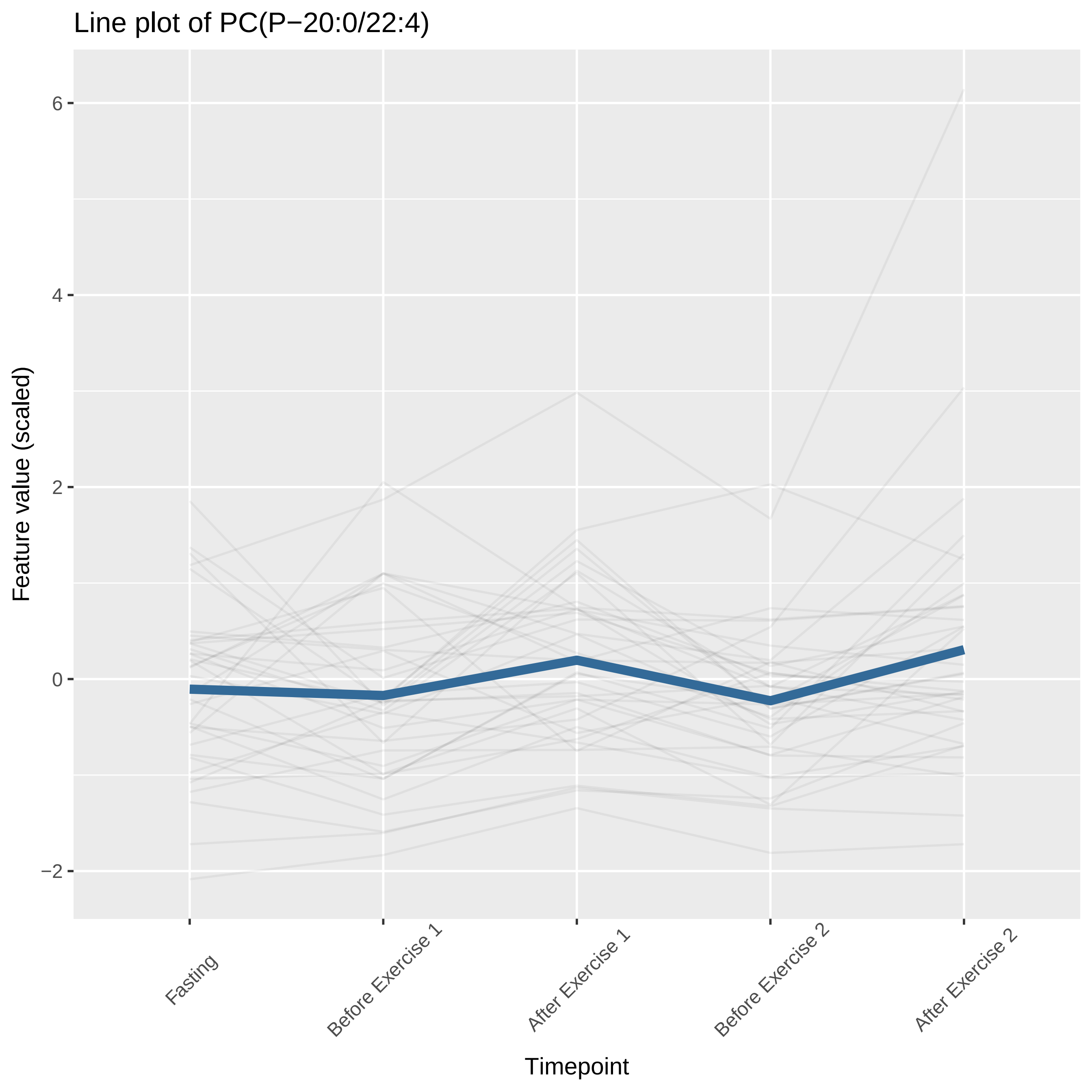

Line plot of PC(P−20:0/22:4)
Feature value (scaled)
6
4
2
0
−2
Fasting
Before Exercise 1
After Exercise 1
Before Exercise 2
After Exercise 2
Timepoint

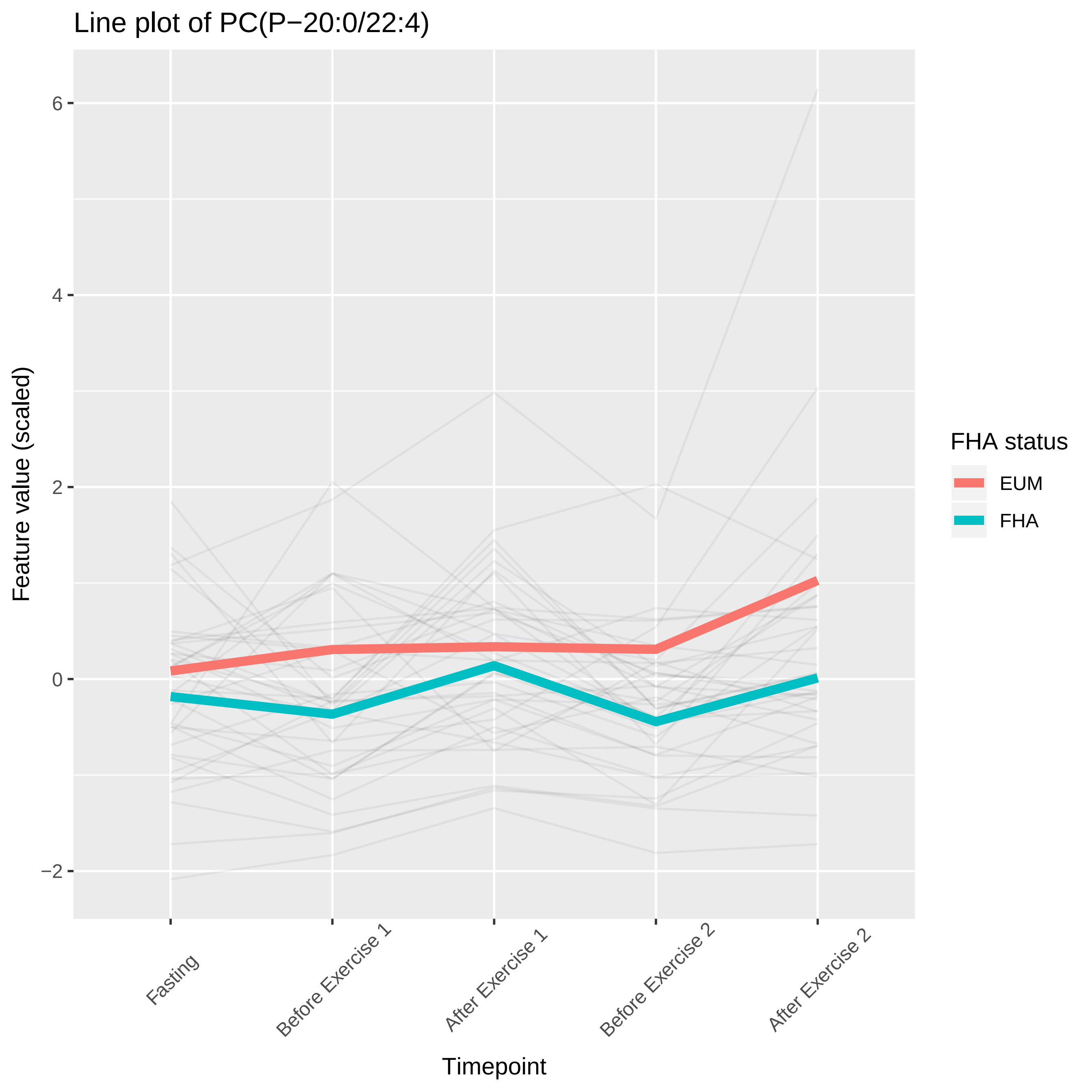

Line plot of PC(P−20:0/22:4)
Feature value (scaled)
6
4
2
0
−2
Fasting
Before Exercise 1
After Exercise 1
Before Exercise 2
After Exercise 2
Timepoint
FHA status
EUM
FHA

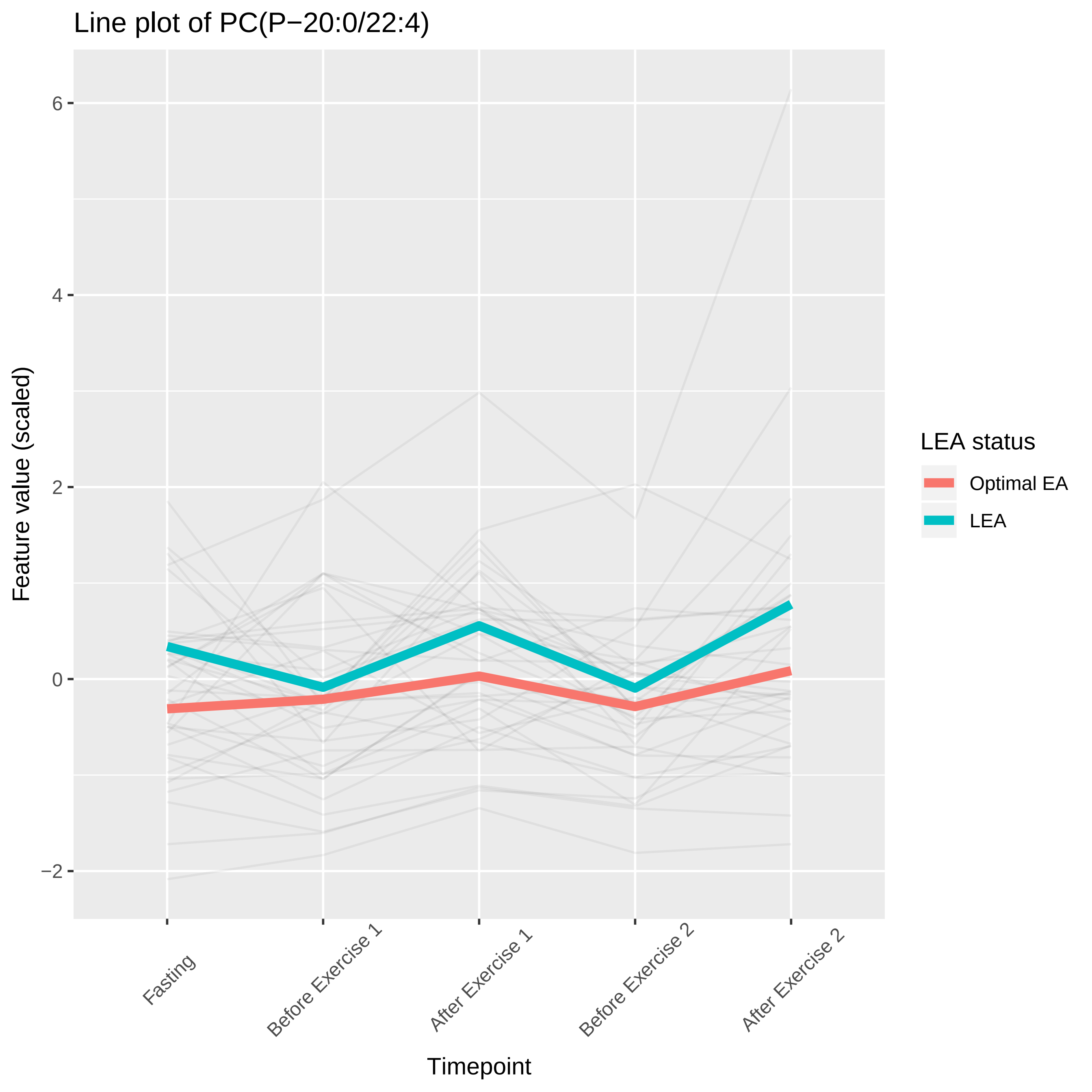
Line plot of PC(P−20:0/22:4)
Feature value (scaled)
6
4
2
0
−2
Fasting
Before Exercise 1
After Exercise 1
Before Exercise 2
After Exercise 2
Timepoint
LEA status
Optimal EA
LEA

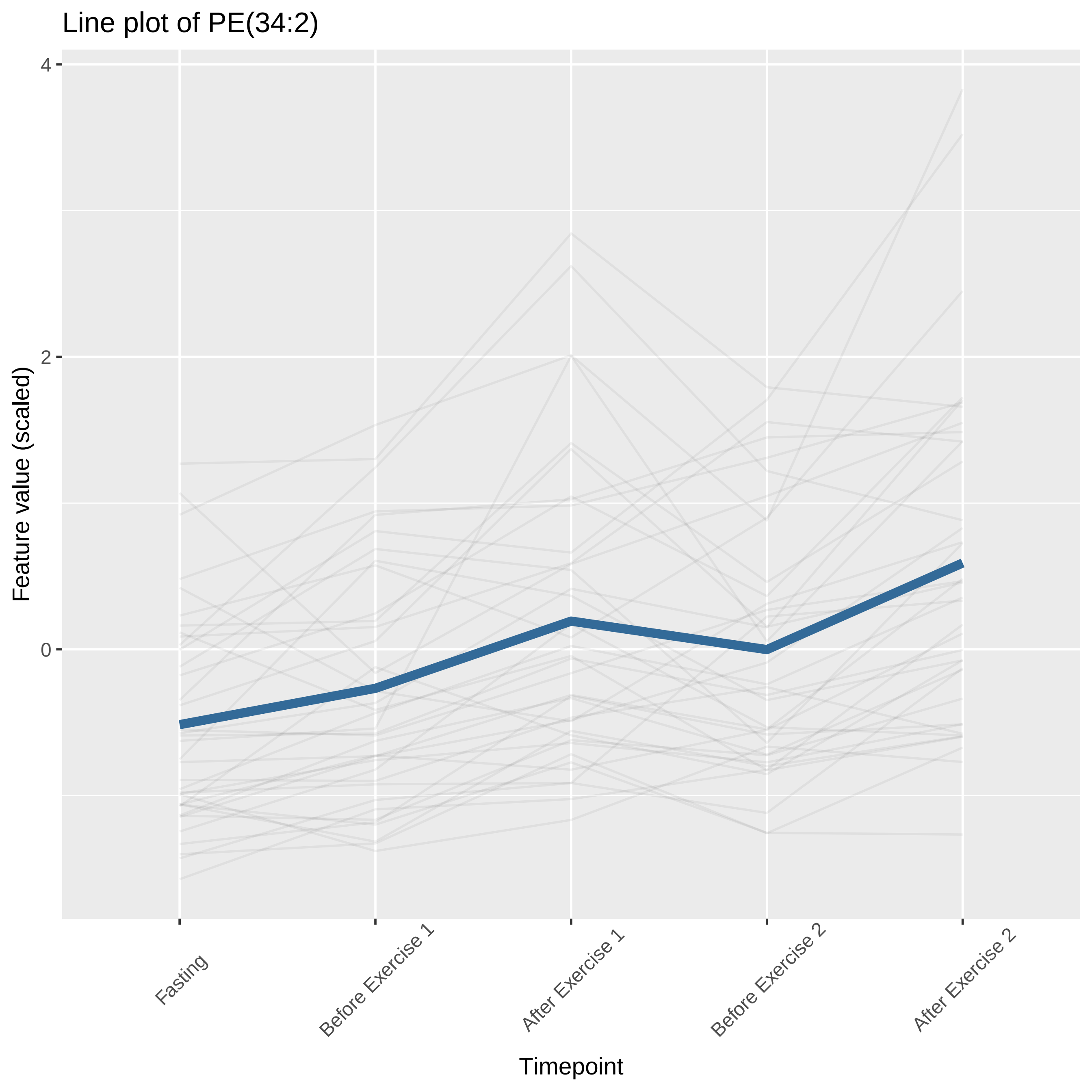

Line plot of PE(34:2)
Feature value (scaled)
4
2
0
Fasting
Before Exercise 1
After Exercise 1
Before Exercise 2
After Exercise 2
Timepoint

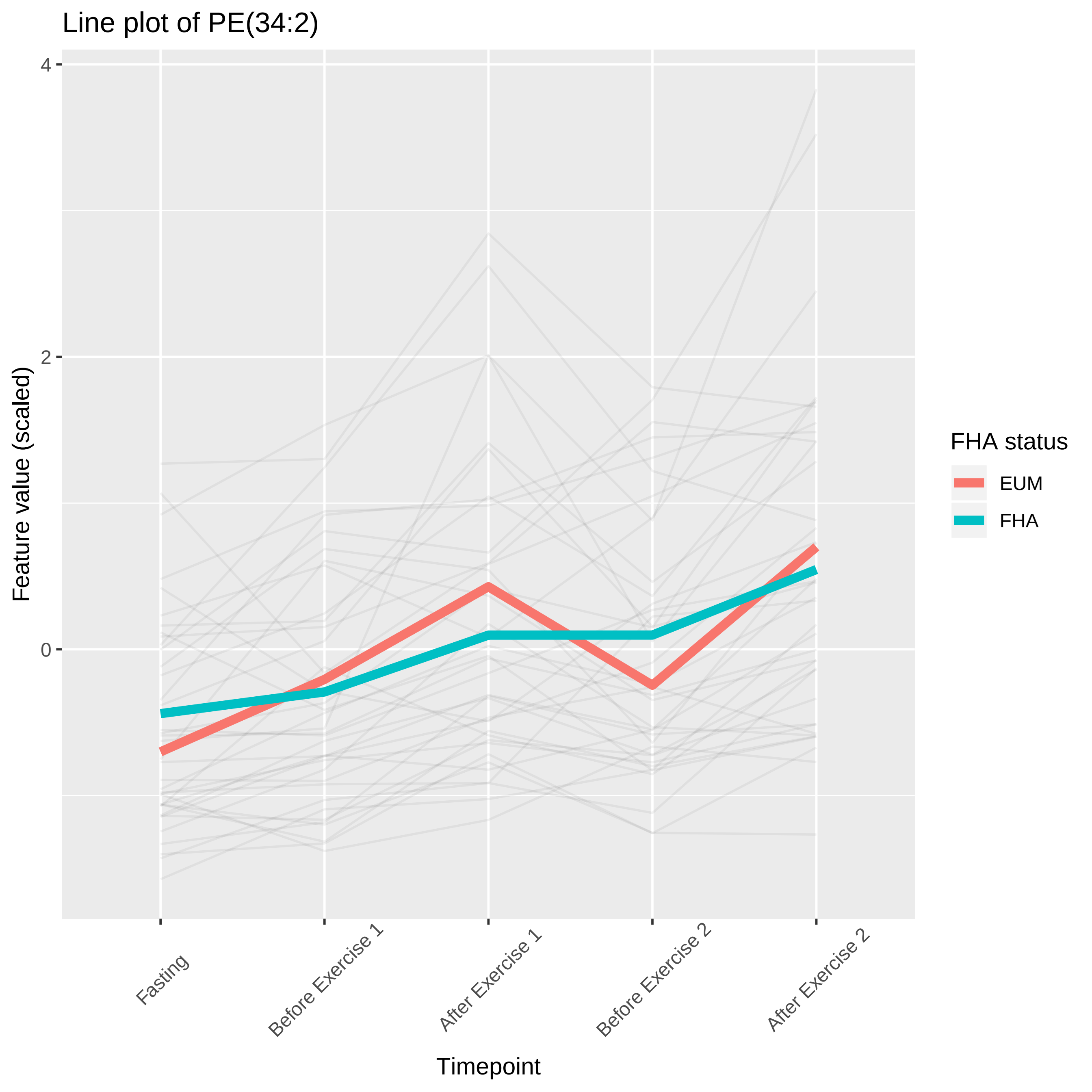
Line plot of PE(34:2)
Feature value (scaled)
4
2
0
Fasting
Before Exercise 1
After Exercise 1
Before Exercise 2
After Exercise 2
Timepoint
FHA status
EUM
FHA

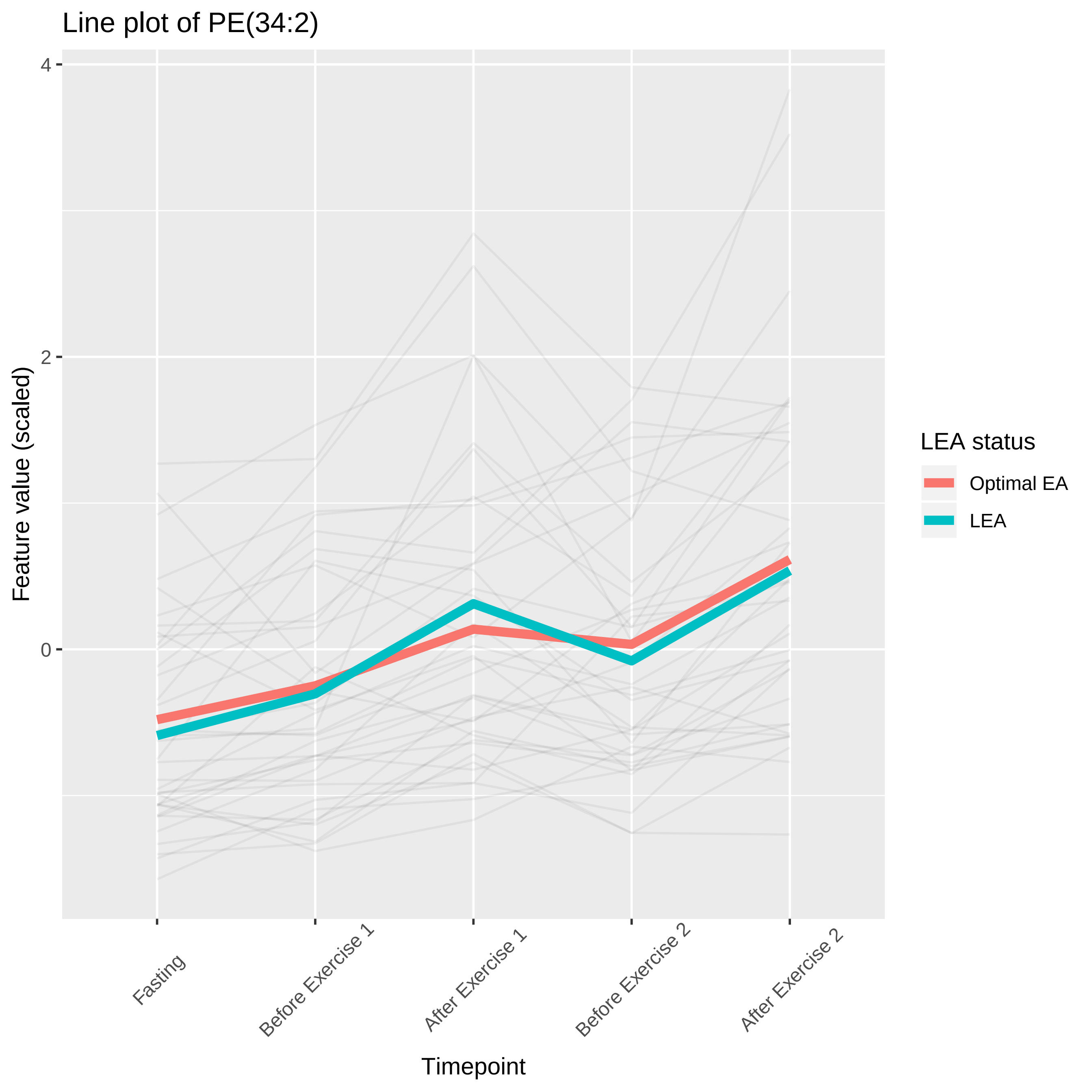

Line plot of PE(34:2)
Feature value (scaled)
4
2
0
Fasting
Before Exercise 1
After Exercise 1
Before Exercise 2
After Exercise 2
Timepoint
LEA status
Optimal EA
LEA

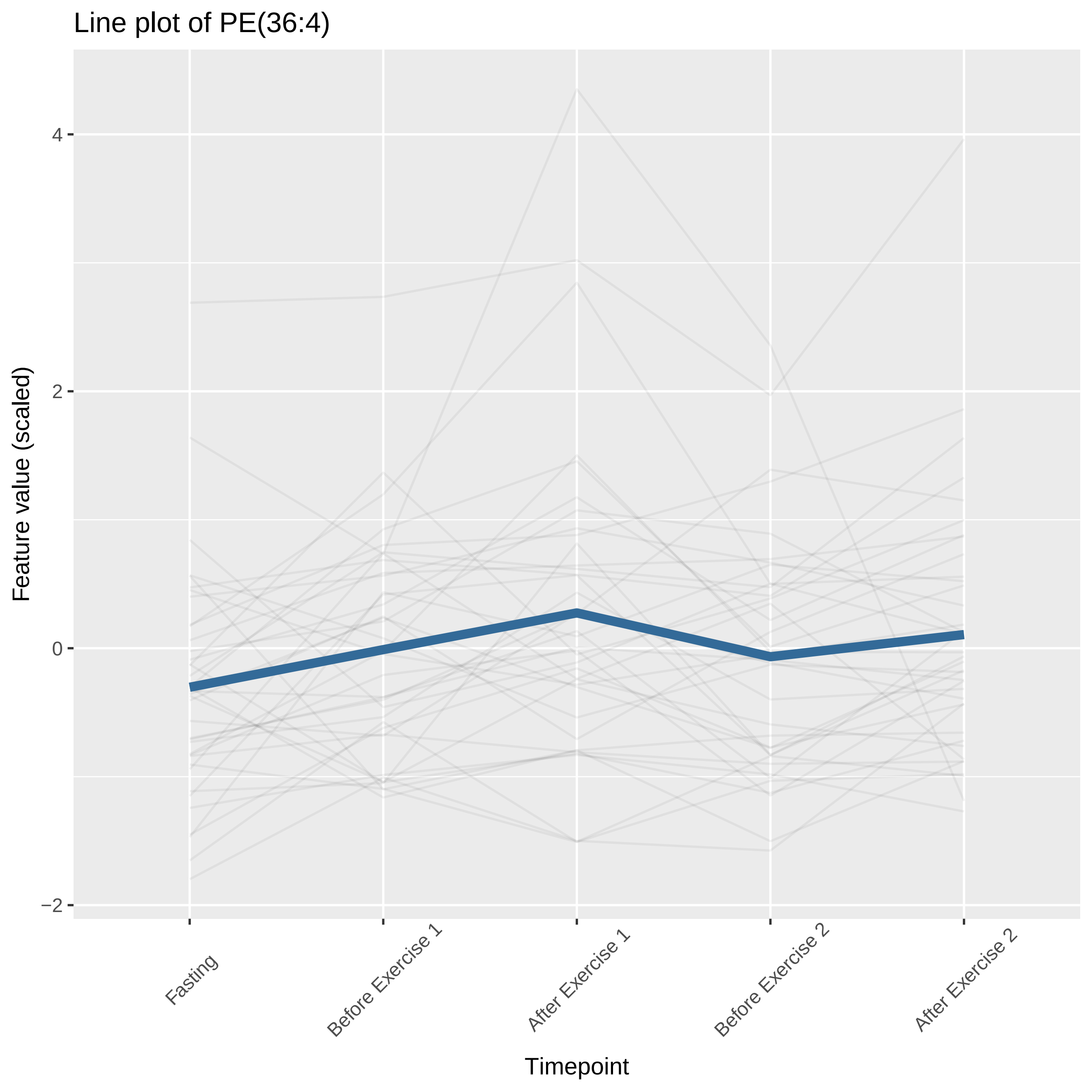
Line plot of PE(36:4)
Feature value (scaled)
4
2
0
−2
Fasting
Before Exercise 1
After Exercise 1
Before Exercise 2
After Exercise 2
Timepoint

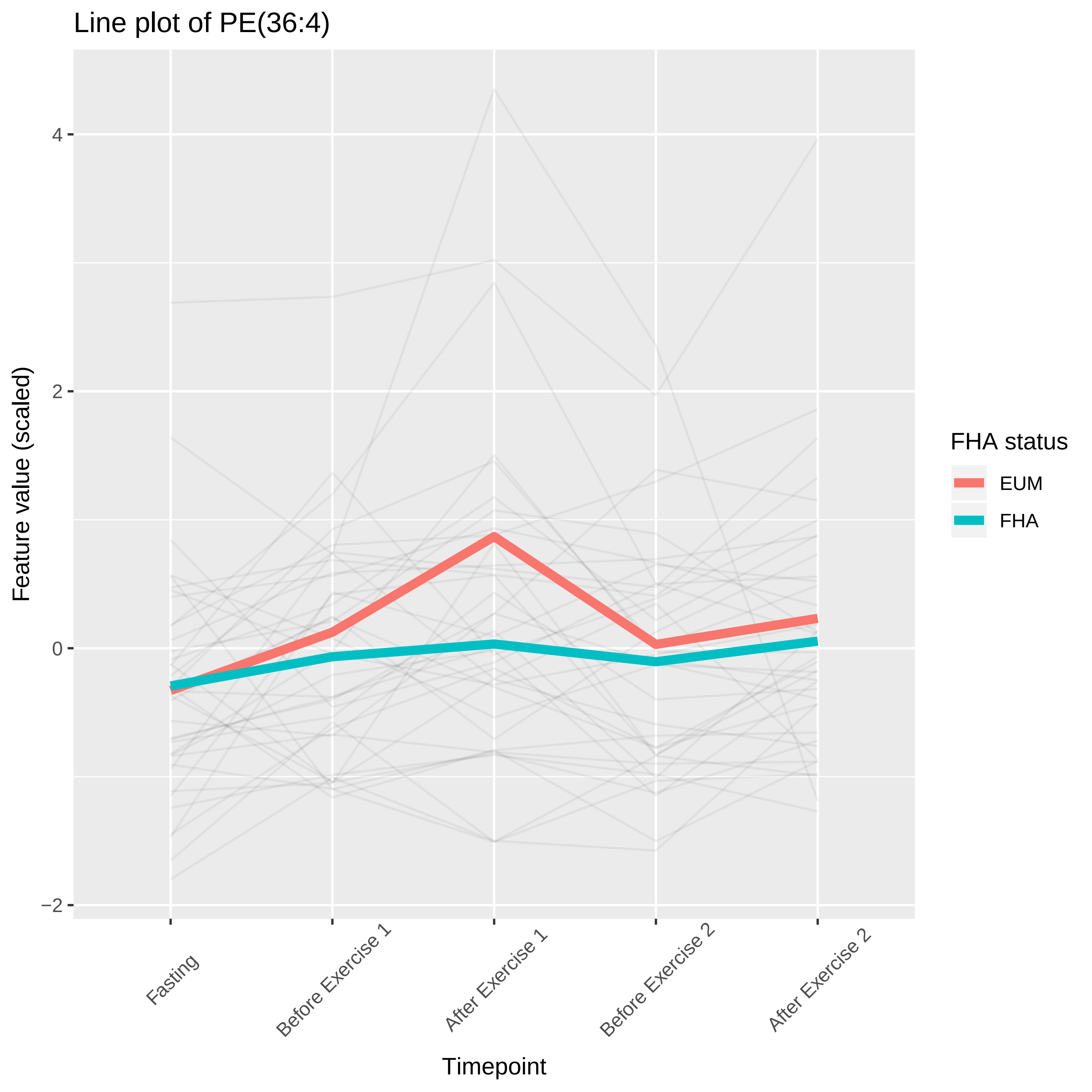

Line plot of PE(36:4)
Feature value (scaled)
4
2
0
−2
Fasting
Before Exercise 1
After Exercise 1
Before Exercise 2
After Exercise 2
Timepoint
FHA status
EUM
FHA

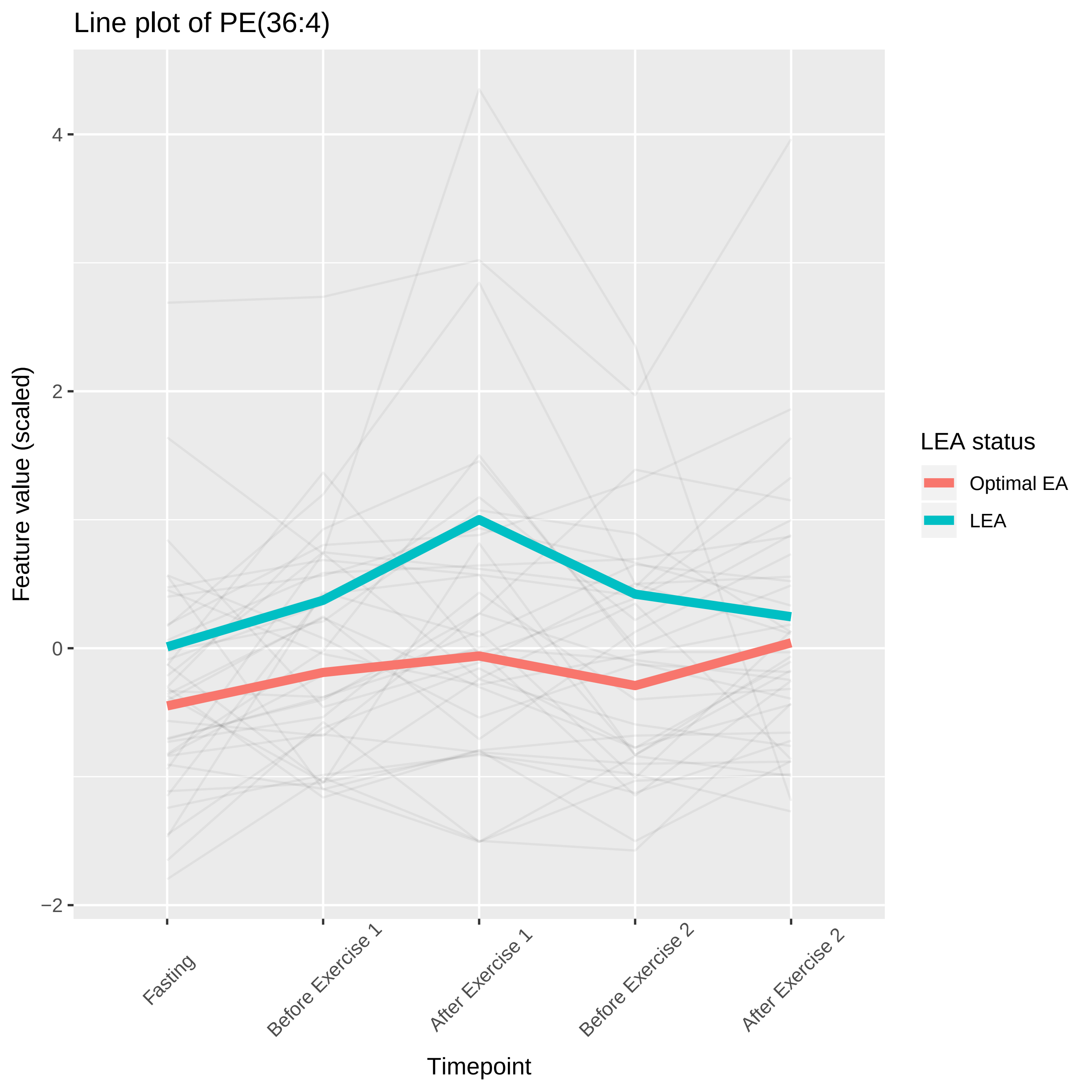

Line plot of PE(36:4)
Feature value (scaled)
4
2
0
−2
Fasting
Before Exercise 1
After Exercise 1
Before Exercise 2
After Exercise 2
Timepoint
LEA status
Optimal EA
LEA

Line plot of PE(38:4)

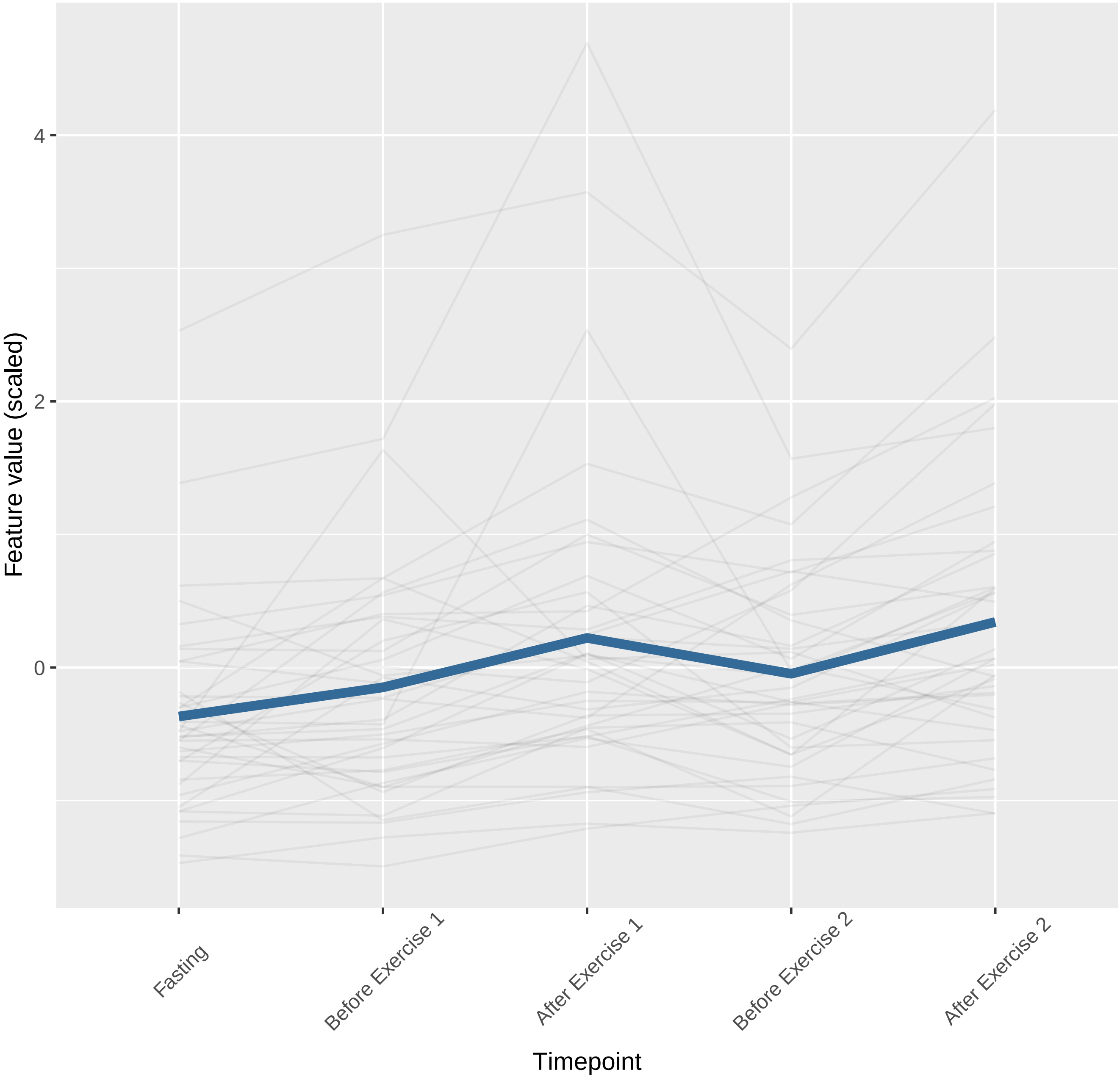

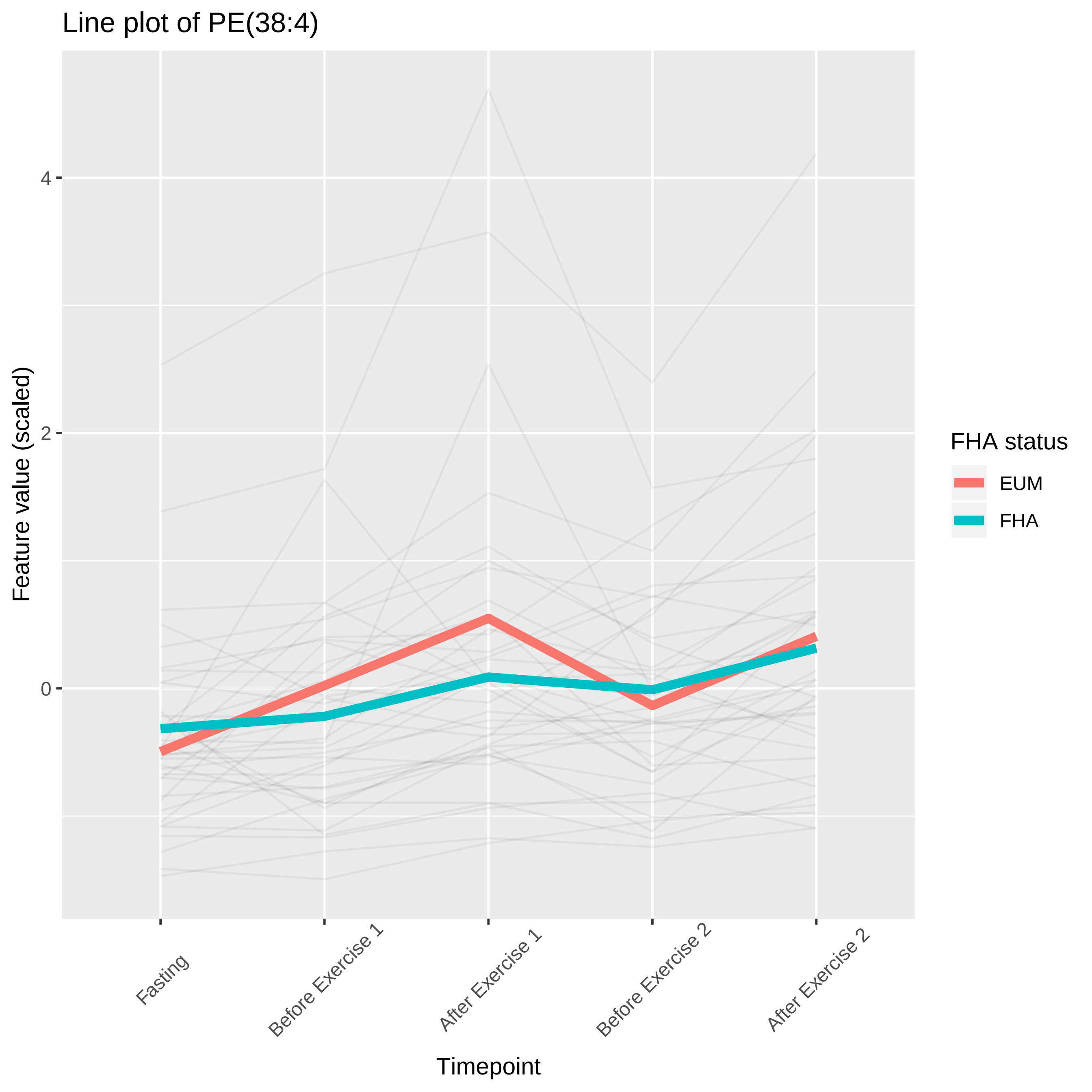

Line plot of PE(38:4)
Feature value (scaled)
4
2
0
Fasting
Before Exercise 1
After Exercise 1
Before Exercise 2
After Exercise 2
Timepoint
FHA status
EUM
FHA

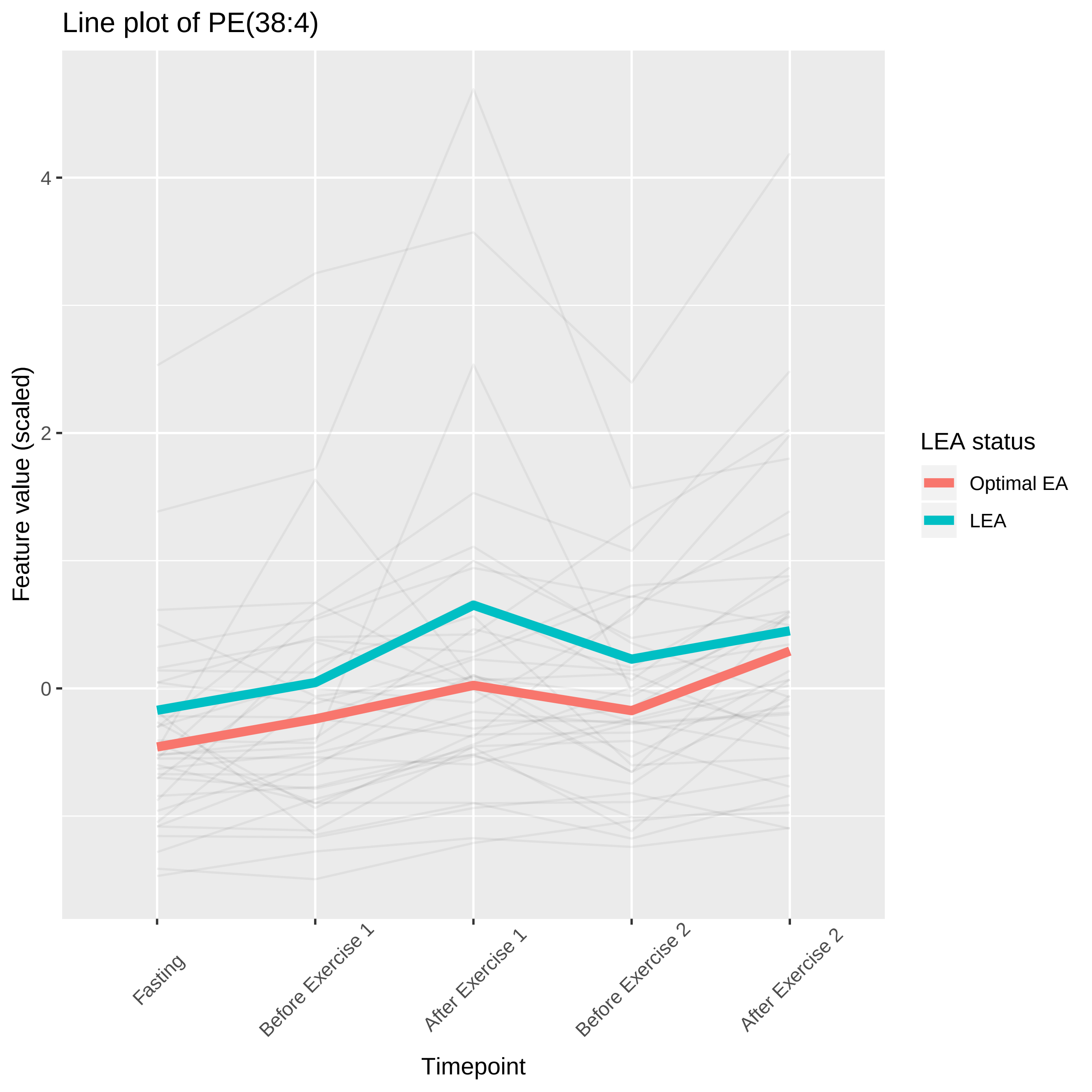
Line plot of PE(38:4)
Feature value (scaled)
4
2
0
Fasting
Before Exercise 1
After Exercise 1
Before Exercise 2
After Exercise 2
Timepoint
LEA status
Optimal EA
LEA

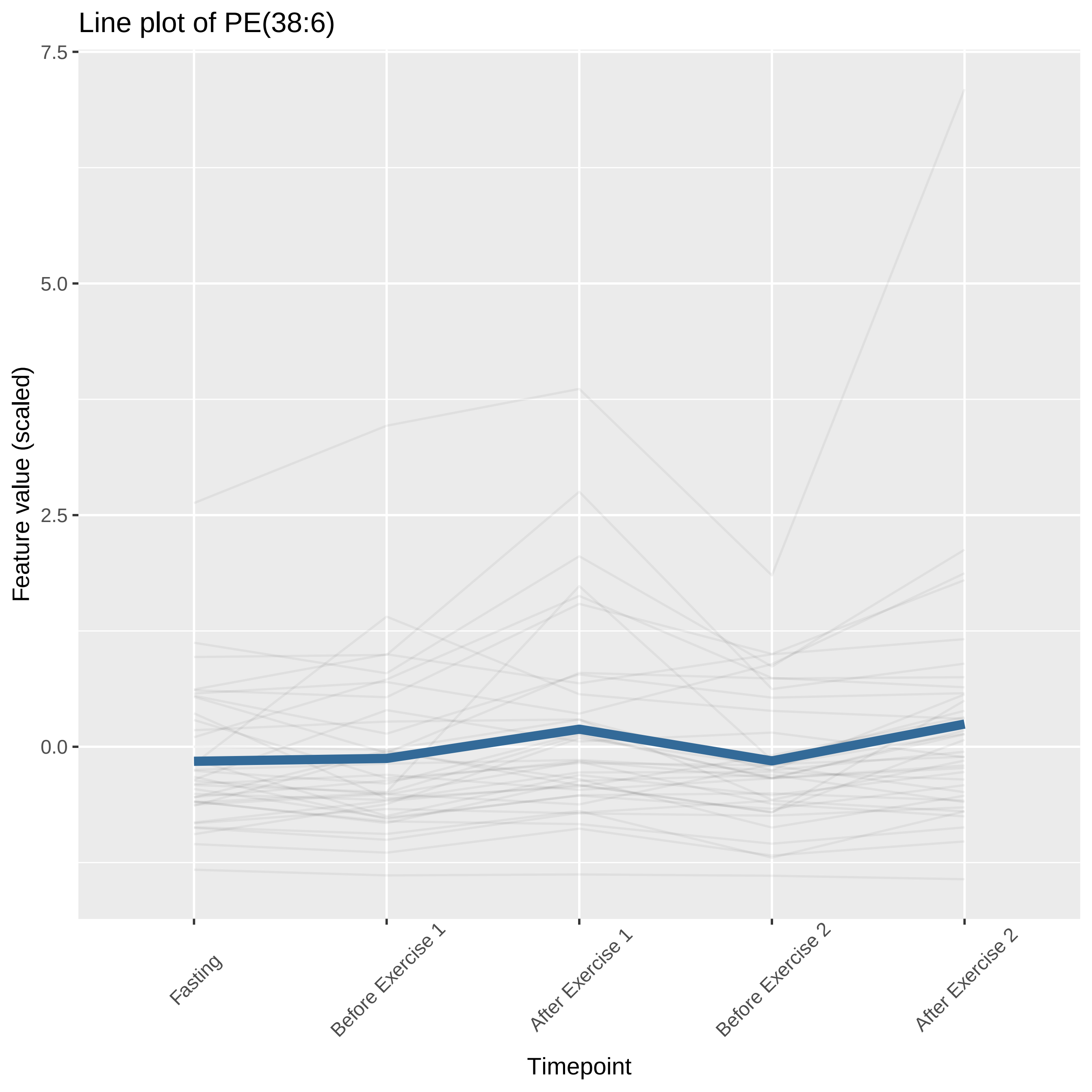

Line plot of PE(38:6)
7.5
5.0
2.5
0.0
Feature value (scaled)
Fasting
Before Exercise 1
After Exercise 1
Before Exercise 2
After Exercise 2
Timepoint

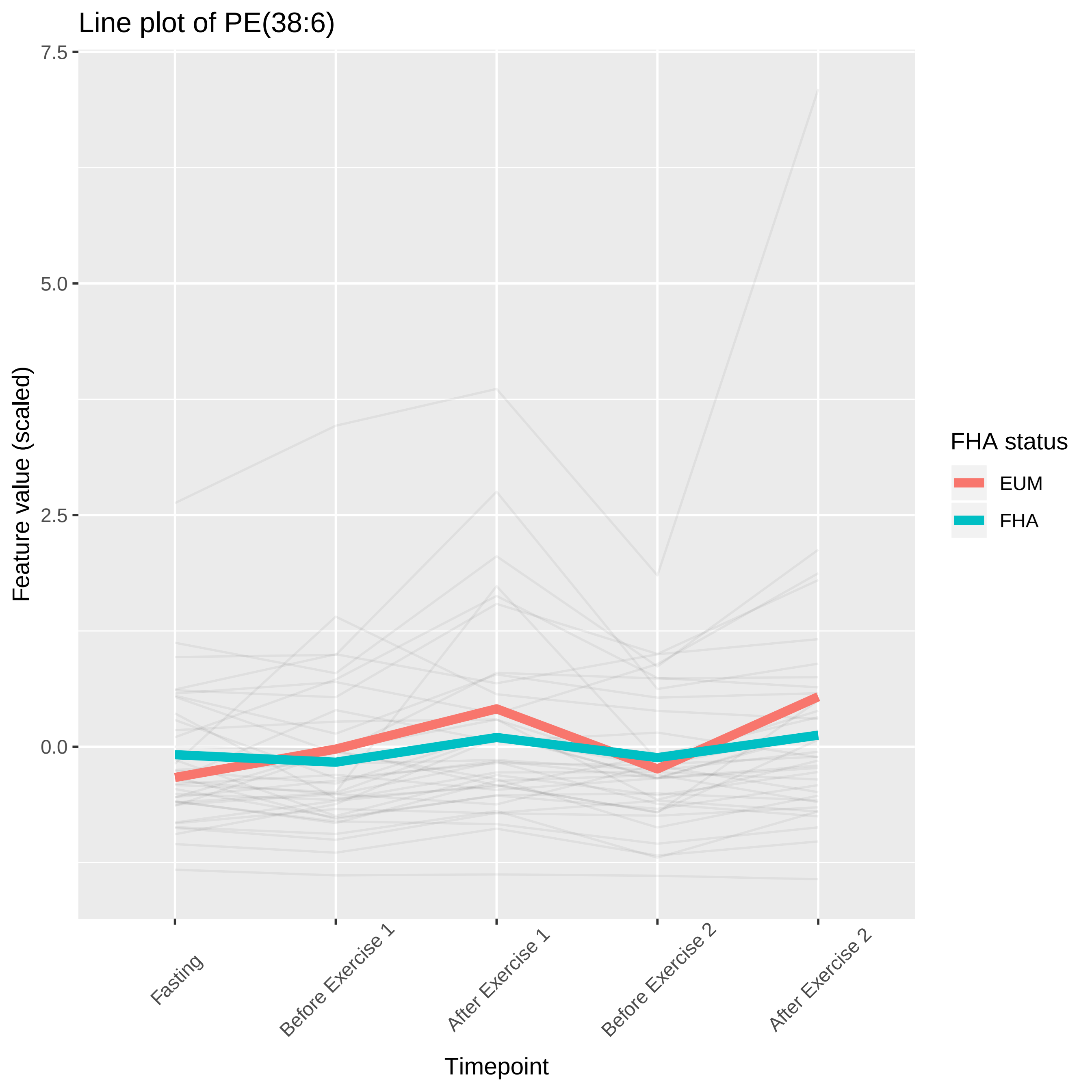

Line plot of PE(38:6)
7.5
5.0
2.5
0.0
Feature value (scaled)
Fasting
Before Exercise 1
After Exercise 1
Before Exercise 2
After Exercise 2
Timepoint
FHA status
EUM
FHA

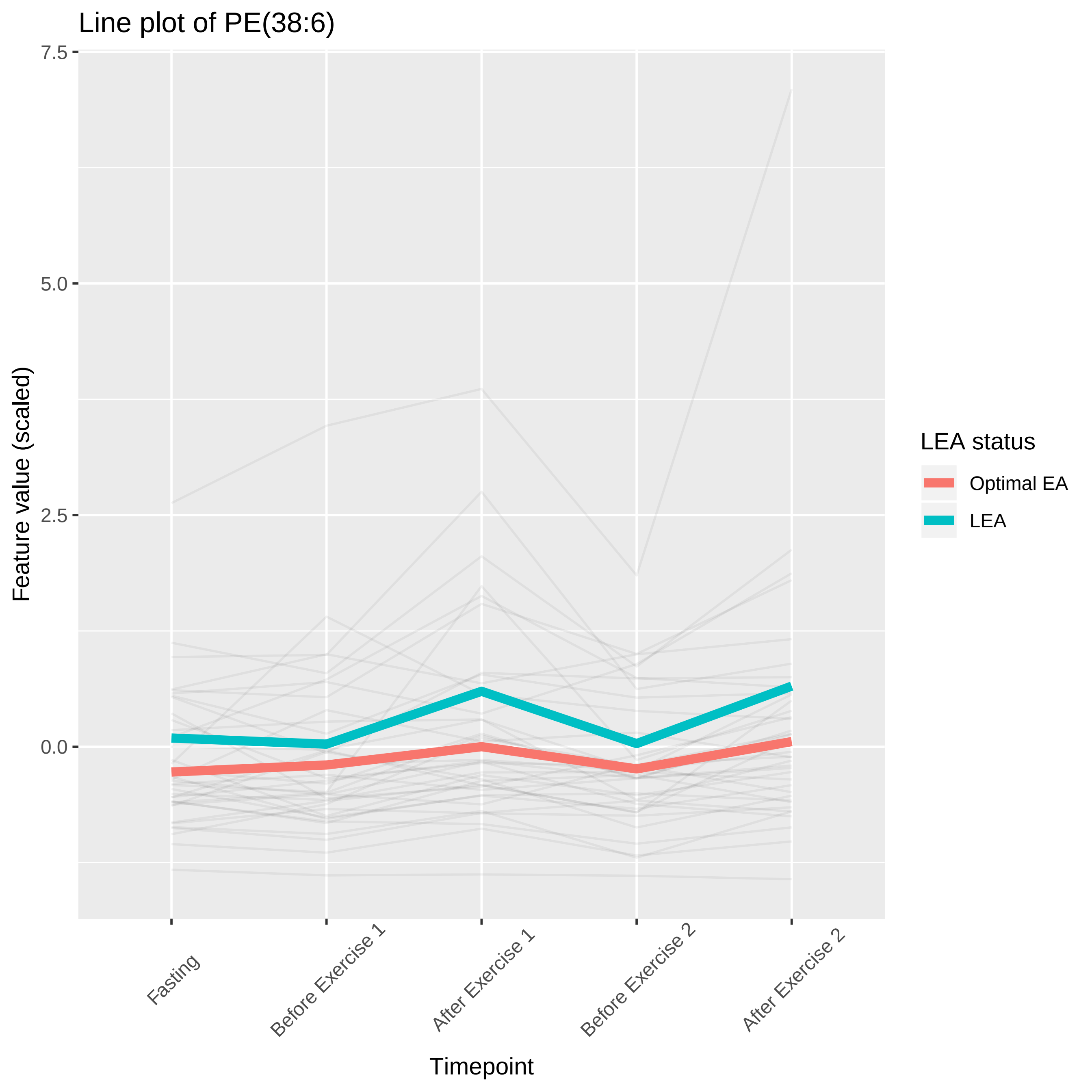

Line plot of PE(38:6)
7.5
5.0
2.5
0.0
Feature value (scaled)
Fasting
Before Exercise 1
After Exercise 1
Before Exercise 2
After Exercise 2
Timepoint
LEA status
Optimal EA
LEA

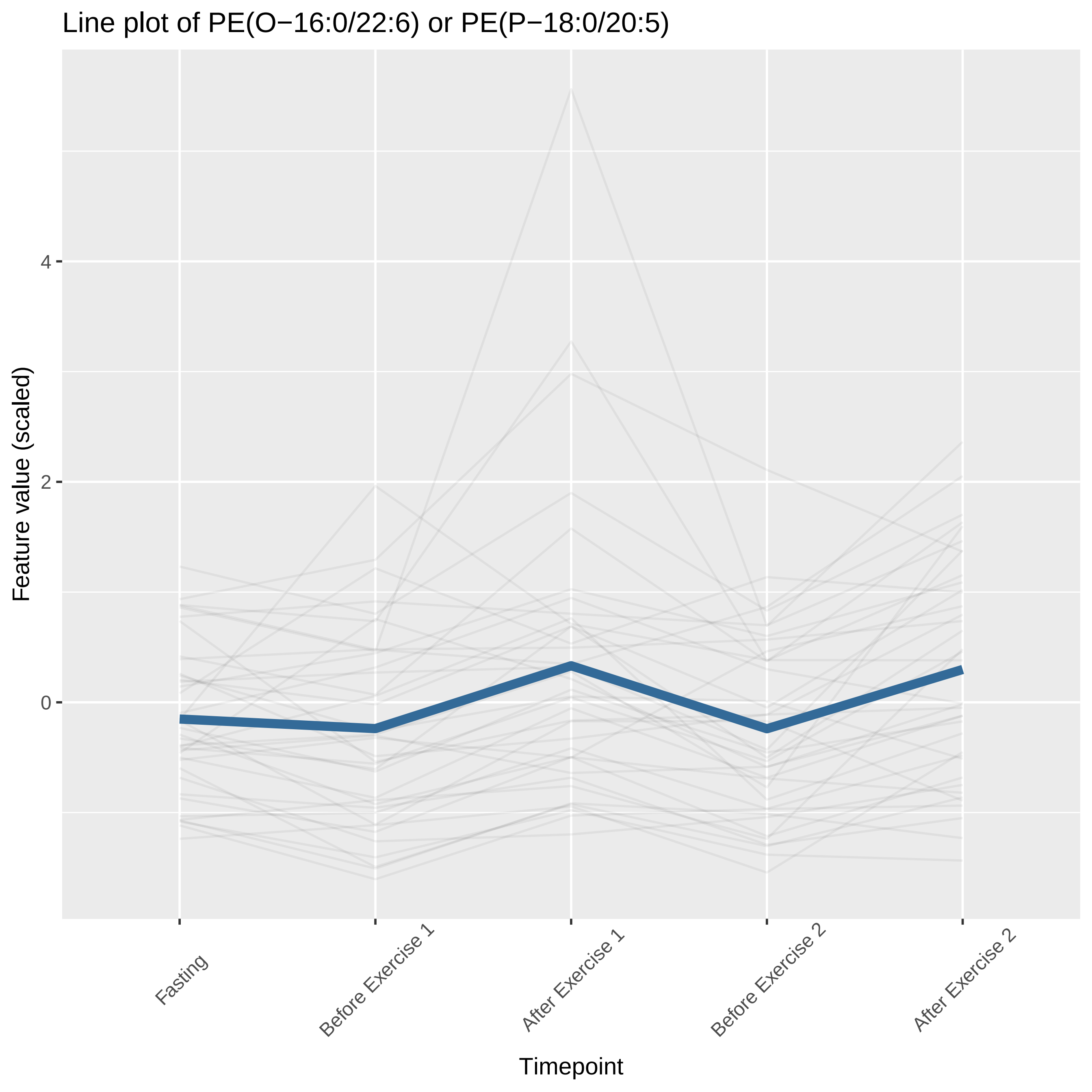

Line plot of PE(O−16:0/22:6) or PE(P−18:0/20:5)
Feature value (scaled)
4
2
0
Fasting
Before Exercise 1
After Exercise 1
Before Exercise 2
After Exercise 2
Timepoint

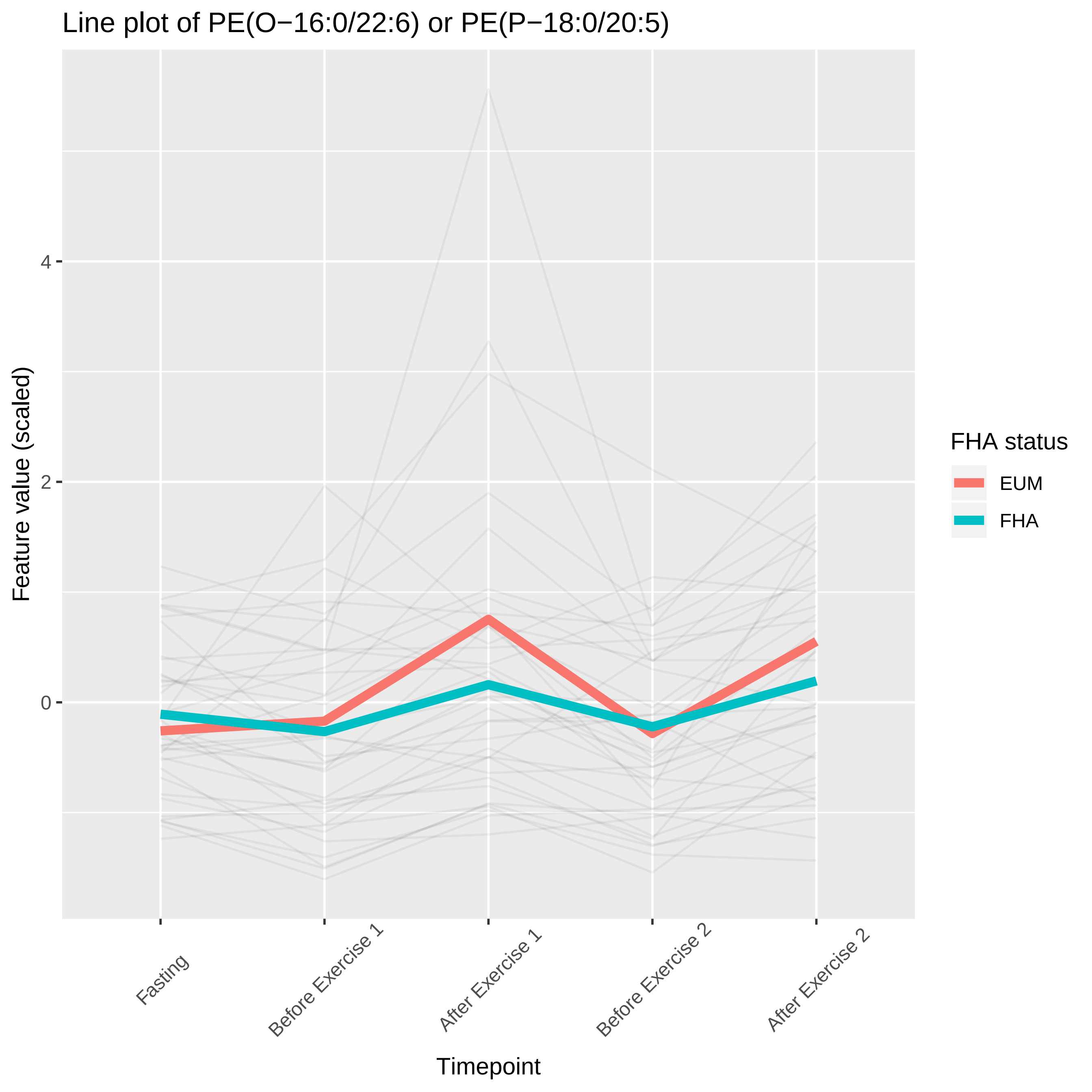
Line plot of PE(O−16:0/22:6) or PE(P−18:0/20:5)
Feature value (scaled)
4
2
0
Fasting
Before Exercise 1
After Exercise 1
Before Exercise 2
After Exercise 2
Timepoint
FHA status
EUM
FHA

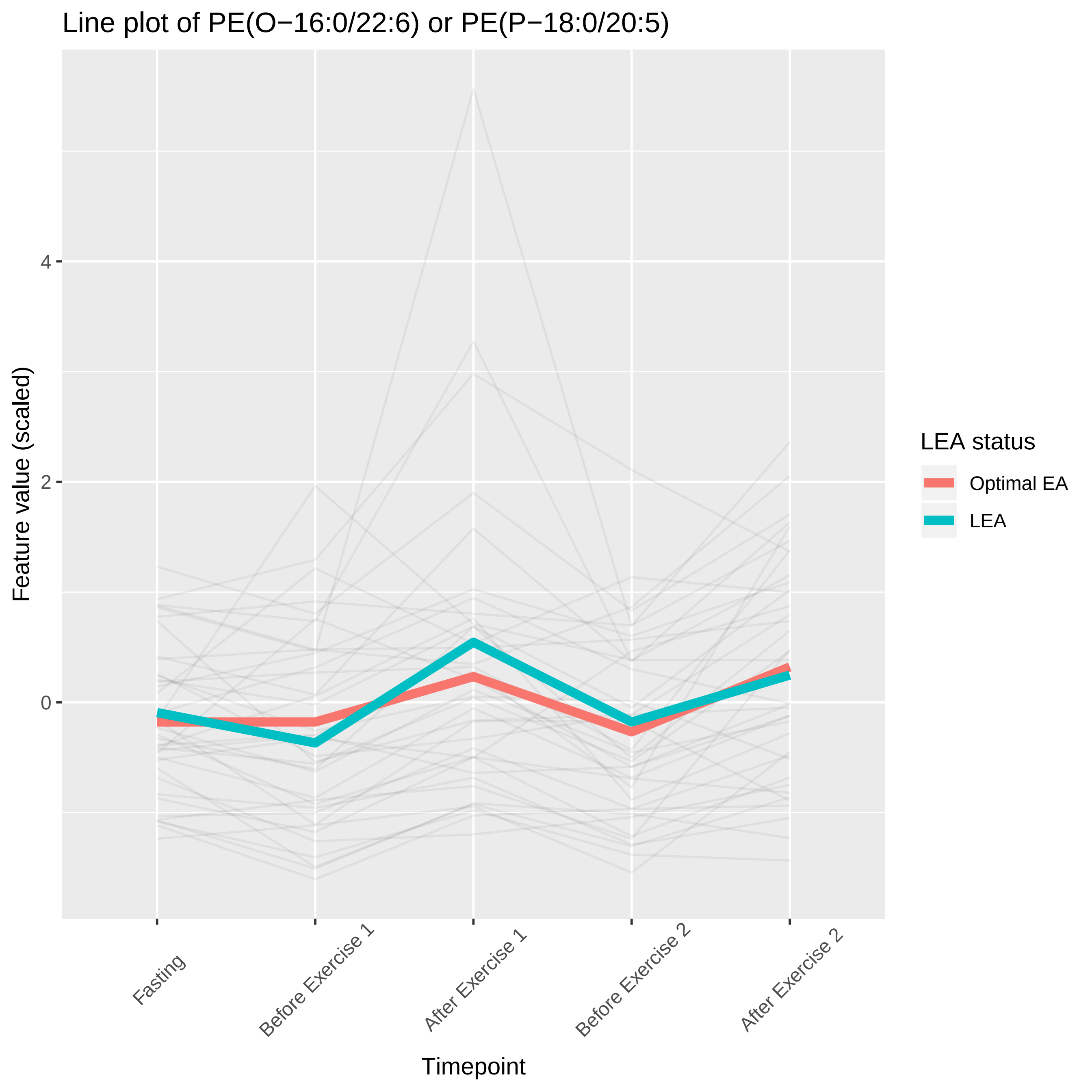
Line plot of PE(O−16:0/22:6) or PE(P−18:0/20:5)
Feature value (scaled)
4
2
0
Fasting
Before Exercise 1
After Exercise 1
Before Exercise 2
After Exercise 2
Timepoint
LEA status
Optimal EA
LEA

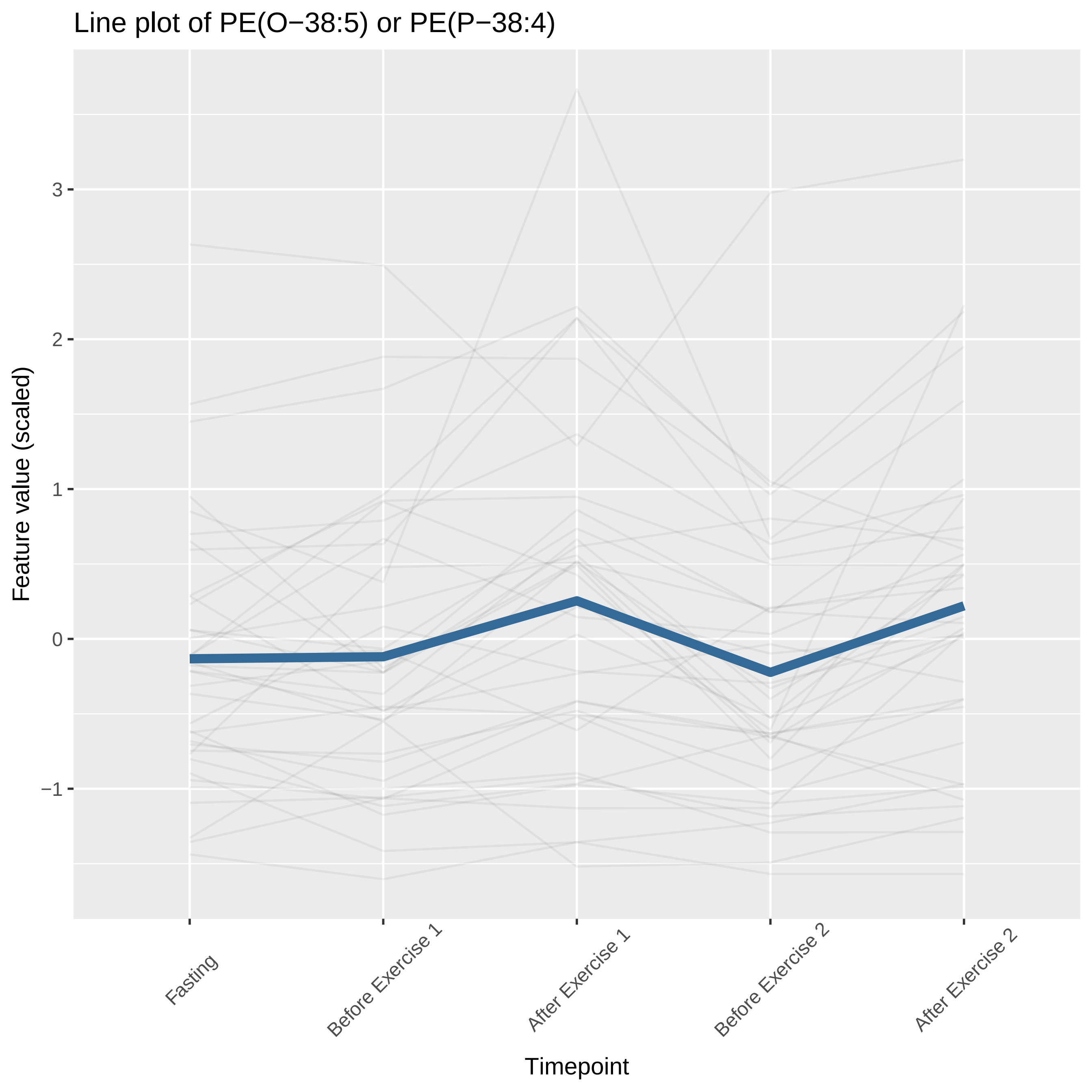

Line plot of PE(O−38:5) or PE(P−38:4)
Feature value (scaled)
3
2
1
0
−1
Fasting
Before Exercise 1
After Exercise 1
Before Exercise 2
After Exercise 2
Timepoint

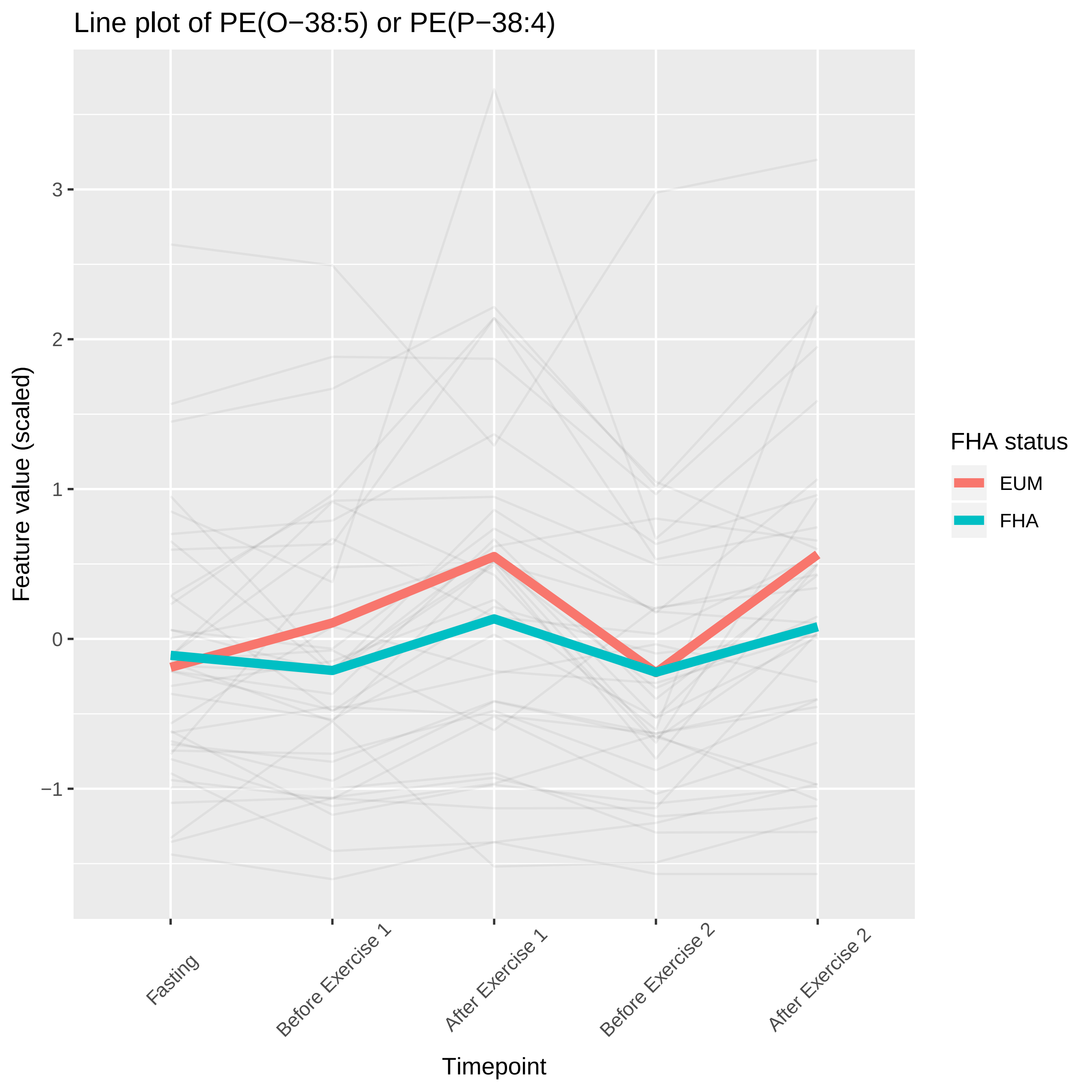

Line plot of PE(O−38:5) or PE(P−38:4)
Feature value (scaled)
3
2
1
0
−1
Fasting
Before Exercise 1
After Exercise 1
Before Exercise 2
After Exercise 2
Timepoint
FHA status
EUM
FHA

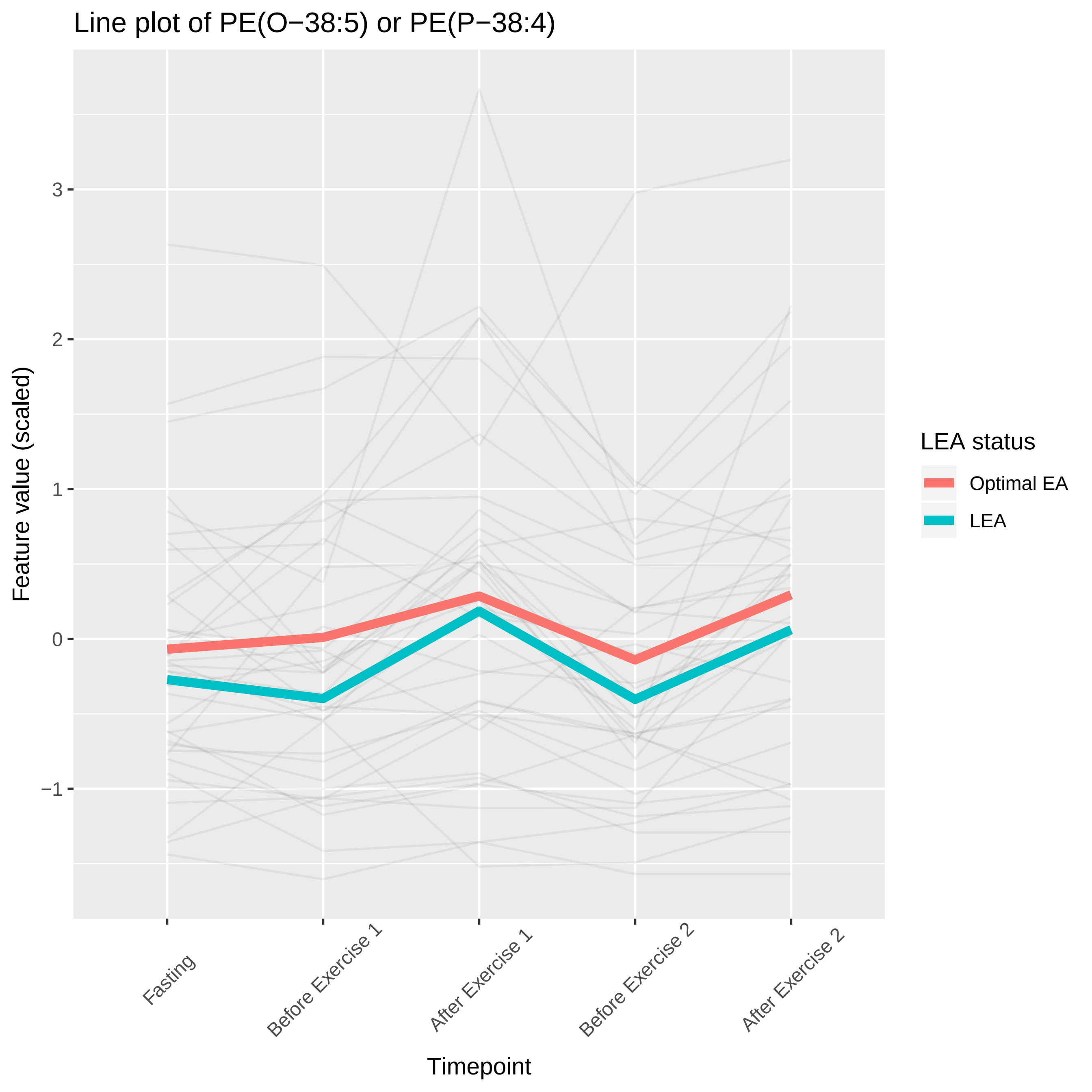

Line plot of PE(O−38:5) or PE(P−38:4)
Feature value (scaled)
3
2
1
0
−1
Fasting
Before Exercise 1
After Exercise 1
Before Exercise 2
After Exercise 2
Timepoint
LEA status
Optimal EA
LEA

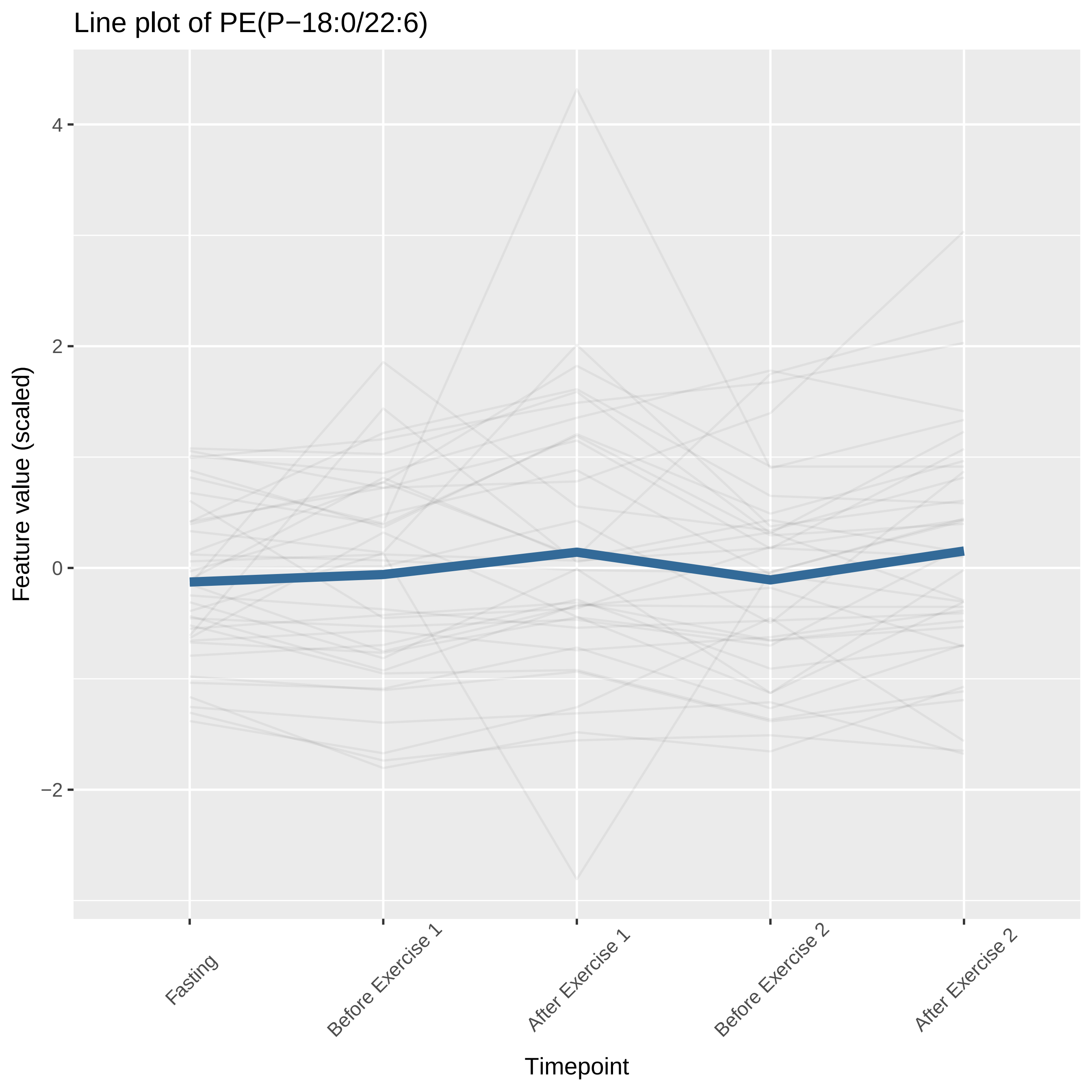

Line plot of PE(P−18:0/22:6)
Feature value (scaled)
4
2
0
−2
Fasting
Before Exercise 1
After Exercise 1
Before Exercise 2
After Exercise 2
Timepoint

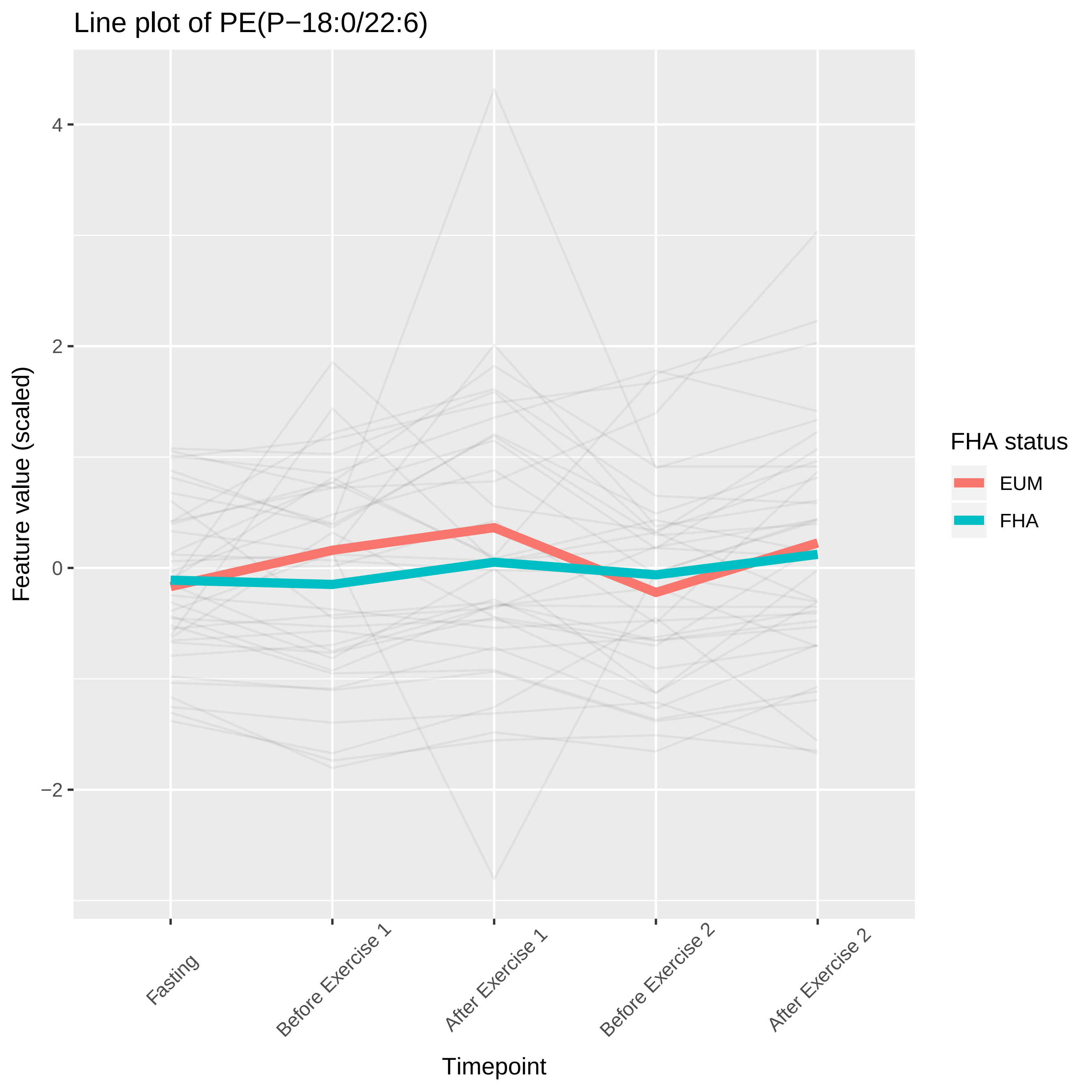

Line plot of PE(P−18:0/22:6)
Feature value (scaled)
4
2
0
−2
Fasting
Before Exercise 1
After Exercise 1
Before Exercise 2
After Exercise 2
Timepoint
FHA status
EUM
FHA

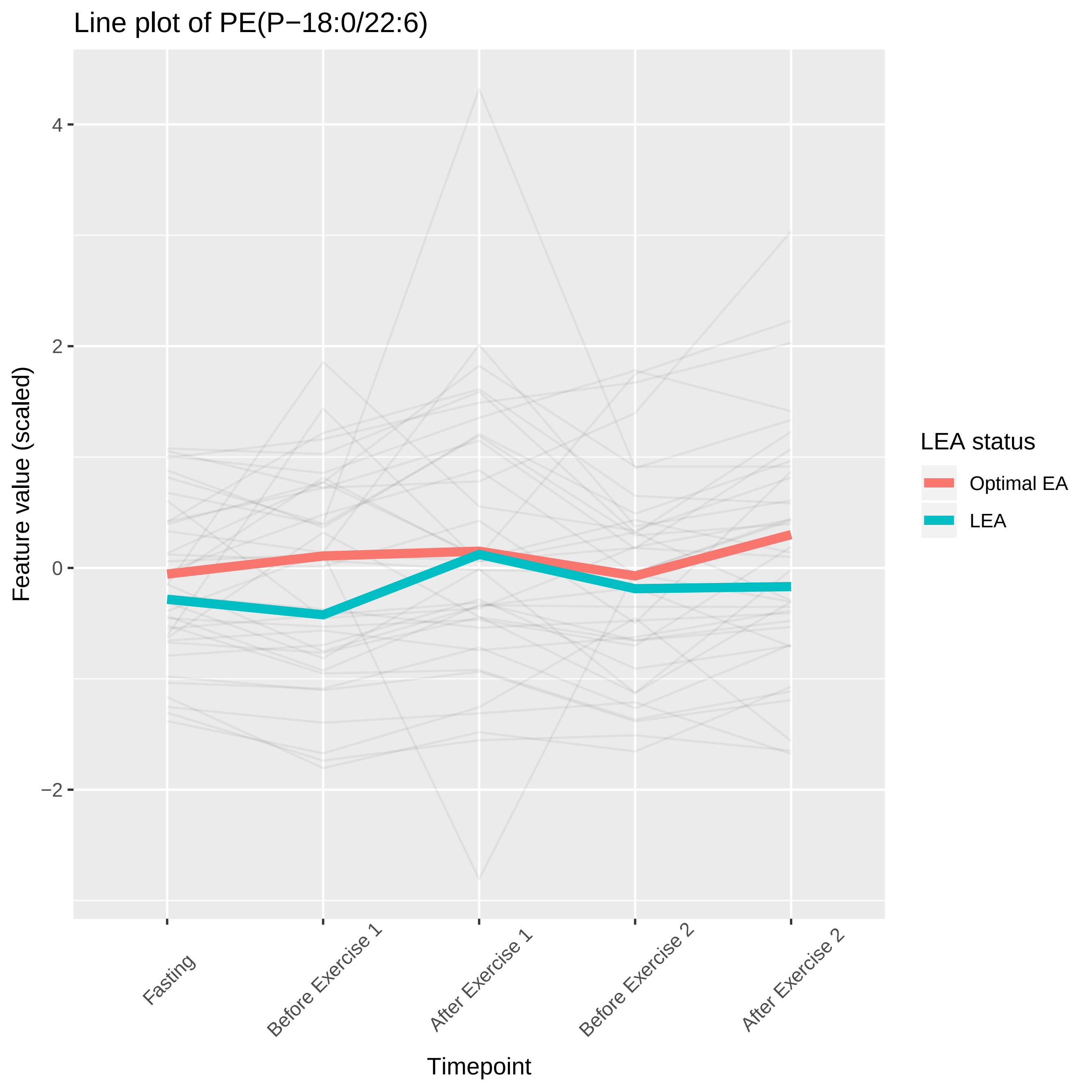

Line plot of PE(P−18:0/22:6)
Feature value (scaled)
4
2
0
−2
Fasting
Before Exercise 1
After Exercise 1
Before Exercise 2
After Exercise 2
Timepoint
LEA status
Optimal EA
LEA

SPHINGOLIPIDS

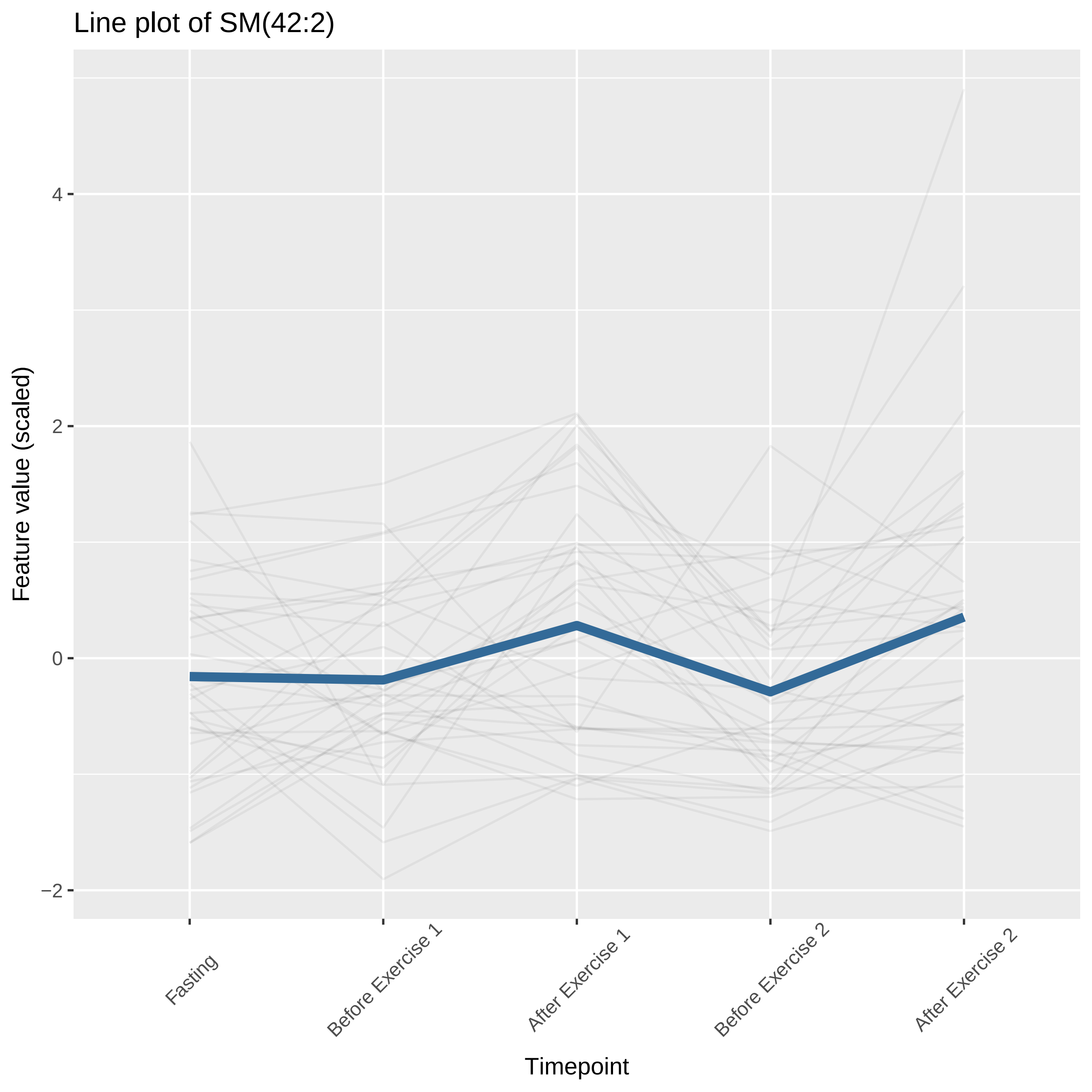

Line plot of SM(42:2)
Feature value (scaled)
4
2
0
−2
Fasting
Before Exercise 1
After Exercise 1
Before Exercise 2
After Exercise 2
Timepoint

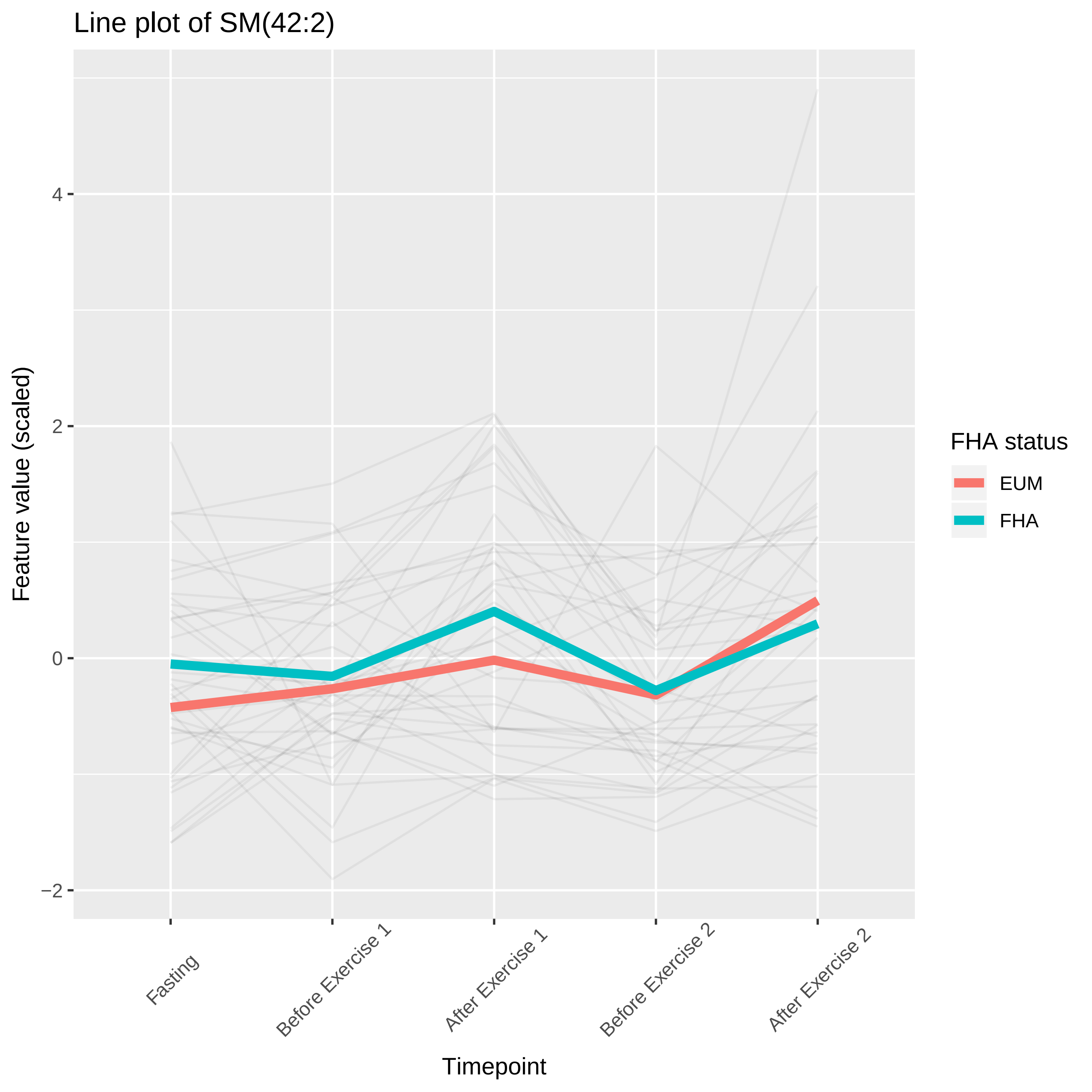

Line plot of SM(42:2)
Feature value (scaled)
4
2
0
−2
Fasting
Before Exercise 1
After Exercise 1
Before Exercise 2
After Exercise 2
Timepoint
FHA status
EUM
FHA

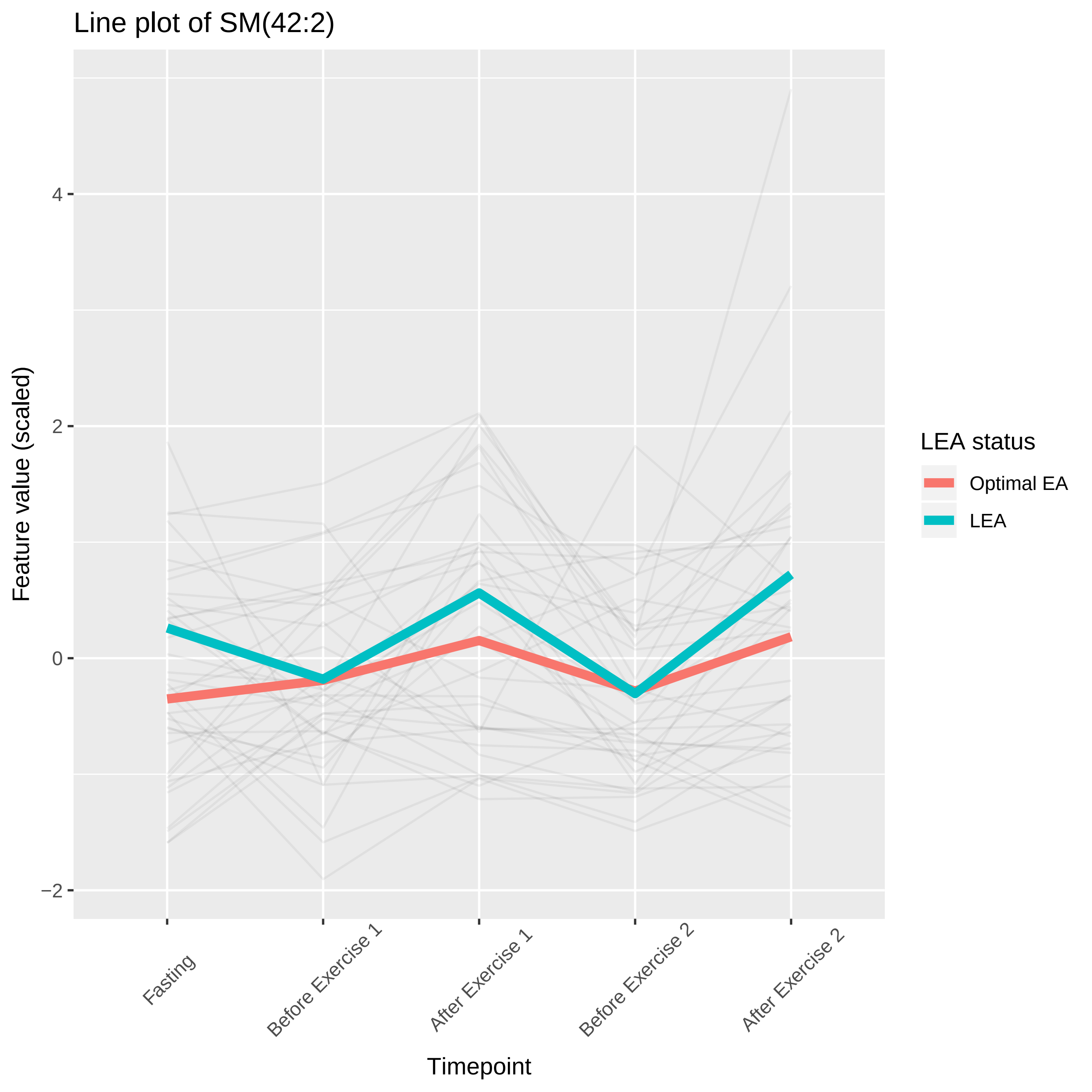

Line plot of SM(42:2)
Feature value (scaled)
4
2
0
−2
Fasting
Before Exercise 1
After Exercise 1
Before Exercise 2
After Exercise 2
Timepoint
LEA status
Optimal EA
LEA

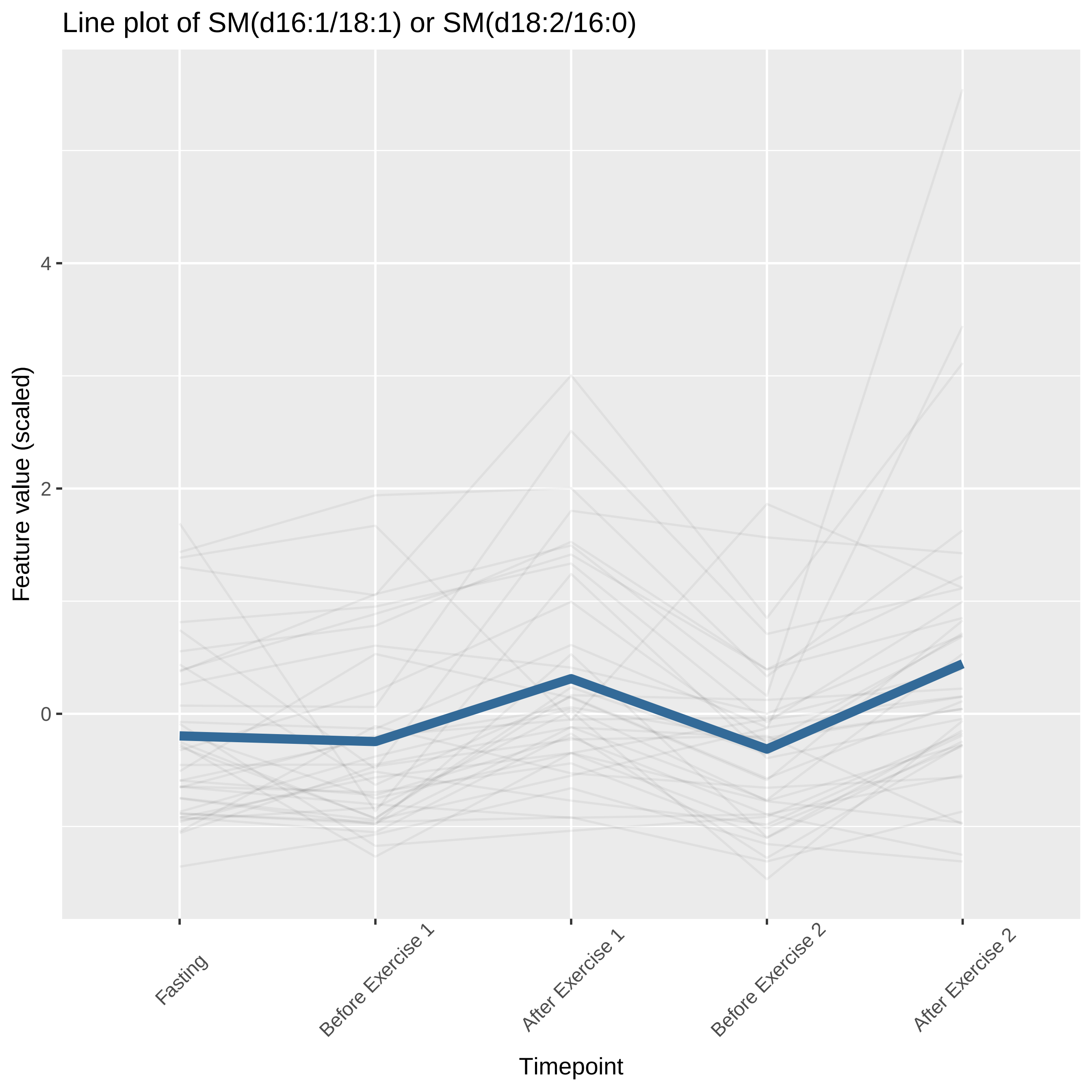
Line plot of SM(d16:1/18:1) or SM(d18:2/16:0)
Feature value (scaled)
4
2
0
Fasting
Before Exercise 1
After Exercise 1
Before Exercise 2
After Exercise 2
Timepoint

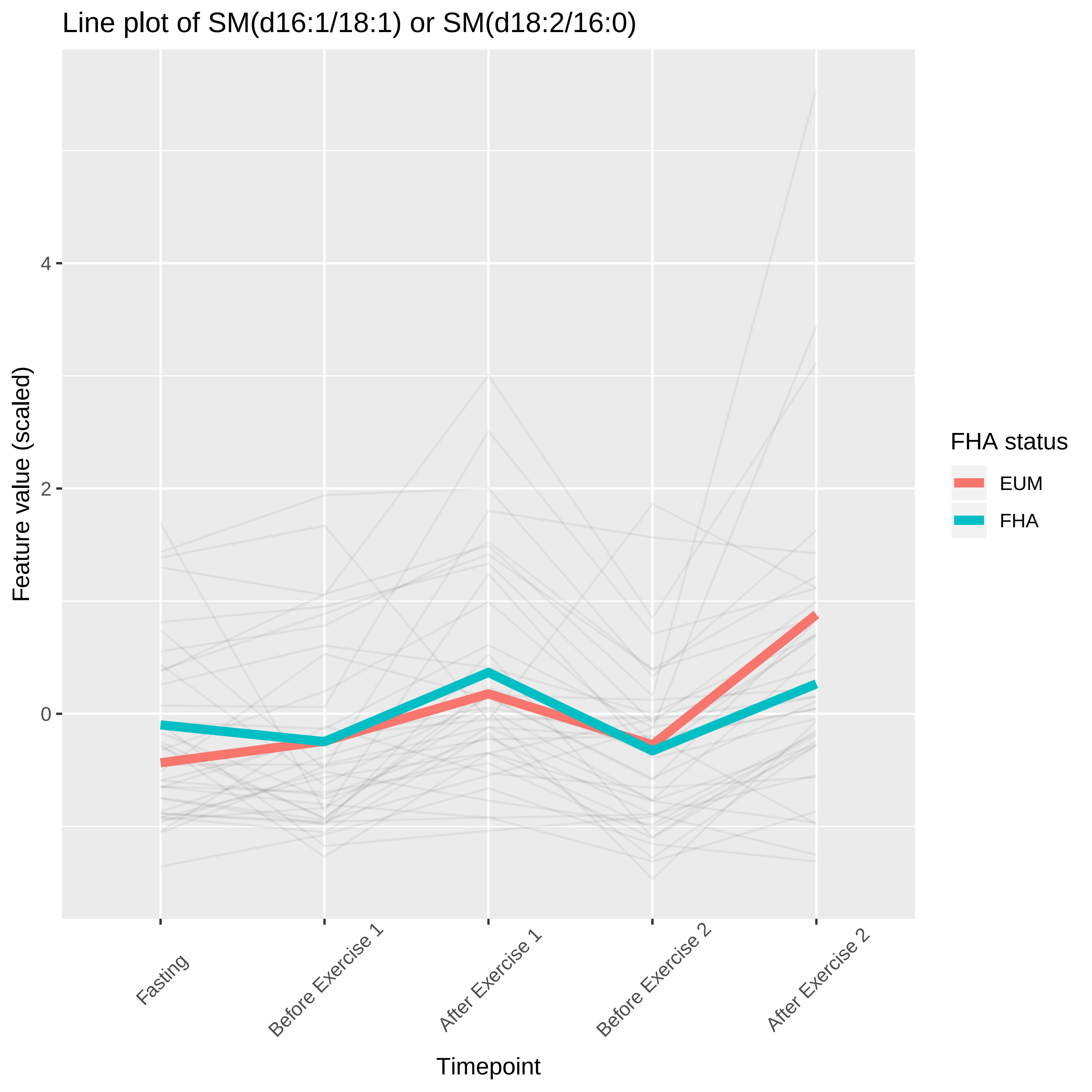
Line plot of SM(d16:1/18:1) or SM(d18:2/16:0)
Feature value (scaled)
4
2
0
Fasting
Before Exercise 1
After Exercise 1
Before Exercise 2
After Exercise 2
Timepoint
FHA status
EUM
FHA

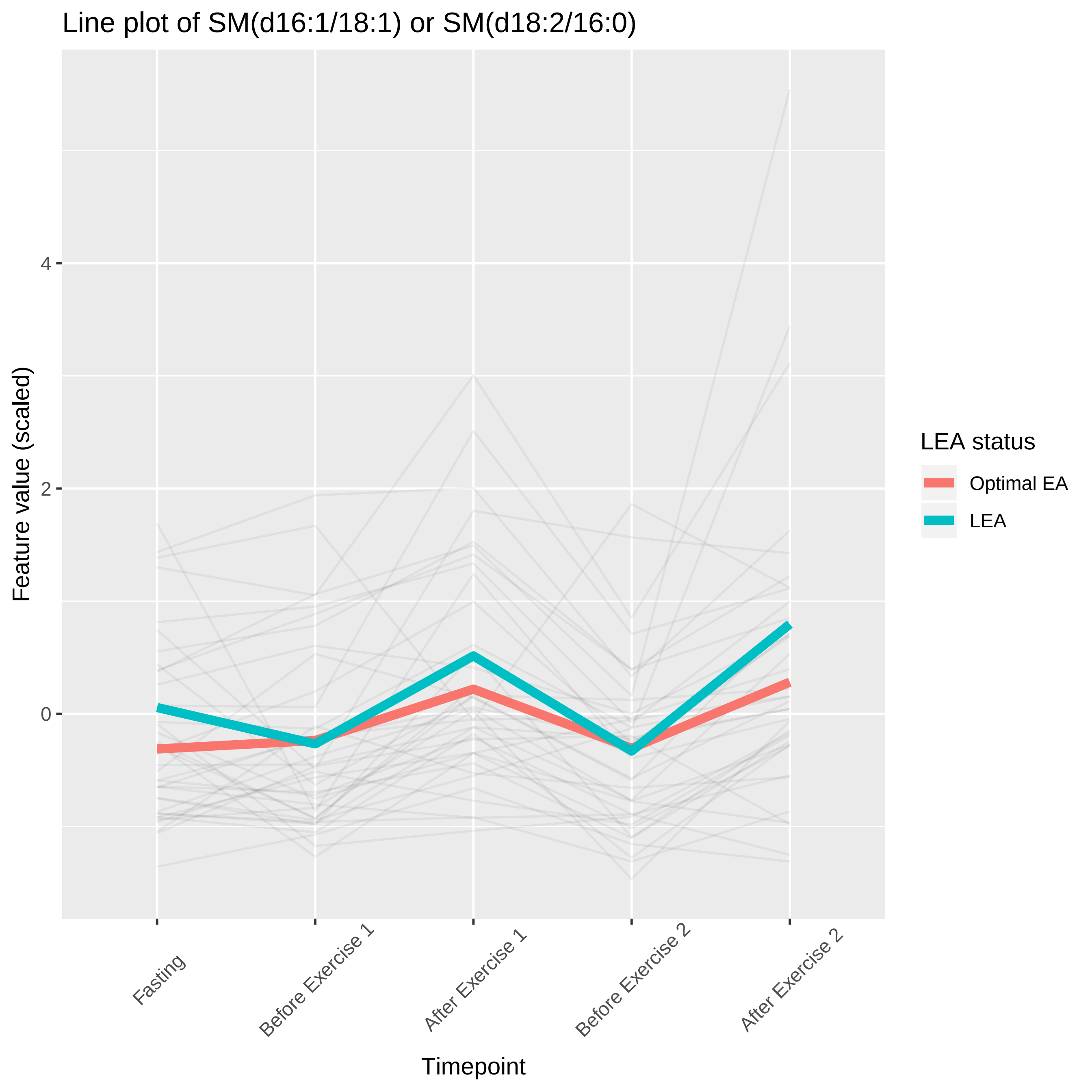

Line plot of SM(d16:1/18:1) or SM(d18:2/16:0)
Feature value (scaled)
4
2
0
Fasting
Before Exercise 1
After Exercise 1
Before Exercise 2
After Exercise 2
Timepoint
LEA status
Optimal EA
LEA

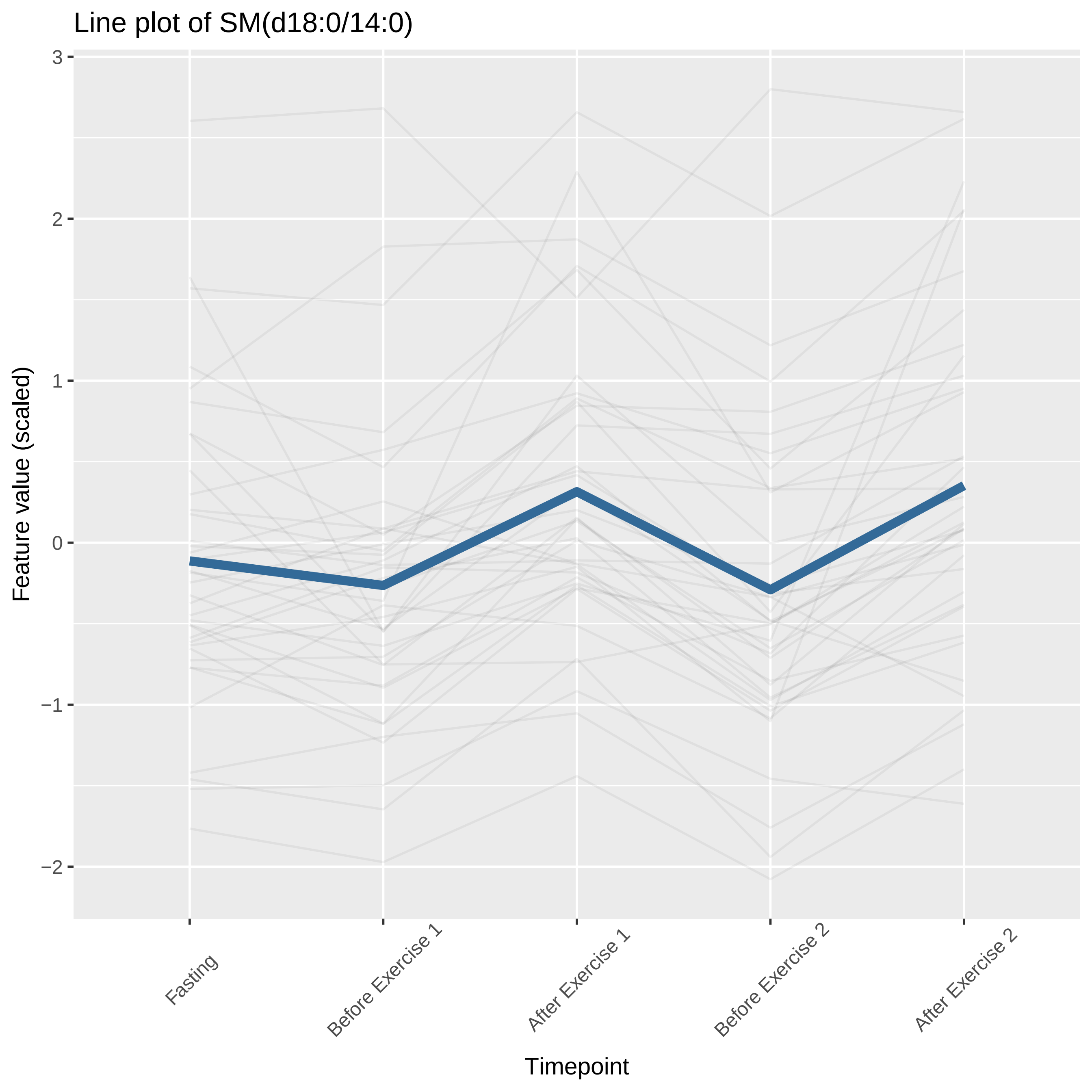

Line plot of SM(d18:0/14:0)
Feature value (scaled)
3
2
1
0
−1
−2
Fasting
Before Exercise 1
After Exercise 1
Before Exercise 2
After Exercise 2
Timepoint

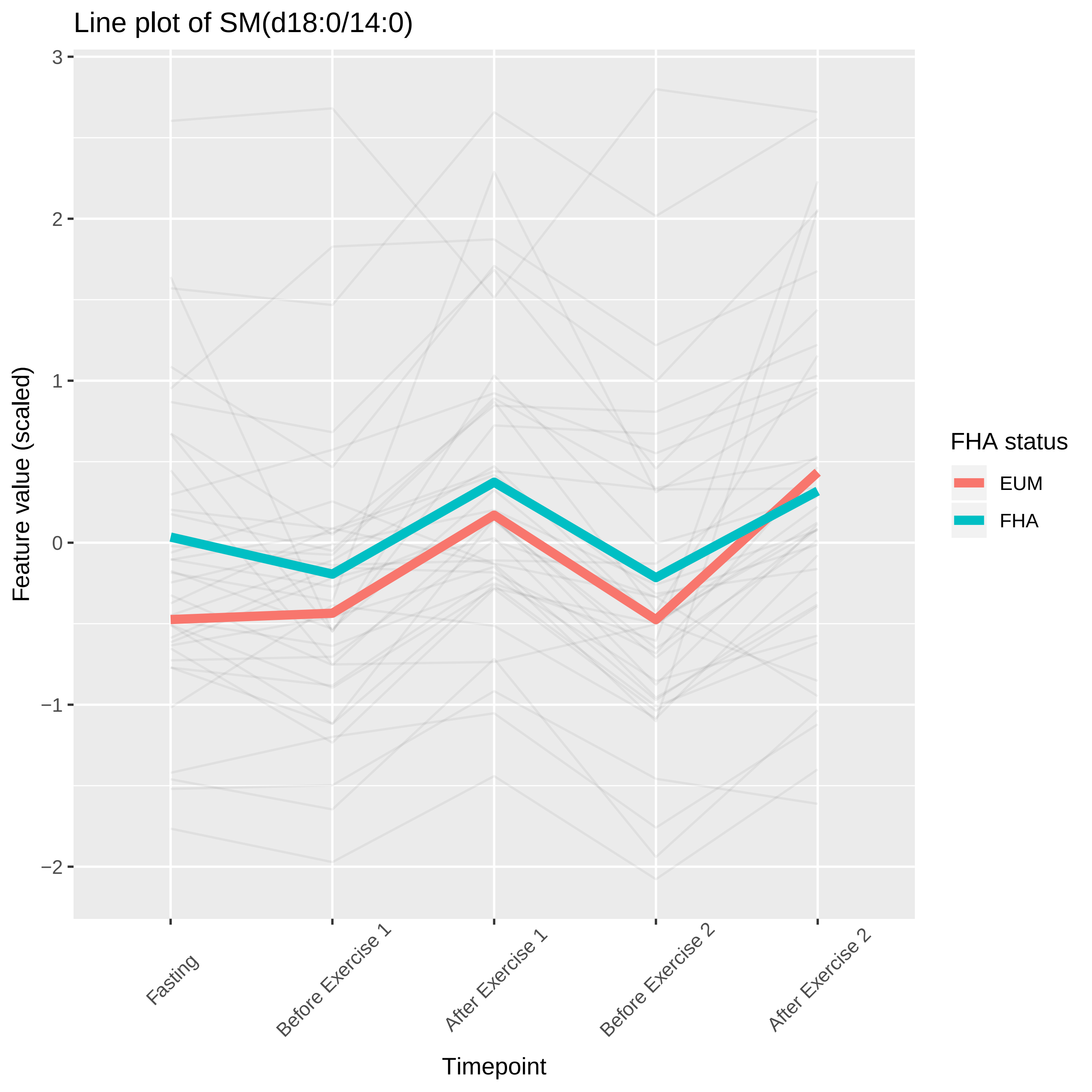
Line plot of SM(d18:0/14:0)
Feature value (scaled)
3
2
1
0
−1
−2
Fasting
Before Exercise 1
After Exercise 1
Before Exercise 2
After Exercise 2
Timepoint
FHA status
EUM
FHA

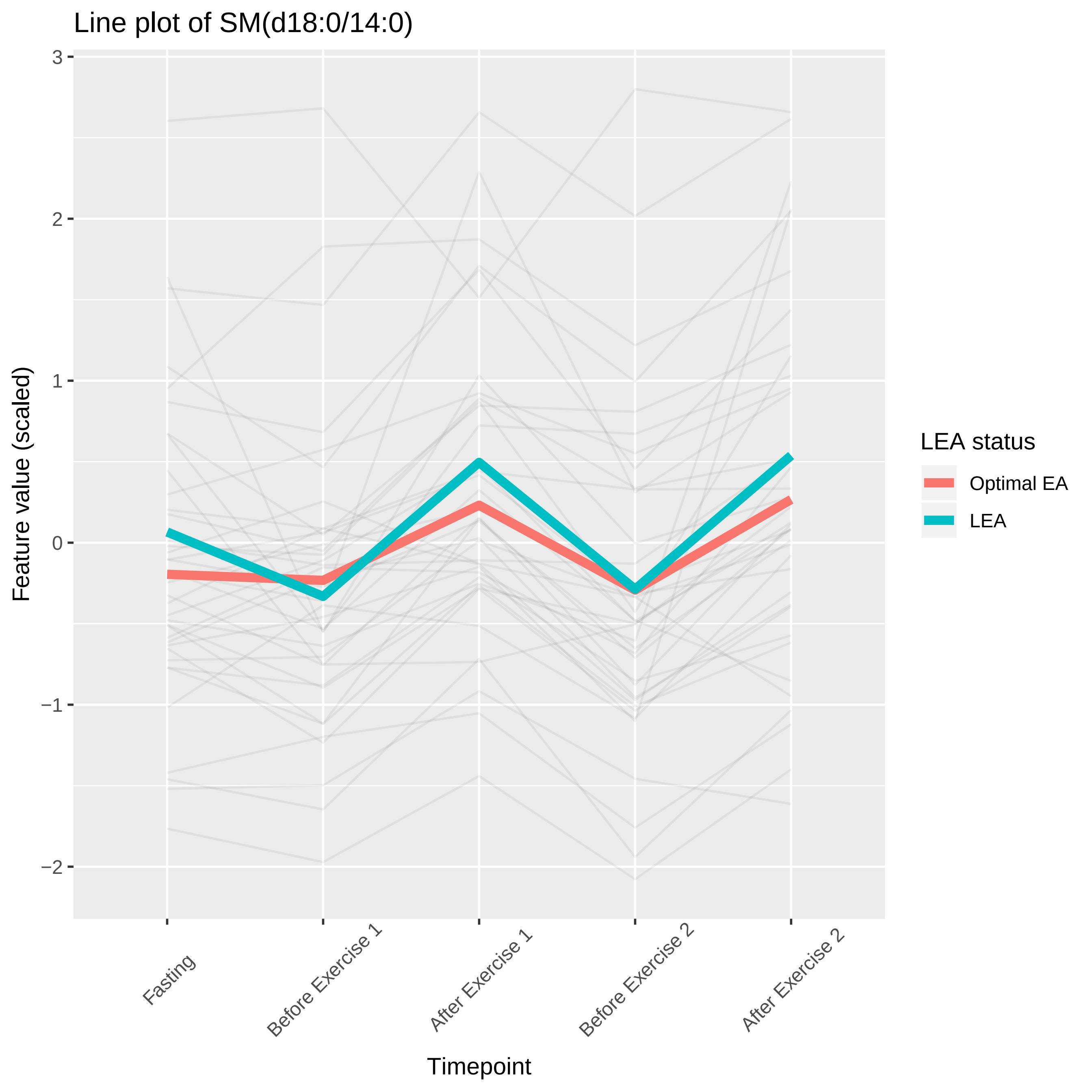

Line plot of SM(d18:0/14:0)
Feature value (scaled)
3
2
1
0
−1
−2
Fasting
Before Exercise 1
After Exercise 1
Before Exercise 2
After Exercise 2
Timepoint
LEA status
Optimal EA
LEA

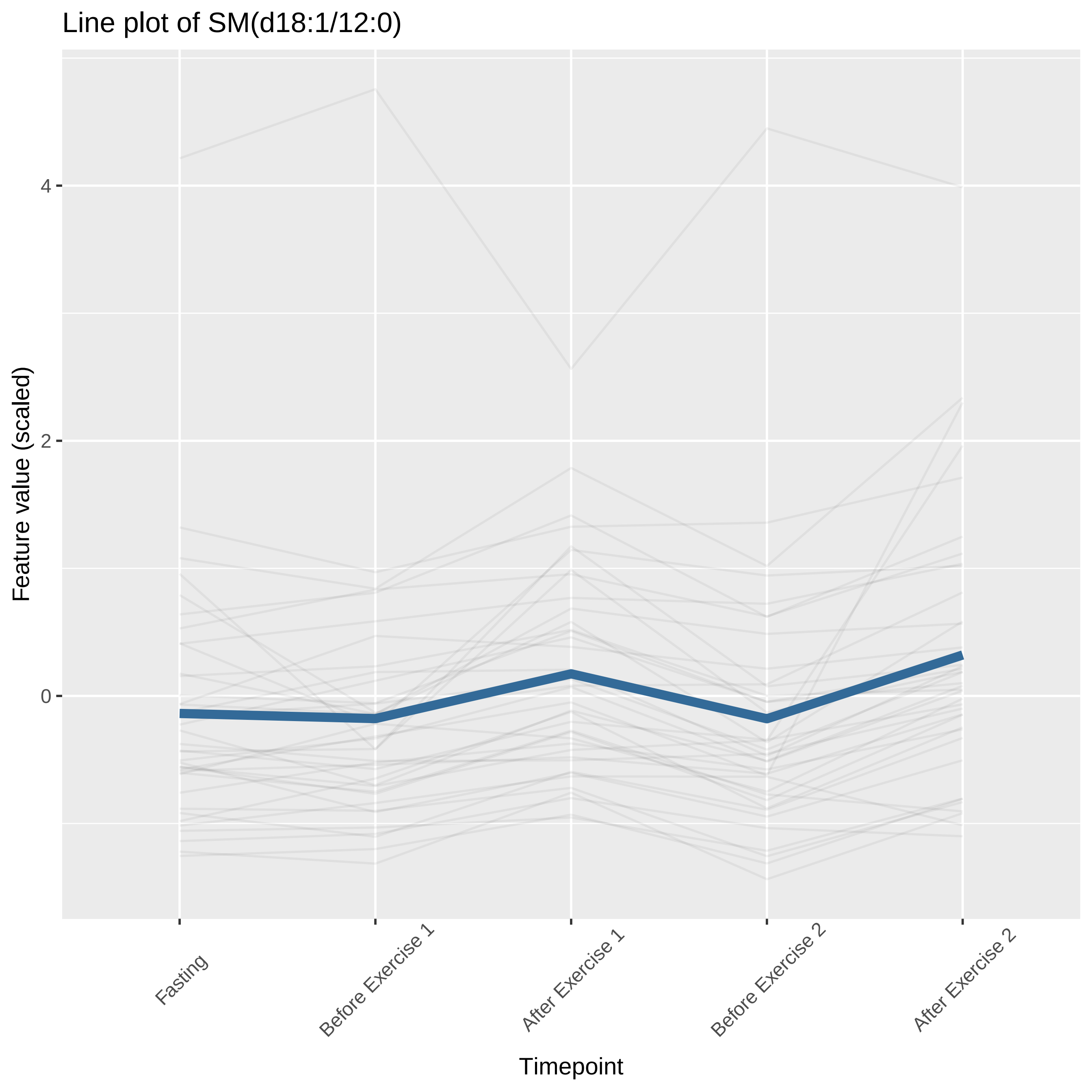

Line plot of SM(d18:1/12:0)
Feature value (scaled)
4
2
0
Fasting
Before Exercise 1
After Exercise 1
Before Exercise 2
After Exercise 2
Timepoint

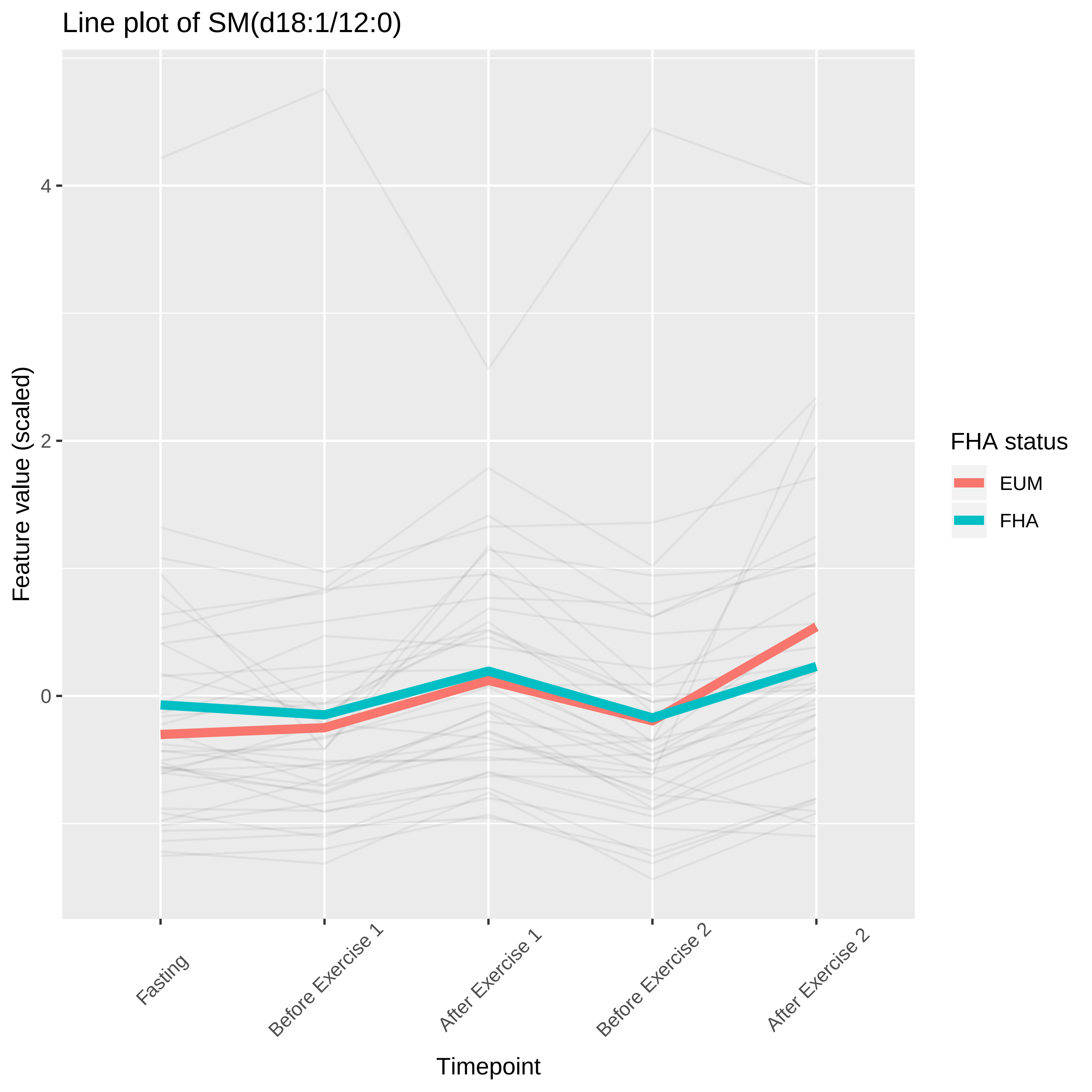

Line plot of SM(d18:1/12:0)
Feature value (scaled)
4
2
0
Fasting
Before Exercise 1
After Exercise 1
Before Exercise 2
After Exercise 2
Timepoint
FHA status
EUM
FHA

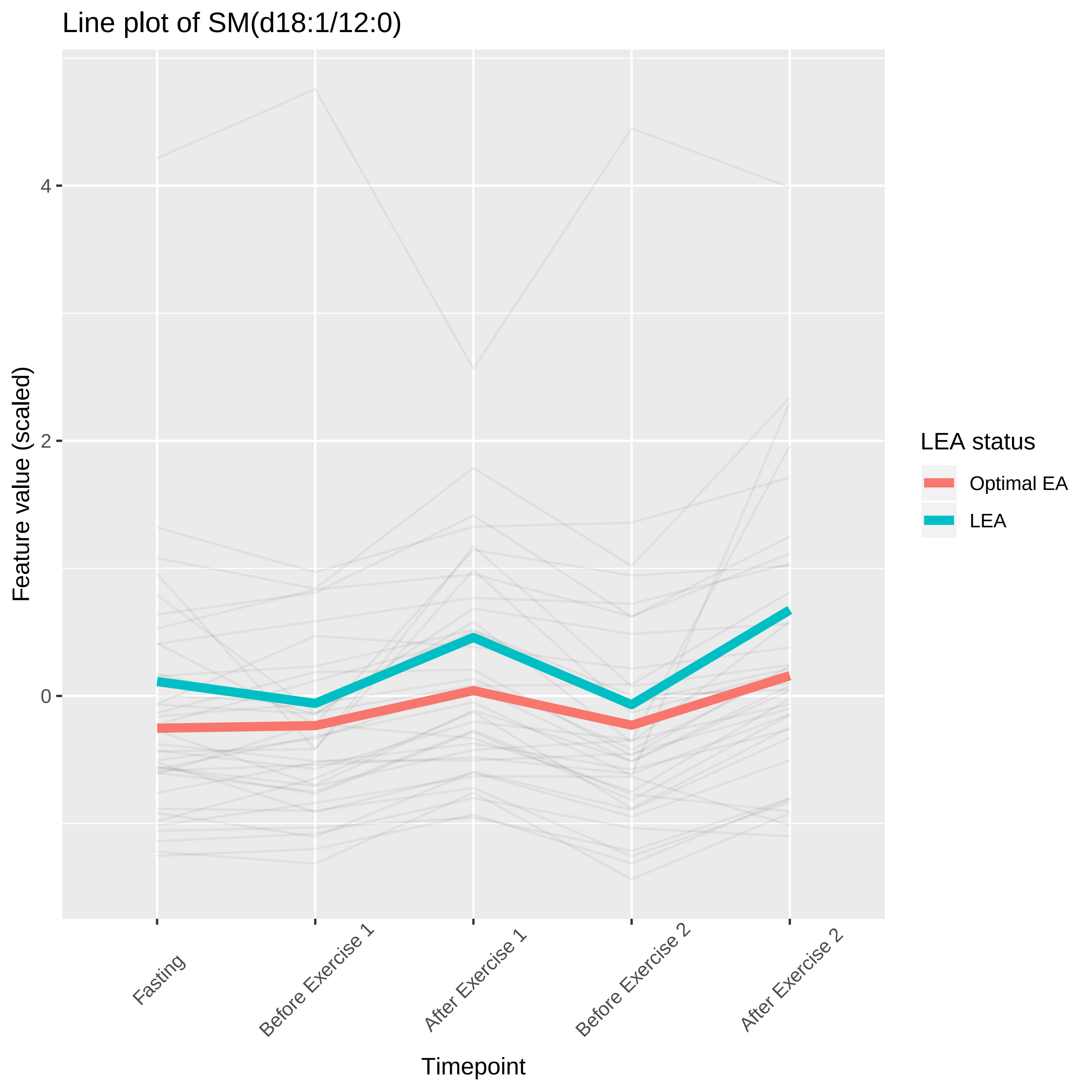

Line plot of SM(d18:1/12:0)
Feature value (scaled)
4
2
0
Fasting
Before Exercise 1
After Exercise 1
Before Exercise 2
After Exercise 2
Timepoint
LEA status
Optimal EA
LEA

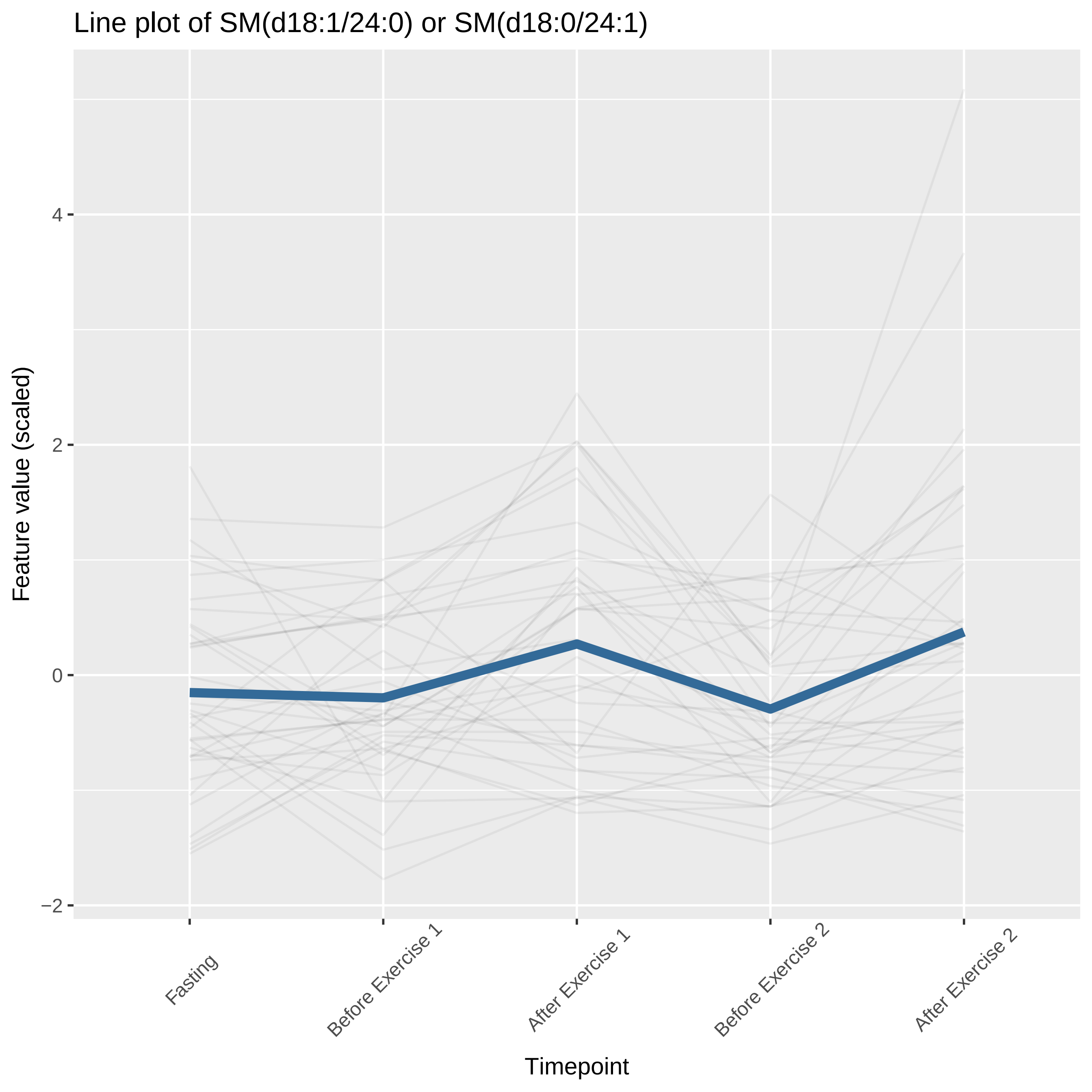

Line plot of SM(d18:1/24:0) or SM(d18:0/24:1)
Feature value (scaled)
4
2
0
−2
Fasting
Before Exercise 1
After Exercise 1
Before Exercise 2
After Exercise 2
Timepoint

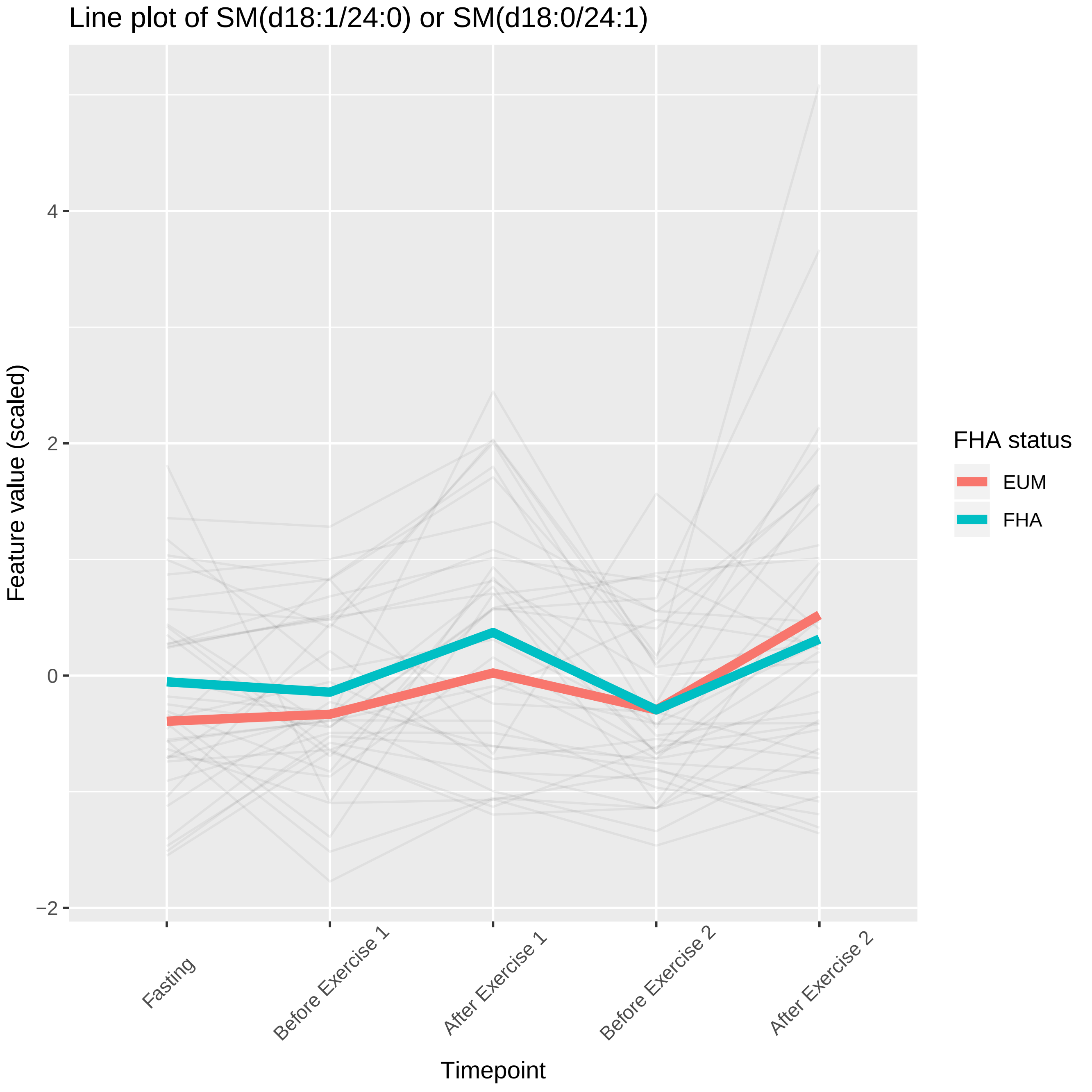
Line plot of SM(d18:1/24:0) or SM(d18:0/24:1)
Feature value (scaled)
4
2
0
−2
Fasting
Before Exercise 1
After Exercise 1
Before Exercise 2
After Exercise 2
Timepoint
FHA status
EUM
FHA

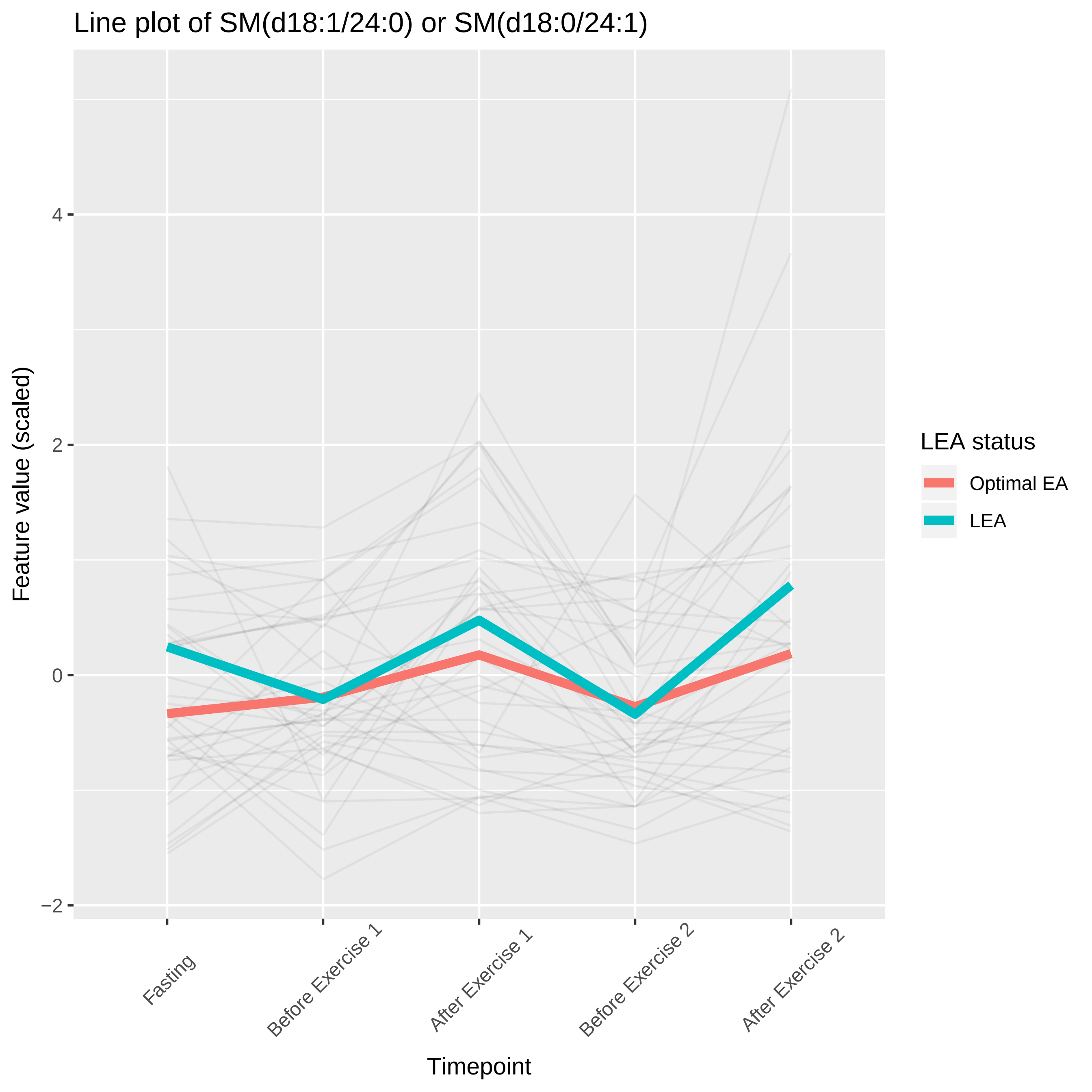
Line plot of SM(d18:1/24:0) or SM(d18:0/24:1)
Feature value (scaled)
4
2
0
−2
Fasting
Before Exercise 1
After Exercise 1
Before Exercise 2
After Exercise 2
Timepoint
LEA status
Optimal EA
LEA

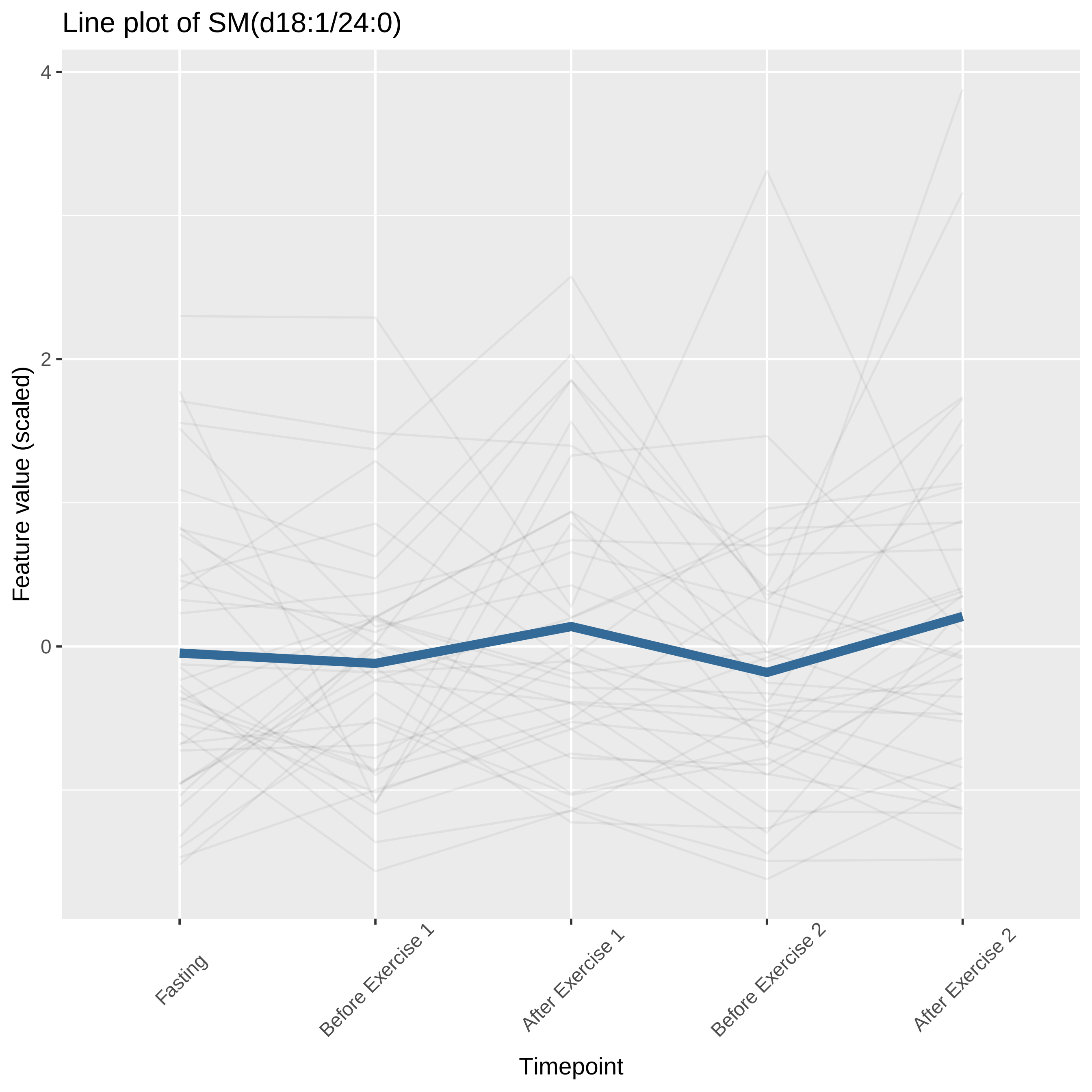
Line plot of SM(d18:1/24:0)
Feature value (scaled)
4
2
0
Fasting
Before Exercise 1
After Exercise 1
Before Exercise 2
After Exercise 2
Timepoint

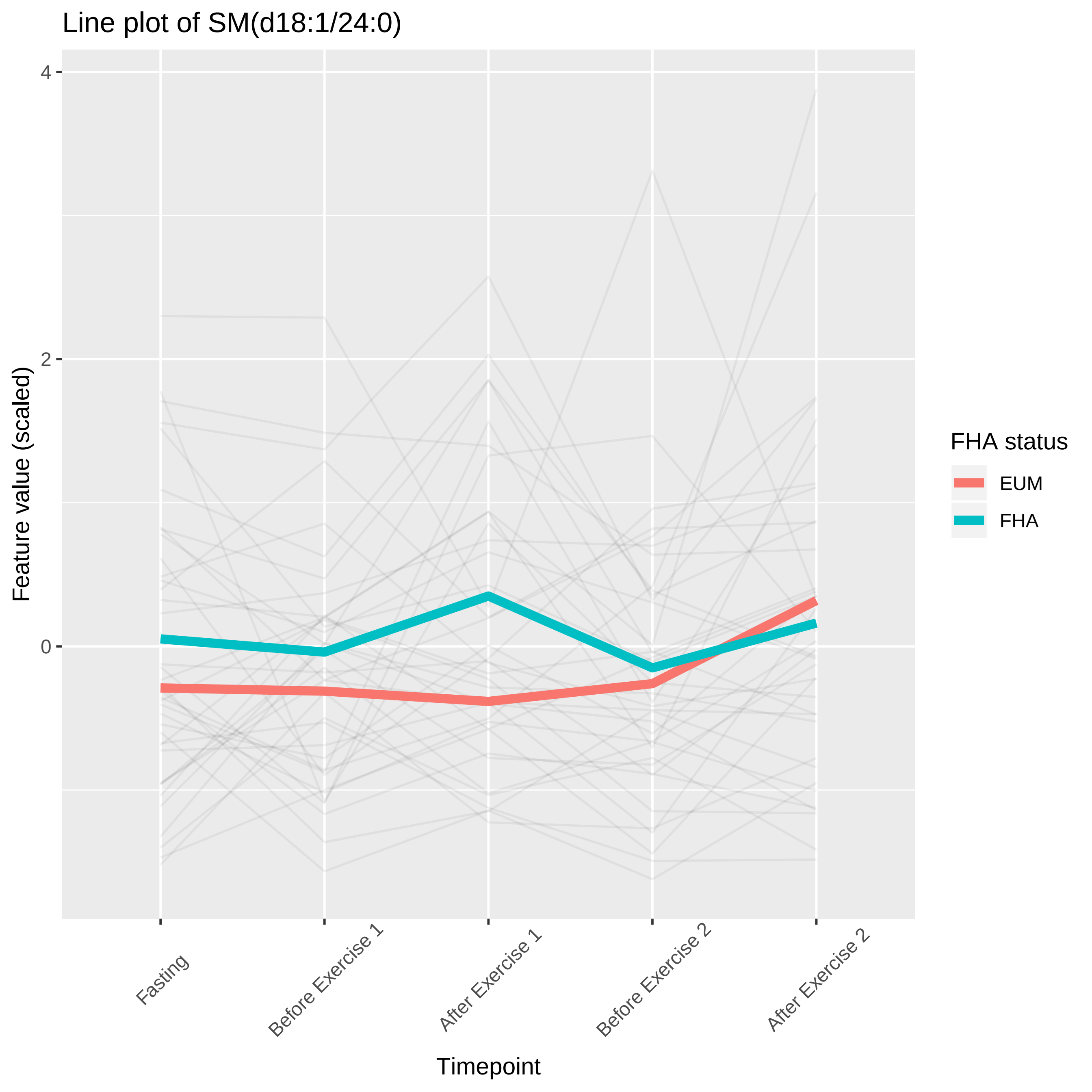

Line plot of SM(d18:1/24:0)
Feature value (scaled)
4
2
0
Fasting
Before Exercise 1
After Exercise 1
Before Exercise 2
After Exercise 2
Timepoint
FHA status
EUM
FHA

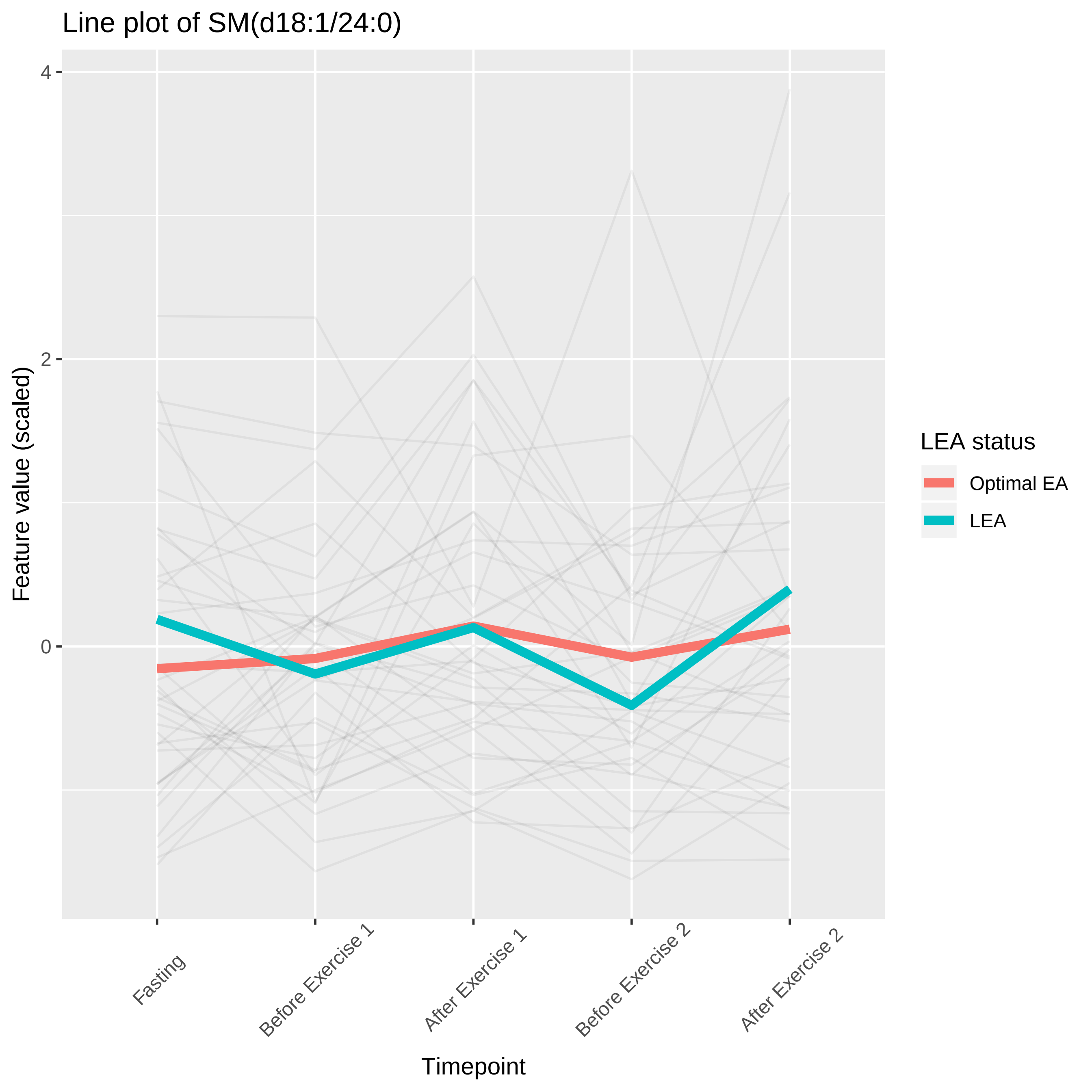

Line plot of SM(d18:1/24:0)
Feature value (scaled)
4
2
0
Fasting
Before Exercise 1
After Exercise 1
Before Exercise 2
After Exercise 2
Timepoint
LEA status
Optimal EA
LEA

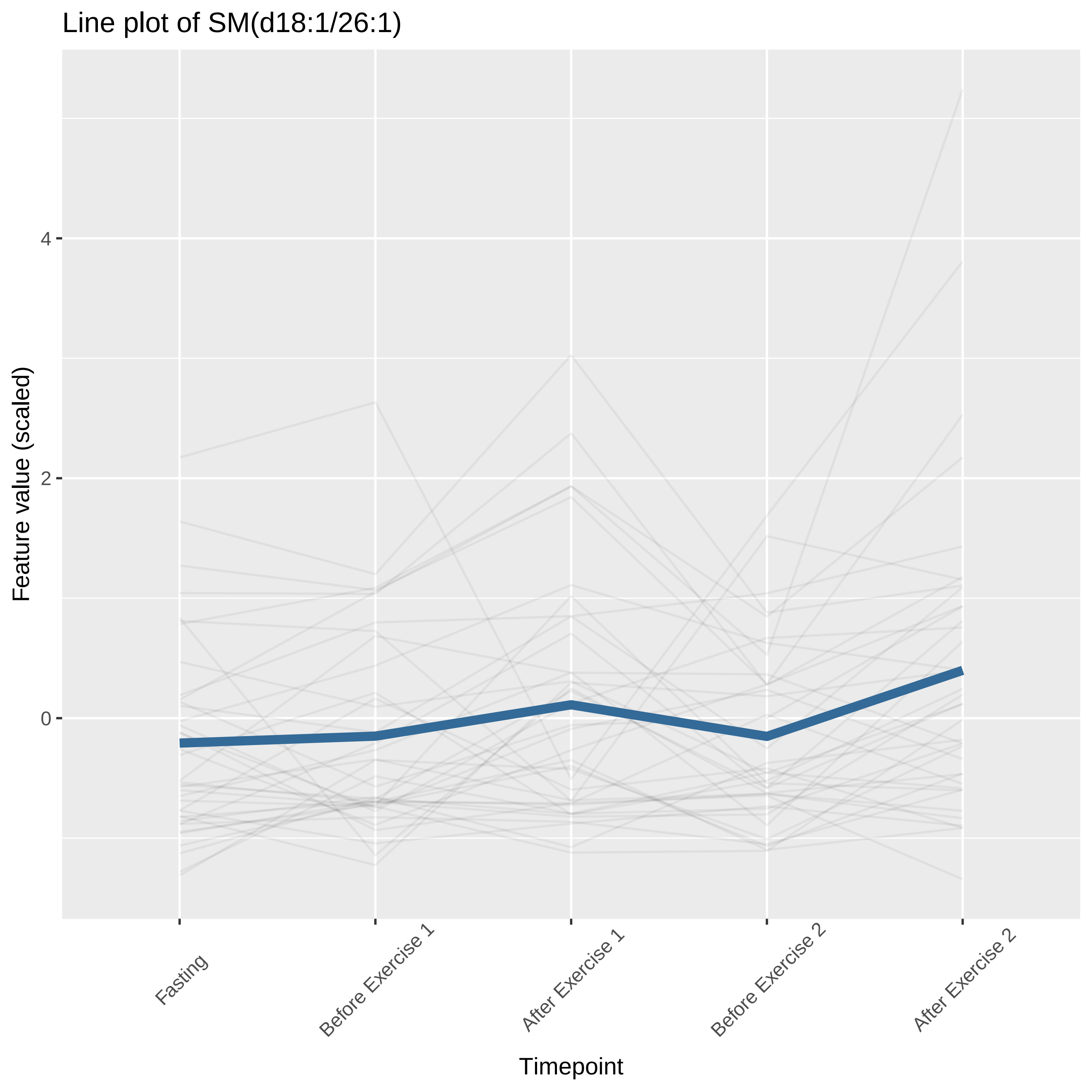

Line plot of SM(d18:1/26:1)
Feature value (scaled)
4
2
0
Fasting
Before Exercise 1
After Exercise 1
Before Exercise 2
After Exercise 2
Timepoint

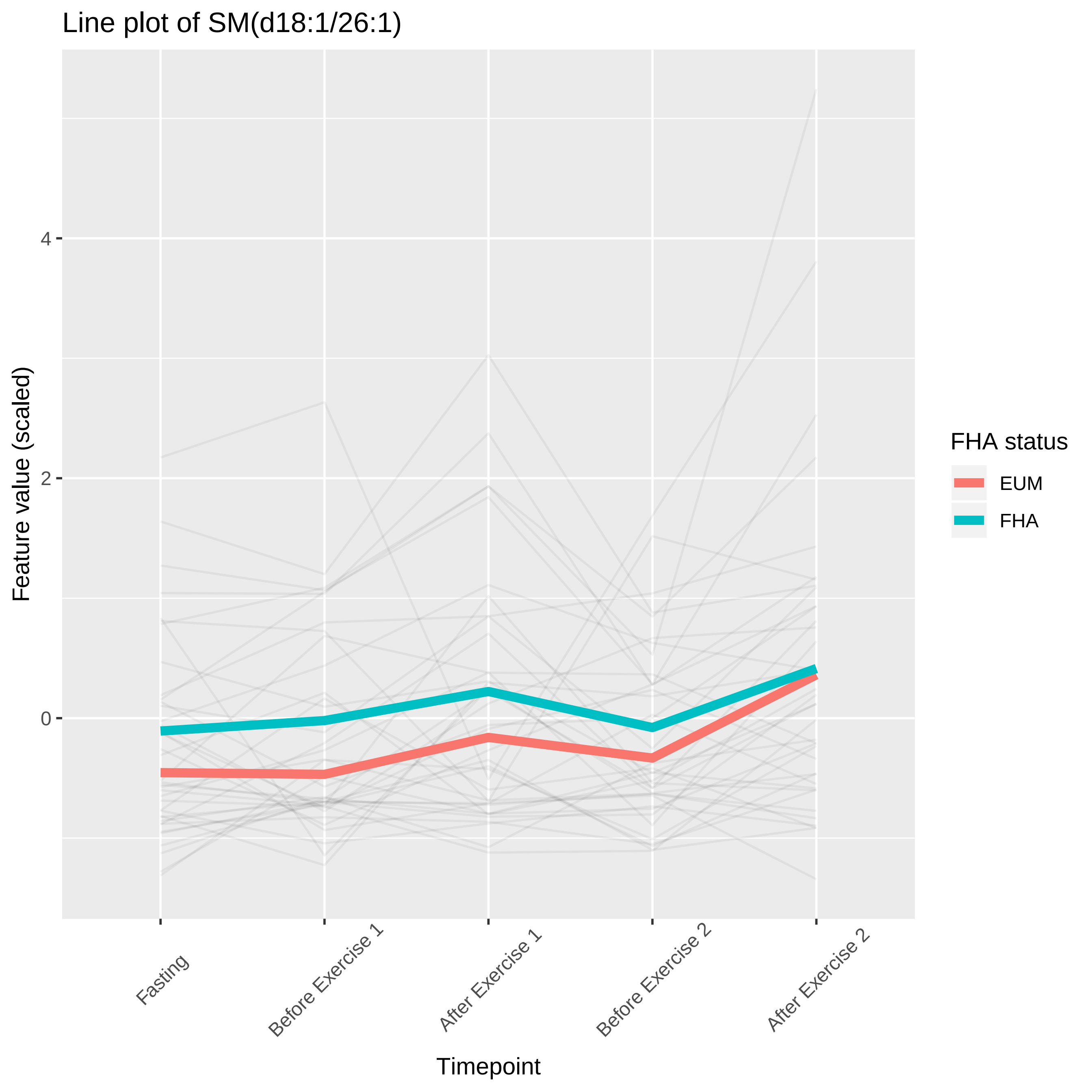

Line plot of SM(d18:1/26:1)
Feature value (scaled)
4
2
0
Fasting
Before Exercise 1
After Exercise 1
Before Exercise 2
After Exercise 2
Timepoint
FHA status
EUM
FHA

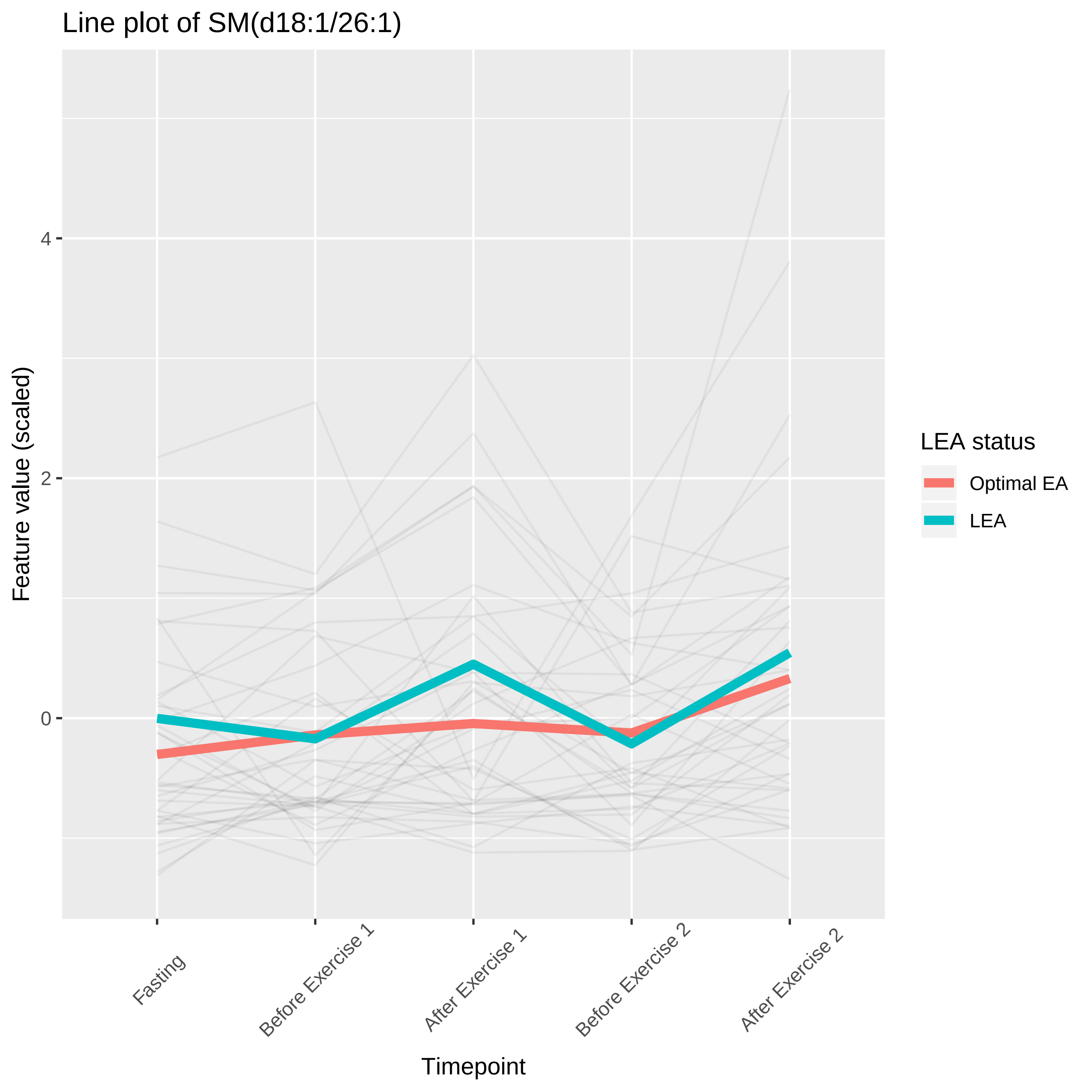

Line plot of SM(d18:1/26:1)
Feature value (scaled)
4
2
0
Fasting
Before Exercise 1
After Exercise 1
Before Exercise 2
After Exercise 2
Timepoint
LEA status
Optimal EA
LEA

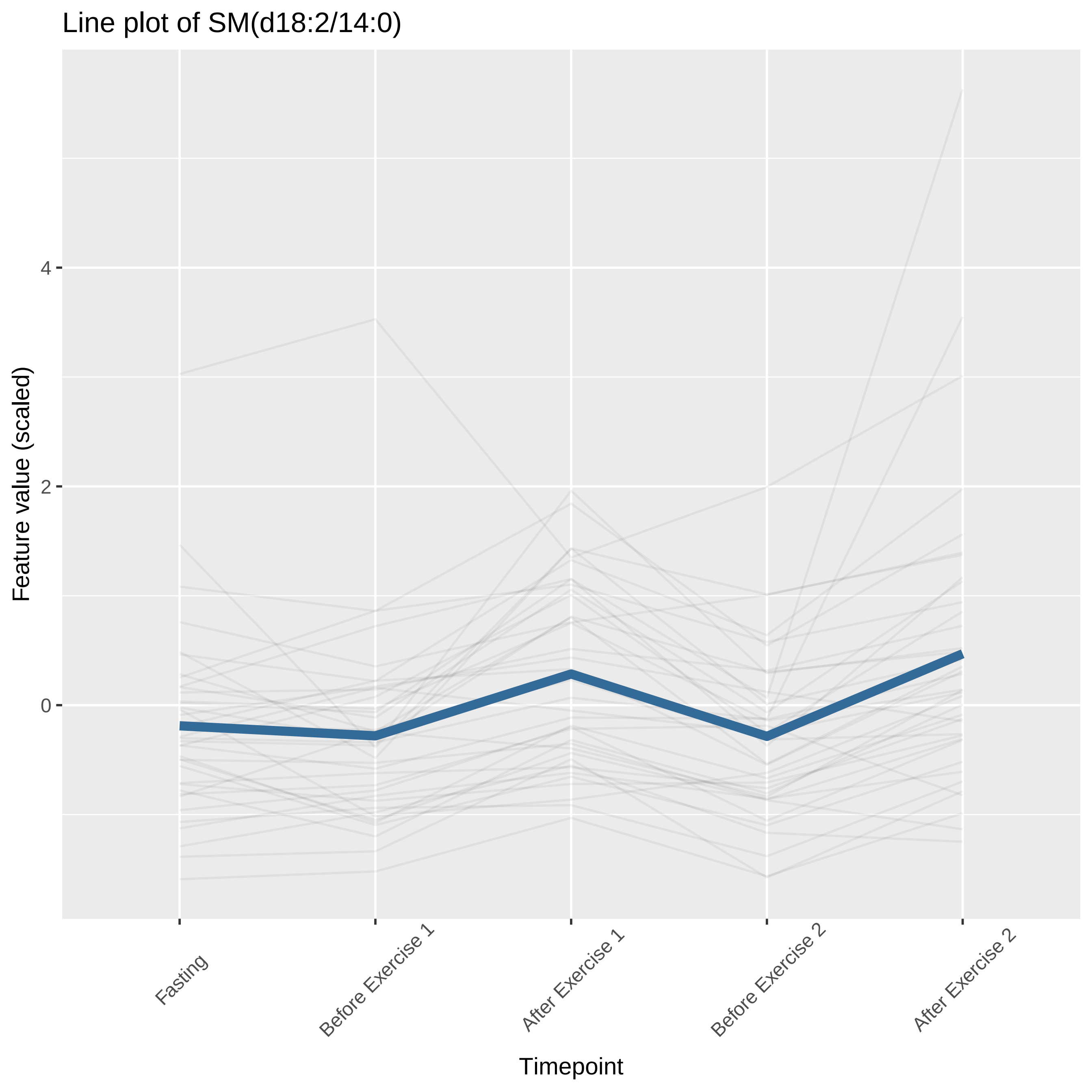
Line plot of SM(d18:2/14:0)
Feature value (scaled)
4
2
0
Fasting
Before Exercise 1
After Exercise 1
Before Exercise 2
After Exercise 2
Timepoint

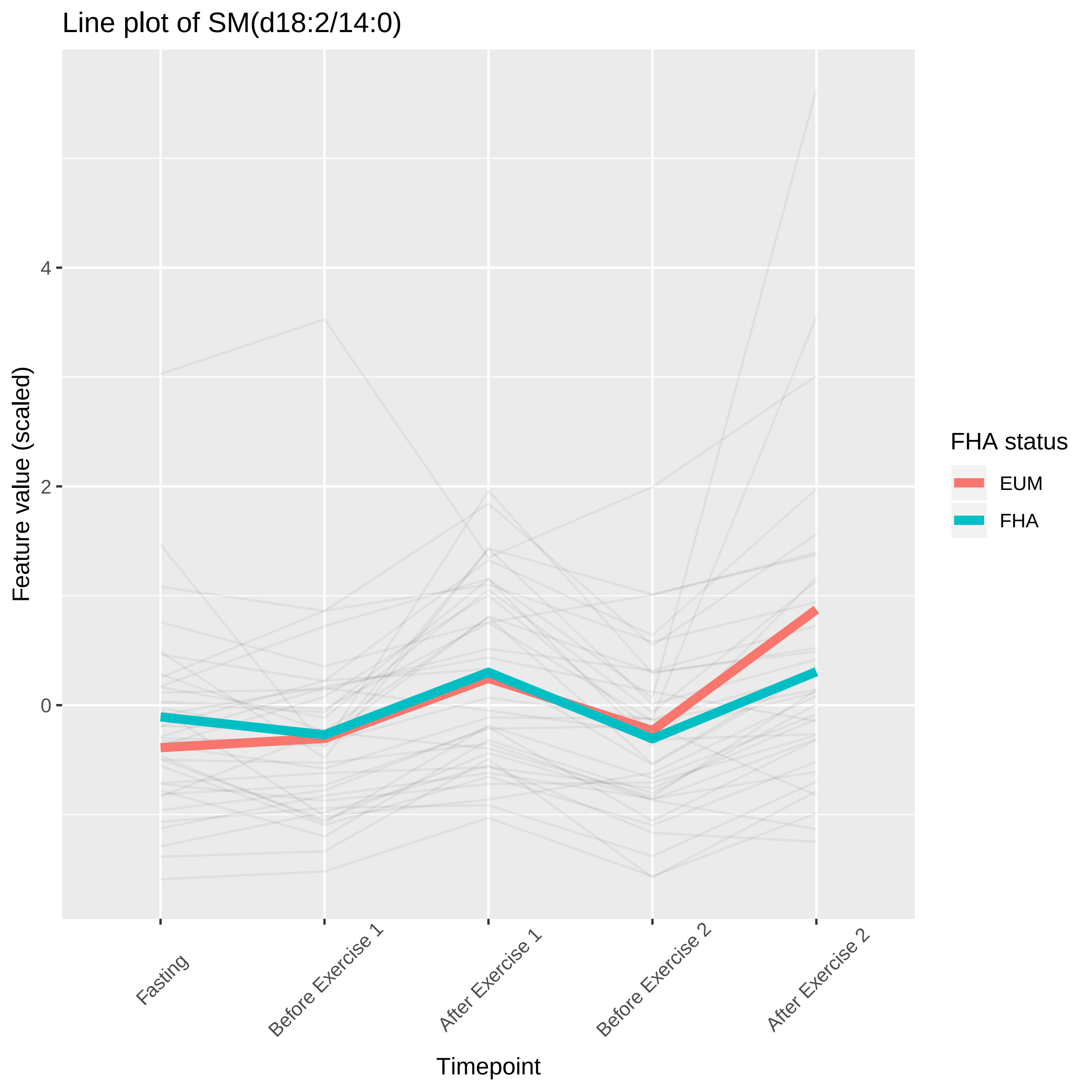

Line plot of SM(d18:2/14:0)
4
2
0
Feature value (scaled)
Fasting
Before Exercise 1
After Exercise 1
Before Exercise 2
After Exercise 2
Timepoint
FHA status
EUM
FHA

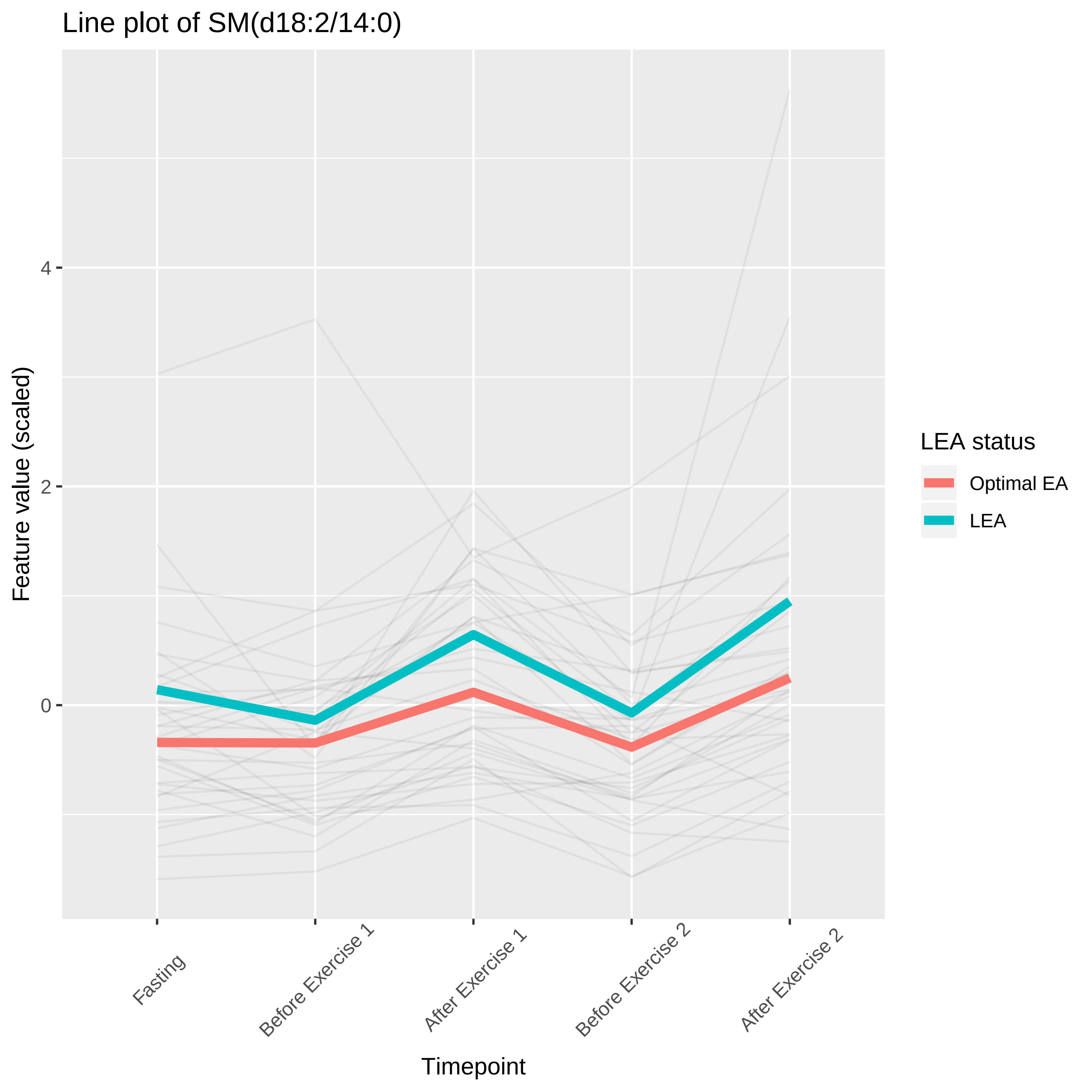
Line plot of SM(d18:2/14:0)
Feature value (scaled)
4
2
0
Fasting
Before Exercise 1
After Exercise 1
Before Exercise 2
After Exercise 2
Timepoint
LEA status
Optimal EA
LEA

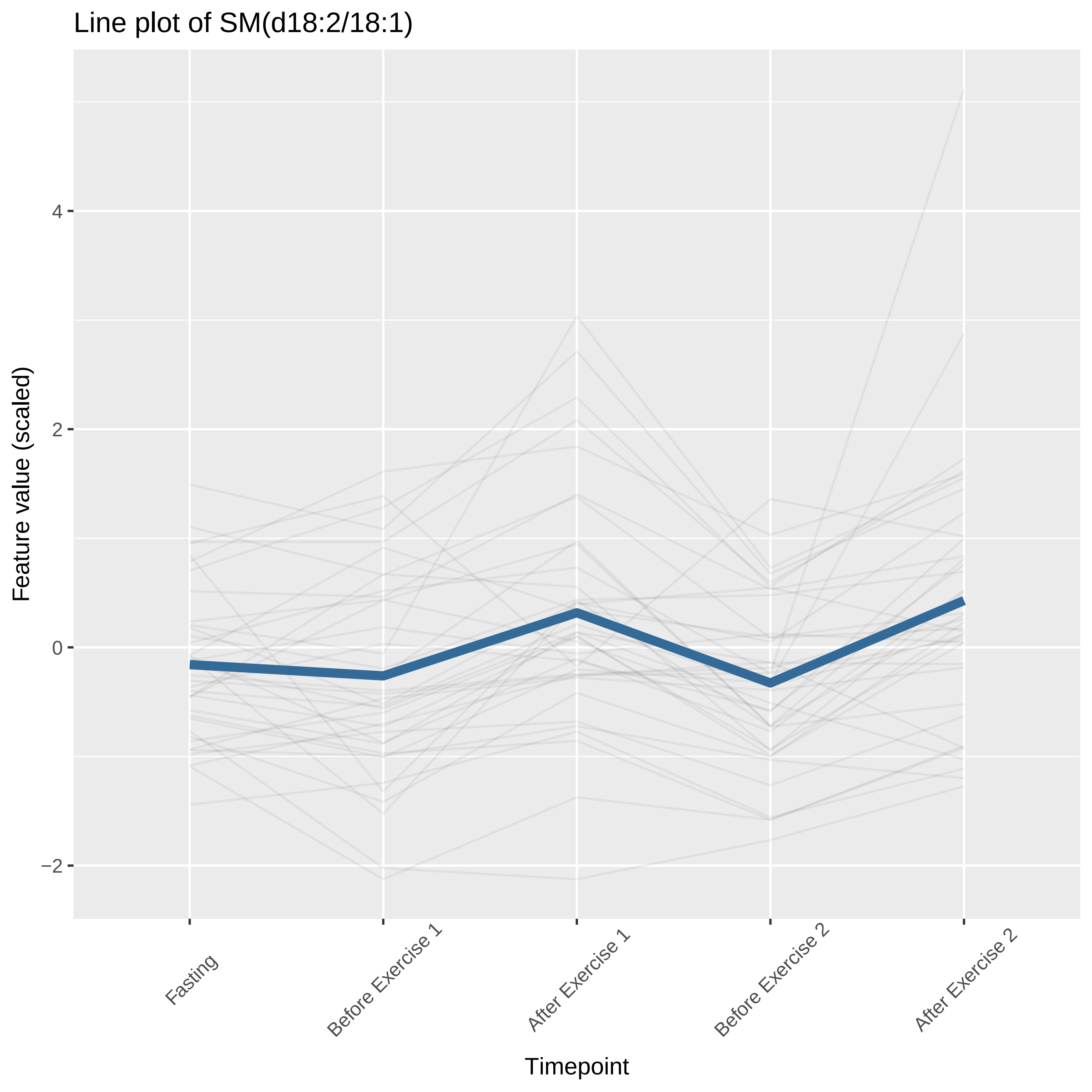

Line plot of SM(d18:2/18:1)
Feature value (scaled)
4
2
0
−2
Fasting
Before Exercise 1
After Exercise 1
Before Exercise 2
After Exercise 2
Timepoint

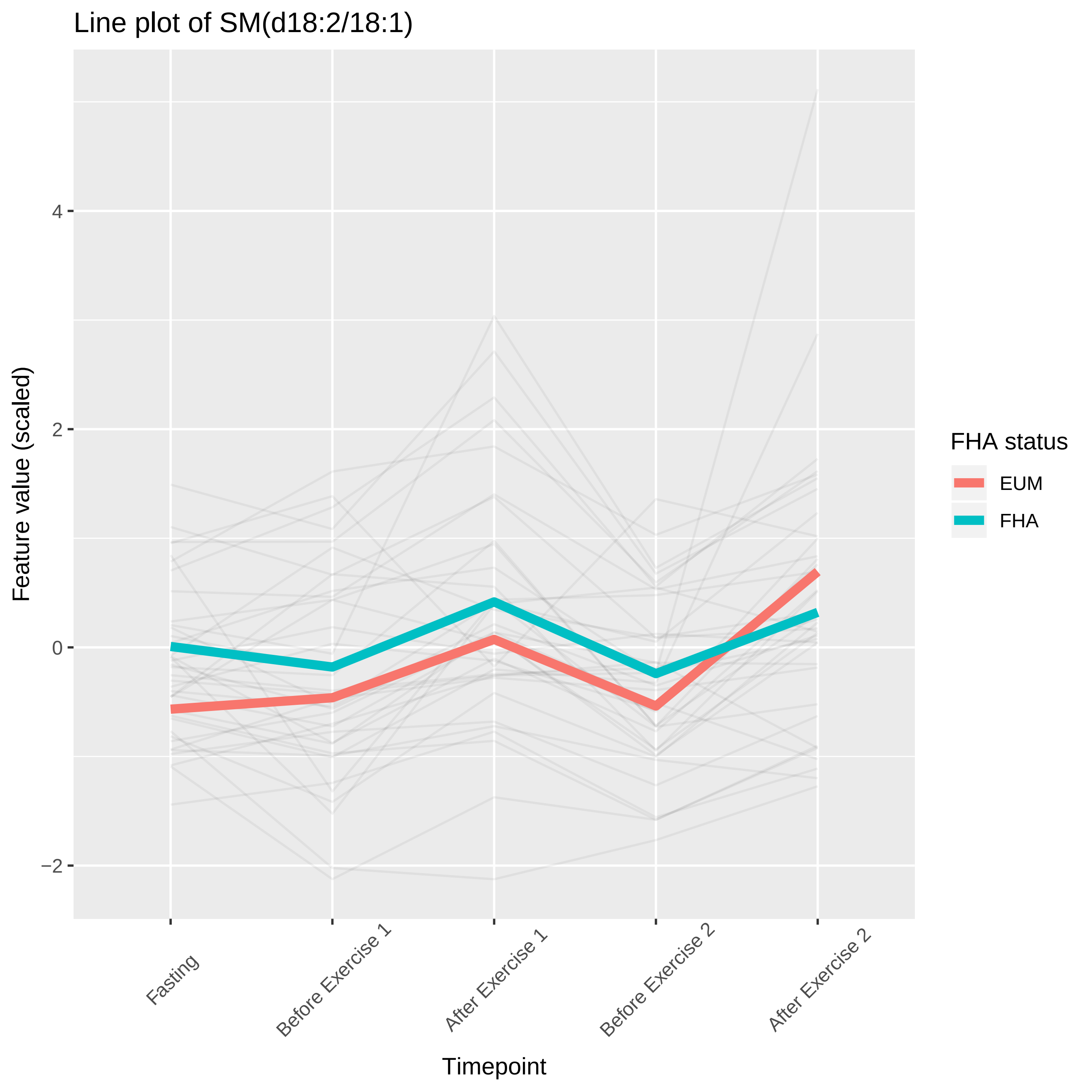

Line plot of SM(d18:2/18:1)
Feature value (scaled)
4
2
0
−2
Fasting
Before Exercise 1
After Exercise 1
Before Exercise 2
After Exercise 2
Timepoint
FHA status
EUM
FHA

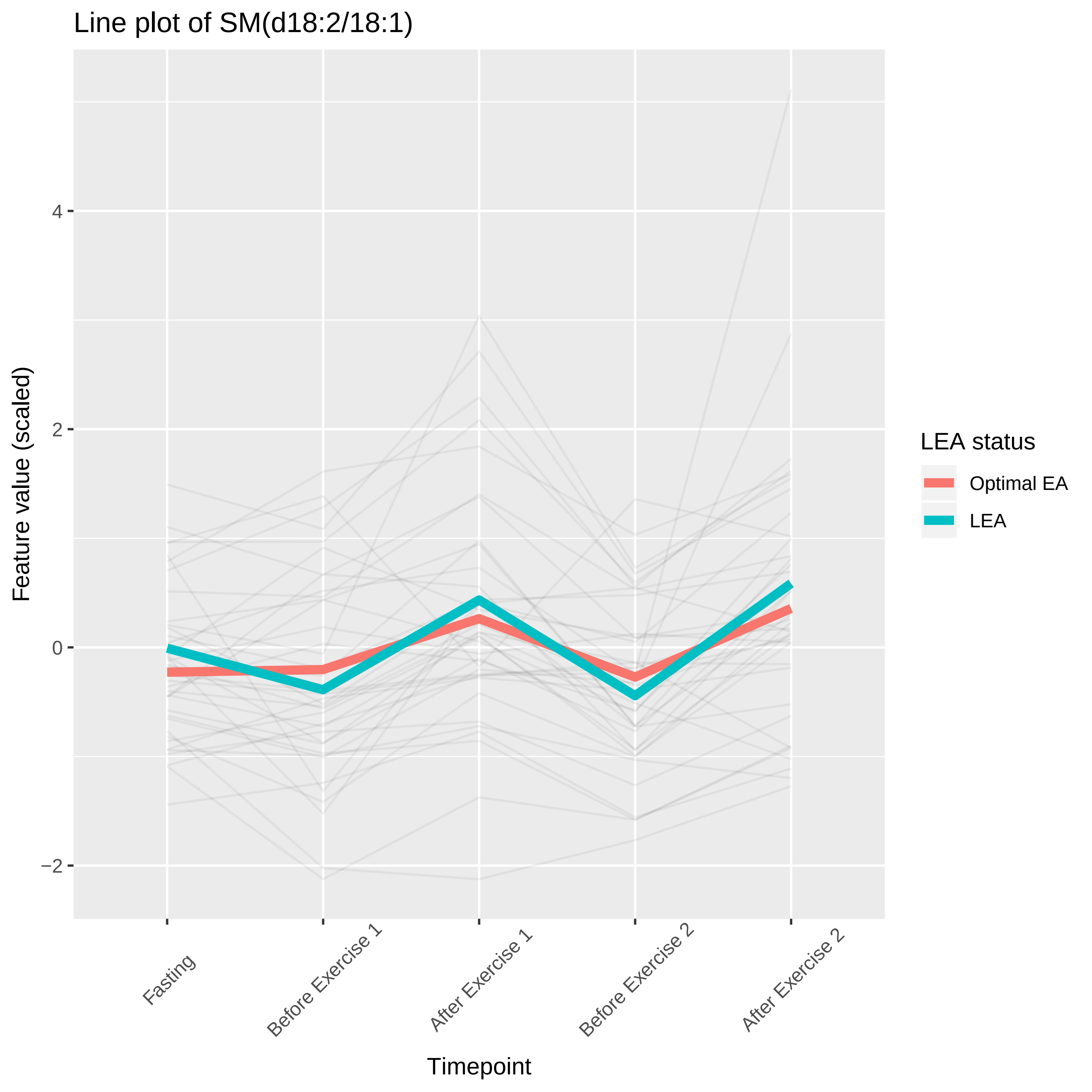

Line plot of SM(d18:2/18:1)
Feature value (scaled)
4
2
0
−2
Fasting
Before Exercise 1
After Exercise 1
Before Exercise 2
After Exercise 2
Timepoint
LEA status
Optimal EA
LEA

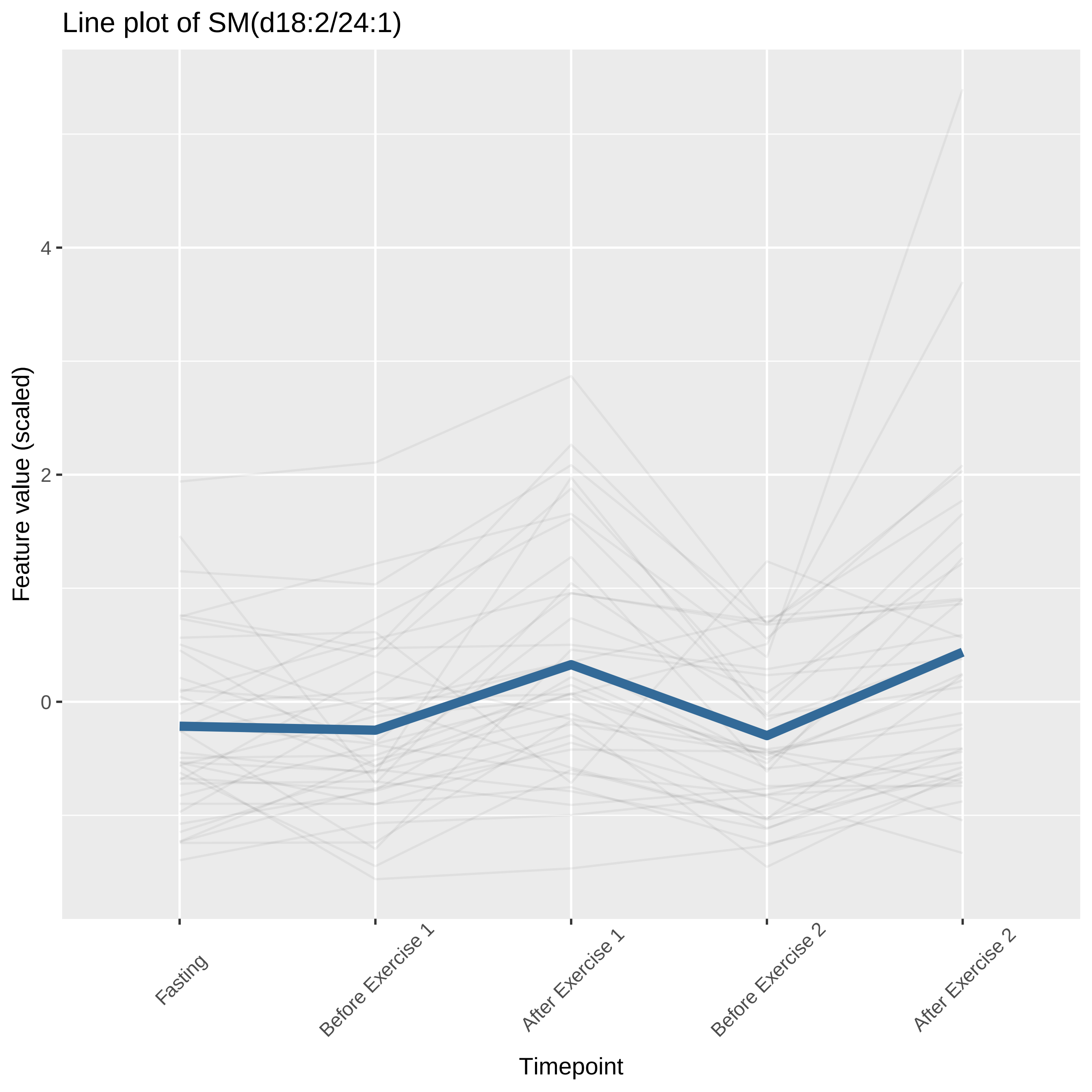

Line plot of SM(d18:2/24:1)
Feature value (scaled)
4
2
0
Fasting
Before Exercise 1
After Exercise 1
Before Exercise 2
After Exercise 2
Timepoint

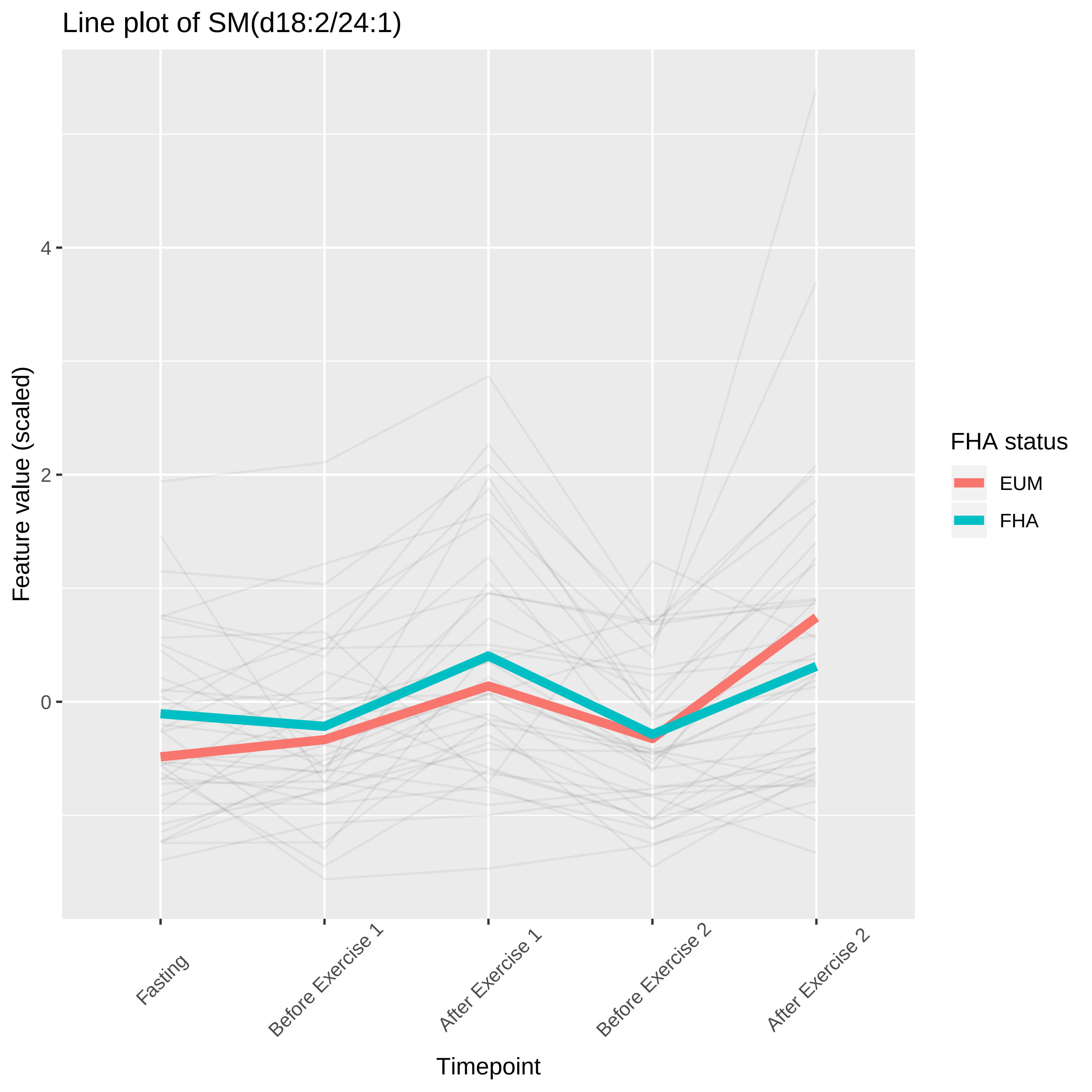

Line plot of SM(d18:2/24:1)
Feature value (scaled)
4
2
0
Fasting
Before Exercise 1
After Exercise 1
Before Exercise 2
After Exercise 2
Timepoint
FHA status
EUM
FHA

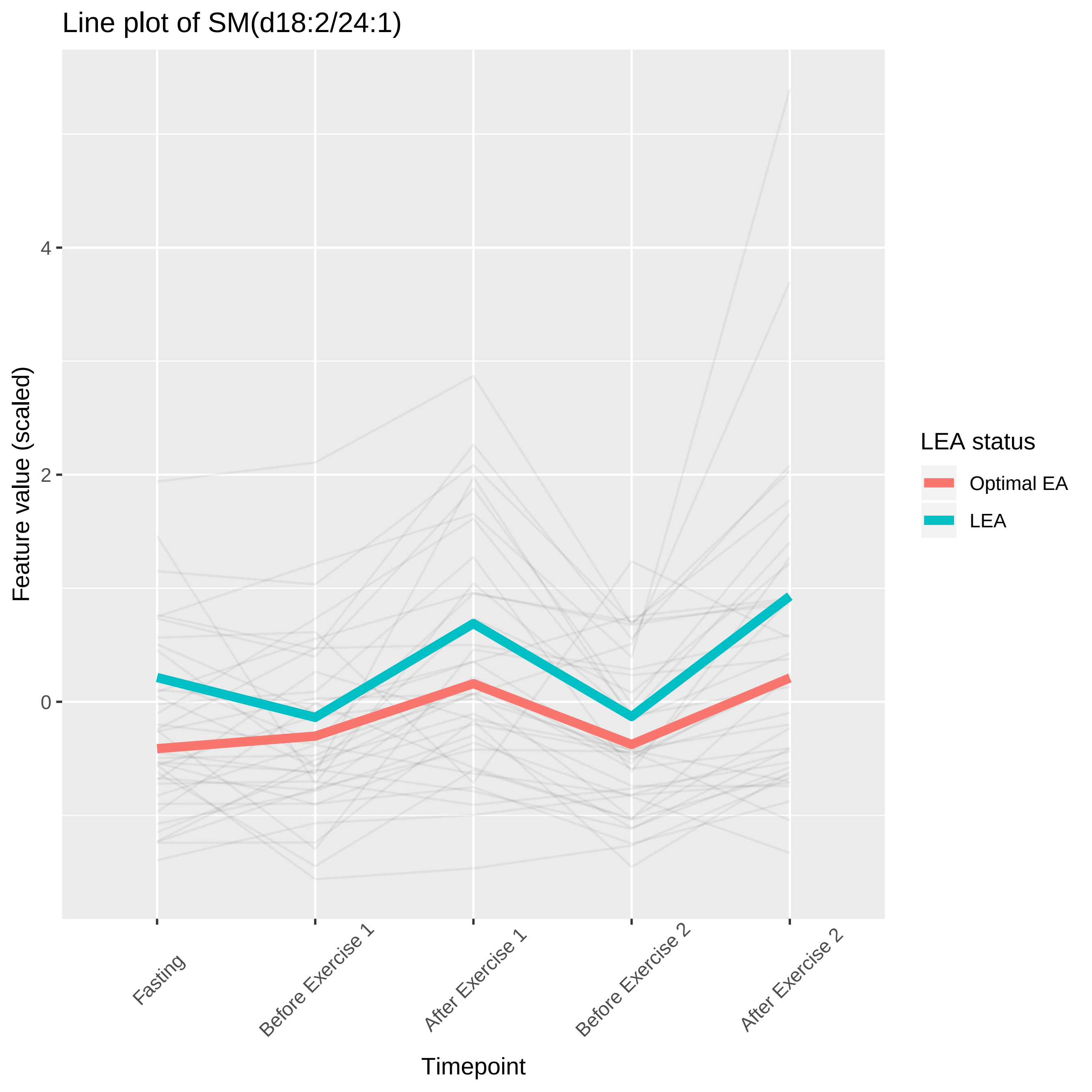

Line plot of SM(d18:2/24:1)
Feature value (scaled)
4
2
0
Fasting
Before Exercise 1
After Exercise 1
Before Exercise 2
After Exercise 2
Timepoint
LEA status
Optimal EA
LEA

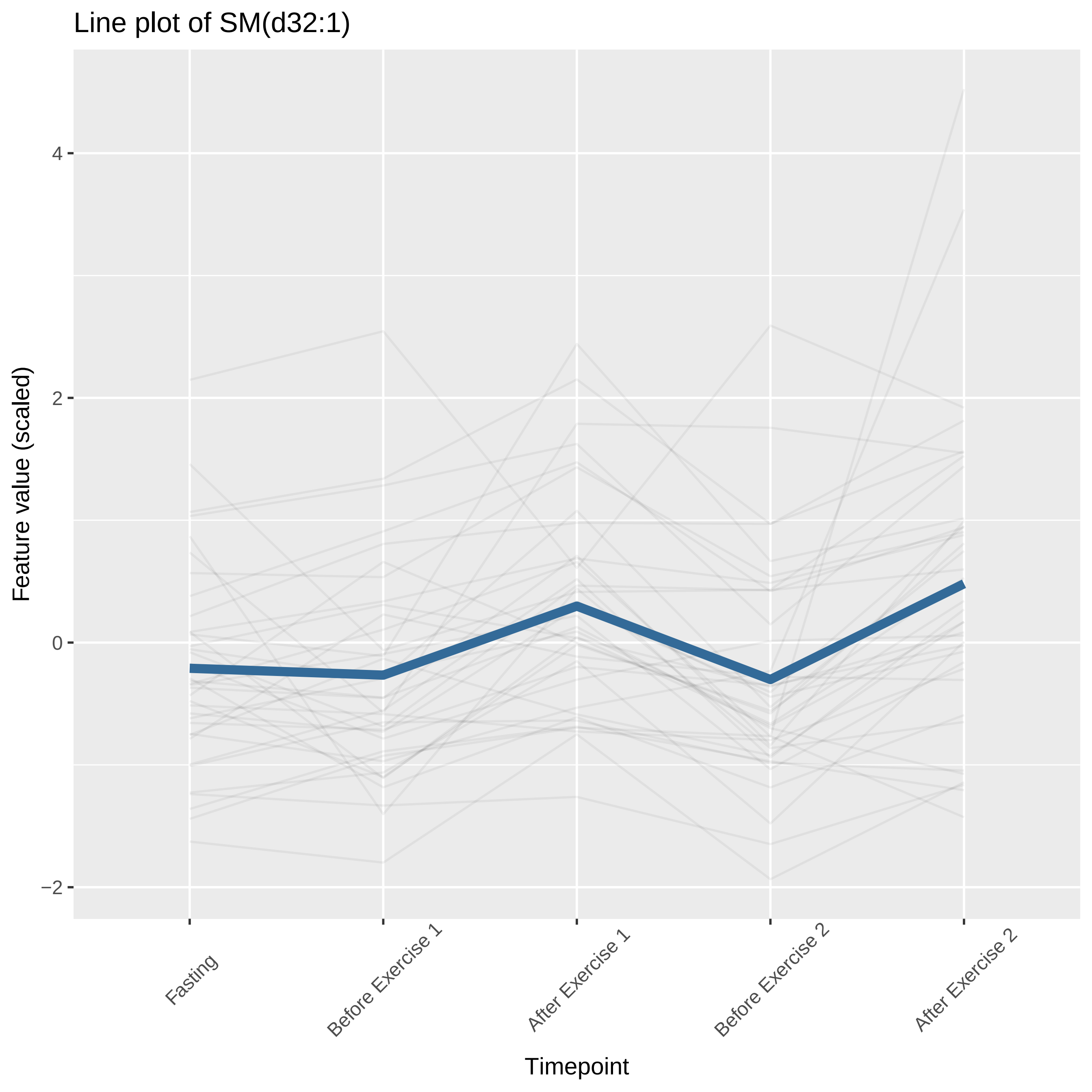

Line plot of SM(d32:1)
Feature value (scaled)
4
2
0
−2
Fasting
Before Exercise 1
After Exercise 1
Before Exercise 2
After Exercise 2
Timepoint

Line plot of SM(d32:1)

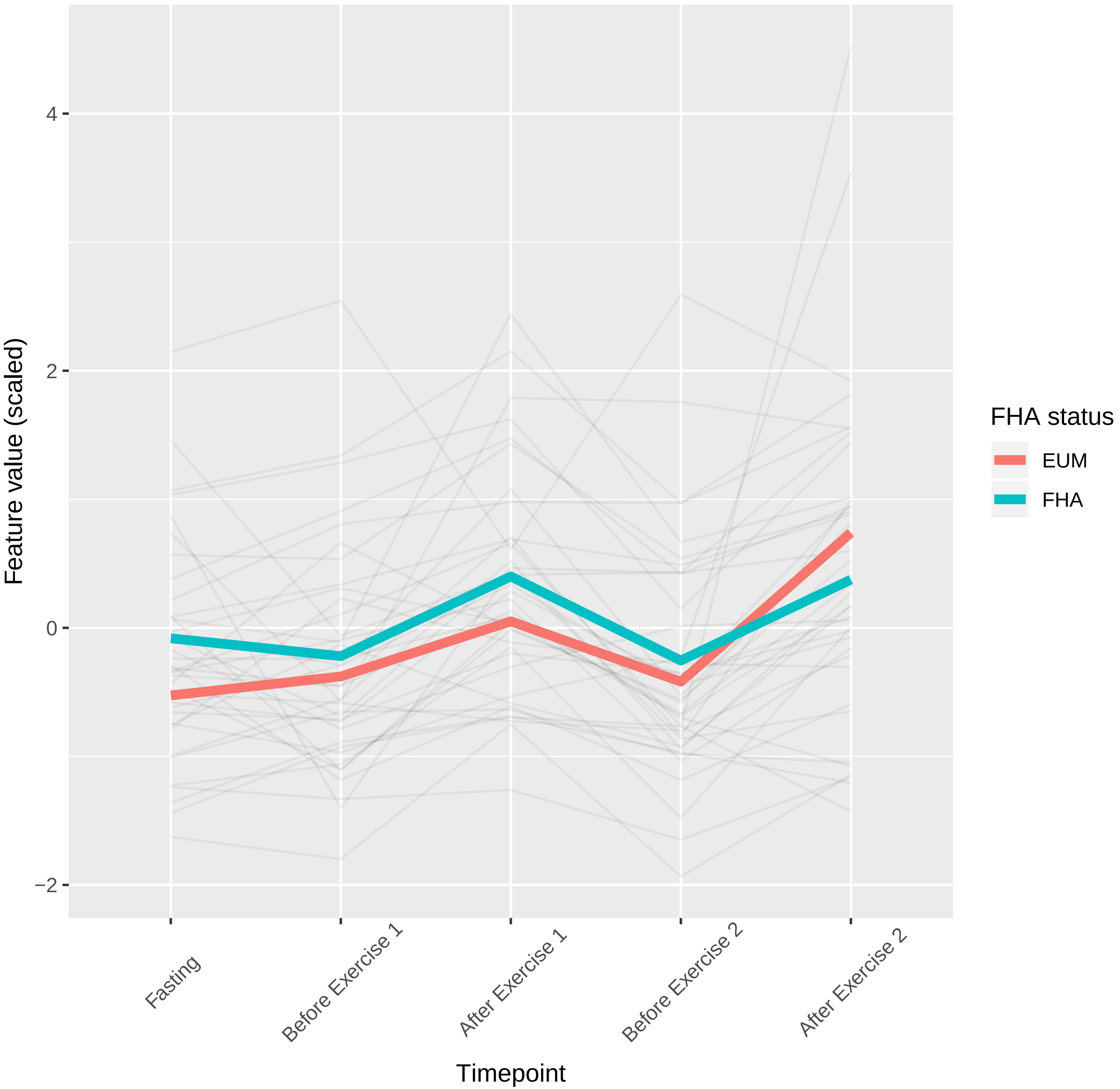

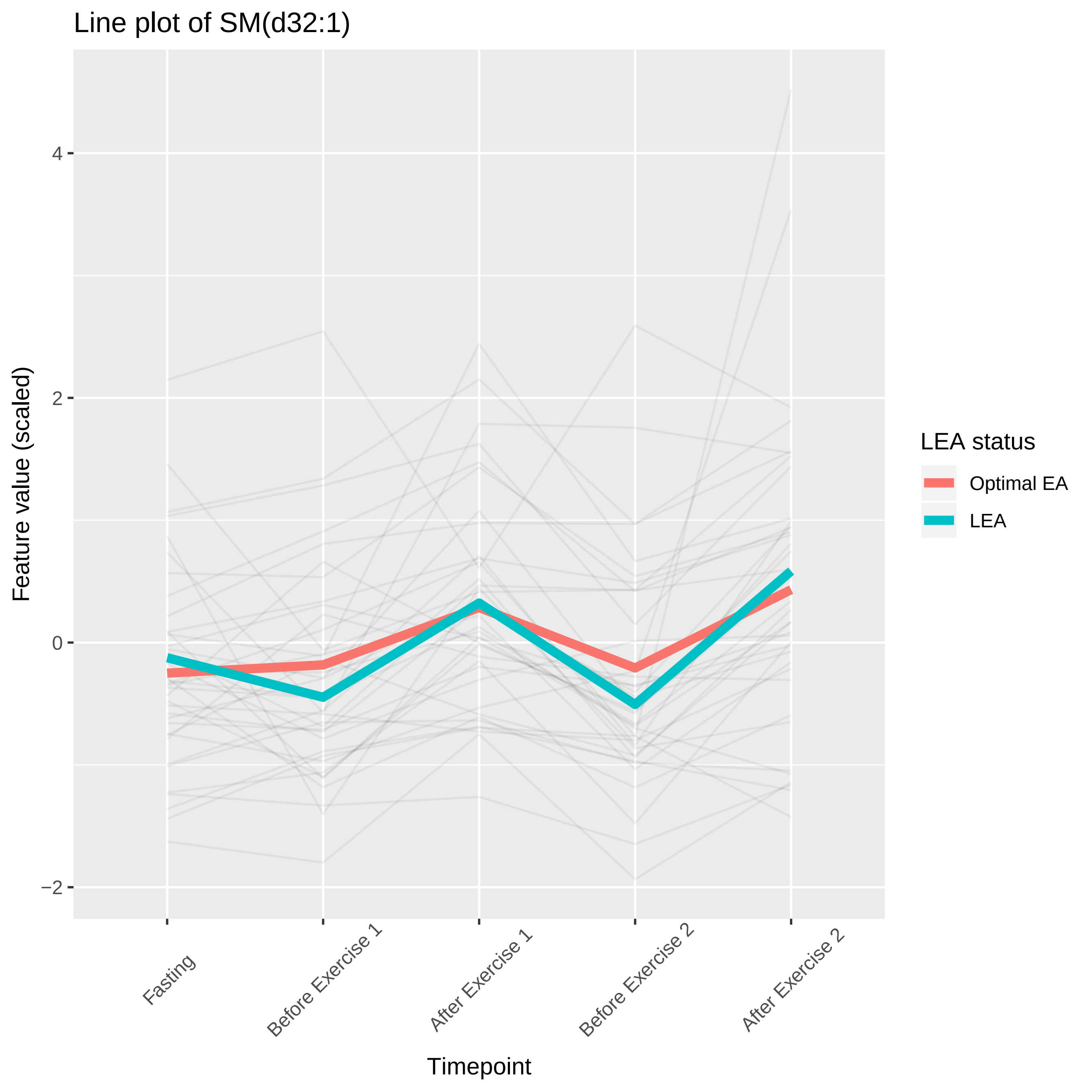

Line plot of SM(d32:1)
Feature value (scaled)
4
2
0
−2
Fasting
Before Exercise 1
After Exercise 1
Before Exercise 2
After Exercise 2
Timepoint
LEA status
Optimal EA
LEA

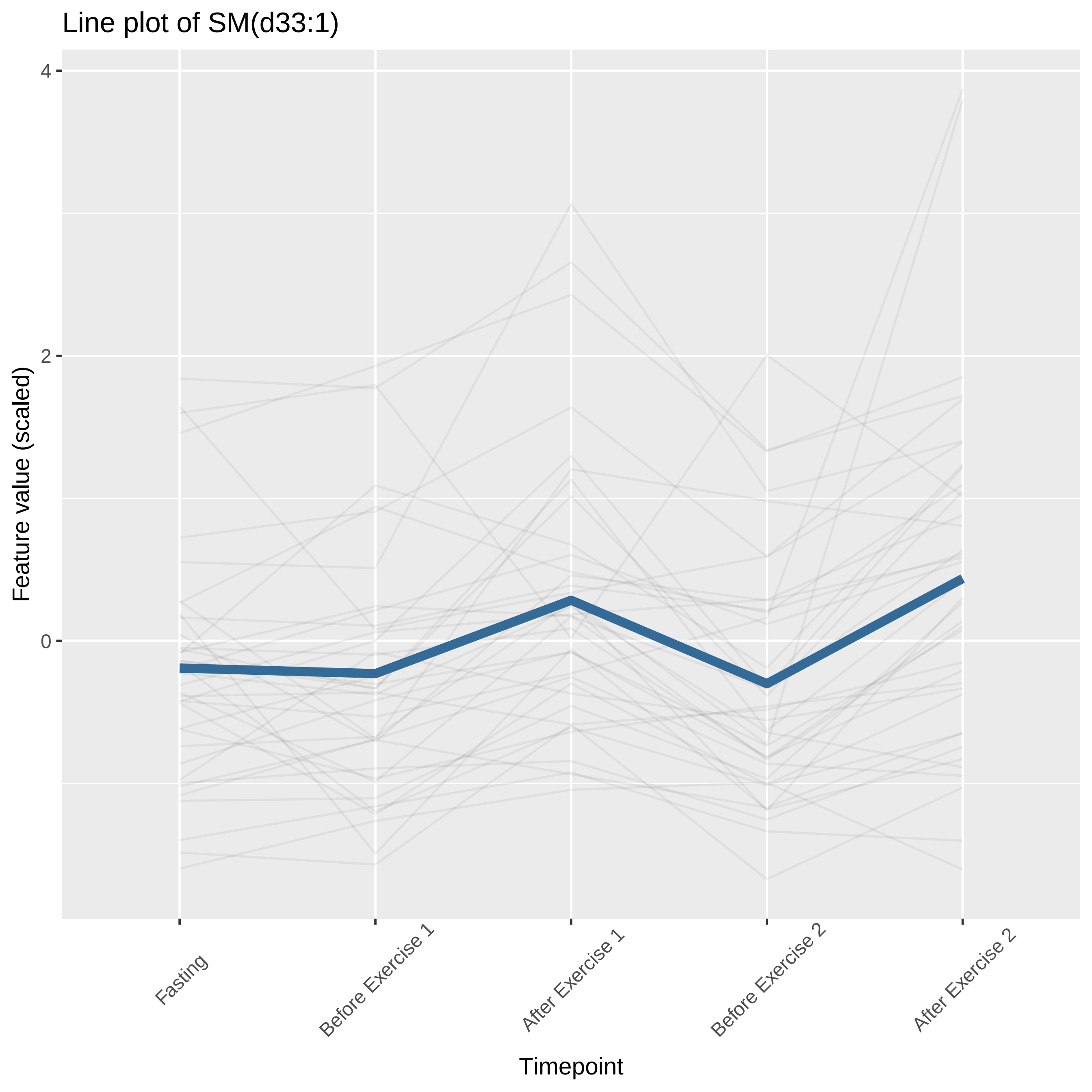

Line plot of SM(d33:1)
Feature value (scaled)
4
2
0
Fasting
Before Exercise 1
After Exercise 1
Before Exercise 2
After Exercise 2
Timepoint

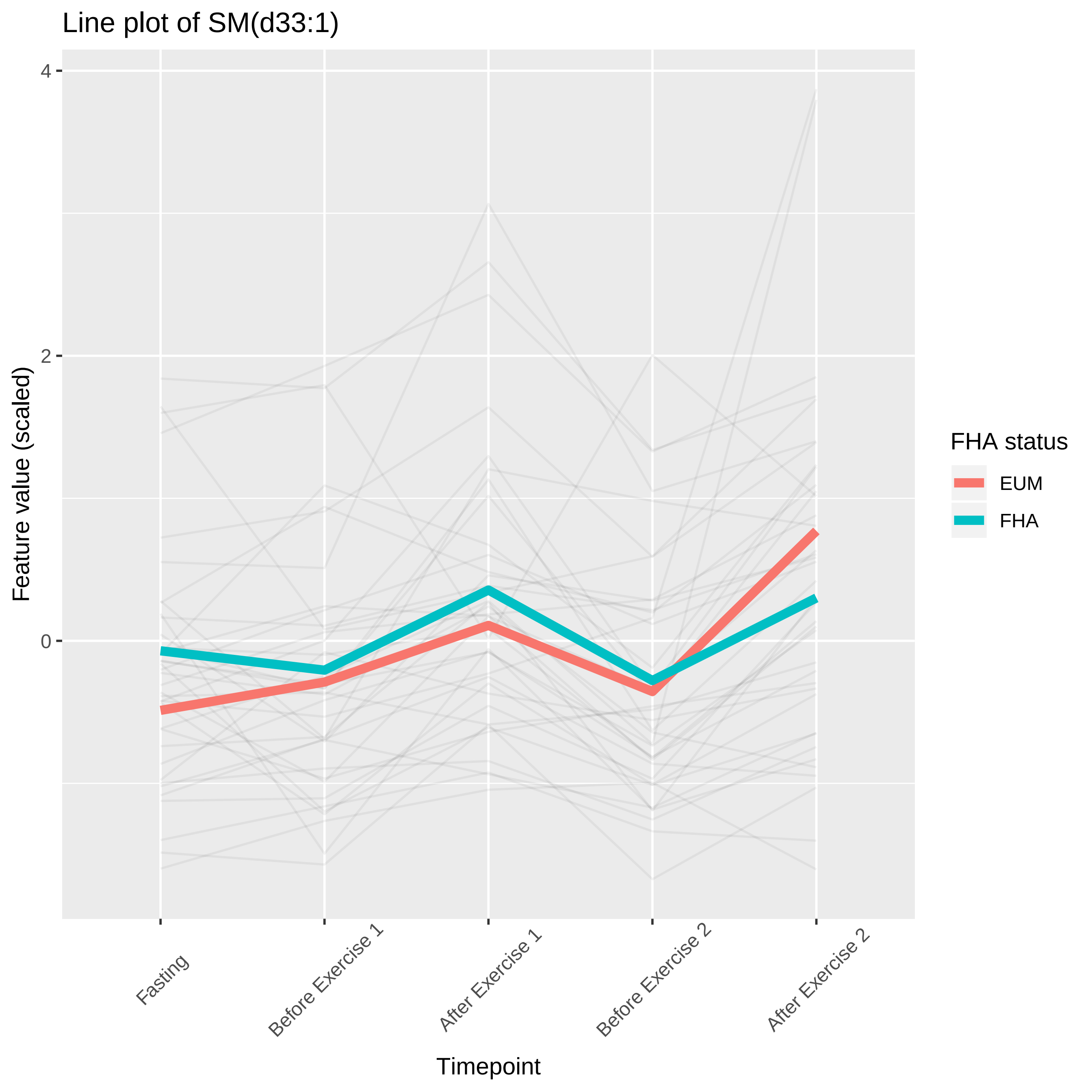
Line plot of SM(d33:1)
Feature value (scaled)
4
2
0
Fasting
Before Exercise 1
After Exercise 1
Before Exercise 2
After Exercise 2
Timepoint
FHA status
EUM
FHA

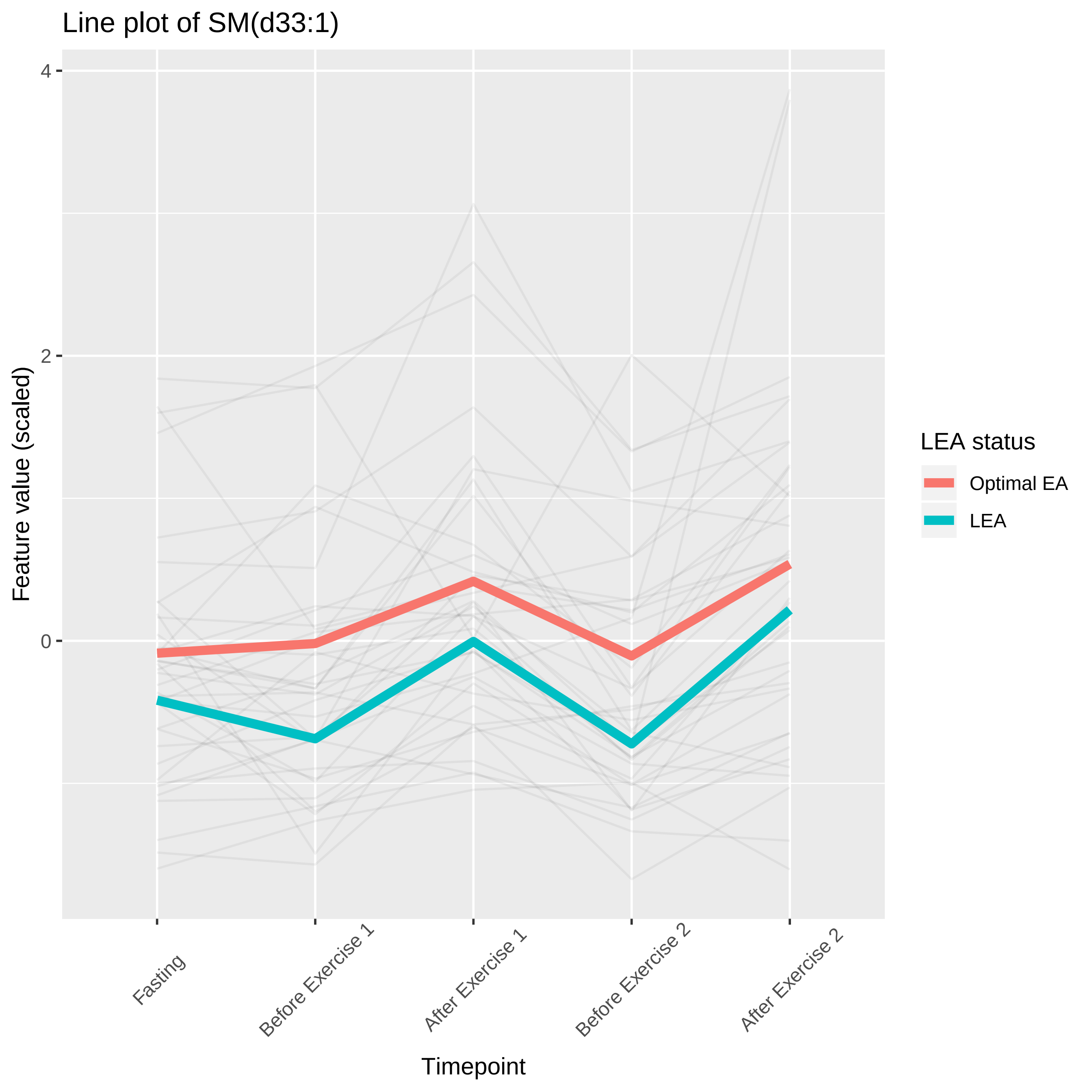
Line plot of SM(d33:1)
4
2
0
Feature value (scaled)
Fasting
Before Exercise 1
After Exercise 1
Before Exercise 2
After Exercise 2
Timepoint
LEA status
Optimal EA
LEA

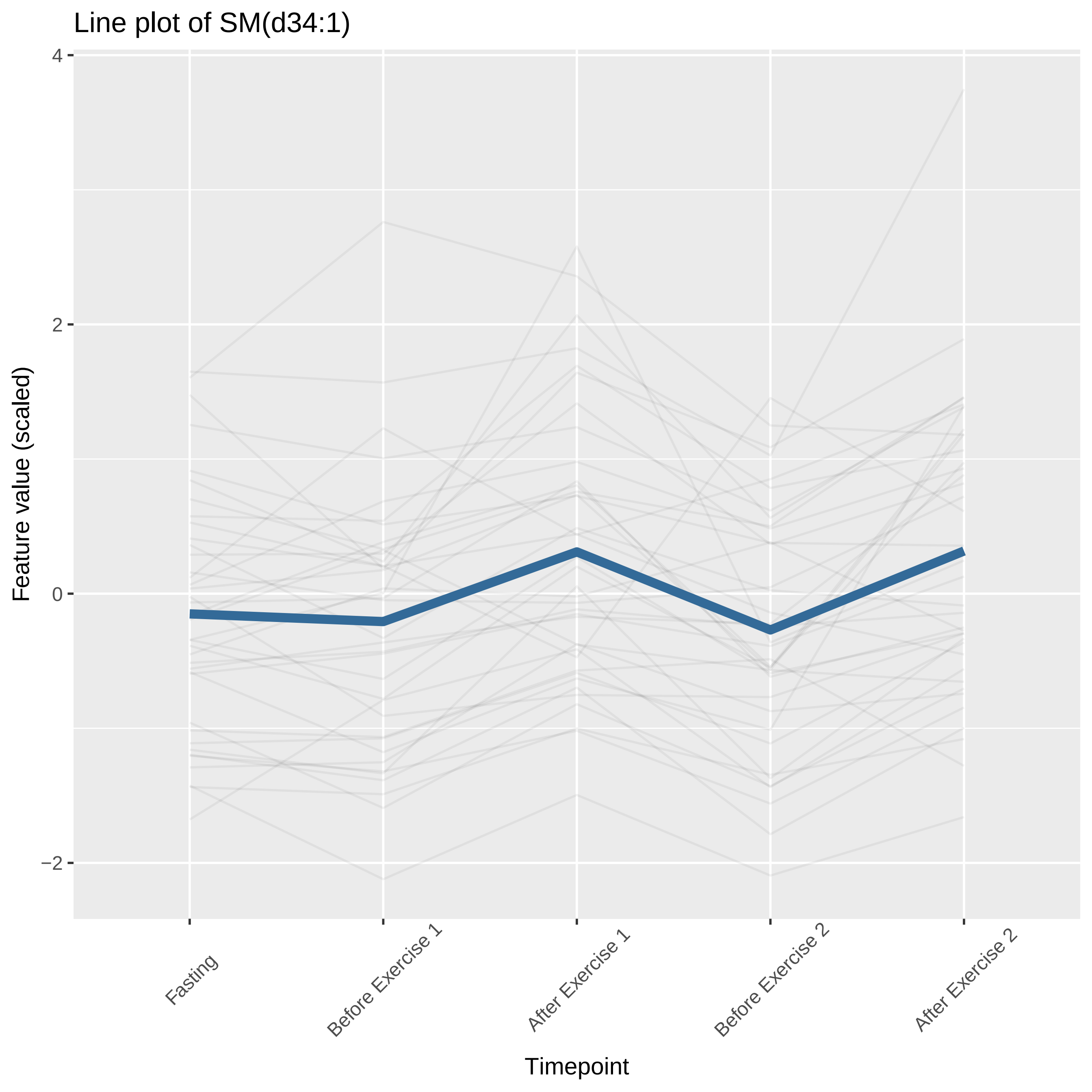
Line plot of SM(d34:1)
Feature value (scaled)
4
2
0
−2
Fasting
Before Exercise 1
After Exercise 1
Before Exercise 2
After Exercise 2
Timepoint

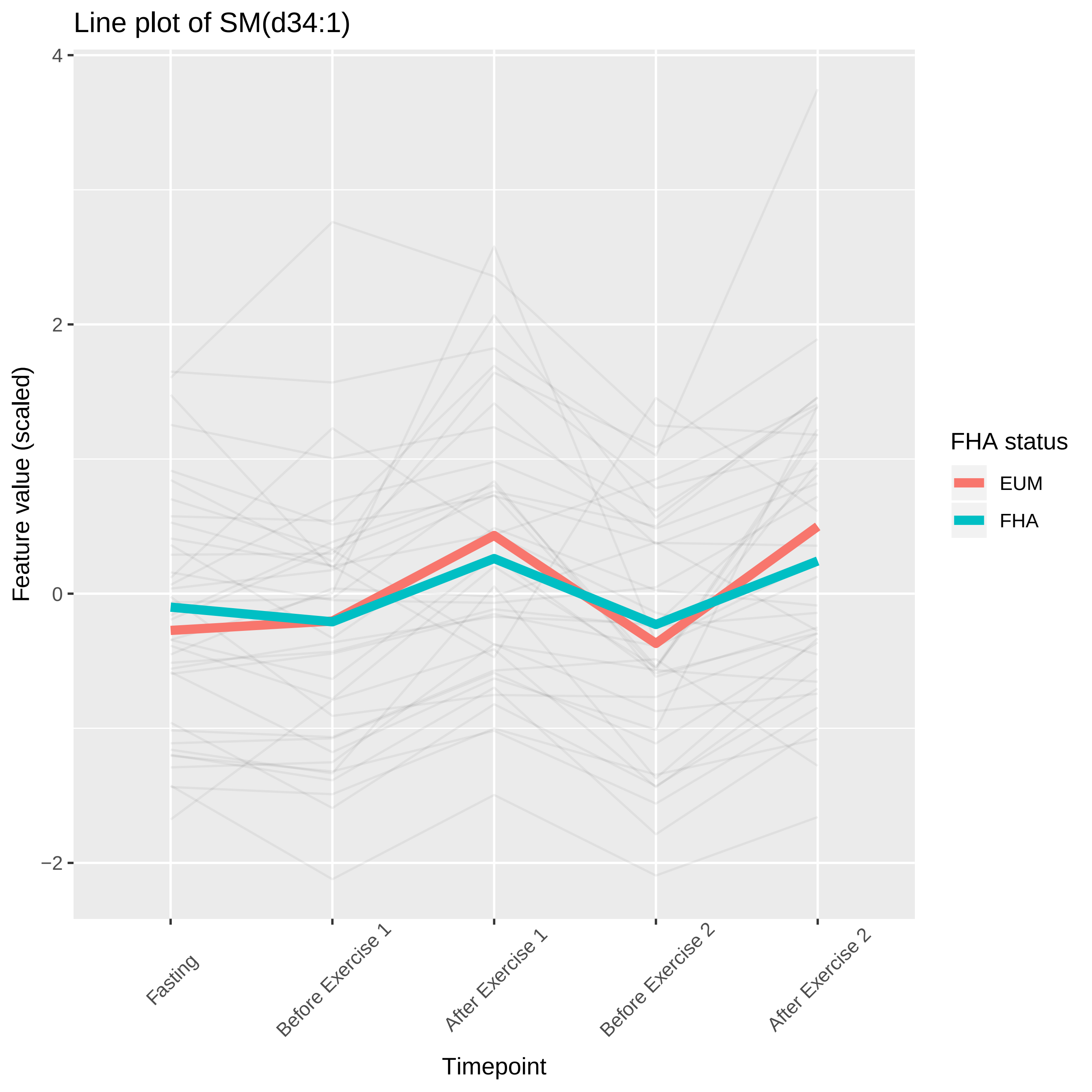

Line plot of SM(d34:1)
Feature value (scaled)
4
2
0
−2
Fasting
Before Exercise 1
After Exercise 1
Before Exercise 2
After Exercise 2
Timepoint
FHA status
EUM
FHA

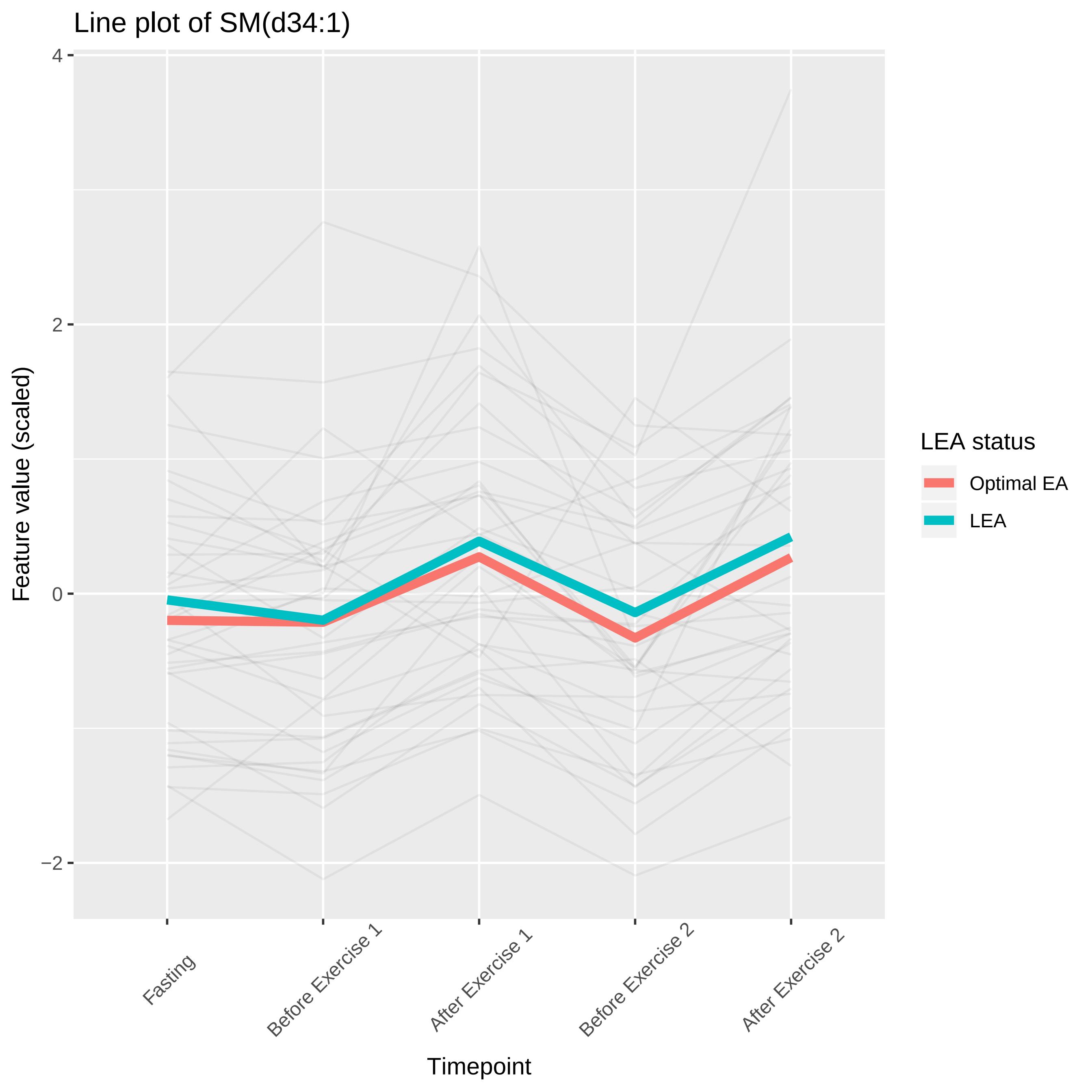

Line plot of SM(d34:1)
Feature value (scaled)
4
2
0
−2
Fasting
Before Exercise 1
After Exercise 1
Before Exercise 2
After Exercise 2
Timepoint
LEA status
Optimal EA
LEA

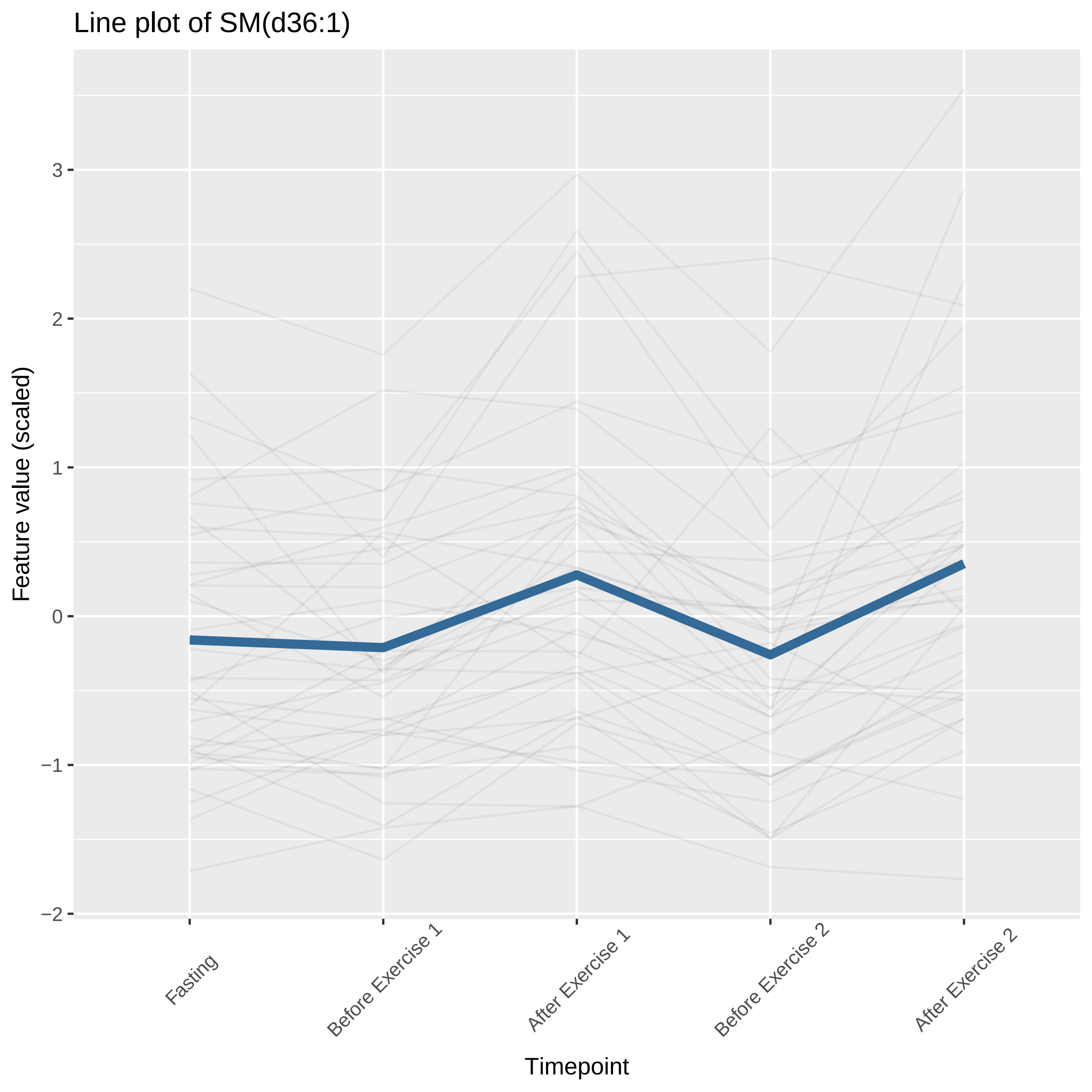

Line plot of SM(d36:1)
Feature value (scaled)
3
2
1
0
−1
−2
Fasting
Before Exercise 1
After Exercise 1
Before Exercise 2
After Exercise 2
Timepoint

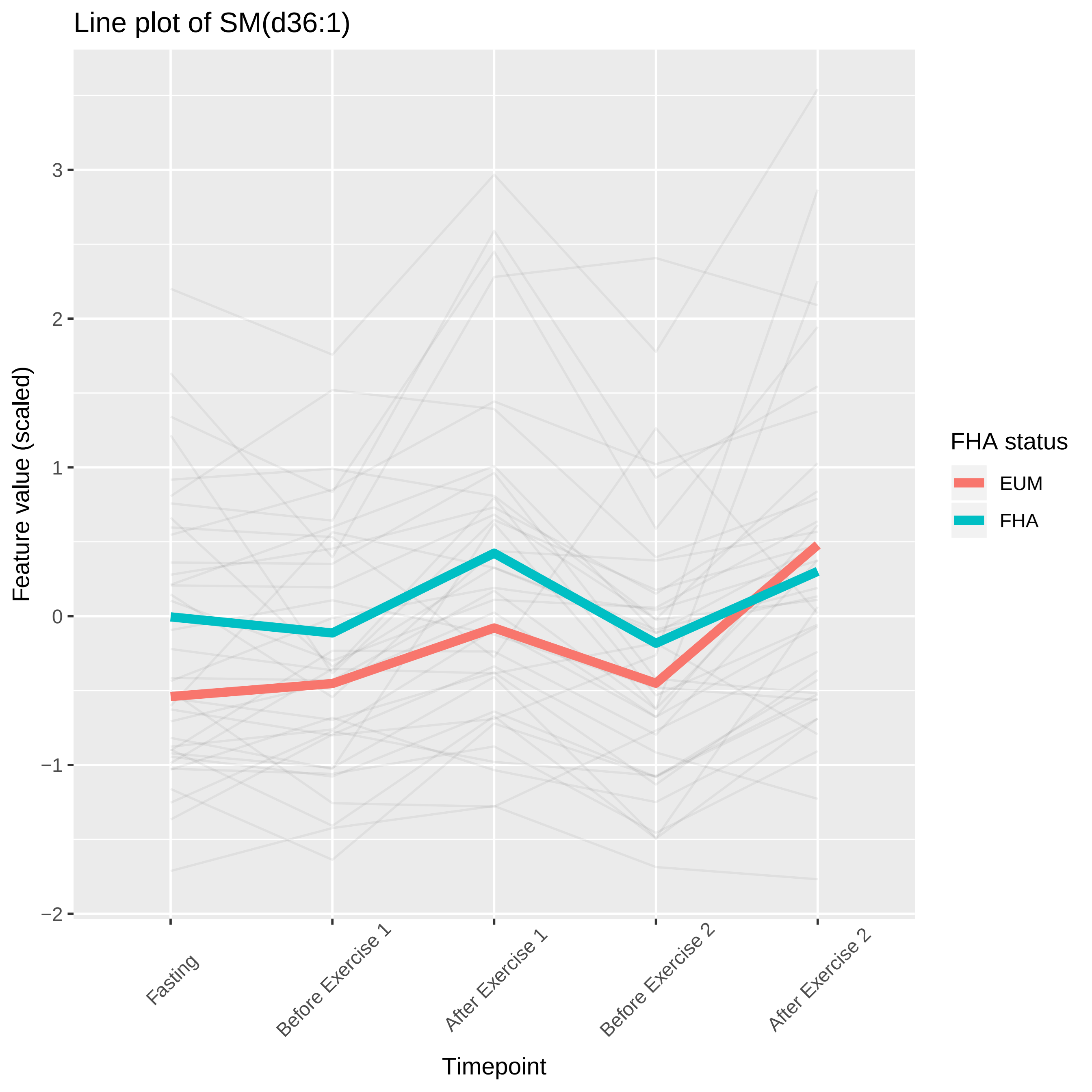

Line plot of SM(d36:1)
Feature value (scaled)
3
2
1
0
−1
−2
Fasting
Before Exercise 1
After Exercise 1
Before Exercise 2
After Exercise 2
Timepoint
FHA status
EUM
FHA

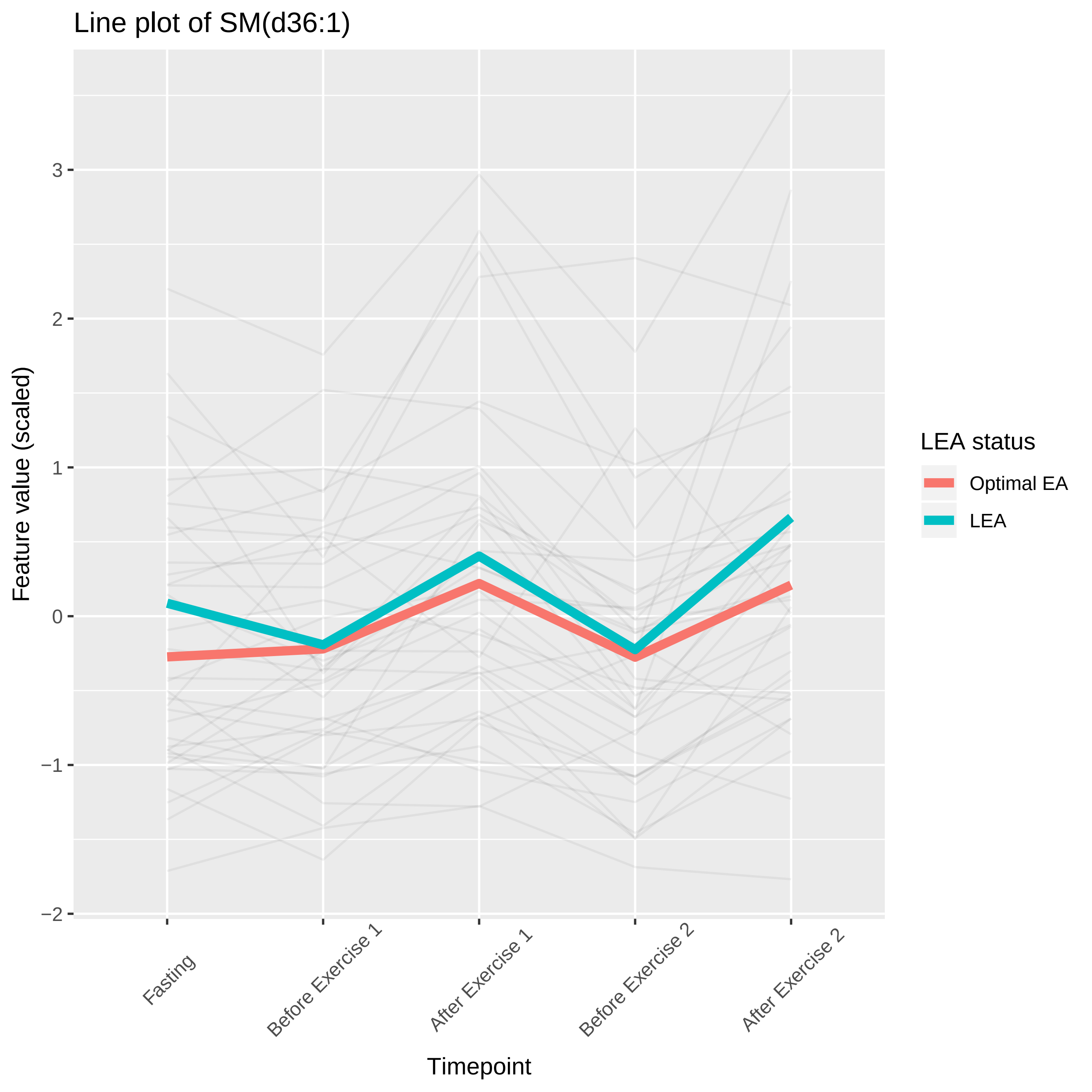
Line plot of SM(d36:1)
Feature value (scaled)
3
2
1
0
−1
−2
Fasting
Before Exercise 1
After Exercise 1
Before Exercise 2
After Exercise 2
Timepoint
LEA status
Optimal EA
LEA

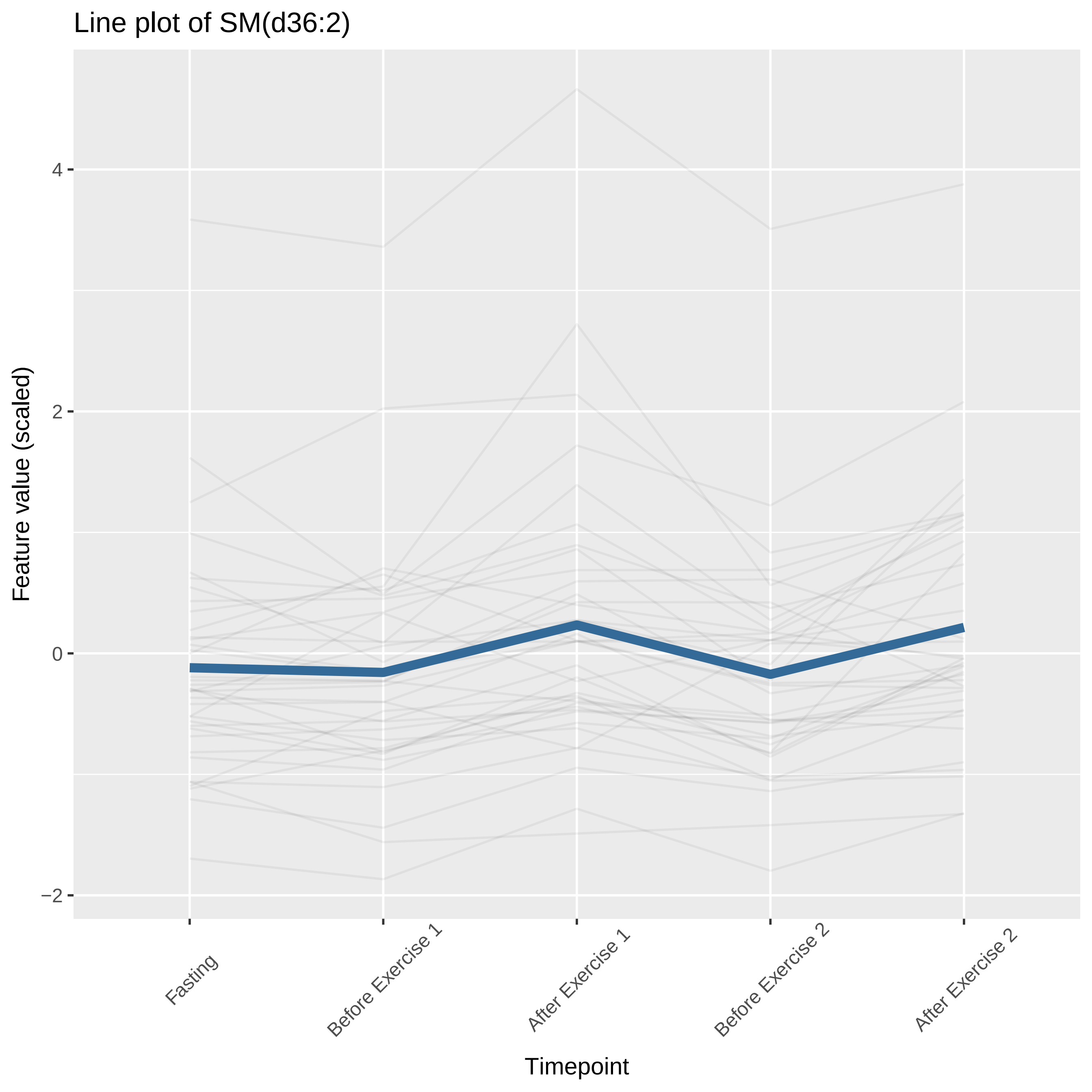

Line plot of SM(d36:2)
Feature value (scaled)
4
2
0
−2
Fasting
Before Exercise 1
After Exercise 1
Before Exercise 2
After Exercise 2
Timepoint

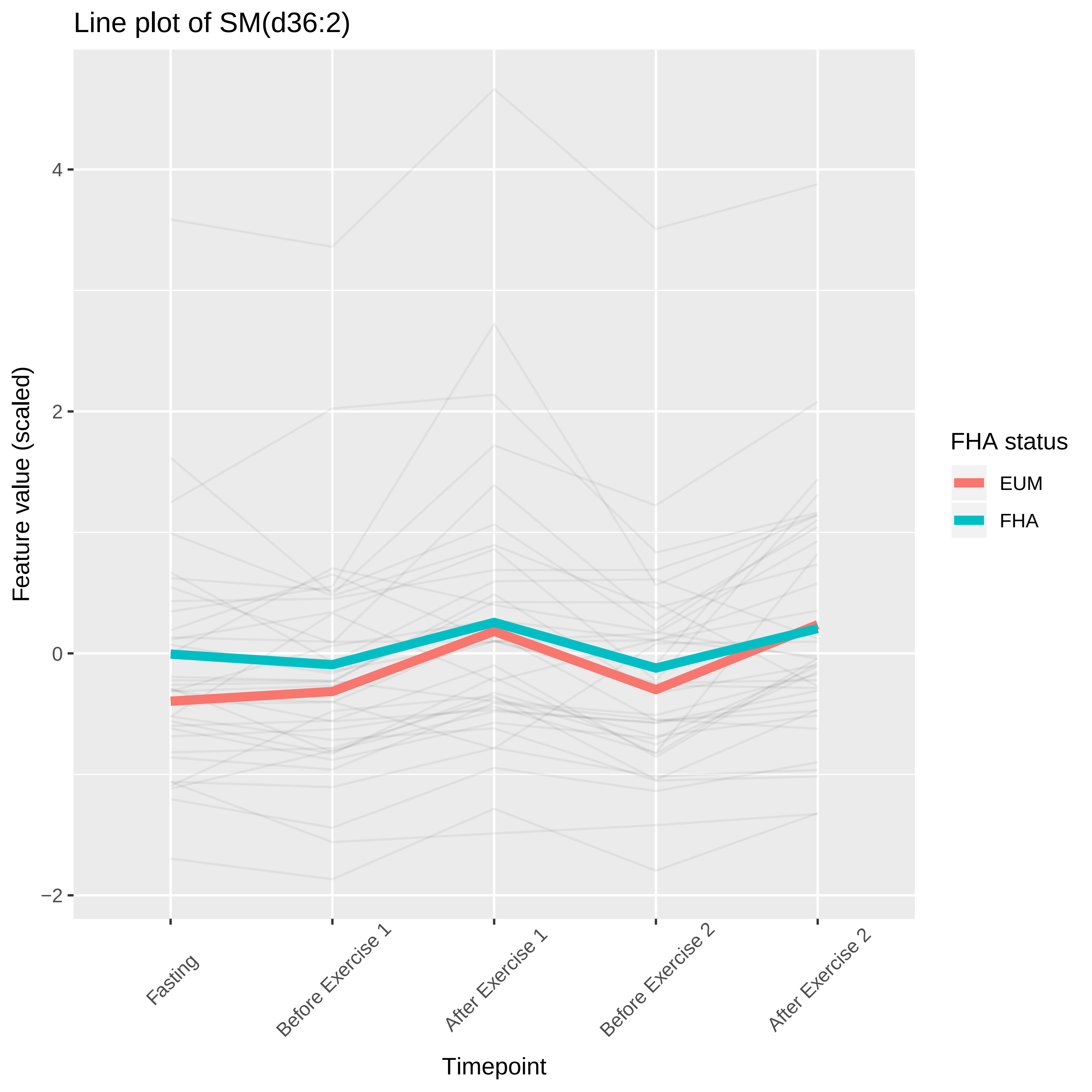
Line plot of SM(d36:2)
Feature value (scaled)
4
2
0
−2
Fasting
Before Exercise 1
After Exercise 1
Before Exercise 2
After Exercise 2
Timepoint
FHA status
EUM
FHA

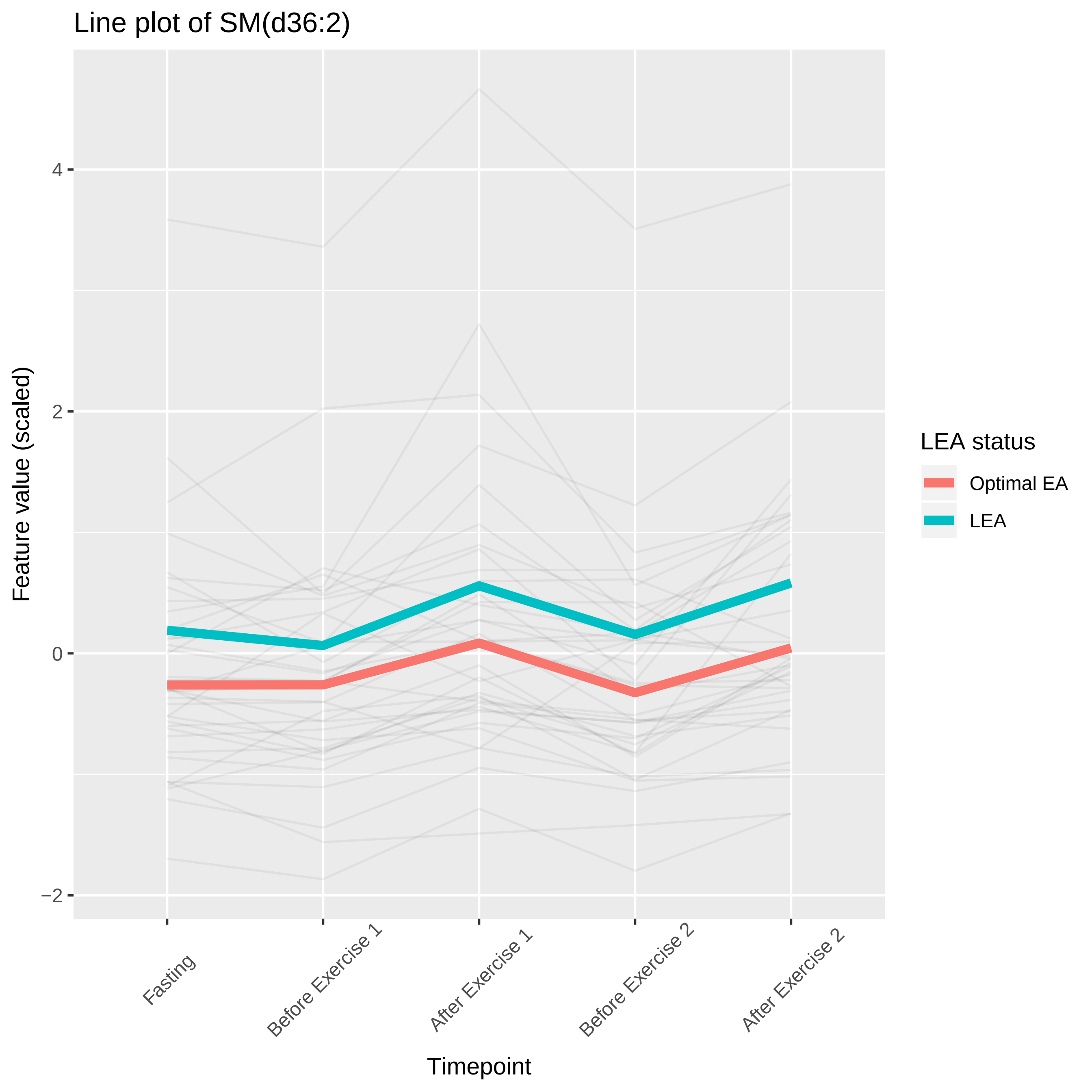
Line plot of SM(d36:2)
Feature value (scaled)
4
2
0
−2
Fasting
Before Exercise 1
After Exercise 1
Before Exercise 2
After Exercise 2
Timepoint
LEA status
Optimal EA
LEA

Line plot of SM(d38:0)

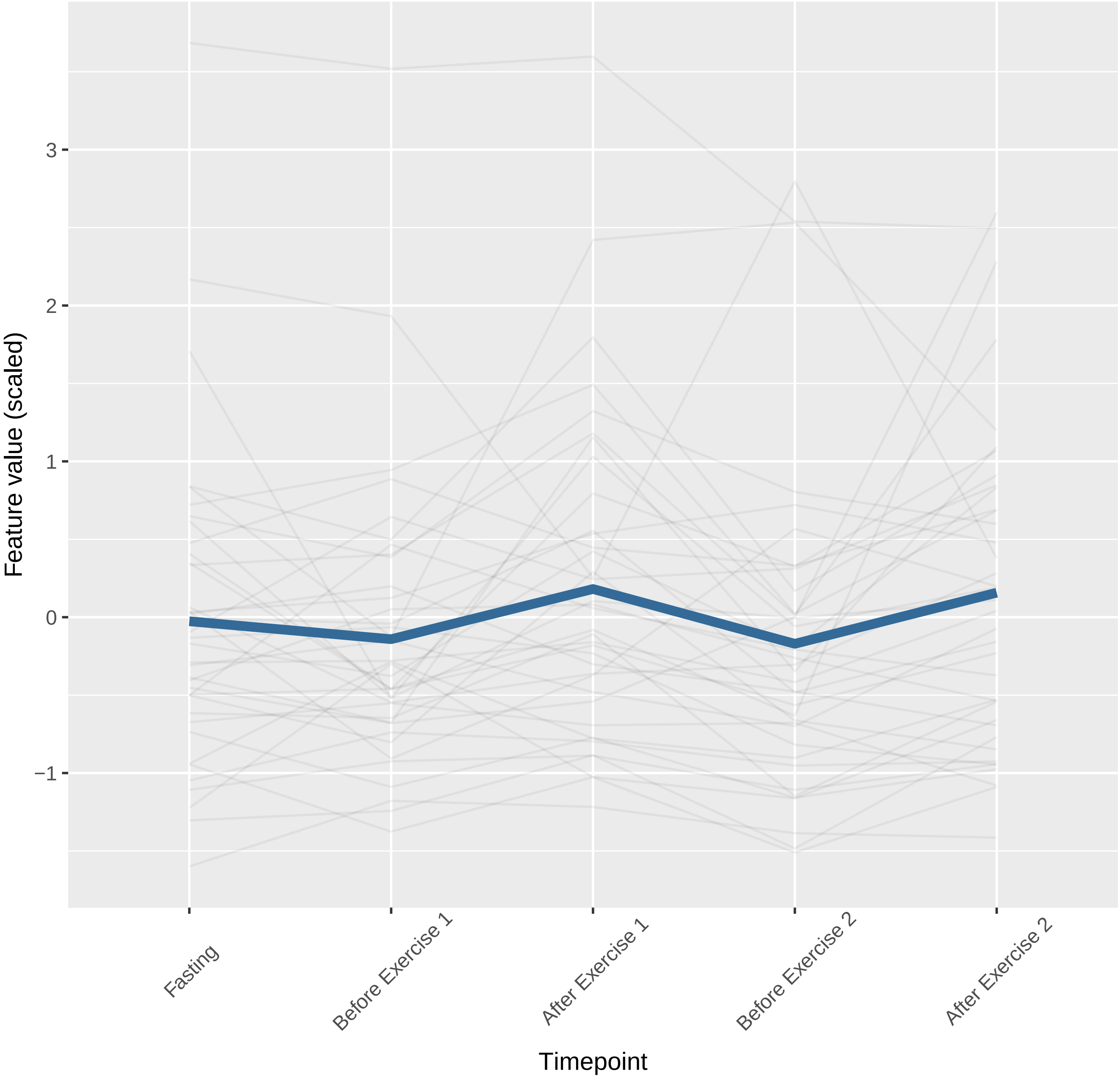

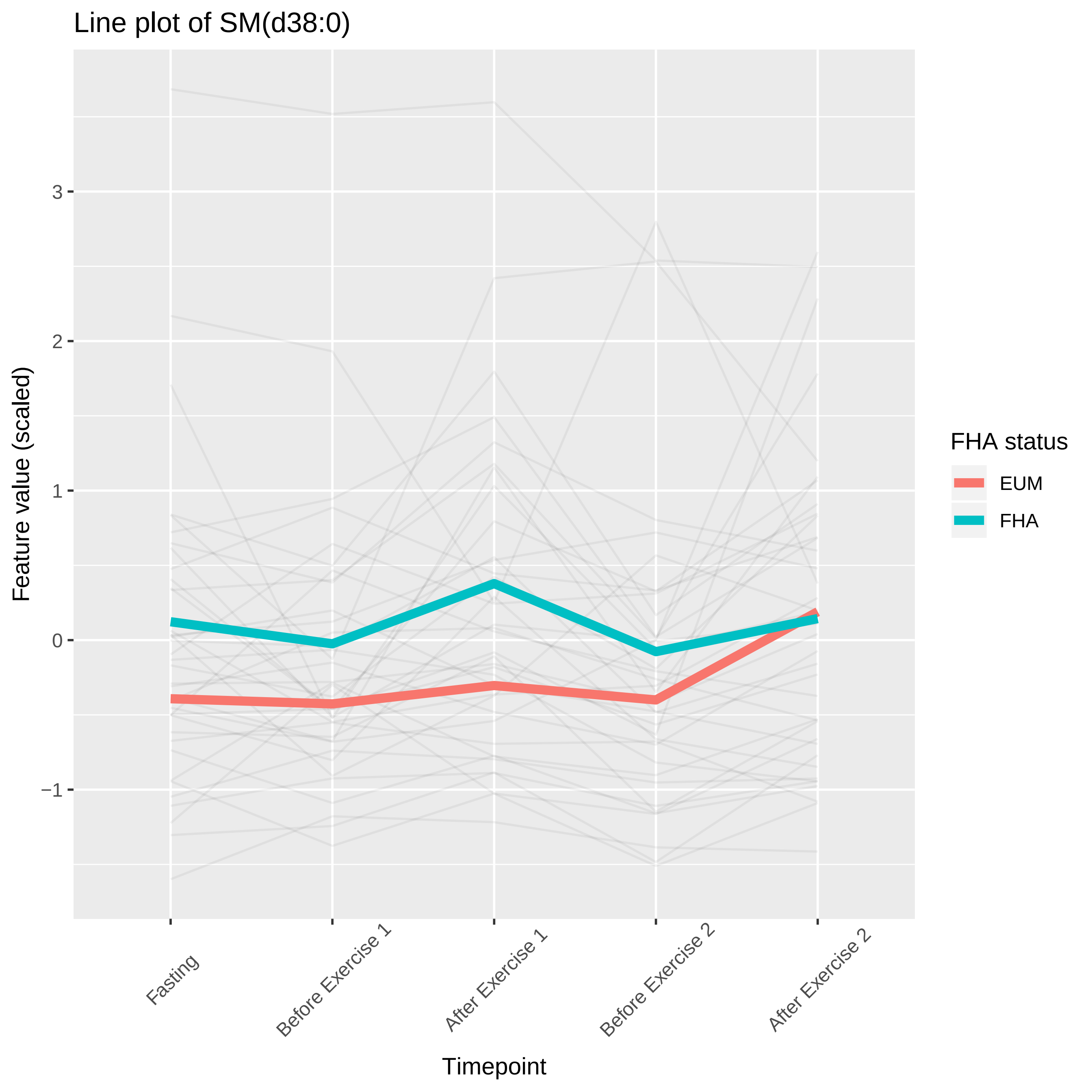

Line plot of SM(d38:0)
Feature value (scaled)
3
2
1
0
−1
Fasting
Before Exercise 1
After Exercise 1
Before Exercise 2
After Exercise 2
Timepoint
FHA status
EUM
FHA

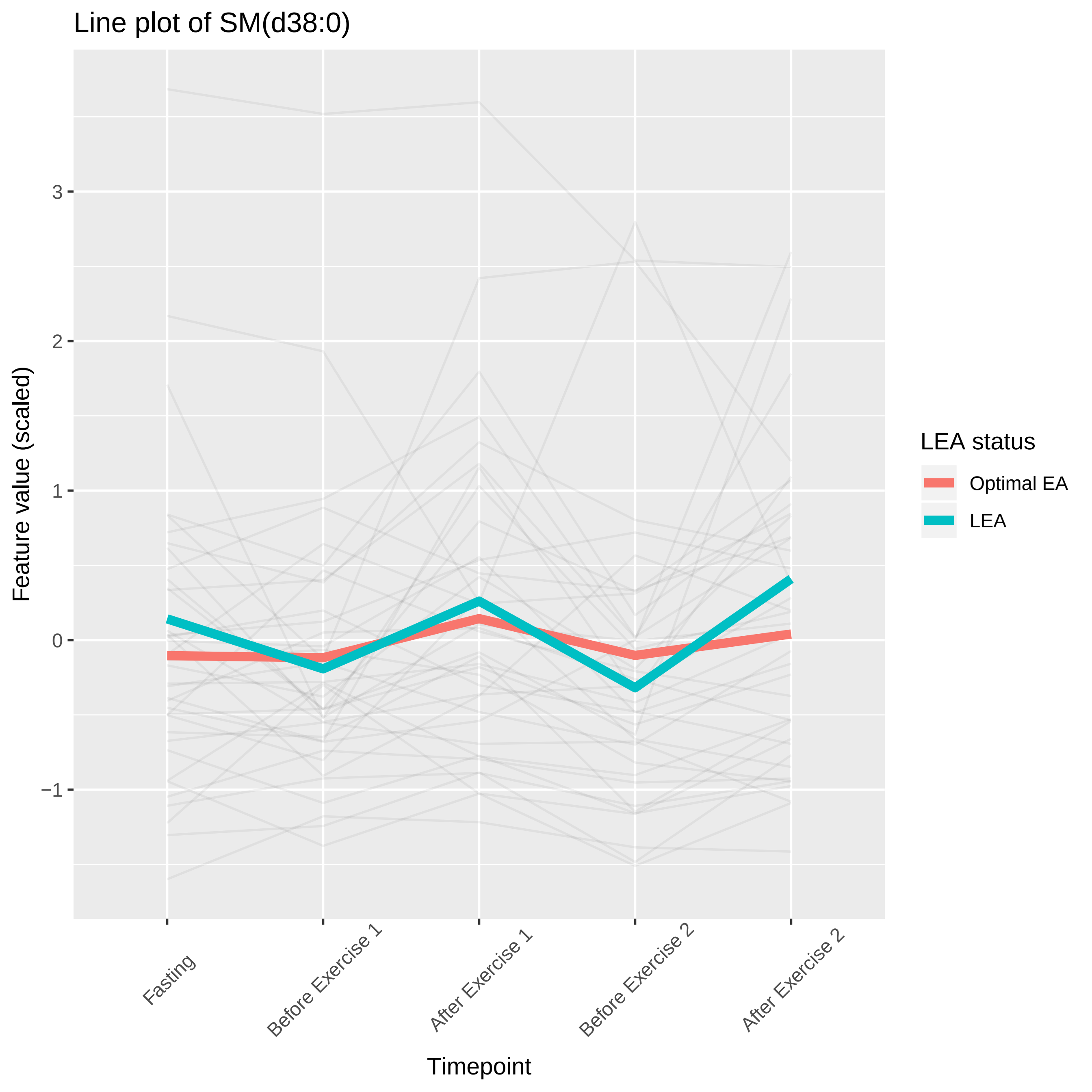
Line plot of SM(d38:0)
Feature value (scaled)
3
2
1
0
−1
Fasting
Before Exercise 1
After Exercise 1
Before Exercise 2
After Exercise 2
Timepoint
LEA status
Optimal EA
LEA

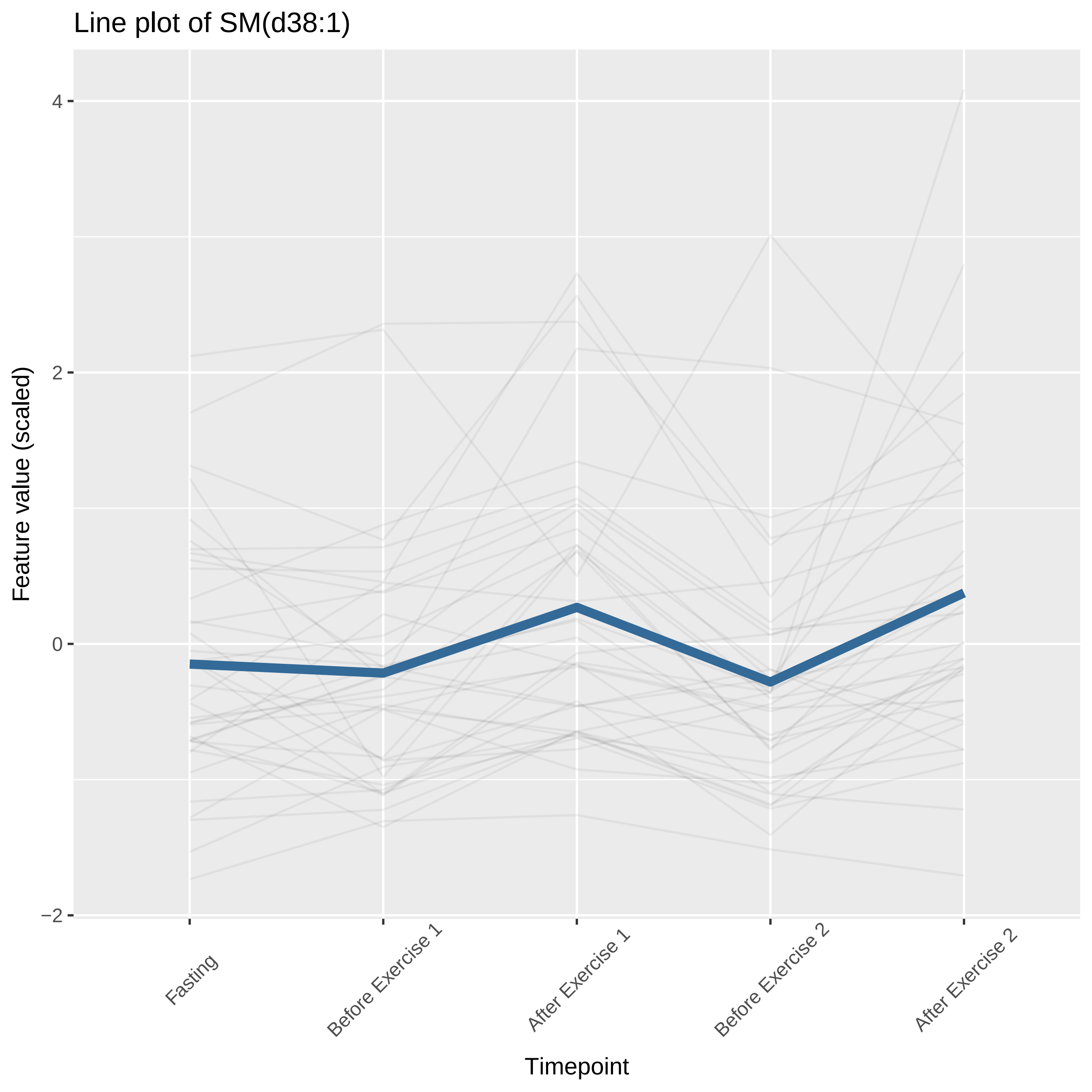

Line plot of SM(d38:1)
Feature value (scaled)
4
2
0
−2
Fasting
Before Exercise 1
After Exercise 1
Before Exercise 2
After Exercise 2
Timepoint

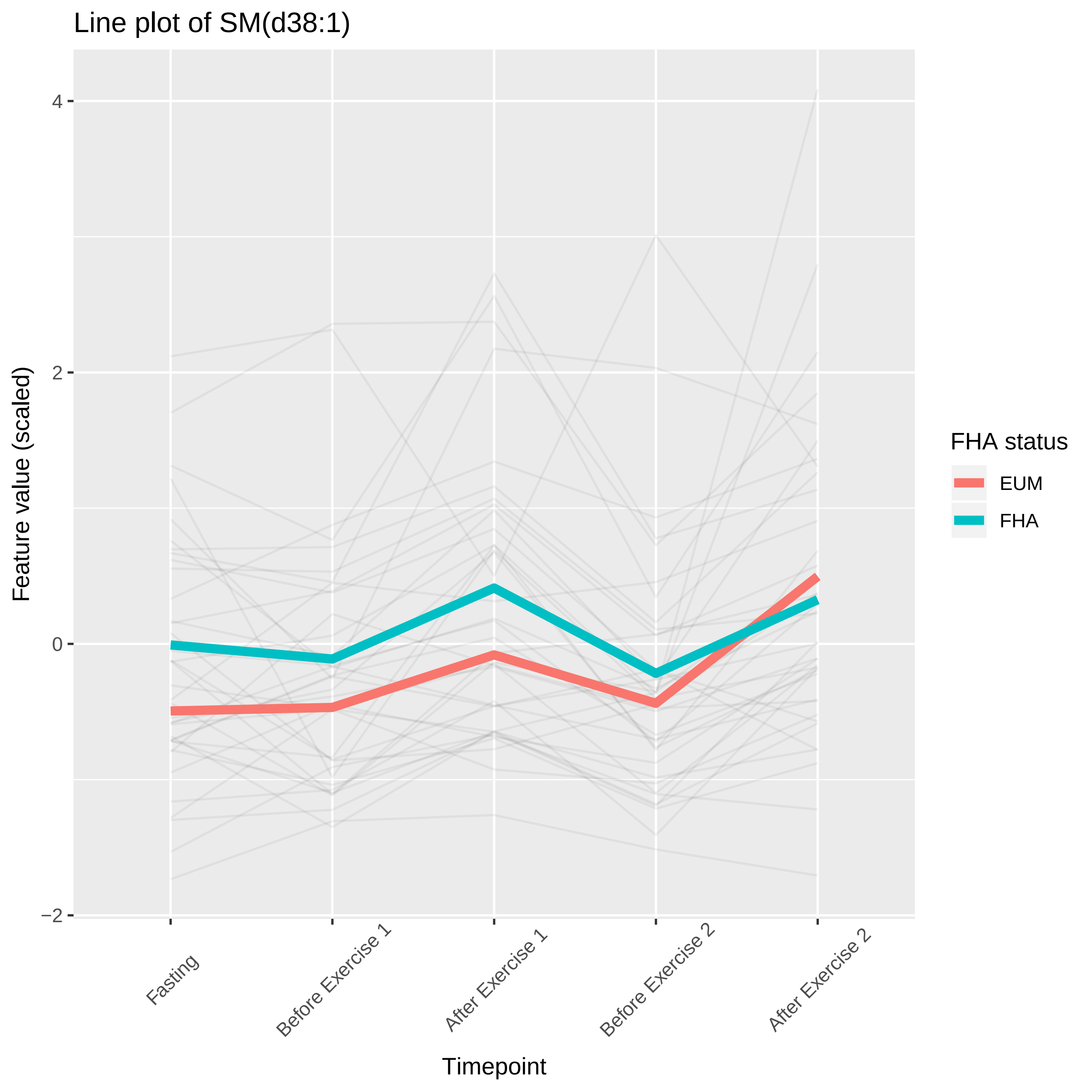

Line plot of SM(d38:1)
Feature value (scaled)
4
2
0
−2
Fasting
Before Exercise 1
After Exercise 1
Before Exercise 2
After Exercise 2
Timepoint
FHA status
EUM
FHA

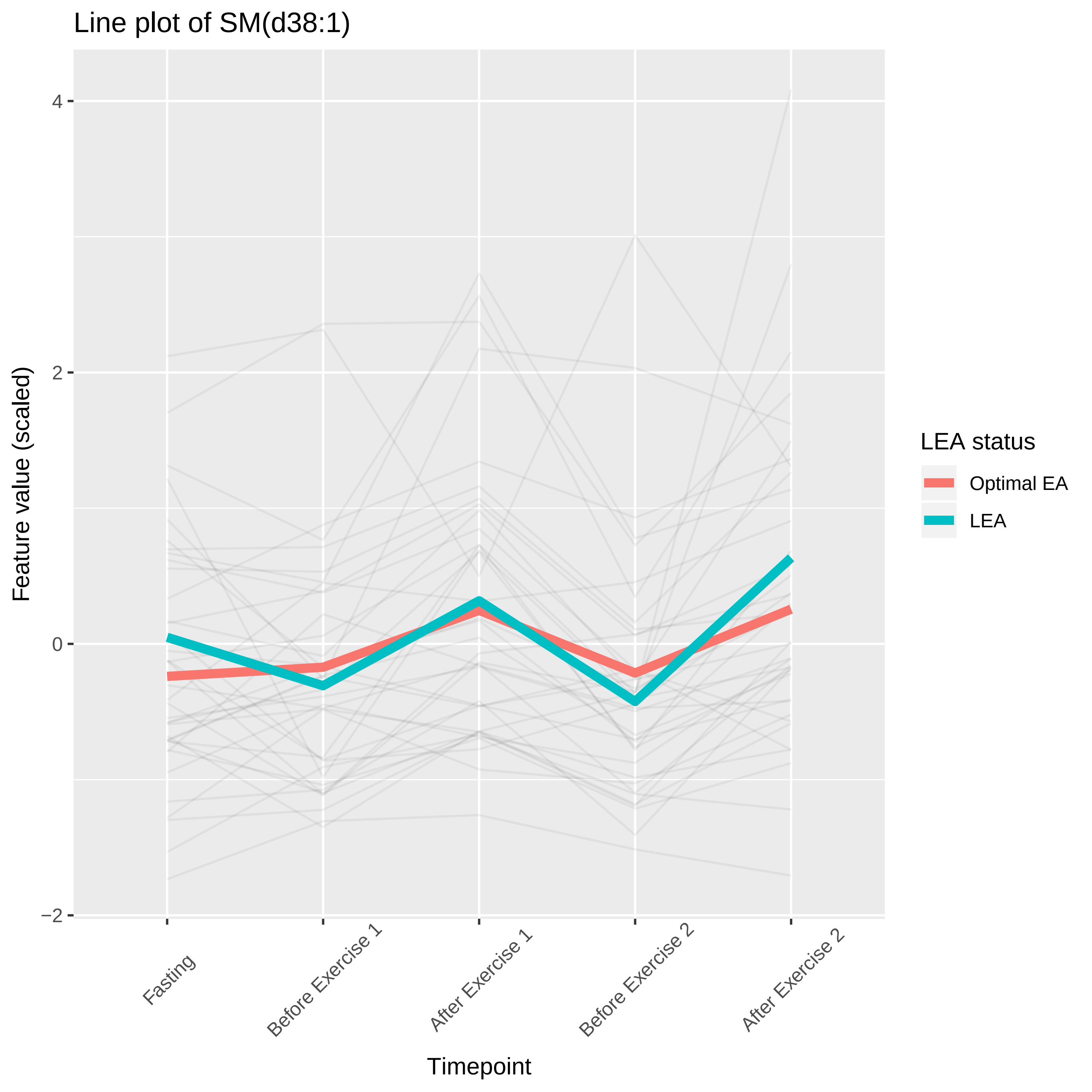
Line plot of SM(d38:1)
Feature value (scaled)
4
2
0
−2
Fasting
Before Exercise 1
After Exercise 1
Before Exercise 2
After Exercise 2
Timepoint
LEA status
Optimal EA
LEA

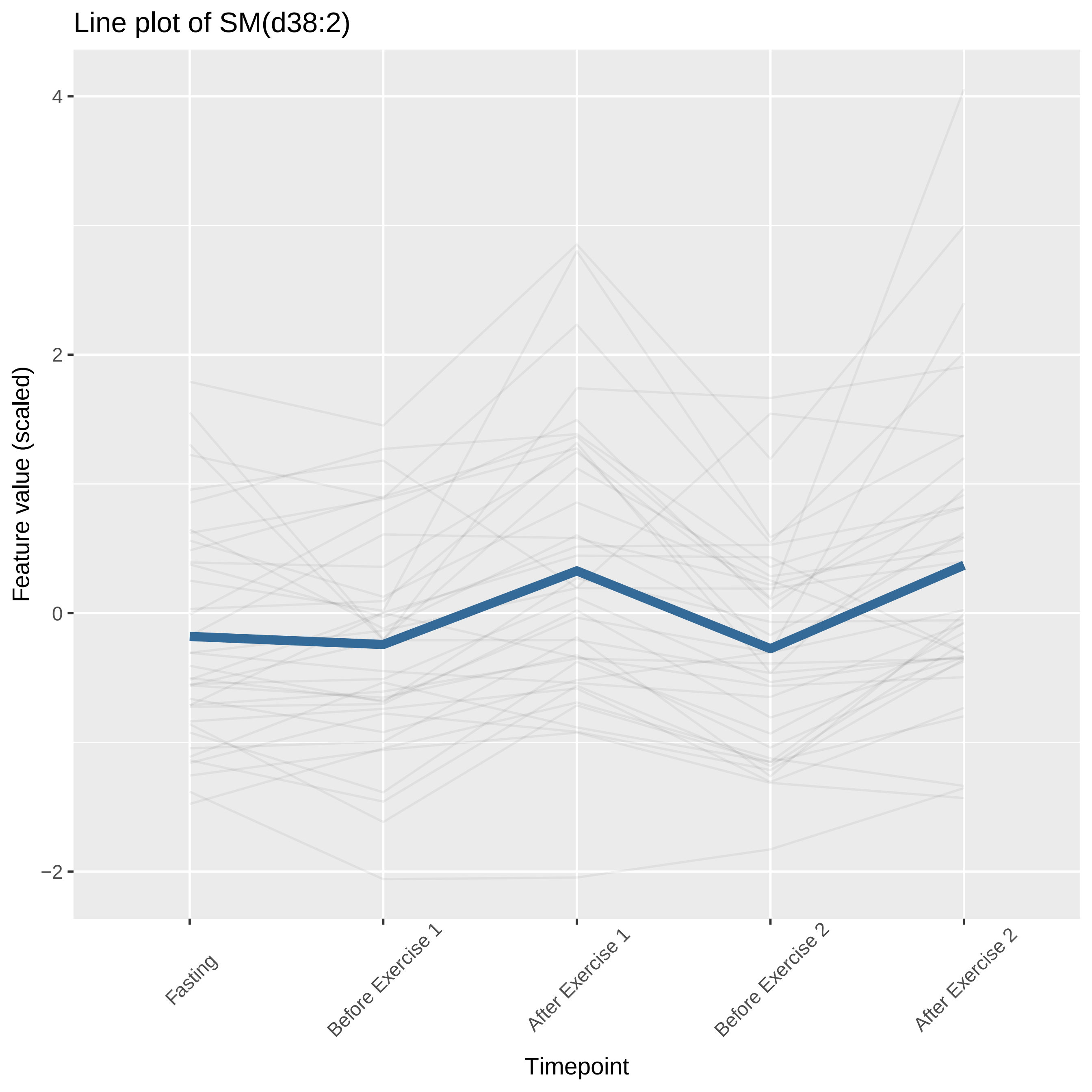

Line plot of SM(d38:2)
Feature value (scaled)
4
2
0
−2
Fasting
Before Exercise 1
After Exercise 1
Before Exercise 2
After Exercise 2
Timepoint

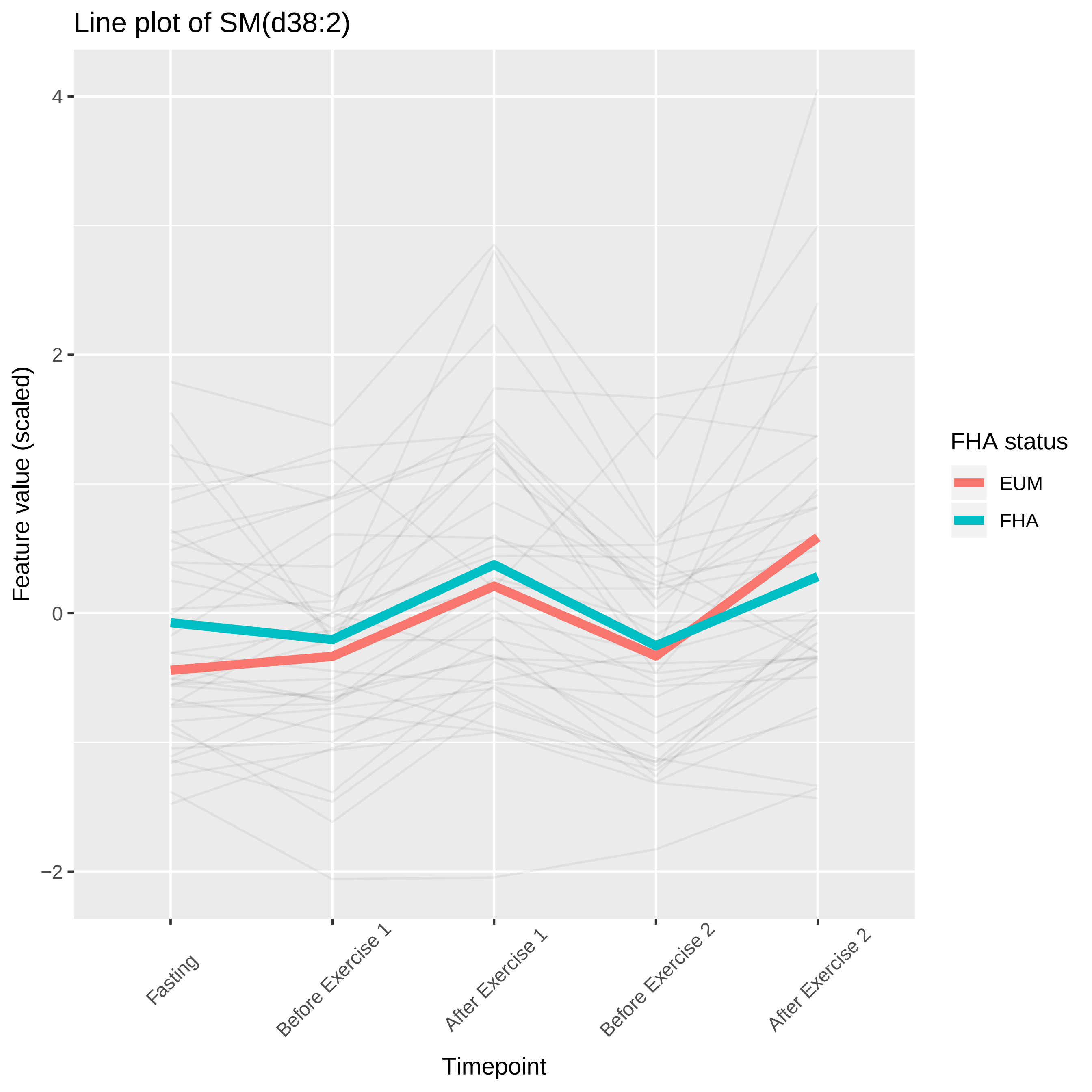

Line plot of SM(d38:2)
Feature value (scaled)
4
2
0
−2
Fasting
Before Exercise 1
After Exercise 1
Before Exercise 2
After Exercise 2
Timepoint
FHA status
EUM
FHA

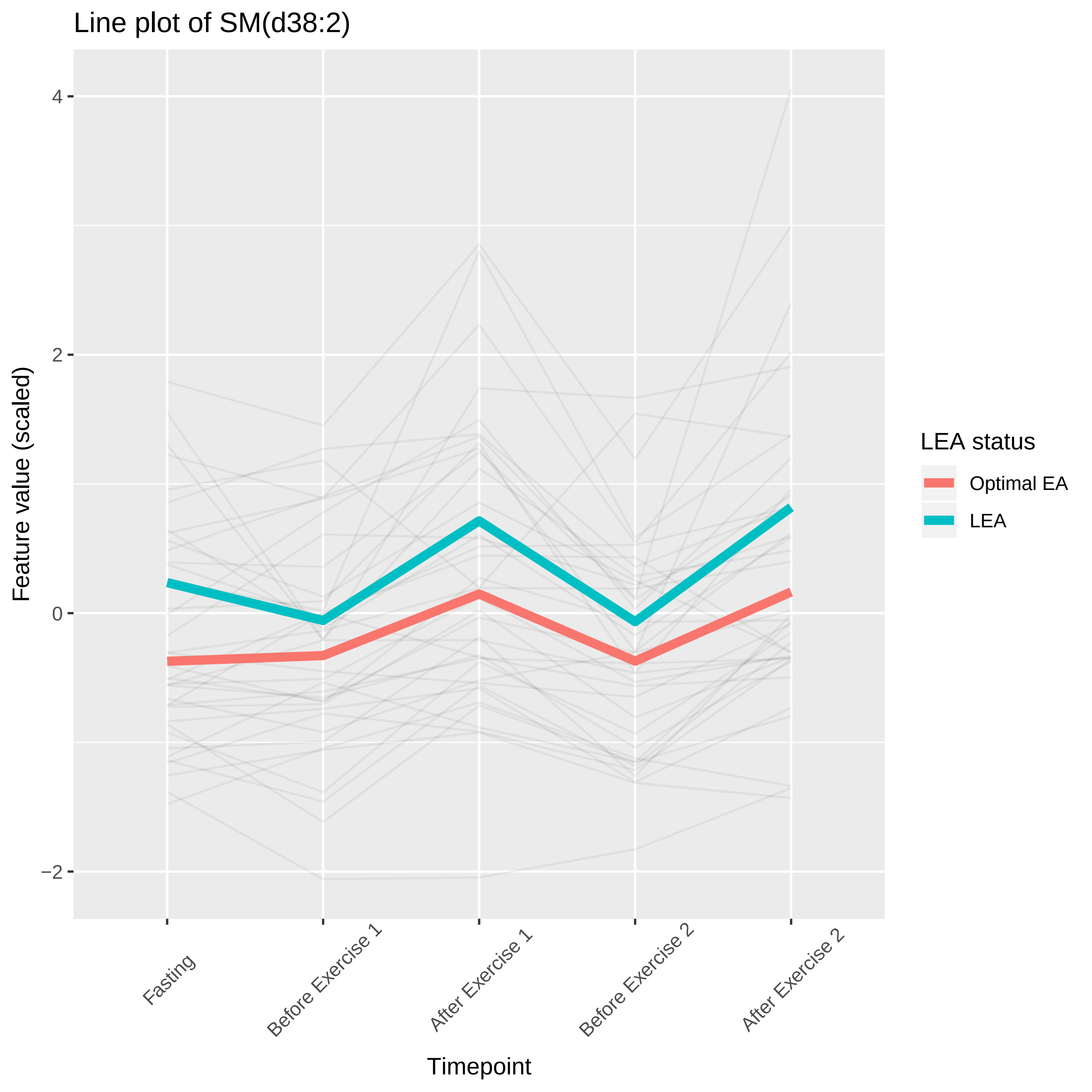

Line plot of SM(d38:2)
4
2
0
−2
Feature value (scaled)
Fasting
Before Exercise 1
After Exercise 1
Before Exercise 2
After Exercise 2
Timepoint
LEA status
Optimal EA
LEA

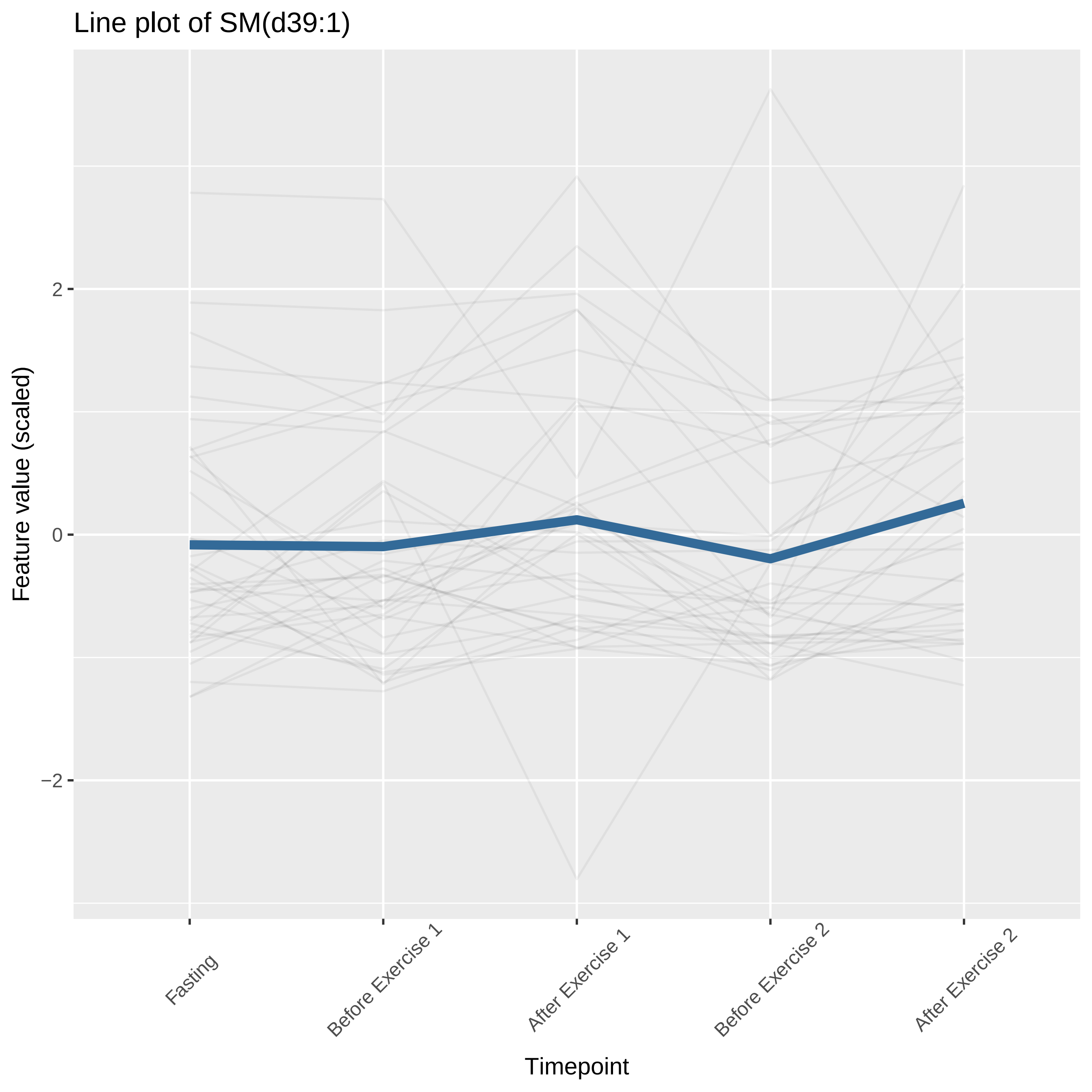

Line plot of SM(d39:1)
Feature value (scaled)
2
0
−2
Fasting
Before Exercise 1
After Exercise 1
Before Exercise 2
After Exercise 2
Timepoint

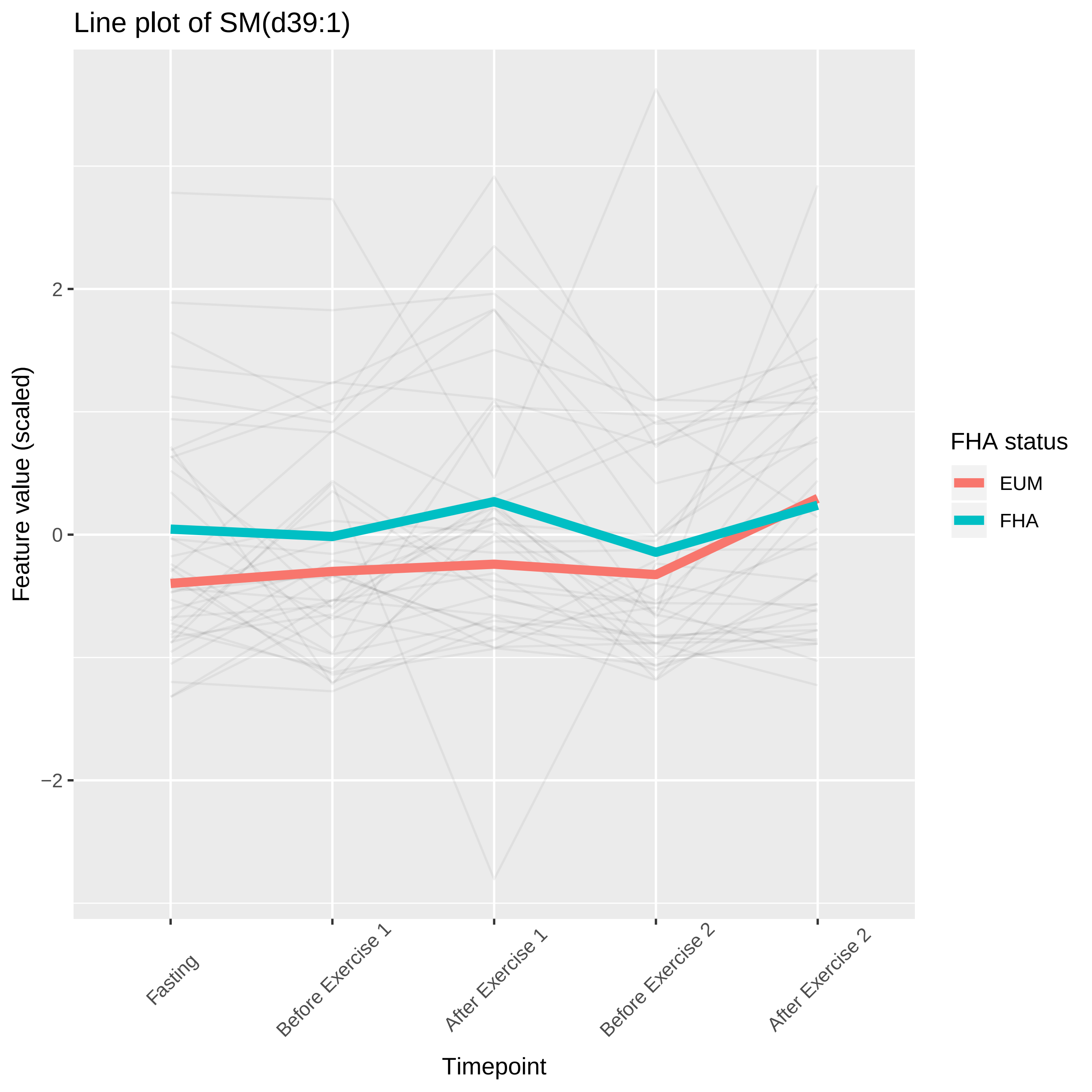

Line plot of SM(d39:1)
Feature value (scaled)
2
0
−2
Fasting
Before Exercise 1
After Exercise 1
Before Exercise 2
After Exercise 2
Timepoint
FHA status
EUM
FHA

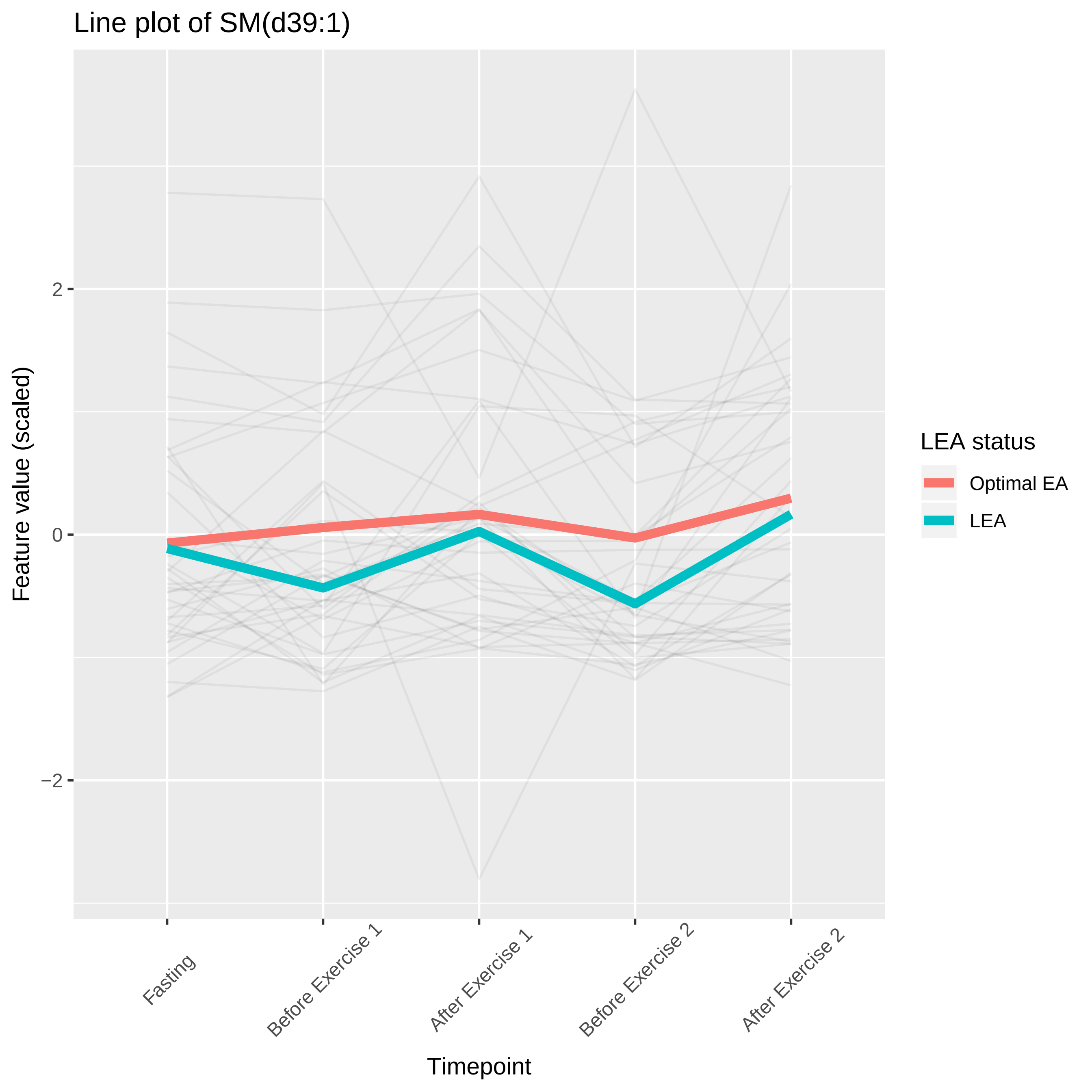

Line plot of SM(d39:1)
Feature value (scaled)
2
0
−2
Fasting
Before Exercise 1
After Exercise 1
Before Exercise 2
After Exercise 2
Timepoint
LEA status
Optimal EA
LEA

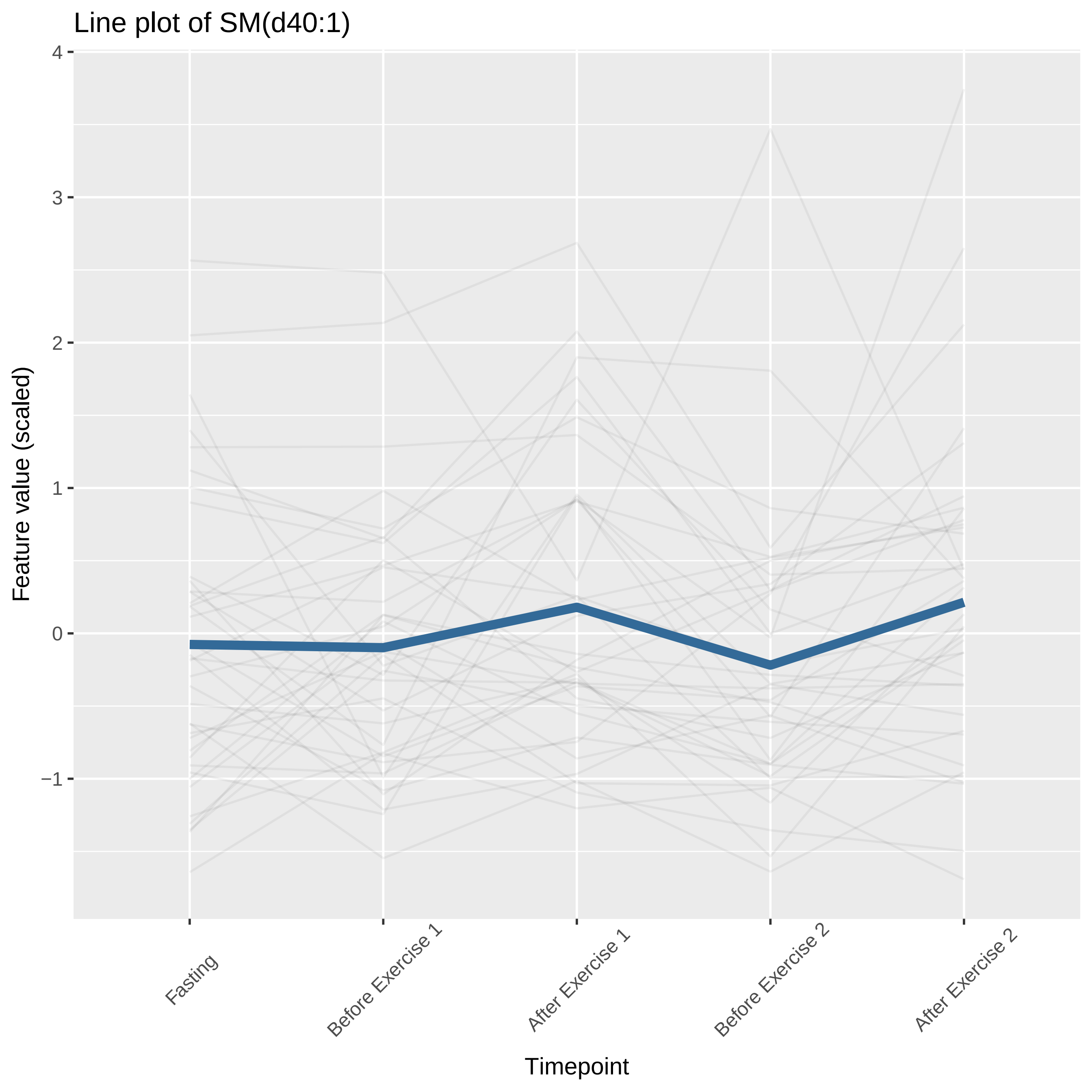

Line plot of SM(d40:1)
Feature value (scaled)
4
3
2
1
0
−1
Fasting
Before Exercise 1
After Exercise 1
Before Exercise 2
After Exercise 2
Timepoint

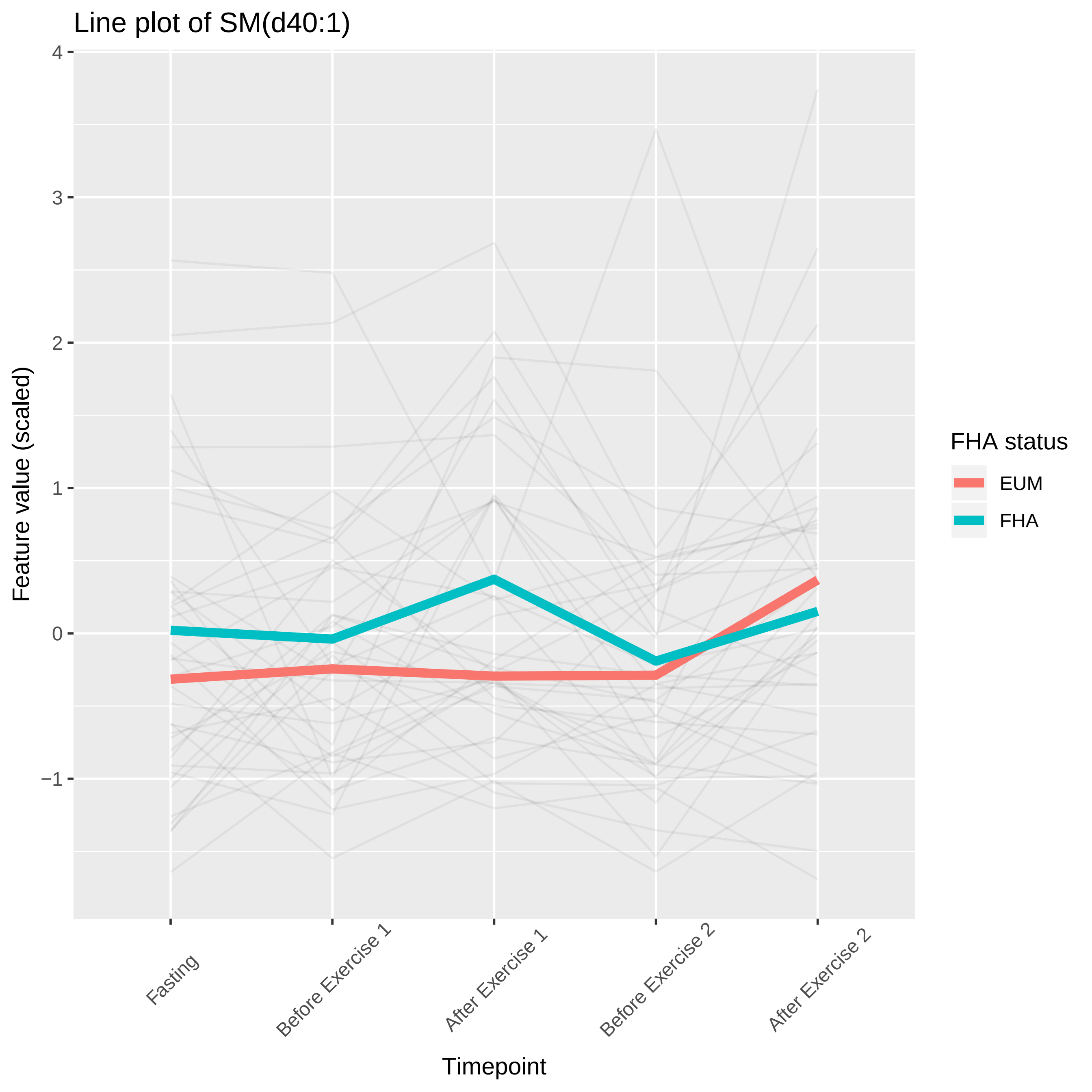

Line plot of SM(d40:1)
Feature value (scaled)
4
3
2
1
0
−1
Fasting
Before Exercise 1
After Exercise 1
Before Exercise 2
After Exercise 2
Timepoint
FHA status
EUM
FHA

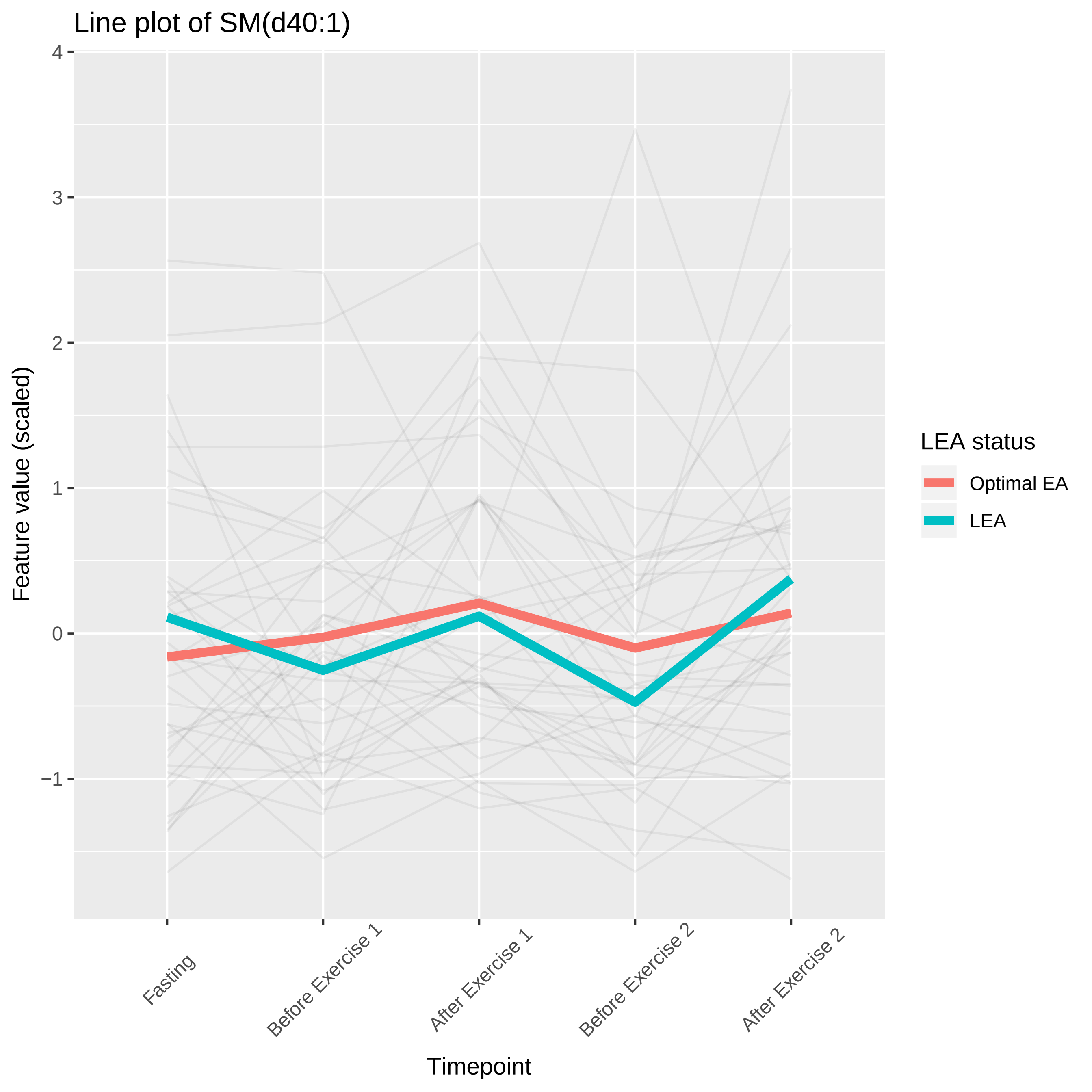
Line plot of SM(d40:1)
Feature value (scaled)
4
3
2
1
0
−1
Fasting
Before Exercise 1
After Exercise 1
Before Exercise 2
After Exercise 2
Timepoint
LEA status
Optimal EA
LEA

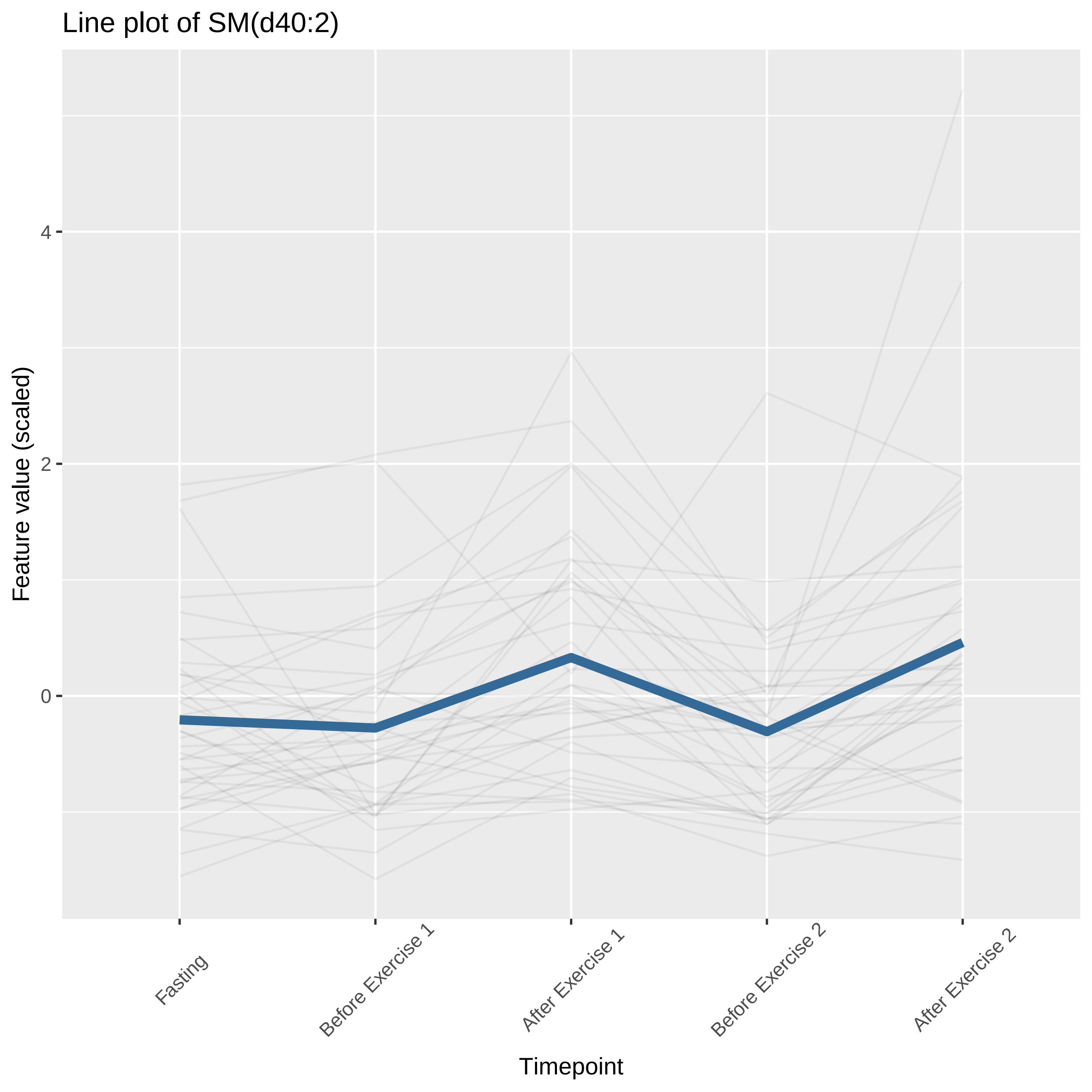

Line plot of SM(d40:2)
Feature value (scaled)
4
2
0
Fasting
Before Exercise 1
After Exercise 1
Before Exercise 2
After Exercise 2
Timepoint

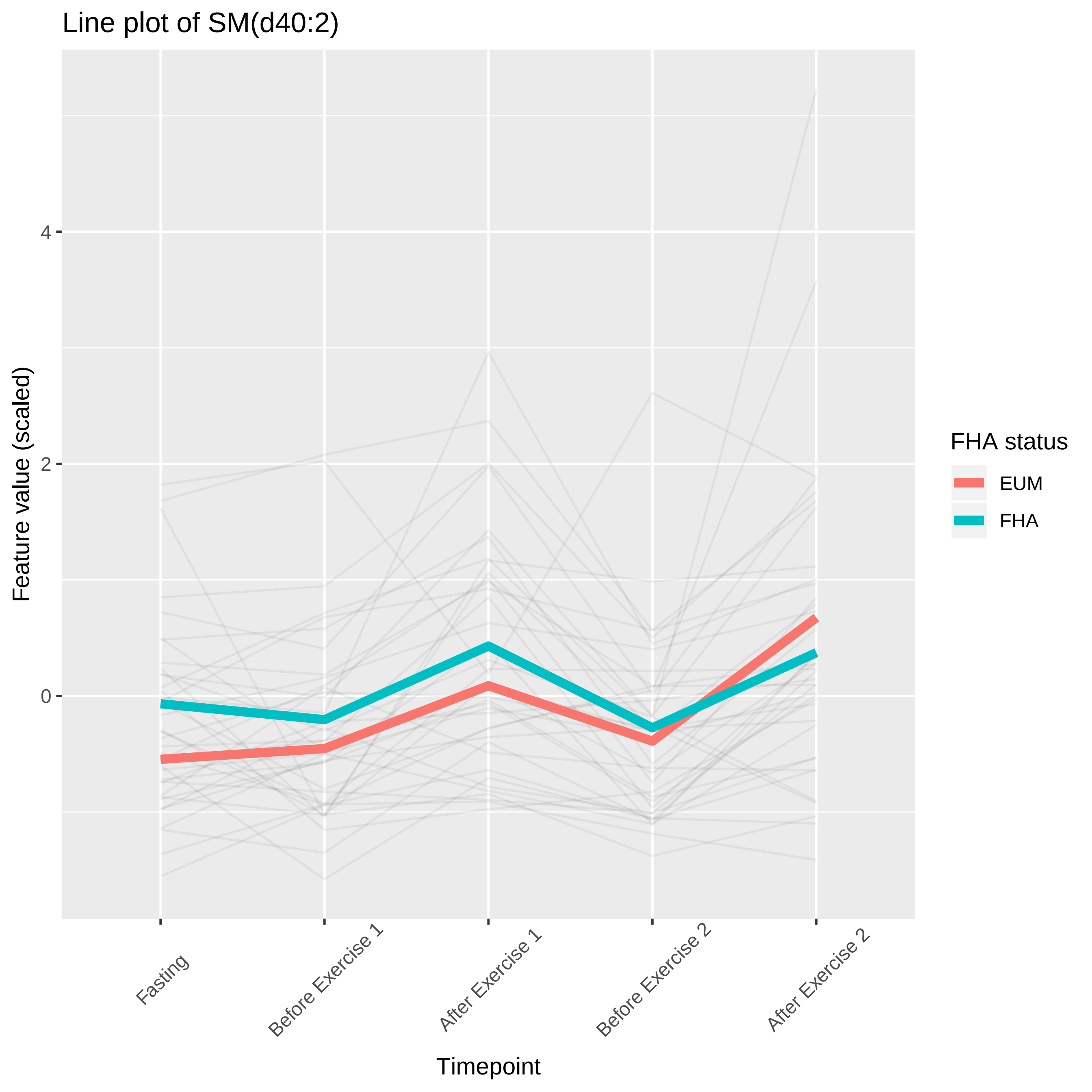

Line plot of SM(d40:2)
Feature value (scaled)
4
2
0
Fasting
Before Exercise 1
After Exercise 1
Before Exercise 2
After Exercise 2
Timepoint
FHA status
EUM
FHA

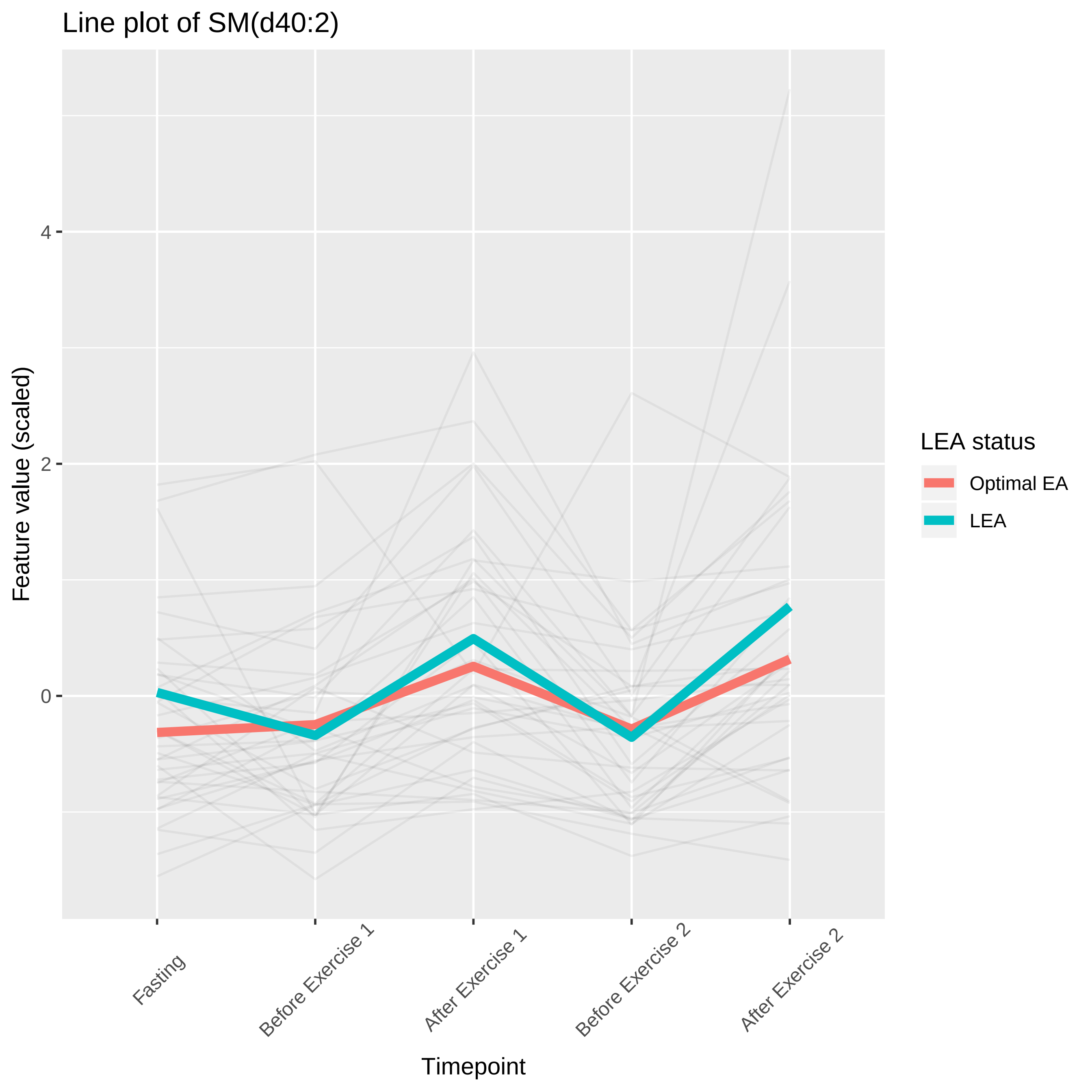

Line plot of SM(d40:2)
Feature value (scaled)
4
2
0
Fasting
Before Exercise 1
After Exercise 1
Before Exercise 2
After Exercise 2
Timepoint
LEA status
Optimal EA
LEA

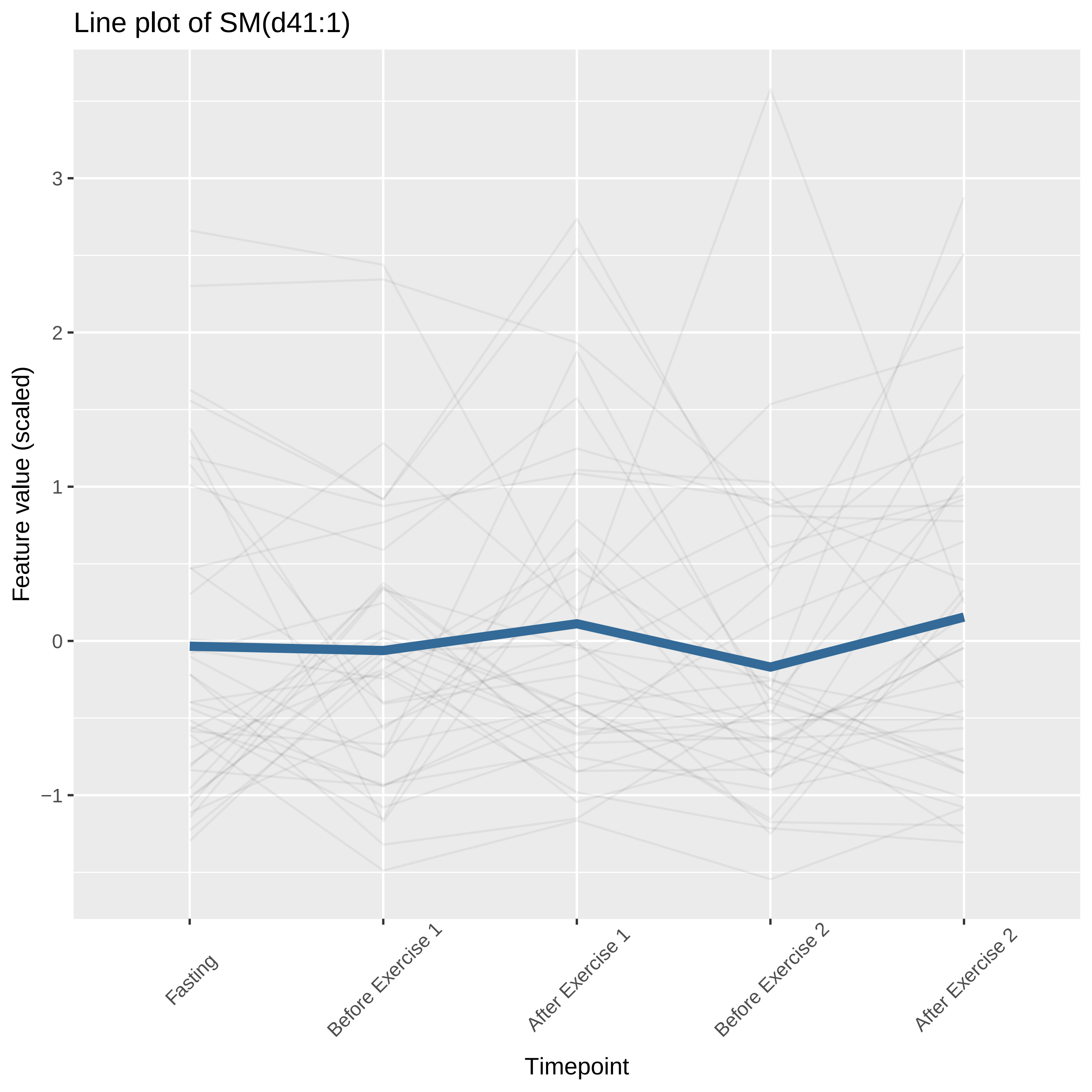

Line plot of SM(d41:1)
Feature value (scaled)
3
2
1
0
−1
Fasting
Before Exercise 1
After Exercise 1
Before Exercise 2
After Exercise 2
Timepoint

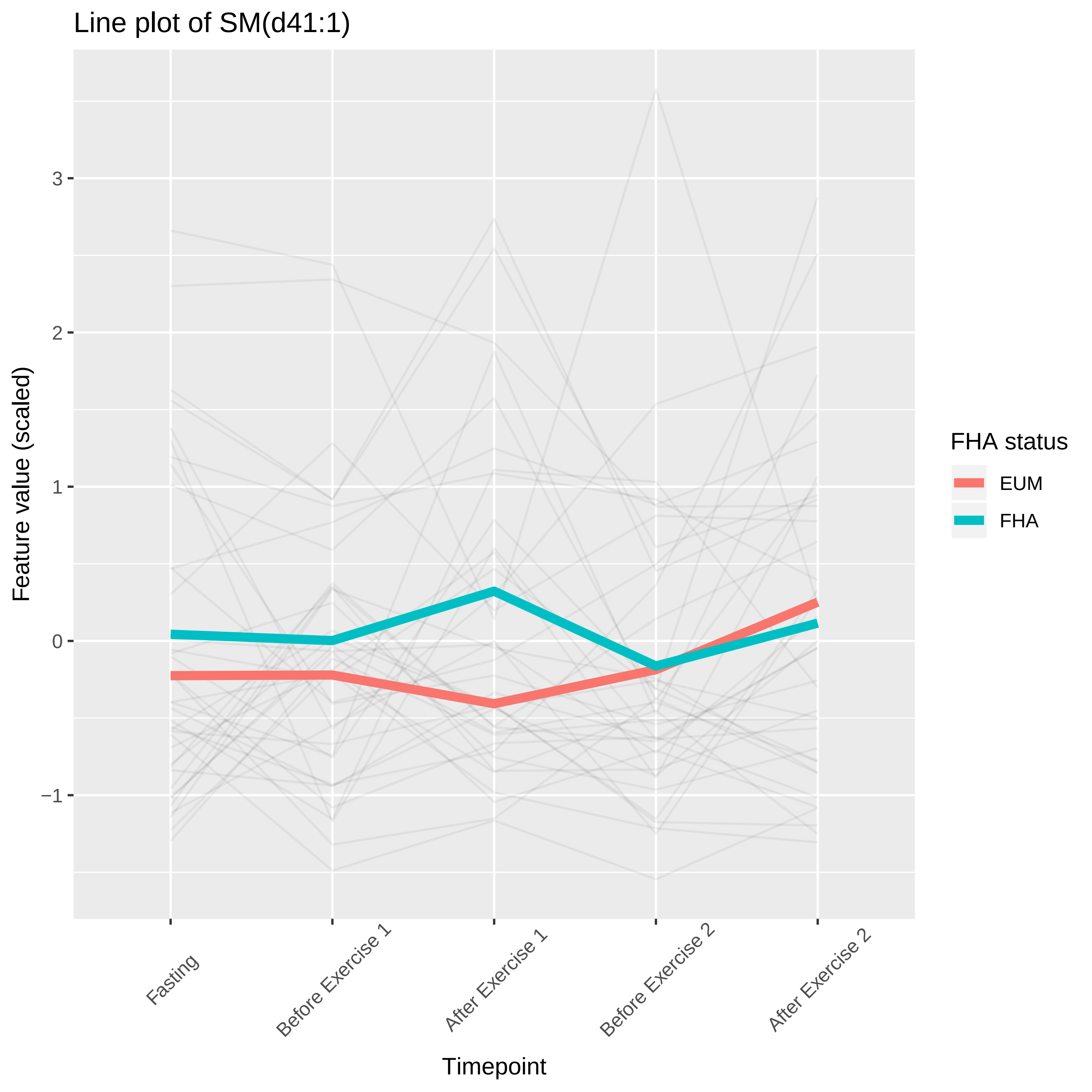
Line plot of SM(d41:1)
Feature value (scaled)
3
2
1
0
−1
Fasting
Before Exercise 1
After Exercise 1
Before Exercise 2
After Exercise 2
Timepoint
FHA status
EUM
FHA

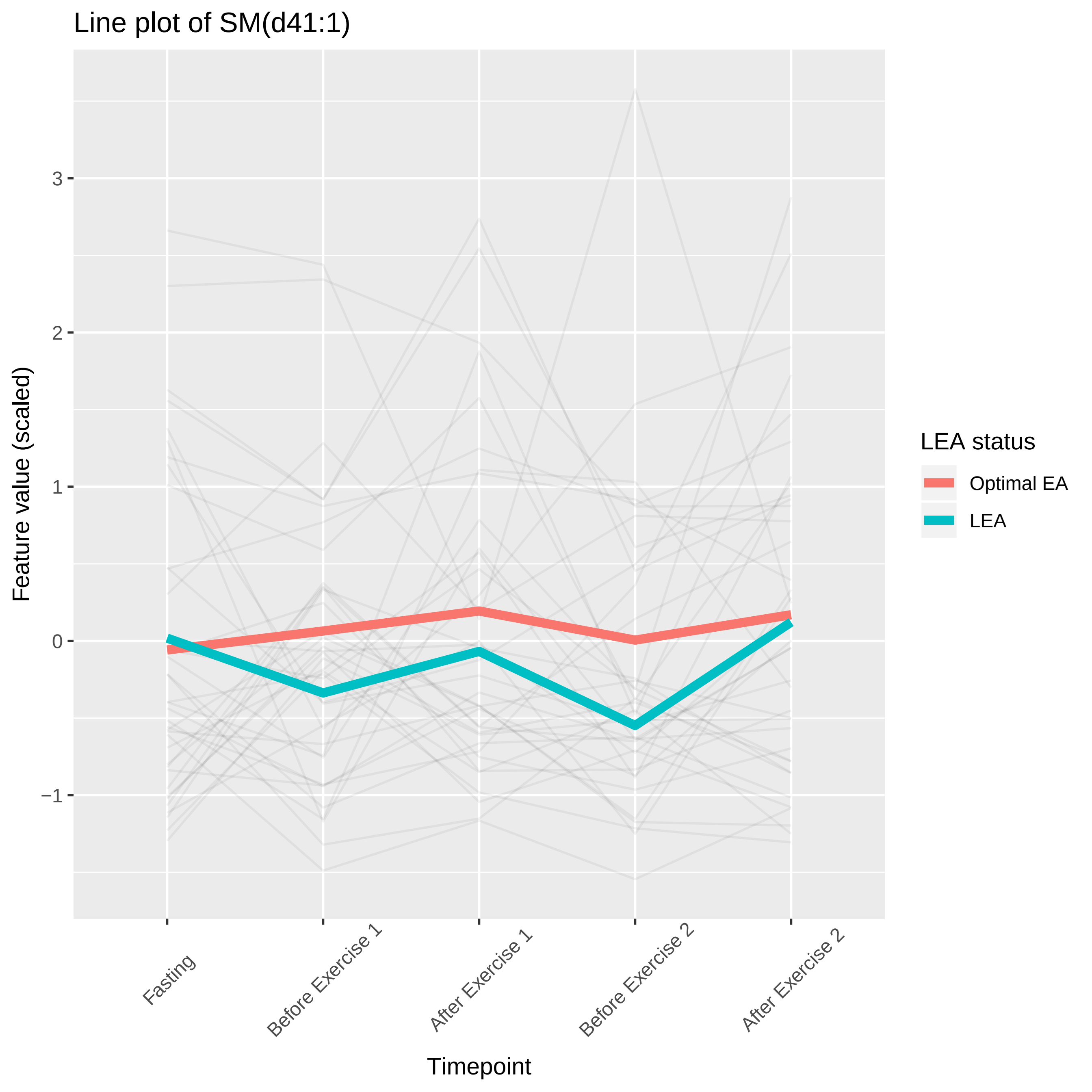

Line plot of SM(d41:1)
Feature value (scaled)
3
2
1
0
−1
Fasting
Before Exercise 1
After Exercise 1
Before Exercise 2
After Exercise 2
Timepoint
LEA status
Optimal EA
LEA

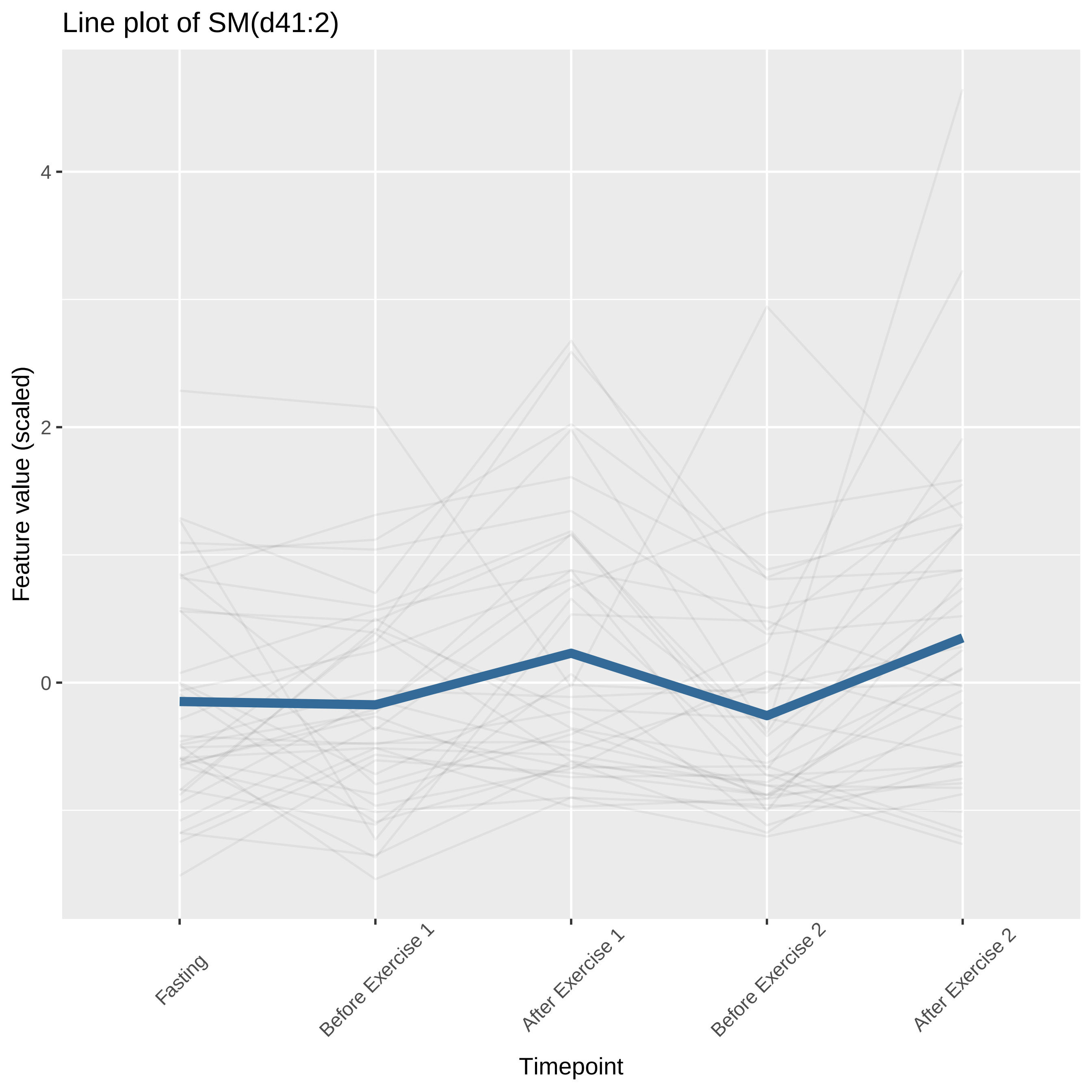

Line plot of SM(d41:2)
Feature value (scaled)
4
2
0
Fasting
Before Exercise 1
After Exercise 1
Before Exercise 2
After Exercise 2
Timepoint

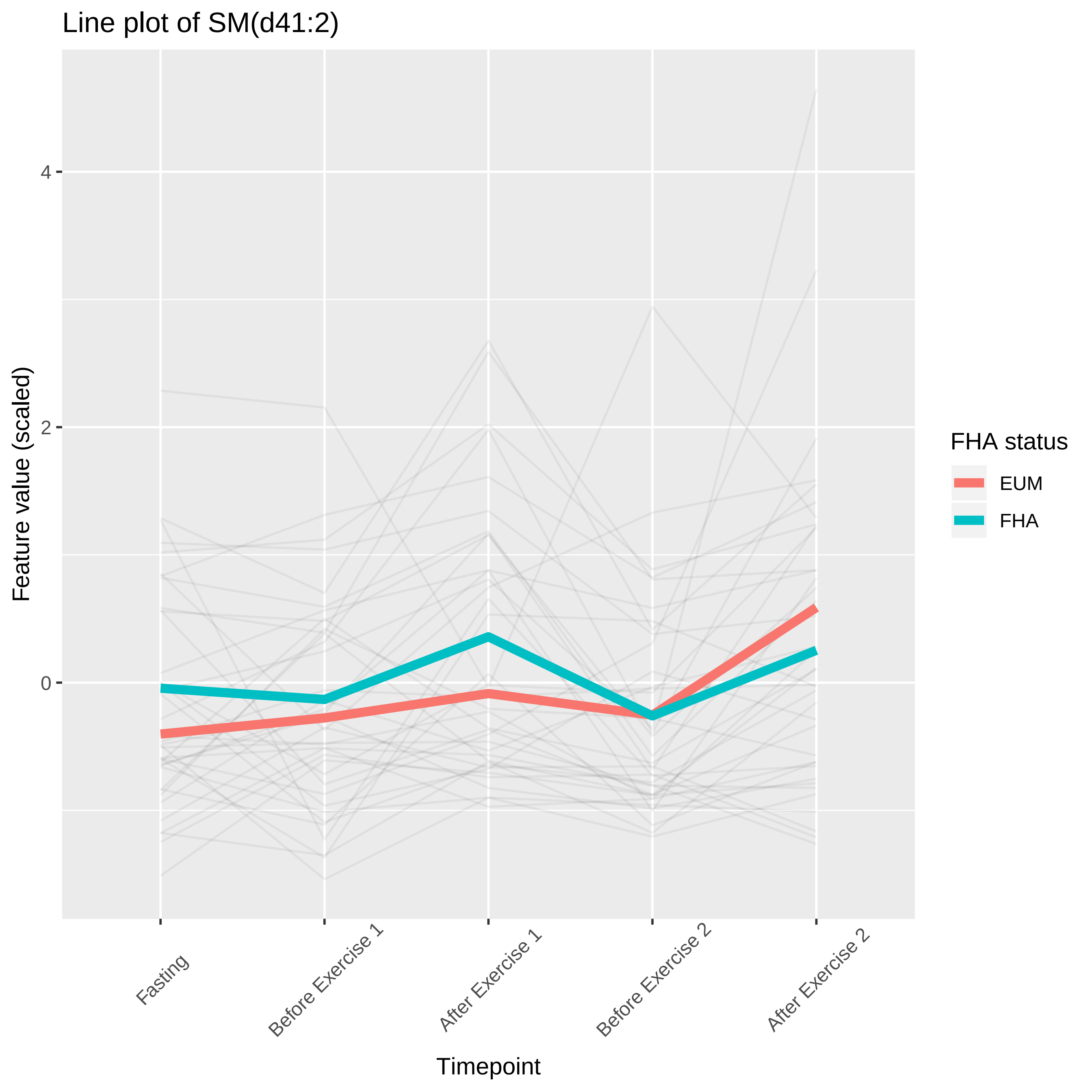
Line plot of SM(d41:2)
Feature value (scaled)
4
2
0
Fasting
Before Exercise 1
After Exercise 1
Before Exercise 2
After Exercise 2
Timepoint
FHA status
EUM
FHA

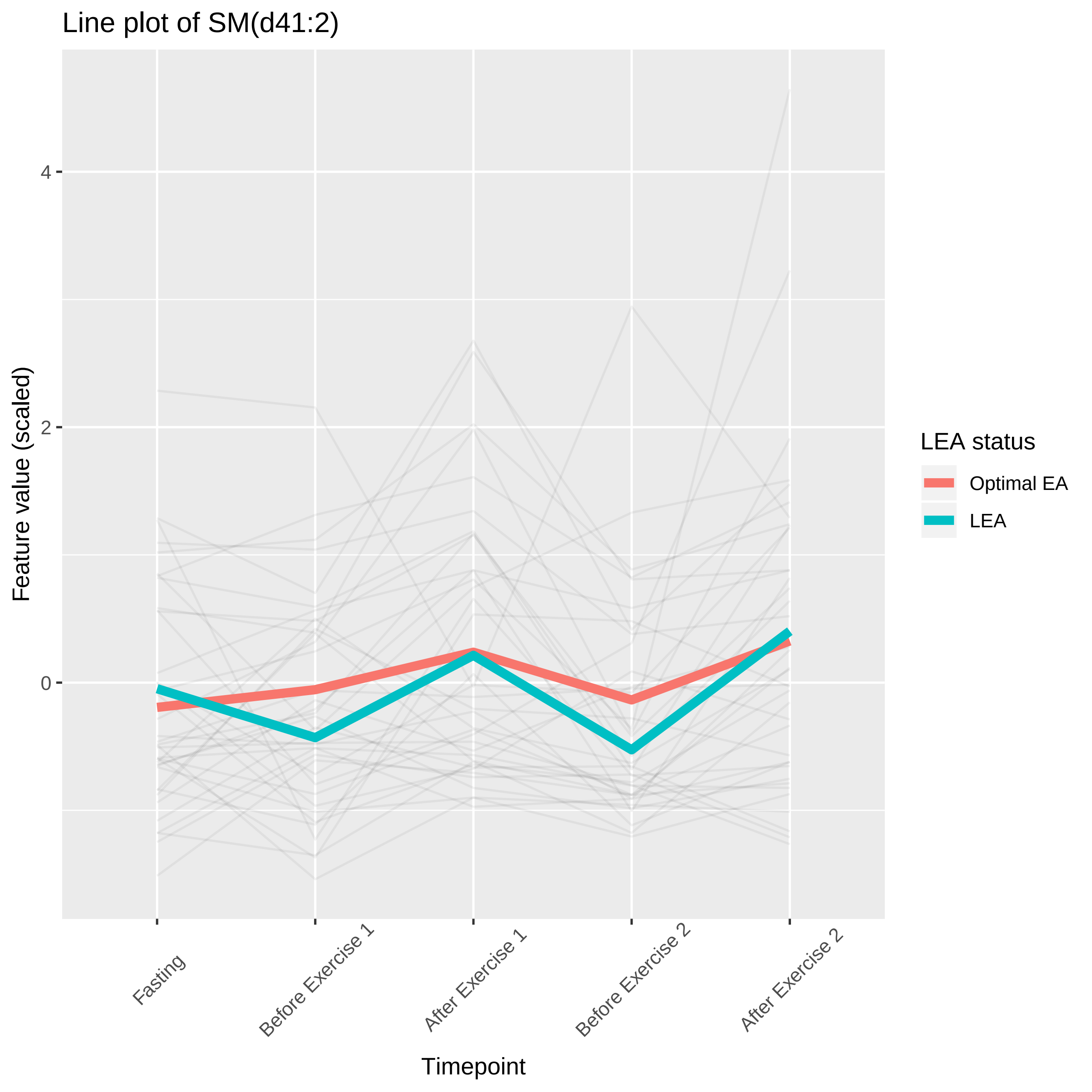

Line plot of SM(d41:2)
Feature value (scaled)
4
2
0
Fasting
Before Exercise 1
After Exercise 1
Before Exercise 2
After Exercise 2
Timepoint
LEA status
Optimal EA
LEA

MISCELLANEOUS

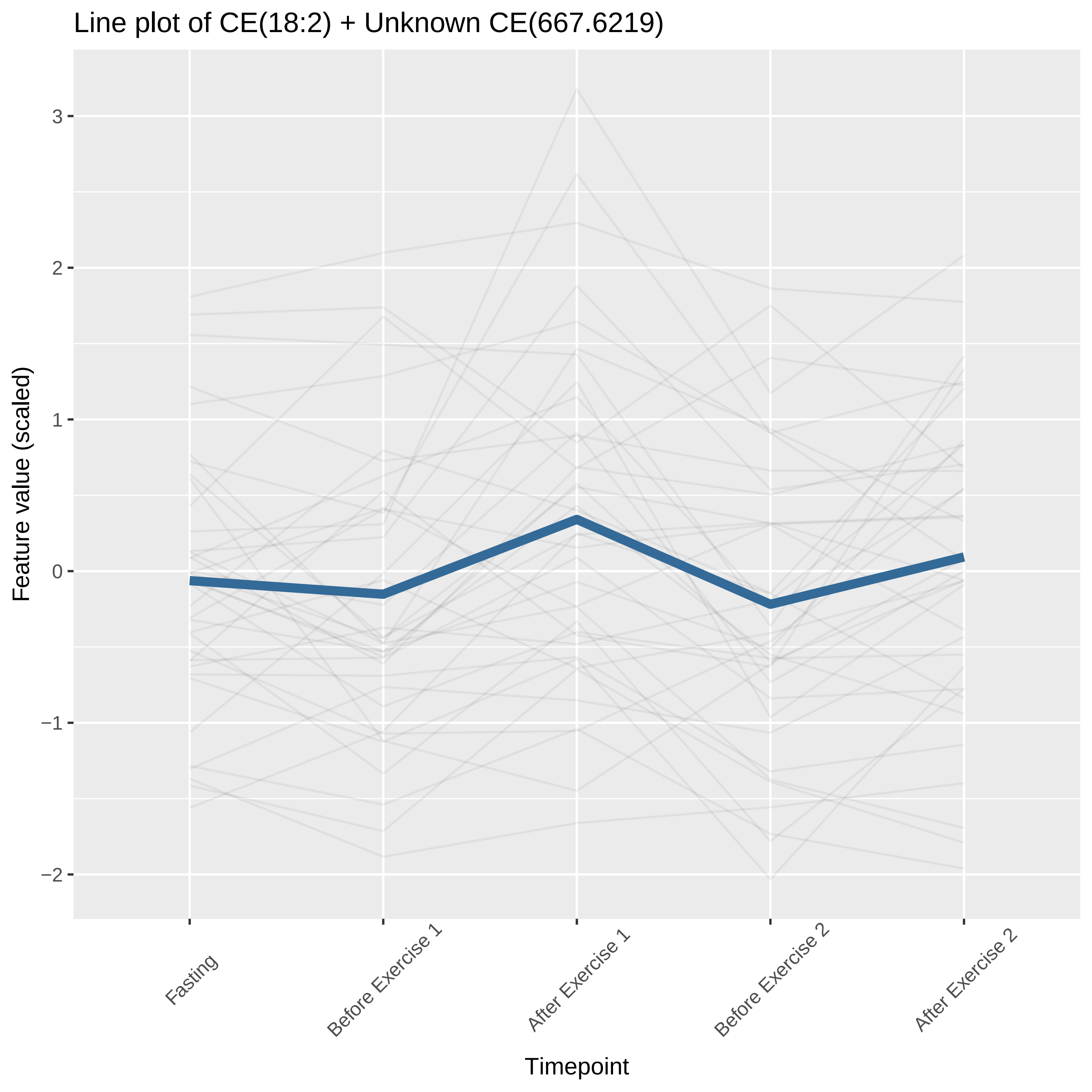
Line plot of CE(18:2) + Unknown CE(667.6219)
Feature value (scaled)
3
2
1
0
−1
−2
Fasting
Before Exercise 1
After Exercise 1
Before Exercise 2
After Exercise 2
Timepoint

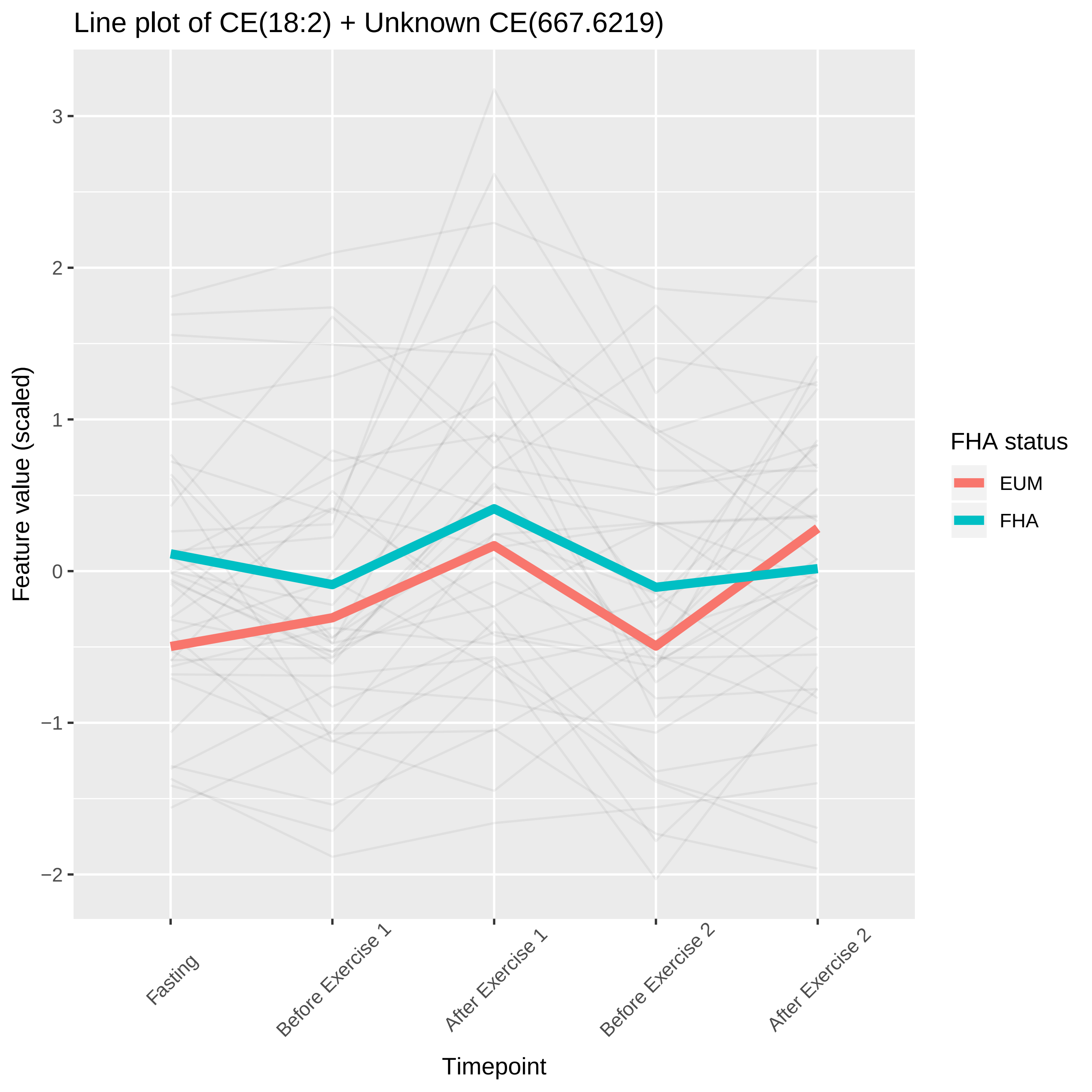
Line plot of CE(18:2) + Unknown CE(667.6219)
Feature value (scaled)
3
2
1
0
−1
−2
Fasting
Before Exercise 1
After Exercise 1
Before Exercise 2
After Exercise 2
Timepoint
FHA status
EUM
FHA

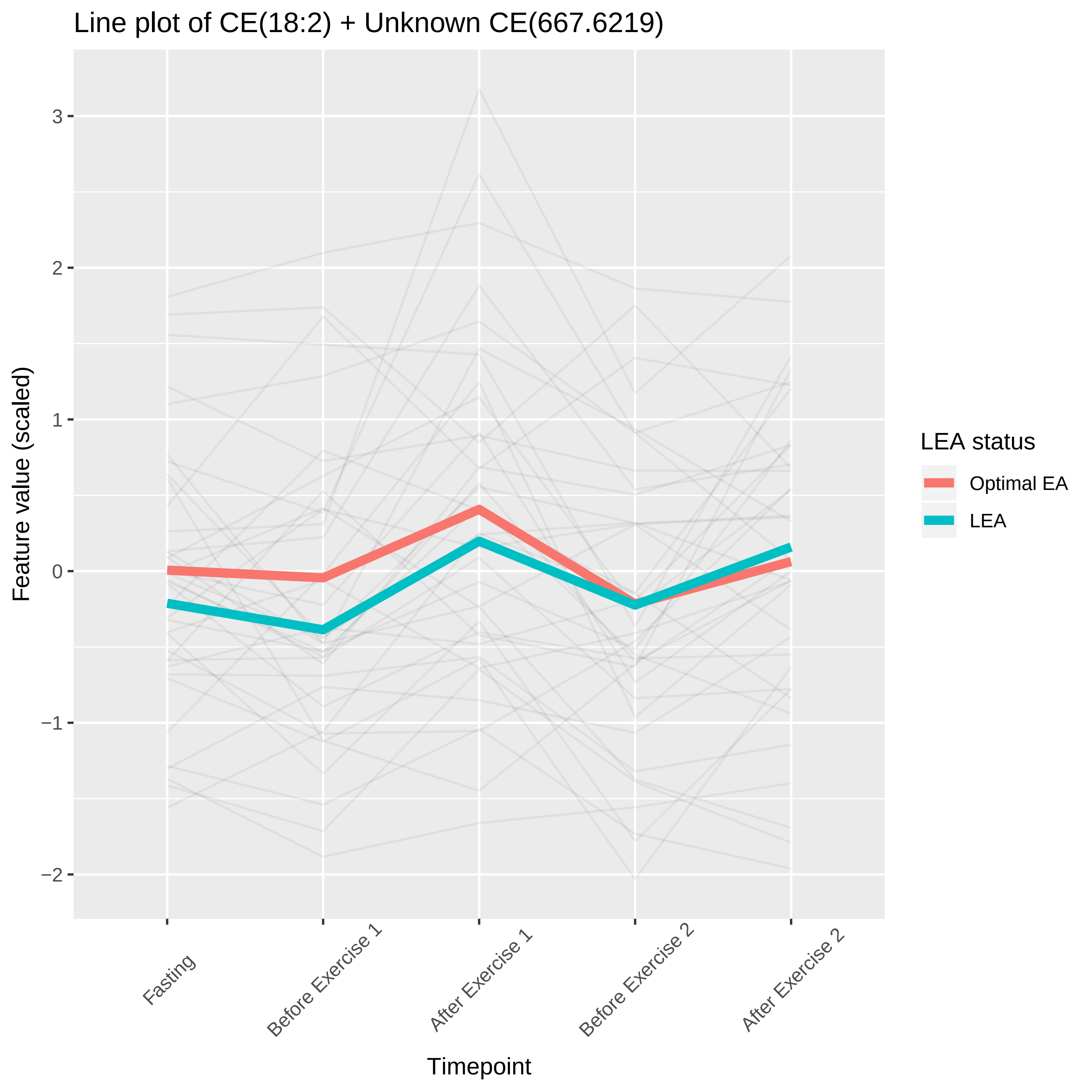
Line plot of CE(18:2) + Unknown CE(667.6219)
Feature value (scaled)
3
2
1
0
−1
−2
Fasting
Before Exercise 1
After Exercise 1
Before Exercise 2
After Exercise 2
Timepoint
LEA status
Optimal EA
LEA

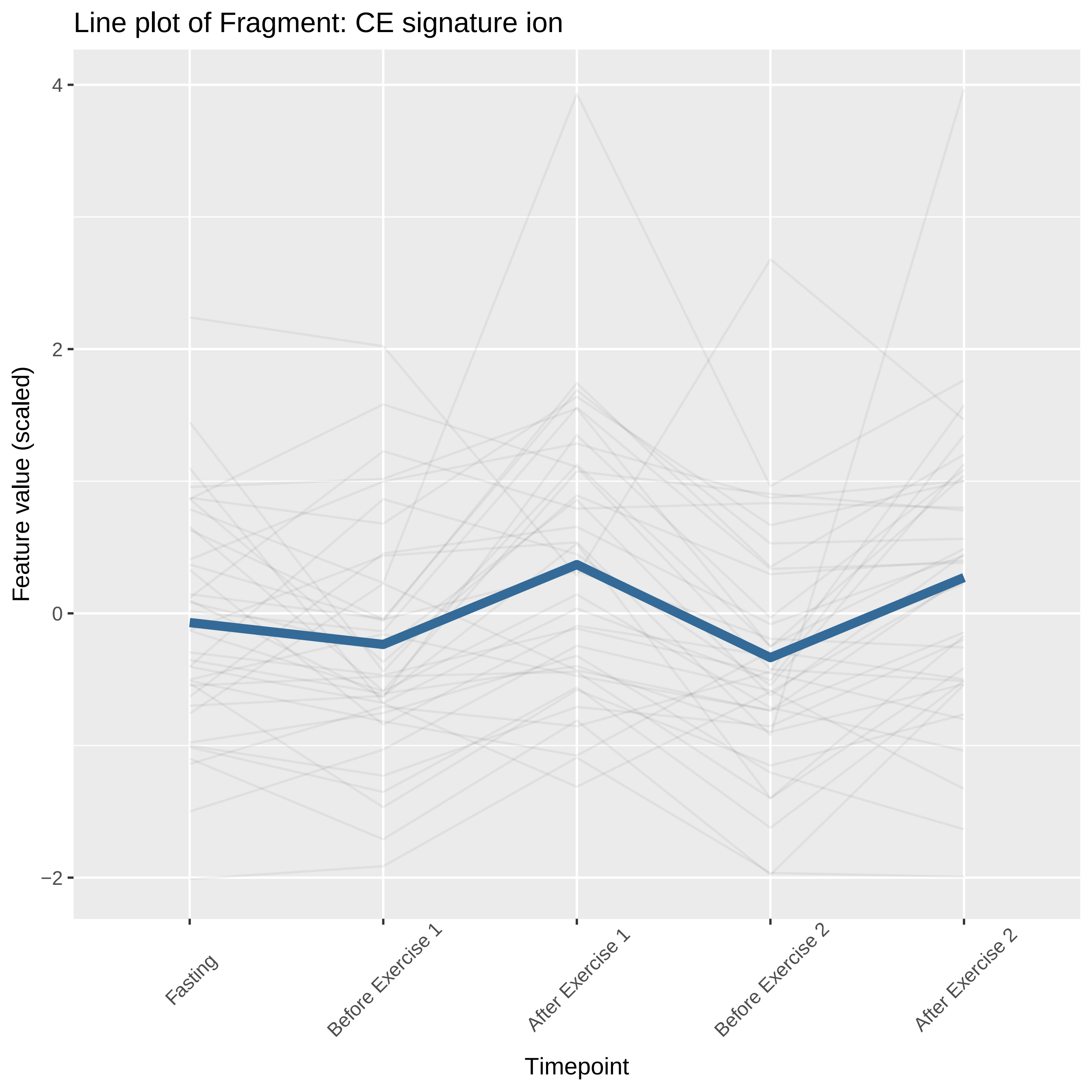

Line plot of Fragment: CE signature ion
Feature value (scaled)
4
2
0
−2
Fasting
Before Exercise 1
After Exercise 1
Before Exercise 2
After Exercise 2
Timepoint

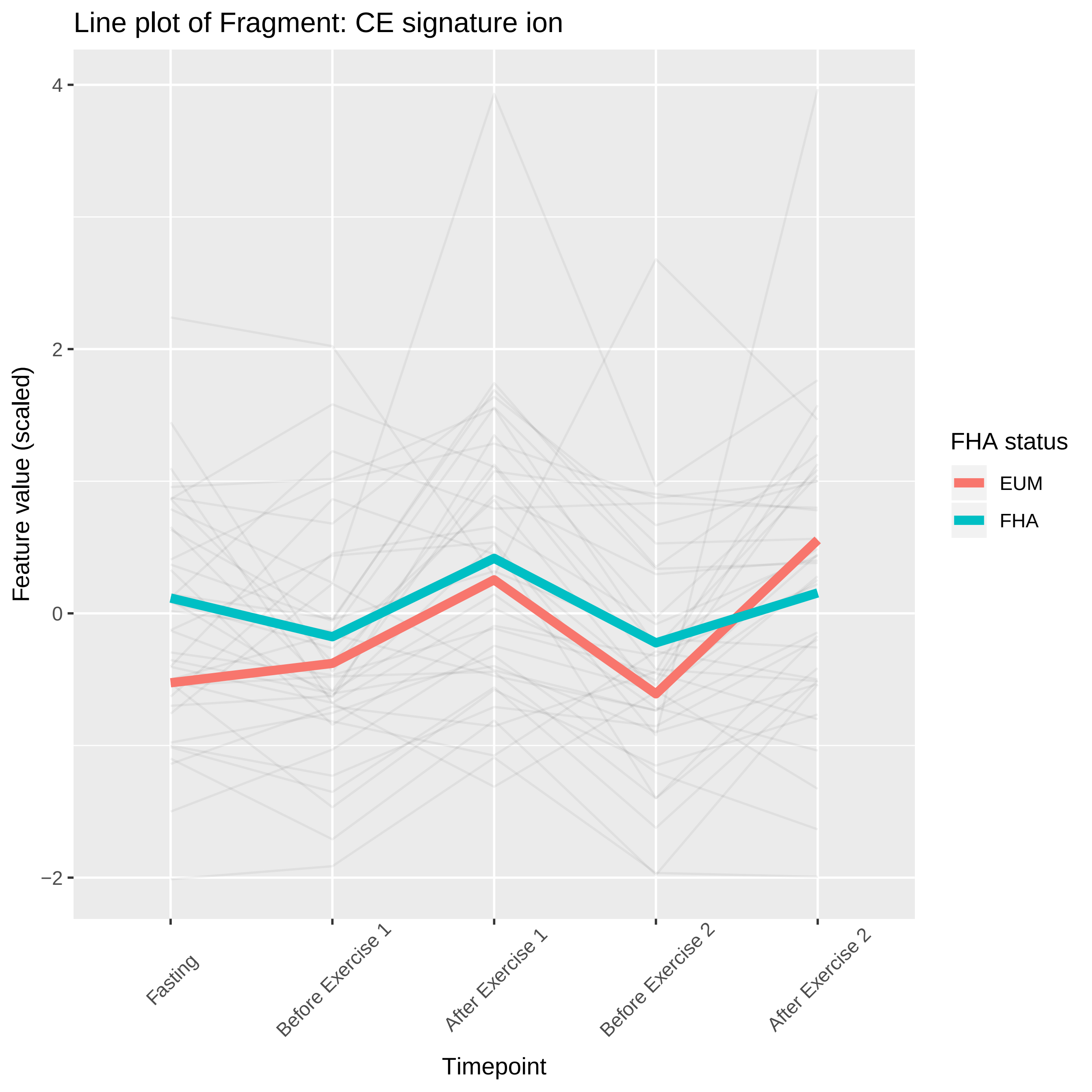

Line plot of Fragment: CE signature ion
Feature value (scaled)
4
2
0
−2
Fasting
Before Exercise 1
After Exercise 1
Before Exercise 2
After Exercise 2
Timepoint
FHA status
EUM
FHA

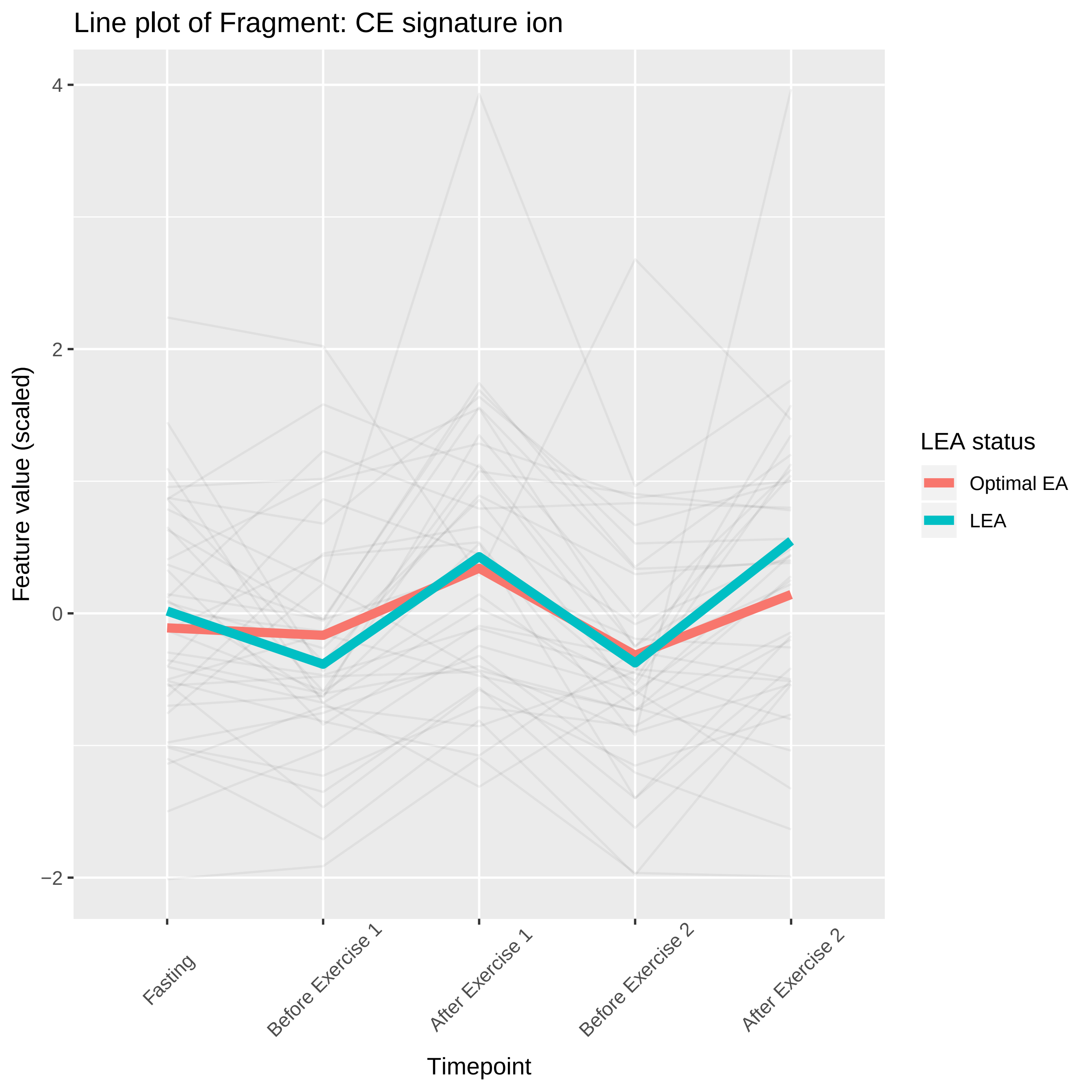
Line plot of Fragment: CE signature ion
Feature value (scaled)
4
2
0
−2
Fasting
Before Exercise 1
After Exercise 1
Before Exercise 2
After Exercise 2
Timepoint
LEA status
Optimal EA
LEA
