# Supplementary figures and images for: Lipidomic profiles, lipid trajectories and clinical biomarkers in female elite endurance athletes
Source: Sci Rep. 2020 Feb 11;10:2349. doi: 10.1038/s41598-020-59127-8 (PMC7012926; doi:10.1038/s41598-020-59127-8)

## LEA Lipidomics traj clusters

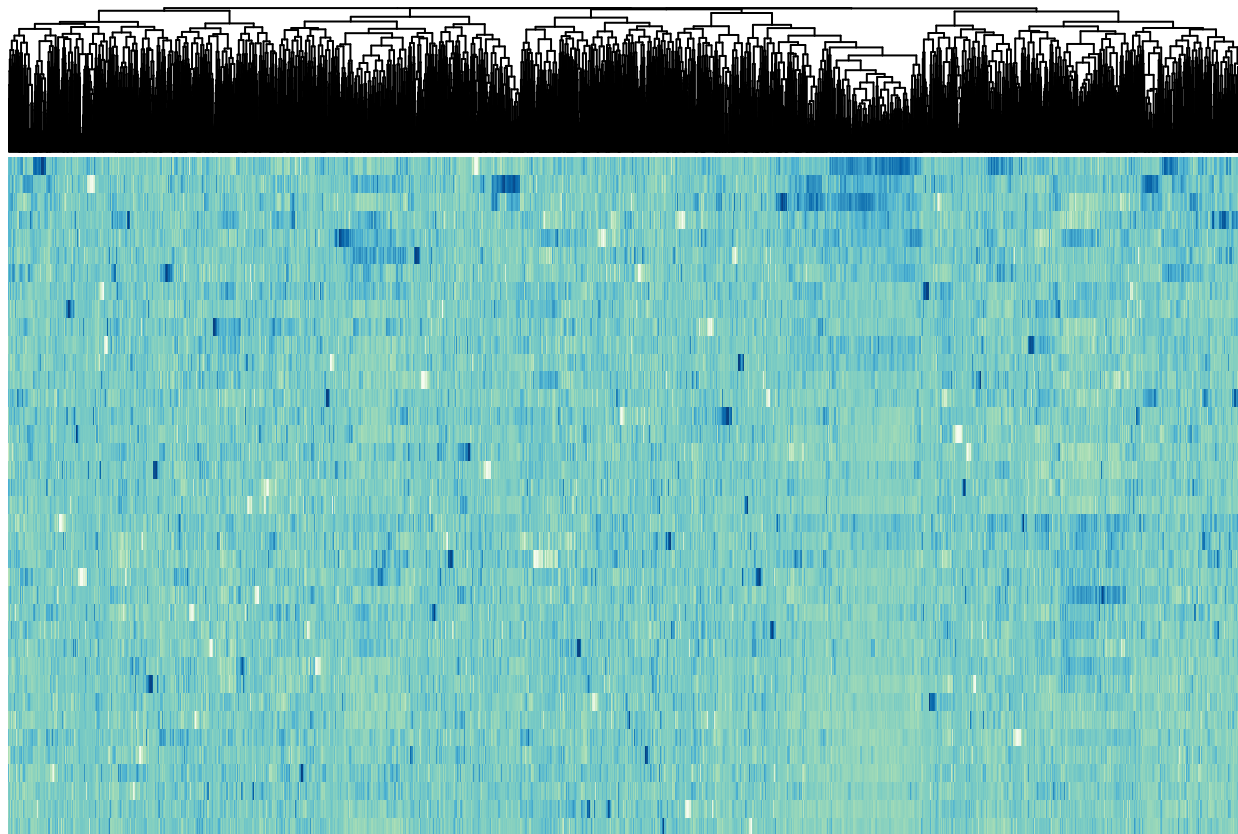

Supplement: Supplementary file 2 — Supplementary information 2. [file 41598_2020_59127_MOESM2_ESM.pdf]
