# Supplementary material for: Lipidomic profiles, lipid trajectories and clinical biomarkers in female elite endurance athletes
Source: Sci Rep. 2020 Feb 11;10:2349. doi: 10.1038/s41598-020-59127-8 (PMC7012926; doi:10.1038/s41598-020-59127-8)

SUPPLEMENTARY TEXT 1

MS spectra for the Steno Diabetes Center in-house library annotated lipids for the pool sample

SM(d33:1): m/z= 689.56; RT=6.63

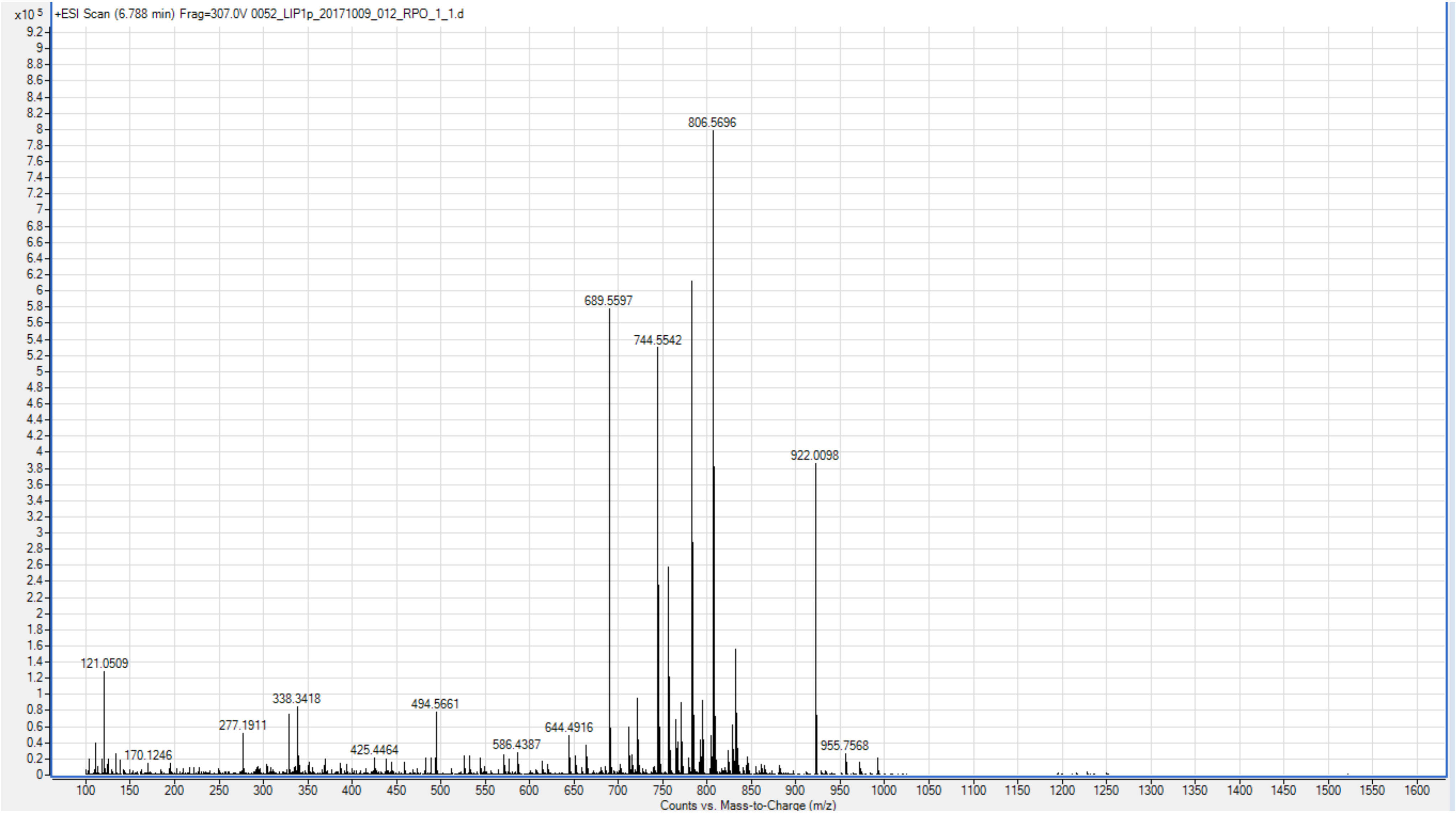

PC(O-36:2): m/z=691.5884; RT=6.4

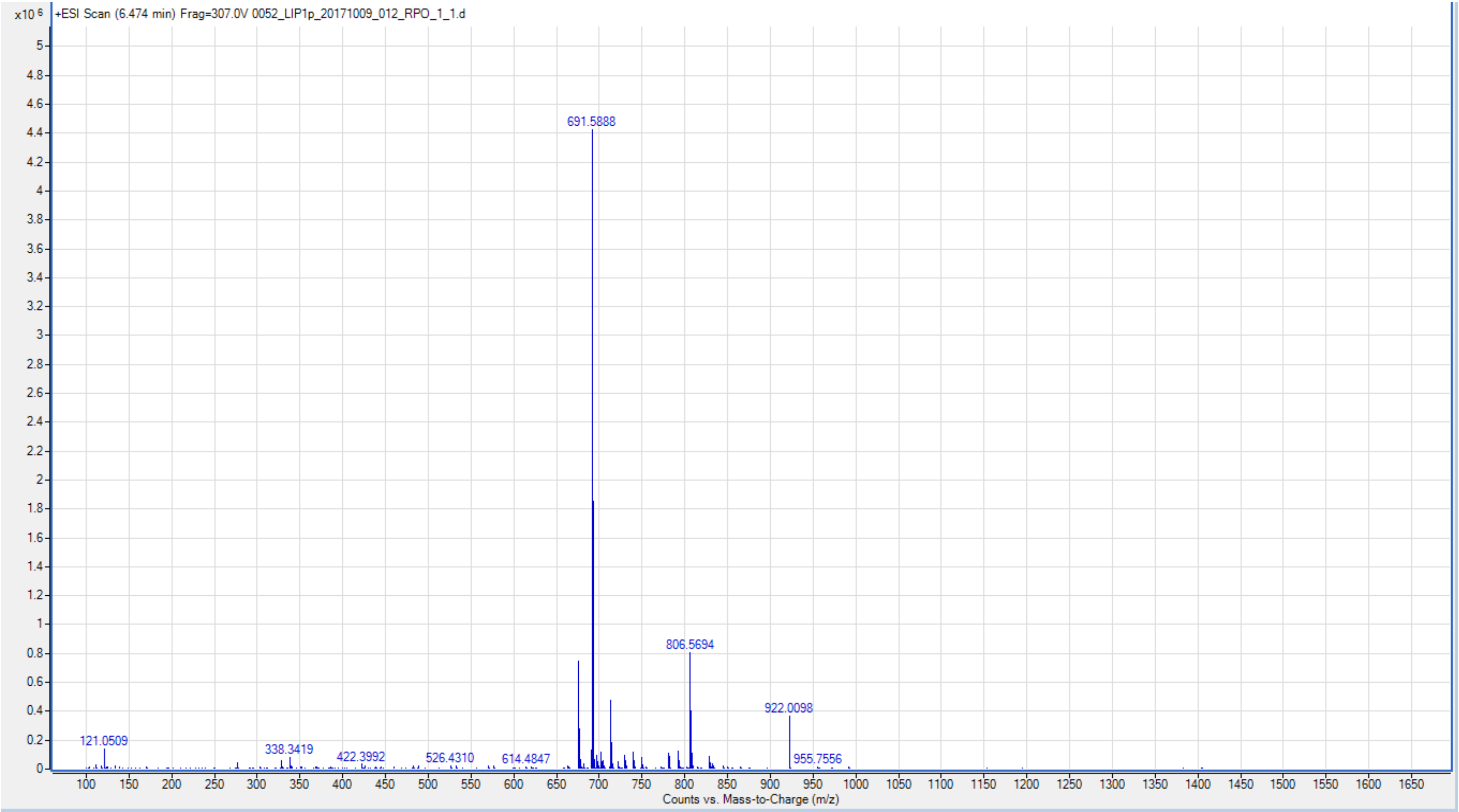

LPC(18:2): m/z=520.34; RT=3.79

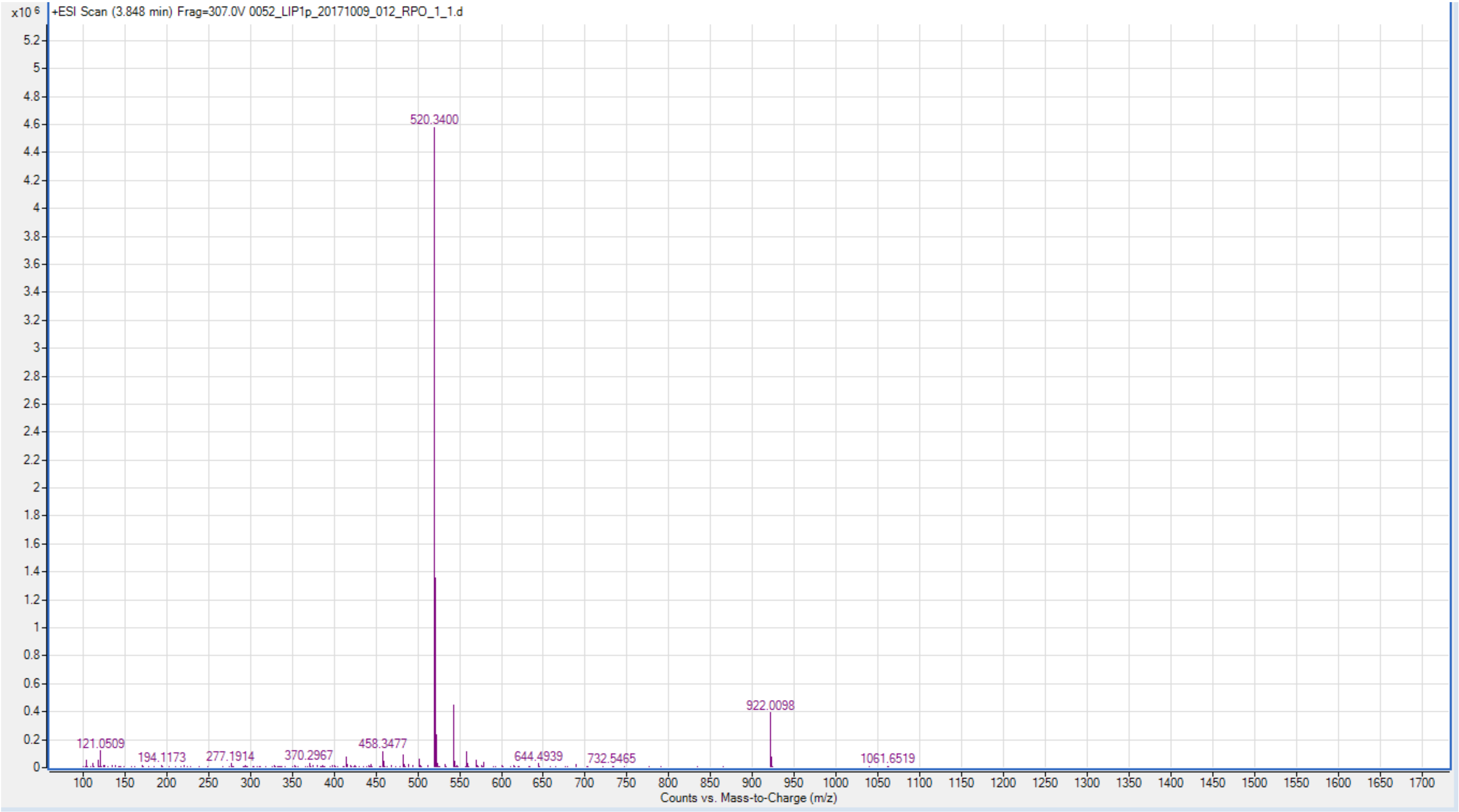

SM(d38:1): m/z=759.64; RT=7.82

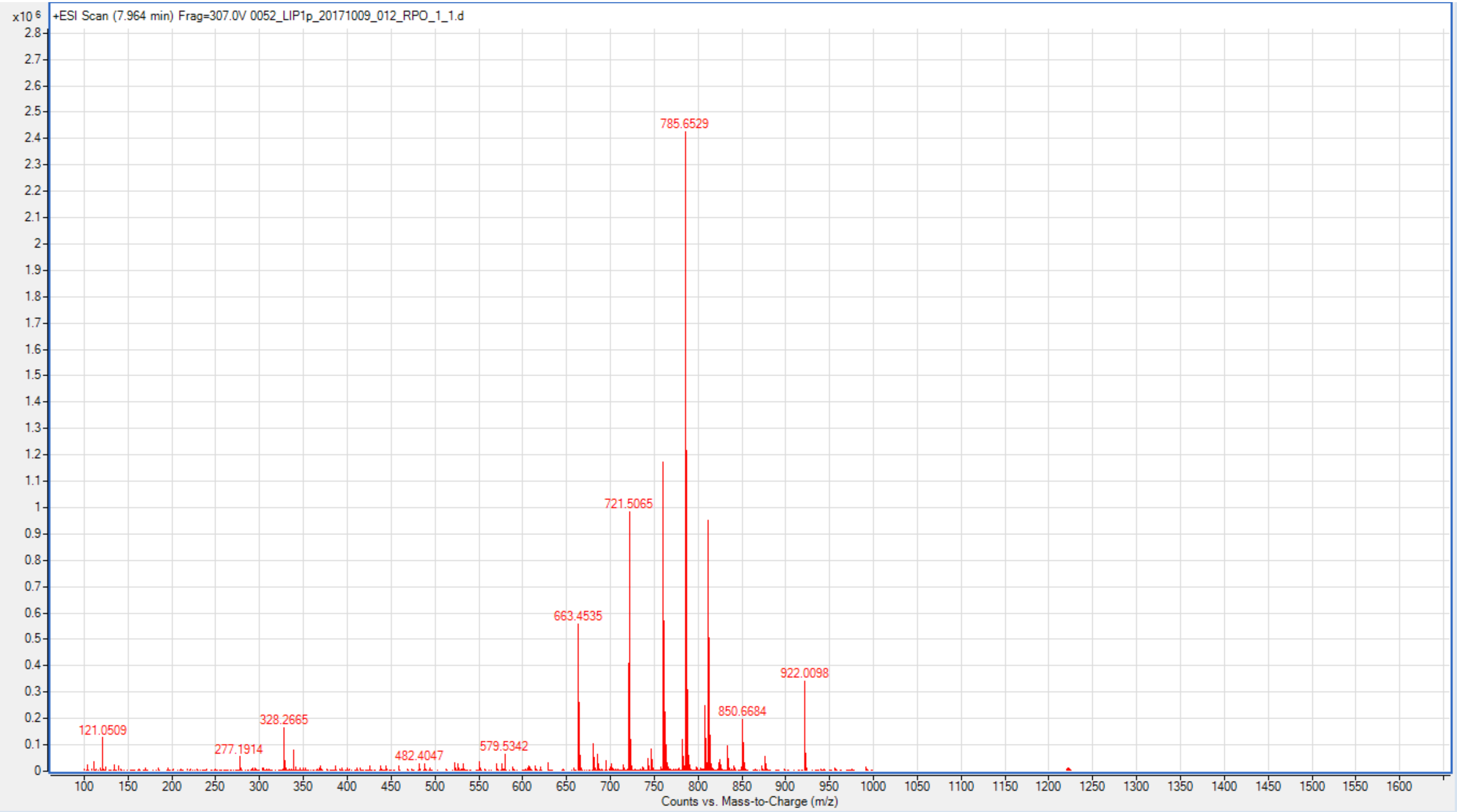

SM(d32:1): m/z=675.54; RT=6.35

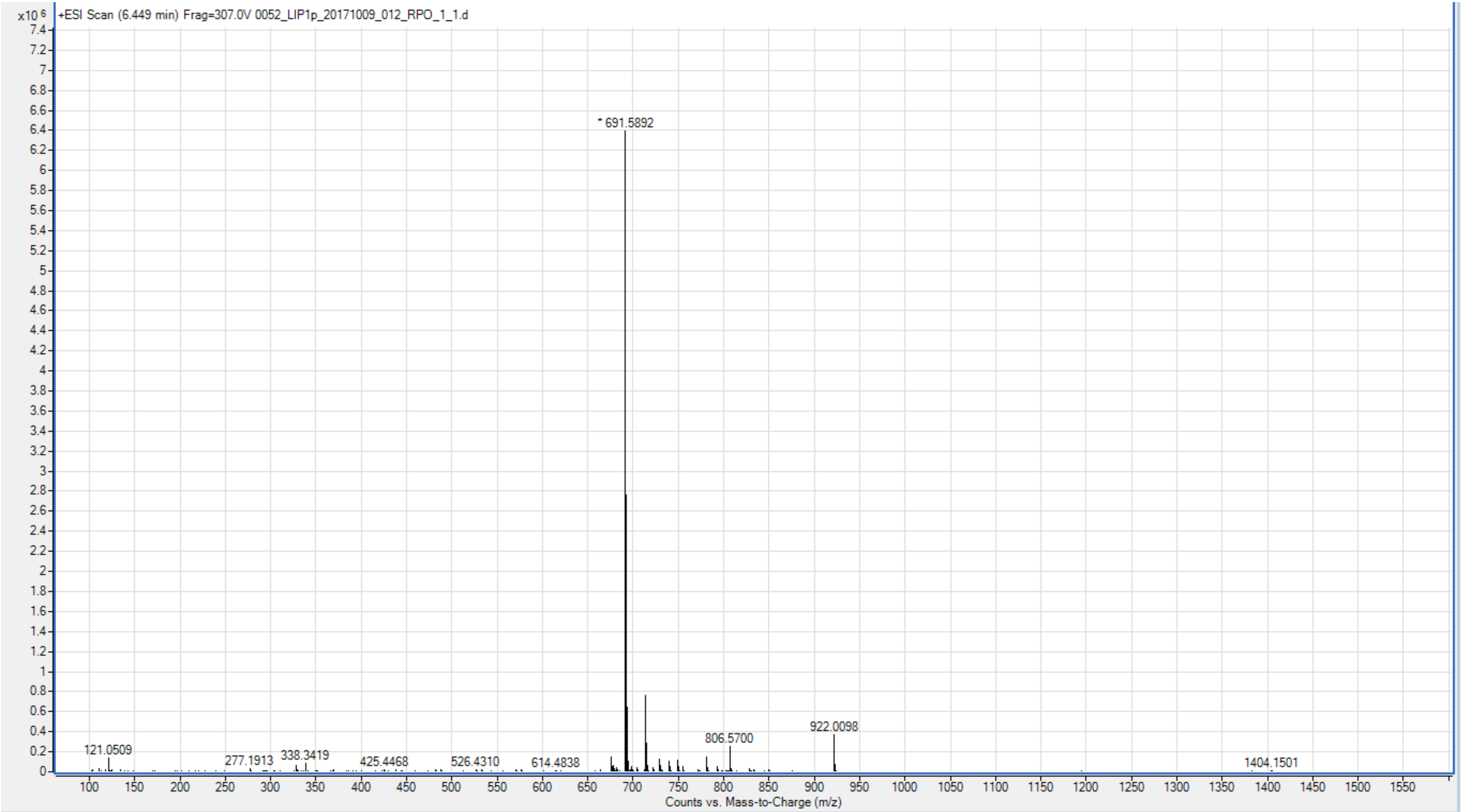

SM(d18:2/18:1): m/z=727.57; RT=6.4

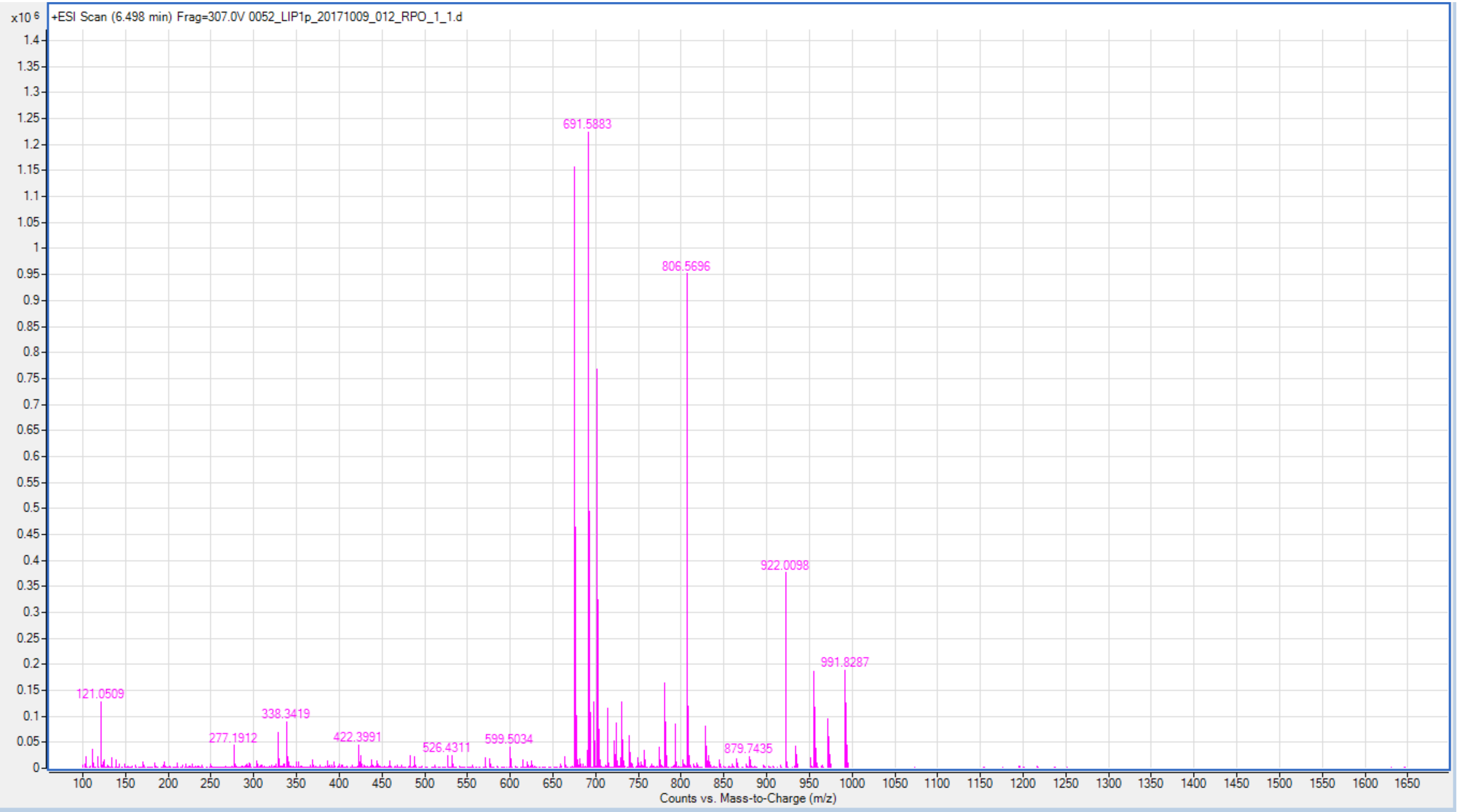

PC(42:6): m/z=862.63; RT=7.11

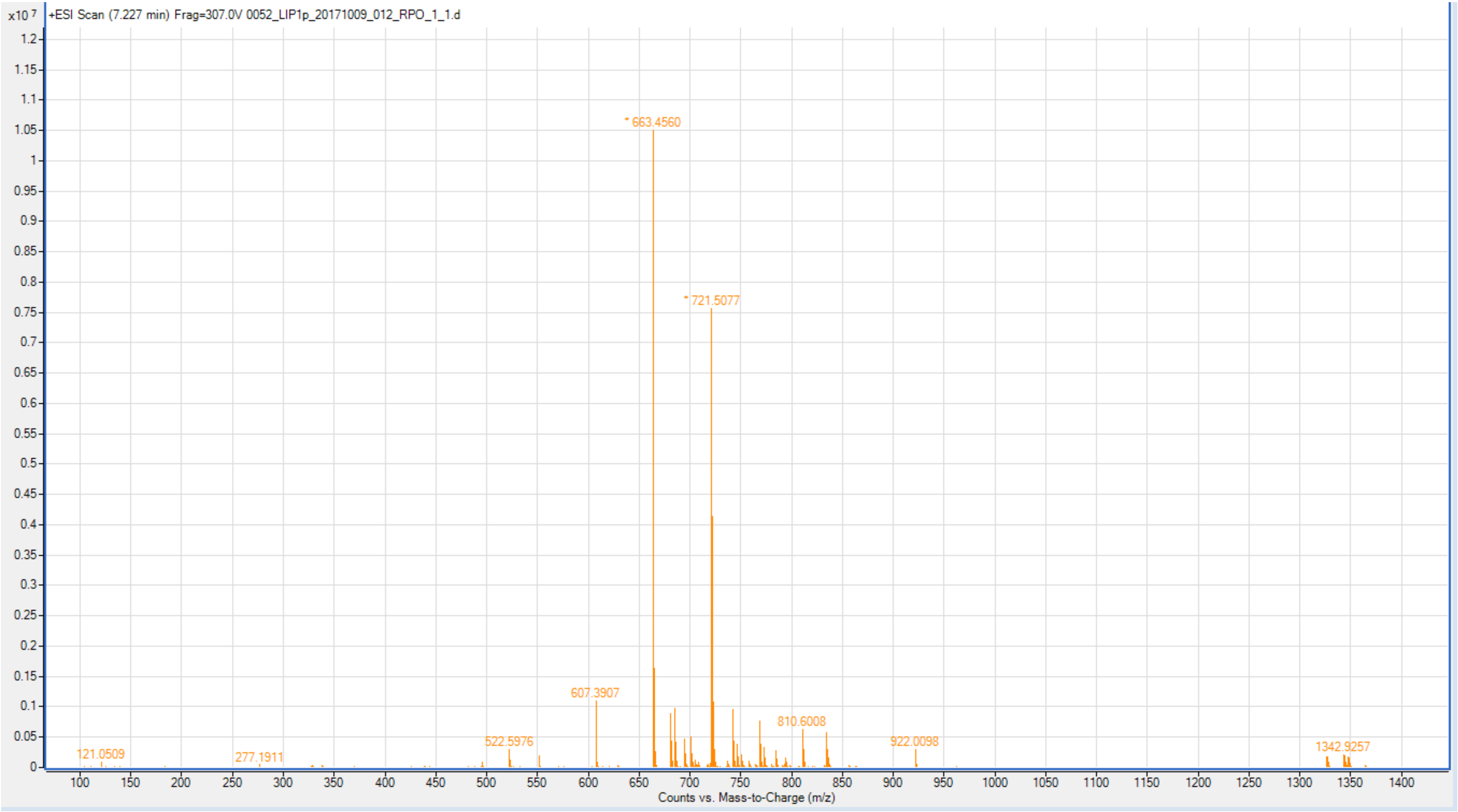

Supplement: Supplementary file 4 — Supplementary information 4. [file 41598_2020_59127_MOESM4_ESM.pdf]
